# Common, intermediate and well‐documented HLA alleles in world populations: CIWD version 3.0.0

**DOI:** 10.1111/tan.13811

**Published:** 2020-01-31

**Authors:** Carolyn K. Hurley, Jane Kempenich, Kim Wadsworth, Jürgen Sauter, Jan A. Hofmann, Daniel Schefzyk, Alexander H. Schmidt, Pablo Galarza, Maria B. R. Cardozo, Malgorzata Dudkiewicz, Lucie Houdova, Pavel Jindra, Betina S. Sorensen, Latha Jagannathan, Ankit Mathur, Tiina Linjama, Tigran Torosian, Rafi Freudenberger, Anastasios Manolis, John Mavrommatis, Nezih Cereb, Sigal Manor, Nira Shriki, Nicoletta Sacchi, Reem Ameen, Raewyn Fisher, Heather Dunckley, Irene Andersen, Ahmed Alaskar, Mohsen Alzahrani, Ali Hajeer, Dunia Jawdat, Grazia Nicoloso, Pawinee Kupatawintu, Louise Cho, Ashminder Kaur, Mats Bengtsson, Jason Dehn

**Affiliations:** ^1^ Department of Oncology Georgetown University Washington District of Columbia; ^2^ National Marrow Donor Program Minneapolis Minnesota; ^3^ DKMS Tübingen Germany; ^4^ Argentine HSC Donors Registry Buenos Aires Argentina; ^5^ Central Unrelated Potential Bone Marrow Donor and Cord Blood Registry POLTRANSPLANT Warsaw Poland; ^6^ University of West Bohemia, New Technologies for the Information Society Pilsen Czech Republic; ^7^ Czech National Marrow Donors Registry and University Hospital Pilsen Pilsen Czech Republic; ^8^ Danish Stem Cell Donors—West Aarhus University Hospital Aarhus Denmark; ^9^ DKMS BMST Foundation India Bangalore India; ^10^ Bangalore Medical Services Trust Bangalore India; ^11^ Finnish Stem Cell Registry Helsinki Finland; ^12^ Fundacja DKMS Warsaw Poland; ^13^ Gift of Life Marrow Registry Florida; ^14^ Hellenic Transplant Organization (HTO) Athens Greece; ^15^ DATRI Blood Stem Cell Donor Registry Chennai India; ^16^ Israel‐Ezer Mizion Bone Marrow Donor Registry Bnei Brak Israel; ^17^ Italian Bone Marrow Donor Registry Genoa Italy; ^18^ Kuwait National Stem Cell Registry Jabriya Kuwait; ^19^ New Zealand Bone Marrow Donor Registry Auckland New Zealand; ^20^ New Zealand Blood Service Auckland New Zealand; ^21^ Norwegian Bone Marrow Donor Registry Oslo Norway; ^22^ Saudi Stem Cell Donor Registry, King Abdullah International Medical Research Center, King Saud bin Abdulaziz University for Health Sciences, King Abdulaziz Medical City, Riyadh Ministry of National Guard Health Affairs Riyadh Saudi Arabia; ^23^ Swiss Blood Stem Cells Bern Switzerland; ^24^ Thai National Stem Cell Donor Registry Bangkok Thailand; ^25^ The Bone Marrow Donor Programme Singapore; ^26^ Tobias Registry of Swedish Bone Marrow Donors and Department of Immunology, Genetics and Pathology Uppsala University Uppsala Sweden

**Keywords:** alleles, ethnic groups, gene frequency, HLA

## Abstract

A catalog of common, intermediate and well‐documented (CIWD) HLA‐A, ‐B, ‐C, ‐DRB1, ‐DRB3, ‐DRB4, ‐DRB5, ‐DQB1 and ‐DPB1 alleles has been compiled from over 8 million individuals using data from 20 unrelated hematopoietic stem cell volunteer donor registries. Individuals are divided into seven geographic/ancestral/ethnic groups and data are summarized for each group and for the total population. P (two‐field) and G group assignments are divided into one of four frequency categories: common (≥1 in 10 000), intermediate (≥1 in 100 000), well‐documented (≥5 occurrences) or not‐CIWD. Overall 26% of alleles in IPD‐IMGT/HLA version 3.31.0 at P group resolution fall into the three CIWD categories. The two‐field catalog includes 18% (n = 545) common, 17% (n = 513) intermediate, and 65% (n = 1997) well‐documented alleles. Full‐field allele frequency data are provided but are limited in value by the variations in resolution used by the registries. A recommended CIWD list is based on the most frequent category in the total or any of the seven geographic/ancestral/ethnic groups. Data are also provided so users can compile a catalog specific to the population groups that they serve. Comparisons are made to three previous CWD reports representing more limited population groups. This catalog, CIWD version 3.0.0, is a step closer to the collection of global HLA frequencies and to a clearer view of HLA diversity in the human population as a whole.

## 1 INTRODUCTION

Identification of the highly polymorphic HLA genes (HLA‐A, ‐B, ‐C, ‐DR, ‐DQ, and ‐DP) in the clinical laboratory (ie, HLA “typing”) ensures that hematopoietic stem cell donor and recipient are HLA matched at high‐resolution, enabling engraftment of donor cells and avoiding detrimental immune responses (eg, graft vs host disease).^1^, ^2^ In solid organ transplantation, evaluation of HLA differences is used to select donors who will not react with or induce donor‐specific antibodies to avoid graft rejection.^3^, ^4^, ^5^ Other uses of HLA typing are to guide immunotherapies,^6^ prevent adverse drug reactions,^7^ diagnose autoimmune diseases,^8^ and inform HLA population genetics.^9^ The frequency of specific HLA alleles in human populations varies, influenced by natural selection.^9^

The almost exponential increase in the known HLA alleles over time^10^, ^11^ has been paralleled by changes in the methods used to type those alleles. Serologic typing of HLA proteins was replaced by DNA‐based methods that identified the presence or absence of specific polymorphisms through the binding of oligonucleotide probes or primers. Next, DNA sequencing, first by Sanger‐based protocols and now by next generation sequencing, has defined the nucleotide sequence of large segments of each HLA gene. Variation in the ability to assign polymorphic residues to one haplotype vs the other has also varied with the reagents and methods used producing, in many cases, ambiguity in the specific genotype carried by an individual. The history of all of these changes in the known allele database and the typing methods is reflected in the wide variety of HLA assignments found in the millions of individuals listed in donor registries around the world.^12^, ^13^

An added challenge is that the method(s) and reagents used are not always linked to the HLA assignment generated, especially within the databases of unrelated hematopoietic stem cell donor registries. This makes it difficult to determine if the assignment truly reflects any unresolved ambiguity. For example, B*51:01:01:01 might be assigned by a laboratory based on a sequence that includes all exons and introns but not the 3′ untranslated region (UTR). Alleles identified later that differ in this UTR (eg, B*51:01:01:02) make the assignment ambiguous. Likewise, it is not clear from the HLA assignment what level of resolution was applied. For example, a two‐field assignment, A*01:01, is not clear as to what alleles are included (eg, A*01:103 because it is included in the A*01:01:01G group) or excluded (A*01:87N because it is a nonexpressed allele and the assignment was provided without the “P” because of registry specifications). This variation has presented a major challenge for determining the frequency of individual alleles.^13^, ^14^

Prompted by the complexity of HLA typing in the face of ever‐increasing allele numbers, attempts have been made to classify alleles based on their frequencies. In this way, if a laboratory resolves an HLA assignment down to several alternative genotypes (ie, ambiguous result), the laboratory might consider the allele frequencies associated with each genotype in making a final assignment without further testing. Initially, this effort was intended to provide guidance for external proficiency testing but rapidly became a reference for clinical decision making. The first classification system called the common and well‐documented (CWD) allele catalog was compiled by the American Society for Histocompatibility and Immunogenetics (ASHI) in 2007^15^ and updated in 2012 as version 2.0.0^16^ (Supporting Information Table S1). This effort was replicated by other worldwide groups, notably the European Federation for Immunogenetics (EFI CWD)^17^ and the China Marrow Donor Program (China CWD).^18^ A fourth study, with subjects overlapping the EFI study, used imputation to predict alleles at two‐field resolution.^19^ While the precise definitions of common and well‐documented differed somewhat among the studies, in general, alleles were classified as common if they were observed in multiple population groups with frequencies greater than 1 in 1000 in groups of at least 1500 individuals. Well‐documented alleles were more restricted in their distribution with unclear frequencies but were observed at least five times by DNA sequencing or three times in a shared haplotype. The remainder of the alleles were classified as not‐CWD.

Solid organ and hematopoietic cell donation and transplantation programs are found in over 100 countries, representing nearly 90% of the worldwide population (https://www.who.int/transplantation/gkt/statistics/en/, October 2019). Typing of HLA to support this activity is challenged by the increasing ethnic diversity of the patient and donor populations including the frequent international source of unrelated hematopoietic cell transplantation donors.^20^, ^21^ For these reasons, an investigation of allele frequencies should take a worldwide focus. The aim of this study, a component of the 18th International HLA and Immunogenetics Workshop, was to collate the most comprehensive and diverse analysis of HLA and estimate frequencies in different geographic/ancestral/ethnic population groups.

## 2 MATERIALS AND METHODS

World Marrow Donor Association unrelated donor registries were invited to participate in sharing HLA data for this study. Donor HLA typing must have met the following conditions to be included: New volunteer donor recruitment testing within the years of 2012‐2018 regardless of current registry availability status; HLA assignment by sequencing (Sanger or next generation DNA sequencing) methods with resolution of at least antigen recognition domain (ARD) exons (ie, Class I exons 2 and 3; Class II exon 2); volunteers included must be consecutive registrants during the period of time of suitable HLA resolution (not just patient‐directed or directed registry upgrade testing); all HLA types during that time period must be submitted including those with allelic ambiguities. Supporting Information Table S2 describes the variations in the HLA nomenclature observed in the dataset.

Twenty registries responded by submitting volunteer donor data for loci (HLA‐A, ‐B, ‐C, ‐DRB1, ‐DRB3, ‐DRB4, ‐DRB5, ‐DQB1 and ‐DPB1) fitting the above conditions (Table 1). Insufficient data were available for HLA‐DQA1 and ‐DPA1 as these loci are not commonly typed by registries. Data were provided as a total allele count assigned to geographic/ancestral/ethnic groups, hereafter “population groups,” if such data was collected (Tables 2a and 2b). Ancestry categorization was defined by each registry and converted into seven population groups for this study: AFA (African/African American), API (Asian/Pacific Islands), EURO (European/European descent), MENA (Middle East/North Coast of Africa), HIS (South or Central America/Hispanic/Latino), NAM (Native American) and UNK (unknown/not asked/multiple ancestries/other).

**Table 1:** Participating registries and number of volunteer donors with HLA assignments contributed

Registry	Number of volunteer donors^a^	World Health Organization region^b^ based on registry location
Argentine HSC Donors Registry	129 879	Americas
Bone Marrow Donor Programme Singapore	32 875	Western Pacific
Central Unrelated Potential Bone Marrow Donor and Cord Blood Registry Poltransplant	6918	European
Czech National Marrow Donors Registry	36 289	European
Danish Stem Cell Donors—West	21 234	European
DKMS (Germany, Polska, UK, India, USA)	5 316 717	Multi‐region
Finnish Stem Cell Registry	20 427	European
Gift of Life Marrow Registry	125 489	Americas
Hellenic Transplant Organization (HTO)	121 088	European
India—DATRI Blood Stem Cell Donor Registry	347 989	South East Asia
Israel‐Ezer Mizion BMDR	138 175	European
Italian Bone Marrow Donor Registry	30 117	European
Saudi Stem Cell Donor Registry	34 729	Eastern Mediterranean
Kuwait National Stem Cell Registry	673	Eastern Mediterranean
New Zealand Bone Marrow Donor Registry	237	Western Pacific
NMDP/Be The Match‐USA and Mexico	1 567 473	Americas
Norwegian Bone Marrow Donor Registry	7202	European
Swiss Blood Stem Cells	84 789	European
Thai National Stem Cell Donor Registry	5000	South East Asia
Tobias Registry of Swedish Bone Marrow Donors	50 502	European
**Total**	**8 077 802**	

a Number of donors varied by locus. The largest number is listed.

b Regions and included countries are defined at http://apps.who.int/medicinedocs/en/d/Jwhozip16e/2.html#Jwhozip16e.2

**Table 2a:** Self‐reported ancestry^a^ of individuals included in the dataset and the number of P group assignments represented by each population group

	Number of HLA assignments (P group, two‐field)						
Ancestry	HLA‐A	HLA‐B	HLA‐C	HLA‐DRB1	HLA‐DRB3/4/5	HLA‐DQB1	HLA‐DPB1
African/African American (AFA)	362 966	370 020	347 465	373 023	72 964	361 723	333 978
Asian/Pacific Islands (API)	1 263 239^b^	1 274 300	1 195 602	1 305 847	117 098	1 299 499	1 080 334
European/European descent (EURO)	11 273 788	11 260 688	10 488 431	11 637 283	635 606	11 507 902	10 655 587
Middle East/North coast of Africa (MENA)	389 811	391 642	379 666	396 117	15 867	400 453	296 490
South or Central America/Hispanic/Latino (HIS)	640 949	655 091	606 689	668 011	193 504	654 807	580 236
Native American populations (NAM)	62 326	63 323	57 232	65 025	16 966	64 730	59 017
Unknown/Not asked/Multiple ancestries/Other (UNK)	1 229 127	1 248 774	1 149 774	1 286 614	128 841	1 195 557	966 013
**Total**	**15 222 206**	**15 263 838**	**14 224 859**	**15 731 920**	**1 180 846**	**15 484 671**	**13 971 655**

a Ancestry designations were determined by each contributing registry.

b Odd number is because of removal of alleles having incorrect format/assignment not based on HLA nomenclature.

**Table 2b:** Self‐reported ancestry^a^ of individuals included in the dataset and the number of total^b^ assignments represented by each population group

	Number of HLA assignments (total)						
Ancestry	HLA‐A	HLA‐B	HLA‐C	HLA‐DRB1	HLA‐DRB3/4/5	HLA‐DQB1	HLA‐DPB1
African/African American (AFA)	388 476	388 579	389 619	389 241	198 134	378 275	336 535
Asian/Pacific Islands (API)	1 291 125^c^	1 298 351	1 255 403	1 318 229	297 151	1 314 598	1 082 177
European/European descent (EURO)	11 929 417	11 941 489	11 827 887	11 938 778	1 329 462	11 735 570	10 680 854
Middle East/North coast of Africa (MENA)	402 447	402 160	403 229	403 373	25 832	402 840	296 914
South or Central America/Hispanic/Latino (HIS)	700 632	700 912	690 043	700 830	400 002	678 680	581 973
Native American populations (NAM)	66 971	66 967	67 072	66 984	39 907	66 309	59 113
Unknown/Not asked/Multiple ancestries/Other (UNK)	1 320 493	1 320 714	1 302 662	1 321 251	226 038	1 227 926	969 187
**Total**	**16 099 561**	**16 119 172**	**15 935 915**	**16 138 686**	**2 516 526**	**15 804 198**	**14 006 753**

a Ancestry designations were determined by each contributing registry.

b Total assignments include HLA assignments at all levels of resolution as submitted by the registries.

c Odd number is because of removal of alleles having incorrect format/assignment not based on HLA nomenclature.

### 2.1 Allele designation and resolution

Assignments include only alleles found in IPD‐IMGT/HLA version 3.31.0 from January 2018.^11^ All assignments are evaluated as submitted with the following exceptions (also described in Supporting Information Table S2): (a) HLA assignments provided as an NMDP multiple allele code,^22^ defined with only alleles in a single G group,^10^ are converted to the respective G nomenclature. (b) Multiple allele‐coded assignments that are not the equivalent of a G assignment (eg, A*01:ANDKJ includes A*01:01 but also A*01:52 which is not included in A*01:01:01G) are merged. (c) Assignments reported at three‐field resolution without the addition of the expected G (eg, A*01:01:01 instead of A*01:01:01G) are merged into the G group assignment. In these cases, it is likely that the assignment was made based on the sequence of the ARD‐encoding exons but confirming information is not available. (d) A summary G value is based on combining all assignments that would be included in a G group (eg, A*01:01:01G total includes assignments of A*01:01:01, A*01:01:01G, A*01:01:01:01, A*01:04N, etc). (e) Two‐field allele summaries are at the resolution level of P groups; known nonexpressed alleles (eg, A*01:04N) are listed separately. The P group includes both G and two‐field assignments (eg, B*07:02P total includes B*07:02, B*07:02P, B*07:02:02, B*07:02:03, B*07:02:01G, all assignments within the B*07:02:01G group except nonexpressed alleles, etc). (f) Data submitted with third‐field (with or without a G) or fourth‐field nomenclature are used for subset analysis showing allele occurrence but the main analysis is evaluated based on the first two fields of the allele name because of inconsistent typing for the third‐ and fourth‐field data across the cohort. (g) Assignment of novel alleles are assignments without a nomenclature designation; these assignments were merged (eg, A*NEW) and included in the analysis.

### 2.2 Assignment of allele frequency categories

The total number of HLA assignments for each locus for each population group is provided in Table 2b. This number is based on the number of individuals assuming two HLA assignments for each locus with the exception of DRB3/DRB4/DRB5 (as described below). The total assignments include HLA assignments at all levels of resolution as submitted by the registries with some alleles removed because of incorrect format or faulty nomenclature. The frequency of each HLA assignment is calculated by dividing the number of alleles for that assignment by the total number of assignments. In cases where assignments are merged to form P or G groups, the individual assignments making up the P or G group (eg, A*02:01:01:01 or A*02:09 assignments when merged to form A*02:01P total) are not included in the total number of assignments for the P or G frequency evaluation (ie, allele assignments are not counted more than once). Supporting Information Tables [Link], [Link] provide the allele counts by population group.

In the 2012 CWD publication (2.0.0 CWD),^16^ common alleles were assigned based on presence in multiple, not necessarily all, population groups with frequencies that were known (Supporting Information Table S1). In general, these alleles were observed at frequencies of >0.001 in reference population groups of at least 1500 individuals. Later evaluations based on European^17^, ^19^ or Chinese populations^18^ were based on two‐field designations. While retaining the category designations, “common” alleles in this study include those present at least 1 in 10 000 in any population group or in the total dataset. This captures almost all the alleles designated as common in the three earlier studies as described in Results. We did not compare 3.0.0 CIWD to the CWD of the German stem cell donors because of the overlap in samples from the DKMS donor center and because the German data were obtained by imputation, not by direct allele counting.^19^

A new category, consistent with that used by the German registry as “well‐documented 1” (WD1),^19^ termed “intermediate” includes alleles found at frequencies less than 1 in 10 000 but at least 1 in 100 000. It should be noted that, because of the smaller groups typed for AFA, MENA, and NAM, it was not possible to assign alleles to the intermediate category as a single category without having met the occurrence threshold for well‐documented. So these groups only have two categories, common and well‐documented.

We have reserved the definition of “well‐documented” to include alleles that have been observed five or more times in unrelated individuals but not at the common or intermediate levels. The well‐documented category is independent of sample sizes of any population group and includes alleles that are rare, but whose existence in multiple random individuals is not in question. We refer to alleles in these three categories as CIWD. HLA‐DRB3/4/5 could not be evaluated for estimated frequency as these loci were reported in total occurrence, but could not sufficiently account for individuals who were not tested or where the gene was not present or homozygous. In addition, greater than 50% of the submitted HLA‐DRB3/4/5 typing contained allelic ambiguities. Given these phenomena, only well‐documented occurrences are provided.

Because alleles common to one or a few population groups do not necessarily reach this minimum frequency in the total population and because the dataset is heavily weighted toward individuals of EURO ancestry, we have chosen to base our summary CIWD assignments on the most frequent category observed within the total or within individual population groups. For example, B*07:12 is well‐documented in the overall population, not‐CIWD in most of the individual population groups but common in AFA so the summary CIWD assignment is common. The tables also provide frequency categories based on the total population as well as categorizations for each group.

## 3 RESULTS

This update to the CWD allele catalog evaluates the frequency of over 15.2 million two‐field P group assignments at each locus of HLA‐A, ‐B, ‐DRB1, and ‐DQB1 in over 8 million individuals whose HLA types are found in 20 worldwide hematopoietic stem cell registries listing their ancestries in seven categories (Tables 1, 2a and 2b). Substantial data are also present for HLA‐C (14.2 million two‐field) and ‐DPB1 (14.0 million) and for HLA‐DRB3/4/5 (1.2 million two‐field assignments). Data used in this analysis are included in Supporting Information tables and will also be posted on a public web site (https://www.ihiw18.org/) so that laboratories might perform their own analyses.

**Figure 1:**
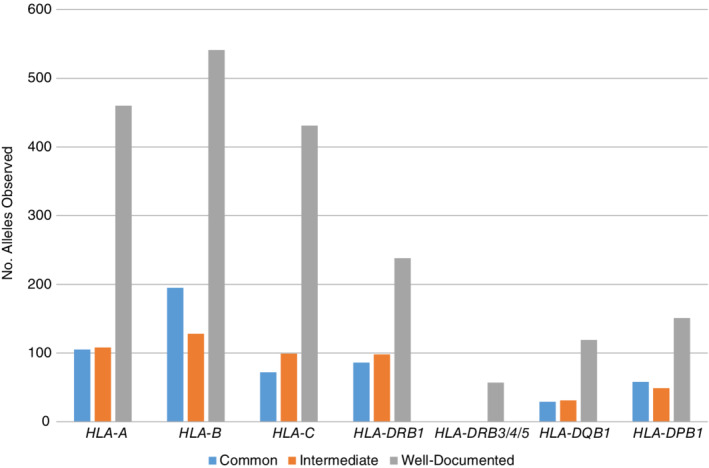
Distribution of HLA alleles into three frequency categories (common, intermediate, well‐documented) at P group (two‐field) resolution

**Figure 2:**
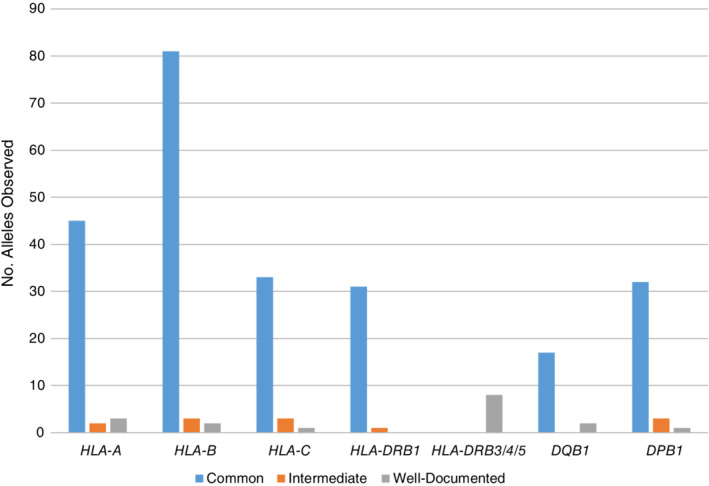
Distribution of HLA alleles into three frequency categories at G group resolution

Table 3 describes the overall frequency of alleles at each locus observed compared with the total number of alleles in IPD‐IMGT/HLA 3.31.0. About half of the known two‐field P group alleles are found within registry databases (eg, 54% HLA‐A alleles); about 25% are assigned as CIWD (eg, 24% HLA‐A alleles). HLA‐DPB1 has the highest percentage of total alleles observed at 69% with 41% overall assigned as CIWD. Figures 1 and 2 describe the number of alleles, at P (two‐field) and G resolution, assigned to each frequency category.

**Table 3:** The representation of total P group two‐field HLA assignments in registry databases

Locus	Total P group alleles in IPD‐IMGT/HLA 3.31.0	Alleles observed in registry dataset	Percentage of total alleles observed	Alleles in CIWD categories	Percentage of alleles assigned as CIWD
HLA‐A	2827	1521	53.8	673	23.8
HLA‐B	3537	1853	52.4	864	24.4
HLA‐C	2494	1366	54.8	602	24.1
HLA‐DRB1	1559	817	52.4	422	27.1
HLA‐DRB3	125	66	52.8	32	25.6
HLA‐DRB4	63	17	27.0	10	15.9
HLA‐DRB5	50	31	62.0	15	30.0
HLA‐DQB1	669	384	57.4	179	26.8
HLA‐DPB1	628	430	68.5	258	41.1

Abbreviations: C, common; I, intermediate; WD, well‐documented.

## 4 CIWD HLA ALLELES AT TWO‐FIELD (P) DESIGNATION

HLA‐B and ‐DQB1 serve as examples of the two‐field P level resolution assignments for the current data compared with previous CWD reports (Table 4).^16^, ^17^, ^18^ The analysis is based on over 15 million two‐field assignments at each locus (Table 2a).

**Table 4:** Overall comparison of HLA P two‐field assignments among CIWD catalogs^a^^,^ b

	2.0.0 CWD^c^			EFI CWD^c^			China CWD^c^		
**HLA‐A**									
**3.0.0 CIWD**	Common n = 63	WD n = 169	Not‐CWD^c^ n = 496	Common n = 35	WD n = 180	Not‐CWD n = 513	Common n = 26	WD n = 112	Not‐CWD n = 590
Common total n = 105	60	43	2	34	36	35	26	28	51
Intermediate n = 108	3	64	41	1	31	76	0	18	90
Well‐documented n = 460	0	59	401	0	106	354	0	21	439
Not‐CIWD^d^ n = 55	0	3	52	0	7	48	0	45	10
**HLA‐B**									
**3.0.0 CIWD**	Common n = 121	WD n = 230	Not‐CWD n = 578	Common n = 62	WD n = 257	Not‐CWD n = 610	Common n = 54	WD n = 143	Not‐CWD n = 732
Common total n = 195	119	74	2	61	60	74	54	60	81
Intermediate n = 128	2	78	48	1	39	88	0	14	114
Well‐documented n = 541	0	71	470	0	143	398	0	23	518
Not‐CIWD n = 65	0	7	58	0	15	50	0	46	19
**HLA‐C**									
**3.0.0 CIWD**	Common n = 36	WD n = 87	Not‐CWD n = 508	Common n = 29	WD n = 158	Not‐CWD n = 444	Common n = 24	WD n = 82	Not‐CWD n = 525
Common total n = 72	36	34	2	26	16	30	24	23	25
Intermediate n = 99	0	41	58	3	28	68	0	18	81
Well‐documented n = 431	0	11	420	0	108	323	0	18	413
Not‐CIWD n = 29	0	1	28	0	6	23	0	23	6
**HLA‐** **DRB1**									
**3.0.0 CIWD**	Common n = 72	WD n = 138	Not‐CWD n = 247	Common n = 42	WD n = 86	Not‐CWD n = 329	Common n = 34	WD n = 93	Not‐CWD n = 330
Common total n = 86	62	24	0	42	18	26	34	25	27
Intermediate n = 98	8	68	22	0	25	73	0	29	69
Well‐documented n = 238	2	40	196	0	37	201	0	16	222
Not‐CIWD n = 35	0	6	29	0	6	29	0	23	12
**HLA‐DRB3/4/5**									
**3.0.0 CIWD**	Common n = 14	WD n = 9	Not‐CWD n = 35	Common	WD	Not‐CWD	Common	WD	Not‐CWD
Common total n = ND	Data insufficient			Not reported			Not reported		
Intermediate n = ND	Data insufficient								
Well‐documented n = 57	14	8	35						
Not‐CIWD n = 1	0	1	0						
**HLA‐** **DQB1**									
**3.0.0 CIWD**	Common n = 19	WD n = 4	Not‐CWD n = 165	Common n = 20	WD n = 42	Not‐CWD n = 126	Common n = 15	WD n = 20	Not‐CWD n = 153
Common total n = 29	19	3	7	17	5	7	15	6	8
Intermediate n = 31	0	0	31	2	8	21	0	4	27
Well‐documented n = 119	0	1	118	1	26	92	0	4	115
Not‐CIWD n = 9	0	0	9	0	3	6	0	6	3
**HLA‐DPB1**									
**3.0.0 CIWD**	Common n = 38	WD n = 11	Not‐CWD n = 210	Common n = 27	WD n = 45	Not‐CWD n = 187	Common	WD	Not‐CWD
Common total n = 58	38	5	15	26	15	17	Not reported		
Intermediate n = 49	0	5	44	1	16	32			
Well‐documented n = 151	0	1	150	0	13	138			
Not‐CIWD n = 1	0	0	1	0	1	0			

Abbreviations: C, common; I, intermediate; WD, well‐documented; ND, not determined.

a Based on the highest frequency for any population. Supporting Information Table S3a lists the alleles and their frequency categories: HLA‐A Supporting Information Table S3a, HLA‐B 3b, HLA‐C 3c, HLA‐DRB1 3d, HLA‐DRB3/4/5 3e, HLA‐DQB1 3f, HLA‐DPB1 3g.

b Alleles observed at least five times in any population in the current dataset are included in this table. Also included are alleles that were observed in previous datasets but observed in this study less than five times. Note that this two‐field designation is the equivalent of a “P” assignment so that alleles within a P group that encode polypeptides that differ outside of the antigen recognition domain are not summed separately. Any nonexpressed alleles that are excluded from P groups are listed separately.

c Some alleles listed in earlier CWD versions are not included because they are within a P group or were condensed into a two‐field assignment.

d Not‐CWD or not‐CIWD based only on alleles categorized as CIWD observed in any catalog.

Slightly more than half (n = 1853) of the two‐field P group HLA‐B alleles of the total 3537 in IPD‐IMGT/HLA 3.31.0 are observed overall in the dataset, but only 864 (24% of total) are assigned to a CIWD category (Table 3, Figure 1). The number of HLA‐B alleles common at the two‐field level are 195 (Table 4, Supporting Information Table 3b). About 60% (119) were considered common in 2.0.0 CWD; 74 were listed as well‐documented in 2.0.0. Two (B*08:12 (AFA), B*37:04 (API)) were considered not‐CWD in 2.0.0 CWD, likely because of the limited population groups included at that time. Comparison to the EFI CWD shows that 121 of the 195 were listed as common (n = 61) or well‐documented (n = 60) and 74 were not included in the EFI CWD listing (Table 4). Comparison to the China CWD shows 114 of the 195 listed as common (n = 54) or well‐documented (n = 60) and 81 not listed. The alleles identified as common in the three earlier CWD reports are largely included in the current common listing (2.0.0 CWD 119/121; EFI CWD 61/62; China CWD 54/54).

The intermediate HLA‐B category includes 128 alleles at the P level; 80 of these were categorized in 2.0.0 CWD as common (n = 2) or well‐documented (n = 78). Of the 48 intermediate alleles not included in 2.0.0 CWD, only eight are listed by EFI CWD or China CWD (assigned as well‐documented).

HLA‐B alleles categorized as well‐documented at two‐field P level resolution total 541; 71 of them are included in 2.0.0 CWD as well‐documented. Of the 470 well‐documented alleles not included in 2.0.0 CWD, 120 are listed by EFI CWD and 13 additional alleles by China CWD as well‐documented. In total, 337 new alleles are added to the well‐documented listing.

Sixty‐five alleles listed in one or more of the previous three reports are not observed in five or more individuals in the current study. All 65 alleles were assigned as well‐documented in the earlier studies: seven in 2.0.0 CWD, 15 in EFI CWD and 46 in China CWD. For example B*40:25, listed as well‐documented in all three prior studies, was only observed once in registry databases (in EURO).

Almost 60% (57%; n = 384) of the two‐field P group HLA‐DQB1 alleles of the total 669 in IPD‐IMGT/HLA 3.31.0 are observed overall in the dataset, but only 179 (27% of total) are assigned to a CIWD category (Table 3, Figure 1). HLA‐DQB1 alleles common at the two‐field level number 29 based on the highest frequency category found in any population group (Table 4, Supporting Information Table 3f). About 66% (n = 19) were considered common in 2.0.0 CWD; three were listed as well‐documented in 2.0.0 CWD. Seven were considered not‐CWD in 2.0.0 CWD. Comparison to the EFI CWD shows that 22 of the 29 were listed as common (n = 17) or well‐documented (n = 5) and seven were not included in the EFI CWD listing (Table 4). Comparison to the China CWD shows 21 of the 29 listed as common (n = 15) or well‐documented (n = 6) and eight not listed. The alleles identified as common in the three earlier CWD reports are largely included in the current common listing (2.0.0 CWD 19/19; EFI CWD 17/20; China CWD 15/15).

The intermediate HLA‐DQB1 category includes 31 alleles at the two‐field P level; none was included in the 2.0.0 CWD. Of the 31 intermediate alleles, only 10 are listed by EFI CWD (two common, eight well‐documented) and four by China CWD (well‐documented).

HLA‐DQB1 alleles categorized as well‐documented at two‐field totaled 119: only one was included in 2.0.0 CWD as well‐documented, 27 by EFI CWD (one common, 26 well‐documented), and four by China CWD (well‐documented). In total, 91 new HLA‐DQB1 alleles were added to the well‐documented listing.

Nine alleles designated as well‐documented in early studies were not included in the 3.0.0 CIWD. For examples, DQB1*03:27, listed as well‐documented in China CWD, was observed three times in this registry study dataset (found in API), and DQB1*06:19, listed as well‐documented in EFI CWD, was observed four times (API/EURO‐2/UNK).

Similar findings are provided for HLA‐A, ‐C, ‐DRB1, and ‐DPB1 (Tables 3 and 4, Figure 1, Supporting Information Table S3a, c, d, g). HLA‐DRB3/4/5 two‐field groups are only categorized as well‐documented because of incomplete information at these loci.

## 5 CIWD HLA ALLELES AT G‐LEVEL DESIGNATION

HLA‐A and ‐DPB1 serve as examples of the G‐level assignments for the current data compared with previous catalogs.^16^, ^17^, ^18^ The analysis is based on over 14 million total assignments at each locus (Table 2b). Changes to the categorization of the G‐level assignments compared with 2.0.0 CWD^16^ are modest.

In 2.0.0 CWD, 40 HLA‐A G group alleles are common and nine are well‐documented (Supporting Information Tables S4 and S5a). The current dataset lists 45 as common (Figure 2). This list also includes ten alleles that had been not designated as a G group at the time of 2.0.0 CWD but that were considered common at that time. Five well‐documented alleles in 2.0.0 CWD (A*02:16:01G, A*11:03, A*24:05:01G, A*24:14, A*66:03) are now classified as common with greatest frequency in AFA, API, HIS, and/or NAM. Two G‐level assignments are now considered intermediate; 2.0.0 CWD listed them as well‐documented. Three G‐level assignments are now well‐documented; previously two were well‐documented and one not‐CWD (A*02:81:01G; observed now in EURO).

HLA‐DPB1 has 32 common G groups; 26 were previously designated as common in 2.0.0 CWD; three had been well‐documented, and three were not‐CWD. One of the latter is a G group (DPB1*04:01:04G) described after 2012. Three alleles are now classified as intermediate and all were previously assigned as well‐documented in 2.0.0 CWD. One G group (DPB1*69:01:01G) remains well‐documented as categorized previously.

Similar findings for HLA‐B, ‐C, ‐DRB1, and ‐DQB1 (Figure 2, Supporting Information Tables S4 and 5b, c, d, f). HLA‐DRB3/4/5 G groups are only categorized as well‐documented because of incomplete information at these loci.

## 6 CIWD HLA ALLELES AT FULL‐FIELD DESIGNATION

It was not possible to consistently determine the frequencies of the alleles reported at full field designations. The typing assignments submitted (Supporting Information Tables [Link], [Link]) illustrate the diversity of HLA assignments that arise from typing volunteer donors over time (2012‐2018) and reflect the DNA sequence‐based typing strategy, including resolution requirements, used by each registry. A good example of this is A*02:01:01:01 which is very common when individuals are typed at a consistent four‐field resolution.^23^, ^24^ In this multiple registry dataset, A*02:01:01:01 was reported only 1294 times while A*02:01:01G was reported over 3.7 million times. Over 40 (n = 43) alleles in the A*02:01:01G group are found in this dataset but individually none are more common than A*02:01:01:01 (eg, second most common A*02:01:01:05 appears 616 times in the total dataset). The total number of assignments of alleles in the G group other than A*02:01:01:01 is 1876. None of the individual alleles in the A*02:01:01G group in Europeans appear in a frequency ≥1 in 10 000. In contrast, A*02:01:01G appears at frequency of >1 in 4 (0.27) in Europeans. Therefore, most registries are typing at a resolution of A*02:01:01G so there are insufficient data to accurately assess the frequency of A*02:01:01:01 in this dataset. A similar situation is observed for other alleles.

## 7 NONEXPRESSED ALLELES

**Figure 3:**
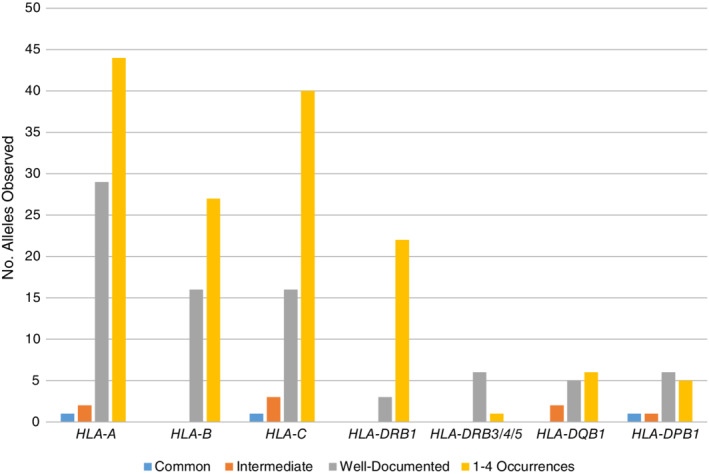
Distribution of nonexpressed HLA alleles into three frequency categories and 1‐4 occurrences at P group resolution

HLA‐A and ‐DRB1 serve as an example of the nonexpressed allele assignments for the current data compared with previous reports (Figure 3, Supporting Information Tables S6 and S7a‐g).^16^, ^17^, ^18^

Seventy‐six nonexpressed two‐field HLA‐A alleles of the total 186 in IPD‐IMGT/HLA 3.31.0 are observed overall in the dataset, but only 32 (17% of total) are assigned to a CIWD category. Most (n = 29) are well‐documented. One allele, A*23:19N, is common in AFA (Supporting Information Table S7a). Two alleles (A*02:53N and A*24:11N) are intermediate, found in the API group. The remaining 44 nonexpressed HLA‐A alleles are each observed one to four times.

In 2.0.0 CWD, 6 well‐documented and 1 common (A*02:53N) nonexpressed HLA‐A alleles are listed; six of these are assigned 3.0.0 CIWD designations. Two of these seven alleles are now classified as intermediate in API (A*02:53N, A*24:11N). One (A*68:11N) has only two entries in the present dataset. The EFI listing has no HLA‐A nonexpressed alleles listed; China CWD lists three as well‐documented: A*02:53N, A*11:69N, A*24:132N. Only A*02:53N is observed in this study.

Of the 25 nonexpressed HLA‐DRB1 alleles observed overall out of the 52 in IPD‐IMGT/HLA 3.31.0, only three are observed more than five times: DRB1*07:10N, DRB1*07:26N and DRB1*12:24N in the EURO group (Supporting Information Tables S6 and 7d). There are no HLA‐DRB1 nonexpressed alleles listed in the three earlier CWD reports.

Similar findings are observed for HLA‐B, ‐C, ‐DQB1, ‐DPB1 (Figure 3, Supporting Information Tables S6 and 7b,c, f, g). HLA‐DRB3/4/5 nonexpressed alleles are only categorized as well‐documented because of incomplete information at these loci. In 2.0.0 CWD, some of these alleles, like DRB4*01:03N (ie, DRB4*01:03:01:02N) and DRB5*01:08N, were designated as common.

## 8 DISCUSSION

This update (3.0.0 CIWD) with over 8 million individuals from 20 worldwide registries represents an enormous increase in the information used to assess HLA frequencies. It is based solely on HLA assignments collected by DNA sequencing covering the time period 2012‐2018. While the number of individuals assessed in the 2012 version of CWD alleles (2.0.0 CWD) was not described, the data from some of the loci evaluated for the EFI and China CWD catalogs derived from less than 1 million individuals (Supporting Information Table S1). Further, two of the earlier reports (2.0.0 and EFI CWD) relied partly on HLA assignments derived from more resolution‐limited DNA‐based typing methods (eg, probe hybridization or sequence‐specific primer typing) collected over earlier time periods. (Note: Advances supporting cost‐effective high volume DNA sequencing and the efforts of registries to grow and diversify their volunteer donor pool have made this CIWD effort possible).

### 8.1 Population groups

Worldwide population diversity is increasing. In the United States, for example, 13% of individuals were born in another country and 25% of individuals list multiple ancestries (https://www.census.gov, October 2018). In Sweden, 25% of the population was either born abroad or had two parents born abroad (https://www.scb.se/en/, 2018). Immigrants represent large portions of the country's population in Saudi Arabia (37%) and Singapore (46%). (http://www.un.org/en/development/desa/population/migration/data/estimates2/data/UN_MigrantStockTotal_2017.xlsx). For this reason, access to worldwide HLA allele frequencies is essential to serve diverse population groups. It should be noted, however, while the allele frequencies for individual population groups are provided here, caution should be observed. Self‐identification of ancestry does not necessarily reflect genetic ancestry.^25^ In cases where the ancestry of a patient or donor may not be known, the laboratory might, for example, rely on the highest frequency CIWD listings to determine if further typing resolution is needed.

The sources of data used for this and the three earlier catalogs differ dramatically although all included some evaluation of unrelated hematopoietic stem cell donors (Supporting Information Table S1). This includes notable overlap of approximately 3.6 million (68% of 5.3 million submitted) donors from DKMS Germany present in the EFI catalog and this analysis. The 3.0.0 CIWD includes only individuals recruited as volunteer donors in hematopoietic stem cell registries. The 20 contributing registries are localized in five of the six World Health Organization geographic regions (Table 1). The African Region is not represented per se, but individuals of recent African ancestry are found within some of the submitted registry data. While the population analyzed is more “global” in nature, clearly future efforts need to be focused on geographic regions under‐represented in this analysis, specifically Africa because of its high genetic diversity^26^ and Asia because it is home to almost 60% of the world's population https://population.un.org/wpp/Publications/Files/WPP2019_Highlights.pdf (October 2019).

### 8.2 Use of CIWD categories

The previous division of alleles into three categories, common, well‐documented and not‐CWD, needed to be reevaluated. In the past, there was a concern that some alleles were either very rare or represented erroneous sequences. For this reason, the well‐documented category captured less common alleles but those confirmed as “real” by several (3‐5) independent DNA sequences. With the inclusion of large international registry data of consecutive donor recruitment and with the focus only on alleles identified by DNA sequencing, it is now possible to estimate the frequency of HLA alleles.

While retaining and expanding the category designations, we suggest that common alleles include those present at ≥1 in 10 000. This broader definition of common in 3.0.0 CIWD captures almost all the alleles designated as common in the three earlier studies (classified as common in 3.0.0 CIWD: 92.0% 2.0.0 CWD, 96% EFI CWD, 100% China CWD; classified as intermediate in 3.0.0 CIWD: 4% 2.0.0 CWD, 2% EFI CWD).

Basing the second frequency category on five occurrences is unrealistic in terms of selecting a set of alleles that should be routinely discriminated by typing reagents. With this criterion, the well‐documented category would include, for example, 669 two‐field P group HLA‐B alleles. Thus, we have introduced a new category, intermediate, capturing alleles present at ≥1 in 100 000 (n = 128 for HLA‐B). We suggest that the intermediate category be included with the common category when typing reagents are developed and/or further testing is required to resolve alternative genotypes. Unfortunately, three groups (AFA, MENA, NAM) did not have sufficient numbers to use the intermediate category, so more focused testing is needed to obtain a better estimate of frequency in these groups. For these three groups, to be conservative, well‐documented alleles might be included when further testing is required to resolve ambiguity until better frequency estimates are obtained.

Well‐documented has been reserved for alleles that are not in the common and intermediate categories but that appear five or more times and are independent of sample size. The well‐documented (n = 541 for HLA‐B P group; n = 2 for HLA‐B G group) and not‐CIWD categories might be used to trigger repeat and/or extended testing when assigned as an unambiguous genotype or when all alternative genotypes include these two categories.

The 3.0.0 CIWD listing captures all of common alleles listed by 2.0.0 CWD, EFI CWD and China CWD within its three frequency categories. Overall, 3.0.0 CIWD includes all but 3% of 2.0.0 CWD well‐documented alleles, 5% of the EFI CWD well‐documented alleles, and 32% of the China CWD well‐documented alleles. Thus, 3.0.0 CIWD bridges the 2.0.0 CWD and EFI catalogs while the China CWD catalog continues to represent a distinct collection of alleles. Future global catalogs should increase their focus on the WHO Western Pacific region.

### 8.3 Resolution

The focus on two‐field P‐level resolution HLA assignments and G resolution assignments is guided by the understanding that protein coding variation within the ARD of the HLA molecules impacts antigenic peptide binding and interactions with T cell receptors and killer immunoglobulin‐like receptors on natural killer cells.^27^, ^28^, ^29^, ^30^ There is no strong evidence that variation outside of these regions alone, with the exception of variation resulting in nonexpressed alleles, plays a role in transplant outcome and disease associations. Recent analyses have focused on potential impact of mismatching patient and donor at noncoding regions and regions outside the ARD, although, because the number of evaluable cases has been small, further investigation is needed.^31^, ^32^, ^33^ While resolution of variation outside the ARD within hematopoietic stem cell registry volunteers allows a more refined analysis of HLA diversity, this level of typing is not commonly used for matching donor and recipient.

When assessing the matching between donor and recipient, an allele may be identified by the laboratory that appears to encode an HLA protein mismatch but is not listed in a CIWD table. Review of the primary CIWD data provided in Supporting Information Tables [Link], [Link] will determine if the allele was observed in the 3.0.0 CIWD dataset. Note that some alleles once thought to be common like A*02:17:01 and B*47:01:01:01 have been deleted from the IPD‐IMGT/HLA database and are no longer listed (http://hla.alleles.org/nomenclature/index.html). Also note that the alleles within a G group are clustered together in the Supporting Information tables so do not appear in numerical order in the tables. If listed in Supporting Information Tables [Link], [Link], it is possible that the allele lies within a P group and so is not listed in Supporting Information Tables S3a‐g. Two examples are C*02:10 which is common and C*14:11 which is intermediate; neither is listed in Supporting Information Table 3c. Both alleles exhibit nucleotide sequence variation in the exons encoding the ARD but are “hidden” within the C*02:02P and C*14:02P groups.

### 8.4 Frequency of alleles within G groups that encode different proteins

Without knowing the extent of the gene sequenced when performing a typing, it is difficult to evaluate the frequency of each allele within a G assignment that encodes a different protein. For example, the A*02:49:01G group includes A*02:49 and A*02:683. Because A*02:683 was first assigned in 2017, A*02:49 was likely assigned based on the sequence of the ARD‐encoding exons only. Thus, there is very little information on how often A*02:683 is present. In a second example, A*02:22:01G includes A*02:22:01:01, A*02:22:01:02, and A*02:104. A*02:22:01 was extended to a fourth field in 2017 but A*02:104 was first described in 2006. In this case, because assignments of A*02:22:01 could have resulted from a failure to consistently include the G when typing for the ARD‐encoding exons only, we cannot be confident that the true frequency of A*02:104 can be evaluated.

However, when the assignment of the primary allele in the G group is at four‐field resolution, a frequency comparison between alleles in the G cluster might be made. This is the case for A*02:05:01G where assignments include A*02:05:01:01 (Supporting Information Table S8). In this case, when the non‐ARD exons are being evaluated, the frequency of G group alleles A*02:179 and A*02:324 compared with that of A*02:05:01:01 can be predicted. As in the case for most G groups,^23^, ^24^ the primary allele is observed most frequently (A*02:05:01:01 appears 53 times in AFA) and secondary alleles at lower frequency (A*02:179 appears 16 times in this group and A*02:324 is not observed). A*02:16:01G (A*02:16, A*02:131) is another example of where the comparison can be made because there are more reports of a secondary allele A*02:131 (n = 125) than the primary allele giving the G group its name (A*02:16, n = 2) in API.

### 8.5 Nonexpressed alleles

The definition of high‐resolution testing requires excluding all known nonexpressed alleles.^34^ Presently there are 464 class I and 124 HLA‐DRB1, ‐DRB3, ‐DRB4, ‐DRB5, ‐DQB1, ‐DPB1 nonexpressed alleles (IPD‐IMGT/HLA version 3.31.0) described. Excluding all nonexpressed alleles that differ outside of the ARD‐encoding domains may require additional DNA testing or a serological assay for expression. An alternative is to use allele frequencies and/or known haplotypes to determine which of the nonexpressed alleles to consider in the testing protocol. Unfortunately, frequency data of null alleles with key variation outside the ARD can be limited and data on haplotypes insufficient for diverse population groups.

The assignment of A*01:01:01G includes the nonexpressed alleles A*01:04:01:01N and A*01:04:01:02N (often listed as A*01:04N in registry databases) together with the very common A*01:01:01:01. The lack of expression results from an insertion in exon 4 in the two nonexpressed alleles which might not be readily tested by the laboratory's routine assays. A*01:04N is observed a total of 6 times in 5.6 million EURO individuals (ie, well‐documented) and not observed in any other registry group, so this frequency might justify a decision to assume these nonexpressed alleles are not present without further testing. However, it is unclear how often exon 4 was truly evaluated by the registries' testing laboratories and whether an accurate estimate of A*01:04N frequency is known. When nonexpressed alleles appear in a specific haplotype,^19^ this can be used to decide on further testing although there are exceptions to usual haplotype associations.

Alleles where variation potentially impacting protein expression has been described but not yet documented by further studies (ie, alleles with a Q designation) total 14 in the CIWD category. Three are intermediate in API (A*32:11Q, C*15:32Q) and EURO (DQB1*02:53Q) and the remainder well‐documented. Because the variations in the three intermediate alleles impact the amino acid sequences of the ARD, in addition to their questionable impact on protein expression, the lack of information on expression should not hinder decisions regarding their relevance in matching. Other, likely rare, Q alleles may carry their key variation outside of the ARD‐encoding exons and it will be difficult to assess matching without clarification of the impact on protein expression.

### 8.6 IMGT/HLA designation of CIWD to extended allele names

In the IPD‐IMGT/HLA database,^11^ the CWD status of alleles has been updated as additional fields are added to the allele name. For example, A*24:20 in the 2.0.0 CWD is categorized as common. As that allele name became extended to include additional fields, the IPD‐IMGT/HLA database assigned the allele with the 01 field(s) (A*24:20:01:01) as common and A*24:20:01:02 as not defined. In some cases, there is, at present, insufficient typing assignments at the four‐field level to determine whether, for example, A*24:20:01:01 is common or not. In the case of common A*30:02:01:01, two other alleles with the same truncated designation in 2.0.0 CWD (A*30:02:01) are also common (A*30:02:01:02, A*30:02:01:03). In the case of A*80:01:01:01, that allele is not common while A*80:01:01:02 is the more common variant. Thus, the IPD‐IMGT/HLA assignments for alleles with longer names than those reported in the prior versions of CIWD need further revision based on the data provided here.

### 8.7 Limitations of the catalog

Although this analysis represents the largest catalog of the frequency of alleles to date, the data are heavily represented from registries in the United States and Germany. This can influence the overall frequency of different alleles outside of what the actual frequency is in the broad geographic/ancestral/ethnic categories. In addition, many parts of the world are not represented by data or include only a small relative number. However, this also provides an opportunity for individual countries, donor registries or individual studies of HLA diversity to compare the HLA data with this global catalog to point out differences at a more local level. Ancestry reporting is performed by many registries at the time of donor recruitment via self‐identification, but is not implemented at some registries which results in a large population group in the unknown category in this analysis. Nomenclature that includes three and four fields accompanies differential practices among registries, both in typing methodology and standardization among testing of donors joining a registry. For this study, we focused on two‐field P‐level resolution summary of nomenclature but could not provide an accurate assessment of frequency among donors that were typed, for example, at four fields, as we could not be confident in the denominator for such assessment. HLA‐DQA1 and ‐DPA1 loci did not have sufficient submission of data, so although not summarized for this study, could offer another phase for the 18th International HLA and Immunogenetics Workshop study. Lastly, the organization and submission of these data focused on alleles, so is not able to provide the context of haplotype data, but again offers additional opportunities for study.

## 9 CONCLUSION

It has been suggested that mutation acts to create a pool of rare alleles that might become important in the defense against a new or evolving pathogen and, therefore, be selected to become more frequent.^35^ The characteristics of variation at the HLA class I loci including alleles that have likely arisen by single point mutations has been described in detail based on the sequences of the alleles submitted to the IPD‐IMGT/HLA database.^36^ Estimates have been made about the total number of HLA alleles (eg, 8‐9 million class I alleles) that would be found^36^ if we were able to sequence the HLA genes from the 7.7 billion humans on earth (https://population.un.org/wpp/, July 2019). The frequency of HLA alleles in different populations reflects that population's evolutionary history^9^ and we see the impact of that history today in the CIWD allele frequencies.

We offer the most comprehensive and collaborative CWD catalog to date through the efforts of the 18th International HLA and Immunogenetics Workshop and the World Marrow Donor Association participating registries. From a catalog that originated to help guide proficiency testing of laboratories to a critical tool for testing development and laboratory clinical decisions and registry policy, we believe the data provided here will continue to be instrumental in the understanding and use of HLA in its many practices for health and research.

## CONFLICT OF INTEREST

Hurley has been a histocompatibility advisor for the NMDP. Georgetown University has filed a patent application on which Hurley is an inventor of the HLA typing and Sanger‐based sequencing technology.

## Supporting information


**Table S1** Comparison of criteria, populations and analysis methods used for the designation of allele frequency categories

**Table S2** Possible DNA‐based HLA assignments for individual who carries the allele HLA‐B*07:02:01:01

**Table S3a‐g** Comparison of HLA P two‐field assignments by locus and allele among CIWD catalogs

**Table S4** Overall comparison of HLA G group assignments among CIWD catalogs

**Table S5a‐g** HLA G level frequencies between CIWD versions

**Table S6** Overall summary of nonexpressed HLA allele assignments

**Table S7a‐g** CIWD status of observed nonexpressed HLA two‐field allele assignments by locus

**Table S8** HLA‐A primary data

**Table S9** HLA‐B primary data

**Table S10** HLA‐C primary data

**Table S11** HLA‐DRB1 primary data

**Table S12** HLA‐DRB3 primary data

**Table S13** HLA‐DRB4 primary data

**Table S14** HLA‐DRB5 primary data

**Table S15** HLA‐DQB1 primary data

**Table S16** HLA‐DPB1 primary data

## Data Availability

The data that support the findings of this study are available in the Supporting Information of this article and will also be posted on the web site of the 18th International HLA & Immunogenetics Workshop (https://www.ihiw18.org/). (Please note that Supporting Information Tables [Link], [Link] should be printed with caution, as they are very large tables).
