# Supplementary material for: Common, intermediate and well‐documented HLA alleles in world populations: CIWD version 3.0.0
Source: HLA. 2020 Jan 31;95(6):516–31. doi: 10.1111/tan.13811 (PMC7317522; doi:10.1111/tan.13811)
Supplement: Supplementary file 1 — Table S1 Comparison of criteria, populations and analysis methods used for the designation of allele frequency categories [file TAN-95-516-s001.docx]

**Supplementary Table 1. Comparison of criteria, populations and analysis methods used for the designation of allele frequency categories**

| **Reference** | **Common** | **Intermediate** | **Well-Documented** | **Population Groups Included** | **Loci /Assignments Included** | **Criteria** |
| --- | --- | --- | --- | --- | --- | --- |
| 3.0.0 CIWD  (this study) | >1/10,000 in any population group or in total population | >1 in 100,000 and <1/10,000 in any population group or in total population | Observed at least 5 times in any population group or in total population | 20 world-wide unrelated hematopoietic stem cell donor registries  8,077,802 total individuals; most contributing to each locus | *A, B, C, DRB1, DRB3/4/5, DQB1, DPB1*  Summary P group and summary G group resolutions as described in Supplementary Table 2 | Allele counting  Defined by DNA sequencing |
| - 1. CWD   Mack et al., 2013 | Observed in multiple populations  Ample data supporting their presence and frequency  Observed at frequencies >1 in 1000 in reference populations of at least 1500 individuals | Not applicable | Not as widely distributed as common alleles  Frequencies less certain than common alleles  Observed five times in unrelated individuals by SBT method or three times by SBT in a specific haplotype in unrelated individuals | Published studies of specific populations, Allele Frequency Net Database, NMDP rare allele list, and individual laboratory reports  139,961 SBT observations used to identify WD alleles  Total number individuals evaluated not described | *A, B, C, DRB1, DRB3/4/5, DQA1, DQB1, DPB1*  Fields varied from 2-4 based on IMGT/HLA database at the time of analysis; G groups | Allele counting, estimation  Defined by variety of DNA typing methods |
| EFI CWD  Sanchez-Mazas et al., 2017 | More than 3 copies observed in at least 3 different populations | Not applicable | At least 5 copies in the total set of populations | HLA-net, DKMS unrelated hematopoietic stem cell donors, Allele Frequency Net Database  639,416 (*HLA-A*)-3,966,984 (*HLA-DRB1*) individuals depending on locus | *A, B, C, DRB1, DQA1, DQB1, DPB1*  Two field | Allele counting, estimation  Defined by variety of DNA typing methods |
| China CWD  He et al., 2018 | >1 in 1000 in reference populations of at least 1500 individuals | Not applicable | Observed five times in unrelated individuals by SBT method or three times in a specific haplotype in unrelated individuals | China unrelated hematopoietic stem cell donor registry, 31 provinces  539,130 (*HLA-C, -DQB1*)-812,211 (*HLA-A, -B, -DRB1*) individuals | *A, B, C, DRB1, DQB1*  Two field | Allele counting  Defined by DNA sequencing |
| Eberhard et al., 2018 | > 1 in 1000 | Not applicable | WD1: Observed with a frequency of at least 1 in 100,000  WD2: Alleles falling into both categories WD3 and WD4  WD3: Observed at least five times  WD4:Observed three times in a specific haplotype | German unrelated hematopoietic stem cell donor registry  5,104,477 individuals | *A, B, C, DRB1, DQB1, DPB1*  Two field | Data based on imputation  Defined by variety of DNA typing methods |

References

Eberhard HP, Schmidt AH, Mytilineos J, Fleischhauer K, Muller CR: Common and well-documented HLA alleles of German stem cell donors by haplotype frequency estimation. HLA 92:206-214, 2018.

He Y, Li J, Mao W, Zhang D, Liu M, Shan X, Zhang B, Zhu C, Shen J, Deng Z, Wang Z, Yu W, Chen Q, Guo W, Su P, Lv R, Li G, Li G, Pei B, Jiao L, Shen G, Liu Y, Feng Z, Su Y, Xie Y, Di W, Liu X, Yang X, Wang J, Qi J, Liu Q, Han Y, He J, Cai J, Zhang Z, Zhu F, Du D: HLA common and well-documented alleles in China. HLA 92:199-205, 2018.

Mack SJ, Cano P, Hollenbach JA, He J, Hurley CK, Middleton D, Moraes ME, Pereira SE, Kempenich JH, Reed EF, Setterholm M, Smith AG, Tilanus MG, Torres M, Varney MD, Voorter CE, Fischer GF, Fleischhauer K, Goodridge D, Klitz W, Little AM, Maiers M, Marsh SG, Muller CR, Noreen H, Rozemuller EH, Sanchez-Mazas A, Senitzer D, Trachtenberg E, Fernandez-Vina M: Common and well-documented HLA alleles: 2012 update to the CWD catalogue. Tissue Antigens 81:194-203, 2013.

Sanchez-Mazas A, Nunes JM, Middleton D, Sauter J, Buhler S, McCabe A, Hofmann J, Baier DM, Schmidt AH, Nicoloso G, Andreani M, Grubic Z, Tiercy JM, Fleischhauer K: Common and well-documented HLA alleles over all of Europe and within European sub-regions: A catalogue from the European Federation for Immunogenetics. HLA 89:104-113, 2017.
