# Supplementary material for: Common, intermediate and well‐documented HLA alleles in world populations: CIWD version 3.0.0
Source: HLA. 2020 Jan 31;95(6):516–31. doi: 10.1111/tan.13811 (PMC7317522; doi:10.1111/tan.13811)
Supplement: Supplementary file 2 — Table S2 Possible DNA‐based HLA assignments for individual who carries the allele HLA‐B*07:02:01:01 [file TAN-95-516-s002.docx]

**Supplementary Table 2. Resolution and merging of DNA-based HLA assignments using, as an example, an individual who carries the allele HLA-B*07:02:01:01^a^**

| Resolution | Assignment | Comments |
| --- | --- | --- |
| Primary data received from registries (Supplementary Tables 8-16) | | |
| Allele | B*07:02:01:01 | A World Health Organization (WHO)-defined designation (Marsh et al., 2010), alleles may be designated with 2-4 field nomenclature depending on how much of the allele has been sequenced and whether other similar alleles have been reported. |
| Three field resolution (i.e., truncated name) | B*07:02:01 | Formally, based on WHO HLA nomenclature guidelines, all alleles with the same digits in fields 1-3 are included. This would exclude alleles that are identical in nucleotide sequence in the antigen recognition domain (ARD) exons but that differ in fields 2 or 3 in their allele name.  In practice, based on typing data submitted to a registry, it is likely that the assignment B*07:02:01 is based on the sequence of the exons encoding the ARD and so is considered equivalent to B*07:02:01G. |
| Two field resolution (i.e., truncated name) | B*07:02 | Technically, this category should include all alleles with names that begin with these two fields.  For the consolidation of the registry assignments in this dataset, this assignment is considered equivalent to B*07:02P. |
| Intermediate resolution^b^ | B*07:CODE | A subset of the B*07 alleles. The subset varies depending on the multiple allele code assigned (Bochtler et al., 2012; <https://hml.nmdp.org/MacUI/> (February 2019)). In this dataset, multiple allele codes that include only alleles within a G group were designed as B*07:02:01G. All other allele subsets were merged into this category. |
| G group resolution | B*07:02:01G | A WHO-defined designation (Marsh et al., 2010), the G designation includes all alleles that have the same nucleotide sequence in the ARD-encoding exons. |
| P group resolution | B*07:02P | A World Health Organization (WHO)-defined designation (Marsh et al., 2010), the P signifies alleles that encode the same protein sequence in the ARD. Non-expressed alleles are excluded. The P group is expressed with two fields.  In practice, this designation could be applied to ambiguous results that include any of the alleles encoding the same protein sequence in the ARD or to a single allele. |
| Analyzed data presented in the manuscript and in Supplementary Tables 3-7 | | |
| Summary G group resolution^b^ | B*07:02:01G total | In the dataset, this category will include assignments of B*07:02:01G and assignments with higher resolution that are included within the B*07:02:01G group e.g., B*07:02:01:01. The summary G group information is used in the manuscript text and tables. |
| Summary P group resolution^b^ | B*07:02P total | In the 3.0.0 CIWD catalog, this is a summary of all P, G and allele designations that would encode the same protein sequence in the ARD. Any non-expressed alleles are not included. The summary P group information is used in the manuscript text and tables. |

^a^ Nomenclature for HLA is described at <http://hla.alleles.org/nomenclature/index.html> (October 2018) (Marsh et al., 2010; Robinson et al., 2015). DNA-based nomenclature has a potential of four fields separated by colons. The first field is related to the serologic assignment / allele family (Holdsworth et al., 2009). The second field refers to nonsynonymous variation causing a change in the amino acid sequence of the HLA protein. The third field refers to synonymous variation in the exons. The fourth field describes intron and 5’ and 3’ untranslated region variation. Antigen recognition domain (ARD)-encoding exons are exons 2 and 3 for class I genes and exon 2 for class II.

^b^ These assignments in the primary data Supplementary Tables 8-16 represent the merger of all HLA assignments that fit within this category.

**References**

Bochtler W, Maiers M, Bakker JN, Baier DM, Hofmann JA, Pingel J, Rist HG, Oudshoorn M, Marsh SG, Muller CR, Hurley CK: An update to the HLA Nomenclature Guidelines of the World Marrow Donor Association, 2012. Bone Marrow Transplant 48:1387-1388, 2013.

Holdsworth R, Hurley CK, Marsh SG, Lau M, Noreen HJ, Kempenich JH, Setterholm M, Maiers M: The HLA dictionary 2008: a summary of HLA-A, -B, -C, -DRB1/3/4/5, and -DQB1 alleles and their association with serologically defined HLA-A, -B, -C, -DR, and -DQ antigens. Tissue Antigens 73:95-170, 2009.

Marsh SG, Albert ED, Bodmer WF, Bontrop RE, Dupont B, Erlich HA, Fernandez-Vina M, Geraghty DE, Holdsworth R, Hurley CK, Lau M, Lee KW, Mach B, Maiers M, Mayr WR, Muller CR, Parham P, Petersdorf EW, Sasazuki T, Strominger JL, Svejgaard A, Terasaki PI, Tiercy JM, Trowsdale J: Nomenclature for factors of the HLA system, 2010. Tissue Antigens 75:291-455, 2010.

Robinson J, Halliwell JA, Hayhurst JD, Flicek P, Parham P, Marsh SG: The IPD and IMGT/HLA database: allele variant databases. Nucleic Acids Res 43:D423-D431, 2015.
