# Supplementary material for: Common, intermediate and well‐documented HLA alleles in world populations: CIWD version 3.0.0
Source: HLA. 2020 Jan 31;95(6):516–31. doi: 10.1111/tan.13811 (PMC7317522; doi:10.1111/tan.13811)
Supplement: Supplementary file 3 — Table S3a‐g Comparison of HLA P two‐field assignments by locus and allele among CIWD catalogs [file TAN-95-516-s003.pdf]

Supplementary Table 3a. Comparison of HLA-A P group two-field assignments among CIWD catalogs [a]

		3.0.0 CIWD									2.0.0 CWD		EFI CWD	China CWD
		Category by Population[b]												
HLA-A IPD-HLA/IMGT 3.31.0	Nomenclature Designation [a]	Highest Frequency	Total	AFA	API	EURO	MENA	HIS	NAM	UNK	HLA-A IPD-IMGT/HLA 3.9.0	Category	Category	Category
A*01:01	P	C	C	C	C	C	C	C	C	C	A*01:01	C	C	C
A*01:02	P	C	C	C	I	C	C	C	C	C	A*01:02	C	C	
A*01:03	P	C	C	C	C	C	C	C	WD	C	A*01:03	C	C	WD
A*01:04N [c]	N	WD	WD			WD					A*01:04N	WD		
A*01:06	P	C	I			I	C	I		I	A*01:06	WD	WD	
A*01:09	P	C	WD	C		WD				WD	A*01:09	WD	WD	
A*01:12	P	I	WD		I						A*01:12	WD		
A*01:14	P	WD	WD			WD					A*01:14			
A*01:15N	N	WD	WD			WD					A*01:15N			
A*01:16N	N	WD	WD			WD				WD	A*01:16N			
A*01:17	P	WD	WD			WD				WD	A*01:17	WD	WD	
A*01:23	P	WD	WD	WD		WD	WD			WD	A*01:23			
A*01:24	P	WD	WD					WD			A*01:24			
A*01:25	P	I	I	WD		I				I	A*01:25	WD	WD	
A*01:26	P	I	WD		I	WD					A*01:26		WD	
A*01:29	P	WD	WD			WD					A*01:29			
A*01:30	P	WD	WD								A*01:30			
A*01:38	P	WD	WD			WD					A*01:38		WD	
A*01:39	P	WD	WD			WD					A*01:39		WD	
A*01:40	P	WD	WD			WD					A*01:40		WD	
A*01:41	P	o [d]	o								A*01:41		WD	
A*01:42	P	WD	WD			WD					A*01:42			
A*01:43	P	I	WD		I						A*01:43			
A*01:44	P	WD	WD			WD					A*01:44			
A*01:46	P	WD	WD							WD	A*01:46			
A*01:48	P	WD	WD							WD	A*01:48			
A*01:51	P	WD	WD			WD					A*01:51			

Supplementary Table 3a. Comparison of HLA-A P group two-field assignments among CIWD catalogs [a]

		3.0.0 CIWD									2.0.0 CWD		EFI CWD	China CWD
		Category by Population[b]												
HLA-A IPD-HLA/IMGT 3.31.0	Nomenclature Designation [a]	Highest Frequency	Total	AFA	API	EURO	MENA	HIS	NAM	UNK	HLA-A IPD-IMGT/HLA 3.9.0	Category	Category	Category
A*01:57N	N	WD	WD			WD					A*01:57N			
A*01:61	P	WD	WD			WD					A*01:61			
A*01:67	P	WD	WD							WD	A*01:67			
A*01:68	P	WD	WD			WD					A*01:68			
A*01:69	P	WD	WD			WD		WD			A*01:69			
A*01:72	P	WD	WD			WD					A*01:72			
A*01:74	P	WD	WD		WD						A*01:74			
A*01:78	P	o	o								A*01:78			WD
A*01:82	P	I	WD		I						A*01:82			
A*01:86	P	WD	WD			WD				WD	A*01:86			
A*01:92	P	WD	WD			WD					A*01:92			
A*01:98	P	WD	WD			WD					A*01:98			
A*01:100	P	WD	WD			WD					A*01:100			
A*01:104	P	WD	WD		WD						A*01:104			
A*01:113	P	WD	WD			WD					A*01:113			
A*01:123N	N	WD	WD			WD					A*01:123N			
A*01:126	P	WD	WD								A*01:126			
A*01:156	P	WD	WD								A*01:156			
A*01:190	P	WD	WD			WD					A*01:190			
A*02:01	P	C	C	C	C	C	C	C	C	C	A*02:01	C	C	C
A*02:02	P	C	C	C	C	C	C	C	C	C	A*02:02	C	C	WD
A*02:03	P	C	C	C	C	I	C	C	C	C	A*02:03	C	WD	C
A*02:04	P	C	C	C	WD	I	WD	C	C	C	A*02:04	C	WD	
A*02:05	P	C	C	C	C	C	C	C	C	C	A*02:05	C	C	C
A*02:06	P	C	C	C	C	C	C	C	C	C	A*02:06	C	WD	C
A*02:07	P	C	C	WD	C	C	C	C	C	C	A*02:07	C	WD	C
A*02:08	P	C	C	WD	C	C	C	I		I	A*02:08	C		WD

Supplementary Table 3a. Comparison of HLA-A P group two-field assignments among CIWD catalogs [a]

		3.0.0 CIWD									2.0.0 CWD		EFI CWD	China CWD
		Category by Population[b]												
HLA-A IPD-HLA/IMGT 3.31.0	Nomenclature Designation [a]	Highest Frequency	Total	AFA	API	EURO	MENA	HIS	NAM	UNK	HLA-A IPD-IMGT/HLA 3.9.0	Category	Category	Category
A*02:10	P	C	I		C	WD				I	A*02:10	C		C
A*02:11	P	C	C	C	C	C	C	C	C	C	A*02:11	C	WD	WD
A*02:12	P	I	I		I	I	WD	I		I	A*02:12	WD		WD
A*02:13	P	C	I	WD	WD	I		C	C	C	A*02:13	WD		
A*02:14	P	C	I	C		WD	C	I		I	A*02:14	C		
A*02:16	P	C	C	WD	C	WD	WD			I	A*02:16	WD		
A*02:17	P	C	C	C	I	C	C	C	C	C	A*02:17	C	WD	WD
A*02:18	P	WD	WD							WD	A*02:18	WD		
A*02:19	P	I	I			WD		I		I	A*02:19	WD		
A*02:20	P	C	C	WD	C	C	C	C	C	C	A*02:20	C	WD	WD
A*02:21	P	WD	WD			WD					A*02:21	WD		
A*02:22	P	C	C	C	I	C	C	C	C	C	A*02:22	C		
A*02:24	P	I	I	WD		I		I		I	A*02:24	C		WD
A*02:25	P	o	o								A*02:25	WD		
A*02:26	P	WD	WD			WD				WD	A*02:26			
A*02:27	P	I	I		WD	I				WD	A*02:27	WD		
A*02:28	P	WD	WD		WD						A*02:28			WD
A*02:29	P	I	I			I		WD		I	A*02:29	WD		
A*02:30	P	C	C	WD	I	C	WD	I		C	A*02:30	C		
A*02:31	P	WD	WD			WD					A*02:31			
A*02:33	P	C	I			WD		C	C	I	A*02:33	WD		
A*02:34	P	WD	WD			WD				WD	A*02:34	WD		
A*02:35	P	C	C	WD	I	C	WD			I	A*02:35	WD		
A*02:36	P	WD	WD			WD				WD	A*02:36	WD		WD
A*02:37	P	WD	WD			WD					A*02:37			
A*02:38	P	I	I			I				WD	A*02:38	WD		
A*02:39	P	I	WD					I		WD	A*02:39	WD		

Supplementary Table 3a. Comparison of HLA-A P group two-field assignments among CIWD catalogs [a]

		3.0.0 CIWD									2.0.0 CWD		EFI CWD	China CWD
		Category by Population[b]												
HLA-A IPD-HLA/IMGT 3.31.0	Nomenclature Designation [a]	Highest Frequency	Total	AFA	API	EURO	MENA	HIS	NAM	UNK	HLA-A IPD-IMGT/HLA 3.9.0	Category	Category	Category
A*02:40	P	WD	WD			WD					A*02:40			
A*02:42	P	WD	WD		WD						A*02:42			WD
A*02:44	P	I	WD			WD		I		I	A*02:44	WD		
A*02:45	P	I	WD			WD		I			A*02:45	WD		
A*02:46	P	WD	WD			WD					A*02:46			
A*02:47	P	WD	WD								A*02:47			
A*02:48	P	WD	WD		WD						A*02:48			WD
A*02:49	P	WD	WD			WD	WD			WD	A*02:49	WD		
A*02:51	P	WD	WD								A*02:51			
A*02:52	P	I	WD			WD		I		I	A*02:52			
A*02:53N	N	I	WD		I					WD	A*02:53N	C		WD
A*02:56	P	I	WD			I					A*02:56			
A*02:57	P	WD	WD					WD			A*02:57			
A*02:58	P	I	WD					I			A*02:58	WD		
A*02:59	P	WD	WD			WD					A*02:59			
A*02:60	P	C	I	C		WD		I		I	A*02:60	C		
A*02:63	P	WD	WD	WD							A*02:63	WD		
A*02:64	P	C	I		I	WD	WD	I	C	I	A*02:64	WD	WD	
A*02:67	P	WD	WD			WD					A*02:67		WD	
A*02:74	P	WD	WD			WD					A*02:74	WD	WD	
A*02:76	P	WD	WD			WD					A*02:76		WD	
A*02:77	P	WD	WD			WD					A*02:77			
A*02:79	P	o	o								A*02:79			WD
A*02:80	P	o	o								A*02:80			WD
A*02:81	P	WD	WD			WD					A*02:81			
A*02:83N	N	WD	WD			WD					A*02:83N			
A*02:84	P	WD	WD	WD					WD	WD	A*02:84	WD		

Supplementary Table 3a. Comparison of HLA-A P group two-field assignments among CIWD catalogs [a]

		3.0.0 CIWD									2.0.0 CWD		EFI CWD	China CWD
		Category by Population[b]												
HLA-A IPD-HLA/IMGT 3.31.0	Nomenclature Designation [a]	Highest Frequency	Total	AFA	API	EURO	MENA	HIS	NAM	UNK	HLA-A IPD-IMGT/HLA 3.9.0	Category	Category	Category
A*02:85	P	C	I	WD		WD	C			I	A*02:85	WD	WD	
A*02:86	P	WD	WD	WD		WD				WD	A*02:86	WD		
A*02:87	P	I	WD			WD		I		WD	A*02:87	WD	WD	
A*02:90	P	WD	WD			WD					A*02:90		WD	WD
A*02:93	P	C	WD			WD	C			WD	A*02:93	WD	WD	WD
A*02:94N	N	WD	WD			WD					A*02:94N			
A*02:95	P	WD	WD			WD					A*02:95		WD	
A*02:96	P	I	WD			WD		WD		I	A*02:96	WD	WD	
A*02:99	P	o	o								A*02:99			WD
A*02:101	P	WD	WD			WD					A*02:101			
A*02:102	P	I	WD				WD			I	A*02:102			
A*02:105	P	WD	WD			WD					A*02:105			
A*02:107	P	WD	WD			WD				WD	A*02:107		WD	
A*02:108	P	o	o								A*02:108			WD
A*02:109	P	WD	WD			WD					A*02:109			
A*02:110	P	WD	WD			WD	WD				A*02:110			
A*02:113N	N	WD	WD								A*02:113N			
A*02:114	P	WD	WD								A*02:114			
A*02:118	P	WD	WD			WD					A*02:118			
A*02:119	P	WD	WD			WD					A*02:119	WD		
A*02:120	P	WD	WD			WD					A*02:120			
A*02:121	P	WD	WD			WD					A*02:121		WD	WD
A*02:122	P	WD	WD	WD							A*02:122	WD		
A*02:123	P	WD	WD			WD				WD	A*02:123	WD		
A*02:125N	N	WD	WD			WD					A*02:125N			
A*02:128	P	WD	WD		WD						A*02:128			
A*02:133	P	WD	WD			WD					A*02:133		WD	

Supplementary Table 3a. Comparison of HLA-A P group two-field assignments among CIWD catalogs [a]

		3.0.0 CIWD									2.0.0 CWD		EFI CWD	China CWD
		Category by Population[b]												
HLA-A IPD-HLA/IMGT 3.31.0	Nomenclature Designation [a]	Highest Frequency	Total	AFA	API	EURO	MENA	HIS	NAM	UNK	HLA-A IPD-IMGT/HLA 3.9.0	Category	Category	Category
A*02:135	P	WD	WD			WD					A*02:135		WD	
A*02:136	P	I	WD					I		WD	A*02:136			
A*02:137	P	I	WD					I			A*02:137	WD		
A*02:138	P	WD	WD			WD					A*02:138		WD	
A*02:139	P	WD	WD			WD					A*02:139		WD	
A*02:141	P	WD	WD			WD	WD				A*02:141		WD	
A*02:147	P	I	WD					I			A*02:147			
A*02:149	P	WD	WD			WD					A*02:149			
A*02:151	P	I	WD			I				WD	A*02:151		WD	
A*02:154	P	WD	WD				WD				A*02:154			
A*02:156	P	WD	WD			WD					A*02:156		WD	
A*02:157	P	WD	WD			WD					A*02:157			
A*02:158	P	WD	WD			WD					A*02:158		WD	
A*02:160	P	WD	WD			WD					A*02:160		WD	
A*02:161	P	WD	WD				WD				A*02:161			
A*02:162	P	WD	WD			WD					A*02:162			
A*02:163	P	WD	WD			WD					A*02:163		WD	
A*02:164	P	WD	WD			WD					A*02:164		WD	
A*02:165	P	WD	WD			WD					A*02:165		WD	
A*02:166	P	WD	WD			WD					A*02:166		WD	
A*02:172	P	WD	WD			WD					A*02:172		WD	
A*02:173	P	WD	WD			WD					A*02:173		WD	
A*02:174	P	WD	WD								A*02:174		WD	
A*02:177	P	WD	WD	WD							A*02:177			
A*02:180	P	WD	WD			WD					A*02:180		WD	
A*02:182	P	WD	WD			WD					A*02:182		WD	
A*02:183	P	WD	WD			WD				WD	A*02:183			

Supplementary Table 3a. Comparison of HLA-A P group two-field assignments among CIWD catalogs [a]

		3.0.0 CIWD									2.0.0 CWD		EFI CWD	China CWD
		Category by Population[b]												
HLA-A IPD-HLA/IMGT 3.31.0	Nomenclature Designation [a]	Highest Frequency	Total	AFA	API	EURO	MENA	HIS	NAM	UNK	HLA-A IPD-IMGT/HLA 3.9.0	Category	Category	Category
A*02:186	P	WD	WD	WD							A*02:186			
A*02:188	P	WD	WD			WD					A*02:188			
A*02:189	P	WD	WD			WD					A*02:189		WD	WD
A*02:190	P	WD	WD			WD					A*02:190			
A*02:194	P	WD	WD			WD					A*02:194			
A*02:195	P	WD	WD			WD					A*02:195			
A*02:197	P	WD	WD			WD					A*02:197			
A*02:198	P	WD	WD			WD					A*02:198		WD	
A*02:199	P	WD	WD			WD					A*02:199		WD	
A*02:203	P	WD	WD			WD					A*02:203			
A*02:205	P	WD	WD			WD					A*02:205		WD	
A*02:206	P	WD	WD			WD					A*02:206			
A*02:209	P	WD	WD								A*02:209			
A*02:210	P	WD	WD			WD					A*02:210		WD	
A*02:211	P	I	WD			WD		I			A*02:211		WD	
A*02:212	P	WD	WD			WD					A*02:212			
A*02:213	P	o	o								A*02:213		WD	
A*02:217	P	WD	WD			WD					A*02:217		WD	
A*02:218	P	WD	WD			WD					A*02:218			
A*02:220	P	o	o								A*02:220		WD	
A*02:221	P	WD	WD			WD					A*02:221		WD	
A*02:227N	N	WD	WD			WD					A*02:227N			
A*02:230	P	o	o								A*02:230			WD
A*02:235	P	WD	WD			WD					A*02:235		WD	
A*02:236	P	WD	WD			WD					A*02:236			
A*02:237	P	WD	WD			WD					A*02:237			
A*02:245	P	WD	WD			WD					A*02:245		WD	

Supplementary Table 3a. Comparison of HLA-A P group two-field assignments among CIWD catalogs [a]

		3.0.0 CIWD									2.0.0 CWD		EFI CWD	China CWD
		Category by Population[b]												
HLA-A IPD-HLA/IMGT 3.31.0	Nomenclature Designation [a]	Highest Frequency	Total	AFA	API	EURO	MENA	HIS	NAM	UNK	HLA-A IPD-IMGT/HLA 3.9.0	Category	Category	Category
A*02:246	P	WD	WD			WD				WD	A*02:246			
A*02:249	P	o	o								A*02:249			WD
A*02:251	P	o	o								A*02:251			WD
A*02:259	P	o	o								A*02:259			WD
A*02:263	P	WD	WD								A*02:263			
A*02:269	P	o	o								A*02:269			WD
A*02:273	P	WD	WD			WD					A*02:273			
A*02:277	P	WD	WD			WD					A*02:277			
A*02:278	P	WD	WD					WD			A*02:278			
A*02:283	P	WD	WD			WD					A*02:283		WD	
A*02:289	P	WD	WD		WD	WD					A*02:289		WD	
A*02:293Q	Q	o	o								A*02:293Q			WD
A*02:299	P	WD	WD			WD					A*02:299			
A*02:306	P	WD	WD			WD					A*02:306			
A*02:312	P	WD	WD								A*02:312			
A*02:325	P	WD	WD			WD					A*02:325			
A*02:326	P	WD	WD			WD					A*02:326			
A*02:332	P	WD	WD			WD					A*02:332			
A*02:338	P	WD	WD			WD					A*02:338			
A*02:342	P	WD	WD			WD					A*02:342			
A*02:343	P	WD	WD			WD					A*02:343			
A*02:344	P	o	o								A*02:344			WD
A*02:346	P	WD	WD			WD					A*02:346			
A*02:359	P	WD	WD								A*02:359			
A*02:367	P	WD	WD			WD					A*02:367			
A*02:380	P	WD	WD			WD					A*02:380			
A*02:382	P	WD	WD				WD				A*02:382			

Supplementary Table 3a. Comparison of HLA-A P group two-field assignments among CIWD catalogs [a]

		3.0.0 CIWD									2.0.0 CWD		EFI CWD	China CWD
		Category by Population[b]												
HLA-A IPD-HLA/IMGT 3.31.0	Nomenclature Designation [a]	Highest Frequency	Total	AFA	API	EURO	MENA	HIS	NAM	UNK	HLA-A IPD-IMGT/HLA 3.9.0	Category	Category	Category
A*02:392	P	WD	WD								A*02:392			
A*02:393	P	WD	WD			WD					A*02:393		WD	
A*02:419	P	o	o								A*02:419			WD
A*02:423	P	WD	WD		WD						A*02:423			
A*02:433	P	WD	WD				WD				A*02:433			
A*02:434	P	WD	WD				WD				A*02:434			
A*02:436	P	I	WD		I						A*02:436			
A*02:478	P	o	o								A*02:478			WD
A*02:484	P	WD	WD			WD					A*02:484			
A*02:498	P	WD	WD			WD					A*02:498			
A*02:514N	N	WD	WD		WD						A*02:514N			
A*02:562	P	WD	WD				WD				A*02:562			
A*02:580	P	WD	WD		WD						A*02:580			
A*02:623	P	I	WD							I	A*02:623			
A*03:01	P	C	C	C	C	C	C	C	C	C	A*03:01	C	C	C
A*03:02	P	C	C	C	C	C	C	C	C	C	A*03:02	C	C	C
A*03:04	P	I	WD			WD		I			A*03:04		WD	
A*03:05	P	I	I			I		I		I	A*03:05	C	WD	WD
A*03:06	P	WD	WD			WD					A*03:06	WD		
A*03:07	P	I	WD	WD		WD		I		WD	A*03:07	WD	WD	
A*03:08	P	I	WD	WD		WD				I	A*03:08	WD	WD	
A*03:09	P	WD	WD								A*03:09	WD		
A*03:10	P	WD	WD		WD						A*03:10			
A*03:12	P	WD	WD			WD					A*03:12			WD
A*03:13	P	WD	WD			WD					A*03:13			
A*03:14	P	WD	WD			WD					A*03:14		WD	
A*03:15	P	WD	WD			WD					A*03:15		WD	

Supplementary Table 3a. Comparison of HLA-A P group two-field assignments among CIWD catalogs [a]

		3.0.0 CIWD									2.0.0 CWD		EFI CWD	China CWD
		Category by Population[b]												
HLA-A IPD-HLA/IMGT 3.31.0	Nomenclature Designation [a]	Highest Frequency	Total	AFA	API	EURO	MENA	HIS	NAM	UNK	HLA-A IPD-IMGT/HLA 3.9.0	Category	Category	Category
A*03:16	P	WD	WD			WD					A*03:16			
A*03:17	P	WD	WD			WD					A*03:17	WD		
A*03:18	P	WD	WD				WD				A*03:18			
A*03:21N	N	WD	WD			WD					A*03:21N	WD		
A*03:22	P	WD	WD			WD					A*03:22	WD	WD	
A*03:23	P	WD	WD			WD					A*03:23			
A*03:25	P	WD	WD			WD					A*03:25		WD	
A*03:27	P	WD	WD			WD					A*03:27		WD	
A*03:28	P	WD	WD			WD					A*03:28		WD	
A*03:29	P	WD	WD			WD				WD	A*03:29		WD	
A*03:33	P	I	I			I				I	A*03:33		WD	
A*03:38	P	WD	WD			WD					A*03:38		WD	
A*03:46	P	WD	WD			WD					A*03:46			
A*03:47	P	WD	WD			WD					A*03:47		WD	
A*03:48	P	WD	WD			WD					A*03:48		WD	
A*03:49	P	WD	WD			WD					A*03:49		WD	
A*03:50	P	WD	WD			WD					A*03:50	WD	WD	
A*03:51	P	WD	WD	WD							A*03:51			
A*03:53	P	WD	WD			WD					A*03:53		WD	
A*03:56	P	WD	WD			WD					A*03:56		WD	
A*03:57	P	WD	WD								A*03:57			
A*03:62	P	WD	WD			WD				WD	A*03:62			
A*03:63	P	WD	WD			WD					A*03:63		WD	
A*03:65	P	WD	WD			WD					A*03:65		WD	
A*03:74	P	WD	WD			WD					A*03:74		WD	
A*03:77	P	o	o								A*03:77			WD
A*03:81	P	WD	WD			WD					A*03:81		WD	

Supplementary Table 3a. Comparison of HLA-A P group two-field assignments among CIWD catalogs [a]

		3.0.0 CIWD									2.0.0 CWD		EFI CWD	China CWD
		Category by Population[b]												
HLA-A IPD-HLA/IMGT 3.31.0	Nomenclature Designation [a]	Highest Frequency	Total	AFA	API	EURO	MENA	HIS	NAM	UNK	HLA-A IPD-IMGT/HLA 3.9.0	Category	Category	Category
A*03:86	P	WD	WD			WD					A*03:86		WD	
A*03:87	P	WD	WD			WD					A*03:87		WD	
A*03:89	P	WD	WD			WD					A*03:89		WD	
A*03:93	P	WD	WD			WD					A*03:93			
A*03:94	P	WD	WD			WD					A*03:94			
A*03:102	P	WD	WD			WD					A*03:102		WD	
A*03:103	P	WD	WD								A*03:103			
A*03:107	P	WD	WD			WD					A*03:107		WD	
A*03:108	P	WD	WD			WD					A*03:108	WD		
A*03:111	P	WD	WD			WD					A*03:111		WD	
A*03:120	P	WD	WD			WD					A*03:120		WD	
A*03:123	P	WD	WD			WD					A*03:123			
A*03:144	P	WD	WD			WD					A*03:144			
A*03:149	P	WD	WD			WD					A*03:149			
A*03:154	P	WD	WD								A*03:154			
A*03:157	P	WD	WD			WD					A*03:157			
A*03:167	P	WD	WD								A*03:167			
A*03:202	P	WD	WD				WD				A*03:202			
A*03:216	P	WD	WD				WD				A*03:216			
A*03:228	P	WD	WD		WD						A*03:228			
A*11:01	P	C	C	C	C	C	C	C	C	C	A*11:01	C	C	C
A*11:02	P	C	C	WD	C	I	WD	I	WD	C	A*11:02	C	WD	C
A*11:03	P	C	C	WD	C	WD	WD	WD	C	I	A*11:03	WD	WD	C
A*11:04	P	C	I		C	I				I	A*11:04	C	WD	WD
A*11:05	P	I	I	WD		I		I		I	A*11:05	WD	WD	
A*11:06	P	WD	WD								A*11:06			WD
A*11:08	P	I	WD			WD		I		WD	A*11:08	WD	WD	

Supplementary Table 3a. Comparison of HLA-A P group two-field assignments among CIWD catalogs [a]

		3.0.0 CIWD									2.0.0 CWD		EFI CWD	China CWD
		Category by Population[b]												
HLA-A IPD-HLA/IMGT 3.31.0	Nomenclature Designation [a]	Highest Frequency	Total	AFA	API	EURO	MENA	HIS	NAM	UNK	HLA-A IPD-IMGT/HLA 3.9.0	Category	Category	Category
A*11:09	P	WD	WD			WD					A*11:09	WD		
A*11:10	P	WD	WD			WD					A*11:10	WD		
A*11:12	P	I	I		WD	I		I		I	A*11:12	WD	WD	WD
A*11:14	P	o	o								A*11:14			WD
A*11:15	P	WD	WD		WD	WD					A*11:15		WD	
A*11:18	P	WD	WD			WD	WD				A*11:18		WD	
A*11:19	P	I	WD		I	WD					A*11:19	WD	WD	WD
A*11:20	P	I	WD		I						A*11:20			
A*11:21N	N	WD	WD								A*11:21N			
A*11:25	P	WD	WD			WD					A*11:25			
A*11:27	P	o	o								A*11:27			WD
A*11:29	P	I	WD			I				WD	A*11:29	WD	WD	
A*11:30	P	WD	WD			WD					A*11:30	WD	WD	
A*11:32	P	WD	WD								A*11:32			WD
A*11:36	P	o	o								A*11:36			WD
A*11:37	P	WD	WD			WD					A*11:37		WD	
A*11:39	P	WD	WD			WD					A*11:39			
A*11:41	P	WD	WD								A*11:41			
A*11:44	P	I	WD		I						A*11:44			
A*11:45	P	WD	WD				WD				A*11:45			
A*11:46	P	o	o								A*11:46		WD	
A*11:47	P	WD	WD			WD					A*11:47		WD	
A*11:56	P	o	o								A*11:56			WD
A*11:58	P	I	WD		I						A*11:58			
A*11:60	P	o	o								A*11:60			WD
A*11:61	P	o	o								A*11:61			WD
A*11:63	P	o	o								A*11:63		WD	

Supplementary Table 3a. Comparison of HLA-A P group two-field assignments among CIWD catalogs [a]

		3.0.0 CIWD									2.0.0 CWD		EFI CWD	China CWD
		Category by Population[b]												
HLA-A IPD-HLA/IMGT 3.31.0	Nomenclature Designation [a]	Highest Frequency	Total	AFA	API	EURO	MENA	HIS	NAM	UNK	HLA-A IPD-IMGT/HLA 3.9.0	Category	Category	Category
A*11:64	P	WD	WD		WD						A*11:64			
A*11:67	P	WD	WD			WD					A*11:67			
A*11:69N	N	o	o								A*11:69N			WD
A*11:70	P	WD	WD			WD					A*11:70			
A*11:74	P	WD	WD			WD					A*11:74			
A*11:81	P	WD	WD			WD					A*11:81			
A*11:87	P	o	o								A*11:87			WD
A*11:88	P	o	o								A*11:88			WD
A*11:89	P	I	WD		I						A*11:89			
A*11:91	P	WD	WD			WD					A*11:91			
A*11:92	P	WD	WD			WD					A*11:92			
A*11:109N	N	WD	WD			WD					A*11:109N			
A*11:117	P	WD	WD			WD					A*11:117			
A*11:135	P	o	o								A*11:135		WD	
A*11:159	P	WD	WD		WD						A*11:159			
A*11:160	P	WD	WD								A*11:160			
A*11:161	P	WD	WD		WD						A*11:161			
A*11:183	P	I	WD		I						A*11:183			
A*11:187	P	WD	WD		WD						A*11:187			
A*11:211	P	WD	WD		WD						A*11:211			
A*11:221	P	WD	WD			WD					A*11:221			
A*23:01	P	C	C	C	C	C	C	C	C	C	A*23:01	C	C	C
A*23:02	P	WD	WD	WD							A*23:02		WD	
A*23:03	P	WD	WD	WD		WD					A*23:03	WD		
A*23:04	P	WD	WD	WD							A*23:04	WD		
A*23:05	P	C	I	WD		WD		C	C	I	A*23:05	WD		
A*23:06	P	WD	WD	WD		WD				WD	A*23:06	WD		

Supplementary Table 3a. Comparison of HLA-A P group two-field assignments among CIWD catalogs [a]

		3.0.0 CIWD									2.0.0 CWD		EFI CWD	China CWD
		Category by Population[b]												
HLA-A IPD-HLA/IMGT 3.31.0	Nomenclature Designation [a]	Highest Frequency	Total	AFA	API	EURO	MENA	HIS	NAM	UNK	HLA-A IPD-IMGT/HLA 3.9.0	Category	Category	Category
A*23:11N	N	WD	WD	WD							A*23:11N			
A*23:12	P	WD	WD			WD					A*23:12			
A*23:13	P	WD	WD			WD					A*23:13	WD	WD	
A*23:14	P	WD	WD								A*23:14			
A*23:15	P	C	WD	C		WD				I	A*23:15	WD		
A*23:19N	N	C	WD	C						WD	A*23:19N	WD		
A*23:23	P	WD	WD			WD					A*23:23		WD	
A*23:26	P	o	o								A*23:26			WD
A*23:30	P	WD	WD				WD				A*23:30			
A*23:39	P	WD	WD				WD				A*23:39			
A*23:47	P	WD	WD			WD					A*23:47			
A*23:48	P	WD	WD			WD					A*23:48			
A*23:53	P	WD	WD			WD					A*23:53			
A*23:56	P	WD	WD								A*23:56			
A*24:02	P	C	C	C	C	C	C	C	C	C	A*24:02	C	C	C
A*24:03	P	C	C	C	C	C	C	C	C	C	A*24:03	C	C	C
A*24:04	P	I	WD		I	WD					A*24:04	WD	WD	WD
A*24:05	P	C	I	WD	I	WD		C	C	I	A*24:05	WD	WD	WD
A*24:06	P	WD	WD		WD	WD					A*24:06	WD	WD	WD
A*24:07	P	C	C	C	C	C	C	C	C	C	A*24:07	C	C	C
A*24:08	P	I	WD		I					WD	A*24:08	WD	WD	WD
A*24:09N	N	WD	WD			WD					A*24:09N	WD		
A*24:10	P	C	C		C	WD	WD			C	A*24:10	C	WD	WD
A*24:11N	N	I	WD		I	WD					A*24:11N	WD		
A*24:13	P	I	WD		I	WD		I		WD	A*24:13	WD		WD
A*24:14	P	C	I	WD	WD	WD		C	C	I	A*24:14	WD		
A*24:15	P	WD	WD					WD			A*24:15	WD		

Supplementary Table 3a. Comparison of HLA-A P group two-field assignments among CIWD catalogs [a]

		3.0.0 CIWD									2.0.0 CWD		EFI CWD	China CWD
		Category by Population[b]												
HLA-A IPD-HLA/IMGT 3.31.0	Nomenclature Designation [a]	Highest Frequency	Total	AFA	API	EURO	MENA	HIS	NAM	UNK	HLA-A IPD-IMGT/HLA 3.9.0	Category	Category	Category
A*24:17	P	C	C	WD	C	WD	WD		C	I	A*24:17	C		WD
A*24:18	P	WD	WD			WD					A*24:18			
A*24:20	P	C	I		C	WD	WD			C	A*24:20	C		C
A*24:21	P	WD	WD		WD	WD				WD	A*24:21			WD
A*24:22	P	C	I	WD		WD		C	C	I	A*24:22	WD		
A*24:23	P	C	I	C	WD	WD		I	C	I	A*24:23	WD		
A*24:24	P	WD	WD	WD							A*24:24			
A*24:25	P	C	C	WD	WD	WD		C	C	C	A*24:25	C		
A*24:26	P	I	I	WD		WD	WD	I		I	A*24:26	WD		
A*24:28	P	I	WD			WD		I		WD	A*24:28			WD
A*24:29	P	I	I			I	WD			WD	A*24:29	WD		
A*24:30	P	I	WD		I	WD					A*24:30	WD		WD
A*24:31	P	I	I			I				WD	A*24:31	WD		
A*24:32	P	I	WD			WD				I	A*24:32	WD		
A*24:35	P	WD	WD			WD					A*24:35	WD		
A*24:36N	N	WD	WD			WD					A*24:36N			
A*24:37	P	I	WD		I					WD	A*24:37	WD		
A*24:38	P	I	WD					I		WD	A*24:38	WD		
A*24:39	P	WD	WD			WD					A*24:39			
A*24:42	P	WD	WD			WD					A*24:42			
A*24:43	P	I	WD			I				WD	A*24:43	WD		
A*24:46	P	WD	WD								A*24:46			
A*24:47	P	WD	WD			WD					A*24:47	WD		
A*24:50	P	WD	WD								A*24:50			
A*24:52	P	WD	WD			WD	WD			WD	A*24:52			WD
A*24:53	P	C	I			WD		C	C	I	A*24:53	WD		
A*24:56	P	I	I			I	WD			WD	A*24:56	WD		

Supplementary Table 3a. Comparison of HLA-A P group two-field assignments among CIWD catalogs [a]

		3.0.0 CIWD									2.0.0 CWD		EFI CWD	China CWD
		Category by Population[b]												
HLA-A IPD-HLA/IMGT 3.31.0	Nomenclature Designation [a]	Highest Frequency	Total	AFA	API	EURO	MENA	HIS	NAM	UNK	HLA-A IPD-IMGT/HLA 3.9.0	Category	Category	Category
A*24:57	P	I	WD			WD				I	A*24:57	WD		
A*24:58	P	I	I			I				WD	A*24:58	WD		
A*24:59	P	o	o								A*24:59			WD
A*24:64	P	I	WD		I						A*24:64	WD		WD
A*24:68	P	WD	WD		WD						A*24:68			WD
A*24:71	P	WD	WD			WD					A*24:71			
A*24:72	P	I	I			I					A*24:72	WD		
A*24:81	P	I	WD			WD				I	A*24:81	WD		
A*24:84N	N	WD	WD			WD					A*24:84N			
A*24:85	P	o	o								A*24:85			WD
A*24:87	P	WD	WD			WD				WD	A*24:87			
A*24:90N	N	WD	WD			WD					A*24:90N	WD		
A*24:91	P	o	o								A*24:91			WD
A*24:93	P	o	o								A*24:93			WD
A*24:95	P	C	WD			WD	WD	C		I	A*24:95	WD		
A*24:97	P	I	WD		I						A*24:97			
A*24:98	P	o	o								A*24:98			WD
A*24:103	P	WD	WD			WD					A*24:103			
A*24:104	P	WD	WD			WD					A*24:104			
A*24:105	P	WD	WD			WD					A*24:105			
A*24:106	P	WD	WD			WD					A*24:106			
A*24:107	P	WD	WD								A*24:107			
A*24:108	P	WD	WD								A*24:108			
A*24:110	P	WD	WD			WD					A*24:110			
A*24:112	P	o	o								A*24:112			WD
A*24:118	P	WD	WD			WD					A*24:118			
A*24:120	P	WD	WD								A*24:120			

Supplementary Table 3a. Comparison of HLA-A P group two-field assignments among CIWD catalogs [a]

		3.0.0 CIWD									2.0.0 CWD		EFI CWD	China CWD
		Category by Population[b]												
HLA-A IPD-HLA/IMGT 3.31.0	Nomenclature Designation [a]	Highest Frequency	Total	AFA	API	EURO	MENA	HIS	NAM	UNK	HLA-A IPD-IMGT/HLA 3.9.0	Category	Category	Category
A*24:121	P	WD	WD			WD					A*24:121			
A*24:122	P	WD	WD			WD					A*24:122			
A*24:123	P	WD	WD				WD				A*24:123			
A*24:128	P	o	o								A*24:128			WD
A*24:130	P	WD	WD		WD						A*24:130			
A*24:132N	N	o	o								A*24:132N			WD
A*24:138	P	WD	WD								A*24:138			
A*24:140	P	WD	WD		WD						A*24:140			
A*24:141	P	o	o								A*24:141			WD
A*24:145	P	I	WD			WD				I	A*24:145			
A*24:146	P	WD	WD				WD				A*24:146			
A*24:152	P	o	o								A*24:152			WD
A*24:159	P	WD	WD					WD			A*24:159			
A*24:164	P	WD	WD			WD					A*24:164			
A*24:165	P	WD	WD		WD						A*24:165			
A*24:168	P	WD	WD			WD					A*24:168			
A*24:169	P	WD	WD			WD					A*24:169			
A*24:173	P	WD	WD							WD	A*24:173			
A*24:175	P	I	WD			WD		WD		I	A*24:175			
A*24:176	P	WD	WD			WD					A*24:176			
A*24:180	P	WD	WD					WD			A*24:180			
A*24:192	P	WD	WD								A*24:192			
A*24:193	P	WD	WD			WD					A*24:193			
A*24:194	P	WD	WD								A*24:194			
A*24:196	P	I	WD		I						A*24:196			
A*24:203	P	WD	WD			WD					A*24:203			
A*24:205	P	WD	WD								A*24:205			

Supplementary Table 3a. Comparison of HLA-A P group two-field assignments among CIWD catalogs [a]

		3.0.0 CIWD									2.0.0 CWD		EFI CWD	China CWD
		Category by Population[b]												
HLA-A IPD-HLA/IMGT 3.31.0	Nomenclature Designation [a]	Highest Frequency	Total	AFA	API	EURO	MENA	HIS	NAM	UNK	HLA-A IPD-IMGT/HLA 3.9.0	Category	Category	Category
A*24:206	P	WD	WD			WD					A*24:206			
A*24:207	P	WD	WD								A*24:207			
A*24:220	P	WD	WD								A*24:220			
A*24:225	P	o	o								A*24:225			WD
A*24:226	P	WD	WD			WD					A*24:226			
A*24:228	P	WD	WD			WD					A*24:228			
A*24:234	P	WD	WD								A*24:234			
A*24:252N	N	WD	WD			WD	WD				A*24:252N			
A*25:01	P	C	C	C	C	C	C	C	C	C	A*25:01	C	C	WD
A*25:02	P	I	WD			I				WD	A*25:02	WD	WD	
A*25:03	P	WD	WD			WD					A*25:03		WD	
A*25:04	P	WD	WD			WD					A*25:04			
A*25:08	P	WD	WD			WD					A*25:08		WD	
A*25:11	P	WD	WD			WD					A*25:11			
A*25:12N	N	WD	WD			WD					A*25:12N			
A*25:13	P	WD	WD								A*25:13		WD	
A*25:14	P	WD	WD			WD					A*25:14		WD	
A*25:16	P	WD	WD			WD					A*25:16			
A*25:24	P	WD	WD					WD			A*25:24			
A*26:01	P	C	C	C	C	C	C	C	C	C	A*26:01	C	C	C
A*26:02	P	C	I	WD	C	WD		WD		C	A*26:02	WD	WD	WD
A*26:03	P	C	I		C	WD				C	A*26:03	C	WD	WD
A*26:05	P	WD	WD		WD					WD	A*26:05		WD	
A*26:06	P	WD	WD							WD	A*26:06			
A*26:07	P	I	I		I	WD		I		WD	A*26:07	WD		
A*26:08	P	C	C	C	I	C	C	C	C	C	A*26:08	C	C	WD
A*26:09	P	I	I			I	WD			I	A*26:09	WD	WD	WD

Supplementary Table 3a. Comparison of HLA-A P group two-field assignments among CIWD catalogs [a]

		3.0.0 CIWD									2.0.0 CWD		EFI CWD	China CWD
		Category by Population[b]												
HLA-A IPD-HLA/IMGT 3.31.0	Nomenclature Designation [a]	Highest Frequency	Total	AFA	API	EURO	MENA	HIS	NAM	UNK	HLA-A IPD-IMGT/HLA 3.9.0	Category	Category	Category
A*26:10	P	WD	WD		WD						A*26:10			
A*26:12	P	C	I	C		WD	WD	I		I	A*26:12	WD	WD	
A*26:13	P	WD	WD			WD					A*26:13	WD	WD	
A*26:14	P	I	WD		I	WD					A*26:14	WD	WD	WD
A*26:15	P	I	I			I				I	A*26:15	WD	WD	
A*26:16	P	WD	WD			WD	WD				A*26:16	WD		
A*26:17	P	C	I		I	WD	C	I		I	A*26:17	WD	WD	WD
A*26:18	P	I	WD		I						A*26:18	WD		WD
A*26:20	P	WD	WD								A*26:20	WD		WD
A*26:27	P	I	WD			WD		I			A*26:27			
A*26:30	P	WD	WD	WD							A*26:30			
A*26:31	P	WD	WD			WD					A*26:31	WD		
A*26:35	P	o	o								A*26:35			WD
A*26:36	P	o	o								A*26:36			WD
A*26:37	P	WD	WD			WD					A*26:37		WD	
A*26:38	P	WD	WD			WD					A*26:38		WD	
A*26:39	P	WD	WD			WD					A*26:39		WD	
A*26:42	P	WD	WD								A*26:42			
A*26:45	P	WD	WD			WD					A*26:45		WD	
A*26:47	P	WD	WD			WD					A*26:47			
A*26:49	P	WD	WD			WD					A*26:49			
A*26:50	P	o	o								A*26:50			WD
A*26:52	P	WD	WD								A*26:52			
A*26:59	P	WD	WD								A*26:59			
A*26:62	P	WD	WD			WD					A*26:62			
A*26:63	P	I	WD		I						A*26:63			
A*26:65	P	WD	WD			WD					A*26:65			

Supplementary Table 3a. Comparison of HLA-A P group two-field assignments among CIWD catalogs [a]

		3.0.0 CIWD									2.0.0 CWD		EFI CWD	China CWD
		Category by Population[b]												
HLA-A IPD-HLA/IMGT 3.31.0	Nomenclature Designation [a]	Highest Frequency	Total	AFA	API	EURO	MENA	HIS	NAM	UNK	HLA-A IPD-IMGT/HLA 3.9.0	Category	Category	Category
A*26:75	P	WD	WD			WD					A*26:75			
A*26:76	P	I	WD		I						A*26:76			
A*26:104	P	WD	WD			WD					A*26:104			
A*29:01	P	C	C	C	C	C	C	C	C	C	A*29:01	C	C	C
A*29:02	P	C	C	C	C	C	C	C	C	C	A*29:02	C	C	WD
A*29:03	P	WD	WD			WD					A*29:03		WD	
A*29:04	P	WD	WD			WD				WD	A*29:04	WD		
A*29:07	P	WD	WD			WD					A*29:07		WD	
A*29:09	P	WD	WD			WD					A*29:09	WD		
A*29:10	P	C	C		WD	C	C	I		I	A*29:10	WD	C	WD
A*29:11	P	WD	WD	WD		WD	WD			WD	A*29:11			
A*29:12	P	WD	WD			WD	WD			WD	A*29:12	WD	WD	
A*29:13	P	WD	WD								A*29:13			
A*29:14	P	WD	WD								A*29:14			
A*29:16	P	WD	WD			WD					A*29:16		WD	
A*29:21	P	WD	WD			WD					A*29:21		WD	
A*29:23	P	WD	WD	WD							A*29:23			
A*29:32	P	WD	WD			WD					A*29:32			
A*29:34	P	WD	WD			WD					A*29:34		WD	
A*29:40	P	WD	WD								A*29:40			
A*29:44	P	WD	WD			WD					A*29:44			
A*29:48	P	WD	WD			WD					A*29:48			
A*29:64	P	WD	WD		WD						A*29:64			
A*29:88	P	WD	WD								A*29:88			
A*30:01	P	C	C	C	C	C	C	C	C	C	A*30:01	C	C	C
A*30:02	P	C	C	C	C	C	C	C	C	C	A*30:02	C	C	WD
A*30:03	P	WD	WD			WD					A*30:03	WD		

Supplementary Table 3a. Comparison of HLA-A P group two-field assignments among CIWD catalogs [a]

		3.0.0 CIWD									2.0.0 CWD		EFI CWD	China CWD
		Category by Population[b]												
HLA-A IPD-HLA/IMGT 3.31.0	Nomenclature Designation [a]	Highest Frequency	Total	AFA	API	EURO	MENA	HIS	NAM	UNK	HLA-A IPD-IMGT/HLA 3.9.0	Category	Category	Category
A*30:04	P	C	C	C	C	C	C	C	C	C	A*30:04	C	C	WD
A*30:07	P	C	WD	C						I	A*30:07	WD		
A*30:08	P	WD	WD	WD			WD				A*30:08	WD		
A*30:09	P	I	WD	WD			WD	I		WD	A*30:09	WD	WD	
A*30:10	P	C	C	C	I	I	C	C	C	C	A*30:10	WD	WD	
A*30:11	P	I	WD		I	WD					A*30:11	WD	WD	
A*30:15	P	WD	WD			WD					A*30:15	WD	WD	
A*30:16	P	WD	WD	WD							A*30:16	WD		
A*30:18	P	WD	WD		WD						A*30:18			WD
A*30:20	P	o	o								A*30:20			WD
A*30:25	P	WD	WD								A*30:25			
A*30:28	P	WD	WD	WD							A*30:28			
A*30:29	P	WD	WD			WD	WD				A*30:29		WD	
A*30:30	P	WD	WD			WD					A*30:30			
A*30:31	P	WD	WD	WD							A*30:31			
A*30:36	P	WD	WD			WD					A*30:36		WD	
A*30:38	P	o	o								A*30:38			WD
A*30:40	P	WD	WD			WD					A*30:40		WD	
A*30:47	P	WD	WD				WD				A*30:47			
A*30:51	P	I	WD					I			A*30:51			
A*30:54	P	WD	WD								A*30:54			
A*30:56	P	WD	WD								A*30:56			
A*30:58	P	WD	WD			WD					A*30:58			
A*30:70N	N	WD	WD	WD							A*30:70N			
A*30:78N	N	WD	WD	WD							A*30:78N			
A*31:01	P	C	C	C	C	C	C	C	C	C	A*31:01	C	C	C
A*31:02	P	C	I	WD		WD		C	C	C	A*31:02	WD	WD	

Supplementary Table 3a. Comparison of HLA-A P group two-field assignments among CIWD catalogs [a]

		3.0.0 CIWD									2.0.0 CWD		EFI CWD	China CWD
		Category by Population[b]												
HLA-A IPD-HLA/IMGT 3.31.0	Nomenclature Designation [a]	Highest Frequency	Total	AFA	API	EURO	MENA	HIS	NAM	UNK	HLA-A IPD-IMGT/HLA 3.9.0	Category	Category	Category
A*31:03	P	o	o								A*31:03	WD		
A*31:04	P	C	I	C		WD	C	WD		I	A*31:04	WD	WD	
A*31:05	P	WD	WD								A*31:05		WD	
A*31:06	P	C	I		C	WD	WD			I	A*31:06	WD	WD	WD
A*31:07	P	I	WD					I			A*31:07			
A*31:08	P	I	I			I				WD	A*31:08	WD	C	
A*31:09	P	C	I			WD		C	C	I	A*31:09	WD		
A*31:11	P	I	WD		I						A*31:11	WD		
A*31:12	P	C	I		C	WD				I	A*31:12	WD		
A*31:13	P	I	WD		I						A*31:13	WD		WD
A*31:14N	N	WD	WD			WD					A*31:14N			
A*31:15	P	C	I	WD		WD		C		I	A*31:15	WD		
A*31:16	P	C	I		C	WD	WD		WD	WD	A*31:16	WD		
A*31:17	P	o	o								A*31:17			WD
A*31:18	P	I	WD					I		WD	A*31:18	WD		
A*31:19	P	WD	WD			WD					A*31:19			
A*31:20	P	WD	WD			WD				WD	A*31:20	WD		
A*31:25	P	WD	WD			WD					A*31:25			
A*31:26	P	WD	WD			WD					A*31:26	WD	WD	
A*31:27	P	WD	WD			WD					A*31:27		WD	
A*31:28	P	WD	WD			WD					A*31:28		WD	
A*31:30	P	WD	WD								A*31:30			WD
A*31:32	P	o	o								A*31:32			WD
A*31:33	P	o	o								A*31:33			WD
A*31:36	P	I	WD					I			A*31:36			
A*31:38	P	WD	WD			WD					A*31:38		WD	
A*31:39	P	WD	WD			WD					A*31:39		WD	

Supplementary Table 3a. Comparison of HLA-A P group two-field assignments among CIWD catalogs [a]

		3.0.0 CIWD									2.0.0 CWD		EFI CWD	China CWD
		Category by Population[b]												
HLA-A IPD-HLA/IMGT 3.31.0	Nomenclature Designation [a]	Highest Frequency	Total	AFA	API	EURO	MENA	HIS	NAM	UNK	HLA-A IPD-IMGT/HLA 3.9.0	Category	Category	Category
A*31:40	P	I	WD			WD				I	A*31:40			
A*31:60N	N	WD	WD								A*31:60N			
A*31:68	P	WD	WD		WD						A*31:68			
A*31:88	P	WD	WD				WD				A*31:88			
A*32:01	P	C	C	C	C	C	C	C	C	C	A*32:01	C	C	C
A*32:02	P	I	WD			WD		I		I	A*32:02	WD	WD	
A*32:03	P	I	I		WD	I				WD	A*32:03	WD		
A*32:04	P	I	I			I		WD		I	A*32:04	WD		
A*32:05	P	WD	WD		WD						A*32:05			
A*32:06	P	WD	WD			WD					A*32:06	WD		
A*32:07	P	WD	WD			WD				WD	A*32:07	WD		
A*32:08	P	WD	WD			WD				WD	A*32:08	WD		
A*32:11Q	Q	I	WD		I						A*32:11Q			
A*32:12	P	WD	WD			WD					A*32:12			
A*32:16	P	WD	WD			WD					A*32:16			
A*32:17	P	WD	WD			WD					A*32:17			
A*32:18	P	WD	WD			WD					A*32:18			
A*32:21	P	WD	WD								A*32:21			
A*32:22	P	WD	WD								A*32:22			
A*32:26	P	WD	WD							WD	A*32:26			
A*32:27N	N	WD	WD			WD					A*32:27N			
A*32:28	P	WD	WD			WD					A*32:28			
A*32:31	P	WD	WD			WD					A*32:31			
A*32:44	P	WD	WD			WD					A*32:44			
A*32:45N	N	WD	WD			WD					A*32:45N			
A*32:47	P	WD	WD								A*32:47			
A*32:55	P	WD	WD			WD					A*32:55			

Supplementary Table 3a. Comparison of HLA-A P group two-field assignments among CIWD catalogs [a]

		3.0.0 CIWD									2.0.0 CWD		EFI CWD	China CWD
		Category by Population[b]												
HLA-A IPD-HLA/IMGT 3.31.0	Nomenclature Designation [a]	Highest Frequency	Total	AFA	API	EURO	MENA	HIS	NAM	UNK	HLA-A IPD-IMGT/HLA 3.9.0	Category	Category	Category
A*32:60	P	WD	WD			WD					A*32:60			
A*32:62	P	WD	WD			WD					A*32:62			
A*33:01	P	C	C	C	C	C	C	C	C	C	A*33:01	C	C	C
A*33:03	P	C	C	C	C	C	C	C	C	C	A*33:03	C	C	C
A*33:04	P	I	WD			WD		WD		I	A*33:04		WD	
A*33:05	P	C	C	C	I	C	C	C	C	C	A*33:05	WD	C	
A*33:08	P	WD	WD								A*33:08			WD
A*33:09	P	WD	WD			WD					A*33:09	WD	WD	
A*33:10	P	I	WD		I						A*33:10			WD
A*33:11	P	WD	WD								A*33:11			
A*33:12	P	WD	WD			WD					A*33:12		WD	
A*33:13	P	WD	WD		WD						A*33:13			
A*33:16	P	WD	WD					WD			A*33:16			
A*33:26	P	C	WD				C				A*33:26			
A*33:27	P	WD	WD			WD					A*33:27		WD	
A*33:30	P	o	o								A*33:30			WD
A*33:33	P	WD	WD				WD				A*33:33			
A*33:34	P	WD	WD			WD					A*33:34			
A*33:48	P	I	WD		I						A*33:48			
A*33:55	P	WD	WD		WD						A*33:55			
A*33:60	P	WD	WD			WD					A*33:60			
A*33:64	P	WD	WD			WD					A*33:64			
A*33:65	P	I	WD		I						A*33:65			
A*34:01	P	C	C	WD	C	I	WD	C	C	C	A*34:01	C		WD
A*34:02	P	C	C	C	I	C	C	C	C	C	A*34:02	C	C	WD
A*34:04	P	WD	WD			WD					A*34:04			
A*34:05	P	C	I		C	WD		I		C	A*34:05	C	WD	

Supplementary Table 3a. Comparison of HLA-A P group two-field assignments among CIWD catalogs [a]

		3.0.0 CIWD									2.0.0 CWD		EFI CWD	China CWD
		Category by Population[b]												
HLA-A IPD-HLA/IMGT 3.31.0	Nomenclature Designation [a]	Highest Frequency	Total	AFA	API	EURO	MENA	HIS	NAM	UNK	HLA-A IPD-IMGT/HLA 3.9.0	Category	Category	Category
A*34:06	P	WD	WD			WD					A*34:06			
A*34:07	P	WD	WD	WD							A*34:07			
A*34:08	P	WD	WD								A*34:08			
A*34:10N	N	WD	WD	WD							A*34:10N			
A*36:01	P	C	C	C	I	I	C	C	C	C	A*36:01	C	C	
A*36:02	P	WD	WD								A*36:02	WD		WD
A*36:03	P	C	I	C			WD	WD		I	A*36:03	WD		
A*36:04	P	I	WD			WD				I	A*36:04	WD	WD	
A*43:01	P	C	I	C	WD	WD	C	WD		I	A*43:01	C	WD	
A*66:01	P	C	C	C	C	C	C	C	C	C	A*66:01	C	C	WD
A*66:02	P	C	C	C	WD	I	WD	C	C	C	A*66:02	C		
A*66:03	P	C	I	C		WD	WD	C	C	C	A*66:03	WD		
A*66:04	P	WD	WD	WD							A*66:04	WD		
A*66:05	P	WD	WD			WD					A*66:05			
A*66:13	P	WD	WD			WD					A*66:13			
A*66:15	P	WD	WD				WD				A*66:15			
A*68:01	P	C	C	C	C	C	C	C	C	C	A*68:01	C	C	C
A*68:02	P	C	C	C	C	C	C	C	C	C	A*68:02	C	C	WD
A*68:03	P	C	C	C	I	I	WD	C	C	C	A*68:03	C	WD	
A*68:04	P	WD	WD							WD	A*68:04			
A*68:05	P	C	C	WD	I	WD		C	C	C	A*68:05	C	WD	
A*68:06	P	I	WD					I		WD	A*68:06	WD		
A*68:07	P	C	I	WD		WD		C	C	I	A*68:07	WD	WD	
A*68:08	P	WD	WD	WD		WD				WD	A*68:08	WD	WD	
A*68:09	P	o	o								A*68:09		WD	
A*68:10	P	C	WD	C						I	A*68:10	WD		
A*68:11N	N	o	o								A*68:11N	WD		

Supplementary Table 3a. Comparison of HLA-A P group two-field assignments among CIWD catalogs [a]

		3.0.0 CIWD									2.0.0 CWD		EFI CWD	China CWD
		Category by Population[b]												
HLA-A IPD-HLA/IMGT 3.31.0	Nomenclature Designation [a]	Highest Frequency	Total	AFA	API	EURO	MENA	HIS	NAM	UNK	HLA-A IPD-IMGT/HLA 3.9.0	Category	Category	Category
A*68:12	P	C	I		WD	I	C	I	C	I	A*68:12	WD	WD	
A*68:13	P	C	WD			WD	C				A*68:13		WD	
A*68:15	P	C	I	C		WD		I		I	A*68:15	WD	WD	
A*68:16	P	I	I			WD		I		I	A*68:16		WD	
A*68:17	P	C	C			I		C	C	C	A*68:17	WD	WD	
A*68:18N	N	WD	WD			WD					A*68:18N			
A*68:19	P	WD	WD			WD					A*68:19			
A*68:20	P	I	WD					I		WD	A*68:20	WD		
A*68:21	P	WD	WD					WD			A*68:21			
A*68:22	P	WD	WD			WD					A*68:22	WD		
A*68:23	P	I	WD			WD		I		I	A*68:23			
A*68:24	P	C	I	WD	C	I	WD	I		I	A*68:24	WD	C	WD
A*68:25	P	I	I			I				WD	A*68:25	WD	WD	
A*68:26	P	WD	WD			WD				WD	A*68:26		WD	
A*68:27	P	WD	WD			WD					A*68:27	WD		
A*68:29	P	WD	WD					WD			A*68:29			
A*68:30	P	I	WD					I			A*68:30	WD		
A*68:31	P	I	WD					I			A*68:31	WD		
A*68:32	P	WD	WD			WD					A*68:32			
A*68:35	P	I	I			I				WD	A*68:35	WD	WD	
A*68:36	P	WD	WD					WD			A*68:36			
A*68:37	P	I	WD			I					A*68:37	WD	WD	
A*68:38	P	WD	WD				WD				A*68:38			WD
A*68:40	P	WD	WD	WD							A*68:40	WD		
A*68:43	P	WD	WD								A*68:43			
A*68:45	P	WD	WD								A*68:45			
A*68:46	P	WD	WD					WD			A*68:46			

Supplementary Table 3a. Comparison of HLA-A P group two-field assignments among CIWD catalogs [a]

		3.0.0 CIWD									2.0.0 CWD		EFI CWD	China CWD
		Category by Population[b]												
HLA-A IPD-HLA/IMGT 3.31.0	Nomenclature Designation [a]	Highest Frequency	Total	AFA	API	EURO	MENA	HIS	NAM	UNK	HLA-A IPD-IMGT/HLA 3.9.0	Category	Category	Category
A*68:54	P	WD	WD	WD						WD	A*68:54			
A*68:55	P	I	WD			WD	WD			I	A*68:55			
A*68:57	P	WD	WD			WD					A*68:57		WD	
A*68:60	P	WD	WD								A*68:60			
A*68:67	P	WD	WD	WD							A*68:67			
A*68:68	P	WD	WD	WD							A*68:68			
A*68:72	P	WD	WD			WD					A*68:72			
A*68:85	P	WD	WD					WD			A*68:85			
A*68:91	P	WD	WD			WD	WD				A*68:91			
A*68:95	P	WD	WD			WD					A*68:95			
A*68:99	P	I	WD		I						A*68:99			
A*68:101	P	WD	WD			WD					A*68:101			
A*68:104	P	WD	WD					WD			A*68:104			
A*69:01	P	C	C	C	C	C	C	C	C	C	A*69:01	C	C	C
A*74:01	P	C	C	C	C	C	C	C	C	C	A*74:01	C	WD	WD
A*74:03	P	C	C	C	I	C	C	C	WD	C	A*74:03	C		WD
A*74:05	P	I	WD		I	WD					A*74:05	WD		WD
A*74:06	P	I	I			I				I	A*74:06	WD		
A*74:07	P	WD	WD								A*74:07			
A*74:08	P	I	WD					I			A*74:08			
A*74:09	P	C	I	C		WD		WD		I	A*74:09	WD		
A*74:10	P	WD	WD	WD							A*74:10	WD		
A*74:11	P	C	I	C		WD		I	WD	I	A*74:11	WD		
A*74:13	P	WD	WD								A*74:13	WD		WD
A*74:16	P	WD	WD	WD							A*74:16			
A*80:01	P	C	C	C	I	C	C	C	C	C	A*80:01	C	WD	

Supplementary Table 3a. Comparison of HLA-A P group two-field assignments among CIWD catalogs [a]

		3.0.0 CIWD									2.0.0 CWD		EFI CWD	China CWD
		Category by Population[b]												
HLA-A IPD-HLA/IMGT 3.31.0	Nomenclature Designation [a]	Highest Frequency	Total	AFA	API	EURO	MENA	HIS	NAM	UNK	HLA-A IPD-IMGT/HLA 3.9.0	Category	Category	Category

C, common; I, intermediate; WD, well-documented

[a] Alleles observed at least five times in any population group in the current dataset are included in this table. The exception are alleles that were observed in previous catalogs but that are not present or are observed less than five times. Note that this two-field designation is the equivalent of a "P" assignment so that expressed alleles within a P group that encode proteins that differ outside of the antigen recognition domain are not listed separately. For example, A*23:17 is within the A*23:01 two-field assignment and is not listed in this table even though it was categorized as well-documented in 2.0.0 CWD. The table does not list all alleles from IPD-IMGT/HLA version 3.31.0.

[b] Population groups include: AFA (African/African American), API (Asian/Pacific Islander), EURO (European/European descent), MENA (Middle East/North Coast of Africa), HIS (Hispanic), NAM (Native American) and UNK (unknown, multiple ancestries or other). Total is the overall population i.e., all groups combined. Highest frequency is the highest CIWD designation among all the individual groups.

[c] Any non-expressed alleles which are excluded from the P group are listed. For example, A*01:04N is within the A*01:01:01G group but is listed separately because it is not expressed.

[d] Not-CIWD, o.

References

Mack SJ, Cano P, Hollenbach JA, He J, Hurley CK, Middleton D, Moraes ME, Pereira SE, Kempenich JH, Reed EF, Setterholm M, Smith AG, Tilanus MG, Torres M, Varney MD, Voorter CE, Fischer GF, Fleischhauer K, Goodridge D, Klitz W, Little AM, Maiers M, Marsh SG, Muller CR, Noreen H, Rozemuller EH, Sanchez-Mazas A, Senitzer D, Trachtenberg E, Fernandez-Vina M: Common and well-documented HLA alleles: 2012 update to the CWD catalogue. Tissue Antigens 81:194-203, 2013.

Sanchez-Mazas A, Nunes JM, Middleton D, Sauter J, Buhler S, McCabe A, Hofmann J, Baier DM, Schmidt AH, Nicoloso G, Andreani M, Grubic Z, Tiercy JM, Fleischhauer K: Common and well-documented HLA alleles over all of Europe and within European sub-regions: A catalogue from the European Federation for Immunogenetics. HLA 89:104-113, 2017.

He Y, Li J, Mao W, Zhang D, Liu M, Shan X, Zhang B, Zhu C, Shen J, Deng Z, Wang Z, Yu W, Chen Q, Guo W, Su P, Lv R, Li G, Li G, Pei B, Jiao L, Shen G, Liu Y, Feng Z, Su Y, Xie Y, Di W, Liu X, Yang X, Wang J, Qi J, Liu Q, Han Y, He J, Cai J, Zhang Z, Zhu F, Du D: HLA common and well-documented alleles in China. HLA 92:199-205, 2018.

Supplementary Table 3b. Comparison of HLA-B P group two-field assignments among CIWD catalogs [a]

		3.0.0 CIWD									2.0.0 CWD		EFI CWD	China CWD
		Category by Population [b]												
HLA-B IPD-HLA/IMGT 3.31.0	Nomenclature Designation [a]	Highest Frequency	Total	AFA	API	EURO	MENA	HIS	NAM	UNK	HLA-B IPD-IMGT/HLA 3.9.0	Category	Category	Category
B*07:02	P	C	C	C	C	C	C	C	C	C	B*07:02	C	C	C
B*07:03	P	I	I			I		WD		I	B*07:03	WD	WD	
B*07:04	P	C	C	WD	WD	C	WD	I		C	B*07:04	C	C	
B*07:05	P	C	C	C	C	C	C	C	C	C	B*07:05	C	C	C
B*07:07	P	I	I	WD		I	WD	I		I	B*07:07	WD	WD	
B*07:08	P	WD	WD			WD					B*07:08	WD	WD	WD
B*07:09	P	C	I	C	I	I		I	C	I	B*07:09	WD	WD	
B*07:10	P	C	I	WD	I	C	C	I		C	B*07:10	C	C	WD
B*07:12	P	C	WD	C						WD	B*07:12	WD		
B*07:13	P	WD	WD	WD							B*07:13	WD		
B*07:14	P	C	I			I	C	C	WD	I	B*07:14	WD	WD	
B*07:15	P	I	I			I				WD	B*07:15	WD	WD	
B*07:16	P	WD	WD			WD					B*07:16	WD	WD	
B*07:17	P	WD	WD								B*07:17			
B*07:18	P	I	WD		I						B*07:18		WD	WD
B*07:19	P	WD	WD			WD					B*07:19			
B*07:20	P	I	WD			WD		I		WD	B*07:20	WD	WD	
B*07:22	P	I	WD			WD				I	B*07:22			
B*07:23	P	WD	WD			WD				WD	B*07:23	WD	WD	
B*07:24	P	WD	WD			WD					B*07:24		WD	
B*07:26	P	I	WD		WD	WD		I		WD	B*07:26	WD	WD	
B*07:29	P	WD	WD								B*07:29			
B*07:31	P	WD	WD			WD					B*07:31		WD	
B*07:33	P	WD	WD		WD	WD					B*07:33		WD	
B*07:36	P	WD	WD		WD	WD				WD	B*07:36	WD	WD	
B*07:37	P	I	WD			WD				I	B*07:37	WD	WD	
B*07:38	P	WD	WD			WD					B*07:38		WD	
B*07:42	P	WD	WD			WD				WD	B*07:42	WD		

Supplementary Table 3b. Comparison of HLA-B P group two-field assignments among CIWD catalogs [a]

		3.0.0 CIWD									2.0.0 CWD		EFI CWD	China CWD
		Category by Population [b]												
**HLA-B IPD-HLA/IMGT 3.31.0**	**Nomenclature Designation [a]**	**Highest Frequency**	Total	**AFA**	**API**	**EURO**	**MENA**	**HIS**	**NAM**	**UNK**	**HLA-B IPD-IMGT/HLA 3.9.0**	**Category**	**Category**	**Category**
B*07:43	P	o [c]	o								B*07:43	WD		
B*07:46	P	I	WD			WD				I	B*07:46	WD	WD	
B*07:47	P	I	I			I				WD	B*07:47		WD	
B*07:48	P	WD	WD			WD	WD				B*07:48		WD	WD
B*07:51	P	I	WD			WD		I			B*07:51	WD		
B*07:53	P	WD	WD			WD					B*07:53			
B*07:56	P	WD	WD							WD	B*07:56			
B*07:57	P	WD	WD			WD					B*07:57		WD	
B*07:62	P	WD	WD			WD					B*07:62		WD	
B*07:63	P	WD	WD			WD					B*07:63		WD	
B*07:67N	N	WD	WD			WD					B*07:67N		WD	
B*07:68	P	WD	WD			WD					B*07:68		WD	
B*07:71	P	WD	WD								B*07:71			
B*07:73	P	WD	WD			WD					B*07:73		WD	
B*07:74	P	WD	WD			WD					B*07:74		WD	
B*07:77	P	WD	WD				WD				B*07:77			
B*07:80	P	WD	WD		WD						B*07:80			
B*07:81	P	WD	WD		WD						B*07:81			WD
B*07:85	P	WD	WD								B*07:85			
B*07:86	P	WD	WD			WD					B*07:86		WD	
B*07:87	P	WD	WD			WD					B*07:87		WD	
B*07:88	P	WD	WD			WD					B*07:88		WD	
B*07:90	P	o	o								B*07:90		WD	
B*07:91	P	o	o								B*07:91			WD
B*07:92	P	WD	WD			WD					B*07:92		WD	
B*07:94	P	WD	WD								B*07:94			
B*07:95	P	WD	WD			WD					B*07:95		WD	
B*07:96	P	WD	WD			WD					B*07:96		WD	

Supplementary Table 3b. Comparison of HLA-B P group two-field assignments among CIWD catalogs [a]

		3.0.0 CIWD									2.0.0 CWD		EFI CWD	China CWD
		Category by Population [b]												
HLA-B IPD-HLA/IMGT 3.31.0	Nomenclature Designation [a]	Highest Frequency	Total	AFA	API	EURO	MENA	HIS	NAM	UNK	HLA-B IPD-IMGT/HLA 3.9.0	Category	Category	Category
B*07:98	P	WD	WD			WD					B*07:98		WD	
B*07:102	P	WD	WD			WD					B*07:102		WD	
B*07:103	P	WD	WD	WD							B*07:103			
B*07:104	P	WD	WD								B*07:104			
B*07:105	P	WD	WD		WD						B*07:105			
B*07:106	P	WD	WD				WD				B*07:106			
B*07:109	P	WD	WD			WD					B*07:109		WD	
B*07:110	P	WD	WD			WD					B*07:110		WD	
B*07:112	P	WD	WD			WD					B*07:112			
B*07:113	P	WD	WD			WD					B*07:113			
B*07:116	P	o	o								B*07:116			WD
B*07:122	P	WD	WD			WD					B*07:122			
B*07:125	P	o	o								B*07:125		WD	
B*07:131	P	WD	WD			WD					B*07:131			
B*07:132	P	WD	WD			WD					B*07:132			
B*07:136	P	WD	WD			WD					B*07:136			
B*07:143	P	WD	WD				WD				B*07:143			
B*07:151	P	WD	WD			WD					B*07:151			
B*07:158	P	WD	WD								B*07:158			
B*07:165	P	WD	WD			WD					B*07:165		WD	
B*07:181N	N	WD	WD			WD					B*07:181N			
B*07:188	P	WD	WD			WD					B*07:188			
B*07:193	P	WD	WD			WD					B*07:193			
B*07:206	P	WD	WD			WD					B*07:206			
B*07:208	P	WD	WD			WD					B*07:208			
B*07:222	P	WD	WD		WD						B*07:222			
B*07:237	P	WD	WD								B*07:237			
B*08:01	P	C	C	C	C	C	C	C	C	C	B*08:01	C	C	C

Supplementary Table 3b. Comparison of HLA-B P group two-field assignments among CIWD catalogs [a]

		3.0.0 CIWD									2.0.0 CWD		EFI CWD	China CWD
		Category by Population [b]												
HLA-B IPD-HLA/IMGT 3.31.0	Nomenclature Designation [a]	Highest Frequency	Total	AFA	API	EURO	MENA	HIS	NAM	UNK	HLA-B IPD-IMGT/HLA 3.9.0	Category	Category	Category
B*08:02	P	WD	WD			WD					B*08:02	WD	WD	
B*08:03	P	WD	WD								B*08:03	WD	WD	
B*08:04	P	I	WD			WD				I	B*08:04	WD	WD	
B*08:05	P	o	o								B*08:05		WD	
B*08:07	P	WD	WD			WD					B*08:07	WD	WD	
B*08:09	P	I	I	WD		I		I		I	B*08:09	WD	WD	
B*08:11	P	WD	WD			WD					B*08:11			
B*08:12	P	C	WD	C		WD				WD	B*08:12		WD	
B*08:13	P	WD	WD			WD				WD	B*08:13	WD		
B*08:14	P	WD	WD			WD					B*08:14		WD	
B*08:15	P	WD	WD			WD					B*08:15		WD	
B*08:16	P	WD	WD	WD							B*08:16			
B*08:18	P	I	I	WD		I		I		I	B*08:18	WD	WD	
B*08:20	P	WD	WD			WD					B*08:20	WD		
B*08:21	P	o	o								B*08:21		WD	
B*08:22	P	WD	WD			WD					B*08:22			
B*08:23	P	WD	WD	WD		WD					B*08:23	WD	WD	
B*08:24	P	I	WD			I					B*08:24		WD	
B*08:25	P	WD	WD			WD					B*08:25		WD	
B*08:26	P	WD	WD							WD	B*08:26			
B*08:28	P	WD	WD			WD					B*08:28		WD	
B*08:31	P	WD	WD			WD					B*08:31			
B*08:33	P	I	WD			WD		I		I	B*08:33		WD	
B*08:34	P	WD	WD			WD					B*08:34		WD	
B*08:35	P	WD	WD			WD					B*08:35	WD	WD	
B*08:36	P	WD	WD								B*08:36			
B*08:37	P	WD	WD			WD					B*08:37		WD	
B*08:39	P	WD	WD			WD					B*08:39		WD	

Supplementary Table 3b. Comparison of HLA-B P group two-field assignments among CIWD catalogs [a]

		3.0.0 CIWD									2.0.0 CWD		EFI CWD	China CWD
		Category by Population [b]												
HLA-B IPD-HLA/IMGT 3.31.0	Nomenclature Designation [a]	Highest Frequency	Total	AFA	API	EURO	MENA	HIS	NAM	UNK	HLA-B IPD-IMGT/HLA 3.9.0	Category	Category	Category
B*08:41	P	WD	WD			WD					B*08:41			
B*08:50	P	WD	WD								B*08:50			
B*08:52	P	WD	WD			WD					B*08:52		WD	
B*08:56	P	WD	WD								B*08:56			
B*08:59	P	I	WD		I						B*08:59			
B*08:63	P	WD	WD			WD					B*08:63		WD	
B*08:71	P	WD	WD			WD					B*08:71			
B*08:75	P	WD	WD			WD					B*08:75			
B*08:78	P	WD	WD			WD				WD	B*08:78			
B*08:88	P	WD	WD			WD					B*08:88			
B*08:97	P	WD	WD			WD					B*08:97		WD	
B*08:104	P	WD	WD			WD					B*08:104			
B*08:110	P	WD	WD			WD					B*08:110			
B*13:01	P	C	C	C	C	C	C	C	C	C	B*13:01	C	C	C
B*13:02	P	C	C	C	C	C	C	C	C	C	B*13:02	C	C	C
B*13:03	P	WD	WD	WD		WD	WD				B*13:03	WD		
B*13:04	P	WD	WD								B*13:04	WD		
B*13:06	P	WD	WD								B*13:06			
B*13:08	P	WD	WD			WD					B*13:08			
B*13:10	P	WD	WD			WD					B*13:10	WD		
B*13:11	P	WD	WD			WD					B*13:11			
B*13:12	P	I	WD		I					WD	B*13:12	WD		
B*13:14	P	WD	WD			WD					B*13:14			
B*13:16	P	o	o								B*13:16			WD
B*13:45	P	WD	WD			WD					B*13:45			
B*13:47	P	WD	WD			WD					B*13:47			
B*13:57	P	WD	WD		WD						B*13:57			
B*14:01	P	C	C	C	C	C	C	C	C	C	B*14:01	C	C	WD

Supplementary Table 3b. Comparison of HLA-B P group two-field assignments among CIWD catalogs [a]

		3.0.0 CIWD									2.0.0 CWD		EFI CWD	China CWD
		Category by Population [b]												
HLA-B IPD-HLA/IMGT 3.31.0	Nomenclature Designation [a]	Highest Frequency	Total	AFA	API	EURO	MENA	HIS	NAM	UNK	HLA-B IPD-IMGT/HLA 3.9.0	Category	Category	Category
B*14:02	P	C	C	C	C	C	C	C	C	C	B*14:02	C	C	C
B*14:03	P	C	I	C	WD	I	C	C	C	C	B*14:03	C	WD	
B*14:05	P	C	WD	C		WD				I	B*14:05	WD		
B*14:06	P	WD	WD			WD				WD	B*14:06		WD	
B*14:07N	N	WD	WD			WD					B*14:07N		WD	
B*14:08	P	WD	WD			WD					B*14:08		WD	
B*14:09	P	WD	WD	WD							B*14:09	WD		
B*14:10	P	WD	WD			WD					B*14:10		WD	
B*14:18	P	o	o								B*14:18		WD	
B*14:19	P	WD	WD			WD					B*14:19		WD	
B*14:20	P	WD	WD			WD					B*14:20			
B*14:23	P	WD	WD								B*14:23			
B*14:29	P	WD	WD			WD					B*14:29			
B*14:32	P	WD	WD			WD					B*14:32			
B*15:01	P	C	C	C	C	C	C	C	C	C	B*15:01	C	C	C
B*15:01N [e]	N	WD	WD			WD					B*15:01N	WD		
B*15:02	P	C	C	C	C	I	C	C	C	C	B*15:02	C	C	C
B*15:03	P	C	C	C	C	C	C	C	C	C	B*15:03	C	WD	WD
B*15:04	P	C	C	C	C	I		C	C	C	B*15:04	WD		
B*15:05	P	C	C	WD	C	WD	WD	C	C	C	B*15:05	WD		C
B*15:06	P	C	I		C	WD		I		I	B*15:06	WD		
B*15:07	P	C	C	C	C	C	WD	C	C	C	B*15:07	C	WD	C
B*15:08	P	C	C	WD	C	C	C	C	C	C	B*15:08	C		WD
B*15:09	P	C	C	WD	I	C	C	C	C	C	B*15:09	C	WD	WD
B*15:10	P	C	C	C	C	C	C	C	C	C	B*15:10	C	C	WD
B*15:11	P	C	C		C	I	C	I		C	B*15:11	C	WD	C
B*15:12	P	C	C		C	WD		I		C	B*15:12	C		C
B*15:13	P	C	C	C	C	I	WD	I		C	B*15:13	C	WD	WD

Supplementary Table 3b. Comparison of HLA-B P group two-field assignments among CIWD catalogs [a]

		3.0.0 CIWD									2.0.0 CWD		EFI CWD	China CWD
		Category by Population [b]												
**HLA-B IPD-HLA/IMGT 3.31.0**	**Nomenclature Designation [a]**	**Highest Frequency**	Total	**AFA**	**API**	**EURO**	**MENA**	**HIS**	**NAM**	**UNK**	**HLA-B IPD-IMGT/HLA 3.9.0**	**Category**	**Category**	**Category**
B*15:14	P	WD	WD			WD				WD	B*15:14	WD		
B*15:15	P	C	C	C	I	I	WD	C	C	C	B*15:15	C		WD
B*15:16	P	C	C	C	I	C	C	C	C	C	B*15:16	C	WD	WD
B*15:17	P	C	C	C	C	C	C	C	C	C	B*15:17	C	C	C
B*15:18	P	C	C	C	C	C	C	C	C	C	B*15:18	C	C	C
B*15:20	P	C	I	WD		I		C	C	C	B*15:20	WD		
B*15:21	P	C	C	WD	C	I	WD	I		C	B*15:21	C		WD
B*15:23	P	I	I	WD		I		I		I	B*15:23	WD		
B*15:24	P	C	C	WD	I	C	C	C	C	C	B*15:24	C	WD	
B*15:25	P	C	C	C	C	I	C	I	C	C	B*15:25	C		C
B*15:27	P	C	C		C	I	WD	C		C	B*15:27	WD		C
B*15:28	P	o	o								B*15:28	WD		
B*15:29	P	C	C		C	I	C	I		C	B*15:29	WD		WD
B*15:30	P	C	C	WD	I	I	WD	C	C	C	B*15:30	C		WD
B*15:31	P	C	I	C		WD	C	I	WD	I	B*15:31	WD		
B*15:32	P	C	I		C	WD	WD	WD		I	B*15:32	C		C
B*15:33	P	C	I			I		I	C	I	B*15:33	WD		
B*15:34	P	I	I		I	I	WD	I		I	B*15:34	C		
B*15:35	P	C	C		C	I	WD	C	C	C	B*15:35	C		WD
B*15:37	P	C	I	C		WD	WD	I	WD	I	B*15:37	WD		
B*15:38	P	C	I		C	WD		I		I	B*15:38	WD		WD
B*15:39	P	C	C	WD	I	C	WD	C	C	C	B*15:39	C		WD
B*15:40	P	C	I	WD	WD	WD		C	C	I	B*15:40	WD		
B*15:42	P	WD	WD			WD					B*15:42	WD		
B*15:44	P	I	WD		I					WD	B*15:44	WD		
B*15:45	P	I	I			I				I	B*15:45	WD		
B*15:46	P	I	WD		I						B*15:46	WD		WD
B*15:47	P	C	I	C		WD	WD	I	C	I	B*15:47	WD		

Supplementary Table 3b. Comparison of HLA-B P group two-field assignments among CIWD catalogs [a]

		3.0.0 CIWD									2.0.0 CWD		EFI CWD	China CWD
		Category by Population [b]												
HLA-B IPD-HLA/IMGT 3.31.0	Nomenclature Designation [a]	Highest Frequency	Total	AFA	API	EURO	MENA	HIS	NAM	UNK	HLA-B IPD-IMGT/HLA 3.9.0	Category	Category	Category
B*15:48	P	C	I			WD		C	C	WD	B*15:48	WD		
B*15:52	P	C	WD	C						I	B*15:52	WD		
B*15:53	P	WD	WD								B*15:53	WD		
B*15:54	P	C	WD	C						WD	B*15:54	WD		
B*15:55	P	I	WD				WD	I			B*15:55	WD		
B*15:56	P	WD	WD		WD						B*15:56	WD		
B*15:57	P	WD	WD			WD					B*15:57			
B*15:58	P	I	WD		I					WD	B*15:58	WD		WD
B*15:62	P	WD	WD	WD							B*15:62			
B*15:63	P	C	WD			WD		I	C	I	B*15:63	WD		
B*15:64	P	I	WD		I						B*15:64			
B*15:65	P	WD	WD			WD		WD		WD	B*15:65	WD		WD
B*15:67	P	WD	WD	WD			WD			WD	B*15:67			
B*15:68	P	o	o								B*15:68			WD
B*15:69	P	WD	WD							WD	B*15:69			
B*15:70	P	WD	WD			WD		WD			B*15:70			
B*15:71	P	I	I			I	WD	WD		I	B*15:71	WD		
B*15:72	P	WD	WD		WD						B*15:72			
B*15:73	P	I	I			I	WD			I	B*15:73	WD		
B*15:75	P	C	I		C		WD				B*15:75	WD		
B*15:76	P	WD	WD			WD					B*15:76			
B*15:78	P	WD	WD			WD					B*15:78			WD
B*15:79N	N	WD	WD			WD					B*15:79N		WD	
B*15:80	P	WD	WD								B*15:80			
B*15:82	P	I	I			I				I	B*15:82	WD		
B*15:83	P	WD	WD	WD							B*15:83			
B*15:86	P	o	o								B*15:86			WD
B*15:87	P	WD	WD								B*15:87			

Supplementary Table 3b. Comparison of HLA-B P group two-field assignments among CIWD catalogs [a]

		3.0.0 CIWD									2.0.0 CWD		EFI CWD	China CWD
		Category by Population [b]												
HLA-B IPD-HLA/IMGT 3.31.0	Nomenclature Designation [a]	Highest Frequency	Total	AFA	API	EURO	MENA	HIS	NAM	UNK	HLA-B IPD-IMGT/HLA 3.9.0	Category	Category	Category
B*15:88	P	WD	WD		WD						B*15:88			WD
B*15:89	P	WD	WD		WD						B*15:89			
B*15:96	P	WD	WD			WD					B*15:96			
B*15:98	P	WD	WD			WD					B*15:98			
B*15:106	P	WD	WD		WD						B*15:106			
B*15:108	P	WD	WD			WD		WD			B*15:108			
B*15:110	P	I	WD					I			B*15:110			
B*15:113	P	I	WD		I						B*15:113	WD		
B*15:117	P	WD	WD			WD					B*15:117			
B*15:122	P	WD	WD			WD					B*15:122			
B*15:123	P	WD	WD				WD				B*15:123			
B*15:125	P	I	I			I					B*15:125	WD		
B*15:128	P	WD	WD			WD					B*15:128			
B*15:132	P	WD	WD			WD				WD	B*15:132	WD		
B*15:134	P	o	o								B*15:134			WD
B*15:135	P	I	WD			I				WD	B*15:135			
B*15:141	P	I	WD		I					I	B*15:141	WD		
B*15:142	P	WD	WD			WD					B*15:142			
B*15:147	P	WD	WD			WD				WD	B*15:147			
B*15:150	P	I	WD					I			B*15:150			
B*15:152	P	o	o								B*15:152			WD
B*15:153	P	WD	WD			WD				WD	B*15:153			
B*15:154	P	WD	WD			WD					B*15:154			
B*15:157	P	WD	WD			WD					B*15:157			
B*15:158	P	WD	WD	WD							B*15:158			
B*15:159	P	WD	WD		WD						B*15:159			
B*15:160	P	WD	WD			WD					B*15:160			
B*15:162	P	I	WD				WD			I	B*15:162			

Supplementary Table 3b. Comparison of HLA-B P group two-field assignments among CIWD catalogs [a]

		3.0.0 CIWD									2.0.0 CWD		EFI CWD	China CWD
		Category by Population [b]												
HLA-B IPD-HLA/IMGT 3.31.0	Nomenclature Designation [a]	Highest Frequency	Total	AFA	API	EURO	MENA	HIS	NAM	UNK	HLA-B IPD-IMGT/HLA 3.9.0	Category	Category	Category
B*15:164	P	WD	WD			WD					B*15:164			
B*15:167	P	WD	WD			WD					B*15:167			
B*15:170	P	I	WD		I						B*15:170			
B*15:172	P	WD	WD			WD					B*15:172			
B*15:173	P	WD	WD			WD					B*15:173			
B*15:174	P	WD	WD			WD					B*15:174			
B*15:175	P	WD	WD								B*15:175			
B*15:178	P	o	o								B*15:178			WD
B*15:181N	N	WD	WD			WD					B*15:181N		WD	
B*15:183	P	WD	WD			WD					B*15:183			
B*15:190N	N	WD	WD			WD					B*15:190N			
B*15:192	P	o	o								B*15:192			WD
B*15:204	P	WD	WD		WD						B*15:204			
B*15:206	P	o	o								B*15:206		WD	
B*15:207	P	WD	WD			WD					B*15:207		WD	
B*15:208	P	WD	WD			WD					B*15:208		WD	
B*15:211	P	WD	WD			WD					B*15:211			
B*15:237	P	WD	WD			WD					B*15:237			
B*15:239	P	WD	WD			WD					B*15:239		WD	
B*15:240	P	I	WD		I						B*15:240			
B*15:248	P	WD	WD			WD					B*15:248			
B*15:290	P	I	WD		I						B*15:290			
B*15:319	P	WD	WD		WD						B*15:319			
B*15:335	P	WD	WD		WD						B*15:335			
B*18:01	P	C	C	C	C	C	C	C	C	C	B*18:01	C	C	C
B*18:02	P	C	C		C	WD	WD			C	B*18:02	C	WD	WD
B*18:03	P	C	C	C	I	C	C	C	C	C	B*18:03	C	C	WD
B*18:04	P	C	I	WD		I	WD	C	C	I	B*18:04	WD	C	

Supplementary Table 3b. Comparison of HLA-B P group two-field assignments among CIWD catalogs [a]

		3.0.0 CIWD									2.0.0 CWD		EFI CWD	China CWD
		Category by Population [b]												
HLA-B IPD-HLA/IMGT 3.31.0	Nomenclature Designation [a]	Highest Frequency	Total	AFA	API	EURO	MENA	HIS	NAM	UNK	HLA-B IPD-IMGT/HLA 3.9.0	Category	Category	Category
B*18:05	P	C	C	WD	I	C	C	C	C	C	B*18:05	WD	C	
B*18:06	P	I	WD			WD	WD	I		WD	B*18:06	WD	WD	
B*18:07	P	I	I			I	WD			WD	B*18:07		WD	
B*18:08	P	WD	WD			WD				WD	B*18:08	WD	WD	
B*18:09	P	WD	WD			WD					B*18:09		WD	
B*18:10	P	WD	WD								B*18:10			
B*18:11	P	WD	WD			WD				WD	B*18:11	WD	WD	
B*18:12	P	WD	WD			WD				WD	B*18:12	WD	WD	
B*18:13	P	I	WD			WD				I	B*18:13	WD		
B*18:14	P	I	I			I		WD		WD	B*18:14	WD	WD	
B*18:15	P	WD	WD			WD				WD	B*18:15		WD	
B*18:18	P	I	I			I				I	B*18:18	WD	C	
B*18:19	P	WD	WD			WD	WD				B*18:19			
B*18:20	P	WD	WD			WD		WD		WD	B*18:20	WD	WD	
B*18:22	P	WD	WD		WD						B*18:22			
B*18:25	P	C	WD	C	I	WD				I	B*18:25	WD		
B*18:28	P	WD	WD			WD					B*18:28		WD	
B*18:30	P	WD	WD			WD					B*18:30			
B*18:32	P	WD	WD								B*18:32			
B*18:33	P	WD	WD			WD					B*18:33	WD	WD	
B*18:34	P	WD	WD			WD					B*18:34		WD	
B*18:36	P	WD	WD			WD					B*18:36			
B*18:37	P	WD	WD								B*18:37			
B*18:40	P	WD	WD			WD					B*18:40			
B*18:43	P	WD	WD			WD					B*18:43		WD	
B*18:44	P	WD	WD			WD					B*18:44			
B*18:49	P	WD	WD			WD					B*18:49		WD	
B*18:60	P	WD	WD			WD					B*18:60			

Supplementary Table 3b. Comparison of HLA-B P group two-field assignments among CIWD catalogs [a]

		3.0.0 CIWD									2.0.0 CWD		EFI CWD	China CWD
		Category by Population [b]												
HLA-B IPD-HLA/IMGT 3.31.0	Nomenclature Designation [a]	Highest Frequency	Total	AFA	API	EURO	MENA	HIS	NAM	UNK	HLA-B IPD-IMGT/HLA 3.9.0	Category	Category	Category
B*18:68	P	WD	WD	WD							B*18:68			
B*18:69	P	WD	WD				WD				B*18:69			
B*18:73	P	WD	WD								B*18:73			
B*18:95	P	WD	WD								B*18:95			
B*18:114	P	WD	WD		WD						B*18:114			
B*27:01	P	I	I			I				WD	B*27:01	WD	WD	WD
B*27:02	P	C	C	C	C	C	C	C	C	C	B*27:02	C	C	WD
B*27:03	P	C	C	C	I	C	C	C	C	C	B*27:03	C	C	WD
B*27:04	P	C	C	WD	C	I	C	I	C	C	B*27:04	C	WD	C
B*27:05	P	C	C	C	C	C	C	C	C	C	B*27:05	C	C	C
B*27:06	P	C	C	C	C	WD	WD	I		C	B*27:06	C	WD	WD
B*27:07	P	C	C	C	C	C	C	C	C	C	B*27:07	C	WD	C
B*27:08	P	C	I	WD		I	WD	C	WD	I	B*27:08	C		
B*27:09	P	C	C		I	C	WD	I		I	B*27:09	WD		
B*27:10	P	I	I	WD		I		I		I	B*27:10	WD		
B*27:12	P	C	I	WD		I	C	C		C	B*27:12	WD		
B*27:14	P	C	C	WD	I	C	WD	I		I	B*27:14	WD		WD
B*27:15	P	I	WD		I						B*27:15	WD		WD
B*27:17	P	WD	WD	WD		WD				WD	B*27:17	WD		
B*27:18	P	WD	WD		WD						B*27:18			
B*27:19	P	WD	WD			WD	WD				B*27:19	WD		
B*27:20	P	I	WD		I						B*27:20	WD		
B*27:23	P	WD	WD								B*27:23			
B*27:24	P	WD	WD		WD						B*27:24	WD		WD
B*27:25	P	WD	WD		WD	WD					B*27:25			WD
B*27:26	P	WD	WD	WD							B*27:26			
B*27:27	P	I	WD					I			B*27:27	WD		
B*27:30	P	WD	WD			WD					B*27:30			

Supplementary Table 3b. Comparison of HLA-B P group two-field assignments among CIWD catalogs [a]

		3.0.0 CIWD									2.0.0 CWD		EFI CWD	China CWD
		Category by Population [b]												
HLA-B IPD-HLA/IMGT 3.31.0	Nomenclature Designation [a]	Highest Frequency	Total	AFA	API	EURO	MENA	HIS	NAM	UNK	HLA-B IPD-IMGT/HLA 3.9.0	Category	Category	Category
B*27:32	P	WD	WD			WD					B*27:32			
B*27:34	P	WD	WD			WD					B*27:34			
B*27:35	P	WD	WD			WD				WD	B*27:35			
B*27:36	P	o	o								B*27:36			WD
B*27:41	P	WD	WD							WD	B*27:41			
B*27:42	P	WD	WD			WD				WD	B*27:42			
B*27:43	P	WD	WD		WD						B*27:43			
B*27:46	P	WD	WD								B*27:46			
B*27:47	P	WD	WD			WD					B*27:47			
B*27:48	P	WD	WD			WD					B*27:48			
B*27:49	P	WD	WD			WD					B*27:49			
B*27:51	P	I	WD			I					B*27:51			
B*27:53	P	WD	WD			WD					B*27:53			
B*27:56	P	WD	WD			WD					B*27:56			
B*27:60	P	WD	WD			WD					B*27:60			
B*27:61	P	WD	WD		WD						B*27:61			WD
B*27:70	P	WD	WD			WD					B*27:70		WD	
B*27:73	P	WD	WD			WD					B*27:73		WD	
B*27:80	P	WD	WD								B*27:80			
B*27:82	P	WD	WD			WD					B*27:82			
B*27:83	P	WD	WD			WD					B*27:83		WD	
B*27:84	P	WD	WD								B*27:84			
B*27:85	P	WD	WD			WD					B*27:85		WD	
B*27:90	P	WD	WD			WD					B*27:90			
B*35:01	P	C	C	C	C	C	C	C	C	C	B*35:01	C	C	C
B*35:02	P	C	C	C	C	C	C	C	C	C	B*35:02	C	C	C
B*35:03	P	C	C	C	C	C	C	C	C	C	B*35:03	C	C	C
B*35:04	P	C	C	C	WD	I		C	C	C	B*35:04	C	WD	WD

Supplementary Table 3b. Comparison of HLA-B P group two-field assignments among CIWD catalogs [a]

		3.0.0 CIWD									2.0.0 CWD		EFI CWD	China CWD
		Category by Population [b]												
HLA-B IPD-HLA/IMGT 3.31.0	Nomenclature Designation [a]	Highest Frequency	Total	AFA	API	EURO	MENA	HIS	NAM	UNK	HLA-B IPD-IMGT/HLA 3.9.0	Category	Category	Category
B*35:05	P	C	C	C	C	I	C	C	C	C	B*35:05	C	WD	C
B*35:06	P	C	I	WD		WD		C	C	C	B*35:06	WD		
B*35:08	P	C	C	C	C	C	C	C	C	C	B*35:08	C	C	C
B*35:09	P	C	C		WD	I		C	C	C	B*35:09	WD		
B*35:10	P	C	I	WD	WD	WD		C	C	I	B*35:10	WD		
B*35:11	P	C	I	WD	WD	WD		C	C	I	B*35:11	WD		WD
B*35:12	P	C	C	C	I	I	WD	C	C	C	B*35:12	C	WD	
B*35:13	P	I	WD		I		WD				B*35:13	WD	WD	
B*35:14	P	C	C	WD	I	I	WD	C	C	C	B*35:14	C	C	
B*35:15	P	I	WD		I	WD		I		WD	B*35:15	WD	WD	
B*35:16	P	C	I	WD	I	WD		C	C	C	B*35:16	C		
B*35:17	P	C	C	C	I	I	WD	C	C	C	B*35:17	C	WD	
B*35:18	P	I	WD			WD		I		I	B*35:18	WD		
B*35:19	P	C	C			I		C	C	C	B*35:19	C		
B*35:20	P	C	C	WD	I	I		C	C	C	B*35:20	C	WD	
B*35:21	P	C	I		WD	WD		C	C	C	B*35:21	C	WD	
B*35:22	P	C	I	WD		WD		C	C	I	B*35:22	WD	WD	
B*35:23	P	C	I		I	WD		C	C	I	B*35:23	C		
B*35:24	P	C	I			WD		C	C	I	B*35:24	WD		
B*35:25	P	WD	WD			WD					B*35:25	WD		
B*35:26	P	I	WD					I			B*35:26	WD		
B*35:27	P	I	WD			I				I	B*35:27	WD	WD	
B*35:28	P	C	I			WD		C	C	I	B*35:28	WD		WD
B*35:29	P	I	I			I				WD	B*35:29	WD	WD	WD
B*35:30	P	C	I		C	WD		C	C	I	B*35:30	WD	WD	WD
B*35:31	P	C	I			WD		C	C	I	B*35:31	WD	WD	
B*35:32	P	WD	WD	WD		WD				WD	B*35:32	WD		
B*35:33	P	WD	WD			WD					B*35:33			

Supplementary Table 3b. Comparison of HLA-B P group two-field assignments among CIWD catalogs [a]

		3.0.0 CIWD									2.0.0 CWD		EFI CWD	China CWD
		Category by Population [b]												
HLA-B IPD-HLA/IMGT 3.31.0	Nomenclature Designation [a]	Highest Frequency	Total	AFA	API	EURO	MENA	HIS	NAM	UNK	HLA-B IPD-IMGT/HLA 3.9.0	Category	Category	Category
B*35:34	P	C	WD			WD		I	C	WD	B*35:34	WD		
B*35:36	P	I	WD			I	WD				B*35:36			
B*35:37	P	WD	WD		WD	WD					B*35:37	WD		WD
B*35:38	P	WD	WD			WD	WD				B*35:38			
B*35:41	P	I	I	WD		I		I	WD	I	B*35:41	C		
B*35:43	P	C	C	C	I	I	WD	C	C	C	B*35:43	C		
B*35:44	P	I	WD					I			B*35:44			
B*35:45	P	WD	WD			WD					B*35:45			
B*35:47	P	I	WD					I			B*35:47	WD		
B*35:48	P	C	WD					I	C	WD	B*35:48	WD		
B*35:49	P	I	WD	WD				I		WD	B*35:49	WD		
B*35:55	P	I	I			I		I		I	B*35:55	WD		
B*35:58	P	WD	WD								B*35:58			
B*35:59	P	WD	WD	WD				WD		WD	B*35:59			
B*35:61	P	WD	WD			WD					B*35:61			
B*35:64	P	o	o								B*35:64			WD
B*35:65Q	Q	WD	WD			WD					B*35:65Q		WD	
B*35:68	P	I	WD			WD		I			B*35:68		WD	
B*35:72	P	WD	WD		WD						B*35:72			
B*35:74	P	WD	WD								B*35:74		WD	
B*35:77	P	I	WD			WD				I	B*35:77	WD	WD	
B*35:80	P	WD	WD			WD					B*35:80			
B*35:81	P	WD	WD			WD					B*35:81		WD	
B*35:87	P	WD	WD			WD					B*35:87			
B*35:90	P	WD	WD								B*35:90			
B*35:93	P	WD	WD			WD					B*35:93		WD	
B*35:95	P	WD	WD			WD					B*35:95		WD	
B*35:96	P	WD	WD			WD					B*35:96		WD	

Supplementary Table 3b. Comparison of HLA-B P group two-field assignments among CIWD catalogs [a]

		3.0.0 CIWD									2.0.0 CWD		EFI CWD	China CWD
		Category by Population [b]												
HLA-B IPD-HLA/IMGT 3.31.0	Nomenclature Designation [a]	Highest Frequency	Total	AFA	API	EURO	MENA	HIS	NAM	UNK	HLA-B IPD-IMGT/HLA 3.9.0	Category	Category	Category
B*35:100	P	WD	WD			WD					B*35:100			
B*35:101	P	o	o								B*35:101		WD	
B*35:103	P	WD	WD								B*35:103			
B*35:104	P	WD	WD			WD					B*35:104		WD	
B*35:106	P	WD	WD				WD				B*35:106			
B*35:108	P	WD	WD								B*35:108			
B*35:109	P	I	WD		I						B*35:109			
B*35:110	P	o	o								B*35:110		WD	
B*35:112	P	WD	WD			WD					B*35:112		WD	
B*35:115	P	WD	WD			WD					B*35:115			
B*35:116	P	I	WD					I		WD	B*35:116			
B*35:121	P	I	WD					I			B*35:121			
B*35:123	P	WD	WD								B*35:123			
B*35:128	P	WD	WD								B*35:128			
B*35:132	P	I	WD		I						B*35:132			
B*35:136	P	WD	WD			WD					B*35:136			
B*35:139	P	WD	WD								B*35:139			
B*35:157	P	WD	WD			WD					B*35:157			
B*35:158	P	WD	WD			WD	WD				B*35:158			
B*35:163	P	WD	WD			WD					B*35:163			
B*35:165N	N	WD	WD								B*35:165N			
B*35:167	P	WD	WD		WD	WD					B*35:167			
B*35:174	P	WD	WD				WD				B*35:174			
B*35:178	P	WD	WD								B*35:178			
B*35:183	P	WD	WD			WD					B*35:183			
B*35:189	P	o	o								B*35:189			WD
B*35:191	P	WD	WD							WD	B*35:191			
B*35:193	P	WD	WD				WD				B*35:193			

Supplementary Table 3b. Comparison of HLA-B P group two-field assignments among CIWD catalogs [a]

		3.0.0 CIWD									2.0.0 CWD		EFI CWD	China CWD
		Category by Population [b]												
HLA-B IPD-HLA/IMGT 3.31.0	Nomenclature Designation [a]	Highest Frequency	Total	AFA	API	EURO	MENA	HIS	NAM	UNK	HLA-B IPD-IMGT/HLA 3.9.0	Category	Category	Category
B*35:204	P	WD	WD			WD					B*35:204			
B*35:214	P	WD	WD			WD					B*35:214			
B*35:225	P	WD	WD								B*35:225			
B*35:247	P	WD	WD			WD				WD	B*35:247			
B*35:259	P	WD	WD								B*35:259			
B*35:263	P	WD	WD			WD					B*35:263			
B*35:278	P	WD	WD			WD					B*35:278			
B*35:321	P	WD	WD			WD				WD	B*35:321			
B*37:01	P	C	C	C	C	C	C	C	C	C	B*37:01	C	C	C
B*37:02	P	C	I			I	C			C	B*37:02	WD		WD
B*37:03N	N	WD	WD			WD					B*37:03N	WD	WD	
B*37:04	P	C	I		C	I	WD			I	B*37:04			WD
B*37:05	P	WD	WD		WD						B*37:05			
B*37:07	P	WD	WD			WD					B*37:07	WD		
B*37:09	P	WD	WD								B*37:09			
B*37:10	P	WD	WD			WD					B*37:10			
B*37:16Q	Q	WD	WD			WD					B*37:16Q			
B*37:17	P	WD	WD			WD					B*37:17			
B*37:18	P	WD	WD								B*37:18			
B*37:21	P	WD	WD			WD					B*37:21			
B*37:22	P	WD	WD		WD						B*37:22			
B*37:27	P	WD	WD								B*37:27			
B*37:35	P	WD	WD		WD						B*37:35			
B*37:42N	N	WD	WD			WD					B*37:42N			
B*37:48	P	WD	WD		WD						B*37:48			
B*38:01	P	C	C	C	C	C	C	C	C	C	B*38:01	C	C	C
B*38:02	P	C	C	C	C	I	C	C	C	C	B*38:02	C	WD	C
B*38:03	P	I	WD			WD		I			B*38:03			

Supplementary Table 3b. Comparison of HLA-B P group two-field assignments among CIWD catalogs [a]

		3.0.0 CIWD									2.0.0 CWD		EFI CWD	China CWD
		Category by Population [b]												
HLA-B IPD-HLA/IMGT 3.31.0	Nomenclature Designation [a]	Highest Frequency	Total	AFA	API	EURO	MENA	HIS	NAM	UNK	HLA-B IPD-IMGT/HLA 3.9.0	Category	Category	Category
B*38:06	P	WD	WD								B*38:06	WD		
B*38:09	P	C	I			I		WD		C	B*38:09	WD		
B*38:12	P	WD	WD			WD	WD				B*38:12			
B*38:13	P	WD	WD			WD					B*38:13			
B*38:14	P	I	WD			WD				I	B*38:14			
B*38:15	P	WD	WD		WD						B*38:15			WD
B*38:17	P	WD	WD								B*38:17			
B*38:24	P	I	WD			WD				I	B*38:24			
B*38:27	P	WD	WD							WD	B*38:27			
B*38:31	P	WD	WD			WD					B*38:31			
B*38:32	P	WD	WD			WD					B*38:32			
B*38:35	P	I	WD		I					WD	B*38:35			
B*38:37	P	WD	WD			WD					B*38:37			
B*39:01	P	C	C	C	C	C	C	C	C	C	B*39:01	C	C	C
B*39:02	P	C	C	C	I	I		C	C	C	B*39:02	C [d]		
B*39:03	P	C	C	WD		I		C	C	C	B*39:03	C		
B*39:04	P	I	WD		I					I	B*39:04	WD		WD
B*39:05	P	C	C	C	C	C	C	C	C	C	B*39:05	C	C	C
B*39:06	P	C	C	C	C	C	C	C	C	C	B*39:06	C [d]	C	WD
B*39:07	P	I	WD					I		WD	B*39:07	WD		
B*39:08	P	C	C	WD	I	WD		C	C	C	B*39:08	C		
B*39:09	P	C	C	WD	C	I		C	C	C	B*39:09	WD	WD	WD
B*39:10	P	C	C	C	I	C	C	C	C	C	B*39:10	C	C	WD
B*39:11	P	C	I	WD	I	WD		C	C	C	B*39:11	C	WD	
B*39:12	P	C	I			WD		C	C	I	B*39:12	C	WD	
B*39:13	P	C	I	WD		WD		C	C	C	B*39:13	WD	WD	
B*39:14	P	C	I			WD		C	C	I	B*39:14	WD		
B*39:15	P	C	I		C	WD	WD			I	B*39:15	C	WD	WD

Supplementary Table 3b. Comparison of HLA-B P group two-field assignments among CIWD catalogs [a]

		3.0.0 CIWD									2.0.0 CWD		EFI CWD	China CWD
		Category by Population [b]												
HLA-B IPD-HLA/IMGT 3.31.0	Nomenclature Designation [a]	Highest Frequency	Total	AFA	API	EURO	MENA	HIS	NAM	UNK	HLA-B IPD-IMGT/HLA 3.9.0	Category	Category	Category
B*39:19	P	WD	WD			WD		WD			B*39:19			
B*39:20	P	C	I	WD		WD		C		I	B*39:20	WD		
B*39:22	P	I	WD					I		WD	B*39:22	WD		
B*39:24	P	C	C	C	I	C	C	C	C	C	B*39:24	C	WD	WD
B*39:26	P	WD	WD			WD					B*39:26	WD		
B*39:29	P	WD	WD			WD					B*39:29			
B*39:31	P	C	I	WD	I	C	C	I		I	B*39:31	WD		WD
B*39:33	P	WD	WD			WD					B*39:33			
B*39:34	P	WD	WD								B*39:34			
B*39:36	P	o	o								B*39:36			WD
B*39:37	P	I	WD			WD		I		I	B*39:37	WD		
B*39:38Q	Q	o	o								B*39:38Q			WD
B*39:39	P	I	WD					I			B*39:39	WD		
B*39:40N	N	WD	WD			WD					B*39:40N			
B*39:43	P	WD	WD		WD						B*39:43			
B*39:47	P	WD	WD		WD						B*39:47			
B*39:53	P	WD	WD			WD					B*39:53			
B*39:57	P	WD	WD			WD					B*39:57			
B*39:58	P	o	o								B*39:58			WD
B*39:62	P	WD	WD			WD					B*39:62			
B*39:64	P	o	o								B*39:64		WD	
B*39:68	P	WD	WD								B*39:68			
B*39:75	P	WD	WD					WD			B*39:75			
B*39:79	P	WD	WD			WD				WD	B*39:79			
B*39:93	P	WD	WD			WD					B*39:93			
B*40:01	P	C	C	C	C	C	C	C	C	C	B*40:01	C	C	C
B*40:02	P	C	C	C	C	C	C	C	C	C	B*40:02	C	C	C
B*40:03	P	C	I	WD	C	WD		C	C	C	B*40:03	WD		C

Supplementary Table 3b. Comparison of HLA-B P group two-field assignments among CIWD catalogs [a]

		3.0.0 CIWD									2.0.0 CWD		EFI CWD	China CWD
		Category by Population [b]												
HLA-B IPD-HLA/IMGT 3.31.0	Nomenclature Designation [a]	Highest Frequency	Total	AFA	API	EURO	MENA	HIS	NAM	UNK	HLA-B IPD-IMGT/HLA 3.9.0	Category	Category	Category
B*40:04	P	C	C	C	I	I		C	C	C	B*40:04	WD		
B*40:05	P	C	C	WD	I	I		C	C	C	B*40:05	C		
B*40:06	P	C	C	C	C	C	C	C	C	C	B*40:06	C	C	C
B*40:07	P	WD	WD			WD					B*40:07	WD	WD	
B*40:08	P	C	C	WD	I	I		C	C	C	B*40:08	C	WD	
B*40:09	P	I	WD			WD		I		I	B*40:09	WD	WD	
B*40:10	P	C	I		C	WD		WD		C	B*40:10	WD	WD	WD
B*40:11	P	C	I	WD	WD	WD		C	C	C	B*40:11	WD	WD	WD
B*40:12	P	C	I	C		I	C	I		I	B*40:12	WD	WD	
B*40:13	P	C	I			I		C		C	B*40:13	WD		
B*40:14	P	WD	WD			WD				WD	B*40:14		WD	
B*40:16	P	C	I	C		WD	WD	C	C	I	B*40:16	C	WD	
B*40:18	P	WD	WD			WD					B*40:18		WD	
B*40:19	P	WD	WD			WD					B*40:19	WD	WD	
B*40:20	P	C	I			WD		C	C	I	B*40:20	WD		
B*40:21	P	WD	WD								B*40:21			
B*40:22N	N	WD	WD								B*40:22N	WD		
B*40:23	P	C	I		C	WD				I	B*40:23	WD	WD	
B*40:25	P	o	o								B*40:25	WD	WD	WD
B*40:26	P	C	I		C	WD				WD	B*40:26	WD		
B*40:27	P	C	I	WD	I	WD		C	C	C	B*40:27	C		
B*40:30	P	WD	WD			WD					B*40:30	WD		
B*40:31	P	WD	WD			WD					B*40:31			
B*40:32	P	I	I			I					B*40:32	WD	WD	
B*40:35	P	WD	WD								B*40:35			
B*40:40	P	I	WD		I					WD	B*40:40	WD		WD
B*40:42	P	WD	WD			WD				WD	B*40:42		WD	
B*40:43	P	WD	WD			WD					B*40:43	WD		WD

Supplementary Table 3b. Comparison of HLA-B P group two-field assignments among CIWD catalogs [a]

		3.0.0 CIWD									2.0.0 CWD		EFI CWD	China CWD
		Category by Population [b]												
HLA-B IPD-HLA/IMGT 3.31.0	Nomenclature Designation [a]	Highest Frequency	Total	AFA	API	EURO	MENA	HIS	NAM	UNK	HLA-B IPD-IMGT/HLA 3.9.0	Category	Category	Category
B*40:44	P	I	WD		I						B*40:44			
B*40:47	P	WD	WD			WD					B*40:47		WD	
B*40:48	P	WD	WD		WD						B*40:48			WD
B*40:49	P	WD	WD			WD					B*40:49		WD	WD
B*40:50	P	C	I		C	WD	WD			I	B*40:50	WD	WD	WD
B*40:52	P	WD	WD			WD					B*40:52			
B*40:53	P	WD	WD								B*40:53			
B*40:61	P	WD	WD		WD						B*40:61			
B*40:63	P	WD	WD			WD					B*40:63			
B*40:64	P	I	WD			WD		I		I	B*40:64	WD	WD	
B*40:69	P	o	o								B*40:69			WD
B*40:70	P	o	o								B*40:70			WD
B*40:72	P	WD	WD			WD					B*40:72		WD	
B*40:74	P	WD	WD								B*40:74			
B*40:75	P	o	o								B*40:75			WD
B*40:78	P	WD	WD		WD		WD				B*40:78			WD
B*40:79	P	WD	WD			WD					B*40:79			
B*40:81	P	o	o								B*40:81			WD
B*40:84	P	WD	WD								B*40:84			WD
B*40:87	P	WD	WD			WD					B*40:87			
B*40:90	P	C	WD	WD		WD			C	WD	B*40:90	WD		
B*40:94	P	WD	WD			WD					B*40:94	WD	WD	
B*40:95	P	I	WD		I						B*40:95			
B*40:99	P	WD	WD								B*40:99			
B*40:102	P	WD	WD			WD	WD				B*40:102		WD	
B*40:103	P	WD	WD				WD				B*40:103			
B*40:108	P	WD	WD			WD					B*40:108		WD	
B*40:110	P	WD	WD		WD						B*40:110			

Supplementary Table 3b. Comparison of HLA-B P group two-field assignments among CIWD catalogs [a]

		3.0.0 CIWD									2.0.0 CWD		EFI CWD	China CWD
		Category by Population [b]												
HLA-B IPD-HLA/IMGT 3.31.0	Nomenclature Designation [a]	Highest Frequency	Total	AFA	API	EURO	MENA	HIS	NAM	UNK	HLA-B IPD-IMGT/HLA 3.9.0	Category	Category	Category
B*40:111	P	WD	WD					WD			B*40:111			
B*40:113	P	WD	WD			WD					B*40:113			
B*40:114	P	WD	WD			WD					B*40:114	WD		
B*40:115	P	I	WD		I						B*40:115			
B*40:117	P	WD	WD								B*40:117			
B*40:119	P	WD	WD								B*40:119			
B*40:122	P	o	o								B*40:122			WD
B*40:125	P	o	o								B*40:125			WD
B*40:126	P	WD	WD								B*40:126			
B*40:129	P	WD	WD					WD			B*40:129	WD		
B*40:130	P	WD	WD		WD	WD					B*40:130			
B*40:135	P	WD	WD								B*40:135			
B*40:140	P	o	o								B*40:140			WD
B*40:142N	N	WD	WD								B*40:142N			
B*40:153	P	o	o								B*40:153			WD
B*40:155N	N	WD	WD		WD						B*40:155N			
B*40:157	P	WD	WD								B*40:157			
B*40:159	P	WD	WD		WD						B*40:159			
B*40:160	P	WD	WD		WD	WD					B*40:160			
B*40:168	P	WD	WD								B*40:168			
B*40:177	P	WD	WD								B*40:177			
B*40:183	P	I	WD		I						B*40:183			
B*40:197	P	WD	WD			WD					B*40:197		WD	
B*40:200	P	WD	WD			WD					B*40:200			
B*40:201	P	WD	WD			WD					B*40:201			
B*40:203	P	WD	WD			WD					B*40:203			
B*40:207	P	o	o								B*40:207			WD
B*40:226	P	WD	WD					WD			B*40:226			

Supplementary Table 3b. Comparison of HLA-B P group two-field assignments among CIWD catalogs [a]

		3.0.0 CIWD									2.0.0 CWD		EFI CWD	China CWD
		Category by Population [b]												
HLA-B IPD-HLA/IMGT 3.31.0	Nomenclature Designation [a]	Highest Frequency	Total	AFA	API	EURO	MENA	HIS	NAM	UNK	HLA-B IPD-IMGT/HLA 3.9.0	Category	Category	Category
B*40:255	P	o	o								B*40:255			WD
B*40:268	P	o	o								B*40:268			WD
B*40:295	P	I	WD		I						B*40:295			
B*40:310	P	WD	WD		WD						B*40:310			
B*40:334	P	I	WD					I		WD	B*40:334			
B*41:01	P	C	C	C	C	C	C	C	C	C	B*41:01	C	C	C
B*41:02	P	C	C	C	C	C	C	C	C	C	B*41:02	C	C	WD
B*41:03	P	C	I	C		WD		I		I	B*41:03	C		
B*41:05	P	WD	WD			WD					B*41:05			
B*41:06	P	I	WD			WD		I			B*41:06			
B*41:07	P	I	WD					I		WD	B*41:07	WD		
B*41:09	P	WD	WD								B*41:09			
B*41:10	P	WD	WD			WD					B*41:10			
B*41:11	P	WD	WD			WD					B*41:11			
B*41:14	P	WD	WD								B*41:14			
B*41:16	P	WD	WD			WD					B*41:16			
B*41:18	P	WD	WD			WD					B*41:18			
B*41:19	P	WD	WD			WD					B*41:19			
B*42:01	P	C	C	C	C	C	C	C	C	C	B*42:01	C	C	WD
B*42:02	P	C	C	C	I	C	C	C	C	C	B*42:02	C	C	WD
B*42:05	P	I	WD			I					B*42:05	WD	WD	
B*42:07	P	WD	WD				WD				B*42:07			
B*42:10	P	WD	WD	WD							B*42:10			
B*42:18	P	I	WD			WD		I		WD	B*42:18			
B*44:02	P	C	C	C	C	C	C	C	C	C	B*44:02	C	C	C
B*44:03	P	C	C	C	C	C	C	C	C	C	B*44:03	C	C	C
B*44:04	P	C	C	C	WD	C	WD	C	C	C	B*44:04	C	WD	
B*44:05	P	C	C	C	C	C	C	C	C	C	B*44:05	C	C	WD

Supplementary Table 3b. Comparison of HLA-B P group two-field assignments among CIWD catalogs [a]

		3.0.0 CIWD									2.0.0 CWD		EFI CWD	China CWD
		Category by Population [b]												
HLA-B IPD-HLA/IMGT 3.31.0	Nomenclature Designation [a]	Highest Frequency	Total	AFA	API	EURO	MENA	HIS	NAM	UNK	HLA-B IPD-IMGT/HLA 3.9.0	Category	Category	Category
B*44:06	P	I	I		I	I	WD	I		I	B*44:06	WD		
B*44:07	P	C	WD	C						I	B*44:07	WD		
B*44:08	P	I	I	WD		I		WD		I	B*44:08	WD		
B*44:09	P	I	I			I				I	B*44:09	WD		
B*44:10	P	C	I	C		WD		C	C	I	B*44:10	C		
B*44:12	P	o	o								B*44:12	WD		
B*44:13	P	WD	WD			WD				WD	B*44:13			
B*44:15	P	C	WD	C		WD				WD	B*44:15	WD		
B*44:17	P	I	WD			WD	WD			I	B*44:17			
B*44:18	P	WD	WD	WD							B*44:18	WD		
B*44:20	P	WD	WD			WD				WD	B*44:20	WD		
B*44:21	P	I	WD			I	WD	WD		WD	B*44:21	WD		
B*44:22	P	WD	WD			WD					B*44:22	WD		
B*44:23N	N	WD	WD			WD					B*44:23N	WD		
B*44:24	P	WD	WD			WD					B*44:24	WD		
B*44:25	P	WD	WD			WD					B*44:25	WD		
B*44:28	P	WD	WD			WD					B*44:28			
B*44:29	P	C	I		WD	I	C	I		I	B*44:29	WD		WD
B*44:30	P	I	WD					I			B*44:30			
B*44:31	P	WD	WD				WD				B*44:31			
B*44:32	P	WD	WD			WD					B*44:32			
B*44:34	P	WD	WD			WD					B*44:34			
B*44:36	P	WD	WD			WD				WD	B*44:36			
B*44:37	P	WD	WD		WD	WD					B*44:37			
B*44:41	P	WD	WD			WD					B*44:41			
B*44:43	P	WD	WD			WD					B*44:43			
B*44:45	P	WD	WD			WD					B*44:45			
B*44:48	P	WD	WD			WD					B*44:48			

Supplementary Table 3b. Comparison of HLA-B P group two-field assignments among CIWD catalogs [a]

		3.0.0 CIWD									2.0.0 CWD		EFI CWD	China CWD
		Category by Population [b]												
HLA-B IPD-HLA/IMGT 3.31.0	Nomenclature Designation [a]	Highest Frequency	Total	AFA	API	EURO	MENA	HIS	NAM	UNK	HLA-B IPD-IMGT/HLA 3.9.0	Category	Category	Category
B*44:50	P	WD	WD								B*44:50	WD		
B*44:60	P	WD	WD								B*44:60			
B*44:64	P	WD	WD			WD					B*44:64		WD	
B*44:67	P	WD	WD			WD					B*44:67		WD	
B*44:68	P	WD	WD			WD					B*44:68		WD	
B*44:73	P	WD	WD			WD					B*44:73			
B*44:74	P	WD	WD			WD					B*44:74		WD	
B*44:76	P	WD	WD			WD					B*44:76		WD	
B*44:77	P	WD	WD			WD					B*44:77		WD	
B*44:79	P	o	o								B*44:79		WD	
B*44:80	P	WD	WD			WD					B*44:80		WD	
B*44:83	P	WD	WD								B*44:83			
B*44:84	P	WD	WD								B*44:84			
B*44:86	P	WD	WD			WD					B*44:86		WD	
B*44:87	P	I	WD		I						B*44:87			
B*44:88	P	WD	WD			WD					B*44:88		WD	
B*44:91	P	WD	WD			WD					B*44:91		WD	
B*44:92	P	WD	WD			WD					B*44:92		WD	
B*44:96	P	WD	WD			WD					B*44:96		WD	
B*44:98	P	WD	WD							WD	B*44:98			
B*44:99	P	WD	WD								B*44:99			
B*44:104	P	WD	WD			WD					B*44:104			
B*44:107	P	WD	WD			WD					B*44:107		WD	
B*44:111	P	WD	WD			WD					B*44:111			
B*44:115	P	WD	WD			WD					B*44:115		WD	
B*44:116	P	WD	WD			WD					B*44:116			
B*44:120	P	WD	WD			WD					B*44:120			
B*44:122	P	WD	WD			WD					B*44:122		WD	

Supplementary Table 3b. Comparison of HLA-B P group two-field assignments among CIWD catalogs [a]

		3.0.0 CIWD									2.0.0 CWD		EFI CWD	China CWD
		Category by Population [b]												
**HLA-B IPD-HLA/IMGT 3.31.0**	**Nomenclature Designation [a]**	**Highest Frequency**	Total	**AFA**	**API**	**EURO**	**MENA**	**HIS**	**NAM**	**UNK**	**HLA-B IPD-IMGT/HLA 3.9.0**	**Category**	**Category**	**Category**
B*44:146	P	WD	WD			WD					B*44:146		WD	
B*44:166	P	WD	WD			WD					B*44:166			
B*44:205	P	WD	WD			WD					B*44:205			
B*45:01	P	C	C	C	C	C	C	C	C	C	B*45:01	C	C	C
B*45:02	P	WD	WD	WD						WD	B*45:02			
B*46:01	P	C	C	C	C	C	C	C	C	C	B*46:01	C	WD	C
B*46:02	P	o	o								B*46:02	WD		
B*46:09	P	o	o								B*46:09			WD
B*46:18	P	o	o								B*46:18			WD
B*46:19	P	o	o								B*46:19			WD
B*46:22	P	WD	WD		WD						B*46:22			
B*47:01	P	C	C	C	C	C	C	C	C	C	B*47:01	C	C	WD
B*47:02	P	I	I	WD		I				WD	B*47:02	WD	WD	
B*47:03	P	C	I	C		WD	C	WD		I	B*47:03	WD	WD	
B*47:04	P	WD	WD			WD				WD	B*47:04			
B*47:05	P	WD	WD								B*47:05			
B*48:01	P	C	C	C	C	C	C	C	C	C	B*48:01	C	WD	C
B*48:02	P	C	I	C	WD	I		C	C	C	B*48:02	WD	WD	
B*48:03	P	C	C	WD	C	I		C	C	C	B*48:03	C	WD	C
B*48:04	P	C	C		C	WD				I	B*48:04	WD		WD
B*48:05	P	WD	WD	WD			WD				B*48:05	WD		
B*48:06	P	WD	WD					WD			B*48:06	WD		WD
B*48:07	P	C	WD			WD		WD	C	I	B*48:07	WD		
B*48:12	P	I	WD					I			B*48:12	WD		
B*48:13	P	I	WD					I			B*48:13	WD		
B*48:24	P	WD	WD		WD						B*48:24			
B*48:26	P	WD	WD							WD	B*48:26			
B*48:29	P	WD	WD								B*48:29			

Supplementary Table 3b. Comparison of HLA-B P group two-field assignments among CIWD catalogs [a]

		3.0.0 CIWD									2.0.0 CWD		EFI CWD	China CWD
		Category by Population [b]												
HLA-B IPD-HLA/IMGT 3.31.0	Nomenclature Designation [a]	Highest Frequency	Total	AFA	API	EURO	MENA	HIS	NAM	UNK	HLA-B IPD-IMGT/HLA 3.9.0	Category	Category	Category
B*49:01	P	C	C	C	C	C	C	C	C	C	B*49:01	C	C	C
B*49:04	P	WD	WD			WD					B*49:04			
B*49:09	P	WD	WD				WD				B*49:09			
B*49:18	P	WD	WD					WD		WD	B*49:18			
B*50:01	P	C	C	C	C	C	C	C	C	C	B*50:01	C	C	C
B*50:02	P	C	C	C	I	C	C	C	C	C	B*50:02	C	WD	
B*50:04	P	I	WD		I						B*50:04	WD		
B*50:08	P	WD	WD			WD					B*50:08			
B*50:09	P	WD	WD			WD					B*50:09			
B*50:12	P	WD	WD			WD					B*50:12			
B*51:01	P	C	C	C	C	C	C	C	C	C	B*51:01	C	C	C
B*51:11N	N	WD	WD			WD					B*51:11N	WD	WD	
B*51:02	P	C	C	C	C	I	C	C	C	C	B*51:02	C	C	C
B*51:03	P	o	o								B*51:03	WD	WD	
B*51:04	P	I	WD	WD		WD	WD	I		I	B*51:04	WD	WD	WD
B*51:05	P	C	C	WD	I	C	C	C		C	B*51:05	C	C	WD
B*51:06	P	C	C	WD	C	I	WD	I	C	C	B*51:06	C	WD	WD
B*51:07	P	C	C	C	C	C	C	C	C	C	B*51:07	C	C	WD
B*51:08	P	C	C	C	C	C	C	C	C	C	B*51:08	C	C	WD
B*51:09	P	C	C	C	I	C	C	C	C	C	B*51:09	C	WD	WD
B*51:10	P	I	WD		WD	WD		I		WD	B*51:10	WD	WD	
B*51:13	P	C	I			WD		C	C	I	B*51:13	WD	WD	
B*51:14	P	C	I	WD		WD		C	C	I	B*51:14	WD		
B*51:17	P	I	WD		I						B*51:17	WD		
B*51:19	P	C	I		C	WD		I		I	B*51:19	WD	WD	
B*51:20	P	WD	WD			WD					B*51:20		WD	
B*51:21	P	WD	WD			WD					B*51:21	WD	WD	WD
B*51:22	P	C	I		WD	I		C	C	I	B*51:22	WD	WD	WD

Supplementary Table 3b. Comparison of HLA-B P group two-field assignments among CIWD catalogs [a]

		3.0.0 CIWD									2.0.0 CWD		EFI CWD	China CWD
		Category by Population [b]												
HLA-B IPD-HLA/IMGT 3.31.0	Nomenclature Designation [a]	Highest Frequency	Total	AFA	API	EURO	MENA	HIS	NAM	UNK	HLA-B IPD-IMGT/HLA 3.9.0	Category	Category	Category
B*51:23	P	I	WD					I		WD	B*51:23	WD		
B*51:24	P	WD	WD			WD					B*51:24		WD	
B*51:26	P	WD	WD			WD					B*51:26		WD	
B*51:29	P	WD	WD			WD	WD				B*51:29	WD		
B*51:31	P	I	WD			WD		I		I	B*51:31			
B*51:33	P	WD	WD								B*51:33			
B*51:34	P	I	WD		I	WD	WD			WD	B*51:34	WD	WD	WD
B*51:36	P	WD	WD		WD						B*51:36			WD
B*51:37	P	WD	WD							WD	B*51:37			
B*51:38	P	WD	WD			WD					B*51:38		WD	
B*51:39	P	WD	WD		WD						B*51:39			WD
B*51:40	P	WD	WD			WD					B*51:40	WD		
B*51:43	P	I	I			WD				I	B*51:43	WD	WD	
B*51:46	P	WD	WD			WD					B*51:46		WD	
B*51:49	P	I	WD			WD				I	B*51:49	WD	WD	
B*51:50	P	I	WD			I					B*51:50	WD	WD	
B*51:55	P	WD	WD			WD					B*51:55	WD	WD	
B*51:56	P	WD	WD			WD					B*51:56		WD	
B*51:59	P	o	o								B*51:59			WD
B*51:61	P	WD	WD			WD					B*51:61		WD	
B*51:64	P	WD	WD	WD							B*51:64			
B*51:65	P	I	WD			WD	WD	I		WD	B*51:65		WD	
B*51:67	P	WD	WD			WD					B*51:67			
B*51:68	P	WD	WD			WD					B*51:68		WD	
B*51:69	P	WD	WD			WD	WD				B*51:69			
B*51:70	P	WD	WD				WD				B*51:70			
B*51:71	P	WD	WD			WD					B*51:71			
B*51:73	P	WD	WD			WD					B*51:73			

Supplementary Table 3b. Comparison of HLA-B P group two-field assignments among CIWD catalogs [a]

		3.0.0 CIWD									2.0.0 CWD		EFI CWD	China CWD
		Category by Population [b]												
HLA-B IPD-HLA/IMGT 3.31.0	Nomenclature Designation [a]	Highest Frequency	Total	AFA	API	EURO	MENA	HIS	NAM	UNK	HLA-B IPD-IMGT/HLA 3.9.0	Category	Category	Category
B*51:74	P	WD	WD				WD				B*51:74			
B*51:75	P	I	WD			WD		I		WD	B*51:75		WD	
B*51:76	P	WD	WD			WD					B*51:76		WD	
B*51:79	P	WD	WD			WD					B*51:79		WD	
B*51:82	P	WD	WD			WD					B*51:82		WD	
B*51:83	P	WD	WD			WD	WD				B*51:83			
B*51:87	P	WD	WD			WD					B*51:87			
B*51:100	P	WD	WD			WD					B*51:100		WD	
B*51:105	P	WD	WD			WD					B*51:105		WD	
B*51:107	P	WD	WD		WD	WD					B*51:107		WD	
B*51:109	P	WD	WD		WD						B*51:109			
B*51:114	P	WD	WD			WD					B*51:114			
B*51:116	P	WD	WD			WD					B*51:116			
B*51:117	P	WD	WD			WD					B*51:117			
B*51:122	P	WD	WD			WD					B*51:122		WD	
B*51:123	P	WD	WD					WD			B*51:123			
B*51:127	P	WD	WD								B*51:127			
B*51:130	P	o	o								B*51:130			WD
B*51:134	P	WD	WD			WD					B*51:134			
B*51:158	P	I	WD		I	WD					B*51:158			
B*51:177	P	WD	WD			WD					B*51:177			
B*52:01	P	C	C	C	C	C	C	C	C	C	B*52:01	C	C	C
B*52:02	P	I	WD			WD		I			B*52:02	WD		
B*52:03	P	I	WD					I			B*52:03	WD		
B*52:04	P	C	I	WD	C	WD			C	I	B*52:04	WD		WD
B*52:06	P	WD	WD			WD					B*52:06			
B*52:10	P	WD	WD			WD		WD			B*52:10			
B*52:11	P	WD	WD			WD					B*52:11			WD

Supplementary Table 3b. Comparison of HLA-B P group two-field assignments among CIWD catalogs [a]

		3.0.0 CIWD									2.0.0 CWD		EFI CWD	China CWD
		Category by Population [b]												
HLA-B IPD-HLA/IMGT 3.31.0	Nomenclature Designation [a]	Highest Frequency	Total	AFA	API	EURO	MENA	HIS	NAM	UNK	HLA-B IPD-IMGT/HLA 3.9.0	Category	Category	Category
B*52:17	P	WD	WD								B*52:17			
B*52:20	P	o	o								B*52:20			WD
B*52:21	P	WD	WD			WD		WD			B*52:21	WD		
B*52:25	P	WD	WD			WD					B*52:25			
B*52:27	P	WD	WD								B*52:27			
B*52:37	P	WD	WD								B*52:37			
B*53:01	P	C	C	C	C	C	C	C	C	C	B*53:01	C	C	WD
B*53:02	P	o	o								B*53:02	WD		
B*53:04	P	WD	WD			WD					B*53:04	WD		
B*53:05	P	I	I			I	WD			WD	B*53:05	WD		
B*53:06	P	WD	WD			WD					B*53:06	WD		
B*53:09	P	I	WD		I						B*53:09			
B*53:10	P	WD	WD			WD		WD		WD	B*53:10			
B*53:15	P	WD	WD	WD							B*53:15			
B*53:18	P	WD	WD								B*53:18			
B*53:20	P	WD	WD	WD							B*53:20			
B*53:23	P	WD	WD								B*53:23			
B*53:24	P	WD	WD			WD					B*53:24			
B*53:28	P	WD	WD		WD						B*53:28			
B*53:31	P	WD	WD			WD					B*53:31			
B*54:01	P	C	C	WD	C	I	C	I	C	C	B*54:01	C		C
B*54:16	P	o	o								B*54:16			WD
B*55:01	P	C	C	C	C	C	C	C	C	C	B*55:01	C	C	C
B*55:02	P	C	C	WD	C	I	C	I	C	C	B*55:02	C	WD	C
B*55:03	P	I	WD		I						B*55:03			
B*55:04	P	C	I		C	WD				I	B*55:04	WD		WD
B*55:05	P	WD	WD			WD					B*55:05			
B*55:07	P	I	WD		I	WD	WD				B*55:07	WD	WD	WD

Supplementary Table 3b. Comparison of HLA-B P group two-field assignments among CIWD catalogs [a]

		3.0.0 CIWD									2.0.0 CWD		EFI CWD	China CWD
		Category by Population [b]												
HLA-B IPD-HLA/IMGT 3.31.0	Nomenclature Designation [a]	Highest Frequency	Total	AFA	API	EURO	MENA	HIS	NAM	UNK	HLA-B IPD-IMGT/HLA 3.9.0	Category	Category	Category
B*55:09	P	WD	WD			WD					B*55:09		WD	
B*55:11	P	WD	WD			WD		WD			B*55:11		WD	
B*55:12	P	I	WD		I					WD	B*55:12	WD		WD
B*55:14	P	WD	WD			WD					B*55:14		WD	
B*55:16	P	o	o								B*55:16			WD
B*55:17	P	WD	WD			WD					B*55:17		WD	
B*55:18	P	WD	WD								B*55:18			
B*55:20	P	WD	WD			WD					B*55:20		WD	
B*55:21	P	WD	WD				WD				B*55:21			WD
B*55:23	P	WD	WD								B*55:23	WD		
B*55:25	P	WD	WD			WD					B*55:25		WD	
B*55:26	P	o	o								B*55:26			WD
B*55:29	P	WD	WD			WD					B*55:29		WD	
B*55:30	P	o	o								B*55:30			WD
B*55:31	P	WD	WD								B*55:31			
B*55:33	P	WD	WD			WD					B*55:33		WD	
B*55:34	P	o	o								B*55:34			WD
B*55:36	P	o	o								B*55:36		WD	
B*55:43	P	o	o								B*55:43			WD
B*55:45	P	WD	WD			WD					B*55:45		WD	
B*55:50	P	o	o								B*55:50			WD
B*55:78	P	WD	WD		WD						B*55:78			
B*56:01	P	C	C	C	C	C	C	C	C	C	B*56:01	C	C	C
B*56:02	P	C	I		C	WD				C	B*56:02	WD	WD	
B*56:03	P	C	I		C	WD				I	B*56:03	WD	WD	WD
B*56:04	P	C	I		C	WD	WD	WD		C	B*56:04	WD	WD	WD
B*56:05	P	I	WD		I						B*56:05			
B*56:07	P	WD	WD		WD						B*56:07			

Supplementary Table 3b. Comparison of HLA-B P group two-field assignments among CIWD catalogs [a]

		3.0.0 CIWD									2.0.0 CWD		EFI CWD	China CWD
		Category by Population [b]												
HLA-B IPD-HLA/IMGT 3.31.0	Nomenclature Designation [a]	Highest Frequency	Total	AFA	API	EURO	MENA	HIS	NAM	UNK	HLA-B IPD-IMGT/HLA 3.9.0	Category	Category	Category
B*56:09	P	o	o								B*56:09			WD
B*56:10	P	I	WD		I						B*56:10	WD		WD
B*56:13	P	WD	WD			WD					B*56:13			
B*56:14	P	o	o								B*56:14			WD
B*56:15	P	WD	WD			WD		WD			B*56:15	WD	WD	
B*56:16	P	WD	WD			WD					B*56:16	WD	WD	
B*56:18	P	o	o								B*56:18			WD
B*56:21	P	o	o								B*56:21			WD
B*56:22	P	I	WD		I						B*56:22			
B*56:25	P	WD	WD			WD					B*56:25		WD	
B*56:27	P	WD	WD			WD					B*56:27		WD	WD
B*56:33	P	WD	WD			WD					B*56:33			
B*57:01	P	C	C	C	C	C	C	C	C	C	B*57:01	C	C	C
B*57:02	P	C	C	C	I	C	C	C	C	C	B*57:02	C	C	WD
B*57:03	P	C	C	C	C	C	C	C	C	C	B*57:03	C	C	WD
B*57:04	P	C	I	C		WD		I	WD	C	B*57:04	WD	WD	
B*57:05	P	WD	WD	WD							B*57:05	WD		
B*57:08	P	o	o								B*57:08		WD	
B*57:09	P	WD	WD	WD							B*57:09			
B*57:11	P	WD	WD								B*57:11			
B*57:13	P	o	o								B*57:13		WD	
B*57:14	P	WD	WD			WD					B*57:14		WD	
B*57:15	P	WD	WD			WD					B*57:15		WD	
B*57:21	P	WD	WD			WD					B*57:21		WD	
B*57:22	P	WD	WD		WD						B*57:22			
B*57:23	P	WD	WD			WD					B*57:23		WD	
B*57:24	P	WD	WD			WD					B*57:24		WD	
B*57:26	P	WD	WD			WD					B*57:26		WD	

Supplementary Table 3b. Comparison of HLA-B P group two-field assignments among CIWD catalogs [a]

		3.0.0 CIWD									2.0.0 CWD		EFI CWD	China CWD
		Category by Population [b]												
HLA-B IPD-HLA/IMGT 3.31.0	Nomenclature Designation [a]	Highest Frequency	Total	AFA	API	EURO	MENA	HIS	NAM	UNK	HLA-B IPD-IMGT/HLA 3.9.0	Category	Category	Category
B*57:32	P	WD	WD			WD					B*57:32		WD	
B*57:34	P	WD	WD			WD					B*57:34			
B*57:40	P	WD	WD			WD					B*57:40		WD	
B*57:44	P	WD	WD			WD					B*57:44			
B*57:48	P	WD	WD			WD					B*57:48			
B*57:58	P	WD	WD								B*57:58			
B*57:61	P	I	WD		I						B*57:61			
B*58:01	P	C	C	C	C	C	C	C	C	C	B*58:01	C	C	C
B*58:02	P	C	C	C	I	I	C	C	C	C	B*58:02	C	WD	
B*58:04	P	WD	WD	WD							B*58:04	WD		
B*58:05	P	WD	WD			WD					B*58:05			
B*58:06	P	WD	WD								B*58:06	WD		
B*58:08	P	WD	WD		WD						B*58:08			
B*58:12	P	o	o								B*58:12			WD
B*58:16	P	WD	WD			WD					B*58:16			
B*58:18	P	I	WD		I						B*58:18			
B*58:23	P	WD	WD			WD					B*58:23			
B*58:28	P	WD	WD		WD						B*58:28			
B*58:37	P	WD	WD			WD					B*58:37			
B*58:62	P	WD	WD		WD						B*58:62			
B*59:01	P	C	I		C	WD		I		C	B*59:01	C		WD
B*67:01	P	C	C	WD	C	I	WD	C	WD	C	B*67:01	C	WD	C
B*67:02	P	WD	WD								B*67:02	WD		
B*67:03	P	o	o								B*67:03			WD
B*73:01	P	C	C	C	C	C	C	C	C	C	B*73:01	C	C	WD
B*78:01	P	C	C	C	I	I	C	C	C	C	B*78:01	C	C	WD
B*78:02	P	I	I			I		WD		I	B*78:02	WD	WD	WD
B*78:04	P	I	WD					I		WD	B*78:04	WD		

Supplementary Table 3b. Comparison of HLA-B P group two-field assignments among CIWD catalogs [a]

		3.0.0 CIWD									2.0.0 CWD		EFI CWD	China CWD
		Category by Population [b]												
HLA-B IPD-HLA/IMGT 3.31.0	Nomenclature Designation [a]	Highest Frequency	Total	AFA	API	EURO	MENA	HIS	NAM	UNK	HLA-B IPD-IMGT/HLA 3.9.0	Category	Category	Category
B*81:01	P	C	C	C	C	I	C	C	C	C	B*81:01	C	WD	WD
B*81:05	P	WD	WD	WD							B*81:05			
B*82:01	P	C	I	C		WD		C	C	C	B*82:01	C	WD	
B*82:02	P	C	I	C		WD	C	WD		I	B*82:02	WD	WD	
B*83:01	P	WD	WD			WD					B*83:01			

C, common; I, intermediate; WD, well-documented

[a] Alleles observed at least five times in any population group in the current dataset are included in this table. The exception are alleles that were observed in previous catalogs but that are not present or are observed less than five times. Note that this two-field designation is the equivalent of a "P" assignment so that alleles within a G group that encode proteins that differ outside of the antigen recognition domain are not listed separately (e.g., B*81:02 is within the B*81:01P group assignment and is not listed in this table even though it was categorized as well-documented in 2.0.0 CWD). The table does not list all alleles from IPD-IMGT/HLA version 3.31.0.

[b] Population groups include: AFA (African/African American), API (Asian/Pacific Islander), EURO (European/European descent), MENA (Middle East/North Coast of Africa), HIS (Hispanic), NAM (Native American) and UNK (unknown, multiple ancestries or other). Total is the overall population i.e., all groups combined. Highest frequency is the highest CIWD designation among all the individual groups.

[c] Not-CIWD, o.

[d] CWD 2.0.0 lists two alleles, B*39:06:01 (WD) and B*39:06:02 (common). The category reported is the most frequent listed.

[e] Any non-expressed alleles which are excluded from the P group are listed. For example, B*15:01:01:02N (sometimes listed by registries as B*15:01N) is within the B*15:01:01G group but is listed separately because it is not expressed.

Supplementary Table 3c. Comparison of HLA-C P group two-field assignments among CIWD catalogs [a]

		3.0.0 CIWD									2.0.0 CWD		EFI CWD	China CWD
		Category by Population [b]												
HLA-C IPD-HLA/IMGT 3.31.0	Nomenclature Designation [a]	Highest Frequency	Total	AFA	API	EURO	MENA	HIS	NAM	UNK	HLA-C IPD-IMGT/HLA 3.9.0	Category	Category	Category
C*01:02	P	C	C	C	C	C	C	C	C	C	C*01:02	C	C	C
C*01:03	P	C	I		C	WD	C			I	C*01:03	WD	WD	C
C*01:04	P	WD	WD		WD						C*01:04			
C*01:06	P	I	WD		I	WD				WD	C*01:06	WD		WD
C*01:07	P	I	I			I				WD	C*01:07	WD	WD	
C*01:08	P	C	I		C					WD	C*01:08	WD		WD
C*01:10	P	I	WD			WD		I		WD	C*01:10	WD		WD
C*01:12	P	WD	WD			WD					C*01:12			
C*01:13	P	WD	WD			WD					C*01:13			
C*01:14	P	WD	WD								C*01:14			
C*01:17	P	C	I		C	WD	WD				C*01:17	WD	WD	WD
C*01:22	P	I	WD			WD		I		WD	C*01:22		WD	WD
C*01:27	P	WD	WD			WD				WD	C*01:27	WD		
C*01:30	P	WD	WD								C*01:30			WD
C*01:32	P	I	I			I				WD	C*01:32		WD	
C*01:33	P	WD	WD								C*01:33		WD	
C*01:45	P	WD	WD			WD					C*01:45		WD	
C*01:50	P	o [c]	o								C*01:50			WD
C*01:51	P	WD	WD								C*01:51			
C*01:52	P	WD	WD			WD					C*01:52		WD	
C*01:54	P	WD	WD			WD					C*01:54			
C*01:58	P	WD	WD			WD	WD				C*01:58		WD	WD
C*01:59	P	WD	WD			WD					C*01:59		WD	
C*01:62	P	WD	WD			WD					C*01:62			
C*01:63	P	o	o								C*01:63		WD	
C*01:67	P	WD	WD								C*01:67			
C*01:70	P	WD	WD			WD					C*01:70			
C*01:74	P	WD	WD			WD					C*01:74			

Supplementary Table 3c. Comparison of HLA-C P group two-field assignments among CIWD catalogs [a]

		3.0.0 CIWD									2.0.0 CWD		EFI CWD	China CWD
		Category by Population [b]												
HLA-C IPD-HLA/IMGT 3.31.0	Nomenclature Designation [a]	Highest Frequency	Total	AFA	API	EURO	MENA	HIS	NAM	UNK	HLA-C IPD-IMGT/HLA 3.9.0	Category	Category	Category
C*01:96	P	WD	WD								C*01:96			
C*01:103	P	WD	WD			WD					C*01:103			
C*01:121Q	Q	WD	WD								C*01:121Q			
C*02:02	P	C	C	C	C	C	C	C	C	C	C*02:02	C	C	C
C*02:03	P	WD	WD			WD					C*02:03			
C*02:04	P	WD	WD			WD			WD		C*02:04	WD	WD	
C*02:05	P	WD	WD								C*02:05			
C*02:06	P	I	I			I		I		I	C*02:06	WD	WD	
C*02:07	P	I	I			I	WD	I		I	C*02:07	WD	WD	
C*02:08	P	I	I		I	WD	WD	I		WD	C*02:08	WD	WD	
C*02:12	P	WD	WD	WD							C*02:12			
C*02:13	P	WD	WD			WD					C*02:13			
C*02:14	P	C	I	C		WD		I		I	C*02:14	WD		
C*02:16	P	WD	WD	WD		WD					C*02:16			
C*02:19	P	I	WD			WD				I	C*02:19	WD		
C*02:21	P	I	WD			WD	WD	I		I	C*02:21			
C*02:23	P	WD	WD			WD					C*02:23			
C*02:24	P	WD	WD			WD					C*02:24			
C*02:25Q	Q	WD	WD			WD					C*02:25Q		WD	
C*02:26	P	I	WD			WD				I	C*02:26			
C*02:27	P	I	WD	WD		WD		I		WD	C*02:27			
C*02:30	P	WD	WD			WD					C*02:30			
C*02:31	P	WD	WD			WD					C*02:31			
C*02:32	P	WD	WD			WD					C*02:32			
C*02:34	P	WD	WD			WD					C*02:34			
C*02:35	P	WD	WD							WD	C*02:35			
C*02:38N	N	WD	WD			WD					C*02:38N		WD	
C*02:40	P	WD	WD			WD					C*02:40			

Supplementary Table 3c. Comparison of HLA-C P group two-field assignments among CIWD catalogs [a]

		3.0.0 CIWD									2.0.0 CWD		EFI CWD	China CWD
		Category by Population [b]												
HLA-C IPD-HLA/IMGT 3.31.0	Nomenclature Designation [a]	Highest Frequency	Total	AFA	API	EURO	MENA	HIS	NAM	UNK	HLA-C IPD-IMGT/HLA 3.9.0	Category	Category	Category
C*02:43	P	WD	WD			WD					C*02:43			
C*02:44	P	WD	WD			WD					C*02:44			
C*02:45	P	WD	WD								C*02:45			
C*02:46	P	WD	WD			WD		WD			C*02:46			
C*02:48	P	WD	WD			WD					C*02:48			
C*02:49	P	WD	WD	WD							C*02:49			
C*02:55	P	WD	WD			WD					C*02:55			
C*02:56	P	WD	WD			WD					C*02:56			
C*02:67Q	Q	WD	WD			WD					C*02:67Q			
C*02:87	P	WD	WD			WD					C*02:87			
C*02:92N	N	WD	WD			WD					C*02:92N			
C*03:02	P	C	C	C	C	C	C	C	C	C	C*03:02	C	C	C
C*03:03	P	C	C	C	C	C	C	C	C	C	C*03:03	C	C	C
C*03:04	P	C	C	C	C	C	C	C	C	C	C*03:04	C	C	C
C*03:05	P	C	C	C	I	I	WD	C	C	C	C*03:05	C		
C*03:06	P	C	C	C	I	I	WD	C	C	C	C*03:06	C		
C*03:07	P	C	I	WD		I		C	WD	I	C*03:07	WD		WD
C*03:08	P	C	I			WD	WD	C	C	I	C*03:08	WD		
C*03:09	P	I	I	WD		I				I	C*03:09	WD		
C*03:10	P	I	I	WD		I				I	C*03:10	WD		
C*03:11	P	WD	WD			WD				WD	C*03:11			
C*03:13	P	WD	WD			WD					C*03:13			
C*03:14	P	I	I			I		I		I	C*03:14	WD		
C*03:16	P	I	I			I	WD	I		I	C*03:16	WD		WD
C*03:17	P	C	WD		C	WD				I	C*03:17	WD		WD
C*03:18	P	WD	WD			WD				WD	C*03:18			
C*03:19	P	C	I			I	C	I		I	C*03:19	WD		
C*03:21	P	I	WD		I						C*03:21			WD

Supplementary Table 3c. Comparison of HLA-C P group two-field assignments among CIWD catalogs [a]

		3.0.0 CIWD									2.0.0 CWD		EFI CWD	China CWD
		Category by Population [b]												
HLA-C IPD-HLA/IMGT 3.31.0	Nomenclature Designation [a]	Highest Frequency	Total	AFA	API	EURO	MENA	HIS	NAM	UNK	HLA-C IPD-IMGT/HLA 3.9.0	Category	Category	Category
C*03:26	P	WD	WD			WD					C*03:26			
C*03:28	P	WD	WD			WD					C*03:28			WD
C*03:30	P	WD	WD			WD					C*03:30			
C*03:32	P	WD	WD		WD	WD					C*03:32			WD
C*03:35	P	WD	WD			WD					C*03:35	WD		WD
C*03:36	P	I	WD		I					I	C*03:36	WD		WD
C*03:37	P	WD	WD		WD						C*03:37			
C*03:38	P	C	WD	C		WD		I	C	I	C*03:38			WD
C*03:39	P	o	o								C*03:39			WD
C*03:40	P	I	WD			WD		I		I	C*03:40	WD		WD
C*03:41	P	WD	WD		WD	WD					C*03:41			WD
C*03:42	P	WD	WD			WD					C*03:42			
C*03:43	P	WD	WD		WD	WD					C*03:43			WD
C*03:44	P	I	I			I				WD	C*03:44			
C*03:45	P	I	WD			WD		I		I	C*03:45			
C*03:46	P	WD	WD			WD					C*03:46			WD
C*03:47	P	I	WD			WD		I		I	C*03:47			
C*03:48	P	o	o								C*03:48			WD
C*03:49	P	WD	WD		WD						C*03:49			
C*03:51	P	WD	WD			WD					C*03:51		WD	
C*03:53	P	WD	WD			WD					C*03:53		WD	
C*03:54	P	WD	WD			WD					C*03:54			
C*03:56	P	WD	WD		WD						C*03:56			WD
C*03:59	P	WD	WD			WD					C*03:59			
C*03:60	P	WD	WD								C*03:60			
C*03:64	P	WD	WD			WD					C*03:64			WD
C*03:69	P	WD	WD			WD					C*03:69			WD
C*03:70	P	WD	WD			WD					C*03:70			

Supplementary Table 3c. Comparison of HLA-C P group two-field assignments among CIWD catalogs [a]

		3.0.0 CIWD									2.0.0 CWD		EFI CWD	China CWD
		Category by Population [b]												
HLA-C IPD-HLA/IMGT 3.31.0	Nomenclature Designation [a]	Highest Frequency	Total	AFA	API	EURO	MENA	HIS	NAM	UNK	HLA-C IPD-IMGT/HLA 3.9.0	Category	Category	Category
C*03:75	P	WD	WD			WD					C*03:75			
C*03:76	P	o	o								C*03:76			WD
C*03:78	P	I	WD					I			C*03:78			
C*03:81	P	WD	WD								C*03:81			
C*03:85	P	I	WD		I						C*03:85			WD
C*03:86	P	WD	WD								C*03:86			
C*03:87	P	I	WD			WD		I			C*03:87			
C*03:88	P	C	I		C		WD				C*03:88	WD		
C*03:91	P	WD	WD			WD					C*03:91			
C*03:94	P	I	WD		I						C*03:94			
C*03:103	P	WD	WD								C*03:103			
C*03:107	P	o	o								C*03:107			WD
C*03:109	P	WD	WD			WD					C*03:109			
C*03:113	P	WD	WD			WD					C*03:113			
C*03:115	P	WD	WD			WD					C*03:115			
C*03:116	P	WD	WD								C*03:116			
C*03:117	P	WD	WD			WD					C*03:117			
C*03:120	P	WD	WD			WD					C*03:120			
C*03:122	P	WD	WD			WD					C*03:122			
C*03:126	P	WD	WD			WD					C*03:126			
C*03:127	P	WD	WD			WD					C*03:127			
C*03:136	P	WD	WD			WD					C*03:136		WD	
C*03:139	P	WD	WD								C*03:139			
C*03:140	P	WD	WD		WD					WD	C*03:140			
C*03:143	P	I	WD			WD		I		I	C*03:143			
C*03:147	P	WD	WD			WD					C*03:147			
C*03:148	P	WD	WD			WD					C*03:148		WD	
C*03:151	P	WD	WD			WD					C*03:151		WD	

Supplementary Table 3c. Comparison of HLA-C P group two-field assignments among CIWD catalogs [a]

		3.0.0 CIWD									2.0.0 CWD		EFI CWD	China CWD
		Category by Population [b]												
HLA-C IPD-HLA/IMGT 3.31.0	Nomenclature Designation [a]	Highest Frequency	Total	AFA	API	EURO	MENA	HIS	NAM	UNK	HLA-C IPD-IMGT/HLA 3.9.0	Category	Category	Category
C*03:153	P	I	WD		I						C*03:153			
C*03:154	P	WD	WD			WD					C*03:154		WD	
C*03:159	P	WD	WD								C*03:159			
C*03:198	P	o	o								C*03:198			WD
C*03:204	P	o	o								C*03:204			WD
C*03:210	P	o	o								C*03:210			WD
C*03:211	P	o	o								C*03:211			WD
C*03:251	P	WD	WD								C*03:251			
C*03:258	P	WD	WD		WD						C*03:258			
C*04:01	P	C	C	C	C	C	C	C	C	C	C*04:01	C	C	C
C*04:03	P	C	C	C	C	C	C	C	C	C	C*04:03	C	C	C
C*04:04	P	C	C	C	WD	I	C	C	C	C	C*04:04	C		
C*04:05	P	WD	WD			WD				WD	C*04:05	WD		
C*04:06	P	C	I		C	WD				C	C*04:06	WD		WD
C*04:07	P	C	C	C	I	I	C	C	WD	C	C*04:07	C	WD	
C*04:08	P	C	I	C		WD		I		I	C*04:08	WD		WD
C*04:09N	N	C	I	WD		I		C	C	I	C*04:09N	C	WD	
C*04:10	P	C	I		C	WD	WD	I	WD	I	C*04:10	WD		
C*04:11	P	WD	WD			WD				WD	C*04:11			
C*04:12	P	WD	WD			WD					C*04:12			
C*04:13	P	C	I	C		WD		I	WD	I	C*04:13	WD		
C*04:14	P	WD	WD	WD		WD	WD				C*04:14			
C*04:15	P	WD	WD			WD				WD	C*04:15			
C*04:16	P	WD	WD			WD					C*04:16			
C*04:17	P	I	WD					I			C*04:17			
C*04:19	P	C	WD					C	C	WD	C*04:19	WD		
C*04:23	P	WD	WD								C*04:23			
C*04:24	P	WD	WD			WD					C*04:24			

Supplementary Table 3c. Comparison of HLA-C P group two-field assignments among CIWD catalogs [a]

		3.0.0 CIWD									2.0.0 CWD		EFI CWD	China CWD
		Category by Population [b]												
HLA-C IPD-HLA/IMGT 3.31.0	Nomenclature Designation [a]	Highest Frequency	Total	AFA	API	EURO	MENA	HIS	NAM	UNK	HLA-C IPD-IMGT/HLA 3.9.0	Category	Category	Category
C*04:26	P	I	WD		I						C*04:26			
C*04:27	P	C	WD	C						WD	C*04:27	WD		
C*04:29	P	WD	WD		WD	WD	WD			WD	C*04:29			
C*04:32	P	WD	WD			WD					C*04:32			
C*04:33	P	WD	WD	WD	WD	WD	WD	WD		WD	C*04:33			
C*04:34	P	WD	WD	WD							C*04:34			
C*04:36	P	WD	WD	WD							C*04:36			
C*04:37	P	WD	WD			WD					C*04:37			
C*04:39	P	WD	WD				WD				C*04:39			
C*04:40	P	WD	WD	WD							C*04:40			
C*04:45	P	WD	WD			WD					C*04:45			
C*04:46	P	I	WD					I			C*04:46			
C*04:47	P	WD	WD			WD					C*04:47			
C*04:52	P	WD	WD			WD					C*04:52			
C*04:54	P	WD	WD			WD					C*04:54			
C*04:55	P	WD	WD			WD					C*04:55			
C*04:56	P	WD	WD			WD					C*04:56			
C*04:59Q	Q	WD	WD			WD					C*04:59Q		WD	
C*04:62	P	o	o								C*04:62		WD	
C*04:63	P	WD	WD			WD					C*04:63			
C*04:65	P	WD	WD			WD					C*04:65			
C*04:69	P	o	o								C*04:69			WD
C*04:70	P	WD	WD								C*04:70			WD
C*04:71	P	WD	WD			WD					C*04:71			
C*04:75	P	WD	WD			WD					C*04:75		WD	
C*04:77	P	WD	WD			WD					C*04:77			
C*04:81	P	o	o								C*04:81			WD
C*04:86	P	WD	WD			WD					C*04:86			

Supplementary Table 3c. Comparison of HLA-C P group two-field assignments among CIWD catalogs [a]

		3.0.0 CIWD									2.0.0 CWD		EFI CWD	China CWD
		Category by Population [b]												
HLA-C IPD-HLA/IMGT 3.31.0	Nomenclature Designation [a]	Highest Frequency	Total	AFA	API	EURO	MENA	HIS	NAM	UNK	HLA-C IPD-IMGT/HLA 3.9.0	Category	Category	Category
C*04:87	P	WD	WD								C*04:87			
C*04:93N	N	WD	WD			WD					C*04:93N		WD	
C*04:95N	N	WD	WD			WD					C*04:95N			
C*04:96	P	WD	WD			WD					C*04:96		WD	
C*04:104	P	WD	WD								C*04:104		WD	
C*04:108	P	WD	WD								C*04:108			
C*04:113	P	WD	WD								C*04:113			
C*04:114	P	WD	WD			WD					C*04:114			
C*04:118	P	WD	WD			WD					C*04:118		WD	
C*04:121	P	WD	WD								C*04:121			
C*04:125	P	WD	WD			WD					C*04:125			
C*04:130	P	o	o								C*04:130			WD
C*04:131	P	WD	WD					WD			C*04:131			
C*04:132	P	WD	WD			WD	WD				C*04:132			
C*04:139	P	WD	WD			WD					C*04:139			
C*04:141	P	WD	WD				WD				C*04:141			
C*04:150	P	WD	WD			WD					C*04:150			
C*04:153	P	WD	WD			WD					C*04:153			
C*04:155	P	WD	WD								C*04:155			
C*04:163	P	WD	WD		WD						C*04:163			
C*04:180	P	WD	WD								C*04:180			
C*04:204	P	WD	WD		WD						C*04:204			
C*04:207	P	WD	WD		WD						C*04:207			
C*04:209	P	WD	WD								C*04:209			
C*04:218	P	WD	WD								C*04:218			
C*05:01	P	C	C	C	C	C	C	C	C	C	C*05:01	C	C	C
C*05:04	P	I	WD			WD		I		WD	C*05:04	WD	WD	
C*05:05	P	I	I			I		WD		I	C*05:05	WD	WD	

Supplementary Table 3c. Comparison of HLA-C P group two-field assignments among CIWD catalogs [a]

		3.0.0 CIWD									2.0.0 CWD		EFI CWD	China CWD
		Category by Population [b]												
HLA-C IPD-HLA/IMGT 3.31.0	Nomenclature Designation [a]	Highest Frequency	Total	AFA	API	EURO	MENA	HIS	NAM	UNK	HLA-C IPD-IMGT/HLA 3.9.0	Category	Category	Category
C*05:06	P	WD	WD			WD					C*05:06		WD	
C*05:07N	N	I	I			I		I		I	C*05:07N	WD	WD	
C*05:08	P	I	I			I		I		I	C*05:08	WD	WD	
C*05:09	P	C	I	WD		I		C	C	C	C*05:09	WD	WD	
C*05:10	P	WD	WD			WD				WD	C*05:10	WD		
C*05:11	P	WD	WD	WD		WD				WD	C*05:11		WD	
C*05:13	P	WD	WD			WD					C*05:13		WD	
C*05:14	P	I	WD			WD				I	C*05:14	WD	WD	
C*05:15	P	WD	WD			WD					C*05:15		WD	
C*05:16	P	WD	WD			WD					C*05:16		WD	
C*05:18	P	WD	WD			WD	WD				C*05:18		WD	
C*05:19	P	WD	WD			WD					C*05:19		WD	
C*05:22	P	WD	WD			WD					C*05:22		WD	
C*05:24	P	WD	WD			WD					C*05:24		WD	
C*05:26	P	WD	WD			WD	WD				C*05:26			
C*05:27	P	WD	WD			WD					C*05:27			
C*05:28	P	WD	WD			WD					C*05:28		WD	
C*05:29	P	WD	WD			WD				WD	C*05:29		WD	
C*05:32	P	WD	WD			WD					C*05:32		WD	
C*05:33	P	WD	WD			WD					C*05:33			
C*05:34	P	WD	WD			WD					C*05:34		WD	
C*05:35	P	WD	WD			WD					C*05:35			
C*05:36	P	WD	WD			WD				WD	C*05:36			
C*05:39	P	WD	WD			WD					C*05:39		WD	
C*05:43	P	WD	WD			WD					C*05:43			
C*05:44	P	WD	WD			WD					C*05:44		WD	
C*05:46	P	WD	WD			WD					C*05:46			
C*05:47	P	WD	WD			WD					C*05:47		WD	

Supplementary Table 3c. Comparison of HLA-C P group two-field assignments among CIWD catalogs [a]

		3.0.0 CIWD									2.0.0 CWD		EFI CWD	China CWD
		Category by Population [b]												
HLA-C IPD-HLA/IMGT 3.31.0	Nomenclature Designation [a]	Highest Frequency	Total	AFA	API	EURO	MENA	HIS	NAM	UNK	HLA-C IPD-IMGT/HLA 3.9.0	Category	Category	Category
C*05:51Q	Q	WD	WD			WD					C*05:51Q			
C*05:55	P	WD	WD			WD					C*05:55			
C*05:65	P	WD	WD			WD					C*05:65			
C*05:67	P	WD	WD			WD					C*05:67			
C*05:77	P	WD	WD			WD					C*05:77			
C*05:80	P	WD	WD			WD					C*05:80			
C*05:97	P	WD	WD			WD					C*05:97			
C*05:98	P	WD	WD			WD					C*05:98			
C*05:99N	N	WD	WD			WD					C*05:99N			
C*05:104	P	WD	WD			WD					C*05:104			
C*05:111	P	WD	WD			WD					C*05:111			
C*06:02	P	C	C	C	C	C	C	C	C	C	C*06:02	C	C	C
C*06:03	P	WD	WD			WD					C*06:03			
C*06:04	P	I	I			I				WD	C*06:04	WD		WD
C*06:06	P	C	I	WD	WD	I	C	I		I	C*06:06	WD		WD
C*06:07	P	I	I			I		WD		I	C*06:07	WD		
C*06:08	P	C	WD	C		WD		I		I	C*06:08	WD		
C*06:09	P	C	WD	C		WD		I		WD	C*06:09	WD		
C*06:10	P	WD	WD			WD					C*06:10			
C*06:11	P	WD	WD			WD	WD				C*06:11			
C*06:12	P	WD	WD								C*06:12			
C*06:13	P	WD	WD			WD					C*06:13			
C*06:14	P	WD	WD			WD					C*06:14			
C*06:16N	N	WD	WD			WD					C*06:16N			
C*06:18	P	I	WD			I					C*06:18			
C*06:23	P	WD	WD			WD					C*06:23			
C*06:26	P	WD	WD			WD					C*06:26			
C*06:27	P	C	I		C	WD	WD			WD	C*06:27	WD		

Supplementary Table 3c. Comparison of HLA-C P group two-field assignments among CIWD catalogs [a]

		3.0.0 CIWD									2.0.0 CWD		EFI CWD	China CWD
		Category by Population [b]												
HLA-C IPD-HLA/IMGT 3.31.0	Nomenclature Designation [a]	Highest Frequency	Total	AFA	API	EURO	MENA	HIS	NAM	UNK	HLA-C IPD-IMGT/HLA 3.9.0	Category	Category	Category
C*06:28	P	WD	WD			WD					C*06:28			
C*06:30	P	WD	WD			WD					C*06:30			
C*06:32	P	WD	WD			WD					C*06:32			
C*06:34	P	WD	WD			WD	WD				C*06:34			
C*06:35	P	I	WD		I						C*06:35			
C*06:37	P	WD	WD								C*06:37			
C*06:38	P	WD	WD			WD					C*06:38			
C*06:39	P	WD	WD								C*06:39			
C*06:47	P	WD	WD			WD					C*06:47			
C*06:50	P	WD	WD			WD					C*06:50			
C*06:53	P	WD	WD				WD				C*06:53			
C*06:54	P	WD	WD			WD					C*06:54			
C*06:58	P	WD	WD			WD					C*06:58			
C*06:59	P	WD	WD			WD				WD	C*06:59			
C*06:65	P	WD	WD			WD					C*06:65		WD	
C*06:66	P	WD	WD			WD					C*06:66			
C*06:70	P	WD	WD			WD					C*06:70			
C*06:71	P	WD	WD			WD					C*06:71			
C*06:79N	N	I	WD		I						C*06:79N			
C*06:86	P	WD	WD			WD					C*06:86			
C*06:87	P	WD	WD			WD					C*06:87			
C*06:91	P	WD	WD			WD					C*06:91		WD	
C*06:96	P	WD	WD		WD						C*06:96			
C*06:103	P	o	o								C*06:103			WD
C*06:104	P	o	o								C*06:104			WD
C*06:106	P	o	o								C*06:106			WD
C*06:115	P	WD	WD		WD						C*06:115			
C*06:127	P	I	WD		I						C*06:127			

Supplementary Table 3c. Comparison of HLA-C P group two-field assignments among CIWD catalogs [a]

		3.0.0 CIWD									2.0.0 CWD		EFI CWD	China CWD
		Category by Population [b]												
HLA-C IPD-HLA/IMGT 3.31.0	Nomenclature Designation [a]	Highest Frequency	Total	AFA	API	EURO	MENA	HIS	NAM	UNK	HLA-C IPD-IMGT/HLA 3.9.0	Category	Category	Category
C*06:138	P	WD	WD								C*06:138			
C*07:01	P	C	C	C	C	C	C	C	C	C	C*07:01	C	C	C
C*07:02	P	C	C	C	C	C	C	C	C	C	C*07:02	C	C	C
C*07:04	P	C	C	C	C	C	C	C	C	C	C*07:04	C	C	C
C*07:05	P	C	I	C		WD	WD	I	WD	I	C*07:05	WD	WD	
C*07:07	P	I	I			I	WD	I		I	C*07:07	WD	C	
C*07:08	P	o	o								C*07:08		WD	
C*07:09	P	WD	WD								C*07:09			
C*07:10	P	I	I			I				I	C*07:10	WD	WD	
C*07:12	P	I	I			I				I	C*07:12	WD	WD	
C*07:13	P	I	WD		I	WD	WD			WD	C*07:13	WD	WD	
C*07:15	P	I	WD			I					C*07:15		WD	
C*07:16	P	I	WD			WD				I	C*07:16		WD	WD
C*07:17	P	C	I	WD		I		C	C	I	C*07:17	WD	WD	
C*07:19	P	C	I	C		I	C	I		I	C*07:19	WD	WD	WD
C*07:21	P	I	WD			WD				I	C*07:21	WD	WD	
C*07:22	P	I	I			I				I	C*07:22	WD	WD	
C*07:24	P	WD	WD			WD				WD	C*07:24	WD	WD	
C*07:25	P	I	I			I				I	C*07:25	WD	WD	
C*07:26	P	C	C	WD	C	WD	WD	WD	C	I	C*07:26	WD	WD	WD
C*07:27	P	C	I		C	I	C	I		I	C*07:27	WD	WD	WD
C*07:28	P	WD	WD	WD		WD					C*07:28			
C*07:29	P	C	I		C	WD				I	C*07:29	WD	WD	
C*07:31	P	WD	WD			WD					C*07:31		WD	
C*07:32N	N	WD	WD			WD				WD	C*07:32N	WD	WD	
C*07:33N	N	WD	WD			WD					C*07:33N			
C*07:35	P	C	WD	C				I		I	C*07:35	WD		
C*07:36	P	I	I			I	WD				C*07:36		WD	

Supplementary Table 3c. Comparison of HLA-C P group two-field assignments among CIWD catalogs [a]

		3.0.0 CIWD									2.0.0 CWD		EFI CWD	China CWD
		Category by Population [b]												
HLA-C IPD-HLA/IMGT 3.31.0	Nomenclature Designation [a]	Highest Frequency	Total	AFA	API	EURO	MENA	HIS	NAM	UNK	HLA-C IPD-IMGT/HLA 3.9.0	Category	Category	Category
C*07:37	P	WD	WD			WD					C*07:37		WD	
C*07:38	P	WD	WD			WD					C*07:38			
C*07:39	P	WD	WD			WD					C*07:39			
C*07:40	P	I	I			I				WD	C*07:40	WD	WD	
C*07:43	P	I	WD		I						C*07:43	WD		WD
C*07:44	P	WD	WD			WD					C*07:44			
C*07:46	P	I	I			I				WD	C*07:46	WD	WD	
C*07:48	P	I	WD		I						C*07:48			
C*07:49	P	WD	WD			WD					C*07:49		WD	
C*07:51	P	WD	WD		WD	WD				WD	C*07:51		WD	WD
C*07:54	P	WD	WD			WD					C*07:54		WD	
C*07:55N	N	WD	WD			WD					C*07:55N		WD	
C*07:56	P	I	WD		I						C*07:56	WD		WD
C*07:57	P	WD	WD			WD					C*07:57			
C*07:60	P	I	I			I					C*07:60	WD	WD	
C*07:61N	N	WD	WD	WD							C*07:61N	WD		
C*07:63	P	I	WD		I						C*07:63			WD
C*07:67	P	o	o								C*07:67	WD		WD
C*07:68	P	I	I			I				WD	C*07:68		C	
C*07:70	P	WD	WD			WD					C*07:70			
C*07:72	P	WD	WD			WD				WD	C*07:72	WD		
C*07:73	P	WD	WD			WD					C*07:73			
C*07:75	P	WD	WD			WD					C*07:75		WD	
C*07:76	P	WD	WD			WD					C*07:76		WD	
C*07:79	P	WD	WD			WD					C*07:79		WD	
C*07:80	P	WD	WD			WD	WD	WD		WD	C*07:80			
C*07:83	P	WD	WD			WD					C*07:83		WD	
C*07:85	P	WD	WD			WD					C*07:85		WD	

Supplementary Table 3c. Comparison of HLA-C P group two-field assignments among CIWD catalogs [a]

		3.0.0 CIWD									2.0.0 CWD		EFI CWD	China CWD
		Category by Population [b]												
HLA-C IPD-HLA/IMGT 3.31.0	Nomenclature Designation [a]	Highest Frequency	Total	AFA	API	EURO	MENA	HIS	NAM	UNK	HLA-C IPD-IMGT/HLA 3.9.0	Category	Category	Category
C*07:88	P	WD	WD			WD					C*07:88		WD	
C*07:89	P	WD	WD								C*07:89		WD	
C*07:90	P	WD	WD			WD					C*07:90		WD	
C*07:91	P	WD	WD			WD					C*07:91		WD	
C*07:92	P	WD	WD			WD					C*07:92		WD	
C*07:93	P	WD	WD			WD	WD			WD	C*07:93		WD	
C*07:95	P	WD	WD			WD					C*07:95		WD	
C*07:96	P	WD	WD			WD					C*07:96		WD	
C*07:97	P	WD	WD			WD					C*07:97		WD	
C*07:100	P	WD	WD			WD					C*07:100		WD	
C*07:101	P	WD	WD			WD					C*07:101		WD	
C*07:102	P	WD	WD			WD					C*07:102			
C*07:104N	N	WD	WD			WD					C*07:104N	WD		
C*07:105	P	WD	WD			WD					C*07:105		WD	
C*07:106	P	WD	WD			WD					C*07:106		WD	
C*07:108	P	WD	WD			WD					C*07:108		WD	
C*07:112	P	WD	WD	WD							C*07:112			
C*07:114	P	WD	WD			WD					C*07:114		WD	
C*07:116	P	WD	WD			WD					C*07:116		WD	
C*07:117	P	WD	WD			WD					C*07:117		WD	
C*07:118	P	WD	WD			WD					C*07:118		WD	
C*07:119	P	WD	WD			WD				WD	C*07:119			
C*07:120	P	WD	WD			WD					C*07:120		WD	
C*07:121Q	Q	WD	WD			WD					C*07:121Q		WD	
C*07:122	P	WD	WD			WD					C*07:122			
C*07:123	P	WD	WD								C*07:123			
C*07:124	P	WD	WD			WD				WD	C*07:124		WD	
C*07:126	P	WD	WD			WD					C*07:126			

Supplementary Table 3c. Comparison of HLA-C P group two-field assignments among CIWD catalogs [a]

		3.0.0 CIWD									2.0.0 CWD		EFI CWD	China CWD
		Category by Population [b]												
HLA-C IPD-HLA/IMGT 3.31.0	Nomenclature Designation [a]	Highest Frequency	Total	AFA	API	EURO	MENA	HIS	NAM	UNK	HLA-C IPD-IMGT/HLA 3.9.0	Category	Category	Category
C*07:127	P	WD	WD			WD					C*07:127		WD	
C*07:128	P	WD	WD								C*07:128			
C*07:130	P	WD	WD		WD	WD					C*07:130		WD	
C*07:133	P	o	o								C*07:133		WD	
C*07:137	P	WD	WD			WD					C*07:137		WD	
C*07:138	P	WD	WD			WD					C*07:138		WD	
C*07:141	P	WD	WD								C*07:141			
C*07:143	P	WD	WD			WD					C*07:143		WD	
C*07:147	P	WD	WD			WD					C*07:147			
C*07:148	P	WD	WD			WD					C*07:148		WD	
C*07:149	P	WD	WD			WD					C*07:149			
C*07:151	P	WD	WD			WD					C*07:151			
C*07:154	P	WD	WD		WD						C*07:154			WD
C*07:155	P	WD	WD			WD					C*07:155		WD	
C*07:165	P	WD	WD			WD					C*07:165		WD	
C*07:169	P	o	o								C*07:169			WD
C*07:172	P	WD	WD			WD					C*07:172		WD	WD
C*07:173	P	WD	WD	WD		WD					C*07:173			
C*07:179	P	WD	WD			WD					C*07:179		WD	
C*07:180	P	WD	WD			WD					C*07:180		WD	
C*07:181	P	WD	WD								C*07:181			
C*07:184	P	WD	WD	WD							C*07:184			
C*07:192	P	WD	WD			WD					C*07:192		WD	
C*07:196	P	WD	WD								C*07:196			
C*07:198N	N	WD	WD								C*07:198N			
C*07:199	P	WD	WD		WD	WD					C*07:199			
C*07:205	P	WD	WD			WD					C*07:205		WD	
C*07:206	P	WD	WD			WD					C*07:206			

Supplementary Table 3c. Comparison of HLA-C P group two-field assignments among CIWD catalogs [a]

		3.0.0 CIWD									2.0.0 CWD		EFI CWD	China CWD
		Category by Population [b]												
HLA-C IPD-HLA/IMGT 3.31.0	Nomenclature Designation [a]	Highest Frequency	Total	AFA	API	EURO	MENA	HIS	NAM	UNK	HLA-C IPD-IMGT/HLA 3.9.0	Category	Category	Category
C*07:207	P	o	o								C*07:207		WD	
C*07:209	P	WD	WD			WD					C*07:209			
C*07:212	P	WD	WD			WD					C*07:212		WD	
C*07:214	P	WD	WD			WD					C*07:214			
C*07:218	P	WD	WD			WD					C*07:218		WD	
C*07:222	P	WD	WD			WD					C*07:222			
C*07:223	P	WD	WD								C*07:223			
C*07:224	P	WD	WD								C*07:224			
C*07:227N	N	WD	WD								C*07:227N			
C*07:232	P	I	WD		I						C*07:232			
C*07:234	P	WD	WD			WD					C*07:234			
C*07:241	P	WD	WD			WD					C*07:241		WD	
C*07:242	P	WD	WD			WD					C*07:242		WD	
C*07:243	P	WD	WD								C*07:243			
C*07:247	P	WD	WD								C*07:247			
C*07:250	P	WD	WD			WD					C*07:250			
C*07:252	P	WD	WD			WD					C*07:252			
C*07:256	P	WD	WD			WD					C*07:256			
C*07:257	P	WD	WD			WD					C*07:257			
C*07:261	P	WD	WD			WD					C*07:261			
C*07:262	P	WD	WD			WD					C*07:262			
C*07:279	P	WD	WD			WD					C*07:279			
C*07:285	P	WD	WD			WD					C*07:285			
C*07:287	P	WD	WD			WD					C*07:287			
C*07:293	P	WD	WD								C*07:293			
C*07:298	P	WD	WD			WD					C*07:298			
C*07:301	P	WD	WD			WD					C*07:301			
C*07:303	P	WD	WD			WD					C*07:303			

Supplementary Table 3c. Comparison of HLA-C P group two-field assignments among CIWD catalogs [a]

		3.0.0 CIWD									2.0.0 CWD		EFI CWD	China CWD
		Category by Population [b]												
HLA-C IPD-HLA/IMGT 3.31.0	Nomenclature Designation [a]	Highest Frequency	Total	AFA	API	EURO	MENA	HIS	NAM	UNK	HLA-C IPD-IMGT/HLA 3.9.0	Category	Category	Category
C*07:312	P	WD	WD								C*07:312			
C*07:314	P	WD	WD								C*07:314			
C*07:326	P	WD	WD					WD			C*07:326			
C*07:335	P	WD	WD			WD					C*07:335			
C*07:339	P	I	WD		I						C*07:339			
C*07:340	P	I	WD		I						C*07:340			
C*07:360	P	WD	WD			WD					C*07:360			
C*07:368	P	WD	WD								C*07:368			
C*07:369	P	WD	WD								C*07:369			
C*07:391	P	WD	WD			WD					C*07:391			
C*07:409	P	WD	WD								C*07:409			
C*07:450	P	WD	WD			WD					C*07:450			
C*07:452N	N	WD	WD			WD					C*07:452N			
C*08:01	P	C	C	C	C	C	C	C	C	C	C*08:01	C	C	C
C*08:02	P	C	C	C	C	C	C	C	C	C	C*08:02	C	C	C
C*08:03	P	C	C	C	C	C	C	C	C	C	C*08:03	C		C
C*08:04	P	C	C	C	I	I	C	C	C	C	C*08:04	C	WD	
C*08:05	P	I	WD	WD		WD				I	C*08:05	WD		
C*08:06	P	C	WD		I	WD			C	I	C*08:06	WD		WD
C*08:07	P	WD	WD								C*08:07			
C*08:08	P	I	WD			WD		I		I	C*08:08			
C*08:10	P	C	I			WD		C	C	I	C*08:10	WD		WD
C*08:11	P	I	WD			WD		I	WD	WD	C*08:11			
C*08:12	P	WD	WD			WD				WD	C*08:12	WD	WD	
C*08:13	P	I	WD	WD				I			C*08:13	WD		
C*08:15	P	WD	WD			WD				WD	C*08:15		WD	
C*08:21	P	o	o								C*08:21			WD
C*08:23	P	WD	WD			WD					C*08:23			

Supplementary Table 3c. Comparison of HLA-C P group two-field assignments among CIWD catalogs [a]

		3.0.0 CIWD									2.0.0 CWD		EFI CWD	China CWD
		Category by Population [b]												
HLA-C IPD-HLA/IMGT 3.31.0	Nomenclature Designation [a]	Highest Frequency	Total	AFA	API	EURO	MENA	HIS	NAM	UNK	HLA-C IPD-IMGT/HLA 3.9.0	Category	Category	Category
C*08:25	P	WD	WD								C*08:25			
C*08:27	P	o	o								C*08:27			WD
C*08:28	P	WD	WD			WD					C*08:28		WD	
C*08:30	P	WD	WD			WD				WD	C*08:30			
C*08:32	P	WD	WD	WD							C*08:32			
C*08:33	P	WD	WD			WD					C*08:33		WD	
C*08:35	P	I	WD					I		WD	C*08:35			
C*08:41	P	o	o								C*08:41			WD
C*08:43	P	WD	WD	WD							C*08:43			
C*08:44	P	o	o								C*08:44			WD
C*08:50	P	WD	WD		WD						C*08:50			
C*08:51	P	WD	WD			WD					C*08:51			
C*08:53	P	WD	WD			WD					C*08:53			
C*08:54	P	WD	WD								C*08:54			
C*08:76	P	WD	WD								C*08:76			
C*08:92	P	WD	WD			WD					C*08:92			
C*08:103	P	WD	WD			WD					C*08:103			
C*08:127N	N	WD	WD		WD						C*08:127N			
C*12:02	P	C	C	C	C	C	C	C	C	C	C*12:02	C	C	C
C*12:03	P	C	C	C	C	C	C	C	C	C	C*12:03	C	C	C
C*12:04	P	C	C	WD	C	I	WD	I	C	C	C*12:04	C		WD
C*12:05	P	C	C	WD	C	I	C	I		I	C*12:05	WD		WD
C*12:06	P	WD	WD			WD					C*12:06			
C*12:07	P	WD	WD	WD							C*12:07			
C*12:08	P	WD	WD								C*12:08			
C*12:09	P	C	I		C	WD	WD			I	C*12:09	WD		WD
C*12:11	P	WD	WD			WD					C*12:11			
C*12:12	P	C	I	WD		WD	WD	C	C	I	C*12:12	WD		

Supplementary Table 3c. Comparison of HLA-C P group two-field assignments among CIWD catalogs [a]

		3.0.0 CIWD									2.0.0 CWD		EFI CWD	China CWD
		Category by Population [b]												
HLA-C IPD-HLA/IMGT 3.31.0	Nomenclature Designation [a]	Highest Frequency	Total	AFA	API	EURO	MENA	HIS	NAM	UNK	HLA-C IPD-IMGT/HLA 3.9.0	Category	Category	Category
C*12:13	P	I	I			I				I	C*12:13	WD		
C*12:14	P	WD	WD			WD					C*12:14			
C*12:16	P	I	I			I				WD	C*12:16	WD		
C*12:18	P	I	WD		I						C*12:18			WD
C*12:19	P	WD	WD	WD							C*12:19			
C*12:20	P	WD	WD								C*12:20			
C*12:21	P	WD	WD			WD					C*12:21			
C*12:24	P	WD	WD			WD				WD	C*12:24			
C*12:27	P	WD	WD				WD				C*12:27			
C*12:28	P	WD	WD			WD					C*12:28			
C*12:30	P	I	I			I					C*12:30			
C*12:33	P	WD	WD			WD					C*12:33			
C*12:34	P	I	I			I				WD	C*12:34			
C*12:38	P	I	WD		I						C*12:38			
C*12:40	P	WD	WD		WD						C*12:40			
C*12:41	P	I	WD		I						C*12:41			
C*12:42Q	Q	WD	WD			WD					C*12:42Q		WD	
C*12:43	P	WD	WD			WD					C*12:43			
C*12:45	P	WD	WD			WD					C*12:45			
C*12:47	P	WD	WD			WD				WD	C*12:47			
C*12:48	P	WD	WD			WD					C*12:48			
C*12:54	P	WD	WD		WD	WD					C*12:54			
C*12:59	P	WD	WD	WD							C*12:59			
C*12:60	P	I	WD		I						C*12:60			
C*12:61	P	WD	WD								C*12:61			
C*12:62	P	WD	WD			WD					C*12:62			
C*12:64	P	WD	WD								C*12:64			
C*12:67	P	WD	WD			WD					C*12:67			

Supplementary Table 3c. Comparison of HLA-C P group two-field assignments among CIWD catalogs [a]

		3.0.0 CIWD									2.0.0 CWD		EFI CWD	China CWD
		Category by Population [b]												
HLA-C IPD-HLA/IMGT 3.31.0	Nomenclature Designation [a]	Highest Frequency	Total	AFA	API	EURO	MENA	HIS	NAM	UNK	HLA-C IPD-IMGT/HLA 3.9.0	Category	Category	Category
C*12:72	P	I	WD		I						C*12:72			
C*12:78	P	WD	WD								C*12:78			
C*12:86	P	WD	WD			WD					C*12:86			
C*12:90	P	WD	WD			WD					C*12:90			
C*12:98	P	WD	WD				WD			WD	C*12:98			
C*12:99	P	WD	WD			WD					C*12:99			
C*12:101	P	I	WD		I						C*12:101			
C*12:136	P	WD	WD		WD						C*12:136			
C*12:139	P	WD	WD				WD				C*12:139			
C*12:149	P	WD	WD								C*12:149			
C*12:162	P	WD	WD		WD						C*12:162			
C*14:02	P	C	C	C	C	C	C	C	C	C	C*14:02	C	C	C
C*14:03	P	C	C	C	C	C	C	C	C	C	C*14:03	C	C	C
C*14:04	P	I	I	WD		I				I	C*14:04	WD	WD	
C*14:05	P	I	I			I				WD	C*14:05	WD	WD	
C*14:06	P	I	WD	WD		WD		I		WD	C*14:06			
C*14:10	P	WD	WD	WD							C*14:10			
C*14:12	P	I	WD		I						C*14:12			WD
C*14:18	P	WD	WD			WD					C*14:18		WD	
C*14:24	P	I	WD		I						C*14:24			WD
C*14:25	P	WD	WD			WD					C*14:25		WD	WD
C*14:29	P	WD	WD		WD						C*14:29			WD
C*14:32	P	WD	WD			WD					C*14:32		WD	
C*14:41	P	WD	WD			WD					C*14:41		WD	
C*14:50	P	WD	WD			WD					C*14:50			
C*14:66	P	I	WD					I			C*14:66			
C*14:67	P	WD	WD								C*14:67			
C*15:02	P	C	C	C	C	C	C	C	C	C	C*15:02	C	C	C

Supplementary Table 3c. Comparison of HLA-C P group two-field assignments among CIWD catalogs [a]

		3.0.0 CIWD									2.0.0 CWD		EFI CWD	China CWD
		Category by Population [b]												
HLA-C IPD-HLA/IMGT 3.31.0	Nomenclature Designation [a]	Highest Frequency	Total	AFA	API	EURO	MENA	HIS	NAM	UNK	HLA-C IPD-IMGT/HLA 3.9.0	Category	Category	Category
C*15:03	P	I	WD			WD		I		I	C*15:03	WD		
C*15:04	P	C	C	WD	C	C	C	C	C	C	C*15:04	C	WD	WD
C*15:05	P	C	C	C	C	C	C	C	C	C	C*15:05	C	C	C
C*15:06	P	C	C	WD	I	C	C	C	C	C	C*15:06	C	C	WD
C*15:07	P	C	C		C	WD	WD			I	C*15:07	WD	WD	WD
C*15:08	P	I	WD		I	WD		I		I	C*15:08	WD	WD	
C*15:09	P	C	C	WD	I	I	C	C	C	C	C*15:09	C	C	WD
C*15:10	P	I	WD		I	WD					C*15:10		WD	
C*15:11	P	I	I	WD	I	I	WD	I		I	C*15:11	WD	C	WD
C*15:12	P	o	o								C*15:12			WD
C*15:15	P	WD	WD			WD					C*15:15		WD	
C*15:16	P	I	WD			WD		I		I	C*15:16			
C*15:17	P	I	WD			WD		I			C*15:17		WD	WD
C*15:21	P	o	o								C*15:21			WD
C*15:22	P	C	WD	C						WD	C*15:22	WD		
C*15:23	P	WD	WD			WD					C*15:23		WD	
C*15:24	P	WD	WD			WD					C*15:24		WD	
C*15:25	P	WD	WD	WD			WD				C*15:25			
C*15:26	P	WD	WD		WD						C*15:26			WD
C*15:27	P	I	WD					I			C*15:27			
C*15:30	P	WD	WD			WD	WD				C*15:30		WD	
C*15:32Q	Q	I	WD		I						C*15:32Q			
C*15:38	P	WD	WD			WD					C*15:38			
C*15:40	P	WD	WD		WD						C*15:40			
C*15:42	P	I	WD		I						C*15:42			
C*15:44	P	WD	WD			WD					C*15:44			
C*15:52	P	WD	WD							WD	C*15:52			
C*15:56	P	o	o								C*15:56		WD	

Supplementary Table 3c. Comparison of HLA-C P group two-field assignments among CIWD catalogs [a]

		3.0.0 CIWD									2.0.0 CWD		EFI CWD	China CWD
		Category by Population [b]												
HLA-C IPD-HLA/IMGT 3.31.0	Nomenclature Designation [a]	Highest Frequency	Total	AFA	API	EURO	MENA	HIS	NAM	UNK	HLA-C IPD-IMGT/HLA 3.9.0	Category	Category	Category
C*15:57	P	WD	WD		WD						C*15:57			
C*15:58	P	I	WD		I						C*15:58			
C*15:68	P	WD	WD		WD						C*15:68			
C*15:72	P	WD	WD								C*15:72			
C*15:85	P	WD	WD								C*15:85			
C*15:102	P	WD	WD							WD	C*15:102			
C*15:122N	N	WD	WD								C*15:122N			
C*15:124	P	WD	WD		WD						C*15:124			
C*16:01	P	C	C	C	C	C	C	C	C	C	C*16:01	C	C	WD
C*16:02	P	C	C	C	C	C	C	C	C	C	C*16:02	C	C	C
C*16:04	P	C	C	C	C	C	C	C	C	C	C*16:04	C	C	WD
C*16:07	P	WD	WD	WD		WD					C*16:07			
C*16:08	P	WD	WD	WD							C*16:08			
C*16:09	P	I	WD					I			C*16:09			
C*16:12	P	I	WD		I						C*16:12			
C*16:13	P	WD	WD			WD					C*16:13		WD	
C*16:14	P	WD	WD			WD					C*16:14		WD	
C*16:15	P	C	WD			WD	C			WD	C*16:15		WD	
C*16:16Q	Q	WD	WD			WD					C*16:16Q		WD	
C*16:18	P	WD	WD			WD					C*16:18		WD	
C*16:19	P	I	WD		I						C*16:19			
C*16:21	P	WD	WD			WD					C*16:21		WD	
C*16:22	P	WD	WD	WD							C*16:22			
C*16:24	P	WD	WD								C*16:24			
C*16:25	P	WD	WD	WD		WD					C*16:25			
C*16:26	P	WD	WD			WD					C*16:26			
C*16:30N	N	I	WD		I						C*16:30N			
C*16:33	P	WD	WD							WD	C*16:33			

Supplementary Table 3c. Comparison of HLA-C P group two-field assignments among CIWD catalogs [a]

		3.0.0 CIWD									2.0.0 CWD		EFI CWD	China CWD
		Category by Population [b]												
HLA-C IPD-HLA/IMGT 3.31.0	Nomenclature Designation [a]	Highest Frequency	Total	AFA	API	EURO	MENA	HIS	NAM	UNK	HLA-C IPD-IMGT/HLA 3.9.0	Category	Category	Category
C*16:37	P	WD	WD							WD	C*16:37			
C*16:38	P	WD	WD			WD				WD	C*16:38			
C*16:46	P	WD	WD	WD			WD				C*16:46			
C*16:50	P	WD	WD	WD						WD	C*16:50			
C*16:52	P	WD	WD			WD					C*16:52			
C*16:74	P	WD	WD			WD					C*16:74			
C*16:88	P	WD	WD		WD						C*16:88			
C*17:01	P	C	C	C	C	C	C	C	C	C	C*17:01	C	C	C
C*17:04	P	I	WD	WD						I	C*17:04			
C*17:07	P	WD	WD			WD					C*17:07		WD	
C*17:23	P	WD	WD								C*17:23			
C*18:01	P	C	C	C	I	C	C	C	C	C	C*18:01	C	WD	WD
C*18:03	P	WD	WD	WD						WD	C*18:03			
C*18:04	P	WD	WD	WD							C*18:04			
C*18:06	P	WD	WD	WD							C*18:06			

C, common; I, intermediate; WD, well-documented

[a] Alleles observed at least five times in any population group in the current dataset are included in this table. The exception are alleles that were observed in previous catalogs but that are not present or are observed less than five times. Note that this two-field designation is the equivalent of a "P" assignment so that alleles within a P group that encode proteins that differ outside of the antigen recognition domain are not listed separately (e.g., C*02:10 is within the C*02:02P two-field assignment and is not listed in this table even though it was categorized as common in 2.0.0 CWD). The table does not list all alleles from IPD-IMGT/HLA version 3.31.0.

[b] Population groups include: AFA (African/African American), API (Asian/Pacific Islander), EURO (European/European descent), MENA (Middle East/North Coast of Africa), HIS (Hispanic), NAM (Native American) and UNK (unknown, multiple ancestries or other). Total is the overall population i.e., all groups combined. Highest frequency is the highest CIWD designation among all the individual groups.

[c] Not-CIWD, o.

Supplementary Table 3d. Comparison of HLA-DRB1 P group two-field assignments among CIWD catalogs [a]

		3.0.0 CIWD									2.0.0 CWD		EFI CWD	China CWD
		Category by Population [b]												
**HLA-DRB1 IPD-HLA/IMGT 3.31.0**	**Nomenclature Designation [a]**	**Highest Frequency**	Total	**AFA**	**API**	**EURO**	**MENA**	**HIS**	**NAM**	**UNK**	**HLA-DRB1 IPD-IMGT/HLA 3.9.0**	**Category**	**Category**	**Category**
DRB1*01:01	P	C	C	C	C	C	C	C	C	C	DRB1*01:01	C	C	C
DRB1*01:02	P	C	C	C	C	C	C	C	C	C	DRB1*01:02	C	C	C
DRB1*01:03	P	C	C	C	C	C	C	C	C	C	DRB1*01:03	C	C	
DRB1*01:04	P	WD	WD								DRB1*01:04	WD	WD	
DRB1*01:06	P	o [c]	o								DRB1*01:06		WD	
DRB1*01:07	P	I	I			I				WD	DRB1*01:07	WD	WD	
DRB1*01:08	P	o	o								DRB1*01:08		WD	
DRB1*01:09	P	o	o								DRB1*01:09		WD	
DRB1*01:10	P	WD	WD			WD					DRB1*01:10		WD	
DRB1*01:11	P	WD	WD			WD					DRB1*01:11	WD	WD	
DRB1*01:14	P	o	o								DRB1*01:14			WD
DRB1*01:16	P	WD	WD			WD					DRB1*01:16			
DRB1*01:18	P	WD	WD			WD					DRB1*01:18		WD	
DRB1*01:20	P	WD	WD			WD					DRB1*01:20		WD	
DRB1*01:22	P	WD	WD			WD					DRB1*01:22		WD	
DRB1*01:24	P	WD	WD			WD					DRB1*01:24		WD	
DRB1*01:26	P	WD	WD	WD							DRB1*01:26			
DRB1*01:27	P	WD	WD			WD					DRB1*01:27		WD	
DRB1*01:29	P	WD	WD			WD					DRB1*01:29		WD	
DRB1*03:01	P	C	C	C	C	C	C	C	C	C	DRB1*03:01	C	C	C
DRB1*03:02	P	C	C	C	C	C	C	C	C	C	DRB1*03:02	C	C	
DRB1*03:03	P	WD	WD	WD						WD	DRB1*03:03	WD		
DRB1*03:04	P	C	C		WD	C		I		I	DRB1*03:04	WD	WD	
DRB1*03:05	P	I	I	WD	I	I				I	DRB1*03:05	WD		WD
DRB1*03:06	P	I	I	WD	I	I				WD	DRB1*03:06	WD		WD
DRB1*03:07	P	I	WD			WD	WD	I		WD	DRB1*03:07	WD		
DRB1*03:08	P	WD	WD			WD					DRB1*03:08			WD
DRB1*03:11	P	WD	WD			WD					DRB1*03:11			WD

Supplementary Table 3d. Comparison of HLA-DRB1 P group two-field assignments among CIWD catalogs [a]

		3.0.0 CIWD									2.0.0 CWD		EFI CWD	China CWD
		Category by Population [b]												
**HLA-DRB1 IPD-HLA/IMGT 3.31.0**	**Nomenclature Designation [a]**	**Highest Frequency**	Total	**AFA**	**API**	**EURO**	**MENA**	**HIS**	**NAM**	**UNK**	**HLA-DRB1 IPD-IMGT/HLA 3.9.0**	**Category**	**Category**	**Category**
DRB1*03:13	P	I	WD			I				WD	DRB1*03:13			
DRB1*03:15	P	C	I	WD	WD	I		C	C	I	DRB1*03:15	WD		
DRB1*03:16	P	I	WD			WD		I			DRB1*03:16	WD		
DRB1*03:17	P	WD	WD			WD					DRB1*03:17			
DRB1*03:19	P	WD	WD			WD					DRB1*03:19			
DRB1*03:22	P	WD	WD			WD					DRB1*03:22	WD		
DRB1*03:23	P	I	I			I				WD	DRB1*03:23	WD		
DRB1*03:24	P	WD	WD			WD					DRB1*03:24			
DRB1*03:25	P	WD	WD			WD					DRB1*03:25			
DRB1*03:27	P	WD	WD								DRB1*03:27			WD
DRB1*03:29	P	WD	WD	WD							DRB1*03:29			
DRB1*03:30	P	WD	WD			WD					DRB1*03:30			
DRB1*03:36	P	WD	WD			WD					DRB1*03:36			
DRB1*03:37	P	I	WD		WD	WD				I	DRB1*03:37			
DRB1*03:39	P	WD	WD			WD					DRB1*03:39			
DRB1*03:42	P	WD	WD			WD					DRB1*03:42			
DRB1*03:43	P	WD	WD			WD					DRB1*03:43			
DRB1*03:44	P	WD	WD			WD					DRB1*03:44			
DRB1*03:47	P	WD	WD			WD					DRB1*03:47			
DRB1*03:58	P	WD	WD			WD					DRB1*03:58			
DRB1*03:69	P	WD	WD			WD					DRB1*03:69			
DRB1*03:72	P	o	o								DRB1*03:72		WD	
DRB1*03:73	P	WD	WD			WD					DRB1*03:73			
DRB1*03:76	P	WD	WD			WD					DRB1*03:76		WD	
DRB1*03:84	P	WD	WD			WD					DRB1*03:84			
DRB1*03:93	P	WD	WD			WD					DRB1*03:93			
DRB1*03:100	P	WD	WD			WD					DRB1*03:100			
DRB1*04:01	P	C	C	C	C	C	C	C	C	C	DRB1*04:01	C	C	C

Supplementary Table 3d. Comparison of HLA-DRB1 P group two-field assignments among CIWD catalogs [a]

		3.0.0 CIWD									2.0.0 CWD		EFI CWD	China CWD
		Category by Population [b]												
**HLA-DRB1 IPD-HLA/IMGT 3.31.0**	**Nomenclature Designation [a]**	**Highest Frequency**	Total	**AFA**	**API**	**EURO**	**MENA**	**HIS**	**NAM**	**UNK**	**HLA-DRB1 IPD-IMGT/HLA 3.9.0**	**Category**	**Category**	**Category**
DRB1*04:02	P	C	C	C	C	C	C	C	C	C	DRB1*04:02	C	C	C
DRB1*04:03	P	C	C	C	C	C	C	C	C	C	DRB1*04:03	C	C	C
DRB1*04:04	P	C	C	C	C	C	C	C	C	C	DRB1*04:04	C	C	C
DRB1*04:05	P	C	C	C	C	C	C	C	C	C	DRB1*04:05	C	C	C
DRB1*04:06	P	C	C	C	C	C	C	C	C	C	DRB1*04:06	C	C	C
DRB1*04:07	P	C	C	C	C	C	C	C	C	C	DRB1*04:07	C	C	C
DRB1*04:08	P	C	C	C	C	C	C	C	C	C	DRB1*04:08	C	C	C
DRB1*04:09	P	I	I	WD	I	I		I		I	DRB1*04:09	C	WD	
DRB1*04:10	P	C	C	C	C	I	C	C	C	C	DRB1*04:10	C	WD	C
DRB1*04:11	P	C	C	C	C	C	WD	C	C	C	DRB1*04:11	C	WD	WD
DRB1*04:12	P	o	o								DRB1*04:12	WD		
DRB1*04:13	P	C	I		I	I	C			I	DRB1*04:13	WD	WD	WD
DRB1*04:14	P	I	I			I				I	DRB1*04:14	WD	WD	
DRB1*04:15	P	WD	WD			WD				WD	DRB1*04:15	WD	WD	
DRB1*04:16	P	WD	WD			WD					DRB1*04:16	WD		
DRB1*04:17	P	C	I	WD		WD		C	C	I	DRB1*04:17	C		WD
DRB1*04:18	P	C	I		C					WD	DRB1*04:18	WD		
DRB1*04:19	P	I	WD			WD		I			DRB1*04:19	WD		
DRB1*04:21	P	WD	WD			WD					DRB1*04:21		WD	
DRB1*04:22	P	o	o								DRB1*04:22	WD		
DRB1*04:23	P	I	WD			WD		I			DRB1*04:23	WD		
DRB1*04:25	P	I	WD					I			DRB1*04:25	WD		
DRB1*04:26	P	I	WD			WD				I	DRB1*04:26	WD	WD	
DRB1*04:27	P	WD	WD								DRB1*04:27			
DRB1*04:31	P	WD	WD			WD					DRB1*04:31		WD	
DRB1*04:32	P	o	o								DRB1*04:32		WD	
DRB1*04:33	P	WD	WD			WD					DRB1*04:33	WD		
DRB1*04:34	P	WD	WD			WD					DRB1*04:34		WD	

Supplementary Table 3d. Comparison of HLA-DRB1 P group two-field assignments among CIWD catalogs [a]

		3.0.0 CIWD									2.0.0 CWD		EFI CWD	China CWD
		Category by Population [b]												
**HLA-DRB1 IPD-HLA/IMGT 3.31.0**	**Nomenclature Designation [a]**	**Highest Frequency**	Total	**AFA**	**API**	**EURO**	**MENA**	**HIS**	**NAM**	**UNK**	**HLA-DRB1 IPD-IMGT/HLA 3.9.0**	**Category**	**Category**	**Category**
DRB1*04:35	P	WD	WD			WD				WD	DRB1*04:35	WD	WD	
DRB1*04:36	P	WD	WD		WD						DRB1*04:36			
DRB1*04:37	P	I	WD			WD		I		WD	DRB1*04:37		WD	
DRB1*04:38	P	I	I			I		WD		I	DRB1*04:38	C	WD	WD
DRB1*04:39	P	WD	WD			WD					DRB1*04:39			
DRB1*04:40	P	WD	WD			WD				WD	DRB1*04:40	WD		
DRB1*04:41	P	I	WD		I					I	DRB1*04:41	WD		
DRB1*04:42	P	WD	WD		WD	WD					DRB1*04:42			
DRB1*04:43	P	WD	WD			WD					DRB1*04:43			
DRB1*04:44	P	WD	WD			WD					DRB1*04:44		WD	
DRB1*04:50	P	I	WD			I				WD	DRB1*04:50	WD	WD	
DRB1*04:51	P	WD	WD		WD						DRB1*04:51	WD		
DRB1*04:54	P	I	WD					I			DRB1*04:54	WD		
DRB1*04:56	P	WD	WD		WD	WD					DRB1*04:56		WD	
DRB1*04:59	P	WD	WD			WD					DRB1*04:59			WD
DRB1*04:60	P	WD	WD			WD					DRB1*04:60		WD	
DRB1*04:62	P	I	WD		I						DRB1*04:62			
DRB1*04:64	P	WD	WD			WD					DRB1*04:64		WD	
DRB1*04:68	P	WD	WD					WD			DRB1*04:68			
DRB1*04:70	P	WD	WD			WD					DRB1*04:70		WD	
DRB1*04:71	P	WD	WD			WD					DRB1*04:71		WD	
DRB1*04:72	P	WD	WD			WD				WD	DRB1*04:72			
DRB1*04:76	P	WD	WD			WD					DRB1*04:76			
DRB1*04:79	P	WD	WD					WD			DRB1*04:79			
DRB1*04:83	P	WD	WD			WD					DRB1*04:83		WD	
DRB1*04:88	P	I	WD		I	WD					DRB1*04:88		WD	WD
DRB1*04:93	P	o	o								DRB1*04:93			WD
DRB1*04:95	P	WD	WD		WD						DRB1*04:95			

Supplementary Table 3d. Comparison of HLA-DRB1 P group two-field assignments among CIWD catalogs [a]

		3.0.0 CIWD									2.0.0 CWD		EFI CWD	China CWD
		Category by Population [b]												
**HLA-DRB1 IPD-HLA/IMGT 3.31.0**	**Nomenclature Designation [a]**	**Highest Frequency**	Total	**AFA**	**API**	**EURO**	**MENA**	**HIS**	**NAM**	**UNK**	**HLA-DRB1 IPD-IMGT/HLA 3.9.0**	**Category**	**Category**	**Category**
DRB1*04:96	P	WD	WD			WD	WD				DRB1*04:96		WD	
DRB1*04:101	P	WD	WD								DRB1*04:101			
DRB1*04:105	P	I	WD		I	WD					DRB1*04:105		WD	
DRB1*04:127	P	WD	WD							WD	DRB1*04:127			
DRB1*07:01	P	C	C	C	C	C	C	C	C	C	DRB1*07:01	C	C	C
DRB1*07:03	P	C	I		C	WD	WD			I	DRB1*07:03	WD		
DRB1*07:05	P	I	I			I		WD		I	DRB1*07:05	WD		
DRB1*07:07	P	I	I			I				WD	DRB1*07:07	WD		
DRB1*07:10N	N	WD	WD			WD					DRB1*07:10N			
DRB1*07:11	P	I	WD			WD		I		WD	DRB1*07:11	WD		WD
DRB1*07:13	P	I	WD		I						DRB1*07:13	WD		WD
DRB1*07:15	P	WD	WD					WD			DRB1*07:15			
DRB1*07:16	P	I	WD			WD		I			DRB1*07:16			
DRB1*07:23	P	WD	WD			WD					DRB1*07:23			
DRB1*07:26N	N	WD	WD			WD					DRB1*07:26N			
DRB1*07:29	P	WD	WD			WD					DRB1*07:29			
DRB1*07:30	P	WD	WD			WD					DRB1*07:30			
DRB1*07:32	P	WD	WD			WD					DRB1*07:32			
DRB1*07:39	P	WD	WD			WD					DRB1*07:39			
DRB1*07:61	P	WD	WD				WD			WD	DRB1*07:61			
DRB1*08:01	P	C	C	C	C	C	C	C	C	C	DRB1*08:01	C	C	WD
DRB1*08:02	P	C	C	C	C	C	C	C	C	C	DRB1*08:02	C	C	C
DRB1*08:03	P	C	C	C	C	C	C	C	C	C	DRB1*08:03	C	C	C
DRB1*08:04	P	C	C	C	C	C	C	C	C	C	DRB1*08:04	C	C	WD
DRB1*08:05	P	I	WD			WD				I	DRB1*08:05	WD	WD	
DRB1*08:06	P	C	C	C	I	C	C	C	C	C	DRB1*08:06	C	C	
DRB1*08:07	P	C	C	WD		I		C	C	C	DRB1*08:07	C	WD	
DRB1*08:08	P	C	WD	C			WD			I	DRB1*08:08	WD		

Supplementary Table 3d. Comparison of HLA-DRB1 P group two-field assignments among CIWD catalogs [a]

		3.0.0 CIWD									2.0.0 CWD		EFI CWD	China CWD
		Category by Population [b]												
HLA-DRB1 IPD-HLA/IMGT 3.31.0	Nomenclature Designation [a]	Highest Frequency	Total	AFA	API	EURO	MENA	HIS	NAM	UNK	HLA-DRB1 IPD-IMGT/HLA 3.9.0	Category	Category	Category
DRB1*08:09	P	C	I		C	WD		WD		I	DRB1*08:09	WD	WD	C
DRB1*08:10	P	C	I	WD	I	I	C	C	C	C	DRB1*08:10	C	C	
DRB1*08:11	P	C	I	C		I		C	C	C	DRB1*08:11	C	WD	
DRB1*08:12	P	I	WD		I						DRB1*08:12	WD		WD
DRB1*08:13	P	I	WD			WD		I		I	DRB1*08:13	WD		
DRB1*08:14	P	I	WD		I						DRB1*08:14	WD		WD
DRB1*08:16	P	WD	WD			WD					DRB1*08:16			
DRB1*08:17	P	I	I			I				WD	DRB1*08:17	WD	WD	
DRB1*08:18	P	I	I		I	WD		I		I	DRB1*08:18	WD	WD	WD
DRB1*08:19	P	I	WD		I						DRB1*08:19	WD		WD
DRB1*08:20	P	WD	WD	WD							DRB1*08:20	WD		
DRB1*08:22	P	WD	WD								DRB1*08:22			
DRB1*08:26	P	WD	WD			WD					DRB1*08:26	WD	WD	
DRB1*08:28	P	WD	WD			WD					DRB1*08:28	WD		
DRB1*08:30	P	o	o								DRB1*08:30			WD
DRB1*08:32	P	o	o								DRB1*08:32			WD
DRB1*08:34	P	WD	WD			WD					DRB1*08:34			
DRB1*08:35	P	o	o								DRB1*08:35			WD
DRB1*08:36	P	o	o								DRB1*08:36			WD
DRB1*08:40	P	o	o								DRB1*08:40		WD	
DRB1*08:41	P	o	o								DRB1*08:41			WD
DRB1*08:44	P	WD	WD			WD					DRB1*08:44		WD	
DRB1*08:82	P	I	WD			WD		I		I	DRB1*08:82			
DRB1*09:01	P	C	C	C	C	C	C	C	C	C	DRB1*09:01	C	C	C
DRB1*09:02	P	WD	WD								DRB1*09:02			
DRB1*09:04	P	I	WD		I						DRB1*09:04	WD		WD
DRB1*09:06	P	I	WD			WD		I		WD	DRB1*09:06	WD		WD
DRB1*09:07	P	o	o								DRB1*09:07			WD

Supplementary Table 3d. Comparison of HLA-DRB1 P group two-field assignments among CIWD catalogs [a]

		3.0.0 CIWD									2.0.0 CWD		EFI CWD	China CWD
		Category by Population [b]												
HLA-DRB1 IPD-HLA/IMGT 3.31.0	Nomenclature Designation [a]	Highest Frequency	Total	AFA	API	EURO	MENA	HIS	NAM	UNK	HLA-DRB1 IPD-IMGT/HLA 3.9.0	Category	Category	Category
DRB1*09:10	P	o	o								DRB1*09:10			WD
DRB1*09:20	P	WD	WD			WD					DRB1*09:20			
DRB1*09:27	P	WD	WD		WD						DRB1*09:27			
DRB1*10:01	P	C	C	C	C	C	C	C	C	C	DRB1*10:01	C	C	C
DRB1*10:02	P	I	WD		I	WD					DRB1*10:02		WD	
DRB1*10:06	P	WD	WD		WD						DRB1*10:06			
DRB1*11:01	P	C	C	C	C	C	C	C	C	C	DRB1*11:01	C	C	C
DRB1*11:02	P	C	C	C	C	C	C	C	C	C	DRB1*11:02	C	C	WD
DRB1*11:03	P	C	C	C	C	C	C	C	C	C	DRB1*11:03	C	C	WD
DRB1*11:04	P	C	C	C	C	C	C	C	C	C	DRB1*11:04	C	C	C
DRB1*11:05	P	I	WD		I					I	DRB1*11:05	WD		
DRB1*11:06	P	C	C	WD	C	I	C	I	C	C	DRB1*11:06	C	WD	C
DRB1*11:07	P	I	WD	WD		WD		I		WD	DRB1*11:07	WD		
DRB1*11:08	P	C	I	WD	C	I	WD		WD	I	DRB1*11:08	WD		WD
DRB1*11:09	P	I	I			I	WD			I	DRB1*11:09	C		
DRB1*11:10	P	C	I	C		WD		I		I	DRB1*11:10	C		
DRB1*11:11	P	C	C	WD	C	I	WD	I		C	DRB1*11:11	C	WD	WD
DRB1*11:12	P	C	C	WD	I	C	C	I		I	DRB1*11:12	C		WD
DRB1*11:13	P	I	I	WD		I	WD	I		I	DRB1*11:13	WD		
DRB1*11:14	P	C	I	C	WD	I		I	WD	I	DRB1*11:14	WD	WD	
DRB1*11:15	P	C	C	WD	WD	C	C	C	C	C	DRB1*11:15	C	WD	WD
DRB1*11:16	P	WD	WD			WD	WD				DRB1*11:16	WD	WD	
DRB1*11:17	P	C	I	C		WD		I	C	I	DRB1*11:17	C		
DRB1*11:18	P	I	I		I	WD		WD		I	DRB1*11:18	WD		
DRB1*11:19	P	I	I		WD	I		I		I	DRB1*11:19	C		WD
DRB1*11:20	P	WD	WD			WD					DRB1*11:20	WD		WD
DRB1*11:21	P	WD	WD			WD					DRB1*11:21			
DRB1*11:23	P	I	WD			WD				I	DRB1*11:23			WD

Supplementary Table 3d. Comparison of HLA-DRB1 P group two-field assignments among CIWD catalogs [a]

		3.0.0 CIWD									2.0.0 CWD		EFI CWD	China CWD
		Category by Population [b]												
**HLA-DRB1 IPD-HLA/IMGT 3.31.0**	**Nomenclature Designation [a]**	**Highest Frequency**	Total	**AFA**	**API**	**EURO**	**MENA**	**HIS**	**NAM**	**UNK**	**HLA-DRB1 IPD-IMGT/HLA 3.9.0**	**Category**	**Category**	**Category**
DRB1*11:24	P	I	I	WD		I	WD	WD		I	DRB1*11:24	WD		
DRB1*11:25	P	WD	WD			WD					DRB1*11:25	WD		
DRB1*11:26	P	WD	WD			WD				WD	DRB1*11:26			
DRB1*11:27	P	I	I		WD	I		I		WD	DRB1*11:27			WD
DRB1*11:28	P	I	I	WD	I	I	WD	I		I	DRB1*11:28	WD		WD
DRB1*11:29	P	I	I	WD	I	I	WD			I	DRB1*11:29	WD	WD	WD
DRB1*11:32	P	WD	WD			WD				WD	DRB1*11:32	WD		
DRB1*11:33	P	WD	WD			WD					DRB1*11:33			
DRB1*11:34	P	I	I			I				WD	DRB1*11:34	WD		
DRB1*11:35	P	WD	WD			WD	WD				DRB1*11:35			
DRB1*11:36	P	I	I		WD	I	WD			I	DRB1*11:36	WD		
DRB1*11:37	P	WD	WD		WD	WD					DRB1*11:37	WD		WD
DRB1*11:39	P	I	I			I				I	DRB1*11:39	WD		WD
DRB1*11:41	P	WD	WD			WD					DRB1*11:41			
DRB1*11:42	P	I	WD			WD	WD	I		I	DRB1*11:42	WD	WD	
DRB1*11:43	P	I	I			I		I		I	DRB1*11:43	WD		
DRB1*11:44	P	WD	WD			WD					DRB1*11:44			
DRB1*11:45	P	WD	WD					WD			DRB1*11:45	WD		
DRB1*11:47	P	C	WD					I	C	WD	DRB1*11:47	WD		
DRB1*11:48	P	WD	WD	WD							DRB1*11:48			
DRB1*11:49	P	WD	WD			WD					DRB1*11:49			
DRB1*11:51	P	WD	WD								DRB1*11:51			
DRB1*11:54	P	o	o								DRB1*11:54			WD
DRB1*11:56	P	WD	WD			WD					DRB1*11:56	WD		
DRB1*11:57	P	o	o								DRB1*11:57			WD
DRB1*11:58	P	WD	WD			WD					DRB1*11:58			
DRB1*11:60	P	WD	WD								DRB1*11:60			
DRB1*11:61	P	WD	WD			WD					DRB1*11:61			

Supplementary Table 3d. Comparison of HLA-DRB1 P group two-field assignments among CIWD catalogs [a]

		3.0.0 CIWD									2.0.0 CWD		EFI CWD	China CWD
		Category by Population [b]												
HLA-DRB1 IPD-HLA/IMGT 3.31.0	Nomenclature Designation [a]	Highest Frequency	Total	AFA	API	EURO	MENA	HIS	NAM	UNK	HLA-DRB1 IPD-IMGT/HLA 3.9.0	Category	Category	Category
DRB1*11:62	P	WD	WD		WD						DRB1*11:62			
DRB1*11:65	P	WD	WD	WD							DRB1*11:65			
DRB1*11:66	P	C	I		C						DRB1*11:66	WD		
DRB1*11:69	P	WD	WD			WD					DRB1*11:69	WD		
DRB1*11:75	P	o	o								DRB1*11:75			WD
DRB1*11:76	P	WD	WD			WD					DRB1*11:76			
DRB1*11:78	P	WD	WD			WD					DRB1*11:78			
DRB1*11:81	P	WD	WD				WD				DRB1*11:81			
DRB1*11:84	P	I	WD			WD		I			DRB1*11:84			
DRB1*11:88	P	WD	WD			WD					DRB1*11:88			
DRB1*11:93	P	WD	WD								DRB1*11:93			
DRB1*11:94	P	WD	WD			WD					DRB1*11:94			
DRB1*11:96	P	WD	WD								DRB1*11:96			
DRB1*11:99	P	WD	WD			WD					DRB1*11:99			
DRB1*11:101	P	WD	WD		WD						DRB1*11:101			WD
DRB1*11:103	P	WD	WD			WD					DRB1*11:103			
DRB1*11:104	P	WD	WD			WD					DRB1*11:104			
DRB1*11:106	P	o	o								DRB1*11:106			WD
DRB1*11:111	P	o	o								DRB1*11:111			WD
DRB1*11:113	P	WD	WD			WD				WD	DRB1*11:113			
DRB1*11:125	P	WD	WD			WD					DRB1*11:125			
DRB1*11:128	P	WD	WD			WD					DRB1*11:128			
DRB1*11:137	P	WD	WD			WD					DRB1*11:137			
DRB1*11:145	P	WD	WD				WD				DRB1*11:145			
DRB1*11:179	P	WD	WD				WD				DRB1*11:179			
DRB1*12:01	P	C	C	C	C	C	C	C	C	C	DRB1*12:01	C	C	C
DRB1*12:02	P	C	C	C	C	C	C	C	C	C	DRB1*12:02	C	C	C
DRB1*12:03	P	I	I			I				I	DRB1*12:03			

Supplementary Table 3d. Comparison of HLA-DRB1 P group two-field assignments among CIWD catalogs [a]

		3.0.0 CIWD									2.0.0 CWD		EFI CWD	China CWD
		Category by Population [b]												
**HLA-DRB1 IPD-HLA/IMGT 3.31.0**	**Nomenclature Designation [a]**	**Highest Frequency**	Total	**AFA**	**API**	**EURO**	**MENA**	**HIS**	**NAM**	**UNK**	**HLA-DRB1 IPD-IMGT/HLA 3.9.0**	**Category**	**Category**	**Category**
DRB1*12:04	P	WD	WD	WD						WD	DRB1*12:04	WD		
DRB1*12:05	P	WD	WD		WD	WD					DRB1*12:05	WD		WD
DRB1*12:08	P	WD	WD		WD						DRB1*12:08			WD
DRB1*12:11	P	WD	WD			WD					DRB1*12:11			
DRB1*12:14	P	o	o								DRB1*12:14			WD
DRB1*12:16	P	o	o								DRB1*12:16			WD
DRB1*12:18	P	o	o								DRB1*12:18			WD
DRB1*12:19	P	o	o								DRB1*12:19			WD
DRB1*12:20	P	WD	WD		WD						DRB1*12:20			WD
DRB1*12:24N	N	WD	WD			WD				WD	DRB1*12:24N			
DRB1*12:25	P	WD	WD	WD						WD	DRB1*12:25			
DRB1*12:27	P	o	o								DRB1*12:27			WD
DRB1*12:28	P	o	o								DRB1*12:28			WD
DRB1*12:48	P	WD	WD			WD					DRB1*12:48			
DRB1*13:01	P	C	C	C	C	C	C	C	C	C	DRB1*13:01	C	C	C
DRB1*13:02	P	C	C	C	C	C	C	C	C	C	DRB1*13:02	C	C	C
DRB1*13:03	P	C	C	C	C	C	C	C	C	C	DRB1*13:03	C	C	WD
DRB1*13:04	P	C	C	C	I	I	WD	C	C	C	DRB1*13:04	C	C	
DRB1*13:05	P	C	C	C	C	C	C	C	C	C	DRB1*13:05	C	C	WD
DRB1*13:06	P	I	I			I		WD		I	DRB1*13:06	WD	WD	
DRB1*13:07	P	I	WD		I	WD	WD			WD	DRB1*13:07	C	WD	WD
DRB1*13:08	P	I	WD		I	WD					DRB1*13:08	WD	WD	WD
DRB1*13:09	P	I	WD			WD		I			DRB1*13:09	WD		
DRB1*13:10	P	I	I	WD		I	WD	I		I	DRB1*13:10	C	WD	
DRB1*13:11	P	I	I		I	I	WD	I		I	DRB1*13:11	WD	WD	
DRB1*13:12	P	C	C	WD	C	WD		I		C	DRB1*13:12	C	WD	C
DRB1*13:13	P	WD	WD		WD						DRB1*13:13	WD		WD
DRB1*13:14	P	I	I	WD	I	I	WD	I		I	DRB1*13:14	C	WD	WD

Supplementary Table 3d. Comparison of HLA-DRB1 P group two-field assignments among CIWD catalogs [a]

		3.0.0 CIWD									2.0.0 CWD		EFI CWD	China CWD
		Category by Population [b]												
HLA-DRB1 IPD-HLA/IMGT 3.31.0	Nomenclature Designation [a]	Highest Frequency	Total	AFA	API	EURO	MENA	HIS	NAM	UNK	HLA-DRB1 IPD-IMGT/HLA 3.9.0	Category	Category	Category
DRB1*13:15	P	C	I			C	WD	WD		I	DRB1*13:15	WD	C	
DRB1*13:16	P	C	I	C		WD	WD	I	WD	I	DRB1*13:16	WD	WD	
DRB1*13:17	P	WD	WD			WD					DRB1*13:17	WD	WD	
DRB1*13:18	P	I	I			I	WD			I	DRB1*13:18	WD	WD	
DRB1*13:19	P	I	I		I	I	WD			WD	DRB1*13:19	WD	WD	WD
DRB1*13:20	P	WD	WD	WD		WD				WD	DRB1*13:20	C	WD	
DRB1*13:21	P	I	I			I	WD	WD		I	DRB1*13:21		WD	
DRB1*13:22	P	I	I			I				WD	DRB1*13:22	WD	WD	
DRB1*13:23	P	WD	WD								DRB1*13:23		WD	
DRB1*13:24	P	WD	WD			WD				WD	DRB1*13:24		WD	
DRB1*13:25	P	WD	WD								DRB1*13:25	WD		
DRB1*13:26	P	WD	WD			WD					DRB1*13:26	WD	WD	
DRB1*13:27	P	WD	WD	WD		WD					DRB1*13:27	WD	WD	
DRB1*13:28	P	WD	WD			WD					DRB1*13:28		WD	
DRB1*13:29	P	WD	WD			WD				WD	DRB1*13:29	WD	WD	
DRB1*13:31	P	C	I	C		WD		I	WD	I	DRB1*13:31	WD		
DRB1*13:32	P	WD	WD								DRB1*13:32		WD	
DRB1*13:33	P	WD	WD			WD					DRB1*13:33			
DRB1*13:34	P	WD	WD								DRB1*13:34			
DRB1*13:36	P	WD	WD			WD				WD	DRB1*13:36	WD		
DRB1*13:37	P	WD	WD	WD							DRB1*13:37	WD		
DRB1*13:38	P	I	WD	WD		I					DRB1*13:38	WD		
DRB1*13:39	P	WD	WD		WD	WD					DRB1*13:39	WD		
DRB1*13:40	P	I	WD			WD		I	WD	WD	DRB1*13:40	WD		
DRB1*13:41	P	WD	WD								DRB1*13:41	WD		
DRB1*13:42	P	I	WD		WD	WD		I			DRB1*13:42	WD		
DRB1*13:48	P	WD	WD			WD					DRB1*13:48			
DRB1*13:49	P	C	I		C	WD					DRB1*13:49	WD		

Supplementary Table 3d. Comparison of HLA-DRB1 P group two-field assignments among CIWD catalogs [a]

		3.0.0 CIWD									2.0.0 CWD		EFI CWD	China CWD
		Category by Population [b]												
HLA-DRB1 IPD-HLA/IMGT 3.31.0	Nomenclature Designation [a]	Highest Frequency	Total	AFA	API	EURO	MENA	HIS	NAM	UNK	HLA-DRB1 IPD-IMGT/HLA 3.9.0	Category	Category	Category
DRB1*13:50	P	WD	WD		WD	WD				WD	DRB1*13:50			WD
DRB1*13:52	P	WD	WD			WD					DRB1*13:52			
DRB1*13:53	P	WD	WD			WD					DRB1*13:53			
DRB1*13:54	P	WD	WD			WD	WD				DRB1*13:54			
DRB1*13:56	P	I	WD			WD		I		WD	DRB1*13:56	WD		
DRB1*13:59	P	C	WD			WD		C	C	I	DRB1*13:59	WD		
DRB1*13:60	P	o	o								DRB1*13:60	WD		
DRB1*13:61	P	WD	WD		WD	WD					DRB1*13:61			
DRB1*13:62	P	WD	WD			WD					DRB1*13:62		WD	
DRB1*13:63	P	WD	WD			WD					DRB1*13:63		WD	
DRB1*13:65	P	WD	WD			WD					DRB1*13:65			
DRB1*13:66	P	WD	WD								DRB1*13:66			
DRB1*13:67	P	WD	WD			WD					DRB1*13:67			
DRB1*13:69	P	WD	WD	WD							DRB1*13:69			
DRB1*13:70	P	WD	WD			WD					DRB1*13:70			
DRB1*13:75	P	WD	WD								DRB1*13:75			
DRB1*13:76	P	o	o								DRB1*13:76	WD		
DRB1*13:77	P	WD	WD								DRB1*13:77			
DRB1*13:79	P	WD	WD			WD				WD	DRB1*13:79			
DRB1*13:80	P	WD	WD			WD					DRB1*13:80			
DRB1*13:81	P	WD	WD								DRB1*13:81	WD		
DRB1*13:82	P	I	WD		I						DRB1*13:82	WD		
DRB1*13:87	P	WD	WD								DRB1*13:87			
DRB1*13:88	P	WD	WD			WD					DRB1*13:88			
DRB1*13:90	P	WD	WD			WD					DRB1*13:90			
DRB1*13:91	P	WD	WD			WD					DRB1*13:91			
DRB1*13:93	P	I	WD			WD				I	DRB1*13:93			
DRB1*13:95	P	WD	WD			WD					DRB1*13:95			

Supplementary Table 3d. Comparison of HLA-DRB1 P group two-field assignments among CIWD catalogs [a]

		3.0.0 CIWD									2.0.0 CWD		EFI CWD	China CWD
		Category by Population [b]												
**HLA-DRB1 IPD-HLA/IMGT 3.31.0**	**Nomenclature Designation [a]**	**Highest Frequency**	Total	**AFA**	**API**	**EURO**	**MENA**	**HIS**	**NAM**	**UNK**	**HLA-DRB1 IPD-IMGT/HLA 3.9.0**	**Category**	**Category**	**Category**
DRB1*13:96	P	WD	WD			WD					DRB1*13:96			
DRB1*13:97	P	WD	WD								DRB1*13:97			
DRB1*13:104	P	WD	WD			WD					DRB1*13:104			
DRB1*13:106	P	WD	WD			WD					DRB1*13:106			
DRB1*13:108	P	WD	WD			WD					DRB1*13:108			
DRB1*13:111	P	WD	WD			WD					DRB1*13:111			
DRB1*13:118	P	o	o								DRB1*13:118			WD
DRB1*13:124	P	WD	WD			WD					DRB1*13:124			
DRB1*13:128	P	I	WD			WD		I			DRB1*13:128			
DRB1*13:150	P	WD	WD			WD					DRB1*13:150			
DRB1*13:155	P	WD	WD								DRB1*13:155			
DRB1*13:158	P	WD	WD			WD					DRB1*13:158			
DRB1*13:187	P	WD	WD			WD					DRB1*13:187			
DRB1*14:01	P	C	C	C	C	C	C	C	C	C	DRB1*14:01	C	C	WD
DRB1*14:02	P	C	C	C	C	C	C	C	C	C	DRB1*14:02	C	C	WD
DRB1*14:03	P	C	C		C	I	C	WD		C	DRB1*14:03	C	WD	C
DRB1*14:04	P	C	C	C	C	C	C	C	C	C	DRB1*14:04	C	C	C
DRB1*14:05	P	C	C	WD	C	I	C	I		C	DRB1*14:05	C	WD	C
DRB1*14:06	P	C	C	C	C	C	C	C	C	C	DRB1*14:06	C		WD
DRB1*14:07	P	C	C	WD	C	C	C	C	C	C	DRB1*14:07	C	WD	C
DRB1*14:08	P	C	I		C	WD				C	DRB1*14:08	WD		
DRB1*14:09	P	I	WD		I						DRB1*14:09	WD		
DRB1*14:10	P	C	I		C	WD			C	I	DRB1*14:10	WD		WD
DRB1*14:11	P	C	I		C	WD	WD	WD		WD	DRB1*14:11	WD		WD
DRB1*14:12	P	C	I		I	WD	C			WD	DRB1*14:12	WD		WD
DRB1*14:13	P	I	WD			WD		I		WD	DRB1*14:13	WD		
DRB1*14:14	P	o	o								DRB1*14:14	WD		
DRB1*14:15	P	C	C	WD	C	WD	WD		C	I	DRB1*14:15	WD		WD

Supplementary Table 3d. Comparison of HLA-DRB1 P group two-field assignments among CIWD catalogs [a]

		3.0.0 CIWD									2.0.0 CWD		EFI CWD	China CWD
		Category by Population [b]												
**HLA-DRB1 IPD-HLA/IMGT 3.31.0**	**Nomenclature Designation [a]**	**Highest Frequency**	Total	**AFA**	**API**	**EURO**	**MENA**	**HIS**	**NAM**	**UNK**	**HLA-DRB1 IPD-IMGT/HLA 3.9.0**	**Category**	**Category**	**Category**
DRB1*14:16	P	I	I			I	WD			I	DRB1*14:16	WD		
DRB1*14:17	P	C	I		WD	I	C	I		I	DRB1*14:17	C	WD	
DRB1*14:18	P	C	I		C	WD				I	DRB1*14:18	C		WD
DRB1*14:19	P	C	I	WD	C	WD				WD	DRB1*14:19	WD		
DRB1*14:20	P	WD	WD			WD				WD	DRB1*14:20	WD		
DRB1*14:21	P	WD	WD			WD				WD	DRB1*14:21	WD		
DRB1*14:22	P	I	WD		I					WD	DRB1*14:22	WD		WD
DRB1*14:23	P	WD	WD			WD				WD	DRB1*14:23			
DRB1*14:24	P	C	I			WD		C	C	I	DRB1*14:24	C		
DRB1*14:25	P	I	WD		I	WD					DRB1*14:25	WD		WD
DRB1*14:27	P	WD	WD								DRB1*14:27			
DRB1*14:28	P	I	WD		I						DRB1*14:28	WD		
DRB1*14:29	P	o	o								DRB1*14:29	WD		
DRB1*14:32	P	WD	WD			WD					DRB1*14:32			
DRB1*14:33	P	I	WD		I	WD	WD			WD	DRB1*14:33	WD		WD
DRB1*14:35	P	WD	WD		WD	WD					DRB1*14:35			
DRB1*14:41	P	WD	WD					WD		WD	DRB1*14:41			
DRB1*14:44	P	WD	WD		WD						DRB1*14:44			WD
DRB1*14:46	P	WD	WD					WD			DRB1*14:46			
DRB1*14:47	P	WD	WD			WD					DRB1*14:47			
DRB1*14:48	P	C	WD			WD		C	WD	I	DRB1*14:48	WD		
DRB1*14:49	P	o	o								DRB1*14:49			WD
DRB1*14:50	P	WD	WD		WD				WD		DRB1*14:50			
DRB1*14:52	P	WD	WD					WD			DRB1*14:52			
DRB1*14:58	P	WD	WD			WD					DRB1*14:58			
DRB1*14:60	P	WD	WD			WD					DRB1*14:60			
DRB1*14:61	P	I	WD		I						DRB1*14:61	WD		WD
DRB1*14:68	P	I	WD		I						DRB1*14:68			

Supplementary Table 3d. Comparison of HLA-DRB1 P group two-field assignments among CIWD catalogs [a]

		3.0.0 CIWD									2.0.0 CWD		EFI CWD	China CWD
		Category by Population [b]												
**HLA-DRB1 IPD-HLA/IMGT 3.31.0**	**Nomenclature Designation [a]**	**Highest Frequency**	Total	**AFA**	**API**	**EURO**	**MENA**	**HIS**	**NAM**	**UNK**	**HLA-DRB1 IPD-IMGT/HLA 3.9.0**	**Category**	**Category**	**Category**
DRB1*14:69	P	I	WD		I						DRB1*14:69			
DRB1*14:70	P	WD	WD			WD				WD	DRB1*14:70	WD		
DRB1*14:75	P	WD	WD			WD					DRB1*14:75			
DRB1*14:79	P	WD	WD								DRB1*14:79			
DRB1*14:86	P	WD	WD			WD					DRB1*14:86			
DRB1*14:87	P	WD	WD			WD					DRB1*14:87			
DRB1*14:90	P	WD	WD								DRB1*14:90			
DRB1*14:95	P	WD	WD			WD					DRB1*14:95			
DRB1*14:97	P	WD	WD			WD					DRB1*14:97			
DRB1*14:99	P	WD	WD			WD					DRB1*14:99			
DRB1*14:101	P	WD	WD			WD					DRB1*14:101			
DRB1*14:106	P	WD	WD					WD			DRB1*14:106			
DRB1*14:109	P	WD	WD							WD	DRB1*14:109			
DRB1*14:110	P	WD	WD				WD				DRB1*14:110			
DRB1*14:111	P	WD	WD			WD					DRB1*14:111			
DRB1*14:114	P	WD	WD	WD							DRB1*14:114			
DRB1*14:122	P	WD	WD								DRB1*14:122			
DRB1*14:126	P	I	WD		I						DRB1*14:126			
DRB1*14:133	P	WD	WD								DRB1*14:133			
DRB1*14:141	P	WD	WD		WD						DRB1*14:141			WD
DRB1*15:01	P	C	C	C	C	C	C	C	C	C	DRB1*15:01	C	C	C
DRB1*15:02	P	C	C	C	C	C	C	C	C	C	DRB1*15:02	C	C	C
DRB1*15:03	P	C	C	C	C	C	C	C	C	C	DRB1*15:03	C	C	WD
DRB1*15:04	P	C	C	WD	C	I	WD	I	C	I	DRB1*15:04	C	WD	C
DRB1*15:05	P	WD	WD			WD					DRB1*15:05			WD
DRB1*15:06	P	C	C	WD	C	I	C	I	C	C	DRB1*15:06	C		WD
DRB1*15:07	P	I	I	WD	WD	I	WD			WD	DRB1*15:07	WD		WD
DRB1*15:10	P	WD	WD			WD					DRB1*15:10	WD		

Supplementary Table 3d. Comparison of HLA-DRB1 P group two-field assignments among CIWD catalogs [a]

		3.0.0 CIWD									2.0.0 CWD		EFI CWD	China CWD
		Category by Population [b]												
**HLA-DRB1 IPD-HLA/IMGT 3.31.0**	**Nomenclature Designation [a]**	**Highest Frequency**	Total	**AFA**	**API**	**EURO**	**MENA**	**HIS**	**NAM**	**UNK**	**HLA-DRB1 IPD-IMGT/HLA 3.9.0**	**Category**	**Category**	**Category**
DRB1*15:11	P	I	WD		I	WD	WD			WD	DRB1*15:11	WD		WD
DRB1*15:13	P	WD	WD			WD					DRB1*15:13			
DRB1*15:14	P	I	WD		I	WD				I	DRB1*15:14	WD		
DRB1*15:15	P	WD	WD		WD	WD					DRB1*15:15			
DRB1*15:18	P	I	I			I					DRB1*15:18	WD		
DRB1*15:20	P	I	WD		I	WD					DRB1*15:20	WD		
DRB1*15:22	P	WD	WD	WD		WD				WD	DRB1*15:22	WD		
DRB1*15:23	P	WD	WD	WD							DRB1*15:23	WD		
DRB1*15:24	P	I	WD			WD				I	DRB1*15:24	WD		
DRB1*15:31	P	WD	WD		WD						DRB1*15:31			
DRB1*15:33	P	WD	WD			WD					DRB1*15:33			
DRB1*15:36	P	WD	WD			WD					DRB1*15:36			
DRB1*15:37	P	WD	WD			WD					DRB1*15:37			
DRB1*15:38	P	I	WD		I						DRB1*15:38	WD		
DRB1*15:40	P	WD	WD			WD					DRB1*15:40			
DRB1*15:47	P	WD	WD		WD						DRB1*15:47			
DRB1*15:49	P	o	o								DRB1*15:49			WD
DRB1*15:55	P	WD	WD			WD					DRB1*15:55			
DRB1*15:56	P	I	WD				WD			I	DRB1*15:56			
DRB1*15:57	P	WD	WD	WD							DRB1*15:57			
DRB1*15:61	P	WD	WD								DRB1*15:61			
DRB1*15:65	P	WD	WD			WD					DRB1*15:65			
DRB1*15:67	P	WD	WD			WD					DRB1*15:67			
DRB1*15:68	P	I	WD		I						DRB1*15:68			
DRB1*15:93	P	WD	WD			WD					DRB1*15:93			
DRB1*15:96	P	WD	WD			WD					DRB1*15:96			
DRB1*16:01	P	C	C	C	C	C	C	C	C	C	DRB1*16:01	C	C	WD
DRB1*16:02	P	C	C	C	C	C	C	C	C	C	DRB1*16:02	C	C	C

Supplementary Table 3d. Comparison of HLA-DRB1 P group two-field assignments among CIWD catalogs [a]

		3.0.0 CIWD									2.0.0 CWD		EFI CWD	China CWD
		Category by Population [b]												
**HLA-DRB1 IPD-HLA/IMGT 3.31.0**	**Nomenclature Designation [a]**	**Highest Frequency**	Total	**AFA**	**API**	**EURO**	**MENA**	**HIS**	**NAM**	**UNK**	**HLA-DRB1 IPD-IMGT/HLA 3.9.0**	**Category**	**Category**	**Category**
DRB1*16:04	P	WD	WD			WD				WD	DRB1*16:04	C		
DRB1*16:05	P	C	I		I	I	C			I	DRB1*16:05	WD		WD
DRB1*16:07	P	I	I			WD	WD	I		I	DRB1*16:07	C		
DRB1*16:08	P	I	WD							I	DRB1*16:08			
DRB1*16:09	P	WD	WD			WD	WD				DRB1*16:09			WD
DRB1*16:10	P	I	WD		I						DRB1*16:10	WD		WD
DRB1*16:12	P	I	WD					I			DRB1*16:12	WD		
DRB1*16:15	P	WD	WD			WD					DRB1*16:15			
DRB1*16:22	P	WD	WD					WD			DRB1*16:22			

C, common; I, intermediate; WD, well-documented

[a] Alleles observed at least five times in any population group in the current dataset are included in this table. The exception are alleles that were observed in previous catalogs but that are not present or are observed less than five times. Note that this two-field designation is the equivalent of a "P" assignment so that alleles within a P group that encode proteins that differ outside of the antigen recognition domain are not listed separately (e.g., DRB1*14:54 is within the DRB1*14:01P assignment and is not listed in this table even though it was categorized as common in 2.0.0 CWD). The table does not list all alleles from IPD-IMGT/HLA version 3.31.0.

[b] Population groups include: AFA (African/African American), API (Asian/Pacific Islands), EURO (European/European descent), MENA (Middle East/North Coast of Africa), HIS (South or Central America/Hispanic/Latino), NAM (Native American populations) and UNK (unknown/not asked/multiple ancestries/other). Total is the overall population i.e., all groups combined. Highest frequency is the highest CIWD designation among all the individual groups.

[c] Not-CIWD, o.

Supplementary Table 3e. Comparison of HLA-DRB3/4/5 P group two-field assignments among CIWD catalogs [a]

		3.0.0 CIWD									2.0.0 CWD		EFI CWD	China CWD
		Category by Population [b]												
HLA-DRB3/4/5 IPD-HLA/IMGT 3.31.0	Nomenclature Designation [a]	Highest Frequency	Total	AFA	API	EURO	MENA	HIS	NAM	UNK	HLA-DRB3/4/5 IPD-IMGT/HLA 3.9.0	Category	Category	Category
DRB3*01:01	P	WD	WD	WD	WD	WD	WD	WD	WD	WD	DRB3*01:01	C	not available	
DRB3*01:02	P	WD	WD			WD				WD	DRB3*01:02	WD		
DRB3*01:03	P	o [d]	o								DRB3*01:03	WD		
DRB3*01:05	P	WD	WD					WD			DRB3*01:05			
DRB3*01:06	P	WD	WD					WD			DRB3*01:06			
DRB3*01:11	P	WD	WD		WD						DRB3*01:11			
DRB3*01:13	P	WD	WD		WD						DRB3*01:13			
DRB3*01:35	P	WD	WD					WD			DRB3*01:35			
DRB3*02:01	P	WD	WD	WD	WD	WD	WD	WD	WD	WD	DRB3*02:01	C		
DRB3*02:02	P	WD	WD	WD	WD	WD	WD	WD	WD	WD	DRB3*02:02	C		
DRB3*02:03	P	WD	WD			WD				WD	DRB3*02:03	WD		
DRB3*02:04	P	WD	WD			WD					DRB3*02:04			
DRB3*02:05	P	WD	WD			WD		WD			DRB3*02:05			
DRB3*02:06	P	WD	WD			WD					DRB3*02:06	WD		
DRB3*02:07	P	WD	WD	WD						WD	DRB3*02:07			
DRB3*02:09	P	WD	WD			WD					DRB3*02:09			
DRB3*02:10	P	WD	WD	WD	WD	WD		WD		WD	DRB3*02:10	C		
DRB3*02:11	P	WD	WD			WD		WD		WD	DRB3*02:11	WD		
DRB3*02:12	P	WD	WD			WD					DRB3*02:12			
DRB3*02:13	P	WD	WD			WD					DRB3*02:13			
DRB3*02:16	P	WD	WD			WD					DRB3*02:16			
DRB3*02:17	P	WD	WD			WD		WD		WD	DRB3*02:17	WD		
DRB3*02:19	P	WD	WD			WD					DRB3*02:19			
DRB3*02:20	P	WD	WD								DRB3*02:20			
DRB3*02:21	P	WD	WD			WD		WD			DRB3*02:21			
DRB3*02:22	P	WD	WD			WD					DRB3*02:22			
DRB3*02:23	P	WD	WD			WD					DRB3*02:23			
DRB3*02:25	P	WD	WD			WD					DRB3*02:25			

Supplementary Table 3e. Comparison of HLA-DRB3/4/5 P group two-field assignments among CIWD catalogs [a]

		3.0.0 CIWD									2.0.0 CWD		EFI CWD	China CWD
		Category by Population [b]												
HLA-DRB3/4/5 IPD-HLA/IMGT 3.31.0	Nomenclature Designation [a]	Highest Frequency	Total	AFA	API	EURO	MENA	HIS	NAM	UNK	HLA-DRB3/4/5 IPD-IMGT/HLA 3.9.0	Category	Category	Category
DRB3*02:32	P	WD	WD		WD	WD					DRB3*02:32			
DRB3*02:51	P	WD	WD					WD			DRB3*02:51			
DRB3*03:01	P	WD	WD	WD	WD	WD	WD	WD	WD	WD	DRB3*03:01	C		
DRB3*03:02	P	WD	WD			WD					DRB3*03:02			
DRB3*03:03	P	WD	WD								DRB3*03:03			
DRB4*01:01	P	WD	WD	WD	WD	WD	WD	WD	WD	WD	DRB4*01:01	C		
DRB4*01:02	P	WD	WD		WD	WD	WD	WD	WD	WD	DRB4*01:02	C		
DRB4*01:03N [c]	N	WD	WD	WD	WD	WD	WD	WD	WD	WD	DRB4*01:03N	C		
DRB4*01:04	P	WD	WD		WD						DRB4*01:04			
DRB4*01:05	P	WD	WD			WD		WD		WD	DRB4*01:05			
DRB4*01:07	P	WD	WD			WD					DRB4*01:07			
DRB4*01:16N	N	WD	WD								DRB4*01:16N			
DRB4*01:17	P	WD	WD			WD					DRB4*01:17			
DRB4*02:01N	N	WD	WD			WD					DRB4*02:01N	C		
DRB4*03:01N	N	WD	WD			WD					DRB4*03:01N	WD		
DRB5*01:01	P	WD	WD	WD	WD	WD	WD	WD	WD	WD	DRB5*01:01	C		
DRB5*01:02	P	WD	WD	WD	WD	WD	WD	WD	WD	WD	DRB5*01:02	C		
DRB5*01:03	P	WD	WD	WD	WD					WD	DRB5*01:03	C		
DRB5*01:04	P	WD	WD								DRB5*01:04			
DRB5*01:05	P	WD	WD			WD					DRB5*01:05			
DRB5*01:06	P	WD	WD								DRB5*01:06			
DRB5*01:08N	N	WD	WD	WD	WD					WD	DRB5*01:08N	C		
DRB5*01:09	P	WD	WD								DRB5*01:09			
DRB5*01:10N	N	WD	WD		WD	WD	WD	WD		WD	DRB5*01:10N	WD		
DRB5*01:11	P	WD	WD		WD						DRB5*01:11			
DRB5*01:15	P	WD	WD		WD						DRB5*01:15			
DRB5*01:20	P	WD	WD								DRB5*01:20			
DRB5*02:02	P	WD	WD	WD	WD	WD	WD	WD	WD	WD	DRB5*02:02	C		

Supplementary Table 3e. Comparison of HLA-DRB3/4/5 P group two-field assignments among CIWD catalogs [a]

		3.0.0 CIWD									2.0.0 CWD		EFI CWD	China CWD
		Category by Population [b]												
**HLA-DRB3/4/5 IPD-HLA/IMGT 3.31.0**	**Nomenclature Designation [a]**	**Highest Frequency**	Total	**AFA**	**API**	**EURO**	**MENA**	**HIS**	**NAM**	**UNK**	**HLA-DRB3/4/5 IPD-IMGT/HLA 3.9.0**	**Category**	**Category**	**Category**
DRB5*02:03	P	WD	WD		WD	WD		WD		WD	DRB5*02:03	WD		
DRB5*02:04	P	WD	WD			WD					DRB5*02:04			

C, common; WD, well-documented

[a] Alleles observed at least five times in any population group in the current dataset are included in this table. The exception are alleles that were observed in previous catalogs but that are not present or are observed less than five times. Note that this two-field designation is the equivalent of a "P" assignment so that alleles within a P group that encode a protein that differs outside of the antigen recognition domain are not listed separately (e.g.,DRB4*01:03 is within the DRB4*01:01P assignment and is not listed in this table even though it was categorized as common in 2.0.0 CWD). The table does not list all alleles from IPD-IMGT/HLA version 3.31.0.

[b] Population groups include: AFA (African/African American), API (Asian/Pacific Islander), EURO (European/European descent), MENA (Middle East/North Coast of Africa), HIS (Hispanic), NAM (Native American) and UNK (unknown, multiple ancestries or other). Total is the overall population i.e., all groups combined. Highest frequency is the highest CIWD designation among all the individual groups.

[c] Any non-expressed alleles which are excluded from the P group are listed. For example, DRB4*01:03:01:02N (sometimes labeled as DRB4*01:03N) is within the DRB4*01:01P group but is listed separately because it is not expressed.

[d] Not-CIWD, o.

Supplementary Table 3f. Comparison of HLA-DQB1 P group two-field assignments among CIWD catalogs [a]

		3.0.0 CIWD									2.0.0 CWD		EFI CWD	China CWD
		Category by Population [b]												
HLA-DQB1 IPD-HLA/IMGT 3.31.0	Nomenclature Designation [a]	Highest Frequency	Total	AFA	API	EURO	MENA	HIS	NAM	UNK	HLA-DQB1 IPD-IMGT/HLA 3.9.0	Category	Category	Category
DQB1*02:01	P	C	C	C	C	C	C	C	C	C	DQB1*02:01	C	C	C
DQB1*02:03	P	C	I	C		WD		I	C	C	DQB1*02:03	C	WD	WD
DQB1*02:05	P	WD	WD			WD				WD	DQB1*02:05			
DQB1*02:07	P	C	I		C	WD				WD	DQB1*02:07			
DQB1*02:12	P	WD	WD			WD	WD			WD	DQB1*02:12		WD	WD
DQB1*02:14	P	I	I			I		WD		I	DQB1*02:14			
DQB1*02:16	P	WD	WD			WD					DQB1*02:16			
DQB1*02:17	P	WD	WD			WD					DQB1*02:17			
DQB1*02:18N	N	WD	WD			WD					DQB1*02:18N		WD	
DQB1*02:19	P	I	WD			WD	WD			I	DQB1*02:19			
DQB1*02:20N	N	WD	WD			WD					DQB1*02:20N		WD	
DQB1*02:21	P	WD	WD			WD					DQB1*02:21		WD	
DQB1*02:23	P	WD	WD			WD					DQB1*02:23			
DQB1*02:24	P	WD	WD			WD					DQB1*02:24		WD	
DQB1*02:26	P	I	WD			WD				I	DQB1*02:26			
DQB1*02:27	P	WD	WD			WD					DQB1*02:27		WD	
DQB1*02:28	P	WD	WD			WD					DQB1*02:28			
DQB1*02:29	P	WD	WD			WD				WD	DQB1*02:29			
DQB1*02:30	P	WD	WD			WD					DQB1*02:30		WD	
DQB1*02:33	P	WD	WD			WD					DQB1*02:33			
DQB1*02:46	P	WD	WD			WD					DQB1*02:46			
DQB1*02:53Q	Q	I	I			I					DQB1*02:53Q		WD	
DQB1*02:63	P	WD	WD			WD					DQB1*02:63			
DQB1*03:01	P	C	C	C	C	C	C	C	C	C	DQB1*03:01	C	C	C
DQB1*03:02	P	C	C	C	C	C	C	C	C	C	DQB1*03:02	C	C	C
DQB1*03:03	P	C	C	C	C	C	C	C	C	C	DQB1*03:03	C	C	C
DQB1*03:04	P	C	C	C	C	C	C	C	C	C	DQB1*03:04	C	C	WD

Supplementary Table 3f. Comparison of HLA-DQB1 P group two-field assignments among CIWD catalogs [a]

		3.0.0 CIWD									2.0.0 CWD		EFI CWD	China CWD
		Category by Population [b]												
HLA-DQB1 IPD-HLA/IMGT 3.31.0	Nomenclature Designation [a]	Highest Frequency	Total	AFA	API	EURO	MENA	HIS	NAM	UNK	HLA-DQB1 IPD-IMGT/HLA 3.9.0	Category	Category	Category
DQB1*03:05	P	C	C	C	C	C	C	C	C	C	DQB1*03:05	C	C	WD
DQB1*03:06	P	WD	WD								DQB1*03:06			
DQB1*03:07	P	o [c]	o								DQB1*03:07		WD	
DQB1*03:08	P	I	WD			WD				I	DQB1*03:08		WD	
DQB1*03:10	P	WD	WD		WD	WD					DQB1*03:10		WD	
DQB1*03:11	P	C	I		C					WD	DQB1*03:11			
DQB1*03:12	P	I	I			I				WD	DQB1*03:12		C	
DQB1*03:13	P	C	I		C	WD				I	DQB1*03:13	WD	WD	WD
DQB1*03:14	P	I	WD		I	WD		I			DQB1*03:14			WD
DQB1*03:16	P	C	WD	C						WD	DQB1*03:16			
DQB1*03:17	P	I	WD		I						DQB1*03:17			WD
DQB1*03:18	P	WD	WD			WD				WD	DQB1*03:18			
DQB1*03:20	P	WD	WD			WD					DQB1*03:20			
DQB1*03:25	P	C	WD			WD		I	C	I	DQB1*03:25			
DQB1*03:26	P	I	WD		I						DQB1*03:26			WD
DQB1*03:27	P	o	o								DQB1*03:27			WD
DQB1*03:30	P	WD	WD			WD					DQB1*03:30		WD	WD
DQB1*03:34	P	o	o								DQB1*03:34			WD
DQB1*03:36	P	WD	WD			WD					DQB1*03:36			
DQB1*03:37	P	I	WD			WD				I	DQB1*03:37		WD	
DQB1*03:38	P	o	o								DQB1*03:38			WD
DQB1*03:40	P	WD	WD			WD					DQB1*03:40			
DQB1*03:44	P	o	o								DQB1*03:44			WD
DQB1*03:45	P	WD	WD			WD					DQB1*03:45			
DQB1*03:48	P	WD	WD			WD					DQB1*03:48			
DQB1*03:53	P	WD	WD				WD				DQB1*03:53			
DQB1*03:54	P	WD	WD								DQB1*03:54			

Supplementary Table 3f. Comparison of HLA-DQB1 P group two-field assignments among CIWD catalogs [a]

		3.0.0 CIWD									2.0.0 CWD		EFI CWD	China CWD
		Category by Population [b]												
HLA-DQB1 IPD-HLA/IMGT 3.31.0	Nomenclature Designation [a]	Highest Frequency	Total	AFA	API	EURO	MENA	HIS	NAM	UNK	HLA-DQB1 IPD-IMGT/HLA 3.9.0	Category	Category	Category
DQB1*03:55	P	WD	WD			WD				WD	DQB1*03:55			
DQB1*03:56	P	WD	WD			WD					DQB1*03:56			
DQB1*03:58	P	I	WD			WD				I	DQB1*03:58			
DQB1*03:59	P	WD	WD			WD					DQB1*03:59			
DQB1*03:62	P	WD	WD		WD						DQB1*03:62			
DQB1*03:63	P	WD	WD			WD					DQB1*03:63			
DQB1*03:64	P	WD	WD			WD					DQB1*03:64			
DQB1*03:65	P	WD	WD			WD					DQB1*03:65			
DQB1*03:67	P	WD	WD			WD				WD	DQB1*03:67			
DQB1*03:68	P	WD	WD			WD					DQB1*03:68			
DQB1*03:69	P	WD	WD								DQB1*03:69			
DQB1*03:70	P	I	WD					I			DQB1*03:70			
DQB1*03:72	P	I	WD					I		I	DQB1*03:72			
DQB1*03:76	P	WD	WD			WD					DQB1*03:76			
DQB1*03:78	P	WD	WD		WD						DQB1*03:78			
DQB1*03:81	P	WD	WD			WD					DQB1*03:81			
DQB1*03:93	P	o	o								DQB1*03:93			WD
DQB1*03:105	P	I	WD					I			DQB1*03:105			
DQB1*03:106	P	I	WD		WD			I			DQB1*03:106			
DQB1*03:109	P	WD	WD			WD					DQB1*03:109			
DQB1*03:114	P	WD	WD			WD					DQB1*03:114			
DQB1*03:118N	N	WD	WD		WD						DQB1*03:118N			
DQB1*03:121	P	WD	WD								DQB1*03:121			
DQB1*03:126	P	WD	WD								DQB1*03:126			
DQB1*03:127	P	WD	WD								DQB1*03:127			
DQB1*03:130	P	WD	WD			WD					DQB1*03:130			
DQB1*03:132	P	I	WD					I			DQB1*03:132			

Supplementary Table 3f. Comparison of HLA-DQB1 P group two-field assignments among CIWD catalogs [a]

		3.0.0 CIWD									2.0.0 CWD		EFI CWD	China CWD
		Category by Population [b]												
HLA-DQB1 IPD-HLA/IMGT 3.31.0	Nomenclature Designation [a]	Highest Frequency	Total	AFA	API	EURO	MENA	HIS	NAM	UNK	HLA-DQB1 IPD-IMGT/HLA 3.9.0	Category	Category	Category
DQB1*03:134	P	WD	WD			WD					DQB1*03:134			
DQB1*03:136	P	WD	WD			WD				WD	DQB1*03:136			
DQB1*03:138	P	WD	WD			WD					DQB1*03:138			
DQB1*03:141	P	WD	WD			WD					DQB1*03:141			
DQB1*03:162	P	WD	WD			WD					DQB1*03:162			
DQB1*03:197Q	Q	WD	WD				WD				DQB1*03:197Q			
DQB1*03:201	P	WD	WD			WD					DQB1*03:201			
DQB1*03:205	P	WD	WD			WD					DQB1*03:205			
DQB1*03:207	P	WD	WD								DQB1*03:207			
DQB1*04:01	P	C	C	WD	C	I	C	C	C	C	DQB1*04:01	C	C	C
DQB1*04:02	P	C	C	C	C	C	C	C	C	C	DQB1*04:02	C	C	C
DQB1*04:03	P	WD	WD			WD					DQB1*04:03			
DQB1*04:04	P	WD	WD			WD					DQB1*04:04			
DQB1*04:11	P	WD	WD			WD					DQB1*04:11			
DQB1*04:12	P	I	WD		I						DQB1*04:12			
DQB1*04:22	P	WD	WD								DQB1*04:22			
DQB1*05:01	P	C	C	C	C	C	C	C	C	C	DQB1*05:01	C	C	C
DQB1*05:02	P	C	C	C	C	C	C	C	C	C	DQB1*05:02	C	C	C
DQB1*05:03	P	C	C	C	C	C	C	C	C	C	DQB1*05:03	C	C	C
DQB1*05:04	P	C	C	C	C	C	C	C	C	C	DQB1*05:04	C	C	WD
DQB1*05:05	P	WD	WD			WD	WD			WD	DQB1*05:05			WD
DQB1*05:06	P	WD	WD		WD						DQB1*05:06			
DQB1*05:09	P	WD	WD			WD					DQB1*05:09			
DQB1*05:11	P	WD	WD			WD					DQB1*05:11			
DQB1*05:12	P	WD	WD			WD					DQB1*05:12			
DQB1*05:20	P	WD	WD			WD					DQB1*05:20			
DQB1*05:23	P	C	I		C						DQB1*05:23			

Supplementary Table 3f. Comparison of HLA-DQB1 P group two-field assignments among CIWD catalogs [a]

		3.0.0 CIWD									2.0.0 CWD		EFI CWD	China CWD
		Category by Population [b]												
HLA-DQB1 IPD-HLA/IMGT 3.31.0	Nomenclature Designation [a]	Highest Frequency	Total	AFA	API	EURO	MENA	HIS	NAM	UNK	HLA-DQB1 IPD-IMGT/HLA 3.9.0	Category	Category	Category
DQB1*05:26	P	WD	WD		WD						DQB1*05:26			
DQB1*05:34	P	WD	WD								DQB1*05:34			
DQB1*05:44	P	WD	WD								DQB1*05:44			
DQB1*05:49	P	WD	WD			WD					DQB1*05:49			
DQB1*05:51	P	WD	WD			WD					DQB1*05:51			
DQB1*05:52	P	WD	WD			WD					DQB1*05:52			
DQB1*05:58	P	WD	WD	WD							DQB1*05:58			
DQB1*05:60	P	WD	WD			WD					DQB1*05:60			
DQB1*05:67	P	WD	WD		WD						DQB1*05:67			
DQB1*05:69	P	WD	WD			WD					DQB1*05:69			
DQB1*05:79	P	WD	WD			WD					DQB1*05:79			
DQB1*05:80	P	WD	WD								DQB1*05:80			
DQB1*05:88	P	WD	WD	WD							DQB1*05:88			
DQB1*06:01	P	C	C	C	C	C	C	C	C	C	DQB1*06:01	C	C	C
DQB1*06:02	P	C	C	C	C	C	C	C	C	C	DQB1*06:02	C	C	C
DQB1*06:03	P	C	C	C	C	C	C	C	C	C	DQB1*06:03	C	C	C
DQB1*06:04	P	C	C	C	C	C	C	C	C	C	DQB1*06:04	C	C	C
DQB1*06:05	P	WD	WD			WD					DQB1*06:05	WD	C	WD
DQB1*06:06	P	o	o								DQB1*06:06		WD	
DQB1*06:07	P	I	I		WD	I	WD	WD		I	DQB1*06:07		C	WD
DQB1*06:08	P	C	C	C	WD	WD	WD	C	C	C	DQB1*06:08	C	WD	
DQB1*06:09	P	C	C	C	C	C	C	C	C	C	DQB1*06:09	C	C	C
DQB1*06:10	P	C	I		C	WD				I	DQB1*06:10	WD		C
DQB1*06:11	P	C	I	C	I	I	WD	C	C	I	DQB1*06:11	WD	WD	WD
DQB1*06:12	P	WD	WD		WD	WD					DQB1*06:12			
DQB1*06:13	P	I	I	WD		I				WD	DQB1*06:13		WD	
DQB1*06:14	P	I	I	WD	WD	I	WD	I		I	DQB1*06:14		WD	

Supplementary Table 3f. Comparison of HLA-DQB1 P group two-field assignments among CIWD catalogs [a]

		3.0.0 CIWD									2.0.0 CWD		EFI CWD	China CWD
		Category by Population [b]												
HLA-DQB1 IPD-HLA/IMGT 3.31.0	Nomenclature Designation [a]	Highest Frequency	Total	AFA	API	EURO	MENA	HIS	NAM	UNK	HLA-DQB1 IPD-IMGT/HLA 3.9.0	Category	Category	Category
DQB1*06:15	P	WD	WD			WD					DQB1*06:15		WD	
DQB1*06:16	P	I	I	WD		I		I		I	DQB1*06:16		WD	
DQB1*06:18	P	WD	WD	WD		WD					DQB1*06:18			
DQB1*06:19	P	o	o								DQB1*06:19		WD	
DQB1*06:20	P	WD	WD	WD		WD					DQB1*06:20		WD	
DQB1*06:21	P	I	I			I					DQB1*06:21		WD	
DQB1*06:22	P	WD	WD	WD		WD				WD	DQB1*06:22			
DQB1*06:23	P	WD	WD			WD					DQB1*06:23		WD	
DQB1*06:26N	N	I	WD	WD		WD				I	DQB1*06:26N			
DQB1*06:27	P	WD	WD			WD				WD	DQB1*06:27		WD	
DQB1*06:28	P	WD	WD			WD					DQB1*06:28		WD	
DQB1*06:29	P	WD	WD			WD					DQB1*06:29		WD	
DQB1*06:31	P	WD	WD			WD	WD				DQB1*06:31		WD	
DQB1*06:32	P	WD	WD			WD					DQB1*06:32		WD	
DQB1*06:33	P	I	I	WD		I				I	DQB1*06:33			
DQB1*06:40	P	WD	WD			WD					DQB1*06:40			
DQB1*06:46	P	WD	WD	WD		WD					DQB1*06:46		WD	
DQB1*06:48	P	C	I		C	WD				WD	DQB1*06:48		WD	
DQB1*06:49	P	C	WD	C						WD	DQB1*06:49			
DQB1*06:50	P	WD	WD	WD							DQB1*06:50			
DQB1*06:53	P	WD	WD		WD	WD					DQB1*06:53		WD	
DQB1*06:55	P	I	WD		I	WD					DQB1*06:55			
DQB1*06:57	P	I	WD		I						DQB1*06:57			
DQB1*06:58	P	WD	WD			WD					DQB1*06:58		WD	
DQB1*06:62	P	WD	WD			WD					DQB1*06:62		WD	
DQB1*06:68	P	WD	WD			WD					DQB1*06:68			
DQB1*06:69	P	WD	WD			WD					DQB1*06:69			

Supplementary Table 3f. Comparison of HLA-DQB1 P group two-field assignments among CIWD catalogs [a]

		3.0.0 CIWD									2.0.0 CWD		EFI CWD	China CWD
		Category by Population [b]												
HLA-DQB1 IPD-HLA/IMGT 3.31.0	Nomenclature Designation [a]	Highest Frequency	Total	AFA	API	EURO	MENA	HIS	NAM	UNK	HLA-DQB1 IPD-IMGT/HLA 3.9.0	Category	Category	Category
DQB1*06:73	P	WD	WD			WD					DQB1*06:73		WD	
DQB1*06:74	P	I	WD		I					I	DQB1*06:74			
DQB1*06:75N	N	WD	WD			WD					DQB1*06:75N		WD	
DQB1*06:77N	N	I	WD		I						DQB1*06:77N			
DQB1*06:78	P	WD	WD			WD					DQB1*06:78			
DQB1*06:79	P	WD	WD			WD					DQB1*06:79		WD	
DQB1*06:81	P	I	WD			WD				I	DQB1*06:81		WD	
DQB1*06:82	P	WD	WD			WD					DQB1*06:82			
DQB1*06:85	P	WD	WD	WD							DQB1*06:85			
DQB1*06:90	P	WD	WD			WD					DQB1*06:90		WD	
DQB1*06:91	P	WD	WD		WD						DQB1*06:91			
DQB1*06:92	P	I	WD		I						DQB1*06:92			
DQB1*06:93	P	WD	WD								DQB1*06:93			
DQB1*06:107	P	o	o								DQB1*06:107			WD
DQB1*06:118	P	WD	WD			WD					DQB1*06:118			
DQB1*06:122	P	WD	WD			WD					DQB1*06:122			
DQB1*06:125	P	WD	WD			WD					DQB1*06:125		WD	
DQB1*06:126	P	WD	WD				WD				DQB1*06:126			
DQB1*06:135	P	WD	WD		WD						DQB1*06:135			
DQB1*06:136	P	WD	WD			WD					DQB1*06:136			
DQB1*06:140	P	I	WD		I						DQB1*06:140			
DQB1*06:142	P	WD	WD		WD						DQB1*06:142			
DQB1*06:144N	N	WD	WD		WD						DQB1*06:144N			
DQB1*06:153	P	WD	WD		WD		WD				DQB1*06:153			
DQB1*06:155	P	WD	WD			WD					DQB1*06:155			
DQB1*06:184	P	WD	WD		WD						DQB1*06:184			

Supplementary Table 3f. Comparison of HLA-DQB1 P group two-field assignments among CIWD catalogs [a]

		3.0.0 CIWD									2.0.0 CWD		EFI CWD	China CWD
		Category by Population [b]												
HLA-DQB1 IPD-HLA/IMGT 3.31.0	Nomenclature Designation [a]	Highest Frequency	Total	AFA	API	EURO	MENA	HIS	NAM	UNK	HLA-DQB1 IPD-IMGT/HLA 3.9.0	Category	Category	Category

C, common; I, intermediate; WD, well-documented

[a] Alleles observed at least five times in any population group in the current dataset are included in this table. The exception are alleles that were observed in previous catalogs but that are not present or are observed less than five times. Note that this two-field designation is the equivalent of a "P" assignment so that alleles within a P group that encode alleles that differ outside of the antigen recognition domain are not listed separately (e.g., DQB1*02:02 is within the DQB1*02:01P assignment and is not listed in this table even though it was categorized as common in 2.0.0 CWD). The table does not list all alleles from IPD-IMGT/HLA version 3.31.0.

[b] Population groups include: AFA (African/African American), API (Asian/Pacific Islander), EURO (European/European descent), MENA (Middle East/North Coast of Africa), HIS (Hispanic), NAM (Native American) and UNK (unknown, multiple ancestries or other). Total is the overall population i.e., all groups combined. Highest frequency is the highest CIWD designation among all the individual groups.

[c] Not-CIWD, o.

Supplementary Table 3g. Comparison of HLA-DPB1 P group two-field assignments among CIWD catalogs [a]

		3.0.0 CIWD									2.0.0 CWD		EFI CWD	China CWD
		Category by Population [b]												
HLA-DPB1 IPD-HLA/IMGT 3.31.0	Nomenclature Designation [a]	Highest Frequency	Total	AFA	API	EURO	MENA	HIS	NAM	UNK	HLA-DPB1 IPD-IMGT/HLA 3.9.0	Category	Category	Category
DPB1*01:01	P	C	C	C	C	C	C	C	C	C	DPB1*01:01	C	C	Not available
DPB1*02:01	P	C	C	C	C	C	C	C	C	C	DPB1*02:01	C	C	
DPB1*02:02	P	C	C	C	C	C	C	C	C	C	DPB1*02:02	C	C	
DPB1*03:01	P	C	C	C	C	C	C	C	C	C	DPB1*03:01	C	C	
DPB1*04:01	P	C	C	C	C	C	C	C	C	C	DPB1*04:01	C	C	
DPB1*04:02	P	C	C	C	C	C	C	C	C	C	DPB1*04:02	C	C	
DPB1*05:01	P	C	C	C	C	C	C	C	C	C	DPB1*05:01	C	C	
DPB1*06:01	P	C	C	C	C	C	C	C	C	C	DPB1*06:01	C	C	
DPB1*08:01	P	WD	WD			WD					DPB1*08:01		WD	
DPB1*09:01	P	C	C	C	C	C	C	C	C	C	DPB1*09:01	C	C	
DPB1*10:01	P	C	C	C	C	C	C	C	C	C	DPB1*10:01	C	C	
DPB1*11:01	P	C	C	C	C	C	C	C	C	C	DPB1*11:01	C	C	
DPB1*13:01	P	C	C	C	C	C	C	C	C	C	DPB1*13:01	C	C	
DPB1*14:01	P	C	C	C	C	C	C	C	C	C	DPB1*14:01	C	C	
DPB1*15:01	P	C	C	C	C	C	C	C	C	C	DPB1*15:01	C	C	
DPB1*16:01	P	C	C	C	C	C	C	C	C	C	DPB1*16:01	C	WD	
DPB1*17:01	P	C	C	C	C	C	C	C	C	C	DPB1*17:01	C	C	
DPB1*18:01	P	C	C	C	I	I	C	C	C	C	DPB1*18:01	C	C	
DPB1*19:01	P	C	C	C	C	C	C	C	C	C	DPB1*19:01	C	C	
DPB1*20:01	P	C	C	C	C	C	C	C	C	C	DPB1*20:01	C	WD	
DPB1*21:01	P	C	C	WD	C	I	WD	I	C	C	DPB1*21:01	C	WD	
DPB1*22:01	P	I	WD		I	WD					DPB1*22:01	WD	WD	
DPB1*23:01	P	C	C	C	C	C	C	C	C	C	DPB1*23:01	C	C	
DPB1*24:01	P	I	I			I	WD	I		I	DPB1*24:01		WD	
DPB1*25:01	P	I	I		I	I		I		I	DPB1*25:01		WD	
DPB1*26:01	P	C	C	C	C	C	C	C	C	C	DPB1*26:01	C	C	
DPB1*27:01	P	C	I	C		WD		C	C	C	DPB1*27:01	C	WD	
DPB1*28:01	P	C	C	C	C	I	C	I	C	C	DPB1*28:01	C	WD	

Supplementary Table 3g. Comparison of HLA-DPB1 P group two-field assignments among CIWD catalogs [a]

		3.0.0 CIWD									2.0.0 CWD		EFI CWD	China CWD
		Category by Population [b]												
HLA-DPB1 IPD-HLA/IMGT 3.31.0	Nomenclature Designation [a]	Highest Frequency	Total	AFA	API	EURO	MENA	HIS	NAM	UNK	HLA-DPB1 IPD-IMGT/HLA 3.9.0	Category	Category	Category
DPB1*29:01	P	C	C	C	C	I	WD	C	C	C	DPB1*29:01	C	WD	
DPB1*30:01	P	C	C	C	I	C	C	C	C	C	DPB1*30:01	C	C	
DPB1*31:01	P	C	I		C	WD		I		C	DPB1*31:01	C	WD	
DPB1*32:01	P	o [c]	o								DPB1*32:01		WD	
DPB1*33:01	P	I	I		I	I	WD	I		I	DPB1*33:01	WD	WD	
DPB1*34:01	P	C	C	C	C	C	C	C	C	C	DPB1*34:01	C	C	
DPB1*35:01	P	C	C	C	WD	C	C	C	C	C	DPB1*35:01	C	WD	
DPB1*36:01	P	C	I		C	I	C			I	DPB1*36:01	WD		
DPB1*37:01	P	C	WD			WD	C				DPB1*37:01			
DPB1*38:01	P	C	I		C	WD				I	DPB1*38:01	WD		
DPB1*39:01	P	C	C	C	I	I	WD	C	C	C	DPB1*39:01	C		
DPB1*40:01	P	C	C	C	WD	I	WD	C	C	C	DPB1*40:01	WD	WD	
DPB1*41:01	P	C	I		C	I	C	I		C	DPB1*41:01		C	
DPB1*45:01	P	C	C	WD	C	C	C	C	C	C	DPB1*45:01	C	C	
DPB1*46:01	P	C	C	C	C	I	C	I		I	DPB1*46:01	C	C	
DPB1*47:01	P	C	I	WD	C	I	C			I	DPB1*47:01	WD	C	
DPB1*48:01	P	C	I		C	WD	WD	I		I	DPB1*48:01		WD	
DPB1*49:01	P	C	I	C	WD	WD	WD	C	C	I	DPB1*49:01	WD		
DPB1*50:01	P	C	I		WD	C	WD	I		C	DPB1*50:01		WD	
DPB1*51:01	P	C	C	WD	C	C	C	I	C	C	DPB1*51:01	C		
DPB1*52:01	P	I	I	WD	I	I				I	DPB1*52:01			
DPB1*53:01	P	C	WD	C				I	C	I	DPB1*53:01			
DPB1*54:01	P	WD	WD	WD		WD				WD	DPB1*54:01			
DPB1*55:01	P	C	I	C	I	WD	WD	C		I	DPB1*55:01	C		
DPB1*56:01	P	WD	WD			WD					DPB1*56:01			
DPB1*57:01	P	I	I	WD	I	I	WD	I		I	DPB1*57:01	WD		
DPB1*59:01	P	C	I		WD	C		I		I	DPB1*59:01	C		
DPB1*60:01	P	WD	WD	WD							DPB1*60:01			

Supplementary Table 3g. Comparison of HLA-DPB1 P group two-field assignments among CIWD catalogs [a]

		3.0.0 CIWD									2.0.0 CWD		EFI CWD	China CWD
		Category by Population [b]												
HLA-DPB1 IPD-HLA/IMGT 3.31.0	Nomenclature Designation [a]	Highest Frequency	Total	AFA	API	EURO	MENA	HIS	NAM	UNK	HLA-DPB1 IPD-IMGT/HLA 3.9.0	Category	Category	Category
DPB1*61:01N	N	C	WD	C				I		I	DPB1*61:01N			
DPB1*63:01	P	C	I	C	WD	I	C	C	C	C	DPB1*63:01	C	C	
DPB1*64:01N	N	I	WD			WD				I	DPB1*64:01N			
DPB1*65:01	P	WD	WD		WD	WD		WD			DPB1*65:01		WD	
DPB1*66:01	P	WD	WD			WD				WD	DPB1*66:01		WD	
DPB1*67:01	P	C	WD			WD	C				DPB1*67:01		WD	
DPB1*68:01	P	WD	WD			WD					DPB1*68:01		WD	
DPB1*69:01	P	WD	WD	WD		WD		WD			DPB1*69:01	WD		
DPB1*70:01	P	I	WD		I	WD					DPB1*70:01		WD	
DPB1*71:01	P	C	I	C	C	I	C	I		I	DPB1*71:01		WD	
DPB1*72:01	P	I	WD		I	WD	WD			I	DPB1*72:01	WD	WD	
DPB1*74:01	P	WD	WD	WD						WD	DPB1*74:01			
DPB1*75:01	P	WD	WD			WD	WD				DPB1*75:01		WD	
DPB1*76:01	P	WD	WD			WD					DPB1*76:01		WD	
DPB1*77:01	P	WD	WD			WD				WD	DPB1*77:01		WD	
DPB1*78:01	P	I	I		I	I	WD	I		I	DPB1*78:01	WD	WD	
DPB1*79:01	P	I	WD			WD		I		WD	DPB1*79:01		WD	
DPB1*80:01	P	C	I	C	I	I	WD	C	C	I	DPB1*80:01		WD	
DPB1*81:01	P	C	I	WD	I	I	C	I		I	DPB1*81:01	C	WD	
DPB1*82:01	P	I	WD					I			DPB1*82:01		WD	
DPB1*83:01	P	I	WD		I	WD					DPB1*83:01		WD	
DPB1*85:01	P	C	C	C	I	I	WD	C	C	C	DPB1*85:01	C		
DPB1*86:01	P	WD	WD	WD							DPB1*86:01			
DPB1*87:01	P	WD	WD			WD				WD	DPB1*87:01			
DPB1*88:01	P	C	I		WD	I	C	WD		I	DPB1*88:01			
DPB1*89:01	P	WD	WD			WD					DPB1*89:01			
DPB1*90:01	P	WD	WD			WD				WD	DPB1*90:01			
DPB1*91:01	P	C	I		C	WD	C			I	DPB1*91:01			

Supplementary Table 3g. Comparison of HLA-DPB1 P group two-field assignments among CIWD catalogs [a]

		3.0.0 CIWD									2.0.0 CWD		EFI CWD	China CWD
		Category by Population [b]												
HLA-DPB1 IPD-HLA/IMGT 3.31.0	Nomenclature Designation [a]	Highest Frequency	Total	AFA	API	EURO	MENA	HIS	NAM	UNK	HLA-DPB1 IPD-IMGT/HLA 3.9.0	Category	Category	Category
DPB1*92:01	P	I	WD		I	I					DPB1*92:01			
DPB1*93:01	P	C	I		C	WD		I		I	DPB1*93:01			
DPB1*95:01	P	WD	WD	WD							DPB1*95:01			
DPB1*96:01	P	WD	WD								DPB1*96:01			
DPB1*98:01	P	WD	WD	WD		WD					DPB1*98:01			
DPB1*99:01	P	I	WD			WD	WD	I		WD	DPB1*99:01			
DPB1*100:01	P	I	I		I	WD		I		I	DPB1*100:01			
DPB1*102:01	P	WD	WD			WD					DPB1*102:01			
DPB1*103:01	P	WD	WD	WD		WD					DPB1*103:01			
DPB1*108:01	P	I	WD		WD	WD		I			DPB1*108:01		WD	
DPB1*109:01	P	I	I			I				WD	DPB1*109:01		WD	
DPB1*110:01	P	WD	WD			WD					DPB1*110:01		WD	
DPB1*112:01	P	WD	WD			WD					DPB1*112:01			
DPB1*113:01	P	I	WD		I	WD	WD				DPB1*113:01		WD	
DPB1*115:01	P	I	I			I				WD	DPB1*115:01		WD	
DPB1*116:01	P	WD	WD			WD				WD	DPB1*116:01		WD	
DPB1*120:01N	N	WD	WD			WD					DPB1*120:01N			
DPB1*121:01	P	WD	WD			WD					DPB1*121:01		WD	
DPB1*122:01	P	WD	WD	WD		WD					DPB1*122:01		WD	
DPB1*123:01	P	WD	WD			WD					DPB1*123:01		WD	
DPB1*125:01	P	I	WD		I	WD					DPB1*125:01			
DPB1*127:01	P	I	I			I					DPB1*127:01		WD	
DPB1*128:01	P	I	I			I		I		I	DPB1*128:01		WD	
DPB1*129:01	P	WD	WD			WD					DPB1*129:01		WD	
DPB1*130:01	P	I	I			I				I	DPB1*130:01		C	
DPB1*132:01	P	I	WD		I	WD	WD	I		WD	DPB1*132:01			
DPB1*134:01	P	WD	WD			WD					DPB1*134:01			
DPB1*136:01	P	C	WD			WD	C				DPB1*136:01			

Supplementary Table 3g. Comparison of HLA-DPB1 P group two-field assignments among CIWD catalogs [a]

		3.0.0 CIWD									2.0.0 CWD		EFI CWD	China CWD
		Category by Population [b]												
HLA-DPB1 IPD-HLA/IMGT 3.31.0	Nomenclature Designation [a]	Highest Frequency	Total	AFA	API	EURO	MENA	HIS	NAM	UNK	HLA-DPB1 IPD-IMGT/HLA 3.9.0	Category	Category	Category
DPB1*137:01	P	WD	WD			WD				WD	DPB1*137:01			
DPB1*139:01	P	I	WD		I						DPB1*139:01			
DPB1*142:01	P	WD	WD		WD						DPB1*142:01			
DPB1*143:01	P	WD	WD	WD							DPB1*143:01			
DPB1*144:01	P	WD	WD			WD					DPB1*144:01			
DPB1*145:01	P	I	WD		WD	WD				I	DPB1*145:01			
DPB1*146:01	P	WD	WD			WD					DPB1*146:01			
DPB1*147:01	P	WD	WD					WD			DPB1*147:01			
DPB1*149:01	P	I	WD			WD				I	DPB1*149:01			
DPB1*150:01	P	C	I		C	WD					DPB1*150:01			
DPB1*151:01	P	WD	WD			WD					DPB1*151:01			
DPB1*152:01	P	WD	WD			WD					DPB1*152:01			
DPB1*153:01	P	I	WD					I		WD	DPB1*153:01			
DPB1*154:01N	N	WD	WD			WD					DPB1*154:01N			
DPB1*155:01	P	WD	WD	WD		WD					DPB1*155:01			
DPB1*156:01	P	I	WD			WD		I			DPB1*156:01			
DPB1*157:01	P	WD	WD			WD					DPB1*157:01			
DPB1*158:01	P	WD	WD			WD					DPB1*158:01			
DPB1*161:01N	N	WD	WD			WD					DPB1*161:01N			
DPB1*163:01	P	WD	WD			WD					DPB1*163:01			
DPB1*164:01	P	WD	WD			WD					DPB1*164:01			
DPB1*165:01	P	WD	WD			WD					DPB1*165:01			
DPB1*166:01	P	I	WD		I						DPB1*166:01			
DPB1*170:01	P	WD	WD			WD					DPB1*170:01			
DPB1*172:01	P	WD	WD			WD		WD			DPB1*172:01			
DPB1*173:01	P	I	WD		I	WD	WD				DPB1*173:01			
DPB1*177:01	P	WD	WD			WD					DPB1*177:01			
DPB1*178:01	P	WD	WD			WD					DPB1*178:01			

Supplementary Table 3g. Comparison of HLA-DPB1 P group two-field assignments among CIWD catalogs [a]

		3.0.0 CIWD									2.0.0 CWD		EFI CWD	China CWD
		Category by Population [b]												
HLA-DPB1 IPD-HLA/IMGT 3.31.0	Nomenclature Designation [a]	Highest Frequency	Total	AFA	API	EURO	MENA	HIS	NAM	UNK	HLA-DPB1 IPD-IMGT/HLA 3.9.0	Category	Category	Category
DPB1*179:01	P	WD	WD			WD					DPB1*179:01			
DPB1*181:01	P	WD	WD				WD				DPB1*181:01			
DPB1*182:01	P	WD	WD		WD	WD					DPB1*182:01			
DPB1*184:01	P	I	WD			WD				I	DPB1*184:01			
DPB1*185:01	P	I	WD			WD				I	DPB1*185:01			
DPB1*186:01	P	WD	WD		WD						DPB1*186:01			
DPB1*187:01	P	WD	WD			WD					DPB1*187:01			
DPB1*188:01	P	WD	WD			WD					DPB1*188:01			
DPB1*189:01	P	WD	WD			WD	WD				DPB1*189:01			
DPB1*190:01	P	I	I			I					DPB1*190:01			
DPB1*191:01	P	I	I			I					DPB1*191:01			
DPB1*192:01	P	I	WD		I						DPB1*192:01			
DPB1*199:01	P	WD	WD			WD					DPB1*199:01			
DPB1*200:01	P	WD	WD			WD					DPB1*200:01			
DPB1*201:01	P	WD	WD			WD	WD				DPB1*201:01			
DPB1*203:01	P	I	WD					I			DPB1*203:01			
DPB1*206:01	P	WD	WD			WD	WD				DPB1*206:01			
DPB1*207:01	P	WD	WD								DPB1*207:01			
DPB1*208:01	P	WD	WD			WD					DPB1*208:01			
DPB1*209:01	P	WD	WD			WD					DPB1*209:01			
DPB1*210:01	P	I	WD					I			DPB1*210:01			
DPB1*211:01	P	WD	WD			WD					DPB1*211:01			
DPB1*212:01	P	WD	WD								DPB1*212:01			
DPB1*214:01	P	WD	WD			WD					DPB1*214:01			
DPB1*215:01	P	I	WD		I	WD				WD	DPB1*215:01			
DPB1*218:01N	N	WD	WD			WD					DPB1*218:01N			
DPB1*220:01	P	WD	WD			WD					DPB1*220:01			
DPB1*221:01	P	WD	WD			WD					DPB1*221:01			

Supplementary Table 3g. Comparison of HLA-DPB1 P group two-field assignments among CIWD catalogs [a]

		3.0.0 CIWD									2.0.0 CWD		EFI CWD	China CWD
		Category by Population [b]												
HLA-DPB1 IPD-HLA/IMGT 3.31.0	Nomenclature Designation [a]	Highest Frequency	Total	AFA	API	EURO	MENA	HIS	NAM	UNK	HLA-DPB1 IPD-IMGT/HLA 3.9.0	Category	Category	Category
DPB1*222:01	P	I	WD			WD				I	DPB1*222:01			
DPB1*224:01	P	WD	WD			WD					DPB1*224:01			
DPB1*225:01	P	WD	WD			WD					DPB1*225:01			
DPB1*227:01	P	WD	WD			WD					DPB1*227:01			
DPB1*228:01	P	WD	WD			WD					DPB1*228:01			
DPB1*233:01	P	WD	WD			WD		WD			DPB1*233:01			
DPB1*235:01	P	WD	WD			WD					DPB1*235:01			
DPB1*236:01	P	WD	WD		WD	WD					DPB1*236:01			
DPB1*238:01	P	I	WD			WD				I	DPB1*238:01			
DPB1*240:01	P	I	WD					I			DPB1*240:01			
DPB1*242:01	P	I	WD		I						DPB1*242:01			
DPB1*244:01	P	WD	WD			WD	WD				DPB1*244:01			
DPB1*245:01	P	WD	WD			WD					DPB1*245:01			
DPB1*246:01	P	WD	WD			WD					DPB1*246:01			
DPB1*249:01	P	WD	WD	WD							DPB1*249:01			
DPB1*256:01	P	WD	WD							WD	DPB1*256:01			
DPB1*257:01	P	WD	WD			WD					DPB1*257:01			
DPB1*258:01	P	WD	WD								DPB1*258:01			
DPB1*259:01	P	WD	WD			WD					DPB1*259:01			
DPB1*260:01	P	WD	WD			WD					DPB1*260:01			
DPB1*264:01	P	WD	WD			WD					DPB1*264:01			
DPB1*265:01	P	WD	WD			WD					DPB1*265:01			
DPB1*266:01	P	WD	WD			WD					DPB1*266:01			
DPB1*269:01	P	I	WD		I						DPB1*269:01			
DPB1*270:01	P	WD	WD			WD					DPB1*270:01			
DPB1*271:01	P	I	WD			WD		I			DPB1*271:01			
DPB1*274:01	P	WD	WD			WD					DPB1*274:01			
DPB1*277:01	P	WD	WD			WD					DPB1*277:01			

Supplementary Table 3g. Comparison of HLA-DPB1 P group two-field assignments among CIWD catalogs [a]

		3.0.0 CIWD									2.0.0 CWD		EFI CWD	China CWD
		Category by Population [b]												
HLA-DPB1 IPD-HLA/IMGT 3.31.0	Nomenclature Designation [a]	Highest Frequency	Total	AFA	API	EURO	MENA	HIS	NAM	UNK	HLA-DPB1 IPD-IMGT/HLA 3.9.0	Category	Category	Category
DPB1*281:01	P	C	WD				C				DPB1*281:01			
DPB1*282:01	P	WD	WD			WD					DPB1*282:01			
DPB1*283:01	P	WD	WD			WD					DPB1*283:01			
DPB1*284:01	P	WD	WD			WD					DPB1*284:01			
DPB1*285:01	P	WD	WD			WD					DPB1*285:01			
DPB1*288:01	P	WD	WD								DPB1*288:01			
DPB1*290:01	P	WD	WD	WD		WD					DPB1*290:01			
DPB1*291:01	P	WD	WD			WD					DPB1*291:01			
DPB1*294:01	P	WD	WD			WD					DPB1*294:01			
DPB1*295:01	P	WD	WD			WD					DPB1*295:01			
DPB1*300:01	P	I	WD		I						DPB1*300:01			
DPB1*301:01	P	WD	WD			WD					DPB1*301:01			
DPB1*303:01	P	WD	WD								DPB1*303:01			
DPB1*305:01	P	WD	WD								DPB1*305:01			
DPB1*309:01	P	WD	WD			WD					DPB1*309:01			
DPB1*312:01	P	WD	WD			WD					DPB1*312:01			
DPB1*313:01	P	WD	WD			WD					DPB1*313:01			
DPB1*314:01	P	WD	WD								DPB1*314:01			
DPB1*318:01	P	WD	WD			WD					DPB1*318:01			
DPB1*319:01	P	WD	WD								DPB1*319:01			
DPB1*320:01	P	WD	WD			WD					DPB1*320:01			
DPB1*321:01	P	WD	WD	WD							DPB1*321:01			
DPB1*329:01	P	WD	WD								DPB1*329:01			
DPB1*333:01	P	WD	WD					WD			DPB1*333:01			
DPB1*339:01	P	WD	WD			WD					DPB1*339:01			
DPB1*340:01	P	WD	WD			WD					DPB1*340:01			
DPB1*345:01	P	WD	WD			WD					DPB1*345:01			
DPB1*348:01	P	WD	WD			WD					DPB1*348:01			

Supplementary Table 3g. Comparison of HLA-DPB1 P group two-field assignments among CIWD catalogs [a]

		3.0.0 CIWD									2.0.0 CWD		EFI CWD	China CWD
		Category by Population [b]												
HLA-DPB1 IPD-HLA/IMGT 3.31.0	Nomenclature Designation [a]	Highest Frequency	Total	AFA	API	EURO	MENA	HIS	NAM	UNK	HLA-DPB1 IPD-IMGT/HLA 3.9.0	Category	Category	Category
DPB1*355:01	P	WD	WD			WD					DPB1*355:01			
DPB1*356:01	P	WD	WD		WD						DPB1*356:01			
DPB1*357:01N	N	WD	WD		WD						DPB1*357:01N			
DPB1*358:01	P	WD	WD			WD					DPB1*358:01			
DPB1*363:01	P	WD	WD		WD						DPB1*363:01			
DPB1*370:01	P	WD	WD								DPB1*370:01			
DPB1*374:01	P	WD	WD								DPB1*374:01			
DPB1*377:01	P	WD	WD			WD					DPB1*377:01			
DPB1*391:01	P	WD	WD		WD						DPB1*391:01			
DPB1*393:01	P	WD	WD			WD					DPB1*393:01			
DPB1*394:01	P	WD	WD		WD						DPB1*394:01			
DPB1*396:01	P	WD	WD								DPB1*396:01			
DPB1*397:01	P	WD	WD			WD					DPB1*397:01			
DPB1*398:01	P	WD	WD			WD					DPB1*398:01			
DPB1*412:01	P	WD	WD			WD					DPB1*412:01			
DPB1*413:01	P	WD	WD			WD					DPB1*413:01			
DPB1*422:01	P	WD	WD								DPB1*422:01			
DPB1*424:01	P	WD	WD								DPB1*424:01			
DPB1*433:01	P	WD	WD							WD	DPB1*433:01			
DPB1*441:01	P	WD	WD	WD							DPB1*441:01			
DPB1*445:01	P	I	WD		I						DPB1*445:01			
DPB1*448:01	P	WD	WD		WD						DPB1*448:01			
DPB1*454:01	P	WD	WD		WD						DPB1*454:01			
DPB1*474:01	P	WD	WD		WD						DPB1*474:01			
DPB1*482:01	P	WD	WD								DPB1*482:01			
DPB1*486:01	P	WD	WD		WD						DPB1*486:01			
DPB1*495:01	P	WD	WD	WD							DPB1*495:01			
DPB1*501:01	P	WD	WD			WD	WD				DPB1*501:01			

Supplementary Table 3g. Comparison of HLA-DPB1 P group two-field assignments among CIWD catalogs [a]

		3.0.0 CIWD									2.0.0 CWD		EFI CWD	China CWD
		Category by Population [b]												
**HLA-DPB1 IPD-HLA/IMGT 3.31.0**	**Nomenclature Designation [a]**	**Highest Frequency**	**Total**	**AFA**	**API**	**EURO**	**MENA**	**HIS**	**NAM**	**UNK**	**HLA-DPB1 IPD-IMGT/HLA 3.9.0**	**Category**	**Category**	**Category**
DPB1*503:01	P	WD	WD								DPB1*503:01			
DPB1*514:01	P	WD	WD			WD					DPB1*514:01			
DPB1*520:01	P	WD	WD		WD						DPB1*520:01			
DPB1*521:01	P	WD	WD								DPB1*521:01			
DPB1*526:01	P	I	WD		I						DPB1*526:01			
DPB1*559:01	P	WD	WD								DPB1*559:01			
DPB1*570:01N	N	WD	WD		WD						DPB1*570:01N			

C, common; I, intermediate; WD, well-documented

[a] Alleles observed at least five times in any population group in the current dataset are included in this table. The exception are alleles that were observed in previous catalogs but that are not present or are observed less than five times. Note that this two-field designation is the equivalent of a "P" assignment so that alleles within a P group that encodes a protein that differs outside of the antigen recognition domain are not listed separately (e.g., DPB1*105:01 is within the DPB1*04:02P assignment and is not listed in this table even though it was categorized as common in 2.0.0 CWD). The table does not list all alleles from IPD-IMGT/HLA version 3.31.0.

[b] Population groups include: AFA (African/African American), API (Asian/Pacific Islander), EURO (European/European descent), MENA (Middle East/North Coast of Africa), HIS (Hispanic), NAM (Native American) and UNK (unknown, multiple ancestries or other). Total is the overall population i.e., all groups combined. Highest frequency is the highest CIWD designation among all the individual groups.

[c] Not-CIWD, o.
