# Supplementary material for: Common, intermediate and well‐documented HLA alleles in world populations: CIWD version 3.0.0
Source: HLA. 2020 Jan 31;95(6):516–31. doi: 10.1111/tan.13811 (PMC7317522; doi:10.1111/tan.13811)
Supplement: Supplementary file 4 — Table S4 Overall comparison of HLA G group assignments among CIWD catalogs [file TAN-95-516-s004.docx]

**Supplementary Table 4. Overall Comparison of HLA G level assignments^a^ between CIWD versions**

|  | **2.0.0 CWD** | | |
| --- | --- | --- | --- |
|  | **HLA-A** | | |
| **3.0.0 CIWD^b^** | **Common n=40** | **WD n=9** | **Not-CWD ^c^ n=1** |
| **Common total n=45** | 40 | 5 | 0 |
| **Intermediate n=2** | 0 | 2 | 0 |
| **Well-documented n=3** | 0 | 2 | 1 |
| **Not-CIWD ^c^ n=0** | 0 | 0 | 0 |

|  | **2.0.0 CWD** | | |
| --- | --- | --- | --- |
|  | **HLA-B** | | |
| **3.0.0 CIWD** | **Common n=70** | **WD n=14** | **Not-CWD n=3** |
| **Common total n=81** | 70 | 10 | 1 |
| **Intermediate n=3** | 0 | 3 | 0 |
| **Well-documented n=2** | 0 | 0 | 2 |
| **Not-CIWD n=1** | 0 | 1 | 0 |

|  | **2.0.0 CWD** | | |
| --- | --- | --- | --- |
|  | **HLA-C** | | |
| **3.0.0 CIWD** | **Common n=31** | **WD n=4** | **Not-CWD n=2** |
| **Common total n=33** | 31 | 2 | 0 |
| **Intermediate n=3** | 0 | 2 | 1 |
| **Well-documented n=1** | 0 | 0 | 1 |
| **Not-CIWD n=0** | 0 | 0 | 0 |

|  | **2.0.0 CWD** | | |
| --- | --- | --- | --- |
|  | **HLA-DRB1** | | |
| **3.0.0 CIWD** | **Common n=31** | **WD n=1** | **Not-CWD n=0** |
| **Common total n=31** | 31 | 0 | 0 |
| **Intermediate n=1** | 0 | 1 | 0 |
| **Well-documented n=0** | 0 | 0 | 0 |
| **Not-CIWD n=0** | 0 | 0 | 0 |

|  | **2.0.0 CWD** | | |
| --- | --- | --- | --- |
|  | **HLA-DRB3/4/5** | | |
| **3.0.0 CIWD** | **Common n=8** | **WD n=0** | **Not-CWD n=0** |
| **Common total n=ND** | insufficient data | | |
| **Intermediate n=ND** | insufficient data | | |
| **Well-documented n=8** | 8 | 0 | 0 |

|  | **2.0.0 CWD** | | |
| --- | --- | --- | --- |
|  | **HLA-DQB1** | | |
| **3.0.0 CIWD** | **Common n=17** | **WD n=0** | **Not-CWD n=2** |
| **Common total n=17** | 17 | 0 | 0 |
| **Intermediate n=0** | 0 | 0 | 0 |
| **Well-documented n=2** | 0 | 0 | 2 |
| **Not-CIWD n=0** | 0 | 0 | 0 |

|  | **2.0.0 CWD** | | |
| --- | --- | --- | --- |
|  | **HLA-DPB1** | | |
| **3.0.0 CIWD** | **Common n=26** | **WD n=7** | **Not-CWD n=3** |
| **Common total n=32** | 26 | 3 | 3 |
| **Intermediate n=3** | 0 | 3 | 0 |
| **Well-documented n=1** | 0 | 1 | 0 |
| **Not-CIWD n=0** | 0 | 0 | 0 |

C, common; I, intermediate; WD, well-documented; ND, not determined

a Based on the highest frequency for any population group. Version 2.0.0 CWD alleles not yet in G group are also included.

b Supplementary Table 5 lists the alleles and their frequency categories: HLA-A Supplementary Table 5a, HLA-B 5b, HLA-C 5c, HLA-DRB1 5d, HLA-DRB3/4/5 5e, HLA-DQB1 5f, HLA-DPB1 5g.

c Not-CIWD or not-CWD based only on alleles categorized as CWD or CIWD observed in any catalog

Reference

Mack SJ, Cano P, Hollenbach JA, He J, Hurley CK, Middleton D, Moraes ME, Pereira SE, Kempenich JH, Reed EF, Setterholm M, Smith AG, Tilanus MG, Torres M, Varney MD, Voorter CE, Fischer GF, Fleischhauer K, Goodridge D, Klitz W, Little AM, Maiers M, Marsh SG, Muller CR, Noreen H, Rozemuller EH, Sanchez-Mazas A, Senitzer D, Trachtenberg E, Fernandez-Vina M: Common and well-documented HLA alleles: 2012 update to the CWD catalogue. Tissue Antigens 81:194-203, 2013.
