# Supplementary material for: Common, intermediate and well‐documented HLA alleles in world populations: CIWD version 3.0.0
Source: HLA. 2020 Jan 31;95(6):516–31. doi: 10.1111/tan.13811 (PMC7317522; doi:10.1111/tan.13811)
Supplement: Supplementary file 5 — Table S5a‐g HLA G level frequencies between CIWD versions [file TAN-95-516-s005.pdf]

Supplementary Table 5a. HLA-A G level frequencies between CIWD versions [a]

| 3.0.0 CIWD Category by Population Group [b] | | | | | | | | | | 2.0.0 CWD | |
|---|---|---|---|---|---|---|---|---|---|---|---|
| HLA-A IPD-HLA/IMGT 3.31.0 | Highest Frequency | Total | AFA | API | EURO | MENA | HIS | NAM | UNK | HLA-A IPD-IMGT/HLA 3.9.0 | Category |
| A*01:01:01G | C | C | C | C | C | C | C | C | C | A*01:01:01G | C |
| A*01:03:01G | C | C | C | C | C | C | C | WD | C | A*01:03 [e] | C |
| A*01:09:01G | WD | WD | WD | | | | | | | A*01:09 | WD |
| A*02:01:01G | C | C | C | C | C | C | C | C | C | A*02:01:01G | C |
| A*02:02:01G | C | C | C | C | C | C | C | C | C | A*02:02 | C |
| A*02:03:01G | C | C | C | C | I | C | C | C | C | A*02:03:01G | C |
| A*02:04:01G | C | C | C | WD | I | WD | C | C | C | A*02:04 | C |
| A*02:05:01G | C | C | C | C | C | C | C | C | C | A*02:05:01G | C |
| A*02:06:01G | C | C | C | C | C | C | C | C | C | A*02:06:01G | C |
| A*02:07:01G | C | C | WD | C | C | C | C | C | C | A*02:07:01G | C |
| A*02:10:01G | C | I | | C | WD | | | | I | A*02:10 | C |
| A*02:11:01G | C | C | C | C | C | C | C | C | C | A*02:11:01G | C |
| A*02:16:01G | C | C | WD | C | WD | WD | | | I | A*02:16:01G | WD |
| A:02:17:01G [c] | C | C | C | I | C | C | C | C | C | A*02:17:01G | C |
| A*02:22:01G | C | C | C | I | C | C | C | C | C | A*02:22:01G | C |
| A*02:49:01G | WD | WD | | | WD | WD | | | WD | A*02:49 | WD |
| A*02:81:01G | WD | WD | | | WD | | | | | A*02:81 | o |
| A*03:01:01G | C | C | C | C | C | C | C | C | C | A*03:01:01G | C |
| A*03:02:01G | C | C | C | C | C | C | C | C | C | A*03:02:01 | C |
| A*11:01:01G | C | C | C | C | C | C | C | C | C | A*11:01:01G | C |
| A*11:02:01G | C | C | WD | C | I | WD | I | WD | C | A*11:02:01G | C |
| A*11:03:01G | C | C | WD | C | WD | WD | WD | C | I | A*11:03 | WD |
| A*11:05:01G | I | I | WD | | I | | I | | I | A*11:05 | WD |
| A*23:01:01G | C | C | C | C | C | C | C | C | C | A*23:01:01G | C |
| A*24:02:01G | C | C | C | C | C | C | C | C | C | A*24:02:01G | C |
| A*24:03:01G | C | C | C | C | C | C | C | C | C | A*24:03:01G | C |
| A*24:05:01G | C | I | WD | I | WD | | C | C | I | A*24:05:01G | WD |
| A*24:14:01G | C | I | WD | WD | WD | | C | C | I | A*24:14 | WD |

**Supplementary Table 5a. HLA-A G level frequencies between CIWD versions [a]**

| 3.0.0 CIWD Category by Population Group [b] | | | | | | | | | | 2.0.0 CWD | |
|---|---|---|---|---|---|---|---|---|---|---|---|
| **HLA-A IPD-HLA/IMGT 3.31.0** | **Highest Frequency** | **Total** | **AFA** | **API** | **EURO** | **MENA** | **HIS** | **NAM** | **UNK** | **HLA-A IPD-IMGT/HLA 3.9.0** | **Category** |
| A*24:20:01G | C | I | | C | WD | WD | | | C | A*24:20 | C |
| A*24:26:01G | I | I | WD | | WD | WD | I | | I | A*24:26 | WD |
| A*25:01:01G | C | C | C | C | C | C | C | C | C | A*25:01:01G | C |
| A*26:01:01G | C | C | C | C | C | C | C | C | C | A*26:01:01G | C |
| A*29:01:01G | C | C | C | C | C | C | C | C | C | A*29:01:01G | C |
| A*29:02:01G | C | C | C | C | C | C | C | C | C | A*29:02:01G | C |
| A*30:01:01G | C | C | C | C | C | C | C | C | C | A*30:01:01G | C |
| A*30:02:01G | C | C | C | C | C | C | C | C | C | A*30:02:01G | C |
| A*30:04:01G | C | C | C | C | C | C | C | C | C | A*30:04:01 | C |
| A*31:01:02G | C | C | C | C | C | C | C | C | C | A*31:01:02G | C |
| A*32:01:01G | C | C | C | C | C | C | C | C | C | A*32:01:01G | C |
| A*33:01:01G | C | C | C | C | C | C | C | C | C | A*33:01:01G | C |
| A*33:03:01G | C | C | C | C | C | C | C | C | C | A*33:03:01G | C |
| A*34:01:01 [d] | C | C | WD | C | I | WD | C | C | C | A*34:01:01 | C |
| A*66:01:01G | C | C | C | C | C | C | C | C | C | A*66:01:01G | C |
| A*66:03:01G | C | I | C | | WD | WD | C | C | C | A*66:03 | WD |
| A*68:01:01G | C | C | C | C | C | C | C | C | C | A*68:01:01G | C |
| A*68:01:02G | C | C | C | C | C | C | C | C | C | A*68:01:02G | C |
| A*68:02:01G | C | C | C | C | C | C | C | C | C | A*68:02:01G | C |
| A*69:01:01G | C | I | WD | I | I | C | C | WD | C | A*69:01 | C |
| A*74:01:01G | C | C | C | C | C | C | C | C | C | A*74:01:01G | C |
| A*80:01:01G | C | C | C | I | C | C | C | C | C | A*80:01 | C |

C, common; I, intermediate; WD, well-documented

[a] Alleles observed at least five times in any population group in the current dataset are included in this table. The exception are alleles that were observed in previous datasets but that are not present or are observed less than five times in this dataset. The table does not list all alleles from IPD-IMGT/HLA version 3.31.0.

**Supplementary Table 5a. HLA-A G level frequencies between CIWD versions [a]**

| 3.0.0 CIWD Category by Population Group [b] | | | | | | | | | | 2.0.0 CWD | |
|---|---|---|---|---|---|---|---|---|---|---|---|
| HLA-A IPD-HLA/IMGT 3.31.0 | Highest Frequency | Total | AFA | API | EURO | MENA | HIS | NAM | UNK | HLA-A IPD-IMGT/HLA 3.9.0 | Category |

[b] Population groups include: AFA (African/African American), API (Asian/Pacific Islands), EURO (European/European descent), MENA (Middle East/North Coast of Africa), HIS (South or Central America/Hispanic/Latino), NAM (Native American populations) and UNK (unknown/not asked/multiple ancestries/other). Total is the overall population i.e., all groups combined. Highest frequency is the highest CIWD designation among all the individual groups.

[c] A*02:17:01G was reported in the dataset but A*02:17:01 was found to be identical to A*02:17:02 so there is only one member in this G group.

[d] Not reported as G in the dataset; however, version 3.31.0 nomenclature has assigned this allele to a G group.

[e] Alleles in this column without a G designation were not assigned as a G group in 2.0.0 CWD. The allele listed is the allele the G group was named after.

**References**

Mack SJ, Cano P, Hollenbach JA, He J, Hurley CK, Middleton D, Moraes ME, Pereira SE, Kempenich JH, Reed EF, Setterholm M, Smith AG, Tilanus MG, Torres M, Varney MD, Voorter CE, Fischer GF, Fleischhauer K, Goodridge D, Klitz W, Little AM, Maiers M, Marsh SG, Muller CR, Noreen H, Rozemuller EH, Sanchez-Mazas A, Senitzer D, Trachtenberg E, Fernandez-Vina M: Common and well-documented HLA alleles: 2012 update to the CWD catalogue. Tissue Antigens 81:194-203, 2013.

**Supplementary Table 5b. HLA-B G level frequencies between CIWD versions [a]**

| 3.0.0 CIWD Category by Population Group [b] | | | | | | | | | | 2.0.0 CWD | |
|---|---|---|---|---|---|---|---|---|---|---|---|
| HLA-B IPD-HLA/IMGT 3.31.0 | Highest Frequency | Total | AFA | API | EURO | MENA | HIS | NAM | UNK | HLA-B IPD-IMGT/HLA 3.9.0 | Category |
| B*07:02:01G | C | C | C | C | C | C | C | C | C | B*07:02:01G | C |
| B*07:05:01G | C | C | C | C | C | C | C | C | C | B*07:05:01G | C |
| B*07:37:01G | I | WD | | | WD | | | | I | B*07:37 [d] | WD |
| B*08:01:01G | C | C | C | C | C | C | C | C | C | B*08:01:01G | C |
| B*13:01:01G | C | C | C | C | C | C | C | C | C | B*13:01:01G | C |
| B*13:02:01G | C | C | C | C | C | C | C | C | C | B*13:02:01G | C |
| B*14:01:01G | C | C | C | C | C | C | C | C | C | B*14:01:01 | C |
| B*14:02:01G | C | C | C | C | C | C | C | C | C | B*14:02:01 | C |
| B*15:01:01G | C | C | C | C | C | C | C | C | C | B*15:01:01G | C |
| B*15:02:01G | C | C | C | C | I | C | C | C | C | B*15:02:01G | C |
| B*15:03:01G | C | C | C | C | C | C | C | C | C | B*15:03:01G | C |
| B*15:04:01G | C | C | WD | C | I | | C | C | C | B*15:04 | WD |
| B*15:07:01G | C | C | WD | C | C | | C | C | C | B*15:07:01G | C |
| B*15:09:01G | C | C | WD | I | C | C | C | C | C | B*15:09 | C |
| B*15:11:01G | C | C | | C | I | C | WD | | C | B*15:11:01G | C |
| B*15:12:01G | C | C | | C | WD | | I | | C | B*15:12:01G | C |
| B*15:16:01G | C | C | C | I | C | C | C | C | C | B*15:16:01 | C |
| B*15:17:01G | C | C | C | C | C | C | C | C | C | B*15:17:01G | C |
| B*15:18:01G | C | C | C | C | C | C | C | C | C | B*15:18:01G | C |
| B*15:21:01G | C | C | WD | C | I | WD | I | | C | B*15:21 | C |
| B*15:25:01G | C | C | WD | C | I | C | I | C | C | B*15:25:01G | C |
| B*15:28:01G | o [c] | o | | | | | | | | B*15:28 | WD |
| B*15:30:01G | C | C | WD | I | I | WD | C | C | C | B*15:30 | C |
| B*15:123:01G | WD | WD | | | | WD | | | | B*15:123 | o |
| B*18:01:01G | C | C | C | C | C | C | C | C | C | B*18:01:01G | C |
| B*18:05:01G | C | C | WD | I | C | C | C | C | C | B*18:05 | WD |
| B*18:18:01G | I | I | | | I | | | | I | B*18:18 | WD |
| B*27:02:01G | C | C | C | C | C | C | C | C | C | B*27:02:01 | C |
| B*27:04:01G | C | C | WD | C | I | C | I | C | C | B*27:04:01G | C |

**Supplementary Table 5b. HLA-B G level frequencies between CIWD versions [a]**

| 3.0.0 CIWD Category by Population Group [b] | | | | | | | | | | 2.0.0 CWD | |
|---|---|---|---|---|---|---|---|---|---|---|---|
| HLA-B IPD-HLA/IMGT 3.31.0 | Highest Frequency | Total | AFA | API | EURO | MENA | HIS | NAM | UNK | HLA-B IPD-IMGT/HLA 3.9.0 | Category |
| B*27:05:02G | C | C | C | C | C | C | C | C | C | B*27:05:02G | C |
| B*27:07:01G | C | C | WD | C | C | C | C | C | C | B*27:07:01G | C |
| B*27:12:01G | C | I | WD | | I | C | C | | C | B*27:12 | WD |
| B*35:01:01G | C | C | C | C | C | C | C | C | C | B*35:01:01G | C |
| B*35:02:01G | C | C | C | C | C | C | C | C | C | B*35:02:01 | C |
| B*35:03:01G | C | C | C | C | C | C | C | C | C | B*35:03:01G | C |
| B*35:05:01G | C | C | C | C | I | C | C | C | C | B*35:05:01 | C |
| B*35:08:01G | C | C | C | C | C | C | C | C | C | B*35:08:01 | C |
| B*35:43:01G | C | C | C | I | I | WD | C | C | C | B*35:43:01G | C |
| B*37:01:01G | C | C | C | C | C | C | C | C | C | B*37:01:01G | C |
| B*38:01:01G | C | C | C | C | C | C | C | C | C | B*38:01:01 | C |
| B*38:02:01G | C | C | C | C | I | C | C | C | C | B*38:02:01G | C |
| B*39:01:01G | C | C | C | C | C | C | C | C | C | B*39:01:01G | C |
| B*39:02:02G | C | C | C | I | I | | C | C | C | B*39:02:02 | C |
| B*39:03:01G | C | C | WD | | I | | C | C | C | B*39:03 | C |
| B*39:05:01G | C | C | C | C | C | C | C | C | C | B*39:05:01 | C |
| B*39:06:02G | C | C | C | C | C | C | C | C | C | B*39:06:02 | C |
| B*39:09:01G | C | C | WD | C | I | | C | C | C | B*39:09 | WD |
| B*39:14:01G | C | I | | | WD | | C | C | I | B*39:14 | WD |
| B*39:31:01G | C | I | WD | I | C | C | I | | I | B*39:31 | WD |
| B*40:01:01G | C | C | C | C | C | C | C | C | C | B*40:01:01G | C |
| B*40:02:01G | C | C | C | C | C | C | C | C | C | B*40:02:01G | C |
| B*40:03:01G | C | I | WD | C | WD | | C | C | C | B*40:03 | WD |
| B*40:05:01G | C | C | WD | I | I | | C | C | C | B*40:05 | C |
| B*40:06:01G | C | C | C | C | C | C | C | C | C | B*40:06:01G | C |
| B*40:10:01G | C | I | | C | WD | | WD | | C | B*40:10:01 | WD |
| B*40:20:01G | C | I | | | WD | | C | C | I | B*40:20 | WD |
| B*40:40:01G | I | WD | | I | | | | | WD | B*40:40 | WD |
| B*40:155:01G | WD | WD | | WD | | | | | | B*40:155 | o |

**Supplementary Table 5b. HLA-B G level frequencies between CIWD versions [a]**

| 3.0.0 CIWD Category by Population Group [b] | | | | | | | | | | 2.0.0 CWD | |
|---|---|---|---|---|---|---|---|---|---|---|---|
| HLA-B IPD-HLA/IMGT 3.31.0 | Highest Frequency | Total | AFA | API | EURO | MENA | HIS | NAM | UNK | HLA-B IPD-IMGT/HLA 3.9.0 | Category |
| B*41:02:01G | C | C | C | C | C | C | C | C | C | B*41:02:01 | C |
| B*42:01:01G | C | C | C | C | C | C | C | C | C | B*42:01:01 | C |
| B*42:02:01G | C | C | C | I | C | C | C | C | C | B*42:02 | C |
| B*44:02:01G | C | C | C | C | C | C | C | C | C | B*44:02:01G | C |
| B*44:03:01G | C | C | C | C | C | C | C | C | C | B*44:03:01G | C |
| B*44:03:02G | C | C | C | C | C | C | C | C | C | B*44:03:02 | C |
| B*44:15:01G | C | WD | C | | WD | | | | WD | B*44:15 | WD |
| B*45:01:01G | C | C | C | C | C | C | C | C | C | B*45:01:01G | C |
| B*46:01:01G | C | C | C | C | C | C | C | C | C | B*46:01:01G | C |
| B*47:01:01G | C | C | C | C | C | C | C | C | C | B*47:01:01G | C |
| B*48:01:01G | C | C | C | C | C | C | C | C | C | B*48:01:01G | C |
| B*49:01:01G | C | C | C | C | C | C | C | C | C | B*49:01:01 | C |
| B*50:01:01G | C | C | C | C | C | C | C | C | C | B*50:01:01 | C |
| B*51:01:01G | C | C | C | C | C | C | C | C | C | B*51:01:01G | C |
| B*51:02:01G | C | C | C | C | I | C | C | C | C | B*51:02:01 | C |
| B*52:01:01G | C | C | C | C | C | C | C | C | C | B*52:01:01G | C |
| B*52:01:02G | C | C | C | C | C | C | C | C | C | B*52:01:02 | C |
| B*53:01:01G | C | C | C | C | C | C | C | C | C | B*53:01:01 | C |
| B*54:01:01G | C | C | WD | C | I | C | I | C | C | B*54:01:01G | C |
| B*55:01:01G | C | C | C | C | C | C | C | C | C | B*55:01:01G | C |
| B*55:02:01G | C | C | WD | C | I | C | I | | C | B*55:02:01G | C |
| B*56:01:01G | C | C | C | C | C | C | C | C | C | B*56:01:01G | C |
| B*57:01:01G | C | C | C | C | C | C | C | C | C | B*57:01:01G | C |
| B*57:03:01G | C | C | C | C | C | C | C | C | C | B*57:03:01 | C |
| B*58:01:01G | C | C | C | C | C | C | C | C | C | B*58:01:01G | C |
| B*59:01:01G | C | I | | C | WD | | I | | C | B*59:01:01G | C |
| B*67:01:02G | C | I | WD | C | I | | I | | I | B*67:01:02 | o |
| B*78:01:01G | C | C | C | I | I | C | C | C | C | B*78:01:01 | C |
| B*81:01:01G | C | C | C | C | I | C | C | C | C | B*81:01:01G | C |

**Supplementary Table 5b. HLA-B G level frequencies between CIWD versions [a]**

| 3.0.0 CIWD Category by Population Group [b] | | | | | | | | | | 2.0.0 CWD | |
|---|---|---|---|---|---|---|---|---|---|---|---|
| **HLA-B IPD-HLA/IMGT 3.31.0** | **Highest Frequency** | **Total** | **AFA** | **API** | **EURO** | **MENA** | **HIS** | **NAM** | **UNK** | **HLA-B IPD-IMGT/HLA 3.9.0** | **Category** |

C, common; I, intermediate; WD, well-documented

[a] Alleles observed at least five times in any population group in the current dataset are included in this table. The exception are alleles that were observed in previous datasets but that are not present or are observed less than five times in 3.0.0 dataset. The table does not list all alleles from IPD-IMGT/HLA version 3.31.0.

[b] Population groups include: AFA (African/African American), API (Asian/Pacific Islands), EURO (European/European descent), MENA (Middle East/North Coast of Africa), HIS (South or Central America/Hispanic/Latino), NAM (Native American populations) and UNK (unknown/not asked/multiple ancestries/other). Total is the overall population i.e., all groups combined. Highest frequency is the highest CIWD designation among all the individual groups.

[c] Observed in dataset but <5 observations.

[d] Alleles in this column without a G designation were not assigned as a G group in 2.0.0 CWD. The allele listed is the allele the G group was named after.

**Supplementary Table 5c. HLA-C G level frequencies between CIWD versions [a]**

| 3.0.0 CIWD Category by Population Group [b] | | | | | | | | | | 2.0.0 CWD | |
|---|---|---|---|---|---|---|---|---|---|---|---|
| HLA-C IPD-IMGT/HLA 3.31.0 | Highest Frequency | Total | AFA | API | EURO | MENA | HIS | NAM | UNK | HLA-C IPD-IMGT/HLA 3.9.0 | Category |
| C*01:02:01G | C | C | C | C | C | C | C | C | C | C*01:02:01G | C |
| C*01:03:01G | C | I | | C | WD | C | | | I | C*01:03:01G | WD |
| C*02:02:02G | C | C | C | C | C | C | C | C | C | C*02:02:02G | C |
| C*02:10:01G | C | C | C | I | C | C | C | C | C | C*02:10 [c] | C |
| C*02:14:01G | C | I | C | | WD | | I | | I | C*02:14 | WD |
| C*03:02:01G | C | C | C | C | C | C | C | C | C | C*03:02:01G | C |
| C*03:03:01G | C | C | C | C | C | C | C | C | C | C*03:03:01G | C |
| C*03:04:01G | C | C | C | C | C | C | C | C | C | C*03:04:01G | C |
| C*03:04:02G | C | C | C | C | C | C | C | C | C | C*03:04:02 | C |
| C*03:05:01G | C | C | C | I | I | WD | C | C | C | C*03:05 | C |
| C*03:14:01G | I | I | | | I | | I | | I | C*03:14 | WD |
| C*03:40:01G | I | WD | | | WD | | I | | I | C*03:40:01 | WD |
| C*04:01:01G | C | C | C | C | C | C | C | C | C | C*04:01:01G | C |
| C*04:03:01G | C | C | C | C | C | C | C | C | C | C*04:03 | C |
| C*04:04:01G | C | C | C | WD | I | C | C | C | C | C*04:04:01 | C |
| C*05:01:01G | C | C | C | C | C | C | C | C | C | C*05:01:01G | C |
| C*06:02:01G | C | C | C | C | C | C | C | C | C | C*06:02:01G | C |
| C*06:127:01G | I | WD | | I | | | | | | C*06:127:01G | o [d] |
| C*07:01:01G | C | C | C | C | C | C | C | C | C | C*07:01:01G | C |
| C*07:02:01G | C | C | C | C | C | C | C | C | C | C*07:02:01G | C |
| C*07:04:01G | C | C | C | C | C | C | C | C | C | C*07:04:01G | C |
| C*07:04:02G | WD | WD | | | WD | | | | | C*07:04:02G | o |
| C*08:01:01G | C | C | C | C | C | C | C | C | C | C*08:01:01G | C |
| C*08:02:01G | C | C | C | C | C | C | C | C | C | C*08:02:01G | C |
| C*08:03:01G | C | C | C | C | C | C | C | C | C | C*08:03:01G | C |
| C*12:02:01G | C | C | C | C | C | C | C | C | C | C*12:02:01G | C |
| C*12:03:01G | C | C | C | C | C | C | C | C | C | C*12:03:01G | C |
| C*14:02:01G | C | C | C | C | C | C | C | C | C | C*14:02:01G | C |

**Supplementary Table 5c. HLA-C G level frequencies between CIWD versions [a]**

| 3.0.0 CIWD Category by Population Group [b] | | | | | | | | | | 2.0.0 CWD | |
|---|---|---|---|---|---|---|---|---|---|---|---|
| **HLA-C IPD-IMGT/HLA 3.31.0** | **Highest Frequency** | **Total** | **AFA** | **API** | **EURO** | **MENA** | **HIS** | **NAM** | **UNK** | **HLA-C IPD-IMGT/HLA 3.9.0** | **Category** |
| C*14:03:01G | C | C | C | C | C | C | C | C | C | C*14:03 | C |
| C*15:02:01G | C | C | C | C | C | C | C | C | C | C*15:02:01G | C |
| C*15:04:01G | C | C | WD | C | C | C | C | C | C | C*15:04 | C |
| C*15:05:01G | C | C | C | C | C | C | C | C | C | C*15:05:01G | C |
| C*16:01:01G | C | C | C | C | C | C | C | C | C | C*16:01:01 | C |
| C*16:02:01G | C | C | C | C | C | C | C | C | C | C*16:02:01G | C |
| C*16:04:01G | C | C | C | C | C | C | C | C | C | C*16:04:01 | C |
| C*17:01:01G | C | C | C | C | C | C | C | C | C | C*17:01:01G | C |
| C*18:01:01G | C | C | C | I | C | C | C | C | C | C*18:01:01G | C |

C, common; I, intermediate; WD, well-documented

[a] Alleles observed at least five times in any population group in the current dataset are included in this table. The exception are alleles that were observed in previous datasets but that are not present or are observed less than five times. The table does not list all alleles from IPD-IMGT/HLA version 3.31.0.

[b] Population groups include: AFA (African/African American), API (Asian/Pacific Islands), EURO (European/European descent), MENA (Middle East/North Coast of Africa), HIS (South or Central America/Hispanic/Latino), NAM (Native American populations) and UNK (unknown/not asked/multiple ancestries/other). Total is the overall population i.e., all groups combined. Highest frequency is the highest CIWD designation among all the individual groups.

[c] Alleles in this column without a G designation were not assigned as G group in 2.0.0 CWD. The allele listed is the allele the G group was named after.

[d] Not CIWD, o

**Supplementary Table 5d. HLA-DRB1 G level frequencies between CIWD versions [a]**

| 3.0.0 CIWD Category by Population Group [b] | | | | | | | | | | 2.0.0 CWD | |
|---|---|---|---|---|---|---|---|---|---|---|---|
| HLA-DRB1 IPD-HLA/IMGT 3.31.0 | Highest Frequency | Total | AFA | API | EURO | MENA | HIS | NAM | UNK | HLA-DRB1 IPD-IMGT/HLA 3.9.0 | Category |
| DRB1*01:01:01G | C | C | C | C | C | C | C | C | C | DRB1*01:01:01 [c] | C |
| DRB1*01:02:01G | C | C | C | C | C | C | C | C | C | DRB1*01:02:01 | C |
| DRB1*03:01:01G | C | C | C | C | C | C | C | C | C | DRB1*03:01:01G | C |
| DRB1*04:01:01G | C | C | C | C | C | C | C | C | C | DRB1*04:01:01 | C |
| DRB1*04:03:01G | C | C | C | C | C | C | C | C | C | DRB1*04:03:01 | C |
| DRB1*04:05:01G | C | C | C | C | C | C | C | C | C | DRB1*04:05:01 | C |
| DRB1*04:06:01G | C | C | C | C | C | C | C | C | C | DRB1*04:06:01G | C |
| DRB1*04:07:01G | C | C | C | C | C | C | C | C | C | DRB1*04:07:01G | C |
| DRB1*04:10:01G | C | C | C | C | I | C | C | C | C | DRB1*04:10:01 | C |
| DRB1*07:01:01G | C | C | C | C | C | C | C | C | C | DRB1*07:01:01G | C |
| DRB1*08:01:01G | C | C | C | C | C | C | C | C | C | DRB1*08:01:01G | C |
| DRB1*08:02:01G | C | C | C | C | C | C | C | C | C | DRB1*08:02:01 | C |
| DRB1*08:03:02G | C | C | C | C | C | C | C | C | C | DRB1*08:03:02 | C |
| DRB1*09:01:02G | C | C | C | C | C | C | C | C | C | DRB1*09:01:02 | C |
| DRB1*10:01:01G | C | C | C | C | C | C | C | C | C | DRB1*10:01:01 | C |
| DRB1*11:01:01G | C | C | C | C | C | C | C | C | C | DRB1*11:01:01G | C |
| DRB1*11:04:01G | C | C | C | C | C | C | C | C | C | DRB1*11:04:01 | C |
| DRB1*11:06:01G | C | C | WD | C | I | C | I | C | C | DRB1*11:06:01G | C |
| DRB1*11:11:01G | C | I | WD | C | I | WD | WD | | I | DRB1*11:11:01G | C |
| DRB1*11:13:01G | I | I | WD | | I | WD | I | | I | DRB1*11:13:01G | WD |
| DRB1*12:01:01G | C | C | C | C | C | C | C | C | C | DRB1*12:01:01G | C |
| DRB1*12:02:01G | C | C | C | C | C | C | C | C | C | DRB1*12:02:01 | C |
| DRB1*13:01:01G | C | C | C | C | C | C | C | C | C | DRB1*13:01:01G | C |
| DRB1*13:02:01G | C | C | C | C | C | C | C | C | C | DRB1*13:02:01 | C |
| DRB1*13:03:01G | C | C | C | C | C | C | C | C | C | DRB1*13:03:01 | C |
| DRB1*14:01:01G | C | C | C | C | C | C | C | C | C | DRB1*14:01:01G | C |
| DRB1*14:02:01G | C | C | C | C | C | C | C | C | C | DRB1*14:02 | C |
| DRB1*14:05:01G | C | C | WD | C | I | C | I | | C | DRB1*14:05:01 | C |
| DRB1*15:01:01G | C | C | C | C | C | C | C | C | C | DRB1*15:01:01G | C |

**Supplementary Table 5d. HLA-DRB1 G level frequencies between CIWD versions [a]**

| 3.0.0 CIWD Category by Population Group [b] | | | | | | | | | | 2.0.0 CWD | |
|---|---|---|---|---|---|---|---|---|---|---|---|
| HLA-DRB1 IPD-HLA/IMGT 3.31.0 | Highest Frequency | Total | AFA | API | EURO | MENA | HIS | NAM | UNK | HLA-DRB1 IPD-IMGT/HLA 3.9.0 | Category |
| DRB1*15:02:01G | C | C | C | C | C | C | C | C | C | DRB1*15:02:01 | C |
| DRB1*15:03:01G | C | C | C | C | C | C | C | C | C | DRB1*15:03:01G | C |
| DRB1*16:02:01G | C | C | C | C | C | C | C | C | C | DRB1*16:02:01 | C |

C, common; I, intermediate; WD, well-documented

[a] Alleles observed at least five times in any population group in the current dataset are included in this table. The exception are alleles that were observed in previous datasets but that are not present or are observed less than five times. The table does not list all alleles from IPD-IMGT/HLA version 3.31.0.

[b] Population groups include: AFA (African/African American), API (Asian/Pacific Islands), EURO (European/European descent), MENA (Middle East/North Coast of Africa), HIS (South or Central America/Hispanic/Latino), NAM (Native American populations) and UNK (unknown/not asked/multiple ancestries/other). Total is the overall population i.e., all groups combined. Highest frequency is the highest CIWD designation among all the individual groups.

[c] Alleles in this column without a G designation were not assigned as G group in 2.0.0 CWD. The allele listed is the allele the G group was named after.

**Supplementary Table 5e. HLA-DRB3/4/5 G level frequencies between CIWD versions [a]**

| 3.0.0 CIWD Category by Population Group [b] | | | | | | | | | | 2.0.0 CWD | |
|---|---|---|---|---|---|---|---|---|---|---|---|
| **HLA-DRB3/4/5 IPD-IMGT/HLA 3.31.0** | **Highest Frequency** | Total | **AFA** | **API** | **EURO** | **MENA** | **HIS** | **NAM** | **UNK** | **HLA-DRB3/4/5 IPD-IMGT/HLA 3.9.0** | **Category** |
| DRB3*01:01:02G | WD | WD | WD | WD | WD | WD | WD | WD | WD | DRB3*01:01:02G | C |
| DRB3*02:01:01G | WD | WD | WD | WD | WD | WD | WD | WD | WD | DRB3*02:01:01G | C |
| DRB3*02:02:01G | WD | WD | WD | WD | WD | WD | WD | WD | WD | DRB3*02:02:01G | C |
| DRB3*03:01:01G | WD | WD | WD | WD | WD | WD | WD | WD | WD | DRB3*03:01:01G | C |
| DRB4*01:01:01G | WD | WD | WD | WD | WD | WD | WD | WD | WD | DRB4*01:01:01G | C |
| DRB5*01:01:01G | WD | WD | WD | WD | WD | WD | WD | WD | WD | DRB5*01:01:01 [c] | C |
| DRB5*01:02:01G | WD | WD | WD | WD | WD | WD | WD | WD | WD | DRB5*01:02:01G | C |
| DRB5*02:02:01G | WD | WD | WD | WD | WD | WD | WD | WD | WD | DRB5*02:02:01G | C |

C, common; WD, well-documented

[a] Because we are uncertain about the denominator, we are unable to estimate frequencies. All assignments observed five or more times are listed as WD. The table does not list all alleles from IPD-IMGT/HLA version 3.31.0.

[b] Population groups include: AFA (African/African American), API (Asian/Pacific Islands), EURO (European/European descent), MENA (Middle East/North Coast of Africa), HIS (South or Central America/Hispanic/Latino), NAM (Native American populations) and UNK (unknown/not asked/multiple ancestries/other). Total is the overall population i.e., all groups combined. Highest frequency is the highest CIWD designation among all the individual groups.

[c] Alleles in this column without a G designation were not assigned as a G group in 2.0.0 CWD. The allele listed is the allele the G group was named after.

**Supplementary Table 5f. HLA-DQB1 G level frequencies between CIWD versions [a]**

| | 3.0.0 CIWD Category by Population Group [b] | | | | | | | | 2.0.0 CWD | |
|---|---|---|---|---|---|---|---|---|---|---|
| HLA-DQB1 IPD-HLA/IMGT 3.31.0 | Highest Frequency | Total | AFA | API | EURO | MENA | HIS | NAM | UNK | HLA-DQB1 IPD-IMGT/HLA 3.9.0 | Category |
| DQB1*02:01:01G | C | C | C | C | C | C | C | C | C | DQB1*02:01:01G | C |
| DQB1*03:01:01G | C | C | C | C | C | C | C | C | C | DQB1*03:01:01G | C |
| DQB1*03:02:01G | C | C | C | C | C | C | C | C | C | DQB1*03:02:01G | C |
| DQB1*03:03:02G | C | C | C | C | C | C | C | C | C | DQB1*03:03:02G | C |
| DQB1*03:04:01G | C | C | C | I | C | C | C | C | C | DQB1*03:04 [c] | C |
| DQB1*03:05:01G | C | C | C | C | C | C | C | C | C | DQB1*03:05:01 | C |
| DQB1*03:10:01G | WD | WD | | | WD | | | | | DQB1*03:10:01 | o [d] |
| DQB1*03:10:02G | WD | WD | | | WD | | | | | DQB1*03:10:02 | o |
| DQB1*04:01:01G | C | C | WD | C | I | C | C | C | C | DQB1*04:01:01G | C |
| DQB1*04:02:01G | C | C | C | C | C | C | C | C | C | DQB1*04:02:01 | C |
| DQB1*05:01:01G | C | C | C | C | C | C | C | C | C | DQB1*05:01:01G | C |
| DQB1*05:02:01G | C | C | C | C | C | C | C | C | C | DQB1*05:02:01G | C |
| DQB1*05:03:01G | C | C | C | C | C | C | C | C | C | DQB1*05:03:01G | C |
| DQB1*05:04:01G | C | C | C | C | C | C | C | C | C | DQB1*05:04 | C |
| DQB1*06:01:01G | C | C | C | C | C | C | C | C | C | DQB1*06:01:01G | C |
| DQB1*06:02:01G | C | C | C | C | C | C | C | C | C | DQB1*06:02:01G | C |
| DQB1*06:03:01G | C | C | C | C | C | C | C | C | C | DQB1*06:03:01G | C |
| DQB1*06:04:01G | C | C | C | C | C | C | C | C | C | DQB1*06:04:01G | C |
| DQB1*06:09:01G | C | C | C | C | C | C | C | C | C | DQB1*06:09 | C |

C, common; I, intermediate; WD, well-documented

[a] Alleles observed at least five times in any population group in the current dataset are included in this table. The exception are alleles that were observed in previous datasets but that are not present or are observed less than five times. The table does not list all alleles from IPD-IMGT/HLA version 3.31.0.

**Supplementary Table 5f. HLA-DQB1 G level frequencies between CIWD versions [a]**

| | 3.0.0 CIWD<br>Category by Population Group [b] | | | | | | | | | 2.0.0 CWD | |
|---|---|---|---|---|---|---|---|---|---|---|---|
| HLA-DQB1 IPD-HLA/IMGT 3.31.0 | Highest Frequency | Total | AFA | API | EURO | MENA | HIS | NAM | UNK | HLA-DQB1 IPD-IMGT/HLA 3.9.0 | Category |

[b] Population groups include: AFA (African/African American), API (Asian/Pacific Islands), EURO (European/European descent), MENA (Middle East/North Coast of Africa), HIS (South or Central America/Hispanic/Latino), NAM (Native American populations) and UNK (unknown/not asked/multiple ancestries/other). Total is the overall population i.e., all groups combined. Highest frequency is the highest CIWD designation among all the individual groups.

[c] Alleles in this column without a G designation were not assigned as a G group in 2.0.0 CWD. The allele listed is the allele the G group was named after.

[d] Not CWD, o

**Supplementary Table 5g. HLA-DPB1 G level frequencies between CIWD versions [a]**

| | 3.0.0 CIWD Category by Population Group [b] | | | | | | | | | 2.0.0 CWD | |
|---|---|---|---|---|---|---|---|---|---|---|---|
| **HLA-DPB1 IPD-HLA/IMGT 3.31.0** | **Highest Frequency** | Total | **AFA** | **API** | **EURO** | **MENA** | **HIS** | **NAM** | **UNK** | **DPB1 IPD-IMGT/HLA 3.9.0** | **Category** |
| DPB1*01:01:01G | C | C | C | C | C | C | C | C | C | DPB1*01:01:01 [c] | C |
| DPB1*01:01:02G | C | C | C | I | C | C | C | C | C | DPB1*01:01:02 | C |
| DPB1*02:01:02G | C | C | C | C | C | C | C | C | C | DPB1*02:01:02 | C |
| DPB1*02:02:01G | C | C | C | C | C | C | C | C | C | DPB1*02:02 | C |
| DPB1*03:01:01G | C | C | C | C | C | C | C | C | C | DPB1*03:01:01G | C |
| DPB1*04:01:01G | C | C | C | C | C | C | C | C | C | DPB1*04:01:01G | C |
| DPB1*04:01:04G | C | I | | | I | | C | | I | | o [d] |
| DPB1*04:02:01G | C | C | C | C | C | C | C | C | C | DPB1*04:02:01G | C |
| DPB1*05:01:01G | C | C | C | C | C | C | C | C | C | DPB1*05:01:01G | C |
| DPB1*06:01:01G | C | C | C | C | C | C | C | C | C | DPB1*06:01 | C |
| DPB1*10:01:01G | C | C | C | C | C | C | C | C | C | DPB1*10:01 | C |
| DPB1*11:01:01G | C | C | C | C | C | C | C | C | C | DPB1*11:01:01 | C |
| DPB1*13:01:01G | C | C | C | C | C | C | C | C | C | DPB1*13:01:01G | C |
| DPB1*14:01:01G | C | C | C | C | C | C | C | C | C | DPB1*14:01 | C |
| DPB1*15:01:01G | C | C | C | C | C | C | C | C | C | DPB1*15:01 | C |
| DPB1*16:01:01G | C | C | C | C | C | C | C | C | C | DPB1*16:01 | C |
| DPB1*17:01:01G | C | C | C | C | C | C | C | C | C | DPB1*17:01:01G | C |
| DPB1*19:01:01G | C | C | C | C | C | C | C | C | C | DPB1*19:01:01G | C |
| DPB1*20:01:01G | C | C | C | C | C | C | C | C | C | DPB1*20:01:01 | C |
| DPB1*23:01:01G | C | C | C | C | C | C | C | C | C | DPB1*23:01:01G | C |
| DPB1*28:01:01G | C | C | C | C | I | C | I | C | C | DPB1*28:01 | C |
| DPB1*30:01:01G | C | C | C | I | C | C | C | C | C | DPB1*30:01 | C |
| DPB1*33:01:01G | I | I | | I | I | WD | I | | I | DPB1*33:01 | WD |
| DPB1*34:01:01G | C | C | C | C | C | C | C | C | C | DPB1*34:01 | C |
| DPB1*39:01:01G | C | C | C | I | I | WD | C | C | C | DPB1*39:01 | C |
| DPB1*40:01:01G | C | C | C | WD | I | WD | C | C | C | DPB1*40:01 | WD |
| DPB1*41:01:01G | C | I | | C | I | C | I | | I | DPB1*41:01 | o |
| DPB1*47:01:01G | C | I | WD | C | I | C | | | I | DPB1*47:01 | WD |
| DPB1*49:01:01G | C | I | C | WD | WD | WD | C | C | I | DPB1*49:01 | WD |

**Supplementary Table 5g. HLA-DPB1 G level frequencies between CIWD versions [a]**

| 3.0.0 CIWD Category by Population Group [b] | | | | | | | | | | 2.0.0 CWD | |
|---|---|---|---|---|---|---|---|---|---|---|---|
| **HLA-DPB1 IPD-HLA/IMGT 3.31.0** | **Highest Frequency** | Total | **AFA** | **API** | **EURO** | **MENA** | **HIS** | **NAM** | **UNK** | **DPB1 IPD-IMGT/HLA 3.9.0** | **Category** |
| DPB1*55:01:01G | C | I | C | I | WD | WD | C | | I | DPB1*55:01 | C |
| DPB1*57:01:01G | I | I | WD | I | I | WD | I | | I | DPB1*57:01 | WD |
| DPB1*69:01:01G | WD | WD | WD | | WD | | WD | | | DPB1*69:01 | WD |
| DPB1*72:01:01G | I | WD | | I | WD | WD | | | I | DPB1*72:01 | WD |
| DPB1*81:01:01G | C | I | WD | I | I | C | I | | I | DPB1*81:01 | C |
| DPB1*85:01:01G | C | I | C | | WD | | I | | I | DPB1*85:01 | C |
| DPB1*91:01:01G | C | I | | C | WD | C | | | I | DPB1*91:01 | o |

C, common; I, intermediate; WD, well-documented

[a] Alleles observed at least five times in any population in the current dataset are included in this table. The exception are alleles that were observed in previous datasets but that are not present or are observed less than five times. The table does not list all alleles from IPD-IMGT/HLA version 3.31.0.
[b] Population groups include: AFA (African/African American), API (Asian/Pacific Islands), EURO (European/European descent), MENA (Middle East/North Coast of Africa), HIS (South or Central America/Hispanic/Latino), NAM (Native American populations) and UNK (unknown/not asked/multiple ancestries/other). Total is the overall population i.e., all groups combined. Highest frequency is the highest CIWD designation among all the individual groups.

[c] Alleles in this column without a G designation were not assigned as a G group in 2.0.0 CWD. The allele listed is the allele the G group was named after.
[d] Not CWD, o. The allele representing this G group was not described in 2012.
