# Supplementary material for: Common, intermediate and well‐documented HLA alleles in world populations: CIWD version 3.0.0
Source: HLA. 2020 Jan 31;95(6):516–31. doi: 10.1111/tan.13811 (PMC7317522; doi:10.1111/tan.13811)
Supplement: Supplementary file 6 — Table S6 Overall summary of nonexpressed HLA allele assignments [file TAN-95-516-s006.docx]

**Supplementary Table 6. Overall summary^a^ of non-expressed HLA allele assignments**

|  | **Total Non-expressed in IMGT 3.31.0** | **Total Non-Expressed Observed in Study** | **% of IMGT Total** | **Common** | **Intermediate** | **Well-Documented** | **Not-CIWD** |
| --- | --- | --- | --- | --- | --- | --- | --- |
| ***HLA-A*** | 186 | 76 | 40.9 | 1 | 2 | 29 | 44 |
| ***HLA-B*** | 144 | 43 | 29.9 | 0 | 0 | 16 | 27 |
| ***HLA-C*** | 134 | 60 | 44.8 | 1 | 3 | 16 | 40 |
| ***HLA-DRB1*** | 52 | 25 | 48.1 | 0 | 0 | 3 | 22 |
| ***HLA-DRB3/4/5*** | 17 | 7 | 41.2 | 0 | 0 | 6 | 1 |
| ***HLA-DQB1*** | 31 | 13 | 41.9 | 0 | 2 | 5 | 6 |
| ***HLA-DPB1*** | 24 | 13 | 54.2 | 1 | 1 | 6 | 5 |

a The summary is derived from data provided in Supplementary Table 7: HLA-A Supplementary Table 7a, HLA-B 7b, HLA-C 7c, HLA-DRB1 7d, HLA-DRB3/4/5 7e, HLA-DQB1 7f, HLA-DPB1 7g.
