# Supplementary material for: Common, intermediate and well‐documented HLA alleles in world populations: CIWD version 3.0.0
Source: HLA. 2020 Jan 31;95(6):516–31. doi: 10.1111/tan.13811 (PMC7317522; doi:10.1111/tan.13811)
Supplement: Supplementary file 7 — Table S7a‐g CIWD status of observed nonexpressed HLA two‐field allele assignments by locus [file TAN-95-516-s007.pdf]

Supplementary Table 7a. CIWD status of observed non-expressed HLA-A assignments [a]

3.0.0 CIWD Category by Population Group [b]										2.0.0 CWD		EFI CWD	China CWD
HLA-A	Highest Frequency	Total	AFA	API	EURO	MENA	HIS	NAM	UNK	HLA-A IPD-IMGT/HLA 3.9.0	Category	Category	Category
A*01:04N	WD	WD			WD					A*01:04N	WD		
A*01:15N	WD	WD			WD					A*01:15N			
A*01:16N	WD	WD			WD				WD	A*01:16N			
A*01:18N	o [d]	o								A*01:18N			
A*01:22N	o	o								A*01:22N			
A*01:31N	o	o								A*01:31N			
A*01:52N [c]	o	o								A*01:52N			
A*01:57N	WD	WD			WD					A*01:57N			
A*01:123N	WD	WD			WD					A*01:123N			
A*01:160N	o	o								A*01:160N			
A*01:162N	o	o								A*01:162N			
A*02:53N	I	WD		I					WD	A*02:53N	C		WD
A*02:83N	WD	WD			WD					A*02:83N			
A*02:88N	o	o								A*02:88N			
A*02:94N	WD	WD			WD					A*02:94N			
A*02:113N	WD	WD								A*02:113N			
A*02:125N	WD	WD			WD					A*02:125N			
A*02:223N	o	o								A*02:223N			
A*02:227N	WD	WD			WD					A*02:227N			
A*02:250N	o	o								A*02:250N			
A*02:350N	o	o								A*02:350N			
A*02:366N	o	o								A*02:366N			
A*02:514N	WD	WD		WD						A*02:514N			
A*03:01:01:02N	o	o								A*03:01:01:02N			
A*03:11N	o	o								A*03:11N			

Supplementary Table 7a. CIWD status of observed non-expressed HLA-A assignments [a]

3.0.0 CIWD Category by Population Group [b]										2.0.0 CWD		EFI CWD	China CWD
HLA-A	Highest Frequency	Total	AFA	API	EURO	MENA	HIS	NAM	UNK	HLA-A IPD-IMGT/HLA 3.9.0	Category	Category	Category
A*03:21N	WD	WD			WD					A*03:21N	WD		
A*03:36N	o	o								A*03:36N			
A*03:68N	o	o								A*03:68N			
A*03:69N	o	o								A*03:69N			
A*03:192N	o	o								A*03:192N			
A*03:197N	o	o								A*03:197N			
A*03:262N	o	o								A*03:262N			
A*03:266N	o	o								A*03:266N			
A*11:21N	WD	WD								A*11:21N			
A*11:69N	o	o								A*11:69N			WD
A*11:109N	WD	WD			WD					A*11:109N			
A*11:115N	o	o								A*11:115N			
A*11:137N	o	o								A*11:137N			
A*11:208N	o	o								A*11:208N			
A*23:07N	o	o								A*23:07N			
A*23:08N	o	o								A*23:08N			
A*23:11N	WD	WD	WD							A*23:11N			
A*23:19N	C	WD	C						WD	A*23:19N			
A*24:09N	WD	WD			WD					A*24:09N	WD		
A*24:11N	I	WD		I	WD					A*24:11N	WD		
A*24:36N	WD	WD			WD					A*24:36N			
A*24:48N	o	o								A*24:48N			
A*24:60N	o	o								A*24:60N			
A*24:84N	WD	WD			WD					A*24:84N			
A*24:86N	o	o								A*24:86N			

Supplementary Table 7a. CIWD status of observed non-expressed HLA-A assignments [a]

3.0.0 CIWD Category by Population Group [b]										2.0.0 CWD		EFI CWD	China CWD
HLA-A	Highest Frequency	Total	AFA	API	EURO	MENA	HIS	NAM	UNK	HLA-A IPD-IMGT/HLA 3.9.0	Category	Category	Category
A*24:90N	WD	WD			WD					A*24:90N	WD		
A*24:132N	o	o								A*24:132N			WD
A*24:252N	WD	WD			WD	WD				A*24:252N			
A*24:312N	o	o								A*24:312N			
A*24:359N	o	o								A*24:359N			
A*25:12N	WD	WD			WD					A*25:12N			
A*26:71N	o	o								A*26:71N			
A*30:59N	o	o								A*30:59N			
A*30:70N	WD	WD	WD							A*30:70N			
A*30:73N	o	o								A*30:73N			
A*30:76N	o	o								A*30:76N			
A*30:78N	WD	WD	WD							A*30:78N			
A*31:01:02:03N	o	o								A*31:01:02:03N			
A*31:14N	WD	WD			WD					A*31:14N			
A*31:60N	WD	WD								A*31:60N			
A*32:19N	o	o								A*32:19N			
A*32:27N	WD	WD			WD					A*32:27N			
A*32:45N	WD	WD			WD					A*32:45N			
A*33:73N	o	o								A*33:73N			
A*33:80N	o	o								A*33:80N			
A*33:96N	o	o								A*33:96N			
A*33:123N	o	o								A*33:123N			
A*34:10N	WD	WD	WD							A*34:10N			
A*68:11N	o	o								A*68:11N	WD		
A*68:18N	WD	WD			WD					A*68:18N			

Supplementary Table 7a. CIWD status of observed non-expressed HLA-A assignments [a]

3.0.0 CIWD Category by Population Group [b]										2.0.0 CWD		EFI CWD	China CWD
HLA-A	Highest Frequency	Total	AFA	API	EURO	MENA	HIS	NAM	UNK	HLA-A IPD-IMGT/HLA 3.9.0	Category	Category	Category
A*68:49N	o	o								A*68:49N			
A*68:59N	o	o								A*68:59N			
A*74:14N	o	o								A*74:14N			

C, common; I, intermediate; WD, well-documented

[a] Null alleles observed at least once in any population group in the current dataset or observed in the earlier catalogs are included in this table. The table does not list all non-expressed alleles from IPD-IMGT/HLA version 3.31.0.

[b] Population groups include: AFA (African/African American), API (Asian/Pacific Islander), EURO (European/European descent), MENA (Middle East/North Coast of Africa), HIS (Hispanic), NAM (Native American) and UNK (unknown, multiple ancestries or other). Total is the overall population i.e., all groups combined. Highest frequency is the highest CIWD designation among all the individual groups.

[c] Registries often reported a truncated assignment rather than the four field name designation. For example, A*02:113:01N, A*02:113:02N and A*02:113 were all reported.

[d] Not CIWD, o

References

Mack SJ, Cano P, Hollenbach JA, He J, Hurley CK, Middleton D, Moraes ME, Pereira SE, Kempenich JH, Reed EF, Setterholm M, Smith AG, Tilanus MG, Torres M, Varney MD, Voorter CE, Fischer GF, Fleischhauer K, Goodridge D, Klitz W, Little AM, Maiers M, Marsh SG, Muller CR, Noreen H, Rozemuller EH, Sanchez-Mazas A, Senitzer D, Trachtenberg E, Fernandez-Vina M: Common and well-documented HLA alleles: 2012 update to the CWD catalogue. Tissue Antigens 81:194-203, 2013.

Supplementary Table 7a. CIWD status of observed non-expressed HLA-A assignments [a]

3.0.0 CIWD Category by Population Group [b]										2.0.0 CWD		EFI CWD	China CWD
HLA-A	Highest Frequency	Total	AFA	API	EURO	MENA	HIS	NAM	UNK	HLA-A IPD-IMGT/HLA 3.9.0	Category	Category	Category

Sanchez-Mazas A, Nunes JM, Middleton D, Sauter J, Buhler S, McCabe A, Hofmann J, Baier DM, Schmidt AH, Nicoloso G, Andreani M, Grubic Z, Tiercy JM, Fleischhauer K: Common and well-documented HLA alleles over all of Europe and within European sub-regions: A catalogue from the European Federation for Immunogenetics. HLA 89:104-113, 2017.

He Y, Li J, Mao W, Zhang D, Liu M, Shan X, Zhang B, Zhu C, Shen J, Deng Z, Wang Z, Yu W, Chen Q, Guo W, Su P, Lv R, Li G, Li G, Pei B, Jiao L, Shen G, Liu Y, Feng Z, Su Y, Xie Y, Di W, Liu X, Yang X, Wang J, Qi J, Liu Q, Han Y, He J, Cai J, Zhang Z, Zhu F, Du D: HLA common and well-documented alleles in China. HLA 92:199-205, 2018.

Supplementary Table 7b. CIWD status of observed non-expressed HLA-B assignments [a]

3.0.0 CIWD Category by Population Group [b]										2.0.0 CWD		EFI CWD	China CWD
HLA-B	Highest Frequency	Total	AFA	API	EURO	MENA	HIS	NAM	UNK	HLA-B IPD-IMGT/HLA 3.9.0	Category	Category	Category
B*07:44N	o [c]	o								B*07:44N			
B*07:67N	WD	WD			WD					B*07:67N		WD	
B*07:111N	o	o								B*07:111N			
B*07:167N	o	o								B*07:167N			
B*07:181N	WD	WD			WD					B*07:181N			
B*07:182N	o	o								B*07:182N			
B*07:231N	o	o								B*07:231N			
B*08:30N	o	o								B*08:30N			
B*08:72N	o	o								B*08:72N			
B*08:86N	o	o								B*08:86N			
B*13:56:01N	o	o								B*13:56:01N			
B*14:07N	WD	WD			WD					B*14:07N		WD	
B*15:01:01:02N	WD	WD			WD					B*15:01:01:02N	WD		
B*15:380N	o	o								B*15:380N			
B*15:79N	WD	WD			WD					B*15:79N		WD	
B*15:181N	WD	WD			WD					B*15:181N		WD	
B*15:190N	WD	WD			WD					B*15:190N			
B*15:272N	o	o								B*15:272N			
B*18:94N	o	o								B*18:94N			
B*27:65N	o	o								B*27:65N			
B*27:94N	o	o								B*27:94N			
B*35:130N	o	o								B*35:130N			
B*35:165N	WD	WD								B*35:165N			
B*35:216N	o	o								B*35:216N			
B*37:03N	WD	WD			WD					B*37:03N	WD	WD	
B*37:33N	o	o								B*37:33N			
B*37:42N	WD	WD			WD					B*37:42N			
B*39:40N [d]	WD	WD			WD					B*39:40N [d]			

Supplementary Table 7b. CIWD status of observed non-expressed HLA-B assignments [a]

3.0.0 CIWD Category by Population Group [b]										2.0.0 CWD		EFI CWD	China CWD
HLA-B	Highest Frequency	Total	AFA	API	EURO	MENA	HIS	NAM	UNK	HLA-B IPD-IMGT/HLA 3.9.0	Category	Category	Category
B*40:22N	WD	WD								B*40:22N	WD		
B*40:23N	o	o								B*40:23N	WD		
B*40:118N	o	o								B*40:118N			
B*40:142N	WD	WD								B*40:142N			
B*40:155N	WD	WD		WD						B*40:155N			
B*40:291N	o	o								B*40:291N			
B*44:23N	WD	WD			WD					B*44:23N			
B*44:217N	o	o								B*44:217N			
B*51:11N	WD	WD			WD					B*51:11N	WD	WD	
B*51:27N	o	o								B*51:27N			
B*51:44N	o	o								B*51:44N			
B*51:98N	o	o								B*51:98N			
B*51:178N	o	o								B*51:178N			
B*55:55N	o	o								B*55:55N			
B*57:79N	o	o								B*57:79N			
B*81:04N	o	o								B*81:04N			

C, common; I, intermediate; WD, well-documented

[a] Null alleles observed at least once in any population group in the current dataset or observed in the earlier catalogs are included in this table. The table does not list all non-expressed alleles from IPD-IMGT/HLA version 3.31.0.

[b] Population groups include: AFA (African/African American), API (Asian/Pacific Islander), EURO (European/European descent), MENA (Middle East/North Coast of Africa), HIS (Hispanic), NAM (Native American) and UNK (unknown, multiple ancestries or other). Total is the overall population i.e., all groups combined. Highest frequency is the highest CIWD designation among all the individual groups.

[c] Not CIWD, o

[d] Registries often reported a truncated assignment rather than the four field name designation. For example, B*39:40:01N, B*39:40:02N and B*39:40 were all reported.

Supplementary Table 7c. CIWD status of observed non-expressed HLA-C assignments [a]

3.0.0 CIWD Category by Population Group [b]										2.0.0 CWD		EFI CWD	China CWD
HLA-C	Highest Frequency	Total	AFA	API	EURO	MENA	HIS	NAM	UNK	HLA-C IPD-IMGT/HLA 3.9.0	Category	Category	Category
C*01:37N	o [c]	o								C*01:37N			
C*01:56N	o	o								C*01:56N			
C*01:117N	o	o								C*01:117N			
C*02:38N	WD	WD			WD					C*02:38N		WD	
C*02:92N	WD	WD			WD					C*02:92N			
C*03:121N	o	o								C*03:121N			
C*03:189N	o	o								C*03:189N			
C*03:316N	o	o								C*03:316N			
C*04:09N	C	I	WD		I		C	C	I	C*04:09N	C	WD	
C*04:88N	o	o								C*04:88N			
C*04:93N	WD	WD			WD					C*04:93N		WD	
C*04:95N	WD	WD			WD					C*04:95N			
C*04:105N	o	o								C*04:105N			
C*04:123N	o	o								C*04:123N			
C*04:173N	o	o								C*04:173N			
C*04:191N	o	o								C*04:191N			
C*04:205N	o	o								C*04:205N			
C*04:215N	o	o								C*04:215N			
C*04:217N	o	o								C*04:217N			
C*04:236N	o	o								C*04:236N			
C*05:07N	I	I			I		I		I	C*05:07N	WD	WD	
C*05:91N	o	o								C*05:91N			
C*05:99N	WD	WD			WD					C*05:99N			
C*05:113N	o	o								C*05:113N			
C*05:128N	o	o								C*05:128N			
C*06:16N	WD	WD			WD					C*06:16N			
C*06:49N	o	o								C*06:49N			

Supplementary Table 7c. CIWD status of observed non-expressed HLA-C assignments [a]

3.0.0 CIWD Category by Population Group [b]										2.0.0 CWD		EFI CWD	China CWD
HLA-C	Highest Frequency	Total	AFA	API	EURO	MENA	HIS	NAM	UNK	HLA-C IPD-IMGT/HLA 3.9.0	Category	Category	Category
C*06:79N	I	WD		I						C*06:79N			
C*06:128N	o	o								C*06:128N			
C*06:152N	o	o								C*06:152N			
C*07:32N	WD	WD			WD				WD	C*07:32N	WD	WD	
C*07:33N	WD	WD			WD					C*07:33N			
C*07:55N	WD	WD			WD					C*07:55N		WD	
C*07:61N	WD	WD	WD							C*07:61N	WD		
C*07:98N	o	o								C*07:98N			
C*07:104N	WD	WD			WD					C*07:104N	WD		
C*07:191N	o	o								C*07:191N			
C*07:198N	WD	WD								C*07:198N			
C*07:227N	WD	WD								C*07:227N			
C*07:264N	o	o								C*07:264N			
C*07:329N	o	o								C*07:329N			
C*07:393N	o	o								C*07:393N			
C*07:452N	WD	WD			WD					C*07:452N			
C*07:476N	o	o								C*07:476N			
C*07:483N	o	o								C*07:483N			
C*07:484N	o	o								C*07:484N			
C*07:491N	o	o								C*07:491N			
C*08:26N	o	o								C*08:26N			
C*08:55N	o	o								C*08:55N			
C*08:127N	WD	WD		WD						C*08:127N			
C*12:46N	o	o								C*12:46N			
C*14:07N	o	o								C*14:07N			
C*14:47N	o	o								C*14:47N			
C*15:92N	o	o								C*15:92N			

Supplementary Table 7c. CIWD status of observed non-expressed HLA-C assignments [a]

3.0.0 CIWD Category by Population Group [b]										2.0.0 CWD		EFI CWD	China CWD
HLA-C	Highest Frequency	Total	AFA	API	EURO	MENA	HIS	NAM	UNK	HLA-C IPD-IMGT/HLA 3.9.0	Category	Category	Category
C*15:115N	o	o								C*15:115N			
C*15:122N	WD	WD								C*15:122N			
C*16:30N	I	WD		I						C*16:30N			
C*16:77N	o	o								C*16:77N			
C*16:89N	o	o								C*16:89N			
C*18:07N	o	o								C*18:07N			

C, common; I, intermediate; WD, well-documented

[a] Null alleles observed at least once in any population group in the current dataset or observed in the earlier catalogs are included in this table. The table does not list all non-expressed alleles from IPD-IMGT/HLA version 3.31.0.

[b] Population groups include: AFA (African/African American), API (Asian/Pacific Islander), EURO (European/European descent), MENA (Middle East/North Coast of Africa), HIS (Hispanic), NAM (Native American) and UNK (unknown, multiple ancestries or other). Total is the overall population i.e., all groups combined. Highest frequency is the highest CIWD designation among all the individual groups.

[c] Not CIWD, o

Supplementary Table 7d. CIWD status of observed non-expressed HLA-DRB1 assignments [a]

3.0.0 CIWD Category by Population Group [b]										2.0.0 CWD		EFI CWD	China CWD
**HLA-DRB1**	**Highest Frequency**	**Total**	**AFA**	**API**	**EUR**	**MENA**	**HIS**	**NAM**	**UNK**	**HLA-DRB1 IPD-IMGT/HLA 3.9.0**	**Category**	**Category**	**Category**
DRB1*01:40N	o [c]	o								DRB1*01:40N			
DRB1*01:68N	o	o								DRB1*01:68N			
DRB1*03:67N	o	o								DRB1*03:67N			
DRB1*03:68N	o	o								DRB1*03:68N			
DRB1*04:94:01N	o	o								DRB1*04:94:01N			
DRB1*04:119N	o	o								DRB1*04:119N			
DRB1*04:142N	o	o								DRB1*04:142N			
DRB1*04:157N	o	o								DRB1*04:157N			
DRB1*04:158N	o	o								DRB1*04:158N			
DRB1*07:10N	WD	WD			WD					DRB1*07:10N			
DRB1*07:26N	WD	WD			WD					DRB1*07:26N			
DRB1*07:58N	o	o								DRB1*07:58N			
DRB1*11:217N	o	o								DRB1*11:217N			
DRB1*12:24N	WD	WD			WD				WD	DRB1*12:24N			
DRB1*13:137N	o	o								DRB1*13:137N			
DRB1*13:142N	o	o								DRB1*13:142N			
DRB1*13:200N	o	o								DRB1*13:200N			
DRB1*14:152N	o	o								DRB1*14:152N			
DRB1*14:166N	o	o								DRB1*14:166N			
DRB1*15:80N	o	o								DRB1*15:80N			
DRB1*15:115N	o	o								DRB1*15:115N			
DRB1*15:129N	o	o								DRB1*15:129N			
DRB1*15:137N	o	o								DRB1*15:137N			
DRB1*16:13N	o	o								DRB1*16:13N			
DRB1*16:21N	o	o								DRB1*16:21N			

C, common; I, intermediate; WD, well-documented

Supplementary Table 7d. CIWD status of observed non-expressed HLA-DRB1 assignments [a]

3.0.0 CIWD Category by Population Group [b]										2.0.0 CWD		EFI CWD	China CWD
HLA-DRB1	Highest Frequency	Total	AFA	API	EUR	MENA	HIS	NAM	UNK	HLA-DRB1 IPD-IMGT/HLA 3.9.0	Category	Category	Category

[a] Null alleles observed at least once in any population group in the current dataset or observed in the earlier catalogs are included in this table. The table does not list all non-expressed alleles from IPD-IMGT/HLA version 3.31.0.

[b] Population groups include: AFA (African/African American), API (Asian/Pacific Islander), EURO (European/European descent), MENA (Middle East/North Coast of Africa), HIS (Hispanic), NAM (Native American) and UNK (unknown, multiple ancestries or other). Total is the overall population i.e., all groups combined. Highest frequency is the highest CIWD designation among all the individual groups.

[c] Not CIWD, o

Supplementary Table 7e. CIWD status of observed non-expressed HLA-DRB3/4/5 assignments [a]

3.0.0 CIWD Category by Population Group [b]										2.0.0 CWD		EFI CWD	China CWD
HLA-DQB1	Highest Frequency	Total	AFA	API	EURO	MENA	HIS	NAM	UNK	HLA-DRB3/4/5 IPD-IMGT/HLA 3.9.0	Category	Category	Category
DRB3*02:29N	o [c]	o								DRB3*02:29N			
DRB4*01:03N [d]	WD	WD	WD	WD	WD	WD	WD	WD	WD	DRB4*01:03N [d]	C		
DRB4*01:16N	WD	WD								DRB4*01:16N			
DRB4*02:01N	WD	WD			WD					DRB4*02:01N	C		
DRB4*03:01N	WD	WD			WD					DRB4*03:01N	WD		
DRB5*01:08N	WD	WD	WD	WD					WD	DRB5*01:08N	C		
DRB5*01:10N	WD	WD		WD	WD	WD	WD		WD	DRB5*01:10N	WD		

C, common; I, intermediate; WD, well-documented

[a] Null alleles observed at least once in any population group in the current dataset or observed in the earlier catalogs are included in this table. The table does not list all non-expressed alleles from IPD-IMGT/HLA version 3.31.0. Because we are uncertain about the denominator, we are unable to estimate frequencies. All assignments observed five or more times are listed as WD. The table does not list all alleles from IPD-IMGT/HLA version 3.31.0.

[b] Population groups include: AFA (African/African American), API (Asian/Pacific Islander), EURO (European/European descent), MENA (Middle East/North Coast of Africa), HIS (Hispanic), NAM (Native American) and UNK (unknown, multiple ancestries or other). Total is the overall population i.e., all groups combined. Highest frequency is the highest CIWD designation among all the individual groups.

[c] Not CIWD, o

[d] DRB4*01:03N includes assignments of DRB4*01:03N, DRB4*01:03:01N, and DRB4*01:03:01:02N.

Supplementary Table 7f. CIWD status of observed non-expressed HLA-DQB1 assignments [a]

3.0.0 CIWD Category by Population Group [b]										2.0.0 CWD		EFI CWD	China CWD
HLA-DQB1	Highest Frequency	Total	AFA	API	EURO	MENA	HIS	NAM	UNK	HLA-DQB1 IPD-IMGT/HLA 3.9.0	Category	Category	Category
DQB1*02:18N	WD	WD			WD					DQB1*02:18N		WD	
DQB1*02:20N	WD	WD			WD					DQB1*02:20N		WD	
DQB1*02:58N	o [c]	o								DQB1*02:58N			
DQB1*03:95N	o	o								DQB1*03:95N			
DQB1*03:118N	WD	WD		WD						DQB1*03:118N			
DQB1*04:41N	o	o								DQB1*04:41N			
DQB1*04:36N	o	o								DQB1*04:36N			
DQB1*06:26N	I	WD	WD		WD				I	DQB1*06:26N			
DQB1*06:75N	WD	WD			WD					DQB1*06:75N		WD	
DQB1*06:77N	I	WD		I						DQB1*06:77N			
DQB1*06:144N	WD	WD		WD						DQB1*06:144N			
DQB1*06:158N	o	o								DQB1*06:158N			
DQB1*06:179N	o	o								DQB1*06:179N			

C, common; I, intermediate; WD, well-documented

[a] Null alleles observed at least once in any population group in the current dataset or observed in the earlier catalogs are included in this table. The table does not list all non-expressed alleles from IPD-IMGT/HLA version 3.31.0.

[b] Population groups include: AFA (African/African American), API (Asian/Pacific Islander), EURO (European/European descent), MENA (Middle East/North Coast of Africa), HIS (Hispanic), NAM (Native American) and UNK (unknown, multiple ancestries or other). Total is the overall population i.e., all groups combined. Highest frequency is the highest CIWD designation among all the individual groups.

[c] Not CIWD, o

Supplementary Table 7g. CIWD status of observed non-expressed HLA-DPB1 assignments [a]

3.0.0 CIWD Category by Population Group [b]										2.0.0 CWD		EFI CWD	China CWD
HLA-DPB1	Highest Frequency	Total	AFA	API	EURO	MENA	HIS	NAM	UNK	HLA-DPB1 IPD-IMGT/HLA 3.9.0	Category	Category	Category
DPB1*61:01N	C	WD	C				I		I	DPB1*61:01N			
DPB1*64:01N	I	WD			WD				I	DPB1*64:01N			
DPB1*120:01N	WD	WD			WD					DPB1*120:01N			
DPB1*154:01N	WD	WD			WD					DPB1*154:01N			
DPB1*159:01N	o [c]	o								DPB1*159:01N			
DPB1*161:01N	WD	WD			WD					DPB1*161:01N			
DPB1*216:01N	o	o								DPB1*216:01N			
DPB1*218:01N	WD	WD			WD					DPB1*218:01N			
DPB1*328:01N	o	o								DPB1*328:01N			
DPB1*357:01N	WD	WD		WD						DPB1*357:01N			
DPB1*382:01N	o	o								DPB1*382:01N			
DPB1*507:01N	o	o								DPB1*507:01N			
DPB1*570:01N	WD	WD		WD						DPB1*570:01N			

C, common; I, intermediate; WD, well-documented

[a] Null alleles observed at least once in any population group in the current dataset or observed in the earlier catalogs are included in this table. The table does not list all non-expressed alleles from IPD-IMGT/HLA version 3.31.0.

[b] Population groups include: AFA (African/African American), API (Asian/Pacific Islander), EURO (European/European descent), MENA (Middle East/North Coast of Africa), HIS (Hispanic), NAM (Native American) and UNK (unknown, multiple ancestries or other). Total is the overall population i.e., all groups combined. Highest frequency is the highest CIWD designation among all the individual groups.

[c] Not CIWD, o
