# Supplementary material for: Common, intermediate and well‐documented HLA alleles in world populations: CIWD version 3.0.0
Source: HLA. 2020 Jan 31;95(6):516–31. doi: 10.1111/tan.13811 (PMC7317522; doi:10.1111/tan.13811)
Supplement: Supplementary file 8 — Table S8 HLA‐A primary data [file TAN-95-516-s008.pdf]

Supplemental Table 8: HLA-A Allele Summary [a]				Allele Count by Population Group [b]								3.0.0 CIWD Category by Population Group [c]								
Allele	Genomic Typing	AlleleID	G group	AFA	API	EURO	MENA	HIS	NAM	UNK	Total	AFA	API	EURO	MENA	HIS	NAM	UNK	Total	Highest Frequency
**A*01:01 total**	**01:01 total**			**16351**	**137296**	**1738123**	**42210**	**48018**	**4820**	**161367**	**2148185**	**C**	**C**	**C**	**C**	**C**	**C**	**C**	**C**	**C**
A*01:01	01:01			22	38	7077	44	14	0	397	7592	WD	I	C	C	I		C	C	C
A*01:01P	01:01P			2	8	1650	8	3	0	14	1685		WD	C	WD			I	C	C
**A*01:01:01G total**	**01:01:01G total**			**16324**	**137237**	**1728999**	**42154**	**47996**	**4820**	**160931**	**2138461**	**C**	**C**	**C**	**C**	**C**	**C**	**C**	**C**	**C**
A*01:01:01G	01:01:01G		01:01:01G	13556	130495	1651061	40798	36760	3834	151106	2027610	C	C	C	C	C	C	C	C	C
A*01:01:01	01:01:01		01:01:01G	713	1613	20350	303	2679	215	2446	28319	C	C	C	C	C	C	C	C	C
A*01:01:01:01	01:01:01:01	HLA00001	01:01:01G	2050	4870	57439	1043	8528	765	7341	82036	C	C	C	C	C	C	C	C	C
A*01:01:01:03	01:01:01:03	HLA14798	01:01:01G	4	257	73	10	19	6	24	393		C	WD	WD	I	WD	I	I	C
A*01:01:01:05	01:01:01:05	HLA16415	01:01:01G	0	0	0	0	0	0	1	1									
A*01:01:01:06	01:01:01:06	HLA16417	01:01:01G	0	0	0	0	0	0	1	1									
A*01:01:01:07	01:01:01:07	HLA16436	01:01:01G	0	0	0	0	2	0	1	3									
A*01:01:01:09	01:01:01:09	HLA16652	01:01:01G	0	0	2	0	2	0	0	4									
A*01:01:38L	01:01:38L	HLA03587	01:01:01G	0	0	1	0	0	0	0	1									
A*01:04N	01:04N	HLA00004	01:01:01G	0	0	6	0	0	0	0	6			WD					WD	WD
A*01:22N	01:22N	HLA02878	01:01:01G	0	0	0	0	0	0	1	1									
A*01:32	01:32	HLA03522	01:01:01G	0	0	5	0	0	0	1	6			WD					WD	WD
A*01:37	01:37	HLA03831	01:01:01G	1	0	59	0	6	0	9	75			WD		WD		WD	WD	WD
A*01:81	01:81	HLA05908	01:01:01G	0	0	1	0	0	0	0	1									
A*01:109	01:109	HLA07807	01:01:01G	0	1	1	0	0	0	0	2									
A*01:155	01:155	HLA11389	01:01:01G	0	0	1	0	0	0	0	1									
A*01:177	01:177	HLA13225	01:01:01G	0	1	0	0	0	0	0	1									
A*01:01:02	01:01:02	HLA01244		0	0	19	0	0	0	4	23			WD					WD	WD
A*01:01:03	01:01:03	HLA01971		0	5	139	0	2	0	1	147		WD	I					WD	I
A*01:01:04	01:01:04	HLA02540		0	0	2	0	0	0	0	2									
A*01:01:05	01:01:05	HLA03131		0	4	6	0	0	0	0	10			WD					WD	WD
A*01:01:06	01:01:06	HLA03742		0	0	4	0	0	0	0	4									
A*01:01:09	01:01:09	HLA04425		0	0	4	0	0	0	0	4									
A*01:01:10	01:01:10	HLA04427		0	0	48	0	0	0	1	49			WD					WD	WD
A*01:01:11	01:01:11	HLA04470		0	0	30	0	0	0	5	35			WD				WD	WD	WD
A*01:01:13	01:01:13	HLA04558		0	0	13	0	0	0	4	17			WD					WD	WD

Supplemental Table 8: HLA-A Allele Summary [a]				Allele Count by Population Group [b]								3.0.0 CIWD Category by Population Group [c]								
Allele	Genomic Typing	AlleleID	G group	AFA	API	EURO	MENA	HIS	NAM	UNK	Total	AFA	API	EURO	MENA	HIS	NAM	UNK	Total	Highest Frequency
A*01:01:14	01:01:14	HLA04804		0	0	1	0	0	0	0	1									
A*01:01:16	01:01:16	HLA05319		0	0	23	0	0	0	0	23			WD					WD	WD
A*01:01:17	01:01:17	HLA05326		0	0	1	1	0	0	1	3									
A*01:01:18	01:01:18	HLA05332		0	0	7	0	0	0	0	7			WD					WD	WD
A*01:01:19	01:01:19	HLA05348		0	0	1	0	0	0	0	1									
A*01:01:20	01:01:20	HLA05546		0	0	1	0	0	0	0	1									
A*01:01:21	01:01:21	HLA05662		0	2	4	1	0	0	0	7								WD	WD
A*01:01:22	01:01:22	HLA05684		0	0	7	0	0	0	0	7			WD					WD	WD
A*01:01:23	01:01:23	HLA05685		0	0	1	1	0	0	0	2									
A*01:01:24	01:01:24	HLA05725		0	0	1	0	0	0	0	1									
A*01:01:25	01:01:25	HLA05726		0	0	1	0	0	0	0	1									
A*01:01:26	01:01:26	HLA05907		0	0	3	0	0	0	0	3									
A*01:01:27	01:01:27	HLA05933		0	0	1	0	0	0	0	1									
A*01:01:28	01:01:28	HLA05934		0	0	1	0	0	0	0	1									
A*01:01:29	01:01:29	HLA05968		0	0	12	0	0	0	0	12			WD					WD	WD
A*01:01:30	01:01:30	HLA05988		0	0	0	1	0	0	0	1									
A*01:01:32	01:01:32	HLA06020		0	0	1	0	0	0	0	1									
A*01:01:33	01:01:33	HLA06097		0	0	37	0	0	0	1	38			WD					WD	WD
A*01:01:34	01:01:34	HLA06103		0	1	1	0	3	0	1	6								WD	WD
A*01:01:39	01:01:39	HLA06745		0	0	1	0	0	0	0	1									
A*01:01:41	01:01:41	HLA06790		0	0	1	0	0	0	0	1									
A*01:01:44	01:01:44	HLA07605		0	0	2	0	0	0	0	2									
A*01:01:45	01:01:45	HLA07674		0	0	3	0	0	0	0	3									
A*01:01:47	01:01:47	HLA07994		0	0	8	0	0	0	3	11			WD					WD	WD
A*01:01:52	01:01:52	HLA08966		0	0	4	0	0	0	0	4									
A*01:01:53	01:01:53	HLA08968		3	0	1	0	0	0	0	4									
A*01:01:54	01:01:54	HLA09414		0	0	3	0	0	0	1	4									
A*01:01:55	01:01:55	HLA09415		0	0	2	0	0	0	0	2									
A*01:01:56	01:01:56	HLA09417		0	0	2	0	0	0	0	2									
A*01:01:60	01:01:60	HLA11306		0	1	0	0	0	0	0	1									

Supplemental Table 8: HLA-A Allele Summary [a]				Allele Count by Population Group [b]								3.0.0 CIWD Category by Population Group [c]								
Allele	Genomic Typing	AlleleID	G group	AFA	API	EURO	MENA	HIS	NAM	UNK	Total	AFA	API	EURO	MENA	HIS	NAM	UNK	Total	Highest Frequency
A*01:01:62	01:01:62	HLA11401		0	0	0	0	0	0	1	1									
A*01:01:65	01:01:65	HLA11990		0	0	1	0	0	0	0	1									
A*01:01:66	01:01:66	HLA11991		0	0	2	0	0	0	0	2									
A*01:01:69	01:01:69	HLA12963		0	0	2	0	0	0	1	3									
A*01:01:71	01:01:71	HLA13620		0	0	1	0	0	0	0	1									
A*01:01:72	01:01:72	HLA13705		0	0	1	0	0	0	0	1									
A*01:01:73	01:01:73	HLA14075		0	0	0	0	0	0	1	1									
A*01:01:75	01:01:75	HLA14640		0	0	0	0	0	0	1	1									
A*01:02	01:02	HLA00002		1916	27	1980	187	1356	132	961	6559	C	I	C	C	C	C	C	C	C
**A*01:03 total**	**01:03 total**			**929**	**159**	**2904**	**1304**	**137**	**6**	**1479**	**6918**	**C**	**C**	**C**	**C**	**C**	**WD**	**C**	**C**	**C**
**A*01:03:01G total**	**01:03:01G total**			**929**	**159**	**2904**	**1304**	**137**	**6**	**1479**	**6918**	**C**	**C**	**C**	**C**	**C**	**WD**	**C**	**C**	**C**
A*01:03	01:03			863	138	2730	1261	126	6	1414	6538	C	C	C	C	C	WD	C	C	C
A*01:03:01G	01:03:01G		01:03:01G	28	18	138	10	1	0	12	207	WD	I	I	WD			WD	I	I
A*01:03:01	01:03:01		01:03:01G	28	0	8	14	1	0	50	101	WD		WD	WD			I	WD	I
A*01:03:01:01	01:03:01:01	HLA00003	01:03:01G	7	1	2	13	0	0	0	23	WD			WD				WD	WD
A*01:03:01:02	01:03:01:02	HLA17076	01:03:01G	3	2	26	6	9	0	3	49			WD	WD	I			WD	I
A*01:06	01:06	HLA01031		2	2	730	87	8	0	30	859			I	C	I		I	I	C
A*01:08	01:08	HLA01292		0	0	1	0	0	0	0	1									
**A*01:09 total**	**01:09 total**			**70**	**4**	**7**	**1**	**1**	**0**	**11**	**94**	**C**		**WD**				**WD**	**WD**	**C**
A*01:09	01:09			49	3	6	1	1	0	7	67	C		WD				WD	WD	C
**A*01:09:01G total**	**01:09:01G total**			**21**	**1**	**1**	**0**	**0**	**0**	**4**	**27**	**WD**							**WD**	**WD**
A*01:09:01	01:09:01		01:09:01G	21	1	1	0	0	0	4	27	WD							WD	WD
A*01:12	01:12	HLA02108		0	22	0	0	0	0	2	24		I						WD	I
A*01:13	01:13	HLA02110		0	1	2	0	0	0	0	3									
A*01:14	01:14	HLA02152		0	1	6	0	0	0	0	7			WD					WD	WD
A*01:15N	01:15N	HLA02183		0	0	10	0	0	0	3	13			WD					WD	WD
A*01:16N	01:16N	HLA02310		0	0	23	0	0	0	5	28			WD				WD	WD	WD
A*01:17	01:17	HLA02373		1	0	38	1	3	1	5	49			WD				WD	WD	WD
A*01:18N	01:18N	HLA02484		0	0	1	0	0	0	0	1									
A*01:21	01:21	HLA02857		0	0	1	0	0	0	0	1									

Supplemental Table 8: HLA-A Allele Summary [a]				Allele Count by Population Group [b]								3.0.0 CIWD Category by Population Group [c]								
Allele	Genomic Typing	AlleleID	G group	AFA	API	EURO	MENA	HIS	NAM	UNK	Total	AFA	API	EURO	MENA	HIS	NAM	UNK	Total	Highest Frequency
A*01:23	01:23	HLA02891		9	1	6	17	0	0	7	40	WD		WD	WD			WD	WD	WD
A*01:24	01:24	HLA02895		0	0	3	0	6	0	0	9					WD			WD	WD
A*01:25	01:25	HLA02897		6	2	160	0	1	0	38	207	WD		I				I	I	I
A*01:26	01:26	HLA02920		0	14	25	0	1	1	0	41		I	WD					WD	I
A*01:28	01:28	HLA02985		0	0	2	0	0	0	1	3									
A*01:29	01:29	HLA03029		0	0	9	0	0	0	1	10			WD					WD	WD
A*01:30	01:30	HLA03030		0	0	4	0	1	0	0	5								WD	WD
A*01:31N	01:31N	HLA03501		0	0	3	1	0	0	0	4									
A*01:35	01:35	HLA03688		0	0	3	0	0	0	0	3									
A*01:38	01:38	HLA03877		0	0	27	0	0	0	1	28			WD					WD	WD
A*01:39	01:39	HLA03879		0	0	10	0	0	0	0	10			WD					WD	WD
A*01:40	01:40	HLA03881		0	0	45	0	0	0	1	46			WD					WD	WD
A*01:41	01:41	HLA03882		0	0	2	0	0	0	0	2									
A*01:42	01:42	HLA03898		0	0	12	0	0	0	0	12			WD					WD	WD
A*01:43	01:43	HLA04122		0	24	1	0	0	0	0	25		I						WD	I
A*01:44	01:44	HLA04151		0	0	5	0	0	0	0	5			WD					WD	WD
A*01:46	01:46	HLA04469		0	0	0	4	0	0	5	9							WD	WD	WD
A*01:48	01:48	HLA04494		0	0	0	0	0	0	12	12							WD	WD	WD
A*01:49	01:49	HLA04525		0	0	2	0	0	0	0	2									
A*01:50	01:50	HLA04527		0	0	1	0	0	0	0	1									
A*01:51	01:51	HLA04546		0	0	28	0	0	0	2	30			WD					WD	WD
**A*01:52N total**	**01:52N total**			**1**	**0**	**1**	**0**	**0**	**0**	**1**	**3**									
A*01:52N	01:52N			0	0	0	0	0	0	1	1									
A*01:52:01N	01:52:01N	HLA04761		1	0	1	0	0	0	0	2									
A*01:54	01:54	HLA04808		0	0	1	0	0	0	1	2									
A*01:55	01:55	HLA04811		0	0	1	0	0	0	0	1									
A*01:57N	01:57N	HLA05321		0	0	8	0	1	0	0	9			WD					WD	WD
A*01:58	01:58	HLA05323		0	2	2	0	0	0	0	4									
A*01:59	01:59	HLA05324		0	0	1	0	0	0	0	1									
A*01:60	01:60	HLA05327		0	0	1	0	0	0	0	1									

Supplemental Table 8: HLA-A Allele Summary [a]				Allele Count by Population Group [b]								3.0.0 CIWD Category by Population Group [c]								
Allele	Genomic Typing	AlleleID	G group	AFA	API	EURO	MENA	HIS	NAM	UNK	Total	AFA	API	EURO	MENA	HIS	NAM	UNK	Total	Highest Frequency
A*01:61	01:61	HLA05329		0	0	9	1	3	0	0	13			WD					WD	WD
A*01:66	01:66	HLA05398		0	0	1	0	0	0	0	1									
**A*01:67 total**	**01:67 total**			**0**	**0**	**4**	**0**	**0**	**0**	**5**	**9**							**WD**	**WD**	**WD**
A*01:67	01:67			0	0	2	0	0	0	2	4									
A*01:67:01	01:67:01	HLA05427		0	0	1	0	0	0	3	4									
A*01:67:02	01:67:02	HLA11215		0	0	1	0	0	0	0	1									
A*01:68	01:68	HLA05429		0	0	6	0	0	0	1	7			WD					WD	WD
**A*01:69 total**	**01:69 total**			**0**	**1**	**16**	**0**	**5**	**0**	**3**	**25**			**WD**		**D**			**WD**	**WD**
A*01:69	01:69			0	0	4	0	0	0	0	4									
A*01:69:01	01:69:01	HLA05432		0	1	7	0	0	0	1	9			WD					WD	WD
A*01:69:02	01:69:02	HLA07473		0	0	5	0	5	0	2	12			WD		WD			WD	WD
A*01:72	01:72	HLA05630		0	0	13	0	0	0	0	13			WD					WD	WD
A*01:73	01:73	HLA05635		0	1	0	0	0	0	0	1									
A*01:74	01:74	HLA05648		0	12	0	0	1	0	0	13		WD						WD	WD
A*01:76	01:76	HLA05670		0	0	1	0	0	0	0	1									
A*01:77	01:77	HLA05838		2	0	2	0	0	0	0	4									
A*01:78	01:78	HLA05894		0	1	0	0	0	0	0	1									
A*01:80	01:80	HLA05906		0	0	0	0	0	0	1	1									
A*01:82	01:82	HLA05932		2	27	2	0	0	0	0	31		I						WD	I
**A*01:83 total**	**01:83 total**			**0**	**0**	**2**	**0**	**0**	**0**	**0**	**2**									
A*01:83:02	01:83:02	HLA08640		0	0	2	0	0	0	0	2									
A*01:86	01:86	HLA06341		0	0	6	3	1	0	11	21			WD				WD	WD	WD
A*01:89	01:89	HLA06558		0	0	4	0	0	0	0	4									
A*01:90	01:90	HLA06561		0	0	1	0	0	0	0	1									
A*01:91	01:91	HLA06569		0	3	1	0	0	0	0	4									
A*01:92	01:92	HLA06570		0	0	5	0	0	0	0	5			WD					WD	WD
A*01:93	01:93	HLA06571		0	0	1	0	0	0	0	1									
A*01:94	01:94	HLA06764		0	0	1	0	0	0	0	1									
A*01:95	01:95	HLA06765		0	0	2	0	0	0	0	2									
A*01:98	01:98	HLA06800		0	2	43	0	0	0	0	45			WD					WD	WD

Supplemental Table 8: HLA-A Allele Summary [a]				Allele Count by Population Group [b]								3.0.0 CIWD Category by Population Group [c]								
Allele	Genomic Typing	AlleleID	G group	AFA	API	EURO	MENA	HIS	NAM	UNK	Total	AFA	API	EURO	MENA	HIS	NAM	UNK	Total	Highest Frequency
A*01:99	01:99	HLA06801		0	0	1	0	0	0	0	1									
A*01:100	01:100	HLA06802		0	1	22	0	1	0	0	24			WD					WD	WD
A*01:101	01:101	HLA06813		0	0	1	0	0	0	1	2									
A*01:104	01:104	HLA07404		0	9	4	0	3	0	4	20		WD						WD	WD
A*01:106	01:106	HLA07597		1	0	2	1	0	0	0	4									
A*01:111	01:111	HLA07991		0	0	1	0	0	0	0	1									
A*01:112	01:112	HLA07993		0	0	1	0	0	0	0	1									
A*01:113	01:113	HLA08248		0	0	12	0	0	0	0	12			WD					WD	WD
A*01:115	01:115	HLA08445		0	0	3	0	1	0	0	4									
A*01:117	01:117	HLA08447		0	0	3	0	0	0	0	3									
A*01:118	01:118	HLA08460		0	0	1	2	0	0	0	3									
A*01:123N	01:123N	HLA08895		0	0	7	0	0	0	0	7			WD					WD	WD
A*01:124	01:124	HLA08920		0	0	2	0	0	0	0	2									
A*01:126	01:126	HLA08967		0	3	3	0	0	0	0	6								WD	WD
A*01:133	01:133	HLA09538		0	0	2	0	0	0	0	2									
A*01:134	01:134	HLA09799		0	0	1	0	0	0	0	1									
A*01:136	01:136	HLA09801		0	0	1	0	0	0	0	1									
A*01:137	01:137	HLA09802		0	0	1	0	0	0	0	1									
A*01:139	01:139	HLA10123		0	0	1	0	0	0	0	1									
A*01:143	01:143	HLA10372		0	0	4	0	0	0	0	4									
A*01:144	01:144	HLA10385		0	3	0	0	0	0	0	3									
A*01:145	01:145	HLA10386		0	0	0	0	0	0	1	1									
A*01:147Q	01:147Q	HLA10914		0	0	1	0	0	0	0	1									
A*01:148	01:148	HLA10978		0	0	1	0	0	0	0	1									
A*01:149	01:149	HLA10979		0	0	2	0	0	0	0	2									
A*01:150	01:150	HLA10980		0	2	0	0	0	0	1	3									
A*01:154	01:154	HLA11400		0	0	1	0	0	0	0	1									
A*01:156	01:156	HLA11799		0	0	4	0	0	0	1	5								WD	WD
A*01:157	01:157	HLA12078		0	0	1	0	0	0	0	1									
A*01:159	01:159	HLA12112		0	0	0	0	0	1	0	1									

Supplemental Table 8: HLA-A Allele Summary [a]				Allele Count by Population Group [b]								3.0.0 CIWD Category by Population Group [c]								
Allele	Genomic Typing	AlleleID	G group	AFA	API	EURO	MENA	HIS	NAM	UNK	Total	AFA	API	EURO	MENA	HIS	NAM	UNK	Total	Highest Frequency
A*01:160N	01:160N	HLA12113		0	0	0	1	0	0	0	1									
A*01:162N	01:162N	HLA12248		0	0	2	0	0	0	0	2									
A*01:163	01:163	HLA12252		0	0	0	0	0	0	1	1									
A*01:167	01:167	HLA12725		0	0	3	0	0	0	0	3									
A*01:169	01:169	HLA12638		0	2	0	0	0	0	0	2									
A*01:170	01:170	HLA12861		0	0	2	0	0	0	0	2									
A*01:171	01:171	HLA12959		0	1	1	0	0	0	0	2									
A*01:172	01:172	HLA12960		0	0	0	0	1	0	0	1									
A*01:173	01:173	HLA12961		0	0	3	0	0	0	0	3									
A*01:175	01:175	HLA12971		0	0	0	0	1	0	0	1									
A*01:180	01:180	HLA13445		0	0	1	0	0	0	0	1									
A*01:182	01:182	HLA13447		0	0	0	0	0	0	1	1									
A*01:183	01:183	HLA13448		0	0	0	0	1	0	0	1									
A*01:184	01:184	HLA13449		0	0	1	0	0	0	0	1									
A*01:185	01:185	HLA13450		0	0	0	0	0	0	1	1									
A*01:188	01:188	HLA13727		0	3	1	0	0	0	0	4									
A*01:189	01:189	HLA13729		0	0	0	1	0	0	0	1									
A*01:190	01:190	HLA13841		0	0	6	0	0	0	0	6			WD					WD	WD
A*01:191	01:191	HLA13845		0	0	2	0	0	0	0	2									
A*01:193	01:193	HLA13954		0	1	0	0	0	0	0	1									
A*01:195	01:195	HLA13976		0	1	1	0	0	0	0	2									
A*01:196	01:196	HLA14106		0	1	0	0	0	0	0	1									
A*01:197	01:197	HLA14283		0	2	0	0	0	0	0	2									
A*01:199	01:199	HLA14462		0	0	0	1	0	0	0	1									
A*01:202	01:202	HLA14916		0	0	1	0	0	0	0	1									
A*01:205	01:205	HLA15247		0	0	1	0	0	0	0	1									
A*01:208Q	01:208Q	HLA15158		0	0	2	0	0	0	0	2									
A*01:209	01:209	HLA15459		0	1	0	0	0	0	0	1									
A*01:221	01:221	HLA16109		0	0	1	0	0	0	0	1									
A*01:223	01:223	HLA16113		0	0	1	0	0	0	0	1									

Supplemental Table 8: HLA-A Allele Summary [a]				Allele Count by Population Group [b]								3.0.0 CIWD Category by Population Group [c]								
Allele	Genomic Typing	AlleleID	G group	AFA	API	EURO	MENA	HIS	NAM	UNK	Total	AFA	API	EURO	MENA	HIS	NAM	UNK	Total	Highest Frequency
A*01:231	01:231	HLA16457		0	1	0	0	0	0	0	1									
A*01:CODE [d]	01:CODE			1229	1573	86859	1309	4893	356	9902	106121	NA	NA	NA	NA	NA	NA	NA	NA	NA
**A*02:01 total**	**02:01 total**			**46155**	**84012**	**3234060**	**69830**	**139179**	**13017**	**288078**	**3874331**	**C**	**C**	**C**	**C**	**C**	**C**	**C**	**C**	**C**
A*02:01	02:01			14	8	12092	197	120	4	604	13039	WD	WD	C	C	C		C	C	C
A*02:01P	02:01P			7	6	4316	11	19	0	15	4374	WD	WD	C	WD	I		I	C	C
**A*02:01:01G total**	**02:01:01G total**			**46087**	**83838**	**3215327**	**69137**	**138693**	**12977**	**286747**	**3852806**	**C**	**C**	**C**	**C**	**C**	**C**	**C**	**C**	**C**
A*02:01:01G	02:01:01G		02:01:01G	41324	78913	3119230	68382	118165	11167	273798	3710979	C	C	C	C	C	C	C	C	C
A*02:01:01	02:01:01		02:01:01G	4731	4744	93981	726	20132	1797	12546	138657	C	C	C	C	C	C	C	C	C
A*02:01:01:01	02:01:01:01	HLA00005	02:01:01G	2	0	925	0	103	0	264	1294			I		C		C	I	C
A*02:01:01:02L	02:01:01:02L	HLA01785	02:01:01G	0	0	11	0	0	0	1	12			WD					WD	WD
A*02:01:01:03	02:01:01:03	HLA03253	02:01:01G	0	0	3	0	0	0	0	3									
A*02:01:01:05	02:01:01:05	HLA13775	02:01:01G	6	2	445	4	104	6	49	616	WD		I		C	WD	I	I	C
A*02:01:01:06	02:01:01:06	HLA14066	02:01:01G	0	0	8	1	0	0	1	10			WD					WD	WD
A*02:01:01:07	02:01:01:07	HLA15498	02:01:01G	0	0	1	0	0	0	0	1									
A*02:01:01:08	02:01:01:08	HLA15499	02:01:01G	5	4	422	8	36	1	37	513	WD		I	WD	I		I	I	I
A*02:01:01:10	02:01:01:10	HLA15762	02:01:01G	0	0	3	0	0	0	0	3									
A*02:01:01:11	02:01:01:11	HLA16397	02:01:01G	7	0	3	0	0	0	5	15	WD						WD	WD	WD
A*02:01:01:12	02:01:01:12	HLA16398	02:01:01G	0	0	0	0	0	0	1	1									
A*02:01:01:13	02:01:01:13	HLA16400	02:01:01G	0	1	0	0	0	0	0	1									
A*02:01:01:14	02:01:01:14	HLA16402	02:01:01G	0	0	0	0	0	0	1	1									
A*02:01:01:15	02:01:01:15	HLA16413	02:01:01G	0	0	0	0	0	0	1	1									
A*02:01:01:16	02:01:01:16	HLA16430	02:01:01G	0	0	2	0	0	0	0	2									
A*02:01:01:17	02:01:01:17	HLA16431	02:01:01G	0	0	1	0	0	0	0	1									
A*02:01:01:18	02:01:01:18	HLA16432	02:01:01G	0	0	63	0	8	0	6	77			WD		I		WD	WD	I
A*02:01:01:19	02:01:01:19	HLA16654	02:01:01G	1	0	3	0	0	0	0	4									
A*02:01:01:20	02:01:01:20	HLA16655	02:01:01G	0	0	3	0	0	0	1	4									
A*02:01:01:21	02:01:01:21	HLA16656	02:01:01G	0	0	2	0	1	0	1	4									
A*02:01:01:22	02:01:01:22	HLA16657	02:01:01G	0	0	10	0	0	0	1	11			WD					WD	WD
A*02:01:01:23	02:01:01:23	HLA16658	02:01:01G	0	0	0	1	0	0	0	1									
A*02:01:08	02:01:08	HLA01664	02:01:01G	1	6	1	0	0	0	1	9		WD						WD	WD

Supplemental Table 8: HLA-A Allele Summary [a]				Allele Count by Population Group [b]								3.0.0 CIWD Category by Population Group [c]								
Allele	Genomic Typing	AlleleID	G group	AFA	API	EURO	MENA	HIS	NAM	UNK	Total	AFA	API	EURO	MENA	HIS	NAM	UNK	Total	Highest Frequency
A*02:01:11	02:01:11	HLA02000	02:01:01G	0	0	0	1	0	0	0	1									
A*02:01:14Q	02:01:14Q	HLA02908	02:01:01G	1	0	22	0	3	1	4	31			WD					WD	WD
A*02:01:15	02:01:15	HLA02914	02:01:01G	0	0	1	0	7	0	1	9					WD			WD	WD
A*02:01:21	02:01:21	HLA03524	02:01:01G	0	0	3	0	0	0	0	3									
A*02:01:79	02:01:79	HLA08056	02:01:01G	0	1	0	0	0	0	0	1									
A*02:01:89	02:01:89	HLA09686	02:01:01G	0	0	0	0	0	1	0	1									
A*02:01:130	02:01:130	HLA16647	02:01:01G	0	0	2	0	0	0	0	2									
A*02:09	02:09	HLA00014	02:01:01G	5	166	97	13	49	2	14	346	WD	C	WD	WD	I		I	I	C
A*02:66	02:66	HLA01781	02:01:01G	2	0	65	0	4	0	5	76			WD				WD	WD	WD
A*02:83N	02:83N	HLA02202	02:01:01G	0	0	5	0	0	0	3	8			WD					WD	WD
A*02:97:01	02:97:01	HLA02509	02:01:01G	0	0	2	0	0	0	1	3									
A*02:140	02:140	HLA03241	02:01:01G	0	0	3	0	0	0	0	3									
A*02:241	02:241	HLA04890	02:01:01G	0	0	5	0	1	0	1	7			WD					WD	WD
A*02:294	02:294	HLA06081	02:01:01G	0	0	0	0	1	0	1	2									
A*02:327	02:327	HLA07104	02:01:01G	0	0	2	0	0	0	0	2									
A*02:397	02:397	HLA08896	02:01:01G	0	0	0	0	3	0	1	4									
A*02:481	02:481	HLA10909	02:01:01G	1	0	0	0	10	0	1	12					I			WD	I
A*02:538	02:538	HLA12179	02:01:01G	0	0	0	1	0	0	0	1									
A*02:629	02:629	HLA14950	02:01:01G	1	0	2	0	0	0	0	3									
A*02:642	02:642	HLA15568	02:01:01G	0	1	0	0	66	2	1	70					I			WD	I
A*02:665	02:665	HLA16406	02:01:01G	0	0	1	0	0	0	0	1									
A*02:01:02	02:01:02	HLA00006		8	48	132	14	8	0	21	231	WD	I	I	WD	I		I	I	I
A*02:01:03	02:01:03	HLA00966		0	0	1	0	0	0	0	1									
A*02:01:04	02:01:04	HLA01032		30	100	790	392	280	33	413	2038	WD	I	I	C	C	C	C	C	C
A*02:01:05	02:01:05	HLA01327		1	4	428	25	21	0	204	683			I	WD	I		C	I	C
A*02:01:09	02:01:09	HLA01682		1	1	252	11	4	0	17	286			I	WD			I	I	I
A*02:01:12	02:01:12	HLA02312		0	0	12	0	0	0	0	12			WD					WD	WD
A*02:01:13	02:01:13	HLA02854		0	0	2	0	0	0	0	2									
A*02:01:18	02:01:18	HLA03199		0	0	76	9	3	0	5	93			WD	WD			WD	WD	WD
A*02:01:19	02:01:19	HLA03218		0	0	1	0	0	0	1	2									

Supplemental Table 8: HLA-A Allele Summary [a]				Allele Count by Population Group [b]								3.0.0 CIWD Category by Population Group [c]								
Allele	Genomic Typing	AlleleID	G group	AFA	API	EURO	MENA	HIS	NAM	UNK	Total	AFA	API	EURO	MENA	HIS	NAM	UNK	Total	Highest Frequency
A*02:01:22	02:01:22	HLA03534		0	0	52	21	0	0	6	79			WD	WD			WD	WD	WD
A*02:01:23	02:01:23	HLA03787		0	0	118	3	0	0	0	121			WD					WD	WD
A*02:01:24	02:01:24	HLA03790		0	0	16	0	2	0	0	18			WD					WD	WD
A*02:01:25	02:01:25	HLA03792		0	0	10	0	0	0	0	10			WD					WD	WD
A*02:01:26	02:01:26	HLA03794		0	0	19	0	0	2	1	22			WD					WD	WD
A*02:01:27	02:01:27	HLA03798		0	0	18	0	0	0	0	18			WD					WD	WD
A*02:01:28	02:01:28	HLA03883		0	0	1	0	0	0	0	1									
A*02:01:29	02:01:29	HLA03887		0	0	19	0	0	0	1	20			WD					WD	WD
A*02:01:30	02:01:30	HLA03889		1	0	28	0	2	0	1	32			WD					WD	WD
A*02:01:31	02:01:31	HLA03897		5	0	36	0	0	0	12	53	WD		WD				WD	WD	WD
A*02:01:32	02:01:32	HLA03943		0	0	17	0	0	0	0	17			WD					WD	WD
A*02:01:33	02:01:33	HLA04421		0	0	3	0	0	0	0	3									
A*02:01:34	02:01:34	HLA04426		0	1	84	1	0	0	0	86			WD					WD	WD
A*02:01:35	02:01:35	HLA04428		1	0	3	0	1	0	0	5								WD	WD
A*02:01:36	02:01:36	HLA04431		0	0	3	1	0	0	0	4									
A*02:01:37	02:01:37	HLA04477		0	0	34	3	1	1	0	39			WD					WD	WD
A*02:01:38	02:01:38	HLA04479		0	0	21	0	1	0	4	26			WD					WD	WD
A*02:01:39	02:01:39	HLA04480		0	0	5	0	1	0	4	10			WD					WD	WD
A*02:01:40	02:01:40	HLA04482		0	1	0	0	0	0	0	1									
A*02:01:41	02:01:41	HLA04484		0	0	13	0	0	0	1	14			WD					WD	WD
A*02:01:42	02:01:42	HLA04557		0	0	5	0	0	0	0	5			WD					WD	WD
A*02:01:43	02:01:43	HLA04674		0	1	12	0	0	0	2	15			WD					WD	WD
A*02:01:45	02:01:45	HLA04814		0	0	3	0	0	0	0	3									
A*02:01:46	02:01:46	HLA05140		0	0	5	0	1	0	1	7			WD					WD	WD
A*02:01:49	02:01:49	HLA05243		0	0	12	0	0	0	0	12			WD					WD	WD
A*02:01:51	02:01:51	HLA05654		0	0	1	0	0	0	0	1									
A*02:01:52	02:01:52	HLA05668		0	0	18	0	1	0	1	20			WD					WD	WD
A*02:01:53	02:01:53	HLA05693		0	0	2	0	0	0	0	2									
A*02:01:54	02:01:54	HLA05698		0	0	2	0	10	0	5	17					I		WD	WD	I
A*02:01:55	02:01:55	HLA06090		0	0	3	0	0	0	0	3									

Supplemental Table 8: HLA-A Allele Summary [a]				Allele Count by Population Group [b]								3.0.0 CIWD Category by Population Group [c]								
Allele	Genomic Typing	AlleleID	G group	AFA	API	EURO	MENA	HIS	NAM	UNK	Total	AFA	API	EURO	MENA	HIS	NAM	UNK	Total	Highest Frequency
A*02:01:57	02:01:57	HLA06567		0	0	2	0	0	0	0	2									
A*02:01:58	02:01:58	HLA06573		0	0	3	0	0	0	0	3									
A*02:01:59	02:01:59	HLA06657		0	0	3	0	0	0	0	3									
A*02:01:60	02:01:60	HLA06661		0	0	3	0	0	0	0	3									
A*02:01:61	02:01:61	HLA06768		0	0	1	0	0	0	3	4									
A*02:01:62	02:01:62	HLA06779		0	0	0	1	0	0	1	2									
A*02:01:64	02:01:64	HLA07283		0	0	3	0	0	0	0	3									
A*02:01:65	02:01:65	HLA07436		0	1	4	0	0	0	0	5								WD	WD
A*02:01:66	02:01:66	HLA07438		0	0	2	0	0	0	0	2									
A*02:01:69	02:01:69	HLA07616		0	0	3	0	0	0	0	3									
A*02:01:70	02:01:70	HLA07617		0	0	1	0	0	0	0	1									
A*02:01:71	02:01:71	HLA07620		0	0	9	0	0	0	0	9			WD					WD	WD
A*02:01:72	02:01:72	HLA07753		0	0	1	0	0	0	0	1									
A*02:01:74	02:01:74	HLA08006		0	0	5	0	0	0	0	5			WD					WD	WD
A*02:01:75	02:01:75	HLA08008		0	0	1	0	0	0	0	1									
A*02:01:76	02:01:76	HLA08014		0	0	4	0	0	0	0	4									
A*02:01:77	02:01:77	HLA08015		0	0	1	0	0	0	0	1									
A*02:01:83	02:01:83	HLA08465		0	0	1	0	0	0	1	2									
A*02:01:84	02:01:84	HLA08497		0	0	1	0	0	0	0	1									
A*02:01:86	02:01:86	HLA08652		0	0	5	0	0	0	1	6			WD					WD	WD
A*02:01:87	02:01:87	HLA09549		0	1	1	0	1	0	0	3									
A*02:01:88	02:01:88	HLA09552		0	0	0	0	2	0	2	4									
A*02:01:90	02:01:90	HLA09810		0	0	1	0	5	0	1	7					WD			WD	WD
A*02:01:92	02:01:92	HLA09813		0	0	1	0	0	0	0	1									
A*02:01:93	02:01:93	HLA09814		0	0	3	1	0	0	0	4									
A*02:01:94	02:01:94	HLA09816		0	0	1	0	0	0	0	1									
A*02:01:100	02:01:100	HLA10396		0	0	2	0	0	0	0	2									
A*02:01:101	02:01:101	HLA10778		0	0	0	0	0	0	1	1									
A*02:01:102	02:01:102	HLA10780		0	0	0	0	0	0	1	1									
A*02:01:103	02:01:103	HLA10782		0	1	0	0	0	0	0	1									

Supplemental Table 8: HLA-A Allele Summary [a]				Allele Count by Population Group [b]								3.0.0 CIWD Category by Population Group [c]								
Allele	Genomic Typing	AlleleID	G group	AFA	API	EURO	MENA	HIS	NAM	UNK	Total	AFA	API	EURO	MENA	HIS	NAM	UNK	Total	Highest Frequency
A*02:01:108	02:01:108	HLA11996		0	0	0	0	0	0	1	1									
A*02:01:111	02:01:111	HLA12117		0	0	2	0	0	0	0	2									
A*02:01:112	02:01:112	HLA12121		0	0	2	0	0	0	0	2									
A*02:01:113	02:01:113	HLA12255		0	0	2	0	0	0	0	2									
A*02:01:114	02:01:114	HLA12256		0	0	1	0	1	0	0	2									
A*02:01:115	02:01:115	HLA12259		0	0	0	0	1	0	0	1									
A*02:01:116	02:01:116	HLA13081		0	0	3	0	0	0	2	5								WD	WD
A*02:01:119	02:01:119	HLA13319		0	0	0	0	1	0	0	1									
A*02:01:120	02:01:120	HLA13462		0	0	0	3	0	0	1	4									
A*02:01:121	02:01:121	HLA14225		0	1	0	0	0	0	0	1									
A*02:01:126	02:01:126	HLA15853		0	0	1	0	0	0	0	1									
**A*02:02 total**	**02:02 total**			**15622**	**322**	**9230**	**2193**	**4335**	**685**	**6070**	**38457**	**C**	**C**	**C**	**C**	**C**	**C**	**C**	**C**	**C**
A*02:02	02:02			8893	136	5051	898	2263	402	3108	20751	C	C	C	C	C	C	C	C	C
A*02:02P	02:02P			0	0	10	0	2	0	0	12			WD					WD	WD
**A*02:02:01G total**	**02:02:01G total**			**6729**	**186**	**4169**	**1295**	**2069**	**283**	**2962**	**17693**	**C**	**C**	**C**	**C**	**C**	**C**	**C**	**C**	**C**
A*02:02:01G	02:02:01G		02:02:01G	1296	114	2160	224	390	2	836	5022	C	I	C	C	C		C	C	C
A*02:02:01	02:02:01		02:02:01G	4156	67	1763	1035	1258	208	1787	10274	C	I	C	C	C	C	C	C	C
A*02:02:01:01	02:02:01:01	HLA00007	02:02:01G	891	5	200	29	293	60	244	1722	C	WD	I	WD	C	C	C	C	C
A*02:02:01:02	02:02:01:02	HLA13987	02:02:01G	386	0	46	7	128	13	95	675	C		WD	WD	C	C	I	I	C
A*02:02:03	02:02:03	HLA13091		0	0	0	0	1	0	0	1									
**A*02:03 total**	**02:03 total**			**78**	**25743**	**499**	**119**	**80**	**45**	**1512**	**28076**	**C**	**C**	**I**	**C**	**C**	**C**	**C**	**C**	**C**
A*02:03	02:03			2	14	5	0	0	0	2	23		I	WD					WD	I
A*02:03P	02:03P			0	1	1	0	0	0	0	2									
**A*02:03:01G total**	**02:03:01G total**			**76**	**25700**	**493**	**119**	**80**	**45**	**1509**	**28022**	**C**	**C**	**I**	**C**	**C**	**C**	**C**	**C**	**C**
A*02:03:01G	02:03:01G		02:03:01G	66	21039	474	109	59	33	1077	22857	C	C	I	C	I	C	C	C	C
A*02:03:01	02:03:01	HLA00008	02:03:01G	10	4660	19	10	21	12	432	5164	WD	C	WD	WD	I	C	C	C	C
A*02:264	02:264	HLA05420	02:03:01G	0	1	0	0	0	0	0	1									
A*02:03:02	02:03:02	HLA01945		0	26	0	0	0	0	1	27		I						WD	I
A*02:03:03	02:03:03	HLA05409		0	1	0	0	0	0	0	1									
A*02:03:04	02:03:04	HLA06091		0	1	0	0	0	0	0	1									

Supplemental Table 8: HLA-A Allele Summary [a]				Allele Count by Population Group [b]								3.0.0 CIWD Category by Population Group [c]								
Allele	Genomic Typing	AlleleID	G group	AFA	API	EURO	MENA	HIS	NAM	UNK	Total	AFA	API	EURO	MENA	HIS	NAM	UNK	Total	Highest Frequency
**A*02:04 total**	**02:04 total**			**141**	**11**	**305**	**6**	**1690**	**370**	**483**	**3006**	**C**	**WD**	**I**	**WD**	**C**	**C**	**C**	**C**	**C**
**A*02:04:01G total**	**02:04:01G total**			**141**	**11**	**305**	**6**	**1690**	**370**	**483**	**3006**	**C**	**WD**	**I**	**WD**	**C**	**C**	**C**	**C**	**C**
A*02:04	02:04	HLA00009	02:04:01G	137	11	276	6	1638	370	423	2861	C	WD	I	WD	C	C	C	C	C
A*02:04:01G	02:04:01G		02:04:01G	4	0	29	0	52	0	58	143			WD		I		I	WD	I
A*02:664	02:664	HLA16399	02:04:01G	0	0	0	0	0	0	2	2									
**A*02:05 total**	**02:05 total**			**6312**	**6944**	**89735**	**10216**	**9237**	**921**	**17916**	**141281**	**C**	**C**	**C**	**C**	**C**	**C**	**C**	**C**	**C**
A*02:05	02:05			1	0	866	14	1	0	98	980			I	WD			I	I	I
A*02:05P	02:05P			0	0	189	1	2	0	0	192			I					I	I
**A*02:05:01G total**	**02:05:01G total**			**6310**	**6940**	**88591**	**10194**	**9233**	**921**	**17817**	**140006**	**C**	**C**	**C**	**C**	**C**	**C**	**C**	**C**	**C**
A*02:05:01G	02:05:01G		02:05:01G	5045	6349	83021	9854	6502	655	16551	127977	C	C	C	C	C	C	C	C	C
A*02:05:01	02:05:01		02:05:01G	1196	571	5262	327	2615	261	1216	11448	C	C	C	C	C	C	C	C	C
A*02:05:01:01	02:05:01:01	HLA00010	02:05:01G	53	19	303	13	115	5	49	557	C	I	I	WD	C	WD	I	I	C
A*02:179	02:179	HLA03833	02:05:01G	16	1	1	0	1	0	1	20	WD							WD	WD
A*02:324	02:324	HLA06900	02:05:01G	0	0	4	0	0	0	0	4									
A*02:05:02	02:05:02	HLA03656		0	0	88	0	1	0	1	90			WD					WD	WD
A*02:05:03	02:05:03	HLA03896		1	1	0	7	0	0	0	9				WD				WD	WD
A*02:05:05	02:05:05	HLA06105		0	3	1	0	0	0	0	4									
**A*02:06 total**	**02:06 total**			**495**	**31484**	**23148**	**1832**	**23754**	**1719**	**9125**	**91557**	**C**	**C**	**C**	**C**	**C**	**C**	**C**	**C**	**C**
A*02:06	02:06			0	2	94	0	12	2	7	117			WD		I		WD	WD	I
A*02:06P	02:06P			0	2	12	0	0	0	0	14			WD					WD	WD
**A*02:06:01G total**	**02:06:01G total**			**495**	**31473**	**23023**	**1832**	**23696**	**1712**	**9101**	**91332**	**C**	**C**	**C**	**C**	**C**	**C**	**C**	**C**	**C**
A*02:06:01G	02:06:01G		02:06:01G	351	26770	21846	1776	15858	1231	7246	75078	C	C	C	C	C	C	C	C	C
A*02:06:01	02:06:01		02:06:01G	96	3231	730	36	5378	314	1397	11182	C	C	I	WD	C	C	C	C	C
A*02:06:01:01	02:06:01:01	HLA00011	02:06:01G	48	1436	439	18	2460	167	449	5017	C	C	I	WD	C	C	C	C	C
A*02:06:01:02	02:06:01:02	HLA13485	02:06:01G	0	9	0	0	0	0	0	9		WD						WD	WD
A*02:06:01:03	02:06:01:03	HLA14087	02:06:01G	0	0	7	0	0	0	1	8			WD					WD	WD
A*02:06:01:04	02:06:01:04	HLA17065	02:06:01G	0	27	1	2	0	0	8	38		I					WD	WD	I
A*02:06:04	02:06:04	HLA02919		0	0	0	0	1	0	0	1									
A*02:06:05	02:06:05	HLA03026		0	0	3	0	31	4	13	51					I		WD	WD	I
A*02:06:07	02:06:07	HLA04062		0	0	11	0	0	0	1	12			WD					WD	WD

Supplemental Table 8: HLA-A Allele Summary [a]				Allele Count by Population Group [b]								3.0.0 CIWD Category by Population Group [c]								
Allele	Genomic Typing	AlleleID	G group	AFA	API	EURO	MENA	HIS	NAM	UNK	Total	AFA	API	EURO	MENA	HIS	NAM	UNK	Total	Highest Frequency
A*02:06:08	02:06:08	HLA04478		0	0	3	0	11	1	2	17					I			WD	I
A*02:06:09	02:06:09	HLA04550		0	0	0	0	2	0	0	2									
A*02:06:10	02:06:10	HLA05640		0	1	0	0	0	0	0	1									
A*02:06:11	02:06:11	HLA07608		0	5	0	0	0	0	0	5		WD						WD	WD
A*02:06:12	02:06:12	HLA08003		0	0	0	0	1	0	0	1									
A*02:06:17	02:06:17	HLA12114		0	0	0	0	0	0	1	1									
A*02:06:18	02:06:18	HLA12558		0	0	1	0	0	0	0	1									
A*02:06:20	02:06:20	HLA13456		0	1	0	0	0	0	0	1									
A*02:06:22	02:06:22	HLA14393		0	0	1	0	0	0	0	1									
**A*02:07 total**	**02:07 total**			**37**	**23911**	**2363**	**485**	**114**	**9**	**2523**	**29442**	**WD**	**C**	**C**	**C**	**C**	**C**	**C**	**C**	**C**
A*02:07	02:07			0	62	14	0	0	0	19	95		I	WD				I	WD	I
A*02:07P	02:07P			0	0	8	0	0	0	0	8			WD					WD	WD
**A*02:07:01G total**	**02:07:01G total**			**37**	**23849**	**2341**	**485**	**114**	**9**	**2504**	**29339**	**WD**	**C**	**C**	**C**	**C**	**C**	**C**	**C**	**C**
A*02:07:01G	02:07:01G		02:07:01G	27	18473	2294	473	86	6	1793	23152	WD	C	C	C	C	WD	C	C	C
A*02:07:01	02:07:01	HLA00012	02:07:01G	10	5375	47	12	28	3	711	6186	WD	C	WD	WD	I		C	C	C
A*02:07:02	02:07:02	HLA07326	02:07:01G	0	1	0	0	0	0	0	1									
A*02:08	02:08	HLA00013		7	278	1560	203	12	1	87	2148	WD	C	C	C	I		I	C	C
**A*02:10 total**	**02:10 total**			**1**	**350**	**8**	**1**	**0**	**0**	**57**	**417**		**C**	**WD**				**I**	**I**	**C**
**A*02:10:01G total**	**02:10:01G total**			**1**	**350**	**8**	**1**	**0**	**0**	**57**	**417**		**C**	**WD**				**I**	**I**	**C**
A*02:10	02:10	HLA00015	02:10:01G	0	146	1	0	0	0	27	174		C					I	I	C
A*02:10:01G	02:10:01G		02:10:01G	1	204	7	1	0	0	30	243		C	WD				I	I	C
**A*02:11 total**	**02:11 total**			**280**	**66094**	**1770**	**1299**	**2576**	**491**	**2085**	**74595**	**C**	**C**	**C**	**C**	**C**	**C**	**C**	**C**	**C**
A*02:11	02:11			0	4	29	0	5	0	1	39			WD		WD			WD	WD
A*02:11P	02:11P			0	0	3	0	1	0	0	4									
**A*02:11:01G total**	**02:11:01G total**			**280**	**66050**	**1738**	**1299**	**2570**	**491**	**2083**	**74511**	**C**	**C**	**C**	**C**	**C**	**C**	**C**	**C**	**C**
A*02:11:01G	02:11:01G		02:11:01G	215	63306	1688	1247	1988	346	1829	70619	C	C	C	C	C	C	C	C	C
A*02:11:01	02:11:01	HLA00016	02:11:01G	65	2744	50	52	580	145	254	3890	C	C	WD	C	C	C	C	C	C
A*02:69	02:69	HLA01903	02:11:01G	0	0	0	0	2	0	0	2									
A*02:11:02	02:11:02	HLA05644		0	6	0	0	0	0	0	6		WD						WD	WD
A*02:11:04	02:11:04	HLA06089		0	6	0	0	0	0	0	6		WD						WD	WD

Supplemental Table 8: HLA-A Allele Summary [a]				Allele Count by Population Group [b]								3.0.0 CIWD Category by Population Group [c]								
Allele	Genomic Typing	AlleleID	G group	AFA	API	EURO	MENA	HIS	NAM	UNK	Total	AFA	API	EURO	MENA	HIS	NAM	UNK	Total	Highest Frequency
A*02:11:05	02:11:05	HLA09880		0	1	0	0	0	0	0	1									
A*02:11:06	02:11:06	HLA10989		0	26	0	0	0	0	0	26		I						WD	I
A*02:11:09	02:11:09	HLA13461		0	1	0	0	0	0	1	2									
A*02:12	02:12	HLA00017		1	20	276	36	50	1	91	475		I	I	WD	I		I	I	I
A*02:13	02:13	HLA00018		12	10	363	1	721	49	170	1326	WD	WD	I		C	C	C	I	C
A*02:14	02:14	HLA00019		178	1	51	84	22	0	43	379	C		WD	C	I		I	I	C
**A*02:16 total**	**02:16 total**			**12**	**4938**	**38**	**23**	**4**	**4**	**69**	**5088**	**WD**	**C**	**WD**	**WD**			**I**	**C**	**C**
**A*02:16:01G total**	**02:16:01G total**			**12**	**4938**	**38**	**23**	**4**	**4**	**69**	**5088**	**WD**	**C**	**WD**	**WD**			**I**	**C**	**C**
A*02:16	02:16	HLA00021	02:16:01G	0	2	0	0	0	0	1	3									
A*02:16:01G	02:16:01G		02:16:01G	10	4811	36	23	3	3	60	4946	WD	C	WD	WD			I	C	C
A*02:131	02:131	HLA03096	02:16:01G	2	125	2	0	1	1	8	139		I					WD	WD	I
**A*02:17 total**	**02:17 total**			**148**	**61**	**11445**	**1890**	**1517**	**345**	**1097**	**16503**	**C**	**I**	**C**	**C**	**C**	**C**	**C**	**C**	**C**
A*02:17	02:17			76	10	3053	473	856	220	637	5325	C	WD	C	C	C	C	C	C	C
A*02:17P	02:17P			0	0	3	0	0	0	0	3									
**A*02:17:01G total**	**02:17:01G total**			**72**	**51**	**8388**	**1417**	**661**	**125**	**460**	**11174**	**C**	**I**	**C**	**C**	**C**	**C**	**C**	**C**	**C**
A*02:17:01G	02:17:01G		02:17:01G	29	43	8051	1377	267	14	353	10134	WD	I	C	C	C	C	C	C	C
A*02:17:02	02:17:02	HLA00023	02:17:01G	43	8	337	40	394	111	107	1040	C	WD	I	WD	C	C	I	I	C
A*02:17:03	02:17:03	HLA09325		0	0	1	0	0	0	0	1									
A*02:18	02:18	HLA00024		0	2	0	0	0	0	8	10							WD	WD	WD
A*02:19	02:19	HLA00025		0	0	61	0	46	0	55	162			WD		I		I	I	I
**A*02:20 total**	**02:20 total**			**38**	**1265**	**1679**	**191**	**681**	**119**	**332**	**4305**	**WD**	**C**	**C**	**C**	**C**	**C**	**C**	**C**	**C**
A*02:20	02:20			2	33	127	22	75	13	21	293		I	I	WD	C	C	I	I	C
A*02:20:01	02:20:01	HLA00026		32	13	1520	109	603	102	284	2663	WD	I	C	C	C	C	C	C	C
A*02:20:02	02:20:02	HLA01260		4	1219	32	60	3	4	27	1349		C	WD	C			I	I	C
A*02:21	02:21	HLA00027		0	0	27	0	2	0	4	33			WD					WD	WD
**A*02:22 total**	**02:22 total**			**165**	**21**	**1908**	**248**	**1182**	**123**	**450**	**4097**	**C**	**I**	**C**	**C**	**C**	**C**	**C**	**C**	**C**
A*02:22	02:22			1	0	17	0	0	0	1	19			WD					WD	WD
**A*02:22:01G total**	**02:22:01G total**			**164**	**21**	**1891**	**248**	**1182**	**123**	**449**	**4078**	**C**	**I**	**C**	**C**	**C**	**C**	**C**	**C**	**C**
A*02:22:01G	02:22:01G		02:22:01G	133	17	1845	240	927	84	396	3642	C	I	C	C	C	C	C	C	C
A*02:22:01	02:22:01		02:22:01G	30	4	46	8	255	39	53	435	WD		WD	WD	C	C	I	I	C

Supplemental Table 8: HLA-A Allele Summary [a]				Allele Count by Population Group [b]								3.0.0 CIWD Category by Population Group [c]								
Allele	Genomic Typing	AlleleID	G group	AFA	API	EURO	MENA	HIS	NAM	UNK	Total	AFA	API	EURO	MENA	HIS	NAM	UNK	Total	Highest Frequency
A*02:104	02:104	HLA02668	02:22:01G	1	0	0	0	0	0	0	1									
**A*02:24 total**	**02:24 total**			**16**	**3**	**712**	**4**	**63**	**2**	**84**	**884**	**WD**		**I**		**I**		**I**	**I**	**I**
A*02:24	02:24			2	0	58	0	9	0	5	74			WD		I		WD	WD	I
A*02:24:01	02:24:01	HLA00030		14	3	653	4	54	2	79	809	WD		I		I		I	I	I
A*02:24:02	02:24:02	HLA05964		0	0	1	0	0	0	0	1									
A*02:25	02:25	HLA00031		0	0	2	0	0	0	0	2									
A*02:26	02:26	HLA00032		0	0	86	2	3	0	8	99			WD				WD	WD	WD
A*02:27	02:27	HLA00033		0	6	963	3	3	0	11	986		WD	I				WD	I	I
A*02:28	02:28	HLA00034		0	12	1	0	0	0	4	17		WD						WD	WD
A*02:29	02:29	HLA00035		0	4	533	0	5	1	29	572			I		WD		I	I	I
**A*02:30 total**	**02:30 total**			**6**	**16**	**4788**	**9**	**17**	**0**	**181**	**5017**	**WD**	**I**	**C**	**WD**	**I**		**C**	**C**	**C**
A*02:30	02:30			5	12	3650	6	11	0	115	3799	WD	WD	C	WD	I		I	C	C
A*02:30:01	02:30:01	HLA00036		1	4	1136	3	6	0	66	1216			I		WD		I	I	I
A*02:30:02	02:30:02	HLA15258		0	0	2	0	0	0	0	2									
A*02:31	02:31	HLA00967		3	0	5	0	2	0	1	11			WD					WD	WD
A*02:33	02:33	HLA01033		2	2	55	1	191	8	82	341			WD		C	C	I	I	C
A*02:34	02:34	HLA01034		0	0	37	0	0	0	10	47			WD				WD	WD	WD
**A*02:35 total**	**02:35 total**			**5**	**94**	**2124**	**15**	**4**	**0**	**24**	**2266**	**WD**	**I**	**C**	**WD**			**I**	**C**	**C**
A*02:35	02:35			0	3	45	0	1	0	0	49			WD					WD	WD
A*02:35:01	02:35:01	HLA01035		5	91	2071	15	3	0	24	2209	WD	I	C	WD			I	C	C
A*02:35:02	02:35:02	HLA01946		0	0	8	0	0	0	0	8			WD					WD	WD
A*02:36	02:36	HLA01036		0	1	34	0	0	0	5	40			WD				WD	WD	WD
A*02:37	02:37	HLA01102		0	0	15	1	1	0	2	19			WD					WD	WD
A*02:38	02:38	HLA01105		4	1	163	0	3	1	12	184			I				WD	I	I
A*02:39	02:39	HLA01109		0	0	3	0	34	4	7	48					I		WD	WD	I
**A*02:40 total**	**02:40 total**			**0**	**0**	**6**	**0**	**0**	**0**	**1**	**7**			**WD**					**WD**	**WD**
A*02:40	02:40			0	0	2	0	0	0	0	2									
A*02:40:01	02:40:01	HLA01121		0	0	4	0	0	0	1	5								WD	WD
A*02:41	02:41	HLA01172		0	1	0	0	0	0	0	1									
A*02:42	02:42	HLA01198		0	5	2	0	0	0	1	8		WD						WD	WD

Supplemental Table 8: HLA-A Allele Summary [a]				Allele Count by Population Group [b]								3.0.0 CIWD Category by Population Group [c]								
Allele	Genomic Typing	AlleleID	G group	AFA	API	EURO	MENA	HIS	NAM	UNK	Total	AFA	API	EURO	MENA	HIS	NAM	UNK	Total	Highest Frequency
A*02:44	02:44	HLA01222		1	1	8	0	44	4	23	81			WD		I		I	WD	I
A*02:45	02:45	HLA01249		0	0	6	0	10	0	1	17			WD		I			WD	I
A*02:46	02:46	HLA01274		0	1	5	0	0	0	1	7			WD					WD	WD
A*02:47	02:47	HLA01324		4	0	0	0	0	0	2	6								WD	WD
A*02:48	02:48	HLA01331		0	5	3	0	0	0	0	8		WD						WD	WD
**A*02:49 total**	**02:49 total**			**0**	**0**	**55**	**9**	**0**	**0**	**9**	**73**			**WD**	**WD**			**WD**	**WD**	**WD**
**A*02:49:01G total**	**02:49:01G total**			**0**	**0**	**55**	**9**	**0**	**0**	**9**	**73**			**WD**	**WD**			**WD**	**WD**	**WD**
A*02:49	02:49	HLA01404	02:49:01G	0	0	51	9	0	0	9	69			WD	WD			WD	WD	WD
A*02:49:01G	02:49:01G		02:49:01G	0	0	3	0	0	0	0	3									
A*02:683	02:683	HLA17047	02:49:01G	0	0	1	0	0	0	0	1									
A*02:51	02:51	HLA01478		0	0	0	0	4	2	2	8								WD	WD
A*02:52	02:52	HLA01533		0	0	25	0	41	0	36	102			WD		I		I	WD	I
A*02:53N	02:53N	HLA01534		0	52	1	0	0	0	6	59		I					WD	WD	I
A*02:54	02:54	HLA01545		0	0	0	0	2	0	0	2									
A*02:55	02:55	HLA01562		0	0	3	0	0	0	0	3									
**A*02:56 total**	**02:56 total**			**0**	**1**	**126**	**1**	**0**	**0**	**4**	**132**			**I**					**WD**	**I**
A*02:56	02:56			0	0	8	1	0	0	1	10			WD					WD	WD
A*02:56:01	02:56:01	HLA01575		0	0	117	0	0	0	3	120			WD					WD	WD
A*02:56:02	02:56:02	HLA03474		0	1	1	0	0	0	0	2									
A*02:57	02:57	HLA01609		0	0	0	0	7	0	1	8					WD			WD	WD
A*02:58	02:58	HLA01610		0	0	1	0	25	0	3	29					I			WD	I
A*02:59	02:59	HLA01666		0	0	5	0	0	0	0	5			WD					WD	WD
**A*02:60 total**	**02:60 total**			**409**	**3**	**25**	**1**	**50**	**4**	**82**	**574**	**C**		**WD**		**I**		**I**	**I**	**C**
A*02:60	02:60			33	1	6	0	8	0	3	51	WD		WD		I			WD	I
A*02:60:01	02:60:01	HLA01665		376	2	19	1	42	4	79	523	C		WD		I		I	I	C
A*02:61	02:61	HLA01699		0	1	0	0	0	0	0	1									
A*02:63	02:63	HLA01735		13	0	0	0	0	0	1	14	WD							WD	WD
**A*02:64 total**	**02:64 total**			**0**	**34**	**75**	**18**	**51**	**8**	**27**	**213**		**I**	**WD**	**WD**	**I**	**C**	**I**	**I**	**C**
A*02:64	02:64			0	27	38	16	40	6	16	143		I	WD	WD	I	WD	I	WD	I
A*02:64:01	02:64:01	HLA01760		0	7	37	2	11	2	11	70		WD	WD		I		WD	WD	I

Supplemental Table 8: HLA-A Allele Summary [a]				Allele Count by Population Group [b]								3.0.0 CIWD Category by Population Group [c]								
Allele	Genomic Typing	AlleleID	G group	AFA	API	EURO	MENA	HIS	NAM	UNK	Total	AFA	API	EURO	MENA	HIS	NAM	UNK	Total	Highest Frequency
**A*02:65 total**	**02:65 total**			**0**	**0**	**2**	**0**	**0**	**0**	**0**	**2**									
A*02:65	02:65	HLA01778		0	0	2	0	0	0	0	2									
A*02:67	02:67	HLA01802		0	0	58	0	0	0	0	58			WD					WD	WD
A*02:68	02:68	HLA01867		0	0	1	0	0	0	0	1									
A*02:70	02:70	HLA01928		1	0	1	0	0	0	0	2									
A*02:72	02:72	HLA01955		0	1	0	0	0	0	0	1									
**A*02:74 total**	**02:74 total**			**1**	**0**	**34**	**0**	**1**	**0**	**1**	**37**			**WD**					**WD**	**WD**
A*02:74	02:74			0	0	2	0	0	0	1	3									
A*02:74:01	02:74:01	HLA02028		0	0	27	0	0	0	0	27			WD					WD	WD
A*02:74:02	02:74:02	HLA02126		1	0	5	0	1	0	0	7			WD					WD	WD
**A*02:76 total**	**02:76 total**			**0**	**0**	**6**	**0**	**0**	**0**	**0**	**6**			**WD**					**WD**	**WD**
A*02:76	02:76			0	0	2	0	0	0	0	2									
A*02:76:01	02:76:01	HLA02081		0	0	3	0	0	0	0	3									
A*02:76:02	02:76:02	HLA07333		0	0	1	0	0	0	0	1									
A*02:77	02:77	HLA02082		2	0	29	0	0	0	2	33			WD					WD	WD
A*02:80	02:80	HLA02160		0	1	0	0	0	0	0	1									
**A*02:81 total**	**02:81 total**			**0**	**0**	**6**	**0**	**0**	**0**	**0**	**6**			**WD**					**WD**	**WD**
**A*02:81:01G total**	**02:81:01G total**			**0**	**0**	**6**	**0**	**0**	**0**	**0**	**6**			**WD**					**WD**	**WD**
A*02:81:01G	02:81:01G		02:81:01G	0	0	6	0	0	0	0	6			WD					WD	WD
A*02:84	02:84	HLA02233		8	0	3	0	0	5	10	26	WD					WD	WD	WD	WD
A*02:85	02:85	HLA02235		10	0	106	91	3	0	20	230	WD		WD	C			I	I	C
**A*02:86 total**	**02:86 total**			**11**	**1**	**59**	**0**	**1**	**2**	**12**	**86**	**WD**		**WD**				**WD**	**WD**	**WD**
A*02:86	02:86			8	1	54	0	0	2	10	75	WD		WD				WD	WD	WD
A*02:86:01	02:86:01	HLA02248		3	0	5	0	1	0	2	11			WD					WD	WD
A*02:87	02:87	HLA02321		1	0	19	1	16	3	8	48			WD		I		WD	WD	I
A*02:88N	02:88N	HLA02337		0	0	4	0	0	0	0	4									
A*02:90	02:90	HLA02374		0	2	34	4	0	0	0	40			WD					WD	WD
A*02:92	02:92	HLA02440		0	0	3	0	0	0	0	3									
**A*02:93 total**	**02:93 total**			**0**	**3**	**42**	**94**	**0**	**0**	**9**	**148**			**WD**	**C**			**WD**	**WD**	**C**
A*02:93	02:93			0	2	38	89	0	0	8	137			WD	C			WD	WD	C

Supplemental Table 8: HLA-A Allele Summary [a]				Allele Count by Population Group [b]								3.0.0 CIWD Category by Population Group [c]								
Allele	Genomic Typing	AlleleID	G group	AFA	API	EURO	MENA	HIS	NAM	UNK	Total	AFA	API	EURO	MENA	HIS	NAM	UNK	Total	Highest Frequency
A*02:93:01	02:93:01	HLA02468		0	1	4	5	0	0	1	11				WD				WD	WD
A*02:94N	02:94N	HLA02476		0	0	28	0	0	0	3	31			WD					WD	WD
A*02:95	02:95	HLA02481		0	4	57	0	0	0	2	63			WD					WD	WD
A*02:96	02:96	HLA02501		0	0	96	0	5	0	31	132			WD		WD		I	WD	I
**A*02:101 total**	**02:101 total**			**0**	**2**	**15**	**1**	**1**	**0**	**2**	**21**			**WD**					**WD**	**WD**
A*02:101	02:101			0	0	4	0	0	0	0	4									
A*02:101:01	02:101:01	HLA02538		0	0	6	1	1	0	1	9			WD					WD	WD
A*02:101:02	02:101:02	HLA06098		0	2	5	0	0	0	1	8			WD					WD	WD
A*02:102	02:102	HLA02541		1	0	2	10	1	0	17	31				WD			I	WD	I
A*02:105	02:105	HLA02703		0	0	5	0	4	1	4	14			WD					WD	WD
A*02:107	02:107	HLA02711		0	0	21	0	0	0	12	33			WD				WD	WD	WD
A*02:108	02:108	HLA02724		0	1	0	0	0	0	0	1									
A*02:109	02:109	HLA02755		0	0	5	0	1	0	0	6			WD					WD	WD
A*02:110	02:110	HLA02776		0	0	8	16	0	0	1	25			WD	WD				WD	WD
**A*02:113N total**	**02:113N total**			**0**	**0**	**4**	**0**	**0**	**0**	**2**	**6**								**WD**	**WD**
A*02:113N	02:113N			0	0	1	0	0	0	0	1									
A*02:113:01N	02:113:01N	HLA02867		0	0	2	0	0	0	2	4									
A*02:113:02N	02:113:02N	HLA12434		0	0	1	0	0	0	0	1									
A*02:114	02:114	HLA02896		0	0	3	1	0	0	1	5								WD	WD
A*02:115	02:115	HLA02899		3	0	0	0	0	0	0	3									
A*02:116	02:116	HLA02901		0	1	1	1	0	0	0	3									
A*02:118	02:118	HLA02935		0	0	8	0	0	0	2	10			WD					WD	WD
A*02:119	02:119	HLA02936		0	0	37	0	0	0	4	41			WD					WD	WD
A*02:120	02:120	HLA02937		0	0	8	0	0	0	0	8			WD					WD	WD
A*02:121	02:121	HLA02953		1	0	15	1	0	0	4	21			WD					WD	WD
A*02:122	02:122	HLA02956		6	0	0	0	3	0	4	13	WD							WD	WD
A*02:123	02:123	HLA02957		0	0	74	0	1	0	8	83			WD				WD	WD	WD
A*02:125N	02:125N	HLA02961		0	0	21	0	0	0	0	21			WD					WD	WD
A*02:127	02:127	HLA03021		0	1	0	0	0	0	0	1									
A*02:128	02:128	HLA03022		0	6	0	0	0	0	0	6		WD						WD	WD

Supplemental Table 8: HLA-A Allele Summary [a]				Allele Count by Population Group [b]								3.0.0 CIWD Category by Population Group [c]								
Allele	Genomic Typing	AlleleID	G group	AFA	API	EURO	MENA	HIS	NAM	UNK	Total	AFA	API	EURO	MENA	HIS	NAM	UNK	Total	Highest Frequency
A*02:133	02:133	HLA03132		0	0	34	0	0	0	2	36			WD					WD	WD
A*02:135	02:135	HLA03145		0	0	20	1	0	0	1	22			WD					WD	WD
A*02:136	02:136	HLA03162		0	0	2	0	17	1	7	27					I		WD	WD	I
A*02:137	02:137	HLA03181		0	0	1	0	15	1	1	18					I			WD	I
A*02:138	02:138	HLA03229		0	0	19	0	0	0	0	19			WD					WD	WD
A*02:139	02:139	HLA03235		0	0	20	0	0	0	0	20			WD					WD	WD
A*02:141	02:141	HLA03284		0	0	13	28	1	0	3	45			WD	WD				WD	WD
A*02:144	02:144	HLA03306		0	0	0	0	0	0	1	1									
A*02:145	02:145	HLA03326		0	0	1	0	0	0	1	2									
A*02:146	02:146	HLA03330		0	0	1	2	0	0	0	3									
A*02:147	02:147	HLA03346		0	0	1	0	20	0	3	24					I			WD	I
A*02:149	02:149	HLA03421		0	0	8	0	0	0	1	9			WD					WD	WD
A*02:150	02:150	HLA03452		0	0	0	1	0	0	1	2									
A*02:151	02:151	HLA03475		0	0	142	0	0	0	7	149			I				WD	WD	I
A*02:152	02:152	HLA03497		0	0	2	0	1	0	0	3									
**A*02:153 total**	**02:153 total**			**0**	**0**	**4**	**0**	**0**	**0**	**0**	**4**									
A*02:153	02:153			0	0	1	0	0	0	0	1									
A*02:153:01	02:153:01	HLA03567		0	0	3	0	0	0	0	3									
A*02:154	02:154	HLA03579		0	0	4	20	0	0	3	27				WD				WD	WD
A*02:156	02:156	HLA03582		0	0	20	0	0	0	0	20			WD					WD	WD
**A*02:157 total**	**02:157 total**			**0**	**0**	**7**	**0**	**0**	**0**	**3**	**10**			**WD**					**WD**	**WD**
A*02:157	02:157			0	0	6	0	0	0	3	9			WD					WD	WD
A*02:157:01	02:157:01	HLA03629		0	0	1	0	0	0	0	1									
A*02:158	02:158	HLA03652		0	0	107	0	4	0	2	113			WD					WD	WD
A*02:159	02:159	HLA03653		1	0	0	0	0	0	0	1									
A*02:160	02:160	HLA03655		0	0	29	0	0	0	0	29			WD					WD	WD
A*02:161	02:161	HLA03657		0	0	0	11	0	0	0	11				WD				WD	WD
A*02:162	02:162	HLA03669		0	0	5	0	0	0	0	5			WD					WD	WD
A*02:163	02:163	HLA03670		0	0	38	0	1	0	1	40			WD					WD	WD
**A*02:164 total**	**02:164 total**			**0**	**0**	**21**	**0**	**0**	**0**	**0**	**21**			**WD**					**WD**	**WD**

Supplemental Table 8: HLA-A Allele Summary [a]				Allele Count by Population Group [b]								3.0.0 CIWD Category by Population Group [c]								
Allele	Genomic Typing	AlleleID	G group	AFA	API	EURO	MENA	HIS	NAM	UNK	Total	AFA	API	EURO	MENA	HIS	NAM	UNK	Total	Highest Frequency
A*02:164	02:164			0	0	19	0	0	0	0	19			WD					WD	WD
A*02:164:01	02:164:01	HLA03671		0	0	2	0	0	0	0	2									
A*02:165	02:165	HLA03672		0	0	5	0	0	0	0	5			WD					WD	WD
A*02:166	02:166	HLA03673		0	0	12	0	1	0	0	13			WD					WD	WD
**A*02:171 total**	**02:171 total**			**0**	**1**	**0**	**0**	**0**	**0**	**0**	**1**									
A*02:171:02	02:171:02	HLA04646		0	1	0	0	0	0	0	1									
A*02:172	02:172	HLA03784		0	0	9	0	0	0	0	9			WD					WD	WD
A*02:173	02:173	HLA03785		0	0	5	0	0	0	0	5			WD					WD	WD
A*02:174	02:174	HLA03786		0	0	4	0	0	0	1	5								WD	WD
A*02:176	02:176	HLA03795		0	0	3	0	0	0	0	3									
A*02:177	02:177	HLA03815		14	0	3	0	0	4	2	23	WD							WD	WD
A*02:180	02:180	HLA03880		0	1	16	0	0	0	1	18			WD					WD	WD
A*02:182	02:182	HLA03886		0	0	13	0	0	0	0	13			WD					WD	WD
A*02:183	02:183	HLA03892		2	0	26	1	1	1	8	39			WD				WD	WD	WD
A*02:185	02:185	HLA03895		0	0	2	0	0	0	0	2									
A*02:186	02:186	HLA03932		9	0	0	0	0	0	2	11	WD							WD	WD
A*02:187	02:187	HLA04063		2	0	0	0	0	0	0	2									
A*02:188	02:188	HLA04064		0	0	42	0	0	0	0	42			WD					WD	WD
A*02:189	02:189	HLA04065		0	0	13	0	0	0	0	13			WD					WD	WD
A*02:190	02:190	HLA03996		1	0	12	0	0	0	1	14			WD					WD	WD
A*02:192	02:192	HLA04120		0	0	3	0	0	0	0	3									
A*02:193	02:193	HLA04121		2	0	0	0	0	0	1	3									
A*02:194	02:194	HLA04149		0	0	27	0	0	0	1	28			WD					WD	WD
A*02:195	02:195	HLA04152		0	0	5	0	0	0	0	5			WD					WD	WD
A*02:196	02:196	HLA04420		0	0	1	0	0	0	0	1									
**A*02:197 total**	**02:197 total**			**0**	**0**	**10**	**0**	**1**	**1**	**1**	**13**			**WD**					**WD**	**WD**
A*02:197	02:197			0	0	10	0	1	1	1	13			WD					WD	WD
A*02:198	02:198	HLA04424		0	0	6	0	0	0	0	6			WD					WD	WD
A*02:199	02:199	HLA04430		0	0	14	0	0	0	1	15			WD					WD	WD
A*02:200	02:200	HLA04928		2	0	0	0	0	0	1	3									

Supplemental Table 8: HLA-A Allele Summary [a]				Allele Count by Population Group [b]								3.0.0 CIWD Category by Population Group [c]								
Allele	Genomic Typing	AlleleID	G group	AFA	API	EURO	MENA	HIS	NAM	UNK	Total	AFA	API	EURO	MENA	HIS	NAM	UNK	Total	Highest Frequency
A*02:201	02:201	HLA04943		0	0	1	0	0	0	0	1									
A*02:202	02:202	HLA04944		0	0	3	1	0	0	0	4									
A*02:203	02:203	HLA04945		0	0	6	0	1	0	0	7			WD					WD	WD
A*02:204	02:204	HLA04948		0	0	1	0	0	0	0	1									
A*02:205	02:205	HLA04949		0	0	12	0	0	0	1	13			WD					WD	WD
A*02:206	02:206	HLA04951		0	0	22	0	0	0	2	24			WD					WD	WD
A*02:209	02:209	HLA04968		0	0	3	2	0	0	1	6								WD	WD
A*02:210	02:210	HLA04969		0	0	8	0	0	0	0	8			WD					WD	WD
**A*02:211 total**	**02:211 total**			**2**	**1**	**16**	**0**	**24**	**2**	**4**	**49**			**WD**		**I**			**WD**	**I**
A*02:211	02:211			1	1	3	0	4	2	1	12								WD	WD
A*02:211:01	02:211:01	HLA04970		1	0	13	0	20	0	3	37			WD		I			WD	I
A*02:212	02:212	HLA04971		0	0	10	0	0	0	0	10			WD					WD	WD
A*02:213	02:213	HLA04972		0	0	4	0	0	0	0	4									
A*02:214	02:214	HLA04973		0	0	2	0	0	0	2	4									
A*02:215	02:215	HLA04975		0	0	1	0	0	0	0	1									
A*02:216	02:216	HLA04976		1	0	0	0	0	0	0	1									
**A*02:217 total**	**02:217 total**			**0**	**0**	**18**	**0**	**0**	**0**	**1**	**19**			**WD**					**WD**	**WD**
A*02:217	02:217			0	0	2	0	0	0	0	2									
A*02:217:01	02:217:01	HLA04984		0	0	9	0	0	0	0	9			WD					WD	WD
A*02:217:02	02:217:02	HLA06526		0	0	7	0	0	0	1	8			WD					WD	WD
A*02:218	02:218	HLA04985		0	0	7	0	0	0	0	7			WD					WD	WD
A*02:219	02:219	HLA04986		0	1	0	0	0	0	0	1									
A*02:220	02:220	HLA04987		0	0	2	0	0	0	0	2									
A*02:221	02:221	HLA04989		0	0	29	1	2	0	4	36			WD					WD	WD
A*02:223N	02:223N	HLA04991		0	0	3	0	0	0	0	3									
A*02:224	02:224	HLA05001		0	0	1	0	0	0	0	1									
A*02:227N	02:227N	HLA05004		0	1	18	0	0	0	0	19			WD					WD	WD
A*02:228	02:228	HLA05005		0	0	1	0	0	0	0	1									
A*02:229	02:229	HLA05020		0	0	1	0	0	0	0	1									
A*02:231	02:231	HLA05037		0	3	0	0	0	0	0	3									

Supplemental Table 8: HLA-A Allele Summary [a]				Allele Count by Population Group [b]								3.0.0 CIWD Category by Population Group [c]								
Allele	Genomic Typing	AlleleID	G group	AFA	API	EURO	MENA	HIS	NAM	UNK	Total	AFA	API	EURO	MENA	HIS	NAM	UNK	Total	Highest Frequency
A*02:232	02:232	HLA05038		0	0	1	0	0	0	0	1									
A*02:233	02:233	HLA05039		0	0	1	0	0	0	0	1									
A*02:235	02:235	HLA05041		0	0	8	0	0	0	0	8			WD					WD	WD
A*02:236	02:236	HLA05042		0	0	5	0	0	0	0	5			WD					WD	WD
A*02:237	02:237	HLA05043		0	0	6	0	0	0	0	6			WD					WD	WD
A*02:238	02:238	HLA05044		0	0	3	0	0	0	0	3									
**A*02:243 total**	**02:243 total**			**0**	**1**	**0**	**0**	**0**	**0**	**0**	**1**									
A*02:243:01	02:243:01	HLA04893		0	1	0	0	0	0	0	1									
A*02:245	02:245	HLA05078		0	0	15	0	0	0	0	15			WD					WD	WD
A*02:246	02:246	HLA05087		0	0	8	2	0	0	5	15			WD				WD	WD	WD
A*02:249	02:249	HLA05225		0	1	0	0	0	0	0	1									
A*02:250N	02:250N	HLA05226		0	0	2	0	0	0	0	2									
A*02:251	02:251	HLA05227		0	1	0	0	0	0	0	1									
A*02:254	02:254	HLA05245		0	0	1	0	0	0	0	1									
A*02:257	02:257	HLA05307		0	0	1	0	0	0	0	1									
A*02:259	02:259	HLA05368		0	1	0	0	0	0	0	1									
A*02:261	02:261	HLA05375		0	1	0	0	0	0	0	1									
A*02:262	02:262	HLA05384		0	0	1	0	0	0	3	4									
A*02:263	02:263	HLA05399		0	0	4	0	1	0	0	5								WD	WD
A*02:268	02:268	HLA05532		0	0	4	0	0	0	0	4									
A*02:269	02:269	HLA05542		0	0	1	0	0	0	0	1									
A*02:270	02:270	HLA05549		0	0	2	1	0	0	0	3									
A*02:273	02:273	HLA05676		0	2	5	0	0	0	0	7			WD					WD	WD
A*02:277	02:277	HLA05696		0	0	5	0	1	0	0	6			WD					WD	WD
A*02:278	02:278	HLA05697		0	0	0	0	5	0	0	5					WD			WD	WD
A*02:280	02:280	HLA05895		0	1	0	0	0	0	0	1									
A*02:281	02:281	HLA05896		0	2	0	0	0	0	0	2									
A*02:283	02:283	HLA05948		0	0	26	0	0	0	4	30			WD					WD	WD
A*02:286	02:286	HLA06008		0	1	0	3	0	0	0	4									
**A*02:289 total**	**02:289 total**			**1**	**5**	**10**	**0**	**0**	**0**	**0**	**16**		**WD**	**WD**					**WD**	**WD**

Supplemental Table 8: HLA-A Allele Summary [a]				Allele Count by Population Group [b]								3.0.0 CIWD Category by Population Group [c]								
Allele	Genomic Typing	AlleleID	G group	AFA	API	EURO	MENA	HIS	NAM	UNK	Total	AFA	API	EURO	MENA	HIS	NAM	UNK	Total	Highest Frequency
A*02:289	02:289			1	2	5	0	0	0	0	8			WD					WD	WD
A*02:289:01	02:289:01	HLA06017		0	3	5	0	0	0	0	8			WD					WD	WD
A*02:292	02:292	HLA06079		0	0	1	0	0	0	0	1									
A*02:293Q	02:293Q	HLA06080		0	1	0	0	0	0	0	1									
A*02:296	02:296	HLA06088		0	0	3	0	0	0	0	3									
A*02:297	02:297	HLA06092		0	3	0	0	0	0	0	3									
A*02:298	02:298	HLA06099		0	1	0	0	0	0	0	1									
A*02:299	02:299	HLA06106		0	0	34	1	0	0	0	35			WD					WD	WD
A*02:302	02:302	HLA06336		0	0	0	0	0	0	1	1									
A*02:306	02:306	HLA06564		2	0	5	0	0	0	1	8			WD					WD	WD
A*02:307	02:307	HLA06565		0	0	2	0	0	0	0	2									
A*02:309	02:309	HLA06572		0	0	1	0	0	0	0	1									
A*02:312	02:312	HLA06747		0	0	4	0	0	0	2	6								WD	WD
A*02:313	02:313	HLA06758		0	0	3	0	0	0	0	3									
A*02:315	02:315	HLA06760		0	1	0	0	0	0	0	1									
A*02:316	02:316	HLA06767		0	0	0	0	0	0	1	1									
A*02:317	02:317	HLA06792		0	0	1	0	0	0	0	1									
A*02:320	02:320	HLA06832		0	0	0	0	0	0	1	1									
A*02:325	02:325	HLA06912		0	0	5	0	0	0	0	5			WD					WD	WD
A*02:326	02:326	HLA06913		0	0	8	0	0	0	1	9			WD					WD	WD
A*02:331	02:331	HLA07291		0	2	0	0	0	0	0	2									
A*02:332	02:332	HLA07292		0	0	7	0	0	0	0	7			WD					WD	WD
A*02:334	02:334	HLA07342		0	0	0	0	1	0	0	1									
A*02:336	02:336	HLA07387		0	0	0	0	1	0	0	1									
A*02:337	02:337	HLA07430		0	0	2	0	0	0	0	2									
A*02:338	02:338	HLA07431		0	0	11	0	1	0	3	15			WD					WD	WD
A*02:339	02:339	HLA07432		0	0	2	0	0	0	0	2									
A*02:340	02:340	HLA07433		0	0	1	0	0	0	0	1									
A*02:341	02:341	HLA07434		0	0	0	0	0	0	1	1									
A*02:342	02:342	HLA07435		0	0	13	0	0	0	0	13			WD					WD	WD

Supplemental Table 8: HLA-A Allele Summary [a]				Allele Count by Population Group [b]								3.0.0 CIWD Category by Population Group [c]								
Allele	Genomic Typing	AlleleID	G group	AFA	API	EURO	MENA	HIS	NAM	UNK	Total	AFA	API	EURO	MENA	HIS	NAM	UNK	Total	Highest Frequency
A*02:343	02:343	HLA07437		0	4	6	0	0	0	0	10			WD					WD	WD
A*02:344	02:344	HLA07607		0	2	0	1	0	0	0	3									
A*02:345	02:345	HLA07611		0	1	0	0	0	0	0	1									
A*02:346	02:346	HLA07612		0	0	7	0	0	0	0	7			WD					WD	WD
A*02:347	02:347	HLA07613		0	0	1	0	0	0	0	1									
A*02:350N	02:350N	HLA07619		0	0	3	0	0	0	0	3									
A*02:352	02:352	HLA07622		2	0	1	0	0	0	0	3									
A*02:353	02:353	HLA07677		0	0	1	0	0	0	0	1									
A*02:354	02:354	HLA07678		0	0	0	0	1	0	0	1									
A*02:355	02:355	HLA07747		0	0	0	1	0	0	0	1									
A*02:359	02:359	HLA08002		0	0	4	0	0	0	3	7								WD	WD
A*02:362	02:362	HLA08009		0	0	1	0	0	0	0	1									
A*02:364	02:364	HLA08011		0	0	1	0	0	0	0	1									
A*02:366N	02:366N	HLA08013		0	0	1	0	0	0	0	1									
A*02:367	02:367	HLA08016		0	0	15	0	0	0	0	15			WD					WD	WD
A*02:368	02:368	HLA08018		0	0	0	0	0	0	1	1									
A*02:372	02:372	HLA08096		0	0	2	0	0	0	0	2									
A*02:374	02:374	HLA08303		0	0	1	0	0	0	0	1									
A*02:377	02:377	HLA08462		0	0	2	0	0	0	0	2									
A*02:379	02:379	HLA08464		0	0	2	0	0	0	0	2									
A*02:380	02:380	HLA08466		0	0	10	0	0	0	1	11			WD					WD	WD
A*02:382	02:382	HLA08493		0	0	0	7	0	0	0	7				WD				WD	WD
A*02:384	02:384	HLA08495		1	0	0	0	0	0	0	1									
A*02:386	02:386	HLA08498		0	0	1	0	0	0	0	1									
A*02:389	02:389	HLA08647		0	0	2	0	0	0	0	2									
A*02:390	02:390	HLA08648		0	0	1	0	0	0	0	1									
A*02:392	02:392	HLA08650		0	0	0	4	0	0	1	5								WD	WD
A*02:393	02:393	HLA08883		0	0	7	0	0	0	0	7			WD					WD	WD
A*02:394	02:394	HLA08888		0	0	1	0	0	0	0	1									
A*02:398	02:398	HLA08972		0	0	0	0	1	0	0	1									

Supplemental Table 8: HLA-A Allele Summary [a]				Allele Count by Population Group [b]								3.0.0 CIWD Category by Population Group [c]								
Allele	Genomic Typing	AlleleID	G group	AFA	API	EURO	MENA	HIS	NAM	UNK	Total	AFA	API	EURO	MENA	HIS	NAM	UNK	Total	Highest Frequency
A*02:406	02:406	HLA09125		0	0	2	0	0	0	0	2									
A*02:407	02:407	HLA09126		0	0	3	0	1	0	0	4									
A*02:409	02:409	HLA09163		0	1	1	0	0	0	0	2									
A*02:410	02:410	HLA09164		1	0	0	0	0	0	0	1									
**A*02:419 total**	**02:419 total**			**0**	**2**	**0**	**0**	**0**	**0**	**0**	**2**									
A*02:419	02:419			0	2	0	0	0	0	0	2									
A*02:423	02:423	HLA09551		0	8	1	0	0	0	0	9		WD						WD	WD
A*02:429	02:429	HLA09692		0	3	0	0	0	0	0	3									
A*02:430	02:430	HLA09693		0	0	3	0	0	0	0	3									
A*02:431	02:431	HLA09715		0	1	0	0	0	0	0	1									
A*02:433	02:433	HLA09808		0	0	0	6	0	0	0	6				WD				WD	WD
A*02:434	02:434	HLA09809		0	0	0	6	0	0	0	6				WD				WD	WD
A*02:435	02:435	HLA09812		0	0	1	0	0	0	0	1									
A*02:436	02:436	HLA09815		0	14	0	0	0	0	0	14		I						WD	I
A*02:461	02:461	HLA10522		0	0	0	0	3	0	0	3									
A*02:474	02:474	HLA10800		0	0	0	0	0	0	1	1									
A*02:483	02:483	HLA10935		0	0	0	0	0	0	2	2									
A*02:484	02:484	HLA10986		0	0	5	0	0	0	0	5			WD					WD	WD
A*02:486	02:486	HLA10988		0	0	2	0	0	0	0	2									
A*02:487	02:487	HLA10990		0	1	0	0	0	0	0	1									
A*02:495	02:495	HLA11405		0	0	1	0	0	0	0	1									
A*02:497	02:497	HLA11555		0	0	0	0	1	0	0	1									
A*02:498	02:498	HLA11408		0	0	5	0	0	0	0	5			WD					WD	WD
A*02:500Q	02:500Q	HLA11410		0	1	0	0	0	0	0	1									
A*02:503	02:503	HLA11413		0	4	0	0	0	0	0	4									
A*02:504	02:504	HLA11414		0	0	0	0	0	0	2	2									
A*02:508	02:508	HLA11643		1	0	0	0	0	0	0	1									
A*02:514N	02:514N	HLA11808		0	8	0	0	0	0	0	8		WD						WD	WD
A*02:515	02:515	HLA11809		0	0	0	0	0	0	1	1									
A*02:517	02:517	HLA11812		1	0	0	0	1	0	0	2									

Supplemental Table 8: HLA-A Allele Summary [a]				Allele Count by Population Group [b]								3.0.0 CIWD Category by Population Group [c]								
Allele	Genomic Typing	AlleleID	G group	AFA	API	EURO	MENA	HIS	NAM	UNK	Total	AFA	API	EURO	MENA	HIS	NAM	UNK	Total	Highest Frequency
A*02:518	02:518	HLA11993		0	0	1	0	0	0	0	1									
A*02:521	02:521	HLA11997		0	0	1	0	0	0	0	1									
A*02:523	02:523	HLA11943		0	0	1	0	0	0	0	1									
**A*02:524 total**	**02:524 total**			**0**	**0**	**3**	**0**	**0**	**0**	**0**	**3**									
A*02:524	02:524			0	0	1	0	0	0	0	1									
A*02:524:01	02:524:01	HLA11962		0	0	1	0	0	0	0	1									
A*02:524:02	02:524:02	HLA14720		0	0	1	0	0	0	0	1									
A*02:526	02:526	HLA12092		0	0	0	1	0	0	1	2									
A*02:527	02:527	HLA12115		0	0	1	0	0	0	0	1									
**A*02:528 total**	**02:528 total**			**0**	**0**	**1**	**0**	**0**	**0**	**0**	**1**									
A*02:528	02:528			0	0	1	0	0	0	0	1									
A*02:529	02:529	HLA12119		0	2	0	0	0	0	0	2									
A*02:530	02:530	HLA12120		0	0	0	0	1	0	0	1									
A*02:531	02:531	HLA12122		2	0	0	0	0	0	0	2									
A*02:536	02:536	HLA12165		0	0	1	0	0	0	0	1									
A*02:541	02:541	HLA12408		0	0	0	0	0	0	1	1									
A*02:543	02:543	HLA12469		0	0	2	0	0	0	0	2									
A*02:545	02:545	HLA12615		0	0	1	0	0	0	0	1									
A*02:548	02:548	HLA12736		0	0	0	0	1	0	0	1									
A*02:549	02:549	HLA12641		0	0	2	0	0	0	0	2									
A*02:550	02:550	HLA12642		0	1	0	0	0	0	0	1									
A*02:551	02:551	HLA12644		0	0	1	0	0	0	0	1									
A*02:552	02:552	HLA12646		0	0	0	0	2	0	1	3									
A*02:554	02:554	HLA12648		0	0	0	0	1	0	0	1									
A*02:556	02:556	HLA12973		0	0	1	0	0	0	0	1									
A*02:560	02:560	HLA13080		0	1	0	0	0	0	0	1									
A*02:561	02:561	HLA13083		0	0	1	0	0	0	0	1									
A*02:562	02:562	HLA13084		0	0	1	7	0	0	0	8				WD				WD	WD
A*02:564	02:564	HLA13086		0	0	1	0	1	0	0	2									
A*02:568	02:568	HLA13092		0	1	0	0	0	0	0	1									

Supplemental Table 8: HLA-A Allele Summary [a]				Allele Count by Population Group [b]								3.0.0 CIWD Category by Population Group [c]								
Allele	Genomic Typing	AlleleID	G group	AFA	API	EURO	MENA	HIS	NAM	UNK	Total	AFA	API	EURO	MENA	HIS	NAM	UNK	Total	Highest Frequency
A*02:569	02:569	HLA13093		0	1	0	0	0	0	0	1									
A*02:574	02:574	HLA13458		0	0	0	0	1	0	0	1									
A*02:579	02:579	HLA13733		0	1	0	0	0	0	0	1									
A*02:580	02:580	HLA13734		0	7	0	0	0	0	0	7		WD						WD	WD
A*02:581	02:581	HLA13849		0	0	0	0	0	0	2	2									
A*02:587	02:587	HLA13772		0	0	2	1	0	0	0	3									
A*02:589	02:589	HLA14004		0	1	0	0	0	0	0	1									
A*02:592	02:592	HLA14133		0	0	2	0	0	0	0	2									
A*02:595	02:595	HLA14136		0	3	0	0	0	0	0	3									
A*02:602	02:602	HLA14396		0	0	0	0	1	0	0	1									
A*02:605Q	02:605Q	HLA14515		0	0	1	0	0	0	0	1									
A*02:611	02:611	HLA14718		0	1	0	0	0	0	0	1									
A*02:612	02:612	HLA14719		0	1	0	0	0	0	0	1									
A*02:616	02:616	HLA14921		0	0	1	0	0	0	0	1									
A*02:623	02:623	HLA14875		0	0	0	0	0	0	16	16							I	WD	I
A*02:626	02:626	HLA14899		0	0	1	0	0	0	0	1									
A*02:639	02:639	HLA15535		0	0	0	0	1	0	0	1									
A*02:646	02:646	HLA15828		0	0	1	0	0	0	0	1									
A*02:673	02:673	HLA16879		0	0	1	0	0	0	0	1									
A*02:CODE	02:CODE			2134	3755	99979	1675	11124	499	12089	131255	NA	NA	NA	NA	NA	NA	NA	NA	NA
**A*03:01 total**	**03:01 total**			**29370**	**63469**	**1592095**	**32064**	**49403**	**4907**	**136849**	**1908157**	**C**	**C**	**C**	**C**	**C**	**C**	**C**	**C**	**C**
A*03:01	03:01			12	11	6403	14	46	0	316	6802	WD	WD	C	WD	I		C	C	C
A*03:01P	03:01P			1	4	1512	4	4	0	5	1530			C				WD	I	C
**A*03:01:01G total**	**03:01:01G total**			**29245**	**63439**	**1583173**	**32036**	**49234**	**4895**	**136406**	**1898428**	**C**	**C**	**C**	**C**	**C**	**C**	**C**	**C**	**C**
A*03:01:01G	03:01:01G		03:01:01G	24144	60337	1517234	31309	37296	3784	127307	1801411	C	C	C	C	C	C	C	C	C
A*03:01:01	03:01:01		03:01:01G	1200	745	17281	181	2901	235	2282	24825	C	C	C	C	C	C	C	C	C
A*03:01:01:01	03:01:01:01	HLA00037	03:01:01G	1497	2314	46439	483	7929	740	6041	65443	C	C	C	C	C	C	C	C	C
**A*03:01N total**	**03:01N total**			**0**	**0**	**1**	**0**	**0**	**0**	**0**	**1**									
A*03:01:01:02N	03:01:01:02N	HLA01718	03:01:01G	0	0	1	0	0	0	0	1									
A*03:01:01:03	03:01:01:03	HLA01960	03:01:01G	13	9	558	15	155	5	63	818	WD	WD	I	WD	C	WD	I	I	C

Supplemental Table 8: HLA-A Allele Summary [a]				Allele Count by Population Group [b]								3.0.0 CIWD Category by Population Group [c]								
Allele	Genomic Typing	AlleleID	G group	AFA	API	EURO	MENA	HIS	NAM	UNK	Total	AFA	API	EURO	MENA	HIS	NAM	UNK	Total	Highest Frequency
A*03:01:01:05	03:01:01:05	HLA13785	03:01:01G	2068	28	1535	43	892	113	641	5320	C	I	C	C	C	C	C	C	C
A*03:01:01:06	03:01:01:06	HLA14216	03:01:01G	0	0	6	0	0	0	0	6			WD					WD	WD
A*03:01:01:07	03:01:01:07	HLA14827	03:01:01G	321	1	7	2	56	17	54	458	C		WD		I	C	I	I	C
A*03:01:01:08	03:01:01:08	HLA15501	03:01:01G	0	0	11	0	0	0	0	11			WD					WD	WD
A*03:01:01:09	03:01:01:09	HLA15759	03:01:01G	0	0	1	0	0	0	0	1									
A*03:01:01:10	03:01:01:10	HLA15761	03:01:01G	0	0	1	0	0	0	0	1									
A*03:01:01:11	03:01:01:11	HLA16662	03:01:01G	1	0	13	0	0	0	1	15			WD					WD	WD
A*03:01:56	03:01:56	HLA14001	03:01:01G	0	0	1	0	0	0	1	2									
A*03:20	03:20	HLA02325	03:01:01G	0	2	59	3	1	0	10	75			WD				WD	WD	WD
A*03:21N	03:21N	HLA02369	03:01:01G	0	0	5	0	1	0	0	6			WD					WD	WD
A*03:26	03:26	HLA02761	03:01:01G	1	3	18	0	3	1	6	32			WD				WD	WD	WD
A*03:132	03:132	HLA07040	03:01:01G	0	0	2	0	0	0	0	2									
A*03:134	03:134	HLA07353	03:01:01G	0	0	1	0	0	0	0	1									
A*03:01:02	03:01:02	HLA00038		94	1	11	3	5	1	16	131	C		WD		WD		I	WD	C
A*03:01:03	03:01:03	HLA00039		15	3	431	2	110	10	62	633	WD		I		C	C	I	I	C
A*03:01:05	03:01:05	HLA02576		0	0	80	0	0	0	1	81			WD					WD	WD
A*03:01:11	03:01:11	HLA03743		0	2	87	0	1	0	4	94			WD					WD	WD
A*03:01:12	03:01:12	HLA03800		0	1	18	0	0	0	0	19			WD					WD	WD
A*03:01:14	03:01:14	HLA03873		0	0	223	0	0	0	4	227			I					I	I
A*03:01:15	03:01:15	HLA03927		0	0	26	0	0	0	3	29			WD					WD	WD
A*03:01:16	03:01:16	HLA04148		0	0	3	0	0	0	4	7								WD	WD
A*03:01:17	03:01:17	HLA04422		0	0	29	0	0	0	0	29			WD					WD	WD
A*03:01:18	03:01:18	HLA05335		0	0	27	0	0	0	1	28			WD					WD	WD
A*03:01:19	03:01:19	HLA05337		0	0	19	0	1	0	0	20			WD					WD	WD
A*03:01:20	03:01:20	HLA05453		0	0	1	0	0	0	0	1									
A*03:01:21	03:01:21	HLA05665		0	0	5	1	0	0	5	11			WD				WD	WD	WD
A*03:01:22	03:01:22	HLA05686		0	0	3	0	0	0	0	3									
A*03:01:23	03:01:23	HLA05689		0	0	6	0	0	0	12	18			WD				WD	WD	WD
A*03:01:24	03:01:24	HLA06328		0	0	1	0	0	0	0	1									
A*03:01:25	03:01:25	HLA06345		0	0	1	0	0	0	0	1									

Supplemental Table 8: HLA-A Allele Summary [a]				Allele Count by Population Group [b]								3.0.0 CIWD Category by Population Group [c]								
Allele	Genomic Typing	AlleleID	G group	AFA	API	EURO	MENA	HIS	NAM	UNK	Total	AFA	API	EURO	MENA	HIS	NAM	UNK	Total	Highest Frequency
A*03:01:28	03:01:28	HLA06562		2	0	9	0	1	0	2	14			WD					WD	WD
A*03:01:30	03:01:30	HLA06766		0	0	1	0	0	0	0	1									
A*03:01:31	03:01:31	HLA06772		0	0	8	0	0	0	0	8			WD					WD	WD
A*03:01:32	03:01:32	HLA07598		0	0	0	0	0	1	0	1									
A*03:01:33	03:01:33	HLA07602		0	0	6	0	0	0	2	8			WD					WD	WD
A*03:01:34	03:01:34	HLA08448		0	0	1	0	0	0	4	5								WD	WD
A*03:01:36	03:01:36	HLA08489		0	0	1	0	0	0	0	1									
A*03:01:38	03:01:38	HLA08641		1	0	4	0	0	0	1	6								WD	WD
A*03:01:39	03:01:39	HLA09118		0	0	5	0	0	0	0	5			WD					WD	WD
A*03:01:42	03:01:42	HLA09545		0	0	2	0	0	0	0	2									
A*03:01:43	03:01:43	HLA09546		0	5	0	1	0	0	0	6		WD						WD	WD
A*03:01:45	03:01:45	HLA10983		0	0	1	0	1	0	0	2									
A*03:01:51	03:01:51	HLA11804		0	0	0	0	1	0	1	2									
A*03:01:52	03:01:52	HLA12554		0	0	0	3	0	0	0	3									
A*03:01:53	03:01:53	HLA12640		0	1	0	0	0	0	0	1									
A*03:01:54	03:01:54	HLA12883		0	2	0	0	0	0	0	2									
A*03:01:55	03:01:55	HLA13452		0	0	4	0	0	0	0	4									
**A*03:02 total**	**03:02 total**			**439**	**6003**	**31494**	**10958**	**1519**	**104**	**8787**	**59304**	**C**	**C**	**C**	**C**	**C**	**C**	**C**	**C**	**C**
A*03:02	03:02			25	487	4021	1298	192	9	1849	7881	WD	C	C	C	C	C	C	C	C
A*03:02P	03:02P			0	0	120	0	0	0	1	121			I					WD	I
**A*03:02:01G total**	**03:02:01G total**			**414**	**5516**	**27352**	**9660**	**1327**	**95**	**6935**	**51299**	**C**	**C**	**C**	**C**	**C**	**C**	**C**	**C**	**C**
A*03:02:01G	03:02:01G		03:02:01G	199	3408	14330	5190	404	27	4701	28259	C	C	C	C	C	C	C	C	C
A*03:02:01	03:02:01	HLA00040	03:02:01G	215	2108	13022	4470	923	68	2234	23040	C	C	C	C	C	C	C	C	C
A*03:02:03	03:02:03	HLA07409		0	0	0	0	0	0	2	2									
A*03:02:04	03:02:04	HLA11969		0	0	1	0	0	0	0	1									
**A*03:04 total**	**03:04 total**			**1**	**0**	**9**	**0**	**8**	**0**	**2**	**20**			**WD**		**I**			**WD**	**I**
A*03:04	03:04			0	0	1	0	0	0	0	1									
A*03:04:01	03:04:01	HLA00042		1	0	6	0	8	0	2	17			WD		I			WD	I
A*03:04:02	03:04:02	HLA06087		0	0	1	0	0	0	0	1									
A*03:04:03	03:04:03	HLA08450		0	0	1	0	0	0	0	1									

Supplemental Table 8: HLA-A Allele Summary [a]				Allele Count by Population Group [b]								3.0.0 CIWD Category by Population Group [c]								
Allele	Genomic Typing	AlleleID	G group	AFA	API	EURO	MENA	HIS	NAM	UNK	Total	AFA	API	EURO	MENA	HIS	NAM	UNK	Total	Highest Frequency
**A*03:05 total**	**03:05 total**			**3**	**1**	**622**	**3**	**28**	**3**	**39**	**699**			**I**		**I**		**I**	**I**	**I**
A*03:05	03:05			3	0	96	0	5	0	4	108			WD		WD			WD	WD
A*03:05:01	03:05:01	HLA01107		0	1	526	3	23	3	35	591			I		I		I	I	I
A*03:06	03:06	HLA01251		2	0	8	0	0	0	2	12			WD					WD	WD
**A*03:07 total**	**03:07 total**			**9**	**0**	**112**	**0**	**16**	**3**	**8**	**148**	**WD**		**WD**		**I**		**WD**	**WD**	**I**
A*03:07	03:07			9	0	105	0	16	3	8	141	WD		WD		I		WD	WD	I
A*03:07:01	03:07:01	HLA01275		0	0	7	0	0	0	0	7			WD					WD	WD
A*03:08	03:08	HLA01302		12	1	110	2	2	2	15	144	WD		WD				I	WD	I
A*03:09	03:09	HLA01477		0	0	3	1	1	0	1	6								WD	WD
A*03:10	03:10	HLA01646		0	9	0	0	0	0	1	10		WD						WD	WD
A*03:11N	03:11N	HLA01714		0	0	4	0	0	0	0	4									
A*03:12	03:12	HLA01771		0	1	5	0	0	0	0	6			WD					WD	WD
A*03:13	03:13	HLA01826		0	0	8	0	0	0	0	8			WD					WD	WD
A*03:14	03:14	HLA01827		0	0	35	0	0	0	0	35			WD					WD	WD
A*03:15	03:15	HLA02109		0	0	52	0	2	0	1	55			WD					WD	WD
A*03:16	03:16	HLA02111		0	0	6	0	1	1	1	9			WD					WD	WD
**A*03:17 total**	**03:17 total**			**1**	**0**	**21**	**0**	**1**	**0**	**4**	**27**			**WD**					**WD**	**WD**
A*03:17	03:17			0	0	10	0	0	0	0	10			WD					WD	WD
A*03:17:01	03:17:01	HLA02197		0	0	11	0	1	0	4	16			WD					WD	WD
A*03:17:02	03:17:02	HLA07998		1	0	0	0	0	0	0	1									
A*03:18	03:18	HLA02290		0	1	2	5	0	0	0	8				WD				WD	WD
**A*03:22 total**	**03:22 total**			**2**	**2**	**113**	**1**	**2**	**0**	**2**	**122**			**WD**					**WD**	**WD**
A*03:22	03:22			0	0	11	0	0	0	0	11			WD					WD	WD
A*03:22:01	03:22:01	HLA02405		2	2	93	1	2	0	2	102			WD					WD	WD
A*03:22:02	03:22:02	HLA06873		0	0	9	0	0	0	0	9			WD					WD	WD
**A*03:23 total**	**03:23 total**			**0**	**0**	**12**	**0**	**0**	**0**	**0**	**12**			**WD**					**WD**	**WD**
A*03:23:01	03:23:01	HLA02528		0	0	2	0	0	0	0	2									
A*03:23:02	03:23:02	HLA06005		0	0	10	0	0	0	0	10			WD					WD	WD
A*03:24	03:24	HLA02590		3	0	0	0	0	0	1	4									
A*03:25	03:25	HLA02683		0	0	10	0	0	0	0	10			WD					WD	WD

Supplemental Table 8: HLA-A Allele Summary [a]				Allele Count by Population Group [b]								3.0.0 CIWD Category by Population Group [c]								
Allele	Genomic Typing	AlleleID	G group	AFA	API	EURO	MENA	HIS	NAM	UNK	Total	AFA	API	EURO	MENA	HIS	NAM	UNK	Total	Highest Frequency
A*03:27	03:27	HLA02779		0	0	8	0	0	0	0	8			WD					WD	WD
A*03:28	03:28	HLA02858		0	0	9	0	0	0	1	10			WD					WD	WD
A*03:29	03:29	HLA02894		0	0	16	0	0	0	5	21			WD				WD	WD	WD
A*03:31	03:31	HLA03002		0	0	3	0	0	0	1	4									
A*03:33	03:33	HLA03082		0	0	249	1	3	0	15	268			I				I	I	I
A*03:34	03:34	HLA03090		0	0	1	0	0	0	0	1									
A*03:36N	03:36N	HLA03119		0	0	0	0	0	0	1	1									
A*03:38	03:38	HLA03140		0	0	5	0	0	0	0	5			WD					WD	WD
A*03:41	03:41	HLA03435		0	1	0	0	0	0	0	1									
**A*03:44 total**	**03:44 total**			**0**	**0**	**3**	**0**	**0**	**0**	**0**	**3**									
A*03:44	03:44			0	0	1	0	0	0	0	1									
A*03:44:01	03:44:01	HLA03525		0	0	2	0	0	0	0	2									
A*03:46	03:46	HLA03568		0	0	5	0	0	0	0	5			WD					WD	WD
A*03:47	03:47	HLA03654		0	0	31	0	0	1	3	35			WD					WD	WD
A*03:48	03:48	HLA03667		0	0	37	0	0	0	0	37			WD					WD	WD
A*03:49	03:49	HLA03668		0	0	9	0	0	0	1	10			WD					WD	WD
A*03:50	03:50	HLA03709		0	0	53	0	0	0	2	55			WD					WD	WD
A*03:51	03:51	HLA03710		13	0	0	0	0	0	2	15	WD							WD	WD
A*03:52	03:52	HLA03870		0	0	2	0	0	0	1	3									
A*03:53	03:53	HLA03874		0	0	12	0	0	0	3	15			WD					WD	WD
A*03:54	03:54	HLA03894		0	0	1	0	0	0	0	1									
A*03:55	03:55	HLA03914		0	0	3	0	0	0	0	3									
A*03:56	03:56	HLA03917		0	0	11	0	0	0	2	13			WD					WD	WD
A*03:57	03:57	HLA03922		1	0	4	0	0	0	0	5								WD	WD
A*03:58	03:58	HLA03929		0	0	1	0	0	0	0	1									
A*03:59	03:59	HLA03931		0	0	3	0	0	0	1	4									
A*03:62	03:62	HLA04142		0	1	12	0	0	0	5	18			WD				WD	WD	WD
A*03:63	03:63	HLA04490		0	0	6	0	0	0	2	8			WD					WD	WD
A*03:65	03:65	HLA04528		0	0	7	0	0	0	0	7			WD					WD	WD
A*03:67	03:67	HLA04559		0	0	1	0	0	0	0	1									

Supplemental Table 8: HLA-A Allele Summary [a]				Allele Count by Population Group [b]								3.0.0 CIWD Category by Population Group [c]								
Allele	Genomic Typing	AlleleID	G group	AFA	API	EURO	MENA	HIS	NAM	UNK	Total	AFA	API	EURO	MENA	HIS	NAM	UNK	Total	Highest Frequency
A*03:68N	03:68N	HLA04561		0	0	2	0	0	0	0	2									
A*03:69N	03:69N	HLA04562		0	0	3	0	0	0	0	3									
A*03:71	03:71	HLA04578		0	0	1	1	0	0	0	2									
A*03:72	03:72	HLA04636		0	0	3	0	0	0	0	3									
A*03:74	03:74	HLA04647		0	0	70	0	0	0	0	70			WD					WD	WD
A*03:77	03:77	HLA05232		0	1	0	0	0	0	0	1									
A*03:79	03:79	HLA05314		0	0	2	0	0	0	0	2									
A*03:81	03:81	HLA05336		0	0	13	0	1	0	0	14			WD					WD	WD
A*03:85	03:85	HLA05441		0	0	0	1	0	0	0	1									
A*03:86	03:86	HLA05456		0	0	5	0	0	0	0	5			WD					WD	WD
A*03:87	03:87	HLA05462		0	0	7	0	0	0	0	7			WD					WD	WD
A*03:88	03:88	HLA05547		0	0	2	0	1	0	1	4									
**A*03:89 total**	**03:89 total**			**1**	**0**	**35**	**0**	**4**	**0**	**1**	**41**			**WD**					**WD**	**WD**
A*03:89	03:89			1	0	33	0	3	0	1	38			WD					WD	WD
A*03:89:01	03:89:01	HLA05552		0	0	2	0	1	0	0	3									
A*03:92	03:92	HLA05631		0	0	2	0	0	0	0	2									
A*03:93	03:93	HLA05636		0	0	19	0	0	0	2	21			WD					WD	WD
A*03:94	03:94	HLA05638		0	0	15	0	0	0	0	15			WD					WD	WD
A*03:95	03:95	HLA05643		0	0	1	0	0	0	0	1									
A*03:96	03:96	HLA05646		0	2	0	0	0	0	2	4									
A*03:97	03:97	HLA05647		0	0	4	0	0	0	0	4									
A*03:98	03:98	HLA05651		0	0	1	0	0	0	0	1									
A*03:99	03:99	HLA05653		0	0	2	0	0	0	0	2									
A*03:100	03:100	HLA05663		0	0	0	1	0	0	0	1									
A*03:101	03:101	HLA05664		0	0	1	0	0	0	0	1									
A*03:102	03:102	HLA05675		0	0	11	0	0	0	0	11			WD					WD	WD
**A*03:103 total**	**03:103 total**			**0**	**1**	**4**	**0**	**0**	**0**	**0**	**5**								**WD**	**WD**
A*03:103	03:103			0	1	4	0	0	0	0	5								WD	WD
A*03:105	03:105	HLA05701		0	0	2	0	0	0	0	2									
A*03:107	03:107	HLA05704		0	0	9	0	0	0	0	9			WD					WD	WD

Supplemental Table 8: HLA-A Allele Summary [a]				Allele Count by Population Group [b]								3.0.0 CIWD Category by Population Group [c]								
Allele	Genomic Typing	AlleleID	G group	AFA	API	EURO	MENA	HIS	NAM	UNK	Total	AFA	API	EURO	MENA	HIS	NAM	UNK	Total	Highest Frequency
A*03:108	03:108	HLA05705		0	0	7	1	0	0	0	8			WD					WD	WD
A*03:110	03:110	HLA05724		0	0	1	0	0	0	0	1									
A*03:111	03:111	HLA05730		0	0	6	0	0	0	0	6			WD					WD	WD
A*03:113	03:113	HLA05935		0	1	0	0	0	0	0	1									
A*03:115	03:115	HLA05990		0	0	1	0	0	0	0	1									
A*03:116	03:116	HLA06095		0	0	0	0	0	0	1	1									
A*03:119	03:119	HLA06550		0	0	2	0	1	0	0	3									
A*03:120	03:120	HLA06551		0	0	6	0	0	0	0	6			WD					WD	WD
A*03:122	03:122	HLA06560		0	0	1	0	0	0	0	1									
**A*03:123 total**	**03:123 total**			**0**	**0**	**31**	**0**	**0**	**0**	**4**	**35**			**WD**					**WD**	**WD**
A*03:123	03:123			0	0	2	0	0	0	0	2									
A*03:123:01	03:123:01	HLA06771		0	0	29	0	0	0	4	33			WD					WD	WD
A*03:124	03:124	HLA06753		0	0	2	0	0	0	0	2									
A*03:125	03:125	HLA06755		0	0	1	0	0	0	1	2									
A*03:126	03:126	HLA06788		0	0	2	0	0	0	0	2									
A*03:127	03:127	HLA06804		0	0	0	0	0	0	2	2									
A*03:128	03:128	HLA06881		0	0	2	0	0	0	0	2									
A*03:130	03:130	HLA06910		0	0	1	0	0	0	0	1									
A*03:133	03:133	HLA07297		1	0	0	0	0	0	0	1									
A*03:135	03:135	HLA07405		0	0	1	0	0	0	0	1									
A*03:136	03:136	HLA07427		0	0	1	0	0	0	0	1									
A*03:137	03:137	HLA07595		0	1	1	0	0	0	0	2									
A*03:139	03:139	HLA07600		0	0	1	0	1	0	1	3									
A*03:140	03:140	HLA07601		0	0	0	0	2	0	0	2									
A*03:141	03:141	HLA07675		0	0	1	0	0	0	0	1									
A*03:142	03:142	HLA07676		0	3	0	0	0	0	0	3									
A*03:144	03:144	HLA07990		0	0	6	0	0	0	0	6			WD					WD	WD
A*03:147	03:147	HLA07999		0	0	2	0	0	0	0	2									
A*03:149	03:149	HLA08001		0	0	5	0	0	0	0	5			WD					WD	WD
A*03:150	03:150	HLA08020		0	0	1	0	0	0	0	1									

Supplemental Table 8: HLA-A Allele Summary [a]				Allele Count by Population Group [b]								3.0.0 CIWD Category by Population Group [c]								
Allele	Genomic Typing	AlleleID	G group	AFA	API	EURO	MENA	HIS	NAM	UNK	Total	AFA	API	EURO	MENA	HIS	NAM	UNK	Total	Highest Frequency
A*03:151	03:151	HLA08281		0	2	0	0	0	0	0	2									
A*03:153	03:153	HLA08449		0	0	1	0	0	0	0	1									
**A*03:154 total**	**03:154 total**			**0**	**2**	**4**	**0**	**0**	**0**	**1**	**7**								**WD**	**WD**
A*03:154	03:154			0	2	3	0	0	0	1	6								WD	WD
A*03:154:01	03:154:01	HLA08451		0	0	1	0	0	0	0	1									
A*03:155	03:155	HLA08452		0	0	1	0	0	0	0	1									
**A*03:157 total**	**03:157 total**			**0**	**0**	**6**	**0**	**0**	**0**	**0**	**6**			**WD**					**WD**	**WD**
A*03:157:01	03:157:01	HLA08487		0	0	2	0	0	0	0	2									
A*03:157:02	03:157:02	HLA12465		0	0	4	0	0	0	0	4									
A*03:158	03:158	HLA08490		1	0	0	0	0	0	0	1									
A*03:163	03:163	HLA09337		0	0	1	0	0	0	1	2									
A*03:166	03:166	HLA09420		0	0	1	0	0	0	0	1									
A*03:167	03:167	HLA09497		0	0	4	1	0	0	0	5								WD	WD
A*03:172	03:172	HLA09804		0	0	3	0	0	0	0	3									
A*03:173	03:173	HLA09805		0	0	3	0	0	0	0	3									
A*03:174	03:174	HLA09806		0	2	0	0	0	0	0	2									
A*03:176	03:176	HLA10387		0	0	3	0	0	0	0	3									
A*03:177	03:177	HLA10388		0	0	0	0	0	0	1	1									
A*03:180	03:180	HLA10398		0	0	1	0	0	0	0	1									
A*03:185	03:185	HLA10982		1	0	0	0	0	0	0	1									
A*03:186	03:186	HLA11310		0	0	0	0	0	0	2	2									
A*03:188	03:188	HLA11314		0	0	2	0	0	0	0	2									
A*03:191	03:191	HLA11805		0	0	2	0	0	0	0	2									
A*03:192N	03:192N	HLA11992		0	0	1	0	0	0	0	1									
A*03:195	03:195	HLA11965		0	0	2	0	0	0	0	2									
A*03:197N	03:197N	HLA11970		0	0	0	1	0	0	0	1									
A*03:198	03:198	HLA12082		0	0	0	1	0	0	0	1									
A*03:201	03:201	HLA12247		0	0	0	0	1	0	1	2									
A*03:202	03:202	HLA12250		0	0	1	13	0	0	0	14				WD				WD	WD
A*03:204	03:204	HLA12400		0	0	1	0	0	0	0	1									

Supplemental Table 8: HLA-A Allele Summary [a]				Allele Count by Population Group [b]								3.0.0 CIWD Category by Population Group [c]								
Allele	Genomic Typing	AlleleID	G group	AFA	API	EURO	MENA	HIS	NAM	UNK	Total	AFA	API	EURO	MENA	HIS	NAM	UNK	Total	Highest Frequency
A*03:206	03:206	HLA12461		0	0	0	0	1	0	0	1									
A*03:207	03:207	HLA12552		0	0	1	0	0	0	1	2									
A*03:210	03:210	HLA12958		0	0	2	0	0	0	0	2									
A*03:212	03:212	HLA12965		0	0	1	0	0	0	0	1									
A*03:213	03:213	HLA12966		0	0	3	0	0	0	1	4									
A*03:214	03:214	HLA12967		1	0	1	0	0	0	0	2									
A*03:215	03:215	HLA12968		0	2	2	0	0	0	0	4									
A*03:216	03:216	HLA12969		0	0	0	5	0	0	0	5				WD				WD	WD
A*03:217	03:217	HLA12972		0	2	0	0	0	0	0	2									
A*03:219	03:219	HLA13079		0	0	0	1	0	0	0	1									
A*03:224	03:224	HLA13619		0	0	1	0	0	0	0	1									
A*03:226	03:226	HLA13647		0	0	1	0	0	0	0	1									
A*03:227	03:227	HLA13701		0	0	1	0	0	0	0	1									
A*03:228	03:228	HLA13730		0	5	0	0	0	0	0	5		WD						WD	WD
A*03:230	03:230	HLA13847		0	0	1	0	0	0	0	1									
A*03:233	03:233	HLA14129		1	0	0	0	0	0	1	2									
A*03:238	03:238	HLA14506		0	0	1	0	0	0	0	1									
A*03:243	03:243	HLA14704		0	0	1	0	0	0	0	1									
A*03:245	03:245	HLA14717		0	0	0	0	0	0	1	1									
A*03:251	03:251	HLA15521		0	0	1	0	0	0	0	1									
A*03:258	03:258	HLA15819		0	0	1	0	0	0	0	1									
A*03:261	03:261	HLA15836		0	0	1	0	0	0	0	1									
A*03:262N	03:262N	HLA15840		0	0	1	0	0	0	0	1									
A*03:266N	03:266N	HLA16071		0	0	4	0	0	0	0	4									
A*03:271	03:271	HLA16204		0	0	1	0	0	0	0	1									
A*03:CODE	03:CODE			3548	1721	105344	1832	6316	587	12119	131467	NA	NA	NA	NA	NA	NA	NA	NA	NA
**A*11:01 total**	**11:01 total**			**5016**	**206873**	**637496**	**26855**	**29261**	**2630**	**73685**	**981816**	**C**	**C**	**C**	**C**	**C**	**C**	**C**	**C**	**C**
A*11:01	11:01			3	35	3310	12	15	0	161	3536		I	C	WD	I		C	C	C
A*11:01P	11:01P			0	7	752	3	4	0	3	769		WD	I					I	I
**A*11:01:01G total**	**11:01:01G total**			**5010**	**206642**	**633330**	**26837**	**29241**	**2630**	**73502**	**977192**	**C**	**C**	**C**	**C**	**C**	**C**	**C**	**C**	**C**

Supplemental Table 8: HLA-A Allele Summary [a]				Allele Count by Population Group [b]								3.0.0 CIWD Category by Population Group [c]								
Allele	Genomic Typing	AlleleID	G group	AFA	API	EURO	MENA	HIS	NAM	UNK	Total	AFA	API	EURO	MENA	HIS	NAM	UNK	Total	Highest Frequency
A*11:01:01G	11:01:01G		11:01:01G	3842	186234	598969	26029	20246	1927	64911	902158	C	C	C	C	C	C	C	C	C
A*11:01:01	11:01:01		11:01:01G	455	8906	12936	313	3685	300	4273	30868	C	C	C	C	C	C	C	C	C
A*11:01:01:01	11:01:01:01	HLA00043	11:01:01G	713	11495	21418	495	5296	403	4314	44134	C	C	C	C	C	C	C	C	C
A*11:21N	11:21N	HLA02052	11:01:01G	0	2	1	0	0	0	2	5								WD	WD
A*11:86	11:86	HLA06027	11:01:01G	0	0	4	0	0	0	1	5								WD	WD
A*11:102	11:102	HLA06527	11:01:01G	0	1	0	0	0	0	0	1									
A*11:108	11:108	HLA07288	11:01:01G	0	1	1	0	14	0	1	17					I			WD	I
A*11:126	11:126	HLA08104	11:01:01G	0	2	1	0	0	0	0	3									
A*11:129	11:129	HLA08309	11:01:01G	0	1	0	0	0	0	0	1									
A*11:01:02	11:01:02	HLA01037		0	46	0	0	0	0	1	47		I						WD	I
A*11:01:04	11:01:04	HLA02025		1	40	0	0	0	0	4	45		I						WD	I
A*11:01:05	11:01:05	HLA02216		0	5	1	0	0	0	2	8		WD						WD	WD
A*11:01:06	11:01:06	HLA02723		0	17	0	0	0	0	0	17		I						WD	I
A*11:01:07	11:01:07	HLA02829		0	6	1	1	0	0	0	8		WD						WD	WD
A*11:01:08	11:01:08	HLA03124		0	0	11	0	0	0	0	11			WD					WD	WD
A*11:01:10	11:01:10	HLA03494		0	0	0	0	0	0	1	1									
A*11:01:11	11:01:11	HLA03803		0	0	15	0	1	0	0	16			WD					WD	WD
A*11:01:13	11:01:13	HLA03910		0	20	2	0	0	0	6	28		I					WD	WD	I
A*11:01:14	11:01:14	HLA03928		0	1	2	0	0	0	0	3									
A*11:01:15	11:01:15	HLA04476		0	3	28	0	0	0	3	34			WD					WD	WD
A*11:01:17	11:01:17	HLA04855		0	0	9	0	0	0	1	10			WD					WD	WD
A*11:01:19	11:01:19	HLA05246		0	2	0	0	0	0	0	2									
A*11:01:20	11:01:20	HLA05318		0	0	1	0	0	0	0	1									
A*11:01:24	11:01:24	HLA05540		0	1	0	0	0	0	0	1									
A*11:01:25	11:01:25	HLA05629		0	20	0	0	0	0	0	20		I						WD	I
A*11:01:27	11:01:27	HLA05727		0	1	5	0	0	0	0	6			WD					WD	WD
A*11:01:30	11:01:30	HLA06740		0	2	0	0	0	0	1	3									
A*11:01:34	11:01:34	HLA07101		0	2	0	0	0	0	0	2									
A*11:01:35	11:01:35	HLA07295		0	5	0	0	0	0	0	5		WD						WD	WD
A*11:01:36	11:01:36	HLA07429		2	0	11	0	0	0	2	15			WD					WD	WD

Supplemental Table 8: HLA-A Allele Summary [a]				Allele Count by Population Group [b]								3.0.0 CIWD Category by Population Group [c]								
Allele	Genomic Typing	AlleleID	G group	AFA	API	EURO	MENA	HIS	NAM	UNK	Total	AFA	API	EURO	MENA	HIS	NAM	UNK	Total	Highest Frequency
A*11:01:38	11:01:38	HLA07575		0	0	6	1	0	0	0	7			WD					WD	WD
A*11:01:39	11:01:39	HLA07577		0	1	0	0	0	0	0	1									
A*11:01:40	11:01:40	HLA07672		0	2	0	0	0	0	0	2									
A*11:01:41	11:01:41	HLA07982		0	1	0	0	0	0	0	1									
A*11:01:42	11:01:42	HLA08257		0	1	2	0	0	0	0	3									
A*11:01:43	11:01:43	HLA08258		0	0	3	0	0	0	0	3									
A*11:01:44	11:01:44	HLA08260		0	1	5	0	0	0	0	6			WD					WD	WD
A*11:01:45	11:01:45	HLA08266		0	3	0	0	0	0	0	3									
A*11:01:48	11:01:48	HLA09523		0	0	0	1	0	0	0	1									
A*11:01:57	11:01:57	HLA10885		0	1	0	0	0	0	0	1									
A*11:01:60	11:01:60	HLA10964		0	1	0	0	0	0	0	1									
A*11:01:61	11:01:61	HLA10965		0	1	0	0	0	0	0	1									
A*11:01:62	11:01:62	HLA10969		0	0	1	0	0	0	0	1									
A*11:01:63	11:01:63	HLA11633		0	1	0	0	0	0	0	1									
A*11:01:65	11:01:65	HLA12104		0	2	1	0	0	0	0	3									
A*11:01:66	11:01:66	HLA12626		0	1	0	0	0	0	0	1									
A*11:01:68	11:01:68	HLA13437		0	1	0	0	0	0	0	1									
A*11:01:69	11:01:69	HLA14388		0	0	1	0	0	0	0	1									
A*11:01:70	11:01:70	HLA14639		0	2	0	0	0	0	0	2									
A*11:107	11:107	HLA07106		0	1	0	0	0	0	0	1									
**A*11:02 total**	**11:02 total**			**21**	**7959**	**203**	**33**	**31**	**5**	**700**	**8952**	**WD**	**C**	**I**	**WD**	**I**	**WD**	**C**	**C**	**C**
A*11:02	11:02			0	24	5	0	0	0	6	35		I	WD				WD	WD	I
**A*11:02:01G total**	**11:02:01G total**			**21**	**7931**	**198**	**33**	**31**	**5**	**693**	**8912**	**WD**	**C**	**I**	**WD**	**I**	**WD**	**C**	**C**	**C**
A*11:02:01G	11:02:01G		11:02:01G	18	6950	191	32	28	5	563	7787	WD	C	I	WD	I	WD	C	C	C
A*11:02:01	11:02:01	HLA00044	11:02:01G	3	981	7	1	3	0	130	1125		C	WD				I	I	C
A*11:02:02	11:02:02	HLA02442		0	4	0	0	0	0	0	4									
A*11:02:04	11:02:04	HLA09301		0	0	0	0	0	0	1	1									
**A*11:03 total**	**11:03 total**			**19**	**1855**	**31**	**26**	**7**	**34**	**60**	**2032**	**WD**	**C**	**WD**	**WD**	**D**	**C**	**I**	**C**	**C**
**A*11:03:01G total**	**11:03:01G total**			**19**	**1855**	**31**	**26**	**7**	**34**	**60**	**2032**	**WD**	**C**	**WD**	**WD**	**D**	**C**	**I**	**C**	**C**
A*11:03	11:03	HLA00045	11:03:01G	11	426	12	4	5	23	32	513	WD	C	WD		WD	C	I	I	C

Supplemental Table 8: HLA-A Allele Summary [a]				Allele Count by Population Group [b]								3.0.0 CIWD Category by Population Group [c]								
**Allele**	**Genomic Typing**	**AlleleID**	**G group**	**AFA**	**API**	**EURO**	**MENA**	**HIS**	**NAM**	**UNK**	**Total**	**AFA**	**API**	**EURO**	**MENA**	**HIS**	**NAM**	**UNK**	**Total**	**Highest Frequency**
A*11:03:01G	11:03:01G		11:03:01G	8	1429	19	22	2	11	28	1519	WD	C	WD	WD		C	I	I	C
A*11:04	11:04	HLA00046		2	316	201	4	0	0	26	549		C	I				I	I	C
**A*11:05 total**	**11:05 total**			**7**	**2**	**252**	**1**	**27**	**2**	**44**	**335**	**WD**		**I**		**I**		**I**	**I**	**I**
**A*11:05:01G total**	**11:05:01G total**			**7**	**2**	**252**	**1**	**27**	**2**	**44**	**335**	**WD**		**I**		**I**		**I**	**I**	**I**
A*11:05	11:05	HLA00047	11:05:01G	6	2	226	1	23	2	39	299	WD		I		I		I	I	I
A*11:05:01G	11:05:01G		11:05:01G	0	0	17	0	0	0	4	21			WD					WD	WD
A*11:05P	11:05P			0	0	1	0	0	0	0	1									
A*11:248	11:248	HLA15608	11:05:01G	1	0	8	0	4	0	1	14			WD					WD	WD
A*11:06	11:06	HLA01104		0	0	4	0	0	0	1	5								WD	WD
A*11:07	11:07	HLA01297		0	0	1	0	0	0	0	1									
A*11:08	11:08	HLA01298		2	2	14	0	20	1	7	46			WD		I		WD	WD	I
A*11:09	11:09	HLA01310		1	0	85	0	0	0	4	90			WD					WD	WD
A*11:10	11:10	HLA01423		0	1	12	1	0	0	0	14			WD					WD	WD
A*11:11	11:11	HLA01547		0	0	2	0	0	0	0	2									
A*11:12	11:12	HLA01556		0	8	399	0	12	0	43	462		WD	I		I		I	I	I
A*11:14	11:14	HLA01647		0	1	0	0	0	0	0	1									
**A*11:15 total**	**11:15 total**			**0**	**6**	**21**	**0**	**0**	**0**	**0**	**27**		**WD**	**WD**					**WD**	**WD**
A*11:15:01	11:15:01	HLA01815		0	6	0	0	0	0	0	6		WD						WD	WD
A*11:15:02	11:15:02	HLA03112		0	0	21	0	0	0	0	21			WD					WD	WD
A*11:16	11:16	HLA01829		0	3	0	0	0	0	0	3									
A*11:17	11:17	HLA01839		0	2	0	0	0	0	0	2									
A*11:18	11:18	HLA01973		0	1	16	19	0	0	0	36			WD	WD				WD	WD
A*11:19	11:19	HLA01987		0	22	6	1	0	0	0	29		I	WD					WD	I
A*11:20	11:20	HLA02045		0	17	2	0	0	0	4	23		I						WD	I
A*11:23	11:23	HLA02124		0	0	0	0	0	0	2	2									
**A*11:24 total**	**11:24 total**			**0**	**0**	**3**	**0**	**0**	**0**	**0**	**3**									
A*11:24:01	11:24:01	HLA02386		0	0	3	0	0	0	0	3									
**A*11:25 total**	**11:25 total**			**0**	**0**	**6**	**0**	**0**	**0**	**1**	**7**			**WD**					**WD**	**WD**
A*11:25	11:25			0	0	4	0	0	0	0	4									
A*11:25:01	11:25:01	HLA02456		0	0	2	0	0	0	0	2									

Supplemental Table 8: HLA-A Allele Summary [a]				Allele Count by Population Group [b]								3.0.0 CIWD Category by Population Group [c]								
Allele	Genomic Typing	AlleleID	G group	AFA	API	EURO	MENA	HIS	NAM	UNK	Total	AFA	API	EURO	MENA	HIS	NAM	UNK	Total	Highest Frequency
A*11:25:02	11:25:02	HLA13835		0	0	0	0	0	0	1	1									
A*11:27	11:27	HLA02530		0	2	0	0	0	0	0	2									
A*11:29	11:29	HLA02601		0	0	132	1	0	0	5	138			I				WD	WD	I
A*11:30	11:30	HLA02775		0	0	32	0	4	0	4	40			WD					WD	WD
A*11:31	11:31	HLA02921		0	0	1	0	0	0	0	1									
**A*11:32 total**	**11:32 total**			**0**	**3**	**3**	**0**	**0**	**0**	**1**	**7**								**WD**	**WD**
A*11:32	11:32			0	1	1	0	0	0	1	3									
A*11:32P	11:32P			0	0	1	0	0	0	0	1									
A*11:32:01	11:32:01	HLA02975		0	2	1	0	0	0	0	3									
A*11:34	11:34	HLA03351		0	0	4	0	0	0	0	4									
A*11:36	11:36	HLA03394		0	1	1	0	0	0	0	2									
A*11:37	11:37	HLA03422		0	1	21	0	0	0	0	22			WD					WD	WD
A*11:39	11:39	HLA03532		0	2	12	0	0	0	1	15			WD					WD	WD
A*11:40	11:40	HLA03576		0	0	1	0	0	0	0	1									
A*11:41	11:41	HLA03686		0	4	0	0	0	0	1	5								WD	WD
A*11:42	11:42	HLA03690		0	1	1	0	0	0	0	2									
A*11:43	11:43	HLA03750		0	1	0	0	0	0	0	1									
A*11:44	11:44	HLA03814		0	56	1	0	0	0	0	57		I						WD	I
A*11:45	11:45	HLA03878		0	0	0	5	0	0	0	5				WD				WD	WD
A*11:46	11:46	HLA03890		0	0	2	0	0	0	0	2									
A*11:47	11:47	HLA03918		0	1	47	0	0	0	1	49			WD					WD	WD
A*11:50Q	11:50Q	HLA04135		0	0	4	0	0	0	0	4									
A*11:51	11:51	HLA04147		0	0	0	0	0	0	1	1									
A*11:54	11:54	HLA04465		0	0	0	4	0	0	0	4									
A*11:58	11:58	HLA04772		0	125	0	0	0	0	2	127		I						WD	I
A*11:60	11:60	HLA05116		0	0	1	0	0	0	0	1									
A*11:63	11:63	HLA05313		0	0	3	0	0	0	0	3									
A*11:64	11:64	HLA05322		0	8	0	0	0	0	0	8		WD						WD	WD
A*11:66	11:66	HLA05450		0	3	0	0	0	0	1	4									
A*11:67	11:67	HLA05455		0	0	7	0	2	0	2	11			WD					WD	WD

Supplemental Table 8: HLA-A Allele Summary [a]				Allele Count by Population Group [b]								3.0.0 CIWD Category by Population Group [c]								
Allele	Genomic Typing	AlleleID	G group	AFA	API	EURO	MENA	HIS	NAM	UNK	Total	AFA	API	EURO	MENA	HIS	NAM	UNK	Total	Highest Frequency
**A*11:70 total**	**11:70 total**			**0**	**0**	**5**	**0**	**0**	**0**	**0**	**5**			**WD**					**WD**	**WD**
A*11:70	11:70			0	0	1	0	0	0	0	1									
A*11:70:01	11:70:01	HLA05545		0	0	1	0	0	0	0	1									
A*11:70:02	11:70:02	HLA11207		0	0	3	0	0	0	0	3									
A*11:71	11:71	HLA05659		0	4	0	0	0	0	0	4									
A*11:72	11:72	HLA05678		0	0	1	2	0	0	0	3									
A*11:73	11:73	HLA05699		0	1	1	0	0	0	1	3									
A*11:74	11:74	HLA05728		0	0	9	0	0	0	0	9			WD					WD	WD
A*11:81	11:81	HLA05931		0	0	21	0	0	0	0	21			WD					WD	WD
A*11:82	11:82	HLA05939		0	0	0	0	0	0	1	1									
A*11:83	11:83	HLA05940		0	0	3	0	0	0	1	4									
A*11:84	11:84	HLA05985		0	0	2	0	0	0	0	2									
A*11:85	11:85	HLA06003		0	1	0	0	0	0	0	1									
A*11:88	11:88	HLA06110		0	2	0	0	0	0	0	2									
A*11:89	11:89	HLA06085		0	27	0	0	0	0	0	27		I						WD	I
**A*11:91 total**	**11:91 total**			**0**	**0**	**10**	**0**	**0**	**0**	**0**	**10**			**WD**					**WD**	**WD**
A*11:91:01	11:91:01	HLA06101		0	0	10	0	0	0	0	10			WD					WD	WD
A*11:92	11:92	HLA06104		0	0	5	0	0	0	1	6			WD					WD	WD
A*11:95	11:95	HLA06339		0	1	0	0	0	0	0	1									
A*11:98	11:98	HLA06367		0	0	2	0	1	0	0	3									
A*11:103	11:103	HLA06555		0	0	3	0	0	0	0	3									
A*11:105	11:105	HLA06809		0	0	1	0	0	0	0	1									
A*11:106	11:106	HLA06797		0	0	2	0	0	0	0	2									
A*11:109N	11:109N	HLA07294		0	0	7	0	0	0	0	7			WD					WD	WD
A*11:111	11:111	HLA07394		0	0	0	1	0	0	0	1									
A*11:112	11:112	HLA07410		0	2	0	0	0	0	0	2									
A*11:115N	11:115N	HLA07574		0	1	0	0	0	0	0	1									
A*11:117	11:117	HLA07578		0	0	5	0	0	0	0	5			WD					WD	WD
A*11:118	11:118	HLA07579		0	1	0	0	0	0	0	1									
**A*11:119 total**	**11:119 total**			**0**	**1**	**1**	**0**	**0**	**0**	**1**	**3**									

Supplemental Table 8: HLA-A Allele Summary [a]				Allele Count by Population Group [b]								3.0.0 CIWD Category by Population Group [c]								
Allele	Genomic Typing	AlleleID	G group	AFA	API	EURO	MENA	HIS	NAM	UNK	Total	AFA	API	EURO	MENA	HIS	NAM	UNK	Total	Highest Frequency
A*11:119:01	11:119:01	HLA07668		0	1	1	0	0	0	1	3									
A*11:122	11:122	HLA07818		0	0	1	0	0	0	0	1									
A*11:131	11:131	HLA08614		0	0	3	0	0	0	0	3									
A*11:132	11:132	HLA08618		0	0	1	0	0	0	0	1									
A*11:134	11:134	HLA08930		0	1	0	0	0	0	0	1									
A*11:135	11:135	HLA08941		0	0	3	0	0	0	0	3									
A*11:136	11:136	HLA08942		0	1	0	0	0	0	0	1									
**A*11:137N total**	**11:137N total**			**0**	**0**	**4**	**0**	**0**	**0**	**0**	**4**									
A*11:137N	11:137N			0	0	4	0	0	0	0	4									
A*11:138	11:138	HLA08944		0	0	0	0	1	0	0	1									
A*11:147	11:147	HLA09780		0	1	0	0	0	0	0	1									
A*11:148	11:148	HLA09781		0	2	0	0	0	0	0	2									
A*11:149	11:149	HLA09782		0	0	1	0	0	0	0	1									
A*11:150	11:150	HLA09788		0	0	3	0	0	0	0	3									
**A*11:153 total**	**11:153 total**			**0**	**0**	**3**	**0**	**0**	**0**	**1**	**4**									
A*11:153:01	11:153:01	HLA10201		0	0	1	0	0	0	0	1									
A*11:153:02	11:153:02	HLA10791		0	0	2	0	0	0	1	3									
A*11:159	11:159	HLA10376		0	8	0	0	0	0	0	8		WD						WD	WD
A*11:160	11:160	HLA10377		0	1	2	2	0	0	0	5								WD	WD
A*11:161	11:161	HLA10378		0	5	0	0	0	0	0	5		WD						WD	WD
A*11:162	11:162	HLA10646		0	0	0	1	0	0	0	1									
A*11:182Q	11:182Q	HLA10942		0	1	0	0	0	0	0	1									
A*11:183	11:183	HLA10955		0	24	0	0	0	0	2	26		I						WD	I
A*11:184	11:184	HLA10966		0	0	4	0	0	0	0	4									
A*11:185	11:185	HLA10967		0	1	0	0	0	0	0	1									
A*11:187	11:187	HLA10970		0	9	0	0	0	0	0	9		WD						WD	WD
A*11:189	11:189	HLA11206		0	0	1	0	0	0	0	1									
A*11:190	11:190	HLA11298		0	2	0	0	0	0	1	3									
A*11:192	11:192	HLA11475		0	0	1	0	0	0	0	1									
A*11:195	11:195	HLA11783		0	1	0	0	0	0	0	1									

Supplemental Table 8: HLA-A Allele Summary [a]				Allele Count by Population Group [b]								3.0.0 CIWD Category by Population Group [c]								
Allele	Genomic Typing	AlleleID	G group	AFA	API	EURO	MENA	HIS	NAM	UNK	Total	AFA	API	EURO	MENA	HIS	NAM	UNK	Total	Highest Frequency
A*11:196	11:196	HLA11784		0	2	0	0	0	0	0	2									
**A*11:199 total**	**11:199 total**			**0**	**1**	**3**	**0**	**0**	**0**	**0**	**4**									
A*11:199	11:199			0	1	3	0	0	0	0	4									
A*11:201	11:201	HLA11984		0	1	0	0	0	0	0	1									
A*11:202	11:202	HLA11986		0	1	0	0	0	0	0	1									
A*11:205	11:205	HLA12103		0	3	0	0	0	0	0	3									
A*11:207	11:207	HLA12243		0	0	1	0	1	0	0	2									
A*11:208N	11:208N	HLA12423		0	1	0	0	0	0	0	1									
A*11:211	11:211	HLA12627		0	10	0	0	0	0	0	10		WD						WD	WD
A*11:214	11:214	HLA12938		0	1	0	0	0	0	0	1									
A*11:219	11:219	HLA13642		0	0	1	0	2	0	0	3									
A*11:221	11:221	HLA13702		0	0	5	0	0	0	0	5			WD					WD	WD
A*11:223	11:223	HLA13723		0	3	0	0	0	0	0	3									
A*11:224	11:224	HLA13945		0	3	0	0	0	0	0	3									
A*11:226	11:226	HLA13948		0	2	1	0	0	0	0	3									
A*11:233	11:233	HLA14430		0	3	0	0	0	0	0	3									
A*11:236	11:236	HLA14708		0	1	0	0	0	0	0	1									
A*11:260	11:260	HLA16632		0	0	1	0	0	0	0	1									
A*11:CODE	11:CODE			545	5653	47279	984	3700	289	6675	65125	NA	NA	NA	NA	NA	NA	NA	NA	NA
**A*23:01 total**	**23:01 total**			**36259**	**6029**	**227588**	**13116**	**19021**	**2209**	**33141**	**337363**	**C**	**C**	**C**	**C**	**C**	**C**	**C**	**C**	**C**
A*23:01	23:01			3	1	1377	10	9	0	105	1505			C	WD	I		I	I	C
A*23:01P	23:01P			2	2	429	2	3	0	2	440			I					I	I
**A*23:01:01G total**	**23:01:01G total**			**36245**	**6025**	**225704**	**13102**	**19007**	**2209**	**33026**	**335318**	**C**	**C**	**C**	**C**	**C**	**C**	**C**	**C**	**C**
A*23:01:01G	23:01:01G		23:01:01G	29796	5685	216097	12813	14564	1654	30261	310870	C	C	C	C	C	C	C	C	C
A*23:01:01	23:01:01		23:01:01G	4960	327	8801	245	3788	465	2267	20853	C	C	C	C	C	C	C	C	C
A*23:01:01:01	23:01:01:01	HLA00048	23:01:01G	293	8	712	30	224	15	191	1473	C	WD	I	WD	C	C	C	I	C
A*23:01:01:02	23:01:01:02	HLA16429	23:01:01G	1	0	5	0	8	0	4	18			WD		I			WD	I
A*23:01:01:03	23:01:01:03	HLA16403	23:01:01G	0	0	0	0	0	0	1	1									
A*23:07N	23:07N	HLA01470	23:01:01G	0	0	1	0	1	0	0	2									
A*23:17	23:17	HLA03165	23:01:01G	1195	5	67	14	416	75	300	2072	C	WD	WD	WD	C	C	C	C	C

Supplemental Table 8: HLA-A Allele Summary [a]				Allele Count by Population Group [b]								3.0.0 CIWD Category by Population Group [c]								
Allele	Genomic Typing	AlleleID	G group	AFA	API	EURO	MENA	HIS	NAM	UNK	Total	AFA	API	EURO	MENA	HIS	NAM	UNK	Total	Highest Frequency
A*23:18	23:18	HLA03170	23:01:01G	0	0	21	0	6	0	2	29			WD		WD			WD	WD
A*23:01:02	23:01:02	HLA03788		0	0	62	1	0	0	1	64			WD					WD	WD
A*23:01:03	23:01:03	HLA05426		5	0	0	0	0	0	0	5	WD							WD	WD
A*23:01:04	23:01:04	HLA05553		3	0	0	0	0	0	2	5								WD	WD
A*23:01:06	23:01:06	HLA06762		0	0	6	0	0	0	2	8			WD					WD	WD
A*23:01:09	23:01:09	HLA07594		0	0	0	0	3	0	0	3									
A*23:01:10	23:01:10	HLA08271		0	0	9	0	0	0	2	11			WD					WD	WD
A*23:01:11	23:01:11	HLA08628		0	0	0	0	0	0	1	1									
A*23:01:12	23:01:12	HLA10382		0	0	1	0	0	0	0	1									
A*23:01:15	23:01:15	HLA12551		0	0	1	0	0	0	0	1									
A*23:01:16	23:01:16	HLA12635		0	1	0	0	0	0	0	1									
A*23:01:18	23:01:18	HLA12948		1	0	0	1	0	0	0	2									
A*23:02	23:02	HLA01038		19	0	0	0	0	0	1	20	WD							WD	WD
**A*23:03 total**	**23:03 total**			**13**	**0**	**9**	**2**	**0**	**0**	**1**	**25**	**WD**		**WD**					**WD**	**WD**
A*23:03	23:03			2	0	0	0	0	0	1	3									
A*23:03:01	23:03:01	HLA01039		4	0	1	0	0	0	0	5								WD	WD
A*23:03:02	23:03:02	HLA03177		7	0	8	2	0	0	0	17	WD		WD					WD	WD
A*23:04	23:04	HLA01108		8	0	3	0	2	0	2	15	WD							WD	WD
A*23:05	23:05	HLA01111		26	2	18	0	104	24	28	202	WD		WD		C	C	I	I	C
A*23:06	23:06	HLA01210		23	0	8	1	3	0	13	48	WD		WD				WD	WD	WD
A*23:08N	23:08N	HLA01492		0	0	1	0	0	0	0	1									
A*23:11N	23:11N	HLA01834		17	0	1	0	0	0	2	20	WD							WD	WD
A*23:12	23:12	HLA01847		1	0	5	1	0	0	2	9			WD					WD	WD
A*23:13	23:13	HLA02328		3	0	80	3	2	0	1	89			WD					WD	WD
**A*23:14 total**	**23:14 total**			**0**	**4**	**4**	**0**	**0**	**0**	**0**	**8**								**WD**	**WD**
A*23:14:02	23:14:02	HLA08277		0	4	4	0	0	0	0	8								WD	WD
A*23:15	23:15	HLA02892		76	0	5	0	2	2	14	99	C		WD				I	WD	C
A*23:19N	23:19N	HLA03183		56	0	1	0	1	0	13	71	C						WD	WD	C
A*23:21	23:21	HLA03691		0	0	0	0	1	0	0	1									
A*23:23	23:23	HLA04485		0	0	7	0	0	0	0	7			WD					WD	WD

Supplemental Table 8: HLA-A Allele Summary [a]				Allele Count by Population Group [b]								3.0.0 CIWD Category by Population Group [c]								
Allele	Genomic Typing	AlleleID	G group	AFA	API	EURO	MENA	HIS	NAM	UNK	Total	AFA	API	EURO	MENA	HIS	NAM	UNK	Total	Highest Frequency
A*23:24	23:24	HLA04487		0	0	2	0	0	0	0	2									
A*23:26	23:26	HLA05236		0	1	0	0	0	0	0	1									
A*23:27	23:27	HLA05433		0	0	1	0	0	0	0	1									
A*23:28	23:28	HLA05435		0	0	4	0	0	0	0	4									
A*23:29	23:29	HLA05449		0	0	0	0	0	0	1	1									
A*23:30	23:30	HLA05524		0	0	2	8	0	0	1	11				WD				WD	WD
A*23:31	23:31	HLA05555		1	0	0	0	0	0	1	2									
A*23:34	23:34	HLA06086		3	0	0	0	0	0	1	4									
A*23:35	23:35	HLA06093		0	0	1	0	0	0	0	1									
A*23:36	23:36	HLA06094		0	0	2	0	0	0	0	2									
**A*23:37 total**	**23:37 total**			**1**	**0**	**0**	**0**	**0**	**0**	**0**	**1**									
A*23:37:02	23:37:02	HLA08272		1	0	0	0	0	0	0	1									
A*23:39	23:39	HLA06547		0	0	1	13	0	0	0	14				WD				WD	WD
A*23:42	23:42	HLA06785		0	0	1	0	0	0	0	1									
A*23:43	23:43	HLA06798		0	0	1	0	0	0	0	1									
A*23:47	23:47	HLA07175		0	0	19	0	0	0	0	19			WD					WD	WD
A*23:48	23:48	HLA07398		0	0	14	0	0	0	0	14			WD					WD	WD
A*23:49	23:49	HLA07403		0	0	2	0	0	0	0	2									
A*23:52	23:52	HLA07986		1	0	0	0	0	0	1	2									
A*23:53	23:53	HLA07987		1	0	5	0	0	0	0	6			WD					WD	WD
A*23:54	23:54	HLA08274		0	0	1	0	0	0	0	1									
A*23:56	23:56	HLA08953		4	0	1	0	0	0	0	5								WD	WD
A*23:57	23:57	HLA09119		0	0	1	0	0	0	0	1									
A*23:60	23:60	HLA09794		0	0	1	1	0	0	0	2									
A*23:63	23:63	HLA11398		0	0	0	1	0	0	0	1									
A*23:64	23:64	HLA11639		0	0	0	0	0	0	1	1									
A*23:67	23:67	HLA12246		0	0	2	0	0	0	0	2									
A*23:68	23:68	HLA12550		0	0	1	0	0	0	0	1									
A*23:72	23:72	HLA14826		0	0	1	0	0	0	0	1									
A*23:73	23:73	HLA14912		0	0	1	0	0	0	0	1									

Supplemental Table 8: HLA-A Allele Summary [a]				Allele Count by Population Group [b]								3.0.0 CIWD Category by Population Group [c]								
Allele	Genomic Typing	AlleleID	G group	AFA	API	EURO	MENA	HIS	NAM	UNK	Total	AFA	API	EURO	MENA	HIS	NAM	UNK	Total	Highest Frequency
A*23:76	23:76	HLA15820		0	0	1	0	0	0	0	1									
A*23:CODE	23:CODE			3912	244	23700	498	2704	259	4818	36135	NA	NA	NA	NA	NA	NA	NA	NA	NA
**A*24:02 total**	**24:02 total**			**9376**	**186507**	**998959**	**48550**	**75846**	**6894**	**114400**	**1440532**	**C**	**C**	**C**	**C**	**C**	**C**	**C**	**C**	**C**
A*24:02	24:02			9	131	5944	41	208	20	388	6741	WD	C	C	C	C	C	C	C	C
A*24:02P	24:02P			2	11	2218	2	12	1	15	2261		WD	C		I		I	C	C
**A*24:02:01G total**	**24:02:01G total**			**9339**	**185630**	**989688**	**48222**	**75450**	**6830**	**113577**	**1428736**	**C**	**C**	**C**	**C**	**C**	**C**	**C**	**C**	**C**
A*24:02:01G	24:02:01G		24:02:01G	7612	170511	948376	47205	56970	5174	105052	1340900	C	C	C	C	C	C	C	C	C
A*24:02:01	24:02:01		24:02:01G	367	3554	10821	253	4278	396	2075	21744	C	C	C	C	C	C	C	C	C
A*24:02:01:01	24:02:01:01	HLA00050	24:02:01G	1207	10712	28196	643	13270	1178	6090	61296	C	C	C	C	C	C	C	C	C
**A*24:02L total**	**24:02L total**			**0**	**138**	**92**	**26**	**23**	**4**	**27**	**310**		**C**	**WD**	**WD**	**I**		**I**	**I**	**C**
A*24:02L	24:02L		24:02:01G	0	0	2	0	0	0	3	5								WD	WD
A*24:02:01L	24:02:01L		24:02:01G	0	2	0	0	0	0	0	2									
A*24:02:01:02L	24:02:01:02L	HLA00051	24:02:01G	0	136	90	26	23	4	24	303		C	WD	WD	I		I	I	C
A*24:02:01:04	24:02:01:04	HLA13780	24:02:01G	27	398	935	82	188	27	128	1785	WD	C	I	C	C	C	I	C	C
A*24:02:01:05	24:02:01:05	HLA13786	24:02:01G	14	52	1074	10	120	4	99	1373	WD	I	I	WD	C		I	I	C
A*24:02:01:06	24:02:01:06	HLA14800	24:02:01G	11	3	8	1	479	39	39	580	WD		WD		C	C	I	I	C
A*24:02:01:07	24:02:01:07	HLA14802	24:02:01G	71	0	0	0	0	0	18	89	C						I	WD	C
A*24:02:01:08	24:02:01:08	HLA14803	24:02:01G	0	1	79	1	1	1	6	89			WD				WD	WD	WD
A*24:02:01:09	24:02:01:09	HLA16411	24:02:01G	0	0	0	0	0	0	2	2									
A*24:02:01:10	24:02:01:10	HLA16439	24:02:01G	0	0	1	0	0	0	0	1									
A*24:02:01:12	24:02:01:12	HLA16837	24:02:01G	0	0	33	0	30	2	4	69			WD		I			WD	I
A*24:02:01:13	24:02:01:13	HLA16862	24:02:01G	0	0	1	0	0	0	0	1									
A*24:02:03Q	24:02:03Q	HLA01616	24:02:01G	0	0	1	0	0	0	0	1									
A*24:02:10	24:02:10	HLA02353	24:02:01G	0	2	0	0	2	0	0	4									
A*24:02:13	24:02:13	HLA02706	24:02:01G	1	115	22	1	17	0	8	164		I	WD		I		WD	I	I
A*24:02:31	24:02:31	HLA04683	24:02:01G	0	11	0	0	0	0	1	12		WD						WD	WD
A*24:02:40	24:02:40	HLA05795	24:02:01G	23	78	0	0	1	0	13	115	WD	I					WD	WD	I
A*24:02:65	24:02:65	HLA09675	24:02:01G	0	0	2	0	0	0	0	2									
A*24:02:79	24:02:79	HLA10828	24:02:01G	0	0	1	0	0	0	0	1									
A*24:02:98	24:02:98	HLA15593	24:02:01G	2	1	0	0	65	4	4	76					I			WD	I

**Supplemental Table 8: HLA-A Allele Summary [a]**				**Allele Count by Population Group [b]**								**3.0.0 CIWD Category by Population Group [c]**								
**Allele**	**Genomic Typing**	**AlleleID**	**G group**	**AFA**	**API**	**EURO**	**MENA**	**HIS**	**NAM**	**UNK**	**Total**	**AFA**	**API**	**EURO**	**MENA**	**HIS**	**NAM**	**UNK**	**Total**	**Highest Frequency**
A*24:09N	24:09N	HLA00059	24:02:01G	0	0	32	0	0	0	3	35			WD					WD	WD
A*24:11N	24:11N	HLA00061	24:02:01G	4	38	14	0	2	1	4	63		I	WD					WD	I
A*24:79	24:79	HLA02984	24:02:01G	0	0	0	0	3	0	2	5								WD	WD
A*24:249	24:249	HLA10206	24:02:01G	0	1	0	0	0	0	0	1									
A*24:271	24:271	HLA10868	24:02:01G	0	3	0	0	0	0	1	4									
A*24:352	24:352	HLA15569	24:02:01G	0	0	0	0	1	0	0	1									
A*24:353	24:353	HLA15573	24:02:01G	0	12	0	0	0	0	1	13		WD						WD	WD
A*24:02:02	24:02:02	HLA00052		4	2	470	0	18	1	56	551			I		I		I	I	I
A*24:02:04	24:02:04	HLA01648		18	24	259	158	151	40	321	971	WD	I	I	C	C	C	C	I	C
A*24:02:05	24:02:05	HLA01828		2	671	90	84	6	1	24	878		C	WD	C	WD		I	I	C
A*24:02:06	24:02:06	HLA01997		0	0	1	0	0	0	0	1									
A*24:02:07	24:02:07	HLA02204		0	0	9	0	0	0	0	9			WD					WD	WD
A*24:02:12	24:02:12	HLA02652		0	0	2	0	0	0	0	2									
A*24:02:14	24:02:14	HLA02869		0	1	98	0	0	0	0	99			WD					WD	WD
A*24:02:15	24:02:15	HLA03196		0	25	1	2	0	0	0	28		I						WD	I
A*24:02:18	24:02:18	HLA03740		0	2	8	0	0	0	0	10			WD					WD	WD
A*24:02:19	24:02:19	HLA03741		3	1	99	0	1	0	6	110			WD				WD	WD	WD
A*24:02:22	24:02:22	HLA04068		0	0	4	14	0	0	2	20				WD				WD	WD
A*24:02:23	24:02:23	HLA04153		0	0	10	3	0	0	2	15			WD					WD	WD
A*24:02:24	24:02:24	HLA04154		0	0	13	0	0	0	1	14			WD					WD	WD
A*24:02:25	24:02:25	HLA04155		0	0	16	0	0	0	0	16			WD					WD	WD
A*24:02:26	24:02:26	HLA04156		0	0	13	0	0	0	0	13			WD					WD	WD
A*24:02:27	24:02:27	HLA04429		0	0	7	0	0	0	9	16			WD				WD	WD	WD
A*24:02:28	24:02:28	HLA04483		0	0	1	0	0	0	0	1									
A*24:02:29	24:02:29	HLA04488		0	0	18	2	0	0	1	21			WD					WD	WD
**A*24:02:34G total**	**24:02:34G total**			**0**	**0**	**1**	**1**	**0**	**0**	**0**	**2**									
A*24:02:34G	24:02:34G		24:02:34G	0	0	1	1	0	0	0	2									
A*24:02:35	24:02:35	HLA05353		1	28	1	17	0	0	2	49		I		WD				WD	I
A*24:02:36	24:02:36	HLA05364		0	0	1	0	0	0	0	1									
A*24:02:37	24:02:37	HLA05461		0	0	1	0	0	0	0	1									

Supplemental Table 8: HLA-A Allele Summary [a]				Allele Count by Population Group [b]								3.0.0 CIWD Category by Population Group [c]								
Allele	Genomic Typing	AlleleID	G group	AFA	API	EURO	MENA	HIS	NAM	UNK	Total	AFA	API	EURO	MENA	HIS	NAM	UNK	Total	Highest Frequency
A*24:02:39	24:02:39	HLA05743		0	1	0	0	0	0	0	1									
A*24:02:47	24:02:47	HLA06544		0	3	0	0	0	0	0	3									
A*24:02:48	24:02:48	HLA06750		0	0	1	1	0	0	0	2									
A*24:02:49	24:02:49	HLA06810		0	3	0	0	0	0	0	3									
A*24:02:53	24:02:53	HLA07424		0	0	9	0	0	0	0	9			WD					WD	WD
A*24:02:55	24:02:55	HLA07563		0	1	0	0	0	0	0	1									
A*24:02:59	24:02:59	HLA08273		0	0	0	0	2	0	1	3									
A*24:02:61	24:02:61	HLA08300		0	1	0	0	0	0	0	1									
A*24:02:62	24:02:62	HLA08633		0	5	12	0	0	0	0	17		WD	WD					WD	WD
A*24:02:63	24:02:63	HLA08634		0	1	0	0	0	0	0	1									
A*24:02:66	24:02:66	HLA09795		0	0	1	0	0	0	0	1									
A*24:02:68	24:02:68	HLA09798		0	2	0	0	0	0	0	2									
A*24:02:71	24:02:71	HLA09905		0	0	1	0	0	1	0	2									
A*24:02:72	24:02:72	HLA10383		0	0	2	3	0	0	1	6								WD	WD
A*24:02:73	24:02:73	HLA10384		2	0	0	0	0	0	0	2									
A*24:02:76	24:02:76	HLA10815		0	0	2	0	0	0	0	2									
A*24:02:85	24:02:85	HLA11213		0	1	0	0	0	0	0	1									
A*24:02:88	24:02:88	HLA11637		0	0	3	0	0	0	1	4									
A*24:02:89	24:02:89	HLA12166		0	0	1	0	0	0	0	1									
A*24:02:90	24:02:90	HLA12409		0	1	0	0	0	0	0	1									
A*24:02:92	24:02:92	HLA12954		0	0	0	0	0	1	0	1									
**A*24:03 total**	**24:03 total**			**230**	**5098**	**21749**	**3493**	**2278**	**229**	**4830**	**37907**	**C**	**C**	**C**	**C**	**C**	**C**	**C**	**C**	**C**
A*24:03	24:03			1	0	195	1	19	1	12	229			I		I		WD	I	I
A*24:03P	24:03P			0	1	70	0	0	0	0	71			WD					WD	WD
**A*24:03:01G total**	**24:03:01G total**			**223**	**5096**	**21471**	**3492**	**2146**	**225**	**4791**	**37444**	**C**	**C**	**C**	**C**	**C**	**C**	**C**	**C**	**C**
A*24:03:01G	24:03:01G		24:03:01G	174	4323	20264	3367	1635	158	4433	34354	C	C	C	C	C	C	C	C	C
A*24:03:01	24:03:01		24:03:01G	27	278	717	86	306	31	164	1609	WD	C	I	C	C	C	C	I	C
A*24:03:01:01	24:03:01:01	HLA00053	24:03:01G	19	97	478	39	133	16	88	870	WD	I	I	WD	C	C	I	I	C
A*24:03:01:02	24:03:01:02	HLA14804	24:03:01G	2	2	5	0	67	15	13	104			WD		I	C	WD	WD	C
A*24:33	24:33	HLA01491	24:03:01G	1	396	7	0	5	5	93	507		C	WD		WD	WD	I	I	C

Supplemental Table 8: HLA-A Allele Summary [a]				Allele Count by Population Group [b]								3.0.0 CIWD Category by Population Group [c]								
**Allele**	**Genomic Typing**	**AlleleID**	**G group**	**AFA**	**API**	**EURO**	**MENA**	**HIS**	**NAM**	**UNK**	**Total**	**AFA**	**API**	**EURO**	**MENA**	**HIS**	**NAM**	**UNK**	**Total**	**Highest Frequency**
A*24:03:02	24:03:02	HLA01040		6	1	13	0	113	3	27	163	WD		WD		C		I	I	C
A*24:04	24:04	HLA00054		0	26	5	1	0	0	1	33		I	WD					WD	I
**A*24:05 total**	**24:05 total**			**10**	**39**	**107**	**4**	**257**	**36**	**77**	**530**	**WD**	**I**	**WD**		**C**	**C**	**I**	**I**	**C**
A*24:05	24:05			5	11	24	1	69	12	13	135	WD	WD	WD		I	C	WD	WD	C
A*24:05P	24:05P			0	0	1	0	0	0	0	1									
**A*24:05:01G total**	**24:05:01G total**			**5**	**28**	**82**	**3**	**188**	**24**	**64**	**394**	**WD**	**I**	**WD**		**C**	**C**	**I**	**I**	**C**
A*24:05:01G	24:05:01G		24:05:01G	5	28	79	3	176	23	62	376	WD	I	WD		C	C	I	I	C
A*24:05:01	24:05:01	HLA00055	24:05:01G	0	0	3	0	12	1	2	18					I			WD	I
A*24:06	24:06	HLA00056		0	9	9	1	0	0	1	20		WD	WD					WD	WD
**A*24:07 total**	**24:07 total**			**184**	**23415**	**1641**	**324**	**227**	**37**	**3376**	**29204**	**C**	**C**	**C**	**C**	**C**	**C**	**C**	**C**	**C**
A*24:07	24:07			76	5970	541	71	80	24	1202	7964	C	C	I	C	C	C	C	C	C
A*24:07:01	24:07:01	HLA00057		108	17439	1100	253	147	13	2174	21234	C	C	I	C	C	C	C	C	C
A*24:07:02	24:07:02	HLA12110		0	6	0	0	0	0	0	6		WD						WD	WD
A*24:08	24:08	HLA00058		0	89	2	0	0	0	11	102		I					WD	WD	I
**A*24:10 total**	**24:10 total**			**4**	**1735**	**88**	**10**	**3**	**1**	**235**	**2076**		**C**	**WD**	**WD**			**C**	**C**	**C**
A*24:10	24:10			1	282	10	1	0	1	46	341		C	WD				I	I	C
A*24:10:01	24:10:01	HLA00060		3	1452	78	9	3	0	189	1734		C	WD	WD			C	C	C
A*24:10:02	24:10:02	HLA08627		0	1	0	0	0	0	0	1									
**A*24:13 total**	**24:13 total**			**0**	**48**	**18**	**2**	**26**	**0**	**6**	**100**		**I**	**WD**		**I**		**WD**	**WD**	**I**
A*24:13	24:13			0	0	5	1	2	0	1	9			WD					WD	WD
A*24:13:01	24:13:01	HLA00063		0	41	4	0	1	0	2	48		I						WD	I
A*24:13:02	24:13:02	HLA03101		0	7	9	1	23	0	3	43		WD	WD		I			WD	I
**A*24:14 total**	**24:14 total**			**11**	**5**	**35**	**1**	**281**	**9**	**48**	**390**	**WD**	**WD**	**WD**		**C**	**C**	**I**	**I**	**C**
**A*24:14:01G total**	**24:14:01G total**			**11**	**5**	**35**	**1**	**281**	**9**	**48**	**390**	**WD**	**WD**	**WD**		**C**	**C**	**I**	**I**	**C**
A*24:14	24:14			11	3	31	1	219	6	41	312	WD		WD		C	WD	I	I	C
A*24:14:01G	24:14:01G		24:14:01G	0	2	4	0	26	0	5	37					I		WD	WD	I
A*24:14:01	24:14:01		24:14:01G	0	0	0	0	34	2	1	37					I			WD	I
A*24:14:01:02	24:14:01:02	HLA14845	24:14:01G	0	0	0	0	2	1	1	4									
A*24:15	24:15	HLA00065		0	0	0	0	7	1	1	9					WD			WD	WD
A*24:17	24:17	HLA00067		8	4923	98	35	4	20	100	5188	WD	C	WD	WD		C	I	C	C

Supplemental Table 8: HLA-A Allele Summary [a]				Allele Count by Population Group [b]								3.0.0 CIWD Category by Population Group [c]								
Allele	Genomic Typing	AlleleID	G group	AFA	API	EURO	MENA	HIS	NAM	UNK	Total	AFA	API	EURO	MENA	HIS	NAM	UNK	Total	Highest Frequency
A*24:18	24:18	HLA00068		0	0	36	0	1	0	0	37			WD					WD	WD
**A*24:20 total**	**24:20 total**			**0**	**572**	**22**	**14**	**4**	**0**	**138**	**750**		**C**	**WD**	**WD**			**C**	**I**	**C**
**A*24:20:01G total**	**24:20:01G total**			**0**	**572**	**22**	**14**	**4**	**0**	**138**	**750**		**C**	**WD**	**WD**			**C**	**I**	**C**
A*24:20	24:20			0	567	20	14	4	0	138	743		C	WD	WD			C	I	C
A*24:20:01G	24:20:01G		24:20:01G	0	5	2	0	0	0	0	7		WD						WD	WD
**A*24:21 total**	**24:21 total**			**0**	**5**	**35**	**0**	**0**	**0**	**7**	**47**		**WD**	**WD**				**WD**	**WD**	**WD**
A*24:21	24:21			0	1	4	0	0	0	0	5								WD	WD
A*24:21:01	24:21:01	HLA00969		0	2	30	0	0	0	7	39			WD				WD	WD	WD
A*24:21:03	24:21:03	HLA13728		0	2	1	0	0	0	0	3									
A*24:22	24:22	HLA00970		5	1	16	1	250	12	32	317	WD		WD		C	C	I	I	C
A*24:23	24:23	HLA01041		81	5	108	2	41	88	111	436	C	WD	WD		I	C	I	I	C
A*24:24	24:24	HLA01042		6	0	0	0	0	0	1	7	WD							WD	WD
A*24:25	24:25	HLA01189		14	10	71	2	1460	75	189	1821	WD	WD	WD		C	C	C	C	C
**A*24:26 total**	**24:26 total**			**6**	**0**	**101**	**39**	**26**	**0**	**18**	**190**	**WD**		**WD**	**WD**	**I**		**I**	**I**	**I**
**A*24:26:01G total**	**24:26:01G total**			**6**	**0**	**101**	**39**	**26**	**0**	**18**	**190**	**WD**		**WD**	**WD**	**I**		**I**	**I**	**I**
A*24:26:01G	24:26:01G		24:26:01G	1	0	30	12	5	0	5	53			WD	WD	WD		WD	WD	WD
A*24:26	24:26	HLA01171	24:26:01G	4	0	71	26	19	0	13	133			WD	WD	I		WD	WD	I
A*24:314	24:314	HLA13634	24:26:01G	1	0	0	1	2	0	0	4									
A*24:28	24:28	HLA01268		1	1	52	0	15	0	7	76			WD		I		WD	WD	I
A*24:29	24:29	HLA01316		0	0	193	6	1	1	9	210			I	WD			WD	I	I
A*24:30	24:30	HLA01317		0	40	64	4	4	0	2	114		I	WD					WD	I
A*24:31	24:31	HLA01330		1	3	431	0	2	0	8	445			I				WD	I	I
A*24:32	24:32	HLA01465		0	4	113	0	3	0	24	144			WD				I	WD	I
A*24:35	24:35	HLA01566		0	0	20	0	1	0	3	24			WD					WD	WD
A*24:36N	24:36N	HLA01583		0	0	18	0	0	0	0	18			WD					WD	WD
A*24:37	24:37	HLA01649		0	32	2	2	0	0	5	41		I					WD	WD	I
A*24:38	24:38	HLA01674		0	1	2	0	27	2	7	39					I		WD	WD	I
A*24:39	24:39	HLA01773		0	0	12	0	0	0	0	12			WD					WD	WD
A*24:41	24:41	HLA01823		0	3	0	0	0	0	0	3									
A*24:42	24:42	HLA01872		0	0	8	0	0	0	0	8			WD					WD	WD

Supplemental Table 8: HLA-A Allele Summary [a]				Allele Count by Population Group [b]								3.0.0 CIWD Category by Population Group [c]								
Allele	Genomic Typing	AlleleID	G group	AFA	API	EURO	MENA	HIS	NAM	UNK	Total	AFA	API	EURO	MENA	HIS	NAM	UNK	Total	Highest Frequency
A*24:43	24:43	HLA01957		0	3	145	0	2	0	5	155			I				WD	WD	I
A*24:46	24:46	HLA01988		0	1	3	0	1	0	2	7								WD	WD
A*24:47	24:47	HLA02037		0	0	16	0	0	0	0	16			WD					WD	WD
A*24:48N	24:48N	HLA02049		0	0	3	0	0	0	1	4									
A*24:49	24:49	HLA02053		0	2	0	0	0	0	0	2									
A*24:50	24:50	HLA02107		0	0	3	0	2	0	0	5								WD	WD
A*24:52	24:52	HLA02181		0	0	10	20	0	0	6	36			WD	WD			WD	WD	WD
A*24:53	24:53	HLA02198		1	3	53	0	224	21	37	339			WD		C	C	I	I	C
A*24:56	24:56	HLA02366		0	0	321	6	1	0	6	334			I	WD			WD	I	I
A*24:57	24:57	HLA02365		0	1	25	0	0	0	30	56			WD				I	WD	I
A*24:58	24:58	HLA02401		0	0	233	0	1	0	11	245			I				WD	I	I
A*24:59	24:59	HLA02417		0	1	1	0	0	0	1	3									
A*24:60N	24:60N	HLA02443		0	0	0	1	0	0	0	1									
A*24:64	24:64	HLA02574		1	16	1	0	0	0	0	18		I						WD	I
A*24:68	24:68	HLA02725		0	10	0	0	0	0	0	10		WD						WD	WD
A*24:69	24:69	HLA02774		0	0	1	0	0	0	0	1									
A*24:70	24:70	HLA02777		0	0	2	0	0	0	0	2									
A*24:71	24:71	HLA02788		0	0	8	2	0	0	0	10			WD					WD	WD
A*24:72	24:72	HLA02860		0	2	168	0	1	0	3	174			I					I	I
A*24:73	24:73	HLA02861		0	0	1	0	0	0	0	1									
**A*24:74 total**	**24:74 total**			**0**	**1**	**2**	**0**	**0**	**0**	**0**	**3**									
A*24:74	24:74			0	0	1	0	0	0	0	1									
A*24:74:01	24:74:01	HLA02862		0	1	0	0	0	0	0	1									
A*24:74:02	24:74:02	HLA09485		0	0	1	0	0	0	0	1									
A*24:77	24:77	HLA02958		0	1	1	0	0	0	0	2									
A*24:80	24:80	HLA03000		0	0	1	0	0	0	0	1									
A*24:81	24:81	HLA03031		0	0	83	0	3	1	17	104			WD				I	WD	I
A*24:82	24:82	HLA03032		0	2	0	0	0	0	0	2									
A*24:84N	24:84N	HLA03154		0	0	8	0	0	0	1	9			WD					WD	WD
A*24:85	24:85	HLA03201		0	1	0	0	0	0	0	1									

Supplemental Table 8: HLA-A Allele Summary [a]				Allele Count by Population Group [b]								3.0.0 CIWD Category by Population Group [c]								
Allele	Genomic Typing	AlleleID	G group	AFA	API	EURO	MENA	HIS	NAM	UNK	Total	AFA	API	EURO	MENA	HIS	NAM	UNK	Total	Highest Frequency
A*24:86N	24:86N	HLA03251		0	0	1	0	0	0	0	1									
A*24:87	24:87	HLA03331		0	1	6	2	1	0	6	16			WD				WD	WD	WD
**A*24:90N total**	**24:90N total**			**2**	**1**	**12**	**0**	**1**	**0**	**3**	**19**			**WD**					**WD**	**WD**
A*24:90N	24:90N			2	0	10	0	1	0	3	16			WD					WD	WD
A*24:90:01N	24:90:01N	HLA03352		0	0	2	0	0	0	0	2									
A*24:90:02N	24:90:02N	HLA13951		0	1	0	0	0	0	0	1									
A*24:91	24:91	HLA03395		0	1	0	0	0	0	0	1									
A*24:95	24:95	HLA03535		3	1	20	7	77	1	44	153			WD	WD	C		I	WD	C
A*24:97	24:97	HLA03569		0	19	0	0	0	0	0	19		I						WD	I
A*24:99	24:99	HLA03618		0	0	4	0	0	0	0	4									
A*24:100	24:100	HLA04894		0	0	1	0	0	0	0	1									
A*24:102	24:102	HLA04896		0	0	0	2	0	0	0	2									
A*24:103	24:103	HLA04897		0	0	9	0	0	0	0	9			WD					WD	WD
A*24:104	24:104	HLA04898		0	0	8	0	0	0	0	8			WD					WD	WD
A*24:105	24:105	HLA04899		0	0	5	0	0	0	0	5			WD					WD	WD
A*24:106	24:106	HLA04905		0	0	74	0	0	0	3	77			WD					WD	WD
A*24:107	24:107	HLA04862		0	0	0	0	2	2	1	5								WD	WD
A*24:108	24:108	HLA04863		0	0	4	2	0	0	1	7								WD	WD
A*24:110	24:110	HLA04908		0	0	17	0	0	0	0	17			WD					WD	WD
A*24:111	24:111	HLA04909		0	0	1	0	0	0	0	1									
A*24:113	24:113	HLA04926		0	0	1	0	0	0	0	1									
A*24:115	24:115	HLA04942		0	0	1	0	0	0	0	1									
A*24:116	24:116	HLA04950		0	0	1	0	1	0	0	2									
A*24:117	24:117	HLA04954		0	0	2	0	0	0	0	2									
A*24:118	24:118	HLA04955		0	0	10	0	0	0	0	10			WD					WD	WD
A*24:120	24:120	HLA04957		0	3	2	0	0	0	0	5								WD	WD
A*24:121	24:121	HLA04958		0	3	16	0	1	0	2	22			WD					WD	WD
A*24:122	24:122	HLA04988		0	0	10	0	0	0	0	10			WD					WD	WD
A*24:123	24:123	HLA05006		0	0	1	6	0	0	1	8				WD				WD	WD
A*24:124	24:124	HLA05007		0	0	0	1	0	0	0	1									

Supplemental Table 8: HLA-A Allele Summary [a]				Allele Count by Population Group [b]								3.0.0 CIWD Category by Population Group [c]								
Allele	Genomic Typing	AlleleID	G group	AFA	API	EURO	MENA	HIS	NAM	UNK	Total	AFA	API	EURO	MENA	HIS	NAM	UNK	Total	Highest Frequency
A*24:126	24:126	HLA05019		0	0	0	0	1	0	1	2									
A*24:128	24:128	HLA04870		0	3	0	0	0	0	0	3									
A*24:130	24:130	HLA04876		0	5	0	0	0	0	0	5		WD						WD	WD
**A*24:135 total**	**24:135 total**			**0**	**1**	**1**	**0**	**1**	**0**	**1**	**4**									
A*24:135:01	24:135:01	HLA05107		0	0	1	0	1	0	0	2									
A*24:135:02	24:135:02	HLA08585		0	1	0	0	0	0	1	2									
A*24:136	24:136	HLA05084		0	0	3	0	1	0	0	4									
A*24:138	24:138	HLA05086		0	0	0	0	4	0	1	5								WD	WD
A*24:140	24:140	HLA05110		0	8	0	0	0	0	0	8		WD						WD	WD
**A*24:142 total**	**24:142 total**			**0**	**1**	**0**	**0**	**0**	**0**	**0**	**1**									
A*24:142	24:142			0	1	0	0	0	0	0	1									
A*24:143	24:143	HLA05240		0	1	0	0	0	0	0	1									
A*24:145	24:145	HLA05550		0	0	6	0	0	0	14	20			WD				I	WD	I
A*24:146	24:146	HLA05673		0	0	0	10	0	0	0	10				WD				WD	WD
A*24:148	24:148	HLA05679		0	0	2	0	0	0	0	2									
A*24:151	24:151	HLA05797		0	1	0	0	0	0	0	1									
A*24:156	24:156	HLA06004		0	2	0	0	0	0	1	3									
A*24:157	24:157	HLA06014		0	0	2	0	0	0	2	4									
A*24:159	24:159	HLA06064		0	0	0	0	7	1	1	9					WD			WD	WD
A*24:164	24:164	HLA06119		0	0	7	0	0	0	0	7			WD					WD	WD
A*24:165	24:165	HLA06196		0	5	0	0	0	0	1	6		WD						WD	WD
A*24:166	24:166	HLA06197		0	2	0	0	0	0	0	2									
A*24:167	24:167	HLA06202		0	0	4	0	0	0	0	4									
A*24:168	24:168	HLA06203		0	0	7	0	0	0	1	8			WD					WD	WD
A*24:169	24:169	HLA06207		0	3	5	0	0	0	2	10			WD					WD	WD
**A*24:172 total**	**24:172 total**			**0**	**0**	**1**	**0**	**0**	**0**	**1**	**2**									
A*24:172:01	24:172:01	HLA06333		0	0	1	0	0	0	1	2									
A*24:173	24:173	HLA06545		0	0	2	0	0	0	7	9							WD	WD	WD
A*24:175	24:175	HLA06556		0	0	13	0	6	0	18	37			WD		WD		I	WD	I
A*24:176	24:176	HLA06557		0	0	10	0	0	0	0	10			WD					WD	WD

Supplemental Table 8: HLA-A Allele Summary [a]				Allele Count by Population Group [b]								3.0.0 CIWD Category by Population Group [c]								
**Allele**	**Genomic Typing**	**AlleleID**	**G group**	**AFA**	**API**	**EURO**	**MENA**	**HIS**	**NAM**	**UNK**	**Total**	**AFA**	**API**	**EURO**	**MENA**	**HIS**	**NAM**	**UNK**	**Total**	**Highest Frequency**
A*24:177	24:177	HLA06568		0	0	1	0	0	0	0	1									
A*24:179	24:179	HLA06751		0	0	0	0	1	0	0	1									
A*24:180	24:180	HLA06752		0	0	0	0	7	1	0	8					WD			WD	WD
A*24:184	24:184	HLA07176		0	0	4	0	0	0	0	4									
A*24:187	24:187	HLA07399		0	0	0	0	0	0	1	1									
A*24:189	24:189	HLA07417		0	1	0	0	0	0	0	1									
A*24:192	24:192	HLA07588		1	0	0	0	4	0	2	7								WD	WD
A*24:193	24:193	HLA07589		0	0	8	0	0	0	0	8			WD					WD	WD
A*24:194	24:194	HLA07590		0	4	0	0	0	0	1	5								WD	WD
A*24:195	24:195	HLA07591		0	0	1	0	0	0	0	1									
A*24:196	24:196	HLA07592		0	16	0	0	0	0	0	16		I						WD	I
A*24:197	24:197	HLA07593		0	0	1	0	0	0	0	1									
A*24:198	24:198	HLA07750		0	4	0	0	0	0	0	4									
A*24:201	24:201	HLA07985		0	1	0	0	0	0	0	1									
A*24:202	24:202	HLA08024		0	0	2	0	0	0	0	2									
A*24:203	24:203	HLA08025		0	0	10	0	0	0	0	10			WD					WD	WD
A*24:204	24:204	HLA08115		0	0	2	0	0	0	0	2									
A*24:205	24:205	HLA08270		0	0	2	0	3	0	1	6								WD	WD
A*24:206	24:206	HLA08275		0	2	5	0	1	0	1	9			WD					WD	WD
**A*24:207 total**	**24:207 total**			**0**	**4**	**0**	**1**	**2**	**0**	**1**	**8**								**WD**	**WD**
A*24:207	24:207			0	4	0	1	2	0	1	8								WD	WD
A*24:209	24:209	HLA08279		0	1	0	0	0	0	0	1									
A*24:213	24:213	HLA08560		0	0	1	0	0	0	0	1									
A*24:214	24:214	HLA08484		0	0	0	0	0	0	1	1									
A*24:215	24:215	HLA08602		0	0	2	0	0	0	0	2									
A*24:216	24:216	HLA08625		0	1	0	0	0	0	0	1									
A*24:218	24:218	HLA08630		0	0	0	0	0	0	1	1									
A*24:219	24:219	HLA08631		0	0	1	0	0	0	0	1									
A*24:220	24:220	HLA08632		0	0	0	1	0	0	4	5								WD	WD
**A*24:226 total**	**24:226 total**			**0**	**0**	**5**	**0**	**0**	**0**	**0**	**5**			**WD**					**WD**	**WD**

Supplemental Table 8: HLA-A Allele Summary [a]				Allele Count by Population Group [b]								3.0.0 CIWD Category by Population Group [c]								
Allele	Genomic Typing	AlleleID	G group	AFA	API	EURO	MENA	HIS	NAM	UNK	Total	AFA	API	EURO	MENA	HIS	NAM	UNK	Total	Highest Frequency
A*24:226	24:226			0	0	2	0	0	0	0	2									
A*24:226:01	24:226:01	HLA08950		0	0	3	0	0	0	0	3									
A*24:228	24:228	HLA08964		0	0	19	0	0	0	0	19			WD					WD	WD
A*24:229	24:229	HLA09078		0	0	4	0	0	0	0	4									
A*24:230	24:230	HLA09109		0	1	0	0	0	0	0	1									
A*24:234	24:234	HLA09495		0	2	4	0	0	0	0	6								WD	WD
A*24:236	24:236	HLA09532		0	0	2	0	1	0	0	3									
A*24:238	24:238	HLA09534		0	0	3	0	0	0	0	3									
A*24:243	24:243	HLA09791		0	0	4	0	0	0	0	4									
A*24:244	24:244	HLA09792		0	1	0	0	0	0	0	1									
A*24:246	24:246	HLA09797		0	1	0	0	0	0	0	1									
A*24:252N	24:252N	HLA10267		0	0	5	5	0	0	0	10			WD	WD				WD	WD
A*24:253	24:253	HLA10381		0	1	0	0	0	0	0	1									
A*24:254	24:254	HLA10444		0	4	0	0	0	0	0	4									
A*24:255	24:255	HLA10448		0	2	0	0	0	0	0	2									
A*24:258	24:258	HLA10673		0	0	1	0	0	0	0	1									
A*24:261	24:261	HLA10819		0	0	1	0	0	0	0	1									
A*24:262	24:262	HLA10827		0	0	0	1	0	0	0	1									
A*24:273	24:273	HLA10893		0	1	1	0	0	0	0	2									
A*24:275	24:275	HLA10975		0	0	0	0	0	0	1	1									
A*24:277	24:277	HLA10977		0	0	2	0	0	0	0	2									
A*24:279	24:279	HLA11212		0	1	0	0	0	0	0	1									
A*24:280	24:280	HLA11397		0	0	3	0	0	0	1	4									
A*24:284	24:284	HLA11795		0	1	0	0	0	0	1	2									
A*24:287	24:287	HLA12077		0	1	0	0	0	0	0	1									
A*24:291	24:291	HLA12459		0	1	0	0	0	0	0	1									
A*24:292	24:292	HLA12633		0	0	0	0	0	0	1	1									
A*24:293	24:293	HLA12634		0	0	0	0	1	0	0	1									
A*24:296	24:296	HLA12949		0	0	0	0	1	0	0	1									
A*24:298	24:298	HLA12951		0	0	0	0	0	0	1	1									

Supplemental Table 8: HLA-A Allele Summary [a]				Allele Count by Population Group [b]								3.0.0 CIWD Category by Population Group [c]								
Allele	Genomic Typing	AlleleID	G group	AFA	API	EURO	MENA	HIS	NAM	UNK	Total	AFA	API	EURO	MENA	HIS	NAM	UNK	Total	Highest Frequency
A*24:300	24:300	HLA12953		0	0	1	0	0	0	0	1									
A*24:301	24:301	HLA12955		0	1	0	0	0	0	0	1									
A*24:302	24:302	HLA12956		0	0	1	0	0	0	0	1									
A*24:307	24:307	HLA13444		0	0	0	0	2	1	0	3									
A*24:308	24:308	HLA13455		0	0	0	0	1	0	0	1									
A*24:309	24:309	HLA13504		0	0	1	0	0	0	0	1									
**A*24:310 total**	**24:310 total**			**0**	**0**	**0**	**0**	**2**	**0**	**0**	**2**									
A*24:310	24:310			0	0	0	0	1	0	0	1									
A*24:310:02	24:310:02	HLA13646		0	0	0	0	1	0	0	1									
A*24:311	24:311	HLA13645		0	1	0	0	0	0	0	1									
A*24:312N	24:312N	HLA13726		0	0	0	1	0	0	0	1									
A*24:318	24:318	HLA13844		0	1	1	0	0	0	0	2									
A*24:320	24:320	HLA13953		0	0	2	0	0	0	0	2									
A*24:321	24:321	HLA13784		0	0	0	0	0	0	1	1									
A*24:324	24:324	HLA14282		0	1	0	0	1	0	0	2									
A*24:330	24:330	HLA14502		0	0	1	0	0	0	0	1									
A*24:332	24:332	HLA14504		0	1	0	0	0	0	0	1									
A*24:339	24:339	HLA14914		0	2	0	0	0	0	0	2									
A*24:345	24:345	HLA15240		0	4	0	0	0	0	0	4									
**A*24:347 total**	**24:347 total**			**0**	**2**	**0**	**0**	**0**	**0**	**0**	**2**									
A*24:347	24:347			0	2	0	0	0	0	0	2									
A*24:356	24:356	HLA15818		0	1	0	1	0	0	0	2									
A*24:359N	24:359N	HLA15838		0	0	1	0	0	0	0	1									
A*24:360	24:360	HLA15957		0	1	1	0	0	0	0	2									
A*24:365	24:365	HLA15953		0	1	0	0	0	0	0	1									
A*24:366	24:366	HLA15954		0	0	0	0	0	0	2	2									
A*24:CODE	24:CODE			1102	7087	97434	2366	9314	810	14586	132699	NA	NA	NA	NA	NA	NA	NA	NA	NA
**A*25:01 total**	**25:01 total**			**1260**	**801**	**311916**	**1803**	**6838**	**515**	**18870**	**342003**	**C**	**C**	**C**	**C**	**C**	**C**	**C**	**C**	**C**
A*25:01	25:01			0	0	1429	0	4	0	50	1483			C				I	I	C
A*25:01P	25:01P			0	0	232	0	0	0	0	232			I					I	I

Supplemental Table 8: HLA-A Allele Summary [a]				Allele Count by Population Group [b]								3.0.0 CIWD Category by Population Group [c]								
Allele	Genomic Typing	AlleleID	G group	AFA	API	EURO	MENA	HIS	NAM	UNK	Total	AFA	API	EURO	MENA	HIS	NAM	UNK	Total	Highest Frequency
**A*25:01:01G total**	**25:01:01G total**			**1260**	**801**	**310224**	**1803**	**6834**	**515**	**18820**	**340257**	**C**	**C**	**C**	**C**	**C**	**C**	**C**	**C**	**C**
A*25:01:01G	25:01:01G		25:01:01G	1003	762	298361	1761	4807	375	17185	324254	C	C	C	C	C	C	C	C	C
A*25:01:01	25:01:01		25:01:01G	251	36	11161	39	1925	137	1556	15105	C	I	C	WD	C	C	C	C	C
A*25:01:01:01	25:01:01:01	HLA00071	25:01:01G	6	3	701	3	102	3	78	896	WD		I		C		I	I	C
A*25:07	25:07	HLA03523	25:01:01G	0	0	1	0	0	0	1	2									
A*25:01:02	25:01:02	HLA01759		0	0	2	0	0	0	0	2									
A*25:01:03	25:01:03	HLA05460		0	0	3	0	0	0	0	3									
A*25:01:04	25:01:04	HLA05706		0	0	1	0	0	0	0	1									
A*25:01:06	25:01:06	HLA07669		0	0	22	0	0	0	0	22			WD					WD	WD
A*25:01:07	25:01:07	HLA07609		0	0	2	0	0	0	0	2									
A*25:01:09	25:01:09	HLA13223		0	0	1	0	0	0	0	1									
A*25:02	25:02	HLA00072		2	0	126	0	0	1	13	142			I				WD	WD	I
A*25:03	25:03	HLA01043		0	0	116	1	0	0	2	119			WD					WD	WD
A*25:04	25:04	HLA01511		0	0	7	0	0	0	1	8			WD					WD	WD
A*25:05	25:05	HLA02607		0	0	3	0	0	0	1	4									
A*25:08	25:08	HLA03565		0	0	6	0	0	0	2	8			WD					WD	WD
A*25:09	25:09	HLA03919		0	0	3	0	0	0	0	3									
A*25:10	25:10	HLA04061		0	0	1	0	0	0	0	1									
A*25:11	25:11	HLA04645		0	0	8	1	0	0	2	11			WD					WD	WD
A*25:12N	25:12N	HLA04762		0	0	8	0	0	0	0	8			WD					WD	WD
A*25:13	25:13	HLA04793		0	0	4	0	1	0	0	5								WD	WD
A*25:14	25:14	HLA05915		0	0	9	0	1	0	2	12			WD					WD	WD
A*25:15	25:15	HLA05943		0	0	1	0	0	0	1	2									
A*25:16	25:16	HLA06190		0	0	10	0	0	0	0	10			WD					WD	WD
A*25:17	25:17	HLA08261		0	0	3	0	0	0	0	3									
A*25:22	25:22	HLA08615		0	0	1	0	0	0	0	1									
A*25:24	25:24	HLA10524		0	0	1	0	7	0	0	8					WD			WD	WD
**A*25:27 total**	**25:27 total**			**0**	**0**	**1**	**0**	**0**	**0**	**0**	**1**									
A*25:27:02	25:27:02	HLA12472		0	0	1	0	0	0	0	1									
A*25:28	25:28	HLA11787		0	0	3	0	0	0	0	3									

Supplemental Table 8: HLA-A Allele Summary [a]				Allele Count by Population Group [b]								3.0.0 CIWD Category by Population Group [c]								
Allele	Genomic Typing	AlleleID	G group	AFA	API	EURO	MENA	HIS	NAM	UNK	Total	AFA	API	EURO	MENA	HIS	NAM	UNK	Total	Highest Frequency
A*25:32	25:32	HLA13643		0	0	1	0	0	0	0	1									
A*25:39	25:39	HLA15835		0	0	1	0	0	0	0	1									
A*25:CODE	25:CODE			104	42	22163	76	856	69	1582	24892	NA	NA	NA	NA	NA	NA	NA	NA	NA
**A*26:01 total**	**26:01 total**			**4915**	**44894**	**400395**	**22233**	**17620**	**1537**	**48091**	**539685**	**C**	**C**	**C**	**C**	**C**	**C**	**C**	**C**	**C**
A*26:01	26:01			39	5	2544	14	33	7	228	2870	C	WD	C	WD	I	C	C	C	C
A*26:01P	26:01P			0	5	707	3	0	0	7	722		WD	I				WD	I	I
**A*26:01:01G total**	**26:01:01G total**			**4746**	**44844**	**397109**	**22197**	**17573**	**1527**	**47824**	**535820**	**C**	**C**	**C**	**C**	**C**	**C**	**C**	**C**	**C**
A*26:01:01G	26:01:01G		26:01:01G	3663	40763	379447	21590	12419	1061	44317	503260	C	C	C	C	C	C	C	C	C
A*26:01:01	26:01:01		26:01:01G	820	2641	10789	445	3587	284	2480	21046	C	C	C	C	C	C	C	C	C
A*26:01:01:01	26:01:01:01	HLA00073	26:01:01G	254	1243	6679	156	1538	180	997	11047	C	C	C	C	C	C	C	C	C
A*26:01:01:02	26:01:01:02	HLA13482	26:01:01G	4	196	123	6	22	1	22	374		C	I	WD	I		I	I	C
A*26:01:01:05	26:01:01:05	HLA16433	26:01:01G	0	0	10	0	0	0	2	12			WD					WD	WD
A*26:01:01:06	26:01:01:06	HLA16667	26:01:01G	0	0	9	0	0	0	1	10			WD					WD	WD
A*26:01:01:07	26:01:01:07	HLA16668	26:01:01G	0	0	2	0	0	0	0	2									
A*26:01:01:08	26:01:01:08	HLA16669	26:01:01G	1	0	14	0	0	1	0	16			WD					WD	WD
A*26:01:07	26:01:07	HLA03583	26:01:01G	4	0	0	0	0	0	0	4									
A*26:01:32	26:01:32	HLA11083	26:01:01G	0	1	0	0	0	0	0	1									
A*26:01:40	26:01:40	HLA15570	26:01:01G	0	0	35	0	3	0	4	42			WD					WD	WD
A*26:26	26:26	HLA02159	26:01:01G	0	0	1	0	4	0	0	5								WD	WD
A*26:99	26:99	HLA10869	26:01:01G	0	0	0	0	0	0	1	1									
A*26:01:02	26:01:02	HLA02177		0	15	2	1	0	0	1	19		I						WD	I
A*26:01:03	26:01:03	HLA02180		1	4	2	0	0	0	0	7								WD	WD
A*26:01:04	26:01:04	HLA02255		128	0	7	6	2	0	22	165	C		WD	WD			I	I	C
A*26:01:05	26:01:05	HLA02828		0	0	2	0	0	0	0	2									
A*26:01:06	26:01:06	HLA03230		0	18	0	0	0	0	0	18		I						WD	I
A*26:01:09	26:01:09	HLA03876		0	0	1	0	0	0	0	1									
A*26:01:11	26:01:11	HLA03891		0	0	4	0	0	0	1	5								WD	WD
A*26:01:12	26:01:12	HLA04144		0	0	0	0	0	0	1	1									
A*26:01:15	26:01:15	HLA05315		0	0	1	1	0	0	0	2									
A*26:01:16	26:01:16	HLA05339		0	0	1	0	0	0	0	1									

Supplemental Table 8: HLA-A Allele Summary [a]				Allele Count by Population Group [b]								3.0.0 CIWD Category by Population Group [c]								
Allele	Genomic Typing	AlleleID	G group	AFA	API	EURO	MENA	HIS	NAM	UNK	Total	AFA	API	EURO	MENA	HIS	NAM	UNK	Total	Highest Frequency
A*26:01:17	26:01:17	HLA05448		0	0	5	4	0	0	0	9			WD					WD	WD
A*26:01:18	26:01:18	HLA05657		0	0	1	0	0	0	0	1									
A*26:01:19	26:01:19	HLA05941		0	0	0	3	0	0	0	3									
A*26:01:20	26:01:20	HLA05944		1	0	4	0	0	0	0	5								WD	WD
A*26:01:21	26:01:21	HLA06021		0	0	3	0	0	0	1	4									
A*26:01:23	26:01:23	HLA07583		0	1	1	0	12	3	5	22					I		WD	WD	I
A*26:01:24	26:01:24	HLA07584		0	0	1	0	0	0	0	1									
A*26:01:27	26:01:27	HLA08613		0	0	0	0	0	0	1	1									
A*26:01:33	26:01:33	HLA11300		0	2	0	0	0	0	0	2									
A*26:01:37	26:01:37	HLA12456		0	0	0	4	0	0	0	4									
**A*26:02 total**	**26:02 total**			**5**	**1077**	**36**	**0**	**7**	**0**	**334**	**1459**	**WD**	**C**	**WD**		**D**		**C**	**I**	**C**
A*26:02	26:02			2	305	5	0	3	0	85	400		C	WD				I	I	C
A*26:02:01	26:02:01	HLA00074		3	772	31	0	4	0	249	1059		C	WD				C	I	C
**A*26:03 total**	**26:03 total**			**1**	**631**	**41**	**1**	**2**	**1**	**300**	**977**		**C**	**WD**				**C**	**I**	**C**
A*26:03	26:03			0	55	3	0	0	0	15	73		I					I	WD	I
A*26:03:01	26:03:01	HLA00075		1	576	38	1	2	1	285	904		C	WD				C	I	C
A*26:05	26:05	HLA00077		0	9	0	0	0	0	6	15		WD					WD	WD	WD
A*26:06	26:06	HLA00078		0	1	0	0	0	0	5	6							WD	WD	WD
**A*26:07 total**	**26:07 total**			**1**	**97**	**40**	**1**	**12**	**0**	**10**	**161**		**I**	**WD**		**I**		**WD**	**I**	**I**
A*26:07	26:07			0	0	6	0	3	0	2	11			WD					WD	WD
A*26:07:01	26:07:01	HLA00079		0	97	2	1	0	0	1	101		I						WD	I
A*26:07:02	26:07:02	HLA01805		1	0	32	0	9	0	7	49			WD		I		WD	WD	I
A*26:08	26:08	HLA00080		93	30	9465	49	669	38	1106	11450	C	I	C	C	C	C	C	C	C
A*26:09	26:09	HLA00081		2	2	316	7	1	1	15	344			I	WD			I	I	I
A*26:10	26:10	HLA00082		0	8	0	0	1	0	1	10		WD						WD	WD
A*26:12	26:12	HLA00084		185	0	49	22	8	3	44	311	C		WD	WD	I		I	I	C
A*26:13	26:13	HLA01044		0	1	63	0	0	0	3	67			WD					WD	WD
A*26:14	26:14	HLA01120		0	38	20	0	0	0	0	58		I	WD					WD	I
A*26:15	26:15	HLA01272		1	2	398	2	3	0	36	442			I				I	I	I
A*26:16	26:16	HLA01347		0	1	25	22	3	0	1	52			WD	WD				WD	WD

Supplemental Table 8: HLA-A Allele Summary [a]				Allele Count by Population Group [b]								3.0.0 CIWD Category by Population Group [c]								
Allele	Genomic Typing	AlleleID	G group	AFA	API	EURO	MENA	HIS	NAM	UNK	Total	AFA	API	EURO	MENA	HIS	NAM	UNK	Total	Highest Frequency
A*26:17	26:17	HLA01351		1	58	84	125	17	1	40	326		I	WD	C	I		I	I	C
A*26:18	26:18	HLA01525		0	17	1	3	0	0	0	21		I						WD	I
A*26:20	26:20	HLA01876		0	3	1	0	0	0	1	5								WD	WD
A*26:27	26:27	HLA02367		0	1	7	0	8	0	1	17			WD		I			WD	I
A*26:28	26:28	HLA02471		0	0	1	0	0	0	0	1									
A*26:30	26:30	HLA02543		6	2	0	0	0	0	3	11	WD							WD	WD
A*26:31	26:31	HLA02581		0	0	13	0	0	0	2	15			WD					WD	WD
A*26:34	26:34	HLA02731		0	1	0	0	0	0	0	1									
A*26:35	26:35	HLA02939		0	2	0	0	0	0	0	2									
A*26:36	26:36	HLA03297		0	2	0	0	0	0	0	2									
A*26:37	26:37	HLA03329		0	0	6	0	0	0	0	6			WD					WD	WD
A*26:38	26:38	HLA03689		0	0	10	1	0	0	1	12			WD					WD	WD
A*26:39	26:39	HLA03793		0	0	50	0	0	0	1	51			WD					WD	WD
A*26:41	26:41	HLA04143		0	0	0	0	2	0	0	2									
A*26:42	26:42	HLA04523		4	0	1	0	0	0	0	5								WD	WD
**A*26:43 total**	**26:43 total**			**0**	**0**	**1**	**0**	**0**	**0**	**0**	**1**									
A*26:43:01	26:43:01	HLA04726		0	0	1	0	0	0	0	1									
A*26:45	26:45	HLA04785		0	0	36	0	0	0	0	36			WD					WD	WD
A*26:47	26:47	HLA05312		0	0	8	0	0	0	0	8			WD					WD	WD
A*26:49	26:49	HLA05341		0	0	6	0	0	0	1	7			WD					WD	WD
A*26:50	26:50	HLA05407		0	3	0	0	0	0	0	3									
A*26:52	26:52	HLA05649		0	3	2	0	0	0	1	6								WD	WD
A*26:54	26:54	HLA05708		0	0	2	0	0	0	0	2									
A*26:55	26:55	HLA05709		0	0	1	0	0	0	0	1									
A*26:59	26:59	HLA06018		0	0	4	0	1	0	0	5								WD	WD
A*26:62	26:62	HLA06205		0	0	5	0	0	0	0	5			WD					WD	WD
A*26:63	26:63	HLA06209		0	37	0	0	0	0	0	37		I						WD	I
A*26:65	26:65	HLA06283		0	3	5	0	0	0	0	8			WD					WD	WD
A*26:68	26:68	HLA06554		0	0	1	0	0	0	0	1									
A*26:71N	26:71N	HLA07393		0	0	1	0	0	0	0	1									

Supplemental Table 8: HLA-A Allele Summary [a]				Allele Count by Population Group [b]								3.0.0 CIWD Category by Population Group [c]								
**Allele**	**Genomic Typing**	**AlleleID**	**G group**	**AFA**	**API**	**EURO**	**MENA**	**HIS**	**NAM**	**UNK**	**Total**	**AFA**	**API**	**EURO**	**MENA**	**HIS**	**NAM**	**UNK**	**Total**	**Highest Frequency**
A*26:75	26:75	HLA08023		0	0	16	0	0	0	0	16			WD					WD	WD
A*26:76	26:76	HLA08028		0	20	0	0	0	0	0	20		I						WD	I
A*26:77	26:77	HLA08256		0	0	2	0	0	0	0	2									
A*26:80	26:80	HLA08607		0	0	2	0	0	0	0	2									
A*26:83	26:83	HLA08946		0	2	0	0	0	0	0	2									
A*26:86	26:86	HLA09479		0	0	0	0	1	0	0	1									
A*26:87	26:87	HLA09490		0	1	0	0	0	0	0	1									
A*26:90	26:90	HLA09510		0	1	1	0	0	0	1	3									
A*26:92	26:92	HLA09568		0	0	1	0	0	0	0	1									
A*26:94	26:94	HLA09807		0	0	2	0	0	0	0	2									
A*26:95	26:95	HLA10379		0	0	1	0	0	0	0	1									
A*26:100	26:100	HLA11299		0	0	2	0	0	0	0	2									
A*26:103	26:103	HLA11987		0	0	1	0	0	0	0	1									
A*26:104	26:104	HLA12073		0	0	5	0	0	0	0	5			WD					WD	WD
A*26:105	26:105	HLA12123		0	1	0	0	0	0	0	1									
A*26:109	26:109	HLA12880		0	0	0	1	0	0	0	1									
A*26:115	26:115	HLA13618		0	0	1	0	0	0	0	1									
A*26:116	26:116	HLA13724		0	2	0	0	0	0	0	2									
A*26:122	26:122	HLA14905		0	3	0	0	0	0	0	3									
A*26:130	26:130	HLA15852		0	0	1	0	0	0	0	1									
A*26:135	26:135	HLA16086		0	0	1	0	0	0	0	1									
A*26:137	26:137	HLA16106		0	0	1	0	0	0	0	1									
A*26:149	26:149	HLA17993		0	0	1	0	0	0	0	1									
A*26:CODE	26:CODE			400	1259	27933	830	2036	149	4313	36920	NA	NA	NA	NA	NA	NA	NA	NA	NA
**A*29:01 total**	**29:01 total**			**524**	**17101**	**33442**	**5539**	**1710**	**110**	**8005**	**66431**	**C**	**C**	**C**	**C**	**C**	**C**	**C**	**C**	**C**
A*29:01	29:01			10	25	688	70	12	0	740	1545	WD	I	I	C	I		C	I	C
A*29:01P	29:01P			0	0	28	1	0	0	0	29			WD					WD	WD
**A*29:01:01G total**	**29:01:01G total**			**514**	**17074**	**32726**	**5468**	**1698**	**110**	**7265**	**64855**	**C**	**C**	**C**	**C**	**C**	**C**	**C**	**C**	**C**
A*29:01:01G	29:01:01G		29:01:01G	469	15766	31231	5285	1290	86	7001	61128	C	C	C	C	C	C	C	C	C
A*29:01:01	29:01:01		29:01:01G	1	25	54	3	5	1	10	99		I	WD		WD		WD	WD	I

Supplemental Table 8: HLA-A Allele Summary [a]				Allele Count by Population Group [b]								3.0.0 CIWD Category by Population Group [c]								
Allele	Genomic Typing	AlleleID	G group	AFA	API	EURO	MENA	HIS	NAM	UNK	Total	AFA	API	EURO	MENA	HIS	NAM	UNK	Total	Highest Frequency
A*29:01:01:01	29:01:01:01	HLA00085	29:01:01G	44	1283	1441	180	403	23	254	3628	C	C	C	C	C	C	C	C	C
A*29:01:06	29:01:06	HLA10957		0	2	0	0	0	0	0	2									
**A*29:02 total**	**29:02 total**			**10838**	**1506**	**269494**	**3967**	**26363**	**2460**	**34115**	**348743**	**C**	**C**	**C**	**C**	**C**	**C**	**C**	**C**	**C**
A*29:02	29:02			5	0	1271	5	56	1	65	1403	WD		C	WD	I		I	I	C
A*29:02P	29:02P			2	1	425	1	5	0	0	434			I		WD			I	I
**A*29:02:01G total**	**29:02:01G total**			**10830**	**1499**	**267569**	**3958**	**26120**	**2448**	**33969**	**346393**	**C**	**C**	**C**	**C**	**C**	**C**	**C**	**C**	**C**
A*29:02:01G	29:02:01G		29:02:01G	8230	1265	244630	3781	18310	1684	29648	307548	C	C	C	C	C	C	C	C	C
A*29:02:01	29:02:01		29:02:01G	1055	117	10973	88	3203	268	2156	17860	C	I	C	C	C	C	C	C	C
A*29:02:01:01	29:02:01:01	HLA00086	29:02:01G	1110	103	11034	74	4127	433	1913	18794	C	I	C	C	C	C	C	C	C
A*29:02:01:02	29:02:01:02	HLA05528	29:02:01G	390	13	879	13	382	49	228	1954	C	I	I	WD	C	C	C	C	C
A*29:02:01:03	29:02:01:03	HLA14084	29:02:01G	42	1	14	1	44	7	16	125	C		WD		I	C	I	WD	C
A*29:02:01:04	29:02:01:04	HLA16671	29:02:01G	1	0	5	0	50	4	5	65			WD		I		WD	WD	I
A*29:02:07	29:02:07	HLA05902	29:02:01G	1	0	0	0	0	0	0	1									
A*29:02:20	29:02:20	HLA17072	29:02:01G	0	0	6	1	3	1	0	11			WD					WD	WD
A*29:26	29:26	HLA05903	29:02:01G	1	0	4	0	0	1	0	6								WD	WD
A*29:46	29:46	HLA08600	29:02:01G	0	0	4	0	0	1	2	7								WD	WD
A*29:95	29:95	HLA16057	29:02:01G	0	0	20	0	1	0	1	22			WD					WD	WD
A*29:02:02	29:02:02	HLA01703		0	0	119	0	3	1	3	126			WD					WD	WD
A*29:02:03	29:02:03	HLA01887		1	6	67	3	178	10	65	330		WD	WD		C	C	I	I	C
A*29:02:04	29:02:04	HLA03923		0	0	14	0	0	0	0	14			WD					WD	WD
A*29:02:06	29:02:06	HLA05451		0	0	8	0	0	0	8	16			WD				WD	WD	WD
A*29:02:08	29:02:08	HLA05977		0	0	4	0	0	0	0	4									
A*29:02:09	29:02:09	HLA06795		0	0	8	0	0	0	5	13			WD				WD	WD	WD
A*29:02:11	29:02:11	HLA07570		0	0	1	0	0	0	0	1									
A*29:02:12	29:02:12	HLA08249		0	0	7	0	0	0	0	7			WD					WD	WD
A*29:02:14	29:02:14	HLA10272		0	0	1	0	0	0	0	1									
A*29:02:15	29:02:15	HLA12619		0	0	0	0	1	0	0	1									
A*29:03	29:03	HLA00087		0	0	17	1	0	0	0	18			WD					WD	WD
A*29:04	29:04	HLA00088		0	0	50	0	1	0	9	60			WD				WD	WD	WD
A*29:06	29:06	HLA01558		0	0	1	0	0	0	0	1									

Supplemental Table 8: HLA-A Allele Summary [a]				Allele Count by Population Group [b]								3.0.0 CIWD Category by Population Group [c]								
Allele	Genomic Typing	AlleleID	G group	AFA	API	EURO	MENA	HIS	NAM	UNK	Total	AFA	API	EURO	MENA	HIS	NAM	UNK	Total	Highest Frequency
A*29:07	29:07	HLA01650		0	0	6	0	0	0	3	9			WD					WD	WD
A*29:09	29:09	HLA01716		3	0	18	0	1	1	1	24			WD					WD	WD
**A*29:10 total**	**29:10 total**			**2**	**11**	**1360**	**328**	**25**	**1**	**121**	**1848**		**WD**	**C**	**C**	**I**		**I**	**C**	**C**
A*29:10	29:10			2	7	1030	266	18	1	91	1415		WD	I	C	I		I	I	C
A*29:10:01	29:10:01	HLA01795		0	4	330	62	7	0	30	433			I	C	WD		I	I	C
A*29:11	29:11	HLA01852		21	1	6	17	3	0	7	55	WD		WD	WD			WD	WD	WD
A*29:12	29:12	HLA02083		0	0	46	18	1	0	11	76			WD	WD			WD	WD	WD
A*29:13	29:13	HLA02084		2	0	2	0	3	0	0	7								WD	WD
A*29:14	29:14	HLA02256		3	0	1	0	1	0	0	5								WD	WD
A*29:15	29:15	HLA02544		2	0	0	1	0	0	0	3									
A*29:16	29:16	HLA02733		0	0	5	0	0	0	0	5			WD					WD	WD
A*29:17	29:17	HLA03025		0	0	4	0	0	0	0	4									
A*29:18	29:18	HLA03467		0	0	1	0	0	0	0	1									
A*29:19	29:19	HLA03560		0	0	1	0	0	0	0	1									
A*29:20	29:20	HLA03916		0	0	3	0	0	0	0	3									
A*29:21	29:21	HLA04560		0	0	44	1	0	0	2	47			WD					WD	WD
A*29:22	29:22	HLA04584		0	0	1	0	0	0	0	1									
A*29:23	29:23	HLA05428		12	0	0	0	0	0	1	13	WD							WD	WD
A*29:24	29:24	HLA05438		0	0	1	0	0	0	0	1									
A*29:28	29:28	HLA06151		0	0	0	1	0	0	0	1									
A*29:29	29:29	HLA06553		0	0	3	0	0	0	0	3									
A*29:30	29:30	HLA06783		0	0	2	0	0	0	0	2									
A*29:32	29:32	HLA07392		0	0	6	3	2	0	2	13			WD					WD	WD
A*29:34	29:34	HLA07666		0	0	18	0	1	0	0	19			WD					WD	WD
A*29:36	29:36	HLA08027		0	0	4	0	0	0	0	4									
A*29:37	29:37	HLA08030		3	0	0	0	0	0	0	3									
A*29:40	29:40	HLA08092		2	0	2	0	1	0	2	7								WD	WD
A*29:42	29:42	HLA08245		0	0	1	0	0	0	0	1									
A*29:44	29:44	HLA08253		0	0	14	0	0	0	0	14			WD					WD	WD
A*29:45	29:45	HLA08254		0	0	2	0	0	0	0	2									

Supplemental Table 8: HLA-A Allele Summary [a]				Allele Count by Population Group [b]								3.0.0 CIWD Category by Population Group [c]								
Allele	Genomic Typing	AlleleID	G group	AFA	API	EURO	MENA	HIS	NAM	UNK	Total	AFA	API	EURO	MENA	HIS	NAM	UNK	Total	Highest Frequency
A*29:48	29:48	HLA08810		0	0	7	0	0	0	0	7			WD					WD	WD
A*29:51	29:51	HLA09128		0	0	4	0	0	0	0	4									
A*29:53	29:53	HLA09410		1	0	0	0	0	0	0	1									
A*29:54	29:54	HLA09520		0	0	2	0	0	0	0	2									
A*29:55	29:55	HLA09824		0	0	2	0	0	0	0	2									
A*29:56	29:56	HLA10450		0	1	0	0	0	0	0	1									
A*29:60	29:60	HLA11075		0	0	1	0	0	0	0	1									
A*29:61	29:61	HLA11204		0	2	0	0	0	0	0	2									
A*29:62	29:62	HLA11205		0	0	3	0	0	0	0	3									
A*29:64	29:64	HLA11295		0	6	0	0	0	0	0	6		WD						WD	WD
A*29:66	29:66	HLA11390		0	0	1	0	0	0	0	1									
A*29:67	29:67	HLA11780		0	0	1	0	0	0	0	1									
A*29:69	29:69	HLA11782		0	0	3	0	0	0	0	3									
A*29:71	29:71	HLA11968		0	0	2	0	0	0	0	2									
A*29:72	29:72	HLA12184		0	1	1	0	0	0	0	2									
A*29:73	29:73	HLA12233		0	0	1	0	0	0	0	1									
A*29:77	29:77	HLA12925		0	0	0	2	0	0	0	2									
A*29:79	29:79	HLA13430		0	1	0	0	0	0	0	1									
A*29:81	29:81	HLA13944		0	0	2	0	0	0	0	2									
A*29:82	29:82	HLA14121		0	3	0	0	0	0	0	3									
A*29:84	29:84	HLA14608		0	0	0	0	0	0	1	1									
A*29:87	29:87	HLA14964		0	0	1	0	1	0	0	2									
A*29:88	29:88	HLA14968		0	0	2	0	4	0	1	7								WD	WD
A*29:90	29:90	HLA15226		0	0	1	0	0	0	0	1									
A*29:91	29:91	HLA15509		0	0	0	0	0	0	1	1									
A*29:109	29:109	HLA17758		0	0	0	0	0	0	1	1									
A*29:CODE	29:CODE			982	353	24990	327	2949	265	3841	33707	NA	NA	NA	NA	NA	NA	NA	NA	NA
**A*30:01 total**	**30:01 total**			**24814**	**18531**	**165306**	**10307**	**11505**	**1366**	**23966**	**255795**	**C**	**C**	**C**	**C**	**C**	**C**	**C**	**C**	**C**
A*30:01	30:01			4	2	1326	1	7	0	69	1409			C		WD		I	I	C
A*30:01P	30:01P			2	0	371	3	1	0	1	378			I					I	I

Supplemental Table 8: HLA-A Allele Summary [a]				Allele Count by Population Group [b]								3.0.0 CIWD Category by Population Group [c]								
Allele	Genomic Typing	AlleleID	G group	AFA	API	EURO	MENA	HIS	NAM	UNK	Total	AFA	API	EURO	MENA	HIS	NAM	UNK	Total	Highest Frequency
**A*30:01:01G total**	**30:01:01G total**			**24806**	**18529**	**163604**	**10301**	**11497**	**1366**	**23895**	**253998**	**C**	**C**	**C**	**C**	**C**	**C**	**C**	**C**	**C**
A*30:01:01G	30:01:01G		30:01:01G	19612	16301	155995	9900	8245	967	21327	232347	C	C	C	C	C	C	C	C	C
A*30:01:01	30:01:01	HLA00089	30:01:01G	5194	2228	7594	401	3252	399	2568	21636	C	C	C	C	C	C	C	C	C
A*30:24	30:24	HLA03097	30:01:01G	0	0	14	0	0	0	0	14			WD					WD	WD
A*30:114	30:114	HLA16675	30:01:01G	0	0	1	0	0	0	0	1									
A*30:01:03	30:01:03	HLA04809		0	0	1	0	0	0	0	1									
A*30:01:04	30:01:04	HLA05681		0	0	2	2	0	0	0	4									
A*30:01:06	30:01:06	HLA08029		1	0	0	0	0	0	0	1									
A*30:01:07	30:01:07	HLA08269		0	0	1	0	0	0	1	2									
A*30:01:08	30:01:08	HLA10451		0	0	1	0	0	0	0	1									
A*30:01:11	30:01:11	HLA14212		1	0	0	0	0	0	0	1									
**A*30:02 total**	**30:02 total**			**23390**	**2326**	**68416**	**5507**	**15031**	**1362**	**18952**	**134984**	**C**	**C**	**C**	**C**	**C**	**C**	**C**	**C**	**C**
A*30:02	30:02			135	5	1686	46	16	6	241	2135	C	WD	C	C	I	WD	C	C	C
A*30:02P	30:02P			2	0	175	5	2	0	2	186			I	WD				I	I
**A*30:02:01G total**	**30:02:01G total**			**23235**	**2321**	**66547**	**5456**	**14999**	**1356**	**18701**	**132615**	**C**	**C**	**C**	**C**	**C**	**C**	**C**	**C**	**C**
A*30:02:01G	30:02:01G		30:02:01G	18162	2119	61576	5205	10601	939	16468	115070	C	C	C	C	C	C	C	C	C
A*30:02:01	30:02:01		30:02:01G	2013	75	1937	87	1748	148	994	7002	C	I	C	C	C	C	C	C	C
A*30:02:01:01	30:02:01:01	HLA00090	30:02:01G	1280	119	2630	156	2013	175	753	7126	C	I	C	C	C	C	C	C	C
A*30:02:01:02	30:02:01:02	HLA11775	30:02:01G	1148	7	380	7	473	68	333	2416	C	WD	I	WD	C	C	C	C	C
A*30:02:01:03	30:02:01:03	HLA12614	30:02:01G	628	1	24	1	164	26	152	996	C		WD		C	C	C	I	C
A*30:33	30:33	HLA04637	30:02:01G	4	0	0	0	0	0	1	5								WD	WD
A*30:02:03	30:02:03	HLA02199		0	0	2	0	13	0	3	18					I			WD	I
A*30:02:04	30:02:04	HLA03142		6	0	0	0	0	0	1	7	WD							WD	WD
A*30:02:05	30:02:05	HLA04643		7	0	0	0	1	0	0	8	WD							WD	WD
A*30:02:06	30:02:06	HLA08037		0	0	5	0	0	0	1	6			WD					WD	WD
A*30:02:09	30:02:09	HLA08622		0	0	0	0	0	0	1	1									
A*30:02:10	30:02:10	HLA08623		1	0	0	0	0	0	1	2									
A*30:02:13	30:02:13	HLA10504		1	0	0	0	0	0	0	1									
A*30:02:14	30:02:14	HLA11316		3	0	0	0	0	0	1	4									
A*30:02:15	30:02:15	HLA11636		0	0	1	0	0	0	0	1									

Supplemental Table 8: HLA-A Allele Summary [a]				Allele Count by Population Group [b]								3.0.0 CIWD Category by Population Group [c]								
Allele	Genomic Typing	AlleleID	G group	AFA	API	EURO	MENA	HIS	NAM	UNK	Total	AFA	API	EURO	MENA	HIS	NAM	UNK	Total	Highest Frequency
A*30:03	30:03	HLA00091		0	0	36	1	0	0	4	41			WD					WD	WD
**A*30:04 total**	**30:04 total**			**1302**	**775**	**17898**	**3093**	**2108**	**241**	**4037**	**29454**	**C**	**C**	**C**	**C**	**C**	**C**	**C**	**C**	**C**
A*30:04	30:04			32	46	1464	204	104	6	299	2155	WD	I	C	C	C	WD	C	C	C
A*30:04P	30:04P			0	0	71	0	0	0	0	71			WD					WD	WD
**A*30:04:01G total**	**30:04:01G total**			**1270**	**729**	**16361**	**2852**	**2004**	**235**	**3737**	**27188**	**C**	**C**	**C**	**C**	**C**	**C**	**C**	**C**	**C**
A*30:04:01G	30:04:01G		30:04:01G	793	441	12934	2386	1130	118	2711	20513	C	C	C	C	C	C	C	C	C
A*30:04:01	30:04:01	HLA00092	30:04:01G	477	288	3427	466	874	117	1026	6675	C	C	C	C	C	C	C	C	C
A*30:04:02	30:04:02	HLA05744		0	0	2	37	0	0	1	40				WD				WD	WD
A*30:07	30:07	HLA00095		100	0	2	0	2	1	15	120	C						I	WD	C
A*30:08	30:08	HLA01112		11	0	1	6	0	0	2	20	WD			WD				WD	WD
A*30:09	30:09	HLA01224		33	0	3	7	14	0	10	67	WD			WD	I		WD	WD	I
A*30:10	30:10	HLA01411		161	18	490	111	894	172	299	2145	C	I	I	C	C	C	C	C	C
**A*30:11 total**	**30:11 total**			**0**	**64**	**11**	**0**	**0**	**0**	**1**	**76**		**I**	**WD**					**WD**	**I**
A*30:11:01	30:11:01	HLA01445		0	0	10	0	0	0	1	11			WD					WD	WD
A*30:11:02	30:11:02	HLA03102		0	64	1	0	0	0	0	65		I						WD	I
A*30:12	30:12	HLA01584		0	0	0	0	2	1	0	3									
A*30:13	30:13	HLA02102		0	0	1	0	0	0	0	1									
A*30:14L	30:14L	HLA02116		0	0	4	0	0	0	0	4									
A*30:15	30:15	HLA02127		0	1	20	1	0	0	2	24			WD					WD	WD
A*30:16	30:16	HLA02439		9	0	1	0	0	0	2	12	WD							WD	WD
A*30:17	30:17	HLA02510		0	0	1	0	0	0	1	2									
A*30:18	30:18	HLA02646		0	8	1	2	0	0	0	11		WD						WD	WD
A*30:19	30:19	HLA02760		0	0	1	1	0	0	0	2									
A*30:22	30:22	HLA02980		0	0	2	0	0	0	0	2									
A*30:23	30:23	HLA02997		0	0	1	0	0	0	0	1									
A*30:25	30:25	HLA03308		0	0	1	1	2	0	1	5								WD	WD
A*30:26	30:26	HLA03502		0	0	2	1	0	0	1	4									
A*30:28	30:28	HLA03564		8	0	0	0	0	0	3	11	WD							WD	WD
A*30:29	30:29	HLA03871		0	0	51	6	1	0	3	61			WD	WD				WD	WD
A*30:30	30:30	HLA04138		1	0	8	0	0	0	0	9			WD					WD	WD

Supplemental Table 8: HLA-A Allele Summary [a]				Allele Count by Population Group [b]								3.0.0 CIWD Category by Population Group [c]								
Allele	Genomic Typing	AlleleID	G group	AFA	API	EURO	MENA	HIS	NAM	UNK	Total	AFA	API	EURO	MENA	HIS	NAM	UNK	Total	Highest Frequency
A*30:31	30:31	HLA04146		5	0	0	0	0	0	1	6	WD							WD	WD
A*30:32	30:32	HLA04522		2	0	0	0	0	0	0	2									
A*30:34	30:34	HLA04651		1	0	1	0	0	0	1	3									
A*30:36	30:36	HLA04850		0	0	18	0	0	0	0	18			WD					WD	WD
A*30:40	30:40	HLA05331		0	0	33	0	0	0	1	34			WD					WD	WD
A*30:41	30:41	HLA05340		0	0	0	1	0	0	0	1									
A*30:42	30:42	HLA05452		0	0	4	0	0	0	0	4									
A*30:44	30:44	HLA05551		0	0	1	0	0	0	0	1									
A*30:45	30:45	HLA05667		3	0	0	0	0	0	0	3									
A*30:46	30:46	HLA05652		0	0	2	0	0	0	0	2									
A*30:47	30:47	HLA06193		0	0	0	5	0	0	0	5				WD				WD	WD
A*30:51	30:51	HLA06206		0	0	2	0	15	3	3	23					I			WD	I
A*30:53	30:53	HLA06742		0	0	4	0	0	0	0	4									
A*30:54	30:54	HLA06749		0	2	1	0	1	0	2	6								WD	WD
A*30:55	30:55	HLA06833		0	1	0	0	0	0	0	1									
A*30:56	30:56	HLA07395		1	0	3	0	0	0	1	5								WD	WD
A*30:57	30:57	HLA07396		0	0	2	0	0	0	0	2									
A*30:58	30:58	HLA07397		0	0	5	0	0	0	0	5			WD					WD	WD
A*30:59N	30:59N	HLA07559		1	0	1	0	0	0	0	2									
A*30:61	30:61	HLA07587		0	0	0	1	0	0	0	1									
A*30:63	30:63	HLA07794		0	0	2	0	0	0	0	2									
A*30:65	30:65	HLA08038		1	0	0	0	0	0	0	1									
A*30:67	30:67	HLA08624		0	0	4	0	0	0	0	4									
A*30:70N	30:70N	HLA08789		7	0	0	0	0	0	1	8	WD							WD	WD
A*30:71	30:71	HLA08949		0	0	1	0	0	0	0	1									
A*30:73N	30:73N	HLA09409		0	0	1	0	0	0	0	1									
A*30:76N	30:76N	HLA09529		0	0	0	1	0	0	1	2									
A*30:78N	30:78N	HLA09705		11	0	2	0	0	0	1	14	WD							WD	WD
A*30:80	30:80	HLA10633		0	0	1	0	0	0	0	1									
A*30:88	30:88	HLA12107		1	0	0	0	0	0	0	1									

Supplemental Table 8: HLA-A Allele Summary [a]				Allele Count by Population Group [b]								3.0.0 CIWD Category by Population Group [c]								
Allele	Genomic Typing	AlleleID	G group	AFA	API	EURO	MENA	HIS	NAM	UNK	Total	AFA	API	EURO	MENA	HIS	NAM	UNK	Total	Highest Frequency
A*30:89	30:89	HLA12174		0	0	1	0	0	0	0	1									
A*30:91	30:91	HLA12245		0	0	0	0	0	0	1	1									
A*30:92	30:92	HLA12549		0	0	1	0	0	0	1	2									
A*30:94	30:94	HLA12947		0	1	0	0	0	0	0	1									
A*30:103	30:103	HLA15520		0	0	0	1	0	0	0	1									
A*30:CODE	30:CODE			2963	669	15473	413	2455	224	3360	25557	NA	NA	NA	NA	NA	NA	NA	NA	NA
**A*31:01 total**	**31:01 total**			**3636**	**38363**	**274467**	**8670**	**31512**	**2958**	**36725**	**396331**	**C**	**C**	**C**	**C**	**C**	**C**	**C**	**C**	**C**
A*31:01	31:01			0	4	1274	3	23	0	41	1345			C		I		I	I	C
A*31:01P	31:01P			0	2	273	2	2	0	1	280			I					I	I
**A*31:01:02G total**	**31:01:02G total**			**3636**	**38358**	**272915**	**8664**	**31407**	**2953**	**36670**	**394603**	**C**	**C**	**C**	**C**	**C**	**C**	**C**	**C**	**C**
A*31:01:02G	31:01:02G		31:01:02G	2758	35119	257878	8469	22392	2132	33148	361896	C	C	C	C	C	C	C	C	C
A*31:01:02	31:01:02		31:01:02G	415	1326	5729	66	3605	320	1714	13175	C	C	C	C	C	C	C	C	C
A*31:01:02:01	31:01:02:01	HLA00097	31:01:02G	458	1909	8907	125	5318	490	1754	18961	C	C	C	C	C	C	C	C	C
A*31:01:02:02	31:01:02:02	HLA12600	31:01:02G	0	0	3	1	1	0	3	8								WD	WD
**A*31:01N**	**31:01N total**			**0**	**1**	**1**	**0**	**0**	**0**	**0**	**2**									
A*31:01:02:03N	31:01:02:03N	HLA13489	31:01:02G	0	1	1	0	0	0	0	2									
A*31:01:02:04	31:01:02:04	HLA15497	31:01:02G	4	2	357	3	89	11	46	512			I		C	C	I	I	C
A*31:01:02:05	31:01:02:05	HLA16401	31:01:02G	0	0	0	0	0	0	1	1									
A*31:01:02:06	31:01:02:06	HLA16404	31:01:02G	0	0	0	0	0	0	1	1									
A*31:14N	31:14N	HLA02651	31:01:02G	0	0	7	0	1	0	1	9			WD					WD	WD
A*31:23	31:23	HLA03417	31:01:02G	0	0	8	0	0	0	2	10			WD					WD	WD
A*31:48	31:48	HLA06122	31:01:02G	1	0	25	0	0	0	0	26			WD					WD	WD
A*31:71	31:71	HLA08929	31:01:02G	0	1	0	0	0	0	0	1									
A*31:119	31:119	HLA16407	31:01:02G	0	0	0	0	1	0	0	1									
A*31:01:04	31:01:04	HLA04466		0	0	3	0	78	5	12	98					C	WD	WD	WD	C
A*31:01:05	31:01:05	HLA04467		0	0	1	0	0	0	0	1									
A*31:01:11	31:01:11	HLA07421		0	0	2	0	0	0	0	2									
A*31:01:12	31:01:12	HLA07568		0	0	1	0	0	0	0	1									
A*31:01:16	31:01:16	HLA08252		0	0	1	1	3	0	0	5								WD	WD
A*31:01:22	31:01:22	HLA10961		0	0	1	0	0	0	0	1									

Supplemental Table 8: HLA-A Allele Summary [a]				Allele Count by Population Group [b]								3.0.0 CIWD Category by Population Group [c]								
Allele	Genomic Typing	AlleleID	G group	AFA	API	EURO	MENA	HIS	NAM	UNK	Total	AFA	API	EURO	MENA	HIS	NAM	UNK	Total	Highest Frequency
A*31:01:23	31:01:23	HLA12237		0	0	1	0	0	0	0	1									
A*31:01:24	31:01:24	HLA12433		0	0	3	0	0	0	1	4									
A*31:01:26	31:01:26	HLA17756		0	0	0	0	0	0	1	1									
A*31:02	31:02	HLA00098		6	4	66	1	735	38	150	1000	WD		WD		C	C	C	I	C
A*31:04	31:04	HLA00100		151	4	17	98	6	4	36	316	C		WD	C	WD		I	I	C
A*31:05	31:05	HLA01199		0	4	0	0	0	0	1	5								WD	WD
A*31:06	31:06	HLA01546		0	390	24	39	0	1	18	472		C	WD	WD			I	I	C
A*31:07	31:07	HLA01603		0	0	1	0	11	0	1	13					I			WD	I
A*31:08	31:08	HLA00066		1	2	439	0	1	0	9	452			I				WD	I	I
A*31:09	31:09	HLA01651		2	1	23	0	240	12	28	306			WD		C	C	I	I	C
A*31:10	31:10	HLA01970		0	0	1	0	1	0	2	4									
A*31:11	31:11	HLA02040		0	13	0	0	0	0	1	14		I						WD	I
A*31:12	31:12	HLA02085		1	684	8	4	0	1	14	712		C	WD				I	I	C
A*31:13	31:13	HLA02355		0	14	1	0	0	0	0	15		I						WD	I
A*31:15	31:15	HLA02737		6	0	41	0	80	0	61	188	WD		WD		C		I	I	C
A*31:16	31:16	HLA02898		1	369	6	12	1	6	7	402		C	WD	WD		WD	WD	I	C
A*31:18	31:18	HLA02934		1	0	2	1	42	2	9	57					I		WD	WD	I
A*31:19	31:19	HLA03016		0	0	5	1	3	0	0	9			WD					WD	WD
A*31:20	31:20	HLA03116		0	0	35	0	0	0	7	42			WD				WD	WD	WD
A*31:21	31:21	HLA03141		0	0	1	0	0	0	0	1									
A*31:24	31:24	HLA03570		0	1	0	0	0	0	0	1									
A*31:25	31:25	HLA03769		0	0	8	0	0	0	0	8			WD					WD	WD
A*31:26	31:26	HLA03920		0	0	81	0	0	0	3	84			WD					WD	WD
A*31:27	31:27	HLA03925		0	0	115	0	0	0	1	116			WD					WD	WD
A*31:28	31:28	HLA04418		0	0	18	2	0	0	4	24			WD					WD	WD
A*31:30	31:30	HLA04746		0	0	1	0	3	0	2	6								WD	WD
A*31:31	31:31	HLA04786		0	0	1	0	0	0	0	1									
A*31:32	31:32	HLA05241		0	1	0	0	0	0	0	1									
A*31:33	31:33	HLA05250		0	1	0	0	0	0	0	1									
A*31:35	31:35	HLA05325		0	0	4	0	0	0	0	4									

Supplemental Table 8: HLA-A Allele Summary [a]				Allele Count by Population Group [b]								3.0.0 CIWD Category by Population Group [c]								
Allele	Genomic Typing	AlleleID	G group	AFA	API	EURO	MENA	HIS	NAM	UNK	Total	AFA	API	EURO	MENA	HIS	NAM	UNK	Total	Highest Frequency
A*31:36	31:36	HLA05345		0	1	2	0	9	2	0	14					I			WD	I
A*31:37	31:37	HLA05346		3	0	0	0	0	0	0	3									
A*31:38	31:38	HLA05447		0	0	7	0	0	0	0	7			WD					WD	WD
A*31:39	31:39	HLA05459		0	0	6	0	0	0	0	6			WD					WD	WD
A*31:40	31:40	HLA05522		0	0	9	0	0	0	19	28			WD				I	WD	I
A*31:41	31:41	HLA05655		0	1	0	0	3	0	0	4									
A*31:42	31:42	HLA05666		0	0	0	0	0	0	1	1									
A*31:43	31:43	HLA05671		0	0	1	0	0	0	2	3									
A*31:44	31:44	HLA05682		0	0	0	0	0	0	1	1									
A*31:47	31:47	HLA05936		0	0	3	0	0	0	0	3									
A*31:51	31:51	HLA06782		1	2	0	0	1	0	0	4									
A*31:54	31:54	HLA06837		0	0	0	0	3	0	0	3									
A*31:60N	31:60N	HLA07522		0	0	4	0	0	0	2	6								WD	WD
A*31:63	31:63	HLA08032		0	0	1	0	0	0	0	1									
A*31:64	31:64	HLA08034		0	1	0	0	0	0	0	1									
A*31:66	31:66	HLA08093		0	0	2	0	0	0	0	2									
A*31:68	31:68	HLA08608		0	7	0	0	2	0	3	12		WD						WD	WD
A*31:69	31:69	HLA08609		0	0	0	0	0	0	1	1									
A*31:75	31:75	HLA09776		0	0	1	0	0	0	0	1									
A*31:77	31:77	HLA09778		1	0	0	0	0	0	0	1									
A*31:80	31:80	HLA10373		0	0	0	0	1	0	0	1									
A*31:84	31:84	HLA10938		0	0	2	0	0	0	0	2									
A*31:85	31:85	HLA10958		1	0	2	0	0	0	0	3									
A*31:86	31:86	HLA10959		0	2	0	0	0	0	0	2									
A*31:88	31:88	HLA11202		0	2	1	16	0	0	0	19				WD				WD	WD
A*31:89	31:89	HLA12099		0	2	0	0	0	0	0	2									
A*31:90	31:90	HLA12918		0	0	0	0	0	0	1	1									
A*31:96	31:96	HLA13428		0	0	0	0	2	0	0	2									
A*31:97	31:97	HLA13372		0	0	0	0	1	0	0	1									
A*31:98	31:98	HLA13722		0	2	0	0	0	0	0	2									

Supplemental Table 8: HLA-A Allele Summary [a]				Allele Count by Population Group [b]								3.0.0 CIWD Category by Population Group [c]								
Allele	Genomic Typing	AlleleID	G group	AFA	API	EURO	MENA	HIS	NAM	UNK	Total	AFA	API	EURO	MENA	HIS	NAM	UNK	Total	Highest Frequency
A*31:100	31:100	HLA13943		0	0	0	0	1	0	0	1									
A*31:101	31:101	HLA13781		0	1	0	0	0	0	0	1									
A*31:102	31:102	HLA14119		0	2	0	0	0	0	0	2									
A*31:105	31:105	HLA14424		0	2	0	0	0	0	0	2									
A*31:109	31:109	HLA14970		0	0	0	0	0	0	2	2									
A*31:123	31:123	HLA17092		0	0	0	0	0	0	1	1									
A*31:CODE	31:CODE			323	930	21289	246	3646	236	3436	30106	NA	NA	NA	NA	NA	NA	NA	NA	NA
**A*32:01 total**	**32:01 total**			**5608**	**31160**	**400758**	**18470**	**16586**	**1641**	**41000**	**515223**	**C**	**C**	**C**	**C**	**C**	**C**	**C**	**C**	**C**
A*32:01	32:01			1007	1107	52176	1827	2004	298	5946	64365	C	C	C	C	C	C	C	C	C
A*32:01P	32:01P			0	1	717	6	2	0	0	726			I	WD				I	I
**A*32:01:01G total**	**32:01:01G total**			**4601**	**30050**	**347797**	**16635**	**14574**	**1341**	**35051**	**450049**	**C**	**C**	**C**	**C**	**C**	**C**	**C**	**C**	**C**
A*32:01:01G	32:01:01G		32:01:01G	3449	27917	326953	16100	9695	896	31671	416681	C	C	C	C	C	C	C	C	C
A*32:01:01	32:01:01		32:01:01G	1105	2086	19496	502	4667	431	3211	31498	C	C	C	C	C	C	C	C	C
A*32:01:01:01	32:01:01:01	HLA00101	32:01:01G	45	47	1334	31	178	12	165	1812	C	I	C	WD	C	C	C	C	C
A*32:01:01:02	32:01:01:02	HLA16405	32:01:01G	0	0	0	0	0	0	1	1									
A*32:01:01:03	32:01:01:03	HLA16414	32:01:01G	0	0	1	0	0	0	0	1									
A*32:01:01:04	32:01:01:04	HLA16672	32:01:01G	0	0	1	0	1	0	0	2									
A*32:01:01:05	32:01:01:05	HLA17075	32:01:01G	0	0	2	0	0	0	0	2									
A*32:01:02	32:01:02	HLA02879	32:01:01G	2	0	9	0	29	2	3	45			WD		I			WD	I
A*32:01:23	32:01:23	HLA14096	32:01:01G	0	0	0	0	4	0	0	4									
A*32:53	32:53	HLA08566	32:01:01G	0	0	1	0	0	0	0	1									
A*32:54	32:54	HLA08570	32:01:01G	0	0	0	2	0	0	0	2									
A*32:01:04	32:01:04	HLA04486		0	1	29	0	1	0	1	32			WD					WD	WD
A*32:01:06	32:01:06	HLA05457		0	0	4	0	0	0	0	4									
A*32:01:08	32:01:08	HLA05979		0	0	3	0	0	0	0	3									
A*32:01:09	32:01:09	HLA06335		0	0	14	1	1	0	0	16			WD					WD	WD
A*32:01:10	32:01:10	HLA06539		0	0	1	0	0	0	0	1									
A*32:01:12	32:01:12	HLA07428		0	0	6	0	4	2	0	12			WD					WD	WD
A*32:01:14	32:01:14	HLA08455		0	0	5	1	0	0	1	7			WD					WD	WD
A*32:01:17	32:01:17	HLA11806		0	0	2	0	0	0	0	2									

Supplemental Table 8: HLA-A Allele Summary [a]				Allele Count by Population Group [b]								3.0.0 CIWD Category by Population Group [c]								
Allele	Genomic Typing	AlleleID	G group	AFA	API	EURO	MENA	HIS	NAM	UNK	Total	AFA	API	EURO	MENA	HIS	NAM	UNK	Total	Highest Frequency
A*32:01:18	32:01:18	HLA12249		0	0	0	0	0	0	1	1									
A*32:01:19	32:01:19	HLA12253		0	1	0	0	0	0	0	1									
A*32:01:20	32:01:20	HLA12254		0	0	1	0	0	0	0	1									
A*32:01:21	32:01:21	HLA12557		0	0	1	0	0	0	0	1									
A*32:01:24	32:01:24	HLA15823		0	0	2	0	0	0	0	2									
A*32:02	32:02	HLA00102		0	0	63	2	27	0	18	110			WD		I		I	WD	I
A*32:03	32:03	HLA00103		0	5	160	1	4	0	11	181		WD	I				WD	I	I
A*32:04	32:04	HLA01045		0	2	615	4	6	0	64	691			I		WD		I	I	I
A*32:05	32:05	HLA01207		0	6	0	0	0	0	0	6		WD						WD	WD
A*32:06	32:06	HLA01228		0	0	13	0	1	0	0	14			WD					WD	WD
A*32:07	32:07	HLA01463		3	0	61	0	3	0	11	78			WD				WD	WD	WD
A*32:08	32:08	HLA01886		0	0	52	0	3	0	11	66			WD				WD	WD	WD
A*32:11Q	32:11Q	HLA02413		0	14	0	0	0	0	0	14		I						WD	I
A*32:12	32:12	HLA02615		0	0	5	0	0	0	0	5			WD					WD	WD
A*32:13	32:13	HLA02649		0	0	1	0	0	0	0	1									
A*32:15	32:15	HLA02798		0	0	1	0	0	0	0	1									
A*32:16	32:16	HLA03236		0	0	63	0	0	0	1	64			WD					WD	WD
A*32:17	32:17	HLA03495		0	0	85	2	1	0	4	92			WD					WD	WD
A*32:18	32:18	HLA03571		0	0	69	0	0	0	2	71			WD					WD	WD
A*32:19N	32:19N	HLA03783		0	0	1	0	1	0	0	2									
A*32:21	32:21	HLA04642		1	0	1	0	3	2	0	7								WD	WD
A*32:22	32:22	HLA04749		0	0	4	0	0	0	2	6								WD	WD
A*32:23	32:23	HLA05121		0	0	1	0	0	0	0	1									
A*32:24	32:24	HLA05347		0	0	0	0	2	0	0	2									
**A*32:26 total**	**32:26 total**			**1**	**0**	**3**	**0**	**2**	**0**	**6**	**12**							**WD**	**WD**	**WD**
A*32:26	32:26			1	0	3	0	2	0	3	9								WD	WD
A*32:26:01	32:26:01	HLA05430		0	0	0	0	0	0	3	3									
A*32:27N	32:27N	HLA05632		0	0	25	0	0	0	1	26			WD					WD	WD
A*32:28	32:28	HLA05639		0	2	31	0	1	0	2	36			WD					WD	WD
A*32:29	32:29	HLA05641		0	0	1	0	0	0	0	1									

Supplemental Table 8: HLA-A Allele Summary [a]				Allele Count by Population Group [b]								3.0.0 CIWD Category by Population Group [c]								
Allele	Genomic Typing	AlleleID	G group	AFA	API	EURO	MENA	HIS	NAM	UNK	Total	AFA	API	EURO	MENA	HIS	NAM	UNK	Total	Highest Frequency
**A*32:30 total**	**32:30 total**			**0**	**0**	**0**	**0**	**0**	**0**	**1**	**1**									
A*32:30	32:30			0	0	0	0	0	0	1	1									
A*32:31	32:31	HLA06007		0	0	7	0	0	0	1	8			WD					WD	WD
**A*32:33 total**	**32:33 total**			**0**	**0**	**3**	**0**	**0**	**0**	**0**	**3**									
A*32:33	32:33			0	0	1	0	0	0	0	1									
A*32:33:01	32:33:01	HLA06371		0	0	2	0	0	0	0	2									
A*32:36	32:36	HLA06789		0	0	0	1	0	0	0	1									
A*32:37	32:37	HLA07185		0	0	4	0	0	0	0	4									
A*32:38	32:38	HLA07603		0	0	1	0	0	0	0	1									
A*32:39	32:39	HLA07604		0	0	1	0	0	0	0	1									
A*32:40	32:40	HLA07606		0	0	1	0	0	0	0	1									
A*32:42	32:42	HLA08035		0	0	1	0	0	0	0	1									
A*32:44	32:44	HLA08047		0	0	11	0	0	0	2	13			WD					WD	WD
A*32:45N	32:45N	HLA08048		0	0	17	0	0	1	3	21			WD					WD	WD
A*32:47	32:47	HLA08453		0	0	4	0	0	0	1	5								WD	WD
A*32:51	32:51	HLA08485		0	0	1	0	1	0	0	2									
A*32:52	32:52	HLA08491		0	0	4	0	0	0	0	4									
**A*32:55 total**	**32:55 total**			**0**	**1**	**8**	**0**	**0**	**0**	**1**	**10**			**WD**					**WD**	**WD**
A*32:55	32:55			0	0	2	0	0	0	0	2									
A*32:55:01	32:55:01	HLA08643		0	0	6	0	0	0	1	7			WD					WD	WD
A*32:55:02	32:55:02	HLA08969		0	1	0	0	0	0	0	1									
A*32:58	32:58	HLA09544		0	0	4	0	0	0	0	4									
A*32:60	32:60	HLA10391		0	0	15	0	0	0	1	16			WD					WD	WD
A*32:62	32:62	HLA10984		0	1	9	0	0	0	0	10			WD					WD	WD
A*32:63	32:63	HLA11217		0	0	0	0	0	0	2	2									
A*32:64	32:64	HLA11301		0	1	0	0	0	0	0	1									
A*32:71	32:71	HLA12970		0	0	0	1	0	0	0	1									
A*32:72	32:72	HLA13243		0	0	1	0	0	0	0	1									
A*32:73	32:73	HLA13453		0	0	1	0	0	0	0	1									
A*32:75	32:75	HLA13848		0	0	0	0	0	0	1	1									

Supplemental Table 8: HLA-A Allele Summary [a]				Allele Count by Population Group [b]								3.0.0 CIWD Category by Population Group [c]								
**Allele**	**Genomic Typing**	**AlleleID**	**G group**	**AFA**	**API**	**EURO**	**MENA**	**HIS**	**NAM**	**UNK**	**Total**	**AFA**	**API**	**EURO**	**MENA**	**HIS**	**NAM**	**UNK**	**Total**	**Highest Frequency**
A*32:86	32:86	HLA15251		0	0	1	0	0	0	0	1									
A*32:98	32:98	HLA16461		0	0	1	0	0	0	0	1									
A*32:CODE	32:CODE			398	617	23076	549	1790	133	3283	29846	NA	NA	NA	NA	NA	NA	NA	NA	NA
**A*33:01 total**	**33:01 total**			**7226**	**1202**	**78237**	**5533**	**12891**	**1095**	**14456**	**120640**	**C**	**C**	**C**	**C**	**C**	**C**	**C**	**C**	**C**
A*33:01	33:01			813	50	5776	248	1291	142	1012	9332	C	I	C	C	C	C	C	C	C
A*33:01P	33:01P			0	0	82	1	1	0	1	85			WD					WD	WD
**A*33:01:01G total**	**33:01:01G total**			**6412**	**1152**	**72361**	**5282**	**11599**	**953**	**13441**	**111200**	**C**	**C**	**C**	**C**	**C**	**C**	**C**	**C**	**C**
A*33:01:01G	33:01:01G		33:01:01G	1804	490	22459	1363	3654	228	5580	35578	C	C	C	C	C	C	C	C	C
A*33:01:01	33:01:01		33:01:01G	4533	657	49581	3894	7750	716	7788	74919	C	C	C	C	C	C	C	C	C
A*33:01:01:01	33:01:01:01	HLA00104	33:01:01G	70	5	310	25	179	9	73	671	C	WD	I	WD	C	C	I	I	C
A*33:01:01:02	33:01:01:02	HLA16434	33:01:01G	0	0	11	0	16	0	0	27			WD		I			WD	I
A*33:01:09	33:01:09	HLA15594	33:01:01G	5	0	0	0	0	0	0	5	WD							WD	WD
A*33:01:02	33:01:02	HLA03885		0	0	9	1	0	0	0	10			WD					WD	WD
A*33:01:03	33:01:03	HLA03926		0	0	9	0	0	0	2	11			WD					WD	WD
A*33:01:05	33:01:05	HLA05660		0	0	0	1	0	0	0	1									
A*33:01:07	33:01:07	HLA08480		1	0	0	0	0	0	0	1									
**A*33:03 total**	**33:03 total**			**17720**	**130258**	**44398**	**6571**	**4432**	**983**	**14607**	**218969**	**C**	**C**	**C**	**C**	**C**	**C**	**C**	**C**	**C**
A*33:03	33:03			8	9	304	0	1	0	31	353	WD	WD	I				I	I	I
A*33:03P	33:03P			3	2	88	3	2	0	0	98			WD					WD	WD
**A*33:03:01G total**	**33:03:01G total**			**17699**	**130197**	**44003**	**6566**	**4429**	**983**	**14574**	**218451**	**C**	**C**	**C**	**C**	**C**	**C**	**C**	**C**	**C**
A*33:03:01G	33:03:01G		33:03:01G	13813	118340	42080	6344	3165	727	11932	196401	C	C	C	C	C	C	C	C	C
A*33:03:01	33:03:01		33:03:01G	3884	11838	1923	221	1264	256	2642	22028	C	C	C	C	C	C	C	C	C
A*33:15	33:15	HLA03246	33:03:01G	1	0	0	0	0	0	0	1									
A*33:25	33:25	HLA03915	33:03:01G	1	17	0	0	0	0	0	18		I						WD	I
A*33:44	33:44	HLA06372	33:03:01G	0	1	0	0	0	0	0	1									
A*33:73N	33:73N	HLA10121	33:03:01G	0	1	0	1	0	0	0	2									
A*33:03:05	33:03:05	HLA04723		1	0	0	2	0	0	0	3									
A*33:03:07	33:03:07	HLA06198		0	35	2	1	0	0	0	38		I						WD	I
A*33:03:08	33:03:08	HLA06204		3	1	1	0	0	0	0	5								WD	WD
A*33:03:09	33:03:09	HLA07284		0	0	0	0	0	0	1	1									

Supplemental Table 8: HLA-A Allele Summary [a]				Allele Count by Population Group [b]								3.0.0 CIWD Category by Population Group [c]								
Allele	Genomic Typing	AlleleID	G group	AFA	API	EURO	MENA	HIS	NAM	UNK	Total	AFA	API	EURO	MENA	HIS	NAM	UNK	Total	Highest Frequency
A*33:03:10	33:03:10	HLA07523		5	1	0	0	0	0	0	6	WD							WD	WD
A*33:03:11	33:03:11	HLA08599		0	2	0	0	0	0	0	2									
A*33:03:16	33:03:16	HLA10375		0	8	0	0	0	0	0	8		WD						WD	WD
A*33:03:25	33:03:25	HLA10962		0	2	0	0	0	0	0	2									
A*33:03:26	33:03:26	HLA11632		0	1	0	0	0	0	0	1									
A*33:03:27	33:03:27	HLA12100		0	1	0	0	0	0	0	1									
A*33:03:28	33:03:28	HLA12620		0	0	0	0	0	0	1	1									
A*33:03:33	33:03:33	HLA18001		1	0	0	0	0	0	0	1									
A*33:04	33:04	HLA00107		0	0	24	1	5	0	14	44			WD		WD		I	WD	I
A*33:05	33:05	HLA00971		43	19	2700	75	78	8	244	3167	C	I	C	C	C	C	C	C	C
A*33:06	33:06	HLA01318		3	0	0	0	1	0	0	4									
A*33:07	33:07	HLA01704		2	0	2	0	0	0	0	4									
A*33:08	33:08	HLA02250		0	2	1	0	1	1	0	5								WD	WD
A*33:09	33:09	HLA02739		0	0	68	0	1	0	0	69			WD					WD	WD
A*33:10	33:10	HLA02807		0	29	0	1	0	0	0	30		I						WD	I
A*33:11	33:11	HLA02918		2	3	0	0	0	0	0	5								WD	WD
A*33:12	33:12	HLA02959		0	0	46	0	1	1	3	51			WD					WD	WD
A*33:13	33:13	HLA02981		0	5	0	0	0	0	0	5		WD						WD	WD
A*33:16	33:16	HLA03286		0	0	0	0	7	0	0	7					WD			WD	WD
**A*33:18 total**	**33:18 total**			**0**	**0**	**0**	**1**	**0**	**0**	**0**	**1**									
A*33:18:01	33:18:01	HLA03349		0	0	0	1	0	0	0	1									
A*33:19	33:19	HLA03388		0	0	0	0	0	0	1	1									
A*33:23	33:23	HLA03566		0	0	1	0	0	0	0	1									
A*33:24	33:24	HLA03817		0	1	0	0	0	0	0	1									
A*33:26	33:26	HLA03921		0	0	2	48	0	0	2	52				C				WD	C
A*33:27	33:27	HLA04057		0	1	74	0	3	0	1	79			WD					WD	WD
A*33:33	33:33	HLA05633		0	0	0	6	0	0	0	6				WD				WD	WD
A*33:34	33:34	HLA05656		0	0	10	0	0	0	0	10			WD					WD	WD
A*33:36	33:36	HLA05965		2	0	0	0	0	0	0	2									
A*33:37	33:37	HLA06011		0	0	3	0	0	0	0	3									

Supplemental Table 8: HLA-A Allele Summary [a]				Allele Count by Population Group [b]								3.0.0 CIWD Category by Population Group [c]								
Allele	Genomic Typing	AlleleID	G group	AFA	API	EURO	MENA	HIS	NAM	UNK	Total	AFA	API	EURO	MENA	HIS	NAM	UNK	Total	Highest Frequency
A*33:42	33:42	HLA06189		0	4	0	0	0	0	0	4									
A*33:48	33:48	HLA06901		0	13	0	0	0	0	0	13		I						WD	I
A*33:50	33:50	HLA07041		0	0	2	0	0	0	0	2									
A*33:51	33:51	HLA07043		0	1	0	0	0	0	0	1									
A*33:52	33:52	HLA07293		0	1	0	0	0	0	1	2									
A*33:54	33:54	HLA07391		0	1	0	1	0	0	0	2									
A*33:55	33:55	HLA07524		0	7	0	0	0	0	0	7		WD						WD	WD
A*33:58	33:58	HLA07567		0	2	0	0	0	0	0	2									
A*33:59	33:59	HLA07795		0	0	1	0	0	0	0	1									
A*33:60	33:60	HLA08042		0	0	6	0	0	0	0	6			WD					WD	WD
A*33:63	33:63	HLA08611		1	0	0	0	0	0	1	2									
A*33:64	33:64	HLA08612		0	0	10	0	0	0	0	10			WD					WD	WD
A*33:65	33:65	HLA08971		0	17	0	0	0	0	0	17		I						WD	I
A*33:68	33:68	HLA09412		0	0	0	0	0	0	1	1									
A*33:70	33:70	HLA09522		0	2	0	0	0	0	0	2									
A*33:71	33:71	HLA09775		0	2	0	0	0	0	0	2									
A*33:72	33:72	HLA09920		0	1	0	0	0	0	0	1									
A*33:80N	33:80N	HLA10850		0	1	0	0	0	0	0	1									
A*33:81	33:81	HLA10851		0	0	2	0	0	0	2	4									
A*33:86	33:86	HLA10956		0	2	0	0	0	0	0	2									
A*33:89	33:89	HLA11778		0	0	1	0	0	0	0	1									
A*33:93	33:93	HLA12101		0	2	0	0	0	0	0	2									
A*33:94	33:94	HLA12240		0	1	0	0	0	0	0	1									
A*33:96N	33:96N	HLA12924		0	1	0	0	0	0	0	1									
A*33:98	33:98	HLA13429		0	1	0	0	0	0	0	1									
A*33:99	33:99	HLA13433		0	1	0	0	0	0	0	1									
A*33:100	33:100	HLA13720		0	1	0	0	0	0	0	1									
A*33:102	33:102	HLA13629		0	1	0	0	0	0	0	1									
A*33:112	33:112	HLA15223		0	2	0	0	0	0	0	2									
A*33:113	33:113	HLA15224		0	1	0	0	0	0	0	1									

Supplemental Table 8: HLA-A Allele Summary [a]				Allele Count by Population Group [b]								3.0.0 CIWD Category by Population Group [c]								
Allele	Genomic Typing	AlleleID	G group	AFA	API	EURO	MENA	HIS	NAM	UNK	Total	AFA	API	EURO	MENA	HIS	NAM	UNK	Total	Highest Frequency
A*33:123N	33:123N	HLA16215		3	0	0	0	0	0	0	3									
A*33:127	33:127	HLA16635		0	0	0	0	0	0	1	1									
A*33:CODE	33:CODE			1589	2719	9967	315	1581	155	3726	20052	NA	NA	NA	NA	NA	NA	NA	NA	NA
**A*34:01 total**	**34:01 total**			**20**	**9387**	**476**	**36**	**139**	**11**	**3075**	**13144**	**WD**	**C**	**I**	**WD**	**C**	**C**	**C**	**C**	**C**
A*34:01	34:01			3	532	24	0	15	0	180	754		C	WD		I		C	I	C
**A*34:01:01G total**	**34:01:01G total**			**17**	**8855**	**452**	**36**	**124**	**11**	**2895**	**12390**	**WD**	**C**	**I**	**WD**	**C**	**C**	**C**	**C**	**C**
A*34:01:01	34:01:01	HLA00108	34:01:01G	17	8855	452	36	124	11	2895	12390	WD	C	I	WD	C	C	C	C	C
**A*34:02 total**	**34:02 total**			**11905**	**78**	**7050**	**651**	**2319**	**336**	**3691**	**26030**	**C**	**I**	**C**	**C**	**C**	**C**	**C**	**C**	**C**
A*34:02	34:02			778	6	536	34	282	24	215	1875	C	WD	I	WD	C	C	C	C	C
A*34:02P	34:02P			0	0	1	0	0	0	0	1									
A*34:02:01	34:02:01	HLA00109		11125	72	6494	617	2037	312	3468	24125	C	I	C	C	C	C	C	C	C
A*34:02:02	34:02:02	HLA05942		1	0	0	0	0	0	1	2									
A*34:02:03	34:02:03	HLA07580		0	0	19	0	0	0	7	26			WD				WD	WD	WD
A*34:02:04	34:02:04	HLA11407		1	0	0	0	0	0	0	1									
A*34:03	34:03	HLA01248		3	0	0	0	0	0	1	4									
A*34:04	34:04	HLA01320		1	0	6	0	0	0	0	7			WD					WD	WD
A*34:05	34:05	HLA01675		1	391	47	4	27	3	157	630		C	WD		I		C	I	C
A*34:06	34:06	HLA01904		0	0	5	0	0	0	1	6			WD					WD	WD
A*34:07	34:07	HLA02363		5	0	0	0	1	0	2	8	WD							WD	WD
A*34:08	34:08	HLA02502		0	0	4	0	0	0	1	5								WD	WD
A*34:09	34:09	HLA05637		0	0	1	0	0	0	0	1									
A*34:10N	34:10N	HLA08617		5	0	0	0	0	0	2	7	WD							WD	WD
A*34:11	34:11	HLA09336		0	2	0	0	0	0	0	2									
A*34:12	34:12	HLA12455		0	0	0	0	0	0	1	1									
A*34:13	34:13	HLA13837		0	0	0	0	1	0	0	1									
A*34:14	34:14	HLA13996		0	1	0	0	0	0	1	2									
A*34:17	34:17	HLA15732		0	1	0	0	0	0	0	1									
A*34:CODE	34:CODE			540	9	496	12	193	20	230	1500	NA	NA	NA	NA	NA	NA	NA	NA	NA
A*36:01	36:01	HLA00110		10464	52	871	245	1317	243	2338	15530	C	I	I	C	C	C	C	C	C
A*36:02	36:02	HLA01290		1	0	4	0	0	0	1	6								WD	WD

Supplemental Table 8: HLA-A Allele Summary [a]				Allele Count by Population Group [b]								3.0.0 CIWD Category by Population Group [c]								
Allele	Genomic Typing	AlleleID	G group	AFA	API	EURO	MENA	HIS	NAM	UNK	Total	AFA	API	EURO	MENA	HIS	NAM	UNK	Total	Highest Frequency
A*36:03	36:03	HLA01497		62	1	4	36	7	2	82	194	C			WD	WD		I	I	C
A*36:04	36:04	HLA01631		0	0	59	1	0	0	23	83			WD				I	WD	I
A*36:05	36:05	HLA04652		2	0	0	0	0	0	0	2									
A*36:CODE	36:CODE			125	0	60	7	29	5	57	283	NA	NA	NA	NA	NA	NA	NA	NA	NA
A*43:01	43:01	HLA00111		52	6	15	186	5	0	19	283	C	WD	WD	C	WD		I	I	C
**A*66:01 total**	**66:01 total**			**5547**	**609**	**54312**	**2367**	**3923**	**387**	**7452**	**74597**	**C**	**C**	**C**	**C**	**C**	**C**	**C**	**C**	**C**
A*66:01	66:01			2	0	387	3	1	0	29	422			I				I	I	I
A*66:01P	66:01P			0	0	82	0	0	0	2	84			WD					WD	WD
**A*66:01:01G total**	**66:01:01G total**			**5545**	**609**	**53843**	**2364**	**3922**	**387**	**7421**	**74091**	**C**	**C**	**C**	**C**	**C**	**C**	**C**	**C**	**C**
A*66:01:01G	66:01:01G		66:01:01G	4379	579	51357	2271	2793	268	6739	68386	C	C	C	C	C	C	C	C	C
A*66:01:01	66:01:01		66:01:01G	1111	29	2320	81	1082	114	645	5382	C	I	C	C	C	C	C	C	C
A*66:01:01:01	66:01:01:01	HLA00112	66:01:01G	55	1	157	12	47	5	36	313	C		I	WD	I	WD	I	I	C
A*66:17	66:17	HLA08116	66:01:01G	0	0	9	0	0	0	1	10			WD					WD	WD
A*66:02	66:02	HLA00113		3208	9	194	9	343	46	694	4503	C	WD	I	WD	C	C	C	C	C
**A*66:03 total**	**66:03 total**			**665**	**2**	**76**	**21**	**113**	**11**	**155**	**1043**	**C**		**WD**	**WD**	**C**	**C**	**C**	**I**	**C**
**A*66:03:01G total**	**66:03:01G total**			**665**	**2**	**76**	**21**	**113**	**11**	**155**	**1043**	**C**		**WD**	**WD**	**C**	**C**	**C**	**I**	**C**
A*66:03	66:03			658	2	74	21	112	11	155	1033	C		WD	WD	C	C	C	I	C
A*66:03:01G	66:03:01G		66:03:01G	7	0	2	0	1	0	0	10	WD							WD	WD
A*66:04	66:04	HLA01375		5	0	0	0	1	0	1	7	WD							WD	WD
A*66:05	66:05	HLA02344		0	0	5	0	0	0	0	5			WD					WD	WD
A*66:06	66:06	HLA02472		0	0	0	0	0	0	1	1									
A*66:12	66:12	HLA04531		0	4	0	0	0	0	0	4									
A*66:13	66:13	HLA04810		0	0	9	0	1	0	1	11			WD					WD	WD
A*66:15	66:15	HLA05317		0	0	1	13	0	0	0	14				WD				WD	WD
A*66:18	66:18	HLA08483		0	0	0	0	0	0	1	1									
A*66:20	66:20	HLA12630		0	0	0	0	1	0	0	1									
A*66:21	66:21	HLA12631		0	0	0	0	1	0	0	1									
A*66:22	66:22	HLA12935		0	0	2	0	0	0	1	3									
A*66:23	66:23	HLA13435		0	0	0	1	0	0	1	2									
A*66:CODE	66:CODE			655	30	8182	160	357	47	698	10129	NA	NA	NA	NA	NA	NA	NA	NA	NA

Supplemental Table 8: HLA-A Allele Summary [a]				Allele Count by Population Group [b]								3.0.0 CIWD Category by Population Group [c]								
**Allele**	**Genomic Typing**	**AlleleID**	**G group**	**AFA**	**API**	**EURO**	**MENA**	**HIS**	**NAM**	**UNK**	**Total**	**AFA**	**API**	**EURO**	**MENA**	**HIS**	**NAM**	**UNK**	**Total**	**Highest Frequency**
**A*68:01 total**	**68:01 total**			**13043**	**59517**	**373870**	**16614**	**31133**	**2773**	**40407**	**537357**	**C**	**C**	**C**	**C**	**C**	**C**	**C**	**C**	**C**
A*68:01	68:01			5797	811	30105	1633	3137	487	7034	49004	C	C	C	C	C	C	C	C	C
A*68:01P	68:01P			0	3	284	3	3	0	4	297			I					I	I
**A*68:01:01G total**	**68:01:01G total**			**5349**	**9734**	**87516**	**8967**	**3442**	**301**	**7427**	**122736**	**C**	**C**	**C**	**C**	**C**	**C**	**C**	**C**	**C**
A*68:01:01G	68:01:01G		68:01:01G	2801	9146	81815	8746	1511	58	5868	109945	C	C	C	C	C	C	C	C	C
A*68:01:01	68:01:01		68:01:01G	941	230	2005	112	725	86	688	4787	C	C	C	C	C	C	C	C	C
A*68:01:01:01	68:01:01:01	HLA00115	68:01:01G	0	0	1	0	0	0	0	1									
A*68:01:01:02	68:01:01:02	HLA05918	68:01:01G	1607	358	3694	109	1206	157	871	8002	C	C	C	C	C	C	C	C	C
A*68:01:01:03	68:01:01:03	HLA16416	68:01:01G	0	0	1	0	0	0	0	1									
**A*68:01:02G total**	**68:01:02G total**			**1890**	**48880**	**255896**	**6004**	**24537**	**1985**	**25915**	**365107**	**C**	**C**	**C**	**C**	**C**	**C**	**C**	**C**	**C**
A*68:01:02G	68:01:02G		68:01:02G	1461	46023	243734	5823	17096	1406	23439	338982	C	C	C	C	C	C	C	C	C
A*68:01:02	68:01:02		68:01:02G	170	1055	4488	77	2875	201	1131	9997	C	C	C	C	C	C	C	C	C
A*68:01:02:01	68:01:02:01	HLA00116	68:01:02G	123	842	1569	39	3939	313	558	7383	C	C	C	WD	C	C	C	C	C
A*68:01:02:02	68:01:02:02	HLA10676	68:01:02G	129	954	5954	65	619	64	764	8549	C	C	C	C	C	C	C	C	C
A*68:01:02:03	68:01:02:03	HLA13997	68:01:02G	7	1	150	0	7	1	21	187	WD		I		WD		I	I	I
A*68:01:02:05	68:01:02:05	HLA17340	68:01:02G	0	0	0	0	0	0	1	1									
A*68:11N	68:11N	HLA00973	68:01:02G	0	0	1	0	0	0	1	2									
A*68:33	68:33	HLA02531	68:01:02G	0	0	0	0	1	0	0	1									
A*68:96	68:96	HLA08803	68:01:02G	0	1	0	0	0	0	0	1									
A*68:152	68:152	HLA15572	68:01:02G	0	4	0	0	0	0	0	4									
A*68:01:03	68:01:03	HLA01726		0	75	1	3	0	0	0	79		I						WD	I
A*68:01:04	68:01:04	HLA02327		0	2	43	0	1	0	21	67			WD				I	WD	I
A*68:01:05	68:01:05	HLA02542		0	0	2	2	0	0	0	4									
A*68:01:06	68:01:06	HLA02889		0	0	6	0	2	0	2	10			WD					WD	WD
A*68:01:08	68:01:08	HLA05443		0	4	0	0	0	0	0	4									
A*68:01:10	68:01:10	HLA06192		0	1	0	0	0	0	0	1									
A*68:01:11	68:01:11	HLA07550		0	0	0	2	3	0	2	7								WD	WD
A*68:01:14	68:01:14	HLA07671		0	0	1	0	0	0	1	2									
A*68:01:17	68:01:17	HLA08620		0	0	4	0	0	0	0	4									
A*68:01:18	68:01:18	HLA08945		0	0	0	0	0	0	1	1									

Supplemental Table 8: HLA-A Allele Summary [a]				Allele Count by Population Group [b]								3.0.0 CIWD Category by Population Group [c]								
**Allele**	**Genomic Typing**	**AlleleID**	**G group**	**AFA**	**API**	**EURO**	**MENA**	**HIS**	**NAM**	**UNK**	**Total**	**AFA**	**API**	**EURO**	**MENA**	**HIS**	**NAM**	**UNK**	**Total**	**Highest Frequency**
A*68:01:19	68:01:19	HLA09413		0	0	4	0	0	0	0	4									
A*68:01:20	68:01:20	HLA09712		6	0	0	0	0	0	0	6	WD							WD	WD
A*68:01:22	68:01:22	HLA11209		0	0	0	0	4	0	0	4									
A*68:01:24	68:01:24	HLA11635		0	4	3	0	0	0	0	7								WD	WD
A*68:01:25	68:01:25	HLA11790		0	0	2	0	0	0	0	2									
A*68:01:26	68:01:26	HLA12106		0	0	1	0	0	0	0	1									
A*68:01:27	68:01:27	HLA12154		1	0	2	0	0	0	0	3									
A*68:01:28	68:01:28	HLA12244		0	0	0	0	3	0	1	4									
A*68:01:29	68:01:29	HLA12939		0	2	0	0	0	0	0	2									
A*68:01:30	68:01:30	HLA12940		0	1	0	0	0	0	0	1									
A*68:01:31	68:01:31	HLA12941		0	0	1	0	1	0	0	2									
**A*68:02 total**	**68:02 total**			**21838**	**734**	**74562**	**3759**	**14677**	**1797**	**16925**	**134292**	**C**	**C**	**C**	**C**	**C**	**C**	**C**	**C**	**C**
A*68:02	68:02			1874	35	5058	256	1784	170	981	10158	C	I	C	C	C	C	C	C	C
A*68:02P	68:02P			0	0	38	1	0	0	1	40			WD					WD	WD
**A*68:02:01G total**	**68:02:01G total**			**19839**	**699**	**69451**	**3502**	**12877**	**1622**	**15908**	**123898**	**C**	**C**	**C**	**C**	**C**	**C**	**C**	**C**	**C**
A*68:02:01G	68:02:01G		68:02:01G	5610	450	49284	2640	3165	128	6045	67322	C	C	C	C	C	C	C	C	C
A*68:02:01	68:02:01		68:02:01G	11300	196	17285	737	6991	1088	8679	46276	C	C	C	C	C	C	C	C	C
A*68:02:01:01	68:02:01:01	HLA00117	68:02:01G	2928	53	2829	122	2689	404	1176	10201	C	I	C	C	C	C	C	C	C
A*68:02:01:02	68:02:01:02	HLA02508	68:02:01G	0	0	17	0	2	1	2	22			WD					WD	WD
A*68:02:01:03	68:02:01:03	HLA02911	68:02:01G	1	0	36	3	30	1	5	76			WD		I		WD	WD	I
A*68:163	68:163	HLA16435	68:02:01G	0	0	0	0	0	0	1	1									
A*68:02:02	68:02:02	HLA03152		116	0	5	0	15	5	34	175	C		WD		I	WD	I	I	C
A*68:02:03	68:02:03	HLA04545		0	0	5	0	0	0	0	5			WD					WD	WD
A*68:02:04	68:02:04	HLA04547		9	0	0	0	1	0	0	10	WD							WD	WD
A*68:02:05	68:02:05	HLA07572		0	0	5	0	0	0	0	5			WD					WD	WD
A*68:02:08	68:02:08	HLA12242		0	0	0	0	0	0	1	1									
**A*68:03 total**	**68:03 total**			**164**	**96**	**587**	**21**	**10269**	**767**	**1550**	**13454**	**C**	**I**	**I**	**WD**	**C**	**C**	**C**	**C**	**C**
A*68:03	68:03			16	5	32	1	1021	60	61	1196	WD	WD	WD		C	C	I	I	C
A*68:03P	68:03P			0	0	1	0	0	0	0	1									
A*68:03:01	68:03:01	HLA00118		148	90	553	20	9246	704	1488	12249	C	I	I	WD	C	C	C	C	C

Supplemental Table 8: HLA-A Allele Summary [a]				Allele Count by Population Group [b]								3.0.0 CIWD Category by Population Group [c]								
Allele	Genomic Typing	AlleleID	G group	AFA	API	EURO	MENA	HIS	NAM	UNK	Total	AFA	API	EURO	MENA	HIS	NAM	UNK	Total	Highest Frequency
A*68:03:02	68:03:02	HLA00119		0	0	0	0	2	0	1	3									
A*68:03:03	68:03:03	HLA05557		0	1	1	0	0	3	0	5								WD	WD
**A*68:04 total**	**68:04 total**			**1**	**0**	**0**	**0**	**3**	**0**	**8**	**12**							**WD**	**WD**	**WD**
A*68:04	68:04			1	0	0	0	1	0	2	4									
A*68:04:02	68:04:02	HLA14972		0	0	0	0	2	0	6	8							WD	WD	WD
A*68:05	68:05	HLA00121		34	20	87	4	1992	110	263	2510	WD	I	WD		C	C	C	C	C
A*68:06	68:06	HLA00122		1	0	3	0	53	2	7	66					I		WD	WD	I
A*68:07	68:07	HLA00123		6	3	23	0	190	13	34	269	WD		WD		C	C	I	I	C
**A*68:08 total**	**68:08 total**			**9**	**0**	**10**	**0**	**0**	**0**	**5**	**24**	**WD**		**WD**				**WD**	**WD**	**WD**
A*68:08	68:08			1	0	2	0	0	0	0	3									
A*68:08:01	68:08:01	HLA00124		8	0	0	0	0	0	2	10	WD							WD	WD
A*68:08:02	68:08:02	HLA03519		0	0	8	0	0	0	3	11			WD					WD	WD
A*68:09	68:09	HLA00125		0	0	1	0	0	0	0	1									
A*68:10	68:10	HLA00972		95	0	3	0	1	0	15	114	C						I	WD	C
A*68:12	68:12	HLA01046		2	8	536	41	59	8	30	684		WD	I	C	I	C	I	I	C
**A*68:13 total**	**68:13 total**			**0**	**2**	**81**	**48**	**0**	**0**	**4**	**135**			**WD**	**C**				**WD**	**C**
A*68:13	68:13			0	1	16	12	0	0	0	29			WD	WD				WD	WD
A*68:13:01	68:13:01	HLA01047		0	1	65	36	0	0	4	106			WD	WD				WD	WD
A*68:15	68:15	HLA01106		112	0	23	0	14	1	20	170	C		WD		I		I	I	C
A*68:16	68:16	HLA01119		1	2	47	0	49	0	63	162			WD		I		I	I	I
A*68:17	68:17	HLA01170		1	3	1027	2	1397	22	1676	4128			I		C	C	C	C	C
A*68:18N	68:18N	HLA01291		0	1	82	0	0	0	2	85			WD					WD	WD
A*68:19	68:19	HLA01303		1	0	5	0	0	0	2	8			WD					WD	WD
A*68:20	68:20	HLA01464		0	1	2	1	34	1	8	47					I		WD	WD	I
**A*68:21 total**	**68:21 total**			**1**	**0**	**0**	**0**	**5**	**0**	**1**	**7**					**D**			**WD**	**WD**
A*68:21:01	68:21:01	HLA01590		1	0	0	0	5	0	1	7					WD			WD	WD
A*68:22	68:22	HLA01595		0	0	39	0	0	0	2	41			WD					WD	WD
A*68:23	68:23	HLA01652		0	2	43	0	63	1	49	158			WD		I		I	WD	I
A*68:24	68:24	HLA01707		8	221	513	27	17	0	29	815	WD	C	I	WD	I		I	I	C
A*68:25	68:25	HLA01822		0	1	184	0	2	1	9	197			I				WD	I	I

Supplemental Table 8: HLA-A Allele Summary [a]				Allele Count by Population Group [b]								3.0.0 CIWD Category by Population Group [c]								
Allele	Genomic Typing	AlleleID	G group	AFA	API	EURO	MENA	HIS	NAM	UNK	Total	AFA	API	EURO	MENA	HIS	NAM	UNK	Total	Highest Frequency
A*68:26	68:26	HLA01918		2	0	28	0	0	0	6	36			WD				WD	WD	WD
**A*68:27 total**	**68:27 total**			**4**	**0**	**10**	**1**	**0**	**0**	**2**	**17**			**WD**					**WD**	**WD**
A*68:27	68:27			1	0	2	0	0	0	0	3									
A*68:27:01	68:27:01	HLA01944		3	0	7	1	0	0	2	13			WD					WD	WD
A*68:27:02	68:27:02	HLA09786		0	0	1	0	0	0	0	1									
A*68:28	68:28	HLA02103		1	0	0	0	0	0	3	4									
A*68:29	68:29	HLA02361		0	0	0	0	6	1	1	8					WD			WD	WD
A*68:30	68:30	HLA02364		1	1	4	0	11	1	1	19					I			WD	I
A*68:31	68:31	HLA02441		2	0	4	0	24	4	3	37					I			WD	I
A*68:32	68:32	HLA02470		0	0	10	0	0	0	2	12			WD					WD	WD
A*68:34	68:34	HLA02595		1	0	0	0	0	0	0	1									
A*68:35	68:35	HLA02624		0	1	214	1	4	0	10	230			I				WD	I	I
A*68:36	68:36	HLA02701		0	0	1	0	6	0	1	8					WD			WD	WD
A*68:37	68:37	HLA02780		0	0	155	0	0	0	3	158			I					WD	I
A*68:38	68:38	HLA02859		0	0	3	14	0	0	0	17				WD				WD	WD
A*68:39	68:39	HLA02940		1	0	0	0	0	0	0	1									
A*68:40	68:40	HLA02982		9	0	0	0	0	0	1	10	WD							WD	WD
A*68:41	68:41	HLA03050		0	0	2	0	0	0	0	2									
**A*68:43 total**	**68:43 total**			**0**	**0**	**3**	**0**	**0**	**0**	**2**	**5**								**WD**	**WD**
A*68:43	68:43			0	0	0	0	0	0	1	1									
A*68:43:01	68:43:01	HLA03340		0	0	3	0	0	0	1	4									
A*68:44	68:44	HLA03533		1	0	0	0	0	0	0	1									
A*68:45	68:45	HLA03561		0	0	2	0	1	0	2	5								WD	WD
A*68:46	68:46	HLA03581		0	0	1	0	6	0	1	8					WD			WD	WD
A*68:49N	68:49N	HLA04774		0	0	1	0	0	0	0	1									
A*68:50	68:50	HLA05123		0	4	0	0	0	0	0	4									
A*68:53	68:53	HLA05342		0	0	2	0	0	0	0	2									
A*68:54	68:54	HLA05350		8	0	1	0	0	0	5	14	WD						WD	WD	WD
**A*68:55 total**	**68:55 total**			**0**	**0**	**16**	**36**	**2**	**0**	**83**	**137**			**WD**	**WD**			**I**	**WD**	**I**
A*68:55	68:55			0	0	4	6	1	0	27	38				WD			I	WD	I

Supplemental Table 8: HLA-A Allele Summary [a]				Allele Count by Population Group [b]								3.0.0 CIWD Category by Population Group [c]								
**Allele**	**Genomic Typing**	**AlleleID**	**G group**	**AFA**	**API**	**EURO**	**MENA**	**HIS**	**NAM**	**UNK**	**Total**	**AFA**	**API**	**EURO**	**MENA**	**HIS**	**NAM**	**UNK**	**Total**	**Highest Frequency**
A*68:55:01	68:55:01	HLA05431		0	0	12	30	0	0	56	98			WD	WD			I	WD	I
A*68:55:02	68:55:02	HLA11988		0	0	0	0	1	0	0	1									
A*68:56	68:56	HLA05437		0	0	2	0	0	0	0	2									
A*68:57	68:57	HLA05442		0	0	12	4	0	0	1	17			WD					WD	WD
A*68:59N	68:59N	HLA05622		0	0	3	0	0	0	0	3									
A*68:60	68:60	HLA05645		4	0	0	0	0	0	2	6								WD	WD
A*68:61	68:61	HLA05650		0	0	1	0	0	0	0	1									
A*68:65	68:65	HLA05710		0	2	1	0	1	0	0	4									
**A*68:66 total**	**68:66 total**			**0**	**1**	**2**	**0**	**0**	**0**	**0**	**3**									
A*68:66	68:66			0	1	2	0	0	0	0	3									
A*68:67	68:67	HLA05938		20	0	0	0	0	0	4	24	WD							WD	WD
A*68:68	68:68	HLA05961		9	0	0	0	0	0	0	9	WD							WD	WD
A*68:69	68:69	HLA05986		0	2	0	0	0	0	1	3									
A*68:70	68:70	HLA06013		0	0	3	0	0	0	0	3									
A*68:71	68:71	HLA06129		0	0	1	0	0	0	0	1									
A*68:72	68:72	HLA06200		0	0	8	0	0	0	0	8			WD					WD	WD
A*68:73	68:73	HLA06340		0	0	3	0	0	0	0	3									
**A*68:75 total**	**68:75 total**			**3**	**0**	**0**	**0**	**1**	**0**	**0**	**4**									
A*68:75	68:75			1	0	0	0	0	0	0	1									
A*68:75:01	68:75:01	HLA06349		0	0	0	0	1	0	0	1									
A*68:75:02	68:75:02	HLA09787		2	0	0	0	0	0	0	2									
A*68:77	68:77	HLA06540		2	0	0	0	0	0	0	2									
A*68:79	68:79	HLA06748		0	0	1	1	1	0	0	3									
A*68:80	68:80	HLA06739		0	0	2	0	0	0	0	2									
A*68:81	68:81	HLA06796		0	0	0	0	1	0	0	1									
A*68:83	68:83	HLA06836		0	0	0	0	2	0	0	2									
A*68:84	68:84	HLA07290		0	0	0	1	0	0	0	1									
A*68:85	68:85	HLA07350		0	0	2	0	5	0	1	8					WD			WD	WD
A*68:86	68:86	HLA07571		0	0	1	0	0	0	0	1									
A*68:89	68:89	HLA08043		0	0	4	0	0	0	0	4									

Supplemental Table 8: HLA-A Allele Summary [a]				Allele Count by Population Group [b]								3.0.0 CIWD Category by Population Group [c]								
**Allele**	**Genomic Typing**	**AlleleID**	**G group**	**AFA**	**API**	**EURO**	**MENA**	**HIS**	**NAM**	**UNK**	**Total**	**AFA**	**API**	**EURO**	**MENA**	**HIS**	**NAM**	**UNK**	**Total**	**Highest Frequency**
A*68:90	68:90	HLA08044		0	0	0	0	0	0	1	1									
A*68:91	68:91	HLA08045		0	0	13	5	0	0	0	18			WD	WD				WD	WD
A*68:93	68:93	HLA08057		0	0	0	0	3	1	0	4									
A*68:95	68:95	HLA08619		0	0	5	0	0	0	0	5			WD					WD	WD
A*68:97	68:97	HLA08940		0	0	0	0	1	0	0	1									
A*68:98	68:98	HLA08948		0	0	0	1	0	0	0	1									
A*68:99	68:99	HLA09108		0	17	2	0	1	0	1	21		I						WD	I
A*68:101	68:101	HLA09659		0	0	7	0	0	0	0	7			WD					WD	WD
**A*68:103 total**	**68:103 total**			**0**	**0**	**0**	**1**	**0**	**0**	**0**	**1**									
A*68:103:02	68:103:02	HLA12080		0	0	0	1	0	0	0	1									
**A*68:104 total**	**68:104 total**			**0**	**0**	**1**	**0**	**7**	**0**	**0**	**8**					**D**			**WD**	**WD**
A*68:104	68:104			0	0	1	0	2	0	0	3									
A*68:104:01	68:104:01	HLA09919		0	0	0	0	5	0	0	5					WD			WD	WD
A*68:106	68:106	HLA10126		1	0	0	0	0	1	0	2									
A*68:110	68:110	HLA10963		2	0	0	0	0	0	0	2									
A*68:111	68:111	HLA10973		0	0	1	0	0	0	0	1									
**A*68:112 total**	**68:112 total**			**0**	**1**	**1**	**0**	**0**	**0**	**0**	**2**									
A*68:112:01	68:112:01	HLA11208		0	0	1	0	0	0	0	1									
A*68:112:02	68:112:02	HLA13840		0	1	0	0	0	0	0	1									
A*68:114	68:114	HLA11791		0	0	2	0	0	0	0	2									
A*68:117	68:117	HLA11971		0	0	0	3	0	0	0	3									
A*68:122	68:122	HLA12454		0	0	0	0	1	0	0	1									
A*68:124	68:124	HLA12548		0	0	0	0	2	0	0	2									
A*68:125	68:125	HLA12623		1	0	0	0	0	0	0	1									
A*68:127	68:127	HLA12628		1	0	0	0	0	0	0	1									
A*68:128	68:128	HLA12927		1	0	0	0	0	0	1	2									
A*68:129	68:129	HLA12936		0	0	1	0	0	0	0	1									
A*68:132	68:132	HLA12943		0	0	0	0	0	0	1	1									
A*68:133	68:133	HLA13439		1	0	0	0	0	0	0	1									
A*68:134	68:134	HLA13725		0	1	0	0	0	0	0	1									

Supplemental Table 8: HLA-A Allele Summary [a]				Allele Count by Population Group [b]								3.0.0 CIWD Category by Population Group [c]								
Allele	Genomic Typing	AlleleID	G group	AFA	API	EURO	MENA	HIS	NAM	UNK	Total	AFA	API	EURO	MENA	HIS	NAM	UNK	Total	Highest Frequency
A*68:136	68:136	HLA13839		0	0	1	0	0	0	0	1									
A*68:139	68:139	HLA14223		0	0	1	0	0	0	0	1									
A*68:140	68:140	HLA14400		1	0	0	0	0	0	0	1									
A*68:146	68:146	HLA14909		0	1	0	0	0	0	0	1									
A*68:148Q	68:148Q	HLA15237		0	0	3	0	0	0	0	3									
A*68:155	68:155	HLA15874		0	0	1	0	0	0	0	1									
A*68:157	68:157	HLA15952		0	1	0	0	0	0	0	1									
A*68:158	68:158	HLA16084		0	0	0	1	0	0	0	1									
A*68:159Q	68:159Q	HLA16120		0	0	2	0	0	0	0	2									
A*68:161	68:161	HLA16449		0	1	0	0	0	0	0	1									
A*68:CODE	68:CODE			3800	1152	39780	875	5414	493	6315	57829	NA	NA	NA	NA	NA	NA	NA	NA	NA
**A*69:01 total**	**69:01 total**			**168**	**381**	**13446**	**2767**	**2089**	**133**	**4969**	**23953**	**C**	**C**	**C**	**C**	**C**	**C**	**C**	**C**	**C**
A*69:01	69:01			160	347	13000	2701	1970	128	4597	22903	C	C	C	C	C	C	C	C	C
A*69:01P	69:01P			0	0	9	1	0	0	4	14			WD					WD	WD
**A*69:01:01G total**	**69:01:01G total**			**8**	**34**	**436**	**65**	**119**	**5**	**368**	**1035**	**WD**	**I**	**I**	**C**	**C**	**WD**	**C**	**I**	**C**
A*69:01:01G	69:01:01G		69:01:01G	3	33	280	0	64	1	119	500		I	I		I		I	I	I
A*69:01:01	69:01:01		69:01:01G	3	0	107	56	34	2	237	439			WD	C	I		C	I	C
A*69:01:01:01	69:01:01:01	HLA00126	69:01:01G	2	1	49	9	20	2	12	95			WD	WD	I		WD	WD	I
A*69:01:01:02	69:01:01:02	HLA16440	69:01:01G	0	0	0	0	1	0	0	1									
A*69:01:02	69:01:02	HLA15518		0	0	1	0	0	0	0	1									
A*69:02	69:02	HLA09785		0	0	1	0	0	0	0	1									
A*69:CODE	69:CODE			8	23	1335	151	55	0	67	1639	NA	NA	NA	NA	NA	NA	NA	NA	NA
**A*74:01 total**	**74:01 total**			**18880**	**1521**	**1976**	**794**	**3587**	**641**	**4867**	**32266**	**C**	**C**	**C**	**C**	**C**	**C**	**C**	**C**	**C**
A*74:01	74:01			2067	10	76	7	480	122	527	3289	C	WD	WD	WD	C	C	C	C	C
A*74:01P	74:01P			1	0	12	0	1	0	1	15			WD					WD	WD
**A*74:01:01G total**	**74:01:01G total**			**16812**	**1511**	**1888**	**787**	**3106**	**519**	**4339**	**28962**	**C**	**C**	**C**	**C**	**C**	**C**	**C**	**C**	**C**
A*74:01:01G	74:01:01G		74:01:01G	14795	1354	1811	779	2576	448	3909	25672	C	C	C	C	C	C	C	C	C
A*74:01:01	74:01:01	HLA00127	74:01:01G	2012	23	75	7	529	69	414	3129	C	I	WD	WD	C	C	C	C	C
**A*74:02 total**	**74:02 total**			**5**	**134**	**2**	**1**	**1**	**2**	**16**	**161**	**WD**	**C**					**I**	**I**	**C**
A*74:02:01	74:02:01		74:01:01G	2	53	0	0	0	0	12	67		I					WD	WD	I

Supplemental Table 8: HLA-A Allele Summary [a]				Allele Count by Population Group [b]								3.0.0 CIWD Category by Population Group [c]								
Allele	Genomic Typing	AlleleID	G group	AFA	API	EURO	MENA	HIS	NAM	UNK	Total	AFA	API	EURO	MENA	HIS	NAM	UNK	Total	Highest Frequency
A*74:02:01:02	74:02:01:02	HLA05527	74:01:01G	3	81	2	1	1	2	4	94		I						WD	I
A*74:03	74:03	HLA00129		358	98	4571	507	107	6	304	5951	C	I	C	C	C	WD	C	C	C
A*74:04	74:04	HLA01115		2	0	0	0	0	0	0	2									
A*74:05	74:05	HLA01254		0	48	6	1	0	0	4	59		I	WD					WD	I
A*74:06	74:06	HLA01422		2	0	252	0	3	0	15	272			I				I	I	I
A*74:07	74:07	HLA01519		0	0	4	0	0	0	2	6								WD	WD
A*74:08	74:08	HLA01544		0	0	1	0	9	0	0	10					I			WD	I
A*74:09	74:09	HLA01653		126	2	5	0	6	0	26	165	C		WD		WD		I	I	C
A*74:10	74:10	HLA01819		5	0	0	0	0	0	2	7	WD							WD	WD
A*74:11	74:11	HLA02254		207	1	7	0	12	6	36	269	C		WD		I	WD	I	I	C
A*74:13	74:13	HLA03499		2	1	2	0	0	0	4	9								WD	WD
A*74:14N	74:14N	HLA03771		0	0	3	0	0	0	0	3									
**A*74:16 total**	**74:16 total**			**12**	**0**	**1**	**0**	**0**	**0**	**2**	**15**	**WD**							**WD**	**WD**
A*74:16	74:16			4	0	0	0	0	0	0	4									
A*74:16:01	74:16:01	HLA08280		4	0	1	0	0	0	0	5								WD	WD
A*74:16:02	74:16:02	HLA11218		4	0	0	0	0	0	2	6								WD	WD
A*74:17	74:17	HLA08456		2	0	0	0	0	0	1	3									
A*74:18	74:18	HLA10117		1	0	0	0	0	0	1	2									
A*74:19	74:19	HLA10137		0	0	0	0	1	0	0	1									
A*74:20	74:20	HLA10390		1	0	0	0	0	0	0	1									
A*74:22	74:22	HLA11315		2	0	0	0	0	0	0	2									
A*74:24	74:24	HLA12957		0	0	1	0	0	0	0	1									
A*74:CODE	74:CODE			1150	48	260	11	256	47	259	2031	NA	NA	NA	NA	NA	NA	NA	NA	NA
**A*80:01 total**	**80:01 total**			**2764**	**25**	**1778**	**225**	**1176**	**149**	**1164**	**7281**	**C**	**I**	**C**	**C**	**C**	**C**	**C**	**C**	**C**
**A*80:01:01G total**	**80:01:01G total**			**2764**	**25**	**1778**	**225**	**1176**	**149**	**1164**	**7281**	**C**	**I**	**C**	**C**	**C**	**C**	**C**	**C**	**C**
A*80:01	80:01			907	6	358	29	345	47	244	1936	C	WD	I	WD	C	C	C	C	C
A*80:01:01G	80:01:01G		80:01:01G	601	6	1147	158	243	7	430	2592	C	WD	I	C	C	C	C	C	C
A*80:01P	80:01P			0	0	2	0	0	0	0	2									
A*80:01:01	80:01:01		80:01:01G	927	7	226	35	401	63	381	2040	C	WD	I	WD	C	C	C	C	C
A*80:01:01:01	80:01:01:01	HLA00130	80:01:01G	2	0	0	0	1	0	0	3									

Supplemental Table 8: HLA-A Allele Summary [a]				Allele Count by Population Group [b]								3.0.0 CIWD Category by Population Group [c]								
**Allele**	**Genomic Typing**	**AlleleID**	**G group**	**AFA**	**API**	**EURO**	**MENA**	**HIS**	**NAM**	**UNK**	**Total**	**AFA**	**API**	**EURO**	**MENA**	**HIS**	**NAM**	**UNK**	**Total**	**Highest Frequency**
A*80:01:01:02	80:01:01:02	HLA08791	80:01:01G	327	6	45	3	186	32	109	708	C	WD	WD		C	C	I	I	C
A*80:CODE	80:CODE			3	0	26	0	15	2	10	56	NA	NA	NA	NA	NA	NA	NA	NA	NA
A*NEW [d]	NEW			0	1	3	0	0	0	0	4	NA	NA	NA	NA	NA	NA	NA	NA	NA
**A*Total [e]**	**Total**			**388476**	**1291125**	**11929417**	**402447**	**700632**	**66971**	**1320493**	**16099561**									

C, common; I, intermediate; WD, well-documented; NA, not applicable

[a] All alleles observed in the current dataset are included in this table. Note that alleles are not in numerical order; alleles within a G group are clustered together. P group "two-field" total (e.g., written as "A*01:01 total") and G group total summary rows are provided. The table does not list all alleles from IPD-IMGT version 3.31.0, if not present in the study dataset.

[b] Population groups include: AFA (African/African American), API (Asian/Pacific Islands), EURO (European/European descent), MENA (Middle East/North Coast of Africa), HIS (South or Central America/Hispanic/Latino), NAM (Native American populations) and UNK (unknown/not asked/multiple ancestries/other). Total is the overall population i.e., all groups combined.

[c] Allele frequency is calculated by dividing the number of times the “allele” of interest is observed in a population by the total number of copies of all the alleles at that particular genetic locus in the population (reported as the last row in this table and also in Table 2b). The total number of copies is calculated by multiplying the number of individuals times two for all loci except DRB3/4/5. For DRB3/4/5, the number of assignments was used as the total. The CIWD status is determined based on the allele frequency. Allele frequency data will be provided on the website of the next International HLA and Immunogenetics Workshop (https://www.ihiw18.org/). Highest frequency is the highest CIWD designation among all the individual groups.

[d] "CODE" is generically defined as a summary category of submitted HLA typing, including NMDP multiple allele codes, with ambiguities that are not within a single P or G group. "NEW" is a summary category for assignments of novel alleles that did not yet receive a nomenclature assignment. The CODE and NEW categories add to the total number of alleles but should not be assigned CIWD designations (labeled as NA, not applicable) as they do not represent a consistent allele designation (i.e., the NEW category may contain alleles with different DNA sequences that are unrelated to one another).

[e] A*Total is the total number of allele assignments for the population group and is based on two times the number of individuals in the group. This number is also listed in Table 2b. It is not the sum of the column as alleles are not counted more than once. For example, when evaluating frequencies at the level of G resolution, individual alleles that make up the G group (e.g., A*80:01:01, A*80:01:01:01, A*80:01:01:02, A*80:01:01G) are not included in the count because these alleles are summed up in the total G designation (e.g., "A*80:01:01G total").
