# Supplementary material for: Common, intermediate and well‐documented HLA alleles in world populations: CIWD version 3.0.0
Source: HLA. 2020 Jan 31;95(6):516–31. doi: 10.1111/tan.13811 (PMC7317522; doi:10.1111/tan.13811)
Supplement: Supplementary file 9 — Table S9 HLA‐B primary data [file TAN-95-516-s009.pdf]

Supplemental Table 9: HLA-B Allele Summary [a]				Allele Count by Population Group [b]								3.0.0 CIWD Category by Population Group [c]								
Allele	Genomic typing	Allele ID	G group	AFA	API	EURO	MENA	HIS	NAM	UNK	Total	AFA	API	EURO	MENA	HIS	NAM	UNK	Total	Highest Frequency
**B*07:02 total**	**07:02 total**			**26182**	**48874**	**1398284**	**14506**	**37930**	**4127**	**115074**	**1644977**	**C**	**C**	**C**	**C**	**C**	**C**	**C**	**C**	**C**
B*07:02	07:02			4	2	4073	8	12	0	157	4256			C	WD	I		C	C	C
B*07:02P	07:02P			1	3	754	3	4	0	3	768			I					I	I
**B*07:02:01G total**	**07:02:01G total**			**26160**	**48865**	**1392994**	**14479**	**37907**	**4126**	**114707**	**1639238**	**C**	**C**	**C**	**C**	**C**	**C**	**C**	**C**	**C**
B*07:02:01G	07:02:01G		07:02:01G	21684	46303	1332475	14129	29042	3293	106417	1553343	C	C	C	C	C	C	C	C	C
B*07:02:01	07:02:01		07:02:01G	4091	2358	54459	327	8027	770	7519	77551	C	C	C	C	C	C	C	C	C
B*07:02:01:01	07:02:01:01	HLA00132	07:02:01G	384	203	5971	23	832	63	758	8234	C	C	C	WD	C	C	C	C	C
B*07:02:01:02	07:02:01:02	HLA16169	07:02:01G	0	0	1	0	0	0	0	1									
B*07:02:01:04	07:02:01:04	HLA16855	07:02:01G	0	0	9	0	0	0	1	10			WD					WD	WD
B*07:02:01:07	07:02:01:07	HLA17071	07:02:01G	0	0	6	0	1	0	1	8			WD					WD	WD
B*07:02:06	07:02:06	HLA02990	07:02:01G	0	0	5	0	0	0	0	5			WD					WD	WD
B*07:02:45	07:02:45	HLA12872	07:02:01G	1	1	58	0	2	0	7	69			WD				WD	WD	WD
B*07:44N	07:44N	HLA02350	07:02:01G	0	0	0	0	2	0	1	3									
B*07:59	07:59	HLA02991	07:02:01G	0	0	3	0	0	0	0	3									
B*07:61	07:61	HLA03166	07:02:01G	0	0	0	0	0	0	1	1									
B*07:120	07:120	HLA06131	07:02:01G	0	0	7	0	0	0	1	8			WD					WD	WD
B*07:129	07:129	HLA06658	07:02:01G	0	0	0	0	0	0	1	1									
B*07:282	07:282	HLA15577	07:02:01G	0	0	0	0	1	0	0	1									
B*07:02:02	07:02:02	HLA00133		0	0	7	0	0	0	0	7			WD					WD	WD
B*07:02:03	07:02:03	HLA00134		12	1	124	1	7	0	5	150	WD		I		WD		WD	WD	I
B*07:02:04	07:02:04	HLA01763		0	0	81	0	1	0	191	273			WD				C	I	C
B*07:02:05	07:02:05	HLA02949		0	0	7	0	0	0	0	7			WD					WD	WD
B*07:02:07	07:02:07	HLA03089		0	0	55	0	0	0	0	55			WD					WD	WD
B*07:02:08	07:02:08	HLA03720		0	0	8	0	0	0	0	8			WD					WD	WD
B*07:02:10	07:02:10	HLA03900		0	0	47	0	0	1	2	50			WD					WD	WD
B*07:02:11	07:02:11	HLA03938		0	0	1	0	1	0	1	3									
B*07:02:12	07:02:12	HLA04042		0	0	3	0	0	0	0	3									
B*07:02:13	07:02:13	HLA04058		5	0	18	4	0	0	1	28	WD		WD					WD	WD
B*07:02:14	07:02:14	HLA04113		0	0	8	0	0	0	0	8			WD					WD	WD
B*07:02:15	07:02:15	HLA04191		0	0	2	0	0	0	0	2									
B*07:02:16	07:02:16	HLA04198		0	0	3	0	0	0	0	3									

Supplemental Table 9: HLA-B Allele Summary [a]				Allele Count by Population Group [b]								3.0.0 CIWD Category by Population Group [c]								
Allele	Genomic typing	Allele ID	G group	AFA	API	EURO	MENA	HIS	NAM	UNK	Total	AFA	API	EURO	MENA	HIS	NAM	UNK	Total	Highest Frequency
B*07:02:17	07:02:17	HLA04238		0	0	0	11	0	0	0	11				WD				WD	WD
B*07:02:18	07:02:18	HLA04246		0	2	0	0	0	0	0	2									
B*07:02:19	07:02:19	HLA04571		0	0	1	0	0	0	1	2									
B*07:02:20	07:02:20	HLA05253		0	0	1	0	0	0	0	1									
B*07:02:21	07:02:21	HLA06678		0	0	16	0	0	0	1	17			WD					WD	WD
B*07:02:22	07:02:22	HLA06679		0	0	1	0	0	0	0	1									
B*07:02:23	07:02:23	HLA06702		0	0	5	0	0	0	0	5			WD					WD	WD
B*07:02:24	07:02:24	HLA06703		0	0	1	0	0	0	0	1									
B*07:02:27	07:02:27	HLA06924		0	0	19	0	0	0	0	19			WD					WD	WD
B*07:02:28	07:02:28	HLA06981		0	0	5	0	0	0	1	6			WD					WD	WD
B*07:02:29	07:02:29	HLA07207		0	0	1	0	0	0	0	1									
B*07:02:30	07:02:30	HLA07467		0	0	1	0	0	0	0	1									
B*07:02:33	07:02:33	HLA08136		0	0	4	0	0	0	0	4									
B*07:02:34	07:02:34	HLA08379		0	1	2	0	0	0	0	3									
B*07:02:35	07:02:35	HLA08385		0	0	3	0	0	0	0	3									
B*07:02:36	07:02:36	HLA08685		0	0	4	0	0	0	0	4									
B*07:02:37	07:02:37	HLA08686		0	0	0	0	0	0	2	2									
B*07:02:42	07:02:42	HLA11839		0	0	0	0	0	0	1	1									
B*07:02:43	07:02:43	HLA12017		0	0	10	0	0	0	0	10			WD					WD	WD
B*07:02:46	07:02:46	HLA13018		0	0	0	0	0	0	1	1									
B*07:02:48	07:02:48	HLA13791		0	0	1	0	0	0	0	1									
B*07:252	07:252	HLA13341		0	0	24	0	0	0	1	25			WD					WD	WD
B*07:03	07:03	HLA00135		0	1	233	0	5	0	15	254			I		WD		I	I	I
B*07:04	07:04	HLA00136		13	11	13038	17	45	3	175	13302	WD	WD	C	WD	I		C	C	C
**B*07:05 total**	**07:05 total**			**3147**	**41501**	**33895**	**5028**	**3360**	**307**	**9358**	**96596**	**C**	**C**	**C**	**C**	**C**	**C**	**C**	**C**	**C**
B*07:05	07:05			0	4	282	2	0	0	42	330			I				I	I	I
B*07:05P	07:05P			1	3	127	2	1	0	0	134			I					WD	I
**B*07:05:01G total**	**07:05:01G total**			**2936**	**41133**	**33306**	**5020**	**3160**	**283**	**9199**	**95037**	**C**	**C**	**C**	**C**	**C**	**C**	**C**	**C**	**C**
B*07:05:01G	07:05:01G		07:05:01G	2615	39424	31904	4882	2576	231	8803	90435	C	C	C	C	C	C	C	C	C
B*07:05:01	07:05:01		07:05:01G	17	609	551	81	126	11	109	1504	WD	C	I	C	C	C	I	I	C
B*07:05:01:01	07:05:01:01	HLA00137	07:05:01G	34	526	567	52	114	6	105	1404	WD	C	I	C	C	WD	I	I	C

Supplemental Table 9: HLA-B Allele Summary [a]				Allele Count by Population Group [b]								3.0.0 CIWD Category by Population Group [c]								
**Allele**	**Genomic typing**	**Allele ID**	**G group**	**AFA**	**API**	**EURO**	**MENA**	**HIS**	**NAM**	**UNK**	**Total**	**AFA**	**API**	**EURO**	**MENA**	**HIS**	**NAM**	**UNK**	**Total**	**Highest Frequency**
B*07:05:01:04	07:05:01:04	HLA16875	07:05:01G	0	1	0	0	0	0	0	1									
B*07:06:01	07:06:01	HLA00138	07:05:01G	270	573	284	5	344	35	182	1693	C	C	I	WD	C	C	C	C	C
B*07:05:02	07:05:02	HLA02317		0	0	40	0	0	0	2	42			WD					WD	WD
B*07:05:05	07:05:05	HLA05390		0	0	1	0	0	0	0	1									
B*07:05:07	07:05:07	HLA11838		0	0	2	0	0	0	0	2									
B*07:05:08	07:05:08	HLA12310		0	11	0	0	0	0	0	11		WD						WD	WD
**B*07:06 total**	**07:06 total**			**480**	**923**	**421**	**9**	**543**	**59**	**297**	**2732**	**C**	**C**	**I**	**WD**	**C**	**C**	**C**	**C**	**C**
B*07:06	07:06			210	344	137	4	199	24	115	1033	C	C	I		C	C	I	I	C
B*07:06:02	07:06:02	HLA14776		0	6	0	0	0	0	0	6		WD						WD	WD
B*07:07	07:07	HLA00139		6	2	413	6	13	1	20	461	WD		I	WD	I		I	I	I
**B*07:08 total**	**07:08 total**			**0**	**0**	**64**	**0**	**1**	**0**	**1**	**66**			**WD**					**WD**	**WD**
B*07:08	07:08			0	0	59	0	1	0	1	61			WD					WD	WD
B*07:08:01	07:08:01	HLA00140		0	0	5	0	0	0	0	5			WD					WD	WD
B*07:09	07:09	HLA00141		136	19	625	3	35	17	70	905	C	I	I		I	C	I	I	C
B*07:10	07:10	HLA00142		12	16	1264	119	24	2	153	1590	WD	I	C	C	I		C	I	C
B*07:12	07:12	HLA00144		54	0	0	0	2	0	12	68	C						WD	WD	C
B*07:13	07:13	HLA00145		13	0	0	0	0	2	3	18	WD							WD	WD
B*07:14	07:14	HLA01049		3	0	686	50	80	5	63	887			I	C	C	WD	I	I	C
B*07:15	07:15	HLA01050		1	0	410	1	0	0	13	425			I				WD	I	I
B*07:16	07:16	HLA01051		0	0	20	0	0	1	2	23			WD					WD	WD
B*07:17	07:17	HLA01110		0	0	2	0	0	0	4	6								WD	WD
**B*07:18 total**	**07:18 total**			**0**	**42**	**4**	**0**	**1**	**0**	**2**	**49**		**I**						**WD**	**I**
B*07:18	07:18			0	1	1	0	0	0	0	2									
B*07:18:01	07:18:01	HLA01116		0	41	1	0	0	0	2	44		I						WD	I
B*07:18:02	07:18:02	HLA03088		0	0	2	0	1	0	0	3									
B*07:19	07:19	HLA01176		0	0	7	0	1	0	2	10			WD					WD	WD
B*07:20	07:20	HLA01184		0	0	17	0	27	1	5	50			WD		I		WD	WD	I
B*07:21	07:21	HLA01253		0	0	2	0	0	0	1	3									
**B*07:22 total**	**07:22 total**			**3**	**1**	**104**	**0**	**1**	**1**	**24**	**134**			**WD**				**I**	**WD**	**I**
B*07:22	07:22			0	0	18	0	0	0	2	20			WD					WD	WD
B*07:22:01	07:22:01	HLA01257		2	1	56	0	1	0	16	76			WD				I	WD	I

Supplemental Table 9: HLA-B Allele Summary [a]				Allele Count by Population Group [b]								3.0.0 CIWD Category by Population Group [c]								
**Allele**	**Genomic typing**	**Allele ID**	**G group**	**AFA**	**API**	**EURO**	**MENA**	**HIS**	**NAM**	**UNK**	**Total**	**AFA**	**API**	**EURO**	**MENA**	**HIS**	**NAM**	**UNK**	**Total**	**Highest Frequency**
B*07:22:02	07:22:02	HLA03260		1	0	30	0	0	1	6	38			WD				WD	WD	WD
B*07:23	07:23	HLA01286		2	0	25	1	0	0	7	35			WD				WD	WD	WD
B*07:24	07:24	HLA01293		0	0	5	0	0	0	4	9			WD					WD	WD
B*07:26	07:26	HLA01362		0	5	98	0	12	1	5	121		WD	WD		I		WD	WD	I
B*07:27	07:27	HLA01435		0	0	1	0	0	0	0	1									
B*07:28	07:28	HLA01516		0	0	1	0	0	0	0	1									
B*07:29	07:29	HLA01548		0	0	3	0	0	0	3	6								WD	WD
B*07:30	07:30	HLA01577		0	1	1	0	0	0	0	2									
B*07:31	07:31	HLA01618		2	0	17	0	0	0	4	23			WD					WD	WD
**B*07:33 total**	**07:33 total**			**2**	**9**	**26**	**0**	**0**	**0**	**0**	**37**		**WD**	**WD**					**WD**	**WD**
B*07:33	07:33			1	2	15	0	0	0	0	18			WD					WD	WD
B*07:33:01	07:33:01	HLA01742		1	0	10	0	0	0	0	11			WD					WD	WD
B*07:33:02	07:33:02	HLA09701		0	5	0	0	0	0	0	5		WD						WD	WD
B*07:33:03	07:33:03	HLA11035		0	2	1	0	0	0	0	3									
B*07:36	07:36	HLA01808		0	8	21	1	1	0	9	40		WD	WD				WD	WD	WD
**B*07:37 total**	**07:37 total**			**0**	**0**	**32**	**0**	**0**	**0**	**28**	**60**			**WD**				**I**	**WD**	**I**
**B*07:37:01G total**	**07:37:01G total**			**0**	**0**	**32**	**0**	**0**	**0**	**28**	**60**			**WD**				**I**	**WD**	**I**
B*07:37	07:37		07:37:01G	0	0	32	0	0	0	28	60			WD				I	WD	I
B*07:38	07:38	HLA01883		0	1	72	0	0	0	0	73			WD					WD	WD
B*07:39	07:39	HLA02089		0	0	1	0	0	0	0	1									
B*07:42	07:42	HLA02175		1	0	53	0	1	0	13	68			WD				WD	WD	WD
B*07:43	07:43	HLA02247		0	2	0	0	0	0	0	2									
B*07:46	07:46	HLA02446		0	0	10	0	0	0	19	29			WD				I	WD	I
B*07:47	07:47	HLA02463		0	0	373	0	0	0	6	379			I				WD	I	I
B*07:48	07:48	HLA02514		0	3	13	37	0	0	1	54			WD	WD				WD	WD
B*07:51	07:51	HLA02713		0	0	6	0	15	2	1	24			WD		I			WD	I
B*07:52	07:52	HLA02796		0	0	1	0	0	0	0	1									
B*07:53	07:53	HLA02805		0	0	10	0	0	0	0	10			WD					WD	WD
B*07:54	07:54	HLA02844		0	1	1	0	0	0	0	2									
B*07:55	07:55	HLA02873		0	0	1	0	0	0	0	1									
**B*07:56 total**	**07:56 total**			**0**	**0**	**4**	**0**	**0**	**0**	**7**	**11**							**WD**	**WD**	**WD**

Supplemental Table 9: HLA-B Allele Summary [a]				Allele Count by Population Group [b]								3.0.0 CIWD Category by Population Group [c]								
Allele	Genomic typing	Allele ID	G group	AFA	API	EURO	MENA	HIS	NAM	UNK	Total	AFA	API	EURO	MENA	HIS	NAM	UNK	Total	Highest Frequency
B*07:56	07:56			0	0	1	0	0	0	0	1									
B*07:56:01	07:56:01	HLA02910		0	0	1	0	0	0	0	1									
B*07:56:02	07:56:02	HLA07464		0	0	2	0	0	0	7	9							WD	WD	WD
B*07:57	07:57	HLA02968		1	2	19	0	0	0	3	25			WD					WD	WD
B*07:62	07:62	HLA03179		0	0	71	0	0	0	1	72			WD					WD	WD
B*07:63	07:63	HLA03237		0	0	32	0	0	0	2	34			WD					WD	WD
B*07:64	07:64	HLA03264		0	0	2	0	0	0	1	3									
B*07:66	07:66	HLA03342		0	0	1	0	0	0	0	1									
B*07:67N	07:67N	HLA03413		0	0	18	0	0	0	0	18			WD					WD	WD
**B*07:68 total**	**07:68 total**			**0**	**4**	**60**	**0**	**1**	**0**	**3**	**68**			**WD**					**WD**	**WD**
B*07:68	07:68			0	2	10	0	0	0	0	12			WD					WD	WD
B*07:68:01	07:68:01	HLA03514		0	0	35	0	0	0	2	37			WD					WD	WD
B*07:68:02	07:68:02	HLA05222		0	1	11	0	0	0	0	12			WD					WD	WD
B*07:68:03	07:68:03	HLA06826		0	1	4	0	1	0	1	7								WD	WD
B*07:70	07:70	HLA03610		0	0	2	0	0	0	1	3									
B*07:71	07:71	HLA03611		0	4	1	0	0	0	2	7								WD	WD
B*07:72	07:72	HLA03622		0	0	2	0	0	0	0	2									
B*07:73	07:73	HLA03658		0	0	25	0	0	0	0	25			WD					WD	WD
B*07:74	07:74	HLA03660		0	0	17	0	0	0	0	17			WD					WD	WD
B*07:76	07:76	HLA03692		0	0	0	0	0	0	1	1									
B*07:77	07:77	HLA03761		0	0	2	5	0	0	0	7				WD				WD	WD
B*07:80	07:80	HLA03936		0	12	1	0	0	0	0	13		WD						WD	WD
B*07:81	07:81	HLA03937		0	6	0	1	0	0	0	7		WD						WD	WD
B*07:82	07:82	HLA03940		0	2	0	0	0	0	0	2									
B*07:85	07:85	HLA04074		0	3	1	0	1	0	0	5								WD	WD
B*07:86	07:86	HLA04079		0	0	9	0	0	1	1	11			WD					WD	WD
B*07:87	07:87	HLA04092		0	0	39	0	1	0	2	42			WD					WD	WD
B*07:88	07:88	HLA04097		0	0	60	0	1	1	1	63			WD					WD	WD
B*07:89	07:89	HLA04099		0	0	0	0	0	0	2	2									
B*07:90	07:90	HLA04109		0	0	4	0	0	0	0	4									
B*07:91	07:91	HLA03981		0	0	1	0	0	0	0	1									

Supplemental Table 9: HLA-B Allele Summary [a]				Allele Count by Population Group [b]								3.0.0 CIWD Category by Population Group [c]								
Allele	Genomic typing	Allele ID	G group	AFA	API	EURO	MENA	HIS	NAM	UNK	Total	AFA	API	EURO	MENA	HIS	NAM	UNK	Total	Highest Frequency
B*07:92	07:92	HLA04172		0	1	37	0	1	0	3	42			WD					WD	WD
B*07:93	07:93	HLA04193		0	0	1	0	0	0	0	1									
B*07:94	07:94	HLA04219		0	4	2	0	0	0	1	7								WD	WD
B*07:95	07:95	HLA04233		0	0	20	0	0	0	1	21			WD					WD	WD
**B*07:96 total**	**07:96 total**			**0**	**0**	**7**	**0**	**0**	**0**	**0**	**7**			**WD**					**WD**	**WD**
B*07:96:01	07:96:01	HLA04241		0	0	7	0	0	0	0	7			WD					WD	WD
B*07:97	07:97	HLA04243		0	1	1	0	0	0	0	2									
B*07:98	07:98	HLA04248		0	0	25	0	0	0	0	25			WD					WD	WD
B*07:99	07:99	HLA04250		0	0	1	0	0	0	0	1									
B*07:101	07:101	HLA04924		0	0	1	0	0	0	0	1									
B*07:102	07:102	HLA04925		0	0	9	0	1	0	0	10			WD					WD	WD
B*07:103	07:103	HLA04929		6	0	0	0	0	0	0	6	WD							WD	WD
B*07:104	07:104	HLA04931		0	0	4	0	1	0	0	5								WD	WD
B*07:105	07:105	HLA04933		0	9	0	0	0	0	1	10		WD						WD	WD
B*07:106	07:106	HLA04935		0	0	3	10	0	0	0	13				WD				WD	WD
B*07:108	07:108	HLA04966		0	0	3	0	0	0	0	3									
B*07:109	07:109	HLA04981		0	0	22	0	0	0	0	22			WD					WD	WD
B*07:110	07:110	HLA04993		0	0	5	0	0	0	0	5			WD					WD	WD
B*07:111N	07:111N	HLA05000		0	0	0	0	0	0	1	1									
B*07:112	07:112	HLA04884		0	0	8	0	0	0	2	10			WD					WD	WD
B*07:113	07:113	HLA04886		0	0	6	0	0	0	2	8			WD					WD	WD
B*07:114	07:114	HLA05094		0	0	1	0	0	0	1	2									
B*07:117	07:117	HLA05531		0	0	0	1	0	0	0	1									
B*07:118	07:118	HLA06041		0	0	0	0	2	1	1	4									
B*07:122	07:122	HLA06224		0	0	7	0	0	0	0	7			WD					WD	WD
B*07:123	07:123	HLA06225		0	1	0	0	0	0	0	1									
B*07:125	07:125	HLA06317		0	0	1	0	0	0	0	1									
B*07:126	07:126	HLA06318		0	0	1	0	0	0	0	1									
B*07:127	07:127	HLA06319		0	0	4	0	0	0	0	4									
B*07:131	07:131	HLA06677		0	0	10	0	0	0	0	10			WD					WD	WD
B*07:132	07:132	HLA06690		0	0	6	0	0	0	0	6			WD					WD	WD

Supplemental Table 9: HLA-B Allele Summary [a]				Allele Count by Population Group [b]								3.0.0 CIWD Category by Population Group [c]								
**Allele**	**Genomic typing**	**Allele ID**	**G group**	**AFA**	**API**	**EURO**	**MENA**	**HIS**	**NAM**	**UNK**	**Total**	**AFA**	**API**	**EURO**	**MENA**	**HIS**	**NAM**	**UNK**	**Total**	**Highest Frequency**
B*07:133	07:133	HLA06691		0	0	0	0	0	0	1	1									
**B*07:136 total**	**07:136 total**			**0**	**1**	**19**	**0**	**1**	**0**	**0**	**21**			**WD**					**WD**	**WD**
B*07:136	07:136			0	0	9	0	0	0	0	9			WD					WD	WD
B*07:136:01	07:136:01	HLA06970		0	0	10	0	0	0	0	10			WD					WD	WD
B*07:136:02	07:136:02	HLA12570		0	1	0	0	1	0	0	2									
B*07:143	07:143	HLA07305		0	2	2	5	0	0	1	10				WD				WD	WD
B*07:145	07:145	HLA07465		0	0	0	0	0	0	1	1									
B*07:147	07:147	HLA07468		0	0	2	0	0	0	0	2									
B*07:148	07:148	HLA07469		0	0	1	0	0	0	0	1									
B*07:149	07:149	HLA07659		0	0	2	0	0	0	0	2									
**B*07:151 total**	**07:151 total**			**0**	**0**	**9**	**1**	**0**	**0**	**0**	**10**			**WD**					**WD**	**WD**
B*07:151	07:151			0	0	4	1	0	0	0	5								WD	WD
B*07:151:01	07:151:01	HLA07662		0	0	5	0	0	0	0	5			WD					WD	WD
B*07:153	07:153	HLA07723		0	0	2	0	0	0	0	2									
B*07:154	07:154	HLA07724		0	0	1	0	0	0	0	1									
B*07:157	07:157	HLA08134		0	0	3	0	0	0	0	3									
B*07:158	07:158	HLA08135		0	0	3	0	2	0	0	5								WD	WD
B*07:160	07:160	HLA08138		0	0	3	0	0	0	1	4									
B*07:163	07:163	HLA08380		0	0	0	2	0	0	0	2									
B*07:165	07:165	HLA08382		0	0	6	0	0	0	0	6			WD					WD	WD
B*07:167N	07:167N	HLA08384		0	0	2	0	0	0	0	2									
B*07:170	07:170	HLA08470		0	0	1	0	0	0	0	1									
B*07:171	07:171	HLA08520		0	1	1	0	0	0	0	2									
B*07:172	07:172	HLA08521		0	0	2	0	0	0	0	2									
B*07:174	07:174	HLA08523		0	0	3	0	0	0	0	3									
B*07:175	07:175	HLA08567		0	0	3	0	0	0	0	3									
B*07:176	07:176	HLA08684		0	0	0	1	0	0	2	3									
B*07:177	07:177	HLA08934		0	0	1	0	0	0	0	1									
B*07:179	07:179	HLA09007		0	0	1	0	0	0	1	2									
B*07:181N	07:181N	HLA09009		0	0	5	0	0	0	1	6			WD					WD	WD
B*07:182N	07:182N	HLA09116		0	1	0	0	0	0	0	1									

Supplemental Table 9: HLA-B Allele Summary [a]				Allele Count by Population Group [b]								3.0.0 CIWD Category by Population Group [c]								
Allele	Genomic typing	Allele ID	G group	AFA	API	EURO	MENA	HIS	NAM	UNK	Total	AFA	API	EURO	MENA	HIS	NAM	UNK	Total	Highest Frequency
B*07:187	07:187	HLA09443		0	0	1	0	0	0	0	1									
B*07:188	07:188	HLA09444		0	0	5	0	0	0	0	5			WD					WD	WD
B*07:189	07:189	HLA09445		0	0	1	0	0	0	0	1									
B*07:193	07:193	HLA09493		0	0	7	0	0	0	0	7			WD					WD	WD
B*07:195	07:195	HLA09589		0	0	1	0	0	0	0	1									
B*07:197	07:197	HLA09591		0	0	1	0	0	0	0	1									
B*07:205	07:205	HLA10420		1	0	0	0	3	0	0	4									
B*07:206	07:206	HLA10423		0	0	7	0	0	0	0	7			WD					WD	WD
B*07:208	07:208	HLA10519		0	0	9	0	0	0	0	9			WD					WD	WD
B*07:210	07:210	HLA10859		0	0	0	1	0	0	1	2									
B*07:211	07:211	HLA11036		0	3	0	0	0	0	0	3									
B*07:213	07:213	HLA11223		0	1	0	0	0	0	0	1									
B*07:216	07:216	HLA11435		3	0	0	0	0	0	0	3									
B*07:217	07:217	HLA11446		0	0	2	0	0	0	0	2									
B*07:218	07:218	HLA11448		0	0	1	0	0	0	0	1									
B*07:220	07:220	HLA11719		0	0	1	0	0	0	0	1									
B*07:221	07:221	HLA11735		0	0	2	0	0	0	0	2									
B*07:222	07:222	HLA11837		0	10	0	0	0	0	0	10		WD						WD	WD
B*07:226	07:226	HLA12171		0	0	2	0	0	0	0	2									
B*07:231N	07:231N	HLA12312		0	0	2	0	0	0	0	2									
B*07:235	07:235	HLA12586		0	0	1	0	0	0	0	1									
B*07:237	07:237	HLA12686		0	0	4	0	0	0	1	5								WD	WD
B*07:238	07:238	HLA12759		0	2	0	0	0	0	0	2									
B*07:239	07:239	HLA12760		0	0	2	0	0	0	0	2									
B*07:243	07:243	HLA13016		0	0	1	0	0	0	1	2									
B*07:245	07:245	HLA13019		0	0	2	0	0	0	0	2									
B*07:246	07:246	HLA13020		0	0	1	0	0	0	0	1									
B*07:247	07:247	HLA13022		0	1	1	0	0	0	0	2									
B*07:255	07:255	HLA13364		0	0	1	0	0	0	0	1									
B*07:258	07:258	HLA14237		0	2	0	0	0	0	0	2									
B*07:261	07:261	HLA14402		0	0	4	0	0	0	0	4									

Supplemental Table 9: HLA-B Allele Summary [a]				Allele Count by Population Group [b]								3.0.0 CIWD Category by Population Group [c]								
Allele	Genomic typing	Allele ID	G group	AFA	API	EURO	MENA	HIS	NAM	UNK	Total	AFA	API	EURO	MENA	HIS	NAM	UNK	Total	Highest Frequency
B*07:262	07:262	HLA14448		0	4	0	0	0	0	0	4									
B*07:264	07:264	HLA14455		0	0	2	0	0	0	0	2									
B*07:270	07:270	HLA15035		0	3	0	0	0	0	0	3									
**B*07:276 total**	**07:276 total**			**0**	**1**	**1**	**0**	**0**	**0**	**1**	**3**									
B*07:276:01	07:276:01	HLA15468		0	1	0	0	0	0	1	2									
B*07:276:02	07:276:02	HLA16224		0	0	1	0	0	0	0	1									
B*07:281	07:281	HLA15740		0	0	1	0	0	0	0	1									
B*07:287	07:287	HLA16056		0	1	0	0	0	0	0	1									
B*07:CODE [d]	07:CODE			1848	759	71557	530	3751	257	6511	85213	NA	NA	NA	NA	NA	NA	NA	NA	NA
**B*08:01 total**	**08:01 total**			**13085**	**32959**	**1173858**	**17335**	**28475**	**3088**	**95155**	**1363955**	**C**	**C**	**C**	**C**	**C**	**C**	**C**	**C**	**C**
B*08:01	08:01			598	423	13098	91	1655	129	2696	18690	C	C	C	C	C	C	C	C	C
B*08:01P	08:01P			0	9	796	6	2	0	6	819		WD	I	WD			WD	I	I
**B*08:01:01G total**	**08:01:01G total**			**12485**	**32521**	**1157664**	**17233**	**26809**	**2959**	**92402**	**1342073**	**C**	**C**	**C**	**C**	**C**	**C**	**C**	**C**	**C**
B*08:01:01G	08:01:01G		08:01:01G	10305	30857	1104332	16863	20303	2367	85907	1270934	C	C	C	C	C	C	C	C	C
B*08:01:01	08:01:01		08:01:01G	1786	1364	42183	321	5295	482	5259	56690	C	C	C	C	C	C	C	C	C
B*08:01:01:01	08:01:01:01	HLA00146	08:01:01G	387	27	10930	26	1174	105	1204	13853	C	I	C	WD	C	C	C	C	C
B*08:01:01:02	08:01:01:02	HLA14850	08:01:01G	7	273	150	22	34	4	23	513	WD	C	I	WD	I		I	I	C
B*08:01:01:05	08:01:01:05	HLA16876	08:01:01G	0	0	1	0	0	0	0	1									
B*08:01:14	08:01:14	HLA06132	08:01:01G	0	0	1	0	0	1	1	3									
B*08:01:20	08:01:20	HLA08776	08:01:01G	0	0	67	1	3	0	8	79			WD				WD	WD	WD
B*08:01:02	08:01:02	HLA02219		1	3	1683	3	9	0	42	1741			C		I		I	C	C
B*08:01:03	08:01:03	HLA02400		0	0	18	0	0	0	1	19			WD					WD	WD
B*08:01:04	08:01:04	HLA02853		1	1	451	0	0	0	3	456			I					I	I
B*08:01:05	08:01:05	HLA03134		0	0	7	0	0	0	0	7			WD					WD	WD
B*08:01:06	08:01:06	HLA03449		0	0	32	0	0	0	2	34			WD					WD	WD
B*08:01:07	08:01:07	HLA03939		0	0	9	0	0	0	0	9			WD					WD	WD
B*08:01:08	08:01:08	HLA04210		0	0	60	0	0	0	0	60			WD					WD	WD
B*08:01:10	08:01:10	HLA04504		0	0	2	0	0	0	0	2									
B*08:01:11	08:01:11	HLA05127		0	0	2	1	0	0	0	3									
B*08:01:12	08:01:12	HLA05362		0	0	2	0	0	0	0	2									
B*08:01:13	08:01:13	HLA05482		0	0	8	0	0	0	0	8			WD					WD	WD

Supplemental Table 9: HLA-B Allele Summary [a]				Allele Count by Population Group [b]								3.0.0 CIWD Category by Population Group [c]								
**Allele**	**Genomic typing**	**Allele ID**	**G group**	**AFA**	**API**	**EURO**	**MENA**	**HIS**	**NAM**	**UNK**	**Total**	**AFA**	**API**	**EURO**	**MENA**	**HIS**	**NAM**	**UNK**	**Total**	**Highest Frequency**
B*08:01:15	08:01:15	HLA06215		0	0	2	0	0	0	0	2									
B*08:01:16	08:01:16	HLA06917		0	0	0	1	0	0	0	1									
B*08:01:17	08:01:17	HLA07637		0	0	7	0	0	0	1	8			WD					WD	WD
B*08:01:18	08:01:18	HLA08341		0	0	3	0	0	0	0	3									
B*08:01:19	08:01:19	HLA08665		0	0	0	0	0	0	1	1									
B*08:01:21	08:01:21	HLA09558		0	0	3	0	0	0	0	3									
B*08:01:22	08:01:22	HLA09893		0	0	3	0	0	0	0	3									
B*08:01:23	08:01:23	HLA10404		0	2	0	0	0	0	0	2									
B*08:01:24	08:01:24	HLA11009		0	0	0	0	0	0	1	1									
B*08:01:25	08:01:25	HLA11232		0	0	2	0	0	0	0	2									
B*08:01:29	08:01:29	HLA11724		0	0	3	0	0	0	0	3									
B*08:01:30	08:01:30	HLA11822		0	0	2	0	0	0	0	2									
B*08:01:32	08:01:32	HLA14395		0	0	1	0	0	0	0	1									
B*08:02	08:02	HLA00147		0	0	14	0	0	0	1	15			WD					WD	WD
B*08:03	08:03	HLA00148		0	1	4	4	1	0	2	12								WD	WD
**B*08:04 total**	**08:04 total**			**0**	**0**	**39**	**1**	**0**	**0**	**35**	**75**			**WD**				**I**	**WD**	**I**
B*08:04	08:04			0	0	38	1	0	0	27	66			WD				I	WD	I
B*08:04:01	08:04:01	HLA00149		0	0	1	0	0	0	8	9							WD	WD	WD
B*08:07	08:07	HLA00974		1	0	19	0	0	0	1	21			WD					WD	WD
B*08:09	08:09	HLA00976		9	3	725	1	10	0	74	822	WD		I		I		I	I	I
B*08:11	08:11	HLA01193		0	0	7	0	2	0	0	9			WD					WD	WD
**B*08:12 total**	**08:12 total**			**93**	**0**	**20**	**1**	**0**	**0**	**12**	**126**	**C**		**WD**				**WD**	**WD**	**C**
B*08:12	08:12			7	0	0	0	0	0	0	7	WD							WD	WD
B*08:12:01	08:12:01	HLA01230		86	0	17	1	0	0	12	116	C		WD				WD	WD	C
B*08:12:03	08:12:03	HLA04186		0	0	3	0	0	0	0	3									
B*08:13	08:13	HLA01352		1	0	32	0	1	0	6	40			WD				WD	WD	WD
B*08:14	08:14	HLA01418		0	0	13	0	0	0	0	13			WD					WD	WD
B*08:15	08:15	HLA01535		0	0	14	0	0	0	1	15			WD					WD	WD
B*08:16	08:16	HLA01641		6	0	0	0	0	0	1	7	WD							WD	WD
B*08:17	08:17	HLA01654		0	0	1	0	0	0	0	1									
B*08:18	08:18	HLA01701		9	1	419	0	13	2	20	464	WD		I		I		I	I	I

Supplemental Table 9: HLA-B Allele Summary [a]				Allele Count by Population Group [b]								3.0.0 CIWD Category by Population Group [c]								
**Allele**	**Genomic typing**	**Allele ID**	**G group**	**AFA**	**API**	**EURO**	**MENA**	**HIS**	**NAM**	**UNK**	**Total**	**AFA**	**API**	**EURO**	**MENA**	**HIS**	**NAM**	**UNK**	**Total**	**Highest Frequency**
B*08:20	08:20	HLA01752		2	0	35	0	1	0	4	42			WD					WD	WD
B*08:21	08:21	HLA01772		0	0	4	0	0	0	0	4									
B*08:22	08:22	HLA01917		0	0	5	0	1	0	1	7			WD					WD	WD
B*08:23	08:23	HLA02132		11	0	38	0	0	0	4	53	WD		WD					WD	WD
B*08:24	08:24	HLA02142		0	0	132	0	0	0	0	132			I					WD	I
B*08:25	08:25	HLA02252		0	0	42	0	0	0	0	42			WD					WD	WD
**B*08:26 total**	**08:26 total**			**0**	**0**	**3**	**0**	**0**	**0**	**11**	**14**							**WD**	**WD**	**WD**
B*08:26	08:26			0	0	1	0	0	0	0	1									
B*08:26:01	08:26:01	HLA02308		0	0	2	0	0	0	11	13							WD	WD	WD
B*08:27	08:27	HLA02406		0	0	1	0	0	0	0	1									
B*08:28	08:28	HLA02490		0	0	20	0	0	0	3	23			WD					WD	WD
B*08:30N	08:30N	HLA02591		0	0	1	0	0	0	1	2									
B*08:31	08:31	HLA02762		0	0	6	0	0	0	0	6			WD					WD	WD
B*08:33	08:33	HLA02849		0	0	41	0	15	0	23	79			WD		I		I	WD	I
B*08:34	08:34	HLA03091		0	0	9	0	0	0	1	10			WD					WD	WD
B*08:35	08:35	HLA03164		0	0	35	0	1	0	3	39			WD					WD	WD
B*08:36	08:36	HLA03265		2	0	0	0	4	0	0	6								WD	WD
B*08:37	08:37	HLA03450		0	0	40	0	1	0	0	41			WD					WD	WD
B*08:39	08:39	HLA03663		1	0	15	0	0	0	1	17			WD					WD	WD
B*08:41	08:41	HLA03902		0	0	19	0	0	0	0	19			WD					WD	WD
B*08:43	08:43	HLA04081		1	2	0	1	0	0	0	4									
B*08:47	08:47	HLA04240		0	0	2	0	0	0	1	3									
B*08:48	08:48	HLA04438		0	0	2	0	0	0	0	2									
B*08:50	08:50	HLA04499		0	0	4	0	1	0	0	5								WD	WD
B*08:51	08:51	HLA04507		0	1	2	0	0	0	0	3									
B*08:52	08:52	HLA04515		0	0	6	0	0	0	0	6			WD					WD	WD
**B*08:53 total**	**08:53 total**			**0**	**0**	**1**	**0**	**0**	**0**	**0**	**1**									
B*08:53	08:53			0	0	1	0	0	0	0	1									
**B*08:56 total**	**08:56 total**			**0**	**0**	**3**	**0**	**0**	**0**	**2**	**5**								**WD**	**WD**
B*08:56	08:56			0	0	1	0	0	0	0	1									
B*08:56:01	08:56:01	HLA04720		0	0	0	0	0	0	2	2									

Supplemental Table 9: HLA-B Allele Summary [a]				Allele Count by Population Group [b]								3.0.0 CIWD Category by Population Group [c]								
Allele	Genomic typing	Allele ID	G group	AFA	API	EURO	MENA	HIS	NAM	UNK	Total	AFA	API	EURO	MENA	HIS	NAM	UNK	Total	Highest Frequency
B*08:56:02	08:56:02	HLA08338		0	0	2	0	0	0	0	2									
B*08:58	08:58	HLA04753		0	0	2	0	0	0	0	2									
**B*08:59 total**	**08:59 total**			**0**	**19**	**3**	**0**	**0**	**0**	**0**	**22**		**I**						**WD**	**I**
B*08:59	08:59			0	1	0	0	0	0	0	1									
B*08:59:01	08:59:01	HLA04788		0	18	1	0	0	0	0	19		I						WD	I
B*08:59:02	08:59:02	HLA08983		0	0	2	0	0	0	0	2									
B*08:61	08:61	HLA04860		0	0	2	0	0	0	0	2									
B*08:62	08:62	HLA05290		0	0	2	1	0	0	0	3									
B*08:63	08:63	HLA05506		0	0	11	0	0	0	0	11			WD					WD	WD
B*08:64	08:64	HLA05510		0	0	1	0	0	0	0	1									
B*08:65	08:65	HLA05720		0	0	0	0	0	0	1	1									
B*08:68	08:68	HLA06259		0	0	0	0	1	0	0	1									
B*08:69	08:69	HLA06260		0	0	2	0	0	0	1	3									
B*08:71	08:71	HLA06682		0	1	25	0	0	0	0	26			WD					WD	WD
B*08:72N	08:72N	HLA06918		0	0	1	0	0	0	0	1									
B*08:75	08:75	HLA06956		0	0	5	0	0	0	0	5			WD					WD	WD
B*08:77	08:77	HLA06996		0	3	1	0	0	0	0	4									
B*08:78	08:78	HLA07191		0	0	5	0	1	0	5	11			WD				WD	WD	WD
B*08:81	08:81	HLA07442		0	0	1	0	0	0	0	1									
B*08:86N	08:86N	HLA07635		0	0	1	0	0	0	0	1									
B*08:87	08:87	HLA07636		0	0	2	0	0	0	0	2									
B*08:88	08:88	HLA07638		0	0	5	0	0	0	0	5			WD					WD	WD
B*08:89	08:89	HLA08041		0	0	2	0	0	0	0	2									
B*08:91	08:91	HLA07912		0	0	1	0	1	0	0	2									
B*08:92	08:92	HLA07913		0	0	3	0	0	0	0	3									
B*08:96	08:96	HLA08340		0	0	0	0	0	0	2	2									
B*08:97	08:97	HLA08343		0	0	13	0	0	0	0	13			WD					WD	WD
B*08:98	08:98	HLA08508		0	0	3	0	0	0	0	3									
B*08:102	08:102	HLA08666		0	0	0	0	0	0	1	1									
B*08:103	08:103	HLA08668		0	0	1	0	0	0	0	1									
B*08:104	08:104	HLA08669		0	0	8	0	0	0	0	8			WD					WD	WD

Supplemental Table 9: HLA-B Allele Summary [a]				Allele Count by Population Group [b]								3.0.0 CIWD Category by Population Group [c]								
Allele	Genomic typing	Allele ID	G group	AFA	API	EURO	MENA	HIS	NAM	UNK	Total	AFA	API	EURO	MENA	HIS	NAM	UNK	Total	Highest Frequency
B*08:108	08:108	HLA09450		0	0	2	0	0	0	0	2									
B*08:110	08:110	HLA09557		0	0	8	0	0	0	0	8			WD					WD	WD
B*08:111	08:111	HLA09874		0	0	2	0	0	0	0	2									
B*08:112	08:112	HLA09892		0	0	1	0	0	0	0	1									
B*08:113	08:113	HLA10192		0	0	2	0	0	0	0	2									
B*08:117	08:117	HLA11010		0	0	1	0	0	0	0	1									
B*08:118	08:118	HLA11011		0	0	0	0	0	0	1	1									
B*08:119	08:119	HLA11012		0	0	0	2	0	0	0	2									
B*08:122	08:122	HLA11476		0	0	1	0	0	0	0	1									
B*08:124	08:124	HLA11652		0	0	1	0	0	0	0	1									
B*08:129	08:129	HLA11823		0	0	3	0	0	0	0	3									
B*08:132	08:132	HLA11937		0	1	3	0	0	0	0	4									
B*08:135	08:135	HLA12268		0	0	2	0	0	0	0	2									
B*08:136	08:136	HLA12269		0	0	2	0	0	0	0	2									
B*08:137	08:137	HLA12475		0	0	3	0	0	0	0	3									
B*08:138	08:138	HLA12607		0	1	1	0	0	0	0	2									
B*08:145	08:145	HLA13343		0	0	0	0	1	0	0	1									
B*08:149	08:149	HLA13469		0	0	1	0	0	0	0	1									
B*08:150	08:150	HLA13470		0	0	2	0	0	0	0	2									
B*08:162	08:162	HLA14926		0	0	2	0	0	0	0	2									
B*08:165	08:165	HLA15164		0	0	1	0	0	0	1	2									
B*08:CODE	08:CODE			448	346	56805	356	2237	186	4406	64784	NA	NA	NA	NA	NA	NA	NA	NA	NA
**B*13:01 total**	**13:01 total**			**58**	**30245**	**2316**	**477**	**95**	**38**	**1987**	**35216**	**C**	**C**	**C**	**C**	**C**	**C**	**C**	**C**	**C**
B*13:01	13:01			6	1242	118	25	11	4	264	1670	WD	C	WD	WD	I		C	C	C
B*13:01P	13:01P			0	0	5	0	0	0	0	5			WD					WD	WD
**B*13:01:01G total**	**13:01:01G total**			**52**	**28993**	**2191**	**452**	**84**	**34**	**1722**	**33528**	**C**	**C**	**C**	**C**	**C**	**C**	**C**	**C**	**C**
B*13:01:01G	13:01:01G		13:01:01G	47	25904	2096	433	65	24	1382	29951	C	C	C	C	I	C	C	C	C
B*13:01:01	13:01:01		13:01:01G	5	3089	95	19	19	10	340	3577	WD	C	WD	WD	I	C	C	C	C
B*13:01:02	13:01:02	HLA03770		0	2	2	0	0	0	0	4									
B*13:01:10	13:01:10	HLA13026		0	8	0	0	0	0	1	9		WD						WD	WD
**B*13:02 total**	**13:02 total**			**3803**	**20350**	**390325**	**12629**	**8855**	**722**	**30541**	**467225**	**C**	**C**	**C**	**C**	**C**	**C**	**C**	**C**	**C**

Supplemental Table 9: HLA-B Allele Summary [a]				Allele Count by Population Group [b]								3.0.0 CIWD Category by Population Group [c]								
Allele	Genomic typing	Allele ID	G group	AFA	API	EURO	MENA	HIS	NAM	UNK	Total	AFA	API	EURO	MENA	HIS	NAM	UNK	Total	Highest Frequency
B*13:02	13:02			29	58	9335	205	10	1	530	10168	WD	I	C	C	I		C	C	C
B*13:02P	13:02P			3	0	482	0	2	0	3	490			I					I	I
**B*13:02:01G total**	**13:02:01G total**			**3771**	**20289**	**380442**	**12424**	**8842**	**720**	**30004**	**456492**	**C**	**C**	**C**	**C**	**C**	**C**	**C**	**C**	**C**
B*13:02:01G	13:02:01G		13:02:01G	3076	17824	366446	12055	6274	533	27712	433920	C	C	C	C	C	C	C	C	C
B*13:02:01	13:02:01		13:02:01G	665	2401	13238	334	2461	178	2205	21482	C	C	C	C	C	C	C	C	C
B*13:02:01:01	13:02:01:01	HLA00153	13:02:01G	30	63	757	35	107	9	86	1087	WD	I	I	WD	C	C	I	I	C
B*13:02:01:03	13:02:01:03	HLA16858	13:02:01G	0	0	0	0	0	0	1	1									
B*13:38	13:38	HLA05118	13:02:01G	0	1	1	0	0	0	0	2									
B*13:02:02	13:02:02	HLA02174		0	0	24	0	1	1	1	27			WD					WD	WD
B*13:02:03	13:02:03	HLA02295		0	0	3	0	0	0	1	4									
B*13:02:04	13:02:04	HLA02809		0	0	19	0	0	0	2	21			WD					WD	WD
B*13:02:06	13:02:06	HLA04083		0	1	2	0	0	0	0	3									
B*13:02:07	13:02:07	HLA04849		0	0	2	0	0	0	0	2									
B*13:02:08	13:02:08	HLA05256		0	0	1	0	0	0	0	1									
B*13:02:10	13:02:10	HLA06235		0	0	1	0	0	0	0	1									
B*13:02:11	13:02:11	HLA06699		0	0	7	0	0	0	0	7			WD					WD	WD
B*13:02:13	13:02:13	HLA07917		0	2	0	0	0	0	0	2									
B*13:02:14	13:02:14	HLA08346		0	0	2	0	0	0	0	2									
B*13:02:15	13:02:15	HLA08347		0	0	3	0	0	0	0	3									
B*13:02:17	13:02:17	HLA12700		0	0	1	0	0	0	0	1									
B*13:02:19	13:02:19	HLA16247		0	0	1	0	0	0	0	1									
B*13:03	13:03	HLA00154		23	4	5	8	0	0	0	40	WD		WD	WD				WD	WD
B*13:04	13:04	HLA00155		0	0	4	0	0	0	1	5								WD	WD
B*13:06	13:06	HLA01276		0	4	0	0	0	0	1	5								WD	WD
B*13:08	13:08	HLA01476		0	0	44	0	0	0	1	45			WD					WD	WD
B*13:10	13:10	HLA01591		0	0	61	0	0	0	0	61			WD					WD	WD
B*13:11	13:11	HLA01632		0	0	8	2	1	0	1	12			WD					WD	WD
**B*13:12 total**	**13:12 total**			**0**	**15**	**0**	**0**	**0**	**0**	**8**	**23**		**I**					**WD**	**WD**	**I**
B*13:12	13:12			0	5	0	0	0	0	2	7		WD						WD	WD
B*13:12:01	13:12:01	HLA01840		0	10	0	0	0	0	5	15		WD					WD	WD	WD
B*13:12:02	13:12:02	HLA08902		0	0	0	0	0	0	1	1									

Supplemental Table 9: HLA-B Allele Summary [a]				Allele Count by Population Group [b]								3.0.0 CIWD Category by Population Group [c]								
**Allele**	**Genomic typing**	**Allele ID**	**G group**	**AFA**	**API**	**EURO**	**MENA**	**HIS**	**NAM**	**UNK**	**Total**	**AFA**	**API**	**EURO**	**MENA**	**HIS**	**NAM**	**UNK**	**Total**	**Highest Frequency**
**B*13:13 total**	**13:13 total**			**0**	**1**	**0**	**0**	**0**	**0**	**0**	**1**									
B*13:13:01	13:13:01	HLA01858		0	1	0	0	0	0	0	1									
B*13:14	13:14	HLA02360		0	0	7	0	0	0	0	7			WD					WD	WD
B*13:15	13:15	HLA02389		0	0	2	0	0	0	0	2									
B*13:16	13:16	HLA02516		0	0	4	0	0	0	0	4									
**B*13:22 total**	**13:22 total**			**0**	**1**	**0**	**0**	**0**	**0**	**0**	**1**									
B*13:22:01	13:22:01	HLA03315		0	1	0	0	0	0	0	1									
B*13:31	13:31	HLA04436		0	0	1	0	0	0	0	1									
B*13:33	13:33	HLA04502		2	0	0	0	0	0	0	2									
B*13:42	13:42	HLA06056		0	1	0	0	0	0	0	1									
B*13:45	13:45	HLA06263		0	0	9	0	1	0	0	10			WD					WD	WD
B*13:46	13:46	HLA06264		0	1	0	0	0	0	0	1									
B*13:47	13:47	HLA06933		0	0	5	0	0	0	0	5			WD					WD	WD
B*13:50	13:50	HLA07033		1	0	0	0	0	0	0	1									
B*13:51	13:51	HLA07094		0	0	0	0	0	0	2	2									
B*13:53	13:53	HLA07693		0	0	2	0	0	0	0	2									
B*13:55	13:55	HLA07892		0	0	1	0	0	0	0	1									
**B*13:56N total**	**13:56N total**			**0**	**0**	**1**	**0**	**0**	**0**	**0**	**1**									
B*13:56:01N	13:56:01N	HLA07915		0	0	1	0	0	0	0	1									
B*13:57	13:57	HLA07916		0	11	0	0	0	0	2	13		WD						WD	WD
B*13:59	13:59	HLA08345		0	0	1	0	0	0	0	1									
B*13:66	13:66	HLA09442		0	0	1	0	1	0	0	2									
B*13:70	13:70	HLA09645		0	0	0	0	1	0	0	1									
B*13:74	13:74	HLA11013		1	0	0	0	0	0	0	1									
B*13:77	13:77	HLA11235		0	1	0	0	0	0	0	1									
B*13:78	13:78	HLA11424		0	1	0	0	0	0	0	1									
B*13:79	13:79	HLA11655		0	0	1	0	0	0	0	1									
B*13:81	13:81	HLA12134		0	0	1	0	0	0	0	1									
B*13:84	13:84	HLA13650		0	0	1	0	0	0	0	1									
B*13:86	13:86	HLA14300		0	0	2	1	0	0	0	3									
B*13:98	13:98	HLA16050		0	0	1	0	0	0	0	1									

Supplemental Table 9: HLA-B Allele Summary [a]				Allele Count by Population Group [b]								3.0.0 CIWD Category by Population Group [c]								
Allele	Genomic typing	Allele ID	G group	AFA	API	EURO	MENA	HIS	NAM	UNK	Total	AFA	API	EURO	MENA	HIS	NAM	UNK	Total	Highest Frequency
B*13:CODE	13:CODE			127	1458	16892	325	656	49	1640	21147	NA	NA	NA	NA	NA	NA	NA	NA	NA
**B*14:01 total**	**14:01 total**			**2900**	**779**	**73385**	**1109**	**5632**	**408**	**8970**	**93183**	**C**	**C**	**C**	**C**	**C**	**C**	**C**	**C**	**C**
B*14:01	14:01			181	40	5116	86	662	38	510	6633	C	I	C	C	C	C	C	C	C
B*14:01P	14:01P			0	0	15	0	1	0	0	16			WD					WD	WD
**B*14:01:01G total**	**14:01:01G total**			**2719**	**739**	**68253**	**1023**	**4969**	**370**	**8459**	**86532**	**C**	**C**	**C**	**C**	**C**	**C**	**C**	**C**	**C**
B*14:01:01G	14:01:01G		14:01:01G	17	41	3236	40	51	2	259	3646	WD	I	C	WD	I		C	C	C
B*14:01:01	14:01:01		14:01:01G	2677	694	64696	982	4844	363	8149	82405	C	C	C	C	C	C	C	C	C
B*14:01:01:01	14:01:01:01	HLA00157	14:01:01G	25	4	320	1	74	5	51	480	WD		I		C	WD	I	I	C
B*14:01:01:02	14:01:01:02	HLA17048	14:01:01G	0	0	1	0	0	0	0	1									
B*14:01:03	14:01:03	HLA09174		0	0	1	0	0	0	0	1									
B*14:01:04	14:01:04	HLA10045		0	0	0	0	0	0	1	1									
**B*14:02 total**	**14:02 total**			**8633**	**2258**	**239888**	**9706**	**27191**	**2022**	**39519**	**329217**	**C**	**C**	**C**	**C**	**C**	**C**	**C**	**C**	**C**
B*14:02	14:02			508	80	15352	564	3020	137	3124	22785	C	I	C	C	C	C	C	C	C
B*14:02P	14:02P			0	0	88	0	0	0	1	89			WD					WD	WD
**B*14:02:01G total**	**14:02:01G total**			**8115**	**2172**	**224383**	**9129**	**23920**	**1866**	**36252**	**305837**	**C**	**C**	**C**	**C**	**C**	**C**	**C**	**C**	**C**
B*14:02:01G	14:02:01G		14:02:01G	786	1097	68400	1862	2711	56	6222	81134	C	C	C	C	C	C	C	C	C
B*14:02:01	14:02:01		14:02:01G	6438	988	147268	7068	17504	1515	28667	209448	C	C	C	C	C	C	C	C	C
B*14:02:01:01	14:02:01:01	HLA00158	14:02:01G	658	86	8547	199	3606	289	1280	14665	C	I	C	C	C	C	C	C	C
B*14:02:01:02	14:02:01:02	HLA13766	14:02:01G	4	0	159	0	8	0	19	190			I		I		I	I	I
B*14:02:01:03	14:02:01:03	HLA14822	14:02:01G	229	1	9	0	91	6	64	400	C		WD		C	WD	I	I	C
B*14:02:02	14:02:02	HLA02150		9	6	58	11	251	19	139	493	WD	WD	WD	WD	C	C	C	I	C
B*14:02:03	14:02:03	HLA04101		0	0	7	0	0	0	0	7			WD					WD	WD
B*14:02:04	14:02:04	HLA04496		0	0	0	1	0	0	2	3									
B*14:02:05	14:02:05	HLA04415		1	0	0	0	0	0	0	1									
B*14:02:13	14:02:13	HLA13012		0	0	0	0	0	0	1	1									
B*14:02:15	14:02:15	HLA16502		0	0	0	1	0	0	0	1									
B*14:03	14:03	HLA00159		929	6	156	71	153	25	206	1546	C	WD	I	C	C	C	C	I	C
B*14:05	14:05	HLA00161		87	0	9	0	4	3	16	119	C		WD				I	WD	C
**B*14:06 total**	**14:06 total**			**0**	**0**	**85**	**1**	**4**	**2**	**10**	**102**			**WD**				**WD**	**WD**	**WD**
B*14:06	14:06			0	0	15	1	0	0	0	16			WD					WD	WD
B*14:06:01	14:06:01	HLA00977		0	0	33	0	0	0	1	34			WD					WD	WD

Supplemental Table 9: HLA-B Allele Summary [a]				Allele Count by Population Group [b]								3.0.0 CIWD Category by Population Group [c]								
Allele	Genomic typing	Allele ID	G group	AFA	API	EURO	MENA	HIS	NAM	UNK	Total	AFA	API	EURO	MENA	HIS	NAM	UNK	Total	Highest Frequency
B*14:06:02	14:06:02	HLA01052		0	0	37	0	4	2	9	52			WD				WD	WD	WD
B*14:07N	14:07N	HLA02166		0	0	15	0	1	0	0	16			WD					WD	WD
**B*14:08 total**	**14:08 total**			**0**	**0**	**7**	**0**	**1**	**0**	**0**	**8**			**WD**					**WD**	**WD**
B*14:08	14:08			0	0	1	0	0	0	0	1									
B*14:08:01	14:08:01	HLA03283		0	0	6	0	1	0	0	7			WD					WD	WD
B*14:09	14:09	HLA03318		8	0	0	0	0	0	1	9	WD							WD	WD
B*14:10	14:10	HLA03639		0	0	37	0	0	0	0	37			WD					WD	WD
B*14:12	14:12	HLA04027		1	0	0	0	0	0	1	2									
B*14:13	14:13	HLA04055		0	0	3	0	0	0	0	3									
B*14:14	14:14	HLA04066		0	0	3	0	0	0	0	3									
B*14:17	14:17	HLA04532		0	0	1	0	0	0	0	1									
B*14:18	14:18	HLA04565		0	0	3	0	0	0	0	3									
B*14:19	14:19	HLA05474		0	0	5	0	0	0	0	5			WD					WD	WD
B*14:20	14:20	HLA05928		0	0	22	0	0	0	0	22			WD					WD	WD
B*14:22	14:22	HLA06324		0	0	1	0	0	0	0	1									
B*14:23	14:23	HLA06325		0	0	3	3	0	0	0	6								WD	WD
B*14:24	14:24	HLA06357		0	0	1	0	0	0	0	1									
B*14:26	14:26	HLA06990		0	0	2	0	0	0	0	2									
B*14:27	14:27	HLA07004		0	0	1	0	0	0	0	1									
B*14:29	14:29	HLA07463		0	0	16	0	0	0	0	16			WD					WD	WD
B*14:31	14:31	HLA07717		0	0	2	2	0	0	0	4									
B*14:32	14:32	HLA07725		0	0	6	0	0	0	0	6			WD					WD	WD
B*14:34	14:34	HLA09585		0	0	1	0	0	0	0	1									
B*14:35	14:35	HLA09586		0	0	0	0	0	0	1	1									
B*14:36	14:36	HLA09587		0	0	1	0	0	0	0	1									
B*14:38	14:38	HLA10417		0	0	0	0	0	0	1	1									
B*14:45	14:45	HLA13014		0	0	3	0	0	0	0	3									
B*14:47	14:47	HLA13544		0	0	0	0	0	0	1	1									
B*14:48	14:48	HLA13662		0	0	0	0	0	0	1	1									
B*14:53	14:53	HLA14490		0	0	1	0	0	0	0	1									
B*14:56	14:56	HLA16820		0	0	1	0	0	0	0	1									

Supplemental Table 9: HLA-B Allele Summary [a]				Allele Count by Population Group [b]								3.0.0 CIWD Category by Population Group [c]								
Allele	Genomic typing	Allele ID	G group	AFA	API	EURO	MENA	HIS	NAM	UNK	Total	AFA	API	EURO	MENA	HIS	NAM	UNK	Total	Highest Frequency
B*14:CODE	14:CODE			444	51	15573	185	1962	107	2776	21098	NA	NA	NA	NA	NA	NA	NA	NA	NA
**B*15:01 total**	**15:01 total**			**3503**	**26750**	**692696**	**4801**	**18544**	**1978**	**57632**	**805904**	**C**	**C**	**C**	**C**	**C**	**C**	**C**	**C**	**C**
B*15:01	15:01			0	10	2356	0	7	1	72	2446		WD	C		WD		I	C	C
B*15:01P	15:01P			0	1	393	0	0	0	2	396			I					I	I
**B*15:01:01G total**	**15:01:01G total**			**3503**	**26462**	**689661**	**4798**	**18530**	**1977**	**57529**	**802460**	**C**	**C**	**C**	**C**	**C**	**C**	**C**	**C**	**C**
B*15:01:01G	15:01:01G		15:01:01G	2738	22931	656657	4671	13051	1462	52011	753521	C	C	C	C	C	C	C	C	C
B*15:01:01	15:01:01		15:01:01G	153	813	4905	25	1096	83	1609	8684	C	C	C	WD	C	C	C	C	C
B*15:01:01:01	15:01:01:01	HLA00162	15:01:01G	567	2704	25694	93	4138	413	3661	37270	C	C	C	C	C	C	C	C	C
**B*15:01N total**	**15:01N total**			**0**	**0**	**7**	**0**	**1**	**0**	**0**	**8**			**WD**					**WD**	**WD**
B*15:01:01:02N	15:01:01:02N	HLA00163	15:01:01G	0	0	7	0	1	0	0	8			WD					WD	WD
B*15:01:01:03	15:01:01:03	HLA11953	15:01:01G	0	0	27	1	1	0	2	31			WD					WD	WD
B*15:01:01:04	15:01:01:04	HLA14080	15:01:01G	33	9	1757	5	171	14	177	2166	WD	WD	C	WD	C	C	C	C	C
B*15:01:01:05	15:01:01:05	HLA14851	15:01:01G	0	0	21	0	1	0	4	26			WD					WD	WD
B*15:01:01:06	15:01:01:06	HLA15405	15:01:01G	10	1	514	3	42	4	48	622	WD		I		I		I	I	I
B*15:01:01:07	15:01:01:07	HLA16170	15:01:01G	0	1	0	0	0	0	0	1									
B*15:01:01:08	15:01:01:08	HLA16171	15:01:01G	0	0	1	0	0	0	0	1									
B*15:01:01:09	15:01:01:09	HLA16172	15:01:01G	0	0	36	0	1	0	0	37			WD					WD	WD
B*15:01:01:11	15:01:01:11	HLA16678	15:01:01G	0	0	11	0	0	0	1	12			WD					WD	WD
B*15:01:01:12	15:01:01:12	HLA16853	15:01:01G	0	0	0	0	0	0	1	1									
B*15:01:06	15:01:06	HLA03005	15:01:01G	0	0	11	0	0	0	4	15			WD					WD	WD
B*15:01:07	15:01:07	HLA03184	15:01:01G	0	0	9	0	0	0	2	11			WD					WD	WD
B*15:102	15:102	HLA02184	15:01:01G	0	2	0	0	0	0	0	2									
B*15:146	15:146	HLA03287	15:01:01G	2	1	4	0	28	1	9	45					I		WD	WD	I
B*15:228	15:228	HLA06903	15:01:01G	0	0	7	0	0	0	0	7			WD					WD	WD
B*15:01:02	15:01:02	HLA00164		0	275	1	2	0	0	3	281		C						I	C
B*15:01:03	15:01:03	HLA00978		0	0	8	0	3	0	2	13			WD					WD	WD
B*15:01:04	15:01:04	HLA01373		0	0	67	0	0	0	1	68			WD					WD	WD
B*15:01:08	15:01:08	HLA03215		0	0	8	0	0	0	0	8			WD					WD	WD
B*15:01:09	15:01:09	HLA03964		0	0	1	0	0	0	0	1									
B*15:01:10	15:01:10	HLA04071		0	0	119	0	0	0	2	121			WD					WD	WD
B*15:01:11	15:01:11	HLA04112		0	0	0	0	0	0	1	1									

Supplemental Table 9: HLA-B Allele Summary [a]				Allele Count by Population Group [b]								3.0.0 CIWD Category by Population Group [c]								
Allele	Genomic typing	Allele ID	G group	AFA	API	EURO	MENA	HIS	NAM	UNK	Total	AFA	API	EURO	MENA	HIS	NAM	UNK	Total	Highest Frequency
B*15:01:12	15:01:12	HLA04216		0	0	11	0	0	0	0	11			WD					WD	WD
B*15:01:13	15:01:13	HLA04454		0	0	4	0	0	0	0	4									
B*15:01:14	15:01:14	HLA04463		0	1	19	1	0	0	1	22			WD					WD	WD
B*15:01:15	15:01:15	HLA04464		0	0	18	0	0	0	0	18			WD					WD	WD
B*15:01:16	15:01:16	HLA04553		0	0	21	0	0	0	0	21			WD					WD	WD
B*15:01:19	15:01:19	HLA05718		0	0	1	0	0	0	0	1									
B*15:01:21	15:01:21	HLA06133		0	0	8	0	0	0	0	8			WD					WD	WD
B*15:01:24	15:01:24	HLA06966		0	0	1	0	2	0	0	3									
B*15:01:25	15:01:25	HLA07457		0	0	1	0	0	0	1	2									
B*15:01:27	15:01:27	HLA07525		0	0	0	0	0	0	1	1									
B*15:01:31	15:01:31	HLA08678		0	0	2	0	0	0	13	15							WD	WD	WD
B*15:01:32	15:01:32	HLA09175		0	0	0	0	3	0	0	3									
B*15:01:33	15:01:33	HLA09575		0	0	1	0	0	0	0	1									
B*15:01:35	15:01:35	HLA11025		0	1	1	0	0	0	1	3									
B*15:01:36	15:01:36	HLA12016		0	0	1	0	0	0	3	4									
**B*15:02 total**	**15:02 total**			**117**	**38192**	**924**	**349**	**142**	**69**	**2288**	**42081**	**C**	**C**	**I**	**C**	**C**	**C**	**C**	**C**	**C**
B*15:02	15:02			3	767	10	3	1	0	17	801		C	WD				I	I	C
B*15:02P	15:02P			0	1	1	0	0	0	0	2									
**B*15:02:01G total**	**15:02:01G total**			**114**	**37420**	**913**	**346**	**141**	**69**	**2271**	**41274**	**C**	**C**	**I**	**C**	**C**	**C**	**C**	**C**	**C**
B*15:02:01G	15:02:01G		15:02:01G	96	31941	879	323	93	45	1609	34986	C	C	I	C	C	C	C	C	C
B*15:02:01	15:02:01	HLA00165	15:02:01G	18	5479	34	23	48	24	662	6288	WD	C	WD	WD	I	C	C	C	C
B*15:02:03	15:02:03	HLA04471		0	1	0	0	0	0	0	1									
B*15:02:04	15:02:04	HLA04473		0	3	0	0	0	0	0	3									
**B*15:03 total**	**15:03 total**			**22255**	**583**	**13315**	**2376**	**6963**	**811**	**8033**	**54336**	**C**	**C**	**C**	**C**	**C**	**C**	**C**	**C**	**C**
B*15:03	15:03			6	1	92	4	5	0	3	111	WD		WD		WD			WD	WD
B*15:03P	15:03P			1	0	37	0	1	0	2	41			WD					WD	WD
**B*15:03:01G total**	**15:03:01G total**			**22237**	**582**	**13160**	**2369**	**6956**	**811**	**8022**	**54137**	**C**	**C**	**C**	**C**	**C**	**C**	**C**	**C**	**C**
B*15:03:01G	15:03:01G		15:03:01G	18520	527	12457	2297	5348	614	7090	46853	C	C	C	C	C	C	C	C	C
B*15:03:01	15:03:01		15:03:01G	249	13	26	1	137	9	68	503	C	I	WD		C	C	I	I	C
B*15:03:01:01	15:03:01:01	HLA00166	15:03:01G	14	4	1	0	6	0	8	33	WD				WD		WD	WD	WD
B*15:03:01:02	15:03:01:02	HLA14102	15:03:01G	3305	28	395	28	1292	178	783	6009	C	I	I	WD	C	C	C	C	C

Supplemental Table 9: HLA-B Allele Summary [a]				Allele Count by Population Group [b]								3.0.0 CIWD Category by Population Group [c]								
Allele	Genomic typing	Allele ID	G group	AFA	API	EURO	MENA	HIS	NAM	UNK	Total	AFA	API	EURO	MENA	HIS	NAM	UNK	Total	Highest Frequency
B*15:103	15:103	HLA02236	15:03:01G	3	0	0	0	0	0	0	3									
B*15:220	15:220	HLA06273	15:03:01G	146	10	281	43	173	10	73	736	C	WD	I	C	C	C	I	I	C
B*15:03:02	15:03:02	HLA03954		1	0	0	0	1	0	0	2									
B*15:03:03	15:03:03	HLA03958		7	0	26	0	0	0	4	37	WD		WD					WD	WD
B*15:03:04	15:03:04	HLA08907		3	0	0	0	0	0	2	5								WD	WD
B*15:03:05	15:03:05	HLA12014		0	0	0	3	0	0	0	3									
**B*15:04 total**	**15:04 total**			**39**	**145**	**669**	**4**	**1508**	**96**	**992**	**3453**	**C**	**C**	**I**		**C**	**C**	**C**	**C**	**C**
B*15:04	15:04			16	8	258	2	483	48	262	1077	WD	WD	I		C	C	C	I	C
B*15:04P	15:04P			0	0	1	0	0	0	0	1									
**B*15:04:01G total**	**15:04:01G total**			**23**	**137**	**409**	**2**	**1025**	**48**	**730**	**2374**	**WD**	**C**	**I**		**C**	**C**	**C**	**C**	**C**
B*15:04:01G	15:04:01G		15:04:01G	2	99	349	0	532	1	606	1589		I	I		C		C	I	C
B*15:04:01	15:04:01		15:04:01G	13	35	47	2	358	29	88	572	WD	I	WD		C	C	I	I	C
B*15:04:01:01	15:04:01:01	HLA00167	15:04:01G	5	0	4	0	50	0	16	75	WD				I		I	WD	I
B*15:04:01:02	15:04:01:02	HLA14852	15:04:01G	3	3	7	0	81	18	20	132			WD		C	C	I	WD	C
B*15:04:04	15:04:04	HLA14857	15:04:01G	0	0	2	0	4	0	0	6								WD	WD
B*15:04:02	15:04:02	HLA11240		0	0	1	0	0	0	0	1									
**B*15:05 total**	**15:05 total**			**24**	**5634**	**114**	**27**	**142**	**24**	**154**	**6119**	**WD**	**C**	**WD**	**WD**	**C**	**C**	**C**	**C**	**C**
B*15:05	15:05			0	228	15	1	27	2	13	286		C	WD		I		WD	I	C
B*15:05P	15:05P			0	0	0	1	0	0	0	1									
B*15:05:01	15:05:01	HLA00168		24	5404	91	25	115	22	141	5822	WD	C	WD	WD	C	C	C	C	C
B*15:05:02	15:05:02	HLA04569		0	0	8	0	0	0	0	8			WD					WD	WD
B*15:05:03	15:05:03	HLA16932		0	2	0	0	0	0	0	2									
B*15:06	15:06	HLA00169		0	180	12	1	13	0	80	286		C	WD		I		I	I	C
**B*15:07 total**	**15:07 total**			**63**	**752**	**5758**	**9**	**338**	**138**	**831**	**7889**	**C**	**C**	**C**	**WD**	**C**	**C**	**C**	**C**	**C**
B*15:07	15:07			29	335	1565	5	110	64	310	2418	WD	C	C	WD	C	C	C	C	C
B*15:07P	15:07P			0	0	0	0	0	0	1	1									
**B*15:07:01G total**	**15:07:01G total**			**34**	**417**	**4193**	**4**	**228**	**74**	**519**	**5469**	**WD**	**C**	**C**		**C**	**C**	**C**	**C**	**C**
B*15:07:01G	15:07:01G		15:07:01G	15	212	3803	4	156	35	402	4627	WD	C	C		C	C	C	C	C
B*15:07:01	15:07:01		15:07:01G	17	200	286	0	66	37	102	708	WD	C	I		I	C	I	I	C
B*15:07:01:01	15:07:01:01	HLA00170	15:07:01G	0	5	3	0	1	2	0	11		WD						WD	WD
B*15:07:01:02	15:07:01:02	HLA16679	15:07:01G	2	0	101	0	5	0	15	123			WD		WD		I	WD	I

Supplemental Table 9: HLA-B Allele Summary [a]				Allele Count by Population Group [b]								3.0.0 CIWD Category by Population Group [c]								
Allele	Genomic typing	Allele ID	G group	AFA	API	EURO	MENA	HIS	NAM	UNK	Total	AFA	API	EURO	MENA	HIS	NAM	UNK	Total	Highest Frequency
B*15:07:03	15:07:03	HLA10692		0	0	0	0	0	0	1	1									
**B*15:08 total**	**15:08 total**			**26**	**3517**	**2408**	**1286**	**301**	**35**	**468**	**8041**	**WD**	**C**	**C**	**C**	**C**	**C**	**C**	**C**	**C**
B*15:08	15:08			19	836	838	413	110	15	172	2403	WD	C	I	C	C	C	C	C	C
B*15:08P	15:08P			0	0	2	0	0	0	0	2									
B*15:08:01	15:08:01	HLA00171		7	2681	1568	873	191	20	296	5636	WD	C	C	C	C	C	C	C	C
**B*15:09 total**	**15:09 total**			**37**	**93**	**3666**	**212**	**459**	**40**	**400**	**4907**	**WD**	**I**	**C**	**C**	**C**	**C**	**C**	**C**	**C**
**B*15:09:01G total**	**15:09:01G total**			**37**	**93**	**3666**	**212**	**459**	**40**	**400**	**4907**	**WD**	**I**	**C**	**C**	**C**	**C**	**C**	**C**	**C**
B*15:09	15:09		15:09:01G	37	61	3459	203	450	40	396	4646	WD	I	C	C	C	C	C	C	C
B*15:09P	15:09P			0	0	1	0	0	0	0	1									
B*15:09:01G	15:09:01G		15:09:01G	0	32	201	8	3	0	4	248		I	I	WD				I	I
B*15:09:01	15:09:01	HLA00172	15:09:01G	0	0	5	1	6	0	0	12			WD		WD			WD	WD
**B*15:10 total**	**15:10 total**			**12912**	**339**	**2706**	**756**	**2620**	**462**	**3541**	**23336**	**C**	**C**	**C**	**C**	**C**	**C**	**C**	**C**	**C**
B*15:10	15:10			949	19	274	16	360	44	227	1889	C	I	I	WD	C	C	C	C	C
B*15:10P	15:10P			0	0	4	0	0	0	1	5								WD	WD
B*15:10:01	15:10:01	HLA00173		11957	320	2428	740	2260	418	3312	21435	C	C	C	C	C	C	C	C	C
B*15:10:02	15:10:02	HLA04448		6	0	0	0	0	0	1	7	WD							WD	WD
**B*15:11 total**	**15:11 total**			**4**	**2511**	**238**	**71**	**13**	**0**	**302**	**3139**		**C**	**I**	**C**	**I**		**C**	**C**	**C**
B*15:11	15:11			0	414	39	9	6	0	28	496		C	WD	WD	WD		I	I	C
B*15:11P	15:11P			0	0	1	0	0	0	0	1									
**B*15:11:01G total**	**15:11:01G total**			**4**	**2097**	**198**	**62**	**7**	**0**	**274**	**2642**		**C**	**I**	**C**	**WD**		**C**	**C**	**C**
B*15:11:01G	15:11:01G		15:11:01G	2	1480	190	59	5	0	190	1926		C	I	C	WD		C	C	C
B*15:11:01	15:11:01	HLA00174	15:11:01G	2	615	8	3	2	0	84	714		C	WD				I	I	C
B*15:367	15:367	HLA13759	15:11:01G	0	2	0	0	0	0	0	2									
**B*15:12 total**	**15:12 total**			**1**	**1973**	**66**	**3**	**17**	**0**	**143**	**2203**		**C**	**WD**		**I**		**C**	**C**	**C**
**B*15:12:01G total**	**15:12:01G total**			**1**	**1973**	**66**	**3**	**17**	**0**	**143**	**2203**		**C**	**WD**		**I**		**C**	**C**	**C**
B*15:12	15:12	HLA00175	15:12:01G	0	413	3	0	3	0	33	452		C					I	I	C
B*15:12:01G	15:12:01G		15:12:01G	1	1539	63	3	12	0	101	1719		C	WD		I		I	C	C
B*15:19	15:19	HLA00182	15:12:01G	0	21	0	0	2	0	9	32		I					WD	WD	I
**B*15:13 total**	**15:13 total**			**88**	**2239**	**135**	**36**	**26**	**3**	**432**	**2959**	**C**	**C**	**I**	**WD**	**I**		**C**	**C**	**C**
B*15:13	15:13			9	178	18	2	4	1	45	257	WD	C	WD				I	I	C
B*15:13:01	15:13:01	HLA00176		79	2061	116	34	22	2	387	2701	C	C	WD	WD	I		C	C	C

Supplemental Table 9: HLA-B Allele Summary [a]				Allele Count by Population Group [b]								3.0.0 CIWD Category by Population Group [c]								
Allele	Genomic typing	Allele ID	G group	AFA	API	EURO	MENA	HIS	NAM	UNK	Total	AFA	API	EURO	MENA	HIS	NAM	UNK	Total	Highest Frequency
B*15:13:02	15:13:02	HLA06965		0	0	1	0	0	0	0	1									
B*15:14	15:14	HLA00177		0	0	111	0	0	0	10	121			WD				WD	WD	WD
B*15:15	15:15	HLA00178		52	56	421	12	5631	307	907	7386	C	I	I	WD	C	C	C	C	C
**B*15:16 total**	**15:16 total**			**6420**	**65**	**3816**	**213**	**2045**	**377**	**2447**	**15383**	**C**	**I**	**C**	**C**	**C**	**C**	**C**	**C**	**C**
B*15:16	15:16			407	7	438	4	252	37	190	1335	C	WD	I		C	C	C	I	C
B*15:16P	15:16P			0	0	1	0	0	0	0	1									
**B*15:16:01G total**	**15:16:01G total**			**6005**	**58**	**3377**	**209**	**1793**	**340**	**2254**	**14036**	**C**	**I**	**C**	**C**	**C**	**C**	**C**	**C**	**C**
B*15:16:01G	15:16:01G		15:16:01G	395	14	707	27	134	0	306	1583	C	I	I	WD	C		C	I	C
B*15:16:01	15:16:01		15:16:01G	4790	41	2492	174	1330	273	1697	10797	C	I	C	C	C	C	C	C	C
B*15:16:01:01	15:16:01:01	HLA00179	15:16:01G	0	0	3	0	0	0	0	3									
B*15:16:01:02	15:16:01:02	HLA14806	15:16:01G	820	3	175	8	329	67	251	1653	C		I	WD	C	C	C	C	C
B*15:16:02	15:16:02	HLA06327		6	0	0	0	0	0	3	9	WD							WD	WD
B*15:16:03	15:16:03	HLA06992		2	0	0	0	0	0	0	2									
**B*15:17 total**	**15:17 total**			**2097**	**12005**	**47557**	**6335**	**3867**	**354**	**8981**	**81196**	**C**	**C**	**C**	**C**	**C**	**C**	**C**	**C**	**C**
B*15:17	15:17			185	203	4013	601	623	40	678	6343	C	C	C	C	C	C	C	C	C
B*15:17P	15:17P			0	1	42	1	0	0	0	44			WD					WD	WD
**B*15:17:01G total**	**15:17:01G total**			**1911**	**11801**	**43415**	**5723**	**3243**	**314**	**8285**	**74692**	**C**	**C**	**C**	**C**	**C**	**C**	**C**	**C**	**C**
B*15:17:01G	15:17:01G		15:17:01G	498	10214	29871	4261	942	20	2370	48176	C	C	C	C	C	C	C	C	C
B*15:17:01	15:17:01		15:17:01G	1053	1097	11635	1289	1539	206	5567	22386	C	C	C	C	C	C	C	C	C
B*15:17:01:01	15:17:01:01	HLA00180	15:17:01G	359	490	1898	173	761	88	345	4114	C	C	C	C	C	C	C	C	C
B*15:17:01:02	15:17:01:02	HLA01439	15:17:01G	1	0	11	0	1	0	3	16			WD					WD	WD
B*15:17:02	15:17:02	HLA02141		1	0	85	10	1	0	18	115			WD	WD			I	WD	I
B*15:17:04	15:17:04	HLA14012		0	0	2	0	0	0	0	2									
**B*15:18 total**	**15:18 total**			**492**	**18871**	**20526**	**876**	**1539**	**266**	**2671**	**45241**	**C**	**C**	**C**	**C**	**C**	**C**	**C**	**C**	**C**
B*15:18	15:18			2	406	395	33	1	0	34	871		C	I	WD			I	I	C
B*15:18P	15:18P			0	0	29	0	0	0	0	29			WD					WD	WD
**B*15:18:01G total**	**15:18:01G total**			**490**	**18466**	**20095**	**843**	**1538**	**266**	**2637**	**44335**	**C**	**C**	**C**	**C**	**C**	**C**	**C**	**C**	**C**
B*15:18:01G	15:18:01G		15:18:01G	373	17318	18728	825	1105	201	2239	40789	C	C	C	C	C	C	C	C	C
B*15:18:01	15:18:01		15:18:01G	115	1126	1337	18	420	62	391	3469	C	C	C	WD	C	C	C	C	C
B*15:18:01:02	15:18:01:02	HLA14853	15:18:01G	0	0	5	0	0	0	0	5			WD					WD	WD
B*15:18:01:03	15:18:01:03	HLA16754	15:18:01G	2	19	25	0	13	3	7	69		I	WD		I		WD	WD	I

Supplemental Table 9: HLA-B Allele Summary [a]				Allele Count by Population Group [b]								3.0.0 CIWD Category by Population Group [c]								
Allele	Genomic typing	Allele ID	G group	AFA	API	EURO	MENA	HIS	NAM	UNK	Total	AFA	API	EURO	MENA	HIS	NAM	UNK	Total	Highest Frequency
B*15:198	15:198	HLA05265	15:18:01G	0	1	0	0	0	0	0	1									
B*15:380N	15:380N	HLA14651	15:18:01G	0	1	0	0	0	0	0	1									
B*15:388	15:388	HLA14874	15:18:01G	0	1	0	0	0	0	0	1									
B*15:18:02	15:18:02	HLA04093		0	0	3	0	0	0	0	3									
B*15:18:03	15:18:03	HLA04023		0	0	4	0	0	0	0	4									
**B*15:20 total**	**15:20 total**			**25**	**0**	**200**	**1**	**411**	**43**	**257**	**937**	**WD**		**I**		**C**	**C**	**C**	**I**	**C**
B*15:20	15:20	HLA00183		25	0	200	1	411	43	257	937	WD		I		C	C	C	I	C
**B*15:21 total**	**15:21 total**			**6**	**3031**	**169**	**14**	**51**	**2**	**837**	**4110**	**WD**	**C**	**I**	**WD**	**I**		**C**	**C**	**C**
**B*15:21:01G total**	**15:21:01G total**			**6**	**3031**	**169**	**14**	**51**	**2**	**837**	**4110**	**WD**	**C**	**I**	**WD**	**I**		**C**	**C**	**C**
B*15:21	15:21		15:21:01G	6	3007	169	14	51	2	837	4086	WD	C	I	WD	I		C	C	C
B*15:21:01G	15:21:01G		15:21:01G	0	24	0	0	0	0	0	24		I						WD	I
B*15:23	15:23	HLA00186		15	4	439	2	19	1	40	520	WD		I		I		I	I	I
**B*15:24 total**	**15:24 total**			**19**	**13**	**4920**	**50**	**104**	**8**	**473**	**5587**	**WD**	**I**	**C**	**C**	**C**	**C**	**C**	**C**	**C**
B*15:24	15:24			4	0	909	7	29	2	95	1046			I	WD	I		I	I	I
B*15:24P	15:24P			0	0	4	0	0	0	0	4									
B*15:24:01	15:24:01	HLA00187		15	13	4004	43	75	6	378	4534	WD	I	C	C	C	WD	C	C	C
B*15:24:02	15:24:02	HLA09307		0	0	3	0	0	0	0	3									
**B*15:25 total**	**15:25 total**			**40**	**9348**	**181**	**48**	**50**	**24**	**406**	**10097**	**C**	**C**	**I**	**C**	**I**	**C**	**C**	**C**	**C**
B*15:25	15:25			12	809	14	2	8	3	44	892	WD	C	WD		I		I	I	C
**B*15:25:01G total**	**15:25:01G total**			**28**	**8536**	**167**	**46**	**42**	**21**	**362**	**9202**	**WD**	**C**	**I**	**C**	**I**	**C**	**C**	**C**	**C**
B*15:25:01G	15:25:01G		15:25:01G	15	7061	163	39	31	12	247	7568	WD	C	I	WD	I	C	C	C	C
B*15:25:01	15:25:01	HLA00188	15:25:01G	13	1474	4	7	11	9	115	1633	WD	C		WD	I	C	I	C	C
B*15:271	15:271	HLA09329	15:25:01G	0	1	0	0	0	0	0	1									
B*15:25:02	15:25:02	HLA03906		0	3	0	0	0	0	0	3									
**B*15:27 total**	**15:27 total**			**2**	**1611**	**147**	**9**	**76**	**0**	**261**	**2106**		**C**	**I**	**WD**	**C**		**C**	**C**	**C**
B*15:27	15:27			0	77	48	0	19	0	33	177		I	WD		I		I	I	I
B*15:27:01	15:27:01	HLA00190		2	1533	38	9	3	0	129	1714		C	WD	WD			I	C	C
B*15:27:02	15:27:02	HLA03103		0	0	61	0	54	0	99	214			WD		I		I	I	I
B*15:27:03	15:27:03	HLA03302		0	1	0	0	0	0	0	1									
**B*15:28 total**	**15:28 total**			**0**	**1**	**0**	**0**	**0**	**0**	**3**	**4**									
**B*15:28:01G total**	**15:28:01G total**			**0**	**1**	**0**	**0**	**0**	**0**	**3**	**4**									

Supplemental Table 9: HLA-B Allele Summary [a]				Allele Count by Population Group [b]								3.0.0 CIWD Category by Population Group [c]								
**Allele**	**Genomic typing**	**Allele ID**	**G group**	**AFA**	**API**	**EURO**	**MENA**	**HIS**	**NAM**	**UNK**	**Total**	**AFA**	**API**	**EURO**	**MENA**	**HIS**	**NAM**	**UNK**	**Total**	**Highest Frequency**
B*15:28	15:28	HLA00191	15:28:01G	0	1	0	0	0	0	3	4									
B*15:29	15:29	HLA00192		4	1036	472	279	22	3	191	2007		C	I	C	I		C	C	C
**B*15:30 total**	**15:30 total**			**29**	**26**	**138**	**5**	**2408**	**118**	**311**	**3035**	**WD**	**I**	**I**	**WD**	**C**	**C**	**C**	**C**	**C**
**B*15:30:01G total**	**15:30:01G total**			**29**	**26**	**138**	**5**	**2408**	**118**	**311**	**3035**	**WD**	**I**	**I**	**WD**	**C**	**C**	**C**	**C**	**C**
B*15:30	15:30		15:30:01G	29	26	138	5	2408	118	311	3035	WD	I	I	WD	C	C	C	C	C
B*15:31	15:31	HLA00194		180	2	94	61	52	5	59	453	C		WD	C	I	WD	I	I	C
**B*15:32 total**	**15:32 total**			**1**	**1364**	**47**	**20**	**6**	**1**	**52**	**1491**		**C**	**WD**	**WD**	**WD**		**I**	**I**	**C**
B*15:32	15:32			0	308	13	2	1	0	19	343		C	WD				I	I	C
B*15:32:01	15:32:01	HLA00195		1	1056	34	18	5	1	33	1148		C	WD	WD	WD		I	I	C
B*15:33	15:33	HLA00196		2	0	598	1	21	7	37	666			I		I	C	I	I	C
B*15:34	15:34	HLA00197		0	79	1089	10	8	2	50	1238		I	I	WD	I		I	I	I
B*15:35	15:35	HLA00198		3	4310	239	15	94	9	1369	6039		C	I	WD	C	C	C	C	C
B*15:36	15:36	HLA00199		0	0	0	0	0	0	1	1									
B*15:37	15:37	HLA00200		277	1	29	6	34	5	76	428	C		WD	WD	I	WD	I	I	C
**B*15:38 total**	**15:38 total**			**1**	**205**	**10**	**1**	**16**	**1**	**18**	**252**		**C**	**WD**		**I**		**I**	**I**	**C**
B*15:38	15:38			0	17	1	0	5	0	1	24		I			WD			WD	I
B*15:38:01	15:38:01	HLA00201		1	188	9	1	11	1	17	228		C	WD		I		I	I	C
**B*15:39 total**	**15:39 total**			**8**	**19**	**1401**	**14**	**657**	**34**	**231**	**2364**	**WD**	**I**	**C**	**WD**	**C**	**C**	**C**	**C**	**C**
B*15:39	15:39			0	0	104	4	67	3	17	195			WD		I		I	I	I
B*15:39P	15:39P			0	0	2	0	0	0	0	2									
B*15:39:01	15:39:01	HLA00202		8	19	1287	10	590	31	213	2158	WD	I	C	WD	C	C	C	C	C
B*15:39:02	15:39:02	HLA05472		0	0	8	0	0	0	1	9			WD					WD	WD
**B*15:40 total**	**15:40 total**			**21**	**5**	**21**	**0**	**278**	**77**	**50**	**452**	**WD**	**WD**	**WD**		**C**	**C**	**I**	**I**	**C**
B*15:40	15:40			19	5	20	0	228	69	45	386	WD	WD	WD		C	C	I	I	C
B*15:40:01	15:40:01	HLA00203		2	0	1	0	50	8	5	66					I	C	WD	WD	C
B*15:42	15:42	HLA00205		0	0	7	0	0	0	0	7			WD					WD	WD
B*15:43	15:43	HLA00206		0	0	2	0	0	0	0	2									
B*15:44	15:44	HLA00207		0	18	1	0	1	0	8	28		I					WD	WD	I
B*15:45	15:45	HLA00208		1	0	149	1	2	0	22	175			I				I	I	I
B*15:46	15:46	HLA00209		0	34	4	1	0	0	4	43		I						WD	I
**B*15:47 total**	**15:47 total**			**123**	**1**	**13**	**10**	**34**	**10**	**37**	**228**	**C**		**WD**	**WD**	**I**	**C**	**I**	**I**	**C**

Supplemental Table 9: HLA-B Allele Summary [a]				Allele Count by Population Group [b]								3.0.0 CIWD Category by Population Group [c]								
Allele	Genomic typing	Allele ID	G group	AFA	API	EURO	MENA	HIS	NAM	UNK	Total	AFA	API	EURO	MENA	HIS	NAM	UNK	Total	Highest Frequency
B*15:47	15:47			27	0	3	0	8	2	1	41	WD				I			WD	I
B*15:47:01	15:47:01	HLA00210		95	1	10	10	26	8	36	186	C		WD	WD	I	C	I	I	C
B*15:47:02	15:47:02	HLA07652		1	0	0	0	0	0	0	1									
B*15:48	15:48	HLA00211		1	3	6	0	142	10	12	174			WD		C	C	WD	I	C
B*15:50	15:50	HLA00979		0	1	1	0	0	0	1	3									
B*15:51	15:51	HLA00980		0	0	3	0	0	0	0	3									
B*15:52	15:52	HLA01053		66	0	1	0	4	0	14	85	C						I	WD	C
B*15:53	15:53	HLA01054		4	1	4	0	0	2	2	13								WD	WD
B*15:54	15:54	HLA01103		57	0	2	2	1	0	8	70	C						WD	WD	C
B*15:55	15:55	HLA01113		3	0	4	18	13	0	4	42				WD	I			WD	I
B*15:56	15:56	HLA01114		0	12	0	0	0	2	1	15		WD						WD	WD
B*15:57	15:57	HLA01117		0	0	6	0	0	0	1	7			WD					WD	WD
B*15:58	15:58	HLA01118		0	74	1	2	0	0	9	86		I					WD	WD	I
B*15:61	15:61	HLA01250		1	0	0	0	0	0	0	1									
B*15:62	15:62	HLA01271		8	0	0	0	0	0	0	8	WD							WD	WD
B*15:63	15:63	HLA01284		0	0	17	2	19	11	19	68			WD		I	C	I	WD	C
**B*15:64 total**	**15:64 total**			**1**	**23**	**4**	**2**	**0**	**0**	**1**	**31**		**I**						**WD**	**I**
B*15:64	15:64			0	3	2	0	0	0	0	5								WD	WD
B*15:64:01	15:64:01	HLA01285		0	20	2	0	0	0	0	22		I						WD	I
B*15:64:02	15:64:02	HLA08373		1	0	0	2	0	0	1	4									
B*15:65	15:65	HLA01426		0	1	50	0	5	0	11	67			WD		WD		WD	WD	WD
B*15:66	15:66	HLA01440		0	2	0	0	0	0	0	2									
B*15:67	15:67	HLA01448		29	0	4	6	1	0	5	45	WD			WD			WD	WD	WD
B*15:68	15:68	HLA01479		0	3	0	0	0	0	1	4									
B*15:69	15:69	HLA01494		0	0	2	0	0	0	8	10							WD	WD	WD
B*15:70	15:70	HLA01536		0	0	7	0	5	0	4	16			WD		WD			WD	WD
B*15:71	15:71	HLA01561		2	2	336	25	6	0	15	386			I	WD	WD		I	I	I
B*15:72	15:72	HLA01567		0	8	0	0	0	0	1	9		WD						WD	WD
B*15:73	15:73	HLA01611		0	2	178	29	2	0	22	233			I	WD			I	I	I
B*15:75	15:75	HLA01690		1	198	0	10	0	3	3	215		C		WD				I	C
B*15:76	15:76	HLA01698		0	0	6	0	0	0	1	7			WD					WD	WD

Supplemental Table 9: HLA-B Allele Summary [a]				Allele Count by Population Group [b]								3.0.0 CIWD Category by Population Group [c]								
Allele	Genomic typing	Allele ID	G group	AFA	API	EURO	MENA	HIS	NAM	UNK	Total	AFA	API	EURO	MENA	HIS	NAM	UNK	Total	Highest Frequency
**B*15:78 total**	**15:78 total**			**1**	**3**	**76**	**0**	**0**	**0**	**4**	**84**			**WD**					**WD**	**WD**
B*15:78	15:78			0	0	4	0	0	0	0	4									
B*15:78:01	15:78:01	HLA01751		1	1	71	0	0	0	4	77			WD					WD	WD
B*15:78:02	15:78:02	HLA03961		0	2	1	0	0	0	0	3									
B*15:79N	15:79N	HLA01767		0	0	39	0	0	0	1	40			WD					WD	WD
B*15:80	15:80	HLA01768		0	1	3	0	0	0	1	5								WD	WD
B*15:82	15:82	HLA01801		2	2	417	1	2	0	16	440			I				I	I	I
B*15:83	15:83	HLA01845		5	0	0	1	0	1	1	8	WD							WD	WD
B*15:85	15:85	HLA01911		0	0	0	0	1	0	0	1									
B*15:86	15:86	HLA01912		0	2	0	0	0	0	0	2									
B*15:87	15:87	HLA01913		0	0	4	1	0	0	0	5								WD	WD
B*15:88	15:88	HLA01915		0	11	1	0	0	0	1	13		WD						WD	WD
B*15:89	15:89	HLA01916		0	10	0	0	0	0	0	10		WD						WD	WD
B*15:95	15:95	HLA02032		3	0	0	0	0	0	0	3									
B*15:96	15:96	HLA02035		0	0	8	0	0	0	0	8			WD					WD	WD
B*15:97	15:97	HLA02099		0	0	4	0	0	0	0	4									
B*15:98	15:98	HLA02113		0	0	5	1	0	0	0	6			WD					WD	WD
B*15:99	15:99	HLA02119		1	0	0	0	0	0	1	2									
B*15:106	15:106	HLA02332		0	5	0	0	0	0	0	5		WD						WD	WD
B*15:107	15:107	HLA02368		1	0	0	0	0	0	0	1									
B*15:108	15:108	HLA02376		0	0	13	0	6	0	0	19			WD		WD			WD	WD
B*15:109	15:109	HLA02387		0	2	1	0	0	0	1	4									
B*15:110	15:110	HLA02421		2	1	0	0	13	3	1	20					I			WD	I
B*15:113	15:113	HLA02483		0	94	1	2	0	0	1	98		I						WD	I
B*15:117	15:117	HLA02618		0	0	18	0	0	0	2	20			WD					WD	WD
B*15:120	15:120	HLA02665		0	0	0	0	0	0	1	1									
B*15:122	15:122	HLA02759		0	0	44	0	0	0	0	44			WD					WD	WD
**B*15:123 total**	**15:123 total**			**2**	**0**	**3**	**16**	**3**	**0**	**0**	**24**				**WD**				**WD**	**WD**
**B*15:123:01G total**	**15:123:01G total**			**2**	**0**	**3**	**16**	**3**	**0**	**0**	**24**				**WD**				**WD**	**WD**
B*15:123:01G	15:123:01G		15:123:01G	2	0	3	16	2	0	0	23				WD				WD	WD
B*15:151	15:151	HLA03470	15:123:01G	0	0	0	0	1	0	0	1									

Supplemental Table 9: HLA-B Allele Summary [a]				Allele Count by Population Group [b]								3.0.0 CIWD Category by Population Group [c]								
Allele	Genomic typing	Allele ID	G group	AFA	API	EURO	MENA	HIS	NAM	UNK	Total	AFA	API	EURO	MENA	HIS	NAM	UNK	Total	Highest Frequency
B*15:125	15:125	HLA02781		0	0	328	0	0	0	3	331			I					I	I
B*15:128	15:128	HLA02875		0	0	6	0	0	0	0	6			WD					WD	WD
B*15:131	15:131	HLA02974		0	0	2	0	0	0	0	2									
B*15:132	15:132	HLA02976		1	1	79	1	1	1	11	95			WD				WD	WD	WD
B*15:133	15:133	HLA03019		0	2	0	0	0	0	0	2									
B*15:135	15:135	HLA03080		0	1	150	1	0	1	7	160			I				WD	WD	I
B*15:141	15:141	HLA03217		0	43	3	0	0	1	18	65		I					I	WD	I
B*15:142	15:142	HLA03244		0	0	37	0	0	0	0	37			WD					WD	WD
B*15:145	15:145	HLA03281		0	0	2	0	0	0	0	2									
B*15:147	15:147	HLA03319		0	0	12	0	0	0	6	18			WD				WD	WD	WD
B*15:150	15:150	HLA03441		0	0	1	0	15	1	2	19					I			WD	I
B*15:153	15:153	HLA03606		0	0	99	0	1	0	8	108			WD				WD	WD	WD
B*15:154	15:154	HLA03614		0	0	24	0	0	0	2	26			WD					WD	WD
B*15:155	15:155	HLA03615		0	0	0	0	1	0	1	2									
B*15:156	15:156	HLA03620		1	0	0	0	0	0	2	3									
B*15:157	15:157	HLA03646		0	0	43	0	0	0	1	44			WD					WD	WD
B*15:158	15:158	HLA03693		6	0	0	0	0	0	2	8	WD							WD	WD
B*15:159	15:159	HLA03708		0	9	0	0	0	0	1	10		WD						WD	WD
B*15:160	15:160	HLA03711		0	0	22	0	0	0	0	22			WD					WD	WD
B*15:161	15:161	HLA03804		0	0	1	1	0	0	1	3									
B*15:162	15:162	HLA03951		0	0	0	8	0	0	53	61				WD			I	WD	I
B*15:163	15:163	HLA03947		0	0	0	1	0	0	0	1									
B*15:164	15:164	HLA03949		0	0	28	0	0	0	0	28			WD					WD	WD
B*15:167	15:167	HLA04084		0	0	8	0	0	0	0	8			WD					WD	WD
B*15:170	15:170	HLA04028		0	13	0	0	0	0	0	13		I						WD	I
B*15:171	15:171	HLA04169		0	0	4	0	0	0	0	4									
B*15:172	15:172	HLA04171		0	0	7	0	0	0	0	7			WD					WD	WD
B*15:173	15:173	HLA04187		0	0	63	1	0	0	1	65			WD					WD	WD
B*15:174	15:174	HLA04213		0	0	5	1	0	0	0	6			WD					WD	WD
B*15:175	15:175	HLA04218		0	0	4	3	0	0	0	7								WD	WD
B*15:176	15:176	HLA04232		0	0	1	0	0	0	0	1									

Supplemental Table 9: HLA-B Allele Summary [a]				Allele Count by Population Group [b]								3.0.0 CIWD Category by Population Group [c]								
Allele	Genomic typing	Allele ID	G group	AFA	API	EURO	MENA	HIS	NAM	UNK	Total	AFA	API	EURO	MENA	HIS	NAM	UNK	Total	Highest Frequency
B*15:178	15:178	HLA04158		0	1	1	1	0	0	0	3									
B*15:180	15:180	HLA04165		0	0	1	0	0	0	0	1									
B*15:181N	15:181N	HLA04457		0	0	8	0	0	0	0	8			WD					WD	WD
B*15:183	15:183	HLA04508		0	0	6	0	0	0	0	6			WD					WD	WD
B*15:184	15:184	HLA04533		1	0	2	0	0	0	1	4									
B*15:185	15:185	HLA04413		0	2	0	0	0	0	0	2									
B*15:190N	15:190N	HLA04770		0	0	5	0	0	0	0	5			WD					WD	WD
B*15:192	15:192	HLA05135		0	1	0	0	0	0	0	1									
B*15:194	15:194	HLA05259		0	1	0	0	0	0	0	1									
B*15:204	15:204	HLA05479		0	7	0	0	0	0	0	7		WD						WD	WD
B*15:206	15:206	HLA05500		0	0	4	0	0	0	0	4									
B*15:207	15:207	HLA05509		0	0	5	0	0	0	0	5			WD					WD	WD
B*15:208	15:208	HLA05516		0	0	9	0	0	0	1	10			WD					WD	WD
B*15:210	15:210	HLA05953		1	0	0	0	0	0	0	1									
B*15:211	15:211	HLA05957		0	0	8	0	0	0	4	12			WD					WD	WD
B*15:212	15:212	HLA06030		0	0	1	0	0	0	0	1									
B*15:217	15:217	HLA06222		0	0	0	0	1	0	1	2									
B*15:218Q	15:218Q	HLA06248		0	0	1	0	0	0	0	1									
B*15:222	15:222	HLA06366		1	0	0	0	0	0	2	3									
B*15:229	15:229	HLA06921		0	0	0	1	0	0	0	1									
B*15:232	15:232	HLA06948		0	0	2	0	0	0	0	2									
B*15:233	15:233	HLA06988		0	0	1	0	0	0	0	1									
B*15:235	15:235	HLA07093		1	0	0	0	0	0	0	1									
B*15:236	15:236	HLA07179		0	0	1	0	0	0	0	1									
B*15:237	15:237	HLA07385		0	1	7	0	0	0	0	8			WD					WD	WD
B*15:238	15:238	HLA07412		0	0	1	1	0	0	0	2									
B*15:239	15:239	HLA07453		0	0	9	0	1	0	0	10			WD					WD	WD
B*15:240	15:240	HLA07649		0	42	0	1	0	1	1	45		I						WD	I
**B*15:242 total**	**15:242 total**			**1**	**0**	**0**	**0**	**0**	**0**	**0**	**1**									
B*15:242	15:242			1	0	0	0	0	0	0	1									
B*15:248	15:248	HLA08068		0	0	7	0	0	0	0	7			WD					WD	WD

Supplemental Table 9: HLA-B Allele Summary [a]				Allele Count by Population Group [b]								3.0.0 CIWD Category by Population Group [c]								
Allele	Genomic typing	Allele ID	G group	AFA	API	EURO	MENA	HIS	NAM	UNK	Total	AFA	API	EURO	MENA	HIS	NAM	UNK	Total	Highest Frequency
B*15:251	15:251	HLA08072		0	0	2	0	0	0	0	2									
B*15:252	15:252	HLA08074		0	0	2	0	0	0	0	2									
B*15:254	15:254	HLA08144		2	0	0	0	0	0	1	3									
B*15:256	15:256	HLA08368		0	0	1	0	0	0	0	1									
B*15:264	15:264	HLA08783		0	0	1	0	0	0	0	1									
B*15:266	15:266	HLA09000		4	0	0	0	0	0	0	4									
B*15:272N	15:272N	HLA09437		0	0	1	0	0	0	0	1									
**B*15:275 total**	**15:275 total**			**0**	**1**	**0**	**0**	**0**	**0**	**0**	**1**									
B*15:275	15:275			0	1	0	0	0	0	0	1									
B*15:276	15:276	HLA09517		0	0	0	0	0	0	1	1									
B*15:277	15:277	HLA09573		0	2	0	0	0	0	0	2									
B*15:278	15:278	HLA09574		0	2	0	0	0	0	0	2									
B*15:279	15:279	HLA09576		0	0	0	0	0	0	2	2									
B*15:280	15:280	HLA09578		0	0	1	0	0	0	0	1									
B*15:282	15:282	HLA09708		1	0	0	0	0	0	0	1									
B*15:285	15:285	HLA10032		0	3	0	0	0	0	0	3									
B*15:287	15:287	HLA10035		0	0	2	0	0	0	0	2									
B*15:288	15:288	HLA10036		0	0	1	0	0	0	0	1									
B*15:290	15:290	HLA10042		0	20	0	0	0	0	0	20		I						WD	I
B*15:293	15:293	HLA10415		0	0	0	0	1	0	0	1									
B*15:309	15:309	HLA11024		0	0	3	0	0	0	0	3									
B*15:310	15:310	HLA11026		0	0	0	0	1	0	0	1									
B*15:312	15:312	HLA11029		2	0	0	0	0	0	0	2									
B*15:313	15:313	HLA11244		1	0	0	0	0	0	0	1									
B*15:315	15:315	HLA11324		0	0	2	0	0	0	0	2									
B*15:317	15:317	HLA11428		0	0	1	0	0	0	0	1									
B*15:319	15:319	HLA11483		0	6	0	0	0	0	0	6		WD						WD	WD
B*15:323	15:323	HLA11660		1	0	0	0	0	0	0	1									
B*15:324	15:324	HLA11728		0	0	3	0	1	0	0	4									
B*15:325	15:325	HLA11774		0	1	0	0	0	0	0	1									
B*15:328	15:328	HLA12132		0	1	0	0	0	0	0	1									

Supplemental Table 9: HLA-B Allele Summary [a]				Allele Count by Population Group [b]								3.0.0 CIWD Category by Population Group [c]								
Allele	Genomic typing	Allele ID	G group	AFA	API	EURO	MENA	HIS	NAM	UNK	Total	AFA	API	EURO	MENA	HIS	NAM	UNK	Total	Highest Frequency
B*15:329	15:329	HLA12133		0	1	0	0	0	0	0	1									
B*15:330	15:330	HLA12285		0	1	0	0	0	0	0	1									
B*15:331	15:331	HLA12289		0	1	0	0	0	0	0	1									
B*15:332	15:332	HLA12290		0	2	0	0	0	0	0	2									
B*15:335	15:335	HLA12299		0	11	0	0	0	0	0	11		WD						WD	WD
B*15:337	15:337	HLA12483		0	1	0	0	0	0	0	1									
B*15:341	15:341	HLA12753		0	1	0	0	0	0	0	1									
B*15:342	15:342	HLA12754		0	2	0	0	0	0	0	2									
B*15:343	15:343	HLA12755		0	1	0	0	0	0	0	1									
B*15:345	15:345	HLA12989		0	1	0	0	0	0	0	1									
B*15:346	15:346	HLA13003		0	0	1	0	0	0	0	1									
B*15:347	15:347	HLA13007		0	1	0	0	0	0	0	1									
B*15:357	15:357	HLA13479		0	1	0	0	0	0	0	1									
B*15:360	15:360	HLA13534		0	1	0	0	0	0	0	1									
B*15:361	15:361	HLA13535		0	1	0	0	0	0	0	1									
B*15:362	15:362	HLA13548		0	0	0	0	0	0	1	1									
**B*15:363 total**	**15:363 total**			**0**	**0**	**0**	**0**	**0**	**0**	**1**	**1**									
B*15:363:02	15:363:02	HLA15635		0	0	0	0	0	0	1	1									
B*15:364	15:364	HLA13857		0	2	0	0	0	0	0	2									
B*15:366	15:366	HLA13859		0	2	0	0	0	0	1	3									
B*15:369	15:369	HLA14015		0	0	0	0	1	0	0	1									
B*15:376	15:376	HLA14309		0	2	0	0	0	0	0	2									
B*15:377Q	15:377Q	HLA14221		0	0	3	0	0	0	0	3									
B*15:378	15:378	HLA14442		0	1	0	0	0	0	0	1									
B*15:387	15:387	HLA14936		0	1	0	0	0	0	0	1									
B*15:390	15:390	HLA14974		0	0	1	0	0	0	0	1									
B*15:391	15:391	HLA14975		0	0	1	0	0	0	0	1									
B*15:392	15:392	HLA14976		0	0	3	0	0	0	1	4									
B*15:395	15:395	HLA15277		0	1	0	0	0	0	0	1									
B*15:406	15:406	HLA16221		0	0	1	0	0	0	0	1									
B*15:417	15:417	HLA16391		0	0	0	0	0	0	1	1									

Supplemental Table 9: HLA-B Allele Summary [a]				Allele Count by Population Group [b]								3.0.0 CIWD Category by Population Group [c]								
Allele	Genomic typing	Allele ID	G group	AFA	API	EURO	MENA	HIS	NAM	UNK	Total	AFA	API	EURO	MENA	HIS	NAM	UNK	Total	Highest Frequency
B*15:434	15:434	HLA17757		0	0	0	0	0	0	1	1									
B*15:CODE	15:CODE			2717	2587	76228	529	3722	357	5669	91809	NA	NA	NA	NA	NA	NA	NA	NA	NA
**B*18:01 total**	**18:01 total**			**11163**	**24982**	**592773**	**21083**	**25357**	**1977**	**53551**	**730886**	**C**	**C**	**C**	**C**	**C**	**C**	**C**	**C**	**C**
B*18:01	18:01			3	1	4682	15	10	2	196	4909			C	WD	I		C	C	C
B*18:01P	18:01P			1	1	1234	3	3	0	8	1250			C				WD	I	C
**B*18:01:01G total**	**18:01:01G total**			**11145**	**24907**	**586651**	**21062**	**25331**	**1974**	**53310**	**724380**	**C**	**C**	**C**	**C**	**C**	**C**	**C**	**C**	**C**
B*18:01:01G	18:01:01G		18:01:01G	9236	23467	564771	20538	19362	1505	50040	688919	C	C	C	C	C	C	C	C	C
B*18:01:01	18:01:01		18:01:01G	608	768	11062	232	2246	168	1379	16463	C	C	C	C	C	C	C	C	C
B*18:01:01:01	18:01:01:01	HLA00213	18:01:01G	941	27	4049	28	2122	167	846	8180	C	I	C	WD	C	C	C	C	C
B*18:01:01:02	18:01:01:02	HLA07768	18:01:01G	259	644	6712	264	1537	125	1008	10549	C	C	C	C	C	C	C	C	C
B*18:01:01:03	18:01:01:03	HLA14812	18:01:01G	101	1	21	0	46	7	33	209	C		WD		I	C	I	I	C
B*18:01:01:04	18:01:01:04	HLA14962	18:01:01G	0	0	1	0	4	0	0	5								WD	WD
B*18:01:01:05	18:01:01:05	HLA16275	18:01:01G	0	0	31	0	0	0	0	31			WD					WD	WD
B*18:01:01:06	18:01:01:06	HLA16637	18:01:01G	0	0	1	0	0	0	0	1									
B*18:01:03	18:01:03	HLA02650	18:01:01G	0	0	0	0	14	2	3	19					I			WD	I
B*18:01:25	18:01:25	HLA16857	18:01:01G	0	0	1	0	0	0	0	1									
**B*18:131 total**	**18:131 total**			**0**	**0**	**2**	**0**	**0**	**0**	**1**	**3**									
B*18:131	18:131		18:01:01G	0	0	2	0	0	0	1	3									
B*18:01:02	18:01:02	HLA01565		10	0	161	0	13	1	24	209	WD		I		I		I	I	I
B*18:01:05	18:01:05	HLA04249		0	0	12	0	0	0	0	12			WD					WD	WD
B*18:01:06	18:01:06	HLA04432		0	72	3	0	0	0	0	75		I						WD	I
B*18:01:07	18:01:07	HLA04453		0	0	1	0	0	0	0	1									
B*18:01:08	18:01:08	HLA04566		0	0	4	0	0	0	9	13							WD	WD	WD
B*18:01:09	18:01:09	HLA05483		0	0	8	0	0	0	0	8			WD					WD	WD
B*18:01:10	18:01:10	HLA06233		4	0	5	0	0	0	3	12			WD					WD	WD
B*18:01:11	18:01:11	HLA06234		0	1	1	0	0	0	0	2									
B*18:01:12	18:01:12	HLA06354		0	0	8	0	0	0	0	8			WD					WD	WD
B*18:01:13	18:01:13	HLA06358		0	0	0	0	0	0	1	1									
B*18:01:16	18:01:16	HLA07691		0	0	0	3	0	0	0	3									
B*18:01:20	18:01:20	HLA11231		0	0	2	0	0	0	0	2									
B*18:01:23	18:01:23	HLA12983		0	0	1	0	0	0	0	1									

Supplemental Table 9: HLA-B Allele Summary [a]				Allele Count by Population Group [b]								3.0.0 CIWD Category by Population Group [c]								
Allele	Genomic typing	Allele ID	G group	AFA	API	EURO	MENA	HIS	NAM	UNK	Total	AFA	API	EURO	MENA	HIS	NAM	UNK	Total	Highest Frequency
B*18:02	18:02	HLA00214		2	1466	75	24	2	1	199	1769		C	WD	WD			C	C	C
B*18:03	18:03	HLA00215		123	35	13495	182	231	29	464	14559	C	I	C	C	C	C	C	C	C
**B*18:04 total**	**18:04 total**			**17**	**2**	**620**	**6**	**133**	**19**	**87**	**884**	**WD**		**I**	**WD**	**C**	**C**	**I**	**I**	**C**
B*18:04	18:04			17	2	584	6	133	19	83	844	WD		I	WD	C	C	I	I	C
B*18:04:01	18:04:01	HLA00216		0	0	36	0	0	0	4	40			WD					WD	WD
**B*18:05 total**	**18:05 total**			**7**	**19**	**1787**	**76**	**135**	**9**	**191**	**2224**	**WD**	**I**	**C**	**C**	**C**	**C**	**C**	**C**	**C**
**B*18:05:01G total**	**18:05:01G total**			**7**	**19**	**1787**	**76**	**135**	**9**	**191**	**2224**	**WD**	**I**	**C**	**C**	**C**	**C**	**C**	**C**	**C**
B*18:05	18:05		18:05:01G	7	19	1787	76	135	9	191	2224	WD	I	C	C	C	C	C	C	C
B*18:06	18:06	HLA00218		1	1	28	15	24	2	8	79			WD	WD	I		WD	WD	I
**B*18:07 total**	**18:07 total**			**0**	**2**	**244**	**23**	**4**	**0**	**12**	**285**			**I**	**WD**			**WD**	**I**	**I**
B*18:07	18:07			0	1	33	4	0	0	2	40			WD					WD	WD
B*18:07:01	18:07:01	HLA00219		0	1	193	19	4	0	10	227			I	WD			WD	I	I
B*18:07:02	18:07:02	HLA04075		0	0	18	0	0	0	0	18			WD					WD	WD
B*18:08	18:08	HLA01055		0	0	31	0	0	0	5	36			WD				WD	WD	WD
B*18:09	18:09	HLA01131		0	0	46	0	0	0	0	46			WD					WD	WD
B*18:10	18:10	HLA01177		0	0	4	1	0	0	1	6								WD	WD
B*18:11	18:11	HLA01270		0	0	33	3	2	0	8	46			WD				WD	WD	WD
**B*18:12 total**	**18:12 total**			**0**	**0**	**51**	**0**	**0**	**0**	**5**	**56**			**WD**				**WD**	**WD**	**WD**
B*18:12	18:12			0	0	41	0	0	0	4	45			WD					WD	WD
B*18:12:01	18:12:01	HLA01281		0	0	10	0	0	0	1	11			WD					WD	WD
B*18:13	18:13	HLA01353		0	0	56	0	0	0	15	71			WD				I	WD	I
B*18:14	18:14	HLA01506		1	1	219	1	6	0	8	236			I		WD		WD	I	I
B*18:15	18:15	HLA01508		0	1	5	2	2	0	8	18			WD				WD	WD	WD
**B*18:18 total**	**18:18 total**			**0**	**0**	**473**	**4**	**3**	**0**	**14**	**494**			**I**				**I**	**I**	**I**
**B*18:18:01G total**	**18:18:01G total**			**0**	**0**	**473**	**4**	**3**	**0**	**14**	**494**			**I**				**I**	**I**	**I**
B*18:18	18:18		18:18:01G	0	0	473	4	3	0	14	494			I				I	I	I
B*18:19	18:19	HLA01782		0	0	7	8	0	0	1	16			WD	WD				WD	WD
B*18:20	18:20	HLA01914		0	0	66	0	5	0	6	77			WD		WD		WD	WD	WD
B*18:22	18:22	HLA02388		0	5	0	0	0	0	0	5		WD						WD	WD
B*18:25	18:25	HLA02783		46	19	8	0	2	0	15	90	C	I	WD				I	WD	C
B*18:26	18:26	HLA02851		2	0	1	1	0	0	0	4									

Supplemental Table 9: HLA-B Allele Summary [a]				Allele Count by Population Group [b]								3.0.0 CIWD Category by Population Group [c]								
**Allele**	**Genomic typing**	**Allele ID**	**G group**	**AFA**	**API**	**EURO**	**MENA**	**HIS**	**NAM**	**UNK**	**Total**	**AFA**	**API**	**EURO**	**MENA**	**HIS**	**NAM**	**UNK**	**Total**	**Highest Frequency**
B*18:27	18:27	HLA02995		0	0	3	0	0	0	0	3									
B*18:28	18:28	HLA03180		0	0	74	0	0	0	1	75			WD					WD	WD
B*18:30	18:30	HLA03462		0	0	8	0	0	1	1	10			WD					WD	WD
B*18:31	18:31	HLA03463		0	0	4	0	0	0	0	4									
B*18:32	18:32	HLA03510		0	0	4	0	1	0	0	5								WD	WD
B*18:33	18:33	HLA03612		0	0	60	3	0	0	0	63			WD					WD	WD
B*18:34	18:34	HLA03717		0	0	51	0	0	0	2	53			WD					WD	WD
B*18:35	18:35	HLA03946		0	0	0	0	0	0	1	1									
B*18:36	18:36	HLA03956		0	0	5	0	0	0	0	5			WD					WD	WD
**B*18:37 total**	**18:37 total**			**0**	**0**	**3**	**3**	**0**	**0**	**0**	**6**								**WD**	**WD**
B*18:37	18:37			0	0	3	3	0	0	0	6								WD	WD
B*18:39	18:39	HLA04091		0	0	3	0	0	0	0	3									
B*18:40	18:40	HLA04106		1	0	28	1	0	0	3	33			WD					WD	WD
B*18:43	18:43	HLA04212		0	0	8	0	0	0	0	8			WD					WD	WD
**B*18:44 total**	**18:44 total**			**0**	**0**	**17**	**1**	**0**	**0**	**0**	**18**			**WD**					**WD**	**WD**
B*18:44	18:44			0	0	2	0	0	0	0	2									
B*18:44:01	18:44:01	HLA04222		0	0	15	1	0	0	0	16			WD					WD	WD
B*18:45	18:45	HLA04223		0	0	3	1	0	0	0	4									
B*18:47	18:47	HLA04510		0	0	2	0	0	0	1	3									
B*18:49	18:49	HLA04805		0	1	6	0	0	0	0	7			WD					WD	WD
B*18:50	18:50	HLA05125		0	3	0	0	0	0	1	4									
B*18:52	18:52	HLA05508		0	0	1	0	0	0	0	1									
B*18:54	18:54	HLA06135		0	0	3	0	0	0	1	4									
B*18:60	18:60	HLA06322		0	0	6	0	0	0	1	7			WD					WD	WD
B*18:63	18:63	HLA06660		0	0	0	1	0	0	0	1									
B*18:65	18:65	HLA06927		0	0	1	0	0	0	0	1									
B*18:67	18:67	HLA07189		0	0	2	0	0	0	0	2									
B*18:68	18:68	HLA07303		8	0	3	0	0	0	2	13	WD							WD	WD
B*18:69	18:69	HLA07632		0	0	0	7	0	0	0	7				WD				WD	WD
**B*18:72 total**	**18:72 total**			**0**	**0**	**0**	**0**	**2**	**0**	**0**	**2**									
B*18:72:02	18:72:02	HLA14824		0	0	0	0	1	0	0	1									

Supplemental Table 9: HLA-B Allele Summary [a]				Allele Count by Population Group [b]								3.0.0 CIWD Category by Population Group [c]								
**Allele**	**Genomic typing**	**Allele ID**	**G group**	**AFA**	**API**	**EURO**	**MENA**	**HIS**	**NAM**	**UNK**	**Total**	**AFA**	**API**	**EURO**	**MENA**	**HIS**	**NAM**	**UNK**	**Total**	**Highest Frequency**
B*18:72:03	18:72:03	HLA16329		0	0	0	0	1	0	0	1									
B*18:73	18:73	HLA07905		0	0	3	3	0	0	0	6								WD	WD
B*18:75	18:75	HLA07907		0	0	1	0	0	0	0	1									
B*18:76	18:76	HLA07908		0	0	1	0	0	0	0	1									
B*18:79	18:79	HLA08392		0	0	0	0	0	0	3	3									
B*18:82	18:82	HLA08506		0	0	1	0	0	0	0	1									
B*18:85	18:85	HLA08982		1	0	0	0	0	0	0	1									
B*18:87	18:87	HLA09345		0	0	1	0	0	0	0	1									
B*18:91	18:91	HLA09556		0	0	1	0	0	0	0	1									
B*18:92	18:92	HLA09885		0	0	2	0	0	0	0	2									
B*18:93	18:93	HLA09889		0	1	0	0	0	0	0	1									
B*18:94N	18:94N	HLA09890		0	0	3	0	0	0	0	3									
B*18:95	18:95	HLA10403		0	0	3	0	0	1	1	5								WD	WD
B*18:98	18:98	HLA11007		4	0	0	0	0	0	0	4									
B*18:102	18:102	HLA11419		0	1	0	0	0	0	1	2									
B*18:104	18:104	HLA11651		0	0	1	0	0	0	0	1									
B*18:106	18:106	HLA12661		0	0	1	0	0	0	0	1									
B*18:111	18:111	HLA13653		0	0	1	0	0	0	0	1									
B*18:113	18:113	HLA13709		0	0	4	0	0	0	0	4									
B*18:114	18:114	HLA13794		0	6	0	0	0	0	0	6		WD						WD	WD
B*18:116	18:116	HLA13796		0	2	0	0	0	0	0	2									
B*18:117	18:117	HLA14434		0	0	1	0	0	0	0	1									
B*18:CODE	18:CODE			1075	682	46618	720	2940	211	4819	57065	NA	NA	NA	NA	NA	NA	NA	NA	NA
B*27:01	27:01	HLA00220		0	0	267	0	2	1	10	280			I				WD	I	I
**B*27:02 total**	**27:02 total**			**193**	**726**	**96061**	**2557**	**1465**	**156**	**4952**	**106110**	**C**	**C**	**C**	**C**	**C**	**C**	**C**	**C**	**C**
B*27:02	27:02			19	38	11373	365	219	24	980	13018	WD	I	C	C	C	C	C	C	C
B*27:02P	27:02P			0	0	16	1	0	0	0	17			WD					WD	WD
**B*27:02:01G total**	**27:02:01G total**			**174**	**688**	**84671**	**2191**	**1246**	**132**	**3972**	**93074**	**C**	**C**	**C**	**C**	**C**	**C**	**C**	**C**	**C**
B*27:02:01G	27:02:01G		27:02:01G	17	115	11607	210	62	4	261	12276	WD	I	C	C	I		C	C	C
B*27:02:01	27:02:01		27:02:01G	155	572	72862	1981	1176	128	3696	80570	C	C	C	C	C	C	C	C	C
B*27:02:01:01	27:02:01:01	HLA00221	27:02:01G	2	1	201	0	8	0	15	227			I		I		I	I	I

Supplemental Table 9: HLA-B Allele Summary [a]				Allele Count by Population Group [b]								3.0.0 CIWD Category by Population Group [c]								
Allele	Genomic typing	Allele ID	G group	AFA	API	EURO	MENA	HIS	NAM	UNK	Total	AFA	API	EURO	MENA	HIS	NAM	UNK	Total	Highest Frequency
B*27:02:01:02	27:02:01:02	HLA16229	27:02:01G	0	0	1	0	0	0	0	1									
B*27:02:02	27:02:02	HLA06955		0	0	1	0	0	0	0	1									
B*27:03	27:03	HLA00222		1386	29	1432	410	432	40	507	4236	C	I	C	C	C	C	C	C	C
**B*27:04 total**	**27:04 total**			**6**	**5768**	**183**	**108**	**18**	**10**	**453**	**6546**	**WD**	**C**	**I**	**C**	**I**	**C**	**C**	**C**	**C**
B*27:04	27:04			0	0	9	0	0	0	0	9			WD					WD	WD
B*27:04P	27:04P			0	0	3	0	0	0	0	3									
**B*27:04:01G total**	**27:04:01G total**			**5**	**5743**	**170**	**107**	**18**	**10**	**452**	**6505**	**WD**	**C**	**I**	**C**	**I**	**C**	**C**	**C**	**C**
B*27:04:01G	27:04:01G		27:04:01G	4	4680	159	93	16	8	319	5279		C	I	C	I	C	C	C	C
B*27:04:01	27:04:01	HLA00223	27:04:01G	1	1063	11	14	2	2	133	1226		C	WD	WD			C	I	C
B*27:04:03	27:04:03	HLA03987		1	24	1	1	0	0	1	28		I						WD	I
B*27:112	27:112	HLA10699		0	1	0	0	0	0	0	1									
**B*27:05 total**	**27:05 total**			**2726**	**9086**	**412750**	**4256**	**12367**	**1554**	**33072**	**475811**	**C**	**C**	**C**	**C**	**C**	**C**	**C**	**C**	**C**
B*27:05	27:05			1	1	1468	0	9	0	48	1527			C		I		I	I	C
B*27:05P	27:05P			1	0	204	0	1	0	1	207			I					I	I
**B*27:05:02G total**	**27:05:02G total**			**2722**	**9066**	**402809**	**4243**	**12331**	**1554**	**32735**	**465460**	**C**	**C**	**C**	**C**	**C**	**C**	**C**	**C**	**C**
B*27:05:02G	27:05:02G		27:05:02G	2240	8359	385618	4162	9387	1229	30452	441447	C	C	C	C	C	C	C	C	C
B*27:05:02	27:05:02		27:05:02G	458	676	16026	78	2755	313	2146	22452	C	C	C	C	C	C	C	C	C
B*27:05:02:01	27:05:02:01	HLA00225	27:05:02G	23	31	1113	3	184	12	132	1498	WD	I	I		C	C	I	I	C
B*27:05:02:02	27:05:02:02	HLA16809	27:05:02G	0	0	1	0	0	0	0	1									
B*27:05:02:05	27:05:02:05	HLA17223	27:05:02G	0	0	1	0	0	0	0	1									
B*27:05:04	27:05:04	HLA01179	27:05:02G	0	0	22	0	1	0	2	25			WD					WD	WD
B*27:13	27:13	HLA00234	27:05:02G	1	0	28	0	4	0	3	36			WD					WD	WD
B*27:05:03	27:05:03	HLA00226		2	15	8080	13	11	0	277	8398		I	C	WD	I		C	C	C
B*27:05:05	27:05:05	HLA01642		0	0	8	0	13	0	3	24			WD		I			WD	I
B*27:05:07	27:05:07	HLA01985		0	0	50	0	2	0	3	55			WD					WD	WD
B*27:05:08	27:05:08	HLA02100		0	0	8	0	0	0	0	8			WD					WD	WD
B*27:05:09	27:05:09	HLA02218		0	0	43	0	0	0	0	43			WD					WD	WD
B*27:05:10	27:05:10	HLA02826		0	0	8	0	0	0	1	9			WD					WD	WD
B*27:05:15	27:05:15	HLA05477		0	0	2	0	0	0	0	2									
B*27:05:18	27:05:18	HLA07634		0	0	42	0	0	0	3	45			WD					WD	WD
B*27:05:20	27:05:20	HLA08661		0	0	1	0	0	0	0	1									

Supplemental Table 9: HLA-B Allele Summary [a]				Allele Count by Population Group [b]								3.0.0 CIWD Category by Population Group [c]								
Allele	Genomic typing	Allele ID	G group	AFA	API	EURO	MENA	HIS	NAM	UNK	Total	AFA	API	EURO	MENA	HIS	NAM	UNK	Total	Highest Frequency
B*27:05:21	27:05:21	HLA08979		0	0	10	0	0	0	0	10			WD					WD	WD
B*27:05:22	27:05:22	HLA08980		0	0	3	0	0	0	0	3									
B*27:05:23	27:05:23	HLA09882		0	0	5	0	0	0	1	6			WD					WD	WD
B*27:05:24	27:05:24	HLA09884		0	0	2	0	0	0	0	2									
B*27:05:25	27:05:25	HLA10198		0	0	1	0	0	0	0	1									
B*27:05:26	27:05:26	HLA10269		0	0	3	0	0	0	0	3									
B*27:05:27	27:05:27	HLA11005		0	0	3	0	0	0	0	3									
B*27:05:30	27:05:30	HLA12266		0	4	0	0	0	0	0	4									
B*27:06	27:06	HLA00227		81	2287	111	13	35	4	449	2980	C	C	WD	WD	I		C	C	C
**B*27:07 total**	**27:07 total**			**70**	**4384**	**4690**	**1079**	**263**	**22**	**1194**	**11702**	**C**	**C**	**C**	**C**	**C**	**C**	**C**	**C**	**C**
B*27:07	27:07			43	407	1605	324	117	13	862	3371	C	C	C	C	C	C	C	C	C
B*27:07P	27:07P			0	0	2	1	0	0	0	3									
**B*27:07:01G total**	**27:07:01G total**			**27**	**3973**	**3083**	**754**	**146**	**9**	**332**	**8324**	**WD**	**C**	**C**	**C**	**C**	**C**	**C**	**C**	**C**
B*27:07:01G	27:07:01G		27:07:01G	20	3722	2858	718	64	1	290	7673	WD	C	C	C	I		C	C	C
B*27:07:01	27:07:01	HLA00228	27:07:01G	7	251	225	36	82	8	42	651	WD	C	I	WD	C	C	I	I	C
B*27:07:03	27:07:03	HLA08329		0	2	0	0	0	0	0	2									
B*27:07:04	27:07:04	HLA09883		0	2	0	0	0	0	0	2									
B*27:08	27:08	HLA00229		6	4	396	20	71	5	113	615	WD		I	WD	C	WD	I	I	C
B*27:09	27:09	HLA00230		4	17	1609	32	24	1	57	1744		I	C	WD	I		I	C	C
B*27:10	27:10	HLA00231		10	1	572	1	11	1	55	651	WD		I		I		I	I	I
B*27:11	27:11	HLA00232		0	2	0	0	0	0	0	2									
**B*27:12 total**	**27:12 total**			**15**	**2**	**1058**	**167**	**77**	**0**	**225**	**1544**	**WD**		**I**	**C**	**C**		**C**	**I**	**C**
**B*27:12:01G total**	**27:12:01G total**			**15**	**2**	**1058**	**167**	**77**	**0**	**225**	**1544**	**WD**		**I**	**C**	**C**		**C**	**I**	**C**
B*27:12	27:12		27:12:01G	15	2	1058	167	77	0	225	1544	WD		I	C	C		C	I	C
B*27:14	27:14	HLA00235		10	25	1464	9	17	4	87	1616	WD	I	C	WD	I		I	C	C
B*27:15	27:15	HLA00236		0	19	0	0	0	0	3	22		I						WD	I
B*27:16	27:16	HLA01056		0	0	1	0	0	0	0	1									
B*27:17	27:17	HLA01130		6	0	34	0	3	0	9	52	WD		WD				WD	WD	WD
B*27:18	27:18	HLA01143		0	7	0	0	0	0	0	7		WD						WD	WD
B*27:19	27:19	HLA01147		0	0	25	22	1	0	2	50			WD	WD				WD	WD
B*27:20	27:20	HLA01173		0	15	1	0	0	0	3	19		I						WD	I

Supplemental Table 9: HLA-B Allele Summary [a]				Allele Count by Population Group [b]								3.0.0 CIWD Category by Population Group [c]								
Allele	Genomic typing	Allele ID	G group	AFA	API	EURO	MENA	HIS	NAM	UNK	Total	AFA	API	EURO	MENA	HIS	NAM	UNK	Total	Highest Frequency
B*27:23	27:23	HLA01348		0	0	4	0	0	0	1	5								WD	WD
B*27:24	27:24	HLA01504		0	11	0	0	0	0	1	12		WD						WD	WD
B*27:25	27:25	HLA01529		0	8	7	2	0	0	0	17		WD	WD					WD	WD
B*27:26	27:26	HLA01952		15	0	3	3	0	0	1	22	WD							WD	WD
B*27:27	27:27	HLA02023		0	1	1	0	14	0	3	19					I			WD	I
B*27:30	27:30	HLA02238		1	0	25	2	2	0	4	34			WD					WD	WD
B*27:32	27:32	HLA02370		0	0	6	0	0	0	0	6			WD					WD	WD
B*27:34	27:34	HLA02473		0	0	11	0	0	0	0	11			WD					WD	WD
B*27:35	27:35	HLA02499		0	0	6	0	0	0	8	14			WD				WD	WD	WD
B*27:36	27:36	HLA02513		0	3	1	0	0	0	0	4									
B*27:37	27:37	HLA02784		0	0	1	0	0	0	0	1									
B*27:39	27:39	HLA03176		0	0	0	0	2	0	1	3									
B*27:41	27:41	HLA03276		0	0	2	0	0	0	10	12							WD	WD	WD
B*27:42	27:42	HLA03278		0	1	5	0	0	0	9	15			WD				WD	WD	WD
B*27:43	27:43	HLA03323		0	9	0	0	0	0	0	9		WD						WD	WD
B*27:45	27:45	HLA03554		0	0	1	0	0	1	0	2									
B*27:46	27:46	HLA03616		0	0	3	1	0	0	1	5								WD	WD
B*27:47	27:47	HLA03659		0	0	15	0	0	0	0	15			WD					WD	WD
B*27:48	27:48	HLA03661		0	0	7	0	0	0	0	7			WD					WD	WD
B*27:49	27:49	HLA03677		0	0	6	0	0	0	0	6			WD					WD	WD
**B*27:50 total**	**27:50 total**			**0**	**0**	**1**	**0**	**0**	**0**	**1**	**2**									
B*27:50	27:50			0	0	0	0	0	0	1	1									
B*27:50:02	27:50:02	HLA12563		0	0	1	0	0	0	0	1									
B*27:51	27:51	HLA03810		0	0	128	0	0	0	0	128			I					WD	I
B*27:52	27:52	HLA03824		0	0	2	0	0	0	0	2									
B*27:53	27:53	HLA03952		0	0	10	2	0	0	0	12			WD					WD	WD
B*27:54	27:54	HLA03955		1	0	0	0	0	0	0	1									
B*27:56	27:56	HLA04053		0	0	11	0	0	0	1	12			WD					WD	WD
B*27:58	27:58	HLA04175		0	0	1	0	0	0	0	1									
B*27:60	27:60	HLA04443		0	0	11	0	0	0	0	11			WD					WD	WD
B*27:61	27:61	HLA04449		0	9	0	3	0	0	1	13		WD						WD	WD

Supplemental Table 9: HLA-B Allele Summary [a]				Allele Count by Population Group [b]								3.0.0 CIWD Category by Population Group [c]								
Allele	Genomic typing	Allele ID	G group	AFA	API	EURO	MENA	HIS	NAM	UNK	Total	AFA	API	EURO	MENA	HIS	NAM	UNK	Total	Highest Frequency
B*27:65N	27:65N	HLA04771		0	0	2	0	0	0	0	2									
B*27:70	27:70	HLA05473		0	0	15	2	0	0	1	18			WD					WD	WD
B*27:72	27:72	HLA05504		0	0	2	0	0	0	0	2									
B*27:73	27:73	HLA05517		0	0	7	0	0	0	0	7			WD					WD	WD
B*27:74	27:74	HLA06183		0	0	1	0	0	0	0	1									
B*27:75	27:75	HLA06212		0	0	0	0	1	0	0	1									
B*27:76	27:76	HLA06230		0	0	1	0	0	0	0	1									
B*27:78	27:78	HLA06256		0	0	3	0	0	0	0	3									
B*27:80	27:80	HLA06353		0	0	3	0	1	0	1	5								WD	WD
B*27:81	27:81	HLA06940		0	0	1	0	0	0	0	1									
B*27:82	27:82	HLA06993		1	0	15	0	0	0	0	16			WD					WD	WD
B*27:83	27:83	HLA07187		0	0	9	0	0	0	0	9			WD					WD	WD
B*27:84	27:84	HLA07188		0	0	3	0	0	0	2	5								WD	WD
B*27:85	27:85	HLA07177		0	1	109	0	0	0	0	110			WD					WD	WD
B*27:86	27:86	HLA07339		0	1	0	0	0	0	0	1									
B*27:88	27:88	HLA07631		0	0	2	0	0	0	0	2									
**B*27:90 total**	**27:90 total**			**0**	**0**	**9**	**0**	**0**	**0**	**0**	**9**			**WD**					**WD**	**WD**
B*27:90	27:90			0	0	1	0	0	0	0	1									
B*27:90:01	27:90:01	HLA07902		0	0	3	0	0	0	0	3									
B*27:90:02	27:90:02	HLA08505		0	0	4	0	0	0	0	4									
B*27:90:03	27:90:03	HLA11819		0	0	1	0	0	0	0	1									
B*27:91	27:91	HLA07903		0	1	0	0	0	0	2	3									
B*27:94N	27:94N	HLA08330		0	1	0	0	0	0	0	1									
B*27:95	27:95	HLA08331		0	0	1	0	0	0	0	1									
B*27:99	27:99	HLA08662		0	0	1	0	0	0	0	1									
B*27:101	27:101	HLA09308		0	0	3	0	0	0	0	3									
B*27:104	27:104	HLA09426		0	0	3	0	1	0	0	4									
B*27:108	27:108	HLA10402		0	1	0	0	0	0	0	1									
B*27:118	27:118	HLA10906		0	0	3	0	0	0	0	3									
B*27:119	27:119	HLA11002		0	0	0	0	1	0	0	1									
B*27:120	27:120	HLA11003		0	1	0	0	0	0	0	1									

Supplemental Table 9: HLA-B Allele Summary [a]				Allele Count by Population Group [b]								3.0.0 CIWD Category by Population Group [c]								
Allele	Genomic typing	Allele ID	G group	AFA	API	EURO	MENA	HIS	NAM	UNK	Total	AFA	API	EURO	MENA	HIS	NAM	UNK	Total	Highest Frequency
B*27:122	27:122	HLA11006		0	0	1	0	0	0	1	2									
B*27:123	27:123	HLA11225		0	0	1	0	0	0	0	1									
B*27:127	27:127	HLA11649		0	0	1	0	0	0	0	1									
B*27:128	27:128	HLA11729		0	0	1	0	0	0	0	1									
B*27:130	27:130	HLA12037		0	3	0	0	0	0	1	4									
B*27:132	27:132	HLA12473		0	0	2	0	0	0	0	2									
B*27:133	27:133	HLA12441		0	0	2	0	0	0	0	2									
B*27:134	27:134	HLA12658		0	0	0	0	0	0	1	1									
B*27:135	27:135	HLA12659		0	0	0	0	0	1	0	1									
B*27:137	27:137	HLA12980		0	0	1	0	0	0	0	1									
B*27:139	27:139	HLA13465		1	0	0	0	0	0	0	1									
B*27:140	27:140	HLA13466		0	0	1	0	0	0	0	1									
B*27:141	27:141	HLA13651		0	0	1	0	0	0	0	1									
B*27:142	27:142	HLA13741		0	0	3	0	0	0	0	3									
B*27:148	27:148	HLA14925		0	0	1	0	0	0	0	1									
B*27:151	27:151	HLA14978		0	0	0	0	0	0	2	2									
B*27:165	27:165	HLA18037		0	1	0	0	0	0	0	1									
B*27:CODE	27:CODE			244	646	36826	252	1116	139	2409	41632	NA	NA	NA	NA	NA	NA	NA	NA	NA
**B*35:01 total**	**35:01 total**			**23840**	**61380**	**628096**	**24407**	**38398**	**3937**	**69383**	**849441**	**C**	**C**	**C**	**C**	**C**	**C**	**C**	**C**	**C**
B*35:01	35:01			7	2	3732	9	15	0	144	3909	WD		C	WD	I		C	C	C
B*35:01P	35:01P			1	5	1296	2	3	0	5	1312		WD	C				WD	I	C
**B*35:01:01G total**	**35:01:01G total**			**23821**	**61372**	**622871**	**24383**	**38377**	**3937**	**69210**	**843971**	**C**	**C**	**C**	**C**	**C**	**C**	**C**	**C**	**C**
B*35:01:01G	35:01:01G		35:01:01G	19667	57534	596537	23848	29211	3051	64271	794119	C	C	C	C	C	C	C	C	C
B*35:01:01	35:01:01		35:01:01G	4152	3834	26139	532	9149	886	4928	49620	C	C	C	C	C	C	C	C	C
B*35:01:01:01	35:01:01:01	HLA00237	35:01:01G	0	0	6	0	0	0	0	6			WD					WD	WD
B*35:01:01:02	35:01:01:02	HLA05354	35:01:01G	0	0	38	0	0	0	4	42			WD					WD	WD
B*35:01:01:05	35:01:01:05	HLA16276	35:01:01G	0	0	113	0	0	0	0	113			WD					WD	WD
B*35:01:01:06	35:01:01:06	HLA16278	35:01:01G	0	0	5	0	0	0	0	5			WD					WD	WD
B*35:01:01:07	35:01:01:07	HLA17063	35:01:01G	0	0	0	0	0	0	1	1									
B*35:01:23	35:01:23	HLA06139	35:01:01G	0	3	0	0	0	0	0	3									
B*35:01:41	35:01:41	HLA10884	35:01:01G	0	1	0	1	0	0	0	2									

Supplemental Table 9: HLA-B Allele Summary [a]				Allele Count by Population Group [b]								3.0.0 CIWD Category by Population Group [c]								
Allele	Genomic typing	Allele ID	G group	AFA	API	EURO	MENA	HIS	NAM	UNK	Total	AFA	API	EURO	MENA	HIS	NAM	UNK	Total	Highest Frequency
B*35:42:01	35:42:01	HLA01578	35:01:01G	0	0	5	2	1	0	0	8			WD					WD	WD
B*35:57	35:57	HLA02091	35:01:01G	0	0	25	0	0	0	2	27			WD					WD	WD
B*35:241	35:241	HLA09923	35:01:01G	0	0	2	0	14	0	2	18					I			WD	I
B*35:250	35:250	HLA10886	35:01:01G	2	0	0	0	0	0	1	3									
B*35:332	35:332	HLA16354	35:01:01G	0	0	1	0	2	0	1	4									
B*35:01:02	35:01:02	HLA01264		1	0	18	0	0	0	2	21			WD					WD	WD
B*35:01:04	35:01:04	HLA02176		5	0	1	0	0	0	1	7	WD							WD	WD
B*35:01:05	35:01:05	HLA02313		0	0	110	0	0	0	17	127			WD				I	WD	I
B*35:01:06	35:01:06	HLA02566		0	0	7	0	0	0	2	9			WD					WD	WD
B*35:01:07	35:01:07	HLA03288		0	0	13	0	0	0	0	13			WD					WD	WD
B*35:01:08	35:01:08	HLA03796		0	0	5	0	0	0	0	5			WD					WD	WD
B*35:01:09	35:01:09	HLA03963		0	0	1	0	2	0	0	3									
B*35:01:10	35:01:10	HLA04114		0	0	0	1	0	0	0	1									
B*35:01:11	35:01:11	HLA04179		0	0	4	11	0	0	1	16				WD				WD	WD
B*35:01:14	35:01:14	HLA04434		0	0	1	0	0	0	0	1									
B*35:01:17	35:01:17	HLA04474		0	0	0	1	0	0	0	1									
B*35:01:19	35:01:19	HLA04544		0	0	17	0	0	0	1	18			WD					WD	WD
B*35:01:26	35:01:26	HLA06651		0	0	1	0	0	0	0	1									
B*35:01:27	35:01:27	HLA06689		5	0	0	0	0	0	0	5	WD							WD	WD
B*35:01:29	35:01:29	HLA07003		0	0	11	0	0	0	0	11			WD					WD	WD
B*35:01:31	35:01:31	HLA07704		0	0	1	0	0	0	0	1									
B*35:01:34	35:01:34	HLA08357		0	0	3	0	0	0	0	3									
B*35:01:38	35:01:38	HLA09571		0	0	1	0	0	0	0	1									
B*35:01:42	35:01:42	HLA11659		0	0	0	0	1	0	0	1									
B*35:01:44	35:01:44	HLA12886		0	1	0	0	0	0	0	1									
**B*35:42 total**	**35:42 total**			**0**	**0**	**8**	**2**	**1**	**0**	**0**	**11**			**WD**					**WD**	**WD**
B*35:42:02	35:42:02	HLA04675		0	0	3	0	0	0	0	3									
**B*35:02 total**	**35:02 total**			**663**	**3666**	**117316**	**10736**	**8816**	**743**	**23651**	**165591**	**C**	**C**	**C**	**C**	**C**	**C**	**C**	**C**	**C**
B*35:02	35:02			8	35	5753	443	227	15	514	6995	WD	I	C	C	C	C	C	C	C
B*35:02P	35:02P			0	0	387	1	1	0	3	392			I					I	I
**B*35:02:01G total**	**35:02:01G total**			**655**	**3631**	**110640**	**10288**	**8585**	**728**	**23128**	**157655**	**C**	**C**	**C**	**C**	**C**	**C**	**C**	**C**	**C**

Supplemental Table 9: HLA-B Allele Summary [a]				Allele Count by Population Group [b]								3.0.0 CIWD Category by Population Group [c]								
Allele	Genomic typing	Allele ID	G group	AFA	API	EURO	MENA	HIS	NAM	UNK	Total	AFA	API	EURO	MENA	HIS	NAM	UNK	Total	Highest Frequency
B*35:02:01G	35:02:01G		35:02:01G	405	2686	87353	8618	4701	384	18023	122170	C	C	C	C	C	C	C	C	C
B*35:02:01	35:02:01		35:02:01G	250	945	23256	1670	3884	344	5105	35454	C	C	C	C	C	C	C	C	C
B*35:02:01:01	35:02:01:01	HLA00238	35:02:01G	0	0	1	0	0	0	0	1									
B*35:02:01:02	35:02:01:02	HLA16279	35:02:01G	0	0	30	0	0	0	0	30			WD					WD	WD
B*35:02:02	35:02:02	HLA02143		0	0	535	4	2	0	6	547			I				WD	I	I
B*35:02:03	35:02:03	HLA03476		0	0	1	0	1	0	0	2									
**B*35:03 total**	**35:03 total**			**1160**	**68091**	**258324**	**16431**	**10384**	**1097**	**22334**	**377821**	**C**	**C**	**C**	**C**	**C**	**C**	**C**	**C**	**C**
B*35:03	35:03			1	9	1897	7	5	0	73	1992		WD	C	WD	WD		I	C	C
B*35:03P	35:03P			0	2	480	2	0	0	0	484			I					I	I
**B*35:03:01G total**	**35:03:01G total**			**1159**	**68069**	**255934**	**16420**	**10379**	**1097**	**22260**	**375318**	**C**	**C**	**C**	**C**	**C**	**C**	**C**	**C**	**C**
B*35:03:01G	35:03:01G		35:03:01G	919	64102	246031	16049	7319	785	20513	355718	C	C	C	C	C	C	C	C	C
B*35:03:01	35:03:01		35:03:01G	219	3786	9010	355	2877	293	1621	18161	C	C	C	C	C	C	C	C	C
B*35:03:01:01	35:03:01:01	HLA00239	35:03:01G	7	141	234	6	68	7	54	517	WD	C	I	WD	I	C	I	I	C
B*35:03:01:02	35:03:01:02	HLA15764	35:03:01G	0	0	0	0	0	0	1	1									
B*35:03:01:03	35:03:01:03	HLA16280	35:03:01G	14	38	643	10	113	11	71	900	WD	I	I	WD	C	C	I	I	C
B*35:03:01:05	35:03:01:05	HLA16812	35:03:01G	0	0	16	0	2	0	0	18			WD					WD	WD
B*35:279	35:279	HLA12877	35:03:01G	0	2	0	0	0	1	0	3									
B*35:03:02	35:03:02	HLA03607		0	0	5	0	0	0	1	6			WD					WD	WD
B*35:03:03	35:03:03	HLA04437		0	1	1	0	0	0	0	2									
B*35:03:05	35:03:05	HLA05491		0	0	1	1	0	0	0	2									
B*35:03:07	35:03:07	HLA05841		0	0	1	0	0	0	0	1									
B*35:03:08	35:03:08	HLA06148		0	0	1	1	0	0	0	2									
B*35:03:09	35:03:09	HLA06220		0	2	1	0	0	0	0	3									
B*35:03:10	35:03:10	HLA06961		0	5	0	0	0	0	0	5		WD						WD	WD
B*35:03:11	35:03:11	HLA07705		0	2	0	0	0	0	0	2									
B*35:03:12	35:03:12	HLA08677		0	0	1	0	0	0	0	1									
B*35:03:15	35:03:15	HLA11828		0	0	1	0	0	0	0	1									
B*35:03:20	35:03:20	HLA12997		0	0	1	0	0	0	0	1									
B*35:03:21	35:03:21	HLA13025		0	1	0	0	0	0	0	1									
**B*35:04 total**	**35:04 total**			**53**	**10**	**425**	**2**	**1378**	**149**	**697**	**2714**	**C**	**WD**	**I**		**C**	**C**	**C**	**C**	**C**
B*35:04	35:04			5	0	137	0	253	8	130	533	WD		I		C	C	I	I	C

Supplemental Table 9: HLA-B Allele Summary [a]				Allele Count by Population Group [b]								3.0.0 CIWD Category by Population Group [c]								
Allele	Genomic typing	Allele ID	G group	AFA	API	EURO	MENA	HIS	NAM	UNK	Total	AFA	API	EURO	MENA	HIS	NAM	UNK	Total	Highest Frequency
B*35:04P	35:04P			0	0	0	0	1	0	0	1									
B*35:04:01	35:04:01	HLA00240		48	9	280	2	1043	136	540	2058	C	WD	I		C	C	C	C	C
B*35:04:02	35:04:02	HLA02260		0	1	8	0	81	5	27	122			WD		C	WD	I	WD	C
**B*35:05 total**	**35:05 total**			**192**	**7838**	**1137**	**71**	**2448**	**158**	**3213**	**15057**	**C**	**C**	**I**	**C**	**C**	**C**	**C**	**C**	**C**
B*35:05	35:05			10	438	209	0	363	12	283	1315	WD	C	I		C	C	C	I	C
**B*35:05:01G total**	**35:05:01G total**			**182**	**7396**	**928**	**71**	**2085**	**146**	**2930**	**13738**	**C**	**C**	**I**	**C**	**C**	**C**	**C**	**C**	**C**
B*35:05:01G	35:05:01G		35:05:01G	7	933	174	0	392	0	461	1967	WD	C	I		C		C	C	C
B*35:05:01	35:05:01		35:05:01G	175	6410	750	71	1667	144	2446	11663	C	C	I	C	C	C	C	C	C
B*35:05:01:01	35:05:01:01	HLA00241	35:05:01G	0	52	0	0	2	0	18	72		I					I	WD	I
B*35:05:01:02	35:05:01:02	HLA16388	35:05:01G	0	1	4	0	20	1	5	31					I		WD	WD	I
B*35:05:01:03	35:05:01:03	HLA16813	35:05:01G	0	0	0	0	4	1	0	5								WD	WD
B*35:05:02	35:05:02	HLA04820		0	4	0	0	0	0	0	4									
B*35:06	35:06	HLA00242		6	2	99	0	273	29	175	584	WD		WD		C	C	C	I	C
**B*35:08 total**	**35:08 total**			**480**	**2156**	**63661**	**9691**	**4601**	**252**	**10528**	**91369**	**C**	**C**	**C**	**C**	**C**	**C**	**C**	**C**	**C**
B*35:08	35:08			24	34	3767	530	490	14	371	5230	WD	I	C	C	C	C	C	C	C
B*35:08P	35:08P			0	0	57	0	0	0	0	57			WD					WD	WD
**B*35:08:01G total**	**35:08:01G total**			**456**	**2122**	**59814**	**9158**	**4109**	**238**	**10156**	**86053**	**C**	**C**	**C**	**C**	**C**	**C**	**C**	**C**	**C**
B*35:08:01G	35:08:01G		35:08:01G	6	1052	3733	325	48	2	193	5359	WD	C	C	C	I		C	C	C
B*35:08:01	35:08:01		35:08:01G	447	1064	55916	8813	4009	234	9937	80420	C	C	C	C	C	C	C	C	C
B*35:08:01:01	35:08:01:01	HLA00244	35:08:01G	3	6	165	20	52	2	26	274		WD	I	WD	I		I	I	I
B*35:08:02	35:08:02	HLA02506		0	0	15	0	1	0	1	17			WD					WD	WD
B*35:08:03	35:08:03	HLA03104		0	0	2	0	1	0	0	3									
B*35:08:05	35:08:05	HLA07335		0	0	2	0	0	0	0	2									
B*35:08:07	35:08:07	HLA11033		0	0	4	0	0	0	0	4									
B*35:08:08	35:08:08	HLA12482		0	0	0	3	0	0	0	3									
**B*35:09 total**	**35:09 total**			**4**	**10**	**508**	**1**	**791**	**17**	**731**	**2062**		**WD**	**I**		**C**	**C**	**C**	**C**	**C**
B*35:09	35:09			0	0	146	0	137	1	122	406			I		C		I	I	C
B*35:09:01	35:09:01	HLA00245		4	10	355	1	631	16	596	1613		WD	I		C	C	C	C	C
B*35:09:02	35:09:02	HLA00246		0	0	7	0	19	0	12	38			WD		I		WD	WD	I
B*35:09:03	35:09:03	HLA07178		0	0	0	0	4	0	1	5								WD	WD
B*35:10	35:10	HLA00247		7	12	61	0	310	24	120	534	WD	WD	WD		C	C	I	I	C

Supplemental Table 9: HLA-B Allele Summary [a]				Allele Count by Population Group [b]								3.0.0 CIWD Category by Population Group [c]								
Allele	Genomic typing	Allele ID	G group	AFA	API	EURO	MENA	HIS	NAM	UNK	Total	AFA	API	EURO	MENA	HIS	NAM	UNK	Total	Highest Frequency
**B*35:11 total**	**35:11 total**			**33**	**10**	**53**	**1**	**196**	**36**	**68**	**397**	**WD**	**WD**	**WD**		**C**	**C**	**I**	**I**	**C**
B*35:11	35:11			3	0	12	0	27	0	8	50			WD		I		WD	WD	I
B*35:11:01	35:11:01	HLA00248		30	9	35	1	166	36	60	337	WD	WD	WD		C	C	I	I	C
B*35:11:02	35:11:02	HLA05273		0	1	4	0	0	0	0	5								WD	WD
B*35:11:03	35:11:03	HLA08393		0	0	2	0	3	0	0	5								WD	WD
**B*35:12 total**	**35:12 total**			**170**	**111**	**756**	**25**	**12232**	**844**	**1776**	**15914**	**C**	**I**	**I**	**WD**	**C**	**C**	**C**	**C**	**C**
B*35:12	35:12			11	5	102	2	1587	100	148	1955	WD	WD	WD		C	C	C	C	C
B*35:12:01	35:12:01	HLA00249		159	105	653	23	10640	744	1628	13952	C	I	I	WD	C	C	C	C	C
B*35:12:02	35:12:02	HLA04183		0	1	0	0	0	0	0	1									
B*35:12:03	35:12:03	HLA06647		0	0	0	0	5	0	0	5					WD			WD	WD
B*35:12:04	35:12:04	HLA16931		0	0	1	0	0	0	0	1									
B*35:13	35:13	HLA00250		0	84	3	6	1	0	1	95		I		WD				WD	I
**B*35:14 total**	**35:14 total**			**11**	**19**	**149**	**11**	**1885**	**106**	**267**	**2448**	**WD**	**I**	**I**	**WD**	**C**	**C**	**C**	**C**	**C**
B*35:14	35:14			1	2	12	1	218	9	9	252			WD		C	C	WD	I	C
B*35:14:01	35:14:01	HLA00251		10	17	86	4	1667	97	252	2133	WD	I	WD		C	C	C	C	C
B*35:14:02	35:14:02	HLA01741		0	0	51	6	0	0	6	63			WD	WD			WD	WD	WD
**B*35:15 total**	**35:15 total**			**0**	**17**	**12**	**0**	**12**	**2**	**6**	**49**		**I**	**WD**		**I**		**WD**	**WD**	**I**
B*35:15	35:15			0	6	4	0	6	1	1	18		WD			WD			WD	WD
B*35:15:01	35:15:01	HLA00252		0	11	5	0	6	1	5	28		WD	WD		WD		WD	WD	WD
B*35:15:02	35:15:02	HLA12238		0	0	3	0	0	0	0	3									
B*35:16	35:16	HLA00253		9	15	61	4	1113	46	140	1388	WD	I	WD		C	C	C	I	C
**B*35:17 total**	**35:17 total**			**82**	**92**	**566**	**33**	**9169**	**518**	**1284**	**11744**	**C**	**I**	**I**	**WD**	**C**	**C**	**C**	**C**	**C**
B*35:17	35:17			37	44	393	10	3568	262	651	4965	WD	I	I	WD	C	C	C	C	C
B*35:17:01	35:17:01	HLA00254		45	48	173	23	5598	256	633	6776	C	I	I	WD	C	C	C	C	C
B*35:17:02	35:17:02	HLA10411		0	0	0	0	3	0	0	3									
B*35:18	35:18	HLA00255		0	0	16	0	14	0	20	50			WD		I		I	WD	I
B*35:19	35:19	HLA00256		0	0	501	0	575	7	753	1836			I		C	C	C	C	C
**B*35:20 total**	**35:20 total**			**34**	**16**	**337**	**1**	**1145**	**72**	**496**	**2101**	**WD**	**I**	**I**		**C**	**C**	**C**	**C**	**C**
B*35:20	35:20			3	1	90	0	173	10	60	337			WD		C	C	I	I	C
B*35:20:01	35:20:01	HLA00257		31	15	206	1	971	62	425	1711	WD	I	I		C	C	C	C	C
B*35:20:02	35:20:02	HLA03014		0	0	41	0	1	0	11	53			WD				WD	WD	WD

Supplemental Table 9: HLA-B Allele Summary [a]				Allele Count by Population Group [b]								3.0.0 CIWD Category by Population Group [c]								
Allele	Genomic typing	Allele ID	G group	AFA	API	EURO	MENA	HIS	NAM	UNK	Total	AFA	API	EURO	MENA	HIS	NAM	UNK	Total	Highest Frequency
B*35:21	35:21	HLA00258		3	7	93	2	363	11	167	646		WD	WD		C	C	C	I	C
B*35:22	35:22	HLA00259		5	4	43	2	231	12	77	374	WD		WD		C	C	I	I	C
B*35:23	35:23	HLA00260		3	19	7	1	235	18	34	317		I	WD		C	C	I	I	C
**B*35:24 total**	**35:24 total**			**3**	**2**	**8**	**0**	**189**	**10**	**21**	**233**			**WD**		**C**	**C**	**I**	**I**	**C**
B*35:24	35:24			0	0	0	0	29	1	2	32					I			WD	I
B*35:24:01	35:24:01	HLA00261		2	2	8	0	152	8	18	190			WD		C	C	I	I	C
B*35:24:02	35:24:02	HLA04497		1	0	0	0	8	1	1	11					I			WD	I
B*35:25	35:25	HLA00262		0	0	5	0	0	0	0	5			WD					WD	WD
B*35:26	35:26	HLA00263		0	1	0	0	23	3	2	29					I			WD	I
B*35:27	35:27	HLA00264		3	1	125	1	4	1	15	150			I				I	WD	I
B*35:28	35:28	HLA00981		3	2	24	2	268	11	39	349			WD		C	C	I	I	C
**B*35:29 total**	**35:29 total**			**3**	**2**	**181**	**0**	**1**	**0**	**6**	**193**			**I**				**WD**	**I**	**I**
B*35:29	35:29			3	0	22	0	0	0	1	26			WD					WD	WD
B*35:29:01	35:29:01	HLA00982		0	2	158	0	1	0	5	166			I				WD	I	I
B*35:29:02	35:29:02	HLA07454		0	0	1	0	0	0	0	1									
B*35:30	35:30	HLA01057		1	261	38	3	137	8	66	514		C	WD		C	C	I	I	C
B*35:31	35:31	HLA01058		1	3	29	1	160	9	33	236			WD		C	C	I	I	C
**B*35:32 total**	**35:32 total**			**6**	**0**	**82**	**0**	**4**	**0**	**10**	**102**	**WD**		**WD**				**WD**	**WD**	**WD**
B*35:32	35:32			2	0	15	0	1	0	1	19			WD					WD	WD
B*35:32:01	35:32:01	HLA01059		4	0	67	0	3	0	9	83			WD				WD	WD	WD
B*35:33	35:33	HLA01060		0	0	5	0	0	0	0	5			WD					WD	WD
B*35:34	35:34	HLA01194		1	0	18	3	20	7	8	57			WD		I	C	WD	WD	C
B*35:36	35:36	HLA01296		0	1	132	27	0	0	0	160			I	WD				WD	I
B*35:37	35:37	HLA01314		0	8	31	0	0	0	1	40		WD	WD					WD	WD
B*35:38	35:38	HLA01503		0	0	46	8	0	0	2	56			WD	WD				WD	WD
B*35:39	35:39	HLA01509		0	0	0	0	2	0	0	2									
B*35:41	35:41	HLA01563		16	3	758	1	14	5	119	916	WD		I		I	WD	I	I	I
**B*35:43 total**	**35:43 total**			**122**	**46**	**557**	**8**	**4968**	**364**	**977**	**7042**	**C**	**I**	**I**	**WD**	**C**	**C**	**C**	**C**	**C**
**B*35:43:01G total**	**35:43:01G total**			**122**	**46**	**557**	**8**	**4968**	**364**	**977**	**7042**	**C**	**I**	**I**	**WD**	**C**	**C**	**C**	**C**	**C**
B*35:43	35:43		35:43:01G	0	0	8	0	9	0	1	18			WD		I			WD	I
B*35:43P	35:43P			0	0	1	0	0	0	0	1									

Supplemental Table 9: HLA-B Allele Summary [a]				Allele Count by Population Group [b]								3.0.0 CIWD Category by Population Group [c]								
Allele	Genomic typing	Allele ID	G group	AFA	API	EURO	MENA	HIS	NAM	UNK	Total	AFA	API	EURO	MENA	HIS	NAM	UNK	Total	Highest Frequency
B*35:43:01G	35:43:01G		35:43:01G	84	30	515	5	3586	239	819	5278	C	I	I	WD	C	C	C	C	C
B*35:43:01	35:43:01	HLA00185	35:43:01G	38	16	33	3	1369	125	157	1741	WD	I	WD		C	C	C	C	C
B*35:79	35:79	HLA03008	35:43:01G	0	0	0	0	4	0	0	4									
B*35:44	35:44	HLA01182		1	0	2	0	28	0	3	34					I			WD	I
B*35:45	35:45	HLA01668		0	0	25	0	0	0	0	25			WD					WD	WD
B*35:46	35:46	HLA01727		0	0	1	0	0	0	0	1									
B*35:47	35:47	HLA01846		1	0	4	0	20	3	3	31					I			WD	I
B*35:48	35:48	HLA01850		0	1	2	0	40	8	5	56					I	C	WD	WD	C
B*35:49	35:49	HLA01888		6	0	2	0	32	2	8	50	WD				I		WD	WD	I
B*35:50	35:50	HLA01896		0	0	1	0	0	0	0	1									
B*35:51	35:51	HLA01897		0	1	1	0	0	0	1	3									
B*35:55	35:55	HLA02031		1	0	120	0	22	3	58	204			I		I		I	I	I
B*35:58	35:58	HLA02128		0	0	0	0	3	0	2	5								WD	WD
**B*35:59 total**	**35:59 total**			**6**	**0**	**1**	**0**	**5**	**3**	**5**	**20**	**WD**				**WD**		**WD**	**WD**	**WD**
B*35:59	35:59			4	0	1	0	4	3	4	16								WD	WD
B*35:59:01	35:59:01	HLA02134		2	0	0	0	1	0	1	4									
**B*35:61 total**	**35:61 total**			**1**	**0**	**9**	**0**	**0**	**0**	**0**	**10**			**WD**					**WD**	**WD**
B*35:61	35:61			0	0	5	0	0	0	0	5			WD					WD	WD
B*35:61:01	35:61:01	HLA02251		0	0	4	0	0	0	0	4									
B*35:61:02	35:61:02	HLA08782		1	0	0	0	0	0	0	1									
B*35:62	35:62	HLA02385		0	0	0	0	0	0	2	2									
B*35:63	35:63	HLA02399		1	0	1	0	0	0	0	2									
**B*35:64 total**	**35:64 total**			**0**	**0**	**1**	**0**	**0**	**0**	**0**	**1**									
B*35:64	35:64			0	0	1	0	0	0	0	1									
B*35:65Q	35:65Q	HLA02411		0	0	46	0	0	0	0	46			WD					WD	WD
**B*35:68 total**	**35:68 total**			**2**	**1**	**23**	**2**	**38**	**2**	**4**	**72**			**WD**		**I**			**WD**	**I**
B*35:68	35:68			0	0	2	0	8	0	0	10					I			WD	I
B*35:68:01	35:68:01	HLA02645		1	1	16	2	0	0	0	20			WD					WD	WD
B*35:68:02	35:68:02	HLA03262		1	0	5	0	30	2	4	42			WD		I			WD	I
B*35:72	35:72	HLA02763		0	7	2	0	0	0	0	9		WD						WD	WD
B*35:74	35:74	HLA02810		0	4	3	0	0	0	0	7								WD	WD

Supplemental Table 9: HLA-B Allele Summary [a]				Allele Count by Population Group [b]								3.0.0 CIWD Category by Population Group [c]								
Allele	Genomic typing	Allele ID	G group	AFA	API	EURO	MENA	HIS	NAM	UNK	Total	AFA	API	EURO	MENA	HIS	NAM	UNK	Total	Highest Frequency
B*35:77	35:77	HLA02977		0	1	103	2	3	0	21	130			WD				I	WD	I
B*35:78	35:78	HLA03003		0	0	3	0	0	0	0	3									
B*35:80	35:80	HLA03078		0	0	7	0	1	0	3	11			WD					WD	WD
B*35:81	35:81	HLA03084		0	0	11	0	0	0	1	12			WD					WD	WD
B*35:82	35:82	HLA03125		0	0	0	0	1	0	1	2									
B*35:84	35:84	HLA03149		0	1	0	0	0	0	0	1									
B*35:85	35:85	HLA03169		0	1	0	0	0	0	0	1									
B*35:86	35:86	HLA03197		0	0	0	0	1	0	0	1									
B*35:87	35:87	HLA03198		0	0	5	0	0	0	2	7			WD					WD	WD
B*35:90	35:90	HLA03321		0	0	4	0	3	0	2	9								WD	WD
B*35:91	35:91	HLA03324		0	1	0	0	0	0	0	1									
B*35:92	35:92	HLA03335		0	0	2	0	0	0	0	2									
B*35:93	35:93	HLA03336		0	1	12	0	0	0	0	13			WD					WD	WD
B*35:95	35:95	HLA03625		0	0	12	0	1	0	0	13			WD					WD	WD
B*35:96	35:96	HLA03662		0	0	5	0	0	0	0	5			WD					WD	WD
B*35:97	35:97	HLA03644		0	1	0	0	0	0	0	1									
B*35:98	35:98	HLA03722		0	0	1	0	0	0	0	1									
B*35:99	35:99	HLA03715		0	0	1	0	2	0	0	3									
B*35:100	35:100	HLA04900		0	0	23	0	1	0	1	25			WD					WD	WD
**B*35:101 total**	**35:101 total**			**0**	**0**	**1**	**0**	**0**	**0**	**0**	**1**									
B*35:101:01	35:101:01	HLA04904		0	0	1	0	0	0	0	1									
B*35:102	35:102	HLA04864		1	0	0	0	1	0	0	2									
B*35:103	35:103	HLA04907		1	0	2	0	2	4	1	10								WD	WD
B*35:104	35:104	HLA04910		0	0	10	0	0	0	0	10			WD					WD	WD
B*35:105	35:105	HLA04911		0	0	0	4	0	0	0	4									
B*35:106	35:106	HLA04913		0	2	0	6	0	0	0	8				WD				WD	WD
**B*35:108 total**	**35:108 total**			**0**	**0**	**3**	**0**	**4**	**1**	**3**	**11**								**WD**	**WD**
B*35:108:01	35:108:01	HLA04916		0	0	3	0	2	0	2	7								WD	WD
B*35:108:02	35:108:02	HLA04921		0	0	0	0	2	1	1	4									
B*35:109	35:109	HLA04919		0	26	2	0	0	0	0	28		I						WD	I
B*35:110	35:110	HLA05063		0	0	1	0	0	0	0	1									

Supplemental Table 9: HLA-B Allele Summary [a]				Allele Count by Population Group [b]								3.0.0 CIWD Category by Population Group [c]								
Allele	Genomic typing	Allele ID	G group	AFA	API	EURO	MENA	HIS	NAM	UNK	Total	AFA	API	EURO	MENA	HIS	NAM	UNK	Total	Highest Frequency
B*35:111	35:111	HLA04922		0	1	0	0	0	0	0	1									
B*35:112	35:112	HLA04923		0	0	6	0	0	0	0	6			WD					WD	WD
B*35:113	35:113	HLA04882		0	0	0	0	2	0	2	4									
B*35:114	35:114	HLA04932		0	2	0	0	0	0	2	4									
B*35:115	35:115	HLA04936		0	0	7	1	0	0	1	9			WD					WD	WD
B*35:116	35:116	HLA04938		1	0	4	0	23	3	13	44					I		WD	WD	I
B*35:117	35:117	HLA04941		0	0	0	0	1	0	0	1									
B*35:119	35:119	HLA04946		1	0	2	0	0	0	0	3									
B*35:120	35:120	HLA04960		0	0	2	0	0	0	0	2									
B*35:121	35:121	HLA04961		0	0	0	0	17	3	2	22					I			WD	I
B*35:122	35:122	HLA04962		0	0	1	0	1	0	1	3									
B*35:123	35:123	HLA04963		0	0	1	0	4	0	0	5								WD	WD
B*35:124	35:124	HLA04965		0	0	1	0	0	0	1	2									
B*35:128	35:128	HLA04992		0	1	0	3	0	0	1	5								WD	WD
B*35:130N	35:130N	HLA04999		0	0	0	1	0	0	0	1									
B*35:132	35:132	HLA04867		0	37	0	0	0	0	2	39		I						WD	I
B*35:136	35:136	HLA04888		0	3	7	0	0	0	0	10			WD					WD	WD
**B*35:137 total**	**35:137 total**			**0**	**2**	**1**	**0**	**0**	**0**	**0**	**3**									
**B*35:137:01G total**	**35:137:01G total**			**0**	**2**	**1**	**0**	**0**	**0**	**0**	**3**									
B*35:137:01G	35:137:01G		35:137:01G	0	2	1	0	0	0	0	3									
B*35:138	35:138	HLA05047		0	0	2	0	0	0	1	3									
B*35:139	35:139	HLA05051		0	1	4	0	0	0	0	5								WD	WD
B*35:143	35:143	HLA05060		0	0	1	0	0	0	0	1									
B*35:144	35:144	HLA05061		0	0	2	0	0	0	0	2									
B*35:148	35:148	HLA05826		0	0	2	0	0	0	1	3									
B*35:149	35:149	HLA05828		0	0	0	0	3	1	0	4									
B*35:152	35:152	HLA05955		0	0	1	0	0	0	0	1									
B*35:155	35:155	HLA06057		0	0	1	0	0	0	0	1									
B*35:156	35:156	HLA06134		0	1	0	1	0	0	0	2									
B*35:157	35:157	HLA06239		0	0	5	0	0	0	0	5			WD					WD	WD
B*35:158	35:158	HLA06261		0	0	27	8	0	0	1	36			WD	WD				WD	WD

Supplemental Table 9: HLA-B Allele Summary [a]				Allele Count by Population Group [b]								3.0.0 CIWD Category by Population Group [c]								
Allele	Genomic typing	Allele ID	G group	AFA	API	EURO	MENA	HIS	NAM	UNK	Total	AFA	API	EURO	MENA	HIS	NAM	UNK	Total	Highest Frequency
B*35:159	35:159	HLA06266		0	1	2	0	1	0	0	4									
B*35:162	35:162	HLA06574		0	0	1	0	0	0	0	1									
B*35:163	35:163	HLA06648		0	0	5	0	0	0	0	5			WD					WD	WD
B*35:165N	35:165N	HLA06685		0	0	3	0	4	0	0	7								WD	WD
B*35:167	35:167	HLA06815		0	11	5	0	0	0	0	16		WD	WD					WD	WD
B*35:169	35:169	HLA06929		0	0	2	0	0	0	0	2									
B*35:170	35:170	HLA06935		0	1	1	0	0	0	0	2									
B*35:171	35:171	HLA06945		0	0	0	0	1	0	0	1									
B*35:174	35:174	HLA06962		0	0	1	17	0	0	0	18				WD				WD	WD
B*35:175	35:175	HLA06964		0	0	0	0	1	0	1	2									
B*35:178	35:178	HLA06999		0	1	4	0	2	0	2	9								WD	WD
B*35:180	35:180	HLA07197		0	0	4	0	0	0	0	4									
B*35:182	35:182	HLA07202		0	0	1	0	0	0	0	1									
B*35:183	35:183	HLA07212		0	0	5	0	0	0	0	5			WD					WD	WD
B*35:184	35:184	HLA07302		0	0	2	0	0	0	0	2									
B*35:189	35:189	HLA07560		0	1	0	0	0	0	0	1									
B*35:190	35:190	HLA07645		0	0	0	0	1	0	0	1									
B*35:191	35:191	HLA07646		0	0	1	0	0	0	6	7							WD	WD	WD
B*35:192	35:192	HLA07651		0	0	2	0	0	0	0	2									
B*35:193	35:193	HLA07692		0	0	0	6	0	0	0	6				WD				WD	WD
B*35:194	35:194	HLA07701		0	0	3	0	0	0	0	3									
B*35:198	35:198	HLA07769		0	0	1	0	0	0	0	1									
B*35:199	35:199	HLA07780		0	0	1	0	0	0	0	1									
B*35:200	35:200	HLA07796		0	0	1	0	0	0	1	2									
B*35:203	35:203	HLA07939		0	0	3	0	0	0	0	3									
B*35:204	35:204	HLA08062		0	0	12	0	0	0	0	12			WD					WD	WD
B*35:206	35:206	HLA08108		0	0	1	0	0	0	1	2									
B*35:208	35:208	HLA08319		0	1	1	0	0	0	0	2									
B*35:210	35:210	HLA08354		0	0	4	0	0	0	0	4									
B*35:211	35:211	HLA08356		0	0	2	0	0	0	0	2									
B*35:212	35:212	HLA08397		0	0	1	0	0	0	0	1									

Supplemental Table 9: HLA-B Allele Summary [a]				Allele Count by Population Group [b]								3.0.0 CIWD Category by Population Group [c]								
Allele	Genomic typing	Allele ID	G group	AFA	API	EURO	MENA	HIS	NAM	UNK	Total	AFA	API	EURO	MENA	HIS	NAM	UNK	Total	Highest Frequency
B*35:214	35:214	HLA08360		0	0	9	0	0	0	1	10			WD					WD	WD
B*35:215	35:215	HLA08361		0	2	0	0	0	0	0	2									
B*35:216N	35:216N	HLA08362		0	0	1	0	1	0	0	2									
B*35:217	35:217	HLA08499		0	0	1	0	0	0	0	1									
B*35:218	35:218	HLA08507		0	0	3	0	0	0	0	3									
B*35:219	35:219	HLA08785		0	0	0	1	1	0	0	2									
B*35:221	35:221	HLA08990		0	0	3	0	0	0	1	4									
B*35:225	35:225	HLA09003		0	0	1	1	1	0	3	6								WD	WD
B*35:228	35:228	HLA09435		0	0	1	0	0	0	0	1									
B*35:232	35:232	HLA09709		0	0	1	0	0	0	0	1									
B*35:233	35:233	HLA09697		0	0	0	0	1	0	0	1									
B*35:234	35:234	HLA09897		0	1	0	0	0	0	0	1									
B*35:235	35:235	HLA09898		0	2	0	1	0	0	0	3									
B*35:236	35:236	HLA09899		0	0	1	0	0	0	1	2									
B*35:237	35:237	HLA09900		0	1	0	0	0	0	0	1									
B*35:238	35:238	HLA09901		0	3	0	0	0	0	0	3									
B*35:239	35:239	HLA09912		0	1	0	0	0	0	0	1									
B*35:240	35:240	HLA09922		0	0	1	0	3	0	0	4									
B*35:242	35:242	HLA10409		0	0	1	0	0	0	0	1									
B*35:243	35:243	HLA10410		0	2	0	0	0	0	0	2									
B*35:247	35:247	HLA10508		0	0	10	0	1	0	5	16			WD				WD	WD	WD
B*35:248	35:248	HLA10516		0	0	2	0	0	0	1	3									
B*35:252	35:252	HLA11027		0	0	1	0	0	0	0	1									
B*35:253	35:253	HLA11326		0	0	0	0	0	0	1	1									
B*35:257	35:257	HLA11826		0	0	1	0	0	0	0	1									
B*35:259	35:259	HLA12008		0	0	3	0	0	0	2	5								WD	WD
B*35:260	35:260	HLA12131		0	0	1	0	0	0	0	1									
B*35:262	35:262	HLA12279		0	3	0	0	0	0	0	3									
B*35:263	35:263	HLA12316		0	0	5	0	0	0	0	5			WD					WD	WD
B*35:265	35:265	HLA12428		0	0	0	0	1	0	0	1									
B*35:266	35:266	HLA12479		0	0	0	0	1	0	0	1									

Supplemental Table 9: HLA-B Allele Summary [a]				Allele Count by Population Group [b]								3.0.0 CIWD Category by Population Group [c]								
**Allele**	**Genomic typing**	**Allele ID**	**G group**	**AFA**	**API**	**EURO**	**MENA**	**HIS**	**NAM**	**UNK**	**Total**	**AFA**	**API**	**EURO**	**MENA**	**HIS**	**NAM**	**UNK**	**Total**	**Highest Frequency**
B*35:268	35:268	HLA12565		0	0	1	0	0	0	0	1									
B*35:269	35:269	HLA12487		0	0	1	0	0	0	0	1									
B*35:274	35:274	HLA12747		0	0	3	0	1	0	0	4									
B*35:277	35:277	HLA12751		0	0	0	0	1	0	0	1									
B*35:278	35:278	HLA12757		0	0	8	0	0	0	0	8			WD					WD	WD
B*35:280	35:280	HLA12976		0	2	0	0	0	0	0	2									
B*35:281	35:281	HLA12996		0	1	0	0	0	0	0	1									
B*35:282	35:282	HLA12998		0	0	1	0	0	0	0	1									
B*35:285	35:285	HLA13345		0	0	2	0	0	0	0	2									
B*35:287	35:287	HLA13476		0	0	0	0	0	1	0	1									
B*35:289	35:289	HLA13538		0	0	1	0	0	0	0	1									
B*35:294	35:294	HLA13657		0	0	2	0	0	0	0	2									
B*35:295	35:295	HLA13658		0	0	1	0	0	0	0	1									
B*35:306	35:306	HLA14201		0	0	0	2	0	0	0	2									
B*35:310	35:310	HLA14438		0	3	0	0	0	0	0	3									
B*35:311	35:311	HLA14443		0	0	2	0	0	0	0	2									
B*35:313	35:313	HLA14684		0	0	3	0	0	0	0	3									
B*35:315	35:315	HLA14931		0	1	0	0	0	0	0	1									
B*35:316	35:316	HLA14992		0	0	2	0	0	0	0	2									
B*35:317	35:317	HLA15262		0	1	0	0	0	0	0	1									
B*35:320	35:320	HLA15281		0	1	0	0	0	0	0	1									
B*35:321	35:321	HLA15292		0	0	6	0	2	0	13	21			WD				WD	WD	WD
B*35:324	35:324	HLA15549		0	0	0	0	0	0	1	1									
B*35:326	35:326	HLA15558		0	1	0	0	0	0	0	1									
B*35:337	35:337	HLA16888		0	0	0	0	0	0	1	1									
B*35:CODE	35:CODE			2275	2621	84017	2352	6628	530	8783	107206	NA	NA	NA	NA	NA	NA	NA	NA	NA
**B*37:01 total**	**37:01 total**			**1997**	**36050**	**152001**	**4148**	**5157**	**543**	**15048**	**214944**	**C**	**C**	**C**	**C**	**C**	**C**	**C**	**C**	**C**
B*37:01	37:01			1	4	711	2	2	0	24	744			I				I	I	I
B*37:01P	37:01P			0	2	210	1	1	0	1	215			I					I	I
**B*37:01:01G total**	**37:01:01G total**			**1995**	**35984**	**150984**	**4143**	**5154**	**541**	**15017**	**213818**	**C**	**C**	**C**	**C**	**C**	**C**	**C**	**C**	**C**
B*37:01:01G	37:01:01G		37:01:01G	1553	32682	140364	3959	3719	382	13442	196101	C	C	C	C	C	C	C	C	C

Supplemental Table 9: HLA-B Allele Summary [a]				Allele Count by Population Group [b]								3.0.0 CIWD Category by Population Group [c]								
Allele	Genomic typing	Allele ID	G group	AFA	API	EURO	MENA	HIS	NAM	UNK	Total	AFA	API	EURO	MENA	HIS	NAM	UNK	Total	Highest Frequency
B*37:01:01	37:01:01		37:01:01G	442	3302	10617	184	1435	159	1575	17714	C	C	C	C	C	C	C	C	C
B*37:01:01:01	37:01:01:01	HLA00265	37:01:01G	0	0	3	0	0	0	0	3									
B*37:01:02	37:01:02	HLA02418		0	0	5	0	0	0	0	5			WD					WD	WD
B*37:01:03	37:01:03	HLA02505		0	57	61	2	0	2	3	125		I	WD					WD	I
B*37:01:06	37:01:06	HLA03908		1	0	18	0	0	0	3	22			WD					WD	WD
B*37:01:07	37:01:07	HLA04184		0	0	6	0	0	0	0	6			WD					WD	WD
B*37:01:08	37:01:08	HLA06994		0	0	2	0	0	0	0	2									
B*37:01:09	37:01:09	HLA08333		0	1	4	0	0	0	0	5								WD	WD
B*37:01:11	37:01:11	HLA13467		0	2	0	0	0	0	0	2									
B*37:02	37:02	HLA00266		0	4	284	122	4	0	220	634			I	C			C	I	C
B*37:03N	37:03N	HLA01267		0	2	21	2	2	0	2	29			WD					WD	WD
**B*37:04 total**	**37:04 total**			**0**	**162**	**194**	**15**	**4**	**1**	**61**	**437**		**C**	**I**	**WD**			**I**	**I**	**C**
B*37:04	37:04			0	7	21	0	0	0	2	30		WD	WD					WD	WD
B*37:04:01	37:04:01	HLA01346		0	63	173	15	4	1	56	312		I	I	WD			I	I	I
B*37:04:02	37:04:02	HLA04738		0	92	0	0	0	0	3	95		I						WD	I
B*37:05	37:05	HLA01359		0	10	2	0	0	0	0	12		WD						WD	WD
**B*37:06 total**	**37:06 total**			**0**	**0**	**4**	**0**	**0**	**0**	**0**	**4**									
B*37:06	37:06			0	0	2	0	0	0	0	2									
B*37:06:01	37:06:01	HLA01949		0	0	2	0	0	0	0	2									
B*37:07	37:07	HLA02007		0	1	28	0	1	0	2	32			WD					WD	WD
B*37:09	37:09	HLA02149		0	0	4	2	0	0	0	6								WD	WD
B*37:10	37:10	HLA02231		0	0	7	0	1	0	0	8			WD					WD	WD
B*37:11	37:11	HLA02479		0	3	1	0	0	0	0	4									
B*37:16Q	37:16Q	HLA04073		0	0	5	0	0	0	0	5			WD					WD	WD
B*37:17	37:17	HLA04208		0	0	20	0	1	0	1	22			WD					WD	WD
B*37:18	37:18	HLA04229		0	1	3	0	0	0	1	5								WD	WD
**B*37:19 total**	**37:19 total**			**0**	**1**	**1**	**0**	**0**	**0**	**0**	**2**									
B*37:19	37:19			0	0	1	0	0	0	0	1									
B*37:19:02	37:19:02	HLA13967		0	1	0	0	0	0	0	1									
B*37:21	37:21	HLA04539		0	0	14	0	0	0	0	14			WD					WD	WD
B*37:22	37:22	HLA04692		0	5	0	0	0	0	0	5		WD						WD	WD

Supplemental Table 9: HLA-B Allele Summary [a]				Allele Count by Population Group [b]								3.0.0 CIWD Category by Population Group [c]								
**Allele**	**Genomic typing**	**Allele ID**	**G group**	**AFA**	**API**	**EURO**	**MENA**	**HIS**	**NAM**	**UNK**	**Total**	**AFA**	**API**	**EURO**	**MENA**	**HIS**	**NAM**	**UNK**	**Total**	**Highest Frequency**
B*37:24	37:24	HLA05487		0	0	4	0	0	0	0	4									
B*37:25	37:25	HLA05719		0	1	0	0	0	0	0	1									
B*37:27	37:27	HLA06231		0	0	4	0	0	0	1	5								WD	WD
B*37:33N	37:33N	HLA07690		0	0	2	0	0	0	0	2									
B*37:34	37:34	HLA07931		0	2	0	0	0	0	0	2									
B*37:35	37:35	HLA08334		0	9	0	0	0	0	0	9		WD						WD	WD
B*37:39	37:39	HLA09887		0	1	0	0	0	0	0	1									
B*37:42N	37:42N	HLA10124		0	0	5	0	0	0	1	6			WD					WD	WD
B*37:44	37:44	HLA10647		0	0	1	0	0	0	0	1									
B*37:48	37:48	HLA11228		0	7	0	0	0	0	0	7		WD						WD	WD
B*37:49	37:49	HLA11420		0	2	0	0	0	0	0	2									
B*37:52	37:52	HLA12175		0	1	3	0	0	0	0	4									
B*37:54	37:54	HLA12660		0	4	0	0	0	0	0	4									
B*37:65	37:65	HLA16473		0	4	0	0	0	0	0	4									
B*37:CODE	37:CODE			79	281	5617	71	263	20	564	6895	NA	NA	NA	NA	NA	NA	NA	NA	NA
**B*38:01 total**	**38:01 total**			**1247**	**3179**	**282636**	**15893**	**12745**	**1004**	**40830**	**357534**	**C**	**C**	**C**	**C**	**C**	**C**	**C**	**C**	**C**
B*38:01	38:01			62	112	13986	855	1295	77	1241	17628	C	I	C	C	C	C	C	C	C
B*38:01P	38:01P			0	0	143	2	0	0	0	145			I					WD	I
**B*38:01:01G total**	**38:01:01G total**			**1185**	**3067**	**268482**	**15029**	**11449**	**927**	**39581**	**339720**	**C**	**C**	**C**	**C**	**C**	**C**	**C**	**C**	**C**
B*38:01:01G	38:01:01G		38:01:01G	20	92	14120	505	150	2	208	15097	WD	I	C	C	C		C	C	C
B*38:01:01	38:01:01		38:01:01G	1165	2975	254360	14524	11299	925	39373	324621	C	C	C	C	C	C	C	C	C
B*38:01:01:01	38:01:01:01	HLA00267	38:01:01G	0	0	2	0	0	0	0	2									
B*38:01:02	38:01:02	HLA02477		0	0	2	0	0	0	0	2									
B*38:01:03	38:01:03	HLA04090		0	0	2	0	0	0	0	2									
B*38:01:04	38:01:04	HLA04217		0	0	3	6	1	0	7	17				WD			WD	WD	WD
B*38:01:05	38:01:05	HLA05962		0	0	0	0	0	0	1	1									
B*38:01:06	38:01:06	HLA07471		0	0	16	0	0	0	0	16			WD					WD	WD
B*38:01:07	38:01:07	HLA07727		0	0	0	1	0	0	0	1									
B*38:01:08	38:01:08	HLA13349		0	0	2	0	0	0	0	2									
**B*38:02 total**	**38:02 total**			**44**	**23374**	**713**	**137**	**178**	**34**	**3068**	**27548**	**C**	**C**	**I**	**C**	**C**	**C**	**C**	**C**	**C**
B*38:02	38:02			1	73	16	0	1	0	17	108		I	WD				I	WD	I

Supplemental Table 9: HLA-B Allele Summary [a]				Allele Count by Population Group [b]								3.0.0 CIWD Category by Population Group [c]								
Allele	Genomic typing	Allele ID	G group	AFA	API	EURO	MENA	HIS	NAM	UNK	Total	AFA	API	EURO	MENA	HIS	NAM	UNK	Total	Highest Frequency
B*38:02P	38:02P			0	0	1	0	0	0	0	1									
**B*38:02:01G total**	**38:02:01G total**			**42**	**22245**	**643**	**136**	**159**	**33**	**2716**	**25974**	**C**	**C**	**I**	**C**	**C**	**C**	**C**	**C**	**C**
B*38:02:01G	38:02:01G		38:02:01G	33	18183	614	122	114	23	1952	21041	WD	C	I	C	C	C	C	C	C
B*38:02:01	38:02:01	HLA00268	38:02:01G	9	4062	29	14	45	10	764	4933	WD	C	WD	WD	I	C	C	C	C
B*38:02:02	38:02:02	HLA00269		1	1056	53	1	18	1	335	1465		C	WD		I		C	I	C
B*38:03	38:03	HLA00270		0	2	8	0	9	0	3	22			WD		I			WD	I
B*38:04	38:04	HLA01137		0	2	1	0	0	0	1	4									
B*38:06	38:06	HLA01265		2	0	2	0	1	3	2	10								WD	WD
B*38:08	38:08	HLA01515		0	0	1	0	0	0	0	1									
B*38:09	38:09	HLA01655		1	2	388	2	5	0	151	549			I		WD		C	I	C
B*38:12	38:12	HLA02394		0	0	27	5	1	0	3	36			WD	WD				WD	WD
B*38:13	38:13	HLA02504		0	1	26	1	0	0	0	28			WD					WD	WD
B*38:14	38:14	HLA02584		0	1	11	0	0	0	17	29			WD				I	WD	I
B*38:15	38:15	HLA02714		0	11	0	0	0	0	2	13		WD						WD	WD
B*38:17	38:17	HLA03051		1	0	3	0	0	0	1	5								WD	WD
B*38:19	38:19	HLA03694		0	0	1	0	0	0	3	4									
B*38:24	38:24	HLA05519		0	0	11	0	1	0	16	28			WD				I	WD	I
B*38:25	38:25	HLA05712		0	0	1	0	0	0	0	1									
B*38:27	38:27	HLA06242		0	0	2	0	0	0	10	12							WD	WD	WD
B*38:28	38:28	HLA06316		0	0	1	0	0	0	0	1									
B*38:29	38:29	HLA06326		0	2	1	0	0	0	0	3									
B*38:31	38:31	HLA06692		0	0	22	0	0	0	1	23			WD					WD	WD
B*38:32	38:32	HLA06830		0	0	6	0	0	0	0	6			WD					WD	WD
B*38:33	38:33	HLA06834		0	0	2	0	0	0	0	2									
B*38:35	38:35	HLA06991		0	13	0	0	0	0	6	19		I					WD	WD	I
B*38:36	38:36	HLA07211		0	0	4	0	0	0	0	4									
B*38:37	38:37	HLA07310		0	0	5	0	0	0	4	9			WD					WD	WD
B*38:41	38:41	HLA08389		0	0	1	1	0	0	0	2									
B*38:44	38:44	HLA10050		0	0	1	0	0	0	0	1									
B*38:45	38:45	HLA10051		0	1	0	0	0	0	0	1									
B*38:47	38:47	HLA10478		0	2	0	0	0	0	0	2									

Supplemental Table 9: HLA-B Allele Summary [a]				Allele Count by Population Group [b]								3.0.0 CIWD Category by Population Group [c]								
Allele	Genomic typing	Allele ID	G group	AFA	API	EURO	MENA	HIS	NAM	UNK	Total	AFA	API	EURO	MENA	HIS	NAM	UNK	Total	Highest Frequency
B*38:48	38:48	HLA10637		0	1	0	0	0	0	0	1									
B*38:56	38:56	HLA12571		0	0	1	0	0	0	0	1									
B*38:60	38:60	HLA14603		0	0	1	0	0	0	0	1									
B*38:61	38:61	HLA14736		0	0	2	0	0	0	0	2									
B*38:64	38:64	HLA15645		0	4	0	0	0	0	0	4									
B*38:CODE	38:CODE			53	198	14406	365	1045	51	2333	18451	NA	NA	NA	NA	NA	NA	NA	NA	NA
**B*39:01 total**	**39:01 total**			**996**	**11462**	**152989**	**3093**	**5425**	**744**	**11962**	**186671**	**C**	**C**	**C**	**C**	**C**	**C**	**C**	**C**	**C**
B*39:01	39:01			0	12	1033	0	44	2	30	1121		WD	I		I		I	I	I
**B*39:01L total**	**39:01L total**			**0**	**0**	**1**	**1**	**0**	**0**	**0**	**2**									
B*39:01P	39:01P			0	0	295	1	0	0	0	296			I					I	I
**B*39:01:01G total**	**39:01:01G total**			**991**	**11447**	**151615**	**3089**	**5239**	**735**	**11906**	**185022**	**C**	**C**	**C**	**C**	**C**	**C**	**C**	**C**	**C**
B*39:01:01G	39:01:01G		39:01:01G	864	10786	147619	3071	4445	641	11302	178728	C	C	C	C	C	C	C	C	C
B*39:01:01	39:01:01		39:01:01G	109	501	3225	12	649	81	455	5032	C	C	C	WD	C	C	C	C	C
B*39:01:01:01	39:01:01:01	HLA00271	39:01:01G	1	0	34	0	1	0	7	43			WD				WD	WD	WD
B*39:01:01:02L	39:01:01:02L	HLA02220	39:01:01G	0	0	1	1	0	0	0	2									
B*39:01:01:03	39:01:01:03	HLA08053	39:01:01G	15	100	727	5	134	12	111	1104	WD	I	I	WD	C	C	I	I	C
B*39:01:01:04	39:01:01:04	HLA14074	39:01:01G	0	1	0	0	0	0	0	1									
B*39:01:01:05	39:01:01:05	HLA16282	39:01:01G	0	0	1	0	0	0	0	1									
B*39:01:01:06	39:01:01:06	HLA16854	39:01:01G	0	0	0	0	0	0	1	1									
B*39:01:01:07	39:01:01:07	HLA17074	39:01:01G	2	0	2	0	9	1	3	17					I			WD	I
B*39:01:03	39:01:03		39:01:01G	0	59	2	0	1	0	27	89		I					I	WD	I
B*39:01:24	39:01:24	HLA16355	39:01:01G	0	0	4	0	0	0	0	4									
B*39:01:04	39:01:04	HLA01123		5	3	14	2	122	6	22	174	WD		WD		C	WD	I	I	C
B*39:01:05	39:01:05	HLA03314		0	0	3	0	19	1	2	25					I			WD	I
B*39:01:07	39:01:07	HLA04041		0	0	4	0	0	0	0	4									
B*39:01:08	39:01:08	HLA04043		0	0	2	0	0	0	0	2									
B*39:01:09	39:01:09	HLA04050		0	0	4	0	0	0	0	4									
B*39:01:10	39:01:10	HLA04177		0	0	9	0	0	0	1	10			WD					WD	WD
B*39:01:12	39:01:12	HLA05514		0	0	5	0	0	0	0	5			WD					WD	WD
B*39:01:14	39:01:14	HLA08283		0	0	2	1	0	0	0	3									
B*39:01:19	39:01:19	HLA11452		0	0	2	0	1	0	1	4									

Supplemental Table 9: HLA-B Allele Summary [a]				Allele Count by Population Group [b]								3.0.0 CIWD Category by Population Group [c]								
Allele	Genomic typing	Allele ID	G group	AFA	API	EURO	MENA	HIS	NAM	UNK	Total	AFA	API	EURO	MENA	HIS	NAM	UNK	Total	Highest Frequency
B*39:01:20	39:01:20	HLA11739		0	0	1	0	0	0	0	1									
**B*39:02 total**	**39:02 total**			**73**	**66**	**192**	**1**	**3476**	**246**	**565**	**4619**	**C**	**I**	**I**		**C**	**C**	**C**	**C**	**C**
B*39:02	39:02			7	7	19	0	395	16	37	481	WD	WD	WD		C	C	I	I	C
B*39:02:01	39:02:01	HLA00274		0	23	1	0	4	0	27	55		I					I	WD	I
**B*39:02:02G total**	**39:02:02G total**			**66**	**36**	**172**	**1**	**3077**	**230**	**501**	**4083**	**C**	**I**	**I**		**C**	**C**	**C**	**C**	**C**
B*39:02:02G	39:02:02G		39:02:02G	2	0	11	0	75	0	31	119			WD		C		I	WD	C
B*39:02:02	39:02:02		39:02:02G	64	36	161	1	2917	227	464	3870	C	I	I		C	C	C	C	C
B*39:02:02:01	39:02:02:01	HLA00275	39:02:02G	0	0	0	0	85	3	6	94					C		WD	WD	C
**B*39:03 total**	**39:03 total**			**7**	**3**	**995**	**0**	**1233**	**20**	**1528**	**3786**	**WD**		**I**		**C**	**C**	**C**	**C**	**C**
**B*39:03:01G total**	**39:03:01G total**			**7**	**3**	**995**	**0**	**1233**	**20**	**1528**	**3786**	**WD**		**I**		**C**	**C**	**C**	**C**	**C**
B*39:03	39:03		39:03:01G	7	3	971	0	1159	20	1469	3629	WD		I		C	C	C	C	C
B*39:03:01G	39:03:01G		39:03:01G	0	0	24	0	73	0	57	154			WD		C		I	WD	C
B*39:03:01	39:03:01		39:03:01G	0	0	0	0	1	0	2	3									
B*39:04	39:04	HLA00277		0	38	4	0	1	0	17	60		I					I	WD	I
**B*39:05 total**	**39:05 total**			**313**	**668**	**2322**	**107**	**17617**	**1166**	**3418**	**25611**	**C**	**C**	**C**	**C**	**C**	**C**	**C**	**C**	**C**
B*39:05	39:05			27	35	303	9	1893	86	304	2657	WD	I	I	WD	C	C	C	C	C
**B*39:05:01G total**	**39:05:01G total**			**286**	**633**	**2019**	**98**	**15724**	**1079**	**3114**	**22953**	**C**	**C**	**C**	**C**	**C**	**C**	**C**	**C**	**C**
B*39:05:01G	39:05:01G		39:05:01G	0	3	0	0	0	0	0	3									
B*39:05:01	39:05:01		39:05:01G	286	630	2019	98	15724	1079	3114	22950	C	C	C	C	C	C	C	C	C
B*39:05:02	39:05:02	HLA04440		0	0	0	0	0	1	0	1									
**B*39:06 total**	**39:06 total**			**578**	**1196**	**59147**	**824**	**13048**	**877**	**7110**	**82780**	**C**	**C**	**C**	**C**	**C**	**C**	**C**	**C**	**C**
B*39:06	39:06			30	27	3177	58	1493	73	355	5213	WD	I	C	C	C	C	C	C	C
B*39:06P	39:06P			0	0	15	0	0	0	0	15			WD					WD	WD
B*39:06:01	39:06:01	HLA00279		21	4	83	1	366	45	127	647	WD		WD		C	C	I	I	C
**B*39:06:02G total**	**39:06:02G total**			**527**	**1165**	**55871**	**765**	**11189**	**759**	**6628**	**76904**	**C**	**C**	**C**	**C**	**C**	**C**	**C**	**C**	**C**
B*39:06:02G	39:06:02G		39:06:02G	17	743	6209	56	250	15	470	7760	WD	C	C	C	C	C	C	C	C
B*39:06:02	39:06:02		39:06:02G	482	401	49140	702	9591	663	6022	67001	C	C	C	C	C	C	C	C	C
B*39:06:02:01	39:06:02:01	HLA00280	39:06:02G	12	2	428	3	40	5	40	530	WD		I		I	WD	I	I	I
B*39:06:02:02	39:06:02:02	HLA16287	39:06:02G	13	10	26	2	1298	74	88	1511	WD	WD	WD		C	C	I	I	C
B*39:06:02:03	39:06:02:03	HLA16286	39:06:02G	3	9	68	2	10	2	8	102		WD	WD		I		WD	WD	I
B*39:06:03	39:06:03	HLA09593		0	0	1	0	0	0	0	1									

Supplemental Table 9: HLA-B Allele Summary [a]				Allele Count by Population Group [b]								3.0.0 CIWD Category by Population Group [c]								
Allele	Genomic typing	Allele ID	G group	AFA	API	EURO	MENA	HIS	NAM	UNK	Total	AFA	API	EURO	MENA	HIS	NAM	UNK	Total	Highest Frequency
B*39:07	39:07	HLA00281		0	1	3	0	35	2	9	50					I		WD	WD	I
B*39:08	39:08	HLA00282		35	26	117	4	2242	215	336	2975	WD	I	WD		C	C	C	C	C
**B*39:09 total**	**39:09 total**			**24**	**631**	**1073**	**0**	**1481**	**60**	**1454**	**4723**	**WD**	**C**	**I**		**C**	**C**	**C**	**C**	**C**
B*39:09	39:09			10	208	405	0	466	28	304	1421	WD	C	I		C	C	C	I	C
**B*39:09:01G total**	**39:09:01G total**			**14**	**423**	**668**	**0**	**1014**	**32**	**1150**	**3301**	**WD**	**C**	**I**		**C**	**C**	**C**	**C**	**C**
B*39:09:01G	39:09:01G		39:09:01G	0	21	45	0	78	0	52	196		I	WD		C		I	I	C
B*39:09:01	39:09:01		39:09:01G	14	398	623	0	931	31	1094	3091	WD	C	I		C	C	C	C	C
B*39:09:01:01	39:09:01:01	HLA00283	39:09:01G	0	4	0	0	4	1	3	12								WD	WD
B*39:09:01:02	39:09:01:02	HLA16753	39:09:01G	0	0	0	0	1	0	0	1									
B*39:09:01:03	39:09:01:03	HLA16852	39:09:01G	0	0	0	0	0	0	1	1									
B*39:09:03	39:09:03	HLA12762		0	0	0	0	1	0	0	1									
**B*39:10 total**	**39:10 total**			**3330**	**66**	**1235**	**856**	**352**	**51**	**984**	**6874**	**C**	**I**	**C**	**C**	**C**	**C**	**C**	**C**	**C**
B*39:10	39:10			406	5	181	48	60	2	140	842	C	WD	I	C	I		C	I	C
B*39:10P	39:10P			0	0	1	0	0	0	0	1									
B*39:10:01	39:10:01	HLA00284		2924	61	1053	808	292	49	844	6031	C	I	I	C	C	C	C	C	C
B*39:11	39:11	HLA00285		27	15	74	0	995	85	197	1393	WD	I	WD		C	C	C	I	C
B*39:12	39:12	HLA00286		3	0	39	0	107	11	90	250			WD		C	C	I	I	C
**B*39:13 total**	**39:13 total**			**18**	**1**	**110**	**0**	**320**	**30**	**179**	**658**	**WD**		**WD**		**C**	**C**	**C**	**I**	**C**
B*39:13	39:13			3	0	34	0	45	3	36	121			WD		I		I	WD	I
B*39:13:01	39:13:01	HLA00287		15	1	74	0	270	27	141	528	WD		WD		C	C	C	I	C
B*39:13:02	39:13:02	HLA02165		0	0	2	0	5	0	2	9					WD			WD	WD
**B*39:14 total**	**39:14 total**			**0**	**2**	**111**	**0**	**209**	**8**	**132**	**462**			**WD**		**C**	**C**	**I**	**I**	**C**
**B*39:14:01G total**	**39:14:01G total**			**0**	**2**	**111**	**0**	**209**	**8**	**132**	**462**			**WD**		**C**	**C**	**I**	**I**	**C**
B*39:14	39:14		39:14:01G	0	2	111	0	209	8	132	462			WD		C	C	I	I	C
B*39:15	39:15	HLA00289		3	235	25	5	3	0	60	331		C	WD	WD			I	I	C
**B*39:19 total**	**39:19 total**			**0**	**1**	**9**	**0**	**5**	**0**	**1**	**16**			**WD**		**WD**			**WD**	**WD**
B*39:19	39:19			0	1	1	0	1	0	0	3									
B*39:19:01	39:19:01	HLA01133		0	0	7	0	0	0	0	7			WD					WD	WD
B*39:19:02	39:19:02	HLA03275		0	0	1	0	4	0	1	6								WD	WD
B*39:20	39:20	HLA01183		17	2	47	0	126	4	26	222	WD		WD		C		I	I	C
B*39:22	39:22	HLA01196		1	0	2	0	25	2	7	37					I		WD	WD	I

Supplemental Table 9: HLA-B Allele Summary [a]				Allele Count by Population Group [b]								3.0.0 CIWD Category by Population Group [c]								
Allele	Genomic typing	Allele ID	G group	AFA	API	EURO	MENA	HIS	NAM	UNK	Total	AFA	API	EURO	MENA	HIS	NAM	UNK	Total	Highest Frequency
B*39:23	39:23	HLA01203		0	4	0	0	0	0	0	4									
**B*39:24 total**	**39:24 total**			**190**	**88**	**6398**	**813**	**242**	**16**	**702**	**8449**	**C**	**I**	**C**	**C**	**C**	**C**	**C**	**C**	**C**
B*39:24	39:24			14	4	550	39	54	1	51	713	WD		I	WD	I		I	I	I
B*39:24P	39:24P			0	0	3	0	0	0	0	3									
B*39:24:01	39:24:01	HLA01221		176	84	5844	774	188	15	650	7731	C	I	C	C	C	C	C	C	C
B*39:24:02	39:24:02	HLA06320		0	0	1	0	0	0	1	2									
B*39:26	39:26	HLA01528		0	0	18	0	0	0	3	21			WD					WD	WD
B*39:28	39:28	HLA01789		0	0	1	0	0	0	0	1									
B*39:29	39:29	HLA01920		0	0	17	0	0	0	1	18			WD					WD	WD
B*39:30	39:30	HLA01924		0	0	1	0	0	0	0	1									
**B*39:31 total**	**39:31 total**			**5**	**15**	**1228**	**109**	**15**	**1**	**61**	**1434**	**WD**	**I**	**C**	**C**	**I**		**I**	**I**	**C**
**B*39:31:01G total**	**39:31:01G total**			**5**	**15**	**1228**	**109**	**15**	**1**	**61**	**1434**	**WD**	**I**	**C**	**C**	**I**		**I**	**I**	**C**
B*39:31	39:31		39:31:01G	5	15	1228	109	15	1	61	1434	WD	I	C	C	I		I	I	C
B*39:32	39:32	HLA01927		0	0	3	0	0	0	0	3									
B*39:33	39:33	HLA02097		0	0	6	0	0	0	1	7			WD					WD	WD
B*39:34	39:34	HLA02163		0	0	2	0	2	0	1	5								WD	WD
B*39:35	39:35	HLA02331		0	0	1	0	0	0	1	2									
B*39:36	39:36	HLA02378		0	3	1	0	0	0	0	4									
B*39:37	39:37	HLA02384		0	0	6	0	39	1	14	60			WD		I		I	WD	I
B*39:38Q	39:38Q	HLA02412		0	2	0	0	0	0	0	2									
**B*39:39 total**	**39:39 total**			**1**	**0**	**2**	**0**	**21**	**2**	**3**	**29**					**I**			**WD**	**I**
B*39:39	39:39			0	0	1	0	3	1	1	6								WD	WD
B*39:39:01	39:39:01	HLA02426		1	0	1	0	18	1	2	23					I			WD	I
**B*39:40N total**	**39:40N total**			**0**	**0**	**6**	**0**	**0**	**0**	**0**	**6**			**WD**					**WD**	**WD**
B*39:40N	39:40N			0	0	2	0	0	0	0	2									
B*39:40:01N	39:40:01N	HLA02436		0	0	1	0	0	0	0	1									
B*39:40:02N	39:40:02N	HLA08388		0	0	3	0	0	0	0	3									
B*39:43	39:43	HLA03006		0	6	2	0	0	0	0	8		WD						WD	WD
B*39:47	39:47	HLA03621		0	5	2	0	0	0	1	8		WD						WD	WD
B*39:49	39:49	HLA03747		0	2	0	0	0	0	1	3									
B*39:51	39:51	HLA04072		0	0	0	1	0	0	0	1									

Supplemental Table 9: HLA-B Allele Summary [a]				Allele Count by Population Group [b]								3.0.0 CIWD Category by Population Group [c]								
Allele	Genomic typing	Allele ID	G group	AFA	API	EURO	MENA	HIS	NAM	UNK	Total	AFA	API	EURO	MENA	HIS	NAM	UNK	Total	Highest Frequency
B*39:52	39:52	HLA04105		0	0	0	1	0	0	0	1									
B*39:53	39:53	HLA04200		0	0	7	1	0	0	0	8			WD					WD	WD
B*39:54	39:54	HLA04161		0	0	0	0	2	0	1	3									
B*39:55	39:55	HLA04451		0	0	0	0	2	0	0	2									
B*39:57	39:57	HLA04796		0	0	7	0	0	0	3	10			WD					WD	WD
B*39:60	39:60	HLA05292		0	2	0	0	0	0	0	2									
B*39:61	39:61	HLA05511		0	0	2	0	0	0	0	2									
B*39:62	39:62	HLA05714		0	0	11	0	1	0	4	16			WD					WD	WD
B*39:64	39:64	HLA06938		0	0	4	0	0	0	0	4									
B*39:65	39:65	HLA06951		0	0	2	0	0	0	0	2									
B*39:68	39:68	HLA07307		0	0	4	0	0	0	2	6								WD	WD
B*39:69	39:69	HLA07472		0	0	1	0	0	0	0	1									
B*39:70	39:70	HLA07663		0	0	0	0	1	0	0	1									
B*39:71	39:71	HLA07664		0	0	2	0	0	0	0	2									
B*39:74	39:74	HLA08140		0	0	2	1	0	0	0	3									
B*39:75	39:75	HLA08142		0	0	0	0	6	0	0	6					WD			WD	WD
B*39:78	39:78	HLA08469		0	0	1	0	0	0	0	1									
B*39:79	39:79	HLA08524		0	0	8	0	0	0	8	16			WD				WD	WD	WD
B*39:81	39:81	HLA09011		0	0	3	0	0	0	1	4									
B*39:82	39:82	HLA09173		0	0	2	0	0	0	0	2									
B*39:89	39:89	HLA11038		2	0	0	0	0	0	0	2									
B*39:90	39:90	HLA11436		0	0	0	0	0	0	1	1									
B*39:91	39:91	HLA11450		0	0	1	0	0	0	0	1									
B*39:93	39:93	HLA11718		0	0	10	0	0	0	0	10			WD					WD	WD
B*39:96	39:96	HLA11845		0	0	0	0	0	0	2	2									
B*39:98	39:98	HLA12314		0	0	0	0	1	0	0	1									
B*39:101	39:101	HLA12981		0	0	1	0	0	0	0	1									
B*39:102	39:102	HLA13024		0	0	4	0	0	0	0	4									
B*39:105	39:105	HLA13546		0	0	0	0	0	0	1	1									
B*39:106	39:106	HLA13799		0	0	0	0	0	0	1	1									
B*39:107	39:107	HLA13979		0	0	3	0	0	0	0	3									

Supplemental Table 9: HLA-B Allele Summary [a]				Allele Count by Population Group [b]								3.0.0 CIWD Category by Population Group [c]								
Allele	Genomic typing	Allele ID	G group	AFA	API	EURO	MENA	HIS	NAM	UNK	Total	AFA	API	EURO	MENA	HIS	NAM	UNK	Total	Highest Frequency
B*39:111	39:111	HLA14449		0	0	2	0	0	0	0	2									
B*39:113	39:113	HLA14452		0	0	0	0	1	0	0	1									
B*39:121	39:121	HLA15773		0	0	1	0	0	0	0	1									
B*39:CODE	39:CODE			100	235	12528	83	2032	140	1550	16668	NA	NA	NA	NA	NA	NA	NA	NA	NA
**B*40:01 total**	**40:01 total**			**3876**	**54309**	**511758**	**5551**	**9875**	**1487**	**50983**	**637839**	**C**	**C**	**C**	**C**	**C**	**C**	**C**	**C**	**C**
B*40:01	40:01			184	1678	5550	28	584	60	1853	9937	C	C	C	WD	C	C	C	C	C
B*40:01P	40:01P			0	0	158	0	4	0	2	164			I					I	I
**B*40:01:01G total**	**40:01:01G total**			**3691**	**52623**	**505760**	**5521**	**9285**	**1426**	**49102**	**627408**	**C**	**C**	**C**	**C**	**C**	**C**	**C**	**C**	**C**
B*40:01:01G	40:01:01G		40:01:01G	3024	46801	481395	5378	7001	1155	45242	589996	C	C	C	C	C	C	C	C	C
B*40:01:01	40:01:01	HLA00291	40:01:01G	0	0	2	0	0	0	1	3									
B*40:01:02	40:01:02		40:01:01G	667	5811	24337	143	2284	271	3858	37371	C	C	C	C	C	C	C	C	C
B*40:01:02:01	40:01:02:01	HLA00292	40:01:01G	0	0	21	0	0	0	0	21			WD					WD	WD
B*40:01:25	40:01:25	HLA07925	40:01:01G	0	1	0	0	0	0	0	1									
B*40:01:42	40:01:42	HLA13218	40:01:01G	0	1	0	0	0	0	0	1									
B*40:01:45	40:01:45	HLA14634	40:01:01G	0	1	0	0	0	0	0	1									
B*40:01:48	40:01:48	HLA15629	40:01:01G	0	0	4	0	0	0	0	4									
B*40:55	40:55	HLA01993	40:01:01G	0	7	0	0	0	0	1	8		WD						WD	WD
B*40:150	40:150	HLA05803	40:01:01G	0	1	1	0	0	0	0	2									
B*40:01:03	40:01:03	HLA01450		0	1	80	1	0	0	11	93			WD				WD	WD	WD
B*40:01:04	40:01:04	HLA01800		1	3	145	1	1	1	12	164			I				WD	I	I
B*40:01:05	40:01:05	HLA01921		0	0	7	0	0	0	0	7			WD					WD	WD
B*40:01:07	40:01:07	HLA04070		0	0	21	0	1	0	0	22			WD					WD	WD
B*40:01:08	40:01:08	HLA04170		0	0	1	0	0	0	0	1									
B*40:01:15	40:01:15	HLA05520		0	0	1	0	0	0	0	1									
B*40:01:18	40:01:18	HLA05924		0	0	6	0	0	0	0	6			WD					WD	WD
B*40:01:20	40:01:20	HLA06253		0	0	1	0	0	0	0	1									
B*40:01:21	40:01:21	HLA06695		0	0	15	0	0	0	0	15			WD					WD	WD
B*40:01:23	40:01:23	HLA06973		0	0	8	0	0	0	0	8			WD					WD	WD
B*40:01:24	40:01:24	HLA07439		0	0	4	0	0	0	1	5								WD	WD
B*40:01:27	40:01:27	HLA08439		0	0	1	0	0	0	1	2									
B*40:01:32	40:01:32	HLA10484		0	1	0	0	0	0	0	1									

Supplemental Table 9: HLA-B Allele Summary [a]				Allele Count by Population Group [b]								3.0.0 CIWD Category by Population Group [c]								
Allele	Genomic typing	Allele ID	G group	AFA	API	EURO	MENA	HIS	NAM	UNK	Total	AFA	API	EURO	MENA	HIS	NAM	UNK	Total	Highest Frequency
B*40:01:40	40:01:40	HLA11931		0	3	0	0	0	0	1	4									
**B*40:02 total**	**40:02 total**			**1602**	**12986**	**176388**	**3414**	**29900**	**2526**	**20365**	**247181**	**C**	**C**	**C**	**C**	**C**	**C**	**C**	**C**	**C**
B*40:02	40:02			0	5	853	0	8	0	29	895		WD	I		I		I	I	I
B*40:02P	40:02P			0	0	204	0	1	0	1	206			I					I	I
**B*40:02:01G total**	**40:02:01G total**			**1602**	**12978**	**175279**	**3414**	**29888**	**2526**	**20332**	**246019**	**C**	**C**	**C**	**C**	**C**	**C**	**C**	**C**	**C**
B*40:02:01G	40:02:01G		40:02:01G	1317	10851	169314	3346	22662	1905	18557	227952	C	C	C	C	C	C	C	C	C
B*40:02:01	40:02:01		40:02:01G	285	2125	5957	68	7215	621	1773	18044	C	C	C	C	C	C	C	C	C
B*40:02:01:01	40:02:01:01	HLA00293	40:02:01G	0	0	4	0	0	0	0	4									
B*40:97	40:97	HLA03746	40:02:01G	0	1	0	0	0	0	1	2									
B*40:176	40:176	HLA07286	40:02:01G	0	1	0	0	11	0	1	13					I			WD	I
B*40:303	40:303	HLA13250	40:02:01G	0	0	4	0	0	0	0	4									
B*40:02:05	40:02:05	HLA03757		0	1	0	0	0	0	0	1									
B*40:02:06	40:02:06	HLA04174		0	0	13	0	0	0	0	13			WD					WD	WD
B*40:02:07	40:02:07	HLA04220		0	1	2	0	0	0	0	3									
B*40:02:08	40:02:08	HLA04543		0	0	4	0	0	0	0	4									
B*40:02:10	40:02:10	HLA04818		0	0	3	0	0	0	0	3									
B*40:02:14	40:02:14	HLA07687		0	0	21	0	0	0	0	21			WD					WD	WD
B*40:02:16	40:02:16	HLA08328		0	0	7	0	0	0	1	8			WD					WD	WD
B*40:02:17	40:02:17	HLA08503		0	0	0	0	0	0	2	2									
B*40:02:21	40:02:21	HLA11646		0	1	0	0	0	0	0	1									
B*40:02:22	40:02:22	HLA11745		0	0	2	0	0	0	0	2									
B*40:02:23	40:02:23	HLA12127		0	0	0	0	3	0	0	3									
**B*40:03 total**	**40:03 total**			**6**	**504**	**33**	**4**	**110**	**28**	**139**	**824**	**WD**	**C**	**WD**		**C**	**C**	**C**	**I**	**C**
**B*40:03:01G total**	**40:03:01G total**			**6**	**504**	**33**	**4**	**110**	**28**	**139**	**824**	**WD**	**C**	**WD**		**C**	**C**	**C**	**I**	**C**
B*40:03	40:03		40:03:01G	3	286	13	1	56	21	90	470		C	WD		I	C	I	I	C
B*40:03:01G	40:03:01G		40:03:01G	3	218	20	3	54	7	49	354		C	WD		I	C	I	I	C
B*40:04	40:04	HLA00295		87	15	506	1	1806	234	792	3441	C	I	I		C	C	C	C	C
**B*40:05 total**	**40:05 total**			**31**	**33**	**247**	**4**	**3272**	**172**	**522**	**4281**	**WD**	**I**	**I**		**C**	**C**	**C**	**C**	**C**
**B*40:05:01G total**	**40:05:01G total**			**31**	**33**	**247**	**4**	**3272**	**172**	**522**	**4281**	**WD**	**I**	**I**		**C**	**C**	**C**	**C**	**C**
B*40:05	40:05		40:05:01G	29	28	237	4	3092	164	485	4039	WD	I	I		C	C	C	C	C
B*40:05:01G	40:05:01G		40:05:01G	0	0	6	0	62	0	28	96			WD		I		I	WD	I

Supplemental Table 9: HLA-B Allele Summary [a]				Allele Count by Population Group [b]								3.0.0 CIWD Category by Population Group [c]								
Allele	Genomic typing	Allele ID	G group	AFA	API	EURO	MENA	HIS	NAM	UNK	Total	AFA	API	EURO	MENA	HIS	NAM	UNK	Total	Highest Frequency
B*40:05:01	40:05:01		40:05:01G	0	0	0	0	8	1	0	9					I			WD	I
B*40:05:01:01	40:05:01:01	HLA00296	40:05:01G	2	5	4	0	110	7	9	137		WD			C	C	WD	WD	C
**B*40:06 total**	**40:06 total**			**325**	**102239**	**5556**	**3143**	**756**	**306**	**3600**	**115925**	**C**	**C**	**C**	**C**	**C**	**C**	**C**	**C**	**C**
B*40:06	40:06			167	8684	1262	654	436	212	1819	13234	C	C	C	C	C	C	C	C	C
B*40:06P	40:06P			0	2	9	0	0	0	0	11			WD					WD	WD
**B*40:06:01G total**	**40:06:01G total**			**158**	**93547**	**4285**	**2489**	**320**	**94**	**1781**	**102674**	**C**	**C**	**C**	**C**	**C**	**C**	**C**	**C**	**C**
B*40:06:01G	40:06:01G		40:06:01G	114	89168	3989	2346	178	29	1421	97245	C	C	C	C	C	C	C	C	C
B*40:06:01	40:06:01		40:06:01G	3	141	78	31	4	5	22	284		C	WD	WD		WD	I	I	C
B*40:06:01:01	40:06:01:01	HLA00297	40:06:01G	1	888	13	1	9	0	148	1060		C	WD		I		C	I	C
B*40:06:01:02	40:06:01:02	HLA01430	40:06:01G	39	3100	203	110	126	56	185	3819	C	C	I	C	C	C	C	C	C
B*40:06:03	40:06:03	HLA05355	40:06:01G	0	1	0	0	0	0	0	1									
B*40:06:04	40:06:04		40:06:01G	1	249	2	1	3	4	5	265		C					WD	I	C
B*40:06:09	40:06:09	HLA11224		0	2	0	0	0	0	0	2									
B*40:06:12	40:06:12	HLA13739		0	3	0	0	0	0	0	3									
B*40:06:14	40:06:14	HLA13968		0	1	0	0	0	0	0	1									
B*40:07	40:07	HLA00298		0	2	83	2	1	0	2	90			WD					WD	WD
B*40:08	40:08	HLA00299		32	22	168	4	2330	158	420	3134	WD	I	I		C	C	C	C	C
B*40:09	40:09	HLA00300		0	0	12	0	14	0	19	45			WD		I		I	WD	I
**B*40:10 total**	**40:10 total**			**2**	**560**	**34**	**3**	**6**	**0**	**373**	**978**		**C**	**WD**		**WD**		**C**	**I**	**C**
B*40:10	40:10			0	34	2	0	1	0	22	59		I					I	WD	I
**B*40:10:01G total**	**40:10:01G total**			**2**	**526**	**32**	**3**	**5**	**0**	**351**	**919**		**C**	**WD**		**WD**		**C**	**I**	**C**
B*40:10:01G	40:10:01G		40:10:01G	0	42	11	0	0	0	22	75		I	WD				I	WD	I
B*40:10:01	40:10:01		40:10:01G	2	389	20	3	4	0	276	694		C	WD				C	I	C
B*40:10:01:02	40:10:01:02	HLA13770	40:10:01G	0	95	1	0	1	0	53	150		I					I	WD	I
**B*40:11 total**	**40:11 total**			**18**	**10**	**70**	**0**	**903**	**44**	**151**	**1196**	**WD**	**WD**	**WD**		**C**	**C**	**C**	**I**	**C**
B*40:11	40:11			2	0	8	0	86	6	6	108			WD		C	WD	WD	WD	C
B*40:11:01	40:11:01	HLA00302		15	9	61	0	812	38	143	1078	WD	WD	WD		C	C	C	I	C
B*40:11:02	40:11:02	HLA05126		1	1	1	0	5	0	2	10					WD			WD	WD
B*40:12	40:12	HLA00303		336	3	133	121	16	4	59	672	C		I	C	I		I	I	C
B*40:13	40:13	HLA00304		0	1	151	0	145	0	175	472			I		C		C	I	C
**B*40:14 total**	**40:14 total**			**0**	**0**	**64**	**0**	**1**	**0**	**6**	**71**			**WD**				**WD**	**WD**	**WD**

Supplemental Table 9: HLA-B Allele Summary [a]				Allele Count by Population Group [b]								3.0.0 CIWD Category by Population Group [c]								
Allele	Genomic typing	Allele ID	G group	AFA	API	EURO	MENA	HIS	NAM	UNK	Total	AFA	API	EURO	MENA	HIS	NAM	UNK	Total	Highest Frequency
B*40:14	40:14			0	0	5	0	0	0	0	5			WD					WD	WD
B*40:14:01	40:14:01	HLA00305		0	0	1	0	0	0	0	1									
B*40:14:02	40:14:02	HLA01667		0	0	31	0	0	0	0	31			WD					WD	WD
B*40:14:03	40:14:03	HLA01816		0	0	27	0	1	0	6	34			WD				WD	WD	WD
B*40:16	40:16	HLA00307		392	3	34	12	106	10	111	668	C		WD	WD	C	C	I	I	C
B*40:18	40:18	HLA00309		0	0	5	0	0	0	0	5			WD					WD	WD
B*40:19	40:19	HLA00310		0	0	29	0	1	0	3	33			WD					WD	WD
**B*40:20 total**	**40:20 total**			**3**	**1**	**19**	**1**	**196**	**11**	**31**	**262**			**WD**		**C**	**C**	**I**	**I**	**C**
**B*40:20:01G total**	**40:20:01G total**			**3**	**1**	**19**	**1**	**196**	**11**	**31**	**262**			**WD**		**C**	**C**	**I**	**I**	**C**
B*40:20	40:20		40:20:01G	3	1	19	1	196	11	31	262			WD		C	C	I	I	C
B*40:21	40:21	HLA00983		0	1	3	0	0	0	1	5								WD	WD
B*40:22N	40:22N	HLA00984		0	0	4	0	0	0	1	5								WD	WD
B*40:23	40:23	HLA01062		2	996	16	4	0	2	24	1044		C	WD				I	I	C
B*40:25	40:25	HLA01064		0	0	1	0	0	0	0	1									
B*40:26	40:26	HLA01122		1	315	6	1	0	0	6	329		C	WD				WD	I	C
**B*40:27 total**	**40:27 total**			**11**	**14**	**71**	**1**	**1109**	**35**	**143**	**1384**	**WD**	**I**	**WD**		**C**	**C**	**C**	**I**	**C**
B*40:27	40:27			1	3	9	0	153	2	6	174			WD		C		WD	I	C
B*40:27:01	40:27:01	HLA01129		10	11	61	1	956	33	137	1209	WD	WD	WD		C	C	C	I	C
B*40:27:02	40:27:02	HLA05954		0	0	1	0	0	0	0	1									
B*40:28	40:28	HLA01140		0	0	0	0	2	1	1	4									
B*40:29	40:29	HLA01223		0	2	0	0	0	0	0	2									
B*40:30	40:30	HLA01232		0	0	10	0	0	0	2	12			WD					WD	WD
B*40:31	40:31	HLA01246		0	0	24	0	1	0	3	28			WD					WD	WD
B*40:32	40:32	HLA01269		1	2	155	0	0	2	4	164			I					I	I
B*40:33	40:33	HLA01287		0	0	4	0	0	0	0	4									
**B*40:35 total**	**40:35 total**			**0**	**0**	**4**	**2**	**0**	**0**	**1**	**7**								**WD**	**WD**
B*40:35:01	40:35:01	HLA01329		0	0	3	2	0	0	1	6								WD	WD
B*40:35:02	40:35:02	HLA10999		0	0	1	0	0	0	0	1									
B*40:36	40:36	HLA01482		0	0	0	0	1	0	0	1									
B*40:37	40:37	HLA01488		0	0	0	0	2	0	1	3									
B*40:38	40:38	HLA01490		0	2	1	0	0	0	1	4									

Supplemental Table 9: HLA-B Allele Summary [a]				Allele Count by Population Group [b]								3.0.0 CIWD Category by Population Group [c]								
Allele	Genomic typing	Allele ID	G group	AFA	API	EURO	MENA	HIS	NAM	UNK	Total	AFA	API	EURO	MENA	HIS	NAM	UNK	Total	Highest Frequency
**B*40:40 total**	**40:40 total**			**0**	**55**	**4**	**4**	**1**	**1**	**6**	**71**		**I**					**WD**	**WD**	**I**
**B*40:40:01G total**	**40:40:01G total**			**0**	**55**	**4**	**4**	**1**	**1**	**6**	**71**		**I**					**WD**	**WD**	**I**
B*40:40	40:40	HLA01510	40:40:01G	0	8	2	1	1	0	2	14		WD						WD	WD
B*40:40:01G	40:40:01G		40:40:01G	0	38	2	3	0	1	4	48		I						WD	I
B*40:229	40:229	HLA08928	40:40:01G	0	9	0	0	0	0	0	9		WD						WD	WD
B*40:42	40:42	HLA01527		0	0	103	0	0	0	5	108			WD				WD	WD	WD
B*40:43	40:43	HLA01600		0	3	8	2	2	0	0	15			WD					WD	WD
B*40:44	40:44	HLA01604		0	13	0	0	0	0	0	13		I						WD	I
B*40:46	40:46	HLA01740		0	0	4	0	0	0	0	4									
B*40:47	40:47	HLA01748		0	0	15	0	0	0	0	15			WD					WD	WD
B*40:48	40:48	HLA01765		0	11	0	0	0	0	2	13		WD						WD	WD
B*40:49	40:49	HLA01898		0	0	31	0	0	0	0	31			WD					WD	WD
B*40:50	40:50	HLA01899		1	592	7	8	1	2	18	629		C	WD	WD			I	I	C
B*40:51	40:51	HLA01919		0	0	1	0	0	0	0	1									
B*40:52	40:52	HLA01976		0	4	5	0	0	0	2	11			WD					WD	WD
B*40:53	40:53	HLA01979		0	2	0	4	0	0	0	6								WD	WD
B*40:54	40:54	HLA01992		0	3	0	0	0	0	1	4									
B*40:61	40:61	HLA02249		0	5	0	0	0	0	0	5		WD						WD	WD
B*40:62	40:62	HLA02383		0	3	1	0	0	0	0	4									
B*40:63	40:63	HLA02458		0	0	5	0	0	0	0	5			WD					WD	WD
B*40:64	40:64	HLA02482		1	0	44	0	64	3	29	141			WD		I		I	WD	I
B*40:67	40:67	HLA02588		0	0	1	0	0	0	0	1									
B*40:69	40:69	HLA02678		0	1	0	0	0	0	0	1									
**B*40:70 total**	**40:70 total**			**0**	**4**	**0**	**0**	**0**	**0**	**0**	**4**									
B*40:70	40:70			0	1	0	0	0	0	0	1									
B*40:70:01	40:70:01	HLA02693		0	3	0	0	0	0	0	3									
**B*40:72 total**	**40:72 total**			**0**	**0**	**21**	**0**	**0**	**0**	**2**	**23**			**WD**					**WD**	**WD**
B*40:72	40:72			0	0	1	0	0	0	0	1									
B*40:72:02	40:72:02	HLA03344		0	0	20	0	0	0	2	22			WD					WD	WD
B*40:73	40:73	HLA02864		0	0	2	0	0	0	0	2									
B*40:74	40:74	HLA02866		0	0	2	4	0	0	0	6								WD	WD

Supplemental Table 9: HLA-B Allele Summary [a]				Allele Count by Population Group [b]								3.0.0 CIWD Category by Population Group [c]								
Allele	Genomic typing	Allele ID	G group	AFA	API	EURO	MENA	HIS	NAM	UNK	Total	AFA	API	EURO	MENA	HIS	NAM	UNK	Total	Highest Frequency
B*40:75	40:75	HLA02884		0	2	0	0	0	0	0	2									
B*40:76	40:76	HLA02906		0	1	0	0	0	0	1	2									
B*40:78	40:78	HLA02989		0	9	2	6	0	0	2	19		WD		WD				WD	WD
B*40:79	40:79	HLA03160		0	0	5	0	0	0	0	5			WD					WD	WD
B*40:80	40:80	HLA03173		0	0	3	0	0	0	0	3									
B*40:81	40:81	HLA03205		0	1	0	0	0	0	1	2									
B*40:84	40:84	HLA03231		0	4	1	0	0	0	0	5								WD	WD
**B*40:87 total**	**40:87 total**			**0**	**0**	**12**	**0**	**0**	**0**	**0**	**12**			**WD**					**WD**	**WD**
B*40:87	40:87			0	0	2	0	0	0	0	2									
B*40:87:01	40:87:01	HLA03256		0	0	10	0	0	0	0	10			WD					WD	WD
B*40:90	40:90	HLA03312		25	1	11	0	1	16	13	67	WD		WD			C	WD	WD	C
B*40:94	40:94	HLA03584		1	0	67	1	0	0	3	72			WD					WD	WD
B*40:95	40:95	HLA03628		0	17	0	0	0	0	0	17		I						WD	I
B*40:98	40:98	HLA03780		0	0	2	0	0	0	0	2									
B*40:99	40:99	HLA04387		0	0	0	0	4	0	2	6								WD	WD
B*40:102	40:102	HLA04903		0	0	12	6	1	0	0	19			WD	WD				WD	WD
B*40:103	40:103	HLA04912		0	0	0	5	0	0	0	5				WD				WD	WD
B*40:105	40:105	HLA04917		0	1	3	0	0	0	0	4									
B*40:107	40:107	HLA04920		1	0	0	0	2	1	0	4									
B*40:108	40:108	HLA05153		0	0	6	0	0	0	0	6			WD					WD	WD
B*40:109	40:109	HLA04930		0	3	0	0	0	0	0	3									
B*40:110	40:110	HLA04880		0	10	0	0	0	0	0	10		WD						WD	WD
B*40:111	40:111	HLA04934		1	1	0	0	6	2	1	11					WD			WD	WD
B*40:112	40:112	HLA04937		0	1	0	0	0	0	0	1									
B*40:113	40:113	HLA04939		0	0	6	0	0	0	0	6			WD					WD	WD
**B*40:114 total**	**40:114 total**			**0**	**1**	**19**	**0**	**0**	**0**	**2**	**22**			**WD**					**WD**	**WD**
B*40:114	40:114			0	1	7	0	0	0	0	8			WD					WD	WD
B*40:114:01	40:114:01	HLA04940		0	0	12	0	0	0	2	14			WD					WD	WD
B*40:115	40:115	HLA04947		0	24	0	1	0	0	0	25		I						WD	I
B*40:116	40:116	HLA04977		0	0	3	0	0	0	0	3									
B*40:117	40:117	HLA04979		0	1	4	0	2	0	0	7								WD	WD

Supplemental Table 9: HLA-B Allele Summary [a]				Allele Count by Population Group [b]								3.0.0 CIWD Category by Population Group [c]								
Allele	Genomic typing	Allele ID	G group	AFA	API	EURO	MENA	HIS	NAM	UNK	Total	AFA	API	EURO	MENA	HIS	NAM	UNK	Total	Highest Frequency
B*40:118N	40:118N	HLA04997		0	0	3	0	0	0	0	3									
B*40:119	40:119	HLA05010		0	0	1	0	1	0	3	5								WD	WD
B*40:120	40:120	HLA05011		0	0	3	0	0	0	0	3									
B*40:121	40:121	HLA05018		0	0	1	0	0	0	0	1									
B*40:122	40:122	HLA05021		0	3	0	0	0	0	0	3									
B*40:126	40:126	HLA04873		0	0	4	0	0	0	1	5								WD	WD
B*40:128	40:128	HLA04877		0	2	0	0	0	0	0	2									
B*40:129	40:129	HLA04878		0	0	0	0	6	0	3	9					WD			WD	WD
**B*40:130 total**	**40:130 total**			**0**	**7**	**5**	**0**	**0**	**0**	**3**	**15**		**WD**	**WD**					**WD**	**WD**
B*40:130	40:130			0	0	1	0	0	0	0	1									
B*40:130:01	40:130:01	HLA04881		0	6	1	0	0	0	1	8		WD						WD	WD
B*40:130:02	40:130:02	HLA10521		0	1	3	0	0	0	2	6								WD	WD
B*40:131	40:131	HLA05050		0	2	0	0	0	0	0	2									
B*40:134	40:134	HLA05093		0	2	0	0	0	0	0	2									
B*40:135	40:135	HLA05096		0	0	3	0	0	1	2	6								WD	WD
B*40:142N	40:142N	HLA05284		0	1	4	0	0	0	0	5								WD	WD
B*40:145	40:145	HLA05305		0	0	1	0	0	0	0	1									
B*40:148	40:148	HLA05537		0	2	0	0	0	0	0	2									
**B*40:155N total**	**40:155N total**			**0**	**6**	**0**	**0**	**0**	**0**	**0**	**6**		**WD**						**WD**	**WD**
**B*40:155:01G total**	**40:155:01G total**			**0**	**6**	**0**	**0**	**0**	**0**	**0**	**6**		**WD**						**WD**	**WD**
B*40:155N	40:155N		40:155:01G	0	2	0	0	0	0	0	2									
B*40:155:01G	40:155:01G		40:155:01G	0	1	0	0	0	0	0	1									
B*40:155:02N	40:155:02N	HLA11085	40:155:01G	0	3	0	0	0	0	0	3									
B*40:156	40:156	HLA05856		0	0	2	0	0	0	0	2									
B*40:157	40:157	HLA05859		0	1	3	0	0	0	1	5								WD	WD
B*40:159	40:159	HLA06025		0	6	0	0	0	0	0	6		WD						WD	WD
**B*40:160 total**	**40:160 total**			**0**	**5**	**8**	**0**	**0**	**0**	**0**	**13**		**WD**	**WD**					**WD**	**WD**
B*40:160	40:160			0	1	1	0	0	0	0	2									
B*40:160:01	40:160:01	HLA06228		0	4	1	0	0	0	0	5								WD	WD
B*40:160:02	40:160:02	HLA08325		0	0	6	0	0	0	0	6			WD					WD	WD
B*40:161	40:161	HLA06281		0	1	0	0	0	0	0	1									

Supplemental Table 9: HLA-B Allele Summary [a]				Allele Count by Population Group [b]								3.0.0 CIWD Category by Population Group [c]								
Allele	Genomic typing	Allele ID	G group	AFA	API	EURO	MENA	HIS	NAM	UNK	Total	AFA	API	EURO	MENA	HIS	NAM	UNK	Total	Highest Frequency
B*40:165	40:165	HLA06681		0	1	0	0	0	0	0	1									
B*40:168	40:168	HLA06914		0	0	4	0	1	0	0	5								WD	WD
B*40:170	40:170	HLA06939		0	0	2	0	0	0	0	2									
B*40:172	40:172	HLA06982		0	0	3	0	0	0	1	4									
B*40:175	40:175	HLA07285		0	0	1	0	1	0	2	4									
B*40:177	40:177	HLA07304		0	3	1	1	0	0	1	6								WD	WD
B*40:181	40:181	HLA07440		0	0	2	0	0	0	0	2									
B*40:183	40:183	HLA07626		0	16	0	0	0	0	0	16		I						WD	I
B*40:187	40:187	HLA07682		0	0	2	0	0	0	0	2									
B*40:190	40:190	HLA07686		0	1	0	0	0	0	0	1									
B*40:195	40:195	HLA07891		0	0	1	0	0	0	0	1									
B*40:196	40:196	HLA07893		0	0	0	0	0	0	1	1									
B*40:197	40:197	HLA07894		0	0	6	0	1	0	0	7			WD					WD	WD
B*40:198	40:198	HLA07895		0	0	3	0	0	0	0	3									
B*40:199	40:199	HLA07896		0	1	0	0	0	0	0	1									
B*40:200	40:200	HLA07897		0	0	8	0	0	0	0	8			WD					WD	WD
B*40:201	40:201	HLA07898		0	0	9	0	0	0	1	10			WD					WD	WD
B*40:203	40:203	HLA07900		0	0	6	0	0	0	0	6			WD					WD	WD
B*40:207	40:207	HLA07962		0	1	0	0	0	0	0	1									
B*40:217	40:217	HLA08323		0	0	4	0	0	0	0	4									
B*40:219	40:219	HLA08353		0	0	1	0	0	0	0	1									
B*40:224	40:224	HLA08658		0	0	0	0	3	0	0	3									
B*40:225	40:225	HLA08659		0	0	0	0	1	0	0	1									
B*40:226	40:226	HLA08660		0	0	0	1	6	1	2	10					WD			WD	WD
B*40:228	40:228	HLA08884		0	1	0	0	0	0	0	1									
B*40:232	40:232	HLA08978		0	0	1	0	0	0	0	1									
B*40:234	40:234	HLA09423		0	0	1	0	0	0	1	2									
B*40:235	40:235	HLA09425		0	1	0	0	0	0	0	1									
B*40:240	40:240	HLA09555		0	0	1	0	0	0	0	1									
B*40:245	40:245	HLA09702		0	1	0	0	0	0	0	1									
B*40:268	40:268	HLA10729		0	1	0	0	0	0	0	1									

Supplemental Table 9: HLA-B Allele Summary [a]				Allele Count by Population Group [b]								3.0.0 CIWD Category by Population Group [c]								
Allele	Genomic typing	Allele ID	G group	AFA	API	EURO	MENA	HIS	NAM	UNK	Total	AFA	API	EURO	MENA	HIS	NAM	UNK	Total	Highest Frequency
B*40:273	40:273	HLA10997		0	0	1	0	0	0	0	1									
B*40:274	40:274	HLA10998		0	0	0	0	2	0	0	2									
B*40:282	40:282	HLA11418		0	1	0	0	0	0	0	1									
B*40:283	40:283	HLA11455		0	0	0	0	2	0	0	2									
B*40:287	40:287	HLA11721		0	3	0	0	0	0	0	3									
B*40:288	40:288	HLA11813		0	0	1	0	0	0	0	1									
B*40:290	40:290	HLA11815		0	0	1	0	0	0	2	3									
B*40:291N	40:291N	HLA11816		0	2	0	0	0	0	0	2									
B*40:292	40:292	HLA11817		0	0	0	0	0	0	1	1									
B*40:293	40:293	HLA12000		0	0	1	0	0	0	0	1									
B*40:295	40:295	HLA12128		0	39	0	1	0	0	0	40		I						WD	I
B*40:296	40:296	HLA12264		0	0	1	0	2	0	0	3									
**B*40:298 total**	**40:298 total**			**0**	**0**	**0**	**0**	**3**	**0**	**0**	**3**									
B*40:298	40:298			0	0	0	0	3	0	0	3									
B*40:306	40:306	HLA13384		0	1	0	0	0	0	0	1									
B*40:309	40:309	HLA13740		0	0	0	0	1	0	0	1									
B*40:310	40:310	HLA13850		0	6	0	0	0	0	0	6		WD						WD	WD
B*40:311	40:311	HLA13852		0	1	0	0	0	0	1	2									
B*40:313	40:313	HLA13965		0	1	0	0	0	0	0	1									
B*40:318	40:318	HLA14242		0	1	0	0	0	0	0	1									
B*40:321	40:321	HLA14229		0	0	1	0	0	0	0	1									
B*40:322	40:322	HLA14383		0	0	1	0	0	0	0	1									
B*40:331	40:331	HLA14924		0	0	0	0	1	0	0	1									
B*40:334	40:334	HLA14981		0	0	1	0	12	0	9	22					I		WD	WD	I
B*40:340	40:340	HLA15541		0	1	0	0	0	0	0	1									
B*40:347	40:347	HLA16266		0	0	1	0	0	0	0	1									
B*40:348	40:348	HLA16466		0	1	0	0	0	0	0	1									
B*40:CODE	40:CODE			351	4282	54471	578	3384	280	4555	67901	NA	NA	NA	NA	NA	NA	NA	NA	NA
**B*41:01 total**	**41:01 total**			**1943**	**2807**	**58236**	**10552**	**6982**	**520**	**14336**	**95376**	**C**	**C**	**C**	**C**	**C**	**C**	**C**	**C**	**C**
B*41:01	41:01			742	877	21934	2964	2712	260	5448	34937	C	C	C	C	C	C	C	C	C
B*41:01P	41:01P			0	0	32	0	0	0	0	32			WD					WD	WD

Supplemental Table 9: HLA-B Allele Summary [a]				Allele Count by Population Group [b]								3.0.0 CIWD Category by Population Group [c]								
Allele	Genomic typing	Allele ID	G group	AFA	API	EURO	MENA	HIS	NAM	UNK	Total	AFA	API	EURO	MENA	HIS	NAM	UNK	Total	Highest Frequency
B*41:01:01	41:01:01	HLA00312		1201	1929	36269	7582	4270	260	8888	60399	C	C	C	C	C	C	C	C	C
B*41:01:02	41:01:02	HLA11644		0	0	1	0	0	0	0	1									
B*41:01:03	41:01:03	HLA12491		0	0	0	6	0	0	0	6				WD				WD	WD
B*41:01:05	41:01:05	HLA16928		0	1	0	0	0	0	0	1									
**B*41:02 total**	**41:02 total**			**2203**	**629**	**75559**	**2614**	**3308**	**332**	**8632**	**93277**	**C**	**C**	**C**	**C**	**C**	**C**	**C**	**C**	**C**
B*41:02	41:02			150	32	3709	168	416	29	358	4862	C	I	C	C	C	C	C	C	C
B*41:02P	41:02P			0	0	14	0	0	0	0	14			WD					WD	WD
**B*41:02:01G total**	**41:02:01G total**			**2052**	**597**	**71829**	**2446**	**2891**	**303**	**8270**	**88388**	**C**	**C**	**C**	**C**	**C**	**C**	**C**	**C**	**C**
B*41:02:01G	41:02:01G		41:02:01G	247	201	25236	696	366	10	2623	29379	C	C	C	C	C	C	C	C	C
B*41:02:01	41:02:01	HLA00313	41:02:01G	1805	396	46593	1750	2525	293	5647	59009	C	C	C	C	C	C	C	C	C
B*41:02:02	41:02:02	HLA03965		0	0	1	0	0	0	0	1									
B*41:02:03	41:02:03	HLA05515		0	0	1	0	0	0	1	2									
B*41:02:04	41:02:04	HLA06697		0	0	1	0	0	0	0	1									
B*41:02:05	41:02:05	HLA09424		1	0	4	0	0	0	2	7								WD	WD
B*41:02:06	41:02:06	HLA12653		0	0	0	0	1	0	1	2									
**B*41:03 total**	**41:03 total**			**250**	**0**	**16**	**1**	**11**	**4**	**56**	**338**	**C**		**WD**		**I**		**I**	**I**	**C**
B*41:03	41:03			17	0	0	1	4	0	2	24	WD							WD	WD
B*41:03:01	41:03:01	HLA00314		233	0	12	0	7	4	54	310	C		WD		WD		I	I	C
B*41:03:02	41:03:02	HLA02922		0	0	4	0	0	0	0	4									
B*41:04	41:04	HLA01256		3	0	0	0	0	0	0	3									
B*41:05	41:05	HLA01288		0	0	14	0	0	0	1	15			WD					WD	WD
B*41:06	41:06	HLA01452		0	0	14	0	11	0	0	25			WD		I			WD	I
B*41:07	41:07	HLA02144		2	0	2	0	44	3	11	62					I		WD	WD	I
B*41:09	41:09	HLA03905		0	0	3	3	0	0	0	6								WD	WD
B*41:10	41:10	HLA04094		0	0	8	0	0	0	0	8			WD					WD	WD
B*41:11	41:11	HLA04236		0	0	8	0	0	0	0	8			WD					WD	WD
B*41:14	41:14	HLA05538		0	0	3	1	0	0	1	5								WD	WD
B*41:15	41:15	HLA05533		0	0	1	0	0	0	0	1									
B*41:16	41:16	HLA05833		0	1	23	0	0	0	0	24			WD					WD	WD
B*41:18	41:18	HLA06252		0	0	7	0	0	0	0	7			WD					WD	WD
B*41:19	41:19	HLA06694		0	0	6	0	0	0	0	6			WD					WD	WD

Supplemental Table 9: HLA-B Allele Summary [a]				Allele Count by Population Group [b]								3.0.0 CIWD Category by Population Group [c]								
Allele	Genomic typing	Allele ID	G group	AFA	API	EURO	MENA	HIS	NAM	UNK	Total	AFA	API	EURO	MENA	HIS	NAM	UNK	Total	Highest Frequency
B*41:21	41:21	HLA08290		0	0	1	1	0	0	0	2									
B*41:22	41:22	HLA08502		0	0	2	0	0	0	0	2									
B*41:23	41:23	HLA08654		0	0	0	0	0	0	1	1									
B*41:27	41:27	HLA09310		0	0	1	0	0	0	0	1									
B*41:28	41:28	HLA09554		0	0	0	0	0	0	3	3									
B*41:30	41:30	HLA10400		0	0	3	0	0	0	0	3									
B*41:31	41:31	HLA10401		0	0	0	1	0	0	0	1									
B*41:32	41:32	HLA10995		0	0	0	0	1	0	0	1									
B*41:40	41:40	HLA13994		0	0	1	0	0	0	0	1									
B*41:44	41:44	HLA14228		0	0	1	0	0	0	0	1									
B*41:51	41:51	HLA16881		0	0	0	2	0	0	0	2									
B*41:CODE	41:CODE			44	13	4811	69	289	20	475	5721	NA	NA	NA	NA	NA	NA	NA	NA	NA
**B*42:01 total**	**42:01 total**			**18521**	**136**	**2337**	**1179**	**3299**	**588**	**4986**	**31046**	**C**	**C**	**C**	**C**	**C**	**C**	**C**	**C**	**C**
B*42:01	42:01			1227	6	163	47	369	51	263	2126	C	WD	I	C	C	C	C	C	C
B*42:01P	42:01P			0	0	1	0	1	0	0	2									
**B*42:01:01G total**	**42:01:01G total**			**17294**	**130**	**2173**	**1132**	**2929**	**537**	**4723**	**28918**	**C**	**C**	**C**	**C**	**C**	**C**	**C**	**C**	**C**
B*42:01:01	42:01:01	HLA00315	42:01:01G	17294	130	2173	1132	2929	537	4723	28918	C	C	C	C	C	C	C	C	C
**B*42:02 total**	**42:02 total**			**2726**	**49**	**1553**	**287**	**903**	**103**	**942**	**6563**	**C**	**I**	**C**	**C**	**C**	**C**	**C**	**C**	**C**
**B*42:02:01G total**	**42:02:01G total**			**2726**	**49**	**1553**	**287**	**903**	**103**	**942**	**6563**	**C**	**I**	**C**	**C**	**C**	**C**	**C**	**C**	**C**
B*42:02	42:02		42:02:01G	1973	30	1018	244	585	73	624	4547	C	I	I	C	C	C	C	C	C
B*42:02P	42:02P			0	0	2	0	0	0	0	2									
B*42:02:01G	42:02:01G		42:02:01G	333	17	447	33	107	4	186	1127	C	I	I	WD	C		C	I	C
B*42:02:01	42:02:01		42:02:01G	12	0	1	5	10	1	19	48	WD			WD	I		I	WD	I
B*42:02:01:01	42:02:01:01	HLA00316	42:02:01G	1	0	0	0	0	0	0	1									
B*42:02:01:02	42:02:01:02	HLA13483	42:02:01G	407	2	85	5	201	25	113	838	C		WD	WD	C	C	I	I	C
**B*42:05 total**	**42:05 total**			**0**	**0**	**135**	**1**	**1**	**0**	**3**	**140**			**I**					**WD**	**I**
B*42:05	42:05			0	0	6	0	0	0	0	6			WD					WD	WD
B*42:05:01	42:05:01	HLA01712		0	0	129	1	1	0	3	134			I					WD	I
B*42:07	42:07	HLA02311		0	0	4	17	0	0	0	21				WD				WD	WD
B*42:08	42:08	HLA02390		2	0	0	0	0	0	0	2									
B*42:09	42:09	HLA02643		1	0	0	0	0	0	0	1									

Supplemental Table 9: HLA-B Allele Summary [a]				Allele Count by Population Group [b]								3.0.0 CIWD Category by Population Group [c]								
Allele	Genomic typing	Allele ID	G group	AFA	API	EURO	MENA	HIS	NAM	UNK	Total	AFA	API	EURO	MENA	HIS	NAM	UNK	Total	Highest Frequency
B*42:10	42:10	HLA03645		5	0	1	0	4	1	1	12	WD							WD	WD
B*42:13	42:13	HLA04168		2	0	1	0	0	0	1	4									
B*42:14	42:14	HLA05308		1	0	0	0	0	0	0	1									
B*42:15	42:15	HLA06223		1	0	0	0	0	0	0	1									
B*42:16	42:16	HLA06286		0	0	0	1	0	0	0	1									
B*42:17	42:17	HLA07959		0	0	0	0	0	0	2	2									
B*42:18	42:18	HLA08981		0	0	6	0	8	0	7	21			WD		I		WD	WD	I
B*42:20	42:20	HLA09313		0	0	0	0	1	0	0	1									
B*42:21	42:21	HLA13541		0	0	0	0	0	0	1	1									
B*42:22	42:22	HLA14236		1	0	0	0	0	0	0	1									
B*42:CODE	42:CODE			924	4	316	11	233	24	252	1764	NA	NA	NA	NA	NA	NA	NA	NA	NA
**B*44:02 total**	**44:02 total**			**6023**	**8871**	**875511**	**16344**	**24376**	**2516**	**81297**	**1014938**	**C**	**C**	**C**	**C**	**C**	**C**	**C**	**C**	**C**
B*44:02	44:02			0	0	2522	7	18	0	151	2698			C	WD	I		C	C	C
**B*44:02S total**	**44:02S total**			**0**	**0**	**2**	**0**	**1**	**0**	**1**	**4**									
B*44:02P	44:02P			0	3	663	3	3	0	1	673			I					I	I
**B*44:02:01G total**	**44:02:01G total**			**6022**	**8868**	**872020**	**16334**	**24335**	**2515**	**81105**	**1011199**	**C**	**C**	**C**	**C**	**C**	**C**	**C**	**C**	**C**
B*44:02:01G	44:02:01G		44:02:01G	4992	8174	829365	16004	18663	2024	75682	954904	C	C	C	C	C	C	C	C	C
B*44:02:01	44:02:01		44:02:01G	32	12	1638	6	146	7	208	2049	WD	WD	C	WD	C	C	C	C	C
B*44:02:01:01	44:02:01:01	HLA00318	44:02:01G	884	648	36166	305	5095	457	4654	48209	C	C	C	C	C	C	C	C	C
B*44:02:01:02S	44:02:01:02S	HLA01606	44:02:01G	0	0	2	0	1	0	1	4									
B*44:02:01:03	44:02:01:03	HLA08401	44:02:01G	104	27	3763	14	319	23	442	4692	C	I	C	WD	C	C	C	C	C
B*44:02:01:04	44:02:01:04	HLA15763	44:02:01G	2	0	17	0	1	0	2	22			WD					WD	WD
B*44:02:01:05	44:02:01:05	HLA15765	44:02:01G	0	0	5	0	0	0	1	6			WD					WD	WD
B*44:02:01:06	44:02:01:06	HLA16683	44:02:01G	0	0	1	0	0	0	0	1									
B*44:02:46	44:02:46	HLA16859	44:02:01G	0	0	1	0	0	0	0	1									
B*44:27:01	44:27:01	HLA01489	44:02:01G	8	7	1062	5	110	4	115	1311	WD	WD	I	WD	C		I	I	C
B*44:02:02	44:02:02	HLA01247		0	0	33	0	0	0	10	43			WD				WD	WD	WD
B*44:02:03	44:02:03	HLA01487		0	0	1	0	0	0	0	1									
B*44:02:05	44:02:05	HLA03412		0	0	3	0	0	0	2	5								WD	WD
B*44:02:06	44:02:06	HLA03953		0	0	14	0	19	1	13	47			WD		I		WD	WD	I
B*44:02:07	44:02:07	HLA03960		0	0	12	0	0	0	2	14			WD					WD	WD

Supplemental Table 9: HLA-B Allele Summary [a]				Allele Count by Population Group [b]								3.0.0 CIWD Category by Population Group [c]								
Allele	Genomic typing	Allele ID	G group	AFA	API	EURO	MENA	HIS	NAM	UNK	Total	AFA	API	EURO	MENA	HIS	NAM	UNK	Total	Highest Frequency
B*44:02:09	44:02:09	HLA04116		0	0	10	0	0	0	0	10			WD					WD	WD
B*44:02:10	44:02:10	HLA04196		0	0	9	0	0	0	0	9			WD					WD	WD
B*44:02:12	44:02:12	HLA04568		1	0	9	0	1	0	3	14			WD					WD	WD
B*44:02:15	44:02:15	HLA06000		0	0	5	0	0	0	6	11			WD				WD	WD	WD
B*44:02:18	44:02:18	HLA06646		0	0	6	0	0	0	0	6			WD					WD	WD
B*44:02:19	44:02:19	HLA06985		0	0	4	0	0	0	1	5								WD	WD
B*44:02:20	44:02:20	HLA06998		0	0	1	0	0	0	0	1									
B*44:02:22	44:02:22	HLA07450		0	0	6	0	0	0	0	6			WD					WD	WD
B*44:02:24	44:02:24	HLA07641		0	0	2	0	0	0	0	2									
B*44:02:33	44:02:33	HLA12004		0	0	2	0	0	0	0	2									
B*44:02:34	44:02:34	HLA12564		0	0	4	0	0	0	0	4									
**B*44:27 total**	**44:27 total**			**8**	**7**	**1241**	**5**	**110**	**4**	**118**	**1493**	**WD**	**WD**	**C**	**WD**	**C**		**I**	**I**	**C**
B*44:27	44:27			0	0	179	0	0	0	3	182			I					I	I
B*44:173	44:173	HLA09169		0	0	6	0	0	0	0	6			WD					WD	WD
**B*44:03 total**	**44:03 total**			**18016**	**75358**	**487012**	**9241**	**38112**	**3896**	**58231**	**689866**	**C**	**C**	**C**	**C**	**C**	**C**	**C**	**C**	**C**
B*44:03	44:03			8661	4053	135235	2845	17919	2178	27996	198887	C	C	C	C	C	C	C	C	C
B*44:03P	44:03P			0	2	92	0	0	0	0	94			WD					WD	WD
**B*44:03:01G total**	**44:03:01G total**			**7462**	**6425**	**344824**	**4721**	**19081**	**1367**	**27559**	**411439**	**C**	**C**	**C**	**C**	**C**	**C**	**C**	**C**	**C**
B*44:03:01G	44:03:01G		44:03:01G	3954	4920	317468	4449	8415	327	21920	361453	C	C	C	C	C	C	C	C	C
B*44:03:01	44:03:01		44:03:01G	1933	814	12900	164	5977	550	3323	25661	C	C	C	C	C	C	C	C	C
B*44:03:01:01	44:03:01:01	HLA00319	44:03:01G	1462	686	14199	92	4404	442	2222	23507	C	C	C	C	C	C	C	C	C
B*44:03:01:02	44:03:01:02	HLA14090	44:03:01G	66	4	182	15	261	47	74	649	C		I	WD	C	C	I	I	C
B*44:03:01:03	44:03:01:03	HLA14807	44:03:01G	45	0	1	0	11	1	9	67	C				I		WD	WD	C
B*44:03:01:04	44:03:01:04	HLA16269	44:03:01G	1	0	1	0	0	0	0	2									
B*44:03:01:09	44:03:01:09	HLA16856	44:03:01G	0	1	27	1	11	0	8	48			WD		I		WD	WD	I
B*44:03:03	44:03:03	HLA02825	44:03:01G	1	0	43	0	1	0	3	48			WD					WD	WD
B*44:03:04	44:03:04	HLA03840	44:03:01G	0	0	3	0	1	0	0	4									
**B*44:03:02G total**	**44:03:02G total**			**1881**	**64866**	**6832**	**1675**	**1112**	**351**	**2662**	**79379**	**C**	**C**	**C**	**C**	**C**	**C**	**C**	**C**	**C**
B*44:03:02G	44:03:02G		44:03:02G	337	41349	3313	824	159	21	848	46851	C	C	C	C	C	C	C	C	C
B*44:03:02	44:03:02	HLA00320	44:03:02G	1544	23516	3519	851	953	330	1814	32527	C	C	C	C	C	C	C	C	C
B*44:03:27	44:03:27	HLA13336	44:03:02G	0	1	0	0	0	0	0	1									

Supplemental Table 9: HLA-B Allele Summary [a]				Allele Count by Population Group [b]								3.0.0 CIWD Category by Population Group [c]								
Allele	Genomic typing	Allele ID	G group	AFA	API	EURO	MENA	HIS	NAM	UNK	Total	AFA	API	EURO	MENA	HIS	NAM	UNK	Total	Highest Frequency
B*44:03:05	44:03:05	HLA03907		0	0	1	0	0	0	0	1									
B*44:03:06	44:03:06	HLA04103		0	0	2	0	0	0	0	2									
B*44:03:08	44:03:08	HLA04252		8	0	0	0	0	0	4	12	WD							WD	WD
B*44:03:09	44:03:09	HLA04754		0	0	4	0	0	0	0	4									
B*44:03:10	44:03:10	HLA04815		2	0	1	0	0	0	2	5								WD	WD
B*44:03:11	44:03:11	HLA04847		0	0	7	0	0	0	1	8			WD					WD	WD
B*44:03:13	44:03:13	HLA06262		0	12	0	0	0	0	1	13		WD						WD	WD
B*44:03:14	44:03:14	HLA06645		0	0	2	0	0	0	0	2									
B*44:03:19	44:03:19	HLA08471		0	0	6	0	0	0	3	9			WD					WD	WD
B*44:03:22	44:03:22	HLA11238		0	0	3	0	0	0	0	3									
B*44:03:23	44:03:23	HLA11477		0	0	3	0	0	0	2	5								WD	WD
B*44:03:25	44:03:25	HLA12990		0	0	0	0	0	0	1	1									
B*44:03:32	44:03:32	HLA14227		1	0	0	0	0	0	0	1									
B*44:03:33	44:03:33	HLA14397		1	0	0	0	0	0	0	1									
B*44:04	44:04	HLA00321		45	12	6135	18	153	19	493	6875	C	WD	C	WD	C	C	C	C	C
**B*44:05 total**	**44:05 total**			**159**	**159**	**59620**	**1191**	**1004**	**60**	**3728**	**65921**	**C**	**C**	**C**	**C**	**C**	**C**	**C**	**C**	**C**
B*44:05	44:05			15	9	3684	99	108	4	187	4106	WD	WD	C	C	C		C	C	C
B*44:05P	44:05P			0	0	22	0	1	0	0	23			WD					WD	WD
B*44:05:01	44:05:01	HLA00322		144	150	55909	1092	894	56	3524	61769	C	C	C	C	C	C	C	C	C
B*44:05:02	44:05:02	HLA04563		0	0	1	0	0	0	0	1									
B*44:05:03	44:05:03	HLA06944		0	0	4	0	1	0	17	22							I	WD	I
B*44:06	44:06	HLA00323		2	100	777	35	45	4	47	1010		I	I	WD	I		I	I	I
B*44:07	44:07	HLA00324		88	1	2	3	1	1	15	111	C						I	WD	C
B*44:08	44:08	HLA00325		6	1	195	0	5	1	22	230	WD		I		WD		I	I	I
B*44:09	44:09	HLA00326		3	1	223	1	3	1	31	263			I				I	I	I
B*44:10	44:10	HLA00327		492	0	22	0	71	9	117	711	C		WD		C	C	I	I	C
B*44:13	44:13	HLA01066		0	0	15	0	2	0	6	23			WD				WD	WD	WD
**B*44:15 total**	**44:15 total**			**108**	**2**	**10**	**1**	**1**	**0**	**11**	**133**	**C**		**WD**				**WD**	**WD**	**C**
**B*44:15:01G total**	**44:15:01G total**			**108**	**2**	**10**	**1**	**1**	**0**	**11**	**133**	**C**		**WD**				**WD**	**WD**	**C**
B*44:15	44:15		44:15:01G	108	2	10	1	1	0	11	133	C		WD				WD	WD	C
B*44:17	44:17	HLA01148		0	0	61	13	1	0	26	101			WD	WD			I	WD	I

Supplemental Table 9: HLA-B Allele Summary [a]				Allele Count by Population Group [b]								3.0.0 CIWD Category by Population Group [c]								
Allele	Genomic typing	Allele ID	G group	AFA	API	EURO	MENA	HIS	NAM	UNK	Total	AFA	API	EURO	MENA	HIS	NAM	UNK	Total	Highest Frequency
B*44:18	44:18	HLA01144		23	0	2	0	0	0	3	28	WD							WD	WD
B*44:20	44:20	HLA01195		1	0	30	0	1	0	6	38			WD				WD	WD	WD
B*44:21	44:21	HLA01197		0	0	131	8	6	0	10	155			I	WD	WD		WD	WD	I
B*44:22	44:22	HLA01313		0	0	9	1	0	0	2	12			WD					WD	WD
B*44:23N	44:23N	HLA01341		0	0	42	0	4	1	4	51			WD					WD	WD
B*44:24	44:24	HLA01354		0	0	9	0	0	0	1	10			WD					WD	WD
B*44:25	44:25	HLA01427		0	0	57	0	1	0	1	59			WD					WD	WD
**B*44:28 total**	**44:28 total**			**2**	**0**	**9**	**1**	**0**	**0**	**1**	**13**			**WD**					**WD**	**WD**
B*44:28	44:28			0	0	1	0	0	0	0	1									
B*44:28:01	44:28:01	HLA01521		2	0	0	0	0	0	0	2									
B*44:28:02	44:28:02	HLA03203		0	0	8	1	0	0	1	10			WD					WD	WD
B*44:29	44:29	HLA01526		3	7	1086	190	9	2	45	1342		WD	I	C	I		I	I	C
B*44:30	44:30	HLA01530		0	0	1	0	12	4	0	17					I			WD	I
B*44:31	44:31	HLA01569		0	0	0	13	0	0	0	13				WD				WD	WD
B*44:32	44:32	HLA01617		0	0	28	0	0	0	1	29			WD					WD	WD
**B*44:34 total**	**44:34 total**			**0**	**0**	**23**	**0**	**0**	**0**	**4**	**27**			**WD**					**WD**	**WD**
B*44:34	44:34			0	0	7	0	0	0	1	8			WD					WD	WD
B*44:34:01	44:34:01	HLA01700		0	0	14	0	0	0	3	17			WD					WD	WD
B*44:34:02	44:34:02	HLA08517		0	0	2	0	0	0	0	2									
B*44:36	44:36	HLA01784		0	0	20	0	0	0	6	26			WD				WD	WD	WD
**B*44:37 total**	**44:37 total**			**0**	**9**	**8**	**0**	**0**	**0**	**0**	**17**		**WD**	**WD**					**WD**	**WD**
B*44:37	44:37			0	1	2	0	0	0	0	3									
B*44:37:01	44:37:01	HLA01790		0	0	6	0	0	0	0	6			WD					WD	WD
B*44:37:02	44:37:02	HLA07699		0	8	0	0	0	0	0	8		WD						WD	WD
**B*44:41 total**	**44:41 total**			**0**	**0**	**30**	**1**	**0**	**0**	**0**	**31**			**WD**					**WD**	**WD**
B*44:41	44:41			0	0	5	0	0	0	0	5			WD					WD	WD
B*44:41:01	44:41:01	HLA02098		0	0	25	1	0	0	0	26			WD					WD	WD
B*44:42	44:42	HLA02206		0	0	1	0	0	0	0	1									
**B*44:43 total**	**44:43 total**			**0**	**0**	**5**	**0**	**1**	**0**	**1**	**7**			**WD**					**WD**	**WD**
B*44:43:01	44:43:01	HLA02234		0	0	5	0	1	0	1	7			WD					WD	WD
B*44:45	44:45	HLA02316		0	0	10	0	0	0	0	10			WD					WD	WD

Supplemental Table 9: HLA-B Allele Summary [a]				Allele Count by Population Group [b]								3.0.0 CIWD Category by Population Group [c]								
**Allele**	**Genomic typing**	**Allele ID**	**G group**	**AFA**	**API**	**EURO**	**MENA**	**HIS**	**NAM**	**UNK**	**Total**	**AFA**	**API**	**EURO**	**MENA**	**HIS**	**NAM**	**UNK**	**Total**	**Highest Frequency**
B*44:46	44:46	HLA02343		0	0	1	0	1	0	0	2									
B*44:47	44:47	HLA02464		0	1	0	0	0	0	0	1									
B*44:48	44:48	HLA02644		0	0	35	0	0	0	2	37			WD					WD	WD
**B*44:50 total**	**44:50 total**			**3**	**0**	**2**	**0**	**4**	**0**	**2**	**11**								**WD**	**WD**
B*44:50	44:50			2	0	1	0	1	0	2	6								WD	WD
B*44:50:01	44:50:01	HLA02695		1	0	1	0	3	0	0	5								WD	WD
B*44:51	44:51	HLA02700		0	0	4	0	0	0	0	4									
**B*44:53 total**	**44:53 total**			**0**	**0**	**1**	**0**	**1**	**0**	**0**	**2**									
B*44:53	44:53			0	0	1	0	1	0	0	2									
B*44:55	44:55	HLA03001		0	0	2	0	0	0	0	2									
B*44:57	44:57	HLA03133		0	0	3	0	0	0	0	3									
**B*44:59 total**	**44:59 total**			**0**	**0**	**2**	**0**	**1**	**0**	**0**	**3**									
B*44:59	44:59			0	0	1	0	0	0	0	1									
B*44:59:01	44:59:01	HLA03190		0	0	1	0	1	0	0	2									
B*44:60	44:60	HLA03259		0	0	4	2	0	0	2	8								WD	WD
B*44:62	44:62	HLA03358		0	0	2	0	0	0	0	2									
B*44:63	44:63	HLA03359		0	0	0	0	0	0	1	1									
**B*44:64 total**	**44:64 total**			**0**	**0**	**23**	**0**	**2**	**0**	**0**	**25**			**WD**					**WD**	**WD**
B*44:64	44:64			0	0	1	0	0	0	0	1									
B*44:64:02	44:64:02	HLA03393		0	0	22	0	2	0	0	24			WD					WD	WD
B*44:65	44:65	HLA03536		0	1	2	0	0	0	1	4									
B*44:67	44:67	HLA03627		0	0	8	0	0	0	0	8			WD					WD	WD
B*44:68	44:68	HLA03718		0	0	8	0	0	0	0	8			WD					WD	WD
**B*44:69 total**	**44:69 total**			**0**	**1**	**1**	**0**	**0**	**0**	**0**	**2**									
B*44:69:01	44:69:01	HLA03695		0	0	1	0	0	0	0	1									
B*44:69:02	44:69:02	HLA15272		0	1	0	0	0	0	0	1									
B*44:70	44:70	HLA03903		0	0	3	0	0	0	0	3									
B*44:71	44:71	HLA03950		0	0	2	0	0	0	0	2									
B*44:72	44:72	HLA03962		0	1	1	0	0	0	0	2									
B*44:73	44:73	HLA03973		0	0	11	0	0	0	0	11			WD					WD	WD
B*44:74	44:74	HLA03974		0	0	28	0	0	0	0	28			WD					WD	WD

Supplemental Table 9: HLA-B Allele Summary [a]				Allele Count by Population Group [b]								3.0.0 CIWD Category by Population Group [c]								
Allele	Genomic typing	Allele ID	G group	AFA	API	EURO	MENA	HIS	NAM	UNK	Total	AFA	API	EURO	MENA	HIS	NAM	UNK	Total	Highest Frequency
B*44:75	44:75	HLA04044		0	0	1	0	0	0	0	1									
B*44:76	44:76	HLA04056		0	0	21	0	1	0	1	23			WD					WD	WD
B*44:77	44:77	HLA04082		0	0	11	0	0	0	0	11			WD					WD	WD
B*44:78	44:78	HLA04085		0	0	1	0	0	0	0	1									
**B*44:79 total**	**44:79 total**			**0**	**0**	**4**	**0**	**0**	**0**	**0**	**4**									
B*44:79	44:79			0	0	1	0	0	0	0	1									
B*44:79:01	44:79:01	HLA04086		0	0	3	0	0	0	0	3									
B*44:80	44:80	HLA04096		0	0	15	0	0	0	0	15			WD					WD	WD
B*44:83	44:83	HLA04111		0	0	4	4	0	0	1	9								WD	WD
**B*44:84 total**	**44:84 total**			**1**	**0**	**3**	**1**	**0**	**0**	**0**	**5**								**WD**	**WD**
B*44:84:01	44:84:01	HLA04173		0	0	0	1	0	0	0	1									
B*44:84:02	44:84:02	HLA10150		1	0	3	0	0	0	0	4									
B*44:85	44:85	HLA04194		1	0	1	0	1	0	0	3									
B*44:86	44:86	HLA04215		0	0	41	0	0	0	0	41			WD					WD	WD
B*44:87	44:87	HLA04224		0	14	0	0	0	0	0	14		I						WD	I
B*44:88	44:88	HLA04228		0	0	7	0	0	0	0	7			WD					WD	WD
B*44:89	44:89	HLA04455		0	0	4	0	0	0	0	4									
B*44:91	44:91	HLA04501		0	0	10	0	0	0	1	11			WD					WD	WD
B*44:92	44:92	HLA04514		0	0	6	0	0	0	0	6			WD					WD	WD
B*44:95	44:95	HLA04536		0	0	4	0	0	0	0	4									
B*44:96	44:96	HLA04540		0	0	8	0	0	0	0	8			WD					WD	WD
B*44:98	44:98	HLA04542		0	0	3	0	1	0	9	13							WD	WD	WD
B*44:99	44:99	HLA04556		0	0	4	0	1	0	3	8								WD	WD
B*44:103	44:103	HLA05049		0	0	0	1	1	0	0	2									
B*44:104	44:104	HLA05052		0	0	5	0	0	0	0	5			WD					WD	WD
B*44:105	44:105	HLA05055		0	0	2	0	0	0	1	3									
B*44:107	44:107	HLA05062		0	0	5	0	0	0	0	5			WD					WD	WD
B*44:109	44:109	HLA05099		0	0	1	0	0	0	0	1									
B*44:110	44:110	HLA05406		0	0	3	0	0	0	0	3									
B*44:111	44:111	HLA05534		0	0	7	0	0	0	0	7			WD					WD	WD
B*44:112	44:112	HLA05717		0	1	0	0	0	0	0	1									

Supplemental Table 9: HLA-B Allele Summary [a]				Allele Count by Population Group [b]								3.0.0 CIWD Category by Population Group [c]								
Allele	Genomic typing	Allele ID	G group	AFA	API	EURO	MENA	HIS	NAM	UNK	Total	AFA	API	EURO	MENA	HIS	NAM	UNK	Total	Highest Frequency
B*44:113	44:113	HLA05829		0	0	1	0	0	0	0	1									
B*44:115	44:115	HLA05877		0	0	10	0	0	0	2	12			WD					WD	WD
B*44:116	44:116	HLA05926		0	0	6	0	0	0	0	6			WD					WD	WD
B*44:117	44:117	HLA06045		0	0	0	0	0	0	1	1									
B*44:120	44:120	HLA06218		0	0	7	0	0	0	0	7			WD					WD	WD
B*44:122	44:122	HLA06244		0	0	8	0	1	0	0	9			WD					WD	WD
B*44:124	44:124	HLA06246		0	3	0	0	0	0	0	3									
B*44:125	44:125	HLA06247		0	0	1	0	0	0	0	1									
**B*44:126 total**	**44:126 total**			**0**	**0**	**1**	**0**	**0**	**0**	**0**	**1**									
B*44:126	44:126			0	0	1	0	0	0	0	1									
B*44:130	44:130	HLA06642		0	0	0	0	0	0	1	1									
B*44:132	44:132	HLA06976		0	0	2	0	0	0	0	2									
B*44:135	44:135	HLA07090		0	0	2	0	0	0	0	2									
B*44:137	44:137	HLA07194		0	1	2	0	0	0	0	3									
B*44:138Q	44:138Q	HLA07343		0	0	1	0	0	0	1	2									
B*44:140	44:140	HLA07447		0	0	1	0	0	0	0	1									
B*44:143	44:143	HLA07451		0	2	0	0	0	0	0	2									
B*44:145	44:145	HLA07642		0	0	1	0	0	0	0	1									
B*44:146	44:146	HLA07696		0	0	9	0	0	0	0	9			WD					WD	WD
B*44:147	44:147	HLA07697		0	0	1	0	0	0	0	1									
B*44:148	44:148	HLA07698		1	0	1	0	0	0	0	2									
B*44:152	44:152	HLA07812		0	0	4	0	0	0	0	4									
B*44:153	44:153	HLA08060		0	1	0	0	0	0	0	1									
B*44:155	44:155	HLA08131		0	0	1	0	0	0	0	1									
B*44:158	44:158	HLA08349		0	0	1	0	0	0	0	1									
B*44:163	44:163	HLA08674		0	0	0	0	0	0	2	2									
B*44:165	44:165	HLA08687		0	0	0	0	0	0	2	2									
B*44:166	44:166	HLA08809		0	0	6	0	0	0	0	6			WD					WD	WD
B*44:169	44:169	HLA08985		0	0	3	0	0	0	0	3									
B*44:170	44:170	HLA08986		0	0	1	0	0	0	0	1									
B*44:177	44:177	HLA09594		0	0	3	0	0	0	1	4									

Supplemental Table 9: HLA-B Allele Summary [a]				Allele Count by Population Group [b]								3.0.0 CIWD Category by Population Group [c]								
Allele	Genomic typing	Allele ID	G group	AFA	API	EURO	MENA	HIS	NAM	UNK	Total	AFA	API	EURO	MENA	HIS	NAM	UNK	Total	Highest Frequency
B*44:180	44:180	HLA09706		0	0	2	0	0	0	0	2									
B*44:181	44:181	HLA09711		0	1	0	0	0	0	0	1									
B*44:182	44:182	HLA10143		0	0	1	0	0	0	0	1									
B*44:185	44:185	HLA10406		0	0	2	0	0	0	0	2									
B*44:186	44:186	HLA10407		0	0	2	0	0	0	0	2									
B*44:188	44:188	HLA10992		0	0	3	0	0	0	0	3									
B*44:189	44:189	HLA11015		0	0	1	0	1	0	0	2									
B*44:191	44:191	HLA11017		1	0	0	0	0	0	0	1									
**B*44:192 total**	**44:192 total**			**0**	**1**	**1**	**0**	**0**	**0**	**0**	**2**									
B*44:192	44:192			0	1	1	0	0	0	0	2									
B*44:197	44:197	HLA11824		0	0	0	1	0	0	0	1									
B*44:200	44:200	HLA12003		0	0	1	0	0	0	0	1									
**B*44:205 total**	**44:205 total**			**0**	**1**	**5**	**0**	**0**	**0**	**0**	**6**			**WD**					**WD**	**WD**
B*44:205:01	44:205:01	HLA12270		0	0	5	0	0	0	0	5			WD					WD	WD
B*44:205:02	44:205:02	HLA12987		0	1	0	0	0	0	0	1									
B*44:206	44:206	HLA12272		0	0	1	0	0	0	0	1									
**B*44:210 total**	**44:210 total**			**0**	**0**	**1**	**0**	**0**	**0**	**0**	**1**									
B*44:210	44:210			0	0	1	0	0	0	0	1									
B*44:212	44:212	HLA12424		0	0	1	0	0	0	0	1									
B*44:215	44:215	HLA12991		0	0	0	0	1	0	0	1									
B*44:216	44:216	HLA12995		0	0	1	0	0	0	0	1									
B*44:217N	44:217N	HLA12992		0	0	1	0	0	0	0	1									
B*44:221	44:221	HLA13539		0	0	2	0	0	0	0	2									
B*44:223	44:223	HLA13656		0	0	0	0	0	0	1	1									
B*44:226	44:226	HLA14005		0	0	2	0	0	0	0	2									
B*44:227	44:227	HLA14007		0	3	0	0	0	0	0	3									
B*44:229	44:229	HLA14079		0	0	3	0	0	0	0	3									
B*44:247	44:247	HLA15270		0	1	0	0	0	0	0	1									
B*44:254	44:254	HLA15555		0	0	0	0	0	0	1	1									
B*44:258	44:258	HLA15879		0	0	0	0	0	0	3	3									
B*44:260	44:260	HLA16242		0	0	1	0	0	0	0	1									

Supplemental Table 9: HLA-B Allele Summary [a]				Allele Count by Population Group [b]								3.0.0 CIWD Category by Population Group [c]								
Allele	Genomic typing	Allele ID	G group	AFA	API	EURO	MENA	HIS	NAM	UNK	Total	AFA	API	EURO	MENA	HIS	NAM	UNK	Total	Highest Frequency
B*44:CODE	44:CODE			1699	1330	81624	744	5381	441	9051	100270	NA	NA	NA	NA	NA	NA	NA	NA	NA
**B*45:01 total**	**45:01 total**			**17480**	**985**	**53933**	**1981**	**10947**	**1240**	**10826**	**97392**	**C**	**C**	**C**	**C**	**C**	**C**	**C**	**C**	**C**
B*45:01	45:01			3	0	235	2	1	0	10	251			I				WD	I	I
B*45:01P	45:01P			1	1	89	3	1	0	1	96			WD					WD	WD
**B*45:01:01G total**	**45:01:01G total**			**17474**	**984**	**53609**	**1976**	**10945**	**1240**	**10815**	**97043**	**C**	**C**	**C**	**C**	**C**	**C**	**C**	**C**	**C**
B*45:01:01G	45:01:01G		45:01:01G	13681	862	50170	1878	7809	895	9218	84513	C	C	C	C	C	C	C	C	C
B*45:01:01	45:01:01	HLA00329	45:01:01G	3790	122	3439	98	3136	345	1597	12527	C	I	C	C	C	C	C	C	C
B*45:07	45:07	HLA01940	45:01:01G	3	0	0	0	0	0	0	3									
B*45:01:02	45:01:02	HLA11318		1	0	0	0	0	0	0	1									
B*45:01:03	45:01:03	HLA18018		1	0	0	0	0	0	0	1									
B*45:02	45:02	HLA00330		8	0	0	0	0	0	5	13	WD						WD	WD	WD
B*45:04	45:04	HLA01343		1	0	0	0	2	0	0	3									
B*45:06	45:06	HLA01607		1	0	0	0	0	0	0	1									
B*45:08	45:08	HLA03258		0	0	1	0	0	0	0	1									
B*45:09	45:09	HLA03299		2	0	1	0	0	0	0	3									
B*45:11	45:11	HLA04481		0	0	2	0	0	0	0	2									
B*45:14	45:14	HLA07683		0	0	4	0	0	0	0	4									
B*45:16	45:16	HLA13735		1	0	0	0	0	0	0	1									
B*45:17	45:17	HLA13964		0	0	0	0	1	0	0	1									
B*45:CODE	45:CODE			426	18	1623	39	305	30	299	2740	NA	NA	NA	NA	NA	NA	NA	NA	NA
**B*46:01 total**	**46:01 total**			**41**	**28921**	**2163**	**387**	**111**	**8**	**3030**	**34661**	**C**	**C**	**C**	**C**	**C**	**C**	**C**	**C**	**C**
B*46:01	46:01			0	7	12	0	1	0	4	24		WD	WD					WD	WD
B*46:01P	46:01P			0	1	3	0	0	0	0	4									
**B*46:01:01G total**	**46:01:01G total**			**41**	**28904**	**2148**	**387**	**110**	**8**	**3024**	**34622**	**C**	**C**	**C**	**C**	**C**	**C**	**C**	**C**	**C**
B*46:01:01G	46:01:01G		46:01:01G	30	21753	2092	377	81	4	2093	26430	WD	C	C	C	C		C	C	C
B*46:01:01	46:01:01	HLA00331	46:01:01G	11	7151	56	10	29	4	931	8192	WD	C	WD	WD	I		C	C	C
B*46:01:02	46:01:02	HLA02685		0	6	0	0	0	0	2	8		WD						WD	WD
B*46:01:03	46:01:03	HLA04503		0	1	0	0	0	0	0	1									
B*46:01:07	46:01:07	HLA06360		0	1	0	0	0	0	0	1									
B*46:01:18	46:01:18	HLA10923		0	1	0	0	0	0	0	1									
B*46:11	46:11	HLA02905		0	2	0	0	0	0	1	3									

Supplemental Table 9: HLA-B Allele Summary [a]				Allele Count by Population Group [b]								3.0.0 CIWD Category by Population Group [c]								
Allele	Genomic typing	Allele ID	G group	AFA	API	EURO	MENA	HIS	NAM	UNK	Total	AFA	API	EURO	MENA	HIS	NAM	UNK	Total	Highest Frequency
**B*46:13 total**	**46:13 total**			**0**	**1**	**0**	**0**	**0**	**0**	**0**	**1**									
B*46:13:01	46:13:01	HLA03249		0	1	0	0	0	0	0	1									
B*46:14	46:14	HLA03271		0	0	1	0	0	0	0	1									
B*46:20	46:20	HLA03909		0	0	0	0	0	0	1	1									
B*46:22	46:22	HLA04163		0	7	0	0	0	0	0	7		WD						WD	WD
B*46:29	46:29	HLA07280		0	1	0	0	0	0	0	1									
B*46:32	46:32	HLA08314		0	0	2	0	0	0	0	2									
B*46:43	46:43	HLA10033		0	1	0	0	0	0	0	1									
B*46:45	46:45	HLA10489		0	1	0	0	0	0	0	1									
B*46:61	46:61	HLA12286		0	1	0	0	0	0	0	1									
B*46:62	46:62	HLA12287		0	2	0	0	0	0	0	2									
B*46:63	46:63	HLA12288		0	1	0	0	0	0	0	1									
B*46:CODE	46:CODE			11	1913	191	34	12	4	255	2420	NA	NA	NA	NA	NA	NA	NA	NA	NA
**B*47:01 total**	**47:01 total**			**597**	**632**	**34780**	**782**	**1540**	**124**	**3143**	**41598**	**C**	**C**	**C**	**C**	**C**	**C**	**C**	**C**	**C**
B*47:01	47:01			338	86	9082	184	750	79	1318	11837	C	I	C	C	C	C	C	C	C
B*47:01P	47:01P			0	0	7	0	0	0	0	7			WD					WD	WD
**B*47:01:01G total**	**47:01:01G total**			**259**	**546**	**25690**	**598**	**790**	**45**	**1825**	**29753**	**C**	**C**	**C**	**C**	**C**	**C**	**C**	**C**	**C**
B*47:01:01G	47:01:01G		47:01:01G	152	476	23547	558	347	13	1525	26618	C	C	C	C	C	C	C	C	C
B*47:01:01	47:01:01		47:01:01G	23	24	955	13	138	10	147	1310	WD	I	I	WD	C	C	C	I	C
B*47:01:01:03	47:01:01:03	HLA14088	47:01:01G	84	46	1188	27	305	22	153	1825	C	I	I	WD	C	C	C	C	C
B*47:01:03	47:01:03	HLA16392		0	0	1	0	0	0	0	1									
B*47:02	47:02	HLA00333		6	3	1062	0	1	1	10	1083	WD		I				WD	I	I
B*47:03	47:03	HLA00334		261	0	53	159	7	1	48	529	C		WD	C	WD		I	I	C
B*47:04	47:04	HLA01481		0	0	6	0	0	0	6	12			WD				WD	WD	WD
B*47:05	47:05	HLA01953		4	0	0	0	0	0	1	5								WD	WD
B*47:08	47:08	HLA06219		0	0	3	0	0	0	0	3									
B*47:10	47:10	HLA12994		0	0	0	0	2	0	0	2									
B*47:CODE	47:CODE			9	0	762	1	22	2	49	845	NA	NA	NA	NA	NA	NA	NA	NA	NA
**B*48:01 total**	**48:01 total**			**203**	**9146**	**8134**	**960**	**12351**	**898**	**5162**	**36854**	**C**	**C**	**C**	**C**	**C**	**C**	**C**	**C**	**C**
B*48:01	48:01			0	1	37	0	7	0	3	48			WD		WD			WD	WD
B*48:01P	48:01P			0	0	4	0	0	0	0	4									

Supplemental Table 9: HLA-B Allele Summary [a]				Allele Count by Population Group [b]								3.0.0 CIWD Category by Population Group [c]								
Allele	Genomic typing	Allele ID	G group	AFA	API	EURO	MENA	HIS	NAM	UNK	Total	AFA	API	EURO	MENA	HIS	NAM	UNK	Total	Highest Frequency
**B*48:01:01G total**	**48:01:01G total**			**203**	**9145**	**8093**	**960**	**12344**	**898**	**5159**	**36802**	**C**	**C**	**C**	**C**	**C**	**C**	**C**	**C**	**C**
B*48:01:01G	48:01:01G		48:01:01G	141	7429	7700	942	8526	628	4234	29600	C	C	C	C	C	C	C	C	C
B*48:01:01	48:01:01		48:01:01G	62	1716	393	18	3818	270	925	7202	C	C	I	WD	C	C	C	C	C
**B*48:02 total**	**48:02 total**			**40**	**5**	**321**	**1**	**677**	**120**	**423**	**1587**	**C**	**WD**	**I**		**C**	**C**	**C**	**I**	**C**
B*48:02	48:02			2	0	121	0	124	15	82	344			I		C	C	I	I	C
B*48:02:01	48:02:01	HLA00336		38	5	199	1	547	104	341	1235	WD	WD	I		C	C	C	I	C
B*48:02:02	48:02:02	HLA04461		0	0	0	0	1	1	0	2									
B*48:02:03	48:02:03	HLA08378		0	0	1	0	5	0	0	6					WD			WD	WD
**B*48:03 total**	**48:03 total**			**5**	**842**	**184**	**1**	**526**	**27**	**296**	**1881**	**WD**	**C**	**I**		**C**	**C**	**C**	**C**	**C**
B*48:03	48:03			0	45	42	0	64	2	39	192		I	WD		I		I	I	I
B*48:03:01	48:03:01	HLA00337		5	797	140	1	442	25	256	1666	WD	C	I		C	C	C	C	C
B*48:03:02	48:03:02	HLA02727		0	0	2	0	20	0	1	23					I			WD	I
**B*48:04 total**	**48:04 total**			**0**	**3736**	**9**	**0**	**1**	**0**	**104**	**3850**		**C**	**WD**				**I**	**C**	**C**
B*48:04	48:04			0	1372	3	0	1	0	75	1451		C					I	I	C
B*48:04:01	48:04:01	HLA00338		0	2363	6	0	0	0	29	2398		C	WD				I	C	C
B*48:04:02	48:04:02	HLA13543		0	1	0	0	0	0	0	1									
B*48:05	48:05	HLA00339		20	0	0	11	0	0	2	33	WD			WD				WD	WD
B*48:06	48:06	HLA00986		3	1	0	0	5	0	1	10					WD			WD	WD
B*48:07	48:07	HLA01069		2	1	6	0	7	13	24	53			WD		WD	C	I	WD	C
B*48:11	48:11	HLA02104		0	1	0	0	0	0	0	1									
B*48:12	48:12	HLA02112		1	0	4	0	19	2	2	28					I			WD	I
B*48:13	48:13	HLA02200		1	0	2	0	10	1	3	17					I			WD	I
B*48:20	48:20	HLA04245		0	1	0	0	0	0	0	1									
B*48:21	48:21	HLA04433		0	0	0	0	0	0	1	1									
B*48:24	48:24	HLA05475		0	6	0	0	0	0	0	6		WD						WD	WD
B*48:26	48:26	HLA07209		0	0	2	0	0	0	6	8							WD	WD	WD
B*48:27	48:27	HLA07210		0	0	0	0	1	0	0	1									
B*48:29	48:29	HLA08903		0	1	0	0	3	1	2	7								WD	WD
B*48:33	48:33	HLA10421		0	1	0	0	0	0	0	1									
B*48:36	48:36	HLA12018		0	0	0	0	1	0	0	1									
B*48:38	48:38	HLA14316		0	0	0	0	1	0	0	1									

Supplemental Table 9: HLA-B Allele Summary [a]				Allele Count by Population Group [b]								3.0.0 CIWD Category by Population Group [c]								
Allele	Genomic typing	Allele ID	G group	AFA	API	EURO	MENA	HIS	NAM	UNK	Total	AFA	API	EURO	MENA	HIS	NAM	UNK	Total	Highest Frequency
B*48:39	48:39	HLA14985		0	0	1	0	1	0	2	4									
B*48:41	48:41	HLA15642		0	1	0	0	0	0	0	1									
B*48:CODE	48:CODE			15	224	936	48	639	33	306	2201	NA	NA	NA	NA	NA	NA	NA	NA	NA
**B*49:01 total**	**49:01 total**			**12237**	**5417**	**168106**	**14358**	**15754**	**1339**	**24188**	**241399**	**C**	**C**	**C**	**C**	**C**	**C**	**C**	**C**	**C**
B*49:01	49:01			5220	466	31050	2845	6409	629	9538	56157	C	C	C	C	C	C	C	C	C
B*49:01P	49:01P			1	0	116	1	2	0	0	120			WD					WD	WD
**B*49:01:01G total**	**49:01:01G total**			**7016**	**4951**	**136925**	**11511**	**9343**	**710**	**14650**	**185106**	**C**	**C**	**C**	**C**	**C**	**C**	**C**	**C**	**C**
B*49:01:01G	49:01:01G		49:01:01G	2432	4038	105974	9434	3407	106	8544	133935	C	C	C	C	C	C	C	C	C
B*49:01:01	49:01:01		49:01:01G	4584	913	30947	2076	5936	604	6106	51166	C	C	C	C	C	C	C	C	C
B*49:01:01:01	49:01:01:01	HLA00340	49:01:01G	0	0	2	1	0	0	0	3									
B*49:01:01:02	49:01:01:02	HLA17557	49:01:01G	0	0	2	0	0	0	0	2									
B*49:01:06	49:01:06	HLA10996		0	0	1	0	0	0	0	1									
B*49:38	49:38	HLA13402		0	0	14	1	0	0	0	15			WD					WD	WD
B*49:02	49:02	HLA01252		0	0	2	0	1	0	1	4									
**B*49:04 total**	**49:04 total**			**0**	**0**	**6**	**0**	**0**	**0**	**0**	**6**			**WD**					**WD**	**WD**
B*49:04	49:04			0	0	4	0	0	0	0	4									
B*49:04:01	49:04:01	HLA02209		0	0	2	0	0	0	0	2									
B*49:06	49:06	HLA04098		0	0	1	0	0	0	0	1									
B*49:09	49:09	HLA04538		0	0	0	8	0	0	0	8				WD				WD	WD
B*49:10	49:10	HLA04706		0	0	0	0	2	0	0	2									
B*49:11	49:11	HLA05484		0	0	2	2	0	0	0	4									
B*49:16	49:16	HLA06227		0	0	3	0	1	0	0	4									
B*49:17	49:17	HLA06352		0	0	0	0	1	0	0	1									
**B*49:18 total**	**49:18 total**			**0**	**0**	**4**	**0**	**6**	**0**	**8**	**18**					**WD**		**WD**	**WD**	**WD**
B*49:18:02	49:18:02	HLA14986		0	0	4	0	6	0	8	18					WD		WD	WD	WD
B*49:20	49:20	HLA06932		0	0	1	0	0	0	0	1									
B*49:21	49:21	HLA07958		0	0	1	0	0	0	0	1									
B*49:23	49:23	HLA08324		0	0	1	0	0	0	0	1									
B*49:25	49:25	HLA08670		0	0	0	0	0	0	3	3									
B*49:31	49:31	HLA11445		0	0	1	0	0	0	1	2									
B*49:37	49:37	HLA12975		1	0	0	0	0	0	0	1									

Supplemental Table 9: HLA-B Allele Summary [a]				Allele Count by Population Group [b]								3.0.0 CIWD Category by Population Group [c]								
Allele	Genomic typing	Allele ID	G group	AFA	API	EURO	MENA	HIS	NAM	UNK	Total	AFA	API	EURO	MENA	HIS	NAM	UNK	Total	Highest Frequency
B*49:41	49:41	HLA14433		0	0	1	0	0	0	0	1									
B*49:CODE	49:CODE			402	46	9682	375	978	49	1369	12901	NA	NA	NA	NA	NA	NA	NA	NA	NA
**B*50:01 total**	**50:01 total**			**3639**	**13457**	**124242**	**20314**	**9468**	**863**	**19612**	**191595**	**C**	**C**	**C**	**C**	**C**	**C**	**C**	**C**	**C**
B*50:01	50:01			86	484	7035	790	205	17	591	9208	C	C	C	C	C	C	C	C	C
B*50:01P	50:01P			0	2	231	1	1	0	1	236			I					I	I
**B*50:01:01G total**	**50:01:01G total**			**3553**	**12970**	**116975**	**19523**	**9262**	**846**	**19020**	**182149**	**C**	**C**	**C**	**C**	**C**	**C**	**C**	**C**	**C**
B*50:01:01G	50:01:01G		50:01:01G	1500	9231	75060	14967	4127	258	11806	116949	C	C	C	C	C	C	C	C	C
B*50:01:01	50:01:01		50:01:01G	1939	3663	40965	4468	4765	566	7025	63391	C	C	C	C	C	C	C	C	C
B*50:01:01:01	50:01:01:01	HLA00341	50:01:01G	79	74	819	88	275	19	160	1514	C	I	I	C	C	C	C	I	C
B*50:01:01:02	50:01:01:02	HLA16418	50:01:01G	35	2	131	0	95	3	29	295	WD		I		C		I	I	C
B*50:01:02	50:01:02	HLA04100		0	0	1	0	0	0	0	1									
B*50:01:04	50:01:04	HLA10514		0	1	0	0	0	0	0	1									
B*50:02	50:02	HLA00342		64	35	3479	422	1460	50	1653	7163	C	I	C	C	C	C	C	C	C
B*50:04	50:04	HLA01070		0	61	1	0	1	0	0	63		I						WD	I
B*50:06	50:06	HLA04108		0	0	0	0	0	0	1	1									
B*50:07	50:07	HLA04180		0	1	0	1	0	0	0	2									
B*50:08	50:08	HLA04182		0	0	9	0	0	0	0	9			WD					WD	WD
B*50:09	50:09	HLA04239		0	0	5	0	0	0	0	5			WD					WD	WD
B*50:12	50:12	HLA05499		0	0	13	0	0	0	0	13			WD					WD	WD
B*50:14	50:14	HLA06835		0	0	1	0	0	0	0	1									
B*50:17	50:17	HLA07968		1	0	0	0	0	0	0	1									
B*50:18	50:18	HLA08559		0	0	2	0	0	0	0	2									
B*50:32	50:32	HLA09316		0	0	0	0	1	0	0	1									
B*50:35	50:35	HLA10399		0	0	0	0	1	0	0	1									
B*50:40	50:40	HLA12560		0	0	1	0	0	0	0	1									
B*50:41	50:41	HLA12561		0	0	0	1	0	0	0	1									
B*50:44	50:44	HLA14601		0	0	1	0	0	0	0	1									
B*50:CODE	50:CODE			138	93	5766	191	583	30	762	7563	NA	NA	NA	NA	NA	NA	NA	NA	NA
**B*51:01 total**	**51:01 total**			**8927**	**89847**	**620443**	**47811**	**38568**	**3716**	**66227**	**875539**	**C**	**C**	**C**	**C**	**C**	**C**	**C**	**C**	**C**
B*51:01	51:01			7	129	4598	22	17	0	198	4971	WD	I	C	WD	I		C	C	C
B*51:01P	51:01P			1	11	1390	5	7	0	6	1420		WD	C	WD	WD		WD	I	C

Supplemental Table 9: HLA-B Allele Summary [a]				Allele Count by Population Group [b]								3.0.0 CIWD Category by Population Group [c]								
**Allele**	**Genomic typing**	**Allele ID**	**G group**	**AFA**	**API**	**EURO**	**MENA**	**HIS**	**NAM**	**UNK**	**Total**	**AFA**	**API**	**EURO**	**MENA**	**HIS**	**NAM**	**UNK**	**Total**	**Highest Frequency**
**B*51:01:01G total**	**51:01:01G total**			**8845**	**87187**	**614250**	**47767**	**38508**	**3715**	**65481**	**865753**	**C**	**C**	**C**	**C**	**C**	**C**	**C**	**C**	**C**
B*51:01:01G	51:01:01G		51:01:01G	6671	79969	584231	46826	26742	2623	59421	806483	C	C	C	C	C	C	C	C	C
B*51:01:01	51:01:01		51:01:01G	959	2796	11089	408	5048	387	2905	23592	C	C	C	C	C	C	C	C	C
B*51:01:01:01	51:01:01:01	HLA00344	51:01:01G	1208	4319	18724	529	6624	700	3118	35222	C	C	C	C	C	C	C	C	C
B*51:01:01:02	51:01:01:02	HLA12698	51:01:01G	0	0	2	1	0	0	0	3									
B*51:01:01:03	51:01:01:03	HLA16059	51:01:01G	0	0	5	0	0	0	0	5			WD					WD	WD
B*51:01:01:04	51:01:01:04	HLA16294	51:01:01G	0	0	34	0	0	0	0	34			WD					WD	WD
B*51:01:01:05	51:01:01:05	HLA16295	51:01:01G	0	0	1	0	0	0	0	1									
B*51:01:01:06	51:01:01:06	HLA16296	51:01:01G	0	0	4	0	0	0	0	4									
B*51:01:01:09	51:01:01:09	HLA16300	51:01:01G	0	0	1	0	0	0	0	1									
B*51:01:01:10	51:01:01:10	HLA16301	51:01:01G	0	0	2	0	0	0	0	2									
B*51:01:01:11	51:01:01:11	HLA16302	51:01:01G	0	0	8	0	0	0	0	8			WD					WD	WD
B*51:01:01:14	51:01:01:14	HLA16688	51:01:01G	0	0	27	1	0	0	4	32			WD					WD	WD
B*51:01:01:17	51:01:01:17	HLA16691	51:01:01G	0	0	1	0	0	0	0	1									
B*51:01:01:18	51:01:01:18	HLA16692	51:01:01G	0	0	1	0	0	0	0	1									
B*51:01:01:19	51:01:01:19	HLA16693	51:01:01G	0	0	2	0	0	0	0	2									
B*51:01:01:20	51:01:01:20	HLA16694	51:01:01G	1	1	0	0	0	0	0	2									
B*51:01:01:21	51:01:01:21	HLA16695	51:01:01G	0	0	16	0	2	0	3	21			WD					WD	WD
B*51:01:05	51:01:05	HLA01579	51:01:01G	1	98	2	0	2	0	7	110		I					WD	WD	I
B*51:01:07	51:01:07	HLA02232	51:01:01G	2	0	24	0	18	1	6	51			WD		I		WD	WD	I
B*51:01:35	51:01:35	HLA08395	51:01:01G	0	0	2	0	17	0	1	20					I			WD	I
B*51:01:44	51:01:44	HLA10460	51:01:01G	0	1	0	0	0	0	1	2									
B*51:01:56	51:01:56	HLA16744	51:01:01G	0	0	1	0	0	0	1	2									
B*51:11N	51:11N	HLA00356	51:01:01G	2	0	17	0	2	0	3	24			WD					WD	WD
B*51:32	51:32	HLA01657	51:01:01G	1	0	9	2	16	1	5	34			WD		I		WD	WD	I
B*51:48	51:48	HLA02885	51:01:01G	0	1	0	0	0	0	0	1									
B*51:51	51:51	HLA03123	51:01:01G	0	1	6	0	0	0	1	8			WD					WD	WD
B*51:193	51:193	HLA13998	51:01:01G	0	1	41	0	37	3	5	87			WD		I		WD	WD	I
B*51:01:02	51:01:02	HLA00345		72	2474	119	13	32	1	524	3235	C	C	WD	WD	I		C	C	C
B*51:01:03	51:01:03	HLA01126		0	0	20	0	2	0	3	25			WD					WD	WD
B*51:01:04	51:01:04	HLA01149		1	2	27	0	0	0	1	31			WD					WD	WD

Supplemental Table 9: HLA-B Allele Summary [a]				Allele Count by Population Group [b]								3.0.0 CIWD Category by Population Group [c]								
Allele	Genomic typing	Allele ID	G group	AFA	API	EURO	MENA	HIS	NAM	UNK	Total	AFA	API	EURO	MENA	HIS	NAM	UNK	Total	Highest Frequency
B*51:01:09	51:01:09	HLA03273		0	30	0	0	0	0	6	36		I					WD	WD	I
B*51:01:10	51:01:10	HLA03797		0	0	8	0	0	0	1	9			WD					WD	WD
B*51:01:12	51:01:12	HLA04206		0	0	0	1	0	0	0	1									
B*51:01:13	51:01:13	HLA04235		0	1	0	0	0	0	0	1									
B*51:01:14	51:01:14	HLA04505		0	1	0	0	0	0	0	1									
B*51:01:15	51:01:15	HLA04513		0	0	0	0	1	0	0	1									
B*51:01:19	51:01:19	HLA05494		0	1	27	0	0	0	1	29			WD					WD	WD
B*51:01:20	51:01:20	HLA05715		0	1	0	2	0	0	2	5								WD	WD
B*51:01:21	51:01:21	HLA05998		0	0	3	0	1	0	1	5								WD	WD
B*51:01:22	51:01:22	HLA06023		0	0	1	0	0	0	0	1									
B*51:01:25	51:01:25	HLA06288		0	0	3	0	0	0	0	3									
B*51:01:26	51:01:26	HLA06364		0	0	1	0	1	0	1	3									
B*51:01:27	51:01:27	HLA06936		0	0	1	0	0	0	0	1									
B*51:01:28	51:01:28	HLA06979		0	0	3	1	0	0	0	4									
B*51:01:30	51:01:30	HLA07203		0	0	1	0	0	0	0	1									
B*51:01:32	51:01:32	HLA07655		0	0	3	0	1	0	1	5								WD	WD
B*51:01:33	51:01:33	HLA07656		0	0	1	0	0	0	1	2									
B*51:01:34	51:01:34	HLA07716		0	4	1	0	0	0	0	5								WD	WD
B*51:01:36	51:01:36	HLA08525		0	0	0	0	0	0	2	2									
B*51:01:37	51:01:37	HLA08575		0	3	0	0	0	0	0	3									
B*51:01:38	51:01:38	HLA08680		3	0	0	0	0	0	1	4									
B*51:01:42	51:01:42	HLA09584		0	0	1	0	0	0	0	1									
B*51:01:43	51:01:43	HLA10120		0	1	1	0	0	0	0	2									
B*51:01:50	51:01:50	HLA11834		0	1	0	0	0	0	0	1									
B*51:01:52	51:01:52	HLA13009		0	1	0	0	0	0	0	1									
B*51:01:53	51:01:53	HLA13011		0	0	1	0	0	0	0	1									
**B*51:02 total**	**51:02 total**			**331**	**3502**	**486**	**248**	**3017**	**274**	**994**	**8852**	**C**	**C**	**I**	**C**	**C**	**C**	**C**	**C**	**C**
B*51:02	51:02			20	182	44	6	348	33	57	690	WD	C	WD	WD	C	C	I	I	C
B*51:02P	51:02P			0	3	2	0	0	0	0	5								WD	WD
**B*51:02:01G total**	**51:02:01G total**			**251**	**2674**	**391**	**230**	**2661**	**238**	**824**	**7269**	**C**	**C**	**I**	**C**	**C**	**C**	**C**	**C**	**C**
B*51:02:01G	51:02:01G		51:02:01G	4	276	20	4	55	4	39	402		C	WD		I		I	I	C

Supplemental Table 9: HLA-B Allele Summary [a]				Allele Count by Population Group [b]								3.0.0 CIWD Category by Population Group [c]								
Allele	Genomic typing	Allele ID	G group	AFA	API	EURO	MENA	HIS	NAM	UNK	Total	AFA	API	EURO	MENA	HIS	NAM	UNK	Total	Highest Frequency
B*51:02:01	51:02:01		51:02:01G	247	2398	371	226	2606	234	785	6867	C	C	I	C	C	C	C	C	C
B*51:02:02	51:02:02	HLA00347		60	643	49	12	8	3	113	888	C	C	WD	WD	I		I	I	C
B*51:03	51:03	HLA00348		0	0	2	0	0	0	0	2									
B*51:04	51:04	HLA00349		5	2	30	7	57	2	42	145	WD		WD	WD	I		I	WD	I
B*51:05	51:05	HLA00350		7	21	4369	795	71	4	326	5593	WD	I	C	C	C		C	C	C
**B*51:06 total**	**51:06 total**			**20**	**9586**	**132**	**38**	**26**	**18**	**406**	**10226**	**WD**	**C**	**I**	**WD**	**I**	**C**	**C**	**C**	**C**
B*51:06	51:06			3	509	6	1	2	0	18	539		C	WD				I	I	C
B*51:06P	51:06P			0	1	0	0	0	0	0	1									
B*51:06:01	51:06:01	HLA00351		17	9076	100	37	24	18	388	9660	WD	C	WD	WD	I	C	C	C	C
B*51:06:02	51:06:02	HLA06268		0	0	26	0	0	0	0	26			WD					WD	WD
**B*51:07 total**	**51:07 total**			**49**	**1078**	**8067**	**1269**	**484**	**34**	**1044**	**12025**	**C**	**C**	**C**	**C**	**C**	**C**	**C**	**C**	**C**
B*51:07	51:07			2	53	626	107	75	4	64	931		I	I	C	C		I	I	C
B*51:07P	51:07P			0	0	2	0	0	0	0	2									
B*51:07:01	51:07:01	HLA00352		47	1025	7439	1162	409	30	980	11092	C	C	C	C	C	C	C	C	C
**B*51:08 total**	**51:08 total**			**228**	**752**	**14658**	**2259**	**1118**	**150**	**3076**	**22241**	**C**	**C**	**C**	**C**	**C**	**C**	**C**	**C**	**C**
B*51:08	51:08			72	137	5031	645	413	73	1022	7393	C	C	C	C	C	C	C	C	C
B*51:08P	51:08P			0	0	14	0	0	0	0	14			WD					WD	WD
B*51:08:01	51:08:01	HLA00353		156	615	9612	1614	705	77	2054	14833	C	C	C	C	C	C	C	C	C
B*51:08:02	51:08:02	HLA11032		0	0	1	0	0	0	0	1									
**B*51:09 total**	**51:09 total**			**220**	**57**	**2234**	**61**	**97**	**16**	**315**	**3000**	**C**	**I**	**C**	**C**	**C**	**C**	**C**	**C**	**C**
B*51:09	51:09			13	1	129	4	11	1	19	178	WD		I		I		I	I	I
B*51:09:01	51:09:01	HLA00354		207	56	2105	57	85	11	291	2812	C	I	C	C	C	C	C	C	C
B*51:09:02	51:09:02	HLA03105		0	0	0	0	1	4	5	10							WD	WD	WD
B*51:10	51:10	HLA00355		4	5	7	0	70	3	13	102		WD	WD		I		WD	WD	I
B*51:12	51:12	HLA00357		0	0	1	0	0	0	0	1									
**B*51:13 total**	**51:13 total**			**0**	**3**	**72**	**1**	**218**	**16**	**107**	**417**			**WD**		**C**	**C**	**I**	**I**	**C**
B*51:13	51:13			0	1	24	0	46	2	25	98			WD		I		I	WD	I
B*51:13P	51:13P			0	0	1	0	0	0	0	1									
B*51:13:01	51:13:01	HLA00358		0	2	46	0	123	8	75	254			WD		C	C	I	I	C
B*51:13:02	51:13:02	HLA01127		0	0	1	1	49	6	7	64					I	WD	WD	WD	I
B*51:14	51:14	HLA00359		6	1	55	0	280	21	97	460	WD		WD		C	C	I	I	C

Supplemental Table 9: HLA-B Allele Summary [a]				Allele Count by Population Group [b]								3.0.0 CIWD Category by Population Group [c]								
Allele	Genomic typing	Allele ID	G group	AFA	API	EURO	MENA	HIS	NAM	UNK	Total	AFA	API	EURO	MENA	HIS	NAM	UNK	Total	Highest Frequency
B*51:16	51:16	HLA00361		0	0	1	0	0	0	0	1									
B*51:17	51:17	HLA01071		0	23	0	0	0	1	1	25		I						WD	I
B*51:18	51:18	HLA01072		0	0	1	0	0	0	0	1									
B*51:19	51:19	HLA01073		0	342	68	0	12	1	15	438		C	WD		I		I	I	C
B*51:20	51:20	HLA01132		0	0	38	0	2	0	2	42			WD					WD	WD
B*51:21	51:21	HLA01134		0	1	13	3	0	0	0	17			WD					WD	WD
B*51:22	51:22	HLA01243		3	5	257	1	107	9	26	408		WD	I		C	C	I	I	C
B*51:23	51:23	HLA01245		1	3	1	1	10	1	5	22					I		WD	WD	I
**B*51:24 total**	**51:24 total**			**0**	**0**	**21**	**1**	**0**	**0**	**0**	**22**			**WD**					**WD**	**WD**
B*51:24	51:24			0	0	3	0	0	0	0	3									
B*51:24:03	51:24:03	HLA04518		0	0	4	0	0	0	0	4									
B*51:24:04	51:24:04	HLA05723		0	0	7	1	0	0	0	8			WD					WD	WD
B*51:24:05	51:24:05	HLA10043		0	0	7	0	0	0	0	7			WD					WD	WD
B*51:26	51:26	HLA01417		0	0	38	0	1	0	2	41			WD					WD	WD
B*51:27N	51:27N	HLA01474		0	0	1	0	0	0	0	1									
B*51:29	51:29	HLA01568		0	0	25	8	2	0	3	38			WD	WD				WD	WD
B*51:31	51:31	HLA01626		1	1	10	0	29	2	14	57			WD		I		I	WD	I
B*51:33	51:33	HLA01676		0	0	4	1	0	0	0	5								WD	WD
B*51:34	51:34	HLA01678		1	30	20	11	0	0	5	67		I	WD	WD			WD	WD	I
B*51:36	51:36	HLA02003		0	7	0	0	0	0	1	8		WD						WD	WD
B*51:37	51:37	HLA02020		0	1	3	0	0	0	9	13							WD	WD	WD
B*51:38	51:38	HLA02167		0	0	48	0	0	0	1	49			WD					WD	WD
B*51:39	51:39	HLA02297		0	6	0	0	1	0	0	7		WD						WD	WD
B*51:40	51:40	HLA02419		0	0	14	0	0	0	4	18			WD					WD	WD
B*51:42	51:42	HLA02558		0	0	2	0	0	0	0	2									
B*51:43	51:43	HLA02620		0	0	108	0	1	0	64	173			WD				I	I	I
B*51:44N	51:44N	HLA02667		0	0	4	0	0	0	0	4									
B*51:46	51:46	HLA02754		0	0	18	0	0	0	0	18			WD					WD	WD
B*51:49	51:49	HLA02933		0	0	47	1	0	0	25	73			WD				I	WD	I
B*51:50	51:50	HLA03099		0	0	143	0	1	0	2	146			I					WD	I
B*51:53	51:53	HLA03277		1	0	0	0	0	0	0	1									

Supplemental Table 9: HLA-B Allele Summary [a]				Allele Count by Population Group [b]								3.0.0 CIWD Category by Population Group [c]								
**Allele**	**Genomic typing**	**Allele ID**	**G group**	**AFA**	**API**	**EURO**	**MENA**	**HIS**	**NAM**	**UNK**	**Total**	**AFA**	**API**	**EURO**	**MENA**	**HIS**	**NAM**	**UNK**	**Total**	**Highest Frequency**
B*51:54	51:54	HLA03279		0	0	0	1	0	0	0	1									
B*51:55	51:55	HLA03282		0	0	10	0	1	0	2	13			WD					WD	WD
**B*51:56 total**	**51:56 total**			**0**	**0**	**33**	**0**	**0**	**0**	**4**	**37**			**WD**					**WD**	**WD**
B*51:56	51:56			0	0	2	0	0	0	0	2									
B*51:56:01	51:56:01	HLA03313		0	0	12	0	0	0	4	16			WD					WD	WD
B*51:56:02	51:56:02	HLA05999		0	0	18	0	0	0	0	18			WD					WD	WD
B*51:56:03	51:56:03	HLA13344		0	0	1	0	0	0	0	1									
B*51:57	51:57	HLA03355		0	0	2	0	0	0	1	3									
B*51:58	51:58	HLA03456		0	1	0	0	0	0	0	1									
B*51:60	51:60	HLA03465		0	0	2	0	0	0	0	2									
**B*51:61 total**	**51:61 total**			**0**	**0**	**35**	**1**	**0**	**0**	**1**	**37**			**WD**					**WD**	**WD**
B*51:61	51:61			0	0	7	1	0	0	0	8			WD					WD	WD
B*51:61:01	51:61:01	HLA03468		0	0	26	0	0	0	1	27			WD					WD	WD
B*51:61:02	51:61:02	HLA08107		0	0	2	0	0	0	0	2									
**B*51:63 total**	**51:63 total**			**0**	**0**	**3**	**0**	**0**	**0**	**0**	**3**									
B*51:63	51:63			0	0	3	0	0	0	0	3									
B*51:64	51:64	HLA03608		11	0	4	0	0	0	0	15	WD							WD	WD
B*51:65	51:65	HLA03617		1	0	89	5	12	0	6	113			WD	WD	I		WD	WD	I
B*51:66	51:66	HLA03665		0	0	3	0	0	0	0	3									
B*51:67	51:67	HLA03674		0	0	8	0	0	0	0	8			WD					WD	WD
B*51:68	51:68	HLA03676		0	0	37	0	0	0	0	37			WD					WD	WD
B*51:69	51:69	HLA03716		0	1	5	31	0	0	2	39			WD	WD				WD	WD
B*51:70	51:70	HLA03719		0	0	1	8	0	0	0	9				WD				WD	WD
B*51:71	51:71	HLA03901		0	0	5	0	0	0	0	5			WD					WD	WD
B*51:72	51:72	HLA03971		0	0	1	0	0	0	0	1									
B*51:73	51:73	HLA03972		0	0	5	0	0	0	0	5			WD					WD	WD
B*51:74	51:74	HLA04045		0	0	2	7	0	0	0	9				WD				WD	WD
B*51:75	51:75	HLA04080		0	0	26	0	35	0	12	73			WD		I		WD	WD	I
B*51:76	51:76	HLA04102		0	0	6	0	0	0	0	6			WD					WD	WD
**B*51:78 total**	**51:78 total**			**0**	**1**	**1**	**0**	**0**	**0**	**0**	**2**									
B*51:78:01	51:78:01	HLA04015		0	1	1	0	0	0	0	2									

Supplemental Table 9: HLA-B Allele Summary [a]				Allele Count by Population Group [b]								3.0.0 CIWD Category by Population Group [c]								
Allele	Genomic typing	Allele ID	G group	AFA	API	EURO	MENA	HIS	NAM	UNK	Total	AFA	API	EURO	MENA	HIS	NAM	UNK	Total	Highest Frequency
B*51:79	51:79	HLA04199		0	0	18	0	0	0	2	20			WD					WD	WD
B*51:80	51:80	HLA04204		0	0	3	0	0	0	0	3									
B*51:81	51:81	HLA04225		0	0	0	0	1	0	1	2									
B*51:82	51:82	HLA04231		0	0	9	0	0	0	0	9			WD					WD	WD
B*51:83	51:83	HLA04251		1	1	6	21	0	0	0	29			WD	WD				WD	WD
B*51:84	51:84	HLA04166		0	1	0	0	0	0	0	1									
B*51:85	51:85	HLA04441		0	0	3	0	0	0	0	3									
B*51:86	51:86	HLA04444		0	3	0	0	0	0	0	3									
B*51:87	51:87	HLA04495		1	0	5	0	0	0	0	6			WD					WD	WD
B*51:89	51:89	HLA04649		0	0	2	0	0	0	0	2									
B*51:90	51:90	HLA04704		0	0	2	0	0	0	0	2									
B*51:91	51:91	HLA04705		0	0	0	0	1	0	0	1									
B*51:93	51:93	HLA04716		0	2	0	0	0	0	1	3									
B*51:94	51:94	HLA04718		0	0	1	0	0	0	2	3									
B*51:95	51:95	HLA04791		0	0	0	3	0	0	1	4									
B*51:97	51:97	HLA05464		0	0	1	0	0	0	0	1									
B*51:98N	51:98N	HLA05468		0	0	2	0	0	0	0	2									
B*51:100	51:100	HLA05495		0	0	5	0	0	0	0	5			WD					WD	WD
B*51:105	51:105	HLA05836		0	0	19	0	0	0	1	20			WD					WD	WD
**B*51:106 total**	**51:106 total**			**0**	**0**	**1**	**0**	**0**	**0**	**0**	**1**									
B*51:106	51:106			0	0	1	0	0	0	0	1									
B*51:107	51:107	HLA05959		0	5	6	0	0	0	0	11		WD	WD					WD	WD
B*51:109	51:109	HLA06270		0	12	0	0	0	0	0	12		WD						WD	WD
B*51:112	51:112	HLA06369		0	0	1	0	0	0	0	1									
B*51:114	51:114	HLA06675		0	0	5	0	2	0	2	9			WD					WD	WD
B*51:115	51:115	HLA06676		0	0	3	1	0	0	0	4									
B*51:116	51:116	HLA06698		0	0	5	0	0	0	0	5			WD					WD	WD
B*51:117	51:117	HLA06701		0	0	12	0	0	0	1	13			WD					WD	WD
B*51:119	51:119	HLA06959		0	0	1	0	0	0	0	1									
B*51:120	51:120	HLA06969		0	0	2	0	0	0	0	2									
B*51:122	51:122	HLA07002		0	0	6	0	0	0	0	6			WD					WD	WD

Supplemental Table 9: HLA-B Allele Summary [a]				Allele Count by Population Group [b]								3.0.0 CIWD Category by Population Group [c]								
Allele	Genomic typing	Allele ID	G group	AFA	API	EURO	MENA	HIS	NAM	UNK	Total	AFA	API	EURO	MENA	HIS	NAM	UNK	Total	Highest Frequency
B*51:123	51:123	HLA07180		0	0	0	0	7	1	1	9					WD			WD	WD
B*51:124	51:124	HLA07181		0	1	0	0	0	0	0	1									
B*51:127	51:127	HLA07461		3	0	0	0	2	0	2	7								WD	WD
B*51:131	51:131	HLA07715		0	0	1	0	0	0	0	1									
B*51:134	51:134	HLA08076		0	0	14	0	0	0	0	14			WD					WD	WD
B*51:135	51:135	HLA08077		0	1	0	0	0	0	0	1									
B*51:136	51:136	HLA08078		0	0	4	0	0	0	0	4									
B*51:137	51:137	HLA08130		0	1	0	0	0	0	0	1									
B*51:139	51:139	HLA08375		2	0	0	0	0	0	0	2									
B*51:141	51:141	HLA08391		0	0	3	0	0	0	0	3									
B*51:143	51:143	HLA08436		0	0	1	0	0	0	0	1									
B*51:145	51:145	HLA08653		0	0	2	0	0	0	0	2									
B*51:146	51:146	HLA08681		0	0	0	1	0	0	2	3									
B*51:147	51:147	HLA08682		0	0	3	0	0	0	0	3									
B*51:148	51:148	HLA08780		1	0	0	0	0	0	0	1									
B*51:150	51:150	HLA09001		0	0	2	0	0	0	0	2									
B*51:152	51:152	HLA09441		0	0	3	0	0	0	0	3									
B*51:153	51:153	HLA09489		0	2	0	0	0	0	0	2									
B*51:154	51:154	HLA09582		0	1	0	0	0	0	0	1									
**B*51:158 total**	**51:158 total**			**0**	**13**	**8**	**0**	**0**	**0**	**0**	**21**		**I**	**WD**					**WD**	**I**
B*51:158	51:158			0	1	0	0	0	0	0	1									
B*51:158:01	51:158:01	HLA10517		0	12	0	0	0	0	0	12		WD						WD	WD
B*51:158:02	51:158:02	HLA11833		0	0	8	0	0	0	0	8			WD					WD	WD
B*51:162	51:162	HLA10759		0	0	0	0	0	0	1	1									
B*51:167	51:167	HLA10991		0	0	1	0	0	0	0	1									
B*51:168	51:168	HLA11031		0	0	0	0	3	1	0	4									
B*51:171	51:171	HLA11433		0	0	0	0	0	0	1	1									
B*51:173Q	51:173Q	HLA11480		0	0	1	0	0	0	0	1									
**B*51:174 total**	**51:174 total**			**0**	**0**	**1**	**0**	**0**	**0**	**0**	**1**									
B*51:174	51:174			0	0	1	0	0	0	0	1									
B*51:177	51:177	HLA11835		0	0	5	0	0	0	0	5			WD					WD	WD

Supplemental Table 9: HLA-B Allele Summary [a]				Allele Count by Population Group [b]								3.0.0 CIWD Category by Population Group [c]								
Allele	Genomic typing	Allele ID	G group	AFA	API	EURO	MENA	HIS	NAM	UNK	Total	AFA	API	EURO	MENA	HIS	NAM	UNK	Total	Highest Frequency
B*51:178N	51:178N	HLA12015		0	0	1	0	0	0	0	1									
B*51:179	51:179	HLA11949		0	0	4	0	0	0	0	4									
B*51:181	51:181	HLA12301		0	0	2	0	0	0	0	2									
B*51:185	51:185	HLA12606		0	0	2	0	0	0	0	2									
B*51:187	51:187	HLA13027		0	0	2	0	0	0	0	2									
B*51:188	51:188	HLA13338		0	0	1	0	0	0	0	1									
B*51:191	51:191	HLA14017		0	3	0	0	0	0	0	3									
B*51:194	51:194	HLA14155		0	1	0	0	0	0	0	1									
B*51:203	51:203	HLA15034		0	0	1	0	0	0	0	1									
B*51:204	51:204	HLA14987		0	0	0	0	0	0	1	1									
B*51:206	51:206	HLA14989		0	0	0	0	0	0	1	1									
B*51:208	51:208	HLA15392		0	0	2	0	0	0	1	3									
B*51:210	51:210	HLA15739		0	0	1	0	0	0	0	1									
B*51:213	51:213	HLA15972		0	0	1	0	0	0	1	2									
B*51:221	51:221	HLA16877		0	1	0	0	0	0	0	1									
B*51:CODE	51:CODE			558	1553	29623	833	3209	218	4515	40509	NA	NA	NA	NA	NA	NA	NA	NA	NA
**B*52:01 total**	**52:01 total**			**6378**	**78865**	**124392**	**14417**	**15809**	**1476**	**26082**	**267419**	**C**	**C**	**C**	**C**	**C**	**C**	**C**	**C**	**C**
B*52:01	52:01			345	18	1069	22	947	59	285	2745	C	I	I	WD	C	C	C	C	C
B*52:01P	52:01P			0	3	297	0	1	0	2	303			I					I	I
**B*52:01:01G total**	**52:01:01G total**			**1015**	**78411**	**120920**	**14014**	**7093**	**747**	**23004**	**245204**	**C**	**C**	**C**	**C**	**C**	**C**	**C**	**C**	**C**
B*52:01:01G	52:01:01G		52:01:01G	825	73397	115473	13447	4987	534	21321	229984	C	C	C	C	C	C	C	C	C
B*52:01:01	52:01:01		52:01:01G	189	5012	5374	567	2103	211	1680	15136	C	C	C	C	C	C	C	C	C
B*52:01:01:01	52:01:01:01	HLA00362	52:01:01G	0	0	3	0	0	0	0	3									
B*52:01:01:02	52:01:01:02	HLA05917	52:01:01G	0	0	61	0	0	0	0	61			WD					WD	WD
B*52:01:01:03	52:01:01:03	HLA10677	52:01:01G	1	1	9	0	3	2	3	19			WD					WD	WD
B*52:01:08	52:01:08	HLA06159	52:01:01G	0	1	0	0	0	0	0	1									
**B*52:01:02G total**	**52:01:02G total**			**5018**	**343**	**2101**	**378**	**7765**	**669**	**2782**	**19056**	**C**	**C**	**C**	**C**	**C**	**C**	**C**	**C**	**C**
B*52:01:02G	52:01:02G		52:01:02G	195	193	236	25	230	2	226	1107	C	C	I	WD	C		C	I	C
B*52:01:02	52:01:02		52:01:02G	4823	150	1865	353	7535	667	2556	17949	C	C	C	C	C	C	C	C	C
B*52:01:04	52:01:04	HLA01677		0	0	0	0	2	1	0	3									
B*52:01:05	52:01:05	HLA04115		0	7	1	1	0	0	2	11		WD						WD	WD

Supplemental Table 9: HLA-B Allele Summary [a]				Allele Count by Population Group [b]								3.0.0 CIWD Category by Population Group [c]								
Allele	Genomic typing	Allele ID	G group	AFA	API	EURO	MENA	HIS	NAM	UNK	Total	AFA	API	EURO	MENA	HIS	NAM	UNK	Total	Highest Frequency
B*52:01:06	52:01:06	HLA05486		0	5	0	0	0	0	0	5		WD						WD	WD
B*52:01:07	52:01:07	HLA05927		0	57	1	1	0	0	1	60		I						WD	I
B*52:01:09	52:01:09	HLA06221		0	0	2	0	0	0	3	5								WD	WD
B*52:01:11	52:01:11	HLA07707		0	2	0	1	0	0	0	3									
B*52:01:12	52:01:12	HLA08065		0	2	0	0	0	0	0	2									
B*52:01:13	52:01:13	HLA08364		0	0	1	0	1	0	0	2									
B*52:01:14	52:01:14	HLA08366		0	11	0	0	0	0	0	11		WD						WD	WD
B*52:01:16	52:01:16	HLA08675		0	1	0	0	0	0	0	1									
B*52:01:17	52:01:17	HLA08989		0	0	0	0	0	0	1	1									
B*52:01:18	52:01:18	HLA09902		0	3	0	0	0	0	0	3									
B*52:01:19	52:01:19	HLA10031		0	0	0	0	0	0	1	1									
B*52:01:20	52:01:20	HLA10761		0	0	0	0	0	0	1	1									
B*52:01:21	52:01:21	HLA12283		0	1	0	0	0	0	0	1									
B*52:01:23	52:01:23	HLA12999		0	1	0	0	0	0	0	1									
**B*52:02 total**	**52:02 total**			**0**	**0**	**5**	**0**	**27**	**2**	**0**	**34**			**WD**		**I**			**WD**	**I**
B*52:02	52:02			0	0	2	0	5	1	0	8					WD			WD	WD
B*52:02:01	52:02:01	HLA01141		0	0	3	0	22	1	0	26					I			WD	I
B*52:03	52:03	HLA01295		0	0	2	0	13	0	0	15					I			WD	I
B*52:04	52:04	HLA01580		5	1506	9	4	0	10	23	1557	WD	C	WD			C	I	I	C
B*52:05	52:05	HLA01627		0	0	1	0	0	0	1	2									
**B*52:06 total**	**52:06 total**			**0**	**2**	**29**	**0**	**0**	**0**	**0**	**31**			**WD**					**WD**	**WD**
B*52:06	52:06			0	0	2	0	0	0	0	2									
B*52:06:02	52:06:02	HLA03009		0	0	26	0	0	0	0	26			WD					WD	WD
B*52:06:03	52:06:03	HLA13000		0	2	1	0	0	0	0	3									
B*52:08	52:08	HLA02212		0	0	3	0	0	0	0	3									
**B*52:10 total**	**52:10 total**			**0**	**2**	**5**	**0**	**7**	**2**	**4**	**20**			**WD**		**WD**			**WD**	**WD**
B*52:10:01	52:10:01	HLA02585		0	2	4	0	7	2	4	19					WD			WD	WD
B*52:10:02	52:10:02	HLA06978		0	0	1	0	0	0	0	1									
B*52:11	52:11	HLA02793		0	3	6	3	0	0	0	12			WD					WD	WD
B*52:17	52:17	HLA04450		0	4	0	0	0	0	1	5								WD	WD
B*52:19	52:19	HLA04498		0	3	0	0	0	0	0	3									

Supplemental Table 9: HLA-B Allele Summary [a]				Allele Count by Population Group [b]								3.0.0 CIWD Category by Population Group [c]								
Allele	Genomic typing	Allele ID	G group	AFA	API	EURO	MENA	HIS	NAM	UNK	Total	AFA	API	EURO	MENA	HIS	NAM	UNK	Total	Highest Frequency
B*52:20	52:20	HLA04733		0	2	0	0	0	0	1	3									
**B*52:21 total**	**52:21 total**			**0**	**0**	**5**	**0**	**6**	**0**	**4**	**15**			**WD**		**WD**			**WD**	**WD**
B*52:21	52:21			0	0	5	0	6	0	4	15			WD		WD			WD	WD
**B*52:25 total**	**52:25 total**			**0**	**0**	**24**	**0**	**0**	**0**	**0**	**24**			**WD**					**WD**	**WD**
B*52:25	52:25			0	0	17	0	0	0	0	17			WD					WD	WD
B*52:25:01	52:25:01	HLA06963		0	0	3	0	0	0	0	3									
B*52:25:02	52:25:02	HLA15274		0	0	4	0	0	0	0	4									
B*52:27	52:27	HLA07336		0	4	2	0	0	0	0	6								WD	WD
B*52:28	52:28	HLA07823		0	4	0	0	0	0	0	4									
B*52:30	52:30	HLA08064		0	1	0	0	0	0	0	1									
**B*52:31 total**	**52:31 total**			**0**	**0**	**1**	**0**	**0**	**0**	**0**	**1**									
B*52:31:01	52:31:01	HLA08363		0	0	1	0	0	0	0	1									
B*52:37	52:37	HLA11022		0	4	1	0	0	0	0	5								WD	WD
B*52:39	52:39	HLA11427		0	1	0	0	0	0	0	1									
B*52:40	52:40	HLA11732		0	0	1	0	0	0	0	1									
B*52:48	52:48	HLA13478		0	0	0	0	1	0	0	1									
B*52:50	52:50	HLA13613		0	0	1	0	0	0	0	1									
B*52:51	52:51	HLA13851		0	0	1	0	0	0	0	1									
B*52:54	52:54	HLA14092		0	1	0	0	0	0	0	1									
B*52:58	52:58	HLA14439		0	1	0	0	0	0	0	1									
B*52:59	52:59	HLA14441		0	1	0	0	0	0	0	1									
B*52:60	52:60	HLA14728		0	2	0	0	0	0	0	2									
B*52:61	52:61	HLA14732		0	0	1	0	0	0	0	1									
B*52:62	52:62	HLA15965		0	1	0	0	0	0	0	1									
B*52:63	52:63	HLA15966		0	1	0	0	0	0	0	1									
B*52:CODE	52:CODE			217	1325	9702	710	984	80	1553	14571	NA	NA	NA	NA	NA	NA	NA	NA	NA
**B*53:01 total**	**53:01 total**			**42405**	**1386**	**34181**	**5821**	**9969**	**1408**	**16966**	**112136**	**C**	**C**	**C**	**C**	**C**	**C**	**C**	**C**	**C**
B*53:01	53:01			2428	23	1826	153	1095	98	643	6266	C	I	C	C	C	C	C	C	C
B*53:01P	53:01P			3	0	141	3	3	0	1	151			I					WD	I
**B*53:01:01G total**	**53:01:01G total**			**39947**	**1353**	**32211**	**5665**	**8869**	**1310**	**16315**	**105670**	**C**	**C**	**C**	**C**	**C**	**C**	**C**	**C**	**C**
B*53:01:01G	53:01:01G		53:01:01G	14896	967	20254	4354	3385	320	7938	52114	C	C	C	C	C	C	C	C	C

Supplemental Table 9: HLA-B Allele Summary [a]				Allele Count by Population Group [b]								3.0.0 CIWD Category by Population Group [c]								
Allele	Genomic typing	Allele ID	G group	AFA	API	EURO	MENA	HIS	NAM	UNK	Total	AFA	API	EURO	MENA	HIS	NAM	UNK	Total	Highest Frequency
B*53:01:01	53:01:01	HLA00364	53:01:01G	25016	386	11956	1311	5483	990	8373	53515	C	C	C	C	C	C	C	C	C
B*53:37	53:37	HLA11955	53:01:01G	35	0	1	0	1	0	4	41	WD							WD	WD
B*53:01:02	53:01:02	HLA01922		1	0	0	0	0	0	0	1									
B*53:01:03	53:01:03	HLA02114		0	10	2	0	0	0	0	12		WD						WD	WD
B*53:01:05	53:01:05	HLA04244		17	0	0	0	1	0	4	22	WD							WD	WD
B*53:01:06	53:01:06	HLA07643		5	0	1	0	0	0	0	6	WD							WD	WD
B*53:01:07	53:01:07	HLA07702		2	0	0	0	0	0	2	4									
B*53:01:10	53:01:10	HLA11039		0	0	0	0	0	0	1	1									
B*53:01:11	53:01:11	HLA13537		2	0	0	0	1	0	0	3									
B*53:02	53:02	HLA00365		0	0	1	0	0	0	0	1									
B*53:04	53:04	HLA00987		0	4	14	4	2	0	4	28			WD					WD	WD
**B*53:05 total**	**53:05 total**			**0**	**2**	**235**	**10**	**2**	**0**	**13**	**262**			**I**	**WD**			**WD**	**I**	**I**
B*53:05	53:05			0	1	202	9	2	0	12	226			I	WD			WD	I	I
B*53:05:01	53:05:01	HLA01178		0	1	33	1	0	0	1	36			WD					WD	WD
B*53:06	53:06	HLA01261		0	0	75	1	0	0	0	76			WD					WD	WD
**B*53:08 total**	**53:08 total**			**2**	**0**	**0**	**0**	**0**	**0**	**0**	**2**									
B*53:08	53:08			2	0	0	0	0	0	0	2									
B*53:09	53:09	HLA01522		0	55	0	0	0	0	0	55		I						WD	I
B*53:10	53:10	HLA02078		0	0	11	0	5	0	5	21			WD		WD		WD	WD	WD
B*53:12	53:12	HLA02636		0	0	1	0	0	0	0	1									
B*53:13	53:13	HLA02942		0	0	3	0	0	0	1	4									
B*53:15	53:15	HLA03209		6	0	0	0	0	0	0	6	WD							WD	WD
B*53:16	53:16	HLA03274		0	0	2	0	0	0	1	3									
**B*53:17 total**	**53:17 total**			**0**	**0**	**2**	**0**	**0**	**0**	**1**	**3**									
B*53:17:02	53:17:02	HLA06050		0	0	2	0	0	0	1	3									
B*53:18	53:18	HLA03609		4	0	0	0	0	0	1	5								WD	WD
B*53:19	53:19	HLA04095		0	0	2	0	0	0	0	2									
B*53:20	53:20	HLA04462		6	0	0	0	0	0	1	7	WD							WD	WD
B*53:21	53:21	HLA04412		0	0	1	0	0	0	0	1									
B*53:22	53:22	HLA04713		0	0	0	0	0	0	1	1									
B*53:23	53:23	HLA04817		0	0	3	0	1	0	1	5								WD	WD

Supplemental Table 9: HLA-B Allele Summary [a]				Allele Count by Population Group [b]								3.0.0 CIWD Category by Population Group [c]								
Allele	Genomic typing	Allele ID	G group	AFA	API	EURO	MENA	HIS	NAM	UNK	Total	AFA	API	EURO	MENA	HIS	NAM	UNK	Total	Highest Frequency
B*53:24	53:24	HLA05862		0	0	6	2	0	0	1	9			WD					WD	WD
B*53:25	53:25	HLA06687		1	0	0	0	0	0	0	1									
B*53:26	53:26	HLA06934		2	0	0	0	0	0	0	2									
B*53:27	53:27	HLA07192		1	0	0	0	0	0	0	1									
B*53:28	53:28	HLA08066		0	10	0	0	0	0	0	10		WD						WD	WD
B*53:29	53:29	HLA08355		0	0	0	0	1	0	1	2									
B*53:30	53:30	HLA09439		1	0	0	0	0	0	0	1									
B*53:31	53:31	HLA09681		0	0	5	0	0	0	0	5			WD					WD	WD
B*53:33	53:33	HLA11021		0	2	1	0	0	0	1	4									
B*53:36	53:36	HLA11827		0	0	1	0	0	0	0	1									
B*53:38	53:38	HLA12282		0	0	1	0	0	0	0	1									
B*53:CODE	53:CODE			2469	50	3267	301	867	93	1635	8682	NA	NA	NA	NA	NA	NA	NA	NA	NA
**B*54:01 total**	**54:01 total**			**9**	**9154**	**840**	**400**	**36**	**7**	**1576**	**12022**	**WD**	**C**	**I**	**C**	**I**	**C**	**C**	**C**	**C**
B*54:01	54:01			0	3	3	0	0	0	0	6								WD	WD
B*54:01P	54:01P			0	1	0	0	0	0	0	1									
**B*54:01:01G total**	**54:01:01G total**			**9**	**9149**	**837**	**400**	**36**	**7**	**1576**	**12014**	**WD**	**C**	**I**	**C**	**I**	**C**	**C**	**C**	**C**
B*54:01:01G	54:01:01G		54:01:01G	9	6734	805	396	31	5	1060	9040	WD	C	I	C	I	WD	C	C	C
B*54:01:01	54:01:01	HLA00367	54:01:01G	0	2415	32	4	5	2	516	2974		C	WD		WD		C	C	C
B*54:01:07	54:01:07	HLA13484		0	1	0	0	0	0	0	1									
B*54:03	54:03	HLA02027		0	0	0	1	0	0	0	1									
B*54:10	54:10	HLA02716		0	1	0	0	0	0	0	1									
B*54:20	54:20	HLA04719		0	1	0	0	0	0	0	1									
B*54:23	54:23	HLA05401		0	1	0	0	0	0	0	1									
B*54:33	54:33	HLA12012		0	1	0	0	0	0	0	1									
B*54:CODE	54:CODE			1	672	57	4	1	1	82	818	NA	NA	NA	NA	NA	NA	NA	NA	NA
**B*55:01 total**	**55:01 total**			**1374**	**20917**	**174535**	**10202**	**5843**	**582**	**18338**	**231791**	**C**	**C**	**C**	**C**	**C**	**C**	**C**	**C**	**C**
B*55:01	55:01			704	1534	47367	2662	2792	368	9561	64988	C	C	C	C	C	C	C	C	C
B*55:01P	55:01P			0	0	77	2	0	0	0	79			WD					WD	WD
**B*55:01:01G total**	**55:01:01G total**			**670**	**19242**	**127000**	**7531**	**3048**	**214**	**8767**	**166472**	**C**	**C**	**C**	**C**	**C**	**C**	**C**	**C**	**C**
B*55:01:01G	55:01:01G		55:01:01G	376	18263	116297	7241	1358	82	7077	150694	C	C	C	C	C	C	C	C	C
B*55:01:01	55:01:01	HLA00368	55:01:01G	281	978	10526	289	1681	131	1655	15541	C	C	C	C	C	C	C	C	C

Supplemental Table 9: HLA-B Allele Summary [a]				Allele Count by Population Group [b]								3.0.0 CIWD Category by Population Group [c]								
Allele	Genomic typing	Allele ID	G group	AFA	API	EURO	MENA	HIS	NAM	UNK	Total	AFA	API	EURO	MENA	HIS	NAM	UNK	Total	Highest Frequency
B*55:01:03	55:01:03	HLA02324	55:01:01G	13	1	176	1	9	1	35	236	WD		I		I		I	I	I
B*55:85	55:85	HLA16755	55:01:01G	0	0	1	0	0	0	0	1									
B*55:01:02	55:01:02	HLA02137		0	0	23	0	0	0	4	27			WD					WD	WD
B*55:01:04	55:01:04	HLA02335		0	137	14	7	2	0	3	163		C	WD	WD				I	C
B*55:01:05	55:01:05	HLA04185		0	0	14	0	0	0	0	14			WD					WD	WD
B*55:01:06	55:01:06	HLA04214		0	1	37	0	1	0	3	42			WD					WD	WD
B*55:01:09	55:01:09	HLA07657		0	2	0	0	0	0	0	2									
B*55:01:10	55:01:10	HLA08679		0	0	1	0	0	0	0	1									
B*55:01:15	55:01:15	HLA13798		0	0	2	0	0	0	0	2									
B*55:01:16	55:01:16	HLA14733		0	1	0	0	0	0	0	1									
**B*55:02 total**	**55:02 total**			**21**	**7346**	**545**	**104**	**32**	**7**	**1349**	**9404**	**WD**	**C**	**I**	**C**	**I**	**C**	**C**	**C**	**C**
B*55:02	55:02			6	1633	96	22	10	4	391	2162	WD	C	WD	WD	I		C	C	C
B*55:02P	55:02P			0	0	5	1	0	0	0	6			WD					WD	WD
**B*55:02:01G total**	**55:02:01G total**			**15**	**5692**	**444**	**81**	**22**	**3**	**956**	**7213**	**WD**	**C**	**I**	**C**	**I**		**C**	**C**	**C**
B*55:02:01G	55:02:01G		55:02:01G	6	4236	407	76	15	2	453	5195	WD	C	I	C	I		C	C	C
B*55:02:01	55:02:01		55:02:01G	9	1423	35	5	7	1	498	1978	WD	C	WD	WD	WD		C	C	C
B*55:02:01:01	55:02:01:01	HLA00369	55:02:01G	0	1	2	0	0	0	0	3									
B*55:02:01:02	55:02:01:02	HLA12581	55:02:01G	0	32	0	0	0	0	5	37		I					WD	WD	I
B*55:02:03	55:02:03	HLA03513		0	20	0	0	0	0	2	22		I						WD	I
B*55:02:07	55:02:07	HLA10412		0	1	0	0	0	0	0	1									
B*55:03	55:03	HLA00370		0	35	0	0	1	0	1	37		I						WD	I
B*55:04	55:04	HLA00371		0	211	5	2	1	0	44	263		C	WD				I	I	C
B*55:05	55:05	HLA00372		1	0	16	0	2	0	1	20			WD					WD	WD
B*55:07	55:07	HLA00374		0	45	16	5	1	0	0	67		I	WD	WD				WD	I
B*55:08	55:08	HLA00375		0	0	1	0	0	0	0	1									
B*55:09	55:09	HLA01180		0	0	60	0	0	0	0	60			WD					WD	WD
B*55:10	55:10	HLA01205		0	1	0	0	0	0	1	2									
B*55:11	55:11	HLA01507		0	0	26	0	5	0	1	32			WD		WD			WD	WD
B*55:12	55:12	HLA01553		0	81	3	0	0	0	10	94		I					WD	WD	I
B*55:14	55:14	HLA01732		0	0	7	0	0	0	0	7			WD					WD	WD
B*55:17	55:17	HLA02092		0	0	34	0	0	0	2	36			WD					WD	WD

Supplemental Table 9: HLA-B Allele Summary [a]				Allele Count by Population Group [b]								3.0.0 CIWD Category by Population Group [c]								
**Allele**	**Genomic typing**	**Allele ID**	**G group**	**AFA**	**API**	**EURO**	**MENA**	**HIS**	**NAM**	**UNK**	**Total**	**AFA**	**API**	**EURO**	**MENA**	**HIS**	**NAM**	**UNK**	**Total**	**Highest Frequency**
B*55:18	55:18	HLA02093		0	3	0	0	0	0	4	7								WD	WD
B*55:20	55:20	HLA02320		0	0	7	0	0	0	0	7			WD					WD	WD
B*55:21	55:21	HLA02518		0	1	2	8	0	0	1	12				WD				WD	WD
B*55:22	55:22	HLA02539		0	1	1	0	0	0	2	4									
B*55:23	55:23	HLA02671		0	3	1	0	0	0	1	5								WD	WD
B*55:24	55:24	HLA02677		0	0	2	0	0	0	0	2									
B*55:25	55:25	HLA02782		0	0	47	0	0	0	1	48			WD					WD	WD
B*55:26	55:26	HLA02845		0	3	0	0	0	0	0	3									
B*55:28	55:28	HLA03098		0	0	1	0	0	0	0	1									
B*55:29	55:29	HLA03195		0	0	5	0	0	0	0	5			WD					WD	WD
B*55:30	55:30	HLA03228		0	2	0	0	0	0	0	2									
B*55:31	55:31	HLA03322		0	0	2	3	0	0	0	5								WD	WD
B*55:33	55:33	HLA03424		0	0	19	0	1	0	2	22			WD					WD	WD
B*55:34	55:34	HLA03503		0	1	0	0	0	0	0	1									
B*55:36	55:36	HLA04047		0	0	1	0	0	0	0	1									
B*55:37	55:37	HLA03983		0	0	0	1	0	0	0	1									
B*55:40	55:40	HLA04821		0	0	3	0	0	0	0	3									
B*55:44	55:44	HLA05478		0	0	0	0	1	0	0	1									
B*55:45	55:45	HLA05507		0	0	13	0	0	0	0	13			WD					WD	WD
B*55:49	55:49	HLA06053		0	1	0	0	0	0	1	2									
B*55:50	55:50	HLA06519		0	1	0	0	0	0	0	1									
B*55:51	55:51	HLA06584		0	3	0	0	0	0	0	3									
B*55:52	55:52	HLA06920		0	1	0	0	0	0	0	1									
B*55:54	55:54	HLA07314		0	0	2	0	0	0	0	2									
B*55:55N	55:55N	HLA07459		0	0	1	0	0	0	0	1									
B*55:56	55:56	HLA07710		0	0	3	0	0	0	0	3									
B*55:59	55:59	HLA09580		0	2	0	0	0	0	0	2									
B*55:60	55:60	HLA10041		0	4	0	0	0	0	0	4									
B*55:64	55:64	HLA11431		0	0	0	0	2	0	0	2									
B*55:67	55:67	HLA11831		0	1	0	0	0	0	0	1									
B*55:69	55:69	HLA12297		0	1	0	0	0	0	0	1									

Supplemental Table 9: HLA-B Allele Summary [a]				Allele Count by Population Group [b]								3.0.0 CIWD Category by Population Group [c]								
**Allele**	**Genomic typing**	**Allele ID**	**G group**	**AFA**	**API**	**EURO**	**MENA**	**HIS**	**NAM**	**UNK**	**Total**	**AFA**	**API**	**EURO**	**MENA**	**HIS**	**NAM**	**UNK**	**Total**	**Highest Frequency**
B*55:72	55:72	HLA13179		0	1	0	0	0	0	0	1									
B*55:73	55:73	HLA13363		0	0	1	0	0	0	0	1									
B*55:78	55:78	HLA14444		0	8	0	0	0	0	0	8		WD						WD	WD
B*55:CODE	55:CODE			92	502	11886	244	531	53	1504	14812	NA	NA	NA	NA	NA	NA	NA	NA	NA
**B*56:01 total**	**56:01 total**			**882**	**9321**	**105001**	**1014**	**2845**	**335**	**7007**	**126405**	**C**	**C**	**C**	**C**	**C**	**C**	**C**	**C**	**C**
B*56:01	56:01			0	4	449	0	1	0	20	474			I				I	I	I
B*56:01P	56:01P			0	1	74	0	0	0	0	75			WD					WD	WD
**B*56:01:01G total**	**56:01:01G total**			**882**	**9314**	**104428**	**1014**	**2844**	**335**	**6987**	**125804**	**C**	**C**	**C**	**C**	**C**	**C**	**C**	**C**	**C**
B*56:01:01G	56:01:01G		56:01:01G	677	8618	100793	994	2021	239	6283	119625	C	C	C	C	C	C	C	C	C
B*56:01:01	56:01:01		56:01:01G	135	416	2156	9	501	59	453	3729	C	C	C	WD	C	C	C	C	C
B*56:01:01:01	56:01:01:01	HLA00376	56:01:01G	0	34	5	0	0	0	4	43		I	WD					WD	I
B*56:01:01:02	56:01:01:02	HLA12582	56:01:01G	49	184	940	7	239	28	168	1615	C	C	I	WD	C	C	C	C	C
B*56:01:01:03	56:01:01:03	HLA14086	56:01:01G	21	62	511	4	83	9	78	768	WD	I	I		C	C	I	I	C
B*56:01:01:04	56:01:01:04	HLA16261	56:01:01G	0	0	23	0	0	0	1	24			WD					WD	WD
B*56:01:02	56:01:02	HLA04209		0	0	21	0	0	0	0	21			WD					WD	WD
B*56:01:03	56:01:03	HLA04552		0	0	13	0	0	0	0	13			WD					WD	WD
B*56:01:04	56:01:04	HLA04819		0	0	13	0	0	0	0	13			WD					WD	WD
B*56:01:06	56:01:06	HLA09722		0	0	3	0	0	0	0	3									
B*56:01:07	56:01:07	HLA10777		0	1	0	0	0	0	0	1									
B*56:01:08	56:01:08	HLA12296		0	1	0	0	0	0	0	1									
**B*56:02 total**	**56:02 total**			**2**	**372**	**13**	**0**	**3**	**0**	**277**	**667**		**C**	**WD**				**C**	**I**	**C**
B*56:02	56:02			2	308	8	0	2	0	220	540		C	WD				C	I	C
B*56:02:01	56:02:01	HLA00377		0	64	5	0	1	0	55	125		I	WD				I	WD	I
B*56:02:02	56:02:02	HLA14399		0	0	0	0	0	0	2	2									
B*56:03	56:03	HLA00378		0	337	8	0	0	0	55	400		C	WD				I	I	C
B*56:04	56:04	HLA00379		1	1147	45	6	7	1	154	1361		C	WD	WD	WD		C	I	C
**B*56:05 total**	**56:05 total**			**0**	**16**	**0**	**0**	**0**	**0**	**1**	**17**		**I**						**WD**	**I**
B*56:05	56:05			0	1	0	0	0	0	0	1									
B*56:05:01	56:05:01	HLA00380		0	8	0	0	0	0	0	8		WD						WD	WD
B*56:05:02	56:05:02	HLA01719		0	7	0	0	0	0	1	8		WD						WD	WD
B*56:06	56:06	HLA00988		0	0	1	0	1	0	0	2									

Supplemental Table 9: HLA-B Allele Summary [a]				Allele Count by Population Group [b]								3.0.0 CIWD Category by Population Group [c]								
Allele	Genomic typing	Allele ID	G group	AFA	API	EURO	MENA	HIS	NAM	UNK	Total	AFA	API	EURO	MENA	HIS	NAM	UNK	Total	Highest Frequency
B*56:07	56:07	HLA00989		0	6	1	0	0	0	1	8		WD						WD	WD
B*56:09	56:09	HLA01625		0	1	0	0	0	0	0	1									
B*56:10	56:10	HLA01658		0	32	0	0	0	0	2	34		I						WD	I
B*56:11	56:11	HLA01688		0	2	0	2	0	0	0	4									
B*56:13	56:13	HLA01892		0	0	5	0	2	0	3	10			WD					WD	WD
B*56:14	56:14	HLA02002		0	1	0	0	0	0	0	1									
B*56:15	56:15	HLA02043		1	0	24	0	7	2	0	34			WD		WD			WD	WD
B*56:16	56:16	HLA02261		1	0	43	0	0	1	4	49			WD					WD	WD
B*56:17	56:17	HLA02565		0	0	0	0	1	0	1	2									
**B*56:20 total**	**56:20 total**			**0**	**0**	**3**	**0**	**0**	**0**	**1**	**4**									
B*56:20	56:20			0	0	2	0	0	0	1	3									
B*56:20:01	56:20:01	HLA02852		0	0	1	0	0	0	0	1									
B*56:21	56:21	HLA03085		0	0	1	0	0	0	0	1									
B*56:22	56:22	HLA03266		0	13	0	0	0	0	0	13		I						WD	I
B*56:25	56:25	HLA04077		0	1	35	0	0	0	3	39			WD					WD	WD
B*56:26	56:26	HLA04024		0	0	1	0	1	0	0	2									
B*56:27	56:27	HLA04555		0	1	58	0	2	0	3	64			WD					WD	WD
B*56:29	56:29	HLA04806		0	0	1	0	0	0	0	1									
B*56:31	56:31	HLA06060		0	0	3	0	0	0	0	3									
B*56:33	56:33	HLA07460		0	0	9	0	0	0	0	9			WD					WD	WD
B*56:34	56:34	HLA07711		0	1	0	1	0	0	0	2									
B*56:36	56:36	HLA08059		0	0	1	0	0	0	0	1									
B*56:37	56:37	HLA08516		0	0	4	0	0	0	0	4									
B*56:39	56:39	HLA08999		0	0	2	0	0	0	0	2									
B*56:41	56:41	HLA09579		0	0	4	0	0	0	0	4									
B*56:44	56:44	HLA11832		0	0	1	0	0	0	0	1									
B*56:45	56:45	HLA12756		0	0	1	0	0	0	0	1									
B*56:46	56:46	HLA13006		0	1	0	0	0	0	0	1									
B*56:CODE	56:CODE			35	122	4451	20	201	15	403	5247	NA	NA	NA	NA	NA	NA	NA	NA	NA
**B*57:01 total**	**57:01 total**			**2476**	**63284**	**389284**	**5266**	**9547**	**1016**	**34365**	**505238**	**C**	**C**	**C**	**C**	**C**	**C**	**C**	**C**	**C**
B*57:01	57:01			3	6	1597	2	4	0	80	1692		WD	C				I	C	C

Supplemental Table 9: HLA-B Allele Summary [a]				Allele Count by Population Group [b]								3.0.0 CIWD Category by Population Group [c]								
Allele	Genomic typing	Allele ID	G group	AFA	API	EURO	MENA	HIS	NAM	UNK	Total	AFA	API	EURO	MENA	HIS	NAM	UNK	Total	Highest Frequency
B*57:01P	57:01P			0	3	327	0	0	0	2	332			I					I	I
**B*57:01:01G total**	**57:01:01G total**			**2473**	**63272**	**387265**	**5132**	**9541**	**1016**	**34280**	**502979**	**C**	**C**	**C**	**C**	**C**	**C**	**C**	**C**	**C**
B*57:01:01G	57:01:01G		57:01:01G	1956	57485	360426	4838	6802	770	30655	462932	C	C	C	C	C	C	C	C	C
B*57:01:01	57:01:01		57:01:01G	517	5787	26833	294	2739	246	3624	40040	C	C	C	C	C	C	C	C	C
B*57:01:01:01	57:01:01:01	HLA00381	57:01:01G	0	0	4	0	0	0	1	5								WD	WD
B*57:79N	57:79N	HLA13633	57:01:01G	0	0	2	0	0	0	0	2									
B*57:01:02	57:01:02	HLA01520		0	0	65	3	0	0	1	69			WD					WD	WD
B*57:01:04	57:01:04	HLA03969		0	0	3	0	0	0	1	4									
B*57:01:06	57:01:06	HLA04456		0	0	5	0	0	0	0	5			WD					WD	WD
B*57:01:07	57:01:07	HLA04755		0	2	2	129	0	0	1	134				C				WD	C
B*57:01:08	57:01:08	HLA05320		0	0	4	0	0	0	0	4									
B*57:01:10	57:01:10	HLA05563		0	0	12	0	0	0	0	12			WD					WD	WD
B*57:01:14	57:01:14	HLA08370		0	0	4	0	0	0	0	4									
B*57:01:15	57:01:15	HLA09723		0	0	0	0	1	0	0	1									
B*57:01:18	57:01:18	HLA11430		0	1	1	0	1	0	0	3									
B*57:01:20	57:01:20	HLA12568		0	0	1	0	0	0	0	1									
**B*57:02 total**	**57:02 total**			**2064**	**45**	**3167**	**497**	**515**	**79**	**811**	**7178**	**C**	**I**	**C**	**C**	**C**	**C**	**C**	**C**	**C**
B*57:02	57:02			87	3	236	38	51	6	37	458	C		I	WD	I	WD	I	I	C
B*57:02P	57:02P			0	0	2	0	0	0	0	2									
B*57:02:01	57:02:01	HLA00382		1969	42	2929	459	464	73	770	6706	C	I	C	C	C	C	C	C	C
B*57:02:02	57:02:02	HLA04435		8	0	0	0	0	0	4	12	WD							WD	WD
**B*57:03 total**	**57:03 total**			**11979**	**217**	**8352**	**933**	**4334**	**909**	**4344**	**31068**	**C**	**C**	**C**	**C**	**C**	**C**	**C**	**C**	**C**
B*57:03	57:03			784	2	530	47	488	76	226	2153	C		I	C	C	C	C	C	C
B*57:03P	57:03P			1	0	4	0	0	0	0	5								WD	WD
**B*57:03:01G total**	**57:03:01G total**			**11194**	**215**	**7816**	**884**	**3846**	**833**	**4118**	**28906**	**C**	**C**	**C**	**C**	**C**	**C**	**C**	**C**	**C**
B*57:03:01G	57:03:01G		57:03:01G	712	80	1811	57	250	3	509	3422	C	I	C	C	C		C	C	C
B*57:03:01	57:03:01		57:03:01G	9374	118	5697	802	2925	670	3273	22859	C	I	C	C	C	C	C	C	C
B*57:03:01:01	57:03:01:01	HLA00383	57:03:01G	565	0	9	2	73	16	109	774	C		WD		C	C	I	I	C
B*57:03:01:02	57:03:01:02	HLA14808	57:03:01G	543	17	299	23	598	144	227	1851	C	I	I	WD	C	C	C	C	C
B*57:03:02	57:03:02	HLA01289		0	0	1	2	0	0	0	3									
B*57:03:03	57:03:03	HLA16226		0	0	1	0	0	0	0	1									

Supplemental Table 9: HLA-B Allele Summary [a]				Allele Count by Population Group [b]								3.0.0 CIWD Category by Population Group [c]								
Allele	Genomic typing	Allele ID	G group	AFA	API	EURO	MENA	HIS	NAM	UNK	Total	AFA	API	EURO	MENA	HIS	NAM	UNK	Total	Highest Frequency
**B*57:04 total**	**57:04 total**			**725**	**3**	**31**	**2**	**35**	**6**	**158**	**960**	**C**		**WD**		**I**	**WD**	**C**	**I**	**C**
B*57:04	57:04			597	3	17	2	26	5	121	771	C		WD		I	WD	I	I	C
B*57:04P	57:04P			0	0	0	0	1	0	0	1									
B*57:04:01	57:04:01	HLA00384		128	0	14	0	8	1	37	188	C		WD		I		I	I	C
B*57:05	57:05	HLA00385		13	0	0	0	0	0	3	16	WD							WD	WD
B*57:07	57:07	HLA01192		1	0	0	0	0	0	0	1									
B*57:09	57:09	HLA01485		10	0	0	0	0	0	1	11	WD							WD	WD
B*57:11	57:11	HLA02676		1	4	4	0	0	0	1	10								WD	WD
B*57:12	57:12	HLA02888		4	0	0	0	0	0	0	4									
B*57:13	57:13	HLA02966		0	0	1	0	0	0	0	1									
**B*57:14 total**	**57:14 total**			**0**	**0**	**19**	**0**	**0**	**0**	**0**	**19**			**WD**					**WD**	**WD**
B*57:14	57:14			0	0	6	0	0	0	0	6			WD					WD	WD
B*57:14:01	57:14:01	HLA03129		0	0	13	0	0	0	0	13			WD					WD	WD
B*57:15	57:15	HLA03147		0	1	16	0	0	0	1	18			WD					WD	WD
B*57:16	57:16	HLA03150		0	0	3	0	0	0	0	3									
B*57:17	57:17	HLA03320		1	0	0	0	0	0	0	1									
B*57:18	57:18	HLA03506		0	1	0	0	0	0	0	1									
B*57:19	57:19	HLA03507		0	0	1	0	0	0	1	2									
B*57:20	57:20	HLA03666		0	0	1	0	0	0	0	1									
B*57:21	57:21	HLA03675		0	0	12	0	0	0	0	12			WD					WD	WD
B*57:22	57:22	HLA03904		0	5	0	0	0	0	0	5		WD						WD	WD
B*57:23	57:23	HLA04046		0	0	9	0	0	0	0	9			WD					WD	WD
B*57:24	57:24	HLA03984		0	0	18	0	0	0	0	18			WD					WD	WD
B*57:26	57:26	HLA04203		0	1	9	0	0	0	0	10			WD					WD	WD
B*57:27	57:27	HLA04452		0	0	0	0	1	0	0	1									
B*57:31	57:31	HLA04848		0	0	2	0	0	0	0	2									
B*57:32	57:32	HLA05424		0	0	6	0	0	0	0	6			WD					WD	WD
B*57:34	57:34	HLA05503		0	0	8	0	0	0	0	8			WD					WD	WD
B*57:35	57:35	HLA05513		0	0	1	0	0	0	0	1									
B*57:38	57:38	HLA05958		0	0	1	0	0	0	0	1									
B*57:39	57:39	HLA06229		0	0	0	2	0	0	0	2									

Supplemental Table 9: HLA-B Allele Summary [a]				Allele Count by Population Group [b]								3.0.0 CIWD Category by Population Group [c]								
**Allele**	**Genomic typing**	**Allele ID**	**G group**	**AFA**	**API**	**EURO**	**MENA**	**HIS**	**NAM**	**UNK**	**Total**	**AFA**	**API**	**EURO**	**MENA**	**HIS**	**NAM**	**UNK**	**Total**	**Highest Frequency**
B*57:40	57:40	HLA06240		0	0	17	0	0	0	0	17			WD					WD	WD
B*57:41	57:41	HLA06241		0	0	1	0	0	0	1	2									
B*57:42	57:42	HLA06249		3	0	0	0	0	0	0	3									
B*57:43	57:43	HLA06250		0	2	0	0	0	0	1	3									
B*57:44	57:44	HLA06315		0	0	5	0	0	0	0	5			WD					WD	WD
B*57:45	57:45	HLA06683		0	0	1	0	0	0	0	1									
B*57:47	57:47	HLA06700		0	0	0	0	0	0	1	1									
B*57:48	57:48	HLA06883		0	0	5	0	0	0	0	5			WD					WD	WD
B*57:49	57:49	HLA06942		0	1	1	0	0	0	0	2									
B*57:52	57:52	HLA06989		0	0	4	0	0	0	0	4									
B*57:54	57:54	HLA07456		0	0	4	0	0	0	0	4									
B*57:56	57:56	HLA07708		0	0	1	0	0	0	0	1									
B*57:58	57:58	HLA08073		0	0	4	0	0	0	1	5								WD	WD
B*57:60	57:60	HLA08371		0	0	1	0	0	0	0	1									
B*57:61	57:61	HLA08927		0	14	0	1	0	0	0	15		I						WD	I
B*57:62	57:62	HLA08997		0	0	1	0	0	0	0	1									
B*57:63	57:63	HLA09303		0	0	0	0	1	0	0	1									
B*57:65	57:65	HLA09577		0	0	1	0	0	0	0	1									
**B*57:67 total**	**57:67 total**			**0**	**0**	**0**	**0**	**0**	**0**	**1**	**1**									
B*57:67:02	57:67:02	HLA14152		0	0	0	0	0	0	1	1									
B*57:69	57:69	HLA10408		0	0	0	0	0	0	1	1									
B*57:70	57:70	HLA11328		0	0	0	1	0	0	0	1									
B*57:73	57:73	HLA12263		0	2	0	0	0	0	0	2									
B*57:75	57:75	HLA13002		0	0	0	1	0	0	0	1									
B*57:76	57:76	HLA13004		0	0	1	0	0	0	0	1									
B*57:77	57:77	HLA13480		0	3	0	0	0	0	0	3									
B*57:78	57:78	HLA13379		0	0	0	1	0	0	0	1									
B*57:81	57:81	HLA14308		0	0	3	0	0	0	0	3									
B*57:83	57:83	HLA14682		0	0	1	0	0	0	0	1									
B*57:86	57:86	HLA16223		0	0	2	0	0	0	0	2									
B*57:CODE	57:CODE			785	640	19233	163	1226	155	2274	24476	NA	NA	NA	NA	NA	NA	NA	NA	NA

Supplemental Table 9: HLA-B Allele Summary [a]				Allele Count by Population Group [b]								3.0.0 CIWD Category by Population Group [c]								
Allele	Genomic typing	Allele ID	G group	AFA	API	EURO	MENA	HIS	NAM	UNK	Total	AFA	API	EURO	MENA	HIS	NAM	UNK	Total	Highest Frequency
**B*58:01 total**	**58:01 total**			**13885**	**63823**	**85923**	**7426**	**7695**	**1019**	**17460**	**197231**	**C**	**C**	**C**	**C**	**C**	**C**	**C**	**C**	**C**
B*58:01	58:01			6	8	854	5	5	0	65	943	WD	WD	I	WD	WD		I	I	I
B*58:01P	58:01P			4	1	245	3	2	0	2	257			I					I	I
**B*58:01:01G total**	**58:01:01G total**			**13875**	**63813**	**84822**	**7416**	**7688**	**1019**	**17393**	**196026**	**C**	**C**	**C**	**C**	**C**	**C**	**C**	**C**	**C**
B*58:01:01G	58:01:01G		58:01:01G	10911	57011	80561	7169	5591	725	15269	177237	C	C	C	C	C	C	C	C	C
B*58:01:01	58:01:01		58:01:01G	1776	4331	2329	157	1285	147	1365	11390	C	C	C	C	C	C	C	C	C
B*58:01:01:01	58:01:01:01	HLA00386	58:01:01G	1156	2403	1883	89	804	145	725	7205	C	C	C	C	C	C	C	C	C
B*58:01:01:03	58:01:01:03	HLA16308	58:01:01G	0	0	9	0	0	0	0	9			WD					WD	WD
B*58:01:01:04	58:01:01:04	HLA16752	58:01:01G	2	2	40	1	7	1	10	63			WD		WD		WD	WD	WD
B*58:01:06	58:01:06	HLA05370	58:01:01G	0	66	0	0	0	0	15	81		I					I	WD	I
B*58:11	58:11	HLA02201	58:01:01G	30	0	0	0	1	1	9	41	WD						WD	WD	WD
B*58:01:05	58:01:05	HLA05298		0	0	0	1	0	0	0	1									
B*58:01:10	58:01:10	HLA07647		0	1	0	0	0	0	0	1									
B*58:01:11	58:01:11	HLA07648		0	0	1	0	0	0	0	1									
B*58:01:14	58:01:14	HLA09896		0	0	1	0	0	0	0	1									
B*58:01:16	58:01:16	HLA12481		0	0	0	1	0	0	0	1									
**B*58:02 total**	**58:02 total**			**14181**	**71**	**940**	**610**	**2115**	**368**	**3584**	**21869**	**C**	**I**	**I**	**C**	**C**	**C**	**C**	**C**	**C**
B*58:02	58:02			11333	49	635	580	1612	305	2666	17180	C	I	I	C	C	C	C	C	C
B*58:02P	58:02P			0	0	0	0	1	0	0	1									
B*58:02:01	58:02:01	HLA00387		2847	22	305	30	502	63	918	4687	C	I	I	WD	C	C	C	C	C
B*58:02:02	58:02:02	HLA14456		1	0	0	0	0	0	0	1									
B*58:04	58:04	HLA01142		5	0	2	0	0	0	1	8	WD							WD	WD
B*58:05	58:05	HLA01181		0	0	30	0	0	0	1	31			WD					WD	WD
B*58:06	58:06	HLA01312		4	0	0	0	0	0	2	6								WD	WD
B*58:07	58:07	HLA01680		1	0	0	0	0	0	0	1									
**B*58:08 total**	**58:08 total**			**0**	**11**	**4**	**1**	**0**	**0**	**0**	**16**		**WD**						**WD**	**WD**
B*58:08	58:08			0	0	0	1	0	0	0	1									
B*58:08:01	58:08:01	HLA01723		0	9	3	0	0	0	0	12		WD						WD	WD
B*58:08:02	58:08:02	HLA06831		0	2	1	0	0	0	0	3									
B*58:09	58:09	HLA01728		0	0	0	3	0	0	0	3									
B*58:14	58:14	HLA02712		0	1	0	0	0	0	0	1									

Supplemental Table 9: HLA-B Allele Summary [a]				Allele Count by Population Group [b]								3.0.0 CIWD Category by Population Group [c]								
Allele	Genomic typing	Allele ID	G group	AFA	API	EURO	MENA	HIS	NAM	UNK	Total	AFA	API	EURO	MENA	HIS	NAM	UNK	Total	Highest Frequency
**B*58:16 total**	**58:16 total**			**0**	**0**	**5**	**0**	**1**	**0**	**1**	**7**			**WD**					**WD**	**WD**
B*58:16	58:16			0	0	1	0	0	0	0	1									
B*58:16:02	58:16:02	HLA09572		0	0	4	0	1	0	1	6								WD	WD
B*58:18	58:18	HLA03316		0	68	1	0	0	0	0	69		I						WD	I
**B*58:19 total**	**58:19 total**			**0**	**3**	**0**	**0**	**0**	**0**	**1**	**4**									
**B*58:19:01G total**	**58:19:01G total**			**0**	**3**	**0**	**0**	**0**	**0**	**1**	**4**									
B*58:19	58:19	HLA03369	58:19:01G	0	3	0	0	0	0	1	4									
B*58:22	58:22	HLA03968		0	1	0	0	0	0	1	2									
B*58:23	58:23	HLA04087		0	0	19	0	0	0	0	19			WD					WD	WD
B*58:24	58:24	HLA04025		0	0	1	0	0	0	0	1									
B*58:25	58:25	HLA04506		1	0	1	0	0	0	0	2									
B*58:26	58:26	HLA04397		0	1	0	0	0	0	0	1									
**B*58:28 total**	**58:28 total**			**0**	**5**	**0**	**0**	**0**	**0**	**0**	**5**		**WD**						**WD**	**WD**
B*58:28:02	58:28:02	HLA13001		0	5	0	0	0	0	0	5		WD						WD	WD
B*58:33	58:33	HLA06226		0	0	1	2	0	0	1	4									
B*58:34	58:34	HLA06649		0	0	0	1	0	0	0	1									
B*58:35	58:35	HLA07199		1	0	1	1	0	0	0	3									
B*58:37	58:37	HLA08067		0	0	5	0	0	0	0	5			WD					WD	WD
B*58:38	58:38	HLA08365		1	0	0	0	0	0	0	1									
B*58:41	58:41	HLA09302		0	1	0	0	0	0	0	1									
B*58:46	58:46	HLA09698		1	0	0	0	0	0	0	1									
B*58:48	58:48	HLA10146		0	2	0	0	0	0	0	2									
**B*58:59 total**	**58:59 total**			**0**	**1**	**0**	**0**	**0**	**0**	**0**	**1**									
B*58:59:02	58:59:02	HLA14306		0	1	0	0	0	0	0	1									
B*58:60	58:60	HLA12124		1	0	0	0	0	0	0	1									
B*58:62	58:62	HLA12284		0	9	0	0	0	0	0	9		WD						WD	WD
B*58:64	58:64	HLA12435		0	1	0	0	0	0	0	1									
B*58:65	58:65	HLA12566		0	0	0	0	1	0	0	1									
B*58:68	58:68	HLA12749		0	0	0	0	0	0	1	1									
B*58:73	58:73	HLA13856		0	1	0	0	0	0	0	1									
B*58:74	58:74	HLA14234		0	3	0	0	0	0	0	3									

Supplemental Table 9: HLA-B Allele Summary [a]				Allele Count by Population Group [b]								3.0.0 CIWD Category by Population Group [c]								
**Allele**	**Genomic typing**	**Allele ID**	**G group**	**AFA**	**API**	**EURO**	**MENA**	**HIS**	**NAM**	**UNK**	**Total**	**AFA**	**API**	**EURO**	**MENA**	**HIS**	**NAM**	**UNK**	**Total**	**Highest Frequency**
B*58:CODE	58:CODE			853	1339	5115	385	589	64	1110	9455	NA	NA	NA	NA	NA	NA	NA	NA	NA
**B*59:01 total**	**59:01 total**			**2**	**1009**	**37**	**0**	**10**	**3**	**347**	**1408**		**C**	**WD**		**I**		**C**	**I**	**C**
**B*59:01:01G total**	**59:01:01G total**			**2**	**1009**	**37**	**0**	**10**	**3**	**347**	**1408**		**C**	**WD**		**I**		**C**	**I**	**C**
B*59:01	59:01		59:01:01G	0	80	4	0	2	0	33	119		I					I	WD	I
B*59:01:01G	59:01:01G		59:01:01G	0	311	24	0	3	1	91	430		C	WD				I	I	C
B*59:01:01	59:01:01		59:01:01G	2	557	8	0	5	2	210	784		C	WD		WD		C	I	C
B*59:01:01:01	59:01:01:01	HLA00389	59:01:01G	0	4	0	0	0	0	1	5								WD	WD
B*59:01:01:02	59:01:01:02	HLA06075	59:01:01G	0	57	1	0	0	0	12	70		I					WD	WD	I
B*59:CODE	59:CODE			0	49	2	0	0	0	7	58	NA	NA	NA	NA	NA	NA	NA	NA	NA
**B*67:01 total**	**67:01 total**			**14**	**1214**	**601**	**13**	**71**	**5**	**324**	**2242**	**WD**	**C**	**I**	**WD**	**C**	**WD**	**C**	**C**	**C**
B*67:01	67:01			4	98	46	0	16	2	28	194		I	WD		I		I	I	I
B*67:01:01	67:01:01	HLA00390		1	952	70	13	2	0	203	1241		C	WD	WD			C	I	C
**B*67:01:02G total**	**67:01:02G total**			**9**	**164**	**485**	**0**	**53**	**3**	**93**	**807**	**WD**	**C**	**I**		**I**		**I**	**I**	**C**
B*67:01:02G	67:01:02G		67:01:02G	2	56	398	0	28	0	54	538		I	I		I		I	I	I
B*67:01:02	67:01:02	HLA00391	67:01:02G	7	108	87	0	25	3	39	269	WD	I	WD		I		I	I	I
B*67:02	67:02	HLA01374		2	0	1	1	1	0	0	5								WD	WD
B*67:CODE	67:CODE			0	11	1	0	0	0	3	15	NA	NA	NA	NA	NA	NA	NA	NA	NA
B*73:01	73:01	HLA00392		256	231	6845	1558	524	58	1054	10526	C	C	C	C	C	C	C	C	C
B*73:CODE	73:CODE			0	0	164	0	0	0	2	166	NA	NA	NA	NA	NA	NA	NA	NA	NA
**B*78:01 total**	**78:01 total**			**3192**	**24**	**293**	**74**	**737**	**148**	**891**	**5359**	**C**	**I**	**I**	**C**	**C**	**C**	**C**	**C**	**C**
B*78:01	78:01			389	2	55	5	119	15	102	687	C		WD	WD	C	C	I	I	C
**B*78:01:01G total**	**78:01:01G total**			**2803**	**22**	**234**	**69**	**617**	**133**	**788**	**4666**	**C**	**I**	**I**	**C**	**C**	**C**	**C**	**C**	**C**
B*78:01:01G	78:01:01G		78:01:01G	118	15	26	1	21	0	43	224	C	I	WD		I		I	I	C
B*78:01:01	78:01:01		78:01:01G	2482	6	201	67	527	114	680	4077	C	WD	I	C	C	C	C	C	C
B*78:01:01:02	78:01:01:02	HLA16309	78:01:01G	203	1	7	1	69	19	65	365	C		WD		I	C	I	I	C
B*78:01:02	78:01:02	HLA06937		0	0	4	0	1	0	1	6								WD	WD
**B*78:02 total**	**78:02 total**			**3**	**0**	**188**	**3**	**7**	**3**	**21**	**225**			**I**		**WD**		**I**	**I**	**I**
B*78:02	78:02			1	0	17	0	1	0	1	20			WD					WD	WD
B*78:02:01	78:02:01	HLA00394		0	0	31	2	1	0	4	38			WD					WD	WD
B*78:02:02	78:02:02	HLA00395		2	0	140	1	5	3	16	167			I		WD		I	I	I
B*78:03	78:03	HLA00396		0	0	1	0	0	0	0	1									

Supplemental Table 9: HLA-B Allele Summary [a]				Allele Count by Population Group [b]								3.0.0 CIWD Category by Population Group [c]								
**Allele**	**Genomic typing**	**Allele ID**	**G group**	**AFA**	**API**	**EURO**	**MENA**	**HIS**	**NAM**	**UNK**	**Total**	**AFA**	**API**	**EURO**	**MENA**	**HIS**	**NAM**	**UNK**	**Total**	**Highest Frequency**
B*78:04	78:04	HLA00990		1	1	2	0	61	2	7	74					I		WD	WD	I
B*78:05	78:05	HLA01403		0	1	1	0	0	0	0	2									
B*78:08	78:08	HLA12300		0	0	0	0	1	0	0	1									
B*78:09	78:09	HLA13984		0	0	1	0	0	0	0	1									
B*78:CODE	78:CODE			108	0	47	0	34	5	17	211	NA	NA	NA	NA	NA	NA	NA	NA	NA
**B*81:01 total**	**81:01 total**			**7085**	**272**	**433**	**126**	**1046**	**142**	**1724**	**10828**	**C**	**C**	**I**	**C**	**C**	**C**	**C**	**C**	**C**
**B*81:01:01G total**	**81:01:01G total**			**7085**	**272**	**433**	**126**	**1046**	**142**	**1724**	**10828**	**C**	**C**	**I**	**C**	**C**	**C**	**C**	**C**	**C**
B*81:01	81:01	HLA00398	81:01:01G	1142	4	40	2	207	37	279	1711	C		WD		C	C	C	C	C
B*81:01P	81:01P			2	0	2	0	0	0	0	4									
B*81:01:01G	81:01:01G		81:01:01G	5936	238	391	124	839	105	1442	9075	C	C	I	C	C	C	C	C	C
B*81:02	81:02	HLA01809	81:01:01G	5	30	0	0	0	0	3	38	WD	I						WD	I
B*81:04N	81:04N	HLA03261		0	0	1	0	1	0	0	2									
B*81:05	81:05	HLA05306		7	0	0	0	0	0	0	7	WD							WD	WD
B*81:06	81:06	HLA08387		0	0	0	0	0	0	1	1									
B*81:07	81:07	HLA11447		1	0	0	0	0	0	0	1									
B*81:CODE	81:CODE			9	0	1	0	1	0	1	12	NA	NA	NA	NA	NA	NA	NA	NA	NA
B*82:01	82:01	HLA00399		925	4	45	1	213	34	191	1413	C		WD		C	C	C	I	C
**B*82:02 total**	**82:02 total**			**115**	**2**	**23**	**60**	**7**	**3**	**34**	**244**	**C**		**WD**	**C**	**WD**		**I**	**I**	**C**
B*82:02	82:02			50	1	9	7	2	0	12	81	C		WD	WD			WD	WD	C
B*82:02:01	82:02:01	HLA01188		65	1	14	53	5	3	22	163	C		WD	C	WD		I	I	C
B*82:03	82:03	HLA04447		1	0	0	0	0	0	0	1									
B*82:CODE	82:CODE			3	0	2	0	0	0	0	5	NA	NA	NA	NA	NA	NA	NA	NA	NA
B*83:01	83:01	HLA01135		0	0	13	0	0	0	1	14			WD					WD	WD
B*83:CODE	83:CODE			0	0	0	0	0	0	1	1	NA	NA	NA	NA	NA	NA	NA	NA	NA
B*NEW [d]	NEW			0	1	1	0	0	0	0	2	NA	NA	NA	NA	NA	NA	NA	NA	NA
**B*Total [e]**	**Total**			**388579**	**1298351**	**11941489**	**402160**	**700912**	**66967**	**1320714**	**16119172**									

C, common; I, intermediate; WD, well-documented; NA, not applicable

Supplemental Table 9: HLA-B Allele Summary [a]				Allele Count by Population Group [b]								3.0.0 CIWD Category by Population Group [c]								
Allele	Genomic typing	Allele ID	G group	AFA	API	EURO	MENA	HIS	NAM	UNK	Total	AFA	API	EURO	MENA	HIS	NAM	UNK	Total	Highest Frequency

[a] All alleles observed in the current dataset are included in this table. Note that alleles are not in numerical order; alleles within a G group are clustered together. P group "two-field" total (e.g., written as "B*07:02 total") and G group total summary rows are provided. The table does not list all alleles from IPD-IMGT version 3.31.0, if not present in the study dataset.

[b] Population groups include: AFA (African/African American), API (Asian/Pacific Islands), EURO (European/European descent), MENA (Middle East/North Coast of Africa), HIS (South or Central America/Hispanic/Latino), NAM (Native American populations) and UNK (unknown/not asked/multiple ancestries/other). Total is the overall population i.e., all groups combined.

[c] Allele frequency is calculated by dividing the number of times the “allele” of interest is observed in a population by the total number of copies of all the alleles at that particular genetic locus in the population (reported as the last row in this table and also in Table 2b). The total number of copies is calculated by multiplying the number of individuals times two for all loci except DRB3/4/5. For DRB3/4/5, the number of assignments was used as the total. The CIWD status is determined based on the allele frequency. Allele frequency data will be provided on the website of the next International HLA and Immunogenetics Workshop (https://www.ihiw18.org/). Highest frequency is the highest CIWD designation among all the individual groups.

[d] "CODE" is generically defined as a summary category of submitted HLA typing, including NMDP multiple allele codes, with ambiguities that are not within a single P or G group. "NEW" is a summary category for assignments of novel alleles that did not yet receive a nomenclature assignment. The CODE and NEW categories add to the total number of alleles but should not be assigned CIWD designations (labeled as NA, not applicable) as they do not represent a consistent allele designation (i.e., the NEW category may contain alleles with different DNA sequences that are unrelated to one another).

[e] B*Total is the total number of allele assignments for the population group and is based on two times the number of individuals in the group. This number is also listed in Table 2b. It is not the sum of the column as alleles are not counted more than once. For example, when evaluating frequencies at the level of G resolution, individual alleles that make up the G group (e.g., A*80:01:01, A*80:01:01:01, A*80:01:01:02, A*80:01:01G) are not included in the count because these alleles are summed up in the total G designation (e.g., "A*80:01:01G total").
