# Supplementary material for: Common, intermediate and well‐documented HLA alleles in world populations: CIWD version 3.0.0
Source: HLA. 2020 Jan 31;95(6):516–31. doi: 10.1111/tan.13811 (PMC7317522; doi:10.1111/tan.13811)
Supplement: Supplementary file 10 — Table S10 HLA‐C primary data [file TAN-95-516-s010.pdf]

Supplemental Table 10: HLA-C Allele Summary [a]				Allele Count by Population Group [b]								3.0.0 CIWD Category by Population Group [c]								
Allele	Genomic typing	Allele ID	G group	AFA	API	EURO	MENA	HIS	NAM	UNK	Total	AFA	API	EURO	MENA	HIS	NAM	UNK	Total	Highest Frequency
**C*01:02 total**	**01:02 total**			**3139**	**82700**	**386694**	**11546**	**30050**	**2576**	**42956**	**559661**	**C**	**C**	**C**	**C**	**C**	**C**	**C**	**C**	**C**
C*01:02	01:02			2	26	2091	3	44	2	99	2267		I	C		I		I	C	C
C*01:02P	01:02P			0	3	450	1	9	0	4	467			I		I			I	I
**C*01:02:01G total**	**01:02:01G total**			**3133**	**82341**	**383811**	**11538**	**29962**	**2572**	**42801**	**556158**	**C**	**C**	**C**	**C**	**C**	**C**	**C**	**C**	**C**
C*01:02:01G	01:02:01G		01:02:01G	2507	72791	366919	11271	22425	1869	38950	516732	C	C	C	C	C	C	C	C	C
C*01:02:01	01:02:01		01:02:01G	602	9131	15757	245	6893	638	3630	36896	C	C	C	C	C	C	C	C	C
C*01:02:01:01	01:02:01:01	HLA00401	01:02:01G	19	371	1043	19	400	36	194	2082	WD	C	I	WD	C	C	C	C	C
C*01:02:01:03	01:02:01:03	HLA16866	01:02:01G	0	0	10	0	1	0	1	12			WD					WD	WD
C*01:02:01:04	01:02:01:04	HLA17051	01:02:01G	0	0	1	0	3	0	1	5								WD	WD
C*01:02:12	01:02:12	HLA05781	01:02:01G	0	1	0	0	0	0	0	1									
C*01:02:30	01:02:30	HLA12437	01:02:01G	5	5	14	2	237	29	21	313	WD	WD	WD		C	C	I	I	C
C*01:44	01:44	HLA05762	01:02:01G	0	41	13	1	1	0	2	58		I	WD					WD	I
C*01:85	01:85	HLA10566	01:02:01G	0	1	0	0	0	0	0	1									
C*01:127	01:127	HLA15575	01:02:01G	0	0	54	0	2	0	2	58			WD					WD	WD
C*01:02:03	01:02:03	HLA02044		1	327	6	2	0	0	29	365		C	WD				I	I	C
C*01:02:04	01:02:04	HLA02670		2	1	26	2	1	0	3	35			WD					WD	WD
C*01:02:06	01:02:06	HLA03445		0	0	2	0	0	0	1	3									
C*01:02:07	01:02:07	HLA03730		0	0	69	0	0	0	1	70			WD					WD	WD
C*01:02:08	01:02:08	HLA04275		0	0	219	0	0	0	3	222			I					I	I
C*01:02:09	01:02:09	HLA04606		0	0	4	0	0	0	0	4									
C*01:02:10	01:02:10	HLA05209		0	0	2	0	5	0	4	11					WD			WD	WD
C*01:02:16	01:02:16	HLA08145		1	0	2	0	29	2	9	43					I		WD	WD	I
C*01:02:18	01:02:18	HLA08885		0	0	1	0	0	0	0	1									
C*01:02:22	01:02:22	HLA09953		0	0	0	0	0	0	1	1									
C*01:02:27	01:02:27	HLA11850		0	0	1	0	0	0	0	1									
C*01:02:32	01:02:32	HLA12495		0	0	2	0	0	0	0	2									
C*01:02:34	01:02:34	HLA12670		0	0	4	0	0	0	0	4									
C*01:02:36	01:02:36	HLA13526		0	0	2	0	0	0	0	2									
C*01:02:37	01:02:37	HLA14597		0	1	0	0	0	0	0	1									
C*01:02:40	01:02:40	HLA15195		0	0	1	0	0	0	0	1									
C*01:02:41	01:02:41	HLA15929		0	0	1	0	0	0	0	1									

Supplemental Table 10: HLA-C Allele Summary [a]				Allele Count by Population Group [b]								3.0.0 CIWD Category by Population Group [c]								
Allele	Genomic typing	Allele ID	G group	AFA	API	EURO	MENA	HIS	NAM	UNK	Total	AFA	API	EURO	MENA	HIS	NAM	UNK	Total	Highest Frequency
C*01:40	01:40	HLA05418		0	1	0	0	0	0	1	2									
**C*01:03 total**	**01:03 total**			**1**	**1026**	**93**	**48**	**2**	**0**	**106**	**1276**		**C**	**WD**	**C**			**I**	**I**	**C**
**C*01:03:01G total**	**01:03:01G total**			**1**	**1026**	**93**	**48**	**2**	**0**	**106**	**1276**		**C**	**WD**	**C**			**I**	**I**	**C**
C*01:03	01:03	HLA00402	01:03:01G	0	244	7	0	2	0	27	280		C	WD				I	I	C
C*01:03:01G	01:03:01G		01:03:01G	1	782	86	48	0	0	79	996		C	WD	C			I	I	C
C*01:04	01:04	HLA01075		0	6	0	0	0	0	0	6		WD						WD	WD
C*01:05	01:05	HLA01434		0	0	0	0	0	0	1	1									
C*01:06	01:06	HLA01555		0	82	13	2	1	0	10	108		I	WD				WD	WD	I
**C*01:07 total**	**01:07 total**			**0**	**0**	**158**	**0**	**2**	**1**	**5**	**166**			**I**				**WD**	**I**	**I**
C*01:07	01:07			0	0	81	0	1	1	3	86			WD					WD	WD
C*01:07:01	01:07:01	HLA01644		0	0	77	0	1	0	1	79			WD					WD	WD
C*01:07:02	01:07:02	HLA12493		0	0	0	0	0	0	1	1									
C*01:08	01:08	HLA01636		0	152	3	0	0	0	13	168		C					WD	I	C
C*01:09	01:09	HLA01659		0	1	3	0	0	0	0	4									
C*01:10	01:10	HLA01866		0	0	8	0	48	1	7	64			WD		I		WD	WD	I
**C*01:12 total**	**01:12 total**			**0**	**2**	**5**	**4**	**0**	**0**	**1**	**12**			**WD**					**WD**	**WD**
C*01:12	01:12			0	1	1	1	0	0	1	4									
C*01:12:01	01:12:01	HLA02469		0	1	4	3	0	0	0	8								WD	WD
C*01:13	01:13	HLA02478		1	0	6	0	1	0	2	10			WD					WD	WD
C*01:14	01:14	HLA02790		0	1	4	0	0	0	0	5								WD	WD
**C*01:15 total**	**01:15 total**			**0**	**0**	**1**	**0**	**0**	**0**	**0**	**1**									
C*01:15	01:15			0	0	1	0	0	0	0	1									
C*01:16	01:16	HLA02812		1	0	0	0	0	0	0	1									
C*01:17	01:17	HLA02813		3	480	13	21	0	0	4	521		C	WD	WD				I	C
C*01:18	01:18	HLA02814		0	4	0	0	0	0	0	4									
C*01:22	01:22	HLA03363		0	0	21	0	7	0	6	34			WD		I		WD	WD	I
C*01:27	01:27	HLA03707		0	1	57	0	3	0	7	68			WD				WD	WD	WD
C*01:30	01:30	HLA04020		0	3	1	2	0	0	0	6								WD	WD
**C*01:32 total**	**01:32 total**			**0**	**3**	**154**	**0**	**0**	**0**	**7**	**164**			**I**				**WD**	**I**	**I**
C*01:32	01:32			0	3	112	0	0	0	7	122			WD				WD	WD	WD
C*01:32:01	01:32:01	HLA04289		0	0	42	0	0	0	0	42			WD					WD	WD

Supplemental Table 10: HLA-C Allele Summary [a]				Allele Count by Population Group [b]								3.0.0 CIWD Category by Population Group [c]								
Allele	Genomic typing	Allele ID	G group	AFA	API	EURO	MENA	HIS	NAM	UNK	Total	AFA	API	EURO	MENA	HIS	NAM	UNK	Total	Highest Frequency
C*01:33	01:33	HLA04290		0	1	4	0	0	0	0	5								WD	WD
C*01:36	01:36	HLA04756		0	0	1	0	0	0	0	1									
C*01:37N	01:37N	HLA04852		0	1	0	0	0	0	0	1									
C*01:39	01:39	HLA05130		0	0	0	0	0	0	1	1									
C*01:41	01:41	HLA05574		0	1	2	0	0	0	0	3									
C*01:45	01:45	HLA05873		1	0	20	0	0	0	1	22			WD					WD	WD
C*01:51	01:51	HLA06305		0	1	4	0	0	0	0	5								WD	WD
C*01:52	01:52	HLA06386		0	0	11	0	0	0	0	11			WD					WD	WD
C*01:54	01:54	HLA06587		0	3	10	0	0	0	4	17			WD					WD	WD
C*01:56N	01:56N	HLA06704		0	2	0	0	0	0	0	2									
C*01:57	01:57	HLA06731		0	1	0	0	0	0	0	1									
C*01:58	01:58	HLA07121		0	0	10	8	0	0	1	19			WD	WD				WD	WD
C*01:59	01:59	HLA07213		0	0	11	0	0	0	0	11			WD					WD	WD
C*01:60	01:60	HLA07214		0	0	4	0	0	0	0	4									
C*01:62	01:62	HLA07475		0	0	8	0	0	0	1	9			WD					WD	WD
**C*01:63 total**	**01:63 total**			**0**	**0**	**2**	**0**	**0**	**0**	**0**	**2**									
C*01:63	01:63			0	0	1	0	0	0	0	1									
C*01:141	01:141	HLA07476		0	0	1	0	0	0	0	1									
C*01:64	01:64	HLA07528		0	1	2	0	0	0	0	3									
C*01:65	01:65	HLA07556		0	0	3	0	0	0	0	3									
C*01:66	01:66	HLA07729		0	0	1	0	0	0	0	1									
C*01:67	01:67	HLA07941		1	0	1	0	3	0	0	5								WD	WD
C*01:70	01:70	HLA08146		0	0	24	0	0	0	2	26			WD					WD	WD
C*01:72	01:72	HLA08178		0	0	1	0	0	0	0	1									
C*01:74	01:74	HLA09012		0	0	16	0	0	0	1	17			WD					WD	WD
C*01:75	01:75	HLA09111		0	0	0	0	0	0	1	1									
C*01:77	01:77	HLA09669		0	1	0	0	0	0	0	1									
C*01:92	01:92	HLA11040		0	0	1	0	0	0	0	1									
C*01:93	01:93	HLA11250		0	0	1	0	0	0	3	4									
C*01:95	01:95	HLA11848		0	1	0	0	0	0	0	1									
C*01:96	01:96	HLA11849		0	0	1	0	0	0	4	5								WD	WD

Supplemental Table 10: HLA-C Allele Summary [a]				Allele Count by Population Group [b]								3.0.0 CIWD Category by Population Group [c]								
Allele	Genomic typing	Allele ID	G group	AFA	API	EURO	MENA	HIS	NAM	UNK	Total	AFA	API	EURO	MENA	HIS	NAM	UNK	Total	Highest Frequency
C*01:97	01:97	HLA11851		0	1	0	1	0	0	0	2									
C*01:99	01:99	HLA11972		0	0	2	0	0	0	0	2									
C*01:100	01:100	HLA12135		0	3	0	0	0	0	0	3									
C*01:101	01:101	HLA12432		0	1	0	0	0	0	0	1									
C*01:102	01:102	HLA12436		0	1	0	0	0	0	0	1									
C*01:103	01:103	HLA12494		0	0	5	0	0	0	0	5			WD					WD	WD
C*01:104	01:104	HLA12497		0	0	0	0	0	0	1	1									
C*01:107	01:107	HLA12738		0	1	1	0	0	0	0	2									
C*01:114	01:114	HLA14019		0	1	0	0	0	0	0	1									
C*01:117N	01:117N	HLA14213		0	0	3	0	0	0	0	3									
C*01:119	01:119	HLA14520		0	0	1	0	0	0	0	1									
C*01:121Q	01:121Q	HLA14993		0	0	2	0	2	0	1	5								WD	WD
C*01:136	01:136	HLA16428		0	1	0	0	0	0	0	1									
C*01:146	01:146	HLA17754		0	0	0	0	0	0	1	1									
C*01:CODE [d]	01:CODE			472	5855	45527	847	4312	458	5656	63127	NA	NA	NA	NA	NA	NA	NA	NA	NA
**C*02:02 total**	**02:02 total**			**29264**	**9442**	**596689**	**12476**	**25268**	**2979**	**49975**	**726093**	**C**	**C**	**C**	**C**	**C**	**C**	**C**	**C**	**C**
C*02:02	02:02			10	6	3851	19	17	1	158	4062	WD	WD	C	WD	I		C	C	C
C*02:02P	02:02P			218	3	768	8	96	4	80	1177	C		I	WD	C		I	I	C
C*02:02:01	02:02:01	HLA00404		1	0	16	0	0	0	0	17			WD					WD	WD
**C*02:02:02G total**	**02:02:02G total**			**6264**	**9202**	**585646**	**11476**	**18909**	**2168**	**42916**	**676581**	**C**	**C**	**C**	**C**	**C**	**C**	**C**	**C**	**C**
C*02:02:02G	02:02:02G		02:02:02G	4774	8192	554005	11002	12828	1481	38521	630803	C	C	C	C	C	C	C	C	C
C*02:02:02	02:02:02		02:02:02G	1063	777	25582	388	4435	514	3490	36249	C	C	C	C	C	C	C	C	C
C*02:02:02:01	02:02:02:01	HLA00405	02:02:02G	416	230	5576	84	1597	172	852	8927	C	C	C	C	C	C	C	C	C
C*02:02:02:02	02:02:02:02	HLA10679	02:02:02G	11	1	399	1	43	1	47	503	WD		I		I		I	I	I
C*02:02:02:03	02:02:02:03	HLA14843	02:02:02G	0	0	38	0	0	0	0	38			WD					WD	WD
C*02:02:02:04	02:02:02:04	HLA15769	02:02:02G	0	0	1	0	0	0	0	1									
C*02:02:02:06	02:02:02:06	HLA16698	02:02:02G	0	0	7	0	0	0	0	7			WD					WD	WD
C*02:02:02:10	02:02:02:10	HLA16702	02:02:02G	0	1	0	0	0	0	0	1									
C*02:02:02:12	02:02:02:12	HLA17059	02:02:02G	0	0	1	0	3	0	0	4									
C*02:02:02:13	02:02:02:13	HLA17160	02:02:02G	0	0	1	0	0	0	0	1									
C*02:29	02:29	HLA04016	02:02:02G	0	1	33	0	3	0	6	43			WD				WD	WD	WD

Supplemental Table 10: HLA-C Allele Summary [a]				Allele Count by Population Group [b]								3.0.0 CIWD Category by Population Group [c]								
**Allele**	**Genomic typing**	**Allele ID**	**G group**	**AFA**	**API**	**EURO**	**MENA**	**HIS**	**NAM**	**UNK**	**Total**	**AFA**	**API**	**EURO**	**MENA**	**HIS**	**NAM**	**UNK**	**Total**	**Highest Frequency**
C*02:69	02:69	HLA09921	02:02:02G	0	0	1	0	0	0	0	1									
C*02:83	02:83	HLA11764	02:02:02G	0	0	2	1	0	0	0	3									
C*02:02:03	02:02:03	HLA00406		0	0	69	21	2	0	7	99			WD	WD			WD	WD	WD
C*02:02:05	02:02:05	HLA01471		0	0	0	0	0	0	1	1									
C*02:02:06	02:02:06	HLA03081		0	0	11	0	0	0	0	11			WD					WD	WD
C*02:02:07	02:02:07	HLA03731		0	4	156	0	1	1	7	169			I				WD	I	I
C*02:02:08	02:02:08	HLA03826		0	0	47	0	0	0	0	47			WD					WD	WD
C*02:02:09	02:02:09	HLA04593		0	0	53	0	0	0	0	53			WD					WD	WD
C*02:02:12	02:02:12	HLA05210		0	0	8	0	0	0	0	8			WD					WD	WD
C*02:02:14	02:02:14	HLA05567		0	0	2	0	0	0	3	5								WD	WD
C*02:02:15	02:02:15	HLA05594		0	0	0	1	0	0	0	1									
C*02:02:16	02:02:16	HLA05606		0	0	0	0	2	0	0	2									
C*02:02:18	02:02:18	HLA06710		0	0	7	0	0	0	0	7			WD					WD	WD
C*02:02:19	02:02:19	HLA06723		0	0	3	0	0	0	0	3									
C*02:02:20	02:02:20	HLA06736		0	0	1	0	0	0	0	1									
C*02:02:22	02:02:22	HLA07237		0	0	3	0	0	0	0	3									
C*02:02:23	02:02:23	HLA08165		0	0	3	0	0	0	0	3									
C*02:02:24	02:02:24	HLA08210		0	0	4	0	0	0	0	4									
C*02:02:26	02:02:26	HLA09202		0	0	1	0	0	0	0	1									
C*02:02:27	02:02:27	HLA11867		0	0	2	0	1	0	0	3									
C*02:02:28	02:02:28	HLA12195		0	0	1	0	0	0	0	1									
**C*02:10 total**	**02:10 total**			**22771**	**227**	**6037**	**951**	**6240**	**805**	**6803**	**43834**	**C**	**C**	**C**	**C**	**C**	**C**	**C**	**C**	**C**
C*02:10	02:10			18076	150	3857	811	4664	666	4840	33064	C	C	C	C	C	C	C	C	C
**C*02:10:01G total**	**02:10:01G total**			**4695**	**77**	**2180**	**140**	**1576**	**139**	**1963**	**10770**	**C**	**I**	**C**	**C**	**C**	**C**	**C**	**C**	**C**
C*02:10:01G	02:10:01G		02:10:01G	2214	59	1876	107	588	10	1231	6085	C	I	C	C	C	C	C	C	C
C*02:10:01	02:10:01		02:10:01G	828	7	101	24	347	31	322	1660	C	WD	WD	WD	C	C	C	C	C
C*02:10:01:01	02:10:01:01	HLA02120	02:10:01G	1537	11	188	9	599	92	386	2822	C	WD	I	WD	C	C	C	C	C
C*02:10:01:02	02:10:01:02	HLA14218	02:10:01G	116	0	15	0	41	5	23	200	C		WD		I	WD	I	I	C
C*02:10:01:03	02:10:01:03	HLA14809	02:10:01G	0	0	0	0	1	1	1	3									
C*02:03	02:03	HLA00408		4	0	20	0	0	0	3	27			WD					WD	WD
C*02:04	02:04	HLA01344		0	0	6	0	4	5	3	18			WD			WD		WD	WD

Supplemental Table 10: HLA-C Allele Summary [a]				Allele Count by Population Group [b]								3.0.0 CIWD Category by Population Group [c]								
**Allele**	**Genomic typing**	**Allele ID**	**G group**	**AFA**	**API**	**EURO**	**MENA**	**HIS**	**NAM**	**UNK**	**Total**	**AFA**	**API**	**EURO**	**MENA**	**HIS**	**NAM**	**UNK**	**Total**	**Highest Frequency**
**C*02:05 total**	**02:05 total**			**4**	**0**	**4**	**0**	**0**	**0**	**2**	**10**								**WD**	**WD**
C*02:05	02:05			0	0	0	0	0	0	1	1									
C*02:05:02	02:05:02	HLA10259		4	0	2	0	0	0	0	6								WD	WD
C*02:05:03	02:05:03	HLA10640		0	0	2	0	0	0	1	3									
**C*02:06 total**	**02:06 total**			**3**	**2**	**1115**	**0**	**11**	**3**	**31**	**1165**			**I**		**I**		**I**	**I**	**I**
C*02:06	02:06			1	1	742	0	10	3	26	783			I		I		I	I	I
C*02:06:01	02:06:01	HLA01673		2	1	373	0	1	0	5	382			I				WD	I	I
C*02:07	02:07	HLA01715		2	1	187	31	32	0	29	282			I	WD	I		I	I	I
C*02:08	02:08	HLA01739		0	85	113	15	11	0	13	237		I	WD	WD	I		WD	I	I
C*02:11	02:11	HLA02170		0	0	1	0	0	0	0	1									
C*02:12	02:12	HLA02240		9	0	0	0	0	0	2	11	WD							WD	WD
C*02:13	02:13	HLA02423		0	1	36	3	0	0	1	41			WD					WD	WD
**C*02:14 total**	**02:14 total**			**201**	**0**	**10**	**0**	**13**	**0**	**47**	**271**	**C**		**WD**		**I**		**I**	**I**	**C**
**C*02:14:01G total**	**02:14:01G total**			**201**	**0**	**10**	**0**	**13**	**0**	**47**	**271**	**C**		**WD**		**I**		**I**	**I**	**C**
C*02:14	02:14		02:14:01G	132	0	5	0	7	0	27	171	C		WD		I		I	I	C
C*02:14:01G	02:14:01G		02:14:01G	27	0	5	0	3	0	8	43	WD		WD				WD	WD	WD
C*02:14:01	02:14:01	HLA02430	02:14:01G	12	0	0	0	2	0	1	15	WD							WD	WD
C*02:14:02	02:14:02	HLA12913	02:14:01G	30	0	0	0	1	0	11	42	WD						WD	WD	WD
C*02:15	02:15	HLA02559		0	0	1	0	0	0	1	2									
**C*02:16 total**	**02:16 total**			**7**	**1**	**69**	**3**	**0**	**0**	**4**	**84**	**WD**		**WD**					**WD**	**WD**
C*02:16	02:16			0	0	4	2	0	0	0	6								WD	WD
C*02:16:01	02:16:01			7	0	0	0	0	0	3	10	WD							WD	WD
C*02:16:02	02:16:02	HLA03589		0	1	65	1	0	0	1	68			WD					WD	WD
C*02:17	02:17	HLA02764		3	0	0	0	0	0	0	3									
C*02:19	02:19	HLA03024		0	0	61	0	0	0	46	107			WD				I	WD	I
C*02:20	02:20	HLA03079		0	0	1	0	0	0	0	1									
C*02:21	02:21	HLA03252		0	0	9	6	8	0	31	54			WD	WD	I		I	WD	I
C*02:23	02:23	HLA03539		0	0	11	0	0	0	0	11			WD					WD	WD
C*02:24	02:24	HLA03728		0	0	26	0	0	0	0	26			WD					WD	WD
C*02:25Q	02:25Q	HLA03739		0	0	13	0	0	0	0	13			WD					WD	WD
**C*02:26 total**	**02:26 total**			**2**	**1**	**21**	**3**	**4**	**2**	**28**	**61**			**WD**				**I**	**WD**	**I**

Supplemental Table 10: HLA-C Allele Summary [a]				Allele Count by Population Group [b]								3.0.0 CIWD Category by Population Group [c]								
Allele	Genomic typing	Allele ID	G group	AFA	API	EURO	MENA	HIS	NAM	UNK	Total	AFA	API	EURO	MENA	HIS	NAM	UNK	Total	Highest Frequency
C*02:26	02:26			0	0	1	0	1	0	1	3									
C*02:26:01	02:26:01	HLA03704		1	0	9	0	0	0	8	18			WD				WD	WD	WD
C*02:26:02	02:26:02	HLA04699		0	1	10	0	3	2	5	21			WD				WD	WD	WD
C*02:26:03	02:26:03	HLA06397		1	0	1	3	0	0	14	19							I	WD	I
**C*02:27 total**	**02:27 total**			**14**	**0**	**28**	**0**	**11**	**0**	**6**	**59**	**WD**		**WD**		**I**		**WD**	**WD**	**I**
C*02:27	02:27			1	0	4	0	3	0	0	8								WD	WD
C*02:27:01	02:27:01	HLA03705		13	0	23	0	8	0	6	50	WD		WD		I		WD	WD	I
C*02:27:02	02:27:02	HLA05139		0	0	1	0	0	0	0	1									
C*02:28	02:28	HLA03801		0	0	0	0	0	0	4	4									
C*02:30	02:30	HLA04277		0	0	17	0	0	0	0	17			WD					WD	WD
C*02:31	02:31	HLA04284		0	0	9	1	1	0	3	14			WD					WD	WD
C*02:32	02:32	HLA04764		0	0	8	0	0	0	0	8			WD					WD	WD
C*02:34	02:34	HLA04823		0	0	5	0	0	0	1	6			WD					WD	WD
C*02:35	02:35	HLA04830		0	0	4	0	0	0	7	11							WD	WD	WD
**C*02:36 total**	**02:36 total**			**0**	**0**	**1**	**0**	**0**	**0**	**0**	**1**									
C*02:36	02:36			0	0	1	0	0	0	0	1									
C*02:38N	02:38N	HLA05189		0	0	56	0	0	0	0	56			WD					WD	WD
**C*02:40 total**	**02:40 total**			**0**	**0**	**15**	**0**	**0**	**1**	**0**	**16**			**WD**					**WD**	**WD**
C*02:40	02:40			0	0	4	0	0	0	0	4									
C*02:40:01	02:40:01	HLA05311		0	0	11	0	0	0	0	11			WD					WD	WD
C*02:40:02	02:40:02	HLA11665		0	0	0	0	0	1	0	1									
**C*02:43 total**	**02:43 total**			**0**	**0**	**10**	**0**	**0**	**0**	**2**	**12**			**WD**					**WD**	**WD**
C*02:43	02:43			0	0	9	0	0	0	2	11			WD					WD	WD
C*02:43:01	02:43:01	HLA05584		0	0	1	0	0	0	0	1									
C*02:44	02:44	HLA05618		0	0	14	0	1	0	1	16			WD					WD	WD
C*02:45	02:45	HLA05733		0	0	2	4	0	0	1	7								WD	WD
C*02:46	02:46	HLA05735		0	0	5	0	6	0	3	14			WD		WD			WD	WD
C*02:48	02:48	HLA06724		0	0	5	0	0	0	0	5			WD					WD	WD
C*02:49	02:49	HLA06898		8	0	1	0	0	0	0	9	WD							WD	WD
C*02:51	02:51	HLA07020		0	0	4	0	0	0	0	4									
**C*02:53 total**	**02:53 total**			**0**	**0**	**1**	**0**	**0**	**0**	**0**	**1**									

Supplemental Table 10: HLA-C Allele Summary [a]				Allele Count by Population Group [b]								3.0.0 CIWD Category by Population Group [c]								
Allele	Genomic typing	Allele ID	G group	AFA	API	EURO	MENA	HIS	NAM	UNK	Total	AFA	API	EURO	MENA	HIS	NAM	UNK	Total	Highest Frequency
C*02:53	02:53			0	0	1	0	0	0	0	1									
C*02:54	02:54	HLA07236		0	0	1	0	0	0	0	1									
**C*02:55 total**	**02:55 total**			**2**	**0**	**13**	**0**	**0**	**0**	**0**	**15**			**WD**					**WD**	**WD**
C*02:55	02:55			1	0	7	0	0	0	0	8			WD					WD	WD
C*02:55:01	02:55:01	HLA07360		0	0	5	0	0	0	0	5			WD					WD	WD
C*02:55:02	02:55:02	HLA14029		1	0	1	0	0	0	0	2									
C*02:56	02:56	HLA07493		0	0	19	0	0	0	1	20			WD					WD	WD
C*02:57	02:57	HLA07944		0	0	2	0	0	0	0	2									
C*02:60	02:60	HLA08209		0	0	3	0	0	0	0	3									
C*02:62	02:62	HLA08212		0	0	1	0	0	0	1	2									
C*02:64	02:64	HLA08533		0	0	4	0	0	0	0	4									
C*02:67Q	02:67Q	HLA09035		0	0	5	0	0	0	2	7			WD					WD	WD
C*02:73	02:73	HLA10438		1	0	0	0	0	0	1	2									
C*02:74	02:74	HLA10439		0	0	1	0	1	0	0	2									
C*02:75	02:75	HLA11106		0	0	1	0	0	0	0	1									
C*02:76	02:76	HLA11107		2	0	0	0	0	0	0	2									
C*02:77	02:77	HLA11108		0	0	2	0	0	0	0	2									
C*02:78	02:78	HLA11109		0	0	1	0	0	0	0	1									
C*02:80	02:80	HLA11122		0	0	2	0	0	0	0	2									
C*02:81	02:81	HLA11260		0	0	4	0	0	0	0	4									
C*02:84	02:84	HLA11868		0	0	1	0	0	0	0	1									
C*02:85	02:85	HLA12093		0	0	3	0	0	0	0	3									
C*02:86	02:86	HLA12095		0	0	0	1	0	0	0	1									
C*02:87	02:87	HLA12186		0	0	5	0	0	0	0	5			WD					WD	WD
C*02:91	02:91	HLA12708		0	0	0	2	0	0	0	2									
C*02:92N	02:92N	HLA12731		0	0	17	0	0	0	0	17			WD					WD	WD
C*02:93	02:93	HLA12779		0	0	2	0	0	0	0	2									
C*02:94	02:94	HLA12778		1	0	0	0	0	0	0	1									
C*02:96	02:96	HLA13109		0	0	0	0	1	0	0	1									
C*02:97	02:97	HLA13110		0	0	0	0	1	1	0	2									
C*02:98	02:98	HLA13111		0	0	4	0	0	0	0	4									

Supplemental Table 10: HLA-C Allele Summary [a]				Allele Count by Population Group [b]								3.0.0 CIWD Category by Population Group [c]								
Allele	Genomic typing	Allele ID	G group	AFA	API	EURO	MENA	HIS	NAM	UNK	Total	AFA	API	EURO	MENA	HIS	NAM	UNK	Total	Highest Frequency
C*02:104	02:104	HLA13568		0	0	2	0	0	0	0	2									
C*02:107	02:107	HLA13867		0	0	1	0	0	0	0	1									
C*02:109	02:109	HLA14335		0	0	1	0	0	0	0	1									
C*02:112	02:112	HLA14538		0	1	0	0	0	0	0	1									
C*02:114	02:114	HLA14753		0	1	0	0	0	0	0	1									
C*02:117	02:117	HLA15056		0	1	0	0	0	0	0	1									
C*02:CODE	02:CODE			2682	510	65389	623	3539	455	6319	79517	NA	NA	NA	NA	NA	NA	NA	NA	NA
**C*03:02 total**	**03:02 total**			**6523**	**62962**	**37989**	**5253**	**2552**	**411**	**8409**	**124099**	**C**	**C**	**C**	**C**	**C**	**C**	**C**	**C**	**C**
C*03:02	03:02			566	2206	2857	310	249	44	1307	7539	C	C	C	C	C	C	C	C	C
C*03:02P	03:02P			1	0	93	0	1	0	1	96			WD					WD	WD
**C*03:02:01G total**	**03:02:01G total**			**5956**	**60734**	**35038**	**4942**	**2302**	**367**	**7101**	**116440**	**C**	**C**	**C**	**C**	**C**	**C**	**C**	**C**	**C**
C*03:02:01G	03:02:01G		03:02:01G	4781	55853	33889	4802	1703	273	6217	107518	C	C	C	C	C	C	C	C	C
C*03:02:01	03:02:01	HLA00410	03:02:01G	0	0	0	0	0	0	1	1									
C*03:02:02	03:02:02		03:02:01G	298	1111	311	26	134	21	214	2115	C	C	I	WD	C	C	C	C	C
C*03:02:02:01	03:02:02:01	HLA01543	03:02:01G	876	3672	767	111	448	73	652	6599	C	C	I	C	C	C	C	C	C
C*03:02:02:02	03:02:02:02	HLA05731	03:02:01G	1	3	67	3	16	0	12	102			WD		I		WD	WD	I
C*03:02:02:03	03:02:02:03	HLA09569	03:02:01G	0	55	1	0	0	0	4	60		I						WD	I
C*03:02:03	03:02:03	HLA02800	03:02:01G	0	0	1	0	1	0	1	3									
C*03:02:07	03:02:07	HLA06126	03:02:01G	0	0	2	0	0	0	0	2									
C*03:146	03:146	HLA07814	03:02:01G	0	40	0	0	0	0	0	40		I						WD	I
C*03:02:05	03:02:05	HLA04287		0	0	1	0	0	0	0	1									
C*03:02:06	03:02:06	HLA04605		0	3	0	1	0	0	0	4									
C*03:02:09	03:02:09	HLA07730		0	17	0	0	0	0	0	17		I						WD	I
C*03:02:10	03:02:10	HLA09615		0	1	0	0	0	0	0	1									
C*03:02:13	03:02:13	HLA11874		0	1	0	0	0	0	0	1									
**C*03:03 total**	**03:03 total**			**4500**	**31913**	**561294**	**6889**	**20513**	**1798**	**48297**	**675204**	**C**	**C**	**C**	**C**	**C**	**C**	**C**	**C**	**C**
C*03:03	03:03			15	11	2259	1	14	2	75	2377	WD	WD	C		I		I	C	C
C*03:03P	03:03P			0	0	498	2	2	0	1	503			I					I	I
**C*03:03:01G total**	**03:03:01G total**			**4175**	**31744**	**558068**	**6876**	**20473**	**1783**	**48088**	**671207**	**C**	**C**	**C**	**C**	**C**	**C**	**C**	**C**	**C**
C*03:03:01G	03:03:01G		03:03:01G	3392	27856	532306	6721	15381	1365	44094	631115	C	C	C	C	C	C	C	C	C
C*03:03:01	03:03:01		03:03:01G	456	2286	13664	93	2879	234	2256	21868	C	C	C	C	C	C	C	C	C

Supplemental Table 10: HLA-C Allele Summary [a]				Allele Count by Population Group [b]								3.0.0 CIWD Category by Population Group [c]								
Allele	Genomic typing	Allele ID	G group	AFA	API	EURO	MENA	HIS	NAM	UNK	Total	AFA	API	EURO	MENA	HIS	NAM	UNK	Total	Highest Frequency
C*03:03:01:01	03:03:01:01	HLA00411	03:03:01G	326	1602	12095	62	2213	184	1737	18219	C	C	C	C	C	C	C	C	C
C*03:03:01:04	03:03:01:04	HLA16705	03:03:01G	0	0	1	0	0	0	0	1									
C*03:03:01:06	03:03:01:06	HLA16707	03:03:01G	0	0	0	0	0	0	1	1									
C*03:03:17	03:03:17	HLA07377	03:03:01G	1	0	1	0	0	0	0	2									
C*03:171	03:171	HLA08582	03:03:01G	0	0	1	0	0	0	0	1									
C*03:03:02	03:03:02	HLA00412		7	1	351	0	4	3	36	402	WD		I				I	I	I
C*03:03:03	03:03:03	HLA01355		0	5	2	0	0	0	0	7		WD						WD	WD
C*03:03:04	03:03:04	HLA01855		300	58	36	9	14	10	81	508	C	I	WD	WD	I	C	I	I	C
C*03:03:05	03:03:05	HLA02567		3	0	35	0	1	0	12	51			WD				WD	WD	WD
C*03:03:07	03:03:07	HLA04264		0	0	28	0	0	0	0	28			WD					WD	WD
C*03:03:10	03:03:10	HLA04281		0	10	1	1	0	0	0	12		WD						WD	WD
C*03:03:12	03:03:12	HLA05215		0	80	2	0	0	0	0	82		I						WD	I
C*03:03:15	03:03:15	HLA07024		0	1	0	0	0	0	0	1									
C*03:03:16	03:03:16	HLA07025		0	0	5	0	3	0	3	11			WD					WD	WD
C*03:03:19	03:03:19	HLA07954		0	2	0	0	0	0	0	2									
C*03:03:20	03:03:20	HLA09199		0	0	3	0	0	0	0	3									
C*03:03:21	03:03:21	HLA09726		0	0	1	0	0	0	0	1									
C*03:03:25	03:03:25	HLA11115		0	0	2	0	0	0	1	3									
C*03:03:27	03:03:27	HLA12510		0	0	1	0	0	0	0	1									
C*03:03:29	03:03:29	HLA13263		0	0	1	0	2	0	0	3									
C*03:03:30	03:03:30	HLA14605		0	0	1	0	0	0	0	1									
C*03:03:31	03:03:31	HLA14681		0	1	0	0	0	0	0	1									
**C*03:04 total**	**03:04 total**			**18307**	**45715**	**774735**	**8219**	**39516**	**4170**	**77579**	**968241**	**C**	**C**	**C**	**C**	**C**	**C**	**C**	**C**	**C**
C*03:04	03:04			697	108	6796	69	309	48	615	8642	C	I	C	C	C	C	C	C	C
C*03:04P	03:04P			0	0	251	1	4	1	0	257			I					I	I
**C*03:04:01G total**	**03:04:01G total**			**7432**	**44595**	**765180**	**7493**	**37151**	**3745**	**73998**	**939594**	**C**	**C**	**C**	**C**	**C**	**C**	**C**	**C**	**C**
C*03:04:01G	03:04:01G		03:04:01G	5590	37352	724225	7254	24941	2622	65827	867811	C	C	C	C	C	C	C	C	C
C*03:04:01	03:04:01		03:04:01G	1841	7236	40821	239	12210	1122	8163	71632	C	C	C	C	C	C	C	C	C
C*03:04:01:01	03:04:01:01	HLA00413	03:04:01G	0	0	116	0	0	0	4	120			WD					WD	WD
C*03:04:01:02	03:04:01:02	HLA04253	03:04:01G	0	0	16	0	0	0	1	17			WD					WD	WD
C*03:100	03:100	HLA05788	03:04:01G	0	7	0	0	0	0	3	10		WD						WD	WD

Supplemental Table 10: HLA-C Allele Summary [a]				Allele Count by Population Group [b]								3.0.0 CIWD Category by Population Group [c]								
Allele	Genomic typing	Allele ID	G group	AFA	API	EURO	MENA	HIS	NAM	UNK	Total	AFA	API	EURO	MENA	HIS	NAM	UNK	Total	Highest Frequency
C*03:101	03:101	HLA05870	03:04:01G	0	0	0	0	0	1	0	1									
C*03:252	03:252	HLA11762	03:04:01G	1	0	2	0	0	0	0	3									
**C*03:04:02G total**	**03:04:02G total**			**10141**	**136**	**2235**	**656**	**2037**	**374**	**2881**	**18460**	**C**	**C**	**C**	**C**	**C**	**C**	**C**	**C**	**C**
C*03:04:02G	03:04:02G		03:04:02G	110	4	83	9	16	1	43	266	C		WD	WD	I		I	I	C
C*03:04:02	03:04:02	HLA00414	03:04:02G	10031	132	2152	647	2021	373	2838	18194	C	C	C	C	C	C	C	C	C
C*03:04:04	03:04:04	HLA02356		1	855	15	0	2	0	55	928		C	WD				I	I	C
C*03:04:05	03:04:05	HLA02522		0	1	37	0	0	0	3	41			WD					WD	WD
C*03:04:06	03:04:06	HLA02819		26	0	2	0	0	1	4	33	WD							WD	WD
C*03:04:07	03:04:07	HLA02820		0	0	4	0	0	0	0	4									
C*03:04:08	03:04:08	HLA04260		0	0	25	0	0	0	1	26			WD					WD	WD
C*03:04:09	03:04:09	HLA04261		0	0	9	0	0	0	0	9			WD					WD	WD
C*03:04:10	03:04:10	HLA04263		0	8	0	0	0	0	0	8		WD						WD	WD
C*03:04:11	03:04:11	HLA04267		0	1	48	0	0	0	1	50			WD					WD	WD
C*03:04:13	03:04:13	HLA04278		0	0	47	0	0	0	2	49			WD					WD	WD
C*03:04:14	03:04:14	HLA04255		0	0	11	0	0	0	0	11			WD					WD	WD
C*03:04:15	03:04:15	HLA04589		0	0	14	0	0	0	0	14			WD					WD	WD
C*03:04:16	03:04:16	HLA04603		0	0	3	0	0	0	0	3									
C*03:04:17	03:04:17	HLA04622		0	0	13	0	0	0	1	14			WD					WD	WD
C*03:04:18	03:04:18	HLA04629		8	0	2	0	0	0	2	12	WD							WD	WD
C*03:04:19	03:04:19	HLA04736		0	0	5	0	0	0	1	6			WD					WD	WD
C*03:04:21	03:04:21	HLA06400		0	6	0	0	0	0	0	6		WD						WD	WD
C*03:04:22	03:04:22	HLA06445		0	0	4	0	1	0	0	5								WD	WD
C*03:04:23	03:04:23	HLA06728		0	0	1	0	0	0	0	1									
C*03:04:24	03:04:24	HLA07246		0	0	2	0	0	0	0	2									
C*03:04:25	03:04:25	HLA07348		0	0	2	0	0	0	0	2									
C*03:04:26	03:04:26	HLA07763		0	0	1	0	0	0	0	1									
C*03:04:27	03:04:27	HLA07952		0	0	19	0	9	0	13	41			WD		I		WD	WD	I
C*03:04:28	03:04:28	HLA07956		0	1	1	0	0	0	0	2									
C*03:04:29	03:04:29	HLA08166		0	0	1	0	0	0	0	1									
C*03:04:30	03:04:30	HLA08167		0	0	1	0	0	0	0	1									
C*03:04:33	03:04:33	HLA09039		2	0	0	0	0	0	0	2									

Supplemental Table 10: HLA-C Allele Summary [a]				Allele Count by Population Group [b]								3.0.0 CIWD Category by Population Group [c]								
Allele	Genomic typing	Allele ID	G group	AFA	API	EURO	MENA	HIS	NAM	UNK	Total	AFA	API	EURO	MENA	HIS	NAM	UNK	Total	Highest Frequency
C*03:04:34	03:04:34	HLA09040		0	0	1	0	0	0	0	1									
C*03:04:35	03:04:35	HLA09042		0	0	0	0	0	0	1	1									
C*03:04:37	03:04:37	HLA09972		0	3	0	0	0	0	0	3									
C*03:04:42	03:04:42	HLA10094		0	0	5	0	0	0	0	5			WD					WD	WD
C*03:04:45	03:04:45	HLA11113		0	0	0	0	3	1	0	4									
C*03:04:46	03:04:46	HLA11343		0	0	0	0	0	0	1	1									
C*03:04:47	03:04:47	HLA13264		0	1	0	0	0	0	0	1									
**C*03:05 total**	**03:05 total**			**242**	**78**	**520**	**22**	**7091**	**845**	**1263**	**10061**	**C**	**I**	**I**	**WD**	**C**	**C**	**C**	**C**	**C**
**C*03:05:01G total**	**03:05:01G total**			**242**	**78**	**520**	**22**	**7091**	**845**	**1263**	**10061**	**C**	**I**	**I**	**WD**	**C**	**C**	**C**	**C**	**C**
C*03:05	03:05	HLA00415	03:05:01G	240	75	497	22	6934	840	1213	9821	C	I	I	WD	C	C	C	C	C
C*03:05:01G	03:05:01G		03:05:01G	2	3	23	0	157	5	50	240			WD		C	WD	I	I	C
**C*03:06 total**	**03:06 total**			**45**	**56**	**361**	**11**	**5096**	**198**	**739**	**6506**	**C**	**I**	**I**	**WD**	**C**	**C**	**C**	**C**	**C**
C*03:06	03:06			21	27	252	7	2505	121	432	3365	WD	I	I	WD	C	C	C	C	C
C*03:06P	03:06P			0	0	1	0	0	0	0	1									
C*03:06:01	03:06:01	HLA00416		24	29	108	4	2590	77	307	3139	WD	I	WD		C	C	C	C	C
C*03:06:02	03:06:02	HLA12785		0	0	0	0	1	0	0	1									
**C*03:07 total**	**03:07 total**			**6**	**2**	**195**	**1**	**158**	**5**	**61**	**428**	**WD**		**I**		**C**	**WD**	**I**	**I**	**C**
C*03:07	03:07			6	1	189	1	156	5	60	418	WD		I		C	WD	I	I	C
C*03:07:01	03:07:01	HLA00417		0	1	6	0	2	0	1	10			WD					WD	WD
C*03:08	03:08	HLA00418		2	0	75	5	129	9	40	260			WD	WD	C	C	I	I	C
C*03:09	03:09	HLA00419		8	0	186	0	2	1	26	223	WD		I				I	I	I
C*03:10	03:10	HLA01076		5	1	238	0	3	1	33	281	WD		I				I	I	I
**C*03:11 total**	**03:11 total**			**2**	**1**	**62**	**0**	**3**	**0**	**7**	**75**			**WD**				**WD**	**WD**	**WD**
C*03:11	03:11			0	1	9	0	1	0	1	12			WD					WD	WD
C*03:11:02	03:11:02	HLA02375		2	0	53	0	2	0	6	63			WD				WD	WD	WD
**C*03:13 total**	**03:13 total**			**2**	**2**	**20**	**1**	**0**	**0**	**2**	**27**			**WD**					**WD**	**WD**
C*03:13	03:13			1	0	2	0	0	0	0	3									
C*03:13:01	03:13:01	HLA01412		1	1	18	1	0	0	2	23			WD					WD	WD
C*03:13:02	03:13:02	HLA09991		0	1	0	0	0	0	0	1									
**C*03:14 total**	**03:14 total**			**4**	**1**	**486**	**0**	**14**	**0**	**52**	**557**			**I**		**I**		**I**	**I**	**I**
**C*03:14:01G total**	**03:14:01G total**			**4**	**1**	**486**	**0**	**14**	**0**	**52**	**557**			**I**		**I**		**I**	**I**	**I**

Supplemental Table 10: HLA-C Allele Summary [a]				Allele Count by Population Group [b]								3.0.0 CIWD Category by Population Group [c]								
**Allele**	**Genomic typing**	**Allele ID**	**G group**	**AFA**	**API**	**EURO**	**MENA**	**HIS**	**NAM**	**UNK**	**Total**	**AFA**	**API**	**EURO**	**MENA**	**HIS**	**NAM**	**UNK**	**Total**	**Highest Frequency**
C*03:14	03:14	HLA01425	03:14:01G	4	1	486	0	14	0	52	557			I		I		I	I	I
C*03:15	03:15	HLA01581		0	3	0	0	0	0	0	3									
C*03:16	03:16	HLA01660		3	3	316	19	17	0	22	380			I	WD	I		I	I	I
**C*03:17 total**	**03:17 total**			**1**	**135**	**5**	**0**	**2**	**0**	**14**	**157**		**C**	**WD**				**I**	**WD**	**C**
C*03:17	03:17			1	131	5	0	2	0	14	153		C	WD				I	WD	C
C*03:17:01	03:17:01	HLA01906		0	4	0	0	0	0	0	4									
**C*03:18 total**	**03:18 total**			**0**	**0**	**32**	**0**	**0**	**1**	**13**	**46**			**WD**				**WD**	**WD**	**WD**
C*03:18	03:18			0	0	13	0	0	1	2	16			WD					WD	WD
C*03:18:01	03:18:01	HLA01925		0	0	19	0	0	0	8	27			WD				WD	WD	WD
C*03:18:02	03:18:02	HLA11117		0	0	0	0	0	0	3	3									
C*03:19	03:19	HLA02133		4	0	378	97	7	4	59	549			I	C	I		I	I	C
C*03:21	03:21	HLA02391		0	13	0	0	0	0	0	13		I						WD	I
C*03:22Q	03:22Q	HLA02414		0	0	2	0	0	0	0	2									
C*03:23	03:23	HLA02444		0	3	0	0	0	0	1	4									
C*03:24	03:24	HLA02467		0	3	0	0	0	0	0	3									
C*03:26	03:26	HLA02526		0	1	26	0	0	0	2	29			WD					WD	WD
C*03:27	03:27	HLA02594		0	0	0	0	1	0	1	2									
C*03:28	03:28	HLA02599		0	1	18	0	0	0	1	20			WD					WD	WD
C*03:30	03:30	HLA02617		0	0	8	0	0	0	0	8			WD					WD	WD
C*03:31	03:31	HLA02648		0	0	2	0	0	0	0	2									
C*03:32	03:32	HLA02681		0	6	19	0	1	0	0	26		WD	WD					WD	WD
C*03:33	03:33	HLA02715		0	0	0	0	0	0	1	1									
C*03:34	03:34	HLA02729		0	1	2	0	0	0	1	4									
**C*03:35 total**	**03:35 total**			**0**	**0**	**41**	**3**	**2**	**0**	**4**	**50**			**WD**					**WD**	**WD**
C*03:35	03:35			0	0	34	3	1	0	3	41			WD					WD	WD
C*03:35:01	03:35:01	HLA02758		0	0	4	0	1	0	1	6								WD	WD
C*03:35:02	03:35:02	HLA15901		0	0	3	0	0	0	0	3									
C*03:36	03:36	HLA02817		0	62	0	0	0	0	16	78		I					I	WD	I
**C*03:37 total**	**03:37 total**			**0**	**7**	**4**	**0**	**0**	**0**	**0**	**11**		**WD**						**WD**	**WD**
C*03:37	03:37			0	1	3	0	0	0	0	4									
C*03:37:01	03:37:01	HLA02818		0	6	0	0	0	0	0	6		WD						WD	WD

Supplemental Table 10: HLA-C Allele Summary [a]				Allele Count by Population Group [b]								3.0.0 CIWD Category by Population Group [c]								
Allele	Genomic typing	Allele ID	G group	AFA	API	EURO	MENA	HIS	NAM	UNK	Total	AFA	API	EURO	MENA	HIS	NAM	UNK	Total	Highest Frequency
C*03:37:02	03:37:02	HLA08536		0	0	1	0	0	0	0	1									
**C*03:38 total**	**03:38 total**			**47**	**3**	**9**	**0**	**20**	**7**	**17**	**103**	**C**		**WD**		**I**	**C**	**I**	**WD**	**C**
C*03:38	03:38			4	0	1	0	4	0	2	11								WD	WD
C*03:38:01	03:38:01	HLA02821		43	0	4	0	16	6	14	83	C				I	WD	I	WD	C
C*03:38:02	03:38:02	HLA03048		0	3	4	0	0	1	1	9								WD	WD
C*03:39	03:39	HLA02843		0	2	0	0	0	0	0	2									
**C*03:40 total**	**03:40 total**			**2**	**1**	**79**	**0**	**7**	**0**	**14**	**103**			**WD**		**I**		**I**	**WD**	**I**
C*03:40	03:40			1	0	16	0	0	0	0	17			WD					WD	WD
**C*03:40:01G total**	**03:40:01G total**			**0**	**1**	**62**	**0**	**7**	**0**	**14**	**84**			**WD**		**I**		**I**	**WD**	**I**
C*03:40:01G	03:40:01G		03:40:01G	0	0	26	0	5	0	7	38			WD		WD		WD	WD	WD
C*03:40:01	03:40:01	HLA02881	03:40:01G	0	1	26	0	0	0	4	31			WD					WD	WD
C*03:279	03:279	HLA13188	03:40:01G	0	0	10	0	2	0	3	15			WD					WD	WD
C*03:40:02	03:40:02	HLA07015		0	0	1	0	0	0	0	1									
C*03:40:04	03:40:04	HLA12860		1	0	0	0	0	0	0	1									
**C*03:41 total**	**03:41 total**			**0**	**6**	**11**	**0**	**2**	**0**	**0**	**19**		**WD**	**WD**					**WD**	**WD**
C*03:41	03:41			0	2	6	0	1	0	0	9			WD					WD	WD
C*03:41:01	03:41:01	HLA02927		0	4	3	0	0	0	0	7								WD	WD
C*03:41:02	03:41:02	HLA11976		0	0	2	0	1	0	0	3									
C*03:42	03:42	HLA02993		0	0	52	0	0	0	1	53			WD					WD	WD
**C*03:43 total**	**03:43 total**			**0**	**10**	**26**	**1**	**0**	**0**	**3**	**40**		**WD**	**WD**					**WD**	**WD**
C*03:43	03:43			0	0	2	0	0	0	0	2									
C*03:43:01	03:43:01	HLA02994		0	10	23	1	0	0	2	36		WD	WD					WD	WD
C*03:43:02	03:43:02	HLA03544		0	0	1	0	0	0	1	2									
C*03:44	03:44	HLA02998		0	0	395	1	0	0	11	407			I				WD	I	I
C*03:45	03:45	HLA03027		0	0	16	0	9	0	28	53			WD		I		I	WD	I
C*03:46	03:46	HLA02987		0	4	9	0	0	0	1	14			WD					WD	WD
C*03:47	03:47	HLA03163		1	0	8	0	53	1	22	85			WD		I		I	WD	I
C*03:48	03:48	HLA03242		0	1	0	0	0	0	0	1									
C*03:49	03:49	HLA03362		0	9	4	1	0	0	3	17		WD						WD	WD
C*03:51	03:51	HLA03723		0	0	56	0	0	0	1	57			WD					WD	WD
C*03:52	03:52	HLA03724		0	0	1	0	0	0	0	1									

Supplemental Table 10: HLA-C Allele Summary [a]				Allele Count by Population Group [b]								3.0.0 CIWD Category by Population Group [c]								
Allele	Genomic typing	Allele ID	G group	AFA	API	EURO	MENA	HIS	NAM	UNK	Total	AFA	API	EURO	MENA	HIS	NAM	UNK	Total	Highest Frequency
C*03:53	03:53	HLA03725		0	0	31	0	0	0	0	31			WD					WD	WD
C*03:54	03:54	HLA03729		0	0	16	0	0	0	0	16			WD					WD	WD
C*03:55	03:55	HLA03733		0	0	2	0	0	0	0	2									
C*03:56	03:56	HLA03737		0	8	0	0	0	0	0	8		WD						WD	WD
**C*03:57 total**	**03:57 total**			**0**	**0**	**1**	**0**	**0**	**0**	**0**	**1**									
C*03:57	03:57			0	0	1	0	0	0	0	1									
C*03:58	03:58	HLA03713		0	4	0	0	0	0	0	4									
C*03:59	03:59	HLA03827		0	0	5	0	0	0	0	5			WD					WD	WD
C*03:60	03:60	HLA03828		0	4	0	0	0	0	1	5								WD	WD
C*03:63	03:63	HLA04256		0	0	1	0	0	0	0	1									
**C*03:64 total**	**03:64 total**			**0**	**0**	**10**	**1**	**1**	**0**	**1**	**13**			**WD**					**WD**	**WD**
C*03:64:01	03:64:01	HLA04257		0	0	9	1	0	0	1	11			WD					WD	WD
C*03:64:02	03:64:02	HLA08206		0	0	1	0	1	0	0	2									
C*03:65	03:65	HLA04259		0	0	2	0	0	0	0	2									
C*03:66	03:66	HLA04262		0	0	1	0	0	0	0	1									
C*03:69	03:69	HLA04269		0	0	11	0	0	0	0	11			WD					WD	WD
C*03:70	03:70	HLA04271		0	0	31	0	0	0	0	31			WD					WD	WD
C*03:71	03:71	HLA04273		0	0	1	2	0	0	0	3									
C*03:73	03:73	HLA04279		0	0	2	0	0	0	0	2									
C*03:74	03:74	HLA04280		0	0	1	0	0	0	0	1									
C*03:75	03:75	HLA04282		3	0	18	0	3	0	1	25			WD					WD	WD
C*03:76	03:76	HLA04283		0	1	0	0	0	0	0	1									
C*03:77	03:77	HLA04595		0	1	0	0	2	0	0	3									
C*03:78	03:78	HLA04618		1	1	0	0	8	1	2	13					I			WD	I
C*03:79	03:79	HLA04633		0	0	1	0	0	0	0	1									
**C*03:80 total**	**03:80 total**			**0**	**0**	**1**	**0**	**0**	**0**	**0**	**1**									
C*03:80:02	03:80:02	HLA15897		0	0	1	0	0	0	0	1									
C*03:81	03:81	HLA04758		0	0	4	0	2	0	0	6								WD	WD
C*03:82	03:82	HLA04777		0	0	4	0	0	0	0	4									
C*03:83	03:83	HLA04794		0	0	1	0	0	0	0	1									
C*03:85	03:85	HLA05111		0	22	1	0	0	0	0	23		I						WD	I

Supplemental Table 10: HLA-C Allele Summary [a]				Allele Count by Population Group [b]								3.0.0 CIWD Category by Population Group [c]								
**Allele**	**Genomic typing**	**Allele ID**	**G group**	**AFA**	**API**	**EURO**	**MENA**	**HIS**	**NAM**	**UNK**	**Total**	**AFA**	**API**	**EURO**	**MENA**	**HIS**	**NAM**	**UNK**	**Total**	**Highest Frequency**
C*03:86	03:86	HLA05122		0	3	1	0	0	0	2	6								WD	WD
**C*03:87 total**	**03:87 total**			**0**	**0**	**23**	**0**	**7**	**0**	**2**	**32**			**WD**		**I**			**WD**	**I**
C*03:87	03:87			0	0	10	0	0	0	0	10			WD					WD	WD
C*03:87:01	03:87:01	HLA05124		0	0	12	0	0	0	2	14			WD					WD	WD
C*03:87:02	03:87:02	HLA12784		0	0	1	0	7	0	0	8					I			WD	I
C*03:88	03:88	HLA05162		1	157	0	9	0	2	2	171		C		WD				I	C
C*03:90	03:90	HLA05171		0	2	0	0	0	0	0	2									
**C*03:91 total**	**03:91 total**			**0**	**0**	**11**	**0**	**0**	**0**	**0**	**11**			**WD**					**WD**	**WD**
C*03:91	03:91			0	0	2	0	0	0	0	2									
C*03:91:01	03:91:01	HLA05197		0	0	8	0	0	0	0	8			WD					WD	WD
C*03:91:02	03:91:02	HLA11116		0	0	1	0	0	0	0	1									
C*03:92	03:92	HLA05202		0	0	1	0	0	0	0	1									
C*03:93	03:93	HLA05371		0	0	1	0	0	0	1	2									
C*03:94	03:94	HLA05396		0	21	0	1	0	0	0	22		I						WD	I
C*03:95	03:95	HLA05605		0	0	4	0	0	0	0	4									
C*03:98	03:98	HLA05747		0	1	0	0	0	0	0	1									
C*03:103	03:103	HLA06067		0	0	2	0	3	0	0	5								WD	WD
C*03:104	03:104	HLA06074		0	0	2	0	0	0	0	2									
C*03:108	03:108	HLA06310		0	0	0	0	1	0	0	1									
C*03:109	03:109	HLA06398		0	0	22	0	1	0	0	23			WD					WD	WD
C*03:112	03:112	HLA06419		0	0	1	0	0	0	0	1									
**C*03:113 total**	**03:113 total**			**0**	**0**	**6**	**0**	**0**	**0**	**0**	**6**			**WD**					**WD**	**WD**
C*03:113	03:113			0	0	3	0	0	0	0	3									
C*03:113:01	03:113:01	HLA06711		0	0	3	0	0	0	0	3									
C*03:114	03:114	HLA06712		0	0	0	0	3	0	0	3									
C*03:115	03:115	HLA06713		0	0	23	0	0	0	0	23			WD					WD	WD
**C*03:116 total**	**03:116 total**			**0**	**4**	**1**	**0**	**0**	**0**	**0**	**5**								**WD**	**WD**
C*03:116:01	03:116:01	HLA06726		0	4	1	0	0	0	0	5								WD	WD
C*03:117	03:117	HLA06727		0	0	10	0	2	0	1	13			WD					WD	WD
C*03:120	03:120	HLA07007		0	0	20	0	0	0	0	20			WD					WD	WD
C*03:121N	03:121N	HLA07008		4	0	0	0	0	0	0	4									

Supplemental Table 10: HLA-C Allele Summary [a]				Allele Count by Population Group [b]								3.0.0 CIWD Category by Population Group [c]								
Allele	Genomic typing	Allele ID	G group	AFA	API	EURO	MENA	HIS	NAM	UNK	Total	AFA	API	EURO	MENA	HIS	NAM	UNK	Total	Highest Frequency
C*03:122	03:122	HLA07013		0	0	14	0	0	0	0	14			WD					WD	WD
C*03:123	03:123	HLA07014		0	0	0	0	1	1	0	2									
C*03:126	03:126	HLA07027		1	0	20	0	0	0	0	21			WD					WD	WD
C*03:127	03:127	HLA07029		0	0	7	0	0	0	0	7			WD					WD	WD
C*03:129	03:129	HLA07118		0	0	1	0	2	0	0	3									
C*03:130	03:130	HLA07119		0	0	4	0	0	0	0	4									
C*03:132	03:132	HLA07140		0	1	0	1	0	0	1	3									
C*03:135	03:135	HLA07239		0	0	1	0	0	0	0	1									
C*03:136	03:136	HLA07240		0	0	10	0	0	0	0	10			WD					WD	WD
C*03:137	03:137	HLA07241		0	0	3	0	0	0	0	3									
C*03:139	03:139	HLA07355		0	4	4	1	0	0	0	9								WD	WD
C*03:140	03:140	HLA07495		1	6	3	0	2	0	5	17		WD					WD	WD	WD
C*03:141	03:141	HLA07496		0	0	2	0	0	0	0	2									
C*03:143	03:143	HLA07498		0	0	5	0	8	0	16	29			WD		I		I	WD	I
C*03:145	03:145	HLA07537		0	0	2	0	0	0	0	2									
C*03:147	03:147	HLA07940		0	1	6	0	0	0	1	8			WD					WD	WD
C*03:148	03:148	HLA07945		0	0	10	0	0	0	0	10			WD					WD	WD
C*03:149	03:149	HLA07946		0	0	2	0	0	0	0	2									
C*03:150	03:150	HLA07947		0	0	2	0	0	0	0	2									
C*03:151	03:151	HLA07948		0	0	15	1	0	0	0	16			WD					WD	WD
C*03:152	03:152	HLA07949		0	0	1	0	0	0	0	1									
C*03:153	03:153	HLA07951		0	14	0	0	0	1	0	15		I						WD	I
C*03:154	03:154	HLA07953		0	0	5	0	0	0	0	5			WD					WD	WD
C*03:155	03:155	HLA07955		0	0	0	2	0	0	0	2									
C*03:156	03:156	HLA07957		0	0	1	0	0	0	0	1									
C*03:158	03:158	HLA08175		0	3	0	0	0	0	0	3									
C*03:159	03:159	HLA08176		0	0	3	0	0	0	3	6								WD	WD
C*03:160	03:160	HLA08214		0	0	3	0	0	0	0	3									
C*03:161	03:161	HLA08215		0	0	2	0	0	0	0	2									
C*03:163	03:163	HLA08217		0	3	0	0	0	0	1	4									
C*03:165	03:165	HLA08477		0	0	3	0	0	0	0	3									

Supplemental Table 10: HLA-C Allele Summary [a]				Allele Count by Population Group [b]								3.0.0 CIWD Category by Population Group [c]								
Allele	Genomic typing	Allele ID	G group	AFA	API	EURO	MENA	HIS	NAM	UNK	Total	AFA	API	EURO	MENA	HIS	NAM	UNK	Total	Highest Frequency
C*03:166	03:166	HLA08530		0	0	1	0	0	0	0	1									
C*03:170	03:170	HLA08545		0	0	1	0	0	0	0	1									
C*03:172	03:172	HLA08764		0	0	0	0	1	0	0	1									
C*03:173	03:173	HLA08766		0	0	3	0	0	0	0	3									
C*03:174	03:174	HLA08767		0	0	1	0	0	0	3	4									
C*03:175	03:175	HLA08768		0	0	0	0	0	0	3	3									
C*03:176	03:176	HLA08812		0	0	0	0	0	0	1	1									
C*03:177	03:177	HLA08813		0	0	1	0	0	0	0	1									
C*03:178	03:178	HLA08814		0	0	0	0	1	1	0	2									
C*03:180	03:180	HLA08816		0	0	1	0	0	0	0	1									
**C*03:184 total**	**03:184 total**			**0**	**0**	**2**	**0**	**0**	**0**	**0**	**2**									
C*03:184	03:184			0	0	2	0	0	0	0	2									
**C*03:186 total**	**03:186 total**			**0**	**0**	**0**	**0**	**1**	**0**	**1**	**2**									
C*03:186	03:186			0	0	0	0	1	0	1	2									
C*03:187	03:187	HLA09198		0	1	0	0	0	0	0	1									
C*03:188	03:188	HLA09200		0	0	1	0	0	0	0	1									
C*03:189N	03:189N	HLA09201		0	0	1	0	0	0	0	1									
C*03:191	03:191	HLA09597		0	0	0	0	0	1	0	1									
C*03:193	03:193	HLA09625		0	1	0	0	0	0	0	1									
C*03:197	03:197	HLA09943		0	1	0	0	0	0	0	1									
C*03:198	03:198	HLA09945		2	1	0	0	0	0	0	3									
C*03:210	03:210	HLA09979		0	0	1	0	0	0	0	1									
C*03:214	03:214	HLA10101		0	0	2	0	0	0	0	2									
C*03:215	03:215	HLA10102		0	0	0	0	1	0	0	1									
C*03:216	03:216	HLA10104		0	1	0	0	0	0	0	1									
C*03:217	03:217	HLA10106		0	0	3	0	0	0	0	3									
C*03:220	03:220	HLA10258		0	0	1	0	0	0	3	4									
C*03:222	03:222	HLA10442		0	1	0	0	0	0	0	1									
C*03:233	03:233	HLA10590		0	1	1	0	0	0	0	2									
C*03:243	03:243	HLA11264		0	0	2	0	0	0	0	2									
C*03:244Q	03:244Q	HLA11265		0	0	1	0	0	0	0	1									

Supplemental Table 10: HLA-C Allele Summary [a]				Allele Count by Population Group [b]								3.0.0 CIWD Category by Population Group [c]								
Allele	Genomic typing	Allele ID	G group	AFA	API	EURO	MENA	HIS	NAM	UNK	Total	AFA	API	EURO	MENA	HIS	NAM	UNK	Total	Highest Frequency
C*03:247	03:247	HLA11572		0	0	1	0	0	0	0	1									
C*03:248	03:248	HLA11578		0	1	0	0	0	0	0	1									
C*03:250	03:250	HLA11668		0	0	4	0	0	0	0	4									
C*03:251	03:251	HLA11711		0	0	4	1	0	0	0	5								WD	WD
C*03:255	03:255	HLA11872		0	0	0	0	1	0	0	1									
C*03:257	03:257	HLA11875		0	0	0	0	1	0	0	1									
C*03:258	03:258	HLA12023		0	5	0	0	0	0	0	5		WD						WD	WD
C*03:259	03:259	HLA11975		0	0	2	0	0	0	0	2									
**C*03:263 total**	**03:263 total**			**0**	**0**	**0**	**0**	**1**	**0**	**0**	**1**									
C*03:263	03:263			0	0	0	0	1	0	0	1									
C*03:264	03:264	HLA12331		1	0	0	0	0	0	0	1									
C*03:269	03:269	HLA12511		0	0	0	1	0	0	0	1									
C*03:271	03:271	HLA12730		0	0	0	0	0	0	1	1									
C*03:272	03:272	HLA12782		0	1	2	0	0	0	0	3									
C*03:274	03:274	HLA12786		0	1	0	0	0	0	0	1									
C*03:275	03:275	HLA12787		0	0	1	0	0	0	0	1									
C*03:278	03:278	HLA13028		0	0	1	0	0	0	0	1									
C*03:282	03:282	HLA13260		0	0	0	0	2	0	0	2									
C*03:283	03:283	HLA13261		0	0	0	0	1	0	0	1									
C*03:286	03:286	HLA13420		0	0	1	0	0	0	0	1									
C*03:291	03:291	HLA13571		0	0	1	0	0	0	0	1									
C*03:296	03:296	HLA13670		1	0	0	0	0	0	0	1									
C*03:297	03:297	HLA13671		0	0	1	0	0	0	0	1									
C*03:298	03:298	HLA13802		0	3	0	0	0	0	0	3									
C*03:301	03:301	HLA14030		0	2	0	0	0	0	0	2									
C*03:305	03:305	HLA14261		0	1	0	0	0	0	0	1									
C*03:307	03:307	HLA14338		0	0	2	0	0	0	0	2									
C*03:308	03:308	HLA14537		0	0	1	0	0	0	1	2									
C*03:309	03:309	HLA14540		0	0	1	0	0	0	0	1									
C*03:316N	03:316N	HLA14756		0	0	0	0	0	0	3	3									
C*03:317	03:317	HLA14860		0	0	4	0	0	0	0	4									

Supplemental Table 10: HLA-C Allele Summary [a]				Allele Count by Population Group [b]								3.0.0 CIWD Category by Population Group [c]								
Allele	Genomic typing	Allele ID	G group	AFA	API	EURO	MENA	HIS	NAM	UNK	Total	AFA	API	EURO	MENA	HIS	NAM	UNK	Total	Highest Frequency
C*03:339	03:339	HLA15917		0	0	1	0	0	0	0	1									
C*03:CODE	03:CODE			3213	8759	138196	1249	9269	1163	15038	176887	NA	NA	NA	NA	NA	NA	NA	NA	NA
**C*04:01 total**	**04:01 total**			**67016**	**133186**	**1231317**	**66166**	**99890**	**8733**	**155605**	**1761913**	**C**	**C**	**C**	**C**	**C**	**C**	**C**	**C**	**C**
C*04:01	04:01			19	15	8266	25	58	0	509	8892	WD	I	C	WD	I		C	C	C
C*04:01P	04:01P			3	9	2875	11	7	0	20	2925		WD	C	WD	I		I	C	C
**C*04:01:01G total**	**04:01:01G total**			**67005**	**133148**	**1220374**	**66087**	**99872**	**8744**	**155060**	**1750290**	**C**	**C**	**C**	**C**	**C**	**C**	**C**	**C**	**C**
C*04:01:01G	04:01:01G		04:01:01G	54893	123519	1167870	64371	75008	6469	144159	1636289	C	C	C	C	C	C	C	C	C
C*04:01:01	04:01:01		04:01:01G	3356	2511	16169	551	7071	552	2959	33169	C	C	C	C	C	C	C	C	C
C*04:01:01:01	04:01:01:01	HLA00420	04:01:01G	8397	5823	22600	616	14270	1421	5984	59111	C	C	C	C	C	C	C	C	C
C*04:01:01:02	04:01:01:02	HLA01328	04:01:01G	1	1	1	0	0	0	0	3									
C*04:01:01:05	04:01:01:05	HLA08080	04:01:01G	116	123	4209	18	740	59	581	5846	C	I	C	WD	C	C	C	C	C
C*04:01:01:06	04:01:01:06	HLA13527	04:01:01G	207	560	8763	528	2632	229	1071	13990	C	C	C	C	C	C	C	C	C
C*04:01:01:08	04:01:01:08	HLA16711	04:01:01G	1	0	4	0	0	0	0	5								WD	WD
C*04:01:01:10	04:01:01:10	HLA16713	04:01:01G	0	0	0	0	1	0	0	1									
C*04:01:01:12	04:01:01:12	HLA17055	04:01:01G	4	0	2	1	2	0	4	13								WD	WD
C*04:01:78	04:01:78	HLA15574	04:01:01G	0	0	0	0	2	0	0	2									
C*04:01:79	04:01:79	HLA15631	04:01:01G	0	0	38	0	1	1	9	49			WD				WD	WD	WD
C*04:09N	04:09N	HLA01451	04:01:01G	26	1	689	1	133	13	125	988	WD		I		C	C	I	I	C
C*04:30	04:30	HLA03122	04:01:01G	0	0	13	1	1	0	0	15			WD					WD	WD
C*04:41	04:41	HLA03832	04:01:01G	0	0	2	0	0	0	0	2									
C*04:82	04:82	HLA05916	04:01:01G	1	608	9	0	8	0	165	791		C	WD		I		C	I	C
C*04:144	04:144	HLA09076	04:01:01G	3	1	0	0	1	0	1	6								WD	WD
C*04:146	04:146	HLA09502	04:01:01G	0	0	0	0	2	0	2	4									
C*04:161	04:161	HLA10255	04:01:01G	0	0	1	0	0	0	0	1									
C*04:195	04:195	HLA12912	04:01:01G	0	1	0	0	0	0	0	1									
C*04:226	04:226	HLA14220	04:01:01G	0	0	2	0	0	0	0	2									
C*04:267	04:267	HLA16729	04:01:01G	0	0	2	0	0	0	0	2									
C*04:01:02	04:01:02	HLA00421		1	0	0	0	0	0	0	1									
C*04:01:03	04:01:03	HLA02086		1	0	5	1	62	0	9	78			WD		I		WD	WD	I
C*04:01:04	04:01:04	HLA02434		0	0	0	4	1	0	0	5								WD	WD
C*04:01:07	04:01:07	HLA03734		0	0	3	0	0	0	0	3									

Supplemental Table 10: HLA-C Allele Summary [a]				Allele Count by Population Group [b]								3.0.0 CIWD Category by Population Group [c]								
Allele	Genomic typing	Allele ID	G group	AFA	API	EURO	MENA	HIS	NAM	UNK	Total	AFA	API	EURO	MENA	HIS	NAM	UNK	Total	Highest Frequency
C*04:01:08	04:01:08	HLA03736		2	2	90	1	1	0	3	99			WD					WD	WD
C*04:01:09	04:01:09	HLA03802		2	1	18	7	0	0	4	32			WD	WD				WD	WD
C*04:01:10	04:01:10	HLA03805		0	0	284	1	1	0	6	292			I				WD	I	I
C*04:01:11	04:01:11	HLA04254		1	1	1	0	0	0	0	3									
C*04:01:12	04:01:12	HLA04286		2	0	0	0	0	0	2	4									
C*04:01:13	04:01:13	HLA04590		0	0	31	0	0	0	0	31			WD					WD	WD
C*04:01:14	04:01:14	HLA04626		0	0	4	0	0	0	3	7								WD	WD
C*04:01:15	04:01:15	HLA04574		0	0	7	0	0	0	0	7			WD					WD	WD
C*04:01:16	04:01:16	HLA04759		0	0	14	0	0	0	0	14			WD					WD	WD
C*04:01:20	04:01:20	HLA05219		0	0	1	0	1	0	0	2									
C*04:01:21	04:01:21	HLA05403		0	0	0	0	1	0	0	1									
C*04:01:22	04:01:22	HLA05560		0	0	2	0	0	0	0	2									
C*04:01:23	04:01:23	HLA05569		0	1	4	2	0	0	1	8								WD	WD
C*04:01:24	04:01:24	HLA05586		0	0	1	0	0	0	0	1									
C*04:01:25	04:01:25	HLA05599		0	0	2	0	0	0	0	2									
C*04:01:26	04:01:26	HLA05610		0	0	1	0	0	0	0	1									
C*04:01:27	04:01:27	HLA06402		0	1	0	0	0	0	0	1									
C*04:01:28	04:01:28	HLA06404		0	0	2	16	0	0	103	121				WD			I	WD	I
C*04:01:29	04:01:29	HLA06446		0	0	1	6	0	0	2	9				WD				WD	WD
C*04:01:30	04:01:30	HLA06532		0	0	3	1	0	0	1	5								WD	WD
C*04:01:33	04:01:33	HLA07022		0	0	0	0	14	0	3	17					I			WD	I
C*04:01:34	04:01:34	HLA07126		0	0	2	0	0	0	0	2									
C*04:01:37	04:01:37	HLA07502		0	0	3	0	0	0	0	3									
C*04:01:39	04:01:39	HLA07973		0	3	0	0	0	0	0	3									
C*04:01:41	04:01:41	HLA08226		0	1	0	4	0	0	0	5								WD	WD
C*04:01:43	04:01:43	HLA08826		0	0	1	0	1	0	0	2									
C*04:01:44	04:01:44	HLA08827		0	0	0	0	1	1	2	4									
C*04:01:46	04:01:46	HLA09044		1	0	0	0	0	0	0	1									
C*04:01:47	04:01:47	HLA09048		0	2	0	0	0	0	0	2									
C*04:01:48	04:01:48	HLA09050		3	0	0	0	0	0	0	3									
C*04:01:49	04:01:49	HLA09205		1	0	0	0	0	0	0	1									

Supplemental Table 10: HLA-C Allele Summary [a]				Allele Count by Population Group [b]								3.0.0 CIWD Category by Population Group [c]								
Allele	Genomic typing	Allele ID	G group	AFA	API	EURO	MENA	HIS	NAM	UNK	Total	AFA	API	EURO	MENA	HIS	NAM	UNK	Total	Highest Frequency
C*04:01:50	04:01:50	HLA09206		0	0	0	0	0	1	0	1									
C*04:01:52	04:01:52	HLA09631		0	0	3	0	0	0	0	3									
C*04:01:55	04:01:55	HLA10536		0	0	0	1	0	0	0	1									
C*04:01:56	04:01:56	HLA10598		0	0	2	0	0	0	0	2									
C*04:01:60	04:01:60	HLA11584		0	0	0	0	1	0	1	2									
C*04:01:61	04:01:61	HLA11585		1	0	0	0	0	0	0	1									
C*04:01:63	04:01:63	HLA12201		0	0	1	0	1	0	0	2									
C*04:01:64	04:01:64	HLA12348		0	0	0	0	1	0	0	1									
C*04:01:65	04:01:65	HLA12517		0	0	2	0	0	0	0	2									
C*04:01:66	04:01:66	HLA12717		0	0	1	0	0	0	0	1									
C*04:01:67	04:01:67	HLA13267		0	0	0	0	0	0	1	1									
C*04:01:75	04:01:75	HLA15189		0	1	0	0	0	0	0	1									
C*04:01:80	04:01:80	HLA15913		0	2	0	0	0	0	0	2									
C*04:01:81	04:01:81	HLA15803		0	0	2	0	0	0	0	2									
**C*04:03 total**	**04:03 total**			**75**	**24089**	**2012**	**355**	**196**	**50**	**2096**	**28873**	**C**	**C**	**C**	**C**	**C**	**C**	**C**	**C**	**C**
C*04:03	04:03			21	2844	386	41	43	13	395	3743	WD	C	I	C	I	C	C	C	C
C*04:03P	04:03P			0	0	1	0	0	0	0	1									
**C*04:03:01G total**	**04:03:01G total**			**54**	**21242**	**1625**	**314**	**153**	**37**	**1701**	**25126**	**C**	**C**	**C**	**C**	**C**	**C**	**C**	**C**	**C**
C*04:03:01G	04:03:01G		04:03:01G	0	516	0	0	0	0	0	516		C						I	C
C*04:03:01	04:03:01	HLA00423	04:03:01G	54	20726	1625	314	153	37	1701	24610	C	C	C	C	C	C	C	C	C
C*04:03:02	04:03:02	HLA09716		0	3	0	0	0	0	0	3									
**C*04:04 total**	**04:04 total**			**180**	**10**	**580**	**108**	**309**	**199**	**558**	**1944**	**C**	**WD**	**I**	**C**	**C**	**C**	**C**	**C**	**C**
C*04:04	04:04			11	1	50	8	34	7	29	140	WD		WD	WD	I	C	I	WD	C
**C*04:04:01G total**	**04:04:01G total**			**169**	**9**	**529**	**100**	**257**	**191**	**523**	**1778**	**C**	**WD**	**I**	**C**	**C**	**C**	**C**	**C**	**C**
C*04:04:01G	04:04:01G		04:04:01G	2	2	21	2	13	2	12	54			WD		I		WD	WD	I
C*04:04:01	04:04:01		04:04:01G	167	7	504	97	238	186	500	1699	C	WD	I	C	C	C	C	C	C
C*04:04:01:01	04:04:01:01	HLA00424	04:04:01G	0	0	3	1	5	3	11	23					WD		WD	WD	WD
C*04:04:01:02	04:04:01:02	HLA16644	04:04:01G	0	0	1	0	1	0	0	2									
C*04:04:02	04:04:02	HLA01831		0	0	1	0	18	1	6	26					I		WD	WD	I
C*04:05	04:05	HLA00425		3	1	97	0	1	0	5	107			WD				WD	WD	WD
**C*04:06 total**	**04:06 total**			**1**	**1055**	**43**	**3**	**4**	**1**	**198**	**1305**		**C**	**WD**				**C**	**I**	**C**

Supplemental Table 10: HLA-C Allele Summary [a]				Allele Count by Population Group [b]								3.0.0 CIWD Category by Population Group [c]								
Allele	Genomic typing	Allele ID	G group	AFA	API	EURO	MENA	HIS	NAM	UNK	Total	AFA	API	EURO	MENA	HIS	NAM	UNK	Total	Highest Frequency
C*04:06	04:06			1	1046	43	3	4	1	198	1296		C	WD				C	I	C
C*04:06:01	04:06:01	HLA00426		0	9	0	0	0	0	0	9		WD						WD	WD
C*04:07	04:07	HLA01078		775	13	179	211	93	6	393	1670	C	I	I	C	C	WD	C	C	C
C*04:08	04:08	HLA01311		107	0	22	0	7	2	28	166	C		WD		I		I	I	C
C*04:10	04:10	HLA01645		2	1141	21	8	7	6	37	1222		C	WD	WD	I	WD	I	I	C
C*04:11	04:11	HLA01711		1	0	80	0	0	1	8	90			WD				WD	WD	WD
C*04:12	04:12	HLA01825		0	0	18	0	2	0	3	23			WD					WD	WD
C*04:13	04:13	HLA01851		199	2	19	1	18	6	53	298	C		WD		I	WD	I	I	C
C*04:14	04:14	HLA01853		5	0	10	16	0	0	0	31	WD		WD	WD				WD	WD
**C*04:15 total**	**04:15 total**			**4**	**1**	**66**	**1**	**4**	**1**	**13**	**90**			**WD**				**WD**	**WD**	**WD**
C*04:15	04:15			0	0	6	0	1	0	0	7			WD					WD	WD
C*04:15:01	04:15:01	HLA01859		4	0	0	0	0	0	1	5								WD	WD
C*04:15:02	04:15:02	HLA03106		0	1	60	1	3	1	12	78			WD				WD	WD	WD
C*04:16	04:16	HLA02193		0	3	28	0	0	0	1	32			WD					WD	WD
C*04:17	04:17	HLA02241		1	0	1	0	23	1	2	28					I			WD	I
C*04:19	04:19	HLA02511		1	0	4	0	101	10	13	129					C	C	WD	WD	C
C*04:20	04:20	HLA02525		0	0	1	0	0	0	0	1									
C*04:23	04:23	HLA02621		0	1	4	0	0	0	0	5								WD	WD
C*04:24	04:24	HLA02622		0	0	24	0	3	0	4	31			WD					WD	WD
C*04:25	04:25	HLA02822		0	1	1	0	0	0	0	2									
C*04:26	04:26	HLA02824		4	59	1	0	0	0	2	66		I						WD	I
C*04:27	04:27	HLA02831		54	0	1	0	1	1	6	63	C						WD	WD	C
C*04:29	04:29	HLA03077		0	10	24	12	1	1	5	53		WD	WD	WD			WD	WD	WD
C*04:32	04:32	HLA03214		1	0	9	0	1	0	0	11			WD					WD	WD
C*04:33	04:33	HLA03364		6	6	25	12	6	0	7	62	WD	WD	WD	WD	WD		WD	WD	WD
C*04:34	04:34	HLA03404		6	0	0	0	0	0	0	6	WD							WD	WD
C*04:35	04:35	HLA03430		0	0	3	0	0	0	0	3									
C*04:36	04:36	HLA03437		5	2	4	0	0	0	2	13	WD							WD	WD
C*04:37	04:37	HLA03527		0	1	70	0	0	0	2	73			WD					WD	WD
C*04:39	04:39	HLA03735		0	0	1	9	0	0	1	11				WD				WD	WD
C*04:40	04:40	HLA03699		13	0	0	1	0	0	1	15	WD							WD	WD

Supplemental Table 10: HLA-C Allele Summary [a]				Allele Count by Population Group [b]								3.0.0 CIWD Category by Population Group [c]								
**Allele**	**Genomic typing**	**Allele ID**	**G group**	**AFA**	**API**	**EURO**	**MENA**	**HIS**	**NAM**	**UNK**	**Total**	**AFA**	**API**	**EURO**	**MENA**	**HIS**	**NAM**	**UNK**	**Total**	**Highest Frequency**
**C*04:42 total**	**04:42 total**			**0**	**0**	**1**	**1**	**0**	**0**	**0**	**2**									
C*04:42:02	04:42:02	HLA11853		0	0	1	1	0	0	0	2									
C*04:43	04:43	HLA04276		0	0	3	0	0	0	1	4									
C*04:45	04:45	HLA04300		0	0	21	0	0	0	0	21			WD					WD	WD
C*04:46	04:46	HLA04301		0	0	2	0	23	0	2	27					I			WD	I
C*04:47	04:47	HLA04303		0	0	27	0	0	0	0	27			WD					WD	WD
C*04:48	04:48	HLA04309		0	0	1	0	0	0	0	1									
C*04:49	04:49	HLA04395		0	0	1	0	0	0	0	1									
C*04:50	04:50	HLA04610		0	0	2	0	0	0	1	3									
C*04:52	04:52	HLA04617		4	0	7	0	1	0	4	16			WD					WD	WD
C*04:53	04:53	HLA04625		2	0	0	0	0	0	0	2									
C*04:54	04:54	HLA04695		0	1	12	1	0	0	2	16			WD					WD	WD
C*04:55	04:55	HLA04696		0	0	5	0	0	0	0	5			WD					WD	WD
C*04:56	04:56	HLA04763		0	0	32	0	0	0	0	32			WD					WD	WD
C*04:58	04:58	HLA04838		0	1	3	0	0	0	0	4									
C*04:59Q	04:59Q	HLA04841		0	0	24	0	1	0	1	26			WD					WD	WD
C*04:61	04:61	HLA04843		0	0	3	0	0	0	1	4									
C*04:62	04:62	HLA04851		0	0	4	0	0	0	0	4									
C*04:63	04:63	HLA05166		0	0	12	0	0	0	3	15			WD					WD	WD
**C*04:64 total**	**04:64 total**			**0**	**0**	**1**	**0**	**0**	**0**	**0**	**1**									
C*04:64:02	04:64:02	HLA08223		0	0	1	0	0	0	0	1									
C*04:65	04:65	HLA05170		0	0	24	4	3	0	3	34			WD					WD	WD
C*04:66	04:66	HLA05175		0	0	1	0	0	0	0	1									
C*04:67	04:67	HLA05191		0	0	1	0	0	0	0	1									
C*04:69	04:69	HLA05218		0	2	1	0	0	0	0	3									
C*04:70	04:70	HLA05414		0	2	1	0	0	0	2	5								WD	WD
C*04:71	04:71	HLA05543		0	0	5	0	3	0	4	12			WD					WD	WD
C*04:72	04:72	HLA05573		0	0	3	0	0	0	0	3									
C*04:73	04:73	HLA05592		0	0	1	0	0	0	0	1									
C*04:75	04:75	HLA05604		0	0	14	0	0	0	2	16			WD					WD	WD
C*04:77	04:77	HLA05739		0	0	63	0	0	0	1	64			WD					WD	WD

Supplemental Table 10: HLA-C Allele Summary [a]				Allele Count by Population Group [b]								3.0.0 CIWD Category by Population Group [c]								
Allele	Genomic typing	Allele ID	G group	AFA	API	EURO	MENA	HIS	NAM	UNK	Total	AFA	API	EURO	MENA	HIS	NAM	UNK	Total	Highest Frequency
C*04:78	04:78	HLA05745		0	0	3	0	0	0	0	3									
C*04:81	04:81	HLA05883		0	3	0	0	0	0	0	3									
C*04:83	04:83	HLA05947		0	0	3	0	0	0	0	3									
C*04:85	04:85	HLA06385		0	0	1	0	1	0	0	2									
C*04:86	04:86	HLA06401		0	0	18	0	0	0	0	18			WD					WD	WD
C*04:87	04:87	HLA06403		1	3	0	1	0	0	0	5								WD	WD
C*04:88N	04:88N	HLA06422		0	0	0	1	0	0	1	2									
C*04:89	04:89	HLA06436		0	3	0	0	0	0	1	4									
C*04:90	04:90	HLA06665		0	0	1	0	0	0	0	1									
C*04:91	04:91	HLA06730		0	0	2	0	0	0	0	2									
C*04:93N	04:93N	HLA07009		0	0	7	0	0	0	1	8			WD					WD	WD
**C*04:94 total**	**04:94 total**			**0**	**1**	**1**	**0**	**0**	**0**	**1**	**3**									
C*04:94:01	04:94:01	HLA07010		0	1	1	0	0	0	1	3									
C*04:95N	04:95N	HLA07017		0	0	6	0	0	0	0	6			WD					WD	WD
C*04:96	04:96	HLA07018		0	1	25	0	0	0	0	26			WD					WD	WD
C*04:97	04:97	HLA07019		0	0	0	0	1	0	0	1									
**C*04:98 total**	**04:98 total**			**0**	**0**	**0**	**0**	**0**	**0**	**1**	**1**									
C*04:98:02	04:98:02	HLA09045		0	0	0	0	0	0	1	1									
C*04:99	04:99	HLA07026		0	0	3	0	0	0	0	3									
C*04:102	04:102	HLA07120		0	0	1	0	0	0	0	1									
C*04:104	04:104	HLA07142		0	0	4	0	0	0	1	5								WD	WD
C*04:105N	04:105N	HLA07243		0	0	2	0	0	0	0	2									
C*04:107	04:107	HLA07406		0	1	0	0	0	0	0	1									
C*04:108	04:108	HLA07499		0	0	4	2	0	0	0	6								WD	WD
C*04:109	04:109	HLA07500		0	0	0	0	1	0	0	1									
C*04:110	04:110	HLA07501		0	0	3	0	0	0	0	3									
C*04:112	04:112	HLA07755		0	0	2	0	0	0	0	2									
C*04:113	04:113	HLA07970		1	0	1	0	3	1	2	8								WD	WD
C*04:114	04:114	HLA07971		4	0	6	0	0	0	1	11			WD					WD	WD
C*04:116	04:116	HLA07974		0	0	1	1	0	0	0	2									
C*04:117	04:117	HLA07975		0	4	0	0	0	0	0	4									

Supplemental Table 10: HLA-C Allele Summary [a]				Allele Count by Population Group [b]								3.0.0 CIWD Category by Population Group [c]								
Allele	Genomic typing	Allele ID	G group	AFA	API	EURO	MENA	HIS	NAM	UNK	Total	AFA	API	EURO	MENA	HIS	NAM	UNK	Total	Highest Frequency
C*04:118	04:118	HLA08022		0	0	7	0	0	0	0	7			WD					WD	WD
C*04:120	04:120	HLA08171		0	0	2	0	0	0	0	2									
C*04:121	04:121	HLA08172		4	0	0	0	0	0	1	5								WD	WD
C*04:122	04:122	HLA08173		0	0	1	0	2	0	0	3									
C*04:123N	04:123N	HLA08174		0	1	0	0	0	0	0	1									
C*04:124	04:124	HLA08221		0	0	2	0	2	0	0	4									
C*04:125	04:125	HLA08222		0	0	7	0	0	0	0	7			WD					WD	WD
C*04:126	04:126	HLA08224		0	0	1	0	0	0	0	1									
C*04:127	04:127	HLA08227		0	0	3	0	0	0	0	3									
C*04:128	04:128	HLA08413		0	0	0	0	2	0	0	2									
C*04:130	04:130	HLA08542		0	1	0	0	0	0	0	1									
C*04:131	04:131	HLA08543		0	1	3	0	6	0	0	10					WD			WD	WD
C*04:132	04:132	HLA08544		0	0	5	7	0	0	0	12			WD	WD				WD	WD
C*04:133	04:133	HLA08822		0	1	1	0	0	0	0	2									
C*04:134	04:134	HLA08823		0	0	0	0	0	0	1	1									
C*04:137	04:137	HLA08829		0	0	0	3	0	0	0	3									
C*04:138	04:138	HLA08830		0	0	0	0	0	0	1	1									
C*04:139	04:139	HLA08832		0	0	8	0	0	0	0	8			WD					WD	WD
C*04:141	04:141	HLA09046		0	0	0	5	1	0	2	8				WD				WD	WD
C*04:142	04:142	HLA09047		0	0	4	0	0	0	0	4									
C*04:143	04:143	HLA09049		0	0	2	0	0	0	0	2									
C*04:145	04:145	HLA09176		0	0	1	0	0	0	0	1									
C*04:150	04:150	HLA09630		0	0	8	0	0	0	0	8			WD					WD	WD
C*04:153	04:153	HLA09907		0	2	6	0	0	0	2	10			WD					WD	WD
C*04:155	04:155	HLA10109		2	0	1	0	0	0	2	5								WD	WD
C*04:163	04:163	HLA10537		0	5	1	0	0	0	0	6		WD						WD	WD
C*04:166	04:166	HLA11120		0	1	0	0	0	0	1	2									
C*04:169	04:169	HLA11345		0	4	0	0	0	0	0	4									
C*04:171	04:171	HLA11573		0	1	0	0	0	0	0	1									
C*04:172	04:172	HLA11583		0	2	0	0	0	0	0	2									
C*04:173N	04:173N	HLA11670		0	0	1	0	0	0	0	1									

Supplemental Table 10: HLA-C Allele Summary [a]				Allele Count by Population Group [b]								3.0.0 CIWD Category by Population Group [c]								
Allele	Genomic typing	Allele ID	G group	AFA	API	EURO	MENA	HIS	NAM	UNK	Total	AFA	API	EURO	MENA	HIS	NAM	UNK	Total	Highest Frequency
C*04:177	04:177	HLA12149		1	0	1	1	0	0	0	3									
C*04:178	04:178	HLA12203		0	0	1	0	0	0	0	1									
**C*04:180 total**	**04:180 total**			**3**	**0**	**3**	**0**	**0**	**0**	**3**	**9**								**WD**	**WD**
C*04:180	04:180			2	0	1	0	0	0	2	5								WD	WD
C*04:180:01	04:180:01	HLA12347		1	0	0	0	0	0	1	2									
C*04:180:02	04:180:02	HLA13806		0	0	2	0	0	0	0	2									
C*04:181	04:181	HLA12349		0	0	1	0	0	0	0	1									
C*04:182	04:182	HLA12513		0	0	0	0	2	2	0	4									
C*04:185	04:185	HLA12516		0	0	1	0	1	0	0	2									
C*04:186	04:186	HLA12702		0	0	1	0	0	0	1	2									
C*04:188	04:188	HLA12714		0	1	1	1	0	0	0	3									
C*04:191N	04:191N	HLA12728		0	0	1	0	0	0	0	1									
C*04:193	04:193	HLA12791		0	0	0	0	2	0	0	2									
C*04:194	04:194	HLA12792		1	1	0	0	0	1	0	3									
C*04:197	04:197	HLA13268		0	0	0	0	1	0	0	1									
C*04:200	04:200	HLA13426		0	0	0	0	1	0	0	1									
C*04:202	04:202	HLA13672		1	0	0	0	0	0	0	1									
C*04:204	04:204	HLA13674		0	6	0	0	0	0	0	6		WD						WD	WD
C*04:205N	04:205N	HLA13675		0	1	0	0	0	0	0	1									
C*04:206	04:206	HLA13676		0	0	3	0	0	0	0	3									
C*04:207	04:207	HLA13805		0	9	0	0	0	0	0	9		WD						WD	WD
C*04:209	04:209	HLA13808		0	1	4	0	0	0	3	8								WD	WD
C*04:210	04:210	HLA13878		0	1	0	0	0	0	0	1									
C*04:213	04:213	HLA14031		0	2	0	0	0	0	0	2									
C*04:214	04:214	HLA14032		0	2	0	0	0	0	0	2									
C*04:215N	04:215N	HLA14033		0	1	0	0	0	0	0	1									
C*04:217N	04:217N	HLA14110		0	0	1	2	0	0	0	3									
C*04:218	04:218	HLA14263		0	0	3	0	0	0	2	5								WD	WD
C*04:221	04:221	HLA14340		0	0	1	0	0	0	0	1									
C*04:236N	04:236N	HLA15144		0	0	3	0	0	0	0	3									
C*04:239	04:239	HLA14997		0	0	1	0	0	0	1	2									

Supplemental Table 10: HLA-C Allele Summary [a]				Allele Count by Population Group [b]								3.0.0 CIWD Category by Population Group [c]								
Allele	Genomic typing	Allele ID	G group	AFA	API	EURO	MENA	HIS	NAM	UNK	Total	AFA	API	EURO	MENA	HIS	NAM	UNK	Total	Highest Frequency
C*04:261	04:261	HLA16556		0	1	0	0	0	0	0	1									
C*04:CODE	04:CODE			8314	7020	143648	3862	13268	1500	22400	200012	NA	NA	NA	NA	NA	NA	NA	NA	NA
**C*05:01 total**	**05:01 total**			**9321**	**9180**	**756088**	**9233**	**30844**	**2799**	**74466**	**891931**	**C**	**C**	**C**	**C**	**C**	**C**	**C**	**C**	**C**
C*05:01	05:01			1	2	2907	5	15	1	149	3080			C	WD	I		C	C	C
C*05:01P	05:01P			0	4	743	4	2	0	4	757			I					I	I
**C*05:01:01G total**	**05:01:01G total**			**9314**	**9174**	**752088**	**9223**	**30815**	**2795**	**74258**	**887667**	**C**	**C**	**C**	**C**	**C**	**C**	**C**	**C**	**C**
C*05:01:01G	05:01:01G		05:01:01G	7134	8330	703068	8970	21361	2058	66569	817490	C	C	C	C	C	C	C	C	C
C*05:01:01	05:01:01		05:01:01G	947	338	21018	124	3984	284	3743	30438	C	C	C	C	C	C	C	C	C
C*05:01:01:01	05:01:01:01	HLA00427	05:01:01G	271	25	3657	22	1865	133	643	6616	C	I	C	WD	C	C	C	C	C
C*05:01:01:02	05:01:01:02	HLA03586	05:01:01G	958	479	24158	100	3555	319	3265	32834	C	C	C	C	C	C	C	C	C
C*05:01:01:04	05:01:01:04	HLA15770	05:01:01G	0	0	1	0	0	0	0	1									
C*05:01:01:05	05:01:01:05	HLA15772	05:01:01G	0	0	1	0	1	0	1	3									
C*05:01:01:08	05:01:01:08	HLA17049	05:01:01G	0	0	15	0	9	1	3	28			WD		I			WD	I
C*05:01:01:09	05:01:01:09	HLA17633	05:01:01G	0	0	0	0	0	0	1	1									
C*05:01:04	05:01:04	HLA03191	05:01:01G	0	0	1	0	0	0	0	1									
C*05:01:05	05:01:05	HLA03978	05:01:01G	0	0	85	2	5	0	9	101			WD		WD		WD	WD	WD
C*05:01:15	05:01:15	HLA05880	05:01:01G	0	2	4	2	1	0	1	10								WD	WD
C*05:03	05:03	HLA01169	05:01:01G	0	0	4	0	0	0	0	4									
C*05:37	05:37	HLA04856	05:01:01G	2	0	55	3	34	0	18	112			WD		I		I	WD	I
C*05:53	05:53	HLA06125	05:01:01G	1	0	14	0	0	0	2	17			WD					WD	WD
C*05:93	05:93	HLA09646	05:01:01G	0	0	1	0	0	0	0	1									
C*05:108	05:108	HLA12591	05:01:01G	1	0	6	0	0	0	3	10			WD					WD	WD
C*05:01:02	05:01:02	HLA01998		0	0	81	0	3	0	12	96			WD				WD	WD	WD
C*05:01:03	05:01:03	HLA02292		4	0	29	0	3	0	7	43			WD				WD	WD	WD
C*05:01:07	05:01:07	HLA04293		0	0	12	0	1	0	1	14			WD					WD	WD
C*05:01:08	05:01:08	HLA04296		0	0	105	1	0	1	16	123			WD				I	WD	I
C*05:01:09	05:01:09	HLA04305		0	0	1	0	0	0	0	1									
C*05:01:11	05:01:11	HLA04391		2	0	62	0	2	1	7	74			WD				WD	WD	WD
C*05:01:12	05:01:12	HLA04798		0	0	3	0	0	0	1	4									
C*05:01:16	05:01:16	HLA06411		0	0	26	0	0	0	10	36			WD				WD	WD	WD
C*05:01:17	05:01:17	HLA07011		0	0	1	0	3	1	0	5								WD	WD

Supplemental Table 10: HLA-C Allele Summary [a]				Allele Count by Population Group [b]								3.0.0 CIWD Category by Population Group [c]								
Allele	Genomic typing	Allele ID	G group	AFA	API	EURO	MENA	HIS	NAM	UNK	Total	AFA	API	EURO	MENA	HIS	NAM	UNK	Total	Highest Frequency
C*05:01:19	05:01:19	HLA07976		0	0	2	0	0	0	0	2									
C*05:01:20	05:01:20	HLA07979		0	0	8	0	0	0	0	8			WD					WD	WD
C*05:01:21	05:01:21	HLA08156		0	0	1	0	0	0	1	2									
C*05:01:22	05:01:22	HLA08475		0	0	1	0	0	0	0	1									
C*05:01:23	05:01:23	HLA08532		0	0	1	0	0	0	0	1									
C*05:01:24	05:01:24	HLA08730		0	0	9	0	0	0	0	9			WD					WD	WD
C*05:01:27	05:01:27	HLA09609		0	0	5	0	0	0	0	5			WD					WD	WD
C*05:01:28	05:01:28	HLA12187		0	0	2	0	0	0	0	2									
C*05:01:30	05:01:30	HLA12332		0	0	1	0	0	0	0	1									
**C*05:04 total**	**05:04 total**			**0**	**0**	**16**	**0**	**34**	**0**	**11**	**61**			**WD**		**I**		**WD**	**WD**	**I**
C*05:04	05:04			0	0	4	0	13	0	3	20					I			WD	I
C*05:04:01	05:04:01	HLA01174		0	0	8	0	21	0	8	37			WD		I		WD	WD	I
C*05:04:02	05:04:02	HLA08736		0	0	4	0	0	0	0	4									
**C*05:05 total**	**05:05 total**			**4**	**1**	**376**	**1**	**5**	**0**	**45**	**432**			**I**		**WD**		**I**	**I**	**I**
C*05:05	05:05			4	1	336	1	5	0	44	391			I		WD		I	I	I
C*05:05:01	05:05:01	HLA01602		0	0	40	0	0	0	1	41			WD					WD	WD
C*05:06	05:06	HLA01661		0	0	24	0	1	0	1	26			WD					WD	WD
C*05:07N	05:07N	HLA01832		3	3	331	2	8	1	34	382			I		I		I	I	I
C*05:08	05:08	HLA01843		0	0	455	0	7	2	22	486			I		I		I	I	I
**C*05:09 total**	**05:09 total**			**8**	**2**	**251**	**2**	**386**	**31**	**138**	**818**	**WD**		**I**		**C**	**C**	**C**	**I**	**C**
C*05:09	05:09			0	1	45	0	60	5	5	116			WD		I	WD	WD	WD	I
C*05:09:01	05:09:01	HLA01856		8	1	205	2	326	26	132	700	WD		I		C	C	C	I	C
C*05:09:03	05:09:03	HLA12042		0	0	1	0	0	0	1	2									
C*05:10	05:10	HLA01907		1	0	48	0	1	0	8	58			WD				WD	WD	WD
C*05:11	05:11	HLA02148		7	0	28	1	1	0	7	44	WD		WD				WD	WD	WD
C*05:13	05:13	HLA02408		0	1	108	1	1	0	2	113			WD					WD	WD
C*05:14	05:14	HLA02586		0	0	46	0	1	0	14	61			WD				I	WD	I
C*05:15	05:15	HLA02753		0	0	20	0	0	0	1	21			WD					WD	WD
C*05:16	05:16	HLA02785		0	0	71	0	0	0	2	73			WD					WD	WD
C*05:17	05:17	HLA02979		0	0	1	0	0	0	0	1									
**C*05:18 total**	**05:18 total**			**0**	**0**	**18**	**20**	**0**	**0**	**3**	**41**			**WD**	**WD**				**WD**	**WD**

Supplemental Table 10: HLA-C Allele Summary [a]				Allele Count by Population Group [b]								3.0.0 CIWD Category by Population Group [c]								
Allele	Genomic typing	Allele ID	G group	AFA	API	EURO	MENA	HIS	NAM	UNK	Total	AFA	API	EURO	MENA	HIS	NAM	UNK	Total	Highest Frequency
C*05:18	05:18			0	0	4	4	0	0	0	8								WD	WD
C*05:18:01	05:18:01	HLA03212		0	0	12	16	0	0	3	31			WD	WD				WD	WD
C*05:18:03	05:18:03	HLA07031		0	0	2	0	0	0	0	2									
C*05:19	05:19	HLA03219		0	0	14	0	0	0	1	15			WD					WD	WD
C*05:20	05:20	HLA03438		0	0	1	0	0	0	1	2									
C*05:21	05:21	HLA03517		0	0	1	0	0	0	0	1									
**C*05:22 total**	**05:22 total**			**1**	**0**	**58**	**0**	**0**	**0**	**0**	**59**			**WD**					**WD**	**WD**
C*05:22	05:22			1	0	6	0	0	0	0	7			WD					WD	WD
C*05:22:01	05:22:01	HLA03531		0	0	17	0	0	0	0	17			WD					WD	WD
C*05:22:02	05:22:02	HLA07225		0	0	35	0	0	0	0	35			WD					WD	WD
C*05:24	05:24	HLA03634		0	0	5	0	0	0	0	5			WD					WD	WD
C*05:25	05:25	HLA03681		0	0	1	0	0	0	1	2									
C*05:26	05:26	HLA04297		0	0	6	16	0	0	0	22			WD	WD				WD	WD
C*05:27	05:27	HLA04302		0	0	8	0	0	0	0	8			WD					WD	WD
C*05:28	05:28	HLA04304		0	0	22	0	0	0	0	22			WD					WD	WD
**C*05:29 total**	**05:29 total**			**0**	**1**	**33**	**2**	**4**	**0**	**5**	**45**			**WD**				**WD**	**WD**	**WD**
C*05:29	05:29			0	0	8	2	2	0	1	13			WD					WD	WD
C*05:29:01	05:29:01	HLA04308		0	1	25	0	2	0	4	32			WD					WD	WD
C*05:30	05:30	HLA04310		0	0	1	0	0	0	0	1									
C*05:32	05:32	HLA04587		1	0	45	2	0	0	2	50			WD					WD	WD
C*05:33	05:33	HLA04596		0	0	9	0	0	0	0	9			WD					WD	WD
C*05:34	05:34	HLA04608		0	0	52	0	0	0	1	53			WD					WD	WD
C*05:35	05:35	HLA04677		0	0	8	0	0	0	3	11			WD					WD	WD
C*05:36	05:36	HLA04701		1	0	34	0	0	0	5	40			WD				WD	WD	WD
C*05:38	05:38	HLA04799		0	0	3	0	0	0	0	3									
C*05:39	05:39	HLA04801		0	0	35	0	0	0	2	37			WD					WD	WD
C*05:41	05:41	HLA04803		0	0	1	0	0	0	0	1									
C*05:42	05:42	HLA04832		0	0	1	0	0	0	0	1									
C*05:43	05:43	HLA05213		0	0	6	0	1	0	0	7			WD					WD	WD
**C*05:44 total**	**05:44 total**			**0**	**0**	**7**	**0**	**0**	**0**	**1**	**8**			**WD**					**WD**	**WD**
C*05:44:01	05:44:01	HLA05310		0	0	3	0	0	0	0	3									

Supplemental Table 10: HLA-C Allele Summary [a]				Allele Count by Population Group [b]								3.0.0 CIWD Category by Population Group [c]								
**Allele**	**Genomic typing**	**Allele ID**	**G group**	**AFA**	**API**	**EURO**	**MENA**	**HIS**	**NAM**	**UNK**	**Total**	**AFA**	**API**	**EURO**	**MENA**	**HIS**	**NAM**	**UNK**	**Total**	**Highest Frequency**
C*05:44:02	05:44:02	HLA08199		0	0	4	0	0	0	1	5								WD	WD
C*05:46	05:46	HLA05571		0	0	17	0	0	0	0	17			WD					WD	WD
C*05:47	05:47	HLA05585		0	0	16	0	0	0	0	16			WD					WD	WD
C*05:49	05:49	HLA05593		0	0	1	0	0	0	0	1									
C*05:51Q	05:51Q	HLA05852		0	0	6	0	0	0	1	7			WD					WD	WD
C*05:52	05:52	HLA06054		0	0	3	0	0	0	0	3									
C*05:55	05:55	HLA06383		0	0	13	0	0	0	3	16			WD					WD	WD
**C*05:58 total**	**05:58 total**			**0**	**0**	**1**	**0**	**0**	**0**	**0**	**1**									
C*05:58:02	05:58:02	HLA07222		0	0	1	0	0	0	0	1									
C*05:59	05:59	HLA06720		0	0	1	0	0	0	0	1									
C*05:60	05:60	HLA07052		0	0	4	0	0	0	0	4									
C*05:61	05:61	HLA07063		0	1	0	0	0	0	0	1									
C*05:63	05:63	HLA07082		0	0	2	0	0	0	0	2									
**C*05:64 total**	**05:64 total**			**0**	**0**	**2**	**0**	**0**	**0**	**0**	**2**									
C*05:64	05:64			0	0	1	0	0	0	0	1									
C*05:64:02	05:64:02	HLA09191		0	0	1	0	0	0	0	1									
C*05:65	05:65	HLA07109		0	0	12	0	0	1	1	14			WD					WD	WD
C*05:67	05:67	HLA07134		0	0	9	0	0	0	0	9			WD					WD	WD
C*05:68	05:68	HLA07135		0	0	1	0	0	0	0	1									
C*05:74	05:74	HLA07488		0	0	1	0	0	0	1	2									
C*05:75	05:75	HLA07977		0	0	1	0	0	0	0	1									
C*05:76	05:76	HLA07978		0	0	1	0	0	0	0	1									
C*05:77	05:77	HLA07980		0	0	6	0	0	0	1	7			WD					WD	WD
C*05:80	05:80	HLA08198		0	0	24	0	0	0	0	24			WD					WD	WD
C*05:81	05:81	HLA08200		0	0	2	0	0	0	0	2									
C*05:82	05:82	HLA08733		0	0	2	0	0	0	0	2									
C*05:84	05:84	HLA08735		0	0	0	0	0	0	1	1									
C*05:87	05:87	HLA09025		0	0	1	0	0	0	0	1									
C*05:89	05:89	HLA09189		0	0	3	0	0	0	0	3									
C*05:91N	05:91N	HLA09192		0	0	1	0	0	0	0	1									
C*05:97	05:97	HLA10079		0	0	5	0	0	0	0	5			WD					WD	WD

Supplemental Table 10: HLA-C Allele Summary [a]				Allele Count by Population Group [b]								3.0.0 CIWD Category by Population Group [c]								
**Allele**	**Genomic typing**	**Allele ID**	**G group**	**AFA**	**API**	**EURO**	**MENA**	**HIS**	**NAM**	**UNK**	**Total**	**AFA**	**API**	**EURO**	**MENA**	**HIS**	**NAM**	**UNK**	**Total**	**Highest Frequency**
C*05:98	05:98	HLA10437		0	0	6	0	0	0	0	6			WD					WD	WD
C*05:99N	05:99N	HLA10664		0	0	6	0	0	0	1	7			WD					WD	WD
C*05:100	05:100	HLA11093		0	0	1	0	0	0	0	1									
**C*05:103 total**	**05:103 total**			**1**	**0**	**0**	**0**	**0**	**0**	**0**	**1**									
C*05:103P	05:103P			1	0	0	0	0	0	0	1									
C*05:104	05:104	HLA12188		0	0	21	0	0	0	0	21			WD					WD	WD
C*05:105	05:105	HLA12405		0	0	0	0	0	0	1	1									
C*05:109	05:109	HLA12706		0	0	2	0	0	0	0	2									
C*05:111	05:111	HLA12726		0	0	5	0	0	0	0	5			WD					WD	WD
C*05:113N	05:113N	HLA12687		0	0	0	1	0	0	0	1									
C*05:116	05:116	HLA13100		0	0	1	0	0	0	0	1									
C*05:118	05:118	HLA13529		0	0	1	0	0	0	0	1									
C*05:120	05:120	HLA13564		1	0	0	0	0	0	0	1									
C*05:121	05:121	HLA13752		0	0	0	0	2	0	1	3									
C*05:122	05:122	HLA13753		0	0	0	0	0	0	1	1									
C*05:128N	05:128N	HLA14606		1	0	0	0	0	0	0	1									
C*05:131	05:131	HLA15047		0	0	1	0	0	0	0	1									
C*05:132	05:132	HLA15048		0	0	2	0	0	0	0	2									
C*05:CODE	05:CODE			1180	382	61100	496	3436	364	6533	73491	NA	NA	NA	NA	NA	NA	NA	NA	NA
**C*06:02 total**	**06:02 total**			**29557**	**122838**	**1057665**	**43692**	**37816**	**3750**	**102420**	**1397738**	**C**	**C**	**C**	**C**	**C**	**C**	**C**	**C**	**C**
C*06:02	06:02			150	121	17611	503	41	16	1291	19733	C	I	C	C	I	C	C	C	C
C*06:02P	06:02P			5	36	2308	321	8	1	1371	4050	WD	I	C	C	I		C	C	C
**C*06:02:01G total**	**06:02:01G total**			**29398**	**122664**	**1037518**	**42512**	**37739**	**3730**	**99733**	**1373294**	**C**	**C**	**C**	**C**	**C**	**C**	**C**	**C**	**C**
C*06:02:01G	06:02:01G		06:02:01G	23890	115164	992544	41530	28265	2792	92646	1296831	C	C	C	C	C	C	C	C	C
C*06:02:01	06:02:01		06:02:01G	1311	1887	11992	241	2309	211	1742	19693	C	C	C	C	C	C	C	C	C
C*06:02:01:01	06:02:01:01	HLA00430	06:02:01G	3454	5046	27453	435	4755	514	4267	45924	C	C	C	C	C	C	C	C	C
C*06:02:01:02	06:02:01:02	HLA02561	06:02:01G	173	531	3703	282	1437	132	655	6913	C	C	C	C	C	C	C	C	C
C*06:02:01:03	06:02:01:03	HLA10027	06:02:01G	556	36	1806	24	969	79	418	3888	C	I	C	WD	C	C	C	C	C
C*06:02:01:08	06:02:01:08	HLA17054	06:02:01G	0	0	16	0	0	0	3	19			WD					WD	WD
C*06:02:03	06:02:03	HLA03018	06:02:01G	13	0	0	0	4	2	2	21	WD							WD	WD
C*06:02:29	06:02:29	HLA09984	06:02:01G	1	0	0	0	0	0	0	1									

Supplemental Table 10: HLA-C Allele Summary [a]				Allele Count by Population Group [b]								3.0.0 CIWD Category by Population Group [c]								
Allele	Genomic typing	Allele ID	G group	AFA	API	EURO	MENA	HIS	NAM	UNK	Total	AFA	API	EURO	MENA	HIS	NAM	UNK	Total	Highest Frequency
C*06:73	06:73	HLA07557	06:02:01G	0	0	1	0	0	0	0	1									
C*06:146	06:146	HLA12675	06:02:01G	0	0	2	0	0	0	0	2									
C*06:176	06:176	HLA14894	06:02:01G	0	0	1	0	0	0	0	1									
C*06:02:04	06:02:04	HLA04291		0	0	4	0	0	0	0	4									
C*06:02:05	06:02:05	HLA04292		0	0	11	0	0	0	0	11			WD					WD	WD
C*06:02:06	06:02:06	HLA05174		0	0	34	1	0	0	2	37			WD					WD	WD
C*06:02:07	06:02:07	HLA05176		0	0	5	0	25	2	5	37			WD		I		WD	WD	I
C*06:02:08	06:02:08	HLA05195		0	0	20	0	0	0	5	25			WD				WD	WD	WD
C*06:02:09	06:02:09	HLA05602		0	0	2	0	0	0	0	2									
C*06:02:11	06:02:11	HLA05783		0	0	1	0	0	0	0	1									
C*06:02:12	06:02:12	HLA06388		0	0	49	0	0	0	1	50			WD					WD	WD
C*06:02:14	06:02:14	HLA06442		0	0	1	0	0	0	0	1									
C*06:02:15	06:02:15	HLA06707		0	0	2	0	0	0	1	3									
C*06:02:16	06:02:16	HLA06715		0	0	1	0	0	0	1	2									
C*06:02:17	06:02:17	HLA07066		0	0	0	1	0	0	1	2									
C*06:02:18	06:02:18	HLA07216		0	0	8	0	0	0	0	8			WD					WD	WD
C*06:02:20	06:02:20	HLA07832		0	0	2	0	2	1	0	5								WD	WD
C*06:02:22	06:02:22	HLA08698		0	0	6	0	0	0	1	7			WD					WD	WD
C*06:02:25	06:02:25	HLA09016		0	0	0	4	0	0	0	4									
C*06:02:26	06:02:26	HLA09181		0	0	3	0	0	0	0	3									
C*06:02:27	06:02:27	HLA09725		0	0	3	0	0	0	0	3									
C*06:02:31	06:02:31	HLA10057		2	0	1	0	0	0	0	3									
C*06:02:33	06:02:33	HLA10062		0	4	4	0	0	0	0	8								WD	WD
C*06:02:37	06:02:37	HLA11251		0	7	4	0	1	0	0	12		WD						WD	WD
C*06:02:38	06:02:38	HLA11439		0	0	2	350	0	0	6	358				C			WD	I	C
C*06:02:39	06:02:39	HLA12136		1	0	1	0	0	0	0	2									
C*06:02:40	06:02:40	HLA12138		0	0	4	0	0	0	0	4									
C*06:02:43	06:02:43	HLA12766		0	3	1	0	0	0	0	4									
C*06:02:44	06:02:44	HLA13408		0	0	1	0	0	0	0	1									
C*06:02:45	06:02:45	HLA14611		1	0	0	0	0	0	0	1									
C*06:02:46	06:02:46	HLA15881		0	1	0	0	0	0	0	1									

Supplemental Table 10: HLA-C Allele Summary [a]				Allele Count by Population Group [b]								3.0.0 CIWD Category by Population Group [c]								
Allele	Genomic typing	Allele ID	G group	AFA	API	EURO	MENA	HIS	NAM	UNK	Total	AFA	API	EURO	MENA	HIS	NAM	UNK	Total	Highest Frequency
C*06:17	06:17	HLA02641		0	2	58	0	0	0	2	62			WD					WD	WD
**C*06:03 total**	**06:03 total**			**0**	**2**	**40**	**2**	**0**	**0**	**2**	**46**			**WD**					**WD**	**WD**
C*06:03	06:03			0	0	24	0	0	0	0	24			WD					WD	WD
C*06:03:01	06:03:01	HLA00431		0	0	8	2	0	0	1	11			WD					WD	WD
C*06:03:02	06:03:02	HLA07492		0	2	8	0	0	0	1	11			WD					WD	WD
**C*06:04 total**	**06:04 total**			**0**	**1**	**333**	**2**	**2**	**1**	**7**	**346**			**I**				**WD**	**I**	**I**
C*06:04	06:04			0	0	236	2	1	1	7	247			I				WD	I	I
C*06:04:01	06:04:01	HLA00432		0	0	97	0	1	0	0	98			WD					WD	WD
C*06:04:02	06:04:02	HLA13971		0	1	0	0	0	0	0	1									
C*06:06	06:06	HLA01258		8	9	309	79	7	0	42	454	WD	WD	I	C	I		I	I	C
C*06:07	06:07	HLA01301		0	0	260	0	6	1	24	291			I		WD		I	I	I
C*06:08	06:08	HLA01628		71	0	14	0	20	3	22	130	C		WD		I		I	WD	C
C*06:09	06:09	HLA01681		58	2	6	0	38	0	13	117	C		WD		I		WD	WD	C
C*06:10	06:10	HLA01824		0	0	5	0	0	0	0	5			WD					WD	WD
C*06:11	06:11	HLA01880		0	2	10	8	1	0	2	23			WD	WD				WD	WD
C*06:12	06:12	HLA02178		0	4	0	0	0	0	1	5								WD	WD
C*06:13	06:13	HLA02239		0	0	8	0	0	0	0	8			WD					WD	WD
C*06:14	06:14	HLA02708		0	0	77	0	4	0	4	85			WD					WD	WD
C*06:15	06:15	HLA02832		0	3	0	0	0	0	1	4									
C*06:16N	06:16N	HLA02833		0	0	11	0	1	0	1	13			WD					WD	WD
C*06:18	06:18	HLA03429		0	0	133	0	0	0	3	136			I					WD	I
C*06:19	06:19	HLA03526		0	0	2	0	0	0	1	3									
C*06:20	06:20	HLA03624		0	1	0	0	0	0	0	1									
C*06:22	06:22	HLA03682		0	0	1	0	0	0	0	1									
C*06:23	06:23	HLA03697		0	1	101	0	0	0	2	104			WD					WD	WD
C*06:25	06:25	HLA04294		0	0	2	0	0	0	0	2									
C*06:26	06:26	HLA04295		0	0	9	0	1	0	1	11			WD					WD	WD
C*06:27	06:27	HLA04299		0	299	7	8	0	0	10	324		C	WD	WD			WD	I	C
C*06:28	06:28	HLA04307		0	0	56	0	0	0	2	58			WD					WD	WD
C*06:30	06:30	HLA04697		0	3	61	1	2	0	4	71			WD					WD	WD
C*06:32	06:32	HLA04745		2	0	5	0	0	0	0	7			WD					WD	WD

Supplemental Table 10: HLA-C Allele Summary [a]				Allele Count by Population Group [b]								3.0.0 CIWD Category by Population Group [c]								
Allele	Genomic typing	Allele ID	G group	AFA	API	EURO	MENA	HIS	NAM	UNK	Total	AFA	API	EURO	MENA	HIS	NAM	UNK	Total	Highest Frequency
**C*06:34 total**	**06:34 total**			**0**	**2**	**12**	**14**	**0**	**0**	**4**	**32**			**WD**	**WD**				**WD**	**WD**
C*06:34	06:34			0	0	7	12	0	0	2	21			WD	WD				WD	WD
C*06:34:01	06:34:01	HLA04828		0	0	5	2	0	0	2	9			WD					WD	WD
C*06:34:02	06:34:02	HLA14020		0	2	0	0	0	0	0	2									
C*06:35	06:35	HLA04835		0	36	0	1	0	0	0	37		I						WD	I
C*06:36	06:36	HLA04837		0	0	1	0	0	0	0	1									
C*06:37	06:37	HLA04844		0	4	3	1	1	0	0	9								WD	WD
C*06:38	06:38	HLA05164		0	0	32	0	0	3	3	38			WD					WD	WD
C*06:39	06:39	HLA05168		0	0	4	0	0	0	1	5								WD	WD
C*06:41	06:41	HLA05192		0	0	3	0	0	0	0	3									
**C*06:42 total**	**06:42 total**			**0**	**0**	**2**	**0**	**0**	**0**	**0**	**2**									
C*06:42:01	06:42:01	HLA05207		0	0	2	0	0	0	0	2									
**C*06:43 total**	**06:43 total**			**0**	**0**	**4**	**0**	**0**	**0**	**0**	**4**									
C*06:43	06:43			0	0	1	0	0	0	0	1									
C*06:43:02	06:43:02	HLA09180		0	0	3	0	0	0	0	3									
C*06:44	06:44	HLA05397		0	0	0	2	0	0	0	2									
C*06:47	06:47	HLA05559		0	0	23	0	0	0	0	23			WD					WD	WD
C*06:49N	06:49N	HLA05576		2	0	1	1	0	0	0	4									
C*06:50	06:50	HLA05583		0	0	5	0	0	0	1	6			WD					WD	WD
C*06:51	06:51	HLA05598		1	0	0	0	0	0	0	1									
C*06:52	06:52	HLA05619		0	0	2	0	0	0	0	2									
**C*06:53 total**	**06:53 total**			**0**	**0**	**3**	**6**	**0**	**0**	**0**	**9**				**WD**				**WD**	**WD**
C*06:53	06:53			0	0	0	1	0	0	0	1									
C*06:53:01	06:53:01	HLA05734		0	0	2	5	0	0	0	7				WD				WD	WD
C*06:53:02	06:53:02	HLA08473		0	0	1	0	0	0	0	1									
C*06:54	06:54	HLA05737		0	4	37	0	0	0	1	42			WD					WD	WD
C*06:58	06:58	HLA06275		0	0	9	0	0	0	2	11			WD					WD	WD
C*06:59	06:59	HLA06407		0	0	11	0	0	0	8	19			WD				WD	WD	WD
C*06:60	06:60	HLA06440		0	2	0	0	0	0	0	2									
C*06:61	06:61	HLA06441		0	0	2	0	0	0	0	2									
C*06:62	06:62	HLA06714		0	0	1	0	0	0	0	1									

Supplemental Table 10: HLA-C Allele Summary [a]				Allele Count by Population Group [b]								3.0.0 CIWD Category by Population Group [c]								
Allele	Genomic typing	Allele ID	G group	AFA	API	EURO	MENA	HIS	NAM	UNK	Total	AFA	API	EURO	MENA	HIS	NAM	UNK	Total	Highest Frequency
C*06:63	06:63	HLA06732		0	0	1	0	0	0	0	1									
C*06:64	06:64	HLA06734		0	0	2	0	0	0	0	2									
C*06:65	06:65	HLA07046		0	0	10	0	0	0	0	10			WD					WD	WD
C*06:66	06:66	HLA07065		0	0	8	0	0	0	1	9			WD					WD	WD
C*06:67	06:67	HLA07069		0	0	0	0	2	0	0	2									
C*06:69	06:69	HLA07131		0	0	1	0	0	0	0	1									
**C*06:70 total**	**06:70 total**			**0**	**0**	**9**	**1**	**0**	**0**	**0**	**10**			**WD**					**WD**	**WD**
C*06:70	06:70			0	0	2	1	0	0	0	3									
C*06:70:01	06:70:01	HLA07478		0	0	7	0	0	0	0	7			WD					WD	WD
C*06:71	06:71	HLA07479		0	0	17	0	0	0	0	17			WD					WD	WD
C*06:75	06:75	HLA07740		0	1	0	0	0	0	0	1									
**C*06:76 total**	**06:76 total**			**0**	**0**	**2**	**0**	**1**	**0**	**0**	**3**									
C*06:76:01	06:76:01	HLA07815		0	0	1	0	0	0	0	1									
C*06:76:02	06:76:02	HLA08205		0	0	1	0	1	0	0	2									
C*06:77	06:77	HLA07826		0	0	3	1	0	0	0	4									
C*06:78	06:78	HLA07827		0	0	4	0	0	0	0	4									
C*06:79N	06:79N	HLA07830		0	27	0	0	0	0	0	27		I						WD	I
C*06:80	06:80	HLA07831		0	1	1	0	0	0	0	2									
C*06:81	06:81	HLA07833		0	0	1	0	0	0	0	1									
C*06:82	06:82	HLA07834		0	1	0	0	0	0	0	1									
C*06:84	06:84	HLA08148		0	3	0	0	0	0	0	3									
C*06:85	06:85	HLA08149		0	0	1	0	0	0	0	1									
C*06:86	06:86	HLA08181		0	0	16	0	0	0	0	16			WD					WD	WD
C*06:87	06:87	HLA08182		0	0	17	0	0	0	2	19			WD					WD	WD
C*06:89	06:89	HLA08695		0	2	2	0	0	0	0	4									
C*06:90	06:90	HLA08696		0	0	2	1	0	0	0	3									
C*06:91	06:91	HLA08697		0	0	14	0	0	0	0	14			WD					WD	WD
C*06:92	06:92	HLA08700		2	0	0	0	0	0	1	3									
C*06:95	06:95	HLA09015		0	0	2	0	0	0	0	2									
C*06:96	06:96	HLA09017		0	7	4	1	0	0	3	15		WD						WD	WD
C*06:97	06:97	HLA09179		0	0	0	0	0	0	1	1									

Supplemental Table 10: HLA-C Allele Summary [a]				Allele Count by Population Group [b]								3.0.0 CIWD Category by Population Group [c]								
Allele	Genomic typing	Allele ID	G group	AFA	API	EURO	MENA	HIS	NAM	UNK	Total	AFA	API	EURO	MENA	HIS	NAM	UNK	Total	Highest Frequency
C*06:98	06:98	HLA09182		1	0	0	0	0	0	0	1									
C*06:101	06:101	HLA09599		0	0	3	0	0	0	0	3									
C*06:103	06:103	HLA09938		0	3	0	0	0	0	0	3									
C*06:104	06:104	HLA09967		0	1	0	0	0	0	0	1									
**C*06:106 total**	**06:106 total**			**0**	**1**	**2**	**1**	**0**	**0**	**0**	**4**									
C*06:106:01	06:106:01	HLA09971		0	0	2	0	0	0	0	2									
C*06:106:02	06:106:02	HLA09973		0	1	0	1	0	0	0	2									
C*06:113	06:113	HLA10061		0	0	0	1	0	0	1	2									
C*06:115	06:115	HLA10075		0	9	0	0	0	0	0	9		WD						WD	WD
C*06:122	06:122	HLA10603		0	0	1	0	0	0	1	2									
C*06:124	06:124	HLA10924		0	2	0	0	0	0	0	2									
C*06:125	06:125	HLA11041		0	0	1	0	0	0	0	1									
C*06:126	06:126	HLA11045		1	0	0	0	0	0	0	1									
**C*06:127 total**	**06:127 total**			**3**	**20**	**2**	**1**	**0**	**0**	**2**	**28**		**I**						**WD**	**I**
C*06:127	06:127			0	4	0	0	0	0	1	5								WD	WD
**C*06:127:01G total**	**06:127:01G total**			**3**	**16**	**2**	**1**	**0**	**0**	**0**	**22**		**I**						**WD**	**I**
C*06:127:01G	06:127:01G		06:127:01G	0	13	0	0	0	0	0	13		I						WD	I
C*06:127:01	06:127:01		06:127:01G	3	2	2	1	0	0	0	8								WD	WD
C*06:127:01:01	06:127:01:01	HLA11046	06:127:01G	0	1	0	0	0	0	0	1									
C*06:127:02	06:127:02	HLA12318		0	0	0	0	0	0	1	1									
C*06:128N	06:128N	HLA11050		1	0	0	0	1	2	0	4									
C*06:129	06:129	HLA11438		0	0	1	0	0	0	0	1									
C*06:131	06:131	HLA11457		0	1	0	0	0	0	0	1									
**C*06:132 total**	**06:132 total**			**0**	**0**	**2**	**0**	**0**	**0**	**2**	**4**									
C*06:132:01	06:132:01	HLA11577		0	0	2	0	0	0	2	4									
C*06:133	06:133	HLA12020		0	0	0	0	0	0	1	1									
C*06:136	06:136	HLA12096		0	0	1	0	0	0	0	1									
C*06:138	06:138	HLA12317		2	0	4	0	0	0	0	6								WD	WD
C*06:139	06:139	HLA12320		0	0	0	0	1	0	0	1									
C*06:140	06:140	HLA12321		0	0	3	0	0	0	0	3									
C*06:142	06:142	HLA12498		0	1	0	0	0	0	0	1									

Supplemental Table 10: HLA-C Allele Summary [a]				Allele Count by Population Group [b]								3.0.0 CIWD Category by Population Group [c]								
Allele	Genomic typing	Allele ID	G group	AFA	API	EURO	MENA	HIS	NAM	UNK	Total	AFA	API	EURO	MENA	HIS	NAM	UNK	Total	Highest Frequency
C*06:144	06:144	HLA12656		0	0	1	0	0	0	0	1									
C*06:148	06:148	HLA12696		0	0	3	0	0	0	0	3									
C*06:150	06:150	HLA13031		2	0	0	0	0	0	0	2									
C*06:151	06:151	HLA13032		0	0	1	0	0	0	0	1									
C*06:152N	06:152N	HLA13033		0	4	0	0	0	0	0	4									
**C*06:155 total**	**06:155 total**			**0**	**0**	**0**	**0**	**1**	**0**	**3**	**4**									
**C*06:155:01G total**	**06:155:01G total**			**0**	**0**	**0**	**0**	**1**	**0**	**3**	**4**									
C*06:155:01	06:155:01		06:155:01G	0	0	0	0	1	0	3	4									
C*06:158	06:158	HLA13551		0	0	1	0	0	0	0	1									
C*06:162	06:162	HLA13666		0	1	0	0	0	0	0	1									
C*06:164	06:164	HLA13746		0	2	0	0	0	0	0	2									
C*06:174	06:174	HLA14738		0	0	1	0	0	0	0	1									
C*06:177	06:177	HLA15036		0	1	0	0	0	0	0	1									
C*06:185	06:185	HLA15207		0	0	1	0	0	0	0	1									
C*06:189	06:189	HLA15423		0	1	0	0	0	0	0	1									
C*06:203	06:203	HLA17504		0	1	0	0	0	0	0	1									
C*06:CODE	06:CODE			4287	4955	122747	2299	6062	736	15249	156335	NA	NA	NA	NA	NA	NA	NA	NA	NA
**C*07:01 total**	**07:01 total**			**41415**	**89717**	**1523480**	**39220**	**60391**	**6244**	**143555**	**1904022**	**C**	**C**	**C**	**C**	**C**	**C**	**C**	**C**	**C**
C*07:01	07:01			20	6	8003	31	24	0	353	8437	WD	WD	C	WD	I		C	C	C
C*07:01P	07:01P			11	0	3153	16	5	0	14	3199	WD		C	WD	WD		I	C	C
**C*07:01:01G total**	**07:01:01G total**			**41326**	**89655**	**1511817**	**39158**	**60342**	**6242**	**143089**	**1891629**	**C**	**C**	**C**	**C**	**C**	**C**	**C**	**C**	**C**
C*07:01:01G	07:01:01G		07:01:01G	33952	84827	1434796	38057	45216	4655	132142	1773645	C	C	C	C	C	C	C	C	C
C*07:01:01	07:01:01		07:01:01G	1070	310	23957	245	3745	294	2853	32474	C	C	C	C	C	C	C	C	C
C*07:01:01:01	07:01:01:01	HLA00433	07:01:01G	2444	540	47295	480	8058	724	6145	65686	C	C	C	C	C	C	C	C	C
C*07:01:01:02	07:01:01:02	HLA07937	07:01:01G	1	0	26	0	0	0	4	31			WD					WD	WD
C*07:01:01:04	07:01:01:04	HLA14091	07:01:01G	5	185	18	1	5	1	25	240	WD	C	WD		WD		I	I	C
C*07:01:01:05	07:01:01:05	HLA14810	07:01:01G	0	0	3	0	0	0	0	3									
C*07:01:01:06	07:01:01:06	HLA15767	07:01:01G	1	0	123	0	5	2	12	143			I		WD		WD	WD	I
C*07:01:01:08	07:01:01:08	HLA16181	07:01:01G	0	0	5	0	0	0	0	5			WD					WD	WD
C*07:01:01:09	07:01:01:09	HLA16650	07:01:01G	4	1	20	3	23	2	10	63			WD		I		WD	WD	I
C*07:01:01:15	07:01:01:15	HLA16818	07:01:01G	0	0	2	0	0	0	0	2									

Supplemental Table 10: HLA-C Allele Summary [a]				Allele Count by Population Group [b]								3.0.0 CIWD Category by Population Group [c]								
Allele	Genomic typing	Allele ID	G group	AFA	API	EURO	MENA	HIS	NAM	UNK	Total	AFA	API	EURO	MENA	HIS	NAM	UNK	Total	Highest Frequency
C*07:01:01:17	07:01:01:17	HLA16868	07:01:01G	0	0	1	0	0	0	0	1									
C*07:01:01:18	07:01:01:18	HLA17058	07:01:01G	0	0	2	0	1	0	1	4									
C*07:01:01:19	07:01:01:19	HLA17978	07:01:01G	0	0	0	0	0	0	1	1									
C*07:01:02	07:01:02	HLA00992	07:01:01G	1164	519	2400	219	1583	271	671	6827	C	C	C	C	C	C	C	C	C
C*07:01:09	07:01:09	HLA03015	07:01:01G	0	0	113	0	40	0	14	167			WD		I		I	I	I
**C*07:06 total**	**07:06 total**			**298**	**3226**	**269**	**50**	**295**	**81**	**328**	**4547**	**C**	**C**	**I**	**C**	**C**	**C**	**C**	**C**	**C**
C*07:06	07:06		07:01:01G	298	3226	269	50	295	81	328	4547	C	C	I	C	C	C	C	C	C
C*07:18	07:18	HLA01672	07:01:01G	2387	47	2783	103	1371	212	881	7784	C	I	C	C	C	C	C	C	C
C*07:52	07:52	HLA03135	07:01:01G	0	0	1	0	0	0	2	3									
C*07:337	07:337	HLA10114	07:01:01G	0	0	1	0	0	0	0	1									
C*07:419	07:419	HLA13202	07:01:01G	0	0	2	0	0	0	0	2									
C*07:01:03	07:01:03	HLA01865		42	0	3	0	13	1	14	73	C				I		I	WD	C
C*07:01:04	07:01:04	HLA02293		0	0	14	0	1	0	1	16			WD					WD	WD
C*07:01:05	07:01:05	HLA02422		11	4	56	5	0	0	5	81	WD		WD	WD			WD	WD	WD
C*07:01:06	07:01:06	HLA02523		0	0	1	0	0	0	0	1									
C*07:01:07	07:01:07	HLA02611		1	0	92	0	0	0	3	96			WD					WD	WD
C*07:01:08	07:01:08	HLA03012		0	0	242	0	4	1	63	310			I				I	I	I
C*07:01:10	07:01:10	HLA04325		0	0	26	0	0	0	0	26			WD					WD	WD
C*07:01:12	07:01:12	HLA04833		0	2	4	6	0	0	0	12				WD				WD	WD
C*07:01:14	07:01:14	HLA05603		0	1	0	0	0	0	0	1									
C*07:01:15	07:01:15	HLA05626		0	0	8	0	0	0	0	8			WD					WD	WD
C*07:01:16	07:01:16	HLA05740		0	0	1	0	0	0	0	1									
C*07:01:17	07:01:17	HLA06390		0	0	1	0	0	0	0	1									
C*07:01:18	07:01:18	HLA06424		0	31	0	0	0	0	0	31		I						WD	I
**C*07:01:20G total**	**07:01:20G total**			**0**	**0**	**3**	**0**	**0**	**0**	**0**	**3**									
C*07:01:20G	07:01:20G		07:01:20G	0	0	3	0	0	0	0	3									
C*07:01:23	07:01:23	HLA07074		0	13	8	0	0	0	1	22		I	WD					WD	I
C*07:01:24	07:01:24	HLA07080		0	0	3	0	0	0	2	5								WD	WD
C*07:01:25	07:01:25	HLA07845		0	0	4	0	0	0	1	5								WD	WD
C*07:01:26	07:01:26	HLA07846		0	0	4	0	0	0	0	4									
C*07:01:28	07:01:28	HLA08442		0	0	5	0	0	0	0	5			WD					WD	WD

Supplemental Table 10: HLA-C Allele Summary [a]				Allele Count by Population Group [b]								3.0.0 CIWD Category by Population Group [c]								
Allele	Genomic typing	Allele ID	G group	AFA	API	EURO	MENA	HIS	NAM	UNK	Total	AFA	API	EURO	MENA	HIS	NAM	UNK	Total	Highest Frequency
C*07:01:29	07:01:29	HLA08709		0	0	1	0	0	0	1	2									
C*07:01:30	07:01:30	HLA08711		0	0	4	0	0	0	1	5								WD	WD
C*07:01:31	07:01:31	HLA08713		4	0	1	2	0	0	0	7								WD	WD
C*07:01:32	07:01:32	HLA09020		0	0	1	0	0	0	0	1									
C*07:01:34	07:01:34	HLA09607		0	0	3	0	0	0	1	4									
C*07:01:35	07:01:35	HLA10065		0	0	0	0	0	0	1	1									
C*07:01:36	07:01:36	HLA10066		0	3	1	0	0	0	0	4									
C*07:01:38	07:01:38	HLA10428		0	0	0	1	0	0	0	1									
C*07:01:40	07:01:40	HLA11051		0	0	0	0	0	0	1	1									
C*07:01:42	07:01:42	HLA11256		0	0	0	0	1	0	3	4									
C*07:01:43	07:01:43	HLA11460		0	0	4	0	0	0	0	4									
C*07:01:44	07:01:44	HLA11776		0	0	4	0	0	0	0	4									
C*07:01:45	07:01:45	HLA12088		0	0	1	1	0	0	0	2									
C*07:01:46	07:01:46	HLA12500		0	0	1	0	0	0	0	1									
C*07:01:47	07:01:47	HLA12705		0	0	5	0	0	0	0	5			WD					WD	WD
C*07:01:48	07:01:48	HLA13038		0	1	0	0	1	0	0	2									
C*07:01:49	07:01:49	HLA13612		0	0	2	0	0	0	0	2									
C*07:01:50	07:01:50	HLA13699		0	0	0	0	0	0	1	1									
C*07:01:51	07:01:51	HLA13706		0	0	1	0	0	0	0	1									
C*07:01:53	07:01:53	HLA14644		0	0	2	0	0	0	0	2									
C*07:01:57	07:01:57	HLA15885		0	1	0	0	0	0	0	1									
C*07:453	07:453	HLA13758		0	0	1	0	0	0	0	1									
**C*07:02 total**	**07:02 total**			**22061**	**158872**	**1344851**	**27801**	**75361**	**6466**	**132296**	**1767708**	**C**	**C**	**C**	**C**	**C**	**C**	**C**	**C**	**C**
C*07:02	07:02			25	97	4971	11	282	21	255	5662	WD	I	C	WD	C	C	C	C	C
C*07:02P	07:02P			1	13	986	3	8	0	6	1017		I	I		I		WD	I	I
**C*07:02:01G total**	**07:02:01G total**			**22025**	**158704**	**1337829**	**27779**	**75017**	**6444**	**131990**	**1759788**	**C**	**C**	**C**	**C**	**C**	**C**	**C**	**C**	**C**
C*07:02:01G	07:02:01G		07:02:01G	16595	141312	1260604	26972	50482	4488	115812	1616265	C	C	C	C	C	C	C	C	C
C*07:02:01	07:02:01		07:02:01G	2285	7870	33314	407	11211	847	8412	64346	C	C	C	C	C	C	C	C	C
C*07:02:01:01	07:02:01:01	HLA00434	07:02:01G	2048	7674	3558	197	7579	633	2505	24194	C	C	C	C	C	C	C	C	C
C*07:02:01:03	07:02:01:03	HLA01902	07:02:01G	1097	1755	40334	203	5692	473	5244	54798	C	C	C	C	C	C	C	C	C
C*07:02:01:05	07:02:01:05	HLA07778	07:02:01G	0	28	0	0	0	0	2	30		I						WD	I

Supplemental Table 10: HLA-C Allele Summary [a]				Allele Count by Population Group [b]								3.0.0 CIWD Category by Population Group [c]								
Allele	Genomic typing	Allele ID	G group	AFA	API	EURO	MENA	HIS	NAM	UNK	Total	AFA	API	EURO	MENA	HIS	NAM	UNK	Total	Highest Frequency
C*07:02:01:07	07:02:01:07	HLA15122	07:02:01G	0	0	1	0	0	0	0	1									
C*07:02:01:10	07:02:01:10	HLA16177	07:02:01G	0	0	1	0	0	0	0	1									
C*07:02:01:12	07:02:01:12	HLA16179	07:02:01G	0	0	2	0	0	0	0	2									
C*07:02:01:13	07:02:01:13	HLA16180	07:02:01G	0	0	0	0	0	0	1	1									
C*07:02:01:16	07:02:01:16	HLA17053	07:02:01G	0	0	2	0	0	0	0	2									
C*07:02:23	07:02:23	HLA06042	07:02:01G	0	0	2	0	0	1	0	3									
C*07:02:53	07:02:53	HLA10612	07:02:01G	0	4	0	0	0	0	1	5								WD	WD
C*07:02:60	07:02:60	HLA11570	07:02:01G	0	1	0	0	0	0	0	1									
C*07:02:70	07:02:70	HLA14377	07:02:01G	0	1	0	0	0	0	0	1									
C*07:02:79	07:02:79	HLA16731	07:02:01G	0	0	1	0	0	0	0	1									
C*07:50	07:50	HLA03004	07:02:01G	0	0	2	0	0	0	0	2									
C*07:66	07:66	HLA03631	07:02:01G	0	41	0	0	0	0	3	44		I						WD	I
C*07:74	07:74	HLA03977	07:02:01G	0	1	4	0	52	2	3	62					I			WD	I
C*07:160	07:160	HLA05889	07:02:01G	0	0	0	0	0	0	1	1									
C*07:308	07:308	HLA09511	07:02:01G	0	0	3	0	0	0	0	3									
C*07:359	07:359	HLA11071	07:02:01G	0	17	1	0	1	0	5	24		I					WD	WD	I
C*07:500	07:500	HLA14897	07:02:01G	0	0	0	0	0	0	1	1									
C*07:02:02	07:02:02	HLA03232		0	4	2	0	1	0	0	7								WD	WD
C*07:02:03	07:02:03	HLA03459		0	0	3	0	0	0	0	3									
C*07:02:04	07:02:04	HLA03638		0	4	12	2	0	0	1	19			WD					WD	WD
C*07:02:06	07:02:06	HLA03976		0	16	1	0	0	0	1	18		I						WD	I
C*07:02:07	07:02:07	HLA04314		0	0	11	0	0	0	0	11			WD					WD	WD
C*07:02:08	07:02:08	HLA04315		1	0	148	0	3	0	10	162			I				WD	I	I
C*07:02:09	07:02:09	HLA04317		0	1	0	0	0	0	0	1									
C*07:02:10	07:02:10	HLA04320		0	0	632	0	0	0	5	637			I				WD	I	I
C*07:02:11	07:02:11	HLA04344		4	1	37	1	1	0	4	48			WD					WD	WD
C*07:02:12	07:02:12	HLA04612		0	1	1	0	0	0	0	2									
C*07:02:13	07:02:13	HLA04630		0	12	0	0	0	0	2	14		WD						WD	WD
C*07:02:14	07:02:14	HLA04824		0	0	11	0	0	0	0	11			WD					WD	WD
C*07:02:16	07:02:16	HLA05190		3	0	26	0	2	0	7	38			WD				WD	WD	WD
C*07:02:18	07:02:18	HLA05580		0	0	76	0	0	0	0	76			WD					WD	WD

Supplemental Table 10: HLA-C Allele Summary [a]				Allele Count by Population Group [b]								3.0.0 CIWD Category by Population Group [c]								
Allele	Genomic typing	Allele ID	G group	AFA	API	EURO	MENA	HIS	NAM	UNK	Total	AFA	API	EURO	MENA	HIS	NAM	UNK	Total	Highest Frequency
C*07:02:20	07:02:20	HLA05732		1	0	5	0	47	0	8	61			WD		I		WD	WD	I
C*07:02:22	07:02:22	HLA05937		0	0	2	0	0	0	0	2									
C*07:02:24	07:02:24	HLA06381		0	0	25	0	0	0	0	25			WD					WD	WD
C*07:02:27	07:02:27	HLA07047		0	0	2	0	0	0	0	2									
C*07:02:28	07:02:28	HLA07060		0	0	24	0	0	0	2	26			WD					WD	WD
C*07:02:30	07:02:30	HLA07731		0	0	1	0	0	0	0	1									
C*07:02:31	07:02:31	HLA07828		0	0	2	0	0	0	0	2									
C*07:02:32	07:02:32	HLA07841		0	0	11	0	0	0	0	11			WD					WD	WD
C*07:02:35	07:02:35	HLA08194		0	0	2	0	0	0	0	2									
C*07:02:36	07:02:36	HLA08196		0	5	3	2	0	0	0	10		WD						WD	WD
C*07:02:37	07:02:37	HLA08721		0	2	0	0	0	0	0	2									
C*07:02:39	07:02:39	HLA08727		0	0	5	0	0	0	0	5			WD					WD	WD
C*07:02:40	07:02:40	HLA09177		0	0	4	0	0	0	0	4									
C*07:02:41	07:02:41	HLA09186		0	0	4	0	0	0	0	4									
C*07:02:42	07:02:42	HLA09604		0	0	0	1	0	0	0	1									
C*07:02:44	07:02:44	HLA09993		0	2	0	0	0	0	0	2									
C*07:02:46	07:02:46	HLA10003		0	1	0	0	0	0	0	1									
C*07:02:49	07:02:49	HLA10009		0	3	0	0	0	0	0	3									
C*07:02:52	07:02:52	HLA10429		0	0	1	2	0	0	0	3									
C*07:02:54	07:02:54	HLA11043		1	0	2	0	0	1	1	5								WD	WD
C*07:02:55	07:02:55	HLA11089		0	6	2	0	0	0	1	9		WD						WD	WD
C*07:02:58	07:02:58	HLA11440		0	0	2	0	0	0	0	2									
C*07:02:62	07:02:62	HLA11855		0	0	2	0	0	0	2	4									
C*07:02:65	07:02:65	HLA12501		0	0	2	0	0	0	1	3									
C*07:02:67	07:02:67	HLA12723		0	0	1	0	0	0	0	1									
C*07:02:69	07:02:69	HLA13522		0	0	1	0	0	0	0	1									
C*07:02:74	07:02:74	HLA15209		0	0	1	0	0	0	0	1									
C*07:02:76	07:02:76	HLA15219		0	0	1	0	0	0	0	1									
**C*07:04 total**	**07:04 total**			**2968**	**22295**	**220635**	**4046**	**4877**	**599**	**16842**	**272262**	**C**	**C**	**C**	**C**	**C**	**C**	**C**	**C**	**C**
C*07:04	07:04			1	3	858	2	4	0	23	891			I				I	I	I
C*07:04P	07:04P			0	0	183	0	1	0	0	184			I					I	I

Supplemental Table 10: HLA-C Allele Summary [a]				Allele Count by Population Group [b]								3.0.0 CIWD Category by Population Group [c]								
**Allele**	**Genomic typing**	**Allele ID**	**G group**	**AFA**	**API**	**EURO**	**MENA**	**HIS**	**NAM**	**UNK**	**Total**	**AFA**	**API**	**EURO**	**MENA**	**HIS**	**NAM**	**UNK**	**Total**	**Highest Frequency**
**C*07:04:01G total**	**07:04:01G total**			**2967**	**22276**	**219555**	**4044**	**4871**	**599**	**16818**	**271130**	**C**	**C**	**C**	**C**	**C**	**C**	**C**	**C**	**C**
C*07:04:01G	07:04:01G		07:04:01G	2360	20278	210218	3958	3429	462	15126	255831	C	C	C	C	C	C	C	C	C
C*07:04:01	07:04:01		07:04:01G	413	1476	6074	55	1020	95	1264	10397	C	C	C	C	C	C	C	C	C
C*07:04:01:01	07:04:01:01	HLA00436	07:04:01G	194	522	3263	27	422	42	428	4898	C	C	C	WD	C	C	C	C	C
C*07:04:01:03	07:04:01:03	HLA16823	07:04:01G	0	0	0	4	0	0	0	4									
**C*07:04:02G total**	**07:04:02G total**			**0**	**0**	**6**	**0**	**0**	**0**	**0**	**6**			**WD**					**WD**	**WD**
C*07:04:02	07:04:02		07:04:02G	0	0	6	0	0	0	0	6			WD					WD	WD
C*07:04:03	07:04:03	HLA03538		0	0	0	0	1	0	0	1									
C*07:04:04	07:04:04	HLA05352		0	15	0	0	0	0	0	15		I						WD	I
C*07:04:05	07:04:05	HLA06444		0	0	2	0	0	0	0	2									
C*07:04:06	07:04:06	HLA06824		0	0	26	0	0	0	1	27			WD					WD	WD
C*07:04:08	07:04:08	HLA08184		0	0	1	0	0	0	0	1									
C*07:04:09	07:04:09	HLA10425		0	0	1	0	0	0	0	1									
C*07:04:10	07:04:10	HLA12713		0	0	2	0	0	0	0	2									
C*07:04:11	07:04:11	HLA15120		0	1	1	0	0	0	0	2									
C*07:05	07:05	HLA00437		228	1	26	33	23	5	54	370	C		WD	WD	I	WD	I	I	C
C*07:07	07:07	HLA00439		1	2	374	20	61	1	73	532			I	WD	I		I	I	I
C*07:08	07:08	HLA00440		0	0	1	0	0	0	0	1									
C*07:09	07:09	HLA00441		0	3	2	2	0	0	0	7								WD	WD
C*07:10	07:10	HLA00442		0	1	391	0	4	0	25	421			I				I	I	I
C*07:12	07:12	HLA00444		1	1	438	0	3	0	28	471			I				I	I	I
C*07:13	07:13	HLA01079		0	88	33	6	0	0	6	133		I	WD	WD			WD	WD	I
C*07:15	07:15	HLA01408		1	4	146	0	0	0	4	155			I					WD	I
C*07:16	07:16	HLA01588		4	0	48	2	0	0	15	69			WD				I	WD	I
**C*07:17 total**	**07:17 total**			**6**	**3**	**174**	**0**	**192**	**26**	**35**	**436**	**WD**		**I**		**C**	**C**	**I**	**I**	**C**
C*07:17	07:17			0	1	37	0	58	7	13	116			WD		I	C	WD	WD	C
C*07:17:01	07:17:01	HLA01662		6	2	135	0	134	19	22	318	WD		I		C	C	I	I	C
C*07:17:02	07:17:02	HLA08720		0	0	2	0	0	0	0	2									
C*07:19	07:19	HLA01720		43	3	306	62	28	0	22	464	C		I	C	I		I	I	C
C*07:21	07:21	HLA01833		2	0	109	0	2	1	25	139			WD				I	WD	I
C*07:22	07:22	HLA01844		1	0	445	3	4	0	15	468			I				I	I	I

Supplemental Table 10: HLA-C Allele Summary [a]				Allele Count by Population Group [b]								3.0.0 CIWD Category by Population Group [c]								
Allele	Genomic typing	Allele ID	G group	AFA	API	EURO	MENA	HIS	NAM	UNK	Total	AFA	API	EURO	MENA	HIS	NAM	UNK	Total	Highest Frequency
C*07:24	07:24	HLA01864		2	2	62	0	0	0	13	79			WD				WD	WD	WD
C*07:25	07:25	HLA01877		0	4	186	0	2	1	17	210			I				I	I	I
**C*07:26 total**	**07:26 total**			**8**	**4280**	**28**	**31**	**6**	**13**	**46**	**4412**	**WD**	**C**	**WD**	**WD**	**WD**	**C**	**I**	**C**	**C**
C*07:26	07:26			7	2061	14	27	5	10	30	2154	WD	C	WD	WD	WD	C	I	C	C
C*07:26:01	07:26:01	HLA01889		1	2218	14	4	1	3	16	2257		C	WD				I	C	C
C*07:26:02	07:26:02	HLA14534		0	1	0	0	0	0	0	1									
**C*07:27 total**	**07:27 total**			**3**	**157**	**203**	**60**	**8**	**1**	**77**	**509**		**C**	**I**	**C**	**I**		**I**	**I**	**C**
C*07:27	07:27			0	5	54	0	1	0	7	67		WD	WD				WD	WD	WD
C*07:27:01	07:27:01	HLA01908		3	141	107	60	7	1	69	388		C	WD	C	I		I	I	C
C*07:27:02	07:27:02	HLA03107		0	11	42	0	0	0	1	54		WD	WD					WD	WD
C*07:28	07:28	HLA01961		19	0	5	2	0	0	4	30	WD		WD					WD	WD
**C*07:29 total**	**07:29 total**			**1**	**479**	**45**	**3**	**0**	**1**	**15**	**544**		**C**	**WD**				**I**	**I**	**C**
C*07:29	07:29			0	280	27	2	0	1	11	321		C	WD				WD	I	C
C*07:29P	07:29P			0	0	1	0	0	0	0	1									
C*07:29:01	07:29:01	HLA02010		1	199	16	1	0	0	4	221		C	WD					I	C
C*07:29:02	07:29:02	HLA14023		0	0	1	0	0	0	0	1									
**C*07:31 total**	**07:31 total**			**0**	**0**	**20**	**0**	**0**	**0**	**1**	**21**			**WD**					**WD**	**WD**
C*07:31	07:31			0	0	2	0	0	0	0	2									
C*07:31:01	07:31:01	HLA02205		0	0	14	0	0	0	1	15			WD					WD	WD
C*07:31:02	07:31:02	HLA08529		0	0	4	0	0	0	0	4									
C*07:32N	07:32N	HLA02354		0	0	87	0	1	0	9	97			WD				WD	WD	WD
C*07:33N	07:33N	HLA02380		0	0	14	0	0	0	3	17			WD					WD	WD
C*07:35	07:35	HLA02425		71	0	1	0	12	3	15	102	C				I		I	WD	C
C*07:36	07:36	HLA02448		0	0	194	11	3	0	4	212			I	WD				I	I
C*07:37	07:37	HLA02459		0	1	12	0	1	0	3	17			WD					WD	WD
**C*07:38 total**	**07:38 total**			**0**	**0**	**37**	**0**	**0**	**0**	**4**	**41**			**WD**					**WD**	**WD**
C*07:38	07:38			0	0	6	0	0	0	1	7			WD					WD	WD
C*07:38:01	07:38:01	HLA02475		0	0	31	0	0	0	3	34			WD					WD	WD
C*07:39	07:39	HLA02564		0	0	6	0	0	0	0	6			WD					WD	WD
C*07:40	07:40	HLA02598		1	1	281	2	4	0	8	297			I				WD	I	I
C*07:41	07:41	HLA02614		0	0	3	0	0	0	0	3									

Supplemental Table 10: HLA-C Allele Summary [a]				Allele Count by Population Group [b]								3.0.0 CIWD Category by Population Group [c]								
**Allele**	**Genomic typing**	**Allele ID**	**G group**	**AFA**	**API**	**EURO**	**MENA**	**HIS**	**NAM**	**UNK**	**Total**	**AFA**	**API**	**EURO**	**MENA**	**HIS**	**NAM**	**UNK**	**Total**	**Highest Frequency**
**C*07:43 total**	**07:43 total**			**0**	**31**	**3**	**0**	**0**	**0**	**4**	**38**		**I**						**WD**	**I**
C*07:43	07:43			0	30	3	0	0	0	4	37		I						WD	I
C*07:43:01	07:43:01	HLA02697		0	1	0	0	0	0	0	1									
C*07:44	07:44	HLA02709		0	0	5	0	0	0	2	7			WD					WD	WD
C*07:45	07:45	HLA02730		0	0	2	0	0	0	0	2									
C*07:46	07:46	HLA02789		2	3	649	2	3	0	13	672			I				WD	I	I
C*07:48	07:48	HLA02835		0	19	0	0	0	0	0	19		I						WD	I
C*07:49	07:49	HLA02943		0	1	48	1	0	1	1	52			WD					WD	WD
C*07:51	07:51	HLA03013		1	6	76	3	3	0	12	101		WD	WD				WD	WD	WD
C*07:53	07:53	HLA03139		0	0	3	0	0	0	0	3									
C*07:54	07:54	HLA03239		0	0	24	0	0	0	1	25			WD					WD	WD
C*07:55N	07:55N	HLA03298		0	0	33	0	0	0	1	34			WD					WD	WD
**C*07:56 total**	**07:56 total**			**1**	**32**	**3**	**0**	**0**	**0**	**2**	**38**		**I**						**WD**	**I**
C*07:56	07:56			0	2	1	0	0	0	1	4									
C*07:56:01	07:56:01	HLA03310		0	0	2	0	0	0	1	3									
C*07:56:02	07:56:02	HLA03753		1	30	0	0	0	0	0	31		I						WD	I
C*07:57	07:57	HLA03365		0	0	16	2	1	0	0	19			WD					WD	WD
C*07:60	07:60	HLA03436		0	0	215	2	1	0	4	222			I					I	I
C*07:61N	07:61N	HLA03439		6	0	1	0	3	4	4	18	WD							WD	WD
C*07:63	07:63	HLA03477		0	16	0	0	0	0	2	18		I						WD	I
C*07:65	07:65	HLA03546		1	0	2	0	0	0	0	3									
C*07:67	07:67	HLA03623		0	3	0	0	0	0	0	3									
C*07:68	07:68	HLA03698		0	1	467	0	1	0	5	474			I				WD	I	I
C*07:70	07:70	HLA03701		1	2	11	4	0	0	3	21			WD					WD	WD
C*07:71	07:71	HLA03702		2	0	0	0	0	0	0	2									
C*07:72	07:72	HLA03703		0	0	28	0	0	0	7	35			WD				WD	WD	WD
C*07:73	07:73	HLA03819		0	0	7	0	0	0	1	8			WD					WD	WD
C*07:75	07:75	HLA04021		0	1	8	0	0	0	0	9			WD					WD	WD
**C*07:76 total**	**07:76 total**			**0**	**1**	**13**	**4**	**1**	**0**	**0**	**19**			**WD**					**WD**	**WD**
C*07:76	07:76			0	0	1	3	1	0	0	5								WD	WD
C*07:76:01	07:76:01	HLA04130		0	1	5	1	0	0	0	7			WD					WD	WD

Supplemental Table 10: HLA-C Allele Summary [a]				Allele Count by Population Group [b]								3.0.0 CIWD Category by Population Group [c]								
Allele	Genomic typing	Allele ID	G group	AFA	API	EURO	MENA	HIS	NAM	UNK	Total	AFA	API	EURO	MENA	HIS	NAM	UNK	Total	Highest Frequency
C*07:76:02	07:76:02	HLA11047		0	0	7	0	0	0	0	7			WD					WD	WD
C*07:77	07:77	HLA04318		0	0	1	3	0	0	0	4									
**C*07:78 total**	**07:78 total**			**0**	**0**	**2**	**0**	**0**	**0**	**0**	**2**									
C*07:78:02	07:78:02	HLA13517		0	0	2	0	0	0	0	2									
C*07:79	07:79	HLA04323		0	0	26	1	0	0	2	29			WD					WD	WD
C*07:80	07:80	HLA04324		0	0	19	6	5	0	9	39			WD	WD	WD		WD	WD	WD
C*07:81	07:81	HLA04328		0	0	2	2	0	0	0	4									
C*07:82	07:82	HLA04329		0	0	3	1	0	0	0	4									
C*07:83	07:83	HLA04330		0	0	9	0	0	0	0	9			WD					WD	WD
C*07:85	07:85	HLA04335		0	0	23	0	1	0	2	26			WD					WD	WD
C*07:87	07:87	HLA04339		0	0	1	0	0	0	0	1									
C*07:88	07:88	HLA04342		0	0	5	0	0	0	0	5			WD					WD	WD
C*07:89	07:89	HLA04343		0	2	4	0	0	0	0	6								WD	WD
C*07:90	07:90	HLA04347		0	0	9	0	0	1	2	12			WD					WD	WD
C*07:91	07:91	HLA04348		0	0	41	0	0	0	0	41			WD					WD	WD
C*07:92	07:92	HLA04396		0	0	17	0	0	0	3	20			WD					WD	WD
C*07:93	07:93	HLA04399		0	0	58	31	1	0	10	100			WD	WD			WD	WD	WD
C*07:95	07:95	HLA04594		0	0	34	0	1	0	2	37			WD					WD	WD
**C*07:96 total**	**07:96 total**			**0**	**0**	**23**	**0**	**0**	**0**	**1**	**24**			**WD**					**WD**	**WD**
C*07:96	07:96			0	0	5	0	0	0	0	5			WD					WD	WD
C*07:96:01	07:96:01	HLA04597		0	0	18	0	0	0	1	19			WD					WD	WD
C*07:97	07:97	HLA04601		0	0	7	0	0	0	0	7			WD					WD	WD
C*07:98N	07:98N	HLA04607		0	0	1	0	0	0	0	1									
C*07:99	07:99	HLA04609		0	0	0	0	2	0	0	2									
C*07:100	07:100	HLA04974		0	0	9	0	0	0	0	9			WD					WD	WD
C*07:101	07:101	HLA04982		0	0	42	0	0	0	1	43			WD					WD	WD
C*07:102	07:102	HLA04983		0	0	5	0	0	0	0	5			WD					WD	WD
C*07:103	07:103	HLA04995		0	0	1	0	0	0	0	1									
C*07:104N	07:104N	HLA04996		0	0	25	0	1	0	3	29			WD					WD	WD
C*07:105	07:105	HLA05012		0	0	6	0	0	0	0	6			WD					WD	WD
C*07:106	07:106	HLA05013		0	0	34	0	0	0	1	35			WD					WD	WD

Supplemental Table 10: HLA-C Allele Summary [a]				Allele Count by Population Group [b]								3.0.0 CIWD Category by Population Group [c]								
Allele	Genomic typing	Allele ID	G group	AFA	API	EURO	MENA	HIS	NAM	UNK	Total	AFA	API	EURO	MENA	HIS	NAM	UNK	Total	Highest Frequency
**C*07:108 total**	**07:108 total**			**0**	**0**	**10**	**0**	**0**	**0**	**1**	**11**			**WD**					**WD**	**WD**
C*07:108:01	07:108:01	HLA05015		0	0	6	0	0	0	0	6			WD					WD	WD
C*07:108:02	07:108:02	HLA10902		0	0	4	0	0	0	1	5								WD	WD
**C*07:109 total**	**07:109 total**			**0**	**0**	**2**	**0**	**0**	**0**	**0**	**2**									
C*07:109	07:109			0	0	2	0	0	0	0	2									
C*07:110	07:110	HLA05017		0	0	1	1	0	0	0	2									
C*07:111	07:111	HLA05022		0	0	0	0	0	0	1	1									
C*07:112	07:112	HLA05023		5	0	0	0	1	0	3	9	WD							WD	WD
C*07:113	07:113	HLA05024		0	0	1	0	0	0	0	1									
C*07:114	07:114	HLA05025		0	3	6	0	0	0	0	9			WD					WD	WD
C*07:116	07:116	HLA05027		0	0	8	0	0	0	0	8			WD					WD	WD
C*07:117	07:117	HLA05028		0	0	101	0	0	0	0	101			WD					WD	WD
C*07:118	07:118	HLA05029		0	0	12	0	0	0	0	12			WD					WD	WD
C*07:119	07:119	HLA05030		0	0	26	2	2	0	10	40			WD				WD	WD	WD
C*07:120	07:120	HLA05031		0	0	41	0	0	0	0	41			WD					WD	WD
C*07:121Q	07:121Q	HLA05032		0	0	7	0	0	0	0	7			WD					WD	WD
C*07:122	07:122	HLA05033		0	0	6	0	0	0	0	6			WD					WD	WD
C*07:123	07:123	HLA05034		0	0	3	0	1	0	1	5								WD	WD
C*07:124	07:124	HLA05035		0	0	19	0	0	0	13	32			WD				WD	WD	WD
C*07:125	07:125	HLA05036		0	0	1	0	0	0	0	1									
C*07:126	07:126	HLA05064		0	0	9	0	0	0	0	9			WD					WD	WD
C*07:127	07:127	HLA05065		0	0	28	0	0	0	0	28			WD					WD	WD
C*07:128	07:128	HLA05066		0	0	3	0	1	0	1	5								WD	WD
C*07:129	07:129	HLA05067		0	0	1	0	1	0	0	2									
C*07:130	07:130	HLA05068		0	5	6	0	0	0	0	11		WD	WD					WD	WD
**C*07:131 total**	**07:131 total**			**0**	**0**	**2**	**0**	**0**	**0**	**0**	**2**									
C*07:131	07:131			0	0	2	0	0	0	0	2									
C*07:132	07:132	HLA05070		0	0	2	0	0	0	0	2									
C*07:133	07:133	HLA05071		0	0	4	0	0	0	0	4									
C*07:134	07:134	HLA05072		0	0	3	0	0	0	0	3									
C*07:135	07:135	HLA05073		0	0	1	0	0	0	0	1									

Supplemental Table 10: HLA-C Allele Summary [a]				Allele Count by Population Group [b]								3.0.0 CIWD Category by Population Group [c]								
**Allele**	**Genomic typing**	**Allele ID**	**G group**	**AFA**	**API**	**EURO**	**MENA**	**HIS**	**NAM**	**UNK**	**Total**	**AFA**	**API**	**EURO**	**MENA**	**HIS**	**NAM**	**UNK**	**Total**	**Highest Frequency**
**C*07:137 total**	**07:137 total**			**0**	**0**	**36**	**0**	**3**	**0**	**0**	**39**			**WD**					**WD**	**WD**
C*07:137	07:137			0	0	1	0	0	0	0	1									
C*07:137:01	07:137:01	HLA05075		0	0	1	0	0	0	0	1									
C*07:137:02	07:137:02	HLA05855		0	0	34	0	3	0	0	37			WD					WD	WD
C*07:138	07:138	HLA05076		0	0	17	0	2	0	1	20			WD					WD	WD
C*07:139	07:139	HLA05077		0	0	3	0	0	0	0	3									
C*07:140	07:140	HLA05089		0	0	2	0	0	0	0	2									
**C*07:141 total**	**07:141 total**			**2**	**0**	**4**	**2**	**1**	**0**	**1**	**10**								**WD**	**WD**
C*07:141:01	07:141:01	HLA05090		1	0	0	0	0	0	0	1									
C*07:141:02	07:141:02	HLA07835		1	0	4	2	1	0	1	9								WD	WD
C*07:142	07:142	HLA05079		0	0	2	0	0	0	0	2									
C*07:143	07:143	HLA05080		0	0	10	0	0	0	0	10			WD					WD	WD
C*07:145	07:145	HLA05082		0	0	3	0	0	0	0	3									
C*07:146	07:146	HLA05083		0	0	1	0	0	0	0	1									
C*07:147	07:147	HLA05138		0	0	38	0	0	0	0	38			WD					WD	WD
C*07:148	07:148	HLA05221		0	1	12	0	0	0	0	13			WD					WD	WD
C*07:149	07:149	HLA05419		0	0	8	0	0	0	0	8			WD					WD	WD
C*07:150Q	07:150Q	HLA05544		0	0	0	0	0	0	1	1									
C*07:151	07:151	HLA05554		0	0	8	0	0	0	2	10			WD					WD	WD
C*07:154	07:154	HLA05780		0	6	0	0	0	0	1	7		WD						WD	WD
C*07:155	07:155	HLA05820		0	0	17	3	1	2	3	26			WD					WD	WD
C*07:163	07:163	HLA06048		0	0	1	0	0	0	0	1									
C*07:165	07:165	HLA06070		0	4	6	0	0	0	0	10			WD					WD	WD
C*07:168	07:168	HLA06170		0	0	1	0	0	0	0	1									
C*07:169	07:169	HLA06171		0	1	0	0	0	0	0	1									
C*07:170	07:170	HLA06277		0	0	1	0	1	0	0	2									
C*07:171	07:171	HLA06306		0	0	1	1	0	0	0	2									
**C*07:172 total**	**07:172 total**			**0**	**1**	**16**	**0**	**3**	**0**	**0**	**20**			**WD**					**WD**	**WD**
C*07:172	07:172			0	0	9	0	3	0	0	12			WD					WD	WD
C*07:172:01	07:172:01	HLA06387		0	1	7	0	0	0	0	8			WD					WD	WD
C*07:173	07:173	HLA06389		6	0	27	0	1	0	1	35	WD		WD					WD	WD

Supplemental Table 10: HLA-C Allele Summary [a]				Allele Count by Population Group [b]								3.0.0 CIWD Category by Population Group [c]								
Allele	Genomic typing	Allele ID	G group	AFA	API	EURO	MENA	HIS	NAM	UNK	Total	AFA	API	EURO	MENA	HIS	NAM	UNK	Total	Highest Frequency
C*07:175	07:175	HLA06392		0	0	1	0	0	0	0	1									
C*07:176	07:176	HLA06408		0	0	1	0	0	0	0	1									
C*07:177	07:177	HLA06409		0	0	2	0	0	0	1	3									
C*07:178	07:178	HLA06410		0	0	2	0	0	0	0	2									
C*07:179	07:179	HLA06423		0	0	16	0	0	0	0	16			WD					WD	WD
C*07:180	07:180	HLA06425		0	0	13	0	0	0	0	13			WD					WD	WD
C*07:181	07:181	HLA06429		0	0	4	0	0	0	1	5								WD	WD
C*07:182	07:182	HLA06430		1	0	0	0	0	0	0	1									
C*07:184	07:184	HLA06449		6	0	0	0	0	0	2	8	WD							WD	WD
C*07:185	07:185	HLA06525		0	0	4	0	0	0	0	4									
C*07:186	07:186	HLA06655		0	0	0	0	0	1	1	2									
C*07:189	07:189	HLA06706		0	0	1	0	0	0	0	1									
C*07:191N	07:191N	HLA06717		0	0	2	0	0	0	0	2									
C*07:192	07:192	HLA06719		0	0	11	0	0	0	1	12			WD					WD	WD
C*07:194	07:194	HLA06735		0	0	3	0	0	0	0	3									
C*07:196	07:196	HLA06896		0	0	4	0	1	0	1	6								WD	WD
C*07:198N	07:198N	HLA07051		0	0	0	0	4	0	1	5								WD	WD
**C*07:199 total**	**07:199 total**			**0**	**8**	**6**	**0**	**0**	**0**	**0**	**14**		**WD**	**WD**					**WD**	**WD**
C*07:199:01	07:199:01	HLA07057		0	8	1	0	0	0	0	9		WD						WD	WD
C*07:199:02	07:199:02	HLA08183		0	0	5	0	0	0	0	5			WD					WD	WD
C*07:200	07:200	HLA07058		0	0	0	0	0	0	2	2									
C*07:201	07:201	HLA07059		0	0	2	0	0	0	0	2									
C*07:202	07:202	HLA07070		0	0	0	1	0	0	0	1									
**C*07:204 total**	**07:204 total**			**0**	**0**	**2**	**0**	**0**	**0**	**1**	**3**									
C*07:204P	07:204P			0	0	0	0	0	0	1	1									
C*07:204:01	07:204:01	HLA07072		0	0	2	0	0	0	0	2									
C*07:205	07:205	HLA07078		0	0	19	0	0	0	0	19			WD					WD	WD
C*07:206	07:206	HLA07079		0	0	8	0	3	0	1	12			WD					WD	WD
C*07:207	07:207	HLA07081		0	0	4	0	0	0	0	4									
C*07:209	07:209	HLA07130		0	0	6	0	0	0	0	6			WD					WD	WD
C*07:211	07:211	HLA07133		0	4	0	0	0	0	0	4									

Supplemental Table 10: HLA-C Allele Summary [a]				Allele Count by Population Group [b]								3.0.0 CIWD Category by Population Group [c]								
Allele	Genomic typing	Allele ID	G group	AFA	API	EURO	MENA	HIS	NAM	UNK	Total	AFA	API	EURO	MENA	HIS	NAM	UNK	Total	Highest Frequency
C*07:212	07:212	HLA07182		1	3	8	0	1	0	3	16			WD					WD	WD
C*07:213	07:213	HLA07215		0	0	0	0	1	0	0	1									
C*07:214	07:214	HLA07217		0	0	5	0	0	0	0	5			WD					WD	WD
C*07:215	07:215	HLA07218		0	0	1	0	0	0	0	1									
C*07:216	07:216	HLA07219		0	0	4	0	0	0	0	4									
C*07:218	07:218	HLA07221		1	2	10	0	0	0	1	14			WD					WD	WD
C*07:222	07:222	HLA07477		0	0	5	0	0	0	0	5			WD					WD	WD
C*07:223	07:223	HLA07481		0	1	4	0	0	0	1	6								WD	WD
C*07:224	07:224	HLA07482		0	3	2	0	0	0	0	5								WD	WD
C*07:226	07:226	HLA07484		0	0	1	0	0	0	0	1									
C*07:227N	07:227N	HLA07486		0	0	4	0	1	0	0	5								WD	WD
C*07:229	07:229	HLA07734		0	1	2	0	1	0	0	4									
C*07:231	07:231	HLA07736		0	2	0	0	0	0	0	2									
C*07:232	07:232	HLA07737		0	14	0	0	0	0	0	14		I						WD	I
C*07:234	07:234	HLA07739		0	0	6	0	0	0	2	8			WD					WD	WD
C*07:238	07:238	HLA07837		0	0	3	0	0	0	0	3									
C*07:240	07:240	HLA07839		0	0	1	0	0	0	0	1									
C*07:241	07:241	HLA07840		0	2	7	0	1	0	1	11			WD					WD	WD
C*07:242	07:242	HLA07842		0	0	13	0	0	0	0	13			WD					WD	WD
C*07:243	07:243	HLA07843		0	0	3	3	0	0	0	6								WD	WD
C*07:244	07:244	HLA07930		0	0	0	0	0	0	1	1									
**C*07:246 total**	**07:246 total**			**0**	**1**	**0**	**0**	**0**	**0**	**0**	**1**									
C*07:246	07:246			0	1	0	0	0	0	0	1									
C*07:247	07:247	HLA08150		0	1	4	0	0	0	0	5								WD	WD
C*07:248	07:248	HLA08151		0	1	2	0	0	0	0	3									
C*07:249	07:249	HLA08152		0	0	3	0	0	0	0	3									
C*07:250	07:250	HLA08153		0	0	13	0	0	0	1	14			WD					WD	WD
C*07:252	07:252	HLA08180		0	0	10	0	0	0	1	11			WD					WD	WD
C*07:254	07:254	HLA08186		0	0	2	0	0	0	1	3									
C*07:255	07:255	HLA08187		1	0	2	0	0	0	0	3									
C*07:256	07:256	HLA08188		0	0	6	0	0	0	0	6			WD					WD	WD

Supplemental Table 10: HLA-C Allele Summary [a]				Allele Count by Population Group [b]								3.0.0 CIWD Category by Population Group [c]								
Allele	Genomic typing	Allele ID	G group	AFA	API	EURO	MENA	HIS	NAM	UNK	Total	AFA	API	EURO	MENA	HIS	NAM	UNK	Total	Highest Frequency
**C*07:257 total**	**07:257 total**			**1**	**0**	**6**	**0**	**0**	**0**	**0**	**7**			**WD**					**WD**	**WD**
C*07:257	07:257			0	0	3	0	0	0	0	3									
C*07:257:01	07:257:01	HLA08189		0	0	1	0	0	0	0	1									
C*07:257:02	07:257:02	HLA12669		0	0	1	0	0	0	0	1									
C*07:257:03	07:257:03	HLA13039		0	0	1	0	0	0	0	1									
C*07:257:04	07:257:04	HLA14324		1	0	0	0	0	0	0	1									
C*07:258	07:258	HLA08190		0	0	1	0	0	0	0	1									
C*07:259	07:259	HLA08191		0	0	2	0	0	0	0	2									
**C*07:260 total**	**07:260 total**			**0**	**0**	**0**	**0**	**1**	**0**	**0**	**1**									
C*07:260	07:260			0	0	0	0	1	0	0	1									
C*07:261	07:261	HLA08193		0	2	5	0	0	0	0	7			WD					WD	WD
C*07:262	07:262	HLA08195		0	0	10	0	1	0	1	12			WD					WD	WD
C*07:264N	07:264N	HLA08402		0	0	0	0	3	0	0	3									
C*07:266	07:266	HLA08474		0	0	0	0	0	0	1	1									
C*07:269	07:269	HLA08527		0	0	1	0	0	0	0	1									
C*07:273	07:273	HLA08691		0	0	3	0	0	0	0	3									
C*07:276	07:276	HLA08704		0	0	3	0	0	0	0	3									
C*07:278	07:278	HLA08706		0	0	1	0	0	0	0	1									
C*07:279	07:279	HLA08707		0	0	5	0	0	0	0	5			WD					WD	WD
C*07:280	07:280	HLA08708		0	0	2	0	0	0	0	2									
C*07:283	07:283	HLA08714		0	0	1	0	0	0	0	1									
C*07:285	07:285	HLA08716		0	0	12	0	0	0	0	12			WD					WD	WD
C*07:286	07:286	HLA08717		0	0	1	0	0	0	0	1									
C*07:287	07:287	HLA08718		0	0	8	0	0	0	1	9			WD					WD	WD
C*07:288	07:288	HLA08719		0	0	0	0	0	0	1	1									
C*07:289	07:289	HLA08723		0	1	0	0	0	0	0	1									
C*07:292	07:292	HLA08726		0	0	3	0	0	0	0	3									
C*07:293	07:293	HLA08833		0	0	2	0	3	0	2	7								WD	WD
C*07:294	07:294	HLA08897		0	0	1	0	0	0	0	1									
C*07:296	07:296	HLA09013		0	0	2	0	0	0	0	2									
C*07:298	07:298	HLA09019		0	0	5	0	0	0	1	6			WD					WD	WD

Supplemental Table 10: HLA-C Allele Summary [a]				Allele Count by Population Group [b]								3.0.0 CIWD Category by Population Group [c]								
**Allele**	**Genomic typing**	**Allele ID**	**G group**	**AFA**	**API**	**EURO**	**MENA**	**HIS**	**NAM**	**UNK**	**Total**	**AFA**	**API**	**EURO**	**MENA**	**HIS**	**NAM**	**UNK**	**Total**	**Highest Frequency**
C*07:301	07:301	HLA09178		0	0	15	0	0	0	1	16			WD					WD	WD
C*07:303	07:303	HLA09184		0	0	6	0	0	0	0	6			WD					WD	WD
C*07:304	07:304	HLA09185		0	0	1	0	1	0	0	2									
C*07:306	07:306	HLA09188		0	0	2	0	0	0	0	2									
C*07:307	07:307	HLA09451		0	3	1	0	0	0	0	4									
C*07:309	07:309	HLA09598		0	1	3	0	0	0	0	4									
C*07:311	07:311	HLA09603		0	0	1	0	0	0	0	1									
C*07:312	07:312	HLA09605		0	0	4	0	0	0	1	5								WD	WD
**C*07:314 total**	**07:314 total**			**0**	**2**	**4**	**0**	**0**	**0**	**1**	**7**								**WD**	**WD**
C*07:314	07:314			0	0	3	0	0	0	1	4									
C*07:314:01	07:314:01	HLA09689		0	1	0	0	0	0	0	1									
C*07:314:02	07:314:02	HLA13259		0	1	1	0	0	0	0	2									
C*07:317	07:317	HLA09906		0	3	0	0	0	0	0	3									
C*07:319	07:319	HLA09997		0	0	1	0	0	0	0	1									
C*07:322	07:322	HLA10014		0	0	1	0	0	0	0	1									
C*07:325	07:325	HLA10053		0	0	0	0	1	0	0	1									
C*07:326	07:326	HLA10054		0	0	0	0	6	0	0	6					WD			WD	WD
C*07:327	07:327	HLA10055		0	4	0	0	0	0	0	4									
C*07:328	07:328	HLA10058		0	0	0	0	0	0	1	1									
C*07:329N	07:329N	HLA10064		0	0	2	0	0	0	0	2									
**C*07:330 total**	**07:330 total**			**0**	**0**	**2**	**0**	**0**	**0**	**0**	**2**									
C*07:330	07:330			0	0	1	0	0	0	0	1									
C*07:330:01	07:330:01	HLA10067		0	0	1	0	0	0	0	1									
C*07:331	07:331	HLA10068		0	0	1	0	0	0	0	1									
C*07:333	07:333	HLA10071		0	0	1	2	0	0	0	3									
C*07:335	07:335	HLA10073		0	0	11	0	0	0	0	11			WD					WD	WD
C*07:336	07:336	HLA10074		0	0	1	0	0	0	0	1									
C*07:338	07:338	HLA10424		0	0	1	0	0	0	0	1									
C*07:339	07:339	HLA10427		0	13	0	0	0	0	0	13		I						WD	I
C*07:340	07:340	HLA10430		0	17	0	0	0	0	0	17		I						WD	I
C*07:342	07:342	HLA10565		0	3	1	0	0	0	0	4									

Supplemental Table 10: HLA-C Allele Summary [a]				Allele Count by Population Group [b]								3.0.0 CIWD Category by Population Group [c]								
Allele	Genomic typing	Allele ID	G group	AFA	API	EURO	MENA	HIS	NAM	UNK	Total	AFA	API	EURO	MENA	HIS	NAM	UNK	Total	Highest Frequency
C*07:351	07:351	HLA10922		0	2	0	0	0	0	1	3									
C*07:352	07:352	HLA10930		0	0	2	0	0	0	1	3									
C*07:353	07:353	HLA10931		0	0	0	0	1	0	0	1									
C*07:354	07:354	HLA10925		0	0	3	0	0	0	0	3									
C*07:356	07:356	HLA11044		0	0	0	0	0	0	1	1									
C*07:357	07:357	HLA11048		0	1	1	0	0	0	0	2									
C*07:358	07:358	HLA11049		0	0	3	0	0	0	0	3									
C*07:360	07:360	HLA11553		0	0	8	0	0	0	3	11			WD					WD	WD
C*07:361	07:361	HLA11252		0	4	0	0	0	0	0	4									
C*07:363	07:363	HLA11254		0	0	1	0	0	0	0	1									
C*07:365	07:365	HLA11336		1	0	0	0	0	0	0	1									
C*07:366	07:366	HLA11337		0	0	2	0	0	0	0	2									
**C*07:368 total**	**07:368 total**			**0**	**1**	**2**	**0**	**0**	**0**	**4**	**7**								**WD**	**WD**
C*07:368	07:368			0	0	0	0	0	0	2	2									
C*07:368:01	07:368:01	HLA11347		0	0	2	0	0	0	2	4									
C*07:368:02	07:368:02	HLA15316		0	1	0	0	0	0	0	1									
C*07:369	07:369	HLA11556		0	0	4	0	0	0	2	6								WD	WD
C*07:370	07:370	HLA11458		0	0	1	0	0	0	0	1									
C*07:376	07:376	HLA11852		0	0	0	1	0	0	0	1									
C*07:378	07:378	HLA11856		0	0	0	1	0	0	0	1									
C*07:379	07:379	HLA12019		0	0	2	0	0	0	0	2									
C*07:381	07:381	HLA12040		0	1	3	0	0	0	0	4									
C*07:384	07:384	HLA12083		0	0	1	0	0	0	0	1									
C*07:386	07:386	HLA12097		0	0	1	0	0	0	0	1									
C*07:387	07:387	HLA12139		0	0	1	0	0	0	0	1									
C*07:390	07:390	HLA12142		0	0	1	0	0	0	0	1									
C*07:391	07:391	HLA12145		0	0	5	0	0	0	0	5			WD					WD	WD
C*07:393N	07:393N	HLA12185		0	1	0	0	0	0	0	1									
C*07:394	07:394	HLA12323		0	0	1	0	0	0	0	1									
C*07:396	07:396	HLA12325		0	1	0	0	0	0	0	1									
C*07:397	07:397	HLA12326		0	2	0	0	0	0	0	2									

Supplemental Table 10: HLA-C Allele Summary [a]				Allele Count by Population Group [b]								3.0.0 CIWD Category by Population Group [c]								
Allele	Genomic typing	Allele ID	G group	AFA	API	EURO	MENA	HIS	NAM	UNK	Total	AFA	API	EURO	MENA	HIS	NAM	UNK	Total	Highest Frequency
C*07:399	07:399	HLA12328		0	0	2	0	0	0	0	2									
C*07:400	07:400	HLA12329		0	0	2	0	0	0	0	2									
C*07:401	07:401	HLA12330		0	0	0	0	0	0	1	1									
C*07:406	07:406	HLA12767		0	0	0	1	0	0	0	1									
C*07:408	07:408	HLA12769		0	0	3	0	0	0	1	4									
C*07:409	07:409	HLA12770		0	0	4	0	0	0	4	8								WD	WD
C*07:411	07:411	HLA13036		0	0	2	0	0	0	0	2									
C*07:412	07:412	HLA13037		0	0	2	0	0	0	0	2									
C*07:413	07:413	HLA13029		0	0	0	0	0	1	0	1									
C*07:415	07:415	HLA13041		0	1	0	0	0	0	0	1									
C*07:417	07:417	HLA13094		0	0	2	0	0	0	0	2									
C*07:418	07:418	HLA13095		0	0	0	0	0	0	1	1									
C*07:420	07:420	HLA13406		0	0	1	0	0	0	0	1									
C*07:428	07:428	HLA13552		0	1	0	0	0	0	0	1									
C*07:429	07:429	HLA13553		0	0	0	3	0	0	0	3									
C*07:433	07:433	HLA13557		0	0	1	0	0	0	0	1									
C*07:434	07:434	HLA13558		0	0	1	0	0	0	0	1									
C*07:438	07:438	HLA13664		0	0	1	0	0	0	0	1									
C*07:439	07:439	HLA13667		0	0	1	0	0	0	0	1									
**C*07:441 total**	**07:441 total**			**0**	**0**	**1**	**0**	**0**	**0**	**0**	**1**									
C*07:441	07:441			0	0	1	0	0	0	0	1									
C*07:442	07:442	HLA13747		0	0	1	0	0	0	0	1									
C*07:443	07:443	HLA13748		0	0	0	0	0	0	1	1									
C*07:444	07:444	HLA13749		0	2	0	0	0	0	0	2									
C*07:447	07:447	HLA13801		0	1	0	0	0	0	0	1									
C*07:448	07:448	HLA13863		0	0	2	0	0	0	1	3									
C*07:450	07:450	HLA13869		0	2	12	0	0	0	0	14			WD					WD	WD
C*07:452N	07:452N	HLA13871		0	0	6	1	0	0	0	7			WD					WD	WD
C*07:455	07:455	HLA14022		0	2	0	0	0	0	0	2									
C*07:456	07:456	HLA14024		0	0	1	0	0	0	0	1									
C*07:457	07:457	HLA14025		0	2	0	0	0	0	0	2									

Supplemental Table 10: HLA-C Allele Summary [a]				Allele Count by Population Group [b]								3.0.0 CIWD Category by Population Group [c]								
Allele	Genomic typing	Allele ID	G group	AFA	API	EURO	MENA	HIS	NAM	UNK	Total	AFA	API	EURO	MENA	HIS	NAM	UNK	Total	Highest Frequency
C*07:463	07:463	HLA14248		0	0	2	0	0	0	0	2									
C*07:464	07:464	HLA14249		0	0	0	0	1	0	0	1									
C*07:465	07:465	HLA14251		0	1	0	0	0	0	0	1									
C*07:470	07:470	HLA14327		0	0	0	0	0	0	1	1									
C*07:473	07:473	HLA14330		0	1	0	0	0	0	0	1									
C*07:476N	07:476N	HLA14250		0	1	0	0	0	0	0	1									
C*07:480	07:480	HLA14521		0	2	0	0	0	0	0	2									
C*07:483N	07:483N	HLA14527		0	0	4	0	0	0	0	4									
C*07:484N	07:484N	HLA14528		1	0	0	0	0	0	1	2									
C*07:485	07:485	HLA14529		0	0	1	0	0	0	0	1									
C*07:488	07:488	HLA14602		0	0	1	0	0	0	0	1									
C*07:490	07:490	HLA14740		0	0	0	0	0	0	1	1									
**C*07:491N total**	**07:491N total**			**0**	**0**	**2**	**0**	**0**	**0**	**0**	**2**									
C*07:491:02N	07:491:02N	HLA15145		0	0	2	0	0	0	0	2									
C*07:493	07:493	HLA14743		0	0	1	0	0	0	0	1									
C*07:496	07:496	HLA14746		0	1	0	0	0	0	0	1									
C*07:498	07:498	HLA14748		0	1	0	0	0	0	0	1									
C*07:504	07:504	HLA15044		0	0	2	0	0	0	0	2									
C*07:506	07:506	HLA15046		0	0	1	0	0	0	0	1									
C*07:508	07:508	HLA15112		0	0	1	0	0	0	0	1									
C*07:514	07:514	HLA15136		0	0	1	0	0	0	0	1									
C*07:519	07:519	HLA15173		0	1	0	0	0	0	0	1									
C*07:525	07:525	HLA15202		0	0	1	0	0	0	0	1									
C*07:528	07:528	HLA15213		0	0	1	0	0	0	0	1									
C*07:529	07:529	HLA15218		0	0	1	0	0	0	0	1									
C*07:541	07:541	HLA15741		0	0	1	0	0	0	0	1									
C*07:543	07:543	HLA15591		0	0	0	0	1	0	0	1									
C*07:548	07:548	HLA15797		0	1	0	0	0	0	0	1									
C*07:550	07:550	HLA15811		0	0	1	0	0	0	0	1									
C*07:552	07:552	HLA15896		0	0	1	0	0	0	0	1									
C*07:555	07:555	HLA15912		0	0	1	0	0	0	0	1									

Supplemental Table 10: HLA-C Allele Summary [a]				Allele Count by Population Group [b]								3.0.0 CIWD Category by Population Group [c]								
Allele	Genomic typing	Allele ID	G group	AFA	API	EURO	MENA	HIS	NAM	UNK	Total	AFA	API	EURO	MENA	HIS	NAM	UNK	Total	Highest Frequency
C*07:558	07:558	HLA15939		0	0	1	0	0	0	0	1									
C*07:563	07:563	HLA15976		0	0	2	0	0	0	0	2									
C*07:573	07:573	HLA16517		0	2	0	0	0	0	0	2									
C*07:CODE	07:CODE			11975	16236	549698	6934	23077	2997	46318	657235	NA	NA	NA	NA	NA	NA	NA	NA	NA
**C*08:01 total**	**08:01 total**			**424**	**51532**	**4872**	**1233**	**14636**	**930**	**8431**	**82058**	**C**	**C**	**C**	**C**	**C**	**C**	**C**	**C**	**C**
C*08:01	08:01			2	6	32	0	5	0	2	47		WD	WD		WD			WD	WD
C*08:01P	08:01P			0	1	5	0	0	0	0	6			WD					WD	WD
**C*08:01:01G total**	**08:01:01G total**			**422**	**51502**	**4834**	**1233**	**14628**	**930**	**8426**	**81975**	**C**	**C**	**C**	**C**	**C**	**C**	**C**	**C**	**C**
C*08:01:01G	08:01:01G		08:01:01G	355	44862	4608	1193	10714	679	7118	69529	C	C	C	C	C	C	C	C	C
C*08:01:01	08:01:01		08:01:01G	67	6368	226	40	3908	251	1275	12135	C	C	I	WD	C	C	C	C	C
C*08:01:03	08:01:03	HLA05786	08:01:01G	0	1	0	0	0	0	0	1									
C*08:01:13	08:01:13	HLA10582	08:01:01G	0	1	0	0	0	0	0	1									
C*08:20	08:20	HLA03632	08:01:01G	0	0	0	0	2	0	0	2									
C*08:22	08:22	HLA03680	08:01:01G	0	256	0	0	4	0	33	293		C					I	I	C
C*08:24	08:24	HLA03763	08:01:01G	0	8	0	0	0	0	0	8		WD						WD	WD
C*08:99	08:99	HLA10619	08:01:01G	0	2	0	0	0	0	0	2									
C*08:148	08:148	HLA16830	08:01:01G	0	4	0	0	0	0	0	4									
C*08:01:02	08:01:02	HLA01586		0	11	1	0	0	0	2	14		WD						WD	WD
C*08:01:06	08:01:06	HLA08157		0	1	0	0	0	0	1	2									
C*08:01:07	08:01:07	HLA08158		0	8	0	0	0	0	0	8		WD						WD	WD
C*08:01:08	08:01:08	HLA08159		0	0	0	0	2	0	0	2									
C*08:01:14	08:01:14	HLA11095		0	2	0	0	0	0	0	2									
C*08:01:16	08:01:16	HLA11571		0	1	0	0	0	0	0	1									
C*08:01:18	08:01:18	HLA12333		0	0	0	0	1	0	0	1									
**C*08:02 total**	**08:02 total**			**13754**	**2901**	**290119**	**10577**	**30263**	**2267**	**45740**	**395621**	**C**	**C**	**C**	**C**	**C**	**C**	**C**	**C**	**C**
C*08:02	08:02			89	49	7397	313	55	17	835	8755	C	I	C	C	I	C	C	C	C
C*08:02P	08:02P			4	0	434	2	1	0	2	443			I					I	I
**C*08:02:01G total**	**08:02:01G total**			**13651**	**2851**	**282270**	**10261**	**30201**	**2250**	**44896**	**386380**	**C**	**C**	**C**	**C**	**C**	**C**	**C**	**C**	**C**
C*08:02:01G	08:02:01G		08:02:01G	10121	2400	252509	9558	20375	1482	37894	334339	C	C	C	C	C	C	C	C	C
C*08:02:01	08:02:01		08:02:01G	1689	208	16731	411	4259	346	4819	28463	C	C	C	C	C	C	C	C	C
C*08:02:01:01	08:02:01:01	HLA00446	08:02:01G	1432	126	9639	270	4642	373	1656	18138	C	C	C	C	C	C	C	C	C

Supplemental Table 10: HLA-C Allele Summary [a]				Allele Count by Population Group [b]								3.0.0 CIWD Category by Population Group [c]								
Allele	Genomic typing	Allele ID	G group	AFA	API	EURO	MENA	HIS	NAM	UNK	Total	AFA	API	EURO	MENA	HIS	NAM	UNK	Total	Highest Frequency
C*08:02:01:02	08:02:01:02	HLA10680	08:02:01G	409	117	3390	22	925	49	527	5439	C	I	C	WD	C	C	C	C	C
C*08:110	08:110	HLA11957	08:02:01G	0	0	1	0	0	0	0	1									
C*08:02:02	08:02:02	HLA04345		0	0	7	0	0	0	1	8			WD					WD	WD
C*08:02:04	08:02:04	HLA05309		3	1	5	0	0	0	2	11			WD					WD	WD
C*08:02:06	08:02:06	HLA06413		3	0	1	1	1	0	2	8								WD	WD
C*08:02:07	08:02:07	HLA07273		0	0	2	0	0	0	0	2									
C*08:02:08	08:02:08	HLA08753		4	0	0	0	2	0	2	8								WD	WD
C*08:02:09	08:02:09	HLA09026		0	0	2	0	0	0	0	2									
C*08:02:10	08:02:10	HLA09194		0	0	1	0	0	0	0	1									
C*08:02:11	08:02:11	HLA11258		0	0	0	0	3	0	0	3									
**C*08:03 total**	**08:03 total**			**48**	**2692**	**4987**	**345**	**1881**	**199**	**1508**	**11660**	**C**	**C**	**C**	**C**	**C**	**C**	**C**	**C**	**C**
C*08:03	08:03			0	10	32	0	5	0	7	54		WD	WD		WD		WD	WD	WD
C*08:03P	08:03P			0	0	3	0	0	0	0	3									
**C*08:03:01G total**	**08:03:01G total**			**48**	**2682**	**4952**	**345**	**1876**	**199**	**1501**	**11603**	**C**	**C**	**C**	**C**	**C**	**C**	**C**	**C**	**C**
C*08:03:01G	08:03:01G		08:03:01G	28	2233	4635	327	1338	132	1317	10010	WD	C	C	C	C	C	C	C	C
C*08:03:01	08:03:01	HLA00447	08:03:01G	20	449	317	18	538	67	184	1593	WD	C	I	WD	C	C	C	I	C
**C*08:04 total**	**08:04 total**			**3723**	**16**	**253**	**47**	**637**	**122**	**903**	**5701**	**C**	**I**	**I**	**C**	**C**	**C**	**C**	**C**	**C**
C*08:04	08:04			503	1	23	2	96	19	111	755	C		WD		C	C	I	I	C
C*08:04:01	08:04:01	HLA00448		3214	15	230	45	541	103	790	4938	C	I	I	C	C	C	C	C	C
C*08:04:02	08:04:02	HLA07521		6	0	0	0	0	0	2	8	WD							WD	WD
C*08:05	08:05	HLA00449		8	0	75	0	1	0	14	98	WD		WD				I	WD	I
C*08:06	08:06	HLA00450		0	16	6	0	0	46	52	120		I	WD			C	I	WD	C
C*08:07	08:07	HLA01136		0	0	4	0	1	0	1	6								WD	WD
**C*08:08 total**	**08:08 total**			**0**	**1**	**15**	**0**	**38**	**1**	**30**	**85**			**WD**		**I**		**I**	**WD**	**I**
C*08:08	08:08			0	0	1	0	13	0	4	18					I			WD	I
C*08:08:01	08:08:01	HLA01238		0	1	14	0	25	1	26	67			WD		I		I	WD	I
C*08:10	08:10	HLA01835		0	1	12	0	144	9	14	180			WD		C	C	I	I	C
C*08:11	08:11	HLA01857		0	0	5	0	26	5	11	47			WD		I	WD	WD	WD	I
C*08:12	08:12	HLA01879		2	0	84	0	1	0	5	92			WD				WD	WD	WD
C*08:13	08:13	HLA02302		14	0	3	0	24	0	2	43	WD				I			WD	I
**C*08:15 total**	**08:15 total**			**1**	**0**	**31**	**0**	**3**	**0**	**8**	**43**			**WD**				**WD**	**WD**	**WD**

Supplemental Table 10: HLA-C Allele Summary [a]				Allele Count by Population Group [b]								3.0.0 CIWD Category by Population Group [c]								
Allele	Genomic typing	Allele ID	G group	AFA	API	EURO	MENA	HIS	NAM	UNK	Total	AFA	API	EURO	MENA	HIS	NAM	UNK	Total	Highest Frequency
C*08:15	08:15			0	0	4	0	1	0	1	6								WD	WD
C*08:15:01	08:15:01	HLA03017		1	0	24	0	2	0	7	34			WD				WD	WD	WD
C*08:15:02	08:15:02	HLA06138		0	0	3	0	0	0	0	3									
**C*08:16 total**	**08:16 total**			**0**	**3**	**0**	**0**	**0**	**0**	**0**	**3**									
C*08:16:01	08:16:01	HLA03280		0	2	0	0	0	0	0	2									
C*08:16:02	08:16:02	HLA07742		0	1	0	0	0	0	0	1									
**C*08:19 total**	**08:19 total**			**0**	**0**	**1**	**0**	**0**	**0**	**0**	**1**									
C*08:19	08:19			0	0	1	0	0	0	0	1									
C*08:21	08:21	HLA03626		0	3	0	0	0	0	0	3									
C*08:23	08:23	HLA03696		0	0	12	0	0	0	3	15			WD					WD	WD
C*08:25	08:25	HLA03767		0	0	3	0	4	0	1	8								WD	WD
C*08:26N	08:26N	HLA03818		0	1	0	0	0	0	0	1									
C*08:27	08:27	HLA03975		0	1	0	0	0	0	0	1									
C*08:28	08:28	HLA04319		0	0	85	4	0	0	2	91			WD					WD	WD
C*08:29	08:29	HLA04332		0	0	4	0	0	0	0	4									
C*08:30	08:30	HLA04393		1	0	36	0	3	0	7	47			WD				WD	WD	WD
C*08:31	08:31	HLA04583		0	0	1	0	0	0	0	1									
C*08:32	08:32	HLA05180		12	0	2	0	0	0	4	18	WD							WD	WD
**C*08:33 total**	**08:33 total**			**0**	**0**	**13**	**0**	**0**	**0**	**1**	**14**			**WD**					**WD**	**WD**
C*08:33	08:33			0	0	2	0	0	0	1	3									
C*08:33:01	08:33:01	HLA05188		0	0	4	0	0	0	0	4									
C*08:33:02	08:33:02	HLA07053		0	0	3	0	0	0	0	3									
C*08:33:03	08:33:03	HLA08201		0	0	4	0	0	0	0	4									
C*08:34	08:34	HLA05196		0	0	2	0	0	0	0	2									
C*08:35	08:35	HLA05395		0	0	1	0	11	0	8	20					I		WD	WD	I
C*08:37	08:37	HLA05579		0	0	1	0	0	0	0	1									
C*08:41	08:41	HLA05790		0	3	0	0	0	0	0	3									
C*08:43	08:43	HLA05874		10	0	0	0	0	1	3	14	WD							WD	WD
C*08:50	08:50	HLA06428		0	10	0	0	0	1	0	11		WD						WD	WD
C*08:51	08:51	HLA06721		0	0	5	0	0	0	0	5			WD					WD	WD
C*08:53	08:53	HLA07050		0	0	33	0	1	0	2	36			WD					WD	WD

Supplemental Table 10: HLA-C Allele Summary [a]				Allele Count by Population Group [b]								3.0.0 CIWD Category by Population Group [c]								
Allele	Genomic typing	Allele ID	G group	AFA	API	EURO	MENA	HIS	NAM	UNK	Total	AFA	API	EURO	MENA	HIS	NAM	UNK	Total	Highest Frequency
C*08:54	08:54	HLA07110		0	0	4	0	3	0	4	11								WD	WD
C*08:55N	08:55N	HLA07345		0	0	1	0	0	0	0	1									
C*08:56	08:56	HLA07363		0	1	0	0	0	0	0	1									
C*08:57	08:57	HLA07489		0	0	1	0	0	0	0	1									
C*08:62	08:62	HLA07938		0	0	1	0	2	0	0	3									
C*08:63	08:63	HLA08088		0	0	1	0	0	0	0	1									
C*08:66	08:66	HLA08427		0	1	0	0	0	0	0	1									
C*08:68	08:68	HLA08729		0	0	2	0	0	0	0	2									
C*08:69	08:69	HLA08731		0	0	1	2	0	0	0	3									
**C*08:72 total**	**08:72 total**			**0**	**2**	**1**	**0**	**0**	**0**	**0**	**3**									
C*08:72	08:72			0	1	0	0	0	0	0	1									
C*08:72:01	08:72:01	HLA08741		0	1	0	0	0	0	0	1									
C*08:72:02	08:72:02	HLA09610		0	0	1	0	0	0	0	1									
C*08:73	08:73	HLA09023		0	0	1	0	0	0	0	1									
C*08:74	08:74	HLA09051		0	0	0	0	0	0	3	3									
C*08:76	08:76	HLA09166		0	0	1	0	2	0	2	5								WD	WD
C*08:90	08:90	HLA10077		0	0	1	0	0	0	0	1									
C*08:91	08:91	HLA10078		0	1	0	0	0	0	0	1									
C*08:92	08:92	HLA10080		0	0	9	0	0	0	0	9			WD					WD	WD
C*08:93	08:93	HLA10081		0	0	1	0	0	0	0	1									
C*08:94	08:94	HLA10260		0	0	3	0	1	0	0	4									
C*08:95	08:95	HLA10615		0	2	0	0	0	0	0	2									
C*08:103	08:103	HLA10915		0	0	5	0	0	0	1	6			WD					WD	WD
C*08:107	08:107	HLA11859		0	0	4	0	0	0	0	4									
C*08:109	08:109	HLA11866		0	1	0	0	0	0	0	1									
C*08:114	08:114	HLA12857		0	0	0	0	1	0	0	1									
C*08:115	08:115	HLA12859		0	0	2	0	0	0	0	2									
C*08:116	08:116	HLA13044		0	0	0	0	0	0	1	1									
C*08:117	08:117	HLA13098		0	0	0	0	0	0	1	1									
C*08:119	08:119	HLA13269		0	0	0	0	3	0	0	3									
C*08:122	08:122	HLA13750		0	1	0	0	0	0	0	1									

Supplemental Table 10: HLA-C Allele Summary [a]				Allele Count by Population Group [b]								3.0.0 CIWD Category by Population Group [c]								
**Allele**	**Genomic typing**	**Allele ID**	**G group**	**AFA**	**API**	**EURO**	**MENA**	**HIS**	**NAM**	**UNK**	**Total**	**AFA**	**API**	**EURO**	**MENA**	**HIS**	**NAM**	**UNK**	**Total**	**Highest Frequency**
C*08:127N	08:127N	HLA14203		0	5	0	0	0	0	0	5		WD						WD	WD
C*08:CODE	08:CODE			1435	4278	29018	493	5340	451	5544	46559	NA	NA	NA	NA	NA	NA	NA	NA	NA
**C*12:02 total**	**12:02 total**			**848**	**87577**	**112273**	**14088**	**6491**	**714**	**21167**	**243158**	**C**	**C**	**C**	**C**	**C**	**C**	**C**	**C**	**C**
C*12:02	12:02			388	7482	29379	4377	2833	417	14364	59240	C	C	C	C	C	C	C	C	C
C*12:02P	12:02P			0	2	68	0	0	0	0	70			WD					WD	WD
**C*12:02:01G total**	**12:02:01G total**			**460**	**80065**	**82811**	**9711**	**3658**	**297**	**6802**	**183804**	**C**	**C**	**C**	**C**	**C**	**C**	**C**	**C**	**C**
C*12:02:01G	12:02:01G		12:02:01G	294	73514	77402	9121	1546	78	5000	166955	C	C	C	C	C	C	C	C	C
C*12:02:01	12:02:01	HLA00453	12:02:01G	0	1	6	0	0	0	2	9			WD					WD	WD
C*12:02:02	12:02:02		12:02:01G	166	6550	5355	590	2112	219	1799	16791	C	C	C	C	C	C	C	C	C
C*12:02:02:01	12:02:02:01	HLA00454	12:02:01G	0	0	48	0	0	0	0	48			WD					WD	WD
C*12:02:02:03	12:02:02:03	HLA17159	12:02:01G	0	0	0	0	0	0	1	1									
C*12:02:03	12:02:03	HLA01145		0	10	7	0	0	0	0	17		WD	WD					WD	WD
C*12:02:04	12:02:04	HLA04620		0	8	0	0	0	0	0	8		WD						WD	WD
C*12:02:06	12:02:06	HLA05178		0	0	2	0	0	0	0	2									
C*12:02:07	12:02:07	HLA07137		0	2	0	0	0	0	0	2									
C*12:02:08	12:02:08	HLA07555		0	0	5	0	0	0	0	5			WD					WD	WD
C*12:02:09	12:02:09	HLA07785		0	2	0	0	0	0	0	2									
C*12:02:11	12:02:11	HLA10435		0	1	0	0	0	0	0	1									
C*12:02:13	12:02:13	HLA12336		0	3	0	0	0	0	0	3									
C*12:02:14	12:02:14	HLA15132		0	2	1	0	0	0	1	4									
**C*12:03 total**	**12:03 total**			**6249**	**41920**	**689079**	**36904**	**26856**	**2107**	**71883**	**874998**	**C**	**C**	**C**	**C**	**C**	**C**	**C**	**C**	**C**
C*12:03	12:03			18	38	11512	362	30	9	942	12911	WD	I	C	C	I	C	C	C	C
C*12:03P	12:03P			5	30	2573	325	3	1	1374	4311	WD	I	C	C			C	C	C
**C*12:03:01G total**	**12:03:01G total**			**6226**	**41830**	**674713**	**35856**	**26818**	**2097**	**69541**	**857081**	**C**	**C**	**C**	**C**	**C**	**C**	**C**	**C**	**C**
C*12:03:01G	12:03:01G		12:03:01G	5104	39262	649953	34917	20360	1561	66336	817493	C	C	C	C	C	C	C	C	C
C*12:03:01	12:03:01		12:03:01G	312	710	8078	297	1967	136	961	12461	C	C	C	C	C	C	C	C	C
C*12:03:01:01	12:03:01:01	HLA00455	12:03:01G	758	1856	16547	640	4445	396	2221	26863	C	C	C	C	C	C	C	C	C
C*12:03:01:02	12:03:01:02	HLA02766	12:03:01G	44	0	0	0	11	3	9	67	C				I		WD	WD	C
C*12:03:01:07	12:03:01:07	HLA16176	12:03:01G	0	0	2	0	0	0	0	2									
C*12:03:01:08	12:03:01:08	HLA17050	12:03:01G	0	0	1	0	0	0	0	1									
C*12:03:01:09	12:03:01:09	HLA17073	12:03:01G	0	0	2	0	0	0	0	2									

Supplemental Table 10: HLA-C Allele Summary [a]				Allele Count by Population Group [b]								3.0.0 CIWD Category by Population Group [c]								
Allele	Genomic typing	Allele ID	G group	AFA	API	EURO	MENA	HIS	NAM	UNK	Total	AFA	API	EURO	MENA	HIS	NAM	UNK	Total	Highest Frequency
C*12:03:06	12:03:06	HLA03285	12:03:01G	0	1	42	1	19	0	6	69			WD		I		WD	WD	I
C*12:23	12:23	HLA03988	12:03:01G	1	0	2	0	7	0	1	11					I			WD	I
C*12:109	12:109	HLA10136	12:03:01G	0	1	0	0	1	0	0	2									
C*12:125	12:125	HLA11490	12:03:01G	0	0	2	0	0	0	1	3									
C*12:143	12:143	HLA12837	12:03:01G	2	0	80	0	6	1	6	95			WD		WD		WD	WD	WD
C*12:160	12:160	HLA13502	12:03:01G	0	0	0	0	1	0	0	1									
C*12:167	12:167	HLA14117	12:03:01G	5	0	4	1	1	0	0	11	WD							WD	WD
C*12:03:03	12:03:03	HLA01885		0	1	37	1	3	0	8	50			WD				WD	WD	WD
C*12:03:04	12:03:04	HLA02428		0	1	4	0	0	0	0	5								WD	WD
C*12:03:05	12:03:05	HLA03127		0	0	68	0	0	0	0	68			WD					WD	WD
C*12:03:08	12:03:08	HLA04337		0	8	33	353	1	0	10	405		WD	WD	C			WD	I	C
C*12:03:09	12:03:09	HLA04600		0	4	8	0	0	0	0	12			WD					WD	WD
C*12:03:10	12:03:10	HLA04836		0	0	46	1	0	0	0	47			WD					WD	WD
C*12:03:11	12:03:11	HLA04840		0	0	13	0	0	0	0	13			WD					WD	WD
C*12:03:12	12:03:12	HLA05172		0	0	0	2	0	0	1	3									
C*12:03:13	12:03:13	HLA05565		0	0	1	0	0	0	0	1									
C*12:03:15	12:03:15	HLA06001		0	0	5	0	0	0	0	5			WD					WD	WD
C*12:03:17	12:03:17	HLA07064		0	1	2	3	1	0	0	7								WD	WD
C*12:03:18	12:03:18	HLA07123		0	0	37	0	0	0	0	37			WD					WD	WD
C*12:03:19	12:03:19	HLA07230		0	2	1	0	0	0	0	3									
C*12:03:20	12:03:20	HLA07318		0	0	2	0	0	0	0	2									
C*12:03:21	12:03:21	HLA07853		0	0	1	0	0	0	0	1									
C*12:03:22	12:03:22	HLA07855		0	0	0	1	0	0	0	1									
C*12:03:23	12:03:23	HLA08535		0	0	4	0	0	0	0	4									
C*12:03:25	12:03:25	HLA08752		0	0	1	0	0	0	0	1									
C*12:03:26	12:03:26	HLA10082		0	4	1	0	0	0	0	5								WD	WD
C*12:03:27	12:03:27	HLA10088		0	0	2	0	0	0	0	2									
C*12:03:29	12:03:29	HLA10434		0	0	9	0	0	0	1	10			WD					WD	WD
C*12:03:32	12:03:32	HLA12773		0	1	1	0	0	0	0	2									
**C*12:03:34G total**	**12:03:34G total**			**0**	**0**	**2**	**0**	**0**	**0**	**0**	**2**									
C*12:03:34G	12:03:34G		12:03:34G	0	0	1	0	0	0	0	1									

Supplemental Table 10: HLA-C Allele Summary [a]				Allele Count by Population Group [b]								3.0.0 CIWD Category by Population Group [c]								
Allele	Genomic typing	Allele ID	G group	AFA	API	EURO	MENA	HIS	NAM	UNK	Total	AFA	API	EURO	MENA	HIS	NAM	UNK	Total	Highest Frequency
C*12:03:34	12:03:34		12:03:34G	0	0	1	0	0	0	0	1									
C*12:03:35	12:03:35	HLA13101		0	0	1	0	0	0	6	7							WD	WD	WD
C*12:03:37	12:03:37	HLA13531		0	0	1	0	0	0	0	1									
C*12:03:41	12:03:41	HLA16273		0	0	1	0	0	0	0	1									
**C*12:04 total**	**12:04 total**			**10**	**4152**	**208**	**12**	**25**	**8**	**336**	**4751**	**WD**	**C**	**I**	**WD**	**I**	**C**	**C**	**C**	**C**
C*12:04	12:04			1	75	34	1	2	0	14	127		I	WD				I	WD	I
C*12:04:01	12:04:01	HLA00456		0	0	6	1	0	0	0	7			WD					WD	WD
C*12:04:02	12:04:02	HLA00457		9	4077	168	10	23	8	322	4617	WD	C	I	WD	I	C	C	C	C
C*12:05	12:05	HLA00458		7	198	1140	460	34	2	75	1916	WD	C	I	C	I		I	C	C
C*12:06	12:06	HLA00459		0	0	14	0	0	0	1	15			WD					WD	WD
C*12:07	12:07	HLA01138		9	0	1	0	0	0	2	12	WD							WD	WD
C*12:08	12:08	HLA01413		0	4	1	0	0	0	0	5								WD	WD
C*12:09	12:09	HLA01705		2	478	13	12	0	0	22	527		C	WD	WD			I	I	C
C*12:11	12:11	HLA01799		0	1	21	1	0	0	0	23			WD					WD	WD
C*12:12	12:12	HLA01878		11	0	66	6	112	19	30	244	WD		WD	WD	C	C	I	I	C
C*12:13	12:13	HLA01954		2	1	289	0	4	0	31	327			I				I	I	I
**C*12:14 total**	**12:14 total**			**0**	**0**	**6**	**1**	**0**	**0**	**0**	**7**			**WD**					**WD**	**WD**
C*12:14:01	12:14:01	HLA01935		0	0	6	0	0	0	0	6			WD					WD	WD
C*12:181	12:181	HLA15168		0	0	0	1	0	0	0	1									
C*12:15	12:15	HLA01969		0	0	1	0	0	0	0	1									
C*12:16	12:16	HLA02304		0	3	1095	3	1	0	9	1111			I				WD	I	I
**C*12:18 total**	**12:18 total**			**0**	**36**	**4**	**2**	**0**	**0**	**2**	**44**		**I**						**WD**	**I**
C*12:18	12:18			0	5	1	0	0	0	0	6		WD						WD	WD
C*12:18:01	12:18:01	HLA02527		0	30	3	2	0	0	2	37		I						WD	I
C*12:18:02	12:18:02	HLA07783		0	1	0	0	0	0	0	1									
C*12:19	12:19	HLA02605		19	0	0	0	0	1	3	23	WD							WD	WD
C*12:20	12:20	HLA02791		0	2	3	0	0	0	1	6								WD	WD
C*12:21	12:21	HLA02842		0	1	37	0	0	0	0	38			WD					WD	WD
C*12:24	12:24	HLA04129		0	3	42	3	0	0	5	53			WD				WD	WD	WD
C*12:25	12:25	HLA04313		0	0	1	0	0	0	0	1									
C*12:27	12:27	HLA04334		0	0	1	23	0	0	0	24				WD				WD	WD

Supplemental Table 10: HLA-C Allele Summary [a]				Allele Count by Population Group [b]								3.0.0 CIWD Category by Population Group [c]								
Allele	Genomic typing	Allele ID	G group	AFA	API	EURO	MENA	HIS	NAM	UNK	Total	AFA	API	EURO	MENA	HIS	NAM	UNK	Total	Highest Frequency
C*12:28	12:28	HLA04336		0	0	31	0	0	0	1	32			WD					WD	WD
C*12:29	12:29	HLA04591		0	0	2	0	0	0	0	2									
C*12:30	12:30	HLA04592		0	0	178	0	0	0	0	178			I					I	I
C*12:33	12:33	HLA04766		0	0	8	0	1	0	0	9			WD					WD	WD
C*12:34	12:34	HLA04825		0	0	328	0	0	0	5	333			I				WD	I	I
C*12:35	12:35	HLA04834		0	0	2	0	0	0	0	2									
C*12:36	12:36	HLA05185		0	0	0	1	0	0	0	1									
C*12:38	12:38	HLA05199		0	15	1	0	0	0	1	17		I						WD	I
C*12:40	12:40	HLA05204		0	5	0	0	0	0	0	5		WD						WD	WD
C*12:41	12:41	HLA05205		0	19	0	0	0	0	0	19		I						WD	I
C*12:42Q	12:42Q	HLA05212		0	0	26	0	0	0	1	27			WD					WD	WD
C*12:43	12:43	HLA05214		0	0	26	1	0	0	0	27			WD					WD	WD
C*12:45	12:45	HLA05570		0	0	51	1	0	0	1	53			WD					WD	WD
C*12:46N	12:46N	HLA05575		0	0	1	0	0	0	0	1									
C*12:47	12:47	HLA05595		0	0	5	0	2	1	6	14			WD				WD	WD	WD
C*12:48	12:48	HLA05608		0	0	7	0	0	0	0	7			WD					WD	WD
C*12:49	12:49	HLA05751		0	2	1	0	0	0	0	3									
C*12:50	12:50	HLA05837		0	0	3	0	0	0	0	3									
C*12:51	12:51	HLA06136		0	0	1	0	0	0	0	1									
C*12:54	12:54	HLA06414		0	6	29	0	0	0	0	35		WD	WD					WD	WD
**C*12:57 total**	**12:57 total**			**0**	**0**	**0**	**1**	**0**	**0**	**0**	**1**									
C*12:57	12:57			0	0	0	1	0	0	0	1									
C*12:58	12:58	HLA06582		0	0	2	0	0	0	0	2									
C*12:59	12:59	HLA06722		9	0	0	0	4	0	2	15	WD							WD	WD
C*12:60	12:60	HLA06897		0	20	0	0	0	0	0	20		I						WD	I
C*12:61	12:61	HLA07055		0	0	3	3	0	0	0	6								WD	WD
C*12:62	12:62	HLA07062		0	0	12	0	0	0	0	12			WD					WD	WD
C*12:64	12:64	HLA07083		0	4	1	0	0	0	0	5								WD	WD
C*12:67	12:67	HLA07223		0	0	6	0	0	0	0	6			WD					WD	WD
C*12:68	12:68	HLA07232		0	0	4	0	0	0	0	4									
C*12:71	12:71	HLA07741		0	2	0	0	0	0	0	2									

Supplemental Table 10: HLA-C Allele Summary [a]				Allele Count by Population Group [b]								3.0.0 CIWD Category by Population Group [c]								
Allele	Genomic typing	Allele ID	G group	AFA	API	EURO	MENA	HIS	NAM	UNK	Total	AFA	API	EURO	MENA	HIS	NAM	UNK	Total	Highest Frequency
C*12:72	12:72	HLA07745		1	13	0	0	0	0	0	14		I						WD	I
C*12:73	12:73	HLA07782		0	0	0	0	2	0	0	2									
C*12:77	12:77	HLA07852		0	0	1	0	0	0	0	1									
C*12:78	12:78	HLA07854		0	0	4	0	0	0	1	5								WD	WD
C*12:82	12:82	HLA08218		0	0	2	0	0	0	0	2									
C*12:85	12:85	HLA08426		0	1	2	0	0	0	1	4									
C*12:86	12:86	HLA08744		0	0	6	0	0	0	0	6			WD					WD	WD
C*12:87	12:87	HLA08745		0	0	3	0	0	0	0	3									
C*12:90	12:90	HLA08748		0	0	9	0	0	0	0	9			WD					WD	WD
C*12:92	12:92	HLA08893		0	0	2	0	0	0	1	3									
C*12:94	12:94	HLA09086		0	0	4	0	0	0	0	4									
C*12:98	12:98	HLA09613		0	0	1	36	0	0	6	43				WD			WD	WD	WD
**C*12:99 total**	**12:99 total**			**0**	**0**	**22**	**0**	**0**	**0**	**0**	**22**			**WD**					**WD**	**WD**
C*12:99	12:99			0	0	16	0	0	0	0	16			WD					WD	WD
C*12:99:01	12:99:01	HLA09614		0	0	6	0	0	0	0	6			WD					WD	WD
C*12:100	12:100	HLA10089		0	0	4	0	0	0	0	4									
C*12:101	12:101	HLA10090		0	33	1	1	0	0	0	35		I						WD	I
C*12:103	12:103	HLA10092		0	1	0	0	0	0	0	1									
C*12:119	12:119	HLA11102		0	0	1	0	0	0	0	1									
C*12:128	12:128	HLA11863		0	1	0	0	0	0	0	1									
C*12:129	12:129	HLA12043		0	0	1	0	0	0	0	1									
C*12:130	12:130	HLA12189		0	2	0	0	0	0	0	2									
C*12:132	12:132	HLA12192		0	3	1	0	0	0	0	4									
C*12:134	12:134	HLA12335		0	1	0	0	0	0	0	1									
C*12:135	12:135	HLA12429		0	0	3	0	0	0	0	3									
C*12:136	12:136	HLA12503		0	5	0	0	0	0	0	5		WD						WD	WD
C*12:137	12:137	HLA12507		0	0	0	0	1	0	0	1									
C*12:139	12:139	HLA12709		0	0	0	5	0	0	0	5				WD				WD	WD
C*12:144	12:144	HLA12774		0	0	1	0	0	0	0	1									
C*12:146	12:146	HLA12856		0	1	0	0	0	0	0	1									
C*12:147	12:147	HLA12866		0	0	4	0	0	0	0	4									

Supplemental Table 10: HLA-C Allele Summary [a]				Allele Count by Population Group [b]								3.0.0 CIWD Category by Population Group [c]								
**Allele**	**Genomic typing**	**Allele ID**	**G group**	**AFA**	**API**	**EURO**	**MENA**	**HIS**	**NAM**	**UNK**	**Total**	**AFA**	**API**	**EURO**	**MENA**	**HIS**	**NAM**	**UNK**	**Total**	**Highest Frequency**
C*12:149	12:149	HLA13043		0	0	1	3	0	0	1	5								WD	WD
C*12:153	12:153	HLA13409		0	0	4	0	0	0	0	4									
C*12:155Q	12:155Q	HLA13416		0	0	1	0	0	0	0	1									
C*12:156	12:156	HLA13419		0	1	2	0	0	0	0	3									
C*12:157	12:157	HLA13565		0	0	1	0	0	0	0	1									
C*12:158	12:158	HLA13566		0	0	1	0	0	0	0	1									
C*12:159	12:159	HLA13567		0	2	0	0	0	0	0	2									
C*12:162	12:162	HLA13754		0	5	0	0	0	0	0	5		WD						WD	WD
C*12:164	12:164	HLA13874		0	2	0	0	0	0	0	2									
C*12:165	12:165	HLA14028		0	1	1	0	0	0	0	2									
C*12:168	12:168	HLA14255		1	0	0	0	0	0	0	1									
C*12:175	12:175	HLA15051		0	1	0	0	0	0	0	1									
C*12:176	12:176	HLA15052		0	1	0	0	0	0	0	1									
C*12:178	12:178	HLA15140		0	0	1	0	0	0	0	1									
C*12:185	12:185	HLA15191		0	0	1	0	0	0	0	1									
C*12:188	12:188	HLA15427		0	0	1	0	0	0	0	1									
**C*12:195 total**	**12:195 total**			**0**	**0**	**0**	**0**	**1**	**0**	**0**	**1**									
C*12:195:02	12:195:02	HLA16939		0	0	0	0	1	0	0	1									
C*12:198	12:198	HLA15920		0	1	0	0	0	0	0	1									
C*12:CODE	12:CODE			1076	4600	96871	3491	4699	497	14752	125986	NA	NA	NA	NA	NA	NA	NA	NA	NA
**C*14:02 total**	**14:02 total**			**5688**	**45419**	**132525**	**12194**	**7737**	**963**	**16146**	**220672**	**C**	**C**	**C**	**C**	**C**	**C**	**C**	**C**	**C**
C*14:02	14:02			8	21	1366	16	11	0	71	1493	WD	I	C	WD	I		I	I	C
C*14:02P	14:02P			0	3	280	1	2	0	0	286			I					I	I
**C*14:02:01G total**	**14:02:01G total**			**5581**	**45392**	**130341**	**12150**	**7665**	**957**	**16014**	**218100**	**C**	**C**	**C**	**C**	**C**	**C**	**C**	**C**	**C**
C*14:02:01G	14:02:01G		14:02:01G	4167	39696	122356	11847	5213	660	13829	197768	C	C	C	C	C	C	C	C	C
C*14:02:01	14:02:01		14:02:01G	692	4394	5577	232	1644	165	1593	14297	C	C	C	C	C	C	C	C	C
C*14:02:01:01	14:02:01:01	HLA00462	14:02:01G	86	1299	2235	64	503	59	394	4640	C	C	C	C	C	C	C	C	C
C*14:02:01:02	14:02:01:02	HLA14811	14:02:01G	636	1	172	7	305	73	198	1392	C		I	WD	C	C	C	I	C
C*14:02:07	14:02:07	HLA06174	14:02:01G	0	1	0	0	0	0	0	1									
C*14:02:15	14:02:15	HLA10145	14:02:01G	0	1	0	0	0	0	0	1									
C*14:31	14:31	HLA06529	14:02:01G	0	0	1	0	0	0	0	1									

Supplemental Table 10: HLA-C Allele Summary [a]				Allele Count by Population Group [b]								3.0.0 CIWD Category by Population Group [c]								
**Allele**	**Genomic typing**	**Allele ID**	**G group**	**AFA**	**API**	**EURO**	**MENA**	**HIS**	**NAM**	**UNK**	**Total**	**AFA**	**API**	**EURO**	**MENA**	**HIS**	**NAM**	**UNK**	**Total**	**Highest Frequency**
C*14:02:02	14:02:02	HLA00463		2	1	156	6	6	0	12	183			I	WD	WD		WD	I	I
C*14:02:03	14:02:03	HLA01721		97	1	17	21	50	6	40	232	C		WD	WD	I	WD	I	I	C
C*14:02:04	14:02:04	HLA02207		0	0	7	0	0	0	1	8			WD					WD	WD
C*14:02:05	14:02:05	HLA05194		0	0	12	0	0	0	1	13			WD					WD	WD
C*14:02:06	14:02:06	HLA05748		0	1	0	0	0	0	0	1									
C*14:02:08	14:02:08	HLA07141		0	0	2	0	0	0	0	2									
C*14:02:09	14:02:09	HLA07274		0	0	0	0	1	0	0	1									
C*14:02:17	14:02:17	HLA11582		0	0	21	0	0	0	0	21			WD					WD	WD
C*14:02:19	14:02:19	HLA13528		0	0	2	0	0	0	0	2									
C*14:11	14:11	HLA03455		0	0	321	0	2	0	7	330			I				WD	I	I
**C*14:03 total**	**14:03 total**			**916**	**3628**	**2678**	**1158**	**396**	**34**	**1885**	**10695**	**C**	**C**	**C**	**C**	**C**	**C**	**C**	**C**	**C**
**C*14:03:01G total**	**14:03:01G total**			**916**	**3628**	**2678**	**1158**	**396**	**34**	**1885**	**10695**	**C**	**C**	**C**	**C**	**C**	**C**	**C**	**C**	**C**
C*14:03	14:03	HLA00464	14:03:01G	908	3530	2541	1106	393	34	1832	10344	C	C	C	C	C	C	C	C	C
C*14:03P	14:03P		14:03:01G	0	0	4	0	0	0	0	4									
C*14:03:01G	14:03:01G		14:03:01G	8	98	133	52	3	0	53	347	WD	I	I	C			I	I	C
C*14:04	14:04	HLA00465		9	1	646	2	3	0	40	701	WD		I				I	I	I
C*14:05	14:05	HLA01469		1	1	265	0	1	0	8	276			I				WD	I	I
C*14:06	14:06	HLA02087		5	1	6	0	37	0	8	57	WD		WD		I		WD	WD	I
C*14:07N	14:07N	HLA02253		0	0	0	0	1	0	0	1									
C*14:10	14:10	HLA03415		8	0	0	0	0	0	3	11	WD							WD	WD
C*14:12	14:12	HLA03820		0	91	2	1	0	0	2	96		I						WD	I
C*14:13	14:13	HLA04327		0	0	1	0	0	0	0	1									
C*14:14	14:14	HLA04341		0	0	1	0	0	0	0	1									
C*14:15	14:15	HLA04619		0	0	1	0	0	0	0	1									
C*14:17	14:17	HLA04778		0	0	3	0	0	0	0	3									
C*14:18	14:18	HLA04784		0	4	13	0	0	0	0	17			WD					WD	WD
**C*14:24 total**	**14:24 total**			**0**	**21**	**0**	**0**	**0**	**0**	**0**	**21**		**I**						**WD**	**I**
C*14:24	14:24			0	1	0	0	0	0	0	1									
C*14:24:01	14:24:01	HLA05891		0	2	0	0	0	0	0	2									
C*14:24:02	14:24:02	HLA07858		0	18	0	0	0	0	0	18		I						WD	I
C*14:25	14:25	HLA05892		0	1	15	0	0	0	0	16			WD					WD	WD

Supplemental Table 10: HLA-C Allele Summary [a]				Allele Count by Population Group [b]								3.0.0 CIWD Category by Population Group [c]								
**Allele**	**Genomic typing**	**Allele ID**	**G group**	**AFA**	**API**	**EURO**	**MENA**	**HIS**	**NAM**	**UNK**	**Total**	**AFA**	**API**	**EURO**	**MENA**	**HIS**	**NAM**	**UNK**	**Total**	**Highest Frequency**
C*14:26	14:26	HLA05921		0	1	0	0	0	0	0	1									
C*14:27	14:27	HLA06128		0	0	0	0	0	0	1	1									
**C*14:28 total**	**14:28 total**			**0**	**0**	**0**	**0**	**1**	**0**	**0**	**1**									
C*14:28:02	14:28:02	HLA12024		0	0	0	0	1	0	0	1									
C*14:29	14:29	HLA06175		0	8	0	0	0	0	1	9		WD						WD	WD
C*14:32	14:32	HLA07125		0	0	6	0	0	0	0	6			WD					WD	WD
C*14:37	14:37	HLA07804		0	0	4	0	0	0	0	4									
C*14:39	14:39	HLA07825		0	2	0	0	0	0	0	2									
C*14:40	14:40	HLA07860		0	0	0	0	0	0	1	1									
C*14:41	14:41	HLA07861		0	0	5	0	0	0	0	5			WD					WD	WD
C*14:43	14:43	HLA08220		0	0	1	0	0	0	0	1									
C*14:44	14:44	HLA08304		0	0	0	0	1	0	1	2									
**C*14:46 total**	**14:46 total**			**0**	**1**	**1**	**0**	**0**	**0**	**0**	**2**									
C*14:46	14:46			0	1	1	0	0	0	0	2									
C*14:47N	14:47N	HLA08819		0	0	1	0	0	0	0	1									
C*14:49	14:49	HLA08821		0	1	0	0	0	0	0	1									
C*14:50	14:50	HLA09203		0	0	5	0	0	0	0	5			WD					WD	WD
C*14:51	14:51	HLA09204		0	1	0	0	0	0	0	1									
C*14:55	14:55	HLA10108		0	0	1	0	0	0	0	1									
C*14:63	14:63	HLA11267		0	3	0	0	0	0	0	3									
C*14:64	14:64	HLA11268		0	0	4	0	0	0	0	4									
C*14:66	14:66	HLA11581		0	0	2	0	17	0	1	20					I			WD	I
C*14:67	14:67	HLA11669		1	4	0	0	0	0	0	5								WD	WD
C*14:69	14:69	HLA12729		0	0	2	0	0	0	0	2									
C*14:74	14:74	HLA15184		0	0	1	0	0	0	0	1									
C*14:76	14:76	HLA15892		0	0	0	1	0	0	0	1									
C*14:81	14:81	HLA15988		0	1	0	0	0	0	0	1									
C*14:CODE	14:CODE			849	2156	13517	642	1186	169	2382	20901	NA	NA	NA	NA	NA	NA	NA	NA	NA
**C*15:02 total**	**15:02 total**			**2271**	**102664**	**232095**	**21990**	**18659**	**1954**	**30364**	**409997**	**C**	**C**	**C**	**C**	**C**	**C**	**C**	**C**	**C**
C*15:02	15:02			1	8	1748	12	13	0	80	1862		WD	C	WD	I		I	C	C
C*15:02P	15:02P			0	8	463	3	2	0	3	479		WD	I					I	I

Supplemental Table 10: HLA-C Allele Summary [a]				Allele Count by Population Group [b]								3.0.0 CIWD Category by Population Group [c]								
Allele	Genomic typing	Allele ID	G group	AFA	API	EURO	MENA	HIS	NAM	UNK	Total	AFA	API	EURO	MENA	HIS	NAM	UNK	Total	Highest Frequency
**C*15:02:01G total**	**15:02:01G total**			**2269**	**102628**	**229687**	**21959**	**18631**	**1951**	**30249**	**407374**	**C**	**C**	**C**	**C**	**C**	**C**	**C**	**C**	**C**
C*15:02:01G	15:02:01G		15:02:01G	1797	97152	218954	21530	14131	1435	28131	383130	C	C	C	C	C	C	C	C	C
C*15:02:01	15:02:01		15:02:01G	112	1354	2758	113	1017	119	490	5963	C	C	C	C	C	C	C	C	C
C*15:02:01:01	15:02:01:01	HLA00467	15:02:01G	334	4062	7254	274	3249	370	1515	17058	C	C	C	C	C	C	C	C	C
C*15:02:01:02	15:02:01:02	HLA12697	15:02:01G	10	2	384	0	83	6	55	540	WD		I		C	WD	I	I	C
C*15:02:01:03	15:02:01:03	HLA12909	15:02:01G	0	21	6	0	0	5	5	37		I	WD			WD	WD	WD	I
C*15:02:01:04	15:02:01:04	HLA16028	15:02:01G	0	0	1	0	0	0	0	1									
C*15:02:01:06	15:02:01:06	HLA16725	15:02:01G	0	7	1	0	0	0	2	10		WD						WD	WD
C*15:02:01:07	15:02:01:07	HLA16726	15:02:01G	0	0	2	0	1	0	0	3									
C*15:02:07	15:02:07	HLA05893	15:02:01G	0	6	0	0	0	0	0	6		WD						WD	WD
C*15:02:19	15:02:19	HLA10156	15:02:01G	0	1	0	0	0	0	0	1									
**C*15:13 total**	**15:13 total**			**16**	**23**	**323**	**42**	**150**	**16**	**50**	**620**	**WD**	**I**	**I**	**C**	**C**	**C**	**I**	**I**	**C**
C*15:13	15:13		15:02:01G	16	21	310	39	146	16	50	598	WD	I	I	WD	C	C	I	I	C
C*15:13:01:01	15:13:01:01	HLA02022	15:02:01G	0	2	11	3	4	0	0	20			WD					WD	WD
C*15:13:01:02	15:13:01:02	HLA16841	15:02:01G	0	0	2	0	0	0	0	2									
C*15:87	15:87	HLA11486	15:02:01G	0	0	3	0	0	0	1	4									
C*15:97	15:97	HLA12183	15:02:01G	0	0	1	0	0	0	0	1									
C*15:02:02	15:02:02	HLA01081		1	1	136	12	8	3	27	188			I	WD	I		I	I	I
C*15:02:03	15:02:03	HLA02424		0	0	45	2	3	0	4	54			WD					WD	WD
C*15:02:04	15:02:04	HLA03146		0	2	0	0	0	0	0	2									
C*15:02:05	15:02:05	HLA04624		0	0	8	1	0	0	0	9			WD					WD	WD
C*15:02:08	15:02:08	HLA07760		0	0	1	0	0	0	0	1									
C*15:02:10	15:02:10	HLA07791		0	9	0	1	0	0	0	10		WD						WD	WD
C*15:02:11	15:02:11	HLA08757		0	0	0	0	1	0	1	2									
C*15:02:13	15:02:13	HLA08759		0	0	3	0	0	0	0	3									
C*15:02:14	15:02:14	HLA08760		0	0	1	0	1	0	0	2									
C*15:02:20	15:02:20	HLA12197		0	0	2	0	0	0	0	2									
C*15:02:22	15:02:22	HLA13254		0	1	0	0	0	0	0	1									
C*15:02:23	15:02:23	HLA13255		0	6	0	0	0	0	0	6		WD						WD	WD
C*15:02:24	15:02:24	HLA13257		0	1	0	0	0	0	0	1									
C*15:02:25	15:02:25	HLA14598		0	0	1	0	0	0	0	1									

Supplemental Table 10: HLA-C Allele Summary [a]				Allele Count by Population Group [b]								3.0.0 CIWD Category by Population Group [c]								
Allele	Genomic typing	Allele ID	G group	AFA	API	EURO	MENA	HIS	NAM	UNK	Total	AFA	API	EURO	MENA	HIS	NAM	UNK	Total	Highest Frequency
C*15:03	15:03	HLA00468		2	0	10	1	19	0	20	52			WD		I		I	WD	I
**C*15:04 total**	**15:04 total**			**34**	**1270**	**5664**	**2865**	**332**	**19**	**1011**	**11195**	**WD**	**C**	**C**	**C**	**C**	**C**	**C**	**C**	**C**
C*15:04	15:04			15	233	2728	1137	150	8	506	4777	WD	C	C	C	C	C	C	C	C
C*15:04P	15:04P			0	0	4	0	0	0	0	4									
**C*15:04:01G total**	**15:04:01G total**			**19**	**1037**	**2932**	**1728**	**182**	**11**	**505**	**6414**	**WD**	**C**	**C**	**C**	**C**	**C**	**C**	**C**	**C**
C*15:04:01G	15:04:01G		15:04:01G	6	435	645	171	24	0	60	1341	WD	C	I	C	I		I	I	C
C*15:04:01	15:04:01	HLA00469	15:04:01G	13	602	2287	1557	158	11	445	5073	WD	C	C	C	C	C	C	C	C
**C*15:05 total**	**15:05 total**			**5943**	**15274**	**42327**	**6796**	**4355**	**429**	**10378**	**85502**	**C**	**C**	**C**	**C**	**C**	**C**	**C**	**C**	**C**
C*15:05	15:05			28	25	1024	68	7	3	173	1328	WD	I	I	C	I		C	I	C
C*15:05P	15:05P			2	2	128	0	1	0	0	133			I					WD	I
**C*15:05:01G total**	**15:05:01G total**			**5893**	**15247**	**41143**	**6728**	**4346**	**426**	**10196**	**83979**	**C**	**C**	**C**	**C**	**C**	**C**	**C**	**C**	**C**
C*15:05:01G	15:05:01G		15:05:01G	4836	14057	39317	6536	3305	307	9667	78025	C	C	C	C	C	C	C	C	C
C*15:05:01	15:05:01	HLA00470	15:05:01G	42	18	222	41	119	13	37	492	C	I	I	C	C	C	I	I	C
C*15:05:02	15:05:02	HLA00471	15:05:01G	945	1169	1604	148	918	104	476	5364	C	C	C	C	C	C	C	C	C
C*15:05:03	15:05:03	HLA01803	15:05:01G	70	0	0	3	4	2	16	95	C						I	WD	C
C*15:29	15:29	HLA05113	15:05:01G	0	3	0	0	0	0	0	3									
C*15:05:04	15:05:04	HLA01909		3	0	0	0	0	0	0	3									
C*15:05:06	15:05:06	HLA06416		12	0	0	0	1	0	7	20	WD						WD	WD	WD
C*15:05:07	15:05:07	HLA08476		0	0	32	0	0	0	0	32			WD					WD	WD
C*15:05:08	15:05:08	HLA09036		3	0	0	0	0	0	2	5								WD	WD
C*15:05:09	15:05:09	HLA09627		2	0	0	0	0	0	0	2									
**C*15:06 total**	**15:06 total**			**7**	**28**	**3256**	**1023**	**129**	**13**	**374**	**4830**	**WD**	**I**	**C**	**C**	**C**	**C**	**C**	**C**	**C**
C*15:06	15:06			1	1	243	107	15	1	25	393			I	C	I		I	I	C
C*15:06P	15:06P			0	0	5	0	0	0	0	5			WD					WD	WD
C*15:06:01	15:06:01	HLA00472		6	27	2983	916	114	12	349	4407	WD	I	C	C	C	C	C	C	C
C*15:06:02	15:06:02	HLA04779		0	0	19	0	0	0	0	19			WD					WD	WD
C*15:06:03	15:06:03	HLA05865		0	0	6	0	0	0	0	6			WD					WD	WD
C*15:07	15:07	HLA00473		2	2003	20	8	4	1	17	2055		C	WD	WD			I	C	C
C*15:08	15:08	HLA00474		0	33	10	1	30	2	16	92		I	WD		I		I	WD	I
C*15:09	15:09	HLA01124		36	46	678	174	2658	156	488	4236	WD	I	I	C	C	C	C	C	C
**C*15:10 total**	**15:10 total**			**0**	**79**	**7**	**0**	**1**	**0**	**2**	**89**		**I**	**WD**					**WD**	**I**

Supplemental Table 10: HLA-C Allele Summary [a]				Allele Count by Population Group [b]								3.0.0 CIWD Category by Population Group [c]								
Allele	Genomic typing	Allele ID	G group	AFA	API	EURO	MENA	HIS	NAM	UNK	Total	AFA	API	EURO	MENA	HIS	NAM	UNK	Total	Highest Frequency
C*15:10:02	15:10:02	HLA03108		0	78	7	0	1	0	2	88		I	WD					WD	I
C*15:10:03	15:10:03	HLA10105		0	1	0	0	0	0	0	1									
C*15:11	15:11	HLA01424		7	36	925	22	14	2	85	1091	WD	I	I	WD	I		I	I	I
C*15:12	15:12	HLA01884		0	3	1	0	0	0	0	4									
C*15:15	15:15	HLA02396		3	2	5	1	0	0	3	14			WD					WD	WD
C*15:16	15:16	HLA02404		0	0	52	2	18	0	40	112			WD		I		I	WD	I
C*15:17	15:17	HLA02438		0	1	5	0	10	1	2	19			WD		I			WD	I
C*15:22	15:22	HLA03813		44	0	1	0	0	1	7	53	C						WD	WD	C
C*15:23	15:23	HLA04312		0	0	25	1	0	0	1	27			WD					WD	WD
C*15:24	15:24	HLA04598		0	1	67	0	1	0	2	71			WD					WD	WD
C*15:25	15:25	HLA04579		5	0	1	7	1	0	3	17	WD			WD				WD	WD
C*15:26	15:26	HLA04639		0	6	0	0	0	0	0	6		WD						WD	WD
C*15:27	15:27	HLA04693		0	0	2	0	11	0	1	14					I			WD	I
C*15:28	15:28	HLA04831		0	0	2	0	0	0	0	2									
C*15:30	15:30	HLA05115		0	0	32	8	0	0	1	41			WD	WD				WD	WD
C*15:32Q	15:32Q	HLA05160		0	14	0	0	0	0	0	14		I						WD	I
C*15:33	15:33	HLA05181		0	3	0	0	0	0	0	3									
C*15:34	15:34	HLA05198		1	0	3	0	0	0	0	4									
C*15:35	15:35	HLA05216		0	0	1	0	0	0	0	1									
C*15:36	15:36	HLA05558		0	0	3	1	0	0	0	4									
C*15:37	15:37	HLA05581		0	0	3	0	0	0	0	3									
C*15:38	15:38	HLA05600		0	0	6	0	0	0	1	7			WD					WD	WD
C*15:39	15:39	HLA05614		0	0	3	0	0	0	0	3									
C*15:40	15:40	HLA05615		0	5	1	1	0	0	1	8		WD						WD	WD
C*15:41	15:41	HLA05625		0	0	1	0	0	0	0	1									
C*15:42	15:42	HLA05750		0	24	0	0	0	1	2	27		I						WD	I
C*15:43	15:43	HLA05755		0	0	2	0	0	0	0	2									
**C*15:44 total**	**15:44 total**			**0**	**0**	**9**	**0**	**0**	**0**	**0**	**9**			**WD**					**WD**	**WD**
C*15:44	15:44			0	0	6	0	0	0	0	6			WD					WD	WD
C*15:44:01	15:44:01	HLA05854		0	0	3	0	0	0	0	3									
C*15:45	15:45	HLA05879		0	0	1	0	0	0	0	1									

Supplemental Table 10: HLA-C Allele Summary [a]				Allele Count by Population Group [b]								3.0.0 CIWD Category by Population Group [c]								
Allele	Genomic typing	Allele ID	G group	AFA	API	EURO	MENA	HIS	NAM	UNK	Total	AFA	API	EURO	MENA	HIS	NAM	UNK	Total	Highest Frequency
C*15:46	15:46	HLA06002		0	0	1	0	0	0	0	1									
C*15:48	15:48	HLA06123		0	0	2	0	0	0	0	2									
C*15:51	15:51	HLA06725		0	0	0	0	1	0	0	1									
C*15:52	15:52	HLA07049		0	0	3	0	1	0	8	12							WD	WD	WD
C*15:54	15:54	HLA07067		0	0	2	0	0	0	0	2									
C*15:56	15:56	HLA07139		0	0	3	0	0	0	0	3									
C*15:57	15:57	HLA07494		0	6	0	0	0	0	0	6		WD						WD	WD
C*15:58	15:58	HLA07787		0	36	1	0	0	0	0	37		I						WD	I
C*15:59	15:59	HLA07788		0	0	1	0	0	0	0	1									
C*15:60	15:60	HLA07790		0	0	0	0	2	1	0	3									
C*15:61	15:61	HLA08170		0	0	0	0	1	0	0	1									
C*15:62	15:62	HLA08534		0	2	0	0	0	0	0	2									
C*15:63	15:63	HLA08694		0	0	1	0	0	0	0	1									
C*15:66	15:66	HLA09037		0	0	2	0	0	0	0	2									
C*15:68	15:68	HLA09619		0	12	0	0	0	0	0	12		WD						WD	WD
C*15:69	15:69	HLA09621		0	0	4	0	0	0	0	4									
C*15:72	15:72	HLA10095		0	2	3	0	0	0	0	5								WD	WD
C*15:73	15:73	HLA10096		0	3	0	0	0	0	0	3									
C*15:74	15:74	HLA10097		0	0	0	1	0	0	0	1									
C*15:76	15:76	HLA10099		0	0	0	0	1	0	0	1									
C*15:77	15:77	HLA10100		0	0	0	0	0	0	1	1									
**C*15:78 total**	**15:78 total**			**0**	**1**	**0**	**0**	**0**	**0**	**0**	**1**									
C*15:78	15:78			0	1	0	0	0	0	0	1									
C*15:79	15:79	HLA10152		0	1	0	0	0	0	0	1									
C*15:80	15:80	HLA10154		0	0	1	0	0	0	0	1									
C*15:85	15:85	HLA10920		0	2	0	0	1	0	2	5								WD	WD
C*15:88	15:88	HLA11574		0	0	2	0	1	0	1	4									
C*15:89	15:89	HLA11575		0	0	1	0	0	0	0	1									
C*15:90	15:90	HLA11576		0	0	1	0	0	0	0	1									
C*15:91	15:91	HLA11666		0	0	1	0	0	0	2	3									
C*15:92N	15:92N	HLA11667		0	1	0	0	0	0	0	1									

Supplemental Table 10: HLA-C Allele Summary [a]				Allele Count by Population Group [b]								3.0.0 CIWD Category by Population Group [c]								
Allele	Genomic typing	Allele ID	G group	AFA	API	EURO	MENA	HIS	NAM	UNK	Total	AFA	API	EURO	MENA	HIS	NAM	UNK	Total	Highest Frequency
C*15:93	15:93	HLA11869		0	0	0	0	2	0	0	2									
C*15:94	15:94	HLA12021		0	0	2	0	0	0	0	2									
C*15:101	15:101	HLA12342		0	4	0	0	0	0	0	4									
C*15:102	15:102	HLA12422		0	0	1	1	0	1	5	8							WD	WD	WD
**C*15:103 total**	**15:103 total**			**0**	**0**	**0**	**0**	**1**	**0**	**0**	**1**									
**C*15:103:01G total**	**15:103:01G total**			**0**	**0**	**0**	**0**	**1**	**0**	**0**	**1**									
C*15:103	15:103	HLA12572	15:103:01G	0	0	0	0	1	0	0	1									
C*15:105Q	15:105Q	HLA12609		0	1	0	0	0	0	0	1									
C*15:107	15:107	HLA13096		0	0	0	0	1	0	0	1									
C*15:109	15:109	HLA13116		0	1	0	0	0	0	0	1									
C*15:111	15:111	HLA13253		0	0	1	0	0	0	0	1									
C*15:112	15:112	HLA13256		0	1	0	0	0	0	0	1									
C*15:113	15:113	HLA13258		0	1	0	0	0	0	0	1									
C*15:115N	15:115N	HLA13570		0	2	0	0	0	0	0	2									
C*15:119	15:119	HLA14336		0	4	0	0	0	0	0	4									
C*15:122N	15:122N	HLA14610		0	1	1	0	2	0	2	6								WD	WD
C*15:124	15:124	HLA15058		0	7	0	0	0	0	0	7		WD						WD	WD
C*15:126	15:126	HLA15117		0	0	1	0	0	0	0	1									
C*15:129	15:129	HLA15200		0	4	0	0	0	0	0	4									
C*15:CODE	15:CODE			745	4268	30763	1444	3207	396	5545	46368	NA	NA	NA	NA	NA	NA	NA	NA	NA
**C*16:01 total**	**16:01 total**			**31231**	**1317**	**251361**	**2883**	**30863**	**3114**	**36426**	**357195**	**C**	**C**	**C**	**C**	**C**	**C**	**C**	**C**	**C**
C*16:01	16:01			677	32	8395	106	377	40	366	9993	C	I	C	C	C	C	C	C	C
C*16:01P	16:01P			3	1	397	4	6	0	1	412			I		WD			I	I
**C*16:01:01G total**	**16:01:01G total**			**30548**	**1282**	**242542**	**2771**	**30463**	**3074**	**36040**	**346720**	**C**	**C**	**C**	**C**	**C**	**C**	**C**	**C**	**C**
C*16:01:01G	16:01:01G		16:01:01G	18138	889	192186	2395	17409	1668	27003	259688	C	C	C	C	C	C	C	C	C
C*16:01:01	16:01:01		16:01:01G	12409	393	50295	376	13052	1405	9036	86966	C	C	C	C	C	C	C	C	C
C*16:01:01:01	16:01:01:01	HLA00475	16:01:01G	0	0	59	0	1	0	1	61			WD					WD	WD
C*16:01:01:02	16:01:01:02	HLA13972	16:01:01G	0	0	0	0	1	0	0	1									
C*16:01:01:03	16:01:01:03	HLA16727	16:01:01G	0	0	1	0	0	0	0	1									
C*16:58	16:58	HLA09503	16:01:01G	1	0	0	0	0	1	0	2									
C*16:97	16:97	HLA14959	16:01:01G	0	0	1	0	0	0	0	1									

Supplemental Table 10: HLA-C Allele Summary [a]				Allele Count by Population Group [b]								3.0.0 CIWD Category by Population Group [c]								
Allele	Genomic typing	Allele ID	G group	AFA	API	EURO	MENA	HIS	NAM	UNK	Total	AFA	API	EURO	MENA	HIS	NAM	UNK	Total	Highest Frequency
C*16:01:02	16:01:02	HLA02041		0	0	2	0	0	0	0	2									
C*16:01:04	16:01:04	HLA04627		0	2	11	2	17	0	9	41			WD		I		WD	WD	I
C*16:01:05	16:01:05	HLA05596		0	0	1	0	0	0	0	1									
C*16:01:07	16:01:07	HLA07136		0	0	1	0	0	0	1	2									
C*16:01:08	16:01:08	HLA08117		0	0	0	0	0	0	1	1									
C*16:01:09	16:01:09	HLA08161		0	0	3	0	0	0	1	4									
C*16:01:10	16:01:10	HLA08204		0	0	1	0	0	0	0	1									
C*16:01:12	16:01:12	HLA09027		1	0	0	0	0	0	0	1									
C*16:01:13	16:01:13	HLA09028		0	0	0	0	0	0	1	1									
C*16:01:14	16:01:14	HLA10086		0	0	1	0	0	0	0	1									
C*16:01:15	16:01:15	HLA10087		2	0	5	0	0	0	4	11			WD					WD	WD
C*16:01:18	16:01:18	HLA11467		0	0	0	0	0	0	1	1									
C*16:01:19	16:01:19	HLA12193		0	0	0	0	0	0	1	1									
C*16:01:20	16:01:20	HLA14607		0	0	1	0	0	0	0	1									
C*16:01:22	16:01:22	HLA15187		0	0	1	0	0	0	0	1									
**C*16:02 total**	**16:02 total**			**735**	**24077**	**48624**	**8939**	**4516**	**571**	**6923**	**94385**	**C**	**C**	**C**	**C**	**C**	**C**	**C**	**C**	**C**
C*16:02	16:02			164	847	8078	1215	992	203	1957	13456	C	C	C	C	C	C	C	C	C
C*16:02P	16:02P			0	1	132	1	1	0	0	135			I					WD	I
**C*16:02:01G total**	**16:02:01G total**			**571**	**23228**	**40405**	**7703**	**3523**	**368**	**4966**	**80764**	**C**	**C**	**C**	**C**	**C**	**C**	**C**	**C**	**C**
C*16:02:01G	16:02:01G		16:02:01G	470	22201	38341	7490	2454	227	4585	75768	C	C	C	C	C	C	C	C	C
C*16:02:01	16:02:01	HLA00476	16:02:01G	101	1027	2064	213	1069	141	381	4996	C	C	C	C	C	C	C	C	C
C*16:02:02	16:02:02	HLA04604		0	0	1	0	0	0	0	1									
C*16:02:03	16:02:03	HLA04829		0	0	0	3	0	0	0	3									
C*16:02:04	16:02:04	HLA04839		0	0	2	11	0	0	0	13				WD				WD	WD
C*16:02:05	16:02:05	HLA05624		0	0	0	6	0	0	0	6				WD				WD	WD
C*16:02:07	16:02:07	HLA06438		0	0	2	0	0	0	0	2									
C*16:02:08	16:02:08	HLA07048		0	0	2	0	0	0	0	2									
C*16:02:11	16:02:11	HLA10085		0	0	2	0	0	0	0	2									
C*16:02:12	16:02:12	HLA11257		0	1	0	0	0	0	0	1									
**C*16:04 total**	**16:04 total**			**381**	**698**	**17725**	**7850**	**811**	**37**	**5607**	**33109**	**C**	**C**	**C**	**C**	**C**	**C**	**C**	**C**	**C**
C*16:04	16:04			12	12	1288	554	99	2	203	2170	WD	WD	C	C	C		C	C	C

Supplemental Table 10: HLA-C Allele Summary [a]				Allele Count by Population Group [b]								3.0.0 CIWD Category by Population Group [c]								
Allele	Genomic typing	Allele ID	G group	AFA	API	EURO	MENA	HIS	NAM	UNK	Total	AFA	API	EURO	MENA	HIS	NAM	UNK	Total	Highest Frequency
C*16:04P	16:04P			0	0	14	0	0	0	0	14			WD					WD	WD
**C*16:04:01G total**	**16:04:01G total**			**369**	**686**	**16422**	**7296**	**712**	**35**	**5404**	**30924**	**C**	**C**	**C**	**C**	**C**	**C**	**C**	**C**	**C**
C*16:04:01G	16:04:01G		16:04:01G	8	342	944	335	12	0	62	1703	WD	C	I	C	I		I	C	C
C*16:04:01	16:04:01		16:04:01G	360	344	15438	6948	690	35	5337	29152	C	C	C	C	C	C	C	C	C
C*16:04:01:01	16:04:01:01	HLA00478	16:04:01G	1	0	39	13	10	0	4	67			WD	WD	I			WD	I
C*16:04:01:02	16:04:01:02	HLA16842	16:04:01G	0	0	1	0	0	0	1	2									
C*16:04:03	16:04:03	HLA12719		0	0	1	0	0	0	0	1									
C*16:06	16:06	HLA01708		0	0	1	0	0	0	0	1									
**C*16:07 total**	**16:07 total**			**6**	**0**	**14**	**0**	**1**	**0**	**2**	**23**	**WD**		**WD**					**WD**	**WD**
C*16:07	16:07			1	0	4	0	0	0	0	5								WD	WD
C*16:07:01	16:07:01	HLA02242		0	0	9	0	1	0	1	11			WD					WD	WD
C*16:07:02	16:07:02	HLA06276		5	0	1	0	0	0	1	7	WD							WD	WD
C*16:08	16:08	HLA02303		7	0	0	0	0	0	0	7	WD							WD	WD
C*16:09	16:09	HLA02686		0	0	1	1	10	0	1	13					I			WD	I
C*16:10	16:10	HLA03115		0	0	1	0	0	0	0	1									
C*16:12	16:12	HLA03425		0	18	1	4	3	0	0	26		I						WD	I
C*16:13	16:13	HLA03726		0	0	12	0	0	0	0	12			WD					WD	WD
C*16:14	16:14	HLA03738		0	0	49	0	0	0	4	53			WD					WD	WD
**C*16:15 total**	**16:15 total**			**1**	**2**	**22**	**95**	**0**	**0**	**7**	**127**			**WD**	**C**			**WD**	**WD**	**C**
C*16:15	16:15			0	1	1	0	0	0	0	2									
C*16:15:01	16:15:01	HLA04321		1	1	21	95	0	0	7	125			WD	C			WD	WD	C
C*16:16Q	16:16Q	HLA04333		1	1	18	0	0	0	0	20			WD					WD	WD
C*16:18	16:18	HLA04599		0	0	19	0	0	0	2	21			WD					WD	WD
C*16:19	16:19	HLA04632		0	37	0	0	0	0	1	38		I						WD	I
C*16:20	16:20	HLA04698		3	0	0	0	0	0	0	3									
C*16:21	16:21	HLA04767		0	0	10	0	0	0	0	10			WD					WD	WD
C*16:22	16:22	HLA05163		7	0	1	0	0	0	1	9	WD							WD	WD
C*16:24	16:24	HLA05182		1	0	4	0	3	0	2	10								WD	WD
C*16:25	16:25	HLA05208		5	0	5	1	0	0	2	13	WD		WD					WD	WD
C*16:26	16:26	HLA05217		0	0	6	1	2	1	1	11			WD					WD	WD
C*16:27	16:27	HLA05521		0	0	3	0	0	0	0	3									

Supplemental Table 10: HLA-C Allele Summary [a]				Allele Count by Population Group [b]								3.0.0 CIWD Category by Population Group [c]								
Allele	Genomic typing	Allele ID	G group	AFA	API	EURO	MENA	HIS	NAM	UNK	Total	AFA	API	EURO	MENA	HIS	NAM	UNK	Total	Highest Frequency
C*16:30N	16:30N	HLA05589		0	31	1	0	0	0	0	32		I						WD	I
C*16:33	16:33	HLA06395		0	0	0	2	0	0	12	14							WD	WD	WD
C*16:35	16:35	HLA06448		0	0	1	0	0	0	0	1									
C*16:37	16:37	HLA06708		0	0	4	0	0	0	11	15							WD	WD	WD
C*16:38	16:38	HLA06778		0	0	55	0	1	0	6	62			WD				WD	WD	WD
**C*16:39 total**	**16:39 total**			**0**	**0**	**0**	**0**	**0**	**0**	**1**	**1**									
C*16:39:01	16:39:01	HLA06890		0	0	0	0	0	0	1	1									
C*16:40	16:40	HLA07054		1	0	0	0	0	0	1	2									
C*16:42	16:42	HLA07112		0	0	0	0	0	0	2	2									
C*16:43	16:43	HLA07229		2	0	0	0	0	0	0	2									
C*16:45	16:45	HLA07744		4	0	0	0	0	0	0	4									
C*16:46	16:46	HLA07848		17	0	4	23	0	0	3	47	WD			WD				WD	WD
C*16:49	16:49	HLA07851		0	0	2	0	0	0	0	2									
C*16:50	16:50	HLA08162		11	0	1	0	0	0	6	18	WD						WD	WD	WD
C*16:51	16:51	HLA08163		0	0	0	0	1	0	0	1									
C*16:52	16:52	HLA08203		0	0	5	0	0	0	0	5			WD					WD	WD
C*16:53	16:53	HLA08811		0	0	1	0	0	0	0	1									
C*16:54	16:54	HLA08892		0	0	1	0	0	0	0	1									
C*16:56	16:56	HLA09115		0	0	1	0	0	0	0	1									
C*16:59	16:59	HLA09612		0	0	0	0	1	0	0	1									
C*16:60	16:60	HLA10083		0	0	2	0	0	0	0	2									
C*16:61	16:61	HLA10433		0	0	0	0	1	0	0	1									
C*16:64	16:64	HLA10927		0	0	2	0	0	0	0	2									
C*16:65	16:65	HLA11090		1	0	0	0	0	0	1	2									
C*16:69	16:69	HLA11098		0	1	0	0	0	0	0	1									
C*16:71	16:71	HLA11862		0	0	0	0	0	0	3	3									
C*16:73	16:73	HLA11973		0	0	3	0	0	0	0	3									
C*16:74	16:74	HLA12044		0	0	7	0	0	0	0	7			WD					WD	WD
C*16:76	16:76	HLA12191		0	0	1	0	0	0	0	1									
C*16:77N	16:77N	HLA12608		0	0	1	1	0	0	0	2									
C*16:78	16:78	HLA12673		0	0	2	0	0	0	0	2									

Supplemental Table 10: HLA-C Allele Summary [a]				Allele Count by Population Group [b]								3.0.0 CIWD Category by Population Group [c]								
**Allele**	**Genomic typing**	**Allele ID**	**G group**	**AFA**	**API**	**EURO**	**MENA**	**HIS**	**NAM**	**UNK**	**Total**	**AFA**	**API**	**EURO**	**MENA**	**HIS**	**NAM**	**UNK**	**Total**	**Highest Frequency**
C*16:82	16:82	HLA13103		0	0	0	0	0	0	1	1									
C*16:84	16:84	HLA13417		0	0	2	0	0	0	0	2									
C*16:85	16:85	HLA13486		0	0	1	0	0	0	0	1									
C*16:86	16:86	HLA13743		2	0	0	0	0	0	0	2									
C*16:88	16:88	HLA14254		0	6	0	0	0	0	0	6		WD						WD	WD
C*16:89N	16:89N	HLA14257		0	1	0	0	0	0	0	1									
C*16:91	16:91	HLA14531		0	1	0	0	0	0	0	1									
C*16:93	16:93	HLA14609		0	0	1	0	0	0	0	1									
C*16:CODE	16:CODE			4339	719	38715	1042	5059	558	6359	56791	NA	NA	NA	NA	NA	NA	NA	NA	NA
**C*17:01 total**	**17:01 total**			**25323**	**3183**	**126706**	**12964**	**11099**	**1210**	**26099**	**206584**	**C**	**C**	**C**	**C**	**C**	**C**	**C**	**C**	**C**
C*17:01	17:01			92	0	518	10	28	0	105	753	C		I	WD	I		I	I	C
C*17:01P	17:01P			0	0	224	3	1	0	0	228			I					I	I
**C*17:01:01G total**	**17:01:01G total**			**25047**	**3148**	**123451**	**12866**	**10475**	**1157**	**25630**	**201774**	**C**	**C**	**C**	**C**	**C**	**C**	**C**	**C**	**C**
C*17:01:01G	17:01:01G		17:01:01G	20941	2979	121004	12512	8516	944	24344	191240	C	C	C	C	C	C	C	C	C
C*17:01:01	17:01:01		17:01:01G	2514	55	634	136	867	108	674	4988	C	I	I	C	C	C	C	C	C
C*17:01:01:02	17:01:01:02	HLA04311	17:01:01G	1457	12	77	14	404	71	337	2372	C	WD	WD	WD	C	C	C	C	C
C*17:01:01:05	17:01:01:05	HLA14101	17:01:01G	105	95	1320	190	572	28	220	2530	C	I	C	C	C	C	C	C	C
C*17:02	17:02	HLA00482	17:01:01G	0	0	1	0	0	0	0	1									
**C*17:03 total**	**17:03 total**			**210**	**41**	**2883**	**89**	**711**	**59**	**414**	**4407**	**C**	**I**	**C**	**C**	**C**	**C**	**C**	**C**	**C**
C*17:03	17:03			180	34	2469	75	595	53	359	3765	C	I	C	C	C	C	C	C	C
C*17:03:01	17:03:01		17:01:01G	30	7	413	14	116	6	55	641	WD	WD	I	WD	C	WD	I	I	C
C*17:03:01:01	17:03:01:01	HLA00993	17:01:01G	0	0	1	0	0	0	0	1									
C*17:38	17:38	HLA17282	17:01:01G	0	0	1	0	0	0	0	1									
C*17:01:02	17:01:02	HLA04628		2	0	0	0	0	0	0	2									
C*17:01:03	17:01:03	HLA04631		0	0	34	0	0	0	2	36			WD					WD	WD
C*17:01:04	17:01:04	HLA05177		1	1	7	8	0	0	2	19			WD	WD				WD	WD
C*17:01:05	17:01:05	HLA05568		1	0	0	0	0	0	0	1									
C*17:01:07	17:01:07	HLA07115		0	0	0	2	0	0	0	2									
C*17:01:09	17:01:09	HLA08208		0	0	3	0	0	0	0	3									
C*17:01:11	17:01:11	HLA11662		0	0	0	0	0	0	1	1									
C*17:04	17:04	HLA02319		28	0	0	1	1	2	15	47	WD						I	WD	I

Supplemental Table 10: HLA-C Allele Summary [a]				Allele Count by Population Group [b]								3.0.0 CIWD Category by Population Group [c]								
Allele	Genomic typing	Allele ID	G group	AFA	API	EURO	MENA	HIS	NAM	UNK	Total	AFA	API	EURO	MENA	HIS	NAM	UNK	Total	Highest Frequency
C*17:05	17:05	HLA03545		0	0	0	0	1	0	0	1									
C*17:07	17:07	HLA05211		0	0	14	0	0	0	1	15			WD					WD	WD
C*17:09	17:09	HLA06427		4	0	0	0	0	0	0	4									
C*17:10	17:10	HLA07068		0	0	3	0	0	0	0	3									
C*17:11	17:11	HLA07233		0	1	1	1	0	0	0	3									
C*17:14	17:14	HLA08531		1	0	0	0	0	0	0	1									
C*17:15	17:15	HLA08750		0	0	1	0	0	0	1	2									
C*17:17	17:17	HLA09031		0	0	1	0	0	0	0	1									
C*17:18	17:18	HLA09032		0	0	1	1	0	0	0	2									
C*17:19	17:19	HLA09033		0	0	1	0	0	0	0	1									
C*17:20	17:20	HLA10056		0	0	2	0	0	0	0	2									
C*17:21	17:21	HLA10093		2	0	0	0	0	0	0	2									
C*17:22	17:22	HLA10262		0	0	0	2	0	0	1	3									
C*17:23	17:23	HLA10510		4	0	0	0	0	0	1	5								WD	WD
C*17:26	17:26	HLA12337		1	0	0	0	0	0	0	1									
C*17:28	17:28	HLA12508		0	0	0	2	0	0	0	2									
C*17:32	17:32	HLA14867		0	0	1	0	0	0	0	1									
C*17:CODE	17:CODE			1545	63	4255	138	881	93	778	7753	NA	NA	NA	NA	NA	NA	NA	NA	NA
**C*18:01 total**	**18:01 total**			**12643**	**110**	**2976**	**714**	**1841**	**259**	**3104**	**21647**	**C**	**I**	**C**	**C**	**C**	**C**	**C**	**C**	**C**
**C*18:01:01G total**	**18:01:01G total**			**12643**	**110**	**2976**	**714**	**1841**	**259**	**3104**	**21647**	**C**	**I**	**C**	**C**	**C**	**C**	**C**	**C**	**C**
C*18:01	18:01	HLA00483	18:01:01G	734	3	42	3	129	16	182	1109	C		WD		C	C	C	I	C
C*18:01P	18:01P		18:01:01G	2	0	9	0	0	0	0	11			WD					WD	WD
C*18:01:01G	18:01:01G		18:01:01G	10682	100	2782	694	1460	198	2672	18588	C	I	C	C	C	C	C	C	C
C*18:02	18:02	HLA00484	18:01:01G	1225	7	143	17	252	45	250	1939	C	WD	I	WD	C	C	C	C	C
C*18:03	18:03	HLA02839		8	0	0	0	1	0	6	15	WD						WD	WD	WD
C*18:04	18:04	HLA05601		17	0	3	0	2	0	3	25	WD							WD	WD
C*18:05	18:05	HLA06443		2	0	0	0	0	0	1	3									
C*18:06	18:06	HLA08690		27	0	0	0	0	0	3	30	WD							WD	WD
C*18:07N	18:07N	HLA09600		1	0	0	0	0	0	0	1									
C*18:09	18:09	HLA12339		1	0	0	0	0	0	0	1									
C*18:10	18:10	HLA14214		0	0	0	0	0	0	1	1									

Supplemental Table 10: HLA-C Allele Summary [a]				Allele Count by Population Group [b]								3.0.0 CIWD Category by Population Group [c]								
Allele	Genomic typing	Allele ID	G group	AFA	API	EURO	MENA	HIS	NAM	UNK	Total	AFA	API	EURO	MENA	HIS	NAM	UNK	Total	Highest Frequency
C*18:CODE	18:CODE			42	0	11	3	17	3	15	91	NA	NA	NA	NA	NA	NA	NA	NA	NA
C*NEW [d]	NEW			0	0	1	0	2	0	0	3	NA	NA	NA	NA	NA	NA	NA	NA	NA
**C*Total [e]**	**Total**			**389619**	**1255403**	**11827887**	**403229**	**690043**	**67072**	**1302662**	**15935915**									

C, common; I, intermediate; WD, well-documented; NA, not applicable

[a] All alleles observed in the current dataset are included in this table. Note that alleles are not in numerical order; alleles within a G group are clustered together. P group "two-field" total (e.g., written as "C*01:02 total") and G group total summary rows are provided. The table does not list all alleles from IPD-IMGT version 3.31.0, if not present in the study dataset.

[b] Population groups include: AFA (African/African American), API (Asian/Pacific Islands), EURO (European/European descent), MENA (Middle East/North Coast of Africa), HIS (South or Central America/Hispanic/Latino), NAM (Native American populations) and UNK (unknown/not asked/multiple ancestries/other). Total is the overall population i.e., all groups combined.

[c] Allele frequency is calculated by dividing the number of times the “allele” of interest is observed in a population by the total number of copies of all the alleles at that particular genetic locus in the population (reported as the last row in this table and also in Table 2b). The total number of copies is calculated by multiplying the number of individuals times two for all loci except DRB3/4/5. For DRB3/4/5, the number of assignments was used as the total. The CIWD status is determined based on the allele frequency. Allele frequency data will be provided on the website of the next International HLA and Immunogenetics Workshop (https://www.ihiw18.org/). Highest frequency is the highest CIWD designation among all the individual groups.

[d] "CODE" is generically defined as a summary category of submitted HLA typing, including NMDP multiple allele codes, with ambiguities that are not within a single P or G group. "NEW" is a summary category for assignments of novel alleles that did not yet receive a nomenclature assignment. The CODE and NEW categories add to the total number of alleles but should not be assigned CIWD designations (labeled as NA, not applicable) as they do not represent a consistent allele designation (i.e., the NEW category may contain alleles with different DNA sequences that are unrelated to one another).

[e] C*Total is the total number of allele assignments for the population group and is based on two times the number of individuals in the group. This number is also listed in Table 2b. It is not the sum of the column as alleles are not counted more than once. For example, when evaluating frequencies at the level of G resolution, individual alleles that make up the G group (e.g., A*80:01:01, A*80:01:01:01, A*80:01:01:02, A*80:01:01G) are not included in the count because these alleles are summed up in the total G designation (e.g., "A*80:01:01G total").
