# Supplementary material for: Common, intermediate and well‐documented HLA alleles in world populations: CIWD version 3.0.0
Source: HLA. 2020 Jan 31;95(6):516–31. doi: 10.1111/tan.13811 (PMC7317522; doi:10.1111/tan.13811)
Supplement: Supplementary file 11 — Table S11 HLA‐DRB1 primary data [file TAN-95-516-s011.pdf]

Supplemental Table 11: HLA-DRB1 Allele Summary [a]				Allele Count by Population Group [b]								3.0.0 CIWD Category by Population Group [c]								
Allele	Genomic Typing	Allele ID	G group	AFA	API	EURO	MENA	HIS	NAM	UNK	Total	AFA	API	EURO	MENA	HIS	NAM	UNK	Total	Highest Frequency
**DRB1*01:01 total**	**01:01 total**			**9405**	**38257**	**1083192**	**13159**	**30699**	**3157**	**80072**	**1257941**	**C**	**C**	**C**	**C**	**C**	**C**	**C**	**C**	**C**
DRB1*01:01	01:01			447	240	24980	416	745	49	1696	28573	C	C	C	C	C	C	C	C	C
DRB1*01:01P	01:01P			0	2	796	1	3	0	1	803			I					I	I
**DRB1*01:01:01G total**	**01:01:01G total**			**8667**	**38001**	**1057137**	**12741**	**29902**	**3102**	**78304**	**1227854**	**C**	**C**	**C**	**C**	**C**	**C**	**C**	**C**	**C**
DRB1*01:01:01G	01:01:01G		01:01:01G	3105	23773	176152	2295	13645	1228	48045	268243	C	C	C	C	C	C	C	C	C
DRB1*01:01:01	01:01:01	HLA00664	01:01:01G	5561	14228	880928	10444	16253	1872	30259	959545	C	C	C	C	C	C	C	C	C
DRB1*01:50	01:50	HLA08604	01:01:01G	0	0	0	2	0	0	0	2									
DRB1*01:67	01:67	HLA12674	01:01:01G	0	0	6	0	0	0	0	6			WD					WD	WD
DRB1*01:77	01:77	HLA15625	01:01:01G	1	0	44	0	4	2	0	51			WD					WD	WD
DRB1*01:82	01:82	HLA17346	01:01:01G	0	0	7	0	0	0	0	7			WD					WD	WD
DRB1*01:01:02	01:01:02	HLA01598		291	0	13	0	8	2	49	363	C		WD		I		I	I	C
DRB1*01:01:03	01:01:03	HLA01869		0	0	1	0	0	0	1	2									
DRB1*01:01:04	01:01:04	HLA03225		0	0	10	0	0	0	1	11			WD					WD	WD
DRB1*01:01:05	01:01:05	HLA03373		0	0	10	0	41	3	3	57			WD		I			WD	I
DRB1*01:01:06	01:01:06	HLA03389		0	0	35	0	0	0	0	35			WD					WD	WD
DRB1*01:01:07	01:01:07	HLA03390		0	0	110	0	0	0	2	112			WD					WD	WD
DRB1*01:01:08	01:01:08	HLA03428		0	0	44	0	0	0	12	56			WD				WD	WD	WD
DRB1*01:01:09	01:01:09	HLA03448		0	0	2	1	0	0	0	3									
DRB1*01:01:10	01:01:10	HLA03912		0	0	16	0	0	0	0	16			WD					WD	WD
DRB1*01:01:11	01:01:11	HLA04370		0	0	1	0	0	0	0	1									
DRB1*01:01:12	01:01:12	HLA04374		0	0	13	0	0	0	1	14			WD					WD	WD
DRB1*01:01:13	01:01:13	HLA04375		0	0	1	0	0	0	0	1									
DRB1*01:01:14	01:01:14	HLA04376		0	0	1	0	0	0	1	2									
DRB1*01:01:15	01:01:15	HLA04385		0	4	0	0	0	0	0	4									
DRB1*01:01:16	01:01:16	HLA04405		0	0	5	0	0	0	0	5			WD					WD	WD
DRB1*01:01:17	01:01:17	HLA04613		0	10	1	0	0	0	0	11		WD						WD	WD
DRB1*01:01:18	01:01:18	HLA04680		0	0	2	0	0	0	0	2									
DRB1*01:01:19	01:01:19	HLA06502		0	0	5	0	0	0	0	5			WD					WD	WD
DRB1*01:01:21	01:01:21	HLA07538		0	0	1	0	0	0	0	1									
DRB1*01:01:24	01:01:24	HLA09217		0	0	1	0	0	0	0	1									
DRB1*01:01:25	01:01:25	HLA09218		0	0	3	0	0	1	1	5								WD	WD

Supplemental Table 11: HLA-DRB1 Allele Summary [a]				Allele Count by Population Group [b]								3.0.0 CIWD Category by Population Group [c]								
Allele	Genomic Typing	Allele ID	G group	AFA	API	EURO	MENA	HIS	NAM	UNK	Total	AFA	API	EURO	MENA	HIS	NAM	UNK	Total	Highest Frequency
DRB1*01:01:27	01:01:27	HLA11690		0	0	1	0	0	0	0	1									
DRB1*01:01:28	01:01:28	HLA11904		0	0	1	0	0	0	0	1									
DRB1*01:01:29	01:01:29	HLA13291		0	0	2	0	0	0	0	2									
**DRB1*01:02 total**	**01:02 total**			**15957**	**2013**	**136773**	**9500**	**21967**	**1672**	**30244**	**218126**	**C**	**C**	**C**	**C**	**C**	**C**	**C**	**C**	**C**
DRB1*01:02	01:02			1232	59	8027	465	2756	122	1649	14310	C	I	C	C	C	C	C	C	C
DRB1*01:02P	01:02P			0	0	135	0	2	0	0	137			I					WD	I
**DRB1*01:02:01G total**	**01:02:01G total**			**14724**	**1899**	**128567**	**9035**	**19205**	**1550**	**28589**	**203569**	**C**	**C**	**C**	**C**	**C**	**C**	**C**	**C**	**C**
DRB1*01:02:01G	01:02:01G		01:02:01G	181	80	5688	231	195	2	204	6581	C	I	C	C	C		C	C	C
DRB1*01:02:01	01:02:01	HLA00665	01:02:01G	14543	1819	122879	8804	19010	1548	28385	196988	C	C	C	C	C	C	C	C	C
DRB1*01:02:02	01:02:02	HLA00666		0	0	2	0	0	0	0	2									
DRB1*01:02:03	01:02:03	HLA01875		0	0	33	0	1	0	5	39			WD				WD	WD	WD
DRB1*01:02:04	01:02:04	HLA02001		0	0	1	0	2	0	0	3									
DRB1*01:02:06	01:02:06	HLA07152		0	55	8	0	1	0	1	65		I	WD					WD	I
DRB1*01:02:11	01:02:11	HLA13599		1	0	0	0	0	0	0	1									
**DRB1*01:03 total**	**01:03 total**			**908**	**185**	**66397**	**227**	**5344**	**427**	**9638**	**83126**	**C**	**C**	**C**	**C**	**C**	**C**	**C**	**C**	**C**
DRB1*01:03	01:03			895	177	63464	219	5227	418	9301	79701	C	C	C	C	C	C	C	C	C
DRB1*01:03P	01:03P			0	0	8	0	2	0	0	10			WD					WD	WD
DRB1*01:03:01	01:03:01	HLA00667		13	8	2925	8	115	9	337	3415	WD	WD	C	WD	C	C	C	C	C
DRB1*01:04	01:04	HLA00668		1	0	3	0	1	0	3	8								WD	WD
DRB1*01:07	01:07	HLA01307		0	0	462	0	0	1	7	470			I				WD	I	I
DRB1*01:09	01:09	HLA01633		0	0	3	0	0	0	0	3									
DRB1*01:10	01:10	HLA01671		0	0	11	0	0	0	0	11			WD					WD	WD
**DRB1*01:11 total**	**01:11 total**			**3**	**0**	**18**	**0**	**1**	**0**	**2**	**24**			**WD**					**WD**	**WD**
DRB1*01:11	01:11			1	0	7	0	0	0	0	8			WD					WD	WD
DRB1*01:11:01	01:11:01	HLA01811		2	0	9	0	1	0	2	14			WD					WD	WD
DRB1*01:11:02	01:11:02	HLA10322		0	0	2	0	0	0	0	2									
DRB1*01:14	01:14	HLA02494		0	2	0	1	0	0	0	3									
DRB1*01:15	01:15	HLA02500		0	0	1	0	1	0	1	3									
DRB1*01:16	01:16	HLA02666		0	0	6	0	0	0	0	6			WD					WD	WD
DRB1*01:17	01:17	HLA03037		0	0	1	0	0	0	0	1									
**DRB1*01:18 total**	**01:18 total**			**0**	**0**	**17**	**0**	**0**	**0**	**0**	**17**			**WD**					**WD**	**WD**

Supplemental Table 11: HLA-DRB1 Allele Summary [a]				Allele Count by Population Group [b]								3.0.0 CIWD Category by Population Group [c]								
Allele	Genomic Typing	Allele ID	G group	AFA	API	EURO	MENA	HIS	NAM	UNK	Total	AFA	API	EURO	MENA	HIS	NAM	UNK	Total	Highest Frequency
DRB1*01:18	01:18			0	0	15	0	0	0	0	15			WD					WD	WD
DRB1*01:18:01	01:18:01	HLA03038		0	0	1	0	0	0	0	1									
DRB1*01:18:02	01:18:02	HLA16585		0	0	1	0	0	0	0	1									
DRB1*01:19	01:19	HLA03040		0	1	0	0	0	0	0	1									
**DRB1*01:20 total**	**01:20 total**			**0**	**2**	**20**	**0**	**0**	**0**	**2**	**24**			**WD**					**WD**	**WD**
DRB1*01:20	01:20			0	0	9	0	0	0	1	10			WD					WD	WD
DRB1*01:20:01	01:20:01	HLA03377		0	2	11	0	0	0	1	14			WD					WD	WD
DRB1*01:22	01:22	HLA03837		0	0	15	0	0	0	0	15			WD					WD	WD
DRB1*01:23	01:23	HLA04037		3	0	0	0	0	0	0	3									
**DRB1*01:24 total**	**01:24 total**			**0**	**0**	**14**	**0**	**0**	**0**	**0**	**14**			**WD**					**WD**	**WD**
DRB1*01:24	01:24			0	0	4	0	0	0	0	4									
DRB1*01:24:01	01:24:01	HLA04371		0	0	4	0	0	0	0	4									
DRB1*01:24:02	01:24:02	HLA08318		0	0	6	0	0	0	0	6			WD					WD	WD
DRB1*01:26	01:26	HLA04373		11	0	1	0	0	0	0	12	WD							WD	WD
DRB1*01:27	01:27	HLA04377		0	0	6	0	0	0	0	6			WD					WD	WD
DRB1*01:28	01:28	HLA04378		0	0	1	0	0	0	0	1									
**DRB1*01:29 total**	**01:29 total**			**0**	**0**	**5**	**0**	**0**	**0**	**0**	**5**			**WD**					**WD**	**WD**
DRB1*01:29:01	01:29:01	HLA04379		0	0	3	0	0	0	0	3									
DRB1*01:29:02	01:29:02	HLA09657		0	0	2	0	0	0	0	2									
DRB1*01:30	01:30	HLA04380		0	0	1	0	0	0	0	1									
DRB1*01:32	01:32	HLA04783		0	0	2	0	0	0	0	2									
DRB1*01:40N	01:40N	HLA07266		0	0	1	0	0	0	0	1									
DRB1*01:43	01:43	HLA07270		0	0	1	0	0	0	0	1									
**DRB1*01:44 total**	**01:44 total**			**0**	**0**	**3**	**0**	**0**	**0**	**0**	**3**									
DRB1*01:44	01:44			0	0	3	0	0	0	0	3									
DRB1*01:46	01:46	HLA08313		0	0	0	0	0	0	3	3									
DRB1*01:54	01:54	HLA08886		0	0	0	0	1	0	0	1									
DRB1*01:57	01:57	HLA09168		0	0	1	0	0	0	0	1									
DRB1*01:58	01:58	HLA09219		0	0	1	0	0	0	0	1									
DRB1*01:59	01:59	HLA09766		0	0	2	0	0	0	0	2									
DRB1*01:68N	01:68N	HLA13293		0	0	1	0	0	0	0	1									

Supplemental Table 11: HLA-DRB1 Allele Summary [a]				Allele Count by Population Group [b]								3.0.0 CIWD Category by Population Group [c]								
**Allele**	**Genomic Typing**	**Allele ID**	**G group**	**AFA**	**API**	**EURO**	**MENA**	**HIS**	**NAM**	**UNK**	**Total**	**AFA**	**API**	**EURO**	**MENA**	**HIS**	**NAM**	**UNK**	**Total**	**Highest Frequency**
DRB1*01:72	01:72	HLA14189		0	0	1	0	0	0	0	1									
DRB1*01:78	01:78	HLA16002		0	0	1	0	0	0	0	1									
DRB1*01:79	01:79	HLA16966		0	0	1	0	0	0	0	1									
DRB1*01:80	01:80	HLA16967		0	1	0	0	0	0	0	1									
DRB1*01:CODE [d]	01:CODE			977	226	25407	332	2846	131	2119	32038	NA	NA	NA	NA	NA	NA	NA	NA	NA
**DRB1*03:01 total**	**03:01 total**			**25025**	**78186**	**1259298**	**36467**	**45511**	**4489**	**117681**	**1566657**	**C**	**C**	**C**	**C**	**C**	**C**	**C**	**C**	**C**
DRB1*03:01	03:01			12531	10697	321883	5866	20234	2609	48112	421932	C	C	C	C	C	C	C	C	C
DRB1*03:01P	03:01P			2	9	1187	12	2	0	2	1214		WD	I	WD				I	I
**DRB1*03:01:01G total**	**03:01:01G total**			**12462**	**67438**	**935810**	**30498**	**25258**	**1880**	**69521**	**1142867**	**C**	**C**	**C**	**C**	**C**	**C**	**C**	**C**	**C**
DRB1*03:01:01G	03:01:01G		03:01:01G	1412	42766	47727	10886	6818	193	40174	149976	C	C	C	C	C	C	C	C	C
DRB1*03:01:01	03:01:01		03:01:01G	10871	24639	880752	19521	17551	1610	28600	983544	C	C	C	C	C	C	C	C	C
DRB1*03:01:01:01	03:01:01:01	HLA00671	03:01:01G	127	20	6427	68	644	62	678	8026	C	I	C	C	C	C	C	C	C
DRB1*03:01:01:02	03:01:01:02	HLA03483	03:01:01G	35	3	373	6	189	13	47	666	WD		I	WD	C	C	I	I	C
DRB1*03:01:08	03:01:08	HLA04577	03:01:01G	17	8	362	12	4	1	7	411	WD	WD	I	WD			WD	I	I
DRB1*03:01:26	03:01:26	HLA16848	03:01:01G	0	0	6	2	0	0	0	8			WD					WD	WD
DRB1*03:124	03:124	HLA13762	03:01:01G	0	2	57	1	52	1	15	128			WD		I		I	WD	I
DRB1*03:132	03:132	HLA15154	03:01:01G	0	0	99	2	0	0	0	101			WD					WD	WD
DRB1*03:137	03:137	HLA15597	03:01:01G	0	0	7	0	0	0	0	7			WD					WD	WD
DRB1*03:01:02	03:01:02	HLA00672		30	36	348	80	11	0	33	538	WD	I	I	C	I		I	I	C
DRB1*03:01:03	03:01:03	HLA02769		0	0	1	0	0	0	0	1									
DRB1*03:01:04	03:01:04	HLA02830		0	1	3	1	1	0	2	8								WD	WD
DRB1*03:01:05	03:01:05	HLA02955		0	0	0	0	5	0	0	5					WD			WD	WD
DRB1*03:01:07	03:01:07	HLA03855		0	0	13	3	0	0	0	16			WD					WD	WD
DRB1*03:01:10	03:01:10	HLA05969		0	0	1	0	0	0	0	1									
DRB1*03:01:11	03:01:11	HLA05992		0	0	3	0	0	0	0	3									
DRB1*03:01:12	03:01:12	HLA06477		0	1	18	0	0	0	0	19			WD					WD	WD
DRB1*03:01:13	03:01:13	HLA06508		0	1	0	0	0	0	0	1									
DRB1*03:01:14	03:01:14	HLA06770		0	0	12	0	0	0	0	12			WD					WD	WD
DRB1*03:01:15	03:01:15	HLA07149		0	0	11	0	0	0	10	21			WD				WD	WD	WD
DRB1*03:01:16	03:01:16	HLA07155		0	0	0	3	0	0	0	3									
DRB1*03:01:17	03:01:17	HLA07158		0	0	5	0	0	0	0	5			WD					WD	WD

Supplemental Table 11: HLA-DRB1 Allele Summary [a]				Allele Count by Population Group [b]								3.0.0 CIWD Category by Population Group [c]								
**Allele**	**Genomic Typing**	**Allele ID**	**G group**	**AFA**	**API**	**EURO**	**MENA**	**HIS**	**NAM**	**UNK**	**Total**	**AFA**	**API**	**EURO**	**MENA**	**HIS**	**NAM**	**UNK**	**Total**	**Highest Frequency**
DRB1*03:01:18	03:01:18	HLA07159		0	0	1	0	0	0	0	1									
DRB1*03:01:20	03:01:20	HLA07872		0	1	1	4	0	0	0	6								WD	WD
DRB1*03:01:21	03:01:21	HLA09062		0	0	0	0	0	0	1	1									
DRB1*03:01:25	03:01:25	HLA13281		0	2	1	0	0	0	0	3									
**DRB1*03:02 total**	**03:02 total**			**22752**	**167**	**2909**	**1331**	**6128**	**1236**	**6224**	**40747**	**C**	**C**	**C**	**C**	**C**	**C**	**C**	**C**	**C**
DRB1*03:02	03:02			2515	8	269	37	864	101	613	4407	C	WD	I	WD	C	C	C	C	C
DRB1*03:02P	03:02P			0	0	7	0	0	0	0	7			WD					WD	WD
DRB1*03:02:01	03:02:01	HLA00673		20226	159	2617	1294	5112	1130	5569	36107	C	C	C	C	C	C	C	C	C
DRB1*03:02:02	03:02:02	HLA00674		11	0	16	0	152	5	42	226	WD		WD		C	WD	I	I	C
DRB1*03:03	03:03	HLA00675		24	0	1	0	0	2	8	35	WD						WD	WD	WD
**DRB1*03:04 total**	**03:04 total**			**4**	**11**	**2038**	**4**	**34**	**1**	**73**	**2165**		**WD**	**C**		**I**		**I**	**C**	**C**
DRB1*03:04	03:04			0	0	141	0	4	0	6	151			I				WD	WD	I
DRB1*03:04:01	03:04:01	HLA00676		4	11	1897	4	30	1	66	2013		WD	C		I		I	C	C
DRB1*03:04:02	03:04:02	HLA06509		0	0	0	0	0	0	1	1									
**DRB1*03:05 total**	**03:05 total**			**7**	**19**	**257**	**3**	**4**	**2**	**27**	**319**	**WD**	**I**	**I**				**I**	**I**	**I**
DRB1*03:05	03:05			1	0	26	0	2	1	1	31			WD					WD	WD
DRB1*03:05:01	03:05:01	HLA00677		5	18	194	3	1	1	24	246	WD	I	I				I	I	I
DRB1*03:05:02	03:05:02	HLA01428		1	0	29	0	1	0	2	33			WD					WD	WD
DRB1*03:05:03	03:05:03	HLA03765		0	1	8	0	0	0	0	9			WD					WD	WD
DRB1*03:06	03:06	HLA00678		5	20	153	3	0	0	13	194	WD	I	I				WD	I	I
DRB1*03:07	03:07	HLA00679		3	2	60	15	29	2	11	122			WD	WD	I		WD	WD	I
DRB1*03:08	03:08	HLA00680		0	0	7	1	0	0	2	10			WD					WD	WD
DRB1*03:10	03:10	HLA00682		0	2	2	0	0	0	0	4									
**DRB1*03:11 total**	**03:11 total**			**0**	**0**	**8**	**2**	**3**	**0**	**2**	**15**			**WD**					**WD**	**WD**
DRB1*03:11	03:11			0	0	1	1	1	0	0	3									
DRB1*03:11:01	03:11:01	HLA00683		0	0	7	1	2	0	2	12			WD					WD	WD
DRB1*03:12	03:12	HLA00684		0	0	1	0	0	0	0	1									
**DRB1*03:13 total**	**03:13 total**			**1**	**0**	**146**	**0**	**4**	**2**	**8**	**161**			**I**				**WD**	**WD**	**I**
DRB1*03:13	03:13			0	0	10	0	1	0	1	12			WD					WD	WD
DRB1*03:13:01	03:13:01	HLA01007		1	0	125	0	3	2	6	137			I				WD	WD	I
DRB1*03:13:02	03:13:02	HLA03684		0	0	11	0	0	0	1	12			WD					WD	WD

Supplemental Table 11: HLA-DRB1 Allele Summary [a]				Allele Count by Population Group [b]								3.0.0 CIWD Category by Population Group [c]								
Allele	Genomic Typing	Allele ID	G group	AFA	API	EURO	MENA	HIS	NAM	UNK	Total	AFA	API	EURO	MENA	HIS	NAM	UNK	Total	Highest Frequency
DRB1*03:14	03:14	HLA01008		0	1	0	0	0	0	1	2									
**DRB1*03:15 total**	**03:15 total**			**8**	**5**	**138**	**4**	**139**	**9**	**26**	**329**	**WD**	**WD**	**I**		**C**	**C**	**I**	**I**	**C**
DRB1*03:15	03:15			7	3	99	4	107	8	20	248	WD		WD		C	C	I	I	C
DRB1*03:15:01	03:15:01	HLA01087		1	2	39	0	32	1	6	81			WD		I		WD	WD	I
DRB1*03:16	03:16	HLA01152		1	0	7	0	14	2	3	27			WD		I			WD	I
DRB1*03:17	03:17	HLA01153		0	0	6	0	0	0	1	7			WD					WD	WD
DRB1*03:18	03:18	HLA01349		0	0	2	0	0	0	0	2									
DRB1*03:19	03:19	HLA01432		1	2	17	1	0	0	1	22			WD					WD	WD
DRB1*03:20	03:20	HLA01455		0	0	1	0	0	0	0	1									
DRB1*03:22	03:22	HLA01557		0	0	9	0	0	0	0	9			WD					WD	WD
DRB1*03:23	03:23	HLA01614		0	1	155	0	0	0	6	162			I				WD	I	I
DRB1*03:24	03:24	HLA01687		0	0	18	0	0	0	0	18			WD					WD	WD
DRB1*03:25	03:25	HLA01692		0	1	5	0	0	0	0	6			WD					WD	WD
DRB1*03:27	03:27	HLA01930		0	4	0	0	0	0	1	5								WD	WD
DRB1*03:28	03:28	HLA01933		0	0	1	0	0	0	0	1									
DRB1*03:29	03:29	HLA02420		6	0	0	0	0	0	0	6	WD							WD	WD
DRB1*03:30	03:30	HLA02603		0	0	6	0	0	0	0	6			WD					WD	WD
DRB1*03:33	03:33	HLA02870		0	1	0	0	0	0	0	1									
DRB1*03:34	03:34	HLA02886		0	0	3	0	0	0	0	3									
DRB1*03:36	03:36	HLA02996		0	0	13	0	0	0	0	13			WD					WD	WD
DRB1*03:37	03:37	HLA03059		2	5	47	3	2	0	16	75		WD	WD				I	WD	I
DRB1*03:39	03:39	HLA03075		1	0	6	0	0	0	1	8			WD					WD	WD
**DRB1*03:41 total**	**03:41 total**			**1**	**0**	**0**	**0**	**0**	**0**	**0**	**1**									
DRB1*03:41	03:41			1	0	0	0	0	0	0	1									
DRB1*03:42	03:42	HLA03749		0	0	5	0	0	0	1	6			WD					WD	WD
DRB1*03:43	03:43	HLA03836		0	0	10	0	0	0	0	10			WD					WD	WD
DRB1*03:44	03:44	HLA03842		0	0	14	1	0	0	0	15			WD					WD	WD
DRB1*03:45	03:45	HLA03843		0	0	1	0	0	0	0	1									
DRB1*03:46	03:46	HLA03844		0	0	1	0	3	0	0	4									
DRB1*03:47	03:47	HLA03859		0	0	16	0	0	0	0	16			WD					WD	WD
DRB1*03:48	03:48	HLA03869		0	0	3	0	0	0	0	3									

Supplemental Table 11: HLA-DRB1 Allele Summary [a]				Allele Count by Population Group [b]								3.0.0 CIWD Category by Population Group [c]								
**Allele**	**Genomic Typing**	**Allele ID**	**G group**	**AFA**	**API**	**EURO**	**MENA**	**HIS**	**NAM**	**UNK**	**Total**	**AFA**	**API**	**EURO**	**MENA**	**HIS**	**NAM**	**UNK**	**Total**	**Highest Frequency**
DRB1*03:49	03:49	HLA04355		0	0	1	0	0	0	0	1									
DRB1*03:50	03:50	HLA04386		0	0	1	0	0	0	0	1									
DRB1*03:51	03:51	HLA04411		0	3	0	0	0	0	0	3									
DRB1*03:54	03:54	HLA05132		0	0	2	0	0	0	0	2									
DRB1*03:55	03:55	HLA05361		0	0	1	0	0	0	0	1									
DRB1*03:58	03:58	HLA05875		0	0	5	0	0	0	3	8			WD					WD	WD
DRB1*03:59	03:59	HLA05949		0	1	0	0	0	0	0	1									
DRB1*03:60	03:60	HLA06307		0	0	1	0	0	0	0	1									
DRB1*03:62	03:62	HLA06494		2	0	0	0	0	0	1	3									
DRB1*03:63	03:63	HLA06495		0	0	1	0	0	0	0	1									
DRB1*03:65	03:65	HLA06908		0	0	2	1	0	0	0	3									
DRB1*03:67N	03:67N	HLA07156		0	0	2	0	0	0	0	2									
DRB1*03:68N	03:68N	HLA07157		0	0	1	0	0	0	0	1									
DRB1*03:69	03:69	HLA07167		0	0	5	0	0	0	0	5			WD					WD	WD
DRB1*03:70	03:70	HLA07169		0	0	2	0	0	0	0	2									
**DRB1*03:71 total**	**03:71 total**			**0**	**1**	**0**	**0**	**0**	**0**	**1**	**2**									
DRB1*03:71	03:71			0	1	0	0	0	0	1	2									
DRB1*03:72	03:72	HLA07253		0	0	3	0	0	0	0	3									
DRB1*03:73	03:73	HLA07255		0	0	11	0	0	0	1	12			WD					WD	WD
DRB1*03:74	03:74	HLA07264		3	0	0	0	0	0	0	3									
DRB1*03:75	03:75	HLA07311		0	0	1	0	0	0	0	1									
DRB1*03:76	03:76	HLA07322		0	0	9	0	0	0	0	9			WD					WD	WD
DRB1*03:79	03:79	HLA07508		1	2	1	0	0	0	0	4									
DRB1*03:81	03:81	HLA04406		0	0	1	0	0	0	0	1									
DRB1*03:84	03:84	HLA08244		0	0	8	0	0	0	0	8			WD					WD	WD
DRB1*03:87	03:87	HLA08854		0	0	3	0	0	0	0	3									
DRB1*03:88	03:88	HLA08875		1	0	0	0	0	0	1	2									
DRB1*03:89	03:89	HLA09063		0	0	2	0	0	0	0	2									
DRB1*03:90	03:90	HLA09214		1	0	2	0	0	1	0	4									
DRB1*03:92	03:92	HLA09759		0	0	2	0	0	0	0	2									
DRB1*03:93	03:93	HLA09761		0	0	7	0	0	0	0	7			WD					WD	WD

Supplemental Table 11: HLA-DRB1 Allele Summary [a]				Allele Count by Population Group [b]								3.0.0 CIWD Category by Population Group [c]								
Allele	Genomic Typing	Allele ID	G group	AFA	API	EURO	MENA	HIS	NAM	UNK	Total	AFA	API	EURO	MENA	HIS	NAM	UNK	Total	Highest Frequency
DRB1*03:95	03:95	HLA10911		0	0	1	0	0	0	0	1									
DRB1*03:96	03:96	HLA11061		0	0	2	0	0	0	0	2									
DRB1*03:99	03:99	HLA11166		0	1	0	0	0	0	0	1									
**DRB1*03:100 total**	**03:100 total**			**0**	**0**	**5**	**0**	**0**	**0**	**0**	**5**			**WD**					**WD**	**WD**
DRB1*03:100:01	03:100:01	HLA11167		0	0	1	0	0	0	0	1									
DRB1*03:100:02	03:100:02	HLA11376		0	0	4	0	0	0	0	4									
DRB1*03:101	03:101	HLA11175		0	1	0	0	0	0	0	1									
DRB1*03:103	03:103	HLA11178		1	0	0	0	0	0	0	1									
DRB1*03:105	03:105	HLA11608		0	0	2	0	0	0	0	2									
DRB1*03:108	03:108	HLA11897		0	0	2	0	0	0	0	2									
DRB1*03:110	03:110	HLA11959		0	0	1	0	0	0	0	1									
DRB1*03:115	03:115	HLA12416		1	0	0	0	0	0	0	1									
DRB1*03:118	03:118	HLA12829		0	0	1	0	0	0	0	1									
DRB1*03:121	03:121	HLA13690		0	0	1	0	0	0	0	1									
DRB1*03:138	03:138	HLA16791		0	1	0	0	0	0	0	1									
DRB1*03:CODE	03:CODE			2986	1592	59527	1572	4316	297	6347	76637	NA	NA	NA	NA	NA	NA	NA	NA	NA
**DRB1*04:01 total**	**04:01 total**			**6707**	**14697**	**881988**	**5693**	**14010**	**2258**	**71870**	**997223**	**C**	**C**	**C**	**C**	**C**	**C**	**C**	**C**	**C**
DRB1*04:01	04:01			591	260	43897	309	1968	177	3819	51021	C	C	C	C	C	C	C	C	C
DRB1*04:01P	04:01P			0	0	56	0	0	0	0	56			WD					WD	WD
**DRB1*04:01:01G total**	**04:01:01G total**			**6116**	**14402**	**837921**	**5382**	**12040**	**2081**	**68043**	**945985**	**C**	**C**	**C**	**C**	**C**	**C**	**C**	**C**	**C**
DRB1*04:01:01G	04:01:01G		04:01:01G	519	9838	183982	834	1431	121	14730	211455	C	C	C	C	C	C	C	C	C
DRB1*04:01:01	04:01:01		04:01:01G	5597	4564	653894	4548	10608	1960	53310	734481	C	C	C	C	C	C	C	C	C
DRB1*04:01:01:01	04:01:01:01	HLA00685	04:01:01G	0	0	34	0	1	0	0	35			WD					WD	WD
DRB1*04:01:01:02	04:01:01:02	HLA15166	04:01:01G	0	0	11	0	0	0	3	14			WD					WD	WD
DRB1*04:01:02	04:01:02	HLA00686		0	35	50	2	2	0	0	89		I	WD					WD	I
DRB1*04:01:03	04:01:03	HLA03066		0	0	23	0	0	0	5	28			WD				WD	WD	WD
DRB1*04:01:04	04:01:04	HLA04661		0	0	3	0	0	0	0	3									
DRB1*04:01:05	04:01:05	HLA04663		0	0	21	0	0	0	0	21			WD					WD	WD
DRB1*04:01:06	04:01:06	HLA04664		0	0	5	0	0	0	0	5			WD					WD	WD
DRB1*04:01:07	04:01:07	HLA06490		0	0	2	0	0	0	0	2									
DRB1*04:01:08	04:01:08	HLA07154		0	0	4	0	0	0	0	4									

Supplemental Table 11: HLA-DRB1 Allele Summary [a]				Allele Count by Population Group [b]								3.0.0 CIWD Category by Population Group [c]								
Allele	Genomic Typing	Allele ID	G group	AFA	API	EURO	MENA	HIS	NAM	UNK	Total	AFA	API	EURO	MENA	HIS	NAM	UNK	Total	Highest Frequency
DRB1*04:01:09	04:01:09	HLA08236		0	0	1	0	0	0	0	1									
DRB1*04:01:10	04:01:10	HLA08797		0	0	3	0	0	0	0	3									
DRB1*04:01:12	04:01:12	HLA09057		0	0	1	0	0	0	0	1									
DRB1*04:01:13	04:01:13	HLA09757		0	0	1	0	0	0	0	1									
DRB1*04:01:14	04:01:14	HLA10662		0	0	0	0	0	0	1	1									
DRB1*04:01:15	04:01:15	HLA11371		0	0	0	0	0	0	1	1									
DRB1*04:01:17	04:01:17	HLA14784		0	0	0	0	0	0	1	1									
**DRB1*04:02 total**	**04:02 total**			**644**	**3951**	**110655**	**16010**	**8726**	**558**	**27469**	**168013**	**C**	**C**	**C**	**C**	**C**	**C**	**C**	**C**	**C**
DRB1*04:02	04:02			64	163	11362	1113	1291	57	1674	15724	C	C	C	C	C	C	C	C	C
DRB1*04:02P	04:02P			0	0	35	2	0	0	0	37			WD					WD	WD
DRB1*04:02:01	04:02:01	HLA00687		580	3788	99258	14895	7435	501	25795	152252	C	C	C	C	C	C	C	C	C
**DRB1*04:03 total**	**04:03 total**			**1263**	**63675**	**89315**	**18935**	**11758**	**952**	**22483**	**208381**	**C**	**C**	**C**	**C**	**C**	**C**	**C**	**C**	**C**
DRB1*04:03	04:03			59	720	4526	868	1243	65	754	8235	C	C	C	C	C	C	C	C	C
DRB1*04:03P	04:03P			0	0	62	0	1	0	0	63			WD					WD	WD
**DRB1*04:03:01G total**	**04:03:01G total**			**1204**	**62916**	**84646**	**18060**	**10514**	**887**	**21725**	**199952**	**C**	**C**	**C**	**C**	**C**	**C**	**C**	**C**	**C**
DRB1*04:03:01G	04:03:01G		04:03:01G	15	8215	1672	166	392	8	1771	12239	WD	C	C	C	C	C	C	C	C
DRB1*04:03:01	04:03:01		04:03:01G	1189	54700	82972	17891	10122	879	19953	187706	C	C	C	C	C	C	C	C	C
DRB1*04:218	04:218	HLA16045	04:03:01G	0	1	2	3	0	0	1	7								WD	WD
DRB1*04:03:02	04:03:02	HLA01009		0	4	15	1	0	0	2	22			WD					WD	WD
DRB1*04:03:03	04:03:03	HLA02717		0	0	54	3	0	0	1	58			WD					WD	WD
DRB1*04:03:04	04:03:04	HLA03172		0	28	0	2	0	0	0	30		I						WD	I
DRB1*04:03:05	04:03:05	HLA04660		0	0	4	0	0	0	0	4									
DRB1*04:03:08	04:03:08	HLA07506		0	5	6	0	0	0	0	11		WD	WD					WD	WD
DRB1*04:03:09	04:03:09	HLA10907		0	1	2	1	0	0	0	4									
DRB1*04:03:10	04:03:10	HLA11162		0	0	0	0	0	0	1	1									
DRB1*04:03:11	04:03:11	HLA14179		0	1	0	0	0	0	0	1									
**DRB1*04:04 total**	**04:04 total**			**3221**	**15656**	**322583**	**6681**	**30979**	**2385**	**39410**	**420915**	**C**	**C**	**C**	**C**	**C**	**C**	**C**	**C**	**C**
DRB1*04:04	04:04			184	624	20485	525	3519	164	2455	27956	C	C	C	C	C	C	C	C	C
DRB1*04:04P	04:04P			0	0	33	1	0	0	1	35			WD					WD	WD
DRB1*04:04:01	04:04:01	HLA00689		3026	15027	302057	6155	27459	2221	36953	392898	C	C	C	C	C	C	C	C	C
DRB1*04:04:02	04:04:02	HLA04039		0	2	1	0	1	0	0	4									

Supplemental Table 11: HLA-DRB1 Allele Summary [a]				Allele Count by Population Group [b]								3.0.0 CIWD Category by Population Group [c]								
Allele	Genomic Typing	Allele ID	G group	AFA	API	EURO	MENA	HIS	NAM	UNK	Total	AFA	API	EURO	MENA	HIS	NAM	UNK	Total	Highest Frequency
DRB1*04:04:03	04:04:03	HLA04659		1	0	3	0	0	0	0	4									
DRB1*04:04:04	04:04:04	HLA04662		9	0	0	0	0	0	0	9	WD							WD	WD
DRB1*04:04:05	04:04:05	HLA04710		0	0	1	0	0	0	0	1									
DRB1*04:04:06	04:04:06	HLA06489		0	3	0	0	0	0	0	3									
DRB1*04:04:07	04:04:07	HLA07551		1	0	1	0	0	0	1	3									
DRB1*04:04:08	04:04:08	HLA08851		0	0	1	0	0	0	0	1									
DRB1*04:04:09	04:04:09	HLA11160		0	0	1	0	0	0	0	1									
**DRB1*04:05 total**	**04:05 total**			**5878**	**27766**	**56671**	**8555**	**13264**	**1471**	**17122**	**130727**	**C**	**C**	**C**	**C**	**C**	**C**	**C**	**C**	**C**
DRB1*04:05	04:05			617	1744	4429	382	2006	111	1358	10647	C	C	C	C	C	C	C	C	C
DRB1*04:05P	04:05P			1	0	45	1	1	0	0	48			WD					WD	WD
**DRB1*04:05:01G total**	**04:05:01G total**			**5248**	**26005**	**52115**	**8170**	**11003**	**1338**	**15687**	**119566**	**C**	**C**	**C**	**C**	**C**	**C**	**C**	**C**	**C**
DRB1*04:05:01G	04:05:01G		04:05:01G	0	286	47	2	47	0	33	415		C	WD		I		I	I	C
DRB1*04:05:01	04:05:01		04:05:01G	5248	25719	52068	8168	10956	1338	15654	119151	C	C	C	C	C	C	C	C	C
DRB1*04:05:03	04:05:03	HLA01551		0	0	30	1	0	0	5	36			WD				WD	WD	WD
DRB1*04:05:04	04:05:04	HLA01605		12	0	47	1	246	20	68	394	WD		WD		C	C	I	I	C
DRB1*04:05:05	04:05:05	HLA03055		0	15	3	0	0	0	1	19		I						WD	I
DRB1*04:05:09	04:05:09	HLA04654		0	0	2	0	8	2	3	15					I			WD	I
DRB1*04:05:13	04:05:13	HLA07300		0	2	0	0	0	0	0	2									
**DRB1*04:06 total**	**04:06 total**			**195**	**9368**	**5811**	**1335**	**1116**	**49**	**2951**	**20825**	**C**	**C**	**C**	**C**	**C**	**C**	**C**	**C**	**C**
DRB1*04:06	04:06			105	3134	1939	424	526	23	1949	8100	C	C	C	C	C	C	C	C	C
DRB1*04:06P	04:06P			0	0	2	0	0	0	0	2									
**DRB1*04:06:01G total**	**04:06:01G total**			**90**	**6234**	**3870**	**911**	**590**	**26**	**1002**	**12723**	**C**	**C**	**C**	**C**	**C**	**C**	**C**	**C**	**C**
DRB1*04:06:01G	04:06:01G		04:06:01G	5	3169	308	374	162	4	415	4437	WD	C	I	C	C		C	C	C
DRB1*04:06:01	04:06:01	HLA00692	04:06:01G	6	2986	166	81	10	4	425	3678	WD	C	I	C	I		C	C	C
DRB1*04:06:02	04:06:02	HLA02172	04:06:01G	79	79	3396	456	418	18	162	4608	C	I	C	C	C	C	C	C	C
**DRB1*04:07 total**	**04:07 total**			**1835**	**2542**	**93549**	**2146**	**44487**	**3642**	**17462**	**165663**	**C**	**C**	**C**	**C**	**C**	**C**	**C**	**C**	**C**
DRB1*04:07	04:07			2	2	343	0	34	6	36	423			I		I	WD	I	I	I
DRB1*04:07P	04:07P			0	0	74	0	2	0	0	76			WD					WD	WD
**DRB1*04:07:01G total**	**04:07:01G total**			**1798**	**2538**	**93048**	**2145**	**44205**	**3602**	**17253**	**164589**	**C**	**C**	**C**	**C**	**C**	**C**	**C**	**C**	**C**
DRB1*04:07:01G	04:07:01G		04:07:01G	1748	2465	90525	2078	42674	3431	16761	159682	C	C	C	C	C	C	C	C	C
DRB1*04:07:01	04:07:01		04:07:01G	49	72	2519	67	1453	163	481	4804	C	I	C	C	C	C	C	C	C

Supplemental Table 11: HLA-DRB1 Allele Summary [a]				Allele Count by Population Group [b]								3.0.0 CIWD Category by Population Group [c]								
Allele	Genomic Typing	Allele ID	G group	AFA	API	EURO	MENA	HIS	NAM	UNK	Total	AFA	API	EURO	MENA	HIS	NAM	UNK	Total	Highest Frequency
DRB1*04:92	04:92	HLA05868	04:07:01G	1	1	4	0	78	8	11	103					C	C	WD	WD	C
DRB1*04:07:02	04:07:02	HLA01453		4	0	2	0	6	3	2	17					WD			WD	WD
DRB1*04:07:03	04:07:03	HLA01706		30	2	82	1	231	27	167	540	WD		WD		C	C	C	I	C
DRB1*04:07:04	04:07:04	HLA04658		1	0	0	0	9	4	4	18					I			WD	I
**DRB1*04:08 total**	**04:08 total**			**357**	**3461**	**55250**	**1158**	**1618**	**119**	**4373**	**66336**	**C**	**C**	**C**	**C**	**C**	**C**	**C**	**C**	**C**
DRB1*04:08	04:08			29	115	3994	88	242	14	318	4800	WD	I	C	C	C	C	C	C	C
DRB1*04:08P	04:08P			0	0	3	0	0	0	0	3									
DRB1*04:08:01	04:08:01	HLA00694		328	3345	51250	1069	1376	105	4055	61528	C	C	C	C	C	C	C	C	C
DRB1*04:08:02	04:08:02	HLA04008		0	1	3	1	0	0	0	5								WD	WD
DRB1*04:09	04:09	HLA00695		11	59	381	3	31	2	64	551	WD	I	I		I		I	I	I
**DRB1*04:10 total**	**04:10 total**			**103**	**3993**	**991**	**318**	**1736**	**172**	**899**	**8212**	**C**	**C**	**I**	**C**	**C**	**C**	**C**	**C**	**C**
DRB1*04:10	04:10			54	1007	514	139	1023	93	466	3296	C	C	I	C	C	C	C	C	C
DRB1*04:10P	04:10P			0	0	1	0	0	0	0	1									
**DRB1*04:10:01G total**	**04:10:01G total**			**48**	**2985**	**476**	**179**	**713**	**79**	**433**	**4913**	**C**	**C**	**I**	**C**	**C**	**C**	**C**	**C**	**C**
DRB1*04:10:01G	04:10:01G		04:10:01G	7	2421	248	87	231	17	216	3227	WD	C	I	C	C	C	C	C	C
DRB1*04:10:01	04:10:01	HLA00696	04:10:01G	32	555	227	90	482	62	206	1654	WD	C	I	C	C	C	C	C	C
DRB1*04:10:03	04:10:03	HLA11952	04:10:01G	0	9	0	0	0	0	9	18		WD					WD	WD	WD
DRB1*04:201	04:201	HLA14002	04:10:01G	9	0	1	2	0	0	2	14	WD							WD	WD
DRB1*04:10:02	04:10:02	HLA07145		1	1	0	0	0	0	0	2									
**DRB1*04:11 total**	**04:11 total**			**469**	**143**	**1848**	**37**	**13866**	**1600**	**3299**	**21262**	**C**	**C**	**C**	**WD**	**C**	**C**	**C**	**C**	**C**
DRB1*04:11	04:11			77	17	662	4	2646	250	679	4335	C	I	I		C	C	C	C	C
DRB1*04:11:01	04:11:01	HLA00697		391	126	1186	33	11215	1350	2614	16915	C	I	I	WD	C	C	C	C	C
DRB1*04:11:02	04:11:02	HLA08241		0	0	0	0	3	0	0	3									
DRB1*04:11:03	04:11:03	HLA09311		1	0	0	0	2	0	6	9							WD	WD	WD
DRB1*04:13	04:13	HLA00699		0	93	169	144	2	3	22	433		I	I	C			I	I	C
DRB1*04:14	04:14	HLA00700		0	1	389	1	2	0	25	418			I				I	I	I
DRB1*04:15	04:15	HLA00701		0	0	108	2	3	0	10	123			WD				WD	WD	WD
DRB1*04:16	04:16	HLA00702		1	0	41	0	3	0	1	46			WD					WD	WD
**DRB1*04:17 total**	**04:17 total**			**12**	**4**	**31**	**0**	**186**	**11**	**79**	**323**	**WD**		**WD**		**C**	**C**	**I**	**I**	**C**
DRB1*04:17	04:17			1	0	9	0	29	0	23	62			WD		I		I	WD	I
DRB1*04:17:01	04:17:01	HLA00703		0	0	15	0	42	0	28	85			WD		I		I	WD	I

Supplemental Table 11: HLA-DRB1 Allele Summary [a]				Allele Count by Population Group [b]								3.0.0 CIWD Category by Population Group [c]								
Allele	Genomic Typing	Allele ID	G group	AFA	API	EURO	MENA	HIS	NAM	UNK	Total	AFA	API	EURO	MENA	HIS	NAM	UNK	Total	Highest Frequency
DRB1*04:17:02	04:17:02	HLA04408		11	4	7	0	115	11	28	176	WD		WD		C	C	I	I	C
DRB1*04:18	04:18	HLA00704		0	439	1	2	0	2	7	451		C					WD	I	C
DRB1*04:19	04:19	HLA00705		0	0	13	0	13	1	3	30			WD		I			WD	I
DRB1*04:20	04:20	HLA00706		0	0	2	0	0	0	1	3									
DRB1*04:21	04:21	HLA00707		0	1	15	0	0	0	0	16			WD					WD	WD
DRB1*04:22	04:22	HLA00708		0	0	1	1	0	0	1	3									
DRB1*04:23	04:23	HLA00709		2	0	24	0	27	0	4	57			WD		I			WD	I
DRB1*04:24	04:24	HLA00710		0	1	0	0	0	0	0	1									
DRB1*04:25	04:25	HLA00711		1	0	2	0	9	0	3	15					I			WD	I
DRB1*04:26	04:26	HLA00712		0	0	73	0	0	0	24	97			WD				I	WD	I
DRB1*04:27	04:27	HLA00713		0	1	4	0	0	0	2	7								WD	WD
DRB1*04:31	04:31	HLA00717		0	0	7	0	0	0	0	7			WD					WD	WD
DRB1*04:33	04:33	HLA01088		0	0	26	0	0	1	2	29			WD					WD	WD
DRB1*04:34	04:34	HLA01167		0	0	19	0	0	0	2	21			WD					WD	WD
DRB1*04:35	04:35	HLA01235		4	3	44	0	1	0	6	58			WD				WD	WD	WD
DRB1*04:36	04:36	HLA01242		0	5	1	0	0	0	0	6		WD						WD	WD
DRB1*04:37	04:37	HLA01338		0	1	18	0	11	0	12	42			WD		I		WD	WD	I
DRB1*04:38	04:38	HLA01345		3	3	192	0	6	0	39	243			I		WD		I	I	I
DRB1*04:39	04:39	HLA01458		0	0	22	0	0	0	2	24			WD					WD	WD
DRB1*04:40	04:40	HLA01454		1	0	75	0	0	0	9	85			WD				WD	WD	WD
DRB1*04:41	04:41	HLA01459		0	39	2	0	1	0	14	56		I					I	WD	I
DRB1*04:42	04:42	HLA01457		0	6	13	0	0	0	0	19		WD	WD					WD	WD
DRB1*04:43	04:43	HLA01499		0	0	16	0	0	0	4	20			WD					WD	WD
**DRB1*04:44 total**	**04:44 total**			**0**	**1**	**9**	**0**	**0**	**0**	**0**	**10**			**WD**					**WD**	**WD**
DRB1*04:44	04:44			0	0	7	0	0	0	0	7			WD					WD	WD
DRB1*04:44:01	04:44:01	HLA01601		0	1	2	0	0	0	0	3									
DRB1*04:50	04:50	HLA01817		0	2	124	1	1	0	5	133			I				WD	WD	I
DRB1*04:51	04:51	HLA02039		0	8	0	0	0	0	0	8		WD						WD	WD
DRB1*04:52	04:52	HLA02054		0	0	4	0	0	0	0	4									
**DRB1*04:53 total**	**04:53 total**			**0**	**0**	**1**	**0**	**0**	**0**	**0**	**1**									
DRB1*04:53	04:53			0	0	1	0	0	0	0	1									

Supplemental Table 11: HLA-DRB1 Allele Summary [a]				Allele Count by Population Group [b]								3.0.0 CIWD Category by Population Group [c]								
Allele	Genomic Typing	Allele ID	G group	AFA	API	EURO	MENA	HIS	NAM	UNK	Total	AFA	API	EURO	MENA	HIS	NAM	UNK	Total	Highest Frequency
DRB1*04:54	04:54	HLA02305		0	0	0	0	19	0	4	23					I			WD	I
DRB1*04:55	04:55	HLA02306		0	0	0	0	1	0	0	1									
**DRB1*04:56 total**	**04:56 total**			**0**	**6**	**39**	**0**	**1**	**1**	**2**	**49**		**WD**	**WD**					**WD**	**WD**
DRB1*04:56	04:56			0	3	14	0	1	0	0	18			WD					WD	WD
DRB1*04:56:01	04:56:01	HLA02314		0	3	25	0	0	1	0	29			WD					WD	WD
DRB1*04:56:02	04:56:02	HLA11068		0	0	0	0	0	0	2	2									
DRB1*04:59	04:59	HLA02580		0	1	17	3	0	0	0	21			WD					WD	WD
DRB1*04:60	04:60	HLA02604		0	1	13	0	3	0	2	19			WD					WD	WD
DRB1*04:62	04:62	HLA02726		0	24	1	0	0	0	0	25		I						WD	I
DRB1*04:64	04:64	HLA02804		0	0	7	0	0	0	1	8			WD					WD	WD
DRB1*04:68	04:68	HLA03070		0	0	0	0	7	0	1	8					WD			WD	WD
DRB1*04:70	04:70	HLA03073		0	0	24	0	0	0	0	24			WD					WD	WD
DRB1*04:71	04:71	HLA03074		0	0	79	0	0	0	0	79			WD					WD	WD
**DRB1*04:72 total**	**04:72 total**			**0**	**0**	**58**	**0**	**1**	**0**	**10**	**69**			**WD**				**WD**	**WD**	**WD**
DRB1*04:72	04:72			0	0	6	0	0	0	3	9			WD					WD	WD
DRB1*04:72:01	04:72:01	HLA03158		0	0	51	0	1	0	7	59			WD				WD	WD	WD
DRB1*04:72:02	04:72:02	HLA04673		0	0	1	0	0	0	0	1									
DRB1*04:74	04:74	HLA03296		0	0	0	0	0	1	1	2									
DRB1*04:75	04:75	HLA03371		0	0	1	0	0	0	2	3									
DRB1*04:76	04:76	HLA03372		0	0	7	0	0	0	0	7			WD					WD	WD
DRB1*04:79	04:79	HLA03993		0	0	0	0	6	0	2	8					WD			WD	WD
DRB1*04:80	04:80	HLA03998		0	2	0	0	0	0	0	2									
DRB1*04:83	04:83	HLA04036		0	0	7	0	1	0	0	8			WD					WD	WD
DRB1*04:84	04:84	HLA04040		0	0	0	0	0	0	1	1									
DRB1*04:87	04:87	HLA04383		0	2	0	0	0	0	2	4									
DRB1*04:88	04:88	HLA04384		0	15	25	0	0	0	1	41		I	WD					WD	I
DRB1*04:89	04:89	HLA04672		0	4	0	0	0	0	0	4									
**DRB1*04:94N total**	**04:94N total**			**0**	**0**	**0**	**0**	**0**	**0**	**1**	**1**									
DRB1*04:94:01N	04:94:01N	HLA06051		0	0	0	0	0	0	1	1									
**DRB1*04:95 total**	**04:95 total**			**0**	**6**	**1**	**1**	**0**	**0**	**3**	**11**		**WD**						**WD**	**WD**
DRB1*04:95	04:95			0	1	0	0	0	0	1	2									

Supplemental Table 11: HLA-DRB1 Allele Summary [a]				Allele Count by Population Group [b]								3.0.0 CIWD Category by Population Group [c]								
**Allele**	**Genomic Typing**	**Allele ID**	**G group**	**AFA**	**API**	**EURO**	**MENA**	**HIS**	**NAM**	**UNK**	**Total**	**AFA**	**API**	**EURO**	**MENA**	**HIS**	**NAM**	**UNK**	**Total**	**Highest Frequency**
DRB1*04:95:01	04:95:01	HLA06296		0	3	0	1	0	0	1	5								WD	WD
DRB1*04:95:02	04:95:02	HLA10160		0	2	1	0	0	0	1	4									
DRB1*04:96	04:96	HLA06488		0	1	39	5	0	0	0	45			WD	WD				WD	WD
**DRB1*04:98 total**	**04:98 total**			**0**	**0**	**2**	**0**	**0**	**0**	**0**	**2**									
DRB1*04:98:02	04:98:02	HLA07312		0	0	2	0	0	0	0	2									
DRB1*04:99	04:99	HLA06845		0	1	0	0	0	0	0	1									
DRB1*04:101	04:101	HLA06875		0	0	1	3	2	0	2	8								WD	WD
DRB1*04:102	04:102	HLA07088		0	0	0	0	2	0	0	2									
**DRB1*04:105 total**	**04:105 total**			**0**	**17**	**6**	**0**	**0**	**0**	**1**	**24**		**I**	**WD**					**WD**	**I**
DRB1*04:105	04:105			0	0	1	0	0	0	0	1									
DRB1*04:105:01	04:105:01	HLA07166		0	0	4	0	0	0	1	5								WD	WD
DRB1*04:105:02	04:105:02	HLA07870		0	17	1	0	0	0	0	18		I						WD	I
DRB1*04:106	04:106	HLA07250		1	0	0	0	0	0	0	1									
DRB1*04:107	04:107	HLA07352		0	1	2	0	0	0	0	3									
DRB1*04:112	04:112	HLA07935		0	0	2	0	0	0	0	2									
DRB1*04:113	04:113	HLA07936		0	0	1	2	0	0	0	3									
DRB1*04:114	04:114	HLA08121		0	0	1	0	0	0	0	1									
DRB1*04:116	04:116	HLA08233		0	0	3	0	1	0	0	4									
DRB1*04:117	04:117	HLA08234		0	0	1	0	0	0	0	1									
DRB1*04:119N	04:119N	HLA08237		0	0	1	0	0	0	0	1									
DRB1*04:122	04:122	HLA08240		0	1	0	0	0	0	0	1									
DRB1*04:123	04:123	HLA08242		0	0	2	0	0	0	0	2									
DRB1*04:127	04:127	HLA08548		0	0	3	0	0	0	7	10							WD	WD	WD
DRB1*04:128	04:128	HLA08794		0	0	1	0	0	0	0	1									
DRB1*04:129	04:129	HLA08795		0	0	1	0	1	0	0	2									
DRB1*04:130	04:130	HLA08796		0	0	1	0	0	0	0	1									
DRB1*04:132	04:132	HLA08799		0	0	2	0	0	0	0	2									
DRB1*04:136	04:136	HLA08843		0	4	0	0	0	0	0	4									
DRB1*04:138	04:138	HLA08846		0	0	1	0	0	0	0	1									
DRB1*04:139	04:139	HLA08847		0	0	4	0	0	0	0	4									
DRB1*04:140	04:140	HLA08848		0	0	1	0	0	0	1	2									

Supplemental Table 11: HLA-DRB1 Allele Summary [a]				Allele Count by Population Group [b]								3.0.0 CIWD Category by Population Group [c]								
Allele	Genomic Typing	Allele ID	G group	AFA	API	EURO	MENA	HIS	NAM	UNK	Total	AFA	API	EURO	MENA	HIS	NAM	UNK	Total	Highest Frequency
DRB1*04:142N	04:142N	HLA08850		0	0	1	0	0	0	0	1									
DRB1*04:143	04:143	HLA08852		0	0	1	0	0	0	0	1									
DRB1*04:144	04:144	HLA08853		0	0	0	0	0	0	1	1									
DRB1*04:145	04:145	HLA08881		0	0	2	0	0	0	0	2									
DRB1*04:148	04:148	HLA09055		0	2	0	0	0	0	0	2									
DRB1*04:149	04:149	HLA09056		0	1	0	0	0	0	0	1									
DRB1*04:150	04:150	HLA09058		0	0	1	0	0	0	0	1									
DRB1*04:152	04:152	HLA09105		0	1	0	0	0	0	0	1									
DRB1*04:155	04:155	HLA09210		0	0	1	0	0	0	0	1									
DRB1*04:156	04:156	HLA09211		0	1	0	0	0	0	0	1									
DRB1*04:157N	04:157N	HLA09306		0	0	0	0	0	0	1	1									
DRB1*04:158N	04:158N	HLA09334		0	0	1	0	1	0	0	2									
DRB1*04:162	04:162	HLA09756		0	1	0	0	0	0	0	1									
DRB1*04:163	04:163	HLA09758		0	1	0	0	0	0	0	1									
DRB1*04:164	04:164	HLA09915		0	0	1	0	0	0	0	1									
DRB1*04:168	04:168	HLA10308		0	0	1	0	0	0	0	1									
DRB1*04:170	04:170	HLA10554		0	0	0	0	2	0	0	2									
DRB1*04:177	04:177	HLA11740		0	0	2	0	0	0	0	2									
DRB1*04:179	04:179	HLA11978		0	0	0	2	0	0	0	2									
DRB1*04:182	04:182	HLA12215		0	0	1	0	0	0	0	1									
DRB1*04:183	04:183	HLA12216		0	1	0	0	0	0	0	1									
DRB1*04:190	04:190	HLA13271		0	0	2	0	0	0	0	2									
DRB1*04:192	04:192	HLA13685		0	0	1	0	0	0	0	1									
DRB1*04:195	04:195	HLA13817		0	2	0	0	0	0	0	2									
DRB1*04:198	04:198	HLA13891		0	1	0	0	0	0	0	1									
DRB1*04:203	04:203	HLA14563		0	0	0	0	1	0	0	1									
DRB1*04:206	04:206	HLA14566		0	3	0	0	0	0	0	3									
DRB1*04:209	04:209	HLA15074		0	0	1	0	0	0	0	1									
DRB1*04:CODE	04:CODE			719	1441	32772	326	5857	373	4788	46276	NA	NA	NA	NA	NA	NA	NA	NA	NA
**DRB1*07:01 total**	**07:01 total**			**38798**	**177782**	**1525694**	**44979**	**71563**	**7000**	**159540**	**2025356**	**C**	**C**	**C**	**C**	**C**	**C**	**C**	**C**	**C**
DRB1*07:01	07:01			4169	2820	87428	2649	9609	574	8145	115394	C	C	C	C	C	C	C	C	C

Supplemental Table 11: HLA-DRB1 Allele Summary [a]				Allele Count by Population Group [b]								3.0.0 CIWD Category by Population Group [c]								
Allele	Genomic Typing	Allele ID	G group	AFA	API	EURO	MENA	HIS	NAM	UNK	Total	AFA	API	EURO	MENA	HIS	NAM	UNK	Total	Highest Frequency
DRB1*07:01P	07:01P			2	7	1904	10	8	0	4	1935		WD	C	WD	I			C	C
**DRB1*07:01:01G total**	**07:01:01G total**			**34624**	**174948**	**1436318**	**42317**	**61946**	**6426**	**151385**	**1907964**	**C**	**C**	**C**	**C**	**C**	**C**	**C**	**C**	**C**
DRB1*07:01:01G	07:01:01G		07:01:01G	7208	131947	439690	24672	17591	1065	71829	694002	C	C	C	C	C	C	C	C	C
DRB1*07:01:01	07:01:01		07:01:01G	27412	42993	996131	17645	44347	5361	79550	1213439	C	C	C	C	C	C	C	C	C
DRB1*07:01:01:01	07:01:01:01	HLA00719	07:01:01G	0	3	179	0	0	0	0	182			I					I	I
DRB1*07:01:01:02	07:01:01:02	HLA03486	07:01:01G	0	0	115	0	0	0	0	115			WD					WD	WD
DRB1*07:01:01:03	07:01:01:03	HLA14836	07:01:01G	4	5	31	0	7	0	4	51		WD	WD		WD			WD	WD
DRB1*07:01:18	07:01:18	HLA13507	07:01:01G	0	0	4	0	1	0	0	5								WD	WD
DRB1*07:01:21	07:01:21	HLA15596	07:01:01G	0	0	4	0	0	0	0	4									
DRB1*07:34	07:34	HLA11772	07:01:01G	0	0	155	0	0	0	2	157			I					WD	I
DRB1*07:72	07:72	HLA15418	07:01:01G	0	0	9	0	0	0	0	9			WD					WD	WD
DRB1*07:01:02	07:01:02	HLA01089		0	0	19	0	0	0	1	20			WD					WD	WD
DRB1*07:01:04	07:01:04	HLA06847		0	0	1	0	0	0	0	1									
DRB1*07:01:05	07:01:05	HLA09665		0	0	11	0	0	0	1	12			WD					WD	WD
DRB1*07:01:06	07:01:06	HLA09668		0	0	2	3	0	0	1	6								WD	WD
DRB1*07:01:07	07:01:07	HLA09690		0	3	1	0	0	0	0	4									
DRB1*07:01:08	07:01:08	HLA11942		0	0	5	0	0	0	0	5			WD					WD	WD
DRB1*07:01:09	07:01:09	HLA13056		3	0	1	0	0	0	1	5								WD	WD
DRB1*07:01:15	07:01:15	HLA13069		0	0	3	0	0	0	2	5								WD	WD
DRB1*07:01:16	07:01:16	HLA13167		0	4	0	0	0	0	0	4									
DRB1*07:01:17	07:01:17	HLA13251		0	0	1	0	0	0	0	1									
DRB1*07:03	07:03	HLA00721		1	1255	13	7	0	1	19	1296		C	WD	WD			I	I	C
DRB1*07:05	07:05	HLA01415		0	0	137	0	5	1	19	162			I		WD		I	I	I
DRB1*07:06	07:06	HLA01514		0	0	1	0	0	0	0	1									
DRB1*07:07	07:07	HLA01620		0	0	186	0	3	0	9	198			I				WD	I	I
DRB1*07:10N	07:10N	HLA02203		1	0	119	0	0	0	3	123			WD					WD	WD
DRB1*07:11	07:11	HLA02495		0	0	22	0	57	2	12	93			WD		I		WD	WD	I
DRB1*07:12	07:12	HLA02735		0	2	0	0	0	0	0	2									
DRB1*07:13	07:13	HLA02912		0	28	2	1	0	0	2	33		I						WD	I
DRB1*07:14	07:14	HLA02932		0	0	2	0	0	0	1	3									
DRB1*07:15	07:15	HLA03136		0	0	1	0	5	0	2	8					WD			WD	WD

Supplemental Table 11: HLA-DRB1 Allele Summary [a]				Allele Count by Population Group [b]								3.0.0 CIWD Category by Population Group [c]								
Allele	Genomic Typing	Allele ID	G group	AFA	API	EURO	MENA	HIS	NAM	UNK	Total	AFA	API	EURO	MENA	HIS	NAM	UNK	Total	Highest Frequency
DRB1*07:16	07:16	HLA03550		0	1	6	0	31	0	4	42			WD		I			WD	I
DRB1*07:20	07:20	HLA06294		1	0	3	0	0	0	0	4									
DRB1*07:21	07:21	HLA06298		0	0	4	0	0	0	0	4									
DRB1*07:22	07:22	HLA07299		0	0	0	0	0	0	1	1									
DRB1*07:23	07:23	HLA09140		0	0	13	0	0	0	0	13			WD					WD	WD
DRB1*07:24	07:24	HLA09335		0	1	0	0	0	0	0	1									
DRB1*07:26N	07:26N	HLA09680		0	0	6	0	0	0	0	6			WD					WD	WD
DRB1*07:29	07:29	HLA10530		0	0	11	0	0	0	0	11			WD					WD	WD
DRB1*07:30	07:30	HLA11753		0	0	26	0	0	0	0	26			WD					WD	WD
DRB1*07:31	07:31	HLA11754		0	0	1	0	0	0	0	1									
DRB1*07:32	07:32	HLA11760		0	0	5	0	0	0	0	5			WD					WD	WD
DRB1*07:33	07:33	HLA11763		0	0	2	0	0	0	0	2									
DRB1*07:37	07:37	HLA13054		0	0	3	0	0	0	0	3									
DRB1*07:39	07:39	HLA13057		0	0	7	0	0	0	0	7			WD					WD	WD
DRB1*07:41	07:41	HLA13062		0	1	1	0	0	0	1	3									
DRB1*07:44	07:44	HLA13067		0	0	3	0	0	0	0	3									
DRB1*07:46	07:46	HLA13070		0	0	2	0	0	0	0	2									
DRB1*07:47	07:47	HLA13071		0	0	2	0	0	0	0	2									
DRB1*07:48	07:48	HLA13072		0	0	4	0	0	0	0	4									
DRB1*07:49	07:49	HLA13073		0	0	3	0	0	0	0	3									
DRB1*07:51	07:51	HLA13075		0	0	2	0	0	0	0	2									
DRB1*07:56	07:56	HLA13164		0	0	2	1	0	0	0	3									
DRB1*07:58N	07:58N	HLA13166		0	0	1	0	0	0	0	1									
DRB1*07:61	07:61	HLA13687		0	0	1	8	0	0	9	18				WD			WD	WD	WD
DRB1*07:64	07:64	HLA13886		0	0	3	0	0	0	0	3									
DRB1*07:65	07:65	HLA14047		0	2	1	0	0	0	0	3									
DRB1*07:67	07:67	HLA14355		0	0	1	0	0	0	0	1									
DRB1*07:77	07:77	HLA16786		0	1	0	0	0	0	0	1									
DRB1*07:CODE	07:CODE			712	555	19456	174	2904	134	1819	25754	NA	NA	NA	NA	NA	NA	NA	NA	NA
**DRB1*08:01 total**	**08:01 total**			**1357**	**3264**	**312103**	**2467**	**7747**	**762**	**24715**	**352415**	**C**	**C**	**C**	**C**	**C**	**C**	**C**	**C**	**C**
DRB1*08:01	08:01			868	579	96712	679	4952	599	10817	115206	C	C	C	C	C	C	C	C	C

Supplemental Table 11: HLA-DRB1 Allele Summary [a]				Allele Count by Population Group [b]								3.0.0 CIWD Category by Population Group [c]								
Allele	Genomic Typing	Allele ID	G group	AFA	API	EURO	MENA	HIS	NAM	UNK	Total	AFA	API	EURO	MENA	HIS	NAM	UNK	Total	Highest Frequency
DRB1*08:01P	08:01P			0	1	214	0	2	0	0	217			I					I	I
**DRB1*08:01:01G total**	**08:01:01G total**			**489**	**2683**	**215141**	**1788**	**2790**	**162**	**13888**	**236941**	**C**	**C**	**C**	**C**	**C**	**C**	**C**	**C**	**C**
DRB1*08:01:01G	08:01:01G		08:01:01G	435	2659	212680	1778	2444	140	13587	233723	C	C	C	C	C	C	C	C	C
DRB1*08:01:01	08:01:01	HLA00723	08:01:01G	54	24	2459	10	345	22	300	3214	C	I	C	WD	C	C	C	C	C
DRB1*08:77	08:77	HLA13774	08:01:01G	0	0	2	0	1	0	1	4									
DRB1*08:01:02	08:01:02	HLA01585		0	0	13	0	1	1	2	17			WD					WD	WD
DRB1*08:01:04	08:01:04	HLA03821		0	0	3	0	0	0	8	11							WD	WD	WD
DRB1*08:01:05	08:01:05	HLA04011		0	1	20	0	2	0	0	23			WD					WD	WD
**DRB1*08:02 total**	**08:02 total**			**740**	**8048**	**13290**	**440**	**48445**	**2960**	**13481**	**87404**	**C**	**C**	**C**	**C**	**C**	**C**	**C**	**C**	**C**
DRB1*08:02	08:02			92	412	2757	54	6508	372	2253	12448	C	C	C	C	C	C	C	C	C
DRB1*08:02P	08:02P			0	0	4	0	0	0	0	4									
**DRB1*08:02:01G total**	**08:02:01G total**			**644**	**7633**	**10508**	**383**	**41711**	**2581**	**11193**	**74653**	**C**	**C**	**C**	**C**	**C**	**C**	**C**	**C**	**C**
DRB1*08:02:01G	08:02:01G		08:02:01G	3	3955	419	13	363	5	238	4996		C	I	WD	C	WD	C	C	C
DRB1*08:02:01	08:02:01		08:02:01G	641	3678	10089	370	41348	2576	10955	69657	C	C	C	C	C	C	C	C	C
DRB1*08:02:02	08:02:02	HLA00725		0	0	3	2	5	0	3	13					WD			WD	WD
DRB1*08:02:03	08:02:03	HLA01416		4	3	18	1	221	7	32	286			WD		C	C	I	I	C
**DRB1*08:03 total**	**08:03 total**			**194**	**30377**	**27278**	**2687**	**1418**	**93**	**5395**	**67442**	**C**	**C**	**C**	**C**	**C**	**C**	**C**	**C**	**C**
DRB1*08:03	08:03			15	1371	1418	169	194	6	377	3550	WD	C	C	C	C	WD	C	C	C
DRB1*08:03P	08:03P			0	0	1	0	0	0	0	1									
**DRB1*08:03:02G total**	**08:03:02G total**			**179**	**29006**	**25826**	**2518**	**1224**	**87**	**5018**	**63858**	**C**	**C**	**C**	**C**	**C**	**C**	**C**	**C**	**C**
DRB1*08:03:02G	08:03:02G		08:03:02G	1	9238	1294	108	15	1	36	10693		C	C	C	I		I	C	C
DRB1*08:03:02	08:03:02		08:03:02G	178	19768	24532	2410	1209	86	4982	53165	C	C	C	C	C	C	C	C	C
DRB1*08:03:03	08:03:03	HLA07331		0	0	33	0	0	0	0	33			WD					WD	WD
**DRB1*08:04 total**	**08:04 total**			**20845**	**1008**	**26381**	**2782**	**5577**	**746**	**7501**	**64840**	**C**	**C**	**C**	**C**	**C**	**C**	**C**	**C**	**C**
DRB1*08:04	08:04			3034	58	3104	170	1075	89	1095	8625	C	I	C	C	C	C	C	C	C
DRB1*08:04P	08:04P			0	0	23	0	0	0	0	23			WD					WD	WD
DRB1*08:04:01	08:04:01	HLA00728		17800	941	23191	2611	4084	634	6303	55564	C	C	C	C	C	C	C	C	C
DRB1*08:04:02	08:04:02	HLA00729		0	0	1	0	0	0	0	1									
DRB1*08:04:03	08:04:03	HLA00730		0	1	0	0	19	4	5	29					I		WD	WD	I
DRB1*08:04:04	08:04:04	HLA01414		9	8	37	1	399	19	97	570	WD	WD	WD		C	C	I	I	C
DRB1*08:04:05	08:04:05	HLA04685		1	0	25	0	0	0	1	27			WD					WD	WD

Supplemental Table 11: HLA-DRB1 Allele Summary [a]				Allele Count by Population Group [b]								3.0.0 CIWD Category by Population Group [c]								
Allele	Genomic Typing	Allele ID	G group	AFA	API	EURO	MENA	HIS	NAM	UNK	Total	AFA	API	EURO	MENA	HIS	NAM	UNK	Total	Highest Frequency
DRB1*08:04:06	08:04:06	HLA06311		1	0	0	0	0	0	0	1									
DRB1*08:05	08:05	HLA00731		0	0	72	0	1	0	15	88			WD				I	WD	I
DRB1*08:06	08:06	HLA00732		1952	16	1916	130	626	73	681	5394	C	I	C	C	C	C	C	C	C
DRB1*08:07	08:07	HLA00733		29	1	489	1	827	73	671	2091	WD		I		C	C	C	C	C
DRB1*08:08	08:08	HLA00734		86	0	3	7	0	0	16	112	C			WD			I	WD	C
DRB1*08:09	08:09	HLA00735		0	841	34	3	6	0	56	940		C	WD		WD		I	I	C
DRB1*08:10	08:10	HLA00736		15	59	1031	68	107	9	133	1422	WD	I	I	C	C	C	C	I	C
DRB1*08:11	08:11	HLA00737		246	3	415	0	91	269	358	1382	C		I		C	C	C	I	C
DRB1*08:12	08:12	HLA00738		0	16	0	0	0	0	2	18		I						WD	I
DRB1*08:13	08:13	HLA00739		0	1	14	0	44	1	24	84			WD		I		I	WD	I
DRB1*08:14	08:14	HLA00740		0	22	0	0	0	0	0	22		I						WD	I
DRB1*08:16	08:16	HLA00742		0	0	7	0	0	0	1	8			WD					WD	WD
DRB1*08:17	08:17	HLA00743		0	1	280	1	0	0	10	292			I				WD	I	I
DRB1*08:18	08:18	HLA00744		0	84	45	3	29	0	33	194		I	WD		I		I	I	I
DRB1*08:19	08:19	HLA00745		0	86	1	1	0	0	1	89		I						WD	I
DRB1*08:20	08:20	HLA00746		6	0	1	0	0	0	3	10	WD							WD	WD
DRB1*08:21	08:21	HLA00747		0	1	0	0	0	0	0	1									
DRB1*08:22	08:22	HLA01282		0	0	4	0	0	0	2	6								WD	WD
**DRB1*08:24 total**	**08:24 total**			**0**	**0**	**2**	**1**	**1**	**0**	**0**	**4**									
DRB1*08:24	08:24			0	0	2	1	1	0	0	4									
DRB1*08:26	08:26	HLA01814		0	0	59	1	1	0	0	61			WD					WD	WD
DRB1*08:28	08:28	HLA01860		0	0	7	1	0	0	4	12			WD					WD	WD
**DRB1*08:30 total**	**08:30 total**			**0**	**1**	**0**	**0**	**0**	**0**	**1**	**2**									
DRB1*08:30:03	08:30:03	HLA06848		0	1	0	0	0	0	1	2									
DRB1*08:32	08:32	HLA02381		0	1	0	0	0	0	0	1									
DRB1*08:34	08:34	HLA03065		0	0	9	3	0	0	0	12			WD					WD	WD
DRB1*08:35	08:35	HLA03384		0	3	0	0	0	0	1	4									
**DRB1*08:36 total**	**08:36 total**			**0**	**3**	**0**	**0**	**0**	**0**	**0**	**3**									
DRB1*08:36	08:36			0	1	0	0	0	0	0	1									
DRB1*08:36:01	08:36:01	HLA03489		0	2	0	0	0	0	0	2									
DRB1*08:38	08:38	HLA04009		0	1	0	0	0	0	0	1									

Supplemental Table 11: HLA-DRB1 Allele Summary [a]				Allele Count by Population Group [b]								3.0.0 CIWD Category by Population Group [c]								
Allele	Genomic Typing	Allele ID	G group	AFA	API	EURO	MENA	HIS	NAM	UNK	Total	AFA	API	EURO	MENA	HIS	NAM	UNK	Total	Highest Frequency
DRB1*08:39	08:39	HLA04124		0	0	1	0	0	0	0	1									
DRB1*08:40	08:40	HLA04797		0	0	4	0	0	0	0	4									
DRB1*08:41	08:41	HLA05851		0	0	1	0	0	0	0	1									
DRB1*08:43	08:43	HLA06491		0	0	2	0	0	0	0	2									
DRB1*08:44	08:44	HLA06492		0	0	12	0	0	0	0	12			WD					WD	WD
**DRB1*08:45 total**	**08:45 total**			**0**	**0**	**1**	**0**	**0**	**0**	**0**	**1**									
DRB1*08:45:01	08:45:01	HLA06493		0	0	1	0	0	0	0	1									
DRB1*08:53	08:53	HLA09097		0	1	0	0	0	0	0	1									
DRB1*08:58	08:58	HLA11766		0	0	1	0	0	0	0	1									
DRB1*08:75	08:75	HLA13279		0	0	1	0	0	0	0	1									
DRB1*08:82	08:82	HLA15004		0	0	14	0	8	0	17	39			WD		I		I	WD	I
DRB1*08:CODE	08:CODE			206	438	3426	15	2425	102	872	7484	NA	NA	NA	NA	NA	NA	NA	NA	NA
**DRB1*09:01 total**	**09:01 total**			**10391**	**47140**	**109415**	**2818**	**8827**	**1125**	**19684**	**199400**	**C**	**C**	**C**	**C**	**C**	**C**	**C**	**C**	**C**
DRB1*09:01	09:01			256	461	1792	52	102	10	265	2938	C	C	C	C	C	C	C	C	C
DRB1*09:01P	09:01P			0	0	32	0	1	0	0	33			WD					WD	WD
**DRB1*09:01:02G total**	**09:01:02G total**			**10135**	**46679**	**107580**	**2766**	**8724**	**1115**	**19418**	**196417**	**C**	**C**	**C**	**C**	**C**	**C**	**C**	**C**	**C**
DRB1*09:01:02G	09:01:02G		09:01:02G	5864	32966	71588	1841	6449	621	13432	132761	C	C	C	C	C	C	C	C	C
DRB1*09:01:02	09:01:02		09:01:02G	4271	13712	35992	925	2275	494	5986	63655	C	C	C	C	C	C	C	C	C
DRB1*09:21	09:21	HLA09928	09:01:02G	0	1	0	0	0	0	0	1									
DRB1*09:01:06	09:01:06	HLA03994		0	0	11	0	0	0	0	11			WD					WD	WD
DRB1*09:01:11	09:01:11	HLA13168		0	0	0	0	0	0	1	1									
**DRB1*09:02 total**	**09:02 total**			**0**	**2**	**4**	**1**	**0**	**0**	**0**	**7**								**WD**	**WD**
DRB1*09:02	09:02			0	0	1	0	0	0	0	1									
DRB1*09:02:01	09:02:01	HLA01513		0	1	0	0	0	0	0	1									
DRB1*09:02:02	09:02:02	HLA03547		0	1	3	1	0	0	0	5								WD	WD
DRB1*09:04	09:04	HLA02018		0	18	1	1	0	0	0	20		I						WD	I
DRB1*09:06	09:06	HLA02682		1	2	12	0	52	3	13	83			WD		I		WD	WD	I
DRB1*09:07	09:07	HLA03301		0	1	0	0	0	0	0	1									
DRB1*09:10	09:10	HLA06036		0	1	0	0	0	0	0	1									
DRB1*09:20	09:20	HLA08429		0	1	104	0	0	0	0	105			WD					WD	WD
DRB1*09:23	09:23	HLA11770		0	0	0	0	0	0	1	1									

Supplemental Table 11: HLA-DRB1 Allele Summary [a]				Allele Count by Population Group [b]								3.0.0 CIWD Category by Population Group [c]								
Allele	Genomic Typing	Allele ID	G group	AFA	API	EURO	MENA	HIS	NAM	UNK	Total	AFA	API	EURO	MENA	HIS	NAM	UNK	Total	Highest Frequency
DRB1*09:27	09:27	HLA14176		0	8	1	0	0	0	0	9		WD						WD	WD
DRB1*09:CODE	09:CODE			562	1296	3362	139	507	49	695	6610	NA	NA	NA	NA	NA	NA	NA	NA	NA
**DRB1*10:01 total**	**10:01 total**			**7762**	**69978**	**103884**	**12255**	**10041**	**1231**	**20021**	**225172**	**C**	**C**	**C**	**C**	**C**	**C**	**C**	**C**	**C**
DRB1*10:01	10:01			691	1091	5733	517	1391	90	1045	10558	C	C	C	C	C	C	C	C	C
DRB1*10:01P	10:01P			0	0	100	7	1	0	0	108			WD	WD				WD	WD
**DRB1*10:01:01G total**	**10:01:01G total**			**7070**	**68803**	**97865**	**11725**	**8648**	**1141**	**18965**	**214217**	**C**	**C**	**C**	**C**	**C**	**C**	**C**	**C**	**C**
DRB1*10:01:01G	10:01:01G		10:01:01G	242	47522	12322	608	752	4	2824	64274	C	C	C	C	C		C	C	C
DRB1*10:01:01	10:01:01		10:01:01G	6824	21254	85316	11080	7869	1133	16122	149598	C	C	C	C	C	C	C	C	C
DRB1*10:01:01:01	10:01:01:01	HLA00750	10:01:01G	4	27	194	37	27	4	19	312		I	I	WD	I		I	I	I
DRB1*10:19	10:19	HLA15606	10:01:01G	0	0	33	0	0	0	0	33			WD					WD	WD
DRB1*10:01:02	10:01:02	HLA01214		0	2	158	0	1	0	9	170			I				WD	I	I
DRB1*10:01:03	10:01:03	HLA03427		0	1	21	0	0	0	0	22			WD					WD	WD
DRB1*10:01:04	10:01:04	HLA09670		0	0	5	0	0	0	1	6			WD					WD	WD
DRB1*10:01:07	10:01:07	HLA13272		0	0	1	0	0	0	0	1									
DRB1*10:01:08	10:01:08	HLA13273		1	81	0	5	0	0	1	88		I		WD				WD	I
DRB1*10:01:09	10:01:09	HLA14464		0	0	1	1	0	0	0	2									
DRB1*10:02	10:02	HLA03054		2	36	7	0	0	0	0	45		I	WD					WD	I
DRB1*10:03	10:03	HLA03391		0	2	0	1	0	0	0	3									
DRB1*10:06	10:06	HLA09672		0	7	1	0	0	0	0	8		WD						WD	WD
DRB1*10:11	10:11	HLA12814		0	0	3	0	0	0	0	3									
DRB1*10:12	10:12	HLA12815		0	0	2	0	0	0	0	2									
DRB1*10:13	10:13	HLA12817		0	0	3	1	0	0	0	4									
DRB1*10:15	10:15	HLA13591		0	1	0	0	0	0	0	1									
DRB1*10:16	10:16	HLA14049		0	2	0	0	0	0	0	2									
DRB1*10:CODE	10:CODE			300	421	2792	35	478	23	481	4530	NA	NA	NA	NA	NA	NA	NA	NA	NA
**DRB1*11:01 total**	**11:01 total**			**29102**	**64904**	**826728**	**29875**	**26609**	**2705**	**76847**	**1056770**	**C**	**C**	**C**	**C**	**C**	**C**	**C**	**C**	**C**
DRB1*11:01	11:01			1122	167	22098	692	535	57	1720	26391	C	C	C	C	C	C	C	C	C
DRB1*11:01P	11:01P			2	2	1914	4	6	0	6	1934			C		WD		WD	C	C
**DRB1*11:01:01G total**	**11:01:01G total**			**3976**	**64476**	**797472**	**27472**	**21397**	**1936**	**68587**	**985316**	**C**	**C**	**C**	**C**	**C**	**C**	**C**	**C**	**C**
DRB1*11:01:01G	11:01:01G		11:01:01G	2163	46810	235211	9113	12899	1208	55560	362964	C	C	C	C	C	C	C	C	C
DRB1*11:01:01	11:01:01		11:01:01G	1809	17635	560481	18349	8420	726	12974	620394	C	C	C	C	C	C	C	C	C

Supplemental Table 11: HLA-DRB1 Allele Summary [a]				Allele Count by Population Group [b]								3.0.0 CIWD Category by Population Group [c]								
Allele	Genomic Typing	Allele ID	G group	AFA	API	EURO	MENA	HIS	NAM	UNK	Total	AFA	API	EURO	MENA	HIS	NAM	UNK	Total	Highest Frequency
DRB1*11:01:01:01	11:01:01:01	HLA00751	11:01:01G	3	27	526	5	68	2	37	668		I	I	WD	I		I	I	I
DRB1*11:01:08	11:01:08	HLA03812	11:01:01G	1	4	1252	5	10	0	16	1288			C	WD	I		I	I	C
DRB1*11:01:29	11:01:29	HLA17342	11:01:01G	0	0	2	0	0	0	0	2									
DRB1*11:01:02	11:01:02	HLA00752		23990	234	5037	1684	4670	711	6513	42839	C	C	C	C	C	C	C	C	C
DRB1*11:01:03	11:01:03	HLA00753		2	13	66	1	0	0	2	84		WD	WD					WD	WD
DRB1*11:01:04	11:01:04	HLA01433		0	0	5	0	1	1	0	7			WD					WD	WD
DRB1*11:01:05	11:01:05	HLA01747		0	8	0	0	0	0	4	12		WD						WD	WD
DRB1*11:01:06	11:01:06	HLA02090		0	2	49	1	0	0	2	54			WD					WD	WD
DRB1*11:01:07	11:01:07	HLA02916		0	1	29	11	0	0	7	48			WD	WD			WD	WD	WD
DRB1*11:01:09	11:01:09	HLA03851		0	0	1	8	0	0	0	9				WD				WD	WD
DRB1*11:01:10	11:01:10	HLA03858		0	0	25	0	0	0	0	25			WD					WD	WD
DRB1*11:01:11	11:01:11	HLA04030		10	0	2	2	0	0	6	20	WD						WD	WD	WD
DRB1*11:01:12	11:01:12	HLA05141		0	1	3	0	0	0	0	4									
DRB1*11:01:13	11:01:13	HLA06483		0	0	15	0	0	0	0	15			WD					WD	WD
DRB1*11:01:16	11:01:16	HLA07263		0	0	11	0	0	0	0	11			WD					WD	WD
DRB1*11:01:18	11:01:18	HLA09069		0	0	1	0	0	0	0	1									
**DRB1*11:02 total**	**11:02 total**			**14257**	**216**	**30046**	**1296**	**7863**	**1007**	**7261**	**61946**	**C**	**C**	**C**	**C**	**C**	**C**	**C**	**C**	**C**
DRB1*11:02	11:02			1845	18	2288	28	1414	108	736	6437	C	I	C	WD	C	C	C	C	C
DRB1*11:02P	11:02P			0	0	6	0	0	0	0	6			WD					WD	WD
DRB1*11:02:01	11:02:01	HLA00754		12401	198	27751	1268	6449	899	6525	55491	C	C	C	C	C	C	C	C	C
DRB1*11:02:02	11:02:02	HLA02213		11	0	1	0	0	0	0	12	WD							WD	WD
**DRB1*11:03 total**	**11:03 total**			**298**	**541**	**106499**	**2810**	**3109**	**187**	**7018**	**120462**	**C**	**C**	**C**	**C**	**C**	**C**	**C**	**C**	**C**
DRB1*11:03	11:03			226	384	69512	1932	2107	149	4756	79066	C	C	C	C	C	C	C	C	C
DRB1*11:03P	11:03P			0	0	50	0	0	0	0	50			WD					WD	WD
DRB1*11:03:01	11:03:01	HLA00755		72	157	36937	878	1002	38	2262	41346	C	C	C	C	C	C	C	C	C
**DRB1*11:04 total**	**11:04 total**			**3326**	**12195**	**421999**	**40304**	**20519**	**1468**	**63240**	**563051**	**C**	**C**	**C**	**C**	**C**	**C**	**C**	**C**	**C**
DRB1*11:04	11:04			294	222	24288	2764	2885	103	2873	33429	C	C	C	C	C	C	C	C	C
DRB1*11:04P	11:04P			0	0	551	3	0	0	3	557			I					I	I
**DRB1*11:04:01G total**	**11:04:01G total**			**1721**	**11960**	**397019**	**37516**	**17532**	**1344**	**60080**	**527172**	**C**	**C**	**C**	**C**	**C**	**C**	**C**	**C**	**C**
DRB1*11:04:01G	11:04:01G		11:04:01G	16	2190	21117	1843	1879	19	16727	43791	WD	C	C	C	C	C	C	C	C
DRB1*11:04:01	11:04:01	HLA00756	11:04:01G	1705	9770	375822	35673	15653	1324	43353	483300	C	C	C	C	C	C	C	C	C

Supplemental Table 11: HLA-DRB1 Allele Summary [a]				Allele Count by Population Group [b]								3.0.0 CIWD Category by Population Group [c]								
**Allele**	**Genomic Typing**	**Allele ID**	**G group**	**AFA**	**API**	**EURO**	**MENA**	**HIS**	**NAM**	**UNK**	**Total**	**AFA**	**API**	**EURO**	**MENA**	**HIS**	**NAM**	**UNK**	**Total**	**Highest Frequency**
DRB1*11:04:15	11:04:15	HLA17430	11:04:01G	0	0	9	0	0	0	0	9			WD					WD	WD
DRB1*11:198	11:198	HLA14631	11:04:01G	0	0	71	0	0	1	0	72			WD					WD	WD
DRB1*11:04:02	11:04:02	HLA00757		1311	11	99	15	99	21	280	1836	C	WD	WD	WD	C	C	C	C	C
DRB1*11:04:03	11:04:03	HLA02245		0	1	34	1	3	0	3	42			WD					WD	WD
DRB1*11:04:04	11:04:04	HLA02848		0	0	4	0	0	0	0	4									
DRB1*11:04:05	11:04:05	HLA03867		0	1	1	5	0	0	1	8				WD				WD	WD
DRB1*11:04:06	11:04:06	HLA05846		0	0	3	0	0	0	0	3									
DRB1*11:05	11:05	HLA00758		0	74	4	0	0	0	15	93		I					I	WD	I
**DRB1*11:06 total**	**11:06 total**			**22**	**3459**	**242**	**48**	**12**	**35**	**171**	**3989**	**WD**	**C**	**I**	**C**	**I**	**C**	**C**	**C**	**C**
DRB1*11:06	11:06			0	16	12	0	0	0	4	32		I	WD					WD	I
**DRB1*11:06:01G total**	**11:06:01G total**			**17**	**3442**	**188**	**48**	**12**	**35**	**158**	**3900**	**WD**	**C**	**I**	**C**	**I**	**C**	**C**	**C**	**C**
DRB1*11:06:01G	11:06:01G		11:06:01G	9	2449	72	24	6	18	83	2661	WD	C	WD	WD	WD	C	I	C	C
DRB1*11:06:01	11:06:01	HLA00759	11:06:01G	8	799	109	23	6	17	62	1024	WD	C	WD	WD	WD	C	I	I	C
DRB1*11:129	11:129	HLA08091	11:06:01G	0	194	7	1	0	0	13	215		C	WD				WD	I	C
DRB1*11:06:02	11:06:02	HLA01683		5	0	42	0	0	0	7	54	WD		WD				WD	WD	WD
DRB1*11:06:03	11:06:03	HLA07873		0	1	0	0	0	0	2	3									
**DRB1*11:07 total**	**11:07 total**			**6**	**0**	**73**	**0**	**8**	**1**	**13**	**101**	**WD**		**WD**		**I**		**WD**	**WD**	**I**
DRB1*11:07	11:07			5	0	53	0	7	1	11	77	WD		WD		WD		WD	WD	WD
DRB1*11:07:01	11:07:01	HLA00760		1	0	19	0	1	0	2	23			WD					WD	WD
DRB1*11:07:02	11:07:02	HLA15005		0	0	1	0	0	0	0	1									
**DRB1*11:08 total**	**11:08 total**			**8**	**981**	**306**	**22**	**1**	**5**	**33**	**1356**	**WD**	**C**	**I**	**WD**		**WD**	**I**	**I**	**C**
DRB1*11:08	11:08			2	6	19	0	1	1	2	31		WD	WD					WD	WD
DRB1*11:08:01	11:08:01	HLA00761		3	970	287	22	0	4	31	1317		C	I	WD			I	I	C
DRB1*11:08:02	11:08:02	HLA00762		3	0	0	0	0	0	0	3									
DRB1*11:08:03	11:08:03	HLA11069		0	5	0	0	0	0	0	5		WD						WD	WD
DRB1*11:09	11:09	HLA00763		0	2	302	9	3	0	53	369			I	WD			I	I	I
**DRB1*11:10 total**	**11:10 total**			**502**	**1**	**30**	**1**	**38**	**4**	**106**	**682**	**C**		**WD**		**I**		**I**	**I**	**C**
DRB1*11:10	11:10			37	0	5	0	5	0	9	56	WD		WD		WD		WD	WD	WD
DRB1*11:10:01	11:10:01	HLA00764		465	1	22	0	33	4	97	622	C		WD		I		I	I	C
DRB1*11:10:02	11:10:02	HLA03443		0	0	3	1	0	0	0	4									
**DRB1*11:11 total**	**11:11 total**			**13**	**1076**	**751**	**40**	**18**	**4**	**242**	**2144**	**WD**	**C**	**I**	**WD**	**I**		**C**	**C**	**C**

Supplemental Table 11: HLA-DRB1 Allele Summary [a]				Allele Count by Population Group [b]								3.0.0 CIWD Category by Population Group [c]								
Allele	Genomic Typing	Allele ID	G group	AFA	API	EURO	MENA	HIS	NAM	UNK	Total	AFA	API	EURO	MENA	HIS	NAM	UNK	Total	Highest Frequency
DRB1*11:11	11:11			7	102	357	10	12	2	132	622	WD	I	I	WD	I		I	I	I
**DRB1*11:11:01G total**	**11:11:01G total**			**5**	**974**	**394**	**30**	**6**	**2**	**109**	**1520**	**WD**	**C**	**I**	**WD**	**WD**		**I**	**I**	**C**
DRB1*11:11:01G	11:11:01G		11:11:01G	1	486	144	17	1	0	21	670		C	I	WD			I	I	C
DRB1*11:11:01	11:11:01	HLA00765	11:11:01G	4	488	250	13	5	2	88	850		C	I	WD	WD		I	I	C
DRB1*11:11:03	11:11:03	HLA11174		1	0	0	0	0	0	1	2									
**DRB1*11:12 total**	**11:12 total**			**6**	**83**	**1753**	**256**	**30**	**1**	**89**	**2218**	**WD**	**I**	**C**	**C**	**I**		**I**	**C**	**C**
DRB1*11:12	11:12			1	0	71	10	8	0	5	95			WD	WD	I		WD	WD	I
DRB1*11:12P	11:12P			0	0	1	0	0	0	0	1									
DRB1*11:12:01	11:12:01	HLA00766		5	83	1681	246	22	1	84	2122	WD	I	C	C	I		I	C	C
**DRB1*11:13 total**	**11:13 total**			**5**	**3**	**858**	**34**	**25**	**0**	**37**	**962**	**WD**		**I**	**WD**	**I**		**I**	**I**	**I**
**DRB1*11:13:01G total**	**11:13:01G total**			**5**	**3**	**858**	**34**	**25**	**0**	**37**	**962**	**WD**		**I**	**WD**	**I**		**I**	**I**	**I**
DRB1*11:13	11:13			2	2	256	10	11	0	15	296			I	WD	I		I	I	I
DRB1*11:13:01G	11:13:01G		11:13:01G	1	0	30	0	2	0	10	43			WD				WD	WD	WD
DRB1*11:13:02	11:13:02	HLA03562	11:13:01G	2	1	572	24	12	0	12	623			I	WD	I		WD	I	I
**DRB1*11:14 total**	**11:14 total**			**41**	**6**	**976**	**3**	**62**	**6**	**99**	**1193**	**C**	**WD**	**I**		**I**	**WD**	**I**	**I**	**C**
DRB1*11:14	11:14			6	0	75	0	11	1	17	110	WD		WD		I		I	WD	I
DRB1*11:14:01	11:14:01	HLA00768		2	6	896	2	46	5	79	1036		WD	I		I	WD	I	I	I
DRB1*11:14:02	11:14:02	HLA02106		33	0	5	1	5	0	3	47	WD		WD		WD			WD	WD
DRB1*11:15	11:15	HLA00769		7	8	1584	147	87	8	133	1974	WD	WD	C	C	C	C	C	C	C
DRB1*11:16	11:16	HLA00770		0	2	81	15	1	0	4	103			WD	WD				WD	WD
DRB1*11:17	11:17	HLA00771		176	1	14	0	14	9	40	254	C		WD		I	C	I	I	C
DRB1*11:18	11:18	HLA00772		4	52	106	4	6	0	16	188		I	WD		WD		I	I	I
**DRB1*11:19 total**	**11:19 total**			**2**	**7**	**653**	**2**	**46**	**1**	**39**	**750**		**WD**	**I**		**I**		**I**	**I**	**I**
DRB1*11:19	11:19			0	0	37	0	7	0	6	50			WD		WD		WD	WD	WD
DRB1*11:19:01	11:19:01	HLA00773		0	7	616	2	39	1	33	698		WD	I		I		I	I	I
DRB1*11:19:03	11:19:03	HLA06500		2	0	0	0	0	0	0	2									
DRB1*11:20	11:20	HLA00774		1	0	29	1	0	0	4	35			WD					WD	WD
DRB1*11:21	11:21	HLA00775		0	0	28	0	0	0	0	28			WD					WD	WD
**DRB1*11:23 total**	**11:23 total**			**0**	**2**	**10**	**1**	**1**	**0**	**16**	**30**			**WD**				**I**	**WD**	**I**
DRB1*11:23	11:23			0	0	1	0	1	0	2	4									
DRB1*11:23:01	11:23:01	HLA00777		0	0	3	1	0	0	0	4									

Supplemental Table 11: HLA-DRB1 Allele Summary [a]				Allele Count by Population Group [b]								3.0.0 CIWD Category by Population Group [c]								
Allele	Genomic Typing	Allele ID	G group	AFA	API	EURO	MENA	HIS	NAM	UNK	Total	AFA	API	EURO	MENA	HIS	NAM	UNK	Total	Highest Frequency
DRB1*11:23:02	11:23:02	HLA09687		0	2	6	0	0	0	14	22			WD				I	WD	I
DRB1*11:24	11:24	HLA00778		5	0	222	15	7	1	18	268	WD		I	WD	WD		I	I	I
DRB1*11:25	11:25	HLA00779		0	0	100	0	0	0	1	101			WD					WD	WD
DRB1*11:26	11:26	HLA00780		0	0	6	1	0	0	6	13			WD				WD	WD	WD
**DRB1*11:27 total**	**11:27 total**			**1**	**6**	**464**	**1**	**12**	**0**	**12**	**496**		**WD**	**I**		**I**		**WD**	**I**	**I**
DRB1*11:27	11:27			0	0	32	0	0	0	0	32			WD					WD	WD
DRB1*11:27:01	11:27:01	HLA00781		0	6	406	0	3	0	7	422		WD	I				WD	I	I
DRB1*11:27:02	11:27:02	HLA01213		0	0	26	1	1	0	3	31			WD					WD	WD
DRB1*11:27:03	11:27:03	HLA06513		1	0	0	0	8	0	2	11					I			WD	I
**DRB1*11:28 total**	**11:28 total**			**6**	**24**	**584**	**5**	**10**	**0**	**26**	**655**	**WD**	**I**	**I**	**WD**	**I**		**I**	**I**	**I**
DRB1*11:28	11:28			1	1	22	1	2	0	2	29			WD					WD	WD
DRB1*11:28:01	11:28:01	HLA00782		5	17	561	4	8	0	24	619	WD	I	I		I		I	I	I
DRB1*11:28:02	11:28:02	HLA03768		0	6	1	0	0	0	0	7		WD						WD	WD
**DRB1*11:29 total**	**11:29 total**			**5**	**35**	**332**	**10**	**4**	**0**	**15**	**401**	**WD**	**I**	**I**	**WD**			**I**	**I**	**I**
DRB1*11:29	11:29			2	8	170	5	3	0	5	193		WD	I	WD			WD	I	I
DRB1*11:29:01	11:29:01	HLA00783		3	27	161	5	1	0	10	207		I	I	WD			WD	I	I
DRB1*11:29:02	11:29:02	HLA13215		0	0	1	0	0	0	0	1									
DRB1*11:30	11:30	HLA00784		0	0	2	0	0	0	0	2									
DRB1*11:32	11:32	HLA00786		2	1	34	0	4	0	10	51			WD				WD	WD	WD
DRB1*11:33	11:33	HLA00787		0	0	11	4	0	0	2	17			WD					WD	WD
DRB1*11:34	11:34	HLA00788		0	0	247	3	1	0	5	256			I				WD	I	I
DRB1*11:35	11:35	HLA01010		0	0	16	20	1	0	1	38			WD	WD				WD	WD
DRB1*11:36	11:36	HLA01090		2	8	262	9	3	1	19	304		WD	I	WD			I	I	I
**DRB1*11:37 total**	**11:37 total**			**1**	**7**	**80**	**2**	**1**	**0**	**4**	**95**		**WD**	**WD**					**WD**	**WD**
DRB1*11:37	11:37			0	1	11	0	0	0	3	15			WD					WD	WD
DRB1*11:37:01	11:37:01	HLA01156		0	6	69	0	1	0	1	77		WD	WD					WD	WD
DRB1*11:37:02	11:37:02	HLA07163		1	0	0	2	0	0	0	3									
DRB1*11:38	11:38	HLA01239		0	0	1	0	0	0	0	1									
DRB1*11:39	11:39	HLA01273		3	2	230	2	4	0	21	262			I				I	I	I
DRB1*11:40	11:40	HLA01279		0	0	1	0	0	0	0	1									
DRB1*11:41	11:41	HLA01300		0	0	10	0	0	0	2	12			WD					WD	WD

Supplemental Table 11: HLA-DRB1 Allele Summary [a]				Allele Count by Population Group [b]								3.0.0 CIWD Category by Population Group [c]								
Allele	Genomic Typing	Allele ID	G group	AFA	API	EURO	MENA	HIS	NAM	UNK	Total	AFA	API	EURO	MENA	HIS	NAM	UNK	Total	Highest Frequency
**DRB1*11:42 total**	**11:42 total**			**0**	**0**	**56**	**13**	**9**	**0**	**14**	**92**			**WD**	**WD**	**I**		**I**	**WD**	**I**
DRB1*11:42	11:42			0	0	55	13	9	0	13	90			WD	WD	I		WD	WD	I
DRB1*11:42:01	11:42:01	HLA01447		0	0	1	0	0	0	1	2									
DRB1*11:43	11:43	HLA01552		1	0	600	0	8	0	21	630			I		I		I	I	I
DRB1*11:44	11:44	HLA01775		0	0	6	1	0	0	0	7			WD					WD	WD
DRB1*11:45	11:45	HLA01779		1	1	2	0	7	0	0	11					WD			WD	WD
**DRB1*11:46 total**	**11:46 total**			**0**	**0**	**1**	**0**	**1**	**0**	**0**	**2**									
DRB1*11:46:01	11:46:01	HLA01794		0	0	1	0	0	0	0	1									
DRB1*11:46:02	11:46:02	HLA06137		0	0	0	0	1	0	0	1									
DRB1*11:47	11:47	HLA01812		4	2	4	1	25	11	10	57					I	C	WD	WD	C
DRB1*11:48	11:48	HLA01841		7	0	2	0	1	0	1	11	WD							WD	WD
**DRB1*11:49 total**	**11:49 total**			**2**	**1**	**18**	**0**	**0**	**0**	**2**	**23**			**WD**					**WD**	**WD**
DRB1*11:49	11:49			0	0	4	0	0	0	0	4									
DRB1*11:49:01	11:49:01	HLA01848		0	1	14	0	0	0	2	17			WD					WD	WD
DRB1*11:49:02	11:49:02	HLA06510		2	0	0	0	0	0	0	2									
DRB1*11:51	11:51	HLA01901		0	0	2	0	2	0	2	6								WD	WD
DRB1*11:53	11:53	HLA01948		0	0	4	0	0	0	0	4									
**DRB1*11:54 total**	**11:54 total**			**0**	**1**	**1**	**0**	**0**	**0**	**1**	**3**									
DRB1*11:54	11:54			0	0	1	0	0	0	0	1									
DRB1*11:54:01	11:54:01	HLA01968		0	1	0	0	0	0	1	2									
DRB1*11:56	11:56	HLA02577		0	0	29	1	1	0	1	32			WD					WD	WD
DRB1*11:57	11:57	HLA02587		0	2	0	0	0	0	0	2									
**DRB1*11:58 total**	**11:58 total**			**0**	**0**	**49**	**3**	**0**	**0**	**3**	**55**			**WD**					**WD**	**WD**
DRB1*11:58	11:58			0	0	5	0	0	0	0	5			WD					WD	WD
DRB1*11:58:01	11:58:01	HLA02718		0	0	38	2	0	0	2	42			WD					WD	WD
DRB1*11:58:02	11:58:02	HLA04666		0	0	6	1	0	0	1	8			WD					WD	WD
DRB1*11:60	11:60	HLA02720		0	0	4	0	0	0	1	5								WD	WD
DRB1*11:61	11:61	HLA02841		0	0	7	0	0	0	0	7			WD					WD	WD
**DRB1*11:62 total**	**11:62 total**			**0**	**10**	**4**	**0**	**0**	**0**	**0**	**14**		**WD**						**WD**	**WD**
DRB1*11:62	11:62			0	1	2	0	0	0	0	3									
DRB1*11:62:01	11:62:01	HLA02856		0	9	2	0	0	0	0	11		WD						WD	WD

Supplemental Table 11: HLA-DRB1 Allele Summary [a]				Allele Count by Population Group [b]								3.0.0 CIWD Category by Population Group [c]								
Allele	Genomic Typing	Allele ID	G group	AFA	API	EURO	MENA	HIS	NAM	UNK	Total	AFA	API	EURO	MENA	HIS	NAM	UNK	Total	Highest Frequency
**DRB1*11:65 total**	**11:65 total**			**9**	**0**	**0**	**0**	**0**	**0**	**3**	**12**	**WD**							**WD**	**WD**
DRB1*11:65:01	11:65:01	HLA03068		9	0	0	0	0	0	3	12	WD							WD	WD
DRB1*11:66	11:66	HLA03072		0	202	4	0	0	0	2	208		C						I	C
DRB1*11:68	11:68	HLA03207		0	0	0	0	0	0	1	1									
DRB1*11:69	11:69	HLA03385		1	0	45	1	0	0	1	48			WD					WD	WD
DRB1*11:73	11:73	HLA03779		0	0	1	0	0	0	0	1									
**DRB1*11:74 total**	**11:74 total**			**0**	**0**	**1**	**0**	**0**	**0**	**0**	**1**									
DRB1*11:74:01	11:74:01	HLA03846		0	0	1	0	0	0	0	1									
DRB1*11:76	11:76	HLA03853		0	0	66	0	1	0	0	67			WD					WD	WD
DRB1*11:78	11:78	HLA03856		0	0	21	0	0	0	0	21			WD					WD	WD
DRB1*11:79	11:79	HLA03866		0	0	1	0	0	0	0	1									
DRB1*11:81	11:81	HLA03913		0	0	2	13	0	0	0	15				WD				WD	WD
DRB1*11:83	11:83	HLA04032		0	0	0	0	1	0	0	1									
**DRB1*11:84 total**	**11:84 total**			**0**	**0**	**21**	**0**	**9**	**0**	**4**	**34**			**WD**		**I**			**WD**	**I**
DRB1*11:84	11:84			0	0	5	0	2	0	1	8			WD					WD	WD
DRB1*11:84:01	11:84:01	HLA04034		0	0	8	0	7	0	2	17			WD		WD			WD	WD
DRB1*11:84:02	11:84:02	HLA07516		0	0	8	0	0	0	1	9			WD					WD	WD
DRB1*11:85	11:85	HLA04400		0	0	1	0	1	0	1	3									
DRB1*11:86	11:86	HLA04404		2	0	0	0	0	0	0	2									
DRB1*11:88	11:88	HLA04615		0	0	10	0	0	0	0	10			WD					WD	WD
DRB1*11:90	11:90	HLA04678		0	0	1	0	0	0	0	1									
DRB1*11:92	11:92	HLA04688		0	3	0	0	0	0	0	3									
DRB1*11:93	11:93	HLA04721		0	0	2	0	1	1	2	6								WD	WD
DRB1*11:94	11:94	HLA04776		0	0	6	0	0	0	0	6			WD					WD	WD
DRB1*11:96	11:96	HLA05379		0	1	3	2	0	0	0	6								WD	WD
DRB1*11:98	11:98	HLA05759		2	0	0	0	0	0	0	2									
DRB1*11:99	11:99	HLA05973		0	0	6	0	0	0	0	6			WD					WD	WD
**DRB1*11:101 total**	**11:101 total**			**0**	**5**	**1**	**0**	**0**	**0**	**0**	**6**		**WD**						**WD**	**WD**
DRB1*11:101	11:101			0	0	1	0	0	0	0	1									
DRB1*11:101:01	11:101:01	HLA06039		0	2	0	0	0	0	0	2									
DRB1*11:101:02	11:101:02	HLA14571		0	3	0	0	0	0	0	3									

Supplemental Table 11: HLA-DRB1 Allele Summary [a]				Allele Count by Population Group [b]								3.0.0 CIWD Category by Population Group [c]								
Allele	Genomic Typing	Allele ID	G group	AFA	API	EURO	MENA	HIS	NAM	UNK	Total	AFA	API	EURO	MENA	HIS	NAM	UNK	Total	Highest Frequency
**DRB1*11:102 total**	**11:102 total**			**0**	**0**	**3**	**0**	**0**	**0**	**1**	**4**									
DRB1*11:102:02	11:102:02	HLA07346		0	0	3	0	0	0	1	4									
**DRB1*11:103 total**	**11:103 total**			**1**	**1**	**11**	**0**	**0**	**0**	**0**	**13**			**WD**					**WD**	**WD**
DRB1*11:103	11:103			1	0	10	0	0	0	0	11			WD					WD	WD
DRB1*11:103:01	11:103:01	HLA06482		0	1	1	0	0	0	0	2									
DRB1*11:104	11:104	HLA06501		0	0	16	0	0	0	0	16			WD					WD	WD
DRB1*11:105	11:105	HLA06511		0	0	1	0	0	0	1	2									
DRB1*11:106	11:106	HLA06521		0	2	0	0	0	0	0	2									
DRB1*11:108	11:108	HLA06667		0	0	3	0	0	0	1	4									
DRB1*11:111	11:111	HLA06860		0	1	0	0	0	0	0	1									
DRB1*11:113	11:113	HLA06773		0	0	8	2	0	0	6	16			WD				WD	WD	WD
DRB1*11:117	11:117	HLA07247		0	2	0	0	0	0	0	2									
DRB1*11:118	11:118	HLA07260		0	3	0	0	0	0	0	3									
DRB1*11:120	11:120	HLA07328		0	0	1	0	0	0	0	1									
DRB1*11:122	11:122	HLA07511		0	0	2	0	0	0	0	2									
DRB1*11:124	11:124	HLA07515		0	0	2	0	0	0	0	2									
DRB1*11:125	11:125	HLA07517		0	0	23	0	0	0	0	23			WD					WD	WD
DRB1*11:128	11:128	HLA07882		0	0	7	0	0	0	1	8			WD					WD	WD
DRB1*11:132	11:132	HLA08551		1	0	0	0	0	0	0	1									
DRB1*11:136	11:136	HLA08868		0	0	1	0	0	0	0	1									
DRB1*11:137	11:137	HLA08869		0	0	15	0	0	0	0	15			WD					WD	WD
DRB1*11:138	11:138	HLA08870		0	0	1	0	0	0	0	1									
DRB1*11:142	11:142	HLA09080		0	0	0	0	0	0	1	1									
DRB1*11:143	11:143	HLA09122		0	1	0	0	0	0	0	1									
DRB1*11:144	11:144	HLA09216		1	0	0	0	0	0	0	1									
DRB1*11:145	11:145	HLA09304		0	0	0	7	0	0	0	7				WD				WD	WD
**DRB1*11:147 total**	**11:147 total**			**0**	**0**	**0**	**1**	**0**	**0**	**0**	**1**									
DRB1*11:147	11:147			0	0	0	1	0	0	0	1									
DRB1*11:148	11:148	HLA09916		0	0	0	0	0	0	1	1									
DRB1*11:150	11:150	HLA10318		0	0	1	0	0	0	0	1									
DRB1*11:152	11:152	HLA10320		0	0	2	0	0	0	0	2									

Supplemental Table 11: HLA-DRB1 Allele Summary [a]				Allele Count by Population Group [b]								3.0.0 CIWD Category by Population Group [c]								
Allele	Genomic Typing	Allele ID	G group	AFA	API	EURO	MENA	HIS	NAM	UNK	Total	AFA	API	EURO	MENA	HIS	NAM	UNK	Total	Highest Frequency
DRB1*11:154	11:154	HLA11060		0	1	0	0	0	0	0	1									
DRB1*11:155	11:155	HLA11171		0	0	0	0	0	0	1	1									
DRB1*11:161	11:161	HLA11744		0	0	2	0	0	0	0	2									
DRB1*11:162	11:162	HLA11746		0	0	1	0	0	0	0	1									
DRB1*11:173	11:173	HLA12838		0	0	1	0	0	0	0	1									
DRB1*11:175	11:175	HLA12840		0	0	2	0	1	0	1	4									
DRB1*11:179	11:179	HLA13288		0	0	0	5	0	0	0	5				WD				WD	WD
DRB1*11:185	11:185	HLA13821		0	0	1	0	0	0	0	1									
DRB1*11:189	11:189	HLA14053		0	0	0	1	0	0	0	1									
**DRB1*11:193 total**	**11:193 total**			**0**	**0**	**1**	**0**	**0**	**0**	**0**	**1**									
DRB1*11:193:01	11:193:01	HLA14182		0	0	1	0	0	0	0	1									
DRB1*11:194	11:194	HLA14186		0	0	0	0	1	0	0	1									
DRB1*11:196	11:196	HLA14421		0	0	1	0	0	0	0	1									
DRB1*11:197	11:197	HLA14572		0	0	1	0	0	0	0	1									
DRB1*11:200	11:200	HLA15006		0	0	0	0	0	0	1	1									
DRB1*11:205	11:205	HLA15707		0	1	0	0	0	0	0	1									
DRB1*11:217N	11:217N	HLA17534		0	0	1	0	0	0	0	1									
DRB1*11:CODE	11:CODE			2782	1335	44556	1483	3143	173	4997	58469	NA	NA	NA	NA	NA	NA	NA	NA	NA
**DRB1*12:01 total**	**12:01 total**			**13148**	**14410**	**223297**	**5173**	**7451**	**982**	**24372**	**288833**	**C**	**C**	**C**	**C**	**C**	**C**	**C**	**C**	**C**
DRB1*12:01	12:01			1	0	888	3	6	0	34	932			I		WD		I	I	I
DRB1*12:01P	12:01P			8	1	208	0	2	0	1	220	WD		I					I	I
**DRB1*12:01:01G total**	**12:01:01G total**			**13139**	**14406**	**222183**	**5167**	**7442**	**982**	**24335**	**287654**	**C**	**C**	**C**	**C**	**C**	**C**	**C**	**C**	**C**
DRB1*12:01:01G	12:01:01G		12:01:01G	13082	14374	222036	5166	7402	979	24286	287325	C	C	C	C	C	C	C	C	C
DRB1*12:01:01	12:01:01		12:01:01G	6	19	23	0	7	1	23	79	WD	I	WD		WD		I	WD	I
DRB1*12:01:01:01	12:01:01:01	HLA00789	12:01:01G	50	8	1	0	17	2	20	98	C	WD			I		I	WD	C
DRB1*12:01:01:03	12:01:01:03	HLA14832	12:01:01G	1	4	117	1	16	0	6	145			WD		I		WD	WD	I
DRB1*12:17	12:17	HLA03485	12:01:01G	0	1	6	0	0	0	0	7			WD					WD	WD
DRB1*12:01:02	12:01:02	HLA01407		0	1	12	3	0	0	1	17			WD					WD	WD
DRB1*12:01:03	12:01:03	HLA04352		0	1	5	0	1	0	0	7			WD					WD	WD
DRB1*12:01:05	12:01:05	HLA09462		0	1	0	0	0	0	1	2									
DRB1*12:01:09	12:01:09	HLA13274		0	0	1	0	0	0	0	1									

Supplemental Table 11: HLA-DRB1 Allele Summary [a]				Allele Count by Population Group [b]								3.0.0 CIWD Category by Population Group [c]								
Allele	Genomic Typing	Allele ID	G group	AFA	API	EURO	MENA	HIS	NAM	UNK	Total	AFA	API	EURO	MENA	HIS	NAM	UNK	Total	Highest Frequency
**DRB1*12:02 total**	**12:02 total**			**1000**	**70680**	**3135**	**658**	**709**	**136**	**6242**	**82560**	**C**	**C**	**C**	**C**	**C**	**C**	**C**	**C**	**C**
DRB1*12:02	12:02			103	3314	97	23	78	10	513	4138	C	C	WD	WD	C	C	C	C	C
**DRB1*12:02:01G total**	**12:02:01G total**			**620**	**67359**	**3017**	**628**	**553**	**95**	**5646**	**77918**	**C**	**C**	**C**	**C**	**C**	**C**	**C**	**C**	**C**
DRB1*12:02:01G	12:02:01G		12:02:01G	9	21272	167	11	4	0	47	21510	WD	C	I	WD			I	C	C
DRB1*12:02:01	12:02:01		12:02:01G	611	46087	2850	617	549	95	5599	56408	C	C	C	C	C	C	C	C	C
DRB1*12:02:02	12:02:02	HLA00791		277	7	21	7	78	31	83	504	C	WD	WD	WD	C	C	I	I	C
**DRB1*12:03 total**	**12:03 total**			**0**	**1**	**125**	**0**	**0**	**1**	**36**	**163**			**I**				**I**	**I**	**I**
DRB1*12:03	12:03			0	0	7	0	0	0	2	9			WD					WD	WD
DRB1*12:03:02	12:03:02	HLA00793		0	1	115	0	0	1	34	151			WD				I	WD	I
DRB1*12:03:03	12:03:03	HLA08114		0	0	3	0	0	0	0	3									
DRB1*12:04	12:04	HLA00794		20	0	3	0	2	1	8	34	WD						WD	WD	WD
DRB1*12:05	12:05	HLA00795		0	13	5	0	0	0	3	21		WD	WD					WD	WD
DRB1*12:07	12:07	HLA01361		0	0	2	0	0	0	0	2									
DRB1*12:08	12:08	HLA01480		0	10	1	0	0	0	1	12		WD						WD	WD
DRB1*12:09	12:09	HLA01806		1	2	0	0	0	0	0	3									
DRB1*12:11	12:11	HLA02055		0	0	40	0	1	0	0	41			WD					WD	WD
DRB1*12:15	12:15	HLA02583		0	1	0	0	0	0	0	1									
**DRB1*12:16 total**	**12:16 total**			**0**	**1**	**0**	**0**	**0**	**0**	**0**	**1**									
DRB1*12:16:01	12:16:01	HLA02988		0	1	0	0	0	0	0	1									
DRB1*12:19	12:19	HLA03604		0	1	0	0	0	0	0	1									
DRB1*12:20	12:20	HLA03997		0	6	0	0	0	0	1	7		WD						WD	WD
DRB1*12:24N	12:24N	HLA05372		0	0	27	0	0	0	10	37			WD				WD	WD	WD
DRB1*12:25	12:25	HLA05561		26	0	2	0	0	0	6	34	WD						WD	WD	WD
DRB1*12:27	12:27	HLA06591		0	3	0	0	0	0	0	3									
DRB1*12:34	12:34	HLA06878		0	0	3	0	1	0	0	4									
DRB1*12:48	12:48	HLA11751		0	0	8	0	0	0	1	9			WD					WD	WD
DRB1*12:52	12:52	HLA12594		0	1	0	0	0	0	0	1									
DRB1*12:53	12:53	HLA12820		0	0	1	0	0	0	0	1									
DRB1*12:58	12:58	HLA13491		0	0	1	0	0	0	0	1									
DRB1*12:CODE	12:CODE			403	981	3712	18	376	30	485	6005	NA	NA	NA	NA	NA	NA	NA	NA	NA
**DRB1*13:01 total**	**13:01 total**			**20540**	**67942**	**779925**	**20293**	**30448**	**3288**	**71206**	**993642**	**C**	**C**	**C**	**C**	**C**	**C**	**C**	**C**	**C**

Supplemental Table 11: HLA-DRB1 Allele Summary [a]				Allele Count by Population Group [b]								3.0.0 CIWD Category by Population Group [c]								
**Allele**	**Genomic Typing**	**Allele ID**	**G group**	**AFA**	**API**	**EURO**	**MENA**	**HIS**	**NAM**	**UNK**	**Total**	**AFA**	**API**	**EURO**	**MENA**	**HIS**	**NAM**	**UNK**	**Total**	**Highest Frequency**
DRB1*13:01	13:01			53	9	4267	20	33	4	238	4624	C	WD	C	WD	I		C	C	C
DRB1*13:01P	13:01P			3	8	700	3	1	0	1	716		WD	I					I	I
**DRB1*13:01:01G total**	**13:01:01G total**			**20435**	**67631**	**774941**	**20270**	**30412**	**3284**	**70953**	**987926**	**C**	**C**	**C**	**C**	**C**	**C**	**C**	**C**	**C**
DRB1*13:01:01G	13:01:01G		13:01:01G	10300	47355	171322	7744	17423	1820	49972	305936	C	C	C	C	C	C	C	C	C
DRB1*13:01:01	13:01:01		13:01:01G	10106	20149	602128	12492	12752	1437	20818	679882	C	C	C	C	C	C	C	C	C
DRB1*13:01:01:01	13:01:01:01	HLA00797	13:01:01G	18	118	837	31	134	14	92	1244	WD	I	I	WD	C	C	I	I	C
DRB1*13:01:01:02	13:01:01:02	HLA14833	13:01:01G	10	9	613	3	94	9	62	800	WD	WD	I		C	C	I	I	C
DRB1*13:01:18	13:01:18	HLA17459	13:01:01G	0	0	4	0	0	0	0	4									
DRB1*13:117	13:117	HLA06820	13:01:01G	1	0	28	0	9	4	8	50			WD		I		WD	WD	I
DRB1*13:190	13:190	HLA12415	13:01:01G	0	0	8	0	0	0	1	9			WD					WD	WD
DRB1*13:215	13:215	HLA14887	13:01:01G	0	0	1	0	0	0	0	1									
DRB1*13:01:03	13:01:03	HLA01689		47	280	7	0	1	0	11	346	C	C	WD				WD	I	C
DRB1*13:01:04	13:01:04	HLA03852		0	1	0	0	0	0	0	1									
DRB1*13:01:05	13:01:05	HLA03864		0	1	1	0	0	0	0	2									
DRB1*13:01:06	13:01:06	HLA04407		0	0	2	0	0	0	0	2									
DRB1*13:01:07	13:01:07	HLA04676		0	1	0	0	0	0	0	1									
DRB1*13:01:08	13:01:08	HLA05822		2	0	2	0	1	0	2	7								WD	WD
DRB1*13:01:10	13:01:10	HLA07259		0	0	4	0	0	0	1	5								WD	WD
DRB1*13:01:11	13:01:11	HLA07510		0	0	1	0	0	0	0	1									
DRB1*13:01:13	13:01:13	HLA07923		0	4	0	0	0	0	0	4									
DRB1*13:01:14	13:01:14	HLA10313		0	7	0	0	0	0	0	7		WD						WD	WD
**DRB1*13:02 total**	**13:02 total**			**25339**	**43830**	**457678**	**17049**	**24472**	**2660**	**61130**	**632158**	**C**	**C**	**C**	**C**	**C**	**C**	**C**	**C**	**C**
DRB1*13:02	13:02			1275	661	19949	621	2932	185	2354	27977	C	C	C	C	C	C	C	C	C
DRB1*13:02P	13:02P			2	0	736	7	1	0	1	747			I	WD				I	I
**DRB1*13:02:01G total**	**13:02:01G total**			**24053**	**43166**	**436923**	**16400**	**21536**	**2475**	**58759**	**603312**	**C**	**C**	**C**	**C**	**C**	**C**	**C**	**C**	**C**
DRB1*13:02:01G	13:02:01G		13:02:01G	565	12616	9172	648	1831	26	12153	37011	C	C	C	C	C	C	C	C	C
DRB1*13:02:01	13:02:01		13:02:01G	23488	30550	427714	15752	19705	2449	46603	566261	C	C	C	C	C	C	C	C	C
DRB1*13:02:01:02	13:02:01:02	HLA17354	13:02:01G	0	0	8	0	0	0	0	8			WD					WD	WD
DRB1*13:208	13:208	HLA13995	13:02:01G	0	0	27	0	0	0	3	30			WD					WD	WD
DRB1*13:236	13:236	HLA16831	13:02:01G	0	0	2	0	0	0	0	2									
DRB1*13:02:02	13:02:02	HLA01155		0	1	67	3	3	0	10	84			WD				WD	WD	WD

Supplemental Table 11: HLA-DRB1 Allele Summary [a]				Allele Count by Population Group [b]								3.0.0 CIWD Category by Population Group [c]								
Allele	Genomic Typing	Allele ID	G group	AFA	API	EURO	MENA	HIS	NAM	UNK	Total	AFA	API	EURO	MENA	HIS	NAM	UNK	Total	Highest Frequency
DRB1*13:02:03	13:02:03	HLA02768		0	0	1	3	0	0	0	4									
DRB1*13:02:05	13:02:05	HLA06496		5	2	0	13	0	0	5	25	WD			WD			WD	WD	WD
DRB1*13:02:06	13:02:06	HLA07878		0	0	0	2	0	0	0	2									
DRB1*13:02:07	13:02:07	HLA07885		3	0	0	0	0	0	0	3									
DRB1*13:02:08	13:02:08	HLA09067		1	0	2	0	0	0	1	4									
**DRB1*13:03 total**	**13:03 total**			**12883**	**1353**	**166671**	**6908**	**8675**	**931**	**18048**	**215469**	**C**	**C**	**C**	**C**	**C**	**C**	**C**	**C**	**C**
DRB1*13:03	13:03			1500	55	10935	441	1631	104	1628	16294	C	I	C	C	C	C	C	C	C
DRB1*13:03P	13:03P			0	0	71	0	0	0	0	71			WD					WD	WD
**DRB1*13:03:01G total**	**13:03:01G total**			**11336**	**1298**	**155648**	**6463**	**7040**	**827**	**16409**	**199021**	**C**	**C**	**C**	**C**	**C**	**C**	**C**	**C**	**C**
DRB1*13:03:01G	13:03:01G		13:03:01G	56	105	1171	68	259	0	1169	2828	C	I	I	C	C		C	C	C
DRB1*13:03:01	13:03:01	HLA00799	13:03:01G	11280	1193	154470	6395	6781	827	15240	196186	C	C	C	C	C	C	C	C	C
DRB1*13:227	13:227	HLA16047	13:03:01G	0	0	7	0	0	0	0	7			WD					WD	WD
DRB1*13:03:02	13:03:02	HLA00800		47	0	16	4	4	0	10	81	C		WD				WD	WD	C
DRB1*13:03:03	13:03:03	HLA04117		0	0	1	0	0	0	1	2									
DRB1*13:04	13:04	HLA00801		4349	41	585	24	2483	361	1470	9313	C	I	I	WD	C	C	C	C	C
**DRB1*13:05 total**	**13:05 total**			**167**	**152**	**23754**	**1961**	**2427**	**123**	**6497**	**35081**	**C**	**C**	**C**	**C**	**C**	**C**	**C**	**C**	**C**
DRB1*13:05	13:05			18	11	2478	77	382	13	327	3306	WD	WD	C	C	C	C	C	C	C
DRB1*13:05P	13:05P			0	0	10	0	0	0	0	10			WD					WD	WD
DRB1*13:05:01	13:05:01	HLA00802		120	141	21265	1884	2043	110	6161	31724	C	C	C	C	C	C	C	C	C
DRB1*13:05:02	13:05:02	HLA02318		29	0	1	0	2	0	9	41	WD						WD	WD	WD
DRB1*13:06	13:06	HLA00803		2	1	343	1	5	1	36	389			I		WD		I	I	I
**DRB1*13:07 total**	**13:07 total**			**1**	**80**	**16**	**28**	**0**	**0**	**9**	**134**		**I**	**WD**	**WD**			**WD**	**WD**	**I**
DRB1*13:07	13:07			0	2	1	1	0	0	0	4									
DRB1*13:07:01	13:07:01	HLA00804		0	78	10	27	0	0	7	122		I	WD	WD			WD	WD	I
DRB1*13:07:02	13:07:02	HLA00805		1	0	5	0	0	0	2	8			WD					WD	WD
DRB1*13:08	13:08	HLA00806		0	53	40	1	0	0	2	96		I	WD					WD	I
DRB1*13:09	13:09	HLA00807		0	0	7	0	18	0	3	28			WD		I			WD	I
DRB1*13:10	13:10	HLA00808		15	1	912	14	15	3	78	1038	WD		I	WD	I		I	I	I
**DRB1*13:11 total**	**13:11 total**			**2**	**30**	**207**	**25**	**58**	**4**	**40**	**366**		**I**	**I**	**WD**	**I**		**I**	**I**	**I**
DRB1*13:11	13:11			1	2	8	1	15	1	2	30			WD		I			WD	I
DRB1*13:11:01	13:11:01	HLA00809		1	28	187	18	43	3	32	312		I	I	WD	I		I	I	I

Supplemental Table 11: HLA-DRB1 Allele Summary [a]				Allele Count by Population Group [b]								3.0.0 CIWD Category by Population Group [c]								
Allele	Genomic Typing	Allele ID	G group	AFA	API	EURO	MENA	HIS	NAM	UNK	Total	AFA	API	EURO	MENA	HIS	NAM	UNK	Total	Highest Frequency
DRB1*13:11:02	13:11:02	HLA03407		0	0	12	6	0	0	6	24			WD	WD			WD	WD	WD
**DRB1*13:12 total**	**13:12 total**			**8**	**1942**	**49**	**2**	**14**	**1**	**166**	**2182**	**WD**	**C**	**WD**		**I**		**C**	**C**	**C**
DRB1*13:12	13:12			0	137	3	0	2	0	9	151		C					WD	WD	C
DRB1*13:12:01	13:12:01	HLA00810		5	1805	46	2	10	1	156	2025	WD	C	WD		I		C	C	C
DRB1*13:12:02	13:12:02	HLA05950		3	0	0	0	2	0	1	6								WD	WD
DRB1*13:13	13:13	HLA00811		0	7	3	0	0	0	2	12		WD						WD	WD
**DRB1*13:14 total**	**13:14 total**			**21**	**17**	**237**	**24**	**12**	**0**	**18**	**329**	**WD**	**I**	**I**	**WD**	**I**		**I**	**I**	**I**
DRB1*13:14	13:14			0	1	14	3	4	0	4	26			WD					WD	WD
DRB1*13:14:01	13:14:01	HLA00812		4	14	200	21	4	0	11	254		I	I	WD			WD	I	I
DRB1*13:14:02	13:14:02	HLA01154		0	2	23	0	1	0	2	28			WD					WD	WD
DRB1*13:14:03	13:14:03	HLA03208		17	0	0	0	3	0	1	21	WD							WD	WD
DRB1*13:15	13:15	HLA00813		2	3	1515	35	5	0	27	1587			C	WD	WD		I	I	C
DRB1*13:16	13:16	HLA00814		172	0	14	8	15	6	45	260	C		WD	WD	I	WD	I	I	C
DRB1*13:17	13:17	HLA00815		0	0	43	0	0	0	1	44			WD					WD	WD
DRB1*13:18	13:18	HLA00816		1	1	195	11	3	0	14	225			I	WD			I	I	I
DRB1*13:19	13:19	HLA00817		0	27	334	18	3	1	11	394		I	I	WD			WD	I	I
DRB1*13:20	13:20	HLA00818		5	2	96	3	1	0	13	120	WD		WD				WD	WD	WD
**DRB1*13:21 total**	**13:21 total**			**0**	**0**	**377**	**18**	**6**	**0**	**14**	**415**			**I**	**WD**	**WD**		**I**	**I**	**I**
DRB1*13:21	13:21			0	0	22	2	0	0	1	25			WD					WD	WD
DRB1*13:21:01	13:21:01	HLA00819		0	0	238	16	6	0	11	271			I	WD	WD		WD	I	I
DRB1*13:21:02	13:21:02	HLA04669		0	0	117	0	0	0	2	119			WD					WD	WD
DRB1*13:22	13:22	HLA00820		2	2	216	2	0	0	11	233			I				WD	I	I
**DRB1*13:23 total**	**13:23 total**			**1**	**0**	**1**	**0**	**1**	**0**	**3**	**6**								**WD**	**WD**
DRB1*13:23:01	13:23:01	HLA00821		1	0	0	0	1	0	3	5								WD	WD
DRB1*13:23:02	13:23:02	HLA05976		0	0	1	0	0	0	0	1									
DRB1*13:24	13:24	HLA00822		0	0	75	4	3	0	6	88			WD				WD	WD	WD
DRB1*13:25	13:25	HLA00823		0	0	4	0	2	0	0	6								WD	WD
**DRB1*13:26 total**	**13:26 total**			**0**	**1**	**32**	**0**	**4**	**0**	**2**	**39**			**WD**					**WD**	**WD**
DRB1*13:26	13:26			0	0	5	0	1	0	1	7			WD					WD	WD
DRB1*13:26:01	13:26:01	HLA00824		0	1	27	0	3	0	0	31			WD					WD	WD
DRB1*13:26:02	13:26:02	HLA08094		0	0	0	0	0	0	1	1									

Supplemental Table 11: HLA-DRB1 Allele Summary [a]				Allele Count by Population Group [b]								3.0.0 CIWD Category by Population Group [c]								
Allele	Genomic Typing	Allele ID	G group	AFA	API	EURO	MENA	HIS	NAM	UNK	Total	AFA	API	EURO	MENA	HIS	NAM	UNK	Total	Highest Frequency
DRB1*13:27	13:27	HLA00825		5	3	7	0	0	0	3	18	WD		WD					WD	WD
DRB1*13:28	13:28	HLA00826		0	0	46	0	0	0	0	46			WD					WD	WD
DRB1*13:29	13:29	HLA00827		2	1	76	0	2	0	8	89			WD				WD	WD	WD
DRB1*13:30	13:30	HLA00828		0	1	0	0	0	0	1	2									
DRB1*13:31	13:31	HLA00829		142	0	7	0	10	5	28	192	C		WD		I	WD	I	I	C
DRB1*13:32	13:32	HLA00830		0	1	4	0	0	0	0	5								WD	WD
**DRB1*13:33 total**	**13:33 total**			**0**	**0**	**30**	**0**	**0**	**0**	**1**	**31**			**WD**					**WD**	**WD**
DRB1*13:33	13:33			0	0	3	0	0	0	0	3									
DRB1*13:33:03	13:33:03	HLA03861		0	0	24	0	0	0	1	25			WD					WD	WD
DRB1*13:33:01	13:33:01	HLA00831		0	0	3	0	0	0	0	3									
DRB1*13:34	13:34	HLA00832		0	0	3	0	0	0	3	6								WD	WD
DRB1*13:35	13:35	HLA01091		0	0	2	0	0	0	0	2									
DRB1*13:36	13:36	HLA01101		3	4	108	0	1	0	8	124			WD				WD	WD	WD
DRB1*13:37	13:37	HLA01150		14	0	3	0	0	0	3	20	WD							WD	WD
DRB1*13:38	13:38	HLA01151		7	0	127	0	1	0	1	136	WD		I					WD	I
DRB1*13:39	13:39	HLA01166		0	6	11	2	0	0	1	20		WD	WD					WD	WD
DRB1*13:40	13:40	HLA01209		2	2	23	0	33	6	12	78			WD		I	WD	WD	WD	I
DRB1*13:41	13:41	HLA01212		1	0	3	0	0	0	2	6								WD	WD
DRB1*13:42	13:42	HLA01236		1	5	38	0	9	0	3	56		WD	WD		I			WD	I
DRB1*13:44	13:44	HLA01240		0	0	1	0	0	0	0	1									
DRB1*13:45	13:45	HLA01241		0	0	1	0	0	0	0	1									
DRB1*13:46	13:46	HLA01350		0	0	1	0	0	0	0	1									
DRB1*13:48	13:48	HLA01442		1	0	6	0	1	0	0	8			WD					WD	WD
DRB1*13:49	13:49	HLA01456		0	389	26	2	2	0	2	421		C	WD					I	C
**DRB1*13:50 total**	**13:50 total**			**4**	**5**	**6**	**2**	**0**	**0**	**5**	**22**		**WD**	**WD**				**WD**	**WD**	**WD**
DRB1*13:50	13:50			2	0	1	0	0	0	0	3									
DRB1*13:50:01	13:50:01	HLA01539		0	5	0	2	0	0	0	7		WD						WD	WD
DRB1*13:50:02	13:50:02	HLA03182		1	0	5	0	0	0	3	9			WD					WD	WD
DRB1*13:50:03	13:50:03	HLA11901		1	0	0	0	0	0	2	3									
DRB1*13:52	13:52	HLA01643		0	0	54	0	0	0	0	54			WD					WD	WD
DRB1*13:53	13:53	HLA01622		1	0	6	0	0	0	1	8			WD					WD	WD

Supplemental Table 11: HLA-DRB1 Allele Summary [a]				Allele Count by Population Group [b]								3.0.0 CIWD Category by Population Group [c]								
Allele	Genomic Typing	Allele ID	G group	AFA	API	EURO	MENA	HIS	NAM	UNK	Total	AFA	API	EURO	MENA	HIS	NAM	UNK	Total	Highest Frequency
DRB1*13:54	13:54	HLA01623		0	0	5	8	0	0	2	15			WD	WD				WD	WD
DRB1*13:56	13:56	HLA01724		0	0	15	0	18	0	5	38			WD		I		WD	WD	I
DRB1*13:57	13:57	HLA01730		0	0	1	0	0	0	0	1									
DRB1*13:59	13:59	HLA01737		4	1	8	0	87	26	16	142			WD		C	C	I	WD	C
**DRB1*13:61 total**	**13:61 total**			**0**	**5**	**65**	**0**	**1**	**0**	**1**	**72**		**WD**	**WD**					**WD**	**WD**
DRB1*13:61	13:61			0	0	5	0	0	0	0	5			WD					WD	WD
DRB1*13:61:01	13:61:01	HLA01791		0	5	54	0	1	0	0	60		WD	WD					WD	WD
DRB1*13:61:02	13:61:02	HLA06497		0	0	6	0	0	0	1	7			WD					WD	WD
DRB1*13:62	13:62	HLA01813		0	0	17	0	0	0	0	17			WD					WD	WD
DRB1*13:63	13:63	HLA01854		0	0	21	2	0	0	0	23			WD					WD	WD
DRB1*13:64	13:64	HLA01932		0	0	1	0	0	0	2	3									
DRB1*13:65	13:65	HLA01991		0	0	5	0	0	0	0	5			WD					WD	WD
**DRB1*13:66 total**	**13:66 total**			**1**	**0**	**2**	**2**	**0**	**0**	**0**	**5**								**WD**	**WD**
DRB1*13:66	13:66			0	0	1	0	0	0	0	1									
DRB1*13:66:02	13:66:02	HLA04689		1	0	1	2	0	0	0	4									
DRB1*13:67	13:67	HLA02030		0	0	25	0	1	0	1	27			WD					WD	WD
DRB1*13:68	13:68	HLA02158		0	0	1	0	0	0	1	2									
DRB1*13:69	13:69	HLA02289		5	0	1	0	0	1	1	8	WD							WD	WD
DRB1*13:70	13:70	HLA02359		0	0	13	0	0	0	0	13			WD					WD	WD
DRB1*13:75	13:75	HLA02702		0	0	4	0	1	0	0	5								WD	WD
DRB1*13:77	13:77	HLA02893		0	0	4	0	1	0	1	6								WD	WD
DRB1*13:78	13:78	HLA02903		0	0	4	0	0	0	0	4									
DRB1*13:79	13:79	HLA02904		1	1	42	0	0	0	8	52			WD				WD	WD	WD
DRB1*13:80	13:80	HLA03034		1	0	5	0	3	0	4	13			WD					WD	WD
DRB1*13:81	13:81	HLA03058		1	0	0	0	1	0	3	5								WD	WD
DRB1*13:82	13:82	HLA03168		1	71	1	0	0	0	0	73		I						WD	I
DRB1*13:86	13:86	HLA03411		0	0	1	0	0	0	0	1									
DRB1*13:87	13:87	HLA03447		0	0	0	4	0	0	1	5								WD	WD
DRB1*13:88	13:88	HLA03640		1	0	15	0	0	0	0	16			WD					WD	WD
DRB1*13:89	13:89	HLA03848		0	0	2	0	0	0	1	3									
DRB1*13:90	13:90	HLA03850		0	0	7	0	0	0	0	7			WD					WD	WD

Supplemental Table 11: HLA-DRB1 Allele Summary [a]				Allele Count by Population Group [b]								3.0.0 CIWD Category by Population Group [c]								
Allele	Genomic Typing	Allele ID	G group	AFA	API	EURO	MENA	HIS	NAM	UNK	Total	AFA	API	EURO	MENA	HIS	NAM	UNK	Total	Highest Frequency
DRB1*13:91	13:91	HLA03857		0	0	11	0	0	0	0	11			WD					WD	WD
DRB1*13:92	13:92	HLA03865		0	0	0	0	1	0	0	1									
DRB1*13:93	13:93	HLA04029		0	0	8	0	0	0	14	22			WD				I	WD	I
DRB1*13:95	13:95	HLA04653		0	0	7	0	0	0	1	8			WD					WD	WD
**DRB1*13:96 total**	**13:96 total**			**1**	**3**	**9**	**0**	**0**	**0**	**0**	**13**			**WD**					**WD**	**WD**
DRB1*13:96	13:96			0	0	3	0	0	0	0	3									
DRB1*13:96:01	13:96:01	HLA04665		1	0	1	0	0	0	0	2									
DRB1*13:96:02	13:96:02	HLA06774		0	3	5	0	0	0	0	8			WD					WD	WD
**DRB1*13:97 total**	**13:97 total**			**1**	**1**	**2**	**1**	**0**	**0**	**0**	**5**								**WD**	**WD**
DRB1*13:97	13:97			0	1	2	0	0	0	0	3									
DRB1*13:97:02	13:97:02	HLA07875		1	0	0	1	0	0	0	2									
DRB1*13:98	13:98	HLA04686		0	2	0	0	0	0	0	2									
DRB1*13:100	13:100	HLA05300		0	0	1	0	0	0	0	1									
DRB1*13:103	13:103	HLA05848		1	0	0	0	0	0	0	1									
DRB1*13:104	13:104	HLA05952		0	0	11	0	0	0	0	11			WD					WD	WD
DRB1*13:105	13:105	HLA05970		0	0	3	0	0	0	0	3									
DRB1*13:106	13:106	HLA05971		0	0	21	0	0	0	1	22			WD					WD	WD
DRB1*13:107	13:107	HLA05972		0	0	1	0	0	0	0	1									
DRB1*13:108	13:108	HLA05974		0	0	10	0	0	0	1	11			WD					WD	WD
DRB1*13:109	13:109	HLA05981		0	0	2	0	0	0	0	2									
DRB1*13:110	13:110	HLA05982		0	0	2	0	0	0	0	2									
DRB1*13:111	13:111	HLA05993		0	0	12	2	1	0	0	15			WD					WD	WD
DRB1*13:112	13:112	HLA05995		0	0	3	0	0	0	0	3									
DRB1*13:118	13:118	HLA06868		0	2	0	0	0	0	0	2									
DRB1*13:124	13:124	HLA07161		0	0	5	0	0	0	0	5			WD					WD	WD
DRB1*13:127	13:127	HLA07170		0	0	3	0	0	0	0	3									
DRB1*13:128	13:128	HLA07184		0	0	5	0	27	2	4	38			WD		I			WD	I
DRB1*13:131	13:131	HLA07258		0	0	1	0	0	0	0	1									
DRB1*13:134	13:134	HLA07301		0	2	0	0	0	0	0	2									
DRB1*13:137N	13:137N	HLA07512		0	0	2	0	0	0	0	2									
DRB1*13:138	13:138	HLA07519		0	0	3	0	0	0	0	3									

Supplemental Table 11: HLA-DRB1 Allele Summary [a]				Allele Count by Population Group [b]								3.0.0 CIWD Category by Population Group [c]								
Allele	Genomic Typing	Allele ID	G group	AFA	API	EURO	MENA	HIS	NAM	UNK	Total	AFA	API	EURO	MENA	HIS	NAM	UNK	Total	Highest Frequency
DRB1*13:139	13:139	HLA07802		0	0	0	0	0	1	0	1									
DRB1*13:141	13:141	HLA07879		0	0	1	3	0	0	0	4									
DRB1*13:142N	13:142N	HLA07880		0	0	3	0	0	0	0	3									
DRB1*13:144	13:144	HLA08856		0	0	0	1	0	0	1	2									
DRB1*13:146	13:146	HLA08861		0	0	2	0	0	0	0	2									
DRB1*13:148	13:148	HLA08864		0	0	1	0	0	0	0	1									
DRB1*13:150	13:150	HLA08871		0	0	5	0	0	0	0	5			WD					WD	WD
DRB1*13:152	13:152	HLA08873		0	0	1	0	0	0	0	1									
DRB1*13:154	13:154	HLA08887		0	0	1	0	0	0	0	1									
DRB1*13:155	13:155	HLA09064		2	0	1	0	0	0	2	5								WD	WD
DRB1*13:157	13:157	HLA09066		0	0	1	0	0	0	1	2									
DRB1*13:158	13:158	HLA09068		0	0	10	0	0	0	0	10			WD					WD	WD
DRB1*13:159	13:159	HLA09213		0	0	1	0	0	0	0	1									
DRB1*13:162	13:162	HLA09658		0	0	1	0	0	0	0	1									
DRB1*13:165	13:165	HLA10314		0	0	1	0	0	0	0	1									
DRB1*13:166	13:166	HLA10315		0	0	1	0	0	0	0	1									
DRB1*13:167	13:167	HLA10317		0	0	1	0	0	0	0	1									
DRB1*13:169	13:169	HLA10658		0	0	1	1	0	0	0	2									
DRB1*13:170	13:170	HLA11168		0	1	0	0	0	0	0	1									
DRB1*13:172	13:172	HLA11170		0	0	0	0	0	0	1	1									
DRB1*13:175	13:175	HLA11373		0	4	0	0	0	0	0	4									
DRB1*13:177	13:177	HLA11377		2	0	0	0	0	0	0	2									
DRB1*13:178	13:178	HLA11378		0	2	0	0	0	0	0	2									
DRB1*13:179	13:179	HLA11379		0	0	0	1	0	0	0	1									
DRB1*13:187	13:187	HLA12059		0	0	5	0	0	0	0	5			WD					WD	WD
DRB1*13:195	13:195	HLA12811		2	0	0	0	0	0	0	2									
DRB1*13:197	13:197	HLA12890		0	0	1	0	0	0	0	1									
DRB1*13:200N	13:200N	HLA13052		0	0	2	0	0	0	0	2									
DRB1*13:205	13:205	HLA13893		0	0	2	0	0	0	0	2									
DRB1*13:211	13:211	HLA14358		0	0	0	0	0	1	0	1									
DRB1*13:222	13:222	HLA15702		0	2	0	0	0	0	0	2									

Supplemental Table 11: HLA-DRB1 Allele Summary [a]				Allele Count by Population Group [b]								3.0.0 CIWD Category by Population Group [c]								
Allele	Genomic Typing	Allele ID	G group	AFA	API	EURO	MENA	HIS	NAM	UNK	Total	AFA	API	EURO	MENA	HIS	NAM	UNK	Total	Highest Frequency
DRB1*13:224	13:224	HLA15704		0	0	2	0	1	0	1	4									
DRB1*13:234	13:234	HLA17038		0	0	1	0	0	0	0	1									
DRB1*13:237	13:237	HLA17169		0	0	1	0	0	0	1	2									
DRB1*13:CODE	13:CODE			4368	1200	55181	1419	4891	301	6460	73820	NA	NA	NA	NA	NA	NA	NA	NA	NA
**DRB1*14:01 total**	**14:01 total**			**6649**	**20054**	**294608**	**16557**	**12321**	**1128**	**35212**	**386529**	**C**	**C**	**C**	**C**	**C**	**C**	**C**	**C**	**C**
DRB1*14:01	14:01			0	0	455	3	1	0	44	503			I				I	I	I
DRB1*14:01P	14:01P			0	2	2053	4	4	0	7	2070			C				WD	C	C
**DRB1*14:01:01G total**	**14:01:01G total**			**6643**	**20043**	**291535**	**16545**	**12314**	**1128**	**35108**	**383316**	**C**	**C**	**C**	**C**	**C**	**C**	**C**	**C**	**C**
DRB1*14:01:01G	14:01:01G		14:01:01G	3630	13560	93537	5152	7336	746	28260	152221	C	C	C	C	C	C	C	C	C
DRB1*14:01:01	14:01:01	HLA00833	14:01:01G	72	185	21072	3626	549	32	577	26113	C	C	C	C	C	C	C	C	C
DRB1*14:54:01	14:54:01		14:01:01G	2941	6297	176926	7767	4429	350	6271	204981	C	C	C	C	C	C	C	C	C
DRB1*14:54:06	14:54:06	HLA16357	14:01:01G	0	1	0	0	0	0	0	1									
DRB1*14:01:02	14:01:02	HLA01278		6	2	29	3	1	0	2	43	WD		WD					WD	WD
DRB1*14:01:04	14:01:04	HLA09343		0	0	1	0	0	0	0	1									
**DRB1*14:54 total**	**14:54 total**			**2941**	**6305**	**177461**	**7769**	**4430**	**350**	**6322**	**205578**	**C**	**C**	**C**	**C**	**C**	**C**	**C**	**C**	**C**
DRB1*14:54	14:54			0	6	532	2	1	0	48	589		WD	I				I	I	I
DRB1*14:54:02	14:54:02	HLA07561		0	0	3	0	0	0	3	6								WD	WD
DRB1*14:54:04	14:54:04	HLA11933		0	1	0	0	0	0	0	1									
**DRB1*14:02 total**	**14:02 total**			**428**	**775**	**4300**	**65**	**14390**	**1921**	**5361**	**27240**	**C**	**C**	**C**	**C**	**C**	**C**	**C**	**C**	**C**
DRB1*14:02	14:02			202	201	1986	24	5710	899	2099	11121	C	C	C	WD	C	C	C	C	C
DRB1*14:02P	14:02P			0	0	1	0	0	0	0	1									
**DRB1*14:02:01G total**	**14:02:01G total**			**226**	**574**	**2305**	**41**	**8664**	**1022**	**3254**	**16086**	**C**	**C**	**C**	**C**	**C**	**C**	**C**	**C**	**C**
DRB1*14:02:01G	14:02:01G		14:02:01G	0	331	0	0	0	0	0	331		C						I	C
DRB1*14:02:01	14:02:01		14:02:01G	226	243	2305	41	8664	1022	3254	15755	C	C	C	C	C	C	C	C	C
DRB1*14:02:02	14:02:02	HLA11176		0	0	8	0	16	0	8	32			WD		I		WD	WD	I
**DRB1*14:03 total**	**14:03 total**			**2**	**1252**	**573**	**291**	**6**	**1**	**282**	**2407**		**C**	**I**	**C**	**WD**		**C**	**C**	**C**
DRB1*14:03	14:03			0	75	21	11	0	0	20	127		I	WD	WD			I	WD	I
DRB1*14:03:01	14:03:01	HLA00835		2	1177	552	280	6	1	262	2280		C	I	C	WD		C	C	C
**DRB1*14:04 total**	**14:04 total**			**297**	**82950**	**11308**	**2121**	**517**	**242**	**3019**	**100454**	**C**	**C**	**C**	**C**	**C**	**C**	**C**	**C**	**C**
DRB1*14:04	14:04			169	24319	5300	1052	271	139	1324	32574	C	C	C	C	C	C	C	C	C
DRB1*14:04P	14:04P			0	0	8	0	0	0	0	8			WD					WD	WD

Supplemental Table 11: HLA-DRB1 Allele Summary [a]				Allele Count by Population Group [b]								3.0.0 CIWD Category by Population Group [c]								
Allele	Genomic Typing	Allele ID	G group	AFA	API	EURO	MENA	HIS	NAM	UNK	Total	AFA	API	EURO	MENA	HIS	NAM	UNK	Total	Highest Frequency
DRB1*14:04:01	14:04:01	HLA00836		128	58628	6000	1069	246	103	1695	67869	C	C	C	C	C	C	C	C	C
DRB1*14:04:03	14:04:03	HLA14050		0	3	0	0	0	0	0	3									
**DRB1*14:05 total**	**14:05 total**			**9**	**6028**	**616**	**224**	**21**	**2**	**780**	**7680**	**WD**	**C**	**I**	**C**	**I**		**C**	**C**	**C**
DRB1*14:05	14:05			0	418	35	14	6	0	59	532		C	WD	WD	WD		I	I	C
**DRB1*14:05:01G total**	**14:05:01G total**			**9**	**5608**	**581**	**210**	**15**	**2**	**721**	**7146**	**WD**	**C**	**I**	**C**	**I**		**C**	**C**	**C**
DRB1*14:05:01G	14:05:01G		14:05:01G	1	558	39	10	0	0	2	610		C	WD	WD				I	C
DRB1*14:05:01	14:05:01		14:05:01G	8	5050	542	200	15	2	719	6536	WD	C	I	C	I		C	C	C
DRB1*14:05:02	14:05:02	HLA01663		0	2	0	0	0	0	0	2									
**DRB1*14:06 total**	**14:06 total**			**216**	**951**	**2097**	**379**	**18560**	**964**	**3474**	**26641**	**C**	**C**	**C**	**C**	**C**	**C**	**C**	**C**	**C**
DRB1*14:06	14:06			20	81	277	26	2592	108	308	3412	WD	I	I	WD	C	C	C	C	C
DRB1*14:06P	14:06P			0	0	2	0	0	0	0	2									
DRB1*14:06:01	14:06:01	HLA00838		196	870	1783	353	15938	856	3123	23119	C	C	C	C	C	C	C	C	C
DRB1*14:06:02	14:06:02	HLA04354		0	0	35	0	30	0	43	108			WD		I		I	WD	I
**DRB1*14:07 total**	**14:07 total**			**31**	**1648**	**2849**	**481**	**706**	**27**	**475**	**6217**	**WD**	**C**	**C**	**C**	**C**	**C**	**C**	**C**	**C**
DRB1*14:07	14:07			2	41	249	28	98	2	28	448		I	I	WD	C		I	I	C
DRB1*14:07P	14:07P			0	0	1	0	0	0	0	1									
DRB1*14:07:01	14:07:01	HLA00839		28	1607	1901	453	605	22	422	5038	WD	C	C	C	C	C	C	C	C
DRB1*14:07:02	14:07:02	HLA01531		1	0	698	0	3	3	25	730			I				I	I	I
DRB1*14:08	14:08	HLA00840		1	211	16	0	1	0	265	494		C	WD				C	I	C
DRB1*14:09	14:09	HLA00841		0	46	3	0	0	0	1	50		I						WD	I
DRB1*14:10	14:10	HLA00842		4	903	13	3	0	9	27	959		C	WD			C	I	I	C
DRB1*14:11	14:11	HLA00843		1	273	94	5	7	0	11	391		C	WD	WD	WD		WD	I	C
**DRB1*14:12 total**	**14:12 total**			**0**	**79**	**83**	**118**	**3**	**0**	**13**	**296**		**I**	**WD**	**C**			**WD**	**I**	**C**
DRB1*14:12	14:12			0	6	6	16	0	0	3	31		WD	WD	WD				WD	WD
DRB1*14:12:01	14:12:01	HLA00844		0	73	77	102	3	0	10	265		I	WD	C			WD	I	C
DRB1*14:13	14:13	HLA00845		1	0	6	0	17	0	13	37			WD		I		WD	WD	I
DRB1*14:15	14:15	HLA00847		5	2495	21	12	1	7	40	2581	WD	C	WD	WD		C	I	C	C
DRB1*14:16	14:16	HLA00848		0	0	236	7	2	0	25	270			I	WD			I	I	I
DRB1*14:17	14:17	HLA00849		2	12	413	58	24	0	53	562		WD	I	C	I		I	I	C
DRB1*14:18	14:18	HLA00850		0	290	9	0	0	0	14	313		C	WD				I	I	C
DRB1*14:19	14:19	HLA00851		5	212	40	3	1	2	9	272	WD	C	WD				WD	I	C

Supplemental Table 11: HLA-DRB1 Allele Summary [a]				Allele Count by Population Group [b]								3.0.0 CIWD Category by Population Group [c]								
**Allele**	**Genomic Typing**	**Allele ID**	**G group**	**AFA**	**API**	**EURO**	**MENA**	**HIS**	**NAM**	**UNK**	**Total**	**AFA**	**API**	**EURO**	**MENA**	**HIS**	**NAM**	**UNK**	**Total**	**Highest Frequency**
DRB1*14:20	14:20	HLA00852		0	3	105	2	1	0	7	118			WD				WD	WD	WD
DRB1*14:21	14:21	HLA00853		0	0	86	0	4	1	6	97			WD				WD	WD	WD
DRB1*14:22	14:22	HLA00854		0	70	3	0	0	0	6	79		I					WD	WD	I
**DRB1*14:23 total**	**14:23 total**			**1**	**4**	**77**	**4**	**3**	**1**	**6**	**96**			**WD**				**WD**	**WD**	**WD**
DRB1*14:23	14:23			0	0	8	2	0	0	0	10			WD					WD	WD
DRB1*14:23:01	14:23:01	HLA00855		1	1	68	2	3	1	5	81			WD				WD	WD	WD
DRB1*14:23:03	14:23:03	HLA04007		0	3	0	0	0	0	0	3									
DRB1*14:23:04	14:23:04	HLA08865		0	0	1	0	0	0	1	2									
DRB1*14:24	14:24	HLA00856		3	4	16	0	244	13	26	306			WD		C	C	I	I	C
DRB1*14:25	14:25	HLA00857		0	44	8	0	0	0	0	52		I	WD					WD	I
DRB1*14:26	14:26	HLA00858		0	1	0	0	0	0	0	1									
**DRB1*14:27 total**	**14:27 total**			**0**	**1**	**1**	**0**	**3**	**0**	**2**	**7**								**WD**	**WD**
DRB1*14:27	14:27			0	1	1	0	3	0	2	7								WD	WD
DRB1*14:28	14:28	HLA00860		0	72	2	0	0	0	4	78		I						WD	I
DRB1*14:29	14:29	HLA00861		0	1	0	0	0	0	1	2									
DRB1*14:31	14:31	HLA00863		0	4	0	0	0	0	0	4									
**DRB1*14:32 total**	**14:32 total**			**0**	**0**	**8**	**1**	**0**	**0**	**3**	**12**			**WD**					**WD**	**WD**
DRB1*14:32	14:32			0	0	1	1	0	0	0	2									
DRB1*14:32:01	14:32:01	HLA00864		0	0	1	0	0	0	0	1									
DRB1*14:32:02	14:32:02	HLA03109		0	0	6	0	0	0	3	9			WD					WD	WD
DRB1*14:33	14:33	HLA01011		0	52	19	31	1	0	5	108		I	WD	WD			WD	WD	I
DRB1*14:35	14:35	HLA01161		0	5	10	0	0	0	0	15		WD	WD					WD	WD
**DRB1*14:38 total**	**14:38 total**			**0**	**0**	**1**	**3**	**0**	**0**	**0**	**4**									
DRB1*14:38	14:38			0	0	1	1	0	0	0	2									
DRB1*14:38:01	14:38:01	HLA01378		0	0	0	2	0	0	0	2									
DRB1*14:41	14:41	HLA01500		1	0	2	0	5	3	5	16					WD		WD	WD	WD
**DRB1*14:44 total**	**14:44 total**			**0**	**13**	**0**	**0**	**0**	**0**	**0**	**13**		**WD**						**WD**	**WD**
DRB1*14:44:01	14:44:01	HLA01629		0	10	0	0	0	0	0	10		WD						WD	WD
DRB1*14:44:02	14:44:02	HLA03036		0	3	0	0	0	0	0	3									
DRB1*14:45	14:45	HLA01630		0	2	0	0	0	0	0	2									
DRB1*14:46	14:46	HLA01693		0	0	0	0	5	0	0	5					WD			WD	WD

Supplemental Table 11: HLA-DRB1 Allele Summary [a]				Allele Count by Population Group [b]								3.0.0 CIWD Category by Population Group [c]								
Allele	Genomic Typing	Allele ID	G group	AFA	API	EURO	MENA	HIS	NAM	UNK	Total	AFA	API	EURO	MENA	HIS	NAM	UNK	Total	Highest Frequency
DRB1*14:47	14:47	HLA01733		0	0	50	0	0	3	3	56			WD					WD	WD
DRB1*14:48	14:48	HLA01734		0	2	11	2	72	5	20	112			WD		C	WD	I	WD	C
DRB1*14:49	14:49	HLA02131		0	1	0	0	0	0	0	1									
DRB1*14:50	14:50	HLA02210		1	11	1	0	0	6	1	20		WD				WD		WD	WD
DRB1*14:52	14:52	HLA02215		0	0	1	0	5	0	3	9					WD			WD	WD
DRB1*14:53	14:53	HLA02348		0	0	1	0	0	0	0	1									
DRB1*14:55	14:55	HLA02409		0	0	4	0	0	0	0	4									
DRB1*14:58	14:58	HLA02487		0	0	6	0	0	0	0	6			WD					WD	WD
DRB1*14:59	14:59	HLA02520		0	0	2	0	0	0	0	2									
DRB1*14:60	14:60	HLA02563		0	0	10	0	0	0	1	11			WD					WD	WD
DRB1*14:61	14:61	HLA02572		0	18	2	0	0	0	3	23		I						WD	I
DRB1*14:64	14:64	HLA02728		0	0	1	0	0	0	0	1									
**DRB1*14:68 total**	**14:68 total**			**0**	**14**	**0**	**0**	**0**	**0**	**0**	**14**		**I**						**WD**	**I**
DRB1*14:68	14:68			0	3	0	0	0	0	0	3									
DRB1*14:68:01	14:68:01	HLA02923		0	11	0	0	0	0	0	11		WD						WD	WD
DRB1*14:69	14:69	HLA03039		0	25	0	0	0	0	0	25		I						WD	I
DRB1*14:70	14:70	HLA03041		1	1	70	0	0	0	11	83			WD				WD	WD	WD
DRB1*14:73	14:73	HLA03044		0	4	0	0	0	0	0	4									
DRB1*14:74	14:74	HLA03052		0	0	1	0	0	0	0	1									
DRB1*14:75	14:75	HLA03194		0	0	8	0	0	0	1	9			WD					WD	WD
DRB1*14:76	14:76	HLA03227		0	2	0	0	0	0	0	2									
DRB1*14:79	14:79	HLA03325		0	3	3	0	0	0	0	6								WD	WD
DRB1*14:82	14:82	HLA03446		0	0	4	0	0	0	0	4									
DRB1*14:83	14:83	HLA03461		1	0	1	0	1	0	1	4									
DRB1*14:86	14:86	HLA03764		0	0	5	0	1	0	0	6			WD					WD	WD
DRB1*14:87	14:87	HLA03845		0	0	27	0	0	0	0	27			WD					WD	WD
DRB1*14:88	14:88	HLA03849		0	0	0	2	0	0	0	2									
DRB1*14:89	14:89	HLA03862		0	0	4	0	0	0	0	4									
DRB1*14:90	14:90	HLA03863		0	0	1	4	0	0	1	6								WD	WD
DRB1*14:94	14:94	HLA04409		0	0	0	0	2	0	0	2									
DRB1*14:95	14:95	HLA04614		0	0	6	0	0	0	0	6			WD					WD	WD

Supplemental Table 11: HLA-DRB1 Allele Summary [a]				Allele Count by Population Group [b]								3.0.0 CIWD Category by Population Group [c]								
Allele	Genomic Typing	Allele ID	G group	AFA	API	EURO	MENA	HIS	NAM	UNK	Total	AFA	API	EURO	MENA	HIS	NAM	UNK	Total	Highest Frequency
DRB1*14:96	14:96	HLA04581		0	1	0	0	0	0	0	1									
DRB1*14:97	14:97	HLA04687		0	0	15	0	0	0	1	16			WD					WD	WD
DRB1*14:99	14:99	HLA04782		0	0	8	0	0	0	0	8			WD					WD	WD
DRB1*14:101	14:101	HLA06010		0	1	5	1	0	0	0	7			WD					WD	WD
DRB1*14:106	14:106	HLA06299		0	0	1	0	5	0	1	7					WD			WD	WD
DRB1*14:108	14:108	HLA06308		0	0	0	0	2	0	0	2									
DRB1*14:109	14:109	HLA06309		0	0	1	0	3	0	6	10							WD	WD	WD
DRB1*14:110	14:110	HLA06480		0	0	0	6	0	0	0	6				WD				WD	WD
DRB1*14:111	14:111	HLA06481		0	0	6	0	0	0	1	7			WD					WD	WD
DRB1*14:114	14:114	HLA06775		5	0	0	0	0	0	0	5	WD							WD	WD
DRB1*14:117	14:117	HLA06907		0	0	1	0	0	0	0	1									
DRB1*14:119	14:119	HLA07172		0	1	0	0	0	0	0	1									
DRB1*14:122	14:122	HLA07381		0	1	3	0	0	0	1	5								WD	WD
DRB1*14:123	14:123	HLA07513		0	0	2	0	0	0	0	2									
**DRB1*14:126 total**	**14:126 total**			**0**	**17**	**0**	**0**	**0**	**0**	**0**	**17**		**I**						**WD**	**I**
DRB1*14:126	14:126			0	1	0	0	0	0	0	1									
DRB1*14:126:01	14:126:01	HLA08086		0	16	0	0	0	0	0	16		I						WD	I
DRB1*14:128	14:128	HLA08553		0	0	3	0	0	0	0	3									
DRB1*14:129	14:129	HLA08565		0	0	3	0	0	0	0	3									
DRB1*14:133	14:133	HLA08860		1	3	2	0	0	0	0	6								WD	WD
DRB1*14:138	14:138	HLA09103		0	4	0	0	0	0	0	4									
DRB1*14:139	14:139	HLA09104		0	0	0	0	0	0	1	1									
DRB1*14:141	14:141	HLA09720		0	7	1	0	0	0	0	8		WD						WD	WD
DRB1*14:142	14:142	HLA09762		0	1	0	0	0	0	0	1									
DRB1*14:145	14:145	HLA10310		0	1	0	0	0	0	0	1									
DRB1*14:151	14:151	HLA11689		0	0	1	0	0	0	0	1									
DRB1*14:152N	14:152N	HLA11710		0	1	3	0	0	0	0	4									
DRB1*14:153	14:153	HLA11752		0	0	0	1	0	0	0	1									
DRB1*14:157	14:157	HLA11906		0	1	1	0	0	0	0	2									
DRB1*14:158	14:158	HLA12218		0	2	0	1	0	0	0	3									
DRB1*14:162	14:162	HLA13593		0	1	0	0	0	0	0	1									

Supplemental Table 11: HLA-DRB1 Allele Summary [a]				Allele Count by Population Group [b]								3.0.0 CIWD Category by Population Group [c]								
Allele	Genomic Typing	Allele ID	G group	AFA	API	EURO	MENA	HIS	NAM	UNK	Total	AFA	API	EURO	MENA	HIS	NAM	UNK	Total	Highest Frequency
DRB1*14:166N	14:166N	HLA13973		0	0	1	0	0	0	2	3									
DRB1*14:169	14:169	HLA14266		0	0	1	0	0	0	1	2									
DRB1*14:170	14:170	HLA14361		0	0	0	0	1	0	0	1									
DRB1*14:171	14:171	HLA14410		0	0	1	0	0	0	0	1									
DRB1*14:175	14:175	HLA15077		0	3	0	0	0	0	0	3									
DRB1*14:178	14:178	HLA15336		0	0	1	0	0	0	0	1									
DRB1*14:CODE	14:CODE			732	1003	18493	1030	1550	134	2392	25334	NA	NA	NA	NA	NA	NA	NA	NA	NA
**DRB1*15:01 total**	**15:01 total**			**9821**	**129365**	**1507494**	**23939**	**41500**	**4043**	**123710**	**1839872**	**C**	**C**	**C**	**C**	**C**	**C**	**C**	**C**	**C**
DRB1*15:01	15:01			5095	15239	411297	5106	19404	2363	52072	510576	C	C	C	C	C	C	C	C	C
DRB1*15:01P	15:01P			0	0	172	0	0	0	0	172			I					I	I
**DRB1*15:01:01G total**	**15:01:01G total**			**4725**	**114098**	**1095584**	**18823**	**22095**	**1680**	**71591**	**1328596**	**C**	**C**	**C**	**C**	**C**	**C**	**C**	**C**	**C**
DRB1*15:01:01G	15:01:01G		15:01:01G	392	83418	42300	4218	5407	140	40393	176268	C	C	C	C	C	C	C	C	C
DRB1*15:01:01	15:01:01		15:01:01G	4207	30597	1046407	14586	15973	1469	30441	1143680	C	C	C	C	C	C	C	C	C
DRB1*15:01:01:01	15:01:01:01	HLA00865	15:01:01G	49	26	3199	14	340	31	375	4034	C	I	C	WD	C	C	C	C	C
DRB1*15:01:01:02	15:01:01:02	HLA03453	15:01:01G	76	48	3504	5	375	40	382	4430	C	I	C	WD	C	C	C	C	C
DRB1*15:01:01:03	15:01:01:03	HLA07374	15:01:01G	0	0	131	0	0	0	0	131			I					WD	I
DRB1*15:01:17	15:01:17	HLA06669	15:01:01G	1	9	4	0	0	0	0	14		WD						WD	WD
DRB1*15:01:32	15:01:32	HLA15611	15:01:01G	0	0	37	0	0	0	0	37			WD					WD	WD
DRB1*15:145	15:145	HLA16750	15:01:01G	0	0	2	0	0	0	0	2									
DRB1*15:01:02	15:01:02	HLA00866		0	1	205	0	0	0	24	230			I				I	I	I
DRB1*15:01:03	15:01:03	HLA01468		0	0	25	0	1	0	3	29			WD					WD	WD
DRB1*15:01:04	15:01:04	HLA01582		0	4	41	0	0	0	5	50			WD				WD	WD	WD
DRB1*15:01:06	15:01:06	HLA02569		0	3	2	0	0	0	0	5								WD	WD
DRB1*15:01:07	15:01:07	HLA04033		0	14	0	0	0	0	1	15		I						WD	I
DRB1*15:01:08	15:01:08	HLA04361		0	0	97	0	0	0	8	105			WD				WD	WD	WD
DRB1*15:01:10	15:01:10	HLA04655		0	0	3	0	0	0	0	3									
DRB1*15:01:11	15:01:11	HLA04656		0	0	6	0	0	0	1	7			WD					WD	WD
DRB1*15:01:12	15:01:12	HLA04657		1	0	17	0	0	0	4	22			WD					WD	WD
DRB1*15:01:13	15:01:13	HLA06473		0	1	9	0	0	0	0	10			WD					WD	WD
DRB1*15:01:14	15:01:14	HLA06476		0	0	2	0	0	0	0	2									
DRB1*15:01:15	15:01:15	HLA06487		0	0	2	0	0	0	0	2									

Supplemental Table 11: HLA-DRB1 Allele Summary [a]				Allele Count by Population Group [b]								3.0.0 CIWD Category by Population Group [c]								
**Allele**	**Genomic Typing**	**Allele ID**	**G group**	**AFA**	**API**	**EURO**	**MENA**	**HIS**	**NAM**	**UNK**	**Total**	**AFA**	**API**	**EURO**	**MENA**	**HIS**	**NAM**	**UNK**	**Total**	**Highest Frequency**
DRB1*15:01:16	15:01:16	HLA06668		0	0	0	10	0	0	1	11				WD				WD	WD
DRB1*15:01:18	15:01:18	HLA07505		0	0	1	0	0	0	0	1									
DRB1*15:01:20	15:01:20	HLA08229		0	0	8	0	0	0	0	8			WD					WD	WD
DRB1*15:01:22	15:01:22	HLA10301		0	2	0	0	0	0	0	2									
DRB1*15:01:25	15:01:25	HLA12364		0	0	23	0	0	0	0	23			WD					WD	WD
DRB1*15:01:27	15:01:27	HLA13814		0	2	0	0	0	0	0	2									
DRB1*15:01:31	15:01:31	HLA15693		0	1	0	0	0	0	0	1									
**DRB1*15:02 total**	**15:02 total**			**1142**	**135036**	**99345**	**13856**	**7830**	**810**	**26660**	**284679**	**C**	**C**	**C**	**C**	**C**	**C**	**C**	**C**	**C**
DRB1*15:02	15:02			102	3704	5688	518	1172	56	1911	13151	C	C	C	C	C	C	C	C	C
DRB1*15:02P	15:02P			0	0	78	1	0	0	1	80			WD					WD	WD
**DRB1*15:02:01G total**	**15:02:01G total**			**1007**	**111474**	**92906**	**12930**	**6631**	**717**	**24312**	**249977**	**C**	**C**	**C**	**C**	**C**	**C**	**C**	**C**	**C**
DRB1*15:02:01G	15:02:01G		15:02:01G	117	67890	21326	1807	952	27	3583	95702	C	C	C	C	C	C	C	C	C
DRB1*15:02:01	15:02:01		15:02:01G	888	43456	71451	11114	5642	688	20691	153930	C	C	C	C	C	C	C	C	C
DRB1*15:02:01:01	15:02:01:01	HLA00867	15:02:01G	2	109	115	8	36	2	35	307		I	WD	WD	I		I	I	I
DRB1*15:02:01:02	15:02:01:02	HLA14828	15:02:01G	0	0	7	0	0	0	0	7			WD					WD	WD
DRB1*15:140	15:140	HLA16027	15:02:01G	0	19	7	1	1	0	3	31		I	WD					WD	I
DRB1*15:02:02	15:02:02	HLA00868		32	19269	666	399	27	36	422	20851	WD	C	I	C	I	C	C	C	C
DRB1*15:02:04	15:02:04	HLA02721		0	32	0	0	0	0	2	34		I						WD	I
DRB1*15:02:05	15:02:05	HLA03254		1	551	7	8	0	1	12	580		C	WD	WD			WD	I	C
DRB1*15:02:08	15:02:08	HLA05129		0	3	0	0	0	0	0	3									
DRB1*15:02:10	15:02:10	HLA09107		0	1	0	0	0	0	0	1									
DRB1*15:02:14	15:02:14	HLA13815		0	1	0	0	0	0	0	1									
DRB1*15:02:15	15:02:15	HLA13889		0	1	0	0	0	0	0	1									
**DRB1*15:03 total**	**15:03 total**			**44994**	**321**	**4318**	**1893**	**6705**	**1272**	**10576**	**70079**	**C**	**C**	**C**	**C**	**C**	**C**	**C**	**C**	**C**
DRB1*15:03	15:03			5514	22	328	62	1124	133	1092	8275	C	I	I	C	C	C	C	C	C
DRB1*15:03P	15:03P			1	0	0	0	0	0	0	1									
**DRB1*15:03:01G total**	**15:03:01G total**			**39476**	**299**	**3990**	**1831**	**5581**	**1138**	**9484**	**61799**	**C**	**C**	**C**	**C**	**C**	**C**	**C**	**C**	**C**
DRB1*15:03:01G	15:03:01G		15:03:01G	8861	174	2522	1634	1205	67	2975	17438	C	C	C	C	C	C	C	C	C
DRB1*15:03:01	15:03:01		15:03:01G	29462	121	1425	195	4170	1029	6276	42678	C	I	C	C	C	C	C	C	C
DRB1*15:03:01:01	15:03:01:01	HLA00870	15:03:01G	537	3	26	0	106	19	117	808	C		WD		C	C	I	I	C
DRB1*15:03:01:02	15:03:01:02	HLA03454	15:03:01G	616	1	17	2	100	23	116	875	C		WD		C	C	I	I	C

Supplemental Table 11: HLA-DRB1 Allele Summary [a]				Allele Count by Population Group [b]								3.0.0 CIWD Category by Population Group [c]								
Allele	Genomic Typing	Allele ID	G group	AFA	API	EURO	MENA	HIS	NAM	UNK	Total	AFA	API	EURO	MENA	HIS	NAM	UNK	Total	Highest Frequency
DRB1*15:03:02	15:03:02	HLA06472		3	0	0	0	0	1	0	4									
DRB1*15:04	15:04	HLA00871		7	2354	432	23	20	8	91	2935	WD	C	I	WD	I	C	I	C	C
DRB1*15:05	15:05	HLA00872		0	4	15	0	1	2	2	24			WD					WD	WD
**DRB1*15:06 total**	**15:06 total**			**17**	**12710**	**304**	**404**	**35**	**28**	**292**	**13790**	**WD**	**C**	**I**	**C**	**I**	**C**	**C**	**C**	**C**
DRB1*15:06	15:06			0	1290	40	43	6	4	28	1411		C	WD	C	WD		I	I	C
DRB1*15:06:01	15:06:01	HLA00873		17	11409	262	360	29	24	263	12364	WD	C	I	C	I	C	C	C	C
DRB1*15:06:02	15:06:02	HLA07864		0	11	2	1	0	0	1	15		WD						WD	WD
**DRB1*15:07 total**	**15:07 total**			**5**	**7**	**150**	**12**	**3**	**0**	**12**	**189**	**WD**	**WD**	**I**	**WD**			**WD**	**I**	**I**
DRB1*15:07	15:07			3	0	18	3	1	0	0	25			WD					WD	WD
DRB1*15:07:01	15:07:01	HLA00874		2	7	131	9	2	0	12	163		WD	I	WD			WD	I	I
DRB1*15:07:02	15:07:02	HLA08230		0	0	1	0	0	0	0	1									
DRB1*15:09	15:09	HLA01159		1	0	2	0	1	0	0	4									
DRB1*15:10	15:10	HLA01211		0	0	16	0	1	0	2	19			WD					WD	WD
DRB1*15:11	15:11	HLA01340		1	15	27	10	4	0	8	65		I	WD	WD			WD	WD	I
DRB1*15:12	15:12	HLA01502		0	0	3	0	0	0	0	3									
DRB1*15:13	15:13	HLA01541		0	0	29	0	0	0	0	29			WD					WD	WD
DRB1*15:14	15:14	HLA01842		0	99	7	0	2	0	24	132		I	WD				I	WD	I
**DRB1*15:15 total**	**15:15 total**			**0**	**5**	**5**	**0**	**0**	**0**	**0**	**10**		**WD**	**WD**					**WD**	**WD**
DRB1*15:15	15:15			0	3	4	0	0	0	0	7								WD	WD
DRB1*15:15:01	15:15:01	HLA01936		0	2	1	0	0	0	0	3									
DRB1*15:18	15:18	HLA02326		1	0	156	1	2	0	3	163			I					I	I
DRB1*15:20	15:20	HLA02571		1	62	16	0	1	1	2	83		I	WD					WD	I
DRB1*15:22	15:22	HLA02575		7	0	41	0	1	0	6	55	WD		WD				WD	WD	WD
DRB1*15:23	15:23	HLA03033		18	0	1	0	1	0	4	24	WD							WD	WD
DRB1*15:24	15:24	HLA03035		0	0	53	0	2	0	44	99			WD				I	WD	I
DRB1*15:25	15:25	HLA03053		0	0	1	0	0	0	0	1									
DRB1*15:27	15:27	HLA03143		0	0	2	2	0	0	0	4									
DRB1*15:28	15:28	HLA03381		0	0	0	0	0	0	2	2									
DRB1*15:31	15:31	HLA03386		0	6	0	0	0	0	1	7		WD						WD	WD
DRB1*15:32	15:32	HLA03635		0	0	0	0	1	0	0	1									
DRB1*15:33	15:33	HLA03766		0	0	27	0	0	0	0	27			WD					WD	WD

Supplemental Table 11: HLA-DRB1 Allele Summary [a]				Allele Count by Population Group [b]								3.0.0 CIWD Category by Population Group [c]								
**Allele**	**Genomic Typing**	**Allele ID**	**G group**	**AFA**	**API**	**EURO**	**MENA**	**HIS**	**NAM**	**UNK**	**Total**	**AFA**	**API**	**EURO**	**MENA**	**HIS**	**NAM**	**UNK**	**Total**	**Highest Frequency**
DRB1*15:35	15:35	HLA04362		0	1	3	0	0	0	0	4									
DRB1*15:36	15:36	HLA04363		0	0	10	0	0	0	0	10			WD					WD	WD
**DRB1*15:37 total**	**15:37 total**			**0**	**0**	**26**	**1**	**0**	**0**	**0**	**27**			**WD**					**WD**	**WD**
DRB1*15:37	15:37			0	0	4	0	0	0	0	4									
DRB1*15:37:01	15:37:01	HLA04364		0	0	17	0	0	0	0	17			WD					WD	WD
DRB1*15:37:02	15:37:02	HLA08593		0	0	5	1	0	0	0	6			WD					WD	WD
DRB1*15:38	15:38	HLA04365		0	75	0	0	0	0	0	75		I						WD	I
DRB1*15:39	15:39	HLA04366		0	0	0	0	0	0	1	1									
DRB1*15:40	15:40	HLA04368		0	0	58	0	0	0	1	59			WD					WD	WD
DRB1*15:41	15:41	HLA04369		0	0	1	0	0	0	0	1									
DRB1*15:43	15:43	HLA04671		0	1	2	0	0	0	1	4									
DRB1*15:47	15:47	HLA05387		0	8	0	0	0	0	0	8		WD						WD	WD
DRB1*15:49	15:49	HLA05392		0	1	0	0	0	0	0	1									
DRB1*15:53	15:53	HLA06471		0	1	0	0	0	0	0	1									
DRB1*15:54	15:54	HLA06474		0	0	1	0	0	0	0	1									
DRB1*15:55	15:55	HLA06475		0	0	8	0	0	0	1	9			WD					WD	WD
DRB1*15:56	15:56	HLA06486		0	0	0	5	0	0	20	25				WD			I	WD	I
DRB1*15:57	15:57	HLA06504		5	0	0	0	0	0	1	6	WD							WD	WD
DRB1*15:59	15:59	HLA07143		0	0	1	0	0	0	0	1									
DRB1*15:60	15:60	HLA07144		0	0	1	0	0	0	0	1									
DRB1*15:61	15:61	HLA07153		0	1	4	0	0	0	0	5								WD	WD
DRB1*15:62	15:62	HLA07164		0	0	1	0	0	0	0	1									
DRB1*15:64	15:64	HLA07248		0	0	4	0	0	0	0	4									
DRB1*15:65	15:65	HLA07249		0	1	5	0	0	0	0	6			WD					WD	WD
**DRB1*15:66 total**	**15:66 total**			**0**	**0**	**1**	**0**	**0**	**0**	**0**	**1**									
DRB1*15:66:01	15:66:01	HLA07275		0	0	1	0	0	0	0	1									
DRB1*15:67	15:67	HLA07337		0	0	21	0	0	0	1	22			WD					WD	WD
DRB1*15:68	15:68	HLA07361		0	39	0	0	0	0	0	39		I						WD	I
DRB1*15:71	15:71	HLA07504		0	2	1	0	0	0	0	3									
DRB1*15:73	15:73	HLA07865		0	3	0	0	0	0	0	3									
DRB1*15:74	15:74	HLA07866		0	0	4	0	0	0	0	4									

Supplemental Table 11: HLA-DRB1 Allele Summary [a]				Allele Count by Population Group [b]								3.0.0 CIWD Category by Population Group [c]								
Allele	Genomic Typing	Allele ID	G group	AFA	API	EURO	MENA	HIS	NAM	UNK	Total	AFA	API	EURO	MENA	HIS	NAM	UNK	Total	Highest Frequency
DRB1*15:75	15:75	HLA07867		0	0	2	0	0	0	0	2									
DRB1*15:76	15:76	HLA08090		0	0	1	0	0	0	1	2									
DRB1*15:77	15:77	HLA08228		0	1	0	0	0	0	0	1									
DRB1*15:78	15:78	HLA08546		1	0	0	0	0	0	0	1									
DRB1*15:80N	15:80N	HLA08583		0	1	0	0	0	0	0	1									
DRB1*15:85	15:85	HLA08837		0	0	3	0	0	0	0	3									
DRB1*15:86	15:86	HLA08838		0	0	2	0	0	0	0	2									
DRB1*15:88	15:88	HLA08913		0	0	4	0	0	0	0	4									
DRB1*15:92	15:92	HLA09054		0	0	3	0	1	0	0	4									
DRB1*15:93	15:93	HLA09208		0	0	11	0	0	0	0	11			WD					WD	WD
DRB1*15:94	15:94	HLA09319		1	0	1	0	1	0	1	4									
DRB1*15:96	15:96	HLA09676		0	1	12	1	1	0	0	15			WD					WD	WD
DRB1*15:102	15:102	HLA10303		0	3	0	0	0	0	0	3									
**DRB1*15:104 total**	**15:104 total**			**0**	**1**	**1**	**0**	**0**	**0**	**0**	**2**									
DRB1*15:104	15:104			0	1	1	0	0	0	0	2									
DRB1*15:107	15:107	HLA11369		0	0	1	0	0	0	0	1									
DRB1*15:109	15:109	HLA11603		0	1	0	0	0	0	0	1									
DRB1*15:115N	15:115N	HLA12576		0	0	1	0	0	0	0	1									
DRB1*15:118	15:118	HLA13153		0	0	0	1	0	0	0	1									
DRB1*15:123	15:123	HLA13374		0	1	0	0	0	0	0	1									
DRB1*15:126	15:126	HLA13888		0	1	0	0	0	0	0	1									
DRB1*15:129N	15:129N	HLA14224		0	1	0	0	0	0	0	1									
DRB1*15:131	15:131	HLA14562		0	0	1	0	0	0	0	1									
DRB1*15:132	15:132	HLA15071		0	1	0	0	0	0	0	1									
DRB1*15:136	15:136	HLA14951		0	0	2	0	0	0	0	2									
DRB1*15:137N	15:137N	HLA14952		1	0	0	0	0	0	0	1									
DRB1*15:CODE	15:CODE			1292	1676	27213	649	2854	163	2958	36805	NA	NA	NA	NA	NA	NA	NA	NA	NA
**DRB1*16:01 total**	**16:01 total**			**939**	**1501**	**327466**	**13810**	**7002**	**585**	**17631**	**368934**	**C**	**C**	**C**	**C**	**C**	**C**	**C**	**C**	**C**
DRB1*16:01	16:01			100	51	14990	654	1277	55	1224	18351	C	I	C	C	C	C	C	C	C
DRB1*16:01P	16:01P			0	0	160	0	2	0	0	162			I					I	I
DRB1*16:01:01	16:01:01	HLA00876		839	1450	312275	13152	5723	530	16402	350371	C	C	C	C	C	C	C	C	C

Supplemental Table 11: HLA-DRB1 Allele Summary [a]				Allele Count by Population Group [b]								3.0.0 CIWD Category by Population Group [c]								
**Allele**	**Genomic Typing**	**Allele ID**	**G group**	**AFA**	**API**	**EURO**	**MENA**	**HIS**	**NAM**	**UNK**	**Total**	**AFA**	**API**	**EURO**	**MENA**	**HIS**	**NAM**	**UNK**	**Total**	**Highest Frequency**
DRB1*16:01:02	16:01:02	HLA00877		0	0	12	2	0	0	3	17			WD					WD	WD
DRB1*16:01:04	16:01:04	HLA09677		0	0	0	1	0	0	0	1									
DRB1*16:01:05	16:01:05	HLA09678		0	0	2	0	0	0	0	2									
DRB1*16:01:06	16:01:06	HLA12398		0	0	0	1	0	0	0	1									
DRB1*16:01:08	16:01:08	HLA13156		0	0	18	0	0	0	2	20			WD					WD	WD
DRB1*16:01:10	16:01:10	HLA14174		0	0	7	0	0	0	0	7			WD					WD	WD
DRB1*16:01:12	16:01:12	HLA16570		0	0	2	0	0	0	0	2									
**DRB1*16:02 total**	**16:02 total**			**5369**	**15430**	**24244**	**3882**	**14948**	**1361**	**8541**	**73775**	**C**	**C**	**C**	**C**	**C**	**C**	**C**	**C**	**C**
DRB1*16:02	16:02			543	681	1948	170	2055	132	900	6429	C	C	C	C	C	C	C	C	C
DRB1*16:02P	16:02P			0	0	14	0	1	0	0	15			WD					WD	WD
**DRB1*16:02:01G total**	**16:02:01G total**			**4826**	**14749**	**22282**	**3712**	**12891**	**1229**	**7639**	**67328**	**C**	**C**	**C**	**C**	**C**	**C**	**C**	**C**	**C**
DRB1*16:02:01G	16:02:01G		16:02:01G	291	5247	5965	430	1502	30	1966	15431	C	C	C	C	C	C	C	C	C
DRB1*16:02:01	16:02:01		16:02:01G	4512	9278	16278	3278	10817	1151	5603	50917	C	C	C	C	C	C	C	C	C
DRB1*16:02:01:01	16:02:01:01	HLA00878	16:02:01G	0	43	0	0	1	1	3	48		I						WD	I
DRB1*16:02:01:02	16:02:01:02	HLA14831	16:02:01G	23	181	39	4	571	47	67	932	WD	C	WD		C	C	I	I	C
DRB1*16:02:02	16:02:02	HLA00879		0	0	0	0	0	0	1	1									
DRB1*16:02:06	16:02:06	HLA14560		0	0	0	0	1	0	1	2									
DRB1*16:03	16:03	HLA00880		0	0	1	0	0	0	0	1									
**DRB1*16:04 total**	**16:04 total**			**0**	**0**	**50**	**1**	**1**	**0**	**11**	**63**			**WD**				**WD**	**WD**	**WD**
DRB1*16:04	16:04			0	0	49	1	1	0	11	62			WD				WD	WD	WD
DRB1*16:04:01	16:04:01	HLA00881		0	0	1	0	0	0	0	1									
**DRB1*16:05 total**	**16:05 total**			**0**	**15**	**553**	**170**	**4**	**0**	**22**	**764**		**I**	**I**	**C**			**I**	**I**	**C**
DRB1*16:05	16:05			0	0	41	7	1	0	1	50			WD	WD				WD	WD
DRB1*16:05:01	16:05:01	HLA00882		0	15	511	163	3	0	21	713		I	I	C			I	I	C
DRB1*16:05:02	16:05:02	HLA01838		0	0	1	0	0	0	0	1									
DRB1*16:07	16:07	HLA00884		0	0	92	5	37	1	46	181			WD	WD	I		I	I	I
DRB1*16:08	16:08	HLA00885		0	0	2	1	0	0	14	17							I	WD	I
**DRB1*16:09 total**	**16:09 total**			**0**	**1**	**69**	**6**	**2**	**0**	**2**	**80**			**WD**	**WD**				**WD**	**WD**
DRB1*16:09	16:09			0	0	19	1	0	0	1	21			WD					WD	WD
DRB1*16:09:01	16:09:01	HLA02130		0	1	50	5	2	0	1	59			WD	WD				WD	WD
DRB1*16:10	16:10	HLA02345		4	91	2	0	0	1	1	99		I						WD	I

Supplemental Table 11: HLA-DRB1 Allele Summary [a]				Allele Count by Population Group [b]								3.0.0 CIWD Category by Population Group [c]								
**Allele**	**Genomic Typing**	**Allele ID**	**G group**	**AFA**	**API**	**EURO**	**MENA**	**HIS**	**NAM**	**UNK**	**Total**	**AFA**	**API**	**EURO**	**MENA**	**HIS**	**NAM**	**UNK**	**Total**	**Highest Frequency**
DRB1*16:12	16:12	HLA03043		0	0	1	0	55	2	4	62					I			WD	I
DRB1*16:13N	16:13N	HLA03062		0	0	0	0	0	0	1	1									
DRB1*16:14	16:14	HLA03995		3	0	0	0	0	0	0	3									
DRB1*16:15	16:15	HLA04359		1	0	6	0	1	0	2	10			WD					WD	WD
DRB1*16:21N	16:21N	HLA09106		0	2	0	0	0	0	0	2									
DRB1*16:22	16:22	HLA09323		0	0	4	0	5	0	2	11					WD			WD	WD
DRB1*16:23	16:23	HLA09460		0	0	1	0	1	0	0	2									
DRB1*16:25	16:25	HLA12804		0	0	2	0	0	0	0	2									
DRB1*16:26	16:26	HLA12806		0	0	1	0	1	0	2	4									
DRB1*16:30	16:30	HLA13152		0	0	1	0	0	0	0	1									
DRB1*16:31	16:31	HLA13154		0	0	1	0	0	0	0	1									
DRB1*16:33	16:33	HLA13160		0	0	1	0	0	0	0	1									
DRB1*16:34	16:34	HLA13245		0	0	1	0	0	0	0	1									
DRB1*16:35	16:35	HLA13585		0	2	0	0	0	0	0	2									
**DRB1*16:38 total**	**16:38 total**			**0**	**4**	**0**	**0**	**0**	**0**	**0**	**4**									
DRB1*16:38	16:38			0	3	0	0	0	0	0	3									
DRB1*16:38:02	16:38:02	HLA17920		0	1	0	0	0	0	0	1									
DRB1*16:CODE	16:CODE			179	218	5598	64	672	49	224	7004	NA	NA	NA	NA	NA	NA	NA	NA	NA
**DRB1*Total**	**Total**			**389241**	**1318229**	**11938778**	**403373**	**700830**	**66984**	**1321251**	**16138686**									

C, common; I, intermediate; WD, well-documented; NA, not applicable

[a] All alleles observed in the current dataset are included in this table. Note that alleles are not in numerical order; alleles within a G group are clustered together. P group "two-field" total (e.g., written as "DRB1*01:01 total") and G group total summary rows are provided. The table does not list all alleles from IPD-IMGT version 3.31.0, if not present in the study dataset.

[b] Population groups include: AFA (African/African American), API (Asian/Pacific Islands), EURO (European/European descent), MENA (Middle East/North Coast of Africa), HIS (South or Central America/Hispanic/Latino), NAM (Native American populations) and UNK (unknown/not asked/multiple ancestries/other). Total is the overall population i.e., all groups combined.

Supplemental Table 11: HLA-DRB1 Allele Summary [a]				Allele Count by Population Group [b]								3.0.0 CIWD Category by Population Group [c]								
Allele	Genomic Typing	Allele ID	G group	AFA	API	EURO	MENA	HIS	NAM	UNK	Total	AFA	API	EURO	MENA	HIS	NAM	UNK	Total	Highest Frequency

[c] Allele frequency is calculated by dividing the number of times the “allele” of interest is observed in a population by the total number of copies of all the alleles at that particular genetic locus in the population (reported as the last row in this table and also in Table 2b). The total number of copies is calculated by multiplying the number of individuals times two for all loci except DRB3/4/5. For DRB3/4/5, the number of assignments was used as the total. The CIWD status is determined based on the allele frequency. Allele frequency data will be provided on the website of the next International HLA and Immunogenetics Workshop (https://www.ihiw18.org/). Highest frequency is the highest CIWD designation among all the individual groups.

[d] "CODE" is generically defined as a summary category of submitted HLA typing, including NMDP multiple allele codes, with ambiguities that are not within a single P or G group. "NEW" is a summary category for assignments of novel alleles that did not yet receive a nomenclature assignment. The CODE and NEW categories add to the total number of alleles but should not be assigned CIWD designations (labeled as NA, not applicable) as they do not represent a consistent allele designation (i.e., the NEW category may contain alleles with different DNA sequences that are unrelated to one another).

[e] DRB1*Total is the total number of allele assignments for the population group and is based on two times the number of individuals in the group. This number is also listed in Table 2b. It is not the sum of the column as alleles are not counted more than once. For example, when evaluating frequencies at the level of G resolution, individual alleles that make up the G group (e.g., A*80:01:01, A*80:01:01:01, A*80:01:01:02, A*80:01:01G) are not included in the count because these alleles are summed up in the total G designation (e.g., "A*80:01:01G total").
