# Supplementary material for: Common, intermediate and well‐documented HLA alleles in world populations: CIWD version 3.0.0
Source: HLA. 2020 Jan 31;95(6):516–31. doi: 10.1111/tan.13811 (PMC7317522; doi:10.1111/tan.13811)
Supplement: Supplementary file 12 — Table S12 HLA‐DRB3 primary data [file TAN-95-516-s012.pdf]

Supplemental Table 12: HLA-DRB3 Allele Summary [a]				Allele Count by Population Group [b]								3.0.0 CIWD Category by Population Group [c]							
Allele	Genomic Typing	Allele ID	G group	AFA	API	EURO	MENA	HIS	NAM	UNK	Total	AFA	API	EURO	MENA	HIS	NAM	UNK	Total
**DRB3*01:01 total**	**01:01 total**			**11527**	**6687**	**111562**	**1436**	**33353**	**2813**	**20489**	**187867**	**WD**	**WD**	**WD**	**WD**	**WD**	**WD**	**WD**	**WD**
DRB3*01:01	01:01			1960	726	22822	376	7296	485	3136	36801	WD	WD	WD	WD	WD	WD	WD	WD
**DRB3*01:01:02G total**	**01:01:02G total**			**9567**	**5941**	**88733**	**1060**	**26057**	**2328**	**17345**	**151031**	**WD**	**WD**	**WD**	**WD**	**WD**	**WD**	**WD**	**WD**
DRB3*01:01:02G	01:01:02G		01:01:02G	8023	4990	72273	865	21819	1947	14836	124753	WD	WD	WD	WD	WD	WD	WD	WD
DRB3*01:01:02	01:01:02		01:01:02G	1540	949	16284	194	4217	377	2493	26054	WD	WD	WD	WD	WD	WD	WD	WD
DRB3*01:01:02:01	01:01:02:01	HLA00887	01:01:02G	4	2	157	1	21	4	15	204			WD		WD		WD	WD
DRB3*01:16	01:16	HLA13234	01:01:02G	0	0	19	0	0	0	1	20			WD					WD
DRB3*01:01:03	01:01:03	HLA00888		0	0	6	0	0	0	0	6			WD					WD
DRB3*01:01:05	01:01:05	HLA03432		0	20	1	0	0	0	8	29		WD					WD	WD
DRB3*01:02	01:02	HLA00890		3	0	21	0	0	0	5	29			WD				WD	WD
DRB3*01:04	01:04	HLA00892		0	0	1	0	1	0	1	3								
DRB3*01:05	01:05	HLA00893		2	0	1	0	18	1	4	26					WD			WD
DRB3*01:06	01:06	HLA01092		1	0	1	0	6	0	0	8					WD			WD
DRB3*01:08	01:08	HLA01462		0	0	0	0	2	0	0	2								
DRB3*01:09	01:09	HLA01505		0	2	0	0	0	0	2	4								
DRB3*01:10	01:10	HLA01518		0	0	4	0	0	0	0	4								
DRB3*01:11	01:11	HLA01818		0	7	1	1	0	0	0	9		WD						WD
DRB3*01:12	01:12	HLA03433		0	3	0	0	0	0	0	3								
DRB3*01:13	01:13	HLA03434		0	8	0	0	0	0	3	11		WD						WD
DRB3*01:23	01:23	HLA15086		0	0	4	0	0	0	0	4								
DRB3*01:25	01:25	HLA15337		0	0	0	0	1	0	0	1								
DRB3*01:27	01:27	HLA15339		0	0	2	0	0	0	0	2								
DRB3*01:29	01:29	HLA15341		1	0	0	0	0	0	0	1								
DRB3*01:31	01:31	HLA15343		0	2	0	0	0	0	0	2								
DRB3*01:35	01:35	HLA15347		0	0	0	0	8	0	0	8					WD			WD
DRB3*01:CODE [d]	01:CODE			21412	11329	126920	1207	36108	4274	16428	217678	NA	NA	NA	NA	NA	NA	NA	NA
**DRB3*02:01 total**	**02:01 total**			**39**	**37**	**2374**	**233**	**611**	**40**	**375**	**3709**	**WD**	**WD**	**WD**	**WD**	**WD**	**WD**	**WD**	**WD**
**DRB3*02:01:01G total**	**02:01:01G total**			**39**	**37**	**2374**	**233**	**611**	**40**	**375**	**3709**	**WD**	**WD**	**WD**	**WD**	**WD**	**WD**	**WD**	**WD**
DRB3*02:01:01G	02:01:01G		02:01:01G	31	35	2042	207	555	35	346	3251	WD	WD	WD	WD	WD	WD	WD	WD
DRB3*02:24	02:24	HLA03574	02:01:01G	8	2	332	26	56	5	29	458	WD		WD	WD	WD	WD	WD	WD
**DRB3*02:02 total**	**02:02 total**			**22758**	**27592**	**142250**	**5251**	**42336**	**3735**	**29165**	**273087**	**WD**	**WD**	**WD**	**WD**	**WD**	**WD**	**WD**	**WD**

Supplemental Table 12: HLA-DRB3 Allele Summary [a]				Allele Count by Population Group [b]								3.0.0 CIWD Category by Population Group [c]							
**Allele**	**Genomic Typing**	**Allele ID**	**G group**	**AFA**	**API**	**EURO**	**MENA**	**HIS**	**NAM**	**UNK**	**Total**	**AFA**	**API**	**EURO**	**MENA**	**HIS**	**NAM**	**UNK**	**Total**
DRB3*02:02	02:02			2	1	0	1	3	0	1	8								WD
**DRB3*02:02:01G total**	**02:02:01G total**			**22755**	**27584**	**142235**	**5248**	**42333**	**3734**	**29162**	**273051**	**WD**	**WD**	**WD**	**WD**	**WD**	**WD**	**WD**	**WD**
DRB3*02:02:01G	02:02:01G		02:02:01G	19766	23756	121610	4407	37095	3265	25855	235754	WD	WD	WD	WD	WD	WD	WD	WD
DRB3*02:02:01	02:02:01		02:02:01G	2977	3820	20531	836	5213	467	3293	37137	WD	WD	WD	WD	WD	WD	WD	WD
DRB3*02:02:01:01	02:02:01:01	HLA00895	02:02:01G	10	2	15	1	3	0	8	39	WD		WD				WD	WD
DRB3*02:02:01:02	02:02:01:02	HLA06593	02:02:01G	2	6	78	4	22	2	5	119		WD	WD		WD		WD	WD
DRB3*02:29N	02:29N	HLA07562	02:02:01G	0	0	1	0	0	0	1	2								
DRB3*02:02:02	02:02:02	HLA01094		1	0	1	2	0	0	0	4								
DRB3*02:02:03	02:02:03	HLA01160		0	0	4	0	0	0	0	4								
DRB3*02:02:04	02:02:04	HLA01608		0	7	0	0	0	0	1	8		WD						WD
DRB3*02:02:06	02:02:06	HLA14222		0	0	11	0	0	1	2	14			WD					WD
DRB3*02:03	02:03	HLA00896		1	1	30	0	1	0	5	38			WD				WD	WD
DRB3*02:04	02:04	HLA00897		0	0	5	0	0	1	1	7			WD					WD
DRB3*02:05	02:05	HLA00898		0	0	7	0	13	1	3	24			WD		WD			WD
DRB3*02:06	02:06	HLA00899		0	1	8	1	0	0	3	13			WD					WD
DRB3*02:07	02:07	HLA00900		6	2	0	0	0	0	5	13	WD						WD	WD
DRB3*02:08	02:08	HLA00901		0	0	1	0	0	0	0	1								
DRB3*02:09	02:09	HLA01095		1	2	7	0	3	0	1	14			WD					WD
DRB3*02:10	02:10	HLA01157		34	7	71	2	42	3	18	177	WD	WD	WD		WD		WD	WD
DRB3*02:11	02:11	HLA01165		1	1	22	1	6	1	5	37			WD		WD		WD	WD
DRB3*02:12	02:12	HLA01185		1	0	31	0	2	0	2	36			WD					WD
DRB3*02:13	02:13	HLA01190		0	0	13	0	0	0	3	16			WD					WD
DRB3*02:15	02:15	HLA01542		0	0	4	0	0	0	0	4								
DRB3*02:16	02:16	HLA01560		0	0	7	0	0	0	1	8			WD					WD
DRB3*02:17	02:17	HLA01570		1	2	68	1	8	1	16	97			WD		WD		WD	WD
DRB3*02:18	02:18	HLA01769		0	0	2	0	0	0	0	2								
DRB3*02:19	02:19	HLA01870		0	1	5	0	0	0	0	6			WD					WD
DRB3*02:20	02:20	HLA02151		0	2	1	0	0	2	0	5								WD
DRB3*02:21	02:21	HLA02410		0	1	9	0	8	0	0	18			WD		WD			WD
**DRB3*02:22 total**	**02:22 total**			**1**	**2**	**7**	**0**	**0**	**0**	**1**	**11**			**WD**					**WD**
DRB3*02:22	02:22			1	1	6	0	0	0	0	8			WD					WD

Supplemental Table 12: HLA-DRB3 Allele Summary [a]				Allele Count by Population Group [b]								3.0.0 CIWD Category by Population Group [c]							
Allele	Genomic Typing	Allele ID	G group	AFA	API	EURO	MENA	HIS	NAM	UNK	Total	AFA	API	EURO	MENA	HIS	NAM	UNK	Total
DRB3*02:22:01	02:22:01	HLA02765		0	1	1	0	0	0	1	3								
DRB3*02:23	02:23	HLA03064		0	0	6	0	0	0	0	6			WD					WD
DRB3*02:25	02:25	HLA03989		0	4	17	0	0	0	1	22			WD					WD
DRB3*02:26	02:26	HLA06515		0	0	4	0	0	0	0	4								
DRB3*02:30	02:30	HLA14373		1	0	0	0	0	0	1	2								
**DRB3*02:31 total**	**02:31 total**			**0**	**1**	**0**	**0**	**0**	**0**	**0**	**1**								
DRB3*02:31:02	02:31:02	HLA15358		0	1	0	0	0	0	0	1								
DRB3*02:32	02:32	HLA14375		1	11	5	0	3	0	3	23		WD	WD					WD
DRB3*02:36	02:36	HLA14624		0	0	1	0	0	0	0	1								
DRB3*02:37	02:37	HLA14856		0	0	1	0	0	0	0	1								
DRB3*02:39	02:39	HLA15012		0	0	0	0	1	0	0	1								
DRB3*02:43	02:43	HLA15355		1	0	0	0	0	0	0	1								
DRB3*02:44	02:44	HLA15356		0	0	1	0	1	0	0	2								
DRB3*02:48	02:48	HLA15362		0	0	0	0	1	0	0	1								
DRB3*02:50	02:50	HLA15364		0	1	0	0	0	1	0	2								
DRB3*02:51	02:51	HLA15365		0	0	0	0	28	1	3	32					WD			WD
DRB3*02:CODE	02:CODE			51186	65945	190855	5141	58030	6026	28158	405341	NA	NA	NA	NA	NA	NA	NA	NA
**DRB3*03:01 total**	**03:01 total**			**8841**	**15229**	**36004**	**1044**	**11457**	**1241**	**9460**	**83276**	**WD**	**WD**	**WD**	**WD**	**WD**	**WD**	**WD**	**WD**
DRB3*03:01	03:01			6241	10315	23778	677	7986	967	6577	56541	WD	WD	WD	WD	WD	WD	WD	WD
**DRB3*03:01:01G total**	**03:01:01G total**			**2600**	**4908**	**12226**	**367**	**3471**	**274**	**2881**	**26727**	**WD**	**WD**	**WD**	**WD**	**WD**	**WD**	**WD**	**WD**
DRB3*03:01:01G	03:01:01G		03:01:01G	1409	2817	6781	176	2026	148	1727	15084	WD	WD	WD	WD	WD	WD	WD	WD
DRB3*03:01:01	03:01:01	HLA00902	03:01:01G	1176	640	5428	171	1410	124	927	9876	WD	WD	WD	WD	WD	WD	WD	WD
DRB3*03:01:03	03:01:03	HLA03573	03:01:01G	15	1451	17	20	35	2	227	1767	WD	WD	WD	WD	WD		WD	WD
DRB3*03:01:05	03:01:05	HLA15369		0	2	0	0	0	0	1	3								
DRB3*03:01:02	03:01:02	HLA01233		0	4	0	0	0	0	1	5								WD
DRB3*03:02	03:02	HLA00903		0	0	8	0	0	0	0	8			WD					WD
DRB3*03:03	03:03	HLA00904		0	2	1	0	2	0	0	5								WD
DRB3*03:04	03:04	HLA14575		1	0	0	0	0	0	0	1								
DRB3*03:05	03:05	HLA14657		0	0	1	0	0	0	0	1								
DRB3*03:06	03:06	HLA14658		0	0	1	0	1	0	0	2								
DRB3*03:08	03:08	HLA15352		0	0	0	0	0	0	1	1								

Supplemental Table 12: HLA-DRB3 Allele Summary [a]				Allele Count by Population Group [b]								3.0.0 CIWD Category by Population Group [c]							
Allele	Genomic Typing	Allele ID	G group	AFA	API	EURO	MENA	HIS	NAM	UNK	Total	AFA	API	EURO	MENA	HIS	NAM	UNK	Total
DRB3*03:09	03:09	HLA15354		0	2	0	0	0	0	0	2								
DRB3*03:11	03:11	HLA15368		0	1	0	0	0	0	0	1								
DRB3*03:CODE	03:CODE			3	6	7	0	2	1	1	20	NA	NA	NA	NA	NA	NA	NA	NA
**DRB3*Total [e]**	**Total**			**115823**	**126891**	**610352**	**14318**	**182053**	**18142**	**104165**	**1171744**								

WD, well-documented; NA, not applicable

[a] All alleles observed in the current dataset are included in this table. Note that alleles are not in numerical order; alleles within a G group are clustered together. P group "two-field" total (e.g., written as "DRB3*01:01 total") and G group total summary rows are provided. The table does not list all alleles from IPD-IMGT version 3.31.0, if not present in the study dataset.

[b] Population groups include: AFA (African/African American), API (Asian/Pacific Islands), EURO (European/European descent), MENA (Middle East/North Coast of Africa), HIS (South or Central America/Hispanic/Latino), NAM (Native American populations) and UNK (unknown/not asked/multiple ancestries/other). Total is the overall population i.e., all groups combined.

[c] Due to high submission of typing with ambiguity and uncertain denominator, only well-documented categories are assigned for this locus. Allele frequency is calculated by dividing the number of times the “allele” of interest is observed in a population by the total number of copies of all the alleles at that particular genetic locus in the population (reported as the last row in this table and also in Table 2b). The total number of copies is calculated by multiplying the number of individuals times two for all loci except DRB3/4/5. For DRB3/4/5, the number of assignments was used as the total. The CIWD status is determined based on the allele frequency. Allele frequency data will be provided on the website of the next International HLA and Immunogenetics Workshop (https://www.ihiw18.org/).

[d] "CODE" is generically defined as a summary category of submitted HLA typing, including NMDP multiple allele codes, with ambiguities that are not within a single P or G group. "NEW" is a summary category for assignments of novel alleles that did not yet receive a nomenclature assignment. The CODE and NEW categories add to the total number of alleles but should not be assigned CIWD designations (labeled as NA, not applicable) as they do not represent a consistent allele designation (i.e., the NEW category may contain alleles with different DNA sequences that are unrelated to one another).

[e] DRB3*Total is the total number of allele assignments for the population group. It is not the sum of the column as alleles are not counted more than once. For example, when evaluating frequencies at the level of G resolution, individual alleles that make up the G group (e.g., A*80:01:01, A*80:01:01:01, A*80:01:01:02, A*80:01:01G) are not included in the count because these alleles are summed up in the total G designation (e.g., "A*80:01:01G total").
