# Supplementary material for: Common, intermediate and well‐documented HLA alleles in world populations: CIWD version 3.0.0
Source: HLA. 2020 Jan 31;95(6):516–31. doi: 10.1111/tan.13811 (PMC7317522; doi:10.1111/tan.13811)
Supplement: Supplementary file 13 — Table S13 HLA‐DRB4 primary data [file TAN-95-516-s013.pdf]

Supplemental Table 13: HLA-DRB4 Allele Summary [a]				Allele Count by Population Group [b]								3.0.0 CIWD Category by Population Group [c]							
Allele	Genomic Typing	Allele ID	G group	AFA	API	EURO	MENA	HIS	NAM	UNK	Total	AFA	API	EURO	MENA	HIS	NAM	UNK	Total
**DRB4*01:01 total**	**01:01 total**			**15841**	**36270**	**210683**	**3932**	**75975**	**6399**	**42566**	**391666**	**WD**	**WD**	**WD**	**WD**	**WD**	**WD**	**WD**	**WD**
DRB4*01:01	01:01			1	0	1	0	0	0	0	2								
**DRB4*01:01:01G total**	**01:01:01G total**			**16101**	**37923**	**221285**	**3921**	**76639**	**6491**	**44229**	**406589**	**WD**	**WD**	**WD**	**WD**	**WD**	**WD**	**WD**	**WD**
DRB4*01:01:01G	01:01:01G		01:01:01G	14455	31733	188352	3233	69019	5746	38899	351437	WD	WD	WD	WD	WD	WD	WD	WD
DRB4*01:01:01	01:01:01		01:01:01G	0	9	1	4	0	0	9	23		WD					WD	WD
DRB4*01:01:01:01	01:01:01:01	HLA00905	01:01:01G	179	17	1298	6	296	37	191	2024	WD	WD	WD	WD	WD	WD	WD	WD
**DRB4*01:03N total**	**01:03N total**			**336**	**2571**	**12288**	**1763**	**106**	**167**	**2112**	**19343**	**WD**	**WD**	**WD**	**WD**	**WD**	**WD**	**WD**	**WD**
DRB4*01:03N	01:03N		01:01:01G	28	285	745	10	135	7	113	1323	WD	WD	WD	WD	WD	WD	WD	WD
DRB4*01:03:01	01:03:01		01:01:01G	1131	3576	19344	573	5559	541	3015	33739	WD	WD	WD	WD	WD	WD	WD	WD
DRB4*01:03:01N	01:03:01N		01:01:01G	0	5	0	0	1	0	3	9		WD						WD
DRB4*01:03:01:02N	01:03:01:02N	HLA00909	01:01:01G	308	2281	11543	95	1628	160	1996	18011	WD	WD	WD	WD	WD	WD	WD	WD
DRB4*01:03:02	01:03:02	HLA00910	01:01:01G	0	17	0	0	1	0	3	21		WD						WD
DRB4*01:06	01:06	HLA01559	01:01:01G	0	0	2	0	0	0	0	2								
**DRB4*01:03 total**	**01:03 total**			**1206**	**4511**	**21029**	**689**	**6660**	**616**	**3467**	**38178**	**WD**	**WD**	**WD**	**WD**	**WD**	**WD**	**WD**	**WD**
DRB4*01:03	01:03			37	508	1157	42	666	56	317	2783	WD	WD	WD	WD	WD	WD	WD	WD
DRB4*01:03:03	01:03:03	HLA01225		38	409	516	72	424	18	130	1607	WD	WD	WD	WD	WD	WD	WD	WD
DRB4*01:03:04	01:03:04	HLA01594		0	1	12	2	10	1	2	28			WD		WD			WD
DRB4*01:02	01:02	HLA00907		1	294	273	8	22	16	107	721		WD	WD	WD	WD	WD	WD	WD
DRB4*01:04	01:04	HLA00911		0	6	0	0	0	0	1	7		WD						WD
DRB4*01:05	01:05	HLA00912		1	4	55	0	9	0	11	80			WD		WD		WD	WD
**DRB4*01:07 total**	**01:07 total**			**0**	**0**	**5**	**0**	**1**	**0**	**1**	**7**			**WD**					**WD**
DRB4*01:07	01:07			0	0	1	0	0	0	1	2								
DRB4*01:07:01	01:07:01	HLA01891		0	0	2	0	1	0	0	3								
DRB4*01:07:02	01:07:02	HLA15019		0	0	2	0	0	0	0	2								
DRB4*01:11	01:11	HLA14659		0	0	1	0	0	0	1	2								
DRB4*01:14	01:14	HLA14662		0	0	2	0	0	0	0	2								
DRB4*01:16N	01:16N	HLA14664		0	0	1	1	4	2	0	8								WD
DRB4*01:17	01:17	HLA14665		1	0	16	0	2	0	3	22			WD					WD
DRB4*01:22	01:22	HLA15015		0	0	0	0	1	0	0	1								
DRB4*01:34	01:34	HLA15381		0	0	1	0	0	0	0	1								
DRB4*01:36	01:36	HLA15383		0	0	1	0	0	0	0	1								

Supplemental Table 13: HLA-DRB4 Allele Summary [a]				Allele Count by Population Group [b]								3.0.0 CIWD Category by Population Group [c]							
Allele	Genomic Typing	Allele ID	G group	AFA	API	EURO	MENA	HIS	NAM	UNK	Total	AFA	API	EURO	MENA	HIS	NAM	UNK	Total
DRB4*01:37	01:37	HLA15429		0	0	1	0	0	0	0	1								
DRB4*01:44	01:44	HLA15715		0	0	0	2	0	0	0	2								
DRB4*01:CODE [d]	01:CODE			28069	60145	241670	3357	78999	8821	33490	454551	NA	NA	NA	NA	NA	NA	NA	NA
DRB4*02:01N	02:01N	HLA00913		1	0	31	0	1	1	3	37			WD					WD
DRB4*03:01N	03:01N	HLA00914		0	0	14	0	0	0	2	16			WD					WD
**DRB4*Total**	**Total**			**44250**	**99290**	**465042**	**7405**	**156778**	**15406**	**78297**	**866468**								

WD, well-documented; NA, not applicable

[a] All alleles observed in the current dataset are included in this table. Note that alleles are not in numerical order; alleles within a G group are clustered together. P group "two-field" total (e.g., written as "DRB4*01:01 total") and G group total summary rows are provided. The table does not list all alleles from IPD-IMGT version 3.31.0, if not present in the study dataset.

[b] Population groups include: AFA (African/African American), API (Asian/Pacific Islands), EURO (European/European descent), MENA (Middle East/North Coast of Africa), HIS (South or Central America/Hispanic/Latino), NAM (Native American populations) and UNK (unknown/not asked/multiple ancestries/other). Total is the overall population i.e., all groups combined.

[c] Due to high submission of typing with ambiguity and uncertain denominator, only well-documented categories are assigned for this locus. Allele frequency is calculated by dividing the number of times the “allele” of interest is observed in a population by the total number of copies of all the alleles at that particular genetic locus in the population (reported as the last row in this table and also in Table 2b). The total number of copies is calculated by multiplying the number of individuals times two for all loci except DRB3/4/5. For DRB3/4/5, the number of assignments was used as the total. The CIWD status is determined based on the allele frequency. Allele frequency data will be provided on the website of the next International HLA and Immunogenetics Workshop (https://www.ihiw18.org/).

[d] "CODE" is generically defined as a summary category of submitted HLA typing, including NMDP multiple allele codes, with ambiguities that are not within a single P or G group. "NEW" is a summary category for assignments of novel alleles that did not yet receive a nomenclature assignment. The CODE and NEW categories add to the total number of alleles but should not be assigned CIWD designations (labeled as NA, not applicable) as they do not represent a consistent allele designation (i.e., the NEW category may contain alleles with different DNA sequences that are unrelated to one another).

[e] DRB4*Total is the total number of allele assignments for the population group. It is not the sum of the column as alleles are not counted more than once. For example, when evaluating frequencies at the level of G resolution, individual alleles that make up the G group (e.g., A*80:01:01, A*80:01:01:01, A*80:01:01:02, A*80:01:01G) are not included in the count because these alleles are summed up in the total G designation (e.g., "A*80:01:01G total").
