# Supplementary material for: Common, intermediate and well‐documented HLA alleles in world populations: CIWD version 3.0.0
Source: HLA. 2020 Jan 31;95(6):516–31. doi: 10.1111/tan.13811 (PMC7317522; doi:10.1111/tan.13811)
Supplement: Supplementary file 14 — Table S14 HLA‐DRB5 primary data [file TAN-95-516-s014.pdf]

Supplemental Table 14: HLA-DRB5 Allele Summary [a]				Allele Count by Population Group [b]								3.0.0 CIWD Category by Population Group [c]							
Allele	Genomic Typing	Allele ID	G group	AFA	API	EURO	MENA	HIS	NAM	UNK	Total	AFA	API	EURO	MENA	HIS	NAM	UNK	Total
**DRB5*01:01 total**	**01:01 total**			**11894**	**16874**	**99254**	**962**	**18472**	**1640**	**18910**	**168006**	**WD**	**WD**	**WD**	**WD**	**WD**	**WD**	**WD**	**WD**
DRB5*01:01	01:01			2104	1847	21727	250	4806	311	3649	34694	WD	WD	WD	WD	WD	WD	WD	WD
**DRB5*01:01:01G total**	**01:01:01G total**			**9788**	**15027**	**77491**	**712**	**13664**	**1328**	**15252**	**133262**	**WD**	**WD**	**WD**	**WD**	**WD**	**WD**	**WD**	**WD**
DRB5*01:01:01G	01:01:01G		01:01:01G	3740	5593	32412	204	5141	554	5198	52842	WD	WD	WD	WD	WD	WD	WD	WD
DRB5*01:01:01	01:01:01	HLA00915	01:01:01G	6048	9434	45079	508	8523	774	10054	80420	WD	WD	WD	WD	WD	WD	WD	WD
DRB5*01:01:02	01:01:02	HLA00916		2	0	36	0	2	1	9	50			WD				WD	WD
**DRB5*01:02 total**	**01:02 total**			**227**	**9642**	**6162**	**805**	**2962**	**256**	**2415**	**22469**	**WD**	**WD**	**WD**	**WD**	**WD**	**WD**	**WD**	**WD**
**DRB5*01:02:01G total**	**01:02:01G total**			**232**	**9857**	**6165**	**805**	**2963**	**256**	**2457**	**22735**	**WD**	**WD**	**WD**	**WD**	**WD**	**WD**	**WD**	**WD**
DRB5*01:02	01:02	HLA00917	01:02:01G	20	1109	827	111	317	33	225	2642	WD	WD	WD	WD	WD	WD	WD	WD
DRB5*01:02:01G	01:02:01G		01:02:01G	207	8533	5335	694	2645	223	2190	19827	WD	WD	WD	WD	WD	WD	WD	WD
DRB5*01:08N	01:08N	HLA00923	01:02:01G	5	215	3	0	1	0	42	266	WD	WD					WD	WD
DRB5*01:03	01:03	HLA00918		12	348	2	2	3	2	49	418	WD	WD					WD	WD
DRB5*01:04	01:04	HLA00919		2	0	0	0	3	0	0	5								WD
DRB5*01:05	01:05	HLA00920		0	2	23	4	1	0	3	33			WD					WD
DRB5*01:06	01:06	HLA00921		0	0	4	0	0	0	1	5								WD
DRB5*01:07	01:07	HLA00922		0	0	4	0	0	0	0	4								
DRB5*01:09	01:09	HLA00924		0	0	3	0	0	1	1	5								WD
DRB5*01:10N	01:10N	HLA01012		3	38	38	7	8	1	9	104		WD	WD	WD	WD		WD	WD
DRB5*01:11	01:11	HLA01638		0	6	2	0	0	0	2	10		WD						WD
DRB5*01:12	01:12	HLA01634		0	0	1	0	0	0	1	2								
DRB5*01:13	01:13	HLA01871		0	0	1	0	0	0	0	1								
DRB5*01:14	01:14	HLA04132		1	0	0	0	0	0	0	1								
DRB5*01:15	01:15	HLA11771		0	6	0	0	0	0	1	7		WD						WD
DRB5*01:17	01:17	HLA14576		0	0	1	0	0	0	0	1								
DRB5*01:18	01:18	HLA14666		0	0	1	0	0	0	0	1								
DRB5*01:20	01:20	HLA15021		0	0	4	0	1	0	1	6								WD
DRB5*01:21	01:21	HLA15023		0	0	1	0	0	0	0	1								
DRB5*01:22	01:22	HLA15024		0	1	0	0	0	0	0	1								
DRB5*01:29	01:29	HLA16593		0	0	0	0	0	0	1	1								
DRB5*01:CODE [d]	01:CODE			24496	42624	134399	1918	31688	3816	19118	258059	NA	NA	NA	NA	NA	NA	NA	NA
**DRB5*02:02 total**	**02:02 total**			**1416**	**936**	**14144**	**411**	**8000**	**638**	**2982**	**28527**	**WD**	**WD**	**WD**	**WD**	**WD**	**WD**	**WD**	**WD**

Supplemental Table 14: HLA-DRB5 Allele Summary [a]				Allele Count by Population Group [b]								3.0.0 CIWD Category by Population Group [c]							
**Allele**	**Genomic Typing**	**Allele ID**	**G group**	**AFA**	**API**	**EURO**	**MENA**	**HIS**	**NAM**	**UNK**	**Total**	**AFA**	**API**	**EURO**	**MENA**	**HIS**	**NAM**	**UNK**	**Total**
DRB5*02:02	02:02			46	160	1991	81	917	71	258	3524	WD	WD	WD	WD	WD	WD	WD	WD
**DRB5*02:02:01G total**	**02:02:01G total**			**1370**	**776**	**12153**	**330**	**7083**	**567**	**2724**	**25003**	**WD**	**WD**	**WD**	**WD**	**WD**	**WD**	**WD**	**WD**
DRB5*02:02:01G	02:02:01G		02:02:01G	1370	776	12133	323	7079	567	2721	24969	WD	WD	WD	WD	WD	WD	WD	WD
DRB5*02:02:01	02:02:01	HLA00926	02:02:01G	0	0	0	7	0	0	1	8				WD				WD
DRB5*02:06	02:06	HLA08085	02:02:01G	0	0	20	0	4	0	2	26			WD					WD
DRB5*02:03	02:03	HLA00927		0	274	10	0	16	1	38	339		WD	WD		WD		WD	WD
DRB5*02:04	02:04	HLA00928		0	0	5	0	0	0	0	5			WD					WD
DRB5*02:08	02:08	HLA15022		0	0	0	0	1	0	0	1								
DRB5*02:12	02:12	HLA15440		0	0	1	0	0	0	0	1								
DRB5*02:13	02:13	HLA15441		1	0	0	0	2	1	0	4								
DRB5*02:CODE	02:CODE			4	4	5	0	13	3	2	31	NA	NA	NA	NA	NA	NA	NA	NA
**DRB5*Total [e]**	**Total**			**38061**	**70970**	**254068**	**4109**	**61171**	**6359**	**43576**	**478314**								

WD, well-documented; NA, not applicable

[a] All alleles observed in the current dataset are included in this table. Note that alleles are not in numerical order; alleles within a G group are clustered together. P group "two-field" total (e.g., written as "DRB5*01:01 total") and G group total summary rows are provided. The table does not list all alleles from IPD-IMGT version 3.31.0, if not present in the study dataset.

[b] Population groups include: AFA (African/African American), API (Asian/Pacific Islands), EURO (European/European descent), MENA (Middle East/North Coast of Africa), HIS (South or Central America/Hispanic/Latino), NAM (Native American populations) and UNK (unknown/not asked/multiple ancestries/other). Total is the overall population i.e., all groups combined.

[c] Due to high submission of typing with ambiguity and uncertain denominator, only well-documented categories are assigned for this locus. Allele frequency is calculated by dividing the number of times the “allele” of interest is observed in a population by the total number of copies of all the alleles at that particular genetic locus in the population (reported as the last row in this table and also in Table 2b). The total number of copies is calculated by multiplying the number of individuals times two for all loci except DRB3/4/5. For DRB3/4/5, the number of assignments was used as the total. The CIWD status is determined based on the allele frequency. Allele frequency data will be provided on the website of the next International HLA and Immunogenetics Workshop (https://www.ihiw18.org/).

Supplemental Table 14: HLA-DRB5 Allele Summary [a]				Allele Count by Population Group [b]								3.0.0 CIWD Category by Population Group [c]							
Allele	Genomic Typing	Allele ID	G group	AFA	API	EURO	MENA	HIS	NAM	UNK	Total	AFA	API	EURO	MENA	HIS	NAM	UNK	Total

[d] "CODE" is generically defined as a summary category of submitted HLA typing, including NMDP multiple allele codes, with ambiguities that are not within a single P or G group. "NEW" is a summary category for assignments of novel alleles that did not yet receive a nomenclature assignment. The CODE and NEW categories add to the total number of alleles but should not be assigned CIWD designations (labeled as NA, not applicable) as they do not represent a consistent allele designation (i.e., the NEW category may contain alleles with different DNA sequences that are unrelated to one another).

[e] DRB3*Total is the total number of allele assignments for the population group. It is not the sum of the column as alleles are not counted more than once. For example, when evaluating frequencies at the level of G resolution, individual alleles that make up the G group (e.g., A*80:01:01, A*80:01:01:01, A*80:01:01:02, A*80:01:01G) are not included in the count because these alleles are summed up in the total G designation (e.g., "A*80:01:01G total").
