# Supplementary material for: Common, intermediate and well‐documented HLA alleles in world populations: CIWD version 3.0.0
Source: HLA. 2020 Jan 31;95(6):516–31. doi: 10.1111/tan.13811 (PMC7317522; doi:10.1111/tan.13811)
Supplement: Supplementary file 15 — Table S15 HLA‐DQB1 primary data [file TAN-95-516-s015.pdf]

Supplemental Table 15: HLA-DQB1 Allele Summary [a]				Allele Count by Population Group [b]								3.0.0 CIWD Category by Population Group [c]								
**Allele**	**Genomic Typing**	**Allele ID**	**G group**	**AFA**	**API**	**EURO**	**MENA**	**HIS**	**NAM**	**UNK**	**Total**	**AFA**	**API**	**EURO**	**MENA**	**HIS**	**NAM**	**UNK**	**Total**	**Highest Frequency**
**DQB1*02:01 total**	**02:01 total**			**77516**	**187155**	**2372591**	**79436**	**111972**	**11248**	**233396**	**3073314**	**C**	**C**	**C**	**C**	**C**	**C**	**C**	**C**	**C**
DQB1*02:01	02:01			33	115	16127	865	30	0	431	17601	WD	I	C	C	I		C	C	C
DQB1*02:01P	02:01P			0	0	23	5	0	0	11	39			WD	WD			WD	WD	WD
**DQB1*02:01:01G total**	**02:01:01G total**			**77356**	**186678**	**2312148**	**75784**	**111784**	**11248**	**232084**	**3007082**	**C**	**C**	**C**	**C**	**C**	**C**	**C**	**C**	**C**
DQB1*02:01:01G	02:01:01G		02:01:01G	45324	139662	761469	39924	67418	7168	176780	1237745	C	C	C	C	C	C	C	C	C
DQB1*02:01:01	02:01:01	HLA00622	02:01:01G	10988	23316	884329	20311	18594	1656	27862	987056	C	C	C	C	C	C	C	C	C
DQB1*02:01:08	02:01:08	HLA08917	02:01:01G	0	1	33	0	1	0	0	35			WD					WD	WD
**DQB1*02:02 total**	**02:02 total**			**21157**	**24054**	**709845**	**18318**	**25919**	**2424**	**28263**	**829980**	**C**	**C**	**C**	**C**	**C**	**C**	**C**	**C**	**C**
DQB1*02:02	02:02			118	356	44040	2777	152	0	833	48276	C	C	C	C	C		C	C	C
DQB1*02:02:01	02:02:01		02:01:01G	18740	19662	560702	12685	24579	2326	24321	663015	C	C	C	C	C	C	C	C	C
DQB1*02:02:01:01	02:02:01:01	HLA00623	02:01:01G	1969	4027	105029	2825	1044	75	3023	117992	C	C	C	C	C	C	C	C	C
DQB1*02:02:01:02	02:02:01:02	HLA13985	02:01:01G	327	2	69	31	144	23	85	681	C		WD	WD	C	C	I	I	C
DQB1*02:02:02	02:02:02	HLA09092	02:01:01G	0	7	3	0	0	0	0	10		WD						WD	WD
DQB1*02:02:03	02:02:03	HLA15617	02:01:01G	3	0	2	0	0	0	1	6								WD	WD
DQB1*02:04	02:04	HLA02136	02:01:01G	3	0	84	0	0	0	8	95			WD				WD	WD	WD
DQB1*02:06	02:06	HLA06141	02:01:01G	0	0	4	0	0	0	0	4									
DQB1*02:10	02:10	HLA08924	02:01:01G	0	0	373	4	4	0	4	385			I					I	I
DQB1*02:48	02:48	HLA12476	02:01:01G	0	0	5	0	0	0	0	5			WD					WD	WD
DQB1*02:59	02:59	HLA13395	02:01:01G	0	0	40	1	0	0	0	41			WD					WD	WD
DQB1*02:64	02:64	HLA13936	02:01:01G	1	0	0	0	0	0	0	1									
DQB1*02:80	02:80	HLA16129	02:01:01G	0	1	0	0	0	0	0	1									
DQB1*02:81	02:81	HLA16137	02:01:01G	0	0	3	0	0	0	0	3									
DQB1*02:82	02:82	HLA16139	02:01:01G	0	0	1	0	0	0	0	1									
DQB1*02:89	02:89	HLA16643	02:01:01G	1	0	0	3	0	0	0	4									
DQB1*02:98	02:98	HLA17423	02:01:01G	0	0	1	0	0	0	0	1									
DQB1*02:99	02:99	HLA17424	02:01:01G	0	0	1	0	0	0	0	1									
DQB1*02:01:02	02:01:02	HLA01934		0	0	1	0	0	0	0	1									
DQB1*02:01:03	02:01:03	HLA05758		0	0	1	0	0	0	0	1									
DQB1*02:01:04	02:01:04	HLA05923		0	0	6	0	0	0	0	6			WD					WD	WD
DQB1*02:01:05	02:01:05	HLA08310		0	0	2	3	0	0	0	5								WD	WD
DQB1*02:01:06	02:01:06	HLA08777		0	0	5	1	0	0	0	6			WD					WD	WD

Supplemental Table 15: HLA-DQB1 Allele Summary [a]				Allele Count by Population Group [b]								3.0.0 CIWD Category by Population Group [c]								
Allele	Genomic Typing	Allele ID	G group	AFA	API	EURO	MENA	HIS	NAM	UNK	Total	AFA	API	EURO	MENA	HIS	NAM	UNK	Total	Highest Frequency
DQB1*02:01:07	02:01:07	HLA08915		0	6	0	0	0	0	1	7		WD						WD	WD
DQB1*02:01:09	02:01:09	HLA09247		0	0	1	0	0	0	0	1									
DQB1*02:01:10	02:01:10	HLA09252		5	0	221	1	6	0	27	260	WD		I		WD		I	I	I
DQB1*02:01:11	02:01:11	HLA09254		3	0	0	0	0	0	0	3									
DQB1*02:01:12	02:01:12	HLA09729		0	0	2	0	0	0	0	2									
DQB1*02:01:14	02:01:14	HLA10278		0	0	4	0	0	0	2	6								WD	WD
DQB1*02:01:15	02:01:15	HLA11674		0	0	0	0	0	0	1	1									
DQB1*02:01:19	02:01:19	HLA12354		1	0	4	0	0	0	0	5								WD	WD
DQB1*02:01:20	02:01:20	HLA13124		0	0	5	0	0	0	5	10			WD				WD	WD	WD
DQB1*02:01:23	02:01:23	HLA13141		0	0	1	0	0	0	1	2									
DQB1*02:03	02:03	HLA00624		930	2	68	3	62	14	209	1288	C		WD		I	C	C	I	C
DQB1*02:05	02:05	HLA02890		0	0	58	0	0	1	7	66			WD				WD	WD	WD
**DQB1*02:07 total**	**02:07 total**			**0**	**320**	**5**	**0**	**0**	**4**	**8**	**337**		**C**	**WD**				**WD**	**I**	**C**
DQB1*02:07	02:07			0	84	2	0	0	4	3	93		I						WD	I
DQB1*02:07:01	02:07:01	HLA08083		0	236	3	0	0	0	5	244		C					WD	I	C
DQB1*02:12	02:12	HLA09127		0	2	36	14	0	0	6	58			WD	WD			WD	WD	WD
DQB1*02:13	02:13	HLA09243		0	0	2	0	0	0	0	2									
**DQB1*02:14 total**	**02:14 total**			**4**	**1**	**197**	**1**	**6**	**1**	**23**	**233**			**I**		**WD**		**I**	**I**	**I**
DQB1*02:14	02:14			1	0	87	1	2	1	12	104			WD				WD	WD	WD
DQB1*02:14:01	02:14:01	HLA09245		3	1	105	0	4	0	10	123			WD				WD	WD	WD
DQB1*02:14:02	02:14:02	HLA12353		0	0	5	0	0	0	1	6			WD					WD	WD
DQB1*02:16	02:16	HLA09248		0	0	5	0	0	0	3	8			WD					WD	WD
DQB1*02:17	02:17	HLA09249		0	1	19	0	0	0	0	20			WD					WD	WD
DQB1*02:18N	02:18N	HLA09250		0	1	19	0	0	0	0	20			WD					WD	WD
DQB1*02:19	02:19	HLA09251		0	0	5	6	0	0	15	26			WD	WD			I	WD	I
DQB1*02:20N	02:20N	HLA09253		0	0	60	0	0	0	2	62			WD					WD	WD
DQB1*02:21	02:21	HLA09255		0	0	10	0	0	0	0	10			WD					WD	WD
DQB1*02:22	02:22	HLA09256		0	0	3	0	0	0	0	3									
DQB1*02:23	02:23	HLA09257		0	0	20	0	0	0	0	20			WD					WD	WD
DQB1*02:24	02:24	HLA09258		0	1	9	0	0	0	0	10			WD					WD	WD
DQB1*02:26	02:26	HLA09260		3	0	99	0	2	0	24	128			WD				I	WD	I

Supplemental Table 15: HLA-DQB1 Allele Summary [a]				Allele Count by Population Group [b]								3.0.0 CIWD Category by Population Group [c]								
Allele	Genomic Typing	Allele ID	G group	AFA	API	EURO	MENA	HIS	NAM	UNK	Total	AFA	API	EURO	MENA	HIS	NAM	UNK	Total	Highest Frequency
DQB1*02:27	02:27	HLA09344		0	0	25	0	0	0	0	25			WD					WD	WD
DQB1*02:28	02:28	HLA09355		0	0	9	0	1	0	2	12			WD					WD	WD
DQB1*02:29	02:29	HLA09357		0	0	54	0	1	0	9	64			WD				WD	WD	WD
DQB1*02:30	02:30	HLA09641		0	0	17	0	0	0	0	17			WD					WD	WD
DQB1*02:31	02:31	HLA09642		0	0	3	0	0	0	0	3									
DQB1*02:33	02:33	HLA09742		0	0	5	0	0	0	0	5			WD					WD	WD
DQB1*02:34	02:34	HLA10279		0	0	4	0	0	0	0	4									
DQB1*02:37	02:37	HLA11133		0	0	1	0	0	0	0	1									
DQB1*02:40	02:40	HLA11675		0	0	1	0	0	0	0	1									
DQB1*02:41	02:41	HLA11883		0	0	1	0	0	0	0	1									
DQB1*02:42	02:42	HLA12208		0	1	0	0	0	0	1	2									
DQB1*02:45	02:45	HLA12523		0	0	0	0	0	0	1	1									
DQB1*02:46	02:46	HLA12524		0	0	6	0	0	0	0	6			WD					WD	WD
DQB1*02:51	02:51	HLA12798		0	3	0	0	0	0	0	3									
DQB1*02:53Q	02:53Q	HLA12878		0	0	165	0	0	0	0	165			I					I	I
DQB1*02:54	02:54	HLA13125		0	0	1	1	1	0	0	3									
DQB1*02:56	02:56	HLA13129		1	0	0	0	0	0	0	1									
DQB1*02:58N	02:58N	HLA13205		0	0	2	0	0	0	0	2									
DQB1*02:61	02:61	HLA13681		0	0	1	0	0	0	0	1									
DQB1*02:62	02:62	HLA13918		0	0	1	0	0	0	0	1									
DQB1*02:63	02:63	HLA13925		0	0	9	0	0	0	0	9			WD					WD	WD
DQB1*02:65	02:65	HLA14230		0	0	0	0	0	0	1	1									
DQB1*02:74	02:74	HLA15322		0	0	0	1	0	0	0	1									
DQB1*02:78	02:78	HLA15993		0	1	0	0	0	0	0	1									
DQB1*02:84	02:84	HLA16187		0	0	1	0	0	0	0	1									
DQB1*02:85	02:85	HLA16322		0	0	1	0	0	0	0	1									
DQB1*02:95	02:95	HLA17166		0	0	1	0	0	0	0	1									
DQB1*02:CODE [d]	02:CODE			2974	2011	37591	212	3808	232	5636	52464	NA	NA	NA	NA	NA	NA	NA	NA	NA
**DQB1*03:01 total**	**03:01 total**			**64549**	**190390**	**2327114**	**98892**	**131200**	**12881**	**245316**	**3070342**	**C**	**C**	**C**	**C**	**C**	**C**	**C**	**C**	**C**
DQB1*03:01	03:01			84	524	64809	6324	178	0	1677	73596	C	C	C	C	C		C	C	C
DQB1*03:01P	03:01P			0	1	808	4	4	0	26	843			I				I	I	I

Supplemental Table 15: HLA-DQB1 Allele Summary [a]				Allele Count by Population Group [b]								3.0.0 CIWD Category by Population Group [c]								
Allele	Genomic Typing	Allele ID	G group	AFA	API	EURO	MENA	HIS	NAM	UNK	Total	AFA	API	EURO	MENA	HIS	NAM	UNK	Total	Highest Frequency
**DQB1*03:01:01G total**	**03:01:01G total**			**56001**	**189759**	**2249467**	**92227**	**128466**	**12536**	**240871**	**2969327**	**C**	**C**	**C**	**C**	**C**	**C**	**C**	**C**	**C**
DQB1*03:01:01G	03:01:01G		03:01:01G	37349	137508	721566	32101	78398	8252	189731	1204905	C	C	C	C	C	C	C	C	C
DQB1*03:01:01	03:01:01		03:01:01G	11574	51376	1512406	59737	44743	3707	47681	1731224	C	C	C	C	C	C	C	C	C
DQB1*03:01:01:01	03:01:01:01	HLA00625	03:01:01G	258	775	7288	36	2998	264	1201	12820	C	C	C	WD	C	C	C	C	C
DQB1*03:01:01:02	03:01:01:02	HLA06613	03:01:01G	0	0	30	0	0	0	0	30			WD					WD	WD
DQB1*03:01:01:03	03:01:01:03	HLA06616	03:01:01G	0	1	306	0	0	0	7	314			I				WD	I	I
DQB1*03:01:01:04	03:01:01:04	HLA15506	03:01:01G	1	2	2	0	0	0	0	5								WD	WD
DQB1*03:01:01:05	03:01:01:05	HLA15507	03:01:01G	17	6	378	2	53	3	40	499	WD	WD	I		I		I	I	I
DQB1*03:01:04	03:01:04	HLA03175	03:01:01G	1809	11	1786	168	433	64	536	4807	C	WD	C	C	C	C	C	C	C
DQB1*03:01:05	03:01:05	HLA05773	03:01:01G	0	2	2	0	0	0	0	4									
DQB1*03:01:09	03:01:09	HLA09147	03:01:01G	0	0	141	1	2	0	0	144			I					WD	I
DQB1*03:01:11	03:01:11	HLA09149	03:01:01G	0	2	3	0	0	0	0	5								WD	WD
DQB1*03:01:26	03:01:26	HLA12737	03:01:01G	0	1	3	2	2	1	1	10								WD	WD
DQB1*03:01:31	03:01:31	HLA13926	03:01:01G	0	0	8	0	0	0	0	8			WD					WD	WD
DQB1*03:01:35	03:01:35	HLA16125	03:01:01G	0	0	1	0	0	0	0	1									
DQB1*03:09	03:09	HLA00635	03:01:01G	195	2	142	26	56	8	67	496	C		I	WD	I	C	I	I	C
**DQB1*03:19 total**	**03:19 total**			**13228**	**125**	**16143**	**434**	**4169**	**556**	**4209**	**38864**	**C**	**I**	**C**	**C**	**C**	**C**	**C**	**C**	**C**
DQB1*03:19	03:19		03:01:01G	8450	81	11077	293	2540	345	2645	25431	C	I	C	C	C	C	C	C	C
DQB1*03:19:01	03:19:01	HLA02690	03:01:01G	4778	44	5066	141	1629	211	1564	13433	C	I	C	C	C	C	C	C	C
DQB1*03:21	03:21	HLA03174	03:01:01G	1	3	68	0	57	4	14	147			WD		I		I	WD	I
DQB1*03:22	03:22	HLA03295	03:01:01G	0	0	88	1	0	0	2	91			WD					WD	WD
DQB1*03:24	03:24	HLA03596	03:01:01G	0	2	6	0	0	0	1	9			WD					WD	WD
DQB1*03:29	03:29	HLA05771	03:01:01G	0	21	11	6	1	0	3	42		I	WD	WD				WD	I
DQB1*03:35	03:35	HLA06176	03:01:01G	0	1	0	0	0	0	0	1									
DQB1*03:42	03:42	HLA08113	03:01:01G	3	0	30	0	86	18	13	150			WD		C	C	I	WD	C
DQB1*03:49	03:49	HLA09143	03:01:01G	2	0	13	1	0	0	0	16			WD					WD	WD
DQB1*03:50	03:50	HLA09144	03:01:01G	0	1	0	0	0	0	0	1									
DQB1*03:94	03:94	HLA10214	03:01:01G	0	1	0	0	0	0	0	1									
DQB1*03:115	03:115	HLA11056	03:01:01G	0	0	24	0	0	4	4	32			WD					WD	WD
DQB1*03:116	03:116	HLA11078	03:01:01G	0	0	2	0	0	0	0	2									
DQB1*03:165	03:165	HLA12574	03:01:01G	1	0	0	0	0	0	0	1									

Supplemental Table 15: HLA-DQB1 Allele Summary [a]				Allele Count by Population Group [b]								3.0.0 CIWD Category by Population Group [c]								
Allele	Genomic Typing	Allele ID	G group	AFA	API	EURO	MENA	HIS	NAM	UNK	Total	AFA	API	EURO	MENA	HIS	NAM	UNK	Total	Highest Frequency
DQB1*03:169	03:169	HLA13051	03:01:01G	0	0	2	0	0	0	1	3									
DQB1*03:182	03:182	HLA13386	03:01:01G	0	0	1	0	0	0	1	2									
DQB1*03:191	03:191	HLA13711	03:01:01G	0	0	67	0	2	0	0	69			WD					WD	WD
DQB1*03:196	03:196	HLA13929	03:01:01G	1	0	17	0	0	0	1	19			WD					WD	WD
DQB1*03:198	03:198	HLA13931	03:01:01G	0	0	0	5	0	0	0	5				WD				WD	WD
DQB1*03:241	03:241	HLA15598	03:01:01G	0	0	2	0	0	0	0	2									
DQB1*03:243	03:243	HLA15585	03:01:01G	12	0	1	0	4	0	3	20	WD							WD	WD
DQB1*03:246	03:246	HLA16128	03:01:01G	0	0	1	0	0	0	0	1									
DQB1*03:253	03:253	HLA16163	03:01:01G	0	0	6	0	2	0	0	8			WD					WD	WD
DQB1*03:01:02	03:01:02	HLA00626		0	2	57	32	1	0	1	93			WD	WD				WD	WD
DQB1*03:01:03	03:01:03	HLA02688		7	3	792	2	10	0	65	879	WD		I		I		I	I	I
DQB1*03:01:06	03:01:06	HLA06142		0	1	0	0	0	0	0	1									
DQB1*03:01:07	03:01:07	HLA09094		1	0	1	0	0	0	0	2									
DQB1*03:01:08	03:01:08	HLA09096		5	15	16	0	1	0	4	41	WD	I	WD					WD	I
DQB1*03:01:13	03:01:13	HLA09233		0	0	6	0	0	0	1	7			WD					WD	WD
DQB1*03:01:14	03:01:14	HLA09235		1	1	53	0	0	0	15	70			WD				I	WD	I
DQB1*03:01:15	03:01:15	HLA09239		0	0	1	0	0	0	0	1									
DQB1*03:01:16	03:01:16	HLA09242		0	0	1	0	0	0	0	1									
DQB1*03:01:17	03:01:17	HLA09634		0	0	3	1	0	0	0	4									
DQB1*03:01:18	03:01:18	HLA09639		0	0	0	0	0	0	4	4									
DQB1*03:01:22	03:01:22	HLA10540		0	0	19	4	0	0	5	28			WD				WD	WD	WD
DQB1*03:01:27	03:01:27	HLA12794		0	0	0	5	0	0	1	6				WD				WD	WD
DQB1*03:01:28	03:01:28	HLA12796		0	0	1	0	0	0	1	2									
DQB1*03:01:29	03:01:29	HLA13122		0	0	1	0	0	0	0	1									
DQB1*03:01:30	03:01:30	HLA13622		0	3	2	0	0	0	0	5								WD	WD
**DQB1*03:02 total**	**03:02 total**			**14783**	**110398**	**1073164**	**43586**	**121773**	**10923**	**134786**	**1509413**	**C**	**C**	**C**	**C**	**C**	**C**	**C**	**C**	**C**
DQB1*03:02	03:02			12	108	15972	1343	95	6	494	18030	WD	I	C	C	C	WD	C	C	C
DQB1*03:02P	03:02P			0	0	7	2	0	0	4	13			WD					WD	WD
**DQB1*03:02:01G total**	**03:02:01G total**			**14734**	**110234**	**1052556**	**42214**	**121485**	**10888**	**133871**	**1485982**	**C**	**C**	**C**	**C**	**C**	**C**	**C**	**C**	**C**
DQB1*03:02:01G	03:02:01G		03:02:01G	8226	82111	310200	16736	71932	6889	103554	599648	C	C	C	C	C	C	C	C	C
DQB1*03:02:01	03:02:01		03:02:01G	6508	28123	742176	25418	49551	3999	30309	886084	C	C	C	C	C	C	C	C	C

Supplemental Table 15: HLA-DQB1 Allele Summary [a]				Allele Count by Population Group [b]								3.0.0 CIWD Category by Population Group [c]								
Allele	Genomic Typing	Allele ID	G group	AFA	API	EURO	MENA	HIS	NAM	UNK	Total	AFA	API	EURO	MENA	HIS	NAM	UNK	Total	Highest Frequency
DQB1*03:02:01:01	03:02:01:01	HLA00627	03:02:01G	0	0	118	53	0	0	2	173			I	C				I	C
DQB1*03:02:01:02	03:02:01:02	HLA15503	03:02:01G	0	0	18	0	0	0	0	18			WD					WD	WD
DQB1*03:02:09	03:02:09	HLA09846	03:02:01G	0	0	11	0	0	0	0	11			WD					WD	WD
DQB1*03:02:12	03:02:12	HLA10217	03:02:01G	0	0	8	0	0	0	3	11			WD					WD	WD
DQB1*03:02:21	03:02:21	HLA16146	03:02:01G	0	0	12	0	0	0	0	12			WD					WD	WD
DQB1*03:02:24	03:02:24	HLA16158	03:02:01G	0	0	1	0	0	0	0	1									
DQB1*03:02:25	03:02:25	HLA17347	03:02:01G	0	0	0	0	0	0	1	1									
DQB1*03:32	03:32	HLA06143	03:02:01G	0	0	1	0	0	0	0	1									
DQB1*03:190	03:190	HLA13708	03:02:01G	0	0	0	7	2	0	0	9				WD				WD	WD
DQB1*03:247	03:247	HLA16130	03:02:01G	0	0	8	0	0	0	1	9			WD					WD	WD
DQB1*03:251	03:251	HLA16143	03:02:01G	0	0	2	0	0	0	0	2									
DQB1*03:263	03:263	HLA17064	03:02:01G	0	0	1	0	0	0	1	2									
DQB1*03:02:02	03:02:02	HLA01770		23	15	4502	17	130	16	364	5067	WD	I	C	WD	C	C	C	C	C
DQB1*03:02:03	03:02:03	HLA02625		14	1	78	10	61	11	26	201	WD		WD	WD	I	C	I	I	C
DQB1*03:02:06	03:02:06	HLA09285		0	38	22	0	0	0	27	87		I	WD				I	WD	I
DQB1*03:02:07	03:02:07	HLA09288		0	0	2	0	1	1	0	4									
DQB1*03:02:08	03:02:08	HLA09741		0	2	1	0	0	0	0	3									
DQB1*03:02:10	03:02:10	HLA10030		0	0	6	0	0	0	0	6			WD					WD	WD
DQB1*03:02:11	03:02:11	HLA10216		0	0	3	0	0	0	0	3									
DQB1*03:02:13	03:02:13	HLA10283		0	0	6	0	0	0	0	6			WD					WD	WD
DQB1*03:02:14	03:02:14	HLA11282		0	0	1	0	0	1	0	2									
DQB1*03:02:15	03:02:15	HLA11887		0	0	0	0	1	0	0	1									
DQB1*03:02:17	03:02:17	HLA12035		0	0	3	0	0	0	0	3									
DQB1*03:02:18	03:02:18	HLA13138		0	0	4	0	0	0	0	4									
DQB1*03:02:19	03:02:19	HLA13140		0	0	1	0	0	0	0	1									
**DQB1*03:03 total**	**03:03 total**			**5478**	**112706**	**513124**	**9275**	**15819**	**1841**	**47409**	**705652**	**C**	**C**	**C**	**C**	**C**	**C**	**C**	**C**	**C**
DQB1*03:03	03:03			18	162	14513	579	35	0	254	15561	WD	C	C	C	I		C	C	C
DQB1*03:03P	03:03P			0	0	7	0	0	0	0	7			WD					WD	WD
**DQB1*03:03:02G total**	**03:03:02G total**			**5458**	**112538**	**497765**	**8695**	**15771**	**1837**	**47124**	**689188**	**C**	**C**	**C**	**C**	**C**	**C**	**C**	**C**	**C**
DQB1*03:03:02G	03:03:02G		03:03:02G	2972	81829	133514	3032	9685	1156	33425	265613	C	C	C	C	C	C	C	C	C
DQB1*03:03:02	03:03:02		03:03:02G	2171	29599	358710	5535	5162	583	12861	414621	C	C	C	C	C	C	C	C	C

Supplemental Table 15: HLA-DQB1 Allele Summary [a]				Allele Count by Population Group [b]								3.0.0 CIWD Category by Population Group [c]								
Allele	Genomic Typing	Allele ID	G group	AFA	API	EURO	MENA	HIS	NAM	UNK	Total	AFA	API	EURO	MENA	HIS	NAM	UNK	Total	Highest Frequency
DQB1*03:03:02:01	03:03:02:01	HLA00629	03:03:02G	310	1056	5381	82	811	80	811	8531	C	C	C	C	C	C	C	C	C
DQB1*03:03:02:02	03:03:02:02	HLA06620	03:03:02G	0	0	5	2	0	0	1	8			WD					WD	WD
DQB1*03:03:02:04	03:03:02:04	HLA12588	03:03:02G	4	4	2	0	113	18	20	161					C	C	I	I	C
DQB1*03:03:04	03:03:04	HLA06822	03:03:02G	1	45	109	44	0	0	3	202		I	WD	C				I	C
DQB1*03:03:09	03:03:09	HLA10223	03:03:02G	0	0	1	0	0	0	0	1									
DQB1*03:31	03:31	HLA05761	03:03:02G	0	0	37	0	0	0	2	39			WD					WD	WD
DQB1*03:33	03:33	HLA06144	03:03:02G	0	0	2	0	0	0	0	2									
DQB1*03:79	03:79	HLA09653	03:03:02G	0	5	3	0	0	0	0	8		WD						WD	WD
DQB1*03:96	03:96	HLA10221	03:03:02G	0	0	1	0	0	0	0	1									
DQB1*03:249	03:249	HLA16132	03:03:02G	0	0	0	0	0	0	1	1									
DQB1*03:03:03	03:03:03	HLA01004		1	4	834	1	3	1	26	870			I				I	I	I
DQB1*03:03:05	03:03:05	HLA09281		0	0	2	0	0	0	2	4									
DQB1*03:03:06	03:03:06	HLA09283		0	1	0	0	0	0	0	1									
DQB1*03:03:07	03:03:07	HLA09650		1	1	2	0	0	1	0	5								WD	WD
DQB1*03:03:11	03:03:11	HLA10280		0	0	1	0	10	2	3	16					I			WD	I
**DQB1*03:04 total**	**03:04 total**			**139**	**146**	**33978**	**1071**	**1911**	**126**	**2862**	**40233**	**C**	**C**	**C**	**C**	**C**	**C**	**C**	**C**	**C**
DQB1*03:04	03:04			53	44	12441	420	728	74	984	14744	C	I	C	C	C	C	C	C	C
**DQB1*03:04:01G total**	**03:04:01G total**			**86**	**102**	**21536**	**651**	**1180**	**51**	**1877**	**25483**	**C**	**I**	**C**	**C**	**C**	**C**	**C**	**C**	**C**
DQB1*03:04:01G	03:04:01G		03:04:01G	0	11	189	0	39	1	110	350		WD	I		I		I	I	I
DQB1*03:04:01	03:04:01	HLA00630	03:04:01G	86	91	21346	651	1141	50	1767	25132	C	I	C	C	C	C	C	C	C
DQB1*03:04:03	03:04:03	HLA16154	03:04:01G	0	0	1	0	0	0	0	1									
DQB1*03:04:02	03:04:02	HLA11278		0	0	1	0	3	1	1	6								WD	WD
**DQB1*03:05 total**	**03:05 total**			**57**	**1528**	**13911**	**4505**	**515**	**40**	**3266**	**23822**	**C**	**C**	**C**	**C**	**C**	**C**	**C**	**C**	**C**
DQB1*03:05	03:05			7	41	1590	367	91	5	159	2260	WD	I	C	C	C	WD	C	C	C
**DQB1*03:05:01G total**	**03:05:01G total**			**46**	**1423**	**11929**	**3987**	**403**	**26**	**3035**	**20849**	**C**	**C**	**C**	**C**	**C**	**C**	**C**	**C**	**C**
DQB1*03:05:01G	03:05:01G		03:05:01G	1	422	1272	154	78	3	1163	3093		C	C	C	C		C	C	C
DQB1*03:05:01	03:05:01	HLA00631	03:05:01G	45	1001	10657	3833	325	23	1872	17756	C	C	C	C	C	C	C	C	C
DQB1*03:05:02	03:05:02	HLA01443		0	11	36	12	1	0	3	63		WD	WD	WD				WD	WD
DQB1*03:05:03	03:05:03	HLA01744		4	1	282	1	15	4	47	354			I		I		I	I	I
DQB1*03:05:04	03:05:04	HLA02497		0	52	74	138	5	5	22	296		I	WD	C	WD	WD	I	I	C
DQB1*03:06	03:06	HLA00632		0	3	1	0	0	0	1	5								WD	WD

Supplemental Table 15: HLA-DQB1 Allele Summary [a]				Allele Count by Population Group [b]								3.0.0 CIWD Category by Population Group [c]								
Allele	Genomic Typing	Allele ID	G group	AFA	API	EURO	MENA	HIS	NAM	UNK	Total	AFA	API	EURO	MENA	HIS	NAM	UNK	Total	Highest Frequency
DQB1*03:08	03:08	HLA00634		1	0	101	0	1	0	17	120			WD				I	WD	I
**DQB1*03:10 total**	**03:10 total**			**0**	**7**	**45**	**1**	**1**	**0**	**0**	**54**		**WD**	**WD**					**WD**	**WD**
DQB1*03:10	03:10			0	3	8	0	0	0	0	11			WD					WD	WD
**DQB1*03:10:01G total**	**03:10:01G total**			**0**	**2**	**23**	**0**	**0**	**0**	**0**	**25**			**WD**					**WD**	**WD**
DQB1*03:10:01G	03:10:01G		03:10:01G	0	0	1	0	0	0	0	1									
DQB1*03:10:01	03:10:01	HLA01164	03:10:01G	0	2	7	0	0	0	0	9			WD					WD	WD
DQB1*03:195	03:195	HLA13919	03:10:01G	0	0	15	0	0	0	0	15			WD					WD	WD
**DQB1*03:10:02G total**	**03:10:02G total**			**0**	**2**	**14**	**1**	**1**	**0**	**0**	**18**			**WD**					**WD**	**WD**
DQB1*03:10:02G	03:10:02G		03:10:02G	0	1	0	0	0	0	0	1									
DQB1*03:10:02	03:10:02		03:10:02G	0	1	14	1	1	0	0	17			WD					WD	WD
DQB1*03:11	03:11	HLA01538		0	250	3	3	1	0	12	269		C					WD	I	C
DQB1*03:12	03:12	HLA01574		1	3	1086	1	0	0	10	1101			I				WD	I	I
DQB1*03:13	03:13	HLA01589		0	174	9	1	0	0	25	209		C	WD				I	I	C
**DQB1*03:14 total**	**03:14 total**			**1**	**35**	**6**	**0**	**9**	**0**	**3**	**54**		**I**	**WD**		**I**			**WD**	**I**
DQB1*03:14	03:14			0	4	1	0	2	0	0	7								WD	WD
DQB1*03:14:01	03:14:01	HLA02026		1	2	0	0	0	0	0	3									
DQB1*03:14:02	03:14:02	HLA09240		0	29	5	0	7	0	3	44		I	WD		I			WD	I
DQB1*03:16	03:16	HLA02208		53	1	1	0	4	0	10	69	C						WD	WD	C
**DQB1*03:17 total**	**03:17 total**			**0**	**20**	**2**	**0**	**1**	**0**	**0**	**23**		**I**						**WD**	**I**
DQB1*03:17	03:17			0	4	1	0	0	0	0	5								WD	WD
DQB1*03:17:01	03:17:01	HLA02323		0	16	1	0	1	0	0	18		I						WD	I
DQB1*03:18	03:18	HLA02372		0	0	12	0	0	0	6	18			WD				WD	WD	WD
DQB1*03:20	03:20	HLA02887		0	0	10	0	0	0	0	10			WD					WD	WD
**DQB1*03:23 total**	**03:23 total**			**0**	**1**	**1**	**0**	**0**	**0**	**1**	**3**									
DQB1*03:23	03:23			0	0	0	0	0	0	1	1									
DQB1*03:23:01	03:23:01	HLA03451		0	1	0	0	0	0	0	1									
DQB1*03:23:02	03:23:02	HLA13580		0	0	1	0	0	0	0	1									
**DQB1*03:25 total**	**03:25 total**			**2**	**0**	**11**	**0**	**56**	**10**	**18**	**97**			**WD**		**I**	**C**	**I**	**WD**	**C**
DQB1*03:25	03:25			1	0	8	0	35	6	6	56			WD		I	WD	WD	WD	I
DQB1*03:25:01	03:25:01	HLA03745		1	0	3	0	21	4	12	41					I		WD	WD	I
DQB1*03:26	03:26	HLA04356		0	15	0	0	0	0	1	16		I						WD	I

Supplemental Table 15: HLA-DQB1 Allele Summary [a]				Allele Count by Population Group [b]								3.0.0 CIWD Category by Population Group [c]								
Allele	Genomic Typing	Allele ID	G group	AFA	API	EURO	MENA	HIS	NAM	UNK	Total	AFA	API	EURO	MENA	HIS	NAM	UNK	Total	Highest Frequency
DQB1*03:27	03:27	HLA05374		0	3	0	0	0	0	0	3									
DQB1*03:28	03:28	HLA05767		0	0	2	0	0	0	0	2									
DQB1*03:30	03:30	HLA05774		0	0	14	0	1	0	0	15			WD					WD	WD
DQB1*03:34	03:34	HLA06145		0	1	0	0	0	0	0	1									
DQB1*03:36	03:36	HLA06177		0	0	6	0	0	0	0	6			WD					WD	WD
DQB1*03:37	03:37	HLA06516		0	0	90	1	0	0	20	111			WD				I	WD	I
DQB1*03:38	03:38	HLA06880		0	1	0	0	0	0	0	1									
DQB1*03:40	03:40	HLA07799		0	0	18	0	0	0	2	20			WD					WD	WD
DQB1*03:44	03:44	HLA08289		0	1	2	1	0	0	0	4									
DQB1*03:45	03:45	HLA08955		0	0	15	0	0	1	0	16			WD					WD	WD
DQB1*03:46	03:46	HLA09093		0	0	1	0	0	0	0	1									
DQB1*03:47	03:47	HLA09095		0	0	2	0	0	0	1	3									
DQB1*03:48	03:48	HLA09129		0	0	6	0	0	0	0	6			WD					WD	WD
DQB1*03:52	03:52	HLA09220		0	0	0	2	0	0	0	2									
DQB1*03:53	03:53	HLA09228		0	0	3	10	0	0	0	13				WD				WD	WD
DQB1*03:54	03:54	HLA09231		0	0	3	2	1	0	1	7								WD	WD
DQB1*03:55	03:55	HLA09232		0	0	30	0	0	0	7	37			WD				WD	WD	WD
DQB1*03:56	03:56	HLA09234		0	0	7	0	0	0	1	8			WD					WD	WD
DQB1*03:57	03:57	HLA09236		0	1	2	0	0	0	0	3									
DQB1*03:58	03:58	HLA09237		0	0	5	1	0	0	17	23			WD				I	WD	I
DQB1*03:59	03:59	HLA09238		0	0	9	0	0	0	0	9			WD					WD	WD
DQB1*03:60	03:60	HLA09241		0	0	4	0	0	0	0	4									
DQB1*03:61	03:61	HLA09261		0	0	1	0	0	0	0	1									
DQB1*03:62	03:62	HLA09263		0	11	0	0	0	0	1	12		WD						WD	WD
DQB1*03:63	03:63	HLA09279		0	0	14	0	0	0	0	14			WD					WD	WD
DQB1*03:64	03:64	HLA09280		0	1	10	0	1	0	2	14			WD					WD	WD
DQB1*03:65	03:65	HLA09282		0	0	11	0	0	0	0	11			WD					WD	WD
DQB1*03:67	03:67	HLA09286		2	0	24	0	1	0	7	34			WD				WD	WD	WD
DQB1*03:68	03:68	HLA09287		0	0	22	0	0	0	0	22			WD					WD	WD
DQB1*03:69	03:69	HLA09289		0	1	2	1	0	0	2	6								WD	WD
DQB1*03:70	03:70	HLA09230		0	0	3	0	12	1	2	18					I			WD	I

Supplemental Table 15: HLA-DQB1 Allele Summary [a]				Allele Count by Population Group [b]								3.0.0 CIWD Category by Population Group [c]								
Allele	Genomic Typing	Allele ID	G group	AFA	API	EURO	MENA	HIS	NAM	UNK	Total	AFA	API	EURO	MENA	HIS	NAM	UNK	Total	Highest Frequency
DQB1*03:71	03:71	HLA09356		0	0	2	0	1	0	0	3									
DQB1*03:72	03:72	HLA09360		0	0	4	0	35	4	14	57					I		I	WD	I
DQB1*03:74	03:74	HLA09635		1	0	1	0	0	0	0	2									
DQB1*03:75	03:75	HLA09636		1	0	0	0	0	0	1	2									
DQB1*03:76	03:76	HLA09637		0	0	5	0	0	0	0	5			WD					WD	WD
DQB1*03:78	03:78	HLA09640		0	11	1	0	0	0	0	12		WD						WD	WD
DQB1*03:81	03:81	HLA09732		0	0	9	0	0	0	1	10			WD					WD	WD
DQB1*03:82	03:82	HLA09773		0	0	1	0	0	0	0	1									
DQB1*03:86	03:86	HLA09848		0	1	0	0	0	0	0	1									
DQB1*03:92	03:92	HLA10210		0	0	2	0	0	0	0	2									
DQB1*03:95N	03:95N	HLA10219		0	0	1	0	0	0	0	1									
DQB1*03:101	03:101	HLA10274		0	2	0	0	0	0	0	2									
DQB1*03:102	03:102	HLA10275		0	0	3	0	0	0	0	3									
DQB1*03:104	03:104	HLA10284		0	2	0	0	0	0	0	2									
DQB1*03:105	03:105	HLA10285		0	0	2	0	7	0	0	9					I			WD	I
DQB1*03:106	03:106	HLA10286		0	7	4	0	11	0	2	24		WD			I			WD	I
DQB1*03:107	03:107	HLA10287		0	0	1	0	0	0	0	1									
DQB1*03:109	03:109	HLA10541		0	0	28	0	0	0	0	28			WD					WD	WD
DQB1*03:111	03:111	HLA10547		0	1	0	0	0	0	0	1									
DQB1*03:112	03:112	HLA10548		0	0	3	0	0	0	0	3									
DQB1*03:113	03:113	HLA10663		0	1	2	0	0	0	0	3									
DQB1*03:114	03:114	HLA10928		0	0	22	1	1	0	1	25			WD					WD	WD
DQB1*03:118N	03:118N	HLA11127		0	9	0	0	0	0	0	9		WD						WD	WD
DQB1*03:119	03:119	HLA11128		0	0	1	0	0	0	0	1									
DQB1*03:120	03:120	HLA11129		0	0	2	0	0	0	0	2									
DQB1*03:121	03:121	HLA11130		0	4	0	0	2	0	0	6								WD	WD
DQB1*03:122	03:122	HLA11131		1	0	0	0	0	0	0	1									
DQB1*03:125	03:125	HLA11143		0	0	2	0	0	0	0	2									
DQB1*03:126	03:126	HLA11144		0	0	3	0	0	0	2	5								WD	WD
DQB1*03:127	03:127	HLA11272		1	0	0	0	2	0	2	5								WD	WD
DQB1*03:130	03:130	HLA11276		0	0	25	0	0	0	0	25			WD					WD	WD

Supplemental Table 15: HLA-DQB1 Allele Summary [a]				Allele Count by Population Group [b]								3.0.0 CIWD Category by Population Group [c]								
Allele	Genomic Typing	Allele ID	G group	AFA	API	EURO	MENA	HIS	NAM	UNK	Total	AFA	API	EURO	MENA	HIS	NAM	UNK	Total	Highest Frequency
DQB1*03:131	03:131	HLA11277		0	0	0	0	2	0	0	2									
DQB1*03:132	03:132	HLA11558		1	0	0	0	12	0	1	14					I			WD	I
DQB1*03:133	03:133	HLA11348		0	0	3	0	0	0	0	3									
DQB1*03:134	03:134	HLA11350		0	0	14	0	0	0	0	14			WD					WD	WD
DQB1*03:136	03:136	HLA11356		1	0	43	0	0	0	7	51			WD				WD	WD	WD
DQB1*03:137	03:137	HLA11357		0	0	3	0	0	0	0	3									
DQB1*03:138	03:138	HLA11358		0	0	14	0	0	0	2	16			WD					WD	WD
DQB1*03:141	03:141	HLA11591		0	0	13	0	0	0	0	13			WD					WD	WD
DQB1*03:142	03:142	HLA11598		0	1	0	0	0	0	0	1									
DQB1*03:143	03:143	HLA11671		0	0	1	0	0	0	0	1									
DQB1*03:145	03:145	HLA11678		0	1	0	0	0	0	0	1									
DQB1*03:149	03:149	HLA12028		0	0	0	0	0	0	1	1									
DQB1*03:150	03:150	HLA12046		2	0	0	0	0	0	1	3									
DQB1*03:153	03:153	HLA12211		0	0	2	0	0	0	0	2									
DQB1*03:154	03:154	HLA12352		0	0	2	0	0	0	0	2									
DQB1*03:157	03:157	HLA12359		0	0	0	0	1	0	0	1									
DQB1*03:159	03:159	HLA12519		0	0	2	0	0	0	0	2									
DQB1*03:160	03:160	HLA12520		0	0	4	0	0	0	0	4									
DQB1*03:162	03:162	HLA12474		0	0	5	0	0	0	0	5			WD					WD	WD
DQB1*03:166	03:166	HLA12788		0	0	2	0	0	0	0	2									
DQB1*03:167	03:167	HLA12795		0	0	0	0	0	0	1	1									
DQB1*03:170	03:170	HLA13118		1	0	0	0	0	0	0	1									
DQB1*03:171	03:171	HLA13119		0	0	1	0	0	0	0	1									
DQB1*03:174	03:174	HLA13123		0	0	2	0	1	0	1	4									
DQB1*03:178	03:178	HLA13139		0	0	0	1	0	0	0	1									
DQB1*03:179	03:179	HLA13246		0	0	1	0	0	0	0	1									
DQB1*03:183	03:183	HLA13578		0	1	0	0	0	0	0	1									
DQB1*03:184	03:184	HLA13579		0	2	2	0	0	0	0	4									
DQB1*03:185	03:185	HLA13581		0	0	1	0	0	0	0	1									
DQB1*03:187	03:187	HLA13678		1	0	0	0	0	0	0	1									
DQB1*03:188	03:188	HLA13679		0	1	2	0	0	0	0	3									

Supplemental Table 15: HLA-DQB1 Allele Summary [a]				Allele Count by Population Group [b]								3.0.0 CIWD Category by Population Group [c]								
Allele	Genomic Typing	Allele ID	G group	AFA	API	EURO	MENA	HIS	NAM	UNK	Total	AFA	API	EURO	MENA	HIS	NAM	UNK	Total	Highest Frequency
DQB1*03:189	03:189	HLA13682		0	0	1	0	0	0	0	1									
DQB1*03:194	03:194	HLA13881		0	0	1	0	0	0	0	1									
DQB1*03:197Q	03:197Q	HLA13930		0	0	0	5	0	0	1	6				WD				WD	WD
DQB1*03:199	03:199	HLA13938		0	1	2	0	0	0	0	3									
DQB1*03:201	03:201	HLA14035		0	0	15	0	0	0	0	15			WD					WD	WD
DQB1*03:204	03:204	HLA14039		0	3	0	0	0	0	0	3									
DQB1*03:205	03:205	HLA14040		0	1	6	0	0	0	0	7			WD					WD	WD
DQB1*03:207	03:207	HLA14160		0	0	0	0	1	3	1	5								WD	WD
DQB1*03:211	03:211	HLA14166		0	0	1	0	0	0	0	1									
DQB1*03:212	03:212	HLA14167		0	1	0	0	0	0	0	1									
DQB1*03:215	03:215	HLA14171		0	0	4	0	0	0	0	4									
DQB1*03:217	03:217	HLA14346		0	0	0	0	0	0	1	1									
DQB1*03:218	03:218	HLA14202		0	0	4	0	0	0	0	4									
DQB1*03:224	03:224	HLA14761		0	1	0	0	0	0	0	1									
DQB1*03:228	03:228	HLA15068		0	2	0	0	0	0	0	2									
DQB1*03:229	03:229	HLA15001		0	0	1	0	0	0	0	1									
DQB1*03:235	03:235	HLA15679		0	0	1	0	0	0	0	1									
DQB1*03:268	03:268	HLA17520		0	1	0	0	0	0	0	1									
DQB1*03:CODE	03:CODE			4001	5992	62316	392	9946	617	11353	94617	NA	NA	NA	NA	NA	NA	NA	NA	NA
**DQB1*04:01 total**	**04:01 total**			**19**	**18612**	**865**	**357**	**83**	**16**	**3161**	**23113**	**WD**	**C**	**I**	**C**	**C**	**C**	**C**	**C**	**C**
DQB1*04:01	04:01			0	18	10	10	1	0	3	42		I	WD	WD				WD	I
**DQB1*04:01:01G total**	**04:01:01G total**			**19**	**18575**	**853**	**336**	**82**	**16**	**3158**	**23039**	**WD**	**C**	**I**	**C**	**C**	**C**	**C**	**C**	**C**
DQB1*04:01:01G	04:01:01G		04:01:01G	10	11666	252	48	39	12	1702	13729	WD	C	I	C	I	C	C	C	C
DQB1*04:01:01	04:01:01		04:01:01G	9	6909	601	288	43	4	1456	9310	WD	C	I	C	I		C	C	C
DQB1*04:01:02	04:01:02	HLA03782		0	19	2	11	0	0	0	32		I		WD				WD	I
**DQB1*04:02 total**	**04:02 total**			**25647**	**20495**	**341850**	**9251**	**66670**	**5930**	**48276**	**518119**	**C**	**C**	**C**	**C**	**C**	**C**	**C**	**C**	**C**
DQB1*04:02	04:02			952	923	29659	740	2124	157	1345	35900	C	C	C	C	C	C	C	C	C
DQB1*04:02P	04:02P			0	0	2	0	0	0	2	4									
**DQB1*04:02:01G total**	**04:02:01G total**			**24694**	**19538**	**312112**	**8511**	**64542**	**5772**	**46915**	**482084**	**C**	**C**	**C**	**C**	**C**	**C**	**C**	**C**	**C**
DQB1*04:02:01G	04:02:01G		04:02:01G	8947	12019	57323	2913	27675	2283	28921	140081	C	C	C	C	C	C	C	C	C
DQB1*04:02:01	04:02:01		04:02:01G	15718	7476	253951	5577	36085	3440	17843	340090	C	C	C	C	C	C	C	C	C

Supplemental Table 15: HLA-DQB1 Allele Summary [a]				Allele Count by Population Group [b]								3.0.0 CIWD Category by Population Group [c]								
Allele	Genomic Typing	Allele ID	G group	AFA	API	EURO	MENA	HIS	NAM	UNK	Total	AFA	API	EURO	MENA	HIS	NAM	UNK	Total	Highest Frequency
DQB1*04:02:01:01	04:02:01:01	HLA00637	04:02:01G	29	43	831	21	782	49	149	1904	WD	I	I	WD	C	C	C	C	C
DQB1*04:02:10	04:02:10	HLA13933	04:02:01G	0	0	1	0	0	0	0	1									
DQB1*04:02:12	04:02:12	HLA16185	04:02:01G	0	0	3	0	0	0	0	3									
DQB1*04:02:13	04:02:13	HLA17165	04:02:01G	0	0	1	0	0	0	1	2									
DQB1*04:13	04:13	HLA09652	04:02:01G	0	0	1	0	0	0	0	1									
DQB1*04:39	04:39	HLA16318	04:02:01G	0	0	0	0	0	0	1	1									
DQB1*04:41N	04:41N	HLA17403	04:02:01G	0	0	1	0	0	0	0	1									
DQB1*04:02:02	04:02:02	HLA05770		0	0	7	0	0	0	0	7			WD					WD	WD
DQB1*04:02:03	04:02:03	HLA09361		0	34	69	0	0	0	3	106		I	WD					WD	I
DQB1*04:02:04	04:02:04	HLA09745		0	0	1	0	3	0	1	5								WD	WD
DQB1*04:02:06	04:02:06	HLA11364		0	0	0	0	1	1	0	2									
DQB1*04:02:07	04:02:07	HLA12048		0	0	0	0	0	0	10	10							WD	WD	WD
DQB1*04:02:08	04:02:08	HLA12049		0	0	1	0	0	0	0	1									
DQB1*04:02:09	04:02:09	HLA13148		1	0	0	0	0	0	0	1									
**DQB1*04:03 total**	**04:03 total**			**0**	**1**	**17**	**0**	**4**	**0**	**1**	**23**			**WD**					**WD**	**WD**
DQB1*04:03	04:03			0	0	2	0	0	0	0	2									
DQB1*04:03:01	04:03:01	HLA03234		0	0	6	0	3	0	0	9			WD					WD	WD
DQB1*04:03:02	04:03:02	HLA04125		0	1	9	0	1	0	1	12			WD					WD	WD
DQB1*04:04	04:04	HLA04390		0	0	98	0	0	0	1	99			WD					WD	WD
DQB1*04:11	04:11	HLA09359		0	0	21	0	0	0	2	23			WD					WD	WD
DQB1*04:12	04:12	HLA09362		0	25	0	0	1	0	2	28		I						WD	I
DQB1*04:18	04:18	HLA10294		2	0	0	0	0	0	1	3									
DQB1*04:19	04:19	HLA10932		0	0	0	0	1	0	0	1									
DQB1*04:20	04:20	HLA11362		0	0	0	0	0	0	1	1									
DQB1*04:21	04:21	HLA11363		0	0	2	0	1	0	1	4									
DQB1*04:22	04:22	HLA11365		4	0	0	0	0	0	1	5								WD	WD
DQB1*04:26	04:26	HLA12802		0	0	1	0	0	0	0	1									
DQB1*04:27	04:27	HLA12803		0	0	3	0	0	0	0	3									
DQB1*04:28	04:28	HLA13146		0	0	1	0	0	0	0	1									
DQB1*04:30	04:30	HLA13939		0	0	3	0	0	0	0	3									
DQB1*04:32	04:32	HLA13982		0	0	2	0	0	0	0	2									

Supplemental Table 15: HLA-DQB1 Allele Summary [a]				Allele Count by Population Group [b]								3.0.0 CIWD Category by Population Group [c]								
Allele	Genomic Typing	Allele ID	G group	AFA	API	EURO	MENA	HIS	NAM	UNK	Total	AFA	API	EURO	MENA	HIS	NAM	UNK	Total	Highest Frequency
DQB1*04:33	04:33	HLA14350		0	0	2	0	0	0	0	2									
DQB1*04:34	04:34	HLA14763		0	1	0	0	0	0	0	1									
DQB1*04:36N	04:36N	HLA15327		0	0	1	0	3	0	0	4									
DQB1*04:CODE	04:CODE			1018	816	4046	31	2409	131	1133	9584	NA	NA	NA	NA	NA	NA	NA	NA	NA
**DQB1*05:01 total**	**05:01 total**			**55148**	**124233**	**1361411**	**36298**	**72282**	**7284**	**135788**	**1792444**	**C**	**C**	**C**	**C**	**C**	**C**	**C**	**C**	**C**
DQB1*05:01	05:01			1953	2694	118968	3200	3280	172	3092	133359	C	C	C	C	C	C	C	C	C
DQB1*05:01P	05:01P			0	0	28	1	0	0	13	42			WD				I	WD	I
**DQB1*05:01:01G total**	**05:01:01G total**			**53194**	**121527**	**1242295**	**33094**	**69002**	**7112**	**132656**	**1658880**	**C**	**C**	**C**	**C**	**C**	**C**	**C**	**C**	**C**
DQB1*05:01:01G	05:01:01G		05:01:01G	19185	87662	243926	13991	30475	2819	82934	480992	C	C	C	C	C	C	C	C	C
DQB1*05:01:01	05:01:01		05:01:01G	33124	33774	995555	18899	36647	4154	49189	1171342	C	C	C	C	C	C	C	C	C
DQB1*05:01:01:01	05:01:01:01	HLA00638	05:01:01G	881	60	2337	155	1873	139	514	5959	C	I	C	C	C	C	C	C	C
DQB1*05:01:01:02	05:01:01:02	HLA06615	05:01:01G	0	0	11	47	0	0	1	59			WD	C				WD	C
DQB1*05:01:01:03	05:01:01:03	HLA12879	05:01:01G	0	0	215	0	0	0	1	216			I					I	I
DQB1*05:01:01:05	05:01:01:05	HLA17371	05:01:01G	0	0	2	0	0	0	0	2									
DQB1*05:01:21	05:01:21	HLA13917	05:01:01G	0	0	8	0	0	0	0	8			WD					WD	WD
DQB1*05:01:22	05:01:22	HLA13920	05:01:01G	0	0	6	0	0	0	0	6			WD					WD	WD
DQB1*05:01:23	05:01:23	HLA16151	05:01:01G	0	0	2	0	0	0	0	2									
DQB1*05:18	05:18	HLA08605	05:01:01G	0	30	18	1	0	0	2	51		I	WD					WD	I
DQB1*05:27	05:27	HLA09509	05:01:01G	1	0	15	0	1	0	2	19			WD					WD	WD
DQB1*05:31	05:31	HLA09828	05:01:01G	0	0	23	1	0	0	1	25			WD					WD	WD
DQB1*05:45	05:45	HLA10236	05:01:01G	2	0	1	0	0	0	0	3									
DQB1*05:84	05:84	HLA12403	05:01:01G	0	0	30	0	0	0	1	31			WD					WD	WD
DQB1*05:103	05:103	HLA13921	05:01:01G	1	0	38	0	3	0	6	48			WD				WD	WD	WD
DQB1*05:104	05:104	HLA13928	05:01:01G	0	0	95	0	0	0	1	96			WD					WD	WD
DQB1*05:107	05:107	HLA13941	05:01:01G	0	0	4	0	3	0	2	9								WD	WD
DQB1*05:133	05:133	HLA16053	05:01:01G	0	0	8	0	0	0	0	8			WD					WD	WD
DQB1*05:137	05:137	HLA16161	05:01:01G	0	0	0	0	0	0	1	1									
DQB1*05:144	05:144	HLA16645	05:01:01G	0	1	0	0	0	0	1	2									
DQB1*05:152	05:152	HLA17344	05:01:01G	0	0	1	0	0	0	0	1									
DQB1*05:01:02	05:01:02	HLA01086		0	0	5	2	0	0	0	7			WD					WD	WD
DQB1*05:01:03	05:01:03	HLA05548		1	0	4	0	0	0	1	6								WD	WD

Supplemental Table 15: HLA-DQB1 Allele Summary [a]				Allele Count by Population Group [b]								3.0.0 CIWD Category by Population Group [c]								
Allele	Genomic Typing	Allele ID	G group	AFA	API	EURO	MENA	HIS	NAM	UNK	Total	AFA	API	EURO	MENA	HIS	NAM	UNK	Total	Highest Frequency
DQB1*05:01:04	05:01:04	HLA09293		0	0	0	1	0	0	0	1									
DQB1*05:01:05	05:01:05	HLA09295		0	0	54	0	0	0	10	64			WD				WD	WD	WD
DQB1*05:01:06	05:01:06	HLA09296		0	0	2	0	0	0	3	5								WD	WD
DQB1*05:01:07	05:01:07	HLA09297		0	0	12	0	0	0	0	12			WD					WD	WD
DQB1*05:01:08	05:01:08	HLA09347		0	0	30	0	0	0	11	41			WD				WD	WD	WD
DQB1*05:01:09	05:01:09	HLA09827		0	2	3	0	0	0	0	5								WD	WD
DQB1*05:01:12	05:01:12	HLA11145		0	9	4	0	0	0	2	15		WD						WD	WD
DQB1*05:01:15	05:01:15	HLA11596		0	0	1	0	0	0	0	1									
DQB1*05:01:16	05:01:16	HLA11679		0	1	1	0	0	0	0	2									
DQB1*05:01:17	05:01:17	HLA11684		0	0	4	0	0	0	0	4									
**DQB1*05:02 total**	**05:02 total**			**10716**	**64280**	**352764**	**22320**	**10427**	**1070**	**29445**	**491022**	**C**	**C**	**C**	**C**	**C**	**C**	**C**	**C**	**C**
DQB1*05:02	05:02			659	1893	28653	1679	344	72	1443	34743	C	C	C	C	C	C	C	C	C
DQB1*05:02P	05:02P			0	0	9	0	0	0	0	9			WD					WD	WD
**DQB1*05:02:01G total**	**05:02:01G total**			**10056**	**62305**	**324086**	**20641**	**10083**	**998**	**27998**	**456167**	**C**	**C**	**C**	**C**	**C**	**C**	**C**	**C**	**C**
DQB1*05:02:01G	05:02:01G		05:02:01G	5698	40810	93432	6328	6072	620	20133	173093	C	C	C	C	C	C	C	C	C
DQB1*05:02:01	05:02:01		05:02:01G	4357	21492	230381	14300	4010	378	7861	282779	C	C	C	C	C	C	C	C	C
DQB1*05:02:01:01	05:02:01:01	HLA00639	05:02:01G	0	0	11	0	0	0	0	11			WD					WD	WD
DQB1*05:02:07	05:02:07	HLA09843	05:02:01G	1	0	165	8	1	0	3	178			I	WD				I	I
DQB1*05:02:11	05:02:11	HLA12699	05:02:01G	0	0	25	0	0	0	0	25			WD					WD	WD
DQB1*05:14	05:14	HLA07330	05:02:01G	0	0	59	1	0	0	0	60			WD					WD	WD
DQB1*05:35	05:35	HLA09838	05:02:01G	0	1	2	2	0	0	0	5								WD	WD
DQB1*05:37	05:37	HLA09841	05:02:01G	0	2	0	0	0	0	0	2									
DQB1*05:46	05:46	HLA10238	05:02:01G	0	0	2	0	0	0	0	2									
DQB1*05:57	05:57	HLA11080	05:02:01G	0	0	2	0	0	0	0	2									
DQB1*05:102	05:102	HLA13915	05:02:01G	0	0	2	2	0	0	0	4									
DQB1*05:106	05:106	HLA13937	05:02:01G	0	0	5	0	0	0	1	6			WD					WD	WD
DQB1*05:02:02	05:02:02	HLA01573		1	80	4	0	0	0	4	89		I						WD	I
DQB1*05:02:04	05:02:04	HLA09348		0	1	7	0	0	0	0	8			WD					WD	WD
DQB1*05:02:06	05:02:06	HLA09832		0	0	1	0	0	0	0	1									
DQB1*05:02:09	05:02:09	HLA11597		0	0	4	0	0	0	0	4									
DQB1*05:02:10	05:02:10	HLA12033		0	1	0	0	0	0	0	1									

Supplemental Table 15: HLA-DQB1 Allele Summary [a]				Allele Count by Population Group [b]								3.0.0 CIWD Category by Population Group [c]								
Allele	Genomic Typing	Allele ID	G group	AFA	API	EURO	MENA	HIS	NAM	UNK	Total	AFA	API	EURO	MENA	HIS	NAM	UNK	Total	Highest Frequency
**DQB1*05:03 total**	**05:03 total**			**6020**	**115198**	**310749**	**19944**	**13227**	**1445**	**36482**	**503065**	**C**	**C**	**C**	**C**	**C**	**C**	**C**	**C**	**C**
DQB1*05:03	05:03			10	144	10072	1460	20	0	253	11959	WD	C	C	C	I		C	C	C
DQB1*05:03P	05:03P			0	1	15	0	0	0	1	17			WD					WD	WD
**DQB1*05:03:01G total**	**05:03:01G total**			**6010**	**114972**	**300607**	**18483**	**13207**	**1445**	**36225**	**490949**	**C**	**C**	**C**	**C**	**C**	**C**	**C**	**C**	**C**
DQB1*05:03:01G	05:03:01G		05:03:01G	3406	90866	105970	6114	8023	971	27857	243207	C	C	C	C	C	C	C	C	C
DQB1*05:03:01	05:03:01		05:03:01G	2602	23898	194525	12360	5184	470	8351	247390	C	C	C	C	C	C	C	C	C
DQB1*05:03:01:01	05:03:01:01	HLA00640	05:03:01G	0	0	18	0	0	0	0	18			WD					WD	WD
DQB1*05:03:01:02	05:03:01:02	HLA06622	05:03:01G	0	0	1	0	0	0	0	1									
DQB1*05:03:03	05:03:03	HLA05769	05:03:01G	0	5	3	0	0	0	0	8		WD						WD	WD
DQB1*05:03:09	05:03:09	HLA09876	05:03:01G	0	0	6	0	0	0	0	6			WD					WD	WD
DQB1*05:08	05:08	HLA05775	05:03:01G	0	14	23	1	0	0	2	40		I	WD					WD	I
DQB1*05:10	05:10	HLA06035	05:03:01G	2	189	10	1	0	4	15	221		C	WD				I	I	C
DQB1*05:78	05:78	HLA12160	05:03:01G	0	0	49	0	0	0	0	49			WD					WD	WD
DQB1*05:96	05:96	HLA13710	05:03:01G	0	0	1	0	0	0	0	1									
DQB1*05:108	05:108	HLA13942	05:03:01G	0	0	1	7	0	0	0	8				WD				WD	WD
DQB1*05:03:02	05:03:02	HLA00641		0	1	25	0	0	0	0	26			WD					WD	WD
DQB1*05:03:05	05:03:05	HLA06823		0	0	1	0	0	0	0	1									
DQB1*05:03:06	05:03:06	HLA09349		0	0	1	0	0	0	0	1									
DQB1*05:03:08	05:03:08	HLA09744		0	47	0	0	0	0	0	47		I						WD	I
DQB1*05:03:11	05:03:11	HLA10553		0	0	11	1	0	0	2	14			WD					WD	WD
DQB1*05:03:12	05:03:12	HLA11146		0	9	0	0	0	0	0	9		WD						WD	WD
DQB1*05:03:13	05:03:13	HLA11148		0	9	0	0	0	0	0	9		WD						WD	WD
DQB1*05:03:14	05:03:14	HLA11594		0	1	1	0	0	0	1	3									
DQB1*05:03:16	05:03:16	HLA11890		0	1	0	0	0	0	0	1									
**DQB1*05:43 total**	**05:43 total**			**0**	**13**	**16**	**0**	**0**	**0**	**0**	**29**		**WD**	**WD**					**WD**	**WD**
DQB1*05:43	05:43			0	12	13	0	0	0	0	25		WD	WD					WD	WD
DQB1*05:43:01	05:43:01	HLA09833		0	1	2	0	0	0	0	3									
DQB1*05:43:02	05:43:02	HLA16144		0	0	1	0	0	0	0	1									
**DQB1*05:04 total**	**05:04 total**			**157**	**269**	**18718**	**520**	**342**	**49**	**1190**	**21245**	**C**	**C**	**C**	**C**	**C**	**C**	**C**	**C**	**C**
**DQB1*05:04:01G total**	**05:04:01G total**			**157**	**269**	**18718**	**520**	**342**	**49**	**1190**	**21245**	**C**	**C**	**C**	**C**	**C**	**C**	**C**	**C**	**C**
DQB1*05:04	05:04	HLA00642	05:04:01G	156	240	18628	519	328	49	1149	21069	C	C	C	C	C	C	C	C	C

Supplemental Table 15: HLA-DQB1 Allele Summary [a]				Allele Count by Population Group [b]								3.0.0 CIWD Category by Population Group [c]								
Allele	Genomic Typing	Allele ID	G group	AFA	API	EURO	MENA	HIS	NAM	UNK	Total	AFA	API	EURO	MENA	HIS	NAM	UNK	Total	Highest Frequency
DQB1*05:04:01G	05:04:01G		05:04:01G	1	29	81	1	14	0	41	167		I	WD		I		I	I	I
DQB1*05:132Q	05:132Q	HLA16014	05:04:01G	0	0	9	0	0	0	0	9			WD					WD	WD
**DQB1*05:05 total**	**05:05 total**			**3**	**0**	**72**	**11**	**0**	**0**	**5**	**91**			**WD**	**WD**			**WD**	**WD**	**WD**
DQB1*05:05	05:05			2	0	18	3	0	0	2	25			WD					WD	WD
DQB1*05:05:01	05:05:01	HLA02689		1	0	54	8	0	0	3	66			WD	WD				WD	WD
**DQB1*05:06 total**	**05:06 total**			**0**	**5**	**0**	**0**	**0**	**0**	**4**	**9**		**WD**						**WD**	**WD**
DQB1*05:06:01	05:06:01	HLA05383		0	3	0	0	0	0	0	3									
DQB1*05:06:02	05:06:02	HLA11592		0	2	0	0	0	0	4	6								WD	WD
DQB1*05:07	05:07	HLA05772		0	0	1	0	0	0	0	1									
DQB1*05:09	05:09	HLA06034		0	3	9	0	0	0	0	12			WD					WD	WD
**DQB1*05:11 total**	**05:11 total**			**0**	**0**	**56**	**1**	**2**	**0**	**4**	**63**			**WD**					**WD**	**WD**
DQB1*05:11	05:11			0	0	8	0	0	0	0	8			WD					WD	WD
DQB1*05:11:01	05:11:01	HLA06287		0	0	5	0	0	0	0	5			WD					WD	WD
DQB1*05:11:02	05:11:02	HLA10288		0	0	43	1	2	0	4	50			WD					WD	WD
DQB1*05:12	05:12	HLA06670		0	0	37	0	0	0	2	39			WD					WD	WD
DQB1*05:15	05:15	HLA07929		0	0	1	0	0	0	0	1									
DQB1*05:16	05:16	HLA08292		0	0	2	1	0	0	0	3									
DQB1*05:19	05:19	HLA09102		3	0	1	0	0	0	0	4									
DQB1*05:20	05:20	HLA09291		0	0	37	0	1	0	3	41			WD					WD	WD
DQB1*05:23	05:23	HLA09351		0	486	2	1	0	0	2	491		C						I	C
DQB1*05:24	05:24	HLA09352		4	0	0	0	0	0	0	4									
DQB1*05:26	05:26	HLA09354		0	8	0	0	1	0	1	10		WD						WD	WD
DQB1*05:28	05:28	HLA09743		0	1	3	0	0	0	0	4									
DQB1*05:29	05:29	HLA09826		0	3	0	0	0	0	0	3									
DQB1*05:34	05:34	HLA09836		0	4	0	0	0	0	1	5								WD	WD
DQB1*05:44	05:44	HLA10112		0	1	2	0	1	0	1	5								WD	WD
DQB1*05:48	05:48	HLA10289		0	0	0	1	0	0	0	1									
DQB1*05:49	05:49	HLA10290		0	0	19	0	0	0	4	23			WD					WD	WD
DQB1*05:51	05:51	HLA10544		0	0	5	0	0	0	2	7			WD					WD	WD
DQB1*05:52	05:52	HLA10549		0	0	46	1	0	0	2	49			WD					WD	WD
DQB1*05:53	05:53	HLA10550		0	0	1	0	0	0	0	1									

Supplemental Table 15: HLA-DQB1 Allele Summary [a]				Allele Count by Population Group [b]								3.0.0 CIWD Category by Population Group [c]								
Allele	Genomic Typing	Allele ID	G group	AFA	API	EURO	MENA	HIS	NAM	UNK	Total	AFA	API	EURO	MENA	HIS	NAM	UNK	Total	Highest Frequency
DQB1*05:54	05:54	HLA10551		0	0	1	0	0	0	0	1									
DQB1*05:55	05:55	HLA10552		0	0	1	0	0	0	0	1									
DQB1*05:58	05:58	HLA11147		5	0	0	0	0	0	0	5	WD							WD	WD
DQB1*05:59	05:59	HLA11149		0	1	0	0	0	0	0	1									
DQB1*05:60	05:60	HLA11557		0	0	7	0	0	0	3	10			WD					WD	WD
DQB1*05:62	05:62	HLA11157		0	0	1	0	0	0	0	1									
DQB1*05:64	05:64	HLA11284		0	1	0	0	0	0	0	1									
**DQB1*05:66 total**	**05:66 total**			**0**	**3**	**0**	**1**	**0**	**0**	**0**	**4**									
DQB1*05:66	05:66			0	1	0	0	0	0	0	1									
DQB1*05:66:01	05:66:01	HLA11359		0	2	0	0	0	0	0	2									
DQB1*05:66:02	05:66:02	HLA14215		0	0	0	1	0	0	0	1									
DQB1*05:67	05:67	HLA11361		0	10	1	1	3	1	4	20		WD						WD	WD
DQB1*05:69	05:69	HLA11595		0	0	14	0	0	0	0	14			WD					WD	WD
DQB1*05:71	05:71	HLA11681		2	0	0	0	0	0	0	2									
DQB1*05:72	05:72	HLA11682		0	0	0	0	3	0	0	3									
DQB1*05:73	05:73	HLA11683		0	0	0	0	1	0	0	1									
DQB1*05:79	05:79	HLA12204		0	0	7	1	0	1	0	9			WD					WD	WD
DQB1*05:80	05:80	HLA12212		3	2	0	1	0	0	1	7								WD	WD
DQB1*05:83	05:83	HLA12358		0	0	3	0	0	0	0	3									
DQB1*05:88	05:88	HLA12801		5	0	0	0	0	0	0	5	WD							WD	WD
**DQB1*05:89 total**	**05:89 total**			**0**	**0**	**0**	**0**	**0**	**0**	**2**	**2**									
DQB1*05:89:02	05:89:02	HLA14347		0	0	0	0	0	0	2	2									
DQB1*05:91	05:91	HLA13143		0	2	0	0	0	0	0	2									
DQB1*05:92	05:92	HLA13144		0	0	4	0	0	0	0	4									
DQB1*05:93	05:93	HLA13577		0	0	2	0	0	0	0	2									
DQB1*05:94	05:94	HLA13582		0	2	0	0	0	0	0	2									
DQB1*05:95	05:95	HLA13683		0	1	0	0	0	0	0	1									
DQB1*05:97	05:97	HLA13811		0	0	2	0	0	0	0	2									
DQB1*05:98	05:98	HLA13812		0	0	1	0	0	0	0	1									
DQB1*05:101	05:101	HLA13885		0	4	0	0	0	0	0	4									
DQB1*05:105	05:105	HLA13935		0	0	2	0	0	0	0	2									

Supplemental Table 15: HLA-DQB1 Allele Summary [a]				Allele Count by Population Group [b]								3.0.0 CIWD Category by Population Group [c]								
Allele	Genomic Typing	Allele ID	G group	AFA	API	EURO	MENA	HIS	NAM	UNK	Total	AFA	API	EURO	MENA	HIS	NAM	UNK	Total	Highest Frequency
DQB1*05:111	05:111	HLA14349		1	0	0	0	0	0	0	1									
DQB1*05:113	05:113	HLA14551		0	1	0	0	0	0	0	1									
DQB1*05:115	05:115	HLA14553		0	1	0	0	0	0	0	1									
DQB1*05:116	05:116	HLA14554		0	2	0	0	0	0	0	2									
DQB1*05:119	05:119	HLA14557		0	0	1	0	0	0	0	1									
DQB1*05:120	05:120	HLA15317		0	1	0	0	0	0	0	1									
DQB1*05:135	05:135	HLA16142		0	0	2	0	0	0	0	2									
DQB1*05:140	05:140	HLA16566		0	1	0	0	0	0	0	1									
DQB1*05:158	05:158	HLA17917		0	4	0	0	0	0	0	4									
DQB1*05:CODE	05:CODE			2828	3532	41466	944	2537	136	4809	56252	NA	NA	NA	NA	NA	NA	NA	NA	NA
**DQB1*06:01 total**	**06:01 total**			**1045**	**203718**	**98834**	**15933**	**7633**	**955**	**22633**	**350751**	**C**	**C**	**C**	**C**	**C**	**C**	**C**	**C**	**C**
DQB1*06:01	06:01			576	41387	55148	6196	4048	546	7452	115353	C	C	C	C	C	C	C	C	C
DQB1*06:01P	06:01P			0	0	1	0	0	0	1	2									
**DQB1*06:01:01G total**	**06:01:01G total**			**469**	**162216**	**43676**	**9730**	**3585**	**409**	**15175**	**235260**	**C**	**C**	**C**	**C**	**C**	**C**	**C**	**C**	**C**
DQB1*06:01:01G	06:01:01G		06:01:01G	318	153769	20048	5358	3231	367	14100	197191	C	C	C	C	C	C	C	C	C
DQB1*06:01:01	06:01:01		06:01:01G	129	8300	22698	4323	342	41	925	36758	C	C	C	C	C	C	C	C	C
DQB1*06:01:03	06:01:03	HLA00645	06:01:01G	22	5	909	47	12	1	51	1047	WD	WD	I	C	I		I	I	C
DQB1*06:01:05	06:01:05	HLA03991	06:01:01G	0	140	16	0	0	0	95	251		C	WD				I	I	C
DQB1*06:01:06	06:01:06	HLA05776	06:01:01G	0	1	0	0	0	0	1	2									
DQB1*06:01:10	06:01:10	HLA09871	06:01:01G	0	0	2	0	0	0	1	3									
DQB1*06:103	06:103	HLA09870	06:01:01G	0	0	1	2	0	0	2	5								WD	WD
DQB1*06:105	06:105	HLA09873	06:01:01G	0	1	1	0	0	0	0	2									
DQB1*06:108	06:108	HLA09929	06:01:01G	0	0	1	0	0	0	0	1									
DQB1*06:01:04	06:01:04	HLA03307		0	2	1	0	0	0	0	3									
DQB1*06:01:08	06:01:08	HLA09224		0	107	7	7	0	0	5	126		I	WD	WD			WD	WD	I
DQB1*06:01:12	06:01:12	HLA11125		0	1	1	0	0	0	0	2									
DQB1*06:01:14	06:01:14	HLA12431		0	5	0	0	0	0	0	5		WD						WD	WD
**DQB1*06:02 total**	**06:02 total**			**65894**	**34541**	**1420381**	**19093**	**47738**	**5430**	**124187**	**1717264**	**C**	**C**	**C**	**C**	**C**	**C**	**C**	**C**	**C**
DQB1*06:02	06:02			575	187	22253	564	307	19	929	24834	C	C	C	C	C	C	C	C	C
DQB1*06:02P	06:02P			0	0	9	0	0	0	7	16			WD				WD	WD	WD
**DQB1*06:02:01G total**	**06:02:01G total**			**65316**	**34294**	**1397572**	**18471**	**47422**	**5408**	**123199**	**1691682**	**C**	**C**	**C**	**C**	**C**	**C**	**C**	**C**	**C**

Supplemental Table 15: HLA-DQB1 Allele Summary [a]				Allele Count by Population Group [b]								3.0.0 CIWD Category by Population Group [c]								
Allele	Genomic Typing	Allele ID	G group	AFA	API	EURO	MENA	HIS	NAM	UNK	Total	AFA	API	EURO	MENA	HIS	NAM	UNK	Total	Highest Frequency
DQB1*06:02:01G	06:02:01G		06:02:01G	34391	21388	327237	6984	26772	3009	83845	503626	C	C	C	C	C	C	C	C	C
DQB1*06:02:01	06:02:01		06:02:01G	30386	12729	1065569	11434	19968	2350	38724	1181160	C	C	C	C	C	C	C	C	C
DQB1*06:02:01:01	06:02:01:01	HLA00646	06:02:01G	515	168	4244	40	617	46	600	6230	C	C	C	WD	C	C	C	C	C
DQB1*06:02:01:03	06:02:01:03	HLA16150	06:02:01G	12	1	12	0	3	1	5	34	WD		WD				WD	WD	WD
DQB1*06:02:12	06:02:12	HLA09932	06:02:01G	0	0	1	0	0	0	0	1									
DQB1*06:02:23	06:02:23	HLA12703	06:02:01G	0	1	2	6	2	1	1	13				WD				WD	WD
DQB1*06:02:26	06:02:26	HLA16155	06:02:01G	0	0	17	0	0	0	0	17			WD					WD	WD
DQB1*06:02:27	06:02:27	HLA17201	06:02:01G	0	0	1	0	0	0	0	1									
DQB1*06:47	06:47	HLA07414	06:02:01G	0	0	9	0	0	0	1	10			WD					WD	WD
DQB1*06:84	06:84	HLA09472	06:02:01G	4	6	343	7	2	0	10	372		WD	I	WD			WD	I	I
DQB1*06:109	06:109	HLA09930	06:02:01G	1	0	7	0	7	0	0	15			WD		I			WD	I
DQB1*06:111	06:111	HLA10167	06:02:01G	1	1	20	0	0	0	0	22			WD					WD	WD
DQB1*06:116	06:116	HLA10246	06:02:01G	0	0	1	0	0	0	0	1									
DQB1*06:117	06:117	HLA10247	06:02:01G	0	0	63	0	0	0	1	64			WD					WD	WD
DQB1*06:127	06:127	HLA10506	06:02:01G	6	0	12	0	51	1	6	76	WD		WD		I		WD	WD	I
DQB1*06:188	06:188	HLA13932	06:02:01G	0	0	16	0	0	0	5	21			WD				WD	WD	WD
DQB1*06:219	06:219	HLA16134	06:02:01G	0	0	6	0	0	0	1	7			WD					WD	WD
DQB1*06:224	06:224	HLA16165	06:02:01G	0	0	4	0	0	0	0	4									
DQB1*06:225	06:225	HLA16166	06:02:01G	0	0	1	0	0	0	0	1									
DQB1*06:226	06:226	HLA16183	06:02:01G	0	0	3	0	0	0	0	3									
DQB1*06:227	06:227	HLA16184	06:02:01G	0	0	1	0	0	0	0	1									
DQB1*06:237	06:237	HLA17291	06:02:01G	0	0	1	0	0	0	0	1									
DQB1*06:240	06:240	HLA17429	06:02:01G	0	0	2	0	0	0	0	2									
DQB1*06:02:02	06:02:02	HLA03156		0	55	476	53	6	2	40	632		I	I	C	WD		I	I	C
DQB1*06:02:03	06:02:03	HLA09366		1	0	1	0	0	0	0	2									
DQB1*06:02:04	06:02:04	HLA09370		1	0	11	0	0	0	0	12			WD					WD	WD
DQB1*06:02:05	06:02:05	HLA09378		0	1	4	0	0	1	0	6								WD	WD
DQB1*06:02:06	06:02:06	HLA09381		0	0	8	0	0	0	1	9			WD					WD	WD
DQB1*06:02:07	06:02:07	HLA09746		0	0	17	0	1	0	3	21			WD					WD	WD
DQB1*06:02:08	06:02:08	HLA09749		0	0	0	5	0	0	0	5				WD				WD	WD
DQB1*06:02:09	06:02:09	HLA09750		1	0	1	0	1	0	1	4									

Supplemental Table 15: HLA-DQB1 Allele Summary [a]				Allele Count by Population Group [b]								3.0.0 CIWD Category by Population Group [c]								
Allele	Genomic Typing	Allele ID	G group	AFA	API	EURO	MENA	HIS	NAM	UNK	Total	AFA	API	EURO	MENA	HIS	NAM	UNK	Total	Highest Frequency
DQB1*06:02:11	06:02:11	HLA09925		0	2	0	0	0	0	0	2									
DQB1*06:02:14	06:02:14	HLA10299		0	0	5	0	0	0	5	10			WD				WD	WD	WD
DQB1*06:02:15	06:02:15	HLA10659		0	1	5	0	0	0	0	6			WD					WD	WD
DQB1*06:02:16	06:02:16	HLA11154		0	1	9	0	1	0	2	13			WD					WD	WD
DQB1*06:02:17	06:02:17	HLA11366		0	0	6	0	0	0	0	6			WD					WD	WD
DQB1*06:02:20	06:02:20	HLA11893		0	0	2	0	0	0	0	2									
DQB1*06:02:24	06:02:24	HLA13149		0	0	1	0	0	0	0	1									
DQB1*06:02:25	06:02:25	HLA13923		0	0	1	0	0	0	0	1									
**DQB1*06:03 total**	**06:03 total**			**9807**	**70202**	**820986**	**23070**	**30079**	**3033**	**71102**	**1028279**	**C**	**C**	**C**	**C**	**C**	**C**	**C**	**C**	**C**
DQB1*06:03	06:03			12	71	11410	644	23	2	288	12450	WD	I	C	C	I		C	C	C
DQB1*06:03P	06:03P			0	0	10	0	0	0	1	11			WD					WD	WD
**DQB1*06:03:01G total**	**06:03:01G total**			**9794**	**70124**	**808947**	**22419**	**30044**	**3031**	**70795**	**1015154**	**C**	**C**	**C**	**C**	**C**	**C**	**C**	**C**	**C**
DQB1*06:03:01G	06:03:01G		06:03:01G	5453	52611	232934	9279	17999	1881	55543	375700	C	C	C	C	C	C	C	C	C
DQB1*06:03:01	06:03:01		06:03:01G	4331	17127	575575	13100	12040	1149	15222	638544	C	C	C	C	C	C	C	C	C
DQB1*06:03:01:01	06:03:01:01	HLA00647	06:03:01G	0	0	0	0	0	0	1	1									
DQB1*06:03:21	06:03:21	HLA13922	06:03:01G	0	0	3	16	0	0	0	19				WD				WD	WD
DQB1*06:03:22	06:03:22	HLA14471	06:03:01G	0	0	3	0	0	0	0	3									
DQB1*06:03:23	06:03:23	HLA16126	06:03:01G	0	0	18	0	0	0	0	18			WD					WD	WD
DQB1*06:03:24	06:03:24	HLA16135	06:03:01G	0	0	1	0	0	0	0	1									
DQB1*06:41	06:41	HLA06179	06:03:01G	9	154	198	16	3	1	16	397	WD	C	I	WD			I	I	C
DQB1*06:44	06:44	HLA06518	06:03:01G	0	4	170	0	1	0	2	177			I					I	I
DQB1*06:110	06:110	HLA10113	06:03:01G	1	228	9	1	0	0	9	248		C	WD				WD	I	C
DQB1*06:185	06:185	HLA13916	06:03:01G	0	0	10	0	0	0	1	11			WD					WD	WD
DQB1*06:187	06:187	HLA13927	06:03:01G	0	0	20	7	1	0	1	29			WD	WD				WD	WD
DQB1*06:223	06:223	HLA16164	06:03:01G	0	0	6	0	0	0	0	6			WD					WD	WD
DQB1*06:03:02	06:03:02	HLA02638		0	0	548	0	6	0	15	569			I		WD		I	I	I
DQB1*06:03:04	06:03:04	HLA09269		1	3	11	1	0	0	0	16			WD					WD	WD
DQB1*06:03:05	06:03:05	HLA09272		0	0	0	0	6	0	1	7					WD			WD	WD
DQB1*06:03:06	06:03:06	HLA09274		0	0	3	0	0	0	0	3									
DQB1*06:03:07	06:03:07	HLA09278		0	2	16	2	0	0	2	22			WD					WD	WD
DQB1*06:03:08	06:03:08	HLA09736		0	0	1	0	0	0	0	1									

Supplemental Table 15: HLA-DQB1 Allele Summary [a]				Allele Count by Population Group [b]								3.0.0 CIWD Category by Population Group [c]								
Allele	Genomic Typing	Allele ID	G group	AFA	API	EURO	MENA	HIS	NAM	UNK	Total	AFA	API	EURO	MENA	HIS	NAM	UNK	Total	Highest Frequency
DQB1*06:03:09	06:03:09	HLA09738		0	0	17	0	0	0	0	17			WD					WD	WD
DQB1*06:03:11	06:03:11	HLA10545		0	0	5	1	0	0	0	6			WD					WD	WD
DQB1*06:03:12	06:03:12	HLA11136		0	0	4	0	0	0	0	4									
DQB1*06:03:13	06:03:13	HLA11137		0	0	5	0	0	0	0	5			WD					WD	WD
DQB1*06:03:14	06:03:14	HLA11138		0	0	1	0	0	0	0	1									
DQB1*06:03:15	06:03:15	HLA11355		0	0	5	3	0	0	0	8			WD					WD	WD
DQB1*06:03:18	06:03:18	HLA12799		0	1	1	0	0	0	0	2									
DQB1*06:03:19	06:03:19	HLA13132		0	1	0	0	0	0	0	1									
DQB1*06:03:26	06:03:26	HLA16188		0	0	2	0	0	0	0	2									
**DQB1*06:04 total**	**06:04 total**			**8255**	**17616**	**367336**	**13049**	**16447**	**1679**	**40890**	**465272**	**C**	**C**	**C**	**C**	**C**	**C**	**C**	**C**	**C**
DQB1*06:04	06:04			104	222	16870	442	63	6	281	17988	C	C	C	C	I	WD	C	C	C
DQB1*06:04P	06:04P			0	0	4	0	0	0	3	7								WD	WD
**DQB1*06:04:01G total**	**06:04:01G total**			**8151**	**17393**	**350216**	**12605**	**16383**	**1673**	**40595**	**447016**	**C**	**C**	**C**	**C**	**C**	**C**	**C**	**C**	**C**
DQB1*06:04:01G	06:04:01G		06:04:01G	5009	13202	152811	6452	10152	1069	33335	222030	C	C	C	C	C	C	C	C	C
DQB1*06:04:01	06:04:01	HLA00648	06:04:01G	3118	4186	196007	6141	6220	604	7245	223521	C	C	C	C	C	C	C	C	C
DQB1*06:34	06:34	HLA03114	06:04:01G	0	0	23	0	0	0	0	23			WD					WD	WD
DQB1*06:36	06:36	HLA03979	06:04:01G	0	1	114	2	0	0	2	119			WD					WD	WD
DQB1*06:38	06:38	HLA04357	06:04:01G	0	0	8	0	0	0	0	8			WD					WD	WD
DQB1*06:39	06:39	HLA04358	06:04:01G	24	4	1241	9	6	0	13	1297	WD		C	WD	WD		I	I	C
DQB1*06:52	06:52	HLA08082	06:04:01G	0	0	7	0	5	0	0	12			WD		WD			WD	WD
DQB1*06:217	06:217	HLA16016	06:04:01G	0	0	5	1	0	0	0	6			WD					WD	WD
DQB1*06:04:02	06:04:02	HLA01005		0	1	117	2	1	0	9	130			WD				WD	WD	WD
DQB1*06:04:03	06:04:03	HLA02602		0	0	86	0	0	0	0	86			WD					WD	WD
DQB1*06:04:05	06:04:05	HLA09244		0	0	1	0	0	0	0	1									
DQB1*06:04:06	06:04:06	HLA09270		0	0	37	0	0	0	2	39			WD					WD	WD
DQB1*06:04:08	06:04:08	HLA09936		0	0	1	0	0	0	0	1									
DQB1*06:04:09	06:04:09	HLA11589		0	0	4	0	0	0	0	4									
**DQB1*06:05 total**	**06:05 total**			**0**	**0**	**11**	**0**	**1**	**0**	**1**	**13**			**WD**					**WD**	**WD**
DQB1*06:05	06:05			0	0	11	0	1	0	0	12			WD					WD	WD
DQB1*06:05:01	06:05:01	HLA00649		0	0	0	0	0	0	1	1									
**DQB1*06:07 total**	**06:07 total**			**1**	**6**	**506**	**7**	**6**	**0**	**29**	**555**		**WD**	**I**	**WD**	**WD**		**I**	**I**	**I**

Supplemental Table 15: HLA-DQB1 Allele Summary [a]				Allele Count by Population Group [b]								3.0.0 CIWD Category by Population Group [c]								
Allele	Genomic Typing	Allele ID	G group	AFA	API	EURO	MENA	HIS	NAM	UNK	Total	AFA	API	EURO	MENA	HIS	NAM	UNK	Total	Highest Frequency
DQB1*06:07	06:07			0	0	36	0	1	0	2	39			WD					WD	WD
DQB1*06:07:01	06:07:01	HLA00652		1	2	378	5	4	0	24	414			I	WD			I	I	I
DQB1*06:07:02	06:07:02	HLA05768		0	4	92	2	1	0	3	102			WD					WD	WD
**DQB1*06:08 total**	**06:08 total**			**1340**	**6**	**69**	**33**	**84**	**20**	**267**	**1819**	**C**	**WD**	**WD**	**WD**	**C**	**C**	**C**	**C**	**C**
DQB1*06:08	06:08			100	1	4	2	12	2	22	143	C				I		I	WD	C
DQB1*06:08:01	06:08:01	HLA00653		1240	4	39	31	72	18	245	1649	C		WD	WD	C	C	C	C	C
DQB1*06:08:02	06:08:02	HLA02609		0	1	26	0	0	0	0	27			WD					WD	WD
**DQB1*06:09 total**	**06:09 total**			**13515**	**25154**	**73514**	**3654**	**6225**	**704**	**14022**	**136788**	**C**	**C**	**C**	**C**	**C**	**C**	**C**	**C**	**C**
DQB1*06:09	06:09			2700	2699	12348	438	999	155	1763	21102	C	C	C	C	C	C	C	C	C
DQB1*06:09P	06:09P			0	0	1	0	0	0	0	1									
**DQB1*06:09:01G total**	**06:09:01G total**			**10813**	**22455**	**61130**	**3214**	**5220**	**549**	**12244**	**115625**	**C**	**C**	**C**	**C**	**C**	**C**	**C**	**C**	**C**
DQB1*06:09:01G	06:09:01G		06:09:01G	5863	17356	23227	2118	2836	319	8817	60536	C	C	C	C	C	C	C	C	C
DQB1*06:09:01	06:09:01		06:09:01G	4794	5033	37499	1087	2292	225	3353	54283	C	C	C	C	C	C	C	C	C
DQB1*06:09:01:01	06:09:01:01	HLA00654	06:09:01G	124	65	326	7	75	5	64	666	C	I	I	WD	C	WD	I	I	C
DQB1*06:09:01:02	06:09:01:02	HLA16360	06:09:01G	5	0	19	0	12	0	2	38	WD		WD		I			WD	I
DQB1*06:88	06:88	HLA09651	06:09:01G	27	1	59	2	5	0	8	102	WD		WD		WD		WD	WD	WD
DQB1*06:09:02	06:09:02	HLA09275		2	0	27	0	0	0	6	35			WD				WD	WD	WD
DQB1*06:09:03	06:09:03	HLA09277		0	0	2	0	5	0	6	13					WD		WD	WD	WD
DQB1*06:09:04	06:09:04	HLA09937		0	0	3	0	0	0	1	4									
DQB1*06:09:05	06:09:05	HLA11140		0	0	3	2	0	0	0	5								WD	WD
DQB1*06:09:06	06:09:06	HLA11677		0	0	0	0	1	0	1	2									
DQB1*06:09:07	06:09:07	HLA17170		0	0	0	0	0	0	1	1									
DQB1*06:10	06:10	HLA00655		0	459	6	0	2	0	35	502		C	WD				I	I	C
**DQB1*06:11 total**	**06:11 total**			**392**	**38**	**363**	**17**	**70**	**10**	**117**	**1007**	**C**	**I**	**I**	**WD**	**C**	**C**	**I**	**I**	**C**
DQB1*06:11	06:11			31	1	28	0	17	0	6	83	WD		WD		I		WD	WD	I
DQB1*06:11:01	06:11:01	HLA00656		360	35	256	17	52	10	103	833	C	I	I	WD	I	C	I	I	C
DQB1*06:11:02	06:11:02	HLA00657		0	2	79	0	1	0	8	90			WD				WD	WD	WD
DQB1*06:11:03	06:11:03	HLA11508		1	0	0	0	0	0	0	1									
DQB1*06:12	06:12	HLA00658		1	5	7	0	2	0	0	15		WD	WD					WD	WD
**DQB1*06:13 total**	**06:13 total**			**5**	**1**	**277**	**2**	**1**	**0**	**8**	**294**	**WD**		**I**				**WD**	**I**	**I**
DQB1*06:13	06:13			2	0	50	0	0	0	2	54			WD					WD	WD

Supplemental Table 15: HLA-DQB1 Allele Summary [a]				Allele Count by Population Group [b]								3.0.0 CIWD Category by Population Group [c]								
Allele	Genomic Typing	Allele ID	G group	AFA	API	EURO	MENA	HIS	NAM	UNK	Total	AFA	API	EURO	MENA	HIS	NAM	UNK	Total	Highest Frequency
DQB1*06:13:01	06:13:01	HLA00659		2	1	225	2	1	0	6	237			I				WD	I	I
DQB1*06:13:02	06:13:02	HLA09377		1	0	2	0	0	0	0	3									
**DQB1*06:14 total**	**06:14 total**			**9**	**5**	**521**	**14**	**11**	**2**	**110**	**672**	**WD**	**WD**	**I**	**WD**	**I**		**I**	**I**	**I**
DQB1*06:14	06:14			1	0	40	0	2	1	1	45			WD					WD	WD
DQB1*06:14:01	06:14:01	HLA00660		7	4	336	3	4	1	71	426	WD		I				I	I	I
DQB1*06:14:02	06:14:02	HLA03431		1	1	145	11	5	0	38	201			I	WD	WD		I	I	I
**DQB1*06:15 total**	**06:15 total**			**0**	**2**	**97**	**0**	**0**	**0**	**1**	**100**			**WD**					**WD**	**WD**
DQB1*06:15	06:15			0	1	13	0	0	0	0	14			WD					WD	WD
DQB1*06:15:01	06:15:01	HLA00661		0	1	24	0	0	0	1	26			WD					WD	WD
DQB1*06:15:02	06:15:02	HLA09368		0	0	60	0	0	0	0	60			WD					WD	WD
DQB1*06:16	06:16	HLA01006		8	0	442	0	11	1	51	513	WD		I		I		I	I	I
DQB1*06:17	06:17	HLA01175		0	0	4	0	0	0	0	4									
**DQB1*06:18 total**	**06:18 total**			**7**	**2**	**10**	**2**	**0**	**0**	**4**	**25**	**WD**		**WD**					**WD**	**WD**
DQB1*06:18	06:18			1	1	1	2	0	0	1	6								WD	WD
DQB1*06:18:01	06:18:01	HLA01446		6	1	9	0	0	0	3	19	WD		WD					WD	WD
**DQB1*06:19 total**	**06:19 total**			**0**	**1**	**2**	**0**	**0**	**0**	**1**	**4**									
DQB1*06:19	06:19			0	0	2	0	0	0	0	2									
DQB1*06:19:01	06:19:01	HLA01460		0	1	0	0	0	0	1	2									
DQB1*06:20	06:20	HLA01612		7	0	51	0	0	0	3	61	WD		WD					WD	WD
DQB1*06:21	06:21	HLA01776		1	2	154	1	1	0	2	161			I					I	I
**DQB1*06:22 total**	**06:22 total**			**11**	**2**	**11**	**2**	**1**	**0**	**7**	**34**	**WD**		**WD**				**WD**	**WD**	**WD**
DQB1*06:22	06:22			4	0	4	0	0	0	0	8								WD	WD
DQB1*06:22:01	06:22:01	HLA01967		4	0	2	1	1	0	2	10								WD	WD
DQB1*06:22:02	06:22:02	HLA09476		3	1	1	0	0	0	4	9								WD	WD
DQB1*06:22:03	06:22:03	HLA11600		0	1	4	1	0	0	1	7								WD	WD
DQB1*06:23	06:23	HLA02005		0	0	34	0	1	0	0	35			WD					WD	WD
DQB1*06:24	06:24	HLA02182		0	0	2	0	0	0	0	2									
DQB1*06:25	06:25	HLA02195		0	0	2	1	0	0	0	3									
DQB1*06:26N	06:26N	HLA02221		5	1	52	0	1	0	15	74	WD		WD				I	WD	I
**DQB1*06:27 total**	**06:27 total**			**1**	**1**	**59**	**0**	**1**	**0**	**5**	**67**			**WD**				**WD**	**WD**	**WD**
DQB1*06:27	06:27			1	0	21	0	1	0	0	23			WD					WD	WD

Supplemental Table 15: HLA-DQB1 Allele Summary [a]				Allele Count by Population Group [b]								3.0.0 CIWD Category by Population Group [c]								
Allele	Genomic Typing	Allele ID	G group	AFA	API	EURO	MENA	HIS	NAM	UNK	Total	AFA	API	EURO	MENA	HIS	NAM	UNK	Total	Highest Frequency
DQB1*06:27:01	06:27:01	HLA02291		0	0	37	0	0	0	5	42			WD				WD	WD	WD
DQB1*06:27:02	06:27:02	HLA10281		0	1	1	0	0	0	0	2									
DQB1*06:28	06:28	HLA02453		0	0	67	0	0	0	2	69			WD					WD	WD
DQB1*06:29	06:29	HLA02696		0	0	6	0	0	0	0	6			WD					WD	WD
DQB1*06:30	06:30	HLA02740		0	0	3	1	0	0	0	4									
DQB1*06:31	06:31	HLA02917		0	1	7	13	0	0	0	21			WD	WD				WD	WD
**DQB1*06:32 total**	**06:32 total**			**1**	**0**	**36**	**0**	**0**	**0**	**2**	**39**			**WD**					**WD**	**WD**
DQB1*06:32	06:32			1	0	25	0	0	0	2	28			WD					WD	WD
DQB1*06:32:01	06:32:01	HLA02950		0	0	11	0	0	0	0	11			WD					WD	WD
DQB1*06:33	06:33	HLA02970		7	1	126	0	2	1	29	166	WD		I				I	I	I
DQB1*06:37	06:37	HLA04119		0	0	0	1	0	0	0	1									
DQB1*06:40	06:40	HLA04737		0	0	5	0	0	0	0	5			WD					WD	WD
DQB1*06:46	06:46	HLA06877		12	0	16	0	0	1	1	30	WD		WD					WD	WD
DQB1*06:48	06:48	HLA07539		1	543	18	2	1	0	10	575		C	WD				WD	I	C
DQB1*06:49	06:49	HLA07820		76	0	1	0	1	0	8	86	C						WD	WD	C
DQB1*06:50	06:50	HLA07922		32	0	0	0	0	0	4	36	WD							WD	WD
**DQB1*06:53 total**	**06:53 total**			**0**	**8**	**23**	**0**	**0**	**0**	**0**	**31**		**WD**	**WD**					**WD**	**WD**
DQB1*06:53	06:53			0	1	19	0	0	0	0	20			WD					WD	WD
DQB1*06:53:01	06:53:01	HLA09222		0	0	4	0	0	0	0	4									
DQB1*06:53:02	06:53:02	HLA15318		0	7	0	0	0	0	0	7		WD						WD	WD
DQB1*06:55	06:55	HLA09225		0	37	5	0	0	0	0	42		I	WD					WD	I
DQB1*06:56	06:56	HLA09226		0	1	0	0	0	0	0	1									
DQB1*06:57	06:57	HLA09227		0	85	0	0	0	0	0	85		I						WD	I
DQB1*06:58	06:58	HLA09229		0	0	15	0	0	0	0	15			WD					WD	WD
DQB1*06:59	06:59	HLA09264		0	0	1	0	0	0	0	1									
DQB1*06:60	06:60	HLA09265		0	0	0	0	1	0	0	1									
DQB1*06:62	06:62	HLA09267		0	0	5	0	0	0	0	5			WD					WD	WD
DQB1*06:63	06:63	HLA09268		0	0	3	0	0	0	0	3									
DQB1*06:64	06:64	HLA09271		0	0	3	0	0	0	0	3									
DQB1*06:68	06:68	HLA09358		0	0	9	0	1	0	2	12			WD					WD	WD
**DQB1*06:69 total**	**06:69 total**			**1**	**0**	**6**	**0**	**2**	**0**	**1**	**10**			**WD**					**WD**	**WD**

Supplemental Table 15: HLA-DQB1 Allele Summary [a]				Allele Count by Population Group [b]								3.0.0 CIWD Category by Population Group [c]								
Allele	Genomic Typing	Allele ID	G group	AFA	API	EURO	MENA	HIS	NAM	UNK	Total	AFA	API	EURO	MENA	HIS	NAM	UNK	Total	Highest Frequency
DQB1*06:69	06:69			1	0	6	0	2	0	1	10			WD					WD	WD
DQB1*06:70	06:70	HLA09365		0	0	2	0	0	0	1	3									
DQB1*06:71	06:71	HLA09367		0	0	3	0	0	0	0	3									
DQB1*06:73	06:73	HLA09371		1	0	20	0	0	0	0	21			WD					WD	WD
DQB1*06:74	06:74	HLA09372		0	40	3	0	0	0	15	58		I					I	WD	I
DQB1*06:75N	06:75N	HLA09373		0	0	23	0	0	0	0	23			WD					WD	WD
DQB1*06:76	06:76	HLA09374		0	0	1	0	0	0	0	1									
DQB1*06:77N	06:77N	HLA09375		0	18	0	0	0	0	0	18		I						WD	I
DQB1*06:78	06:78	HLA09376		0	0	5	0	0	0	2	7			WD					WD	WD
**DQB1*06:79 total**	**06:79 total**			**3**	**0**	**29**	**0**	**0**	**0**	**2**	**34**			**WD**					**WD**	**WD**
DQB1*06:79	06:79			2	0	6	0	0	0	0	8			WD					WD	WD
DQB1*06:79:01	06:79:01	HLA09379		1	0	22	0	0	0	2	25			WD					WD	WD
DQB1*06:79:02	06:79:02	HLA11498		0	0	1	0	0	0	0	1									
DQB1*06:80	06:80	HLA09380		0	0	1	0	0	0	0	1									
DQB1*06:81	06:81	HLA09382		0	1	53	0	0	0	27	81			WD				I	WD	I
DQB1*06:82	06:82	HLA09383		1	0	8	0	0	0	0	9			WD					WD	WD
DQB1*06:83	06:83	HLA09384		0	0	1	0	0	0	1	2									
DQB1*06:85	06:85	HLA09633		8	0	1	3	0	0	0	12	WD							WD	WD
DQB1*06:90	06:90	HLA09733		0	0	28	0	0	0	0	28			WD					WD	WD
DQB1*06:91	06:91	HLA09734		0	5	0	0	0	0	0	5		WD						WD	WD
DQB1*06:92	06:92	HLA09735		0	20	2	2	0	0	1	25		I						WD	I
DQB1*06:93	06:93	HLA09737		0	0	4	0	0	0	3	7								WD	WD
DQB1*06:94	06:94	HLA09740		0	0	1	0	0	0	0	1									
DQB1*06:95	06:95	HLA09747		1	0	0	0	0	0	0	1									
DQB1*06:97	06:97	HLA09751		0	0	2	0	0	0	0	2									
**DQB1*06:99 total**	**06:99 total**			**0**	**3**	**0**	**0**	**0**	**0**	**0**	**3**									
DQB1*06:99:02	06:99:02	HLA11561		0	3	0	0	0	0	0	3									
DQB1*06:100	06:100	HLA09862		0	1	0	0	0	0	0	1									
DQB1*06:106	06:106	HLA09926		0	0	2	0	0	0	0	2									
DQB1*06:114	06:114	HLA10244		0	1	0	0	0	0	0	1									
**DQB1*06:118 total**	**06:118 total**			**1**	**1**	**5**	**0**	**0**	**0**	**0**	**7**			**WD**					**WD**	**WD**

Supplemental Table 15: HLA-DQB1 Allele Summary [a]				Allele Count by Population Group [b]								3.0.0 CIWD Category by Population Group [c]								
Allele	Genomic Typing	Allele ID	G group	AFA	API	EURO	MENA	HIS	NAM	UNK	Total	AFA	API	EURO	MENA	HIS	NAM	UNK	Total	Highest Frequency
DQB1*06:118:02	06:118:02	HLA12031		1	1	5	0	0	0	0	7			WD					WD	WD
DQB1*06:119	06:119	HLA10257		0	0	2	0	0	0	1	3									
DQB1*06:122	06:122	HLA10293		0	1	5	0	0	0	0	6			WD					WD	WD
DQB1*06:123	06:123	HLA10295		0	0	0	4	0	0	0	4									
DQB1*06:124	06:124	HLA10297		0	0	1	0	0	0	0	1									
DQB1*06:125	06:125	HLA10298		0	0	30	0	0	0	0	30			WD					WD	WD
DQB1*06:126	06:126	HLA10300		0	0	0	7	0	0	0	7				WD				WD	WD
DQB1*06:128	06:128	HLA10542		0	0	0	0	1	0	0	1									
DQB1*06:131	06:131	HLA11123		0	0	0	0	1	0	0	1									
DQB1*06:134	06:134	HLA11135		2	0	0	0	1	0	0	3									
DQB1*06:135	06:135	HLA11139		0	7	0	0	0	0	0	7		WD						WD	WD
DQB1*06:136	06:136	HLA11152		0	0	5	0	0	0	0	5			WD					WD	WD
DQB1*06:137	06:137	HLA11153		0	0	1	0	0	0	0	1									
DQB1*06:139	06:139	HLA11156		0	0	1	0	0	0	0	1									
DQB1*06:140	06:140	HLA11273		0	27	0	0	0	0	0	27		I						WD	I
DQB1*06:141	06:141	HLA11280		0	0	1	0	0	0	0	1									
DQB1*06:142	06:142	HLA11281		0	6	0	0	0	0	0	6		WD						WD	WD
DQB1*06:143	06:143	HLA11286		0	0	1	0	0	0	0	1									
DQB1*06:144N	06:144N	HLA11353		0	8	0	0	0	0	2	10		WD						WD	WD
DQB1*06:145	06:145	HLA11354		0	1	2	0	0	0	0	3									
**DQB1*06:146 total**	**06:146 total**			**0**	**0**	**3**	**1**	**0**	**0**	**0**	**4**									
DQB1*06:146	06:146			0	0	1	0	0	0	0	1									
DQB1*06:146:01	06:146:01	HLA11367		0	0	1	1	0	0	0	2									
DQB1*06:146:02	06:146:02	HLA14173		0	0	1	0	0	0	0	1									
DQB1*06:147	06:147	HLA11368		0	0	2	0	0	0	1	3									
DQB1*06:148	06:148	HLA11489		0	4	0	0	0	0	0	4									
DQB1*06:151	06:151	HLA11685		0	0	1	0	0	0	0	1									
DQB1*06:152	06:152	HLA11686		0	0	1	0	0	0	0	1									
DQB1*06:153	06:153	HLA11879		0	5	0	5	0	0	0	10		WD		WD				WD	WD
DQB1*06:155	06:155	HLA11885		0	0	15	0	0	0	0	15			WD					WD	WD
DQB1*06:157	06:157	HLA12025		0	1	0	0	0	0	0	1									

Supplemental Table 15: HLA-DQB1 Allele Summary [a]				Allele Count by Population Group [b]								3.0.0 CIWD Category by Population Group [c]								
Allele	Genomic Typing	Allele ID	G group	AFA	API	EURO	MENA	HIS	NAM	UNK	Total	AFA	API	EURO	MENA	HIS	NAM	UNK	Total	Highest Frequency
DQB1*06:158N	06:158N	HLA12030		0	0	1	0	0	0	1	2									
DQB1*06:159	06:159	HLA12047		0	0	1	0	0	0	0	1									
DQB1*06:160	06:160	HLA12210		0	0	1	0	0	0	0	1									
DQB1*06:162	06:162	HLA12361		0	0	2	0	0	0	0	2									
DQB1*06:164	06:164	HLA12526		0	0	1	0	0	0	2	3									
DQB1*06:166	06:166	HLA12534		0	0	1	0	0	0	0	1									
DQB1*06:169	06:169	HLA13130		0	0	1	2	0	0	1	4									
DQB1*06:170	06:170	HLA13131		0	0	0	0	0	0	2	2									
DQB1*06:171	06:171	HLA13133		2	0	0	0	0	0	0	2									
DQB1*06:172	06:172	HLA13134		0	0	2	0	0	0	0	2									
DQB1*06:173	06:173	HLA13150		0	0	1	0	0	0	0	1									
DQB1*06:176	06:176	HLA13575		0	0	1	0	0	0	0	1									
DQB1*06:177	06:177	HLA13576		0	1	0	0	0	0	0	1									
DQB1*06:178	06:178	HLA13583		0	0	1	0	0	0	0	1									
DQB1*06:179N	06:179N	HLA13584		1	0	0	0	0	0	0	1									
DQB1*06:181	06:181	HLA13677		0	2	0	0	0	0	0	2									
DQB1*06:183	06:183	HLA13813		0	2	0	0	0	0	0	2									
DQB1*06:184	06:184	HLA13882		0	6	0	1	0	0	0	7		WD						WD	WD
**DQB1*06:190 total**	**06:190 total**			**0**	**0**	**3**	**0**	**0**	**0**	**0**	**3**									
DQB1*06:190	06:190			0	0	3	0	0	0	0	3									
DQB1*06:191	06:191	HLA14038		0	0	3	0	0	0	0	3									
DQB1*06:194	06:194	HLA14159		0	0	0	1	0	0	0	1									
DQB1*06:198	06:198	HLA14351		0	0	1	0	0	0	0	1									
DQB1*06:199	06:199	HLA14548		0	0	1	0	0	0	0	1									
DQB1*06:202	06:202	HLA14686		0	0	1	0	0	0	0	1									
**DQB1*06:206 total**	**06:206 total**			**0**	**0**	**1**	**0**	**0**	**0**	**0**	**1**									
DQB1*06:206:02	06:206:02	HLA16189		0	0	1	0	0	0	0	1									
DQB1*06:CODE	06:CODE			5731	2748	82247	808	5173	463	9438	106608	NA	NA	NA	NA	NA	NA	NA	NA	NA
DQB1*NEW [e]	NEW			0	0	2	0	0	0	0	2									
**DQB1*Total**	**Total**			**378275**	**1314598**	**11735570**	**402840**	**678680**	**66309**	**1227926**	**15804198**									

Supplemental Table 15: HLA-DQB1 Allele Summary [a]				Allele Count by Population Group [b]								3.0.0 CIWD Category by Population Group [c]								
Allele	Genomic Typing	Allele ID	G group	AFA	API	EURO	MENA	HIS	NAM	UNK	Total	AFA	API	EURO	MENA	HIS	NAM	UNK	Total	Highest Frequency

C, common; I, intermediate; WD, well-documented; NA, not applicable

[a] All alleles observed in the current dataset are included in this table. Note that alleles are not in numerical order; alleles within a G group are clustered together. P group "two-field" total (e.g., written as "DQB1*02:01 total")and G group total summary rows are provided. The table does not list all alleles from IPD-IMGT version 3.31.0, if not present in the study dataset.

[b] Population groups include: AFA (African/African American), API (Asian/Pacific Islands), EURO (European/European descent), MENA (Middle East/North Coast of Africa), HIS (South or Central America/Hispanic/Latino), NAM (Native American populations) and UNK (unknown/not asked/multiple ancestries/other). Total is the overall population i.e., all groups combined.

[c] Allele frequency is calculated by dividing the number of times the “allele” of interest is observed in a population by the total number of copies of all the alleles at that particular genetic locus in the population (reported as the last row in this table and also in Table 2b). The total number of copies is calculated by multiplying the number of individuals times two for all loci except DRB3/4/5. For DRB3/4/5, the number of assignments was used as the total. The CIWD status is determined based on the allele frequency. Allele frequency data will be provided on the website of the next International HLA and Immunogenetics Workshop (https://www.ihiw18.org/). Highest frequency is the highest CIWD designation among all the individual groups.

[d] "CODE" is generically defined as a summary category of submitted HLA typing, including NMDP multiple allele codes, with ambiguities that are not within a single P or G group. "NEW" is a summary category for assignments of novel alleles that did not yet receive a nomenclature assignment. The CODE and NEW categories add to the total number of alleles but should not be assigned CIWD designations (labeled as NA, not applicable) as they do not represent a consistent allele designation (i.e., the NEW category may contain alleles with different DNA sequences that are unrelated to one another).

[e] DQB1*Total is the total number of allele assignments for the population group and is based on two times the number of individuals in the group. This number is also listed in Table 2b. It is not the sum of the column as alleles are not counted more than once. For example, when evaluating frequencies at the level of G resolution, individual alleles that make up the G group (e.g., A*80:01:01, A*80:01:01:01, A*80:01:01:02, A*80:01:01G) are not included in the count because these alleles are summed up in the total G designation (e.g., "A*80:01:01G total").
