# Supplementary material for: Common, intermediate and well‐documented HLA alleles in world populations: CIWD version 3.0.0
Source: HLA. 2020 Jan 31;95(6):516–31. doi: 10.1111/tan.13811 (PMC7317522; doi:10.1111/tan.13811)
Supplement: Supplementary file 16 — Table S16 HLA‐DPB1 primary data [file TAN-95-516-s016.pdf]

Supplemental Table 16: HLA-DPB1 Allele Summary [a]				Allele Count by Population Group [b]								3.0.0 CIWD Category by Population Group [c]								
Allele	Genomic Typing	Allele ID	G group	AFA	API	EURO	MENA	HIS	NAM	UNK	Total	AFA	API	EURO	MENA	HIS	NAM	UNK	Total	Highest Frequency
**DPB1*01:01 total**	**01:01 total**			**96157**	**43213**	**514372**	**3927**	**29680**	**4448**	**63592**	**755389**	**C**	**C**	**C**	**C**	**C**	**C**	**C**	**C**	**C**
DPB1*01:01	01:01			8449	1878	24111	248	4260	388	4000	43334	C	C	C	C	C	C	C	C	C
DPB1*01:01P	01:01P			0	0	3	0	0	0	0	3									
**DPB1*01:01:01G total**	**01:01:01G total**			**77723**	**41250**	**483370**	**3230**	**23343**	**3728**	**56600**	**689244**	**C**	**C**	**C**	**C**	**C**	**C**	**C**	**C**	**C**
DPB1*01:01:01G	01:01:01G		01:01:01G	38533	31936	258073	1693	13228	1773	33735	378971	C	C	C	C	C	C	C	C	C
DPB1*01:01:01	01:01:01		01:01:01G	39181	9313	225269	1536	10103	1955	22860	310217	C	C	C	C	C	C	C	C	C
DPB1*162:01:02	162:01:02	HLA17156	01:01:01G	3	0	14	1	1	0	1	20			WD					WD	WD
DPB1*417:01	417:01	HLA12847	01:01:01G	6	1	4	0	4	0	2	17	WD							WD	WD
DPB1*462:01	462:01	HLA13715	01:01:01G	0	0	10	0	7	0	2	19			WD		I			WD	I
**DPB1*01:01:02G total**	**01:01:02G total**			**9977**	**85**	**6843**	**449**	**2076**	**331**	**2989**	**22750**	**C**	**I**	**C**	**C**	**C**	**C**	**C**	**C**	**C**
DPB1*01:01:02G	01:01:02G		01:01:02G	5418	57	3397	240	1233	189	1779	12313	C	I	C	C	C	C	C	C	C
DPB1*01:01:02	01:01:02		01:01:01G	4559	28	3446	209	843	142	1210	10437	C	I	C	C	C	C	C	C	C
DPB1*01:01:03	01:01:03	HLA02006		0	0	42	0	0	1	2	45			WD					WD	WD
DPB1*01:01:05	01:01:05	HLA11704		0	0	2	0	0	0	0	2									
DPB1*01:01:06	01:01:06	HLA13312		2	0	0	0	0	0	0	2									
**DPB1*162:01 total**	**162:01 total**			**9**	**0**	**15**	**1**	**2**	**0**	**2**	**29**	**WD**		**WD**					**WD**	**WD**
DPB1*162:01	162:01			6	0	1	0	1	0	1	9	WD							WD	WD
DPB1*01:CODE [d]	01:CODE			1296	395	17888	106	400	26	945	21056	NA	NA	NA	NA	NA	NA	NA	NA	NA
**DPB1*02:01 total**	**02:01 total**			**44508**	**224189**	**1497120**	**58524**	**71055**	**7855**	**139233**	**2042484**	**C**	**C**	**C**	**C**	**C**	**C**	**C**	**C**	**C**
DPB1*02:01	02:01			7282	18728	305459	10910	13012	1233	13331	369955	C	C	C	C	C	C	C	C	C
DPB1*02:01P	02:01P			1	0	41	0	0	0	4	46			WD					WD	WD
**DPB1*02:01:02G total**	**02:01:02G total**			**37160**	**205205**	**1185681**	**47597**	**57618**	**6539**	**125340**	**1665140**	**C**	**C**	**C**	**C**	**C**	**C**	**C**	**C**	**C**
DPB1*02:01:02G	02:01:02G		02:01:02G	22033	167104	456132	15193	39608	4344	95600	800014	C	C	C	C	C	C	C	C	C
DPB1*02:01:02	02:01:02		02:01:02G	15073	37805	728640	32350	17921	2186	29660	863635	C	C	C	C	C	C	C	C	C
DPB1*02:01:02:01	02:01:02:01	HLA00517	02:01:02G	0	0	1	0	0	0	0	1									
DPB1*02:01:02:05	02:01:02:05	HLA16911	02:01:02G	0	0	1	0	0	0	0	1									
DPB1*02:01:02:10	02:01:02:10	HLA16908	02:01:02G	0	0	7	0	0	0	0	7			WD					WD	WD
DPB1*02:01:02:12	02:01:02:12	HLA17103	02:01:02G	0	0	1	0	0	0	0	1									
DPB1*02:01:20	02:01:20	HLA17186	02:01:02G	0	0	2	0	0	0	0	2									
DPB1*141:01	141:01	HLA08802	02:01:02G	3	18	45	0	1	0	4	71		I	WD					WD	I
DPB1*352:01	352:01	HLA11946	02:01:02G	0	4	36	1	1	1	0	43			WD					WD	WD

Supplemental Table 16: HLA-DPB1 Allele Summary [a]				Allele Count by Population Group [b]								3.0.0 CIWD Category by Population Group [c]								
Allele	Genomic Typing	Allele ID	G group	AFA	API	EURO	MENA	HIS	NAM	UNK	Total	AFA	API	EURO	MENA	HIS	NAM	UNK	Total	Highest Frequency
**DPB1*414:01 total**	**414:01 total**			**46**	**197**	**67**	**10**	**16**	**5**	**39**	**380**	**C**	**C**	**WD**	**WD**	**I**	**WD**	**I**	**I**	**C**
DPB1*414:01	414:01		02:01:02G	46	197	67	10	16	5	39	380	C	C	WD	WD	I	WD	I	I	C
**DPB1*416:01 total**	**416:01 total**			**5**	**76**	**712**	**42**	**71**	**3**	**35**	**944**	**WD**	**I**	**I**	**C**	**C**		**I**	**I**	**C**
DPB1*416:01	416:01		02:01:02G	5	71	633	39	69	3	34	854	WD	I	I	C	C		I	I	C
DPB1*416:01:01	416:01:01		02:01:02G	0	5	79	3	2	0	1	90		WD	WD					WD	WD
DPB1*617:01	617:01	HLA16349	02:01:02G	0	1	18	1	0	0	0	20			WD					WD	WD
DPB1*640:01	640:01	HLA16846	02:01:02G	0	0	19	0	0	0	2	21			WD					WD	WD
DPB1*02:01:03	02:01:03	HLA00518		0	0	87	0	0	0	1	88			WD					WD	WD
DPB1*02:01:04	02:01:04	HLA01473		42	195	5551	11	405	83	523	6810	C	C	C	WD	C	C	C	C	C
DPB1*02:01:05	02:01:05	HLA01592		0	32	2	0	0	0	5	39		I					WD	WD	I
DPB1*02:01:06	02:01:06	HLA01615		16	0	2	0	18	0	6	42	WD				I		WD	WD	I
DPB1*02:01:07	02:01:07	HLA03491		0	1	45	5	0	0	2	53			WD	WD				WD	WD
DPB1*02:01:08	02:01:08	HLA08801		0	0	177	0	1	0	2	180			I					I	I
DPB1*02:01:09	02:01:09	HLA09399		0	0	35	0	1	0	10	46			WD				I	WD	I
DPB1*02:01:10	02:01:10	HLA10358		0	0	2	0	0	0	0	2									
DPB1*02:01:11	02:01:11	HLA10560		0	0	4	0	0	0	0	4									
DPB1*02:01:12	02:01:12	HLA10948		0	16	0	0	0	0	7	23		I					WD	WD	I
DPB1*02:01:13	02:01:13	HLA11698		0	12	0	0	0	0	2	14		I						WD	I
DPB1*02:01:14	02:01:14	HLA12223		1	0	0	0	0	0	0	1									
DPB1*02:01:15	02:01:15	HLA12383		0	0	31	0	0	0	0	31			WD					WD	WD
DPB1*02:01:16	02:01:16	HLA12842		1	0	1	0	0	0	0	2									
DPB1*02:01:18	02:01:18	HLA14584		5	0	0	1	0	0	0	6	WD							WD	WD
DPB1*02:01:21	02:01:21	HLA17264		0	0	2	0	0	0	0	2									
**DPB1*02:02 total**	**02:02 total**			**603**	**16154**	**42011**	**479**	**5456**	**398**	**6762**	**71863**	**C**	**C**	**C**	**C**	**C**	**C**	**C**	**C**	**C**
**DPB1*02:02:01G total**	**02:02:01G total**			**603**	**16154**	**42011**	**479**	**5456**	**398**	**6762**	**71863**	**C**	**C**	**C**	**C**	**C**	**C**	**C**	**C**	**C**
DPB1*02:02	02:02		02:02:01G	569	14292	38465	445	5184	388	5813	65156	C	C	C	C	C	C	C	C	C
DPB1*02:02P	02:02P			0	0	3	0	0	0	0	3									
DPB1*02:02:01G	02:02:01G		02:02:01G	22	1587	1779	24	163	9	865	4449	WD	C	C	WD	C	C	C	C	C
DPB1*02:02:01	02:02:01		02:02:01G	12	268	1762	10	109	1	84	2246	WD	C	C	WD	C		I	C	C
DPB1*02:02:01:01	02:02:01:01	HLA00519	02:02:01G	0	0	1	0	0	0	0	1									
DPB1*547:01	547:01	HLA14679	02:02:01G	0	7	1	0	0	0	0	8		WD						WD	WD

Supplemental Table 16: HLA-DPB1 Allele Summary [a]				Allele Count by Population Group [b]								3.0.0 CIWD Category by Population Group [c]								
Allele	Genomic Typing	Allele ID	G group	AFA	API	EURO	MENA	HIS	NAM	UNK	Total	AFA	API	EURO	MENA	HIS	NAM	UNK	Total	Highest Frequency
DPB1*02:CODE	02:CODE			296	109	826	16	227	10	326	1810	NA	NA	NA	NA	NA	NA	NA	NA	NA
**DPB1*03:01 total**	**03:01 total**			**19170**	**56363**	**1091785**	**21128**	**43335**	**4596**	**89989**	**1326366**	**C**	**C**	**C**	**C**	**C**	**C**	**C**	**C**	**C**
DPB1*03:01	03:01			0	19	150	3	8	0	59	239		I	I		I		I	I	I
DPB1*03:01P	03:01P			2	0	31	1	0	0	2	36			WD					WD	WD
**DPB1*03:01:01G total**	**03:01:01G total**			**19167**	**56341**	**1091585**	**21124**	**43327**	**4595**	**89926**	**1326065**	**C**	**C**	**C**	**C**	**C**	**C**	**C**	**C**	**C**
DPB1*03:01:01G	03:01:01G		03:01:01G	18552	55609	1074368	20827	41028	4399	88341	1303124	C	C	C	C	C	C	C	C	C
DPB1*03:01:01	03:01:01		03:01:01G	240	530	7870	70	1219	96	1043	11068	C	C	C	C	C	C	C	C	C
DPB1*03:01:01:04	03:01:01:04	HLA17115	03:01:01G	0	0	1	0	0	0	0	1									
DPB1*03:01:08	03:01:08	HLA13716	03:01:01G	2	5	323	3	36	2	16	387		WD	I		I		I	I	I
**DPB1*104:01 total**	**104:01 total**			**272**	**164**	**1606**	**114**	**546**	**65**	**244**	**3011**	**C**	**C**	**C**	**C**	**C**	**C**	**C**	**C**	**C**
DPB1*104:01	104:01		03:01:01G	174	125	1109	62	340	49	156	2015	C	C	C	C	C	C	C	C	C
DPB1*104:01:01	104:01:01		03:01:01G	98	39	497	52	206	16	88	996	C	I	I	C	C	C	I	I	C
**DPB1*124:01 total**	**124:01 total**			**101**	**32**	**7364**	**108**	**495**	**33**	**276**	**8409**	**C**	**I**	**C**	**C**	**C**	**C**	**C**	**C**	**C**
DPB1*124:01	124:01		03:01:01G	101	32	7192	106	494	33	276	8234	C	I	C	C	C	C	C	C	C
DPB1*124:01:01	124:01:01		03:01:01G	0	0	172	2	1	0	0	175			I					I	I
DPB1*351:01	351:01	HLA11945	03:01:01G	0	1	53	2	3	0	6	65			WD				WD	WD	WD
DPB1*03:01:02	03:01:02	HLA01306		0	0	1	0	0	0	1	2									
DPB1*03:01:03	03:01:03	HLA09407		0	0	5	0	0	0	0	5			WD					WD	WD
DPB1*03:01:04	03:01:04	HLA12394		0	1	7	0	0	0	0	8			WD					WD	WD
DPB1*03:01:05	03:01:05	HLA13316		0	2	0	0	0	0	0	2									
DPB1*03:01:06	03:01:06	HLA13318		1	0	6	0	0	1	1	9			WD					WD	WD
DPB1*03:CODE	03:CODE			53	32	419	7	76	7	123	717	NA	NA	NA	NA	NA	NA	NA	NA	NA
**DPB1*04:01 total**	**04:01 total**			**36759**	**303789**	**4531708**	**117802**	**160009**	**16209**	**357840**	**5524116**	**C**	**C**	**C**	**C**	**C**	**C**	**C**	**C**	**C**
DPB1*04:01	04:01			643	4955	282023	11868	1174	63	4245	304971	C	C	C	C	C	C	C	C	C
DPB1*04:01P	04:01P			0	0	99	0	1	0	8	108			WD				WD	WD	WD
**DPB1*04:01:01G total**	**04:01:01G total**			**36114**	**298816**	**4248567**	**105905**	**158678**	**16133**	**353545**	**5217758**	**C**	**C**	**C**	**C**	**C**	**C**	**C**	**C**	**C**
DPB1*04:01:01G	04:01:01G		04:01:01G	22965	236715	1847776	40233	106066	11385	282554	2547694	C	C	C	C	C	C	C	C	C
DPB1*04:01:01	04:01:01		04:01:01G	12773	61244	2381370	65108	50223	4526	69168	2644412	C	C	C	C	C	C	C	C	C
DPB1*04:01:01:01	04:01:01:01	HLA00521	04:01:01G	252	677	13696	124	1974	191	1590	18504	C	C	C	C	C	C	C	C	C
DPB1*04:01:01:02	04:01:01:02	HLA06633	04:01:01G	38	52	804	14	210	20	88	1226	C	I	I	WD	C	C	I	I	C
DPB1*04:01:01:03	04:01:01:03	HLA16896	04:01:01G	0	0	3	0	0	0	0	3									

Supplemental Table 16: HLA-DPB1 Allele Summary [a]				Allele Count by Population Group [b]								3.0.0 CIWD Category by Population Group [c]								
Allele	Genomic Typing	Allele ID	G group	AFA	API	EURO	MENA	HIS	NAM	UNK	Total	AFA	API	EURO	MENA	HIS	NAM	UNK	Total	Highest Frequency
DPB1*04:01:01:04	04:01:01:04	HLA16897	04:01:01G	0	0	6	0	0	0	0	6			WD					WD	WD
DPB1*04:01:01:06	04:01:01:06	HLA17116	04:01:01G	0	0	32	0	0	0	0	32			WD					WD	WD
DPB1*04:01:31	04:01:31	HLA13719	04:01:01G	0	1	239	7	0	0	3	250			I	WD				I	I
**DPB1*126:01 total**	**126:01 total**			**23**	**77**	**3366**	**308**	**173**	**11**	**73**	**4031**	**WD**	**I**	**C**	**C**	**C**	**C**	**I**	**C**	**C**
DPB1*126:01	126:01		04:01:01G	23	77	3366	308	173	11	73	4031	WD	I	C	C	C	C	I	C	C
DPB1*350:01	350:01	HLA11944	04:01:01G	63	48	953	51	29	0	64	1208	C	I	I	C	I		I	I	C
DPB1*415:01	415:01	HLA12864	04:01:01G	0	2	7	59	0	0	1	69			WD	C				WD	C
DPB1*459:01	459:01	HLA13712	04:01:01G	0	0	31	0	0	0	0	31			WD					WD	WD
DPB1*464:01	464:01	HLA13718	04:01:01G	0	0	281	1	3	0	4	289			I					I	I
DPB1*615:01	615:01	HLA16315	04:01:01G	0	0	3	0	0	0	0	3									
DPB1*04:01:03	04:01:03	HLA07382		0	1	147	0	0	0	2	150			I					I	I
**DPB1*04:01:04G total**	**04:01:04G total**			**0**	**0**	**743**	**3**	**68**	**2**	**18**	**834**			**I**		**C**		**I**	**I**	**C**
DPB1*04:01:04G	04:01:04G		04:01:04G	0	0	48	0	0	0	0	48			WD					WD	WD
DPB1*04:01:04	04:01:04		04:01:04G	0	0	695	3	68	2	18	786			I		C		I	I	C
DPB1*04:01:05	04:01:05	HLA09389		0	0	1	0	0	0	0	1									
DPB1*04:01:06	04:01:06	HLA09393		1	0	20	0	66	4	8	99			WD		C		WD	WD	C
DPB1*04:01:07	04:01:07	HLA09394		1	0	4	0	14	7	3	29					I	C		WD	C
DPB1*04:01:08	04:01:08	HLA10329		0	1	6	17	0	0	1	25			WD	WD				WD	WD
DPB1*04:01:09	04:01:09	HLA10330		0	0	8	0	0	0	0	8			WD					WD	WD
DPB1*04:01:10	04:01:10	HLA10332		0	0	4	0	0	0	0	4									
DPB1*04:01:11	04:01:11	HLA10335		0	1	3	9	0	0	0	13				WD				WD	WD
DPB1*04:01:12	04:01:12	HLA10340		0	0	10	0	0	0	2	12			WD					WD	WD
DPB1*04:01:13	04:01:13	HLA10353		0	0	12	0	0	0	3	15			WD					WD	WD
DPB1*04:01:14	04:01:14	HLA10356		0	0	18	0	0	0	0	18			WD					WD	WD
DPB1*04:01:15	04:01:15	HLA10443		0	8	2	0	0	0	0	10		WD						WD	WD
DPB1*04:01:16	04:01:16	HLA10945		0	0	8	0	0	0	0	8			WD					WD	WD
DPB1*04:01:18	04:01:18	HLA11196		0	0	3	0	0	0	1	4									
DPB1*04:01:19	04:01:19	HLA11383		0	0	6	0	0	0	0	6			WD					WD	WD
DPB1*04:01:20	04:01:20	HLA11384		0	0	6	0	0	0	0	6			WD					WD	WD
DPB1*04:01:21	04:01:21	HLA11616		0	0	3	0	1	0	2	6								WD	WD
DPB1*04:01:22	04:01:22	HLA11915		0	0	1	0	0	0	1	2									

Supplemental Table 16: HLA-DPB1 Allele Summary [a]				Allele Count by Population Group [b]								3.0.0 CIWD Category by Population Group [c]								
**Allele**	**Genomic Typing**	**Allele ID**	**G group**	**AFA**	**API**	**EURO**	**MENA**	**HIS**	**NAM**	**UNK**	**Total**	**AFA**	**API**	**EURO**	**MENA**	**HIS**	**NAM**	**UNK**	**Total**	**Highest Frequency**
DPB1*04:01:23	04:01:23	HLA11916		0	0	2	0	0	0	0	2									
DPB1*04:01:24	04:01:24	HLA12386		0	0	5	0	0	0	0	5			WD					WD	WD
DPB1*04:01:25	04:01:25	HLA12841		0	0	2	0	0	0	0	2									
DPB1*04:01:28	04:01:28	HLA12894		0	0	3	0	6	0	1	10					I			WD	I
DPB1*04:01:29	04:01:29	HLA13296		0	0	1	0	0	0	0	1									
DPB1*04:01:30	04:01:30	HLA13300		0	0	1	0	1	0	0	2									
DPB1*04:01:32	04:01:32	HLA14645		0	7	0	0	0	0	0	7		WD						WD	WD
**DPB1*04:02 total**	**04:02 total**			**43607**	**63385**	**1340262**	**43186**	**160940**	**13684**	**126201**	**1791265**	**C**	**C**	**C**	**C**	**C**	**C**	**C**	**C**	**C**
DPB1*04:02	04:02			107	578	40619	2566	1196	61	869	45996	C	C	C	C	C	C	C	C	C
DPB1*04:02P	04:02P			1	0	52	0	1	0	4	58			WD					WD	WD
**DPB1*04:02:01G total**	**04:02:01G total**			**43499**	**62804**	**1299582**	**40620**	**159725**	**13623**	**125326**	**1745179**	**C**	**C**	**C**	**C**	**C**	**C**	**C**	**C**	**C**
DPB1*04:02:01G	04:02:01G		04:02:01G	28570	50792	899407	25668	107275	9585	103983	1225280	C	C	C	C	C	C	C	C	C
DPB1*04:02:01	04:02:01		04:02:01G	2320	9927	377979	13871	36894	2561	15119	458671	C	C	C	C	C	C	C	C	C
DPB1*04:02:01:01	04:02:01:01	HLA00522	04:02:01G	88	94	2852	125	730	52	365	4306	C	I	C	C	C	C	C	C	C
DPB1*04:02:01:02	04:02:01:02	HLA06634	04:02:01G	425	1195	11184	246	11394	869	2277	27590	C	C	C	C	C	C	C	C	C
DPB1*04:02:01:04	04:02:01:04	HLA17120	04:02:01G	0	0	1	0	0	0	0	1									
DPB1*04:02:10	04:02:10	HLA17180	04:02:01G	0	0	2	0	0	0	0	2									
**DPB1*105:01 total**	**105:01 total**			**12041**	**793**	**8019**	**708**	**3374**	**552**	**3559**	**29046**	**C**	**C**	**C**	**C**	**C**	**C**	**C**	**C**	**C**
DPB1*105:01	105:01		04:02:01G	11621	770	7778	682	3229	542	3457	28079	C	C	C	C	C	C	C	C	C
DPB1*105:01:01	105:01:01		04:02:01G	420	23	241	26	145	10	102	967	C	I	I	WD	C	C	C	I	C
**DPB1*463:01 total**	**463:01 total**			**54**	**3**	**134**	**2**	**58**	**4**	**22**	**277**	**C**		**I**		**I**		**I**	**I**	**C**
DPB1*463:01	463:01		04:02:01G	49	3	114	2	56	4	21	249	C		I		I		I	I	C
DPB1*463:01:01	463:01:01		04:02:01G	5	0	20	0	2	0	1	28	WD		WD					WD	WD
DPB1*571:01	571:01	HLA15391	04:02:01G	1	0	0	0	0	0	0	1									
DPB1*647:01	647:01	HLA17078	04:02:01G	0	0	4	0	0	0	1	5								WD	WD
DPB1*04:02:03	04:02:03	HLA11382		0	1	0	0	9	0	1	11					I			WD	I
DPB1*04:02:04	04:02:04	HLA11622		0	0	0	0	3	0	0	3									
DPB1*04:02:05	04:02:05	HLA11699		0	0	8	0	0	0	0	8			WD					WD	WD
DPB1*04:02:06	04:02:06	HLA11922		0	0	0	0	1	0	1	2									
DPB1*04:02:07	04:02:07	HLA12224		0	2	0	0	5	0	0	7					WD			WD	WD
DPB1*04:02:08	04:02:08	HLA13604		0	0	1	0	0	0	0	1									

Supplemental Table 16: HLA-DPB1 Allele Summary [a]				Allele Count by Population Group [b]								3.0.0 CIWD Category by Population Group [c]								
Allele	Genomic Typing	Allele ID	G group	AFA	API	EURO	MENA	HIS	NAM	UNK	Total	AFA	API	EURO	MENA	HIS	NAM	UNK	Total	Highest Frequency
DPB1*04:CODE	04:CODE			530	90	4298	255	600	31	922	6726	NA	NA	NA	NA	NA	NA	NA	NA	NA
**DPB1*05:01 total**	**05:01 total**			**1679**	**105570**	**193213**	**7024**	**13439**	**1128**	**33728**	**355781**	**C**	**C**	**C**	**C**	**C**	**C**	**C**	**C**	**C**
DPB1*05:01	05:01			28	1296	5060	321	170	28	594	7497	WD	C	C	C	C	C	C	C	C
DPB1*05:01P	05:01P			0	1	3	0	0	0	1	5								WD	WD
**DPB1*05:01:01G total**	**05:01:01G total**			**1651**	**104271**	**188149**	**6703**	**13269**	**1100**	**33132**	**348275**	**C**	**C**	**C**	**C**	**C**	**C**	**C**	**C**	**C**
DPB1*05:01:01G	05:01:01G		05:01:01G	1597	100602	185249	6507	12646	1053	32235	339889	C	C	C	C	C	C	C	C	C
DPB1*05:01:01	05:01:01		05:01:01G	53	3406	2888	193	617	46	826	8029	C	C	C	C	C	C	C	C	C
DPB1*05:01:02	05:01:02	HLA03630	05:01:01G	0	8	9	1	1	0	0	19		WD	WD					WD	WD
DPB1*135:01	135:01	HLA07334	05:01:01G	1	255	3	2	5	1	71	338		C			WD		I	I	C
DPB1*05:01:03	05:01:03	HLA12376		0	1	1	0	0	0	1	3									
DPB1*05:01:06	05:01:06	HLA13298		0	1	0	0	0	0	0	1									
DPB1*05:CODE	05:CODE			31	1027	643	2	152	9	473	2337	NA	NA	NA	NA	NA	NA	NA	NA	NA
**DPB1*06:01 total**	**06:01 total**			**1340**	**545**	**156236**	**956**	**5058**	**527**	**12493**	**177155**	**C**	**C**	**C**	**C**	**C**	**C**	**C**	**C**	**C**
DPB1*06:01	06:01			1091	374	115743	761	4010	428	9303	131710	C	C	C	C	C	C	C	C	C
**DPB1*06:01:01G total**	**06:01:01G total**			**249**	**171**	**40492**	**195**	**1048**	**99**	**3190**	**45444**	**C**	**C**	**C**	**C**	**C**	**C**	**C**	**C**	**C**
DPB1*06:01:01G	06:01:01G		06:01:01G	0	45	298	0	0	0	433	776		I	I				C	I	C
DPB1*06:01:01	06:01:01		06:01:01G	249	126	40194	195	1048	99	2757	44668	C	C	C	C	C	C	C	C	C
DPB1*06:01:02	06:01:02	HLA14938		0	0	1	0	0	0	0	1									
DPB1*06:CODE	06:CODE			2	0	71	0	9	0	14	96	NA	NA	NA	NA	NA	NA	NA	NA	NA
DPB1*08:01	08:01	HLA00525		1	0	27	3	1	0	2	34			WD					WD	WD
DPB1*08:CODE	08:CODE			0	0	2	0	0	0	1	3	NA	NA	NA	NA	NA	NA	NA	NA	NA
**DPB1*09:01 total**	**09:01 total**			**659**	**48074**	**73269**	**4284**	**3316**	**362**	**7699**	**137663**	**C**	**C**	**C**	**C**	**C**	**C**	**C**	**C**	**C**
DPB1*09:01	09:01			115	2101	9489	758	799	64	1113	14439	C	C	C	C	C	C	C	C	C
DPB1*09:01:01	09:01:01	HLA00526		536	45943	63687	3518	2313	282	6510	122789	C	C	C	C	C	C	C	C	C
DPB1*09:01:02	09:01:02	HLA09088		8	29	80	7	204	16	76	420	WD	I	WD	WD	C	C	I	I	C
DPB1*09:01:03	09:01:03	HLA11197		0	1	13	0	0	0	0	14			WD					WD	WD
DPB1*09:01:04	09:01:04	HLA13908		0	0	0	1	0	0	0	1									
DPB1*09:CODE	09:CODE			4	26	140	15	27	0	39	251	NA	NA	NA	NA	NA	NA	NA	NA	NA
**DPB1*10:01 total**	**10:01 total**			**1645**	**8163**	**161416**	**5329**	**9887**	**881**	**14444**	**201765**	**C**	**C**	**C**	**C**	**C**	**C**	**C**	**C**	**C**
DPB1*10:01	10:01			1257	3795	102755	3370	7701	699	9958	129535	C	C	C	C	C	C	C	C	C
DPB1*10:01P	10:01P			0	0	1	0	0	0	0	1									

Supplemental Table 16: HLA-DPB1 Allele Summary [a]				Allele Count by Population Group [b]								3.0.0 CIWD Category by Population Group [c]								
Allele	Genomic Typing	Allele ID	G group	AFA	API	EURO	MENA	HIS	NAM	UNK	Total	AFA	API	EURO	MENA	HIS	NAM	UNK	Total	Highest Frequency
**DPB1*10:01:01G total**	**10:01:01G total**			**388**	**4368**	**58659**	**1959**	**2186**	**182**	**4486**	**72228**	**C**	**C**	**C**	**C**	**C**	**C**	**C**	**C**	**C**
DPB1*10:01:01G	10:01:01G		10:01:01G	23	3398	9200	208	45	3	400	13277	WD	C	C	C	I		C	C	C
DPB1*10:01:01	10:01:01		10:01:01G	365	970	49443	1751	2141	179	4086	58935	C	C	C	C	C	C	C	C	C
DPB1*10:01:01:01	10:01:01:01	HLA00527	10:01:01G	0	0	16	0	0	0	0	16			WD					WD	WD
DPB1*10:01:03	10:01:03	HLA15729		0	0	1	0	0	0	0	1									
DPB1*10:CODE	10:CODE			5	3	157	2	23	3	19	212	NA	NA	NA	NA	NA	NA	NA	NA	NA
**DPB1*11:01 total**	**11:01 total**			**10271**	**595**	**152736**	**1060**	**12660**	**1290**	**15995**	**194607**	**C**	**C**	**C**	**C**	**C**	**C**	**C**	**C**	**C**
DPB1*11:01	11:01			1076	31	8735	82	2195	119	1179	13417	C	I	C	C	C	C	C	C	C
DPB1*11:01P	11:01P			0	0	1	0	0	0	0	1									
**DPB1*11:01:01G total**	**11:01:01G total**			**9190**	**563**	**143972**	**978**	**10327**	**1171**	**14799**	**181000**	**C**	**C**	**C**	**C**	**C**	**C**	**C**	**C**	**C**
DPB1*11:01:01G	11:01:01G		11:01:01G	129	21	7453	35	51	2	119	7810	C	I	C	C	I		C	C	C
DPB1*11:01:01	11:01:01	HLA00528	11:01:01G	9061	542	136519	943	10276	1169	14680	173190	C	C	C	C	C	C	C	C	C
DPB1*11:01:02	11:01:02	HLA00529		4	1	7	0	137	0	15	164			WD		C		I	I	C
DPB1*11:01:03	11:01:03	HLA10952		1	0	21	0	0	0	2	24			WD					WD	WD
DPB1*11:01:04	11:01:04	HLA11707		0	0	0	0	1	0	0	1									
DPB1*11:CODE	11:CODE			19	3	93	1	27	0	40	183	NA	NA	NA	NA	NA	NA	NA	NA	NA
**DPB1*13:01 total**	**13:01 total**			**13056**	**71443**	**203450**	**7320**	**12273**	**1414**	**21520**	**330476**	**C**	**C**	**C**	**C**	**C**	**C**	**C**	**C**	**C**
DPB1*13:01	13:01			0	18	15	0	3	1	9	46		I	WD				WD	WD	I
DPB1*13:01P	13:01P			0	0	17	0	0	0	1	18			WD					WD	WD
**DPB1*13:01:01G total**	**13:01:01G total**			**13056**	**71425**	**203277**	**7320**	**12270**	**1413**	**21509**	**330270**	**C**	**C**	**C**	**C**	**C**	**C**	**C**	**C**	**C**
DPB1*13:01:01G	13:01:01G		13:01:01G	12910	71381	203010	7296	12241	1411	21465	329714	C	C	C	C	C	C	C	C	C
DPB1*13:01:01	13:01:01		13:01:01G	2	18	2	0	0	0	2	24		I						WD	I
DPB1*107:01	107:01	HLA02050	13:01:01G	1	0	0	0	0	0	0	1									
DPB1*133:01	133:01	HLA06672	13:01:01G	143	25	264	24	28	2	41	527	C	I	I	WD	I		I	I	C
DPB1*519:01	519:01	HLA14414	13:01:01G	0	1	1	0	1	0	1	4									
DPB1*13:01:02	13:01:02	HLA09721		0	0	141	0	0	0	1	142			I					I	I
DPB1*13:CODE	13:CODE			50	81	127	2	34	2	68	364	NA	NA	NA	NA	NA	NA	NA	NA	NA
**DPB1*14:01 total**	**14:01 total**			**1995**	**46241**	**147420**	**7652**	**17139**	**1804**	**16289**	**238540**	**C**	**C**	**C**	**C**	**C**	**C**	**C**	**C**	**C**
DPB1*14:01	14:01			974	7980	67242	4185	8215	977	6876	96449	C	C	C	C	C	C	C	C	C
DPB1*14:01P	14:01P			0	0	10	0	0	0	0	10			WD					WD	WD
**DPB1*14:01:01G total**	**14:01:01G total**			**1021**	**38261**	**80165**	**3467**	**8924**	**827**	**9413**	**142078**	**C**	**C**	**C**	**C**	**C**	**C**	**C**	**C**	**C**

Supplemental Table 16: HLA-DPB1 Allele Summary [a]				Allele Count by Population Group [b]								3.0.0 CIWD Category by Population Group [c]								
Allele	Genomic Typing	Allele ID	G group	AFA	API	EURO	MENA	HIS	NAM	UNK	Total	AFA	API	EURO	MENA	HIS	NAM	UNK	Total	Highest Frequency
DPB1*14:01:01G	14:01:01G		14:01:01G	74	20006	8514	447	490	32	2481	32044	C	C	C	C	C	C	C	C	C
DPB1*14:01:01	14:01:01		14:01:01G	947	18195	71612	3020	8431	795	6930	109930	C	C	C	C	C	C	C	C	C
DPB1*14:01:01:01	14:01:01:01	HLA00531	14:01:01G	0	0	1	0	0	0	0	1									
DPB1*498:01	498:01	HLA14196	14:01:01G	0	60	0	0	0	0	2	62		I						WD	I
DPB1*572:01	572:01	HLA15165	14:01:01G	0	0	32	0	2	0	0	34			WD					WD	WD
DPB1*651:01	651:01	HLA17193	14:01:01G	0	0	6	0	1	0	0	7			WD					WD	WD
DPB1*14:01:02	14:01:02	HLA12848		0	0	1	0	0	0	0	1									
DPB1*14:01:03	14:01:03	HLA14366		0	0	2	0	0	0	0	2									
DPB1*14:CODE	14:CODE			2	15	81	9	21	1	15	144	NA	NA	NA	NA	NA	NA	NA	NA	NA
**DPB1*15:01 total**	**15:01 total**			**1847**	**6807**	**80000**	**2521**	**2815**	**270**	**7063**	**101323**	**C**	**C**	**C**	**C**	**C**	**C**	**C**	**C**	**C**
DPB1*15:01	15:01			663	520	25117	865	1084	121	1801	30171	C	C	C	C	C	C	C	C	C
DPB1*15:01P	15:01P			0	0	1	0	0	0	0	1									
**DPB1*15:01:01G total**	**15:01:01G total**			**1184**	**6287**	**54882**	**1656**	**1731**	**149**	**5261**	**71150**	**C**	**C**	**C**	**C**	**C**	**C**	**C**	**C**	**C**
DPB1*15:01:01G	15:01:01G		15:01:01G	44	1180	546	36	30	2	688	2526	C	C	I	C	I		C	C	C
DPB1*15:01:01	15:01:01	HLA00532	15:01:01G	1140	5106	54333	1609	1701	147	4573	68609	C	C	C	C	C	C	C	C	C
DPB1*585:01	585:01	HLA15401	15:01:01G	0	1	3	11	0	0	0	15				WD				WD	WD
DPB1*15:01:02	15:01:02	HLA11709		0	0	0	0	0	0	1	1									
DPB1*15:CODE	15:CODE			6	1	10	2	3	0	5	27	NA	NA	NA	NA	NA	NA	NA	NA	NA
**DPB1*16:01 total**	**16:01 total**			**422**	**4966**	**61067**	**364**	**1558**	**167**	**4497**	**73041**	**C**	**C**	**C**	**C**	**C**	**C**	**C**	**C**	**C**
DPB1*16:01	16:01			172	323	19548	131	553	82	1207	22016	C	C	C	C	C	C	C	C	C
**DPB1*16:01:01G total**	**16:01:01G total**			**250**	**4643**	**41512**	**233**	**1005**	**85**	**3290**	**51018**	**C**	**C**	**C**	**C**	**C**	**C**	**C**	**C**	**C**
DPB1*16:01:01G	16:01:01G		16:01:01G	5	239	3231	17	7	0	34	3533	WD	C	C	WD	I		I	C	C
DPB1*16:01:01	16:01:01	HLA00533	16:01:01G	245	4404	38281	216	998	85	3256	47485	C	C	C	C	C	C	C	C	C
DPB1*16:01:02	16:01:02	HLA11385		0	0	7	0	0	0	0	7			WD					WD	WD
DPB1*16:CODE	16:CODE			12	4	286	0	23	1	54	380	NA	NA	NA	NA	NA	NA	NA	NA	NA
**DPB1*17:01 total**	**17:01 total**			**23590**	**16650**	**174997**	**9056**	**18923**	**2090**	**21517**	**266823**	**C**	**C**	**C**	**C**	**C**	**C**	**C**	**C**	**C**
**DPB1*17:01:01G total**	**17:01:01G total**			**23590**	**16650**	**174997**	**9056**	**18923**	**2090**	**21517**	**266823**	**C**	**C**	**C**	**C**	**C**	**C**	**C**	**C**	**C**
DPB1*17:01	17:01		17:01:01G	5476	4567	97644	5361	5466	503	4490	123507	C	C	C	C	C	C	C	C	C
DPB1*17:01P	17:01P			0	0	17	0	0	0	0	17			WD					WD	WD
DPB1*17:01:01G	17:01:01G		17:01:01G	15725	11751	69830	3375	12401	1464	16276	130822	C	C	C	C	C	C	C	C	C
DPB1*17:01:01	17:01:01		17:01:01G	252	297	6996	241	238	17	176	8217	C	C	C	C	C	C	C	C	C

Supplemental Table 16: HLA-DPB1 Allele Summary [a]				Allele Count by Population Group [b]								3.0.0 CIWD Category by Population Group [c]								
Allele	Genomic Typing	Allele ID	G group	AFA	API	EURO	MENA	HIS	NAM	UNK	Total	AFA	API	EURO	MENA	HIS	NAM	UNK	Total	Highest Frequency
DPB1*17:01:01:01	17:01:01:01	HLA00534	17:01:01G	0	0	7	17	0	0	0	24			WD	WD				WD	WD
DPB1*131:01	131:01	HLA06078	17:01:01G	2137	35	487	62	818	106	573	4218	C	I	I	C	C	C	C	C	C
DPB1*460:01	460:01	HLA13713	17:01:01G	0	0	16	0	0	0	2	18			WD					WD	WD
DPB1*17:CODE	17:CODE			29	6	74	0	25	0	28	162	NA	NA	NA	NA	NA	NA	NA	NA	NA
DPB1*18:01	18:01	HLA00535		18862	89	903	55	2648	531	4489	27577	C	I	I	C	C	C	C	C	C
DPB1*18:CODE	18:CODE			5	0	0	0	1	1	1	8	NA	NA	NA	NA	NA	NA	NA	NA	NA
**DPB1*19:01 total**	**19:01 total**			**964**	**3039**	**73496**	**623**	**2310**	**276**	**5678**	**86386**	**C**	**C**	**C**	**C**	**C**	**C**	**C**	**C**	**C**
**DPB1*19:01:01G total**	**19:01:01G total**			**964**	**3039**	**73496**	**623**	**2310**	**276**	**5678**	**86386**	**C**	**C**	**C**	**C**	**C**	**C**	**C**	**C**	**C**
DPB1*19:01	19:01		19:01:01G	162	918	34654	317	687	79	851	37668	C	C	C	C	C	C	C	C	C
DPB1*19:01P	19:01P			0	0	2	0	0	0	0	2									
DPB1*19:01:01G	19:01:01G		19:01:01G	669	2118	38420	294	1583	194	4758	48036	C	C	C	C	C	C	C	C	C
DPB1*106:01	106:01	HLA02048	19:01:01G	132	3	202	4	25	2	43	411	C		I		I		I	I	C
DPB1*533:01	533:01	HLA14468	19:01:01G	0	0	3	0	0	0	0	3									
DPB1*535:01	535:01	HLA14470	19:01:01G	1	0	215	8	15	1	26	266			I	WD	I		I	I	I
DPB1*19:CODE	19:CODE			0	0	1	0	0	0	3	4	NA	NA	NA	NA	NA	NA	NA	NA	NA
**DPB1*20:01 total**	**20:01 total**			**322**	**235**	**44044**	**102**	**1184**	**150**	**3813**	**49850**	**C**	**C**	**C**	**C**	**C**	**C**	**C**	**C**	**C**
DPB1*20:01	20:01			36	6	2503	7	192	19	220	2983	C	WD	C	WD	C	C	C	C	C
DPB1*20:01P	20:01P			0	0	0	0	0	0	1	1									
**DPB1*20:01:01G total**	**20:01:01G total**			**286**	**229**	**41541**	**95**	**992**	**131**	**3592**	**46866**	**C**	**C**	**C**	**C**	**C**	**C**	**C**	**C**	**C**
DPB1*20:01:01G	20:01:01G		20:01:01G	0	1	0	0	0	0	0	1									
DPB1*20:01:01	20:01:01	HLA00537	20:01:01G	286	228	41541	95	992	131	3592	46865	C	C	C	C	C	C	C	C	C
DPB1*20:CODE	20:CODE			0	1	21	0	6	0	2	30	NA	NA	NA	NA	NA	NA	NA	NA	NA
DPB1*21:01	21:01	HLA00539		12	7875	203	14	22	7	591	8724	WD	C	I	WD	I	C	C	C	C
DPB1*21:CODE	21:CODE			0	20	0	0	0	0	1	21	NA	NA	NA	NA	NA	NA	NA	NA	NA
DPB1*22:01	22:01	HLA00540		0	12	7	0	0	0	4	23		I	WD					WD	I
**DPB1*23:01 total**	**23:01 total**			**315**	**1168**	**84286**	**2178**	**1913**	**161**	**4237**	**94258**	**C**	**C**	**C**	**C**	**C**	**C**	**C**	**C**	**C**
DPB1*23:01	23:01			17	88	14163	625	46	6	145	15090	WD	I	C	C	I	C	C	C	C
**DPB1*23:01:01G total**	**23:01:01G total**			**298**	**1080**	**70114**	**1553**	**1867**	**155**	**4092**	**79159**	**C**	**C**	**C**	**C**	**C**	**C**	**C**	**C**	**C**
DPB1*23:01:01G	23:01:01G		23:01:01G	172	778	28121	543	1218	101	3182	34115	C	C	C	C	C	C	C	C	C
DPB1*23:01:01	23:01:01	HLA00541	23:01:01G	113	297	40736	1002	610	49	842	43649	C	C	C	C	C	C	C	C	C
DPB1*138:01	138:01	HLA07798	23:01:01G	13	5	1257	8	39	5	68	1395	WD	WD	C	WD	I	WD	I	I	C

Supplemental Table 16: HLA-DPB1 Allele Summary [a]				Allele Count by Population Group [b]								3.0.0 CIWD Category by Population Group [c]								
Allele	Genomic Typing	Allele ID	G group	AFA	API	EURO	MENA	HIS	NAM	UNK	Total	AFA	API	EURO	MENA	HIS	NAM	UNK	Total	Highest Frequency
DPB1*23:01:02	23:01:02	HLA11293		0	0	9	0	0	0	0	9			WD					WD	WD
DPB1*23:CODE	23:CODE			1	0	33	1	6	0	8	49	NA	NA	NA	NA	NA	NA	NA	NA	NA
DPB1*24:01	24:01	HLA00542		4	4	443	13	14	0	69	547			I	WD	I		I	I	I
DPB1*25:01	25:01	HLA00543		3	36	409	1	7	1	33	490		I	I		I		I	I	I
DPB1*25:CODE	25:CODE			0	0	7	0	0	0	0	7	NA	NA	NA	NA	NA	NA	NA	NA	NA
**DPB1*26:01 total**	**26:01 total**			**148**	**39869**	**1877**	**523**	**75**	**120**	**1101**	**43713**	**C**	**C**	**C**	**C**	**C**	**C**	**C**	**C**	**C**
DPB1*26:01	26:01			14	359	83	38	12	11	35	552	WD	C	WD	C	I	C	I	I	C
DPB1*26:01P	26:01P			0	1	0	0	0	0	0	1									
DPB1*26:01:02	26:01:02	HLA00545		134	39509	1794	485	63	109	1066	43160	C	C	C	C	C	C	C	C	C
DPB1*26:CODE	26:CODE			0	10	2	0	0	0	0	12	NA	NA	NA	NA	NA	NA	NA	NA	NA
DPB1*27:01	27:01	HLA00546		51	3	84	0	444	104	130	816	C		WD		C	C	C	I	C
DPB1*27:CODE	27:CODE			7	0	0	0	1	0	3	11	NA	NA	NA	NA	NA	NA	NA	NA	NA
**DPB1*28:01 total**	**28:01 total**			**190**	**4095**	**283**	**83**	**38**	**10**	**324**	**5023**	**C**	**C**	**I**	**C**	**I**	**C**	**C**	**C**	**C**
**DPB1*28:01:01G total**	**28:01:01G total**			**190**	**4095**	**283**	**83**	**38**	**10**	**324**	**5023**	**C**	**C**	**I**	**C**	**I**	**C**	**C**	**C**	**C**
DPB1*28:01	28:01	HLA00547	28:01:01G	67	457	39	12	13	4	100	692	C	C	WD	WD	I		C	I	C
DPB1*28:01:01G	28:01:01G		28:01:01G	58	2564	110	20	13	3	94	2862	C	C	I	WD	I		I	C	C
DPB1*296:01	296:01	HLA11552	28:01:01G	65	1074	134	51	12	3	130	1469	C	C	I	C	I		C	C	C
DPB1*29:01	29:01	HLA00548		819	716	150	13	394	111	323	2526	C	C	I	WD	C	C	C	C	C
**DPB1*30:01 total**	**30:01 total**			**2059**	**44**	**1426**	**281**	**365**	**31**	**666**	**4872**	**C**	**I**	**C**	**C**	**C**	**C**	**C**	**C**	**C**
**DPB1*30:01:01G total**	**30:01:01G total**			**2059**	**44**	**1426**	**281**	**365**	**31**	**666**	**4872**	**C**	**I**	**C**	**C**	**C**	**C**	**C**	**C**	**C**
DPB1*30:01	30:01		30:01:01G	2059	43	1426	281	365	31	666	4871	C	I	C	C	C	C	C	C	C
DPB1*30:01:01G	30:01:01G		30:01:01G	0	1	0	0	0	0	0	1									
DPB1*30:CODE	30:CODE			8	0	0	0	0	0	0	8	NA	NA	NA	NA	NA	NA	NA	NA	NA
DPB1*31:01	31:01	HLA00550		3	885	55	2	6	0	136	1087		C	WD		I		C	I	C
**DPB1*33:01 total**	**33:01 total**			**2**	**67**	**398**	**15**	**13**	**1**	**25**	**521**		**I**	**I**	**WD**	**I**		**I**	**I**	**I**
**DPB1*33:01:01G total**	**33:01:01G total**			**2**	**67**	**398**	**15**	**13**	**1**	**25**	**521**		**I**	**I**	**WD**	**I**		**I**	**I**	**I**
DPB1*33:01	33:01		33:01:01G	2	54	383	15	13	1	24	492		I	I	WD	I		I	I	I
DPB1*33:01:01G	33:01:01G		33:01:01G	0	13	14	0	0	0	1	28		I	WD					WD	I
DPB1*33:01:01	33:01:01		33:01:01G	0	0	1	0	0	0	0	1									
DPB1*33:CODE	33:CODE			0	0	2	0	0	0	0	2	NA	NA	NA	NA	NA	NA	NA	NA	NA
**DPB1*34:01 total**	**34:01 total**			**472**	**146**	**3849**	**327**	**280**	**27**	**518**	**5619**	**C**	**C**	**C**	**C**	**C**	**C**	**C**	**C**	**C**

Supplemental Table 16: HLA-DPB1 Allele Summary [a]				Allele Count by Population Group [b]								3.0.0 CIWD Category by Population Group [c]								
Allele	Genomic Typing	Allele ID	G group	AFA	API	EURO	MENA	HIS	NAM	UNK	Total	AFA	API	EURO	MENA	HIS	NAM	UNK	Total	Highest Frequency
**DPB1*34:01:01G total**	**34:01:01G total**			**472**	**146**	**3849**	**327**	**280**	**27**	**518**	**5619**	**C**	**C**	**C**	**C**	**C**	**C**	**C**	**C**	**C**
DPB1*34:01	34:01		34:01:01G	472	137	3849	327	280	27	518	5610	C	C	C	C	C	C	C	C	C
DPB1*34:01:01G	34:01:01G		34:01:01G	0	9	0	0	0	0	0	9		WD						WD	WD
DPB1*34:CODE	34:CODE			0	0	0	0	8	0	1	9	NA	NA	NA	NA	NA	NA	NA	NA	NA
**DPB1*35:01 total**	**35:01 total**			**50**	**9**	**3022**	**32**	**257**	**16**	**315**	**3701**	**C**	**WD**	**C**	**C**	**C**	**C**	**C**	**C**	**C**
DPB1*35:01	35:01			5	0	180	2	44	0	25	256	WD		I		I		I	I	I
DPB1*35:01:01	35:01:01	HLA00554		45	9	2842	30	213	16	290	3445	C	WD	C	C	C	C	C	C	C
DPB1*35:CODE	35:CODE			1	0	10	1	1	0	1	14	NA	NA	NA	NA	NA	NA	NA	NA	NA
DPB1*36:01	36:01	HLA00555		4	142	1028	68	1	0	38	1281		C	I	C			I	I	C
DPB1*37:01	37:01	HLA00556		0	1	13	32	0	0	1	47			WD	C				WD	C
DPB1*37:CODE	37:CODE			0	0	0	2	0	0	0	2	NA	NA	NA	NA	NA	NA	NA	NA	NA
DPB1*38:01	38:01	HLA00557		0	415	9	0	1	0	44	469		C	WD				I	I	C
DPB1*38:CODE	38:CODE			0	1	0	0	0	0	0	1	NA	NA	NA	NA	NA	NA	NA	NA	NA
**DPB1*39:01 total**	**39:01 total**			**1645**	**26**	**132**	**16**	**207**	**28**	**374**	**2428**	**C**	**I**	**I**	**WD**	**C**	**C**	**C**	**C**	**C**
**DPB1*39:01:01G total**	**39:01:01G total**			**1645**	**26**	**132**	**16**	**207**	**28**	**374**	**2428**	**C**	**I**	**I**	**WD**	**C**	**C**	**C**	**C**	**C**
DPB1*39:01	39:01		39:01:01G	1476	20	121	16	189	26	326	2174	C	I	I	WD	C	C	C	C	C
DPB1*39:01:01G	39:01:01G		39:01:01G	43	6	6	0	3	1	10	69	C	WD	WD				I	WD	C
DPB1*584:01	584:01	HLA15505	39:01:01G	126	0	5	0	15	1	38	185	C		WD		I		I	I	C
DPB1*39:CODE	39:CODE			98	0	1	0	11	3	20	133	NA	NA	NA	NA	NA	NA	NA	NA	NA
**DPB1*40:01 total**	**40:01 total**			**4219**	**7**	**152**	**19**	**489**	**91**	**1009**	**5986**	**C**	**WD**	**I**	**WD**	**C**	**C**	**C**	**C**	**C**
**DPB1*40:01:01G total**	**40:01:01G total**			**4219**	**7**	**152**	**19**	**489**	**91**	**1009**	**5986**	**C**	**WD**	**I**	**WD**	**C**	**C**	**C**	**C**	**C**
DPB1*40:01	40:01		40:01:01G	4219	7	152	19	489	91	1009	5986	C	WD	I	WD	C	C	C	C	C
DPB1*40:CODE	40:CODE			10	0	0	0	1	0	2	13	NA	NA	NA	NA	NA	NA	NA	NA	NA
**DPB1*41:01 total**	**41:01 total**			**2**	**373**	**477**	**33**	**18**	**2**	**98**	**1003**		**C**	**I**	**C**	**I**		**C**	**I**	**C**
DPB1*41:01	41:01			0	22	34	2	3	0	9	70		I	WD				WD	WD	I
**DPB1*41:01:01G total**	**41:01:01G total**			**2**	**351**	**443**	**31**	**15**	**2**	**89**	**933**		**C**	**I**	**C**	**I**		**I**	**I**	**C**
DPB1*41:01:01G	41:01:01G		41:01:01G	0	1	0	0	0	0	0	1									
DPB1*41:01:01	41:01:01		41:01:01G	2	350	443	31	15	2	89	932		C	I	C	I		I	I	C
DPB1*41:CODE	41:CODE			0	0	3	0	0	0	1	4	NA	NA	NA	NA	NA	NA	NA	NA	NA
DPB1*45:01	45:01	HLA00564		8	850	2185	297	128	7	339	3814	WD	C	C	C	C	C	C	C	C
DPB1*45:CODE	45:CODE			0	0	2	0	0	0	1	3	NA	NA	NA	NA	NA	NA	NA	NA	NA

Supplemental Table 16: HLA-DPB1 Allele Summary [a]				Allele Count by Population Group [b]								3.0.0 CIWD Category by Population Group [c]								
Allele	Genomic Typing	Allele ID	G group	AFA	API	EURO	MENA	HIS	NAM	UNK	Total	AFA	API	EURO	MENA	HIS	NAM	UNK	Total	Highest Frequency
**DPB1*46:01 total**	**46:01 total**			**118**	**524**	**959**	**32**	**25**	**4**	**82**	**1744**	**C**	**C**	**I**	**C**	**I**		**I**	**C**	**C**
DPB1*46:01	46:01			48	29	302	16	11	2	24	432	C	I	I	WD	I		I	I	C
DPB1*46:01:01	46:01:01	HLA00565		70	495	657	16	14	2	58	1312	C	C	I	WD	I		I	I	C
DPB1*46:CODE	46:CODE			2	0	3	0	0	0	1	6	NA	NA	NA	NA	NA	NA	NA	NA	NA
**DPB1*47:01 total**	**47:01 total**			**27**	**113**	**430**	**131**	**0**	**0**	**64**	**765**	**WD**	**C**	**I**	**C**			**I**	**I**	**C**
**DPB1*47:01:01G total**	**47:01:01G total**			**27**	**113**	**430**	**131**	**0**	**0**	**64**	**765**	**WD**	**C**	**I**	**C**			**I**	**I**	**C**
DPB1*47:01	47:01		47:01:01G	22	108	392	122	0	0	61	705	WD	I	I	C			I	I	C
DPB1*47:01:01G	47:01:01G		47:01:01G	2	5	37	4	0	0	1	49		WD	WD					WD	WD
DPB1*47:01:01	47:01:01		47:01:01G	3	0	1	5	0	0	2	11				WD				WD	WD
DPB1*47:CODE	47:CODE			1	1	0	0	1	0	0	3	NA	NA	NA	NA	NA	NA	NA	NA	NA
DPB1*48:01	48:01	HLA00567		1	349	33	21	7	1	50	462		C	WD	WD	I		I	I	C
DPB1*48:CODE	48:CODE			0	0	0	0	1	0	2	3	NA	NA	NA	NA	NA	NA	NA	NA	NA
**DPB1*49:01 total**	**49:01 total**			**143**	**9**	**68**	**10**	**307**	**15**	**72**	**624**	**C**	**WD**	**WD**	**WD**	**C**	**C**	**I**	**I**	**C**
**DPB1*49:01:01G total**	**49:01:01G total**			**143**	**9**	**68**	**10**	**307**	**15**	**72**	**624**	**C**	**WD**	**WD**	**WD**	**C**	**C**	**I**	**I**	**C**
DPB1*49:01	49:01		49:01:01G	138	9	62	8	297	14	70	598	C	WD	WD	WD	C	C	I	I	C
DPB1*49:01:01G	49:01:01G		49:01:01G	2	0	6	2	0	0	1	11			WD					WD	WD
DPB1*49:01:01	49:01:01		49:01:01G	3	0	0	0	10	1	1	15					I			WD	I
DPB1*49:CODE	49:CODE			0	0	1	0	2	0	0	3	NA	NA	NA	NA	NA	NA	NA	NA	NA
DPB1*50:01	50:01	HLA00569		3	7	1086	7	12	0	98	1213		WD	C	WD	I		C	I	C
DPB1*50:CODE	50:CODE			1	1	1	0	0	0	1	4	NA	NA	NA	NA	NA	NA	NA	NA	NA
DPB1*51:01	51:01	HLA00570		8	212	2173	45	40	10	121	2609	WD	C	C	C	I	C	C	C	C
DPB1*51:CODE	51:CODE			0	1	9	0	0	0	0	10	NA	NA	NA	NA	NA	NA	NA	NA	NA
DPB1*52:01	52:01	HLA00571		13	14	243	0	1	0	38	309	WD	I	I				I	I	I
DPB1*52:CODE	52:CODE			0	0	0	0	0	0	1	1	NA	NA	NA	NA	NA	NA	NA	NA	NA
DPB1*53:01	53:01	HLA00572		51	3	4	0	16	6	13	93	C				I	C	I	WD	C
DPB1*54:01	54:01	HLA00573		15	0	29	0	0	0	5	49	WD		WD				WD	WD	WD
**DPB1*55:01 total**	**55:01 total**			**340**	**15**	**60**	**12**	**62**	**1**	**63**	**553**	**C**	**I**	**WD**	**WD**	**C**		**I**	**I**	**C**
**DPB1*55:01:01G total**	**55:01:01G total**			**340**	**15**	**60**	**12**	**62**	**1**	**63**	**553**	**C**	**I**	**WD**	**WD**	**C**		**I**	**I**	**C**
DPB1*55:01	55:01		55:01:01G	319	10	58	12	62	1	60	522	C	WD	WD	WD	C		I	I	C
DPB1*55:01:01G	55:01:01G		55:01:01G	16	5	2	0	0	0	1	24	WD	WD						WD	WD
DPB1*55:01:01	55:01:01		55:01:01G	5	0	0	0	0	0	2	7	WD							WD	WD

Supplemental Table 16: HLA-DPB1 Allele Summary [a]				Allele Count by Population Group [b]								3.0.0 CIWD Category by Population Group [c]								
Allele	Genomic Typing	Allele ID	G group	AFA	API	EURO	MENA	HIS	NAM	UNK	Total	AFA	API	EURO	MENA	HIS	NAM	UNK	Total	Highest Frequency
DPB1*55:CODE	55:CODE			1	1	1	0	1	0	0	4	NA	NA	NA	NA	NA	NA	NA	NA	NA
DPB1*56:01	56:01	HLA00575		0	2	33	0	0	0	1	36			WD					WD	WD
**DPB1*57:01 total**	**57:01 total**			**7**	**25**	**129**	**6**	**15**	**0**	**11**	**193**	**WD**	**I**	**I**	**WD**	**I**		**I**	**I**	**I**
**DPB1*57:01:01G total**	**57:01:01G total**			**7**	**25**	**129**	**6**	**15**	**0**	**11**	**193**	**WD**	**I**	**I**	**WD**	**I**		**I**	**I**	**I**
DPB1*57:01	57:01	HLA00576	57:01:01G	7	19	114	5	14	0	11	170	WD	I	I	WD	I		I	I	I
DPB1*57:01:01G	57:01:01G		57:01:01G	0	6	15	1	1	0	0	23		WD	WD					WD	WD
DPB1*58:CODE	58:CODE			0	1	0	0	0	0	0	1	NA	NA	NA	NA	NA	NA	NA	NA	NA
DPB1*59:01	59:01	HLA00578		3	7	1300	4	16	2	52	1384		WD	C		I		I	I	C
DPB1*59:CODE	59:CODE			1	0	0	0	0	0	0	1	NA	NA	NA	NA	NA	NA	NA	NA	NA
DPB1*60:01	60:01	HLA00579		6	0	1	0	0	0	1	8	WD							WD	WD
DPB1*61:01N	61:01N	HLA00580		81	1	1	0	13	0	24	120	C				I		I	WD	C
DPB1*62:01	62:01	HLA00581		4	0	0	0	0	0	0	4									
DPB1*63:01	63:01	HLA00582		176	6	624	48	289	18	127	1288	C	WD	I	C	C	C	C	I	C
DPB1*63:CODE	63:CODE			0	0	3	0	0	0	0	3	NA	NA	NA	NA	NA	NA	NA	NA	NA
DPB1*64:01N	64:01N	HLA00583		2	0	57	0	3	0	10	72			WD				I	WD	I
**DPB1*65:01 total**	**65:01 total**			**3**	**5**	**26**	**0**	**5**	**0**	**0**	**39**		**WD**	**WD**		**WD**			**WD**	**WD**
DPB1*65:01	65:01			3	5	23	0	5	0	0	36		WD	WD		WD			WD	WD
DPB1*65:01:01	65:01:01	HLA00584		0	0	3	0	0	0	0	3									
DPB1*65:CODE	65:CODE			1	0	0	0	0	0	1	2	NA	NA	NA	NA	NA	NA	NA	NA	NA
DPB1*66:01	66:01	HLA00585		0	0	24	0	0	0	5	29			WD				WD	WD	WD
DPB1*67:01	67:01	HLA00586		0	2	11	35	0	0	3	51			WD	C				WD	C
DPB1*68:01	68:01	HLA00587		0	4	47	0	1	2	1	55			WD					WD	WD
DPB1*68:CODE	68:CODE			0	1	0	0	0	0	0	1	NA	NA	NA	NA	NA	NA	NA	NA	NA
**DPB1*69:01 total**	**69:01 total**			**25**	**0**	**6**	**0**	**5**	**1**	**4**	**41**	**WD**		**WD**		**WD**			**WD**	**WD**
**DPB1*69:01:01G total**	**69:01:01G total**			**25**	**0**	**6**	**0**	**5**	**1**	**4**	**41**	**WD**		**WD**		**WD**			**WD**	**WD**
DPB1*69:01	69:01		69:01:01G	25	0	6	0	5	1	4	41	WD		WD		WD			WD	WD
DPB1*70:01	70:01	HLA00589		0	11	13	0	1	0	0	25		I	WD					WD	I
**DPB1*71:01 total**	**71:01 total**			**36**	**127**	**915**	**43**	**44**	**3**	**84**	**1252**	**C**	**C**	**I**	**C**	**I**		**I**	**I**	**C**
DPB1*71:01	71:01			31	89	818	39	40	3	80	1100	WD	I	I	C	I		I	I	C
DPB1*71:01:01	71:01:01	HLA00590		5	38	97	4	4	0	4	152	WD	I	WD					I	I
DPB1*71:CODE	71:CODE			1	0	1	0	0	0	0	2	NA	NA	NA	NA	NA	NA	NA	NA	NA

Supplemental Table 16: HLA-DPB1 Allele Summary [a]				Allele Count by Population Group [b]								3.0.0 CIWD Category by Population Group [c]								
Allele	Genomic Typing	Allele ID	G group	AFA	API	EURO	MENA	HIS	NAM	UNK	Total	AFA	API	EURO	MENA	HIS	NAM	UNK	Total	Highest Frequency
**DPB1*72:01 total**	**72:01 total**			**4**	**21**	**59**	**9**	**2**	**0**	**10**	**105**		**I**	**WD**	**WD**			**I**	**WD**	**I**
**DPB1*72:01:01G total**	**72:01:01G total**			**4**	**21**	**59**	**9**	**2**	**0**	**10**	**105**		**I**	**WD**	**WD**			**I**	**WD**	**I**
DPB1*72:01	72:01		72:01:01G	4	18	58	9	2	0	9	100		I	WD	WD			WD	WD	I
DPB1*72:01:01G	72:01:01G		72:01:01G	0	3	1	0	0	0	1	5								WD	WD
DPB1*72:CODE	72:CODE			0	0	1	0	0	0	0	1	NA	NA	NA	NA	NA	NA	NA	NA	NA
DPB1*73:01	73:01	HLA00592		0	0	1	0	0	0	0	1									
DPB1*74:01	74:01	HLA00593		24	1	0	0	0	0	5	30	WD						WD	WD	WD
DPB1*74:CODE	74:CODE			0	0	0	0	1	0	0	1	NA	NA	NA	NA	NA	NA	NA	NA	NA
DPB1*75:01	75:01	HLA00594		1	1	50	10	1	0	3	66			WD	WD				WD	WD
DPB1*76:01	76:01	HLA00595		0	0	47	3	0	0	2	52			WD					WD	WD
DPB1*76:CODE	76:CODE			0	0	0	0	1	0	0	1	NA	NA	NA	NA	NA	NA	NA	NA	NA
DPB1*77:01	77:01	HLA00596		1	0	61	0	0	0	5	67			WD				WD	WD	WD
DPB1*78:01	78:01	HLA00597		1	64	270	26	10	1	25	397		I	I	WD	I		I	I	I
DPB1*79:01	79:01	HLA00598		0	0	33	1	6	1	8	49			WD		I		WD	WD	I
DPB1*80:01	80:01	HLA00599		155	36	183	16	59	13	63	525	C	I	I	WD	C	C	I	I	C
DPB1*80:CODE	80:CODE			0	0	0	0	1	0	0	1	NA	NA	NA	NA	NA	NA	NA	NA	NA
**DPB1*81:01 total**	**81:01 total**			**7**	**80**	**938**	**59**	**23**	**3**	**62**	**1172**	**WD**	**I**	**I**	**C**	**I**		**I**	**I**	**C**
**DPB1*81:01:01G total**	**81:01:01G total**			**7**	**80**	**938**	**59**	**23**	**3**	**62**	**1172**	**WD**	**I**	**I**	**C**	**I**		**I**	**I**	**C**
DPB1*81:01	81:01		81:01:01G	7	66	882	58	22	3	60	1098	WD	I	I	C	I		I	I	C
DPB1*81:01:01G	81:01:01G		81:01:01G	0	14	50	1	1	0	2	68		I	WD					WD	I
DPB1*81:01:01	81:01:01		81:01:01G	0	0	6	0	0	0	0	6			WD					WD	WD
DPB1*82:01	82:01	HLA01002		0	0	2	0	12	0	2	16					I			WD	I
DPB1*82:CODE	82:CODE			13	0	0	0	2	0	5	20	NA	NA	NA	NA	NA	NA	NA	NA	NA
DPB1*83:01	83:01	HLA01084		2	41	18	0	1	0	4	66		I	WD					WD	I
**DPB1*85:01 total**	**85:01 total**			**5077**	**14**	**208**	**7**	**571**	**114**	**1151**	**7142**	**C**	**I**	**I**	**WD**	**C**	**C**	**C**	**C**	**C**
DPB1*85:01	85:01			4976	11	198	6	562	113	1124	6990	C	I	I	WD	C	C	C	C	C
**DPB1*85:01:01G total**	**85:01:01G total**			**101**	**3**	**10**	**1**	**9**	**1**	**27**	**152**	**C**		**WD**		**I**		**I**	**I**	**C**
DPB1*85:01:01G	85:01:01G		85:01:01G	52	3	10	1	1	1	12	80	C		WD				I	WD	C
DPB1*85:01:01	85:01:01		85:01:01G	49	0	0	0	8	0	15	72	C				I		I	WD	C
DPB1*85:CODE	85:CODE			4	0	0	0	0	0	5	9	NA	NA	NA	NA	NA	NA	NA	NA	NA
DPB1*86:01	86:01	HLA01234		5	0	4	2	3	1	1	16	WD							WD	WD

Supplemental Table 16: HLA-DPB1 Allele Summary [a]				Allele Count by Population Group [b]								3.0.0 CIWD Category by Population Group [c]								
Allele	Genomic Typing	Allele ID	G group	AFA	API	EURO	MENA	HIS	NAM	UNK	Total	AFA	API	EURO	MENA	HIS	NAM	UNK	Total	Highest Frequency
DPB1*87:01	87:01	HLA01308		0	0	58	0	1	0	5	64			WD				WD	WD	WD
DPB1*88:01	88:01	HLA01304		3	10	533	349	5	0	36	936		WD	I	C	WD		I	I	C
DPB1*88:CODE	88:CODE			2	0	0	0	1	0	0	3	NA	NA	NA	NA	NA	NA	NA	NA	NA
DPB1*89:01	89:01	HLA01360		4	3	19	0	1	0	0	27			WD					WD	WD
**DPB1*90:01 total**	**90:01 total**			**4**	**3**	**86**	**0**	**3**	**0**	**7**	**103**			**WD**				**WD**	**WD**	**WD**
DPB1*90:01	90:01			4	3	77	0	3	0	7	94			WD				WD	WD	WD
DPB1*90:01:01	90:01:01	HLA01441		0	0	9	0	0	0	0	9			WD					WD	WD
**DPB1*91:01 total**	**91:01 total**			**1**	**660**	**36**	**33**	**2**	**0**	**42**	**774**		**C**	**WD**	**C**			**I**	**I**	**C**
**DPB1*91:01:01G total**	**91:01:01G total**			**1**	**660**	**36**	**33**	**2**	**0**	**42**	**774**		**C**	**WD**	**C**			**I**	**I**	**C**
DPB1*91:01	91:01		91:01:01G	1	532	36	31	2	0	35	637		C	WD	C			I	I	C
DPB1*91:01:01G	91:01:01G		91:01:01G	0	128	0	0	0	0	0	128		C						WD	C
DPB1*91:01:01	91:01:01		91:01:01G	0	0	0	2	0	0	7	9							WD	WD	WD
DPB1*92:01	92:01	HLA01597		1	25	107	0	1	0	2	136		I	I					WD	I
DPB1*93:01	93:01	HLA01637		3	755	31	0	6	1	27	823		C	WD		I		I	I	C
DPB1*94:01	94:01	HLA01669		1	2	1	0	0	0	0	4									
DPB1*95:01	95:01	HLA01670		5	0	0	0	0	0	1	6	WD							WD	WD
DPB1*96:01	96:01	HLA01684		4	0	0	0	0	0	3	7								WD	WD
DPB1*98:01	98:01	HLA01762		11	2	5	0	0	0	1	19	WD		WD					WD	WD
DPB1*99:01	99:01	HLA01830		2	2	26	22	14	2	6	74			WD	WD	I		WD	WD	I
DPB1*99:CODE	99:CODE			0	0	0	0	1	0	0	1	NA	NA	NA	NA	NA	NA	NA	NA	NA
DPB1*100:01	100:01	HLA01837		0	89	16	0	28	2	14	149		I	WD		I		I	I	I
DPB1*102:01	102:01	HLA01947		0	0	23	0	0	0	1	24			WD					WD	WD
DPB1*102:CODE	102:CODE			0	0	0	0	0	0	1	1	NA	NA	NA	NA	NA	NA	NA	NA	NA
DPB1*103:01	103:01	HLA02042		5	0	8	0	0	0	3	16	WD		WD					WD	WD
DPB1*104:CODE	104:CODE			0	0	0	2	0	0	1	3	NA	NA	NA	NA	NA	NA	NA	NA	NA
DPB1*105:CODE	105:CODE			46	1	12	0	8	1	17	85	NA	NA	NA	NA	NA	NA	NA	NA	NA
DPB1*106:CODE	106:CODE			0	0	10	1	2	0	1	14	NA	NA	NA	NA	NA	NA	NA	NA	NA
DPB1*108:01	108:01	HLA02080		0	6	82	0	17	0	4	109		WD	WD		I			WD	I
DPB1*109:01	109:01	HLA02115		1	2	213	0	2	0	8	226			I				WD	I	I
DPB1*109:CODE	109:CODE			0	0	1	0	1	0	1	3	NA	NA	NA	NA	NA	NA	NA	NA	NA
DPB1*110:01	110:01	HLA02243		1	1	28	1	1	0	0	32			WD					WD	WD

Supplemental Table 16: HLA-DPB1 Allele Summary [a]				Allele Count by Population Group [b]								3.0.0 CIWD Category by Population Group [c]								
Allele	Genomic Typing	Allele ID	G group	AFA	API	EURO	MENA	HIS	NAM	UNK	Total	AFA	API	EURO	MENA	HIS	NAM	UNK	Total	Highest Frequency
DPB1*110:CODE	110:CODE			0	0	1	0	0	0	0	1	NA	NA	NA	NA	NA	NA	NA	NA	NA
DPB1*112:01	112:01	HLA02403		1	2	36	0	3	0	3	45			WD					WD	WD
DPB1*113:01	113:01	HLA02447		0	24	16	9	0	0	0	49		I	WD	WD				WD	I
DPB1*115:01	115:01	HLA02493		0	2	201	4	2	0	9	218			I				WD	I	I
DPB1*116:01	116:01	HLA02579		0	2	62	1	3	0	5	73			WD				WD	WD	WD
DPB1*117:01	117:01	HLA02738		1	1	0	0	0	0	1	3									
DPB1*119:01	119:01	HLA03490		0	0	1	0	0	0	0	1									
DPB1*120:01N	120:01N	HLA03492		0	1	17	0	0	0	1	19			WD					WD	WD
DPB1*121:01	121:01	HLA03575		0	1	14	0	0	0	0	15			WD					WD	WD
DPB1*122:01	122:01	HLA03822		9	0	9	0	0	0	1	19	WD		WD					WD	WD
DPB1*122:CODE	122:CODE			0	0	0	0	0	0	1	1	NA	NA	NA	NA	NA	NA	NA	NA	NA
DPB1*123:01	123:01	HLA03838		0	0	26	0	0	0	1	27			WD					WD	WD
DPB1*124:CODE	124:CODE			0	0	8	0	1	0	2	11	NA	NA	NA	NA	NA	NA	NA	NA	NA
DPB1*125:01	125:01	HLA04398		0	77	6	1	0	0	3	87		I	WD					WD	I
DPB1*127:01	127:01	HLA05357		1	1	172	0	0	0	1	175			I					I	I
DPB1*128:01	128:01	HLA05358		0	1	436	0	6	0	12	455			I		I		I	I	I
DPB1*128:CODE	128:CODE			0	0	0	0	0	0	1	1	NA	NA	NA	NA	NA	NA	NA	NA	NA
DPB1*129:01	129:01	HLA05760		0	1	20	2	0	0	2	25			WD					WD	WD
DPB1*129:CODE	129:CODE			0	0	0	0	1	0	0	1	NA	NA	NA	NA	NA	NA	NA	NA	NA
DPB1*130:01	130:01	HLA06049		0	2	639	0	3	0	47	691			I				I	I	I
DPB1*130:CODE	130:CODE			0	0	2	0	0	0	0	2	NA	NA	NA	NA	NA	NA	NA	NA	NA
DPB1*131:CODE	131:CODE			14	0	3	0	4	1	7	29	NA	NA	NA	NA	NA	NA	NA	NA	NA
DPB1*132:01	132:01	HLA06140		2	12	35	6	53	3	7	118		I	WD	WD	I		WD	WD	I
DPB1*134:01	134:01	HLA06673		0	0	11	0	0	0	1	12			WD					WD	WD
DPB1*136:01	136:01	HLA07366		0	1	41	77	0	0	4	123			WD	C				WD	C
DPB1*137:01	137:01	HLA07534		0	1	19	0	4	1	5	30			WD				WD	WD	WD
DPB1*137:CODE	137:CODE			0	0	1	0	0	0	0	1	NA	NA	NA	NA	NA	NA	NA	NA	NA
DPB1*138:CODE	138:CODE			0	0	0	0	1	0	0	1	NA	NA	NA	NA	NA	NA	NA	NA	NA
DPB1*139:01	139:01	HLA07816		0	21	0	0	0	0	0	21		I						WD	I
DPB1*140:01	140:01	HLA08416		0	1	0	0	0	0	0	1									
DPB1*142:01	142:01	HLA09132		0	6	0	0	0	0	0	6		WD						WD	WD

Supplemental Table 16: HLA-DPB1 Allele Summary [a]				Allele Count by Population Group [b]								3.0.0 CIWD Category by Population Group [c]								
Allele	Genomic Typing	Allele ID	G group	AFA	API	EURO	MENA	HIS	NAM	UNK	Total	AFA	API	EURO	MENA	HIS	NAM	UNK	Total	Highest Frequency
DPB1*143:01	143:01	HLA09385		6	0	0	0	0	0	1	7	WD							WD	WD
DPB1*144:01	144:01	HLA09386		0	0	43	0	1	0	0	44			WD					WD	WD
DPB1*145:01	145:01	HLA09477		0	5	5	0	0	0	20	30		WD	WD				I	WD	I
DPB1*146:01	146:01	HLA09388		0	0	23	0	0	0	3	26			WD					WD	WD
DPB1*147:01	147:01	HLA09390		0	0	3	0	5	0	0	8					WD			WD	WD
DPB1*149:01	149:01	HLA09392		0	0	20	0	0	1	11	32			WD				I	WD	I
DPB1*150:01	150:01	HLA09395		0	197	9	1	1	0	1	209		C	WD					I	C
DPB1*150:CODE	150:CODE			0	0	1	0	0	0	0	1	NA	NA	NA	NA	NA	NA	NA	NA	NA
DPB1*151:01	151:01	HLA09396		0	0	18	0	0	0	0	18			WD					WD	WD
DPB1*152:01	152:01	HLA09397		0	0	45	0	0	0	1	46			WD					WD	WD
DPB1*152:CODE	152:CODE			0	0	1	0	0	0	0	1	NA	NA	NA	NA	NA	NA	NA	NA	NA
DPB1*153:01	153:01	HLA09398		0	0	1	1	26	4	7	39					I		WD	WD	I
DPB1*154:01N	154:01N	HLA09400		0	0	39	0	0	0	0	39			WD					WD	WD
**DPB1*155:01 total**	**155:01 total**			**10**	**0**	**22**	**2**	**0**	**1**	**1**	**36**	**WD**		**WD**					**WD**	**WD**
DPB1*155:01	155:01			7	0	0	0	0	0	1	8	WD							WD	WD
DPB1*155:01:01	155:01:01	HLA09401		3	0	0	1	0	1	0	5								WD	WD
DPB1*155:01:02	155:01:02	HLA13905		0	0	22	1	0	0	0	23			WD					WD	WD
DPB1*156:01	156:01	HLA09402		0	1	9	0	12	0	2	24			WD		I			WD	I
DPB1*157:01	157:01	HLA09403		0	0	44	0	0	0	0	44			WD					WD	WD
DPB1*158:01	158:01	HLA09404		0	0	41	0	0	0	3	44			WD					WD	WD
DPB1*159:01N	159:01N	HLA09405		1	0	0	0	1	0	0	2									
DPB1*160:01	160:01	HLA09406		0	1	0	0	0	0	0	1									
DPB1*161:01N	161:01N	HLA09408		0	0	5	0	0	0	2	7			WD					WD	WD
DPB1*163:01	163:01	HLA09767		0	0	14	0	0	0	0	14			WD					WD	WD
DPB1*164:01	164:01	HLA09768		0	0	5	0	0	0	0	5			WD					WD	WD
DPB1*164:CODE	164:CODE			0	0	2	0	0	0	0	2	NA	NA	NA	NA	NA	NA	NA	NA	NA
DPB1*165:01	165:01	HLA09769		1	0	9	0	0	0	0	10			WD					WD	WD
DPB1*166:01	166:01	HLA10026		0	12	0	0	0	0	3	15		I						WD	I
DPB1*167:01	167:01	HLA10263		0	0	1	0	0	0	0	1									
DPB1*170:01	170:01	HLA10324		0	0	10	0	0	0	2	12			WD					WD	WD
DPB1*171:CODE	171:CODE			0	6	0	0	0	0	2	8	NA	NA	NA	NA	NA	NA	NA	NA	NA

Supplemental Table 16: HLA-DPB1 Allele Summary [a]				Allele Count by Population Group [b]								3.0.0 CIWD Category by Population Group [c]								
Allele	Genomic Typing	Allele ID	G group	AFA	API	EURO	MENA	HIS	NAM	UNK	Total	AFA	API	EURO	MENA	HIS	NAM	UNK	Total	Highest Frequency
DPB1*172:01	172:01	HLA10326		0	3	22	0	5	0	3	33			WD		WD			WD	WD
DPB1*173:01	173:01	HLA10327		0	21	9	17	0	0	2	49		I	WD	WD				WD	I
DPB1*174:01	174:01	HLA10328		0	0	1	0	0	0	0	1									
DPB1*175:01	175:01	HLA10331		0	0	0	0	0	0	1	1									
DPB1*176:01	176:01	HLA10333		0	0	4	0	0	0	0	4									
DPB1*177:01	177:01	HLA10334		3	0	29	0	2	0	2	36			WD					WD	WD
DPB1*178:01	178:01	HLA10336		0	0	103	0	0	0	3	106			WD					WD	WD
DPB1*178:CODE	178:CODE			0	0	1	0	0	0	0	1	NA	NA	NA	NA	NA	NA	NA	NA	NA
DPB1*179:01	179:01	HLA10337		0	0	6	0	4	0	3	13			WD					WD	WD
DPB1*180:01	180:01	HLA10338		0	0	1	0	0	0	0	1									
DPB1*181:01	181:01	HLA10339		0	0	1	5	0	0	0	6				WD				WD	WD
DPB1*182:01	182:01	HLA10341		0	9	39	2	1	0	2	53		WD	WD					WD	WD
DPB1*183:01	183:01	HLA10342		0	0	1	0	0	0	0	1									
DPB1*184:01	184:01	HLA10343		0	0	39	0	0	0	10	49			WD				I	WD	I
DPB1*184:CODE	184:CODE			0	1	0	0	0	0	0	1	NA	NA	NA	NA	NA	NA	NA	NA	NA
DPB1*185:01	185:01	HLA10344		0	0	16	0	0	0	12	28			WD				I	WD	I
DPB1*186:01	186:01	HLA10345		0	6	2	0	0	0	0	8		WD						WD	WD
DPB1*187:01	187:01	HLA10346		0	0	13	0	0	0	0	13			WD					WD	WD
DPB1*188:01	188:01	HLA10347		1	3	5	0	0	0	0	9			WD					WD	WD
DPB1*189:01	189:01	HLA10348		0	0	25	6	1	0	2	34			WD	WD				WD	WD
DPB1*190:01	190:01	HLA10349		0	0	241	0	0	0	0	241			I					I	I
DPB1*191:01	191:01	HLA10350		0	0	202	0	1	0	2	205			I					I	I
DPB1*192:01	192:01	HLA10351		0	37	1	0	0	0	0	38		I						WD	I
DPB1*193:01	193:01	HLA10352		0	0	2	0	0	0	0	2									
DPB1*194:01	194:01	HLA10354		0	0	1	0	0	0	0	1									
DPB1*195:01	195:01	HLA10355		0	0	1	0	0	0	1	2									
DPB1*195:CODE	195:CODE			0	0	0	0	0	0	1	1	NA	NA	NA	NA	NA	NA	NA	NA	NA
DPB1*196:01	196:01	HLA10357		0	0	1	0	0	0	0	1									
DPB1*198:01	198:01	HLA10360		0	0	1	0	0	0	0	1									
DPB1*198:CODE	198:CODE			0	0	0	0	0	0	1	1	NA	NA	NA	NA	NA	NA	NA	NA	NA
DPB1*199:01	199:01	HLA10361		0	0	9	0	0	0	4	13			WD					WD	WD

Supplemental Table 16: HLA-DPB1 Allele Summary [a]				Allele Count by Population Group [b]								3.0.0 CIWD Category by Population Group [c]								
Allele	Genomic Typing	Allele ID	G group	AFA	API	EURO	MENA	HIS	NAM	UNK	Total	AFA	API	EURO	MENA	HIS	NAM	UNK	Total	Highest Frequency
DPB1*200:01	200:01	HLA10362		0	1	5	1	0	0	2	9			WD					WD	WD
DPB1*200:CODE	200:CODE			1	0	0	0	0	0	0	1	NA	NA	NA	NA	NA	NA	NA	NA	NA
DPB1*201:01	201:01	HLA10363		1	2	40	5	2	0	0	50			WD	WD				WD	WD
DPB1*201:CODE	201:CODE			0	0	0	0	0	0	1	1	NA	NA	NA	NA	NA	NA	NA	NA	NA
**DPB1*203:01 total**	**203:01 total**			**0**	**0**	**0**	**0**	**6**	**0**	**2**	**8**					**I**			**WD**	**I**
DPB1*203:01	203:01			0	0	0	0	6	0	2	8					I			WD	I
DPB1*205:01	205:01	HLA10367		0	0	0	0	0	0	2	2									
DPB1*206:01	206:01	HLA10368		0	4	13	5	1	0	1	24			WD	WD				WD	WD
DPB1*207:01	207:01	HLA10369		0	0	3	0	0	0	2	5								WD	WD
DPB1*208:01	208:01	HLA10370		0	1	26	0	0	0	1	28			WD					WD	WD
DPB1*209:01	209:01	HLA10371		0	2	8	0	1	0	1	12			WD					WD	WD
DPB1*210:01	210:01	HLA10558		0	0	2	0	25	2	4	33					I			WD	I
DPB1*211:01	211:01	HLA10559		0	0	8	0	1	0	0	9			WD					WD	WD
DPB1*212:01	212:01	HLA10561		0	2	4	0	0	0	0	6								WD	WD
DPB1*214:01	214:01	HLA10943		1	4	84	1	1	0	2	93			WD					WD	WD
DPB1*215:01	215:01	HLA10944		0	25	36	0	0	0	6	67		I	WD				WD	WD	I
DPB1*216:01N	216:01N	HLA10946		0	0	2	0	0	0	0	2									
DPB1*217:01	217:01	HLA10947		0	0	0	0	0	0	2	2									
DPB1*217:CODE	217:CODE			0	1	0	0	0	0	0	1	NA	NA	NA	NA	NA	NA	NA	NA	NA
DPB1*218:01N	218:01N	HLA10949		0	0	11	0	0	0	0	11			WD					WD	WD
DPB1*220:01	220:01	HLA10951		0	0	23	0	0	0	2	25			WD					WD	WD
DPB1*221:01	221:01	HLA10953		0	3	5	0	1	0	0	9			WD					WD	WD
DPB1*222:01	222:01	HLA10954		0	0	13	0	0	0	12	25			WD				I	WD	I
DPB1*224:01	224:01	HLA11182		0	2	7	0	0	0	0	9			WD					WD	WD
DPB1*225:01	225:01	HLA11183		0	0	16	0	1	0	2	19			WD					WD	WD
DPB1*227:01	227:01	HLA11185		0	0	8	0	0	0	0	8			WD					WD	WD
DPB1*227:CODE	227:CODE			3	0	0	0	0	0	0	3	NA	NA	NA	NA	NA	NA	NA	NA	NA
DPB1*228:01	228:01	HLA11186		0	0	5	0	0	0	0	5			WD					WD	WD
DPB1*229:01	229:01	HLA11187		0	0	2	0	0	0	0	2									
DPB1*230:01	230:01	HLA11188		0	0	4	0	0	0	0	4									
DPB1*231:01	231:01	HLA11189		0	0	1	0	0	0	0	1									

Supplemental Table 16: HLA-DPB1 Allele Summary [a]				Allele Count by Population Group [b]								3.0.0 CIWD Category by Population Group [c]								
Allele	Genomic Typing	Allele ID	G group	AFA	API	EURO	MENA	HIS	NAM	UNK	Total	AFA	API	EURO	MENA	HIS	NAM	UNK	Total	Highest Frequency
DPB1*233:01	233:01	HLA11192		3	4	11	0	5	0	3	26			WD		WD			WD	WD
DPB1*234:01	234:01	HLA11193		0	2	1	0	0	0	1	4									
DPB1*235:01	235:01	HLA11194		0	0	5	0	0	0	0	5			WD					WD	WD
**DPB1*236:01 total**	**236:01 total**			**0**	**7**	**24**	**0**	**0**	**0**	**0**	**31**		**WD**	**WD**					**WD**	**WD**
DPB1*236:01	236:01			0	1	11	0	0	0	0	12			WD					WD	WD
DPB1*236:01:01	236:01:01	HLA11195		0	0	13	0	0	0	0	13			WD					WD	WD
DPB1*236:01:02	236:01:02	HLA13904		0	6	0	0	0	0	0	6		WD						WD	WD
DPB1*237:01	237:01	HLA11491		0	0	0	0	1	0	0	1									
DPB1*238:01	238:01	HLA11492		0	0	31	0	1	1	10	43			WD				I	WD	I
DPB1*239:01	239:01	HLA11493		0	0	3	0	0	0	0	3									
DPB1*240:01	240:01	HLA11494		1	0	1	0	8	1	3	14					I			WD	I
DPB1*241:01	241:01	HLA11495		2	0	0	0	0	0	1	3									
DPB1*242:01	242:01	HLA11496		0	29	0	0	0	1	1	31		I						WD	I
DPB1*244:01	244:01	HLA11509		0	0	10	8	1	0	0	19			WD	WD				WD	WD
DPB1*245:01	245:01	HLA11510		0	0	5	0	0	0	0	5			WD					WD	WD
DPB1*246:01	246:01	HLA11511		4	1	6	0	0	0	1	12			WD					WD	WD
DPB1*247:01	247:01	HLA11512		0	0	1	0	0	0	0	1									
DPB1*248:01	248:01	HLA11513		0	0	1	0	0	0	0	1									
DPB1*249:01	249:01	HLA11514		18	0	0	0	0	2	4	24	WD							WD	WD
DPB1*250:01	250:01	HLA11515		0	0	2	0	0	0	1	3									
DPB1*253:01	253:01	HLA11518		0	0	3	0	0	0	0	3									
DPB1*254:01	254:01	HLA11519		0	3	1	0	0	0	0	4									
DPB1*256:01	256:01	HLA11539		1	0	1	0	0	0	6	8							WD	WD	WD
DPB1*257:01	257:01	HLA11540		0	0	17	0	0	0	0	17			WD					WD	WD
DPB1*258:01	258:01	HLA11541		0	0	4	0	0	0	1	5								WD	WD
DPB1*259:01	259:01	HLA11542		0	1	25	0	0	0	0	26			WD					WD	WD
DPB1*260:01	260:01	HLA11543		0	0	68	0	0	0	0	68			WD					WD	WD
DPB1*261:01	261:01	HLA11544		0	0	2	0	0	0	0	2									
DPB1*262:01	262:01	HLA11499		0	0	4	0	0	0	0	4									
DPB1*263:01	263:01	HLA11500		0	0	1	0	0	0	0	1									
DPB1*264:01	264:01	HLA11501		0	0	5	0	0	0	1	6			WD					WD	WD

Supplemental Table 16: HLA-DPB1 Allele Summary [a]				Allele Count by Population Group [b]								3.0.0 CIWD Category by Population Group [c]								
Allele	Genomic Typing	Allele ID	G group	AFA	API	EURO	MENA	HIS	NAM	UNK	Total	AFA	API	EURO	MENA	HIS	NAM	UNK	Total	Highest Frequency
DPB1*265:01	265:01	HLA11502		0	0	45	0	0	0	0	45			WD					WD	WD
DPB1*266:01	266:01	HLA11503		0	4	14	0	0	0	0	18			WD					WD	WD
DPB1*269:01	269:01	HLA11506		0	17	2	0	0	0	0	19		I						WD	I
DPB1*270:01	270:01	HLA11507		0	0	9	0	0	0	0	9			WD					WD	WD
DPB1*271:01	271:01	HLA11521		1	3	27	1	10	0	2	44			WD		I			WD	I
DPB1*272:01	272:01	HLA11522		0	0	2	0	0	0	0	2									
DPB1*274:01	274:01	HLA11524		0	0	14	0	0	0	0	14			WD					WD	WD
DPB1*276:01	276:01	HLA11526		0	0	1	0	0	0	0	1									
DPB1*277:01	277:01	HLA11527		0	0	52	0	0	0	0	52			WD					WD	WD
DPB1*278:01	278:01	HLA11528		2	0	1	0	1	0	0	4									
**DPB1*279:01 total**	**279:01 total**			**0**	**0**	**2**	**0**	**0**	**0**	**0**	**2**									
DPB1*279:01	279:01			0	0	2	0	0	0	0	2									
DPB1*280:01	280:01	HLA11530		0	0	0	0	2	0	0	2									
DPB1*281:01	281:01	HLA11531		0	0	2	41	0	0	2	45				C				WD	C
DPB1*282:01	282:01	HLA11532		0	0	23	0	0	0	0	23			WD					WD	WD
DPB1*283:01	283:01	HLA11533		0	0	25	0	0	0	1	26			WD					WD	WD
DPB1*284:01	284:01	HLA11534		0	1	5	0	0	0	2	8			WD					WD	WD
DPB1*285:01	285:01	HLA11535		0	2	7	1	1	0	0	11			WD					WD	WD
DPB1*287:01	287:01	HLA11537		0	0	3	0	0	0	0	3									
DPB1*288:01	288:01	HLA11538		0	3	3	1	0	0	0	7								WD	WD
DPB1*289:01	289:01	HLA11545		0	0	2	0	0	0	0	2									
DPB1*290:01	290:01	HLA11546		6	0	24	3	0	0	2	35	WD		WD					WD	WD
DPB1*291:01	291:01	HLA11547		0	0	26	1	0	0	0	27			WD					WD	WD
DPB1*292:01	292:01	HLA11548		0	0	0	0	0	0	1	1									
DPB1*294:01	294:01	HLA11550		0	0	9	0	0	1	0	10			WD					WD	WD
DPB1*295:01	295:01	HLA11551		0	0	9	0	0	0	1	10			WD					WD	WD
DPB1*295:CODE	295:CODE			0	0	0	0	1	0	0	1	NA	NA	NA	NA	NA	NA	NA	NA	NA
DPB1*299:01	299:01	HLA11612		0	0	1	0	1	0	0	2									
DPB1*300:01	300:01	HLA11613		0	15	0	0	0	0	1	16		I						WD	I
DPB1*301:01	301:01	HLA11614		0	0	7	0	0	0	0	7			WD					WD	WD
DPB1*303:01	303:01	HLA11617		0	0	3	0	1	0	1	5								WD	WD

Supplemental Table 16: HLA-DPB1 Allele Summary [a]				Allele Count by Population Group [b]								3.0.0 CIWD Category by Population Group [c]								
Allele	Genomic Typing	Allele ID	G group	AFA	API	EURO	MENA	HIS	NAM	UNK	Total	AFA	API	EURO	MENA	HIS	NAM	UNK	Total	Highest Frequency
DPB1*304:01	304:01	HLA11618		0	0	3	0	0	0	0	3									
DPB1*305:01	305:01	HLA11619		0	2	3	0	0	0	1	6								WD	WD
DPB1*306:01	306:01	HLA11620		0	0	2	0	0	0	0	2									
DPB1*307:01	307:01	HLA11621		0	0	0	0	2	0	0	2									
DPB1*308:01	308:01	HLA11623		0	0	1	0	0	0	0	1									
DPB1*309:01	309:01	HLA11624		0	0	24	0	0	0	1	25			WD					WD	WD
DPB1*310:01	310:01	HLA11625		0	0	0	0	3	0	0	3									
DPB1*311:01	311:01	HLA11626		0	0	4	0	0	0	0	4									
DPB1*312:01	312:01	HLA11627		0	3	8	4	1	0	4	20			WD					WD	WD
DPB1*313:01	313:01	HLA11628		0	0	7	0	0	0	1	8			WD					WD	WD
DPB1*314:01	314:01	HLA11629		0	0	4	1	0	0	1	6								WD	WD
DPB1*315:01	315:01	HLA11630		1	0	0	0	0	0	1	2									
DPB1*316:01	316:01	HLA11631		0	1	1	0	0	0	0	2									
DPB1*318:01	318:01	HLA11692		0	0	15	0	0	0	2	17			WD					WD	WD
DPB1*319:01	319:01	HLA11693		0	0	3	0	0	1	2	6								WD	WD
DPB1*320:01	320:01	HLA11694		0	0	10	0	0	0	0	10			WD					WD	WD
DPB1*321:01	321:01	HLA11695		6	0	0	0	0	0	1	7	WD							WD	WD
DPB1*324:01	324:01	HLA11701		0	0	3	0	0	0	0	3									
DPB1*325:01	325:01	HLA11702		0	0	0	0	0	0	2	2									
DPB1*327:01	327:01	HLA11705		2	0	0	0	0	0	0	2									
DPB1*328:01N	328:01N	HLA11706		0	0	4	0	0	0	0	4									
DPB1*329:01	329:01	HLA11708		0	0	2	0	4	0	0	6								WD	WD
DPB1*330:01	330:01	HLA11907		0	2	0	0	0	0	0	2									
DPB1*331:01	331:01	HLA11908		0	1	0	0	0	0	0	1									
DPB1*333:01	333:01	HLA11910		3	0	0	0	5	0	1	9					WD			WD	WD
DPB1*334:01	334:01	HLA11911		0	0	4	0	0	0	0	4									
DPB1*335:01	335:01	HLA11912		0	0	1	0	0	0	0	1									
DPB1*337:01	337:01	HLA11914		0	0	1	0	0	0	0	1									
DPB1*338:01	338:01	HLA11917		0	0	0	0	4	0	0	4									
DPB1*339:01	339:01	HLA11918		0	0	5	0	0	0	1	6			WD					WD	WD
DPB1*340:01	340:01	HLA11919		0	1	14	0	0	0	0	15			WD					WD	WD

Supplemental Table 16: HLA-DPB1 Allele Summary [a]				Allele Count by Population Group [b]								3.0.0 CIWD Category by Population Group [c]								
Allele	Genomic Typing	Allele ID	G group	AFA	API	EURO	MENA	HIS	NAM	UNK	Total	AFA	API	EURO	MENA	HIS	NAM	UNK	Total	Highest Frequency
DPB1*341:01	341:01	HLA11920		0	0	2	0	0	0	0	2									
DPB1*342:01	342:01	HLA11921		0	0	3	0	0	0	0	3									
DPB1*343:01	343:01	HLA11923		0	0	2	0	0	0	0	2									
DPB1*345:01	345:01	HLA11925		0	0	5	0	0	0	0	5			WD					WD	WD
DPB1*346:01	346:01	HLA11926		1	0	0	0	0	0	1	2									
DPB1*347:01	347:01	HLA11927		0	0	0	0	0	0	1	1									
**DPB1*348:01 total**	**348:01 total**			**1**	**0**	**6**	**0**	**0**	**0**	**1**	**8**			**WD**					**WD**	**WD**
DPB1*348:01	348:01			1	0	1	0	0	0	1	3									
DPB1*348:01:01	348:01:01	HLA11928		0	0	5	0	0	0	0	5			WD					WD	WD
DPB1*349:01	349:01	HLA11929		0	0	2	0	1	0	1	4									
DPB1*350:CODE	350:CODE			0	0	0	0	0	0	1	1	NA	NA	NA	NA	NA	NA	NA	NA	NA
**DPB1*354:01 total**	**354:01 total**			**0**	**1**	**2**	**0**	**0**	**0**	**0**	**3**									
DPB1*354:01	354:01			0	1	0	0	0	0	0	1									
DPB1*354:01:02	354:01:02	HLA12374		0	0	2	0	0	0	0	2									
DPB1*355:01	355:01	HLA12062		0	1	6	0	4	0	3	14			WD					WD	WD
DPB1*356:01	356:01	HLA12063		0	5	0	0	0	0	0	5		WD						WD	WD
DPB1*357:01N	357:01N	HLA12064		0	6	2	0	0	0	4	12		WD						WD	WD
DPB1*358:01	358:01	HLA12065		1	1	8	0	0	0	0	10			WD					WD	WD
DPB1*359:01	359:01	HLA12066		0	0	1	0	0	0	0	1									
DPB1*360:01	360:01	HLA12067		0	1	0	2	0	0	0	3									
DPB1*362:01	362:01	HLA12069		0	0	1	0	0	0	0	1									
DPB1*363:01	363:01	HLA12181		0	9	2	0	0	0	1	12		WD						WD	WD
DPB1*364:01	364:01	HLA12222		0	0	0	0	1	0	0	1									
DPB1*365:01	365:01	HLA12225		0	0	1	0	0	0	1	2									
DPB1*366:01	366:01	HLA12226		0	2	0	0	0	0	0	2									
DPB1*367:01	367:01	HLA12227		1	0	1	0	0	0	1	3									
DPB1*368:01	368:01	HLA12228		0	0	0	0	1	0	0	1									
DPB1*369:01	369:01	HLA12229		0	0	1	0	2	0	1	4									
DPB1*370:01	370:01	HLA12230		3	0	2	0	0	0	0	5								WD	WD
DPB1*371:01	371:01	HLA12231		0	0	1	0	1	0	0	2									
DPB1*372:01	372:01	HLA12371		0	0	2	0	0	0	0	2									

Supplemental Table 16: HLA-DPB1 Allele Summary [a]				Allele Count by Population Group [b]								3.0.0 CIWD Category by Population Group [c]								
Allele	Genomic Typing	Allele ID	G group	AFA	API	EURO	MENA	HIS	NAM	UNK	Total	AFA	API	EURO	MENA	HIS	NAM	UNK	Total	Highest Frequency
DPB1*374:01	374:01	HLA12373		0	0	3	0	2	0	1	6								WD	WD
DPB1*375:01	375:01	HLA12375		0	0	4	0	0	0	0	4									
DPB1*376:01	376:01	HLA12378		0	0	1	0	0	0	0	1									
DPB1*377:01	377:01	HLA12379		3	1	6	0	0	0	2	12			WD					WD	WD
DPB1*380:01	380:01	HLA12382		0	3	0	0	0	0	0	3									
DPB1*381:01	381:01	HLA12384		0	1	0	0	0	0	0	1									
DPB1*382:01N	382:01N	HLA12385		1	0	0	0	3	0	0	4									
DPB1*384:01	384:01	HLA12388		1	0	1	0	0	0	0	2									
DPB1*390:01	390:01	HLA12395		0	0	1	0	0	0	0	1									
DPB1*391:01	391:01	HLA12396		0	10	0	0	0	0	0	10		WD						WD	WD
DPB1*392:01	392:01	HLA12397		0	1	0	0	0	0	0	1									
DPB1*393:01	393:01	HLA12425		0	0	5	0	1	0	1	7			WD					WD	WD
DPB1*394:01	394:01	HLA12438		0	8	3	0	0	0	1	12		WD						WD	WD
DPB1*396:01	396:01	HLA12538		0	0	4	0	0	0	1	5								WD	WD
DPB1*397:01	397:01	HLA12539		0	0	6	0	0	0	0	6			WD					WD	WD
DPB1*398:01	398:01	HLA12540		0	2	24	0	0	0	1	27			WD					WD	WD
DPB1*399:01	399:01	HLA12541		0	0	1	0	0	0	1	2									
DPB1*404:01	404:01	HLA12546		0	0	1	0	0	0	0	1									
DPB1*408:01	408:01	HLA12850		0	0	0	0	0	0	2	2									
DPB1*410:01	410:01	HLA12852		0	0	1	0	0	0	0	1									
DPB1*411:01	411:01	HLA12853		0	0	1	0	0	0	0	1									
DPB1*412:01	412:01	HLA12854		0	0	8	0	0	0	0	8			WD					WD	WD
DPB1*413:01	413:01	HLA12855		1	0	17	0	0	0	2	20			WD					WD	WD
DPB1*418:01	418:01	HLA12895		0	0	3	0	1	0	0	4									
DPB1*419:01	419:01	HLA12897		0	0	1	0	0	0	0	1									
DPB1*421:01	421:01	HLA12900		0	0	2	0	0	0	0	2									
DPB1*422:01	422:01	HLA12901		4	1	3	0	0	0	3	11								WD	WD
**DPB1*423:01 total**	**423:01 total**			**2**	**0**	**1**	**0**	**0**	**0**	**0**	**3**									
DPB1*423:01	423:01			2	0	1	0	0	0	0	3									
DPB1*424:01	424:01	HLA13297		0	0	4	0	0	0	2	6								WD	WD
DPB1*424:CODE	424:CODE			0	0	0	0	0	0	1	1	NA	NA	NA	NA	NA	NA	NA	NA	NA

Supplemental Table 16: HLA-DPB1 Allele Summary [a]				Allele Count by Population Group [b]								3.0.0 CIWD Category by Population Group [c]								
Allele	Genomic Typing	Allele ID	G group	AFA	API	EURO	MENA	HIS	NAM	UNK	Total	AFA	API	EURO	MENA	HIS	NAM	UNK	Total	Highest Frequency
DPB1*425:01	425:01	HLA13299		0	0	3	0	0	0	0	3									
DPB1*425:CODE	425:CODE			1	0	0	0	0	0	0	1	NA	NA	NA	NA	NA	NA	NA	NA	NA
DPB1*426:01	426:01	HLA13301		0	0	2	0	0	0	0	2									
DPB1*427:01	427:01	HLA13302		0	0	1	0	0	0	0	1									
DPB1*428:01	428:01	HLA13303		0	0	0	0	1	0	0	1									
DPB1*432:01	432:01	HLA13308		0	0	1	0	0	0	0	1									
DPB1*433:01	433:01	HLA13309		0	0	3	0	0	0	7	10							WD	WD	WD
DPB1*434:01	434:01	HLA13310		4	0	0	0	0	0	0	4									
DPB1*437:01	437:01	HLA13314		0	0	2	0	0	0	2	4									
DPB1*440:01	440:01	HLA13390		0	0	3	0	0	0	0	3									
DPB1*441:01	441:01	HLA13600		5	0	0	0	0	0	0	5	WD							WD	WD
DPB1*442:01	442:01	HLA13601		0	1	0	0	0	0	0	1									
DPB1*444:01	444:01	HLA13603		0	0	0	0	1	0	0	1									
DPB1*445:01	445:01	HLA13605		0	66	0	0	0	0	1	67		I						WD	I
DPB1*446:01	446:01	HLA13606		1	0	0	0	0	0	0	1									
DPB1*447:01	447:01	HLA13607		1	0	2	0	0	0	0	3									
DPB1*448:01	448:01	HLA13609		0	7	0	0	0	0	0	7		WD						WD	WD
DPB1*451:01	451:01	HLA13641		0	0	1	0	0	0	0	1									
DPB1*454:01	454:01	HLA13694		0	9	0	0	0	0	2	11		WD						WD	WD
DPB1*457:01	457:01	HLA13697		0	0	2	0	0	0	0	2									
DPB1*458:01	458:01	HLA13698		0	0	2	0	0	0	0	2									
DPB1*462:CODE	462:CODE			0	0	7	0	24	0	2	33	NA	NA	NA	NA	NA	NA	NA	NA	NA
DPB1*465:01	465:01	HLA13823		0	0	1	0	0	0	0	1									
DPB1*467:01	467:01	HLA13825		0	0	1	1	0	0	2	4									
DPB1*468:01	468:01	HLA13826		0	3	0	0	0	0	0	3									
DPB1*469:01	469:01	HLA13827		0	0	2	0	0	0	0	2									
DPB1*470:01	470:01	HLA13828		0	0	4	0	0	0	0	4									
DPB1*471:01	471:01	HLA13829		0	0	1	0	0	0	0	1									
DPB1*474:01	474:01	HLA13900		0	5	1	0	0	0	0	6		WD						WD	WD
DPB1*475:01	475:01	HLA13901		0	1	0	0	0	0	0	1									
DPB1*476:01	476:01	HLA13902		0	1	1	0	0	0	0	2									

Supplemental Table 16: HLA-DPB1 Allele Summary [a]				Allele Count by Population Group [b]								3.0.0 CIWD Category by Population Group [c]								
Allele	Genomic Typing	Allele ID	G group	AFA	API	EURO	MENA	HIS	NAM	UNK	Total	AFA	API	EURO	MENA	HIS	NAM	UNK	Total	Highest Frequency
DPB1*480:01	480:01	HLA13910		0	1	1	0	0	0	0	2									
DPB1*482:01	482:01	HLA13912		1	3	0	0	0	0	1	5								WD	WD
DPB1*483:01	483:01	HLA13913		0	1	0	0	0	0	0	1									
DPB1*484:01	484:01	HLA14056		0	0	0	0	0	0	1	1									
DPB1*485:01	485:01	HLA14057		0	1	1	0	0	0	0	2									
DPB1*486:01	486:01	HLA14058		0	6	0	0	0	0	0	6		WD						WD	WD
DPB1*487:01	487:01	HLA14059		0	1	0	0	0	0	1	2									
DPB1*488:01	488:01	HLA14060		0	0	0	0	2	0	0	2									
DPB1*489:01	489:01	HLA14061		0	2	1	0	0	0	0	3									
DPB1*490:01	490:01	HLA14062		0	3	1	0	0	0	0	4									
DPB1*492:01	492:01	HLA14064		0	0	2	0	0	0	0	2									
DPB1*493:01	493:01	HLA14065		3	0	0	0	0	0	0	3									
DPB1*495:01	495:01	HLA14191		5	0	1	0	0	0	0	6	WD							WD	WD
DPB1*499:01	499:01	HLA14267		0	2	0	0	0	0	0	2									
DPB1*500:01	500:01	HLA14268		0	0	2	0	0	0	0	2									
DPB1*501:01	501:01	HLA14269		0	0	8	5	0	0	0	13			WD	WD				WD	WD
DPB1*502:01	502:01	HLA14270		0	0	2	0	0	0	0	2									
DPB1*503:01	503:01	HLA14271		0	0	4	0	0	0	1	5								WD	WD
DPB1*504:01	504:01	HLA14272		0	2	0	0	0	0	0	2									
DPB1*505:01	505:01	HLA14273		0	0	3	0	0	0	0	3									
DPB1*507:01N	507:01N	HLA14275		0	3	0	0	0	0	0	3									
DPB1*508:01	508:01	HLA14276		0	0	3	0	0	0	0	3									
DPB1*512:01	512:01	HLA14364		1	0	0	0	0	0	0	1									
DPB1*514:01	514:01	HLA14367		0	0	5	0	1	0	0	6			WD					WD	WD
DPB1*517:01	517:01	HLA14370		0	0	1	0	0	0	0	1									
DPB1*520:01	520:01	HLA14578		0	5	0	0	0	0	0	5		WD						WD	WD
DPB1*521:01	521:01	HLA14579		0	0	4	1	0	0	0	5								WD	WD
DPB1*522:01	522:01	HLA14580		0	1	1	0	0	0	0	2									
DPB1*523:01	523:01	HLA14581		0	0	1	0	0	0	0	1									
DPB1*525:01	525:01	HLA14583		0	0	4	0	0	0	0	4									
DPB1*526:01	526:01	HLA14585		0	17	2	0	0	0	0	19		I						WD	I

Supplemental Table 16: HLA-DPB1 Allele Summary [a]				Allele Count by Population Group [b]								3.0.0 CIWD Category by Population Group [c]								
Allele	Genomic Typing	Allele ID	G group	AFA	API	EURO	MENA	HIS	NAM	UNK	Total	AFA	API	EURO	MENA	HIS	NAM	UNK	Total	Highest Frequency
DPB1*530:01	530:01	HLA14589		0	4	0	0	0	0	0	4									
DPB1*532:01	532:01	HLA14592		0	1	0	0	0	0	0	1									
DPB1*539:01	539:01	HLA14671		0	0	1	0	0	0	0	1									
DPB1*540:01	540:01	HLA14672		0	0	3	0	0	0	0	3									
DPB1*541:01	541:01	HLA14673		0	1	0	0	0	0	0	1									
DPB1*544:01	544:01	HLA14676		0	2	0	0	0	0	0	2									
DPB1*550:01	550:01	HLA14937		0	2	0	0	0	0	0	2									
DPB1*553:01	553:01	HLA15094		0	0	2	0	0	0	0	2									
DPB1*557:01	557:01	HLA15098		0	0	1	0	0	0	0	1									
DPB1*558:01	558:01	HLA15099		2	0	1	0	0	0	0	3									
DPB1*559:01	559:01	HLA15100		0	3	2	0	0	0	0	5								WD	WD
DPB1*566:01	566:01	HLA15107		0	0	3	0	0	0	0	3									
DPB1*567:01	567:01	HLA15108		0	2	1	0	0	0	0	3									
DPB1*568:01	568:01	HLA15110		0	0	0	0	1	0	0	1									
DPB1*570:01N	570:01N	HLA15146		0	5	0	0	0	0	0	5		WD						WD	WD
DPB1*575:01	575:01	HLA15445		0	2	0	0	0	0	0	2									
DPB1*576:01	576:01	HLA15447		0	0	1	0	0	0	0	1									
DPB1*578:01	578:01	HLA15449		0	0	1	0	0	0	0	1									
DPB1*588:01	588:01	HLA15719		0	0	1	0	0	0	0	1									
DPB1*604:01	604:01	HLA16006		0	1	0	0	0	0	0	1									
DPB1*605:01	605:01	HLA16007		0	0	0	0	0	0	1	1									
DPB1*609:01	609:01	HLA16011		0	1	0	0	0	0	0	1									
DPB1*614:01	614:01	HLA16054		0	2	0	0	0	0	0	2									
DPB1*620:01	620:01	HLA16352		0	1	0	0	0	0	0	1									
DPB1*623:01	623:01	HLA16395		0	0	2	0	0	0	1	3									
DPB1*631:01	631:01	HLA16600		0	3	0	0	0	0	0	3									
DPB1*635:01	635:01	HLA16604		0	0	2	0	0	0	0	2									
DPB1*656:01	656:01	HLA17548		0	1	0	0	0	0	0	1									
DPB1*679:01	679:01	HLA17948		0	1	0	0	0	0	0	1									
DPB1*683:01	683:01	HLA17952		0	1	0	0	0	0	0	1									
DPB1*NEW [d]	NEW			0	4	0	0	0	0	1	5	NA	NA	NA	NA	NA	NA	NA	NA	NA

Supplemental Table 16: HLA-DPB1 Allele Summary [a]				Allele Count by Population Group [b]								3.0.0 CIWD Category by Population Group [c]								
Allele	Genomic Typing	Allele ID	G group	AFA	API	EURO	MENA	HIS	NAM	UNK	Total	AFA	API	EURO	MENA	HIS	NAM	UNK	Total	Highest Frequency
DPB1*Total [e]	Total			336535	1082177	10680854	296914	581973	59113	969187	14006753									

C, common; I, intermediate; WD, well-documented; NA, not applicable

[a] All alleles observed in the current dataset are included in this table. Note that alleles are not in numerical order; alleles within a G group are clustered together. P group "two-field" total (e.g., written as "DPB1*01:01 total") and G group total summary rows are provided. The table does not list all alleles from IPD-IMGT version 3.31.0, if not present in the study dataset.

[b] Population groups include: AFA (African/African American), API (Asian/Pacific Islands), EURO (European/European descent), MENA (Middle East/North Coast of Africa), HIS (South or Central America/Hispanic/Latino), NAM (Native American populations) and UNK (unknown/not asked/multiple ancestries/other). Total is the overall population i.e., all groups combined.

[c] Allele frequency is calculated by dividing the number of times the “allele” of interest is observed in a population by the total number of copies of all the alleles at that particular genetic locus in the population (reported as the last row in this table and also in Table 2b). The total number of copies is calculated by multiplying the number of individuals times two for all loci except DRB3/4/5. For DRB3/4/5, the number of assignments was used as the total. The CIWD status is determined based on the allele frequency. Allele frequency data will be provided on the website of the next International HLA and Immunogenetics Workshop (https://www.ihiw18.org/). Highest frequency is the highest CIWD designation among all the individual groups.

[d] "CODE" is generically defined as a summary category of submitted HLA typing, including NMDP multiple allele codes, with ambiguities that are not within a single P or G group. "NEW" is a summary category for assignments of novel alleles that did not yet receive a nomenclature assignment. The CODE and NEW categories add to the total number of alleles but should not be assigned CIWD designations (labeled as NA, not applicable) as they do not represent a consistent allele designation (i.e., the NEW category may contain alleles with different DNA sequences that are unrelated to one another).

[e] DPB1*Total is the total number of allele assignments for the population group and is based on two times the number of individuals in the group. This number is also listed in Table 2b. It is not the sum of the column as alleles are not counted more than once. For example, when evaluating frequencies at the level of G resolution, individual alleles that make up the G group (e.g., A*80:01:01, A*80:01:01:01, A*80:01:01:02, A*80:01:01G) are not included in the count because these alleles are summed up in the total G designation (e.g., "A*80:01:01G total").
